# Investigating plasma lipid profiles in association with Parkinson’s disease risk

**DOI:** 10.1038/s41531-025-00955-8

**Published:** 2025-04-28

**Authors:** Houwen Zhang, Fangzheng Cao, Jialin Yu, Yu Liang, You Wu

**Affiliations:** 1 The Second Clinical Medical College of Zhejiang Chinese Medical University, Hangzhou, Zhejiang China; 2 The Second Affiliated Hospital of Zhejiang Chinese Medical University, Xinhua Hospital of Zhejiang Province, Hangzhou, China

**Keywords:** Movement disorders, Parkinson's disease

## Abstract

Parkinson’s Disease (PD) is associated with lipid metabolic disturbances, but the specific roles of lipids in its pathogenesis are unclear. This Mendelian Randomization (MR) study utilized GWAS data and IVW methods to investigate plasma lipids and PD risk. The genetic predispositions to altered levels of triacylglycerols (TAGs), diacylglycerols (DAGs), phosphatidylcholines (PCs), and phosphatidylethanolamines (PEs) are associated with an increased risk of PD, while the genetic predispositions to sphingomyelin (SM) and lysophosphatidylcholines (LPCs) are associated with a reduced risk of PD. Further research is needed to establish the biological mechanisms underlying these relationships.

## Introduction

Lipids play essential roles beyond energy storage^1^, acting as key components of cell membranes, including membrane rafts and protein anchors, and participating in various signaling and transport processes^2,3^. Parkinson’s Disease (PD) is associated with disturbances in multiple lipid metabolic pathways, including those involving glycerophospholipids^4^, sphingolipids^5^, and phosphatidylethanolamines (PEs)^6^. These disruptions can affect intracerebral signaling, membrane integrity and cell survival, potentially contributing to the pathogenesis of PD^7^. Despite these associations, the precise role of lipids in PD and their potential as therapeutic targets remain insufficiently understood, highlighting the need for further investigation to clarify their impact.

In PD research, the role of plasma lipids presents a complex picture. Some studies suggest that certain lipids, such as high-density lipoprotein (HDL) cholesterol, may provide neuroprotective benefits by slowing disease progression and mitigating neuronal damage through anti-inflammatory mechanisms^8,9^. Conversely, evidence suggests that dysregulated lipid levels, including oxidized low-density lipoprotein (LDL) cholesterol^10^ and specific polyunsaturated fatty acids (PUFAs)^11^, could increase PD risk by exacerbating neuronal dysfunction and death. Another viewpoint highlights the intricate balance between protective and harmful lipid species, such as oxidized cholesterol derivatives (oxysterols), indicating that this balance may be more critical for PD risk than the concentration of any individual lipid type. This complex interplay of lipids in PD underscores the necessity of comprehensive lipidomic analyses to better understand their roles in the disease’s pathology.

To further explore the involvement of specific lipids in PD risk, we employ Mendelian Randomization (MR) analysis^12^, a robust approach for causal inference that helps mitigate biases associated with exposure and confounding factors. In this study, we use MR to investigate the relationship between the genetic predispositions to 179 distinct lipid traits in the plasma lipidome and PD risk, assessing their potential as either protective or risk-enhancing factors.

## Results

### Instrumental Variables (IVs) Selection

We utilized publicly available summary-level data from GWAS to select IVs. The data for selected IVs are detailed in supplementary Table 1. For PD, 3456 SNPs were extracted for the 179 lipid traits (supplementary Table 2). All IVs had F statistics greater than 10, indicating that no weak IVs were included in our study^13^.

### Effects of Lipid Traits on PD Risk

**Fig. 1:**
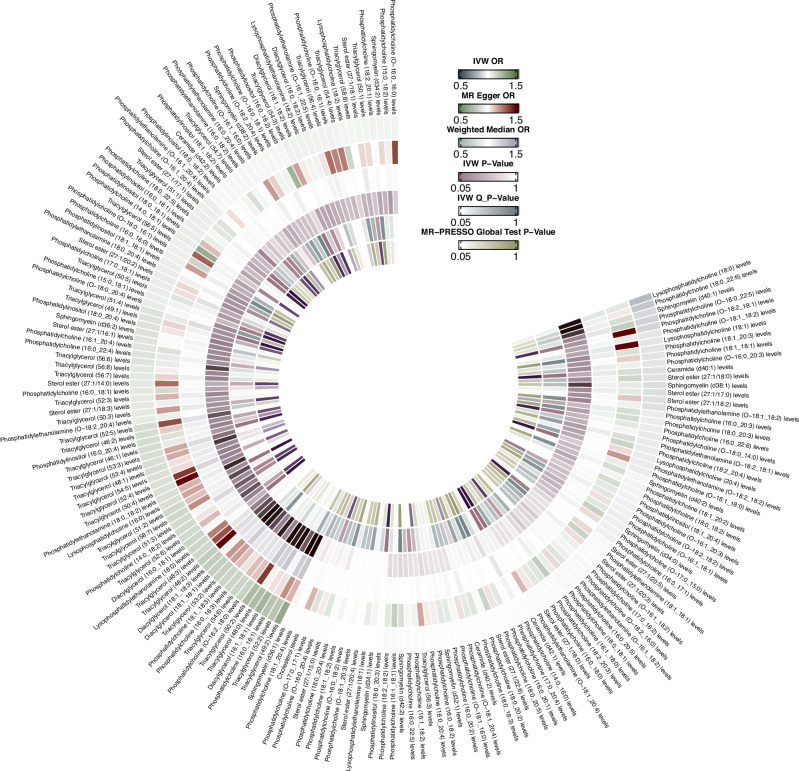
Circular heat map of the complete data of the MR analyses, heterogeneity and pleiotropy testing between the genetic predispositions to 179 lipid traits and PD risk. Circular heat map was generated to visualise the results of Mendelian randomisation analysis conducted on two sample groups, illustrating the correlation effects estimated by three different Mendelian randomization methods, horizontal pleiotropy test and heterogeneity test, with 179 lipid traits as the exposure variable and Parkinson’s Disease (PD) risk as the outcome variable. IVW, inverse variance weighted; Q, Cochran’s Q.

**Fig. 2:**
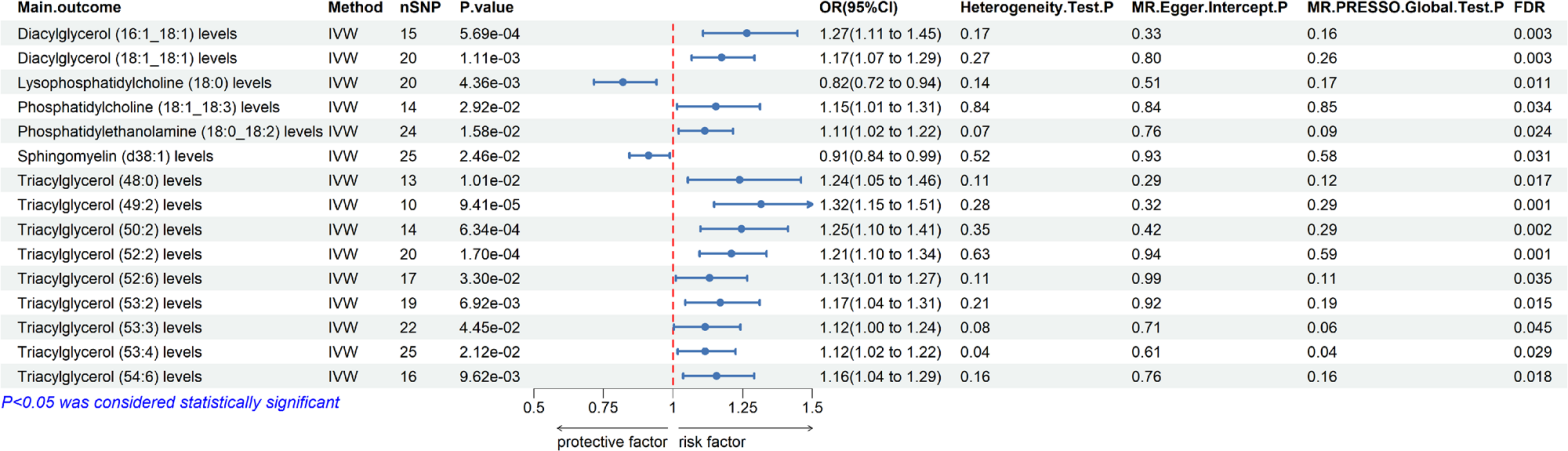
Forest plot of association of the genetic predispositions to lipid traits with risk of PD. The change of lipids in PD is represented by OR and the 95% confidence interval. FDR < 0.05 was found significant after multiple-comparison correction. Heterogeneity Test P < 0.05 indicated the presence of heterogeneity. The presence of horizontal pleiotropy was assessed using MR-Egger Intercept P < 0.05 or MR-PRESSO Global Test P < 0.05. IVW, inverse variance weighted; FDR, false discovery rate.

**Fig. 3:**
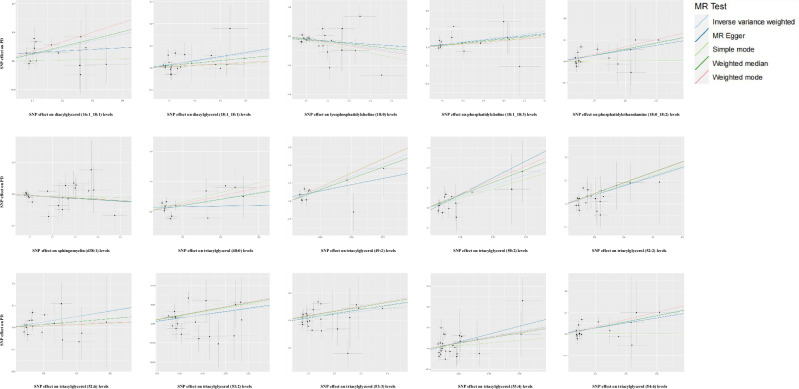
Scatter plot for MR analysis of the correlation effect of significant the genetic predispositions to lipids levels on PD risk. Single nucleotide polymorphism (SNP) associations with lipid traits risk are displayed versus individual SNP associations with PD in black dots. The 95% CI of odd ratio for each SNP is shown by vertical and horizontal lines. The slope of the lines represents the estimated correlation effect of the MR methods.

The effects of 179 lipid traits on PD risk are illustrated in Fig. 1. The genetic predispositions to alterations in plasma levels of diacylglycerols (DAGs: 16:1_18:1, 18:1_18:1), phosphatidylcholine (PC: 18:1_18:3), phosphatidylethanolamine (PE: 18:0_18:2), and multiple triacylglycerols (TAGs: 48:0, 49:2, 50:2, 52:2, 52:6, 53:2, 53:3, 53:4, 54:6) were associated with a potentially increased risk of PD (Figs. 2 and 3). Conversely, the genetic predispositions to alterations in lysophosphatidylcholine (LPC: 18:0) and sphingomyelin (SM: d38:1) levels were associated with a potentially reduced risk of PD (Figs. 2 and 3). Due to inconsistencies in the direction of results across the five MR analytical methods, DAG (18:1_18:3), PC (16:0_16:1, 18:0_22:6), SM (d40:1), and several TAGs (51:2, 51:3, 52:5, 56:6) were excluded, despite showing statistically significant results with the IVW method (supplementary Table 2). All statistically significant results were corrected for false discovery rate (FDR), with *p*-values less than 0.05.

### Effects of PD Risk on Lipid Traits

Reverse MR analysis revealed a potential correlation between the genetic predispositions to PD risk and alterations in DAG (18:1_18:1) (OR = 0.92, 95% CI 0.85–0.99, *P*_IVW_ = 0.02, *P*_Egger intercept_ = 0.95).

### Sensitivity Analyses

**Fig. 4:**
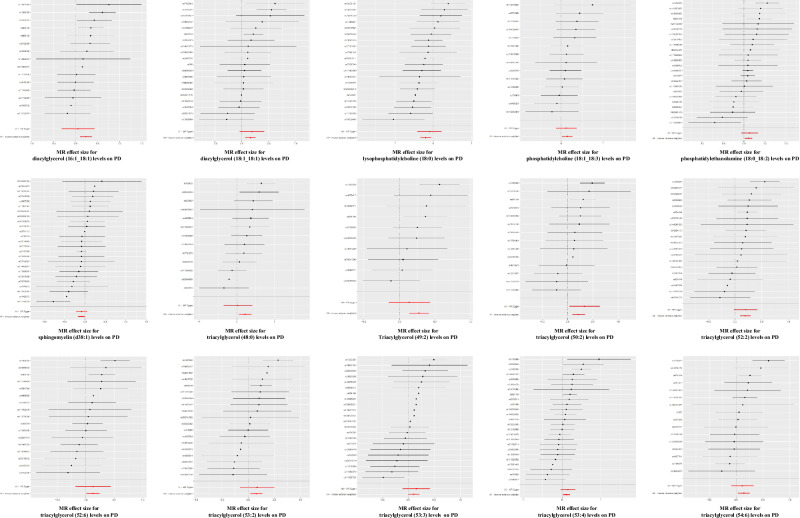
Forest plot of SNPs for MR analysis of the correlation effect of significant genetic predispositions to lipids levels on PD risk. The analysis evaluates the effect of individual single-nucleotide polymorphisms (SNPs) on Parkinson’s disease (PD) risk. The black horizontal solid lines represent the effect estimates of each SNP on PD risk, as determined using the Wald ratio method. Each black line corresponds to a specific SNP located in lipid-related genome-wide association study (GWAS). The red line represents the overall effect estimate of lipid-related SNPs on PD risk, derived from aggregating the effects of all individual SNPs.

**Fig. 5:**
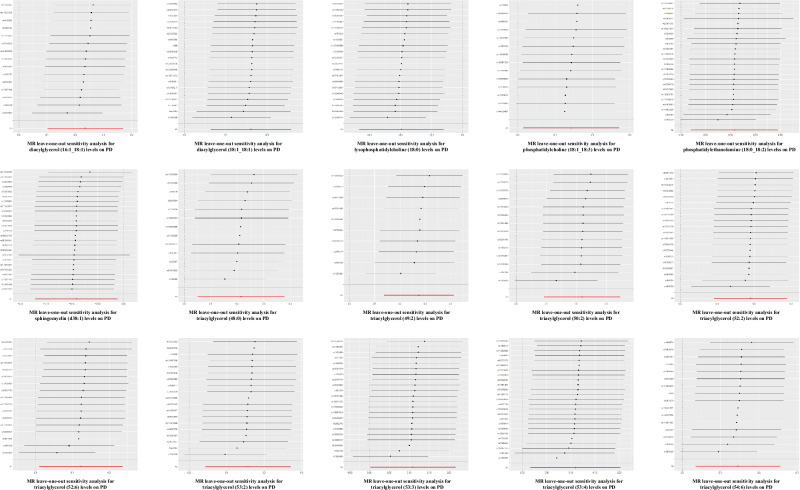
“Leave-one-out” analysis plot for MR analysis of the correlation effect of significant genetic predispositions to lipids levels on PD risk. By sequentially removing each single-nucleotide polymorphisms (SNPs), the meta-analysis effect of the remaining SNPs is recalculated to observe the changes in results following the exclusion of each SNP.

Among all statistically significant results, TAG (53:4) was removed due to significant heterogeneity (*P* = 0.04) and global test results (*P* = 0.037) (Fig. 2). For the remaining results, Cochran’s Q-test indicated no heterogeneity in the selected IVs (*P* > 0.05) (Figs. 2 and 4). Additionally, MR-Egger intercept and MR-PRESSO tests showed no evidence of horizontal pleiotropy or outlier values among the selected IVs for all lipid traits (*P* > 0.05) (Fig. 2). The leave-one-out test further confirmed the reliability of our MR analyses (Fig. 5).

## Discussion

Our study revealed that the genetic predispositions to plasma alterations in DAGs (16:1_18:1, 18:1_18:1), PC (18:1_18:3), PE (18:0_18:2), and several TAGs traits (48:0, 49:2, 50:2, 52:2, 52:6, 53:2, 53:3, 53:4, 54:6) were associated with a potentially increased risk of PD. Conversely, the genetic predispositions to alterations in LPC (18:0) and SM (d38:1) were linked to a decreased PD risk. Reverse MR analysis indicated a negative association between PD and alterations in DAG (18:1_18:1). These findings underscore the complex role of lipid metabolism in PD pathogenesis, as evidenced by our MR analysis.

The association of elevated DAG levels with increased PD risk is consistent with existing literature. Previous studies have suggested that DAG levels may serve as a predictive marker for PD severity within two years^14^. One recent metabolomics study identified plasma DAG levels as a key indicator for predicting the incidence of PD^15^, suggesting that DAG could be a biomarker for both the prognosis and pathogenesis of PD, with a strong correlation to its onset. Additionally, significantly elevated DAG levels have been detected in the frontal cortex of PD patients^16^, and blocking DAG biosynthesis has been shown to alleviate the toxicity of α-synuclein (α-syn), a hallmark protein in PD, further supporting the positive correlation between elevated DAG levels and PD risk^17^. This finding is consistent with our results. Notably, genetic studies have identified a link between the DAG Kinase theta (DGKζ) gene and PD susceptibility, providing genetic evidence that DAG dysregulation is associated with PD risk^18,19^. The DGK family of enzymes catalyzes the conversion of DAG to phosphatidic acid (PA), with multiple DGK isoforms expressed in the brain and nervous system, playing a role in neural signaling and synaptic plasticity^20^. PA is another critical lipid signaling molecule involved in numerous cellular processes and signaling pathways^21^. PA produced by DGKζ can activate PA-dependent effectors such as PI4P5Kα, which is essential for maintaining the PI cycle and regulating actin polymerization^21^. Furthermore, PA can be converted back to DAG by Pah1, a phosphatase, and maintaining the balance between DAG and PA is crucial for physiological functions. Studies have shown that inhibiting Pah1, which converts PA to DAG, reduces DAG production, inhibits lipid droplet formation, and prevents α-syn accumulation and aggregation on these droplets, thereby reducing α-syn toxicity^17^. This mechanism supports our finding that elevated DAG levels are associated with an increased risk of PD. However, in our reverse MR analysis, we observed a potentially negative association between the genetic predispositions to PD risk and the levels of DAG (18:1_18:1). This finding contrasts with studies that have reported a decline in plasma DAG levels in PD patients, which is consistent with our reverse MR analysis^22,23^. Notably, these findings diverge from the elevated DAG levels detected in specific brain regions, such as the frontal and visual cortices^16,24^, suggesting a complex dysregulation of DAG metabolism. This dysregulation may be intricately linked to both the progression and symptomatology of PD, highlighting the multifaceted nature of lipid metabolism in PD pathogenesis.

The role of TAGs in PD remains unclear, with previous studies showing varying results. It has been widely observed that peripheral blood TAG levels are significantly reduced in PD patients^22,25,26^, even before diagnosis^27^. However, other studies have found no significant differences in peripheral blood TAG levels between PD patients and control groups^28^. Variability in these findings may be attributed to differences in sex, race, and the methodologies used for TAG measurement^9,22,24^. In our study, we used single nucleotide polymorphisms (SNPs) as instrumental variables and identified a strong positive correlation between alterations in peripheral blood TAG levels and PD risk. In a Saccharomyces cerevisiae model, cells unable to synthesize triglycerides demonstrated increased tolerance to α-synuclein (α-syn) overexpression, highlighting a link between triglyceride metabolism and α-syn pathology^29^. Additionally, previous research has shown that both rotenone treatment and α-syn overexpression are associated with intracellular triglyceride accumulation, which is stored within lipid droplets^29,30^. α-syn has the ability to bind to these lipid droplets, reducing the turnover of stored triglycerides and enhancing α-syn aggregation^31^.

PC is an essential component of the cell membrane, involved in maintaining membrane integrity and function. A healthy cell membrane is crucial for the normal functioning of neurons^32^. LPC is a bioactive lipid derived from PC through hydrolysis by phospholipase A2 (PLA2). LPC participates in a wide range of physiological and pathological processes, including cell signaling, inflammatory responses, apoptosis, and metabolic regulation. It is found in plasma and various tissues and is involved in multiple cellular processes that could influence PD pathology. Previous studies have suggested that PC may have protective effects on the nervous system. For example, in the context of ApoE4, choline supplementation—a precursor of PC—has been shown to restore metabolic abnormalities associated with ApoE4 and improve cellular function^33^. Furthermore, animal studies using relatively low doses of 6-hydroxydopamine (6-OHDA) to induce partial nigrostriatal lesions have revealed that PC levels in the substantia nigra (SN) are significantly reduced in PD-like animals following induction. This suggests that decreased PC levels in neural tissue may contribute to the pathogenesis of PD^34^. In clinical studies, specific PC species, such as PC 34:2, 46:2, PC 34:5, 36:5, and 38:5, have been found to be reduced in the plasma and frontal cortex of PD patients, particularly in the substantia nigra of male PD patients, pointing to a notable alteration in PC metabolism associated with the disease. However, our research presents conflicting findings, showing that changes in peripheral blood PC levels are positively correlated with PD risk. This observation is consistent with previous studies, which reported that the PC/LPC ratio in the peripheral blood of PD patients is significantly higher than that in healthy controls, remaining elevated across all Hoehn and Yahr (H&Y) stages and throughout the disease course^35^. These findings support the idea that PC may play a more complex role in PD pathogenesis, with elevated plasma PC or reduced LPC potentially contributing to the disease process. In the 6-OHDA-induced PD animal model, it has been reported that most lipid levels in the LPC class are significantly downregulated, while LPC species such as LPC (16:0) and LPC (18:1) are upregulated. This suggests that LPC could be involved in PD pathogenesis, with different LPC species potentially playing distinct roles in disease development^34^. However, there is currently a lack of studies investigating the potential correlation between LPC and PD. Our research found that changes in LPC (18:0) levels were significantly negatively correlated with the risk of PD, suggesting that peripheral blood LPC (18:0) levels may have a protective effect against PD, and the specific mechanisms warrant further investigation. Therefore, although current research indicates that LPC and PC may play important roles in the pathogenesis of PD, further studies are needed to clarify their specific mechanisms and clinical application potential.

Increased levels of PE in the lipid rafts of the frontal cortex have been documented in PD patients^36^. Notably, phosphoethanolamine cytidylyltransferase^37^, an enzyme involved in PE synthesis, is found to be elevated in the SN of PD patients. This finding may help explain our results, where the genetic predispositions to elevated levels of PE (18:0_18:2) were identified as a potential risk factor for PD. The dysregulation of PE metabolism in PD could contribute to alterations in membrane composition and cellular function, potentially playing a role in the pathogenesis of the disease.

SM has been proposed to be involved in PD due to its presence in the myelin sheath and its role in nerve impulse transmission, presynaptic plasticity, and neurotransmitter receptor localization^38^. However, the precise role of SM in PD pathogenesis remains controversial. Evidence suggests that cell membrane lipid microdomains enriched in SM may influence α-Syn aggregation^39^. Specifically, α-Syn binds to vesicle membranes containing cholesterol and SM^40^, creating high-density lipoprotein-like (HDL-like) particles. In these particles, α-Syn partially forms a core structure with a disrupted helical conformation (residues 1–100) while the C-terminal residues (40) remain in a random-coil state^41^. Furthermore, the transfer of oligomeric α-Syn aggregates between neuron-like cells is mediated by extracellular vesicle release, a process governed by the SM/ceramide (Cer) ratio^42^. Reduced neutral sphingomyelinase (nSMase) levels inhibit Cer production in extracellular vesicles, leading to decreased α-Syn transfer. Supporting this, Tsutsumi et al. demonstrated that GW4869, an nSMase2 inhibitor, reduces exosomal release from microglial cells, thereby limiting inflammation^43^. The potential protective effect of the genetic predispositions to SM (d38:1) observed in our study adds to the complexity of sphingolipid metabolism in PD. While overall sphingolipid dysregulation may be a risk factor for PD^44^, certain sphingolipid species, such as SM (d38:1), may have protective roles. This highlights the need to consider individual lipid species and their interactions with other biomolecules and environmental factors, as these interactions could influence the pathological processes of PD.

In our study’s significant findings, the SNPs associated with lipid traits were not found to be located in PD risk-related genes such as SNCA^45^, LRRK2^46^, GBA1^47^, GALC^48^, SMPD1^49^, SREBF1^50^ and PLA2G6^51^. The SNPs selected for this study were based on their association with lipid traits from publicly available GWAS datasets, though they do not cover all genes involved in lipid metabolism or PD risk (supplementary Table 3). While genes like GBA1 and SMPD1 are linked to lipid metabolism and PD risk, their influence on lipid traits in our dataset may not have been sufficient for inclusion as instrumental variables. Additionally, the GWAS data may not have captured all relevant genetic variants or lipid-associated PD risk genes. However, we identified several significant shared SNPs that regulate lipid metabolism and susceptibility to PD, including rs1260326, rs769432, and rs117146578. These SNPs are located in the GCKR, OR3A3, and DRAM1 genes, respectively, with rs1260326 demonstrating the most extensive and significant effect on lipid regulation. The GCKR gene encodes glucose kinase regulatory protein (GKRP), which inhibits the interaction between glucose kinase and its substrate, playing a crucial role in glucose storage and disposal. Variants such as rs1260326 can diminish the inhibitory effect of GKRP on glucose kinase, thereby influencing glucose and lipid metabolism. This effect increases the risk of metabolic diseases like Type 2 Diabetes (T2D) and Non-Alcoholic Fatty Liver Disease (NAFLD)^52–54^. Specifically, the rs1260326 polymorphism in GCKR has been linked to a 21% increase in susceptibility to metabolic syndrome^55,56^. Metabolic syndrome represents a culmination of cardiometabolic abnormalities with a genetic basis, potentially leading to various disorders, including cardiovascular and neurological complications, and a general pro-inflammatory state^57^. Research has also suggested that variants like rs1260326 might be associated with an increased risk of Alzheimer’s Disease (AD) by influencing branched-chain amino acid levels^58^. The OR3A3 gene is part of a large family of G-protein-coupled receptors (GPCRs) that interact with odorant molecules in the nasal cavity^59^. Variants in OR3A3 may affect olfactory sensitivity and perception, potentially impacting conditions related to smell, such as anosmia or other sensory processing disorders. DRAM1 (DNA Damage Regulated Autophagy Modulator 1) encodes a lysosomal membrane protein involved in the regulation of autophagy and apoptosis^60^. DRAM1 participates in mitophagy, a selective autophagic process that removes damaged mitochondria. Mitochondrial dysfunction is closely related to the pathogenesis of PD, and DRAM1’s role in enhancing mitochondrial quality control may protect dopaminergic neurons from damage. By interacting with the BAX protein, DRAM1 regulates the interplay between autophagy and apoptosis. This regulation is particularly important when neurons are under stress or damage, potentially influencing the pathological progression of PD^61,62^. However, a direct association between these gene variants and PD has not yet been established, and such associations may involve polygenic effects that require further investigation.

While our study provides novel insights into the potential correlation relationships between alterations of plasma lipid species and PD risk, it is important to acknowledge certain limitations. The GWAS data utilized in our analysis predominantly originate from the Finnish population, which may limit the applicability of our findings to other ethnic groups. It is also unclear whether individuals with PD are included in this dataset. The genetic predispositions and their associations with lipid profiles and PD risk may vary across populations, and the SNPs used in our study do not overlap with those in the Australian Cadby et al. study^63^, likely due to population differences. Given that the PD risk SNPs in our study were derived from a European cohort, caution is needed when generalizing our findings across diverse populations. Replication studies in varied ethnicities are necessary to validate and extend our results. Secondly, although the MR approach is a powerful tool for assessing potential causal relationships, it is not immune to potential biases. Pleiotropy, where a single genetic variant influences multiple traits, cannot be entirely excluded and may confound the observed associations. While we performed sensitivity analyses to minimize the impact of pleiotropy, residual confounding cannot be completely ruled out. Lastly, our study relied on summary-level data from GWAS and did not have access to individual-level data. This limitation precluded us from conducting more detailed subgroup analyses or exploring potential gene-environment interactions that may modulate the relationship between lipid profiles and PD risk.

## Conclusion

In conclusion, our study employed a comprehensive MR approach to investigate the potential correlation between plasma lipid species and PD risk. We identified several specific lipid species, including the genetic predispositions to TAG, DAG, SM and PC that were potentially associated with an increased risk of PD. Conversely, the certain genetic predispositions to LPC and SM species were found to be potentially associated with a reduced risk of PD.

These findings provide novel insights into the complex role of lipid metabolism in the pathogenesis of PD and highlight the potential of specific lipid species as biomarkers or therapeutic targets. Our results lay the foundation for future mechanistic studies to elucidate the biological pathways through which these lipid species influence PD risk and to explore the potential of lipid-modifying interventions in the prevention or management of PD.

However, it is important to interpret these results in light of the study’s limitations, particularly the limited generalizability to diverse populations and the potential for residual confounding. Future research should focus on replicating these findings in larger, multi-ethnic cohorts, leveraging individual-level data to explore potential effect modifiers, and investigating the molecular mechanisms underlying the observed associations.

## Methods

**Fig. 6:**
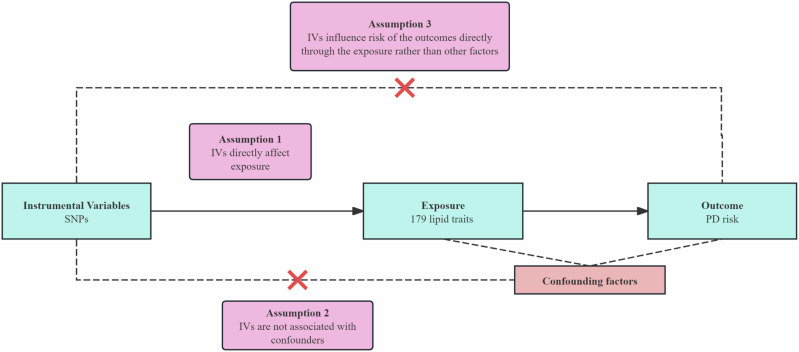
Overview of the study design. SNPs, single-nucleotide polymorphisms; PD, Parkinson’s Disease.

Figure 6 visually outlines the study design, providing a detailed representation.

### Data Sources

The lipidome dataset was sourced from a detailed genome-wide association analysis (GWAS), involving univariate and multivariate genome-wide analyses of 179 lipid species in a cohort of 7174 Finnish participants^64^. To dissect the complex lipidomic landscape, the study employed a clustering analysis strategy that treated glycerolipids and other lipid categories distinctly. Specifically, 44 lipid species, including TAGs and DAGs, were classified under glycerolipids and analyzed separately. The remaining 135 lipid species, which encompassed glycerophospholipids, sphingolipids, and sterols, were grouped and analyzed as another distinct cluster. This methodical approach allowed for a nuanced understanding of the genetic underpinnings across different lipid classes within the plasma lipidome.

The GWAS summary data on PD were obtained from a large-scale meta-analysis conducted by Nalls et al. (2019) from the International Parkinson’s Disease Genomics Consortium^65^. The study involved the analysis of 7.8 million single nucleotide polymorphisms (SNPs) in a combined cohort of 37,688 clinically diagnosed PD cases, 18,618 UK Biobank proxy-cases (self-reported PD), and 1,417,791 controls of European ancestry. Proxy-cases were included to maximize statistical power as the genetic correlation between proxy-cases and clinically diagnosed PD was found to be high (rG > 0.8). Genetic PD risk was defined based on the presence of specific genetic variants (SNPs) associated with PD. The meta-analysis identified 90 independent genome-wide significant association signals across 78 genomic loci, including 38 novel independent risk signals in 37 loci. These risk variants were used to construct polygenic risk scores (PRS) to evaluate an individual’s genetic risk for PD. The PRS, including 1805 variants, explained approximately 16-36% of the heritable risk of PD, depending on the assumed prevalence (0.5-2%). Furthermore, functional enrichment analyses indicated that the identified risk variants were involved in lysosomal pathways and acetylcholine neurotransmitter release in brain tissues, providing insights into the biological mechanisms underlying PD risk.

### Instrument Selection

We applied stringent QC filters to the GWAS data, including removal of SNPs with low call rates (<95%), deviation from Hardy-Weinberg equilibrium (P < 1×10 ^-6^), and low minor allele frequency (<1%). Individuals with high missingness (>5%), outlying heterozygosity rates, or discordant sex information were also excluded. Imputation was performed using the Haplotype Reference Consortium (HRC) panel, and only high-quality imputed SNPs (info score >0.8) were retained for subsequent analyses.

For the MR analyses, SNPs significantly associated with lipid traits were selected, adhering to a genome-wide significance threshold of P < 1 × 10^-5^ ^66^. To address the potential confounding effects of other diseases and environmental factors, we performed a rigorous screening of the SNPs used in our study. For each SNP, we conducted a search in the LDlink database (https://ldlink.nih.gov/?tab=home) to assess its genetic correlation with known factors that could potentially influence PD, such as the use of statin medications, exposure to heavy metals (lead, cadmium), or pesticide exposure. SNPs showing a genome-wide significant association (*P* < 5 × 10 ^-8^) with any of these confounding factors were excluded from subsequent analyses. In cases where a SNP exhibited multiple signals for a single trait, only the strongest signal was selected. Subsequently, SNPs within each trait were clumped to ensure the retention of only independent SNPs. The clumping was based on linkage disequilibrium (LD), with a threshold set at R^2^ < 0.001 and a clumping window of 10,000 kb^67^. A total of 4529 SNPs were selected, with details provided in supplementary Table 1.

In the reverse MR analysis, IVs for PD risk were selected using a stricter *p*-value criterion of less than 5 × 10^-8^ ^68^, adhering to the consensus standard for genome-wide significance. To guarantee the genetic variants’ independence, clumping was utilized, setting the window size to 5000 kb and specifying an R² < 0.01. A total of 23 SNPs were selected, with details provided in supplementary Table 1.

### MR Analysis

Five MR methods were utilized in our study to assess the correlation between the genetic predispositions to lipid traits and PD risk, as represented by odds ratios (OR) and 95% confidence intervals (CI). These methods included inverse variance weighted (IVW)^69^, MR-Egger^70^, weighted median, weighted mode, and simple mode^71^. The IVW method, aggregating Wald ratios weighted by their inverse variances, served as our primary method. Consistency in the direction of potential causal effects across all five methods was required to consider the results reliable. To mitigate the risk of Type 1 errors associated with testing multiple hypotheses, the significance threshold for statistically significant results within each group was adjusted using the FDR correction^72^.

### Sensitivity analysis

To enhance the robustness of our MR estimates, analyses of heterogeneity and horizontal pleiotropy were conducted. Heterogeneity was assessed using Cochran’s Q method^73^, with a significance threshold set at P < 0.05. To investigate potential horizontal pleiotropy, both the MR-Egger intercept test^74^ and the MR-PRESSO global test^75^ were employed. Additionally, a leave-one-out method was utilized to perform a robustness check^76^, thereby confirming the reliability of our findings.

For the purpose of this study, we utilized the software packages “TwoSampleMR” and “MRPRESSO” with R version 4.3.2 to conduct our analyses.

### Ethics Approval Statement

This study was conducted in accordance with the ethical standards of the institutional and national research committee and with the 1964 Helsinki declaration and its later amendments or comparable ethical standards. The data used in this research are publicly accessible and were obtained from established databases which have received approval from appropriate ethics review boards. Ethical clearance for the original studies that produced the genome-wide association data on plasma lipids and PD was obtained from the ethics committees of the respective institutions. No personal information was used, as this study only involved the analysis of anonymized and publicly available summary statistics.

All procedures performed in studies involving human participants were in accordance with ethical standards of the institution at which the studies were conducted. Informed consent was obtained from all individual participants involved in the original studies.

## Supplementary information

Supplementary tables

Financial Disclosures and Conflict of Interest declaration

## Data Availability

The lipid GWAS summary statistics were acquired from the GWAS Catalog at the European Bioinformatics Institute, with accession numbers ranging from GCST90277238 to GCST90277416. The information on PD referenced in our research was sourced from the Medical Research Center-Integrative Epidemiology Unit (MRC-IEU) OpenGWAS database (https://gwas.mrcieu.ac.uk/). Additional information can be found in the referenced original GWAS studies documented within our manuscript.
