# Supplementary material for: Investigating plasma lipid profiles in association with Parkinson’s disease risk
Source: NPJ Parkinsons Dis. 2025 Apr 28;11:99. doi: 10.1038/s41531-025-00955-8 (PMC12038023; doi:10.1038/s41531-025-00955-8)
Supplement: Supplementary file 1 — Supplementary tables [file 41531_2025_955_MOESM1_ESM.pdf]

Supplementary table 1

all SNPs of lipids													
lipid traits	chromosome	base_pair_location	effect_allele	other_allele	beta	standard_error	effect_allele_frequency	rsid	variant_id	info	n	pval	sampleid
Sterol ester (27:1/14:0) levels	1	72791134	A	G	7.99E-01	1.79E-01	2.54E-03	rs41289170	1_72791134_G_A	0.92	7174	8.30E-06	GCST90277238
Sterol ester (27:1/14:0) levels	2	27212506	A	G	8.85E-02	1.88E-02	7.23E-01	rs11608	2_27212506_G_A	1.00	7174	2.53E-06	GCST90277238
Sterol ester (27:1/14:0) levels	2	224951925	T	A	-3.80E-01	7.77E-02	1.25E-02	rs116778355	2_224951925_A_T	0.93	7174	1.00E-06	GCST90277238
Sterol ester (27:1/14:0) levels	3	195507255	C	G	8.96E-02	1.85E-02	7.11E-01	rs2686446	3_195507255_G_C	0.98	7174	1.28E-06	GCST90277238
Sterol ester (27:1/14:0) levels	5	174326694	C	T	8.75E-02	1.93E-02	7.53E-01	rs2964094	5_174326694_T_C	1.00	7174	5.94E-06	GCST90277238
Sterol ester (27:1/14:0) levels	7	17920067	C	G	-1.81E-01	3.75E-02	5.27E-02	rs55764039	7_17920067_G_C	0.99	7174	1.41E-06	GCST90277238
Sterol ester (27:1/14:0) levels	8	125487789	G	C	-8.41E-02	1.70E-02	4.10E-01	rs28601761	8_125487789_C_G	1.00	7174	7.71E-07	GCST90277238
Sterol ester (27:1/14:0) levels	9	27742537	A	T	7.90E-02	1.79E-02	3.75E-01	rs10812660	9_27742537_T_A	0.93	7174	9.81E-06	GCST90277238
Sterol ester (27:1/14:0) levels	10	122133760	G	A	-1.87E-01	4.10E-02	4.66E-02	rs80195215	10_122133760_A_G	0.94	7174	5.46E-06	GCST90277238
Sterol ester (27:1/14:0) levels	11	26008522	C	T	7.71E-02	1.74E-02	3.73E-01	rs12787616	11_26008522_T_C	0.99	7174	9.98E-06	GCST90277238
Sterol ester (27:1/14:0) levels	11	61796827	T	G	-8.05E-02	1.69E-02	4.36E-01	rs4246215	11_61796827_G_T	0.99	7174	1.80E-06	GCST90277238
Sterol ester (27:1/14:0) levels	12	101754541	T	G	-8.68E-02	1.67E-02	5.21E-01	rs7957768	12_101754541_G_T	1.00	7174	2.21E-07	GCST90277238
Sterol ester (27:1/14:0) levels	12	120950756	C	T	-7.96E-02	1.70E-02	5.85E-01	rs1696359	12_120950756_T_C	1.00	7174	2.84E-06	GCST90277238
Sterol ester (27:1/14:0) levels	13	111318086	A	G	-8.03E-02	1.82E-02	3.00E-01	rs2479959	13_111318086_G_A	1.00	7174	9.95E-06	GCST90277238
Sterol ester (27:1/14:0) levels	15	93906166	C	T	4.81E-01	1.03E-01	6.84E-03	rs75922719	15_93906166_T_C	0.95	7174	2.79E-06	GCST90277238
Sterol ester (27:1/14:0) levels	16	11851921	A	C	7.63E-02	1.70E-02	5.78E-01	rs7198817	16_11851921_C_A	1.00	7174	7.55E-06	GCST90277238
Sterol ester (27:1/14:0) levels	19	19269704	G	A	-2.34E-01	3.79E-02	5.35E-02	rs187429064	19_19269704_A_G	0.95	7174	6.89E-10	GCST90277238
Sterol ester (27:1/14:0) levels	19	44908684	C	T	1.42E-01	2.15E-02	1.89E-01	rs429358	19_44908684_T_C	1.00	7174	4.33E-11	GCST90277238
Sterol ester (27:1/14:0) levels	20	1965583	C	A	8.98E-02	1.92E-02	2.64E-01	rs6045698	20_1965583_A_C	0.98	7174	2.96E-06	GCST90277238
Sterol ester (27:1/14:0) levels	20	44413724	T	C	-1.86E-01	3.73E-02	5.24E-02	rs1800961	20_44413724_C_T	1.00	7174	5.99E-07	GCST90277238
Sterol ester (27:1/14:0) levels	22	43503538	C	T	8.59E-02	1.76E-02	3.44E-01	rs62232045	22_43503538_T_C	0.98	7174	1.04E-06	GCST90277238
Sterol ester (27:1/15:0) levels	1	30991480	T	C	-8.55E-02	1.91E-02	6.91E-01	rs1999811	1_30991480_C_T	1.00	6428	7.28E-06	GCST90277239
Sterol ester (27:1/15:0) levels	1	209576196	C	T	4.62E-01	1.02E-01	7.74E-03	rs17014799	1_209576196_T_C	1.00	6428	6.37E-06	GCST90277239
Sterol ester (27:1/15:0) levels	2	98354059	T	A	-4.60E-01	1.00E-01	8.71E-03	rs139857433	2_98354059_A_T	0.92	6428	4.49E-06	GCST90277239
Sterol ester (27:1/15:0) levels	3	39848105	G	A	8.18E-02	1.82E-02	3.73E-01	rs930917	3_39848105_A_G	1.00	6428	7.39E-06	GCST90277239
Sterol ester (27:1/15:0) levels	4	26516933	C	G	-3.30E-01	7.03E-02	1.70E-02	rs113844909	4_26516933_G_C	0.95	6428	2.77E-06	GCST90277239
Sterol ester (27:1/15:0) levels	5	40282763	T	C	-2.32E-01	4.57E-02	3.92E-02	rs115686032	5_40282763_C_T	0.99	6428	3.88E-07	GCST90277239
Sterol ester (27:1/15:0) levels	5	63595335	G	T	3.32E-01	7.38E-02	1.42E-02	rs150680623	5_63595335_T_G	0.99	6428	7.08E-06	GCST90277239
Sterol ester (27:1/15:0) levels	5	65839152	A	G	9.15E-02	2.01E-02	2.59E-01	rs2100040	5_65839152_G_A	1.00	6428	5.57E-06	GCST90277239
Sterol ester (27:1/15:0) levels	5	175777765	T	C	-7.80E-01	1.66E-01	3.54E-03	rs55662279	5_175777765_C_T	0.80	6428	2.72E-06	GCST90277239
Sterol ester (27:1/15:0) levels	7	50678097	T	C	-1.05E-01	2.23E-02	1.98E-01	rs2237478	7_50678097_C_T	1.00	6428	2.55E-06	GCST90277239
Sterol ester (27:1/15:0) levels	7	104180833	C	T	-3.27E-01	7.13E-02	1.65E-02	rs146236963	7_104180833_T_C	0.98	6428	4.49E-06	GCST90277239
Sterol ester (27:1/15:0) levels	8	4035643	C	G	-1.28E-01	2.88E-02	1.09E-01	rs62502692	8_4035643_G_C	0.97	6428	8.94E-06	GCST90277239
Sterol ester (27:1/15:0) levels	8	138441693	G	A	7.96E-02	1.76E-02	5.31E-01	rs985679	8_138441693_A_G	0.99	6428	6.64E-06	GCST90277239
Sterol ester (27:1/15:0) levels	10	129461219	T	C	1.09E-01	2.32E-02	1.78E-01	rs61859797	10_129461219_C_T	1.00	6428	2.63E-06	GCST90277239
Sterol ester (27:1/15:0) levels	11	61796827	T	G	-8.40E-02	1.78E-02	4.36E-01	rs4246215	11_61796827_G_T	0.99	6428	2.52E-06	GCST90277239
Sterol ester (27:1/15:0) levels	14	48229919	T	C	-1.03E-01	2.31E-02	1.82E-01	rs12589429	14_48229919_C_T	0.99	6428	8.08E-06	GCST90277239
Sterol ester (27:1/15:0) levels	17	14664504	A	G	3.67E-01	8.16E-02	1.28E-02	rs77201364	17_14664504_G_A	0.92	6428	6.93E-06	GCST90277239
Sterol ester (27:1/15:0) levels	19	30685856	A	T	-2.54E-01	5.65E-02	2.57E-02	rs140283718	19_30685856_T_A	0.92	6428	7.08E-06	GCST90277239
Sterol ester (27:1/15:0) levels	21	41477170	A	G	8.39E-02	1.85E-02	6.36E-01	rs2104810	21_41477170_G_A	1.00	6428	6.19E-06	GCST90277239
Sterol ester (27:1/15:0) levels	22	36797580	G	C	-1.05E-01	2.38E-02	8.23E-01	rs28582101	22_36797580_C_G	0.95	6428	9.75E-06	GCST90277239
Sterol ester (27:1/16:0) levels	1	55039974	T	G	-3.90E-01	4.64E-02	3.32E-02	rs11591147	1_55039974_G_T	1.00	7174	5.36E-17	GCST90277240
Sterol ester (27:1/16:0) levels	1	56335870	A	G	-2.86E-01	5.29E-02	2.76E-02	rs3005923	1_56335870_G_A	0.92	7174	6.58E-08	GCST90277240
Sterol ester (27:1/16:0) levels	1	154043206	A	G	-2.56E-01	5.67E-02	2.24E-02	rs7517801	1_154043206_G_A	1.00	7174	6.62E-06	GCST90277240
Sterol ester (27:1/16:0) levels	2	21041028	A	G	1.09E-01	1.84E-02	2.85E-01	rs1367117	2_21041028_G_A	1.00	7174	2.74E-09	GCST90277240
Sterol ester (27:1/16:0) levels	3	3342886	C	T	6.67E-01	1.42E-01	3.36E-03	rs56767222	3_3342886_T_C	0.99	7174	2.84E-06	GCST90277240
Sterol ester (27:1/16:0) levels	3	76900144	C	T	-8.70E-02	1.94E-02	2.52E-01	rs13085650	3_76900144_T_C	0.99	7174	7.31E-06	GCST90277240
Sterol ester (27:1/16:0) levels	4	37043069	G	C	7.59E-02	1.65E-02	5.58E-01	rs10031375	4_37043069_C_G	1.00	7174	4.14E-06	GCST90277240
Sterol ester (27:1/16:0) levels	4	72899760	A	G	2.47E-01	4.42E-02	3.78E-02	rs74915447	4_72899760_G_A	0.97	7174	2.55E-08	GCST90277240
Sterol ester (27:1/16:0) levels	4	73424911	T	C	2.24E-01	4.08E-02	4.51E-02	rs79146711	4_73424911_C_T	0.94	7174	4.45E-08	GCST90277240
Sterol ester (27:1/16:0) levels	4	74487710	C	A	3.30E-01	5.07E-02	2.81E-02	rs138270540	4_74487710_A_C	0.96	7174	8.36E-11	GCST90277240

Sterol ester (27:1/16:0) levels	4	164639807	G	T	-1.19E-01	2.47E-02	1.34E-01	rs28627829	4_164639807_T_G	0.96	7174	1.63E-06	GCST90277240
Sterol ester (27:1/16:0) levels	5	118775347	C	T	2.68E-01	5.87E-02	2.18E-02	rs10078182	5_118775347_T_C	0.95	7174	5.22E-06	GCST90277240
Sterol ester (27:1/16:0) levels	6	72631607	T	G	-8.95E-02	2.02E-02	2.17E-01	rs10943045	6_72631607_G_T	1.00	7174	9.57E-06	GCST90277240
Sterol ester (27:1/16:0) levels	6	124502870	G	A	7.96E-02	1.71E-02	5.86E-01	rs1336770	6_124502870_A_G	0.99	7174	3.07E-06	GCST90277240
Sterol ester (27:1/16:0) levels	6	152650134	T	C	2.23E-01	4.63E-02	3.34E-02	rs141836086	6_152650134_C_T	1.00	7174	1.48E-06	GCST90277240
Sterol ester (27:1/16:0) levels	9	136677616	G	C	-8.58E-02	1.77E-02	6.47E-01	rs2236514	9_136677616_C_G	0.95	7174	1.38E-06	GCST90277240
Sterol ester (27:1/16:0) levels	10	7162916	G	A	-7.61E-02	1.69E-02	5.80E-01	rs10795530	10_7162916_A_G	1.00	7174	7.08E-06	GCST90277240
Sterol ester (27:1/16:0) levels	10	52079201	T	C	7.51E-01	1.65E-01	2.76E-03	rs117010856	10_52079201_C_T	0.86	7174	5.80E-06	GCST90277240
Sterol ester (27:1/16:0) levels	11	43971474	A	G	7.88E-02	1.71E-02	6.17E-01	rs7932326	11_43971474_G_A	1.00	7174	4.13E-06	GCST90277240
Sterol ester (27:1/16:0) levels	11	61770929	C	G	-8.27E-02	1.70E-02	4.44E-01	rs174527	11_61770929_G_C	0.98	7174	1.24E-06	GCST90277240
Sterol ester (27:1/16:0) levels	11	61803311	C	T	-2.06E-01	1.68E-02	4.07E-01	rs174547	11_61803311_T_C	1.00	7174	4.77E-34	GCST90277240
Sterol ester (27:1/16:0) levels	11	62406721	G	A	9.43E-02	2.11E-02	8.04E-01	rs3017103	11_62406721_A_G	0.99	7174	8.15E-06	GCST90277240
Sterol ester (27:1/16:0) levels	11	88314021	A	G	-7.35E-02	1.66E-02	4.63E-01	rs538047	11_88314021_G_A	1.00	7174	9.75E-06	GCST90277240
Sterol ester (27:1/16:0) levels	11	116780399	T	C	-1.05E-01	1.92E-02	7.53E-01	rs11604424	11_116780399_C_T	1.00	7174	5.06E-08	GCST90277240
Sterol ester (27:1/16:0) levels	12	4796048	G	A	1.70E-01	3.65E-02	5.63E-02	rs9788072	12_4796048_A_G	0.95	7174	3.41E-06	GCST90277240
Sterol ester (27:1/16:0) levels	12	19677962	T	G	-9.60E-02	1.94E-02	2.46E-01	rs10841310	12_19677962_G_T	0.98	7174	7.87E-07	GCST90277240
Sterol ester (27:1/16:0) levels	12	53231170	G	C	1.09E-01	2.37E-02	1.47E-01	rs7487904	12_53231170_C_G	1.00	7174	3.94E-06	GCST90277240
Sterol ester (27:1/16:0) levels	12	101714807	T	C	-8.50E-02	1.67E-02	4.91E-01	rs10860778	12_101714807_C_T	1.00	7174	3.85E-07	GCST90277240
Sterol ester (27:1/16:0) levels	15	53458377	C	T	8.61E-02	1.67E-02	5.38E-01	rs7167665	15_53458377_T_C	0.99	7174	2.37E-07	GCST90277240
Sterol ester (27:1/16:0) levels	16	73349244	T	C	-8.45E-02	1.70E-02	5.57E-01	rs9924448	16_73349244_C_T	0.97	7174	7.15E-07	GCST90277240
Sterol ester (27:1/16:0) levels	18	65789123	G	A	-3.13E-01	6.91E-02	1.49E-02	rs75708826	18_65789123_A_G	0.98	7174	6.16E-06	GCST90277240
Sterol ester (27:1/16:0) levels	19	11079868	T	C	-2.09E-01	2.81E-02	9.46E-02	rs118068660	19_11079868_C_T	1.00	7174	1.02E-13	GCST90277240
Sterol ester (27:1/16:0) levels	19	15830645	G	A	-9.59E-02	1.92E-02	2.53E-01	rs73005445	19_15830645_A_G	1.00	7174	5.59E-07	GCST90277240
Sterol ester (27:1/16:0) levels	19	44908822	T	C	-4.07E-01	3.70E-02	5.31E-02	rs7412	19_44908822_C_T	1.00	7174	4.75E-28	GCST90277240
Sterol ester (27:1/16:0) levels	19	48224239	T	C	1.30E-01	2.75E-02	1.04E-01	rs56336136	19_48224239_C_T	0.97	7174	2.32E-06	GCST90277240
Sterol ester (27:1/16:0) levels	20	44413724	T	C	-2.22E-01	3.72E-02	5.24E-02	rs1800961	20_44413724_C_T	1.00	7174	2.60E-09	GCST90277240
Sterol ester (27:1/16:1) levels	1	1329726	A	T	-1.66E-01	3.50E-02	6.34E-02	rs142738732	1_1329726_T_A	0.96	7172	2.20E-06	GCST90277241
Sterol ester (27:1/16:1) levels	1	38245682	T	C	1.85E-01	4.00E-02	4.83E-02	rs111656006	1_38245682_C_T	0.97	7172	3.82E-06	GCST90277241
Sterol ester (27:1/16:1) levels	1	212561372	A	C	1.11E-01	2.32E-02	1.53E-01	rs11119973	1_212561372_C_A	1.00	7172	1.84E-06	GCST90277241
Sterol ester (27:1/16:1) levels	2	27508073	C	T	-9.04E-02	1.75E-02	6.51E-01	rs1260326	2_27508073_T_C	1.00	7172	2.33E-07	GCST90277241
Sterol ester (27:1/16:1) levels	2	62072939	C	T	2.43E-01	5.29E-02	2.61E-02	rs7355269	2_62072939_T_C	0.99	7172	4.35E-06	GCST90277241
Sterol ester (27:1/16:1) levels	2	167832629	T	C	1.17E-01	2.44E-02	1.44E-01	rs80227620	2_167832629_C_T	0.95	7172	1.73E-06	GCST90277241
Sterol ester (27:1/16:1) levels	4	14330096	G	T	5.58E-01	1.24E-01	4.90E-03	rs116522970	4_14330096_T_G	0.96	7172	7.01E-06	GCST90277241
Sterol ester (27:1/16:1) levels	4	110207431	T	C	-1.08E-01	2.12E-02	7.97E-01	rs5022521	4_110207431_C_T	0.94	7172	3.39E-07	GCST90277241
Sterol ester (27:1/16:1) levels	5	6743310	C	T	1.86E-01	3.93E-02	9.52E-01	rs274673	5_6743310_T_C	0.97	7172	2.24E-06	GCST90277241
Sterol ester (27:1/16:1) levels	7	138187033	T	C	1.69E-01	3.57E-02	5.75E-02	rs17603855	7_138187033_C_T	0.97	7172	2.13E-06	GCST90277241
Sterol ester (27:1/16:1) levels	9	27742537	A	T	8.01E-02	1.78E-02	3.75E-01	rs10812660	9_27742537_T_A	0.93	7172	7.26E-06	GCST90277241
Sterol ester (27:1/16:1) levels	9	33614216	C	T	7.62E-02	1.69E-02	5.82E-01	rs855500	9_33614216_T_C	1.00	7172	6.24E-06	GCST90277241
Sterol ester (27:1/16:1) levels	9	123419309	C	T	8.28E-02	1.70E-02	5.10E-01	rs10760286	9_123419309_T_C	0.97	7172	1.06E-06	GCST90277241
Sterol ester (27:1/16:1) levels	10	100315722	A	G	-1.56E-01	2.60E-02	1.21E-01	rs603424	10_100315722_G_A	1.00	7172	2.07E-09	GCST90277241
Sterol ester (27:1/16:1) levels	10	100600003	A	G	1.18E-01	2.42E-02	1.41E-01	rs4447106	10_100600003_G_A	0.98	7172	1.01E-06	GCST90277241
Sterol ester (27:1/16:1) levels	12	87990673	T	C	8.73E-02	1.94E-02	2.60E-01	rs7313803	12_87990673_C_T	0.95	7172	6.97E-06	GCST90277241
Sterol ester (27:1/16:1) levels	12	120977587	C	T	7.64E-02	1.68E-02	4.57E-01	rs2464190	12_120977587_T_C	0.99	7172	5.25E-06	GCST90277241
Sterol ester (27:1/16:1) levels	14	89719965	G	A	-1.94E-01	4.10E-02	4.30E-02	rs113130033	14_89719965_A_G	0.98	7172	2.22E-06	GCST90277241
Sterol ester (27:1/16:1) levels	16	11833509	T	C	8.39E-02	1.70E-02	5.75E-01	rs11647818	16_11833509_C_T	1.00	7172	8.02E-07	GCST90277241
Sterol ester (27:1/16:1) levels	16	71095785	A	C	-1.88E-01	3.81E-02	5.24E-02	rs201714080	16_71095785_C_A	0.97	7172	7.60E-07	GCST90277241
Sterol ester (27:1/16:1) levels	17	34678243	C	A	-6.45E-01	1.42E-01	3.78E-03	rs150352030	17_34678243_A_C	0.95	7172	5.67E-06	GCST90277241
Sterol ester (27:1/16:1) levels	17	51561964	T	C	-2.59E-01	5.25E-02	2.75E-02	rs116917626	17_51561964_C_T	0.97	7172	7.87E-07	GCST90277241
Sterol ester (27:1/16:1) levels	19	9621826	C	T	2.19E-01	4.95E-02	3.06E-02	rs117952876	19_9621826_T_C	0.99	7172	9.64E-06	GCST90277241
Sterol ester (27:1/16:1) levels	19	19269704	G	A	-2.17E-01	3.79E-02	5.35E-02	rs187429064	19_19269704_A_G	0.95	7172	9.82E-09	GCST90277241
Sterol ester (27:1/16:1) levels	19	19346108	T	C	-1.42E-01	3.15E-02	7.57E-02	rs58489806	19_19346108_C_T	1.00	7172	6.59E-06	GCST90277241
Sterol ester (27:1/16:1) levels	20	44413724	T	C	-2.04E-01	3.72E-02	5.24E-02	rs1800961	20_44413724_C_T	1.00	7172	4.34E-08	GCST90277241
Sterol ester (27:1/17:0) levels	1	160650361	G	A	1.19E-01	2.56E-02	1.41E-01	rs11265455	1_160650361_A_G	0.90	7047	3.30E-06	GCST90277242
Sterol ester (27:1/17:0) levels	1	210320724	A	C	7.59E-02	1.71E-02	5.41E-01	rs12735602	1_210320724_C_A	1.00	7047	9.09E-06	GCST90277242
Sterol ester (27:1/17:0) levels	2	30180840	C	T	-8.29E-02	1.86E-02	7.05E-01	rs1868072	2_30180840_T_C	0.98	7047	8.47E-06	GCST90277242
Sterol ester (27:1/17:0) levels	2	211666173	A	T	-9.97E-02	2.18E-02	1.83E-01	rs72945021	2_211666173_T_A	0.99	7047	5.01E-06	GCST90277242
Sterol ester (27:1/17:0) levels	3	11833970	G	A	-7.54E-02	1.69E-02	4.61E-01	rs9827613	3_11833970_A_G	0.99	7047	8.51E-06	GCST90277242

Sterol ester (27:1/17:0) levels	4	12263787	G	T	8.61E-02	1.72E-02	5.98E-01	rs13125535	4_12263787_T_G	1.00	7047	5.61E-07	GCST90277242
Sterol ester (27:1/17:0) levels	4	136648128	A	T	-3.10E-01	6.92E-02	1.49E-02	rs79568254	4_136648128_T_A	0.98	7047	7.56E-06	GCST90277242
Sterol ester (27:1/17:0) levels	6	84260623	A	G	1.80E-01	3.94E-02	5.00E-02	rs17791425	6_84260623_G_A	0.97	7047	4.90E-06	GCST90277242
Sterol ester (27:1/17:0) levels	6	122284176	G	A	3.36E-01	7.07E-02	1.60E-02	rs77943189	6_122284176_A_G	0.91	7047	2.05E-06	GCST90277242
Sterol ester (27:1/17:0) levels	7	104334784	G	A	1.18E-01	2.59E-02	1.23E-01	rs10241012	7_104334784_A_G	1.00	7047	4.98E-06	GCST90277242
Sterol ester (27:1/17:0) levels	9	12243520	C	T	1.02E-01	2.27E-02	8.35E-01	rs2382335	9_12243520_T_C	1.00	7047	7.21E-06	GCST90277242
Sterol ester (27:1/17:0) levels	9	86181820	T	C	-2.38E-01	5.35E-02	2.49E-02	rs111566713	9_86181820_C_T	0.98	7047	8.77E-06	GCST90277242
Sterol ester (27:1/17:0) levels	9	133279427	C	T	-1.00E-01	2.10E-02	7.98E-01	rs635634	9_133279427_T_C	1.00	7047	1.84E-06	GCST90277242
Sterol ester (27:1/17:0) levels	9	134243611	T	C	7.87E-02	1.76E-02	4.33E-01	rs10881589	9_134243611_C_T	0.92	7047	8.37E-06	GCST90277242
Sterol ester (27:1/17:0) levels	11	61796827	T	G	-8.46E-02	1.70E-02	4.36E-01	rs4246215	11_61796827_G_T	0.99	7047	6.70E-07	GCST90277242
Sterol ester (27:1/17:0) levels	11	71313656	C	T	3.23E-01	6.90E-02	1.58E-02	rs140015624	11_71313656_T_C	0.93	7047	2.96E-06	GCST90277242
Sterol ester (27:1/17:0) levels	12	8854565	G	T	-8.96E-02	2.02E-02	2.22E-01	rs4503592	12_8854565_T_G	0.99	7047	9.63E-06	GCST90277242
Sterol ester (27:1/17:0) levels	12	46735737	G	A	3.51E-01	7.69E-02	1.23E-02	rs116859210	12_46735737_A_G	1.00	7047	5.04E-06	GCST90277242
Sterol ester (27:1/17:0) levels	12	53118972	T	A	2.04E-01	2.52E-02	1.26E-01	rs2280696	12_53118972_A_T	0.99	7047	6.37E-16	GCST90277242
Sterol ester (27:1/17:0) levels	12	54038501	A	G	-1.92E-01	4.22E-02	4.18E-02	rs113505726	12_54038501_G_A	0.97	7047	5.11E-06	GCST90277242
Sterol ester (27:1/17:0) levels	12	100426307	G	A	1.73E-01	3.76E-02	5.43E-02	rs12296850	12_100426307_A_G	1.00	7047	4.26E-06	GCST90277242
Sterol ester (27:1/17:0) levels	15	48709000	A	T	-1.32E-01	2.66E-02	1.18E-01	rs79528439	15_48709000_T_A	0.97	7047	7.60E-07	GCST90277242
Sterol ester (27:1/17:0) levels	16	292437	C	T	-1.25E-01	2.42E-02	1.42E-01	rs12921107	16_292437_T_C	0.99	7047	2.38E-07	GCST90277242
Sterol ester (27:1/17:0) levels	16	25212475	C	T	1.70E-01	3.58E-02	9.38E-01	rs10775274	16_25212475_T_C	0.94	7047	1.93E-06	GCST90277242
Sterol ester (27:1/17:0) levels	16	31850641	A	G	-2.43E-01	5.32E-02	9.71E-01	rs2358779	16_31850641_G_A	0.91	7047	5.03E-06	GCST90277242
Sterol ester (27:1/17:0) levels	16	76465100	G	C	-1.42E-01	2.80E-02	1.01E-01	rs1002079	16_76465100_C_G	0.98	7047	3.89E-07	GCST90277242
Sterol ester (27:1/17:0) levels	19	44908684	C	T	1.22E-01	2.16E-02	1.89E-01	rs429358	19_44908684_T_C	1.00	7047	1.91E-08	GCST90277242
Sterol ester (27:1/17:0) levels	22	36797580	G	C	-1.08E-01	2.28E-02	8.23E-01	rs28582101	22_36797580_C_G	0.95	7047	2.22E-06	GCST90277242
Sterol ester (27:1/17:1) levels	2	224951925	T	A	-3.57E-01	7.79E-02	1.25E-02	rs116778355	2_224951925_A_T	0.93	7166	4.61E-06	GCST90277243
Sterol ester (27:1/17:1) levels	3	134998561	T	A	-7.86E-02	1.70E-02	4.06E-01	rs40435	3_134998561_A_T	1.00	7166	3.81E-06	GCST90277243
Sterol ester (27:1/17:1) levels	4	137812361	A	C	1.03E-01	2.28E-02	1.63E-01	rs72723967	4_137812361_C_A	0.99	7166	5.81E-06	GCST90277243
Sterol ester (27:1/17:1) levels	5	105868026	A	G	2.18E-01	4.91E-02	3.07E-02	rs79101108	5_105868026_G_A	0.98	7166	9.05E-06	GCST90277243
Sterol ester (27:1/17:1) levels	8	105734207	G	A	8.22E-02	1.83E-02	2.99E-01	rs6469013	8_105734207_A_G	0.99	7166	7.16E-06	GCST90277243
Sterol ester (27:1/17:1) levels	9	27742537	A	T	9.32E-02	1.79E-02	3.75E-01	rs10812660	9_27742537_T_A	0.93	7166	1.91E-07	GCST90277243
Sterol ester (27:1/17:1) levels	10	71286664	C	T	-2.43E-01	5.33E-02	2.68E-02	rs77392374	10_71286664_T_C	0.93	7166	5.17E-06	GCST90277243
Sterol ester (27:1/17:1) levels	12	68935916	A	C	-9.72E-02	2.16E-02	1.86E-01	rs11614909	12_68935916_C_A	0.99	7166	7.04E-06	GCST90277243
Sterol ester (27:1/17:1) levels	12	79317580	G	A	-8.45E-02	1.88E-02	2.72E-01	rs10746106	12_79317580_A_G	0.99	7166	7.24E-06	GCST90277243
Sterol ester (27:1/17:1) levels	12	101754513	A	G	-7.79E-02	1.68E-02	5.21E-01	rs7957655	12_101754513_G_A	1.00	7166	3.41E-06	GCST90277243
Sterol ester (27:1/17:1) levels	14	22802228	T	C	7.80E-02	1.70E-02	5.70E-01	rs7151696	14_22802228_C_T	1.00	7166	4.31E-06	GCST90277243
Sterol ester (27:1/17:1) levels	16	72072066	C	T	-9.29E-02	1.99E-02	2.28E-01	rs217184	16_72072066_T_C	0.99	7166	3.24E-06	GCST90277243
Sterol ester (27:1/17:1) levels	17	34703734	A	G	-6.34E-01	1.42E-01	3.79E-03	rs117691424	17_34703734_G_A	0.96	7166	8.36E-06	GCST90277243
Sterol ester (27:1/17:1) levels	18	55127832	G	A	-9.05E-01	2.02E-01	2.02E-03	rs141647056	18_55127832_A_G	0.85	7166	7.80E-06	GCST90277243
Sterol ester (27:1/17:1) levels	18	70340195	A	G	2.58E-01	5.35E-02	2.85E-02	rs76492272	18_70340195_G_A	0.88	7166	1.50E-06	GCST90277243
Sterol ester (27:1/17:1) levels	19	19269704	G	A	-2.11E-01	3.80E-02	5.35E-02	rs187429064	19_19269704_A_G	0.95	7166	3.10E-08	GCST90277243
Sterol ester (27:1/17:1) levels	19	41730113	C	A	-4.23E-01	9.18E-02	8.39E-03	rs193228861	19_41730113_A_C	0.95	7166	4.14E-06	GCST90277243
Sterol ester (27:1/17:1) levels	20	44413724	T	C	-1.98E-01	3.73E-02	5.24E-02	rs1800961	20_44413724_C_T	1.00	7166	1.08E-07	GCST90277243
Sterol ester (27:1/18:0) levels	1	5333063	A	G	1.75E-01	3.83E-02	5.25E-02	rs113270487	1_5333063_G_A	0.95	7157	5.00E-06	GCST90277244
Sterol ester (27:1/18:0) levels	1	164790578	T	C	4.95E-01	8.97E-02	9.43E-03	rs144944992	1_164790578_C_T	0.96	7157	3.58E-08	GCST90277244
Sterol ester (27:1/18:0) levels	2	131901250	G	T	-3.24E-01	6.28E-02	2.10E-02	rs6738602	2_131901250_T_G	0.86	7157	2.56E-07	GCST90277244
Sterol ester (27:1/18:0) levels	2	162800402	A	T	7.87E-02	1.74E-02	6.23E-01	rs7425339	2_162800402_T_A	1.00	7157	6.17E-06	GCST90277244
Sterol ester (27:1/18:0) levels	2	172761437	T	C	2.67E-01	5.99E-02	1.98E-02	rs141162511	2_172761437_C_T	0.99	7157	8.21E-06	GCST90277244
Sterol ester (27:1/18:0) levels	2	211659523	T	G	-1.01E-01	2.16E-02	1.85E-01	rs13405349	2_211659523_G_T	0.99	7157	3.35E-06	GCST90277244
Sterol ester (27:1/18:0) levels	3	13583299	T	C	-1.81E-01	3.76E-02	5.32E-02	rs55721138	3_13583299_C_T	0.96	7157	1.53E-06	GCST90277244
Sterol ester (27:1/18:0) levels	3	60928407	A	G	8.35E-02	1.82E-02	3.00E-01	rs11130797	3_60928407_G_A	1.00	7157	4.54E-06	GCST90277244
Sterol ester (27:1/18:0) levels	3	137801985	A	G	-1.31E-01	2.80E-02	9.83E-02	rs17482800	3_137801985_G_A	1.00	7157	2.99E-06	GCST90277244
Sterol ester (27:1/18:0) levels	4	37043069	G	C	7.53E-02	1.65E-02	5.58E-01	rs10031375	4_37043069_C_G	1.00	7157	5.46E-06	GCST90277244
Sterol ester (27:1/18:0) levels	5	135996882	C	T	-9.51E-02	2.04E-02	7.63E-01	rs30743	5_135996882_T_C	0.92	7157	2.99E-06	GCST90277244
Sterol ester (27:1/18:0) levels	6	124457214	C	T	9.41E-02	2.02E-02	7.83E-01	rs11154240	6_124457214_T_C	1.00	7157	3.12E-06	GCST90277244
Sterol ester (27:1/18:0) levels	6	154117109	A	G	1.22E-01	2.70E-02	1.14E-01	rs17181296	6_154117109_G_A	0.96	7157	6.76E-06	GCST90277244
Sterol ester (27:1/18:0) levels	7	44542387	C	T	9.71E-02	1.77E-02	3.39E-01	rs17725246	7_44542387_T_C	1.00	7157	4.14E-08	GCST90277244
Sterol ester (27:1/18:0) levels	7	136137436	C	G	8.11E-02	1.80E-02	3.19E-01	rs17242295	7_136137436_G_C	1.00	7157	6.63E-06	GCST90277244
Sterol ester (27:1/18:0) levels	9	133279427	C	T	-1.54E-01	2.07E-02	7.98E-01	rs635634	9_133279427_T_C	1.00	7157	1.31E-13	GCST90277244

Sterol ester (27:1/18:0) levels	11	16850893	A	G	8.36E-02	1.87E-02	2.88E-01	rs366590	11_16850893_G_A	0.98	7157	7.83E-06	GCST90277244
Sterol ester (27:1/18:0) levels	11	48217630	A	C	-1.03E-01	2.25E-02	1.60E-01	rs7130086	11_48217630_C_A	1.00	7157	4.68E-06	GCST90277244
Sterol ester (27:1/18:0) levels	11	61796827	T	G	-1.11E-01	1.69E-02	4.36E-01	rs4246215	11_61796827_G_T	0.99	7157	4.47E-11	GCST90277244
Sterol ester (27:1/18:0) levels	11	116786845	T	C	-1.09E-01	1.96E-02	7.65E-01	rs3741298	11_116786845_C_T	1.00	7157	3.09E-08	GCST90277244
Sterol ester (27:1/18:0) levels	12	53118972	T	A	2.54E-01	2.49E-02	1.26E-01	rs2280696	12_53118972_A_T	0.99	7157	3.69E-24	GCST90277244
Sterol ester (27:1/18:0) levels	12	53924362	C	T	-7.90E-02	1.68E-02	4.82E-01	rs10735853	12_53924362_T_C	1.00	7157	2.61E-06	GCST90277244
Sterol ester (27:1/18:0) levels	12	73198257	A	G	8.90E-02	1.98E-02	2.37E-01	rs10879574	12_73198257_G_A	0.99	7157	7.40E-06	GCST90277244
Sterol ester (27:1/18:0) levels	16	29117117	G	A	9.33E-02	1.92E-02	2.55E-01	rs11646327	16_29117117_A_G	0.99	7157	1.24E-06	GCST90277244
Sterol ester (27:1/18:0) levels	16	56953853	T	C	-1.04E-01	1.87E-02	2.76E-01	rs56228609	16_56953853_C_T	1.00	7157	2.73E-08	GCST90277244
Sterol ester (27:1/18:0) levels	19	15830645	G	A	-1.04E-01	1.92E-02	2.53E-01	rs73005445	19_15830645_A_G	1.00	7157	5.71E-08	GCST90277244
Sterol ester (27:1/18:0) levels	19	44908684	C	T	1.34E-01	2.15E-02	1.89E-01	rs429358	19_44908684_T_C	1.00	7157	5.26E-10	GCST90277244
Sterol ester (27:1/18:0) levels	22	27255754	C	T	8.14E-02	1.71E-02	3.98E-01	rs134783	22_27255754_T_C	1.00	7157	2.12E-06	GCST90277244
Sterol ester (27:1/18:1) levels	1	38265767	G	A	7.91E-02	1.78E-02	4.55E-01	rs12127700	1_38265767_A_G	0.88	7174	9.36E-06	GCST90277245
Sterol ester (27:1/18:1) levels	1	55039974	T	G	-2.98E-01	4.66E-02	3.32E-02	rs11591147	1_55039974_G_T	1.00	7174	1.63E-10	GCST90277245
Sterol ester (27:1/18:1) levels	1	56532951	G	C	2.42E-01	5.46E-02	2.45E-02	rs11588501	1_56532951_C_G	0.97	7174	9.06E-06	GCST90277245
Sterol ester (27:1/18:1) levels	1	154078023	C	T	-2.90E-01	5.71E-02	2.22E-02	rs4845593	1_154078023_T_C	1.00	7174	3.80E-07	GCST90277245
Sterol ester (27:1/18:1) levels	2	21148196	C	T	8.95E-02	1.92E-02	2.57E-01	rs11687710	2_21148196_T_C	1.00	7174	3.09E-06	GCST90277245
Sterol ester (27:1/18:1) levels	2	156522444	T	C	2.95E-01	5.97E-02	2.04E-02	rs115419570	2_156522444_C_T	0.98	7174	7.75E-07	GCST90277245
Sterol ester (27:1/18:1) levels	4	72899760	A	G	2.63E-01	4.43E-02	3.78E-02	rs74915447	4_72899760_G_A	0.97	7174	2.80E-09	GCST90277245
Sterol ester (27:1/18:1) levels	4	73124451	T	C	2.34E-01	4.49E-02	3.81E-02	rs114483871	4_73124451_C_T	0.93	7174	1.85E-07	GCST90277245
Sterol ester (27:1/18:1) levels	4	73947510	C	A	3.38E-01	5.30E-02	2.53E-02	rs182695896	4_73947510_A_C	0.96	7174	1.78E-10	GCST90277245
Sterol ester (27:1/18:1) levels	5	86717236	T	C	-3.84E-01	8.52E-02	9.82E-03	rs41441450	5_86717236_C_T	0.99	7174	6.70E-06	GCST90277245
Sterol ester (27:1/18:1) levels	5	118775347	C	T	2.68E-01	5.87E-02	2.18E-02	rs10078182	5_118775347_T_C	0.95	7174	4.93E-06	GCST90277245
Sterol ester (27:1/18:1) levels	6	32168770	G	T	-8.14E-02	1.69E-02	5.84E-01	rs1061808	6_32168770_T_G	1.00	7174	1.47E-06	GCST90277245
Sterol ester (27:1/18:1) levels	7	19940796	A	T	-1.59E-01	3.48E-02	6.17E-02	rs7804103	7_19940796_T_A	1.00	7174	5.05E-06	GCST90277245
Sterol ester (27:1/18:1) levels	9	27742537	A	T	7.99E-02	1.78E-02	3.75E-01	rs10812660	9_27742537_T_A	0.93	7174	7.44E-06	GCST90277245
Sterol ester (27:1/18:1) levels	9	116950689	A	C	2.88E-01	6.30E-02	1.76E-02	rs151274583	9_116950689_C_A	0.95	7174	4.99E-06	GCST90277245
Sterol ester (27:1/18:1) levels	11	43971474	A	G	7.72E-02	1.71E-02	6.17E-01	rs7932326	11_43971474_G_A	1.00	7174	6.56E-06	GCST90277245
Sterol ester (27:1/18:1) levels	12	19677962	T	G	-1.06E-01	1.94E-02	2.46E-01	rs10841310	12_19677962_G_T	0.98	7174	5.70E-08	GCST90277245
Sterol ester (27:1/18:1) levels	12	53231170	G	C	1.28E-01	2.37E-02	1.47E-01	rs7487904	12_53231170_C_G	1.00	7174	7.01E-08	GCST90277245
Sterol ester (27:1/18:1) levels	12	101714807	T	C	-8.15E-02	1.67E-02	4.91E-01	rs10860778	12_101714807_C_T	1.00	7174	1.16E-06	GCST90277245
Sterol ester (27:1/18:1) levels	12	124663423	G	A	-8.36E-02	1.71E-02	6.03E-01	rs701081	12_124663423_A_G	0.99	7174	1.09E-06	GCST90277245
Sterol ester (27:1/18:1) levels	13	26901722	T	C	7.23E-01	1.62E-01	3.00E-03	rs140055410	13_26901722_C_T	0.85	7174	8.50E-06	GCST90277245
Sterol ester (27:1/18:1) levels	13	31500828	G	A	1.91E-01	4.04E-02	4.56E-02	rs73176681	13_31500828_A_G	0.98	7174	2.33E-06	GCST90277245
Sterol ester (27:1/18:1) levels	13	72602312	TG	CG	1.27E-01	2.81E-02	1.01E-01	rs117802584	13_72602312_CG_TG	0.97	7174	5.82E-06	GCST90277245
Sterol ester (27:1/18:1) levels	14	101393317	C	T	-2.46E-01	5.41E-02	2.43E-02	rs4906111	14_101393317_T_C	1.00	7174	5.51E-06	GCST90277245
Sterol ester (27:1/18:1) levels	16	73349244	T	C	-7.74E-02	1.71E-02	5.57E-01	rs9924448	16_73349244_C_T	0.97	7174	5.93E-06	GCST90277245
Sterol ester (27:1/18:1) levels	17	80953542	A	C	-1.07E-01	1.97E-02	2.29E-01	rs55833830	17_80953542_C_A	0.99	7174	6.21E-08	GCST90277245
Sterol ester (27:1/18:1) levels	18	53959046	C	G	3.18E-01	6.73E-02	1.64E-02	rs117765824	18_53959046_G_C	0.96	7174	2.32E-06	GCST90277245
Sterol ester (27:1/18:1) levels	19	11079868	T	C	-1.72E-01	2.82E-02	9.46E-02	rs118068660	19_11079868_C_T	1.00	7174	1.07E-09	GCST90277245
Sterol ester (27:1/18:1) levels	19	15830645	G	A	-8.72E-02	1.92E-02	2.53E-01	rs73005445	19_15830645_A_G	1.00	7174	5.51E-06	GCST90277245
Sterol ester (27:1/18:1) levels	19	41031633	C	T	-7.89E-02	1.73E-02	6.30E-01	rs10404959	19_41031633_T_C	0.98	7174	5.25E-06	GCST90277245
Sterol ester (27:1/18:1) levels	19	44908822	T	C	-2.81E-01	3.72E-02	5.31E-02	rs7412	19_44908822_C_T	1.00	7174	4.01E-14	GCST90277245
Sterol ester (27:1/18:1) levels	20	44413724	T	C	-2.10E-01	3.72E-02	5.24E-02	rs1800961	20_44413724_C_T	1.00	7174	1.71E-08	GCST90277245
Sterol ester (27:1/18:2) levels	1	37622895	C	G	1.17E-01	2.46E-02	8.65E-01	rs10796911	1_37622895_G_C	1.00	7174	2.06E-06	GCST90277246
Sterol ester (27:1/18:2) levels	1	55039974	T	G	-3.58E-01	4.66E-02	3.32E-02	rs11591147	1_55039974_G_T	1.00	7174	1.69E-14	GCST90277246
Sterol ester (27:1/18:2) levels	2	21100497	G	A	1.04E-01	1.87E-02	2.78E-01	rs17395333	2_21100497_A_G	1.00	7174	3.06E-08	GCST90277246
Sterol ester (27:1/18:2) levels	4	36228194	T	C	-2.09E-01	4.46E-02	3.76E-02	rs61796597	4_36228194_C_T	0.96	7174	2.97E-06	GCST90277246
Sterol ester (27:1/18:2) levels	4	72540456	G	A	2.39E-01	4.75E-02	3.24E-02	rs144064722	4_72540456_A_G	0.98	7174	4.81E-07	GCST90277246
Sterol ester (27:1/18:2) levels	4	73424911	T	C	2.84E-01	4.09E-02	4.51E-02	rs79146711	4_73424911_C_T	0.94	7174	4.02E-12	GCST90277246
Sterol ester (27:1/18:2) levels	4	74505174	T	C	3.46E-01	5.09E-02	2.81E-02	rs116302332	4_74505174_C_T	0.96	7174	1.17E-11	GCST90277246
Sterol ester (27:1/18:2) levels	5	172483791	C	T	-6.38E-01	1.38E-01	3.72E-03	rs73327386	5_172483791_T_C	0.96	7174	3.66E-06	GCST90277246
Sterol ester (27:1/18:2) levels	6	5542174	G	A	7.76E-02	1.69E-02	5.85E-01	rs9405846	6_5542174_A_G	0.99	7174	4.63E-06	GCST90277246
Sterol ester (27:1/18:2) levels	8	9315848	A	G	7.86E-02	1.69E-02	5.12E-01	rs7012814	8_9315848_G_A	0.99	7174	3.22E-06	GCST90277246
Sterol ester (27:1/18:2) levels	9	89816177	A	C	1.06E-01	2.31E-02	8.28E-01	rs9330455	9_89816177_C_A	0.92	7174	4.30E-06	GCST90277246
Sterol ester (27:1/18:2) levels	10	5384541	A	G	-4.63E-01	9.83E-02	7.54E-03	rs117806675	10_5384541_G_A	0.98	7174	2.58E-06	GCST90277246
Sterol ester (27:1/18:2) levels	11	43971474	A	G	7.57E-02	1.71E-02	6.17E-01	rs7932326	11_43971474_G_A	1.00	7174	9.93E-06	GCST90277246

Sterol ester (27:1/18:2) levels	11	61837310	T	A	1.39E-01	1.90E-02	2.59E-01	rs2524299	11_61837310_A_T	1.00	7174	3.25E-13	GCST90277246
Sterol ester (27:1/18:2) levels	11	116780399	T	C	-9.73E-02	1.92E-02	7.53E-01	rs11604424	11_116780399_C_T	1.00	7174	4.37E-07	GCST90277246
Sterol ester (27:1/18:2) levels	12	19768671	T	C	3.25E-01	7.26E-02	1.45E-02	rs80339463	12_19768671_C_T	0.98	7174	7.90E-06	GCST90277246
Sterol ester (27:1/18:2) levels	12	112377818	T	C	2.24E-01	4.91E-02	3.22E-02	rs61941338	12_112377818_C_T	0.94	7174	5.44E-06	GCST90277246
Sterol ester (27:1/18:2) levels	16	88768837	C	A	5.00E-01	1.10E-01	6.10E-03	rs144863685	16_88768837_A_C	0.91	7174	6.12E-06	GCST90277246
Sterol ester (27:1/18:2) levels	17	45856678	C	T	-8.26E-02	1.84E-02	7.14E-01	rs2521811	17_45856678_T_C	0.98	7174	7.51E-06	GCST90277246
Sterol ester (27:1/18:2) levels	19	10308454	T	C	-8.15E-02	1.80E-02	3.12E-01	rs281417	19_10308454_C_T	1.00	7174	6.21E-06	GCST90277246
Sterol ester (27:1/18:2) levels	19	11079868	T	C	-1.94E-01	2.82E-02	9.46E-02	rs118068660	19_11079868_C_T	1.00	7174	6.81E-12	GCST90277246
Sterol ester (27:1/18:2) levels	19	44908822	T	C	-4.64E-01	3.69E-02	5.31E-02	rs7412	19_44908822_C_T	1.00	7174	7.79E-36	GCST90277246
Sterol ester (27:1/18:2) levels	19	44947071	A	G	-1.23E-01	2.24E-02	1.66E-01	rs9304645	19_44947071_G_A	0.99	7174	4.20E-08	GCST90277246
Sterol ester (27:1/18:2) levels	20	44413724	T	C	-2.16E-01	3.72E-02	5.24E-02	rs1800961	20_44413724_C_T	1.00	7174	7.42E-09	GCST90277246
Sterol ester (27:1/18:3) levels	1	55039974	T	G	-2.25E-01	4.67E-02	3.32E-02	rs11591147	1_55039974_G_T	1.00	7174	1.47E-06	GCST90277247
Sterol ester (27:1/18:3) levels	2	27508073	C	T	-1.05E-01	1.75E-02	6.51E-01	rs1260326	2_27508073_T_C	1.00	7174	1.64E-09	GCST90277247
Sterol ester (27:1/18:3) levels	2	62072939	C	T	2.59E-01	5.29E-02	2.61E-02	rs7355269	2_62072939_T_C	0.99	7174	9.93E-07	GCST90277247
Sterol ester (27:1/18:3) levels	2	136640311	G	T	6.05E-01	1.32E-01	4.45E-03	rs137900618	2_136640311_T_G	0.97	7174	5.00E-06	GCST90277247
Sterol ester (27:1/18:3) levels	3	182509337	C	T	1.88E-01	4.22E-02	4.83E-02	rs62294915	3_182509337_T_C	0.88	7174	8.97E-06	GCST90277247
Sterol ester (27:1/18:3) levels	5	50889130	G	A	9.51E-02	2.02E-02	2.28E-01	rs13172653	5_50889130_A_G	0.98	7174	2.62E-06	GCST90277247
Sterol ester (27:1/18:3) levels	6	94453722	T	C	5.01E-01	1.13E-01	5.84E-03	rs12199598	6_94453722_C_T	0.95	7174	8.72E-06	GCST90277247
Sterol ester (27:1/18:3) levels	9	130101985	T	C	2.10E-01	4.36E-02	3.81E-02	rs34500711	9_130101985_C_T	0.96	7174	1.59E-06	GCST90277247
Sterol ester (27:1/18:3) levels	11	61770929	C	G	-8.59E-02	1.71E-02	4.44E-01	rs174527	11_61770929_G_C	0.98	7174	5.00E-07	GCST90277247
Sterol ester (27:1/18:3) levels	11	61843278	A	G	-1.83E-01	1.69E-02	4.10E-01	rs174584	11_61843278_G_A	1.00	7174	4.02E-27	GCST90277247
Sterol ester (27:1/18:3) levels	12	101754513	A	G	-8.49E-02	1.67E-02	5.21E-01	rs7957655	12_101754513_G_A	1.00	7174	3.88E-07	GCST90277247
Sterol ester (27:1/18:3) levels	12	120977112	A	G	9.34E-02	1.70E-02	3.95E-01	rs2255531	12_120977112_G_A	1.00	7174	4.25E-08	GCST90277247
Sterol ester (27:1/18:3) levels	13	51210597	G	A	2.97E-01	6.62E-02	1.67E-02	rs146997505	13_51210597_A_G	0.96	7174	7.42E-06	GCST90277247
Sterol ester (27:1/18:3) levels	13	93173799	C	G	8.52E-02	1.93E-02	2.55E-01	rs319552	13_93173799_G_C	0.96	7174	9.76E-06	GCST90277247
Sterol ester (27:1/18:3) levels	14	89719965	G	A	-2.17E-01	4.10E-02	4.30E-02	rs113130033	14_89719965_A_G	0.98	7174	1.22E-07	GCST90277247
Sterol ester (27:1/18:3) levels	15	33326741	A	C	8.11E-02	1.71E-02	3.75E-01	rs1980102	15_33326741_C_A	1.00	7174	2.27E-06	GCST90277247
Sterol ester (27:1/18:3) levels	19	19268740	T	C	-1.69E-01	3.44E-02	6.26E-02	rs58542926	19_19268740_C_T	1.00	7174	1.01E-06	GCST90277247
Sterol ester (27:1/18:3) levels	19	19347579	G	A	-2.82E-01	3.78E-02	5.38E-02	rs182611493	19_19347579_A_G	0.95	7174	8.55E-14	GCST90277247
Sterol ester (27:1/18:3) levels	19	44908822	T	C	-1.91E-01	3.73E-02	5.31E-02	rs7412	19_44908822_C_T	1.00	7174	3.07E-07	GCST90277247
Sterol ester (27:1/18:3) levels	20	44413724	T	C	-2.40E-01	3.72E-02	5.24E-02	rs1800961	20_44413724_C_T	1.00	7174	1.30E-10	GCST90277247
Sterol ester (27:1/20:2) levels	2	25435229	C	G	-1.80E-01	3.72E-02	9.35E-01	rs6755057	2_25435229_G_C	0.99	5848	1.33E-06	GCST90277248
Sterol ester (27:1/20:2) levels	2	43225044	G	A	1.06E-01	1.97E-02	3.29E-01	rs7933	2_43225044_A_G	0.99	5848	9.14E-08	GCST90277248
Sterol ester (27:1/20:2) levels	2	43847292	T	C	-2.56E-01	2.17E-02	7.73E-01	rs4245791	2_43847292_C_T	1.00	5848	9.13E-32	GCST90277248
Sterol ester (27:1/20:2) levels	2	43853185	G	A	-2.11E-01	3.19E-02	9.98E-02	rs6709904	2_43853185_A_G	0.99	5848	3.77E-11	GCST90277248
Sterol ester (27:1/20:2) levels	2	60821224	C	T	-3.11E-01	6.75E-02	1.96E-02	rs79849855	2_60821224_T_C	0.97	5848	4.07E-06	GCST90277248
Sterol ester (27:1/20:2) levels	2	165115925	A	G	-3.88E-01	8.74E-02	1.20E-02	rs77590808	2_165115925_G_A	0.98	5848	9.32E-06	GCST90277248
Sterol ester (27:1/20:2) levels	2	225135785	A	G	2.93E-01	6.39E-02	2.29E-02	rs36194627	2_225135785_G_A	0.98	5848	4.70E-06	GCST90277248
Sterol ester (27:1/20:2) levels	3	7487632	G	A	1.69E-01	3.57E-02	7.29E-02	rs75823353	3_7487632_A_G	0.98	5848	2.39E-06	GCST90277248
Sterol ester (27:1/20:2) levels	4	73947510	C	A	2.86E-01	5.94E-02	2.53E-02	rs182695896	4_73947510_A_C	0.96	5848	1.52E-06	GCST90277248
Sterol ester (27:1/20:2) levels	4	96997008	C	T	-8.72E-02	1.97E-02	3.20E-01	rs13121156	4_96997008_T_C	1.00	5848	9.25E-06	GCST90277248
Sterol ester (27:1/20:2) levels	5	90954717	T	C	2.27E-01	5.13E-02	3.36E-02	rs74726976	5_90954717_C_T	0.99	5848	9.64E-06	GCST90277248
Sterol ester (27:1/20:2) levels	5	180649403	T	C	2.47E-01	5.53E-02	3.14E-02	rs114838148	5_180649403_C_T	0.94	5848	7.99E-06	GCST90277248
Sterol ester (27:1/20:2) levels	6	5531009	G	C	-9.23E-02	1.90E-02	3.78E-01	rs9504426	6_5531009_C_G	0.99	5848	1.16E-06	GCST90277248
Sterol ester (27:1/20:2) levels	6	138623575	C	T	1.31E-01	2.89E-02	1.19E-01	rs12527805	6_138623575_T_C	0.96	5848	6.29E-06	GCST90277248
Sterol ester (27:1/20:2) levels	7	39081865	T	C	-1.02E-01	2.01E-02	3.14E-01	rs1404999	7_39081865_C_T	1.00	5848	3.99E-07	GCST90277248
Sterol ester (27:1/20:2) levels	7	44532058	A	G	9.94E-02	2.18E-02	2.39E-01	rs55837134	7_44532058_G_A	0.98	5848	4.95E-06	GCST90277248
Sterol ester (27:1/20:2) levels	7	147774030	T	C	-9.56E-02	2.10E-02	2.69E-01	rs700320	7_147774030_C_T	1.00	5848	5.31E-06	GCST90277248
Sterol ester (27:1/20:2) levels	8	23548281	T	C	-1.68E-01	3.62E-02	6.92E-02	rs56276984	8_23548281_C_T	1.00	5848	3.57E-06	GCST90277248
Sterol ester (27:1/20:2) levels	9	133256205	C	G	-1.33E-01	2.58E-02	1.56E-01	rs7853989	9_133256205_G_C	1.00	5848	2.41E-07	GCST90277248
Sterol ester (27:1/20:2) levels	10	7348594	T	C	-2.69E-01	5.87E-02	2.62E-02	rs118137821	10_7348594_C_T	0.96	5848	4.91E-06	GCST90277248
Sterol ester (27:1/20:2) levels	10	29809803	T	C	-1.73E-01	3.81E-02	6.75E-02	rs10826704	10_29809803_C_T	0.96	5848	5.50E-06	GCST90277248
Sterol ester (27:1/20:2) levels	10	53105888	A	G	8.64E-02	1.84E-02	5.56E-01	rs1903973	10_53105888_G_A	1.00	5848	2.62E-06	GCST90277248
Sterol ester (27:1/20:2) levels	10	69935534	C	T	-1.91E-01	4.26E-02	5.46E-02	rs2244334	10_69935534_T_C	0.90	5848	7.39E-06	GCST90277248
Sterol ester (27:1/20:2) levels	12	120617356	C	T	-9.42E-02	1.98E-02	3.13E-01	rs6490297	12_120617356_T_C	0.99	5848	2.04E-06	GCST90277248
Sterol ester (27:1/20:2) levels	15	79180180	G	A	-9.02E-02	1.97E-02	6.09E-01	rs4778971	15_79180180_A_G	0.94	5848	4.61E-06	GCST90277248
Sterol ester (27:1/20:2) levels	16	26717568	C	A	9.87E-02	1.98E-02	6.70E-01	rs7202845	16_26717568_A_C	0.99	5848	6.41E-07	GCST90277248

Sterol ester (27:1/20:2) levels	19	44908684	C	T	1.62E-01	2.37E-02	1.89E-01	rs429358	19_44908684_T_C	1.00	5848	9.20E-12	GCST90277248
Sterol ester (27:1/20:3) levels	1	55170567	C	T	-2.45E-01	4.17E-02	4.20E-02	rs72660594	1_55170567_T_C	0.99	7171	4.38E-09	GCST90277249
Sterol ester (27:1/20:3) levels	2	10043013	G	C	-5.16E-01	1.07E-01	6.23E-03	rs79047909	2_10043013_C_G	0.96	7171	1.38E-06	GCST90277249
Sterol ester (27:1/20:3) levels	2	173386140	A	G	2.53E-01	5.63E-02	2.46E-02	rs6761859	2_173386140_G_A	0.94	7171	7.01E-06	GCST90277249
Sterol ester (27:1/20:3) levels	2	173688057	C	G	3.16E-01	6.68E-02	1.72E-02	rs112478356	2_173688057_G_C	0.90	7171	2.29E-06	GCST90277249
Sterol ester (27:1/20:3) levels	3	14644646	A	C	-1.52E-01	3.36E-02	6.68E-02	rs9310455	3_14644646_C_A	1.00	7171	5.99E-06	GCST90277249
Sterol ester (27:1/20:3) levels	3	72433728	G	A	3.92E-01	8.07E-02	1.20E-02	rs79771052	3_72433728_A_G	0.91	7171	1.25E-06	GCST90277249
Sterol ester (27:1/20:3) levels	4	72803111	A	G	2.72E-01	5.38E-02	2.56E-02	rs77645768	4_72803111_G_A	0.97	7171	4.39E-07	GCST90277249
Sterol ester (27:1/20:3) levels	4	73424911	T	C	1.86E-01	4.10E-02	4.51E-02	rs79146711	4_73424911_C_T	0.94	7171	5.45E-06	GCST90277249
Sterol ester (27:1/20:3) levels	4	73947510	C	A	2.85E-01	5.31E-02	2.53E-02	rs182695896	4_73947510_A_C	0.96	7171	8.01E-08	GCST90277249
Sterol ester (27:1/20:3) levels	5	38698557	G	A	-8.12E-02	1.72E-02	3.51E-01	rs1428498	5_38698557_A_G	1.00	7171	2.48E-06	GCST90277249
Sterol ester (27:1/20:3) levels	6	109151613	G	C	2.91E-01	5.98E-02	2.26E-02	rs117754141	6_109151613_C_G	0.92	7171	1.12E-06	GCST90277249
Sterol ester (27:1/20:3) levels	9	19882242	G	C	-9.31E-02	1.98E-02	2.51E-01	rs4977342	9_19882242_C_G	0.95	7171	2.47E-06	GCST90277249
Sterol ester (27:1/20:3) levels	9	109704483	A	G	3.76E-01	7.79E-02	1.15E-02	rs117079226	9_109704483_G_A	0.98	7171	1.47E-06	GCST90277249
Sterol ester (27:1/20:3) levels	9	131892383	C	T	-4.65E-01	9.66E-02	7.79E-03	rs80325645	9_131892383_T_C	0.96	7171	1.53E-06	GCST90277249
Sterol ester (27:1/20:3) levels	9	132336145	C	A	-1.34E-01	2.95E-02	9.08E-02	rs7036889	9_132336145_A_C	0.99	7171	5.37E-06	GCST90277249
Sterol ester (27:1/20:3) levels	11	61835886	T	A	-1.70E-01	1.90E-02	2.58E-01	rs2727271	11_61835886_A_T	1.00	7171	3.98E-19	GCST90277249
Sterol ester (27:1/20:3) levels	11	76043576	T	G	-8.41E-02	1.74E-02	4.44E-01	rs624605	11_76043576_G_T	0.94	7171	1.35E-06	GCST90277249
Sterol ester (27:1/20:3) levels	11	88318708	C	G	-8.20E-02	1.67E-02	5.48E-01	rs570929	11_88318708_G_C	1.00	7171	9.70E-07	GCST90277249
Sterol ester (27:1/20:3) levels	14	46693947	A	G	2.89E-01	5.90E-02	2.13E-02	rs111575978	14_46693947_G_A	0.96	7171	9.70E-07	GCST90277249
Sterol ester (27:1/20:3) levels	14	95283517	G	A	-8.89E-02	1.92E-02	7.35E-01	rs2352991	14_95283517_A_G	0.98	7171	3.61E-06	GCST90277249
Sterol ester (27:1/20:3) levels	15	53459505	G	A	8.34E-02	1.68E-02	5.57E-01	rs685850	15_53459505_A_G	0.99	7171	7.01E-07	GCST90277249
Sterol ester (27:1/20:3) levels	16	15038105	G	A	-2.26E-01	1.78E-02	3.15E-01	rs1135999	16_15038105_A_G	1.00	7171	1.90E-36	GCST90277249
Sterol ester (27:1/20:3) levels	16	15773795	G	T	1.12E-01	1.84E-02	2.96E-01	rs56374730	16_15773795_T_G	0.99	7171	1.15E-09	GCST90277249
Sterol ester (27:1/20:3) levels	17	51561964	T	C	-2.38E-01	5.25E-02	2.75E-02	rs116917626	17_51561964_C_T	0.97	7171	5.66E-06	GCST90277249
Sterol ester (27:1/20:3) levels	17	82619804	GT	G	-1.92E-01	4.10E-02	4.37E-02	rs199568207	17_82619804_G_GT	0.96	7171	2.99E-06	GCST90277249
Sterol ester (27:1/20:3) levels	19	15729265	C	A	-9.15E-02	1.93E-02	2.59E-01	rs3752149	19_15729265_A_C	0.99	7171	2.25E-06	GCST90277249
Sterol ester (27:1/20:3) levels	19	19269704	G	A	-2.36E-01	3.79E-02	5.35E-02	rs187429064	19_19269704_A_G	0.95	7171	4.92E-10	GCST90277249
Sterol ester (27:1/20:3) levels	19	44908822	T	C	-2.03E-01	3.73E-02	5.31E-02	rs7412	19_44908822_C_T	1.00	7171	5.69E-08	GCST90277249
Sterol ester (27:1/20:3) levels	20	18988572	T	C	-1.95E-01	4.05E-02	4.88E-02	rs62217148	20_18988572_C_T	0.91	7171	1.43E-06	GCST90277249
Sterol ester (27:1/20:3) levels	20	25297964	A	G	8.52E-02	1.69E-02	4.46E-01	rs7020	20_25297964_G_A	1.00	7171	4.67E-07	GCST90277249
Sterol ester (27:1/20:3) levels	20	44413724	T	C	-2.39E-01	3.72E-02	5.24E-02	rs1800961	20_44413724_C_T	1.00	7171	1.55E-10	GCST90277249
Sterol ester (27:1/20:4) levels	1	40824563	T	C	1.05E-01	2.21E-02	1.66E-01	rs3767941	1_40824563_C_T	0.99	7174	2.01E-06	GCST90277250
Sterol ester (27:1/20:4) levels	1	55039974	T	G	-2.97E-01	4.62E-02	3.32E-02	rs11591147	1_55039974_G_T	1.00	7174	1.30E-10	GCST90277250
Sterol ester (27:1/20:4) levels	1	201955621	C	G	8.44E-02	1.82E-02	2.91E-01	rs3820438	1_201955621_G_C	0.98	7174	3.54E-06	GCST90277250
Sterol ester (27:1/20:4) levels	2	17147264	T	G	-4.14E-01	7.73E-02	1.20E-02	rs186913978	2_17147264_G_T	0.95	7174	8.60E-08	GCST90277250
Sterol ester (27:1/20:4) levels	4	72899760	A	G	1.96E-01	4.39E-02	3.78E-02	rs74915447	4_72899760_G_A	0.97	7174	7.81E-06	GCST90277250
Sterol ester (27:1/20:4) levels	4	73947510	C	A	2.96E-01	5.25E-02	2.53E-02	rs182695896	4_73947510_A_C	0.96	7174	1.82E-08	GCST90277250
Sterol ester (27:1/20:4) levels	4	174018821	C	CT	-3.47E-01	6.53E-02	1.69E-02	rs201013676	4_174018821_CT_C	0.99	7174	1.08E-07	GCST90277250
Sterol ester (27:1/20:4) levels	4	174026533	A	G	-3.44E-01	6.56E-02	1.66E-02	rs6812370	4_174026533_G_A	1.00	7174	1.55E-07	GCST90277250
Sterol ester (27:1/20:4) levels	5	132304843	G	A	7.57E-02	1.69E-02	4.20E-01	rs6860806	5_132304843_A_G	0.99	7174	7.76E-06	GCST90277250
Sterol ester (27:1/20:4) levels	6	124438655	A	T	7.82E-02	1.68E-02	5.32E-01	rs9388335	6_124438655_T_A	0.99	7174	3.35E-06	GCST90277250
Sterol ester (27:1/20:4) levels	8	22193452	C	G	8.90E-01	1.94E-01	2.07E-03	rs7837272	8_22193452_G_C	0.90	7174	4.47E-06	GCST90277250
Sterol ester (27:1/20:4) levels	9	22932648	T	C	3.85E-01	8.54E-02	1.10E-02	rs117825513	9_22932648_C_T	0.96	7174	6.64E-06	GCST90277250
Sterol ester (27:1/20:4) levels	10	13808836	C	T	-1.61E-01	3.63E-02	5.75E-02	rs113603971	10_13808836_T_C	0.95	7174	9.89E-06	GCST90277250
Sterol ester (27:1/20:4) levels	11	12542442	T	C	8.45E-02	1.83E-02	3.04E-01	rs11022403	11_12542442_C_T	0.96	7174	4.15E-06	GCST90277250
Sterol ester (27:1/20:4) levels	11	61594967	G	A	-2.20E-01	4.47E-02	3.69E-02	rs79136768	11_61594967_A_G	0.98	7174	9.10E-07	GCST90277250
Sterol ester (27:1/20:4) levels	11	61744026	T	C	2.12E-01	2.45E-02	1.34E-01	rs3741252	11_61744026_C_T	0.99	7174	6.46E-18	GCST90277250
Sterol ester (27:1/20:4) levels	11	61776027	C	T	-6.48E-01	1.52E-02	4.14E-01	rs174528	11_61776027_T_C	1.00	7174	0.00E+00	GCST90277250
Sterol ester (27:1/20:4) levels	11	62056826	A	G	-3.27E-01	4.18E-02	4.33E-02	rs147981159	11_62056826_G_A	0.96	7174	6.76E-15	GCST90277250
Sterol ester (27:1/20:4) levels	11	62059462	G	A	-2.68E-01	4.49E-02	3.69E-02	rs113248417	11_62059462_A_G	0.98	7174	2.59E-09	GCST90277250
Sterol ester (27:1/20:4) levels	11	62454004	A	G	1.46E-01	1.97E-02	2.56E-01	rs4382917	11_62454004_G_A	0.92	7174	1.24E-13	GCST90277250
Sterol ester (27:1/20:4) levels	11	68794860	T	C	-1.53E-01	2.34E-02	1.56E-01	rs2229738	11_68794860_C_T	0.94	7174	7.01E-11	GCST90277250
Sterol ester (27:1/20:4) levels	13	38255030	T	A	-7.51E-02	1.69E-02	6.01E-01	rs646299	13_38255030_A_T	0.99	7174	9.30E-06	GCST90277250
Sterol ester (27:1/20:4) levels	13	110137139	T	A	-7.66E-02	1.68E-02	4.31E-01	rs9555673	13_110137139_A_T	0.98	7174	5.31E-06	GCST90277250
Sterol ester (27:1/20:4) levels	14	98823032	T	C	3.20E-01	7.09E-02	1.38E-02	rs117084735	14_98823032_C_T	0.99	7174	6.51E-06	GCST90277250
Sterol ester (27:1/20:4) levels	16	15038105	G	A	-1.01E-01	1.78E-02	3.15E-01	rs1135999	16_15038105_A_G	1.00	7174	1.23E-08	GCST90277250

Sterol ester (27:1/20:4) levels	16	57423359	G	A	-1.01E-01	2.19E-02	1.72E-01	rs223841	16_57423359_A_G	1.00	7174	4.23E-06	GCST90277250
Sterol ester (27:1/20:4) levels	16	87676514	C	T	1.78E-01	3.53E-02	5.99E-02	rs117186386	16_87676514_T_C	0.98	7174	4.60E-07	GCST90277250
Sterol ester (27:1/20:4) levels	18	44179195	C	T	1.05E-01	2.14E-02	1.84E-01	rs635196	18_44179195_T_C	1.00	7174	1.05E-06	GCST90277250
Sterol ester (27:1/20:4) levels	18	73089879	C	A	-9.05E-02	2.02E-02	7.88E-01	rs2469034	18_73089879_A_C	1.00	7174	7.35E-06	GCST90277250
Sterol ester (27:1/20:4) levels	19	7662522	A	G	-1.72E-01	3.65E-02	6.12E-02	rs138482835	19_7662522_G_A	0.91	7174	2.39E-06	GCST90277250
Sterol ester (27:1/20:4) levels	19	19269704	G	A	-2.54E-01	3.75E-02	5.35E-02	rs187429064	19_19269704_A_G	0.95	7174	1.21E-11	GCST90277250
Sterol ester (27:1/20:4) levels	19	44908822	T	C	-3.10E-01	3.68E-02	5.31E-02	rs7412	19_44908822_C_T	1.00	7174	4.29E-17	GCST90277250
Sterol ester (27:1/20:4) levels	20	25297964	A	G	7.47E-02	1.67E-02	4.46E-01	rs7020	20_25297964_G_A	1.00	7174	8.25E-06	GCST90277250
Sterol ester (27:1/20:4) levels	20	44413724	T	C	-1.67E-01	3.69E-02	5.24E-02	rs1800961	20_44413724_C_T	1.00	7174	5.95E-06	GCST90277250
Sterol ester (27:1/20:5) levels	1	8828055	T	C	-7.93E-02	1.75E-02	3.54E-01	rs4908782	1_8828055_C_T	0.99	7174	5.59E-06	GCST90277251
Sterol ester (27:1/20:5) levels	1	169678964	A	G	1.24E-01	2.57E-02	1.20E-01	rs34343838	1_169678964_G_A	0.99	7174	1.33E-06	GCST90277251
Sterol ester (27:1/20:5) levels	1	204934830	C	T	-1.25E-01	2.71E-02	1.07E-01	rs2625222	1_204934830_T_C	0.99	7174	3.84E-06	GCST90277251
Sterol ester (27:1/20:5) levels	3	176095628	C	T	1.60E-01	3.58E-02	6.35E-02	rs71629271	3_176095628_T_C	0.91	7174	8.36E-06	GCST90277251
Sterol ester (27:1/20:5) levels	4	37392086	G	A	-8.53E-02	1.66E-02	5.38E-01	rs2054498	4_37392086_A_G	1.00	7174	2.88E-07	GCST90277251
Sterol ester (27:1/20:5) levels	4	189640546	G	C	-3.07E-01	6.86E-02	1.70E-02	rs59807465	4_189640546_C_G	0.87	7174	7.85E-06	GCST90277251
Sterol ester (27:1/20:5) levels	5	23468036	A	G	-9.35E-02	2.11E-02	2.05E-01	rs4490551	5_23468036_G_A	0.96	7174	9.25E-06	GCST90277251
Sterol ester (27:1/20:5) levels	5	85519569	A	C	1.52E-01	3.13E-02	7.77E-02	rs143678376	5_85519569_C_A	0.98	7174	1.12E-06	GCST90277251
Sterol ester (27:1/20:5) levels	5	113784003	G	A	-8.20E-02	1.76E-02	4.00E-01	rs12515163	5_113784003_A_G	0.93	7174	3.21E-06	GCST90277251
Sterol ester (27:1/20:5) levels	6	156440204	G	T	7.91E-02	1.70E-02	4.55E-01	rs980814	6_156440204_T_G	0.96	7174	3.55E-06	GCST90277251
Sterol ester (27:1/20:5) levels	7	132693734	A	C	-7.65E-02	1.70E-02	4.09E-01	rs7780677	7_132693734_C_A	1.00	7174	7.21E-06	GCST90277251
Sterol ester (27:1/20:5) levels	8	3077293	C	G	1.69E-01	3.80E-02	5.14E-02	rs75897070	8_3077293_G_C	0.97	7174	8.62E-06	GCST90277251
Sterol ester (27:1/20:5) levels	8	132397243	C	G	-1.06E-01	2.26E-02	1.75E-01	rs11986253	8_132397243_G_C	0.96	7174	3.00E-06	GCST90277251
Sterol ester (27:1/20:5) levels	8	142670127	GGAGA	G	-4.26E-01	9.57E-02	8.67E-03	rs200322055	8_142670127_G_GGAG	0.88	7174	8.75E-06	GCST90277251
Sterol ester (27:1/20:5) levels	9	16421864	CCT	C	-7.60E-02	1.67E-02	4.64E-01	rs5896690	9_16421864_C_CCT	0.99	7174	5.48E-06	GCST90277251
Sterol ester (27:1/20:5) levels	11	42657198	C	T	1.11E-01	2.30E-02	1.55E-01	rs7947954	11_42657198_T_C	1.00	7174	1.55E-06	GCST90277251
Sterol ester (27:1/20:5) levels	11	61481911	A	C	-1.01E-01	2.14E-02	8.03E-01	rs3019200	11_61481911_C_A	1.00	7174	2.37E-06	GCST90277251
Sterol ester (27:1/20:5) levels	11	61770929	C	G	-1.29E-01	1.71E-02	4.44E-01	rs174527	11_61770929_G_C	0.98	7174	4.44E-14	GCST90277251
Sterol ester (27:1/20:5) levels	11	61839211	A	G	-4.11E-01	1.64E-02	4.10E-01	rs174581	11_61839211_G_A	1.00	7174	3.56E-133	GCST90277251
Sterol ester (27:1/20:5) levels	11	62451557	G	A	1.10E-01	1.97E-02	2.57E-01	rs7936002	11_62451557_A_G	0.93	7174	2.53E-08	GCST90277251
Sterol ester (27:1/20:5) levels	11	68908029	T	C	-1.58E-01	2.87E-02	9.80E-02	rs508049	11_68908029_C_T	0.96	7174	3.35E-08	GCST90277251
Sterol ester (27:1/20:5) levels	11	103598670	G	A	1.13E-01	2.44E-02	8.61E-01	rs1481977	11_103598670_A_G	1.00	7174	3.79E-06	GCST90277251
Sterol ester (27:1/20:5) levels	12	101714807	T	C	-8.58E-02	1.68E-02	4.91E-01	rs10860778	12_101714807_C_T	1.00	7174	3.23E-07	GCST90277251
Sterol ester (27:1/20:5) levels	15	53447803	G	C	8.57E-02	1.89E-02	2.62E-01	rs17548303	15_53447803_C_G	0.99	7174	6.25E-06	GCST90277251
Sterol ester (27:1/20:5) levels	16	10434179	G	A	1.07E-01	2.27E-02	1.65E-01	rs62025936	16_10434179_A_G	0.99	7174	2.75E-06	GCST90277251
Sterol ester (27:1/20:5) levels	19	19347579	G	A	-1.81E-01	3.79E-02	5.38E-02	rs182611493	19_19347579_A_G	0.95	7174	1.87E-06	GCST90277251
Sterol ester (27:1/20:5) levels	19	44905307	T	A	-1.33E-01	2.51E-02	1.36E-01	rs449647	19_44905307_A_T	0.95	7174	1.07E-07	GCST90277251
Sterol ester (27:1/20:5) levels	21	41847687	A	G	8.56E-02	1.90E-02	2.68E-01	rs8132104	21_41847687_G_A	1.00	7174	6.98E-06	GCST90277251
Sterol ester (27:1/22:6) levels	1	54369167	T	C	-1.75E-01	3.54E-02	6.10E-02	rs17110517	1_54369167_C_T	0.98	7173	7.85E-07	GCST90277252
Sterol ester (27:1/22:6) levels	1	55039974	T	G	-2.38E-01	4.69E-02	3.32E-02	rs11591147	1_55039974_G_T	1.00	7173	3.90E-07	GCST90277252
Sterol ester (27:1/22:6) levels	1	119349977	A	G	2.45E-01	5.47E-02	2.45E-02	rs77708260	1_119349977_G_A	0.99	7173	7.41E-06	GCST90277252
Sterol ester (27:1/22:6) levels	3	68666055	A	C	3.18E-01	6.60E-02	1.71E-02	rs149229417	3_68666055_C_A	0.95	7173	1.44E-06	GCST90277252
Sterol ester (27:1/22:6) levels	3	185542441	T	C	7.63E-02	1.73E-02	3.85E-01	rs113651041	3_185542441_C_T	1.00	7173	9.97E-06	GCST90277252
Sterol ester (27:1/22:6) levels	4	13024335	C	T	-2.48E-01	5.33E-02	2.55E-02	rs116794590	4_13024335_T_C	0.98	7173	3.28E-06	GCST90277252
Sterol ester (27:1/22:6) levels	4	87901015	G	C	2.45E-01	5.48E-02	2.43E-02	rs72657952	4_87901015_C_G	0.97	7173	8.06E-06	GCST90277252
Sterol ester (27:1/22:6) levels	4	91336094	T	G	-1.98E-01	4.25E-02	4.12E-02	rs115647337	4_91336094_G_T	0.97	7173	3.24E-06	GCST90277252
Sterol ester (27:1/22:6) levels	6	24387133	C	T	2.59E-01	4.99E-02	3.00E-02	rs75101588	6_24387133_T_C	0.98	7173	2.23E-07	GCST90277252
Sterol ester (27:1/22:6) levels	6	120046093	A	G	-1.30E-01	2.93E-02	8.88E-02	rs73769662	6_120046093_G_A	1.00	7173	9.33E-06	GCST90277252
Sterol ester (27:1/22:6) levels	7	17680332	T	G	7.45E-02	1.68E-02	5.81E-01	rs2075083	7_17680332_G_T	0.99	7173	9.42E-06	GCST90277252
Sterol ester (27:1/22:6) levels	11	61818856	CT	C	-2.01E-01	1.69E-02	4.06E-01	rs35473591	11_61818856_C_CT	1.00	7173	2.14E-32	GCST90277252
Sterol ester (27:1/22:6) levels	11	68794860	T	C	-1.14E-01	2.34E-02	1.56E-01	rs2229738	11_68794860_C_T	0.94	7173	1.28E-06	GCST90277252
Sterol ester (27:1/22:6) levels	13	107677268	G	A	8.55E-02	1.83E-02	2.84E-01	rs9559135	13_107677268_A_G	1.00	7173	3.17E-06	GCST90277252
Sterol ester (27:1/22:6) levels	14	20010775	T	C	-1.64E-01	3.66E-02	5.52E-02	rs111751059	14_20010775_C_T	0.99	7173	7.18E-06	GCST90277252
Sterol ester (27:1/22:6) levels	14	72571809	T	C	-2.05E-01	3.82E-02	5.30E-02	rs10150075	14_72571809_C_T	0.95	7173	8.96E-08	GCST90277252
Sterol ester (27:1/22:6) levels	15	64865848	A	G	-2.68E-01	5.36E-02	2.42E-02	rs77456635	15_64865848_G_A	1.00	7173	5.68E-07	GCST90277252
Sterol ester (27:1/22:6) levels	15	93239374	A	G	-9.75E-02	2.05E-02	7.19E-01	rs11633878	15_93239374_G_A	0.83	7173	1.97E-06	GCST90277252
Sterol ester (27:1/22:6) levels	15	101196347	T	G	1.99E-01	4.11E-02	4.49E-02	rs117061362	15_101196347_G_T	0.93	7173	1.26E-06	GCST90277252
Sterol ester (27:1/22:6) levels	17	78966695	T	C	2.13E-01	4.50E-02	3.91E-02	rs72852391	17_78966695_C_T	0.91	7173	2.18E-06	GCST90277252

Sterol ester (27:1/22:6) levels	18	48039184	C	T	-9.26E-02	1.72E-02	3.85E-01	rs4072727	18_48039184_T_C	1.00	7173	7.11E-08	GCST90277252
Sterol ester (27:1/22:6) levels	19	44908822	T	C	-2.69E-01	3.72E-02	5.31E-02	rs7412	19_44908822_C_T	1.00	7173	5.61E-13	GCST90277252
Sterol ester (27:1/22:6) levels	20	57125699	A	G	-1.01E-01	2.27E-02	1.60E-01	rs11696517	20_57125699_G_A	0.99	7173	8.19E-06	GCST90277252
Ceramide (d40:1) levels	2	12194447	G	A	7.48E-02	1.69E-02	4.92E-01	rs12477218	2_12194447_A_G	1.00	7117	9.41E-06	GCST90277253
Ceramide (d40:1) levels	2	27508073	C	T	-9.95E-02	1.75E-02	6.51E-01	rs1260326	2_27508073_T_C	1.00	7117	1.40E-08	GCST90277253
Ceramide (d40:1) levels	2	141867363	T	C	1.95E-01	3.99E-02	4.95E-02	rs114910162	2_141867363_C_T	0.95	7117	9.74E-07	GCST90277253
Ceramide (d40:1) levels	2	200379367	G	A	-4.79E-01	1.08E-01	6.78E-03	rs78573518	2_200379367_A_G	0.92	7117	9.82E-06	GCST90277253
Ceramide (d40:1) levels	4	36311616	C	T	8.99E-02	2.00E-02	2.22E-01	rs10025139	4_36311616_T_C	0.99	7117	7.10E-06	GCST90277253
Ceramide (d40:1) levels	4	151996582	T	G	1.14E-01	2.44E-02	8.61E-01	rs6535831	4_151996582_G_T	0.99	7117	2.85E-06	GCST90277253
Ceramide (d40:1) levels	5	76672693	A	G	8.87E-02	1.99E-02	7.73E-01	rs4704352	5_76672693_G_A	1.00	7117	8.80E-06	GCST90277253
Ceramide (d40:1) levels	7	47368432	G	A	-8.83E-02	1.99E-02	7.68E-01	rs2255744	7_47368432_A_G	1.00	7117	9.31E-06	GCST90277253
Ceramide (d40:1) levels	7	95426286	C	T	8.94E-02	1.95E-02	2.41E-01	rs11982486	7_95426286_T_C	1.00	7117	4.77E-06	GCST90277253
Ceramide (d40:1) levels	8	118268727	A	T	1.23E-01	2.23E-02	1.71E-01	rs28505920	8_118268727_T_A	0.96	7117	3.58E-08	GCST90277253
Ceramide (d40:1) levels	9	71460729	T	C	-1.03E-01	2.21E-02	8.23E-01	rs1346910	9_71460729_C_T	1.00	7117	3.46E-06	GCST90277253
Ceramide (d40:1) levels	10	73731455	A	G	-1.11E-01	2.41E-02	1.44E-01	rs56143221	10_73731455_G_A	0.98	7117	4.14E-06	GCST90277253
Ceramide (d40:1) levels	11	117120135	G	A	-8.81E-02	1.86E-02	7.17E-01	rs12366015	11_117120135_A_G	1.00	7117	2.25E-06	GCST90277253
Ceramide (d40:1) levels	12	111854	A	G	-1.59E-01	3.59E-02	5.85E-02	rs11064535	12_111854_G_A	0.97	7117	9.98E-06	GCST90277253
Ceramide (d40:1) levels	12	67485111	C	T	-2.23E-01	4.83E-02	3.26E-02	rs117873718	12_67485111_T_C	0.92	7117	4.03E-06	GCST90277253
Ceramide (d40:1) levels	14	63768838	T	G	1.48E-01	2.52E-02	1.25E-01	rs7157785	14_63768838_G_T	1.00	7117	5.10E-09	GCST90277253
Ceramide (d40:1) levels	14	98057258	G	T	-2.83E-01	6.34E-02	1.78E-02	rs865934	14_98057258_T_G	1.00	7117	8.11E-06	GCST90277253
Ceramide (d40:1) levels	16	89171782	A	G	-7.92E-02	1.67E-02	5.30E-01	rs12927793	16_89171782_G_A	0.99	7117	2.13E-06	GCST90277253
Ceramide (d40:1) levels	17	81124244	C	T	-8.42E-02	1.90E-02	2.66E-01	rs11868222	17_81124244_T_C	0.98	7117	9.25E-06	GCST90277253
Ceramide (d40:1) levels	18	5754635	T	A	-8.52E-02	1.92E-02	2.67E-01	rs1917921	18_5754635_A_T	0.98	7117	8.91E-06	GCST90277253
Ceramide (d40:1) levels	19	19269704	G	A	-2.32E-01	3.79E-02	5.35E-02	rs187429064	19_19269704_A_G	0.95	7117	1.02E-09	GCST90277253
Ceramide (d40:1) levels	19	19346108	T	C	-1.78E-01	3.16E-02	7.57E-02	rs58489806	19_19346108_C_T	1.00	7117	1.72E-08	GCST90277253
Ceramide (d40:1) levels	19	38556464	A	T	8.76E-02	1.95E-02	2.41E-01	rs2945048	19_38556464_T_A	0.99	7117	7.16E-06	GCST90277253
Ceramide (d40:1) levels	20	12982070	G	A	-2.63E-01	1.74E-02	6.61E-01	rs364585	20_12982070_A_G	1.00	7117	1.02E-50	GCST90277253
Ceramide (d40:1) levels	20	13126078	C	T	1.28E-01	2.35E-02	1.46E-01	rs2236124	20_13126078_T_C	0.99	7117	5.60E-08	GCST90277253
Ceramide (d40:1) levels	20	13295543	A	G	1.73E-01	3.75E-02	5.71E-02	rs56406916	20_13295543_G_A	0.90	7117	3.96E-06	GCST90277253
Ceramide (d40:1) levels	20	32510074	C	T	-8.53E-02	1.70E-02	4.67E-01	rs293566	20_32510074_T_C	1.00	7117	5.58E-07	GCST90277253
Ceramide (d40:1) levels	22	46600357	A	G	-1.13E-01	2.15E-02	1.90E-01	rs5767288	22_46600357_G_A	1.00	7117	1.42E-07	GCST90277253
Ceramide (d40:2) levels	1	240877505	G	A	-1.13E-01	2.38E-02	2.09E-01	rs61834063	1_240877505_A_G	0.99	5287	2.10E-06	GCST90277254
Ceramide (d40:2) levels	2	132139262	A	G	-1.98E-01	4.46E-02	5.94E-02	rs114316759	2_132139262_G_A	0.86	5287	8.85E-06	GCST90277254
Ceramide (d40:2) levels	2	167928158	T	C	2.31E-01	4.94E-02	4.18E-02	rs115833524	2_167928158_C_T	0.95	5287	2.92E-06	GCST90277254
Ceramide (d40:2) levels	2	240329826	C	T	2.49E-01	5.33E-02	3.41E-02	rs147060788	2_240329826_T_C	0.98	5287	3.08E-06	GCST90277254
Ceramide (d40:2) levels	4	147474942	G	A	-1.14E-01	2.53E-02	1.89E-01	rs6537481	4_147474942_A_G	1.00	5287	6.59E-06	GCST90277254
Ceramide (d40:2) levels	7	151291137	A	C	4.25E-01	9.50E-02	1.26E-02	rs79320828	7_151291137_C_A	0.92	5287	7.88E-06	GCST90277254
Ceramide (d40:2) levels	8	6166747	A	G	-4.18E-01	8.81E-02	1.31E-02	rs6559102	8_6166747_G_A	0.96	5287	2.12E-06	GCST90277254
Ceramide (d40:2) levels	9	71444815	T	C	1.60E-01	3.55E-02	8.25E-02	rs10869023	9_71444815_C_T	0.99	5287	6.84E-06	GCST90277254
Ceramide (d40:2) levels	9	76271174	A	C	-3.02E-01	6.74E-02	2.23E-02	rs117064201	9_76271174_C_A	0.93	5287	7.66E-06	GCST90277254
Ceramide (d40:2) levels	11	6300702	A	G	2.14E-01	4.32E-02	5.88E-02	rs139456652	11_6300702_G_A	0.88	5287	8.01E-07	GCST90277254
Ceramide (d40:2) levels	11	36075190	T	C	2.67E-01	5.71E-02	3.41E-02	rs117671667	11_36075190_C_T	0.88	5287	2.83E-06	GCST90277254
Ceramide (d40:2) levels	11	97717674	G	A	-8.95E-02	1.97E-02	4.34E-01	rs629047	11_97717674_A_G	0.98	5287	5.44E-06	GCST90277254
Ceramide (d40:2) levels	11	116778201	C	G	-1.40E-01	2.67E-02	8.49E-01	rs964184	11_116778201_G_C	1.00	5287	1.64E-07	GCST90277254
Ceramide (d40:2) levels	13	76834291	C	T	1.45E-01	3.02E-02	1.23E-01	rs17748108	13_76834291_T_C	0.96	5287	1.72E-06	GCST90277254
Ceramide (d40:2) levels	14	63772911	G	T	2.16E-01	2.87E-02	1.24E-01	rs12878001	14_63772911_T_G	1.00	5287	5.67E-14	GCST90277254
Ceramide (d40:2) levels	15	92566992	A	G	-1.35E-01	2.87E-02	1.35E-01	rs111745969	15_92566992_G_A	0.97	5287	2.59E-06	GCST90277254
Ceramide (d40:2) levels	19	1150087	T	C	-4.25E-01	9.49E-02	1.30E-02	rs111984437	19_1150087_C_T	0.88	5287	7.43E-06	GCST90277254
Ceramide (d40:2) levels	19	19269704	G	A	-2.20E-01	4.44E-02	5.35E-02	rs187429064	19_19269704_A_G	0.95	5287	7.10E-07	GCST90277254
Ceramide (d40:2) levels	19	38559208	C	G	1.06E-01	2.24E-02	2.41E-01	rs12974674	19_38559208_G_C	0.99	5287	2.54E-06	GCST90277254
Ceramide (d40:2) levels	19	48629610	C	G	2.50E-01	5.40E-02	3.05E-02	rs61751862	19_48629610_G_C	1.00	5287	3.75E-06	GCST90277254
Ceramide (d40:2) levels	20	12978039	G	A	-2.28E-01	2.02E-02	6.66E-01	rs438568	20_12978039_A_G	1.00	5287	3.66E-29	GCST90277254
Ceramide (d42:1) levels	1	114581582	C	T	5.69E-01	1.28E-01	4.85E-03	rs62621917	1_114581582_T_C	0.89	7174	9.12E-06	GCST90277255
Ceramide (d42:1) levels	2	20948514	C	A	-7.70E-02	1.69E-02	3.93E-01	rs3923037	2_20948514_A_C	1.00	7174	5.55E-06	GCST90277255
Ceramide (d42:1) levels	2	27508073	C	T	-8.88E-02	1.74E-02	6.51E-01	rs1260326	2_27508073_T_C	1.00	7174	3.64E-07	GCST90277255
Ceramide (d42:1) levels	2	62042163	T	A	2.62E-01	5.84E-02	2.14E-02	rs10201832	2_62042163_A_T	0.99	7174	7.31E-06	GCST90277255
Ceramide (d42:1) levels	2	141867363	T	C	1.86E-01	3.97E-02	4.95E-02	rs114910162	2_141867363_C_T	0.95	7174	2.99E-06	GCST90277255

Ceramide (d42:1) levels	2	200379367	G	A	-5.00E-01	1.08E-01	6.78E-03	rs78573518	2_200379367_A_G	0.92	7174	3.38E-06 GCST90277255
Ceramide (d42:1) levels	3	55192986	C	A	1.15E-01	2.59E-02	8.81E-01	rs11130463	3_55192986_A_C	0.99	7174	8.35E-06 GCST90277255
Ceramide (d42:1) levels	4	40237784	G	T	-8.21E-02	1.77E-02	6.55E-01	rs10033592	4_40237784_T_G	0.98	7174	3.36E-06 GCST90277255
Ceramide (d42:1) levels	4	72372827	A	G	2.37E-01	5.10E-02	2.96E-02	rs114822153	4_72372827_G_A	0.93	7174	3.21E-06 GCST90277255
Ceramide (d42:1) levels	4	131441209	C	T	8.38E-02	1.86E-02	2.77E-01	rs72941422	4_131441209_T_C	1.00	7174	6.55E-06 GCST90277255
Ceramide (d42:1) levels	4	175907374	T	C	1.54E-01	3.40E-02	6.41E-02	rs57099041	4_175907374_C_T	1.00	7174	5.50E-06 GCST90277255
Ceramide (d42:1) levels	5	16573000	C	T	2.69E-01	5.77E-02	2.25E-02	rs186176314	5_16573000_T_C	0.96	7174	3.13E-06 GCST90277255
Ceramide (d42:1) levels	7	95390043	A	G	-7.78E-02	1.69E-02	5.60E-01	rs6977389	7_95390043_G_A	1.00	7174	4.10E-06 GCST90277255
Ceramide (d42:1) levels	8	17632332	T	C	-5.38E-01	1.20E-01	5.97E-03	rs112631157	8_17632332_C_T	0.82	7174	7.37E-06 GCST90277255
Ceramide (d42:1) levels	8	118268727	A	T	1.01E-01	2.23E-02	1.71E-01	rs28505920	8_118268727_T_A	0.96	7174	5.57E-06 GCST90277255
Ceramide (d42:1) levels	9	107021609	G	T	-2.94E-01	6.36E-02	1.86E-02	rs117888980	9_107021609_T_G	0.97	7174	3.74E-06 GCST90277255
Ceramide (d42:1) levels	11	116833462	A	C	-1.11E-01	2.16E-02	8.11E-01	rs7123454	11_116833462_C_A	0.99	7174	2.87E-07 GCST90277255
Ceramide (d42:1) levels	12	67513820	A	C	-2.12E-01	4.78E-02	3.50E-02	rs7970512	12_67513820_C_A	0.87	7174	9.54E-06 GCST90277255
Ceramide (d42:1) levels	12	89259912	A	G	-8.60E-02	1.76E-02	3.37E-01	rs11105228	12_89259912_G_A	1.00	7174	1.13E-06 GCST90277255
Ceramide (d42:1) levels	14	33070106	C	G	3.80E-01	8.27E-02	1.24E-02	rs56368930	14_33070106_G_C	0.83	7174	4.44E-06 GCST90277255
Ceramide (d42:1) levels	16	89171782	A	G	-7.85E-02	1.66E-02	5.30E-01	rs12927793	16_89171782_G_A	0.99	7174	2.34E-06 GCST90277255
Ceramide (d42:1) levels	18	71270815	A	G	-2.09E-01	4.67E-02	3.38E-02	rs72954979	18_71270815_G_A	0.96	7174	7.77E-06 GCST90277255
Ceramide (d42:1) levels	19	18958653	A	G	8.41E-02	1.90E-02	3.03E-01	rs7252628	19_18958653_G_A	0.90	7174	9.98E-06 GCST90277255
Ceramide (d42:1) levels	19	19282905	A	G	-1.66E-01	2.24E-02	1.71E-01	rs8100204	19_19282905_G_A	0.97	7174	1.29E-13 GCST90277255
Ceramide (d42:1) levels	19	38556464	A	T	9.26E-02	1.94E-02	2.41E-01	rs2945048	19_38556464_T_A	0.99	7174	1.88E-06 GCST90277255
Ceramide (d42:1) levels	20	12982070	G	A	-2.23E-01	1.74E-02	6.61E-01	rs364585	20_12982070_A_G	1.00	7174	6.11E-37 GCST90277255
Ceramide (d42:1) levels	20	13126078	C	T	1.48E-01	2.34E-02	1.46E-01	rs2236124	20_13126078_T_C	0.99	7174	2.53E-10 GCST90277255
Ceramide (d42:2) levels	2	21002409	T	C	1.03E-01	1.86E-02	7.28E-01	rs1042034	2_21002409_C_T	1.00	7173	3.54E-08 GCST90277256
Ceramide (d42:2) levels	2	27508073	C	T	-9.48E-02	1.74E-02	6.51E-01	rs1260326	2_27508073_T_C	1.00	7173	5.58E-08 GCST90277256
Ceramide (d42:2) levels	2	120863835	C	A	1.30E-01	2.62E-02	1.16E-01	rs55817533	2_120863835_A_C	0.98	7173	7.05E-07 GCST90277256
Ceramide (d42:2) levels	2	137364592	T	C	3.47E-01	7.15E-02	1.43E-02	rs116327692	2_137364592_C_T	0.97	7173	1.28E-06 GCST90277256
Ceramide (d42:2) levels	3	7468297	T	C	1.21E-01	2.62E-02	8.70E-01	rs779714	3_7468297_C_T	0.88	7173	4.03E-06 GCST90277256
Ceramide (d42:2) levels	3	31247248	A	C	-8.67E-02	1.91E-02	2.60E-01	rs9839625	3_31247248_C_A	0.98	7173	5.88E-06 GCST90277256
Ceramide (d42:2) levels	3	54513590	T	C	1.12E-01	2.37E-02	1.60E-01	rs11713732	3_54513590_C_T	0.92	7173	2.12E-06 GCST90277256
Ceramide (d42:2) levels	3	102651475	T	C	3.62E-01	7.91E-02	1.21E-02	rs142985829	3_102651475_C_T	0.93	7173	4.79E-06 GCST90277256
Ceramide (d42:2) levels	4	151996582	T	G	1.21E-01	2.43E-02	8.61E-01	rs6535831	4_151996582_G_T	0.99	7173	6.38E-07 GCST90277256
Ceramide (d42:2) levels	4	168325002	A	G	-2.09E-01	4.61E-02	3.34E-02	rs6837202	4_168325002_G_A	1.00	7173	6.22E-06 GCST90277256
Ceramide (d42:2) levels	6	56110664	G	C	1.16E-01	2.54E-02	1.34E-01	rs7769878	6_56110664_C_G	0.93	7173	5.19E-06 GCST90277256
Ceramide (d42:2) levels	8	118268727	A	T	1.17E-01	2.22E-02	1.71E-01	rs28505920	8_118268727_T_A	0.96	7173	1.66E-07 GCST90277256
Ceramide (d42:2) levels	9	71470570	A	G	1.27E-01	2.86E-02	9.50E-02	rs11507232	9_71470570_G_A	0.99	7173	9.99E-06 GCST90277256
Ceramide (d42:2) levels	10	8406642	T	C	-8.38E-02	1.73E-02	3.82E-01	rs1572595	10_8406642_C_T	0.98	7173	1.24E-06 GCST90277256
Ceramide (d42:2) levels	10	14273933	G	T	1.23E-01	2.75E-02	1.05E-01	rs61836115	10_14273933_T_G	1.00	7173	8.54E-06 GCST90277256
Ceramide (d42:2) levels	10	63118358	G	C	-7.92E-02	1.67E-02	4.61E-01	rs6479877	10_63118358_C_G	0.99	7173	2.17E-06 GCST90277256
Ceramide (d42:2) levels	10	70843134	C	T	-1.32E-01	2.07E-02	2.06E-01	rs12763964	10_70843134_T_C	1.00	7173	1.88E-10 GCST90277256
Ceramide (d42:2) levels	10	130351351	A	G	9.80E-02	2.11E-02	1.91E-01	rs12356523	10_130351351_G_A	0.99	7173	3.31E-06 GCST90277256
Ceramide (d42:2) levels	11	116831407	C	T	-1.25E-01	2.46E-02	8.64E-01	rs5141	11_116831407_T_C	0.99	7173	3.87E-07 GCST90277256
Ceramide (d42:2) levels	12	121000508	T	C	-9.83E-02	1.74E-02	3.60E-01	rs1169306	12_121000508_C_T	1.00	7173	1.51E-08 GCST90277256
Ceramide (d42:2) levels	14	63888972	C	T	8.15E-02	1.82E-02	6.91E-01	rs7493416	14_63888972_T_C	1.00	7173	7.57E-06 GCST90277256
Ceramide (d42:2) levels	16	52635159	C	T	2.64E-01	5.59E-02	2.39E-02	rs79098941	16_52635159_T_C	0.93	7173	2.39E-06 GCST90277256
Ceramide (d42:2) levels	17	37499294	T	C	1.37E-01	2.97E-02	8.76E-02	rs117704733	17_37499294_C_T	0.97	7173	4.39E-06 GCST90277256
Ceramide (d42:2) levels	17	42347515	C	T	8.38E-02	1.80E-02	3.14E-01	rs6503695	17_42347515_T_C	1.00	7173	3.28E-06 GCST90277256
Ceramide (d42:2) levels	19	19255823	T	C	-1.99E-01	3.44E-02	6.24E-02	rs72999033	19_19255823_C_T	1.00	7173	7.12E-09 GCST90277256
Ceramide (d42:2) levels	19	19269704	G	A	-2.88E-01	3.77E-02	5.35E-02	rs187429064	19_19269704_A_G	0.95	7173	2.75E-14 GCST90277256
Ceramide (d42:2) levels	19	38556464	A	T	8.58E-02	1.94E-02	2.41E-01	rs2945048	19_38556464_T_A	0.99	7173	9.80E-06 GCST90277256
Ceramide (d42:2) levels	20	12982070	G	A	-2.65E-01	1.74E-02	6.61E-01	rs364585	20_12982070_A_G	1.00	7173	7.12E-52 GCST90277256
Ceramide (d42:2) levels	20	13126078	C	T	1.70E-01	2.34E-02	1.46E-01	rs2236124	20_13126078_T_C	0.99	7173	4.26E-13 GCST90277256
Ceramide (d42:2) levels	21	28580399	T	C	2.04E-01	4.38E-02	3.92E-02	rs78709083	21_28580399_C_T	0.98	7173	3.19E-06 GCST90277256
Ceramide (d42:2) levels	22	46600357	A	G	-1.41E-01	2.14E-02	1.90E-01	rs5767288	22_46600357_G_A	1.00	7173	4.31E-11 GCST90277256
Cholesterol levels	1	55039974	T	G	-2.14E-01	4.70E-02	3.32E-02	rs11591147	1_55039974_G_T	1.00	7166	5.51E-06 GCST90277257
Cholesterol levels	1	181952433	C	T	-1.39E-01	3.03E-02	8.32E-02	rs12562141	1_181952433_T_C	1.00	7166	4.52E-06 GCST90277257
Cholesterol levels	2	170149397	T	C	-3.65E-01	7.71E-02	1.24E-02	rs180958041	2_170149397_C_T	0.95	7166	2.22E-06 GCST90277257
Cholesterol levels	2	215511513	G	A	8.54E-02	1.91E-02	2.56E-01	rs34462361	2_215511513_A_G	1.00	7166	8.05E-06 GCST90277257

Cholesterol levels	3	172335874	G	A	-1.08E-01	2.33E-02	1.56E-01	rs669552	3_172335874_A_G	0.99	7166	3.40E-06	GCST90277257
Cholesterol levels	3	194964435	A	G	-4.95E-01	1.09E-01	7.64E-03	rs143214193	3_194964435_G_A	0.85	7166	5.35E-06	GCST90277257
Cholesterol levels	4	72366511	T	C	2.53E-01	5.16E-02	2.87E-02	rs11730766	4_72366511_C_T	0.94	7166	9.67E-07	GCST90277257
Cholesterol levels	4	72803111	A	G	2.42E-01	5.39E-02	2.56E-02	rs77645768	4_72803111_G_A	0.97	7166	7.16E-06	GCST90277257
Cholesterol levels	4	73947510	C	A	2.68E-01	5.33E-02	2.53E-02	rs182695896	4_73947510_A_C	0.96	7166	5.02E-07	GCST90277257
Cholesterol levels	5	126190702	A	C	-2.78E-01	6.10E-02	2.01E-02	rs75059546	5_126190702_C_A	0.95	7166	5.45E-06	GCST90277257
Cholesterol levels	7	50678097	T	C	-9.93E-02	2.11E-02	1.98E-01	rs2237478	7_50678097_C_T	1.00	7166	2.52E-06	GCST90277257
Cholesterol levels	8	11644899	A	G	-8.07E-02	1.81E-02	3.22E-01	rs11781607	8_11644899_G_A	1.00	7166	7.89E-06	GCST90277257
Cholesterol levels	9	2958367	G	A	3.03E-01	6.60E-02	1.75E-02	rs112411704	9_2958367_A_G	0.91	7166	4.51E-06	GCST90277257
Cholesterol levels	9	27742537	A	T	8.60E-02	1.79E-02	3.75E-01	rs10812660	9_27742537_T_A	0.93	7166	1.57E-06	GCST90277257
Cholesterol levels	9	96213656	G	A	1.91E-01	4.13E-02	4.40E-02	rs78721266	9_96213656_A_G	0.99	7166	3.90E-06	GCST90277257
Cholesterol levels	9	121135217	G	A	-1.89E-01	4.01E-02	4.74E-02	rs12552499	9_121135217_A_G	0.99	7166	2.50E-06	GCST90277257
Cholesterol levels	10	51918584	A	G	-1.01E-01	2.12E-02	1.89E-01	rs2879630	10_51918584_G_A	1.00	7166	1.80E-06	GCST90277257
Cholesterol levels	10	52240944	G	A	7.61E-02	1.70E-02	5.93E-01	rs7909144	10_52240944_A_G	1.00	7166	7.66E-06	GCST90277257
Cholesterol levels	11	117120135	G	A	-8.24E-02	1.86E-02	7.17E-01	rs12366015	11_117120135_A_G	1.00	7166	9.91E-06	GCST90277257
Cholesterol levels	12	31810895	A	G	2.35E-01	5.13E-02	2.79E-02	rs1271671	12_31810895_G_A	0.98	7166	4.70E-06	GCST90277257
Cholesterol levels	12	31822701	T	C	2.38E-01	5.15E-02	2.78E-02	rs1259751	12_31822701_C_T	0.98	7166	4.02E-06	GCST90277257
Cholesterol levels	13	23150340	C	T	1.24E-01	2.74E-02	1.03E-01	rs75611814	13_23150340_T_C	0.99	7166	5.82E-06	GCST90277257
Cholesterol levels	15	58420774	C	T	8.77E-02	1.82E-02	3.03E-01	rs11858279	15_58420774_T_C	0.98	7166	1.43E-06	GCST90277257
Cholesterol levels	15	58428969	C	G	-7.94E-02	1.73E-02	4.71E-01	rs11857386	15_58428969_G_C	0.94	7166	4.57E-06	GCST90277257
Cholesterol levels	16	14576910	C	T	1.10E-01	2.28E-02	1.60E-01	rs16963800	16_14576910_T_C	0.99	7166	1.36E-06	GCST90277257
Cholesterol levels	16	57423359	G	A	-9.82E-02	2.22E-02	1.72E-01	rs223841	16_57423359_A_G	1.00	7166	9.81E-06	GCST90277257
Cholesterol levels	18	35844265	C	T	-3.03E-01	6.84E-02	1.54E-02	rs141401656	18_35844265_T_C	0.97	7166	9.94E-06	GCST90277257
Cholesterol levels	18	39850559	C	T	-7.68E-02	1.72E-02	3.89E-01	rs17702736	18_39850559_T_C	0.99	7166	8.24E-06	GCST90277257
Cholesterol levels	19	11079868	T	C	-1.61E-01	2.83E-02	9.46E-02	rs118068660	19_11079868_C_T	1.00	7166	1.16E-08	GCST90277257
Cholesterol levels	19	15830645	G	A	-9.00E-02	1.92E-02	2.53E-01	rs73005445	19_15830645_A_G	1.00	7166	2.94E-06	GCST90277257
Cholesterol levels	19	44908684	C	T	1.06E-01	2.15E-02	1.89E-01	rs429358	19_44908684_T_C	1.00	7166	9.26E-07	GCST90277257
Cholesterol levels	19	49823108	C	A	-2.28E-01	4.22E-02	4.23E-02	rs114243848	19_49823108_A_C	0.96	7166	7.17E-08	GCST90277257
Cholesterol levels	20	40665923	T	C	-1.07E-01	2.30E-02	1.55E-01	rs57405165	20_40665923_C_T	0.99	7166	3.52E-06	GCST90277257
Cholesterol levels	20	44413724	T	C	-1.80E-01	3.74E-02	5.24E-02	rs1800961	20_44413724_C_T	1.00	7166	1.57E-06	GCST90277257
Cholesterol levels	22	50075106	T	C	3.95E-01	8.66E-02	1.00E-02	rs76093749	22_50075106_C_T	0.99	7166	5.10E-06	GCST90277257
Diacylglycerol (16:0_18:1) levels	1	77136442	T	C	-8.08E-02	1.82E-02	5.63E-01	rs6699688	1_77136442_C_T	0.99	6293	9.29E-06	GCST90277258
Diacylglycerol (16:0_18:1) levels	1	184416563	G	C	4.17E-01	9.16E-02	1.10E-02	rs78056622	1_184416563_C_G	0.91	6293	5.51E-06	GCST90277258
Diacylglycerol (16:0_18:1) levels	1	230598232	T	C	1.48E-01	3.20E-02	9.25E-02	rs55857911	1_230598232_C_T	0.92	6293	3.66E-06	GCST90277258
Diacylglycerol (16:0_18:1) levels	2	27508073	C	T	-9.93E-02	1.86E-02	6.51E-01	rs1260326	2_27508073_T_C	1.00	6293	9.81E-08	GCST90277258
Diacylglycerol (16:0_18:1) levels	3	10598526	C	G	1.02E-01	2.25E-02	2.13E-01	rs17033194	3_10598526_G_C	0.96	6293	5.69E-06	GCST90277258
Diacylglycerol (16:0_18:1) levels	5	120107843	A	G	3.74E-01	8.42E-02	1.13E-02	rs115473344	5_120107843_G_A	0.98	6293	8.89E-06	GCST90277258
Diacylglycerol (16:0_18:1) levels	7	24401906	C	T	8.17E-02	1.85E-02	6.56E-01	rs93	7_24401906_T_C	1.00	6293	9.99E-06	GCST90277258
Diacylglycerol (16:0_18:1) levels	7	76683164	G	A	1.91E-01	4.25E-02	4.82E-02	rs117905902	7_76683164_A_G	0.98	6293	7.21E-06	GCST90277258
Diacylglycerol (16:0_18:1) levels	7	157355450	A	G	-6.54E-01	1.37E-01	4.75E-03	rs180999317	7_157355450_G_A	0.86	6293	2.04E-06	GCST90277258
Diacylglycerol (16:0_18:1) levels	8	142752432	A	G	-1.22E-01	2.42E-02	1.58E-01	rs11775636	8_142752432_G_A	1.00	6293	4.60E-07	GCST90277258
Diacylglycerol (16:0_18:1) levels	9	2770506	C	T	2.39E-01	5.30E-02	2.99E-02	rs117730598	9_2770506_T_C	0.96	6293	6.90E-06	GCST90277258
Diacylglycerol (16:0_18:1) levels	9	120350676	T	C	2.31E-01	4.77E-02	3.92E-02	rs72753379	9_120350676_C_T	0.95	6293	1.28E-06	GCST90277258
Diacylglycerol (16:0_18:1) levels	9	132470502	G	A	2.59E-01	5.83E-02	2.59E-02	rs111568723	9_132470502_A_G	0.95	6293	8.97E-06	GCST90277258
Diacylglycerol (16:0_18:1) levels	11	116778201	C	G	-1.86E-01	2.46E-02	8.49E-01	rs964184	11_116778201_G_C	1.00	6293	4.04E-14	GCST90277258
Diacylglycerol (16:0_18:1) levels	12	44462630	T	A	3.79E-01	7.63E-02	1.46E-02	rs118099052	12_44462630_A_T	0.93	6293	7.22E-07	GCST90277258
Diacylglycerol (16:0_18:1) levels	12	72431183	C	T	-8.71E-02	1.90E-02	3.48E-01	rs36094040	12_72431183_T_C	0.99	6293	4.64E-06	GCST90277258
Diacylglycerol (16:0_18:1) levels	14	36679967	A	C	-8.48E-02	1.84E-02	6.23E-01	rs1884216	14_36679967_C_A	1.00	6293	4.29E-06	GCST90277258
Diacylglycerol (16:0_18:1) levels	15	40252330	G	C	-1.73E-01	3.79E-02	6.50E-02	rs117614422	15_40252330_C_G	0.93	6293	4.70E-06	GCST90277258
Diacylglycerol (16:0_18:1) levels	15	93154632	T	C	-8.18E-02	1.83E-02	3.78E-01	rs28689929	15_93154632_C_T	0.99	6293	8.05E-06	GCST90277258
Diacylglycerol (16:0_18:1) levels	16	30484331	A	G	-1.23E-01	2.76E-02	1.25E-01	rs9796793	16_30484331_G_A	0.95	6293	7.82E-06	GCST90277258
Diacylglycerol (16:0_18:1) levels	16	78596301	C	T	2.18E-01	4.90E-02	3.53E-02	rs76606887	16_78596301_T_C	0.98	6293	8.68E-06	GCST90277258
Diacylglycerol (16:0_18:1) levels	18	5149977	G	C	-8.28E-02	1.85E-02	6.51E-01	rs447117	18_5149977_C_G	1.00	6293	7.43E-06	GCST90277258
Diacylglycerol (16:0_18:2) levels	1	186849811	T	G	1.78E-01	4.02E-02	5.80E-02	rs76587848	1_186849811_G_T	0.93	6075	9.36E-06	GCST90277259
Diacylglycerol (16:0_18:2) levels	1	230586281	A	G	9.81E-02	1.98E-02	3.20E-01	rs6687291	1_230586281_G_A	0.97	6075	7.54E-07	GCST90277259
Diacylglycerol (16:0_18:2) levels	1	230712956	T	C	-1.08E-01	2.29E-02	1.96E-01	rs2004776	1_230712956_C_T	1.00	6075	2.83E-06	GCST90277259
Diacylglycerol (16:0_18:2) levels	2	27508073	C	T	-1.02E-01	1.89E-02	6.51E-01	rs1260326	2_27508073_T_C	1.00	6075	6.21E-08	GCST90277259

Diacylglycerol (16:0_18:2) levels	2	42290376	A	C	-9.13E-02	2.06E-02	2.65E-01	rs7569544	2_42290376_C_A	1.00	6075	9.64E-06	GCST90277259
Diacylglycerol (16:0_18:2) levels	2	42953102	T	C	-6.30E-01	1.34E-01	4.92E-03	rs78504564	2_42953102_C_T	0.95	6075	2.49E-06	GCST90277259
Diacylglycerol (16:0_18:2) levels	2	220215369	A	G	3.08E-01	6.38E-02	2.14E-02	rs140314539	2_220215369_G_A	0.98	6075	1.38E-06	GCST90277259
Diacylglycerol (16:0_18:2) levels	3	55269076	G	A	2.93E-01	6.54E-02	1.98E-02	rs149992851	3_55269076_A_G	0.98	6075	7.72E-06	GCST90277259
Diacylglycerol (16:0_18:2) levels	3	188792426	T	G	-1.92E-01	4.15E-02	5.28E-02	rs6810359	3_188792426_G_T	0.95	6075	3.61E-06	GCST90277259
Diacylglycerol (16:0_18:2) levels	5	149755706	T	C	-1.14E-01	2.30E-02	1.96E-01	rs34606692	5_149755706_C_T	1.00	6075	7.38E-07	GCST90277259
Diacylglycerol (16:0_18:2) levels	6	28352897	T	C	-1.16E-01	2.34E-02	1.83E-01	rs758398	6_28352897_C_T	1.00	6075	7.42E-07	GCST90277259
Diacylglycerol (16:0_18:2) levels	6	64409802	A	C	9.35E-02	1.95E-02	3.24E-01	rs11756450	6_64409802_C_A	0.99	6075	1.61E-06	GCST90277259
Diacylglycerol (16:0_18:2) levels	7	73612048	T	C	-1.34E-01	2.61E-02	1.41E-01	rs17145750	7_73612048_C_T	1.00	6075	3.14E-07	GCST90277259
Diacylglycerol (16:0_18:2) levels	8	130543708	C	T	6.69E-01	1.40E-01	4.57E-03	rs111934503	8_130543708_T_C	0.92	6075	1.66E-06	GCST90277259
Diacylglycerol (16:0_18:2) levels	9	8556190	G	T	-1.73E-01	3.89E-02	5.96E-02	rs34792804	9_8556190_T_G	0.97	6075	8.70E-06	GCST90277259
Diacylglycerol (16:0_18:2) levels	9	71279650	G	C	-2.81E-01	6.00E-02	2.42E-02	rs148163011	9_71279650_C_G	0.97	6075	2.83E-06	GCST90277259
Diacylglycerol (16:0_18:2) levels	9	136257994	G	A	-2.53E-01	5.27E-02	3.60E-02	rs112122274	9_136257994_A_G	0.85	6075	1.68E-06	GCST90277259
Diacylglycerol (16:0_18:2) levels	11	23915220	A	C	9.17E-02	2.03E-02	7.17E-01	rs4262724	11_23915220_C_A	1.00	6075	6.46E-06	GCST90277259
Diacylglycerol (16:0_18:2) levels	11	116778201	C	G	-2.25E-01	2.48E-02	8.49E-01	rs964184	11_116778201_G_C	1.00	6075	1.64E-19	GCST90277259
Diacylglycerol (16:0_18:2) levels	12	19924761	A	C	-4.04E-01	8.44E-02	1.17E-02	rs57747810	12_19924761_C_A	0.95	6075	1.70E-06	GCST90277259
Diacylglycerol (16:0_18:2) levels	12	44386579	C	T	3.41E-01	7.12E-02	1.78E-02	rs11182480	12_44386579_T_C	0.93	6075	1.78E-06	GCST90277259
Diacylglycerol (16:0_18:2) levels	15	38675336	C	G	-1.07E-01	2.32E-02	1.92E-01	rs7165355	15_38675336_G_C	1.00	6075	3.92E-06	GCST90277259
Diacylglycerol (16:0_18:2) levels	15	97687850	C	T	8.28E-02	1.85E-02	4.51E-01	rs1032956	15_97687850_T_C	0.98	6075	7.88E-06	GCST90277259
Diacylglycerol (16:0_18:2) levels	16	11584433	A	T	1.11E-01	2.49E-02	1.65E-01	rs2868424	16_11584433_T_A	0.97	6075	8.47E-06	GCST90277259
Diacylglycerol (16:0_18:2) levels	17	1623049	C	T	1.07E-01	2.32E-02	1.96E-01	rs35986054	17_1623049_T_C	0.98	6075	4.30E-06	GCST90277259
Diacylglycerol (16:0_18:2) levels	19	3057222	C	T	1.21E-01	2.64E-02	1.51E-01	rs4806901	19_3057222_T_C	0.92	6075	4.18E-06	GCST90277259
Diacylglycerol (16:0_18:2) levels	19	41202334	T	C	8.20E-02	1.82E-02	5.32E-01	rs7254036	19_41202334_C_T	0.99	6075	7.01E-06	GCST90277259
Diacylglycerol (16:0_18:2) levels	21	44845081	T	C	-5.80E-01	1.25E-01	4.71E-03	rs144838649	21_44845081_C_T	0.96	6075	3.52E-06	GCST90277259
Diacylglycerol (16:1_18:1) levels	1	81671654	G	A	-1.86E-01	4.19E-02	4.80E-02	rs143808674	1_81671654_A_G	0.99	6215	8.76E-06	GCST90277260
Diacylglycerol (16:1_18:1) levels	2	27508073	C	T	-1.12E-01	1.87E-02	6.51E-01	rs1260326	2_27508073_T_C	1.00	6215	2.15E-09	GCST90277260
Diacylglycerol (16:1_18:1) levels	4	124943164	T	C	1.06E-01	2.37E-02	1.81E-01	rs10022344	4_124943164_C_T	0.98	6215	8.41E-06	GCST90277260
Diacylglycerol (16:1_18:1) levels	6	31276082	T	C	-1.07E-01	2.18E-02	2.08E-01	rs2524075	6_31276082_C_T	1.00	6215	9.22E-07	GCST90277260
Diacylglycerol (16:1_18:1) levels	6	43900875	G	A	2.61E-01	5.44E-02	3.06E-02	rs2182380	6_43900875_A_G	0.92	6215	1.65E-06	GCST90277260
Diacylglycerol (16:1_18:1) levels	7	36495127	A	G	2.58E-01	5.53E-02	3.06E-02	rs77134026	7_36495127_G_A	0.85	6215	3.20E-06	GCST90277260
Diacylglycerol (16:1_18:1) levels	8	142752432	A	G	-1.13E-01	2.43E-02	1.58E-01	rs11775636	8_142752432_G_A	1.00	6215	3.05E-06	GCST90277260
Diacylglycerol (16:1_18:1) levels	9	23713682	C	T	1.02E-01	2.24E-02	2.03E-01	rs3829088	9_23713682_T_C	1.00	6215	5.65E-06	GCST90277260
Diacylglycerol (16:1_18:1) levels	9	136257994	G	A	-2.60E-01	5.25E-02	3.60E-02	rs112122274	9_136257994_A_G	0.85	6215	7.58E-07	GCST90277260
Diacylglycerol (16:1_18:1) levels	10	62375635	CA	C	-2.85E-01	6.11E-02	2.27E-02	rs35162925	10_62375635_C_CA	1.00	6215	3.12E-06	GCST90277260
Diacylglycerol (16:1_18:1) levels	11	116778201	C	G	-1.64E-01	2.47E-02	8.49E-01	rs964184	11_116778201_G_C	1.00	6215	3.17E-11	GCST90277260
Diacylglycerol (16:1_18:1) levels	12	44386579	C	T	3.45E-01	6.96E-02	1.78E-02	rs11182480	12_44386579_T_C	0.93	6215	7.16E-07	GCST90277260
Diacylglycerol (16:1_18:1) levels	12	72331215	T	G	-8.86E-02	1.83E-02	4.23E-01	rs7955732	12_72331215_G_T	1.00	6215	1.24E-06	GCST90277260
Diacylglycerol (16:1_18:1) levels	12	96298692	C	A	-2.14E-01	4.56E-02	4.14E-02	rs111750990	12_96298692_A_C	0.96	6215	2.65E-06	GCST90277260
Diacylglycerol (16:1_18:1) levels	15	39983732	A	G	-2.78E-01	6.22E-02	2.09E-02	rs12050879	15_39983732_G_A	1.00	6215	7.99E-06	GCST90277260
Diacylglycerol (16:1_18:1) levels	16	71309806	A	G	-9.31E-02	2.11E-02	2.37E-01	rs4419068	16_71309806_G_A	1.00	6215	9.89E-06	GCST90277260
Diacylglycerol (16:1_18:1) levels	17	66540315	C	T	8.07E-02	1.82E-02	4.28E-01	rs908150	17_66540315_T_C	1.00	6215	9.56E-06	GCST90277260
Diacylglycerol (16:1_18:1) levels	18	30887893	G	C	-2.13E-01	4.16E-02	5.09E-02	rs10502551	18_30887893_C_G	0.96	6215	3.21E-07	GCST90277260
Diacylglycerol (16:1_18:1) levels	18	78352143	A	C	2.67E-01	6.01E-02	2.42E-02	rs147782497	18_78352143_C_A	0.94	6215	9.11E-06	GCST90277260
Diacylglycerol (16:1_18:1) levels	19	30685856	A	T	-2.66E-01	5.71E-02	2.57E-02	rs140283718	19_30685856_T_A	0.92	6215	3.29E-06	GCST90277260
Diacylglycerol (16:1_18:1) levels	19	52961172	C	T	2.06E-01	4.59E-02	4.35E-02	rs78263224	19_52961172_T_C	0.92	6215	7.61E-06	GCST90277260
Diacylglycerol (16:1_18:1) levels	22	40189878	A	C	3.84E-01	8.53E-02	1.18E-02	rs117771237	22_40189878_C_A	0.95	6215	7.02E-06	GCST90277260
Diacylglycerol (18:1_18:1) levels	1	220805303	T	C	1.31E-01	2.39E-02	1.52E-01	rs17596144	1_220805303_C_T	0.99	6884	4.26E-08	GCST90277261
Diacylglycerol (18:1_18:1) levels	2	21002409	T	C	1.04E-01	1.90E-02	7.28E-01	rs1042034	2_21002409_C_T	1.00	6884	4.97E-08	GCST90277261
Diacylglycerol (18:1_18:1) levels	2	27508073	C	T	-1.23E-01	1.78E-02	6.51E-01	rs1260326	2_27508073_T_C	1.00	6884	5.88E-12	GCST90277261
Diacylglycerol (18:1_18:1) levels	2	36549412	T	G	-2.46E-01	5.21E-02	2.77E-02	rs3770813	2_36549412_G_T	1.00	6884	2.37E-06	GCST90277261
Diacylglycerol (18:1_18:1) levels	2	127068957	G	A	-8.35E-02	1.88E-02	2.91E-01	rs1060743	2_127068957_A_G	1.00	6884	8.86E-06	GCST90277261
Diacylglycerol (18:1_18:1) levels	2	128427075	G	A	1.60E-01	3.26E-02	7.45E-02	rs76950187	2_128427075_A_G	0.99	6884	9.58E-07	GCST90277261
Diacylglycerol (18:1_18:1) levels	2	212010621	G	A	-9.83E-02	1.82E-02	6.76E-01	rs10169217	2_212010621_A_G	1.00	6884	7.18E-08	GCST90277261
Diacylglycerol (18:1_18:1) levels	3	87298641	A	G	-1.86E-01	3.98E-02	4.88E-02	rs116011373	3_87298641_G_A	0.96	6884	3.06E-06	GCST90277261
Diacylglycerol (18:1_18:1) levels	3	132015999	G	A	-1.07E-01	2.26E-02	1.65E-01	rs9865586	3_132015999_A_G	0.99	6884	2.33E-06	GCST90277261
Diacylglycerol (18:1_18:1) levels	4	4999649	C	T	1.07E-01	2.15E-02	2.14E-01	rs62291573	4_4999649_T_C	0.94	6884	6.91E-07	GCST90277261
Diacylglycerol (18:1_18:1) levels	4	62344980	T	C	-3.37E-01	7.18E-02	1.43E-02	rs116796527	4_62344980_C_T	0.99	6884	2.68E-06	GCST90277261

Diacylglycerol (18:1_18:1) levels	7	6295952	T	A	9.46E-02	2.04E-02	2.32E-01	rs7782961	7_6295952_A_T	0.97	6884	3.51E-06	GCST90277261
Diacylglycerol (18:1_18:1) levels	7	73597712	A	G	-1.44E-01	2.59E-02	1.22E-01	rs35332062	7_73597712_G_A	1.00	6884	3.11E-08	GCST90277261
Diacylglycerol (18:1_18:1) levels	8	19956018	G	A	2.81E-01	5.80E-02	2.27E-02	rs268	8_19956018_A_G	0.97	6884	1.26E-06	GCST90277261
Diacylglycerol (18:1_18:1) levels	8	125469835	G	A	-7.58E-02	1.71E-02	5.38E-01	rs2954021	8_125469835_A_G	1.00	6884	9.05E-06	GCST90277261
Diacylglycerol (18:1_18:1) levels	11	116618319	G	A	1.05E-01	2.31E-02	1.66E-01	rs12365864	11_116618319_A_G	0.97	6884	5.55E-06	GCST90277261
Diacylglycerol (18:1_18:1) levels	11	116778201	C	G	-2.64E-01	2.34E-02	8.49E-01	rs964184	11_116778201_G_C	1.00	6884	3.74E-29	GCST90277261
Diacylglycerol (18:1_18:1) levels	12	72431183	C	T	-8.29E-02	1.81E-02	3.48E-01	rs36094040	12_72431183_T_C	0.99	6884	4.96E-06	GCST90277261
Diacylglycerol (18:1_18:1) levels	12	96850670	C	A	-3.93E-01	8.07E-02	1.12E-02	rs139500046	12_96850670_A_C	0.99	6884	1.13E-06	GCST90277261
Diacylglycerol (18:1_18:1) levels	14	58138012	C	T	2.99E-01	6.71E-02	1.73E-02	rs137871178	14_58138012_T_C	0.96	6884	8.42E-06	GCST90277261
Diacylglycerol (18:1_18:1) levels	16	82018393	C	T	3.25E-01	7.03E-02	1.74E-02	rs62046303	16_82018393_T_C	0.90	6884	3.80E-06	GCST90277261
Diacylglycerol (18:1_18:1) levels	18	78352143	A	C	2.59E-01	5.65E-02	2.42E-02	rs147782497	18_78352143_C_A	0.94	6884	4.75E-06	GCST90277261
Diacylglycerol (18:1_18:1) levels	19	44892962	T	C	9.44E-02	2.05E-02	2.23E-01	rs157582	19_44892962_C_T	1.00	6884	4.43E-06	GCST90277261
Diacylglycerol (18:1_18:2) levels	1	150251390	A	G	-1.21E-01	2.61E-02	1.29E-01	rs72694990	1_150251390_G_A	0.99	6613	4.00E-06	GCST90277262
Diacylglycerol (18:1_18:2) levels	1	220806736	A	C	1.12E-01	2.24E-02	1.85E-01	rs34621709	1_220806736_C_A	1.00	6613	6.40E-07	GCST90277262
Diacylglycerol (18:1_18:2) levels	1	230156121	C	T	-1.12E-01	2.04E-02	7.69E-01	rs2352723	1_230156121_T_C	1.00	6613	3.76E-08	GCST90277262
Diacylglycerol (18:1_18:2) levels	2	21002409	T	C	9.34E-02	1.95E-02	7.28E-01	rs1042034	2_21002409_C_T	1.00	6613	1.65E-06	GCST90277262
Diacylglycerol (18:1_18:2) levels	2	27508073	C	T	-1.29E-01	1.81E-02	6.51E-01	rs1260326	2_27508073_T_C	1.00	6613	9.98E-13	GCST90277262
Diacylglycerol (18:1_18:2) levels	2	111060064	A	G	1.06E-01	2.29E-02	1.77E-01	rs11684166	2_111060064_G_A	0.99	6613	3.49E-06	GCST90277262
Diacylglycerol (18:1_18:2) levels	2	212078809	G	A	-8.19E-02	1.84E-02	6.60E-01	rs9973939	2_212078809_A_G	1.00	6613	8.26E-06	GCST90277262
Diacylglycerol (18:1_18:2) levels	3	87298641	A	G	-1.82E-01	4.07E-02	4.88E-02	rs116011373	3_87298641_G_A	0.96	6613	7.38E-06	GCST90277262
Diacylglycerol (18:1_18:2) levels	3	171536246	A	G	-4.99E-01	1.11E-01	7.31E-03	rs57500102	3_171536246_G_A	0.92	6613	7.12E-06	GCST90277262
Diacylglycerol (18:1_18:2) levels	3	176180291	C	T	1.49E-01	3.33E-02	7.76E-02	rs114444157	3_176180291_T_C	0.95	6613	7.23E-06	GCST90277262
Diacylglycerol (18:1_18:2) levels	4	4999649	C	T	1.00E-01	2.19E-02	2.14E-01	rs62291573	4_4999649_T_C	0.94	6613	4.75E-06	GCST90277262
Diacylglycerol (18:1_18:2) levels	4	141672748	T	C	-3.01E-01	6.04E-02	2.19E-02	rs17007519	4_141672748_C_T	0.96	6613	6.24E-07	GCST90277262
Diacylglycerol (18:1_18:2) levels	4	163302506	T	G	7.83E-02	1.75E-02	5.32E-01	rs4691907	4_163302506_G_T	1.00	6613	7.60E-06	GCST90277262
Diacylglycerol (18:1_18:2) levels	6	14146172	G	A	-1.12E-01	2.47E-02	1.47E-01	rs7745829	6_14146172_A_G	0.99	6613	5.66E-06	GCST90277262
Diacylglycerol (18:1_18:2) levels	6	28292472	A	T	-9.77E-02	2.09E-02	2.21E-01	rs1233660	6_28292472_T_A	1.00	6613	3.00E-06	GCST90277262
Diacylglycerol (18:1_18:2) levels	6	64441454	A	G	1.29E-01	2.63E-02	1.28E-01	rs75008187	6_64441454_G_A	1.00	6613	9.80E-07	GCST90277262
Diacylglycerol (18:1_18:2) levels	6	154880792	G	C	1.55E-01	3.31E-02	7.40E-02	rs141116593	6_154880792_C_G	0.99	6613	2.82E-06	GCST90277262
Diacylglycerol (18:1_18:2) levels	7	6295952	T	A	9.18E-02	2.08E-02	2.32E-01	rs7782961	7_6295952_A_T	0.97	6613	9.94E-06	GCST90277262
Diacylglycerol (18:1_18:2) levels	7	73598455	G	A	-1.72E-01	2.65E-02	1.22E-01	rs79624003	7_73598455_A_G	1.00	6613	8.98E-11	GCST90277262
Diacylglycerol (18:1_18:2) levels	8	19956018	G	A	3.36E-01	5.82E-02	2.27E-02	rs268	8_19956018_A_G	0.97	6613	8.26E-09	GCST90277262
Diacylglycerol (18:1_18:2) levels	8	43650502	A	G	1.28E-01	2.79E-02	1.14E-01	rs75596627	8_43650502_G_A	0.95	6613	4.62E-06	GCST90277262
Diacylglycerol (18:1_18:2) levels	10	5793091	T	C	-6.04E-01	1.31E-01	4.57E-03	rs117416257	10_5793091_C_T	0.99	6613	3.83E-06	GCST90277262
Diacylglycerol (18:1_18:2) levels	10	88606986	A	G	3.49E-01	7.84E-02	1.29E-02	rs75082203	10_88606986_G_A	0.98	6613	8.40E-06	GCST90277262
Diacylglycerol (18:1_18:2) levels	10	112142696	T	C	-9.02E-02	1.90E-02	2.97E-01	rs72836628	10_112142696_C_T	1.00	6613	2.11E-06	GCST90277262
Diacylglycerol (18:1_18:2) levels	11	17897155	T	G	-6.89E-01	1.54E-01	3.22E-03	rs138578034	11_17897155_G_T	0.94	6613	7.80E-06	GCST90277262
Diacylglycerol (18:1_18:2) levels	11	32530936	G	T	-4.77E-01	1.05E-01	7.47E-03	rs181215046	11_32530936_T_G	0.96	6613	5.32E-06	GCST90277262
Diacylglycerol (18:1_18:2) levels	11	62717059	T	C	-5.59E-01	1.25E-01	5.02E-03	rs142868058	11_62717059_C_T	0.97	6613	8.45E-06	GCST90277262
Diacylglycerol (18:1_18:2) levels	11	116618319	G	A	1.24E-01	2.35E-02	1.66E-01	rs12365864	11_116618319_A_G	0.97	6613	1.58E-07	GCST90277262
Diacylglycerol (18:1_18:2) levels	11	116778201	C	G	-2.91E-01	2.37E-02	8.49E-01	rs964184	11_116778201_G_C	1.00	6613	3.26E-34	GCST90277262
Diacylglycerol (18:1_18:2) levels	12	129796667	A	G	-8.71E-02	1.88E-02	6.83E-01	rs10773708	12_129796667_G_A	1.00	6613	3.71E-06	GCST90277262
Diacylglycerol (18:1_18:2) levels	16	23267056	A	C	-2.51E-01	5.28E-02	3.10E-02	rs55788915	16_23267056_C_A	0.93	6613	1.92E-06	GCST90277262
Diacylglycerol (18:1_18:2) levels	18	62009100	C	T	-8.62E-02	1.89E-02	6.94E-01	rs7239207	18_62009100_T_C	1.00	6613	4.99E-06	GCST90277262
Diacylglycerol (18:1_18:2) levels	19	20106360	T	C	-2.00E-01	4.18E-02	4.53E-02	rs4608457	19_20106360_C_T	0.97	6613	1.81E-06	GCST90277262
Diacylglycerol (18:1_18:2) levels	19	33144357	C	A	8.73E-02	1.91E-02	2.78E-01	rs11670344	19_33144357_A_C	1.00	6613	5.24E-06	GCST90277262
Diacylglycerol (18:1_18:2) levels	19	44892962	T	C	1.18E-01	2.09E-02	2.23E-01	rs157582	19_44892962_C_T	1.00	6613	1.66E-08	GCST90277262
Diacylglycerol (18:1_18:2) levels	19	44971423	T	C	9.62E-02	1.91E-02	3.07E-01	rs11668758	19_44971423_C_T	0.99	6613	4.54E-07	GCST90277262
Phosphatidylcholine (O-18:1_18:2) levels	1	107643461	C	T	-1.12E-01	2.37E-02	1.62E-01	rs6583035	1_107643461_T_C	1.00	6572	2.46E-06	GCST90277263
Phosphatidylcholine (O-18:1_18:2) levels	1	186241818	C	T	2.19E-01	4.86E-02	3.25E-02	rs141217537	1_186241818_T_C	0.96	6572	6.59E-06	GCST90277263
Phosphatidylcholine (O-18:1_18:2) levels	1	196932199	G	A	2.31E-01	5.02E-02	3.18E-02	rs55865364	1_196932199_A_G	0.97	6572	4.22E-06	GCST90277263
Phosphatidylcholine (O-18:1_18:2) levels	2	27508073	C	T	-8.98E-02	1.82E-02	6.51E-01	rs1260326	2_27508073_T_C	1.00	6572	8.83E-07	GCST90277263
Phosphatidylcholine (O-18:1_18:2) levels	2	36315434	A	C	4.90E-01	9.60E-02	7.95E-03	rs7599400	2_36315434_C_A	0.99	6572	3.46E-07	GCST90277263
Phosphatidylcholine (O-18:1_18:2) levels	3	87298641	A	G	-1.91E-01	4.09E-02	4.88E-02	rs116011373	3_87298641_G_A	0.96	6572	3.06E-06	GCST90277263
Phosphatidylcholine (O-18:1_18:2) levels	3	133895761	T	C	1.12E-01	2.32E-02	1.73E-01	rs117518401	3_133895761_C_T	0.98	6572	1.48E-06	GCST90277263
Phosphatidylcholine (O-18:1_18:2) levels	4	141972167	G	A	-2.57E-01	5.61E-02	2.65E-02	rs114441702	4_141972167_A_G	0.93	6572	4.52E-06	GCST90277263
Phosphatidylcholine (O-18:1_18:2) levels	5	153405520	A	G	-1.23E-01	2.68E-02	1.22E-01	rs77442856	5_153405520_G_A	0.98	6572	4.21E-06	GCST90277263

Phosphatidylcholine (O-18:1_18:2) levels	5	178403016	A	G	3.18E-01	7.09E-02	1.61E-02	rs112374014	5_178403016_G_A	0.96	6572	7.33E-06	GCST90277263
Phosphatidylcholine (O-18:1_18:2) levels	7	10662626	C	G	9.56E-02	2.02E-02	2.44E-01	rs6958772	7_10662626_G_C	1.00	6572	2.27E-06	GCST90277263
Phosphatidylcholine (O-18:1_18:2) levels	7	74460551	A	G	-8.08E-02	1.73E-02	5.03E-01	rs13237829	7_74460551_G_A	1.00	6572	3.03E-06	GCST90277263
Phosphatidylcholine (O-18:1_18:2) levels	8	19956018	G	A	2.81E-01	5.94E-02	2.27E-02	rs268	8_19956018_A_G	0.97	6572	2.39E-06	GCST90277263
Phosphatidylcholine (O-18:1_18:2) levels	8	32454379	T	C	2.35E-01	4.91E-02	3.31E-02	rs180761622	8_32454379_C_T	1.00	6572	1.73E-06	GCST90277263
Phosphatidylcholine (O-18:1_18:2) levels	10	24164242	T	C	-1.89E-01	4.21E-02	4.93E-02	rs75224949	10_24164242_C_T	0.91	6572	7.27E-06	GCST90277263
Phosphatidylcholine (O-18:1_18:2) levels	10	110816937	C	T	-1.42E-01	2.76E-02	8.74E-01	rs10749053	10_110816937_T_C	0.92	6572	2.57E-07	GCST90277263
Phosphatidylcholine (O-18:1_18:2) levels	11	116778201	C	G	-1.71E-01	2.40E-02	8.49E-01	rs964184	11_116778201_G_C	1.00	6572	1.43E-12	GCST90277263
Phosphatidylcholine (O-18:1_18:2) levels	13	20969363	C	T	1.01E-01	2.26E-02	1.85E-01	rs61950149	13_20969363_T_C	0.97	6572	7.99E-06	GCST90277263
Phosphatidylcholine (O-18:1_18:2) levels	16	23263670	A	G	-1.65E-01	3.39E-02	7.70E-02	rs112075774	16_23263670_G_A	0.93	6572	1.21E-06	GCST90277263
Phosphatidylcholine (O-18:1_18:2) levels	18	78520543	G	C	1.86E-01	4.11E-02	4.58E-02	rs151145497	18_78520543_C_G	0.99	6572	6.35E-06	GCST90277263
Phosphatidylcholine (O-18:1_18:2) levels	19	20106360	T	C	-1.93E-01	4.27E-02	4.53E-02	rs4608457	19_20106360_C_T	0.97	6572	6.51E-06	GCST90277263
Phosphatidylcholine (O-18:1_18:2) levels	19	54419778	A	G	8.60E-02	1.89E-02	3.53E-01	rs12463051	19_54419778_G_A	0.93	6572	5.58E-06	GCST90277263
Phosphatidylcholine (O-18:1_18:2) levels	20	22581333	CTT	CT	-2.18E-01	4.76E-02	3.53E-02	rs112021763	20_22581333_CT_CTT	0.99	6572	4.92E-06	GCST90277263
Phosphatidylcholine (O-18:1_20:3) levels	1	39937698	T	G	1.34E-01	1.93E-02	2.50E-01	rs7529794	1_39937698_G_T	0.99	7174	4.32E-12	GCST90277264
Phosphatidylcholine (O-18:1_20:3) levels	1	98498507	C	T	8.18E-02	1.78E-02	6.30E-01	rs571344	1_98498507_T_C	0.97	7174	4.23E-06	GCST90277264
Phosphatidylcholine (O-18:1_20:3) levels	1	163474114	A	G	-2.46E-01	5.08E-02	2.96E-02	rs12116819	1_163474114_G_A	0.94	7174	1.24E-06	GCST90277264
Phosphatidylcholine (O-18:1_20:3) levels	2	27508073	C	T	-9.90E-02	1.75E-02	6.51E-01	rs1260326	2_27508073_T_C	1.00	7174	1.56E-08	GCST90277264
Phosphatidylcholine (O-18:1_20:3) levels	2	155599766	T	C	7.50E-02	1.68E-02	5.56E-01	rs2691773	2_155599766_C_T	1.00	7174	8.10E-06	GCST90277264
Phosphatidylcholine (O-18:1_20:3) levels	3	4762993	A	G	-4.42E-01	9.31E-02	8.73E-03	rs62231613	3_4762993_G_A	0.91	7174	2.10E-06	GCST90277264
Phosphatidylcholine (O-18:1_20:3) levels	3	32353724	A	C	8.25E-02	1.82E-02	7.08E-01	rs4955144	3_32353724_C_A	0.99	7174	5.91E-06	GCST90277264
Phosphatidylcholine (O-18:1_20:3) levels	3	77008436	T	G	2.56E-01	5.45E-02	2.45E-02	rs76530357	3_77008436_G_T	0.96	7174	2.70E-06	GCST90277264
Phosphatidylcholine (O-18:1_20:3) levels	3	120300479	T	C	-9.41E-02	2.07E-02	2.03E-01	rs34782816	3_120300479_C_T	0.99	7174	5.65E-06	GCST90277264
Phosphatidylcholine (O-18:1_20:3) levels	4	80232181	C	T	-1.26E-01	2.59E-02	1.17E-01	rs75563920	4_80232181_T_C	1.00	7174	1.29E-06	GCST90277264
Phosphatidylcholine (O-18:1_20:3) levels	4	152971920	G	A	-9.74E-02	2.16E-02	1.89E-01	rs17029364	4_152971920_A_G	0.99	7174	6.68E-06	GCST90277264
Phosphatidylcholine (O-18:1_20:3) levels	5	117857598	G	A	-2.97E-01	5.92E-02	2.11E-02	rs75668753	5_117857598_A_G	0.96	7174	5.49E-07	GCST90277264
Phosphatidylcholine (O-18:1_20:3) levels	6	7298054	A	G	7.97E-02	1.72E-02	3.91E-01	rs2714309	6_7298054_G_A	1.00	7174	3.69E-06	GCST90277264
Phosphatidylcholine (O-18:1_20:3) levels	7	95573476	T	C	-3.40E-01	7.58E-02	1.27E-02	rs76309137	7_95573476_C_T	0.99	7174	7.34E-06	GCST90277264
Phosphatidylcholine (O-18:1_20:3) levels	8	18126210	T	C	2.84E-01	5.90E-02	2.17E-02	rs73200277	8_18126210_C_T	0.95	7174	1.45E-06	GCST90277264
Phosphatidylcholine (O-18:1_20:3) levels	8	79831072	A	G	2.38E-01	5.28E-02	2.64E-02	rs73693512	8_79831072_G_A	0.99	7174	6.97E-06	GCST90277264
Phosphatidylcholine (O-18:1_20:3) levels	8	100059985	G	C	2.77E-01	6.21E-02	1.97E-02	rs117001547	8_100059985_C_G	0.95	7174	8.34E-06	GCST90277264
Phosphatidylcholine (O-18:1_20:3) levels	10	20803487	T	C	1.61E-01	3.61E-02	5.76E-02	rs117646116	10_20803487_C_T	0.98	7174	8.59E-06	GCST90277264
Phosphatidylcholine (O-18:1_20:3) levels	11	40267436	A	C	-5.46E-01	1.19E-01	5.67E-03	rs191695863	11_40267436_C_A	0.88	7174	4.41E-06	GCST90277264
Phosphatidylcholine (O-18:1_20:3) levels	12	17851399	C	A	-7.33E-01	1.63E-01	3.08E-03	rs149105878	12_17851399_A_C	0.83	7174	7.12E-06	GCST90277264
Phosphatidylcholine (O-18:1_20:3) levels	12	26619127	G	A	-1.10E-01	2.41E-02	1.42E-01	rs17477122	12_26619127_A_G	1.00	7174	4.77E-06	GCST90277264
Phosphatidylcholine (O-18:1_20:3) levels	12	32302096	C	T	-8.79E-02	1.94E-02	2.50E-01	rs11051913	12_32302096_T_C	0.99	7174	6.29E-06	GCST90277264
Phosphatidylcholine (O-18:1_20:3) levels	13	81460606	A	G	-7.57E-02	1.67E-02	4.83E-01	rs9565669	13_81460606_G_A	1.00	7174	6.32E-06	GCST90277264
Phosphatidylcholine (O-18:1_20:3) levels	13	111120067	C	T	1.10E-01	2.44E-02	1.37E-01	rs77116141	13_111120067_T_C	1.00	7174	5.99E-06	GCST90277264
Phosphatidylcholine (O-18:1_20:3) levels	15	61598332	T	C	-1.69E-01	3.43E-02	9.37E-01	rs7168957	15_61598332_C_T	1.00	7174	9.02E-07	GCST90277264
Phosphatidylcholine (O-18:1_20:3) levels	16	23623429	A	C	-8.01E-02	1.80E-02	3.77E-01	rs12926135	16_23623429_C_A	0.92	7174	9.11E-06	GCST90277264
Phosphatidylcholine (O-18:1_20:3) levels	17	9294060	C	T	8.58E-02	1.78E-02	5.80E-01	rs62067063	17_9294060_T_C	0.93	7174	1.50E-06	GCST90277264
Phosphatidylcholine (O-18:1_20:3) levels	17	49398516	G	A	2.90E-01	6.54E-02	1.65E-02	rs181353915	17_49398516_A_G	0.97	7174	9.48E-06	GCST90277264
Phosphatidylcholine (O-18:1_20:3) levels	19	1052854	G	A	-4.93E-01	1.10E-01	6.20E-03	rs78117248	19_1052854_A_G	0.94	7174	7.96E-06	GCST90277264
Phosphatidylcholine (O-18:1_20:3) levels	19	54065411	G	T	-3.45E-01	6.73E-02	1.68E-02	rs148313235	19_54065411_T_G	0.95	7174	3.16E-07	GCST90277264
Phosphatidylcholine (O-18:1_20:4) levels	1	39937698	T	G	1.01E-01	1.94E-02	2.50E-01	rs7529794	1_39937698_G_T	0.99	7173	1.65E-07	GCST90277265
Phosphatidylcholine (O-18:1_20:4) levels	1	94942936	A	G	-7.99E-02	1.66E-02	4.63E-01	rs1146461	1_94942936_G_A	1.00	7173	1.57E-06	GCST90277265
Phosphatidylcholine (O-18:1_20:4) levels	1	201132736	T	C	-7.83E-02	1.71E-02	5.75E-01	rs11582724	1_201132736_C_T	0.98	7173	4.64E-06	GCST90277265
Phosphatidylcholine (O-18:1_20:4) levels	2	178827066	C	T	-2.17E-01	4.86E-02	3.12E-02	rs73040346	2_178827066_T_C	1.00	7173	8.28E-06	GCST90277265
Phosphatidylcholine (O-18:1_20:4) levels	3	45251126	G	A	1.31E-01	2.86E-02	9.68E-02	rs56121911	3_45251126_A_G	1.00	7173	5.24E-06	GCST90277265
Phosphatidylcholine (O-18:1_20:4) levels	3	77008436	T	G	2.66E-01	5.45E-02	2.45E-02	rs76530357	3_77008436_G_T	0.96	7173	1.05E-06	GCST90277265
Phosphatidylcholine (O-18:1_20:4) levels	5	51956035	A	C	1.40E-01	3.12E-02	8.38E-02	rs143183807	5_51956035_C_A	0.96	7173	6.94E-06	GCST90277265
Phosphatidylcholine (O-18:1_20:4) levels	5	117857598	G	A	-2.68E-01	5.92E-02	2.11E-02	rs75668753	5_117857598_A_G	0.96	7173	6.12E-06	GCST90277265
Phosphatidylcholine (O-18:1_20:4) levels	6	101082967	T	A	-2.09E-01	4.39E-02	3.77E-02	rs78392466	6_101082967_A_T	0.99	7173	1.98E-06	GCST90277265
Phosphatidylcholine (O-18:1_20:4) levels	7	83961536	C	T	-7.54E-02	1.68E-02	5.61E-01	rs797821	7_83961536_T_C	1.00	7173	7.21E-06	GCST90277265
Phosphatidylcholine (O-18:1_20:4) levels	7	95573476	T	C	-3.51E-01	7.57E-02	1.27E-02	rs76309137	7_95573476_C_T	0.99	7173	3.63E-06	GCST90277265
Phosphatidylcholine (O-18:1_20:4) levels	7	124474397	C	T	3.39E-01	6.57E-02	1.68E-02	rs186790715	7_124474397_T_C	0.98	7173	2.45E-07	GCST90277265
Phosphatidylcholine (O-18:1_20:4) levels	8	11680104	T	A	4.52E-01	1.02E-01	8.10E-03	rs11780085	8_11680104_A_T	0.86	7173	8.92E-06	GCST90277265

Phosphatidylcholine (O-18:1_20:4) levels	8	18416117	A	T	1.77E-01	3.55E-02	6.11E-02	rs1018070	8_18416117_T_A	0.99	7173	5.83E-07	GCST90277265
Phosphatidylcholine (O-18:1_20:4) levels	8	53669039	T	C	1.76E-01	3.80E-02	5.18E-02	rs10095602	8_53669039_C_T	0.99	7173	3.79E-06	GCST90277265
Phosphatidylcholine (O-18:1_20:4) levels	9	33013634	G	A	-1.58E-01	3.37E-02	6.68E-02	rs145366219	9_33013634_A_G	1.00	7173	2.68E-06	GCST90277265
Phosphatidylcholine (O-18:1_20:4) levels	9	91806878	T	C	-8.77E-02	1.98E-02	2.47E-01	rs7048143	9_91806878_C_T	0.96	7173	9.79E-06	GCST90277265
Phosphatidylcholine (O-18:1_20:4) levels	10	70458313	A	C	1.99E-01	4.38E-02	4.43E-02	rs76179782	10_70458313_C_A	0.85	7173	5.97E-06	GCST90277265
Phosphatidylcholine (O-18:1_20:4) levels	10	74861163	C	G	-3.29E-01	6.72E-02	1.60E-02	rs117140365	10_74861163_G_C	0.96	7173	9.83E-07	GCST90277265
Phosphatidylcholine (O-18:1_20:4) levels	12	95853480	T	G	1.13E-01	2.26E-02	1.65E-01	rs77751297	12_95853480_G_T	0.99	7173	6.41E-07	GCST90277265
Phosphatidylcholine (O-18:1_20:4) levels	13	81451428	A	C	-7.87E-02	1.68E-02	5.31E-01	rs7322909	13_81451428_C_A	0.99	7173	3.00E-06	GCST90277265
Phosphatidylcholine (O-18:1_20:4) levels	13	110757928	G	T	7.54E-02	1.69E-02	4.39E-01	rs4000217	13_110757928_T_G	0.99	7173	8.23E-06	GCST90277265
Phosphatidylcholine (O-18:1_20:4) levels	14	36841591	T	C	-1.87E-01	4.08E-02	5.04E-02	rs79713567	14_36841591_C_T	0.87	7173	4.75E-06	GCST90277265
Phosphatidylcholine (O-18:1_20:4) levels	15	88358204	A	G	7.81E-02	1.75E-02	4.07E-01	rs6496477	15_88358204_G_A	0.93	7173	8.70E-06	GCST90277265
Phosphatidylcholine (O-18:1_20:4) levels	16	78457680	T	G	-1.08E-01	2.29E-02	1.63E-01	rs59634655	16_78457680_G_T	0.99	7173	2.46E-06	GCST90277265
Phosphatidylcholine (O-18:1_20:4) levels	19	54124989	T	C	-3.01E-01	6.71E-02	1.63E-02	rs140427655	19_54124989_C_T	1.00	7173	7.53E-06	GCST90277265
Phosphatidylcholine (O-18:1_20:4) levels	20	9964250	A	T	2.17E-01	4.70E-02	3.38E-02	rs76023792	20_9964250_T_A	0.94	7173	3.87E-06	GCST90277265
Phosphatidylcholine (O-18:1_20:4) levels	21	40397531	C	T	-5.00E-01	9.73E-02	8.09E-03	rs77900286	21_40397531_T_C	0.94	7173	2.88E-07	GCST90277265
Phosphatidylcholine (O-18:2_16:0) levels	1	39937698	T	G	1.26E-01	1.93E-02	2.50E-01	rs7529794	1_39937698_G_T	0.99	7174	8.15E-11	GCST90277266
Phosphatidylcholine (O-18:2_16:0) levels	1	246692321	C	G	-9.28E-02	1.80E-02	3.14E-01	rs61852444	1_246692321_G_C	0.99	7174	2.50E-07	GCST90277266
Phosphatidylcholine (O-18:2_16:0) levels	2	27256675	T	C	1.72E-01	3.84E-02	4.91E-02	rs76881617	2_27256675_C_T	0.99	7174	7.48E-06	GCST90277266
Phosphatidylcholine (O-18:2_16:0) levels	3	46995266	G	C	7.84E-02	1.71E-02	3.84E-01	rs11720139	3_46995266_C_G	1.00	7174	4.97E-06	GCST90277266
Phosphatidylcholine (O-18:2_16:0) levels	4	24133433	A	G	9.29E-02	1.93E-02	2.63E-01	rs11727825	4_24133433_G_A	0.97	7174	1.43E-06	GCST90277266
Phosphatidylcholine (O-18:2_16:0) levels	4	77085694	G	A	-2.11E-01	4.62E-02	3.50E-02	rs115972339	4_77085694_A_G	0.98	7174	5.16E-06	GCST90277266
Phosphatidylcholine (O-18:2_16:0) levels	5	2069974	C	A	1.53E-01	3.34E-02	7.02E-02	rs146435288	5_2069974_A_C	0.97	7174	4.37E-06	GCST90277266
Phosphatidylcholine (O-18:2_16:0) levels	6	7298054	A	G	7.96E-02	1.72E-02	3.91E-01	rs2714309	6_7298054_G_A	1.00	7174	3.76E-06	GCST90277266
Phosphatidylcholine (O-18:2_16:0) levels	6	161340057	G	T	-8.11E-02	1.76E-02	3.35E-01	rs6455722	6_161340057_T_G	0.99	7174	4.26E-06	GCST90277266
Phosphatidylcholine (O-18:2_16:0) levels	7	100920067	C	T	-7.57E-02	1.69E-02	4.36E-01	rs9691107	7_100920067_T_C	0.99	7174	7.83E-06	GCST90277266
Phosphatidylcholine (O-18:2_16:0) levels	8	18396999	C	G	1.02E-01	2.13E-02	1.93E-01	rs35583283	8_18396999_G_C	1.00	7174	1.70E-06	GCST90277266
Phosphatidylcholine (O-18:2_16:0) levels	11	20577887	T	C	-8.36E-02	1.77E-02	6.70E-01	rs10833360	11_20577887_C_T	1.00	7174	2.38E-06	GCST90277266
Phosphatidylcholine (O-18:2_16:0) levels	11	41087025	G	C	8.23E-02	1.72E-02	6.33E-01	rs7935110	11_41087025_C_G	1.00	7174	1.70E-06	GCST90277266
Phosphatidylcholine (O-18:2_16:0) levels	11	109005691	C	T	-7.97E-02	1.79E-02	3.17E-01	rs11212840	11_109005691_T_C	0.99	7174	8.07E-06	GCST90277266
Phosphatidylcholine (O-18:2_16:0) levels	12	84167129	C	T	7.91E-02	1.75E-02	6.50E-01	rs6539823	12_84167129_T_C	1.00	7174	6.07E-06	GCST90277266
Phosphatidylcholine (O-18:2_16:0) levels	13	59324563	G	A	-8.64E-02	1.89E-02	2.77E-01	rs9527967	13_59324563_A_G	0.98	7174	5.07E-06	GCST90277266
Phosphatidylcholine (O-18:2_16:0) levels	15	88358204	A	G	7.87E-02	1.75E-02	4.07E-01	rs6496477	15_88358204_G_A	0.93	7174	7.32E-06	GCST90277266
Phosphatidylcholine (O-18:2_16:0) levels	17	49270778	C	T	-8.07E-02	1.68E-02	4.83E-01	rs71379351	17_49270778_T_C	1.00	7174	1.54E-06	GCST90277266
Phosphatidylcholine (O-18:2_16:0) levels	17	61552499	T	C	1.47E-01	3.07E-02	9.29E-02	rs142604671	17_61552499_C_T	0.88	7174	1.56E-06	GCST90277266
Phosphatidylcholine (O-18:2_16:0) levels	17	61714452	C	T	-3.14E-01	6.99E-02	1.51E-02	rs55908410	17_61714452_T_C	0.95	7174	7.15E-06	GCST90277266
Phosphatidylcholine (O-18:2_16:0) levels	17	68496359	C	G	9.53E-02	2.11E-02	1.90E-01	rs72845685	17_68496359_G_C	1.00	7174	6.52E-06	GCST90277266
Phosphatidylcholine (O-18:2_16:0) levels	20	16819023	A	C	-4.68E-01	1.05E-01	7.10E-03	rs138778351	20_16819023_C_A	0.91	7174	9.37E-06	GCST90277266
Phosphatidylcholine (O-18:2_18:1) levels	1	39937698	T	G	1.17E-01	1.93E-02	2.50E-01	rs7529794	1_39937698_G_T	0.99	7174	1.76E-09	GCST90277267
Phosphatidylcholine (O-18:2_18:1) levels	1	171705634	A	G	8.80E-02	1.94E-02	2.57E-01	rs6685742	1_171705634_G_A	1.00	7174	5.73E-06	GCST90277267
Phosphatidylcholine (O-18:2_18:1) levels	1	246693388	A	G	-8.54E-02	1.76E-02	3.39E-01	rs59319733	1_246693388_G_A	1.00	7174	1.20E-06	GCST90277267
Phosphatidylcholine (O-18:2_18:1) levels	2	204687512	T	C	1.98E-01	3.50E-02	6.71E-02	rs149768482	2_204687512_C_T	0.93	7174	1.46E-08	GCST90277267
Phosphatidylcholine (O-18:2_18:1) levels	3	110722896	G	A	-2.07E-01	4.69E-02	3.81E-02	rs73218874	3_110722896_A_G	0.87	7174	9.92E-06	GCST90277267
Phosphatidylcholine (O-18:2_18:1) levels	3	122279149	A	G	2.75E-01	6.14E-02	1.91E-02	rs114796420	3_122279149_G_A	0.99	7174	7.90E-06	GCST90277267
Phosphatidylcholine (O-18:2_18:1) levels	4	77085694	G	A	-2.25E-01	4.61E-02	3.50E-02	rs115972339	4_77085694_A_G	0.98	7174	1.04E-06	GCST90277267
Phosphatidylcholine (O-18:2_18:1) levels	4	182275394	T	C	1.76E-01	3.65E-02	5.51E-02	rs150917153	4_182275394_C_T	0.98	7174	1.45E-06	GCST90277267
Phosphatidylcholine (O-18:2_18:1) levels	4	184839680	G	A	2.22E-01	4.97E-02	3.01E-02	rs72707125	4_184839680_A_G	0.94	7174	7.93E-06	GCST90277267
Phosphatidylcholine (O-18:2_18:1) levels	8	18381562	G	C	-7.51E-02	1.68E-02	4.24E-01	rs17126576	8_18381562_C_G	1.00	7174	8.21E-06	GCST90277267
Phosphatidylcholine (O-18:2_18:1) levels	8	56509388	G	A	2.74E-01	5.64E-02	9.77E-01	rs2610042	8_56509388_A_G	0.99	7174	1.19E-06	GCST90277267
Phosphatidylcholine (O-18:2_18:1) levels	11	20577887	T	C	-8.82E-02	1.77E-02	6.70E-01	rs10833360	11_20577887_C_T	1.00	7174	6.25E-07	GCST90277267
Phosphatidylcholine (O-18:2_18:1) levels	11	30336511	G	A	-2.09E-01	4.44E-02	3.84E-02	rs139683825	11_30336511_A_G	0.98	7174	2.52E-06	GCST90277267
Phosphatidylcholine (O-18:2_18:1) levels	11	61814292	C	T	1.31E-01	1.72E-02	3.83E-01	rs174560	11_61814292_T_C	1.00	7174	3.22E-14	GCST90277267
Phosphatidylcholine (O-18:2_18:1) levels	17	31622748	T	A	-8.12E-02	1.77E-02	6.71E-01	rs414383	17_31622748_A_T	0.99	7174	4.37E-06	GCST90277267
Phosphatidylcholine (O-18:2_18:1) levels	17	49270778	C	T	-7.66E-02	1.68E-02	4.83E-01	rs71379351	17_49270778_T_C	1.00	7174	4.95E-06	GCST90277267
Phosphatidylcholine (O-18:2_18:1) levels	17	68491696	C	A	8.94E-02	1.92E-02	2.52E-01	rs8069148	17_68491696_A_C	1.00	7174	3.14E-06	GCST90277267
Phosphatidylcholine (O-18:2_18:1) levels	18	57256007	A	T	2.60E-01	5.45E-02	2.72E-02	rs111336143	18_57256007_T_A	0.89	7174	1.85E-06	GCST90277267
Phosphatidylcholine (O-18:2_18:2) levels	1	29962031	T	C	-2.16E-01	4.76E-02	3.71E-02	rs76469829	1_29962031_C_T	0.95	6235	5.69E-06	GCST90277268
Phosphatidylcholine (O-18:2_18:2) levels	1	172130754	A	G	3.78E-01	8.42E-02	1.17E-02	rs76513766	1_172130754_G_A	0.99	6235	7.01E-06	GCST90277268

Phosphatidylcholine (O-18:2_18:2) levels	2	56382464	T	A	-1.01E-01	1.87E-02	3.50E-01	rs9967725	2_56382464_A_T	1.00	6235	6.79E-08	GCST90277268
Phosphatidylcholine (O-18:2_18:2) levels	4	150117372	TC	T	4.89E-01	1.02E-01	8.11E-03	rs200932481	4_150117372_T_TC	0.96	6235	1.66E-06	GCST90277268
Phosphatidylcholine (O-18:2_18:2) levels	6	161271441	A	G	-9.87E-02	2.17E-02	2.15E-01	rs4708901	6_161271441_G_A	0.99	6235	5.37E-06	GCST90277268
Phosphatidylcholine (O-18:2_18:2) levels	7	2289982	A	C	8.05E-01	1.77E-01	2.36E-03	rs191895023	7_2289982_C_A	0.89	6235	5.63E-06	GCST90277268
Phosphatidylcholine (O-18:2_18:2) levels	7	95974040	G	C	-1.94E-01	3.98E-02	5.62E-02	rs142999489	7_95974040_C_G	0.97	6235	1.09E-06	GCST90277268
Phosphatidylcholine (O-18:2_18:2) levels	8	116151644	G	C	2.20E-01	4.64E-02	4.20E-02	rs62511275	8_116151644_C_G	0.89	6235	2.08E-06	GCST90277268
Phosphatidylcholine (O-18:2_18:2) levels	9	114386150	T	G	8.28E-02	1.78E-02	4.85E-01	rs10733608	9_114386150_G_T	1.00	6235	3.34E-06	GCST90277268
Phosphatidylcholine (O-18:2_18:2) levels	10	106220847	A	G	-8.34E-02	1.85E-02	3.65E-01	rs10884272	10_106220847_G_A	0.99	6235	6.46E-06	GCST90277268
Phosphatidylcholine (O-18:2_18:2) levels	11	61744026	T	C	1.66E-01	2.63E-02	1.34E-01	rs3741252	11_61744026_C_T	0.99	6235	3.34E-10	GCST90277268
Phosphatidylcholine (O-18:2_18:2) levels	11	61818856	CT	C	-5.35E-01	1.70E-02	4.06E-01	rs35473591	11_61818856_C_CT	1.00	6235	4.23E-202	GCST90277268
Phosphatidylcholine (O-18:2_18:2) levels	11	62056158	C	T	-1.76E-01	2.39E-02	1.73E-01	rs12226389	11_62056158_T_C	0.99	6235	2.15E-13	GCST90277268
Phosphatidylcholine (O-18:2_18:2) levels	11	62451557	G	A	1.34E-01	2.09E-02	2.57E-01	rs7936002	11_62451557_A_G	0.93	6235	1.65E-10	GCST90277268
Phosphatidylcholine (O-18:2_18:2) levels	11	64357915	TG	CG	7.97E-02	1.79E-02	4.73E-01	rs474901	11_64357915_CG_TG	1.00	6235	8.54E-06	GCST90277268
Phosphatidylcholine (O-18:2_18:2) levels	11	75744264	G	A	-1.21E-01	2.12E-02	2.32E-01	rs600626	11_75744264_A_G	0.99	6235	1.23E-08	GCST90277268
Phosphatidylcholine (O-18:2_18:2) levels	12	5112153	C	T	9.72E-02	2.17E-02	7.84E-01	rs529446	12_5112153_T_C	1.00	6235	7.85E-06	GCST90277268
Phosphatidylcholine (O-18:2_18:2) levels	12	98288399	GA	G	1.78E-01	3.97E-02	5.35E-02	rs35599386	12_98288399_G_GA	0.99	6235	7.32E-06	GCST90277268
Phosphatidylcholine (O-18:2_18:2) levels	14	41287117	T	G	-6.53E-01	1.44E-01	4.44E-03	rs79995028	14_41287117_G_T	0.89	6235	5.86E-06	GCST90277268
Phosphatidylcholine (O-18:2_18:2) levels	15	23837617	T	A	3.13E-01	6.43E-02	9.80E-01	rs7179330	15_23837617_A_T	0.97	6235	1.15E-06	GCST90277268
Phosphatidylcholine (O-18:2_18:2) levels	15	71480547	T	C	-1.02E-01	2.24E-02	2.04E-01	rs12905466	15_71480547_C_T	0.99	6235	5.01E-06	GCST90277268
Phosphatidylcholine (O-18:2_18:2) levels	16	75219782	G	C	8.56E-02	1.91E-02	3.29E-01	rs6564238	16_75219782_C_G	0.99	6235	7.56E-06	GCST90277268
Phosphatidylcholine (O-18:2_18:2) levels	17	49470511	A	G	-1.48E-01	3.29E-02	8.01E-02	rs150158785	17_49470511_G_A	0.97	6235	7.51E-06	GCST90277268
Phosphatidylcholine (O-18:2_18:2) levels	18	64269741	A	G	1.18E-01	2.68E-02	1.30E-01	rs1363011	18_64269741_G_A	0.98	6235	1.00E-05	GCST90277268
Phosphatidylcholine (O-18:2_18:2) levels	19	614967	C	T	9.10E-02	2.02E-02	3.41E-01	rs11669519	19_614967_T_C	0.85	6235	6.45E-06	GCST90277268
Phosphatidylcholine (O-18:2_18:2) levels	19	19347579	G	A	-2.08E-01	4.04E-02	5.38E-02	rs182611493	19_19347579_A_G	0.95	6235	2.78E-07	GCST90277268
Phosphatidylcholine (O-18:2_18:2) levels	20	64047793	A	G	-1.04E-01	2.27E-02	1.96E-01	rs12303	20_64047793_G_A	0.98	6235	4.56E-06	GCST90277268
Phosphatidylcholine (O-18:2_18:2) levels	22	23818551	C	T	-3.53E-01	7.14E-02	1.63E-02	rs139516883	22_23818551_T_C	0.96	6235	7.61E-07	GCST90277268
Phosphatidylcholine (O-18:2_20:4) levels	1	39937698	T	G	1.51E-01	2.07E-02	2.50E-01	rs7529794	1_39937698_G_T	0.99	6258	3.79E-13	GCST90277269
Phosphatidylcholine (O-18:2_20:4) levels	1	155135691	A	G	-2.32E-01	4.50E-02	4.29E-02	rs12726330	1_155135691_G_A	1.00	6258	2.54E-07	GCST90277269
Phosphatidylcholine (O-18:2_20:4) levels	2	9689106	G	A	-1.10E-01	2.31E-02	1.97E-01	rs6713691	2_9689106_A_G	0.96	6258	1.76E-06	GCST90277269
Phosphatidylcholine (O-18:2_20:4) levels	2	27508073	C	T	-9.43E-02	1.88E-02	6.51E-01	rs1260326	2_27508073_T_C	1.00	6258	5.12E-07	GCST90277269
Phosphatidylcholine (O-18:2_20:4) levels	2	235266114	G	A	9.72E-02	2.07E-02	7.46E-01	rs4663191	2_235266114_A_G	0.97	6258	2.64E-06	GCST90277269
Phosphatidylcholine (O-18:2_20:4) levels	4	3285732	G	C	1.04E-01	2.29E-02	1.91E-01	rs3135062	4_3285732_C_G	0.99	6258	5.91E-06	GCST90277269
Phosphatidylcholine (O-18:2_20:4) levels	5	54771412	T	C	2.17E-01	4.33E-02	4.89E-02	rs72757442	5_54771412_C_T	0.90	6258	5.83E-07	GCST90277269
Phosphatidylcholine (O-18:2_20:4) levels	6	49191020	T	C	-7.38E-01	1.64E-01	3.39E-03	rs62411861	6_49191020_C_T	0.94	6258	6.88E-06	GCST90277269
Phosphatidylcholine (O-18:2_20:4) levels	6	142892562	A	G	-2.92E-01	5.92E-02	2.44E-02	rs12196051	6_142892562_G_A	0.95	6258	8.50E-07	GCST90277269
Phosphatidylcholine (O-18:2_20:4) levels	6	159557473	C	T	2.77E-01	6.07E-02	2.43E-02	rs149182005	6_159557473_T_C	0.93	6258	5.17E-06	GCST90277269
Phosphatidylcholine (O-18:2_20:4) levels	7	70998624	G	C	3.40E-01	7.27E-02	1.63E-02	rs116987919	7_70998624_C_G	0.97	6258	2.98E-06	GCST90277269
Phosphatidylcholine (O-18:2_20:4) levels	7	116377385	T	A	2.06E-01	4.65E-02	4.02E-02	rs189518615	7_116377385_A_T	0.95	6258	9.08E-06	GCST90277269
Phosphatidylcholine (O-18:2_20:4) levels	8	18433891	A	T	-9.69E-02	1.91E-02	6.62E-01	rs35152003	8_18433891_T_A	0.97	6258	4.05E-07	GCST90277269
Phosphatidylcholine (O-18:2_20:4) levels	9	99053057	T	G	-1.19E-01	2.50E-02	1.51E-01	rs16918167	9_99053057_G_T	1.00	6258	1.96E-06	GCST90277269
Phosphatidylcholine (O-18:2_20:4) levels	15	58391167	G	A	-1.01E-01	1.80E-02	5.73E-01	rs1532085	15_58391167_A_G	1.00	6258	1.93E-08	GCST90277269
Phosphatidylcholine (O-18:2_20:4) levels	15	58431476	T	C	1.09E-01	2.08E-02	2.58E-01	rs1800588	15_58431476_C_T	0.99	6258	1.76E-07	GCST90277269
Phosphatidylcholine (O-18:2_20:4) levels	17	12597341	T	C	8.08E-02	1.80E-02	5.93E-01	rs758186	17_12597341_C_T	0.99	6258	7.19E-06	GCST90277269
Phosphatidylcholine (O-18:2_20:4) levels	17	49966298	T	G	1.46E-01	3.12E-02	9.58E-02	rs79577600	17_49966298_G_T	0.95	6258	2.89E-06	GCST90277269
Phosphatidylcholine (O-18:2_20:4) levels	21	44325499	T	G	6.74E-01	1.51E-01	3.51E-03	rs62220410	21_44325499_G_T	0.99	6258	8.58E-06	GCST90277269
Phosphatidylethanolamine (16:0_18:2) levels	1	230124135	G	A	-1.98E-01	4.40E-02	5.41E-02	rs116537941	1_230124135_A_G	0.95	5172	6.84E-06	GCST90277270
Phosphatidylethanolamine (16:0_18:2) levels	2	27102273	A	G	2.28E-01	4.69E-02	4.68E-02	rs75388820	2_27102273_G_A	0.97	5172	1.19E-06	GCST90277270
Phosphatidylethanolamine (16:0_18:2) levels	2	152605833	A	G	9.33E-02	2.07E-02	6.13E-01	rs6434115	2_152605833_G_A	0.95	5172	6.92E-06	GCST90277270
Phosphatidylethanolamine (16:0_18:2) levels	3	23781447	A	G	-1.03E-01	2.20E-02	2.72E-01	rs9826895	3_23781447_G_A	0.99	5172	2.95E-06	GCST90277270
Phosphatidylethanolamine (16:0_18:2) levels	3	50443604	T	G	1.09E-01	2.18E-02	2.77E-01	rs11917269	3_50443604_G_T	1.00	5172	6.75E-07	GCST90277270
Phosphatidylethanolamine (16:0_18:2) levels	3	73256915	A	T	-9.96E-02	1.96E-02	4.76E-01	rs7636653	3_73256915_T_A	1.00	5172	4.13E-07	GCST90277270
Phosphatidylethanolamine (16:0_18:2) levels	3	142947149	C	T	1.10E-01	2.04E-02	6.29E-01	rs6768813	3_142947149_T_C	0.99	5172	7.74E-08	GCST90277270
Phosphatidylethanolamine (16:0_18:2) levels	4	168564086	G	A	8.86E-02	1.98E-02	4.63E-01	rs10015043	4_168564086_A_G	1.00	5172	7.91E-06	GCST90277270
Phosphatidylethanolamine (16:0_18:2) levels	5	4129081	T	C	-3.55E-01	7.99E-02	1.48E-02	rs115554476	5_4129081_C_T	0.96	5172	9.06E-06	GCST90277270
Phosphatidylethanolamine (16:0_18:2) levels	5	99192949	A	G	-5.25E-01	1.13E-01	9.18E-03	rs149492888	5_99192949_G_A	0.93	5172	3.40E-06	GCST90277270
Phosphatidylethanolamine (16:0_18:2) levels	6	73616238	A	G	-1.08E-01	2.36E-02	2.18E-01	rs35584640	6_73616238_G_A	1.00	5172	4.89E-06	GCST90277270
Phosphatidylethanolamine (16:0_18:2) levels	6	85323731	T	G	8.73E-01	1.95E-01	2.48E-03	rs151000252	6_85323731_G_T	0.92	5172	7.49E-06	GCST90277270

Phosphatidylethanolamine (16:0_18:2) levels	7	50816756	A	G	-1.37E-01	2.68E-02	8.41E-01	rs7776911	7_50816756_G_A	1.00	5172	3.22E-07	GCST90277270
Phosphatidylethanolamine (16:0_18:2) levels	10	127361837	T	C	-1.45E-01	3.23E-02	1.09E-01	rs2296637	10_127361837_C_T	0.97	5172	7.14E-06	GCST90277270
Phosphatidylethanolamine (16:0_18:2) levels	14	46165338	T	C	-9.14E-01	2.06E-01	3.30E-03	rs192233385	14_46165338_C_T	0.95	5172	9.20E-06	GCST90277270
Phosphatidylethanolamine (16:0_18:2) levels	15	38411586	A	C	2.66E-01	5.91E-02	2.87E-02	rs117398262	15_38411586_C_A	0.98	5172	6.72E-06	GCST90277270
Phosphatidylethanolamine (16:0_18:2) levels	17	5310096	T	C	-2.41E-01	4.87E-02	4.43E-02	rs2585266	17_5310096_C_T	0.96	5172	7.67E-07	GCST90277270
Phosphatidylethanolamine (16:0_18:2) levels	17	83090952	A	G	-4.43E-01	9.97E-02	1.05E-02	rs113318060	17_83090952_G_A	0.96	5172	8.86E-06	GCST90277270
Phosphatidylethanolamine (16:0_20:4) levels	1	189909464	A	G	-8.79E-02	1.83E-02	3.09E-01	rs6413913	1_189909464_G_A	1.00	6905	1.50E-06	GCST90277271
Phosphatidylethanolamine (16:0_20:4) levels	1	201480758	C	A	4.23E-01	8.67E-02	1.26E-02	rs111877698	1_201480758_A_C	0.82	6905	1.10E-06	GCST90277271
Phosphatidylethanolamine (16:0_20:4) levels	1	218177981	C	T	8.22E-02	1.80E-02	3.49E-01	rs1338449	1_218177981_T_C	1.00	6905	4.98E-06	GCST90277271
Phosphatidylethanolamine (16:0_20:4) levels	2	27508073	C	T	-1.15E-01	1.78E-02	6.51E-01	rs1260326	2_27508073_T_C	1.00	6905	1.12E-10	GCST90277271
Phosphatidylethanolamine (16:0_20:4) levels	3	50383123	A	C	-8.73E-02	1.92E-02	2.65E-01	rs2236950	3_50383123_C_A	0.99	6905	5.47E-06	GCST90277271
Phosphatidylethanolamine (16:0_20:4) levels	3	126114417	T	C	7.62E-02	1.70E-02	4.97E-01	rs2290053	3_126114417_C_T	1.00	6905	7.24E-06	GCST90277271
Phosphatidylethanolamine (16:0_20:4) levels	4	152031948	A	G	-1.89E-01	3.75E-02	5.75E-02	rs114899411	4_152031948_G_A	0.99	6905	5.08E-07	GCST90277271
Phosphatidylethanolamine (16:0_20:4) levels	5	996057	G	A	-8.96E-02	1.95E-02	2.85E-01	rs326048	5_996057_A_G	0.93	6905	4.60E-06	GCST90277271
Phosphatidylethanolamine (16:0_20:4) levels	5	21867829	G	A	-3.02E-01	6.63E-02	1.73E-02	rs115359603	5_21867829_A_G	0.94	6905	5.26E-06	GCST90277271
Phosphatidylethanolamine (16:0_20:4) levels	5	173674912	C	G	-7.98E-02	1.75E-02	6.28E-01	rs791631	5_173674912_G_C	0.99	6905	5.58E-06	GCST90277271
Phosphatidylethanolamine (16:0_20:4) levels	6	22879225	C	T	1.11E-01	2.49E-02	1.36E-01	rs79211442	6_22879225_T_C	0.99	6905	9.23E-06	GCST90277271
Phosphatidylethanolamine (16:0_20:4) levels	7	50816756	A	G	-1.04E-01	2.34E-02	8.41E-01	rs7776911	7_50816756_G_A	1.00	6905	9.36E-06	GCST90277271
Phosphatidylethanolamine (16:0_20:4) levels	7	153641664	A	T	1.28E-01	2.83E-02	8.95E-01	rs4726320	7_153641664_T_A	0.96	6905	5.80E-06	GCST90277271
Phosphatidylethanolamine (16:0_20:4) levels	8	2533493	C	G	5.60E-01	1.15E-01	5.95E-03	rs146330404	8_2533493_G_C	0.89	6905	1.14E-06	GCST90277271
Phosphatidylethanolamine (16:0_20:4) levels	8	3234062	T	G	-1.16E-01	2.21E-02	1.79E-01	rs1442407	8_3234062_G_T	1.00	6905	1.60E-07	GCST90277271
Phosphatidylethanolamine (16:0_20:4) levels	10	64197077	T	C	2.45E-01	5.40E-02	2.55E-02	rs71508993	10_64197077_C_T	0.98	6905	5.81E-06	GCST90277271
Phosphatidylethanolamine (16:0_20:4) levels	11	61514878	T	C	1.39E-01	2.66E-02	1.16E-01	rs3741259	11_61514878_C_T	0.99	6905	1.86E-07	GCST90277271
Phosphatidylethanolamine (16:0_20:4) levels	11	61814292	C	T	3.65E-01	1.71E-02	3.83E-01	rs174560	11_61814292_T_C	1.00	6905	7.02E-98	GCST90277271
Phosphatidylethanolamine (16:0_20:4) levels	11	62170073	A	G	9.02E-02	1.75E-02	3.87E-01	rs1792909	11_62170073_G_A	1.00	6905	2.60E-07	GCST90277271
Phosphatidylethanolamine (16:0_20:4) levels	12	4892157	G	A	-1.34E-01	2.87E-02	1.02E-01	rs11063393	12_4892157_A_G	0.96	6905	3.22E-06	GCST90277271
Phosphatidylethanolamine (16:0_20:4) levels	12	21197755	G	T	-1.18E-01	2.29E-02	1.66E-01	rs11045856	12_21197755_T_G	1.00	6905	2.49E-07	GCST90277271
Phosphatidylethanolamine (16:0_20:4) levels	12	99719293	C	T	5.44E-01	1.04E-01	6.81E-03	rs188404615	12_99719293_T_C	0.95	6905	1.53E-07	GCST90277271
Phosphatidylethanolamine (16:0_20:4) levels	15	58432593	A	T	8.81E-02	1.86E-02	3.11E-01	rs8034802	15_58432593_T_A	1.00	6905	2.31E-06	GCST90277271
Phosphatidylethanolamine (16:0_20:4) levels	17	38791988	T	C	3.88E-01	7.66E-02	1.28E-02	rs62076866	17_38791988_C_T	0.98	6905	4.38E-07	GCST90277271
Phosphatidylethanolamine (16:0_20:4) levels	18	5841984	G	A	-1.15E-01	2.43E-02	1.54E-01	rs35140833	18_5841984_A_G	0.93	6905	2.30E-06	GCST90277271
Phosphatidylethanolamine (16:0_20:4) levels	19	40833991	C	T	8.70E-02	1.92E-02	7.14E-01	rs12459249	19_40833991_T_C	0.98	6905	5.89E-06	GCST90277271
Phosphatidylethanolamine (16:0_20:4) levels	21	17470749	A	G	9.06E-02	2.01E-02	2.72E-01	rs62242707	21_17470749_G_A	0.91	6905	6.33E-06	GCST90277271
Phosphatidylethanolamine (18:0_18:2) levels	4	93196475	T	C	-2.29E-01	5.11E-02	3.96E-02	rs72666927	4_93196475_C_T	0.96	5497	7.34E-06	GCST90277272
Phosphatidylethanolamine (18:0_18:2) levels	4	154586438	C	T	9.48E-02	2.03E-02	3.23E-01	rs6050	4_154586438_T_C	0.99	5497	3.24E-06	GCST90277272
Phosphatidylethanolamine (18:0_18:2) levels	5	21444102	G	T	-2.12E-01	4.71E-02	4.93E-02	rs148426771	5_21444102_T_G	0.89	5497	6.49E-06	GCST90277272
Phosphatidylethanolamine (18:0_18:2) levels	6	86624181	T	C	2.42E-01	5.26E-02	3.67E-02	rs77191266	6_86624181_C_T	0.95	5497	4.43E-06	GCST90277272
Phosphatidylethanolamine (18:0_18:2) levels	7	102708677	T	A	1.03E-01	2.30E-02	2.20E-01	rs60984271	7_102708677_A_T	0.99	5497	8.34E-06	GCST90277272
Phosphatidylethanolamine (18:0_18:2) levels	8	27843092	A	G	1.34E-01	3.01E-02	1.09E-01	rs55654962	8_27843092_G_A	0.99	5497	8.32E-06	GCST90277272
Phosphatidylethanolamine (18:0_18:2) levels	8	82931796	G	T	2.70E-01	5.67E-02	2.98E-02	rs74939791	8_82931796_T_G	0.98	5497	2.03E-06	GCST90277272
Phosphatidylethanolamine (18:0_18:2) levels	9	127026955	A	G	2.10E-01	4.63E-02	4.52E-02	rs72764354	9_127026955_G_A	0.99	5497	5.86E-06	GCST90277272
Phosphatidylethanolamine (18:0_18:2) levels	10	98416870	A	G	5.27E-01	1.17E-01	7.37E-03	rs7075480	10_98416870_G_A	0.99	5497	7.18E-06	GCST90277272
Phosphatidylethanolamine (18:0_18:2) levels	13	27228661	T	C	1.11E-01	2.23E-02	7.65E-01	rs9507859	13_27228661_C_T	1.00	5497	7.13E-07	GCST90277272
Phosphatidylethanolamine (18:0_18:2) levels	15	57731057	C	T	-1.66E-01	3.54E-02	7.93E-02	rs28416404	15_57731057_T_C	0.98	5497	2.84E-06	GCST90277272
Phosphatidylethanolamine (18:0_18:2) levels	15	97625949	T	C	5.17E-01	1.15E-01	7.52E-03	rs1553262	15_97625949_C_T	0.94	5497	6.57E-06	GCST90277272
Phosphatidylethanolamine (18:0_18:2) levels	18	11445795	G	A	-1.06E-01	2.39E-02	2.07E-01	rs7230887	18_11445795_A_G	0.98	5497	9.29E-06	GCST90277272
Phosphatidylethanolamine (18:0_18:2) levels	18	33463076	T	C	9.90E-02	2.10E-02	2.89E-01	rs7239464	18_33463076_C_T	1.00	5497	2.43E-06	GCST90277272
Phosphatidylethanolamine (18:0_18:2) levels	18	68848836	TG	T	-3.18E-01	7.18E-02	1.86E-02	rs145618222	18_68848836_T_TG	0.94	5497	9.34E-06	GCST90277272
Phosphatidylethanolamine (18:0_18:2) levels	18	80062512	A	G	3.52E-01	7.84E-02	1.51E-02	rs75414245	18_80062512_G_A	0.97	5497	7.28E-06	GCST90277272
Phosphatidylethanolamine (18:0_18:2) levels	19	20083148	T	C	-1.14E-01	2.50E-02	1.85E-01	rs1230353	19_20083148_C_T	0.98	5497	4.92E-06	GCST90277272
Phosphatidylethanolamine (18:0_18:2) levels	21	34985564	G	A	1.41E-01	3.03E-02	8.88E-01	rs2014300	21_34985564_A_G	1.00	5497	3.42E-06	GCST90277272
Phosphatidylethanolamine (18:0_18:2) levels	22	36971495	T	C	-1.21E-01	2.71E-02	1.52E-01	rs17749540	22_36971495_C_T	0.98	5497	7.39E-06	GCST90277272
Phosphatidylethanolamine (18:0_20:4) levels	1	230143835	G	A	8.80E-02	1.98E-02	7.68E-01	rs4846905	1_230143835_A_G	1.00	7064	8.68E-06	GCST90277273
Phosphatidylethanolamine (18:0_20:4) levels	2	27508073	C	T	-1.05E-01	1.76E-02	6.51E-01	rs1260326	2_27508073_T_C	1.00	7064	2.39E-09	GCST90277273
Phosphatidylethanolamine (18:0_20:4) levels	2	227401238	C	T	1.24E-01	2.71E-02	1.08E-01	rs62189362	2_227401238_T_C	0.99	7064	4.45E-06	GCST90277273
Phosphatidylethanolamine (18:0_20:4) levels	3	138147591	G	A	-8.06E-02	1.78E-02	6.53E-01	rs4564909	3_138147591_A_G	1.00	7064	5.94E-06	GCST90277273
Phosphatidylethanolamine (18:0_20:4) levels	3	195507255	C	G	8.99E-02	1.86E-02	7.11E-01	rs2686446	3_195507255_G_C	0.98	7064	1.41E-06	GCST90277273

Phosphatidylethanolamine (18:0_20:4) levels	4	87278332	G	T	-7.86E-02	1.70E-02	5.44E-01	rs6531968	4_87278332_T_G	1.00	7064	3.79E-06	GCST90277273
Phosphatidylethanolamine (18:0_20:4) levels	5	141674766	T	C	-2.41E-01	5.41E-02	2.54E-02	rs79066364	5_141674766_C_T	0.96	7064	8.80E-06	GCST90277273
Phosphatidylethanolamine (18:0_20:4) levels	8	12966162	C	T	9.87E-02	2.11E-02	2.06E-01	rs2946493	8_12966162_T_C	0.98	7064	2.91E-06	GCST90277273
Phosphatidylethanolamine (18:0_20:4) levels	9	137711224	A	G	1.35E-01	2.82E-02	1.08E-01	rs113668788	9_137711224_G_A	0.93	7064	1.81E-06	GCST90277273
Phosphatidylethanolamine (18:0_20:4) levels	12	47729780	G	A	9.03E-02	1.96E-02	2.41E-01	rs7484827	12_47729780_A_G	1.00	7064	4.21E-06	GCST90277273
Phosphatidylethanolamine (18:0_20:4) levels	13	37381120	T	G	1.55E-01	3.28E-02	7.44E-02	rs78414457	13_37381120_G_T	0.96	7064	2.43E-06	GCST90277273
Phosphatidylethanolamine (18:0_20:4) levels	15	58428969	C	G	-8.13E-02	1.74E-02	4.71E-01	rs11857386	15_58428969_G_C	0.94	7064	3.12E-06	GCST90277273
Phosphatidylethanolamine (18:0_20:4) levels	16	56953103	T	C	9.71E-02	1.87E-02	2.79E-01	rs12446515	16_56953103_C_T	1.00	7064	2.30E-07	GCST90277273
Phosphatidylethanolamine (18:0_20:4) levels	17	4480495	T	C	-1.21E-01	2.69E-02	1.11E-01	rs3816689	17_4480495_C_T	0.99	7064	7.13E-06	GCST90277273
Phosphatidylethanolamine (18:0_20:4) levels	18	15033501	T	C	3.72E-01	7.45E-02	1.31E-02	rs146934967	18_15033501_C_T	0.99	7064	5.92E-07	GCST90277273
Phosphatidylethanolamine (18:0_20:4) levels	18	21542677	T	G	-1.21E-01	2.01E-02	7.81E-01	rs12960229	18_21542677_G_T	1.00	7064	1.87E-09	GCST90277273
Phosphatidylethanolamine (18:0_20:4) levels	19	19347579	G	A	-1.69E-01	3.81E-02	5.38E-02	rs182611493	19_19347579_A_G	0.95	7064	9.04E-06	GCST90277273
Phosphatidylethanolamine (18:0_20:4) levels	20	62310086	A	C	-9.05E-02	1.73E-02	3.99E-01	rs2379129	20_62310086_C_A	0.99	7064	1.65E-07	GCST90277273
Phosphatidylethanolamine (18:0_20:4) levels	22	19662612	T	C	-5.32E-01	1.20E-01	5.53E-03	rs144849989	22_19662612_C_T	0.88	7064	9.33E-06	GCST90277273
Phosphatidylethanolamine (18:0_20:4) levels	22	24495324	G	C	8.54E-02	1.70E-02	5.49E-01	rs2070474	22_24495324_C_G	1.00	7064	5.10E-07	GCST90277273
Phosphatidylethanolamine (18:1_18:1) levels	1	63400788	C	T	2.22E-01	5.03E-02	3.04E-02	rs116105185	1_63400788_T_C	0.96	6866	9.97E-06	GCST90277274
Phosphatidylethanolamine (18:1_18:1) levels	1	79358390	A	G	-1.70E-01	3.68E-02	5.90E-02	rs12137781	1_79358390_G_A	0.96	6866	3.83E-06	GCST90277274
Phosphatidylethanolamine (18:1_18:1) levels	1	201408310	G	A	1.17E-01	2.53E-02	8.64E-01	rs1262295	1_201408310_A_G	0.98	6866	3.86E-06	GCST90277274
Phosphatidylethanolamine (18:1_18:1) levels	2	27375230	C	T	-1.15E-01	1.75E-02	6.20E-01	rs4665972	2_27375230_T_C	1.00	6866	6.24E-11	GCST90277274
Phosphatidylethanolamine (18:1_18:1) levels	2	41532438	T	A	-7.90E-02	1.77E-02	3.66E-01	rs4952487	2_41532438_A_T	1.00	6866	8.30E-06	GCST90277274
Phosphatidylethanolamine (18:1_18:1) levels	2	163870869	T	G	7.99E-02	1.78E-02	3.64E-01	rs13418209	2_163870869_G_T	0.99	6866	7.65E-06	GCST90277274
Phosphatidylethanolamine (18:1_18:1) levels	3	68279920	C	T	2.77E-01	6.26E-02	2.03E-02	rs78880935	3_68279920_T_C	0.93	6866	9.75E-06	GCST90277274
Phosphatidylethanolamine (18:1_18:1) levels	3	139875064	C	G	-9.70E-02	2.15E-02	1.94E-01	rs4683767	3_139875064_G_C	1.00	6866	6.52E-06	GCST90277274
Phosphatidylethanolamine (18:1_18:1) levels	6	8749035	A	G	2.20E-01	4.94E-02	3.06E-02	rs74920877	6_8749035_G_A	0.98	6866	8.64E-06	GCST90277274
Phosphatidylethanolamine (18:1_18:1) levels	7	1103491	T	C	1.23E-01	2.64E-02	1.20E-01	rs12113381	7_1103491_C_T	1.00	6866	3.24E-06	GCST90277274
Phosphatidylethanolamine (18:1_18:1) levels	8	37124499	G	A	-1.31E-01	2.79E-02	8.95E-01	rs500246	8_37124499_A_G	0.98	6866	2.98E-06	GCST90277274
Phosphatidylethanolamine (18:1_18:1) levels	10	41829153	G	C	-1.25E-01	2.80E-02	1.10E-01	rs9663572	10_41829153_C_G	0.96	6866	8.13E-06	GCST90277274
Phosphatidylethanolamine (18:1_18:1) levels	11	61803876	G	C	1.84E-01	1.75E-02	3.84E-01	rs174548	11_61803876_C_G	1.00	6866	1.15E-25	GCST90277274
Phosphatidylethanolamine (18:1_18:1) levels	13	65750016	A	C	2.48E-01	5.50E-02	2.54E-02	rs144154975	13_65750016_C_A	0.98	6866	6.56E-06	GCST90277274
Phosphatidylethanolamine (18:1_18:1) levels	14	95471324	C	G	8.76E-02	1.97E-02	2.74E-01	rs8015793	14_95471324_G_C	0.93	6866	8.53E-06	GCST90277274
Phosphatidylethanolamine (18:1_18:1) levels	15	71629500	A	G	-8.12E-02	1.77E-02	4.83E-01	rs950668	15_71629500_G_A	0.91	6866	4.50E-06	GCST90277274
Phosphatidylethanolamine (18:1_18:1) levels	15	97139522	T	C	-8.34E-02	1.86E-02	6.60E-01	rs11853492	15_97139522_C_T	0.93	6866	7.13E-06	GCST90277274
Phosphatidylethanolamine (18:1_18:1) levels	17	79455291	T	C	1.10E-01	2.47E-02	1.40E-01	rs55771840	17_79455291_C_T	0.98	6866	8.48E-06	GCST90277274
Phosphatidylethanolamine (18:1_18:1) levels	18	21040292	A	C	3.31E-01	5.14E-02	2.88E-02	rs17202368	18_21040292_C_A	0.99	6866	1.29E-10	GCST90277274
Phosphatidylethanolamine (18:1_18:1) levels	18	21887951	T	A	3.54E-01	7.57E-02	1.35E-02	rs150543432	18_21887951_A_T	0.95	6866	3.05E-06	GCST90277274
Phosphatidylethanolamine (18:1_18:1) levels	18	52193653	G	C	9.52E-02	2.13E-02	7.88E-01	rs2044350	18_52193653_C_G	0.98	6866	7.87E-06	GCST90277274
Phosphatidylethanolamine (18:1_18:1) levels	19	47816733	T	C	-3.98E-01	8.89E-02	9.83E-03	rs11668274	19_47816733_C_T	0.93	6866	7.89E-06	GCST90277274
Phosphatidylethanolamine (18:1_18:1) levels	20	1953902	G	A	9.17E-02	1.83E-02	3.24E-01	rs6045639	20_1953902_A_G	0.99	6866	5.20E-07	GCST90277274
Phosphatidylethanolamine (18:1_18:1) levels	20	44413724	T	C	-1.71E-01	3.82E-02	5.24E-02	rs1800961	20_44413724_C_T	1.00	6866	8.08E-06	GCST90277274
Phosphatidylethanolamine (18:1_18:1) levels	20	62232970	T	C	-8.27E-02	1.73E-02	4.19E-01	rs6089695	20_62232970_C_T	1.00	6866	1.79E-06	GCST90277274
Phosphatidylethanolamine (18:1_18:1) levels	21	37359926	C	T	8.81E-02	1.69E-02	5.06E-01	rs7281206	21_37359926_T_C	1.00	6866	1.93E-07	GCST90277274
Phosphatidylethanolamine (O-16:1_18:2) levels	1	23639339	T	C	-9.80E-02	2.11E-02	2.27E-01	rs10917404	1_23639339_C_T	1.00	6468	3.32E-06	GCST90277275
Phosphatidylethanolamine (O-16:1_18:2) levels	2	27508073	C	T	-9.74E-02	1.84E-02	6.51E-01	rs1260326	2_27508073_T_C	1.00	6468	1.22E-07	GCST90277275
Phosphatidylethanolamine (O-16:1_18:2) levels	5	86717236	T	C	-4.08E-01	8.94E-02	9.82E-03	rs41441450	5_86717236_C_T	0.99	6468	5.08E-06	GCST90277275
Phosphatidylethanolamine (O-16:1_18:2) levels	5	133494602	A	G	-1.26E-01	2.80E-02	1.14E-01	rs13161129	5_133494602_G_A	0.96	6468	6.63E-06	GCST90277275
Phosphatidylethanolamine (O-16:1_18:2) levels	5	174328650	C	T	1.34E-01	2.77E-02	1.27E-01	rs6866162	5_174328650_T_C	0.92	6468	1.34E-06	GCST90277275
Phosphatidylethanolamine (O-16:1_18:2) levels	6	21056212	A	G	-1.07E-01	2.31E-02	1.75E-01	rs142477396	6_21056212_G_A	0.99	6468	3.77E-06	GCST90277275
Phosphatidylethanolamine (O-16:1_18:2) levels	6	32826204	C	A	-9.80E-02	2.20E-02	8.08E-01	rs1044043	6_32826204_A_C	1.00	6468	8.88E-06	GCST90277275
Phosphatidylethanolamine (O-16:1_18:2) levels	9	15305380	C	G	1.17E-01	2.49E-02	8.56E-01	rs581080	9_15305380_G_C	1.00	6468	2.75E-06	GCST90277275
Phosphatidylethanolamine (O-16:1_18:2) levels	10	71286664	C	T	-2.52E-01	5.67E-02	2.68E-02	rs77392374	10_71286664_T_C	0.93	6468	9.28E-06	GCST90277275
Phosphatidylethanolamine (O-16:1_18:2) levels	12	4009151	A	G	-1.15E-01	2.59E-02	8.66E-01	rs4238010	12_4009151_G_A	1.00	6468	9.44E-06	GCST90277275
Phosphatidylethanolamine (O-16:1_18:2) levels	12	34480655	T	C	1.34E-01	3.01E-02	9.72E-02	rs117207178	12_34480655_C_T	0.97	6468	8.39E-06	GCST90277275
Phosphatidylethanolamine (O-16:1_18:2) levels	15	38647674	A	T	8.10E-02	1.76E-02	4.61E-01	rs12916925	15_38647674_T_A	1.00	6468	4.41E-06	GCST90277275
Phosphatidylethanolamine (O-16:1_18:2) levels	15	58391167	G	A	-8.94E-02	1.77E-02	5.73E-01	rs1532085	15_58391167_A_G	1.00	6468	4.67E-07	GCST90277275
Phosphatidylethanolamine (O-16:1_18:2) levels	15	93906166	C	T	4.76E-01	1.07E-01	6.84E-03	rs75922719	15_93906166_T_C	0.95	6468	8.01E-06	GCST90277275
Phosphatidylethanolamine (O-16:1_18:2) levels	16	11827406	C	T	8.57E-02	1.78E-02	5.70E-01	rs11075022	16_11827406_T_C	1.00	6468	1.60E-06	GCST90277275
Phosphatidylethanolamine (O-16:1_18:2) levels	16	19425616	A	G	-2.47E-01	5.52E-02	2.65E-02	rs138445434	16_19425616_G_A	0.97	6468	7.74E-06	GCST90277275

Phosphatidylethanolamine (O-16:1_18:2) levels	16	54459960	T	C	-1.30E-01	2.71E-02	1.22E-01	rs72815248	16_54459960_C_T	0.96	6468	1.66E-06	GCST90277275
Phosphatidylethanolamine (O-16:1_18:2) levels	16	88735092	A	G	-4.31E-01	7.96E-02	1.28E-02	rs12926159	16_88735092_G_A	0.96	6468	6.10E-08	GCST90277275
Phosphatidylethanolamine (O-16:1_18:2) levels	20	62310086	A	C	-9.46E-02	1.80E-02	3.99E-01	rs2379129	20_62310086_C_A	0.99	6468	1.58E-07	GCST90277275
Phosphatidylethanolamine (O-16:1_18:2) levels	21	23717899	A	G	-3.37E-01	7.53E-02	1.60E-02	rs73897494	21_23717899_G_A	0.89	6468	7.88E-06	GCST90277275
Phosphatidylethanolamine (O-16:1_20:4) levels	1	62497063	G	A	1.07E-01	1.96E-02	7.38E-01	rs1168036	1_62497063_A_G	1.00	6639	4.78E-08	GCST90277276
Phosphatidylethanolamine (O-16:1_20:4) levels	2	41471833	C	G	-8.35E-02	1.82E-02	3.49E-01	rs1911704	2_41471833_G_C	1.00	6639	4.65E-06	GCST90277276
Phosphatidylethanolamine (O-16:1_20:4) levels	2	156288948	A	G	2.55E-01	5.73E-02	2.35E-02	rs115852609	2_156288948_G_A	0.99	6639	8.90E-06	GCST90277276
Phosphatidylethanolamine (O-16:1_20:4) levels	2	213698079	C	A	3.47E-01	7.55E-02	1.56E-02	rs114498622	2_213698079_A_C	0.88	6639	4.32E-06	GCST90277276
Phosphatidylethanolamine (O-16:1_20:4) levels	3	11166089	A	C	2.12E-01	4.77E-02	3.50E-02	rs13063467	3_11166089_C_A	1.00	6639	9.06E-06	GCST90277276
Phosphatidylethanolamine (O-16:1_20:4) levels	3	24997572	A	T	-8.43E-02	1.73E-02	4.79E-01	rs6769712	3_24997572_T_A	1.00	6639	1.07E-06	GCST90277276
Phosphatidylethanolamine (O-16:1_20:4) levels	3	26848389	G	T	8.47E-02	1.83E-02	3.50E-01	rs1488201	3_26848389_T_G	0.99	6639	3.60E-06	GCST90277276
Phosphatidylethanolamine (O-16:1_20:4) levels	4	136284098	A	G	1.32E-01	2.92E-02	9.89E-02	rs11722429	4_136284098_G_A	0.98	6639	5.94E-06	GCST90277276
Phosphatidylethanolamine (O-16:1_20:4) levels	4	169278135	A	C	-1.22E-01	2.60E-02	1.30E-01	rs13101957	4_169278135_C_A	0.98	6639	2.89E-06	GCST90277276
Phosphatidylethanolamine (O-16:1_20:4) levels	5	16862310	C	T	-1.87E-01	4.05E-02	5.24E-02	rs17614174	5_16862310_T_C	0.92	6639	3.75E-06	GCST90277276
Phosphatidylethanolamine (O-16:1_20:4) levels	6	73220880	A	G	2.47E-01	5.13E-02	3.15E-02	rs56144603	6_73220880_G_A	0.99	6639	1.42E-06	GCST90277276
Phosphatidylethanolamine (O-16:1_20:4) levels	6	114209369	C	T	6.46E-01	1.38E-01	4.42E-03	rs139075653	6_114209369_T_C	0.94	6639	3.04E-06	GCST90277276
Phosphatidylethanolamine (O-16:1_20:4) levels	6	139585552	G	T	2.99E-01	6.45E-02	1.82E-02	rs184289628	6_139585552_T_G	0.97	6639	3.67E-06	GCST90277276
Phosphatidylethanolamine (O-16:1_20:4) levels	7	6530143	A	T	9.31E-02	1.88E-02	3.23E-01	rs12113214	7_6530143_T_A	0.95	6639	7.34E-07	GCST90277276
Phosphatidylethanolamine (O-16:1_20:4) levels	8	22701002	G	A	-8.05E-02	1.80E-02	6.22E-01	rs6558177	8_22701002_A_G	0.99	6639	8.36E-06	GCST90277276
Phosphatidylethanolamine (O-16:1_20:4) levels	8	51606091	T	G	4.08E-01	9.03E-02	9.05E-03	rs142336498	8_51606091_G_T	0.98	6639	6.36E-06	GCST90277276
Phosphatidylethanolamine (O-16:1_20:4) levels	8	64641039	T	C	-4.78E-01	1.00E-01	8.45E-03	rs77404354	8_64641039_C_T	0.90	6639	1.84E-06	GCST90277276
Phosphatidylethanolamine (O-16:1_20:4) levels	8	65661212	A	G	-2.62E-01	5.33E-02	2.68E-02	rs13281002	8_65661212_G_A	0.96	6639	9.59E-07	GCST90277276
Phosphatidylethanolamine (O-16:1_20:4) levels	9	13548159	TG	T	2.05E-01	3.98E-02	4.94E-02	rs35745803	9_13548159_T_TG	1.00	6639	2.77E-07	GCST90277276
Phosphatidylethanolamine (O-16:1_20:4) levels	9	132450788	T	A	8.84E-02	1.93E-02	2.86E-01	rs551412	9_132450788_A_T	0.99	6639	4.64E-06	GCST90277276
Phosphatidylethanolamine (O-16:1_20:4) levels	10	5872836	A	G	-1.03E-01	2.24E-02	8.00E-01	rs4335452	10_5872836_G_A	0.95	6639	3.88E-06	GCST90277276
Phosphatidylethanolamine (O-16:1_20:4) levels	10	26331627	G	A	-1.31E-01	2.61E-02	1.27E-01	rs35960238	10_26331627_A_G	0.99	6639	5.37E-07	GCST90277276
Phosphatidylethanolamine (O-16:1_20:4) levels	10	44350663	C	T	2.93E-01	6.46E-02	1.84E-02	rs76916318	10_44350663_T_C	0.99	6639	5.76E-06	GCST90277276
Phosphatidylethanolamine (O-16:1_20:4) levels	10	63094343	A	G	9.46E-02	2.13E-02	2.08E-01	rs7920171	10_63094343_G_A	1.00	6639	8.96E-06	GCST90277276
Phosphatidylethanolamine (O-16:1_20:4) levels	11	61770929	C	G	1.02E-01	1.78E-02	4.44E-01	rs174527	11_61770929_G_C	0.98	6639	9.33E-09	GCST90277276
Phosphatidylethanolamine (O-16:1_20:4) levels	11	61824890	G	A	3.17E-01	1.73E-02	4.08E-01	rs174566	11_61824890_A_G	1.00	6639	1.46E-73	GCST90277276
Phosphatidylethanolamine (O-16:1_20:4) levels	11	62454004	A	G	-1.01E-01	2.06E-02	2.56E-01	rs4382917	11_62454004_G_A	0.92	6639	9.84E-07	GCST90277276
Phosphatidylethanolamine (O-16:1_20:4) levels	11	79640107	G	A	-7.79E-02	1.74E-02	5.61E-01	rs639956	11_79640107_A_G	1.00	6639	8.06E-06	GCST90277276
Phosphatidylethanolamine (O-16:1_20:4) levels	11	116778201	C	G	-1.41E-01	2.41E-02	8.49E-01	rs964184	11_116778201_G_C	1.00	6639	5.34E-09	GCST90277276
Phosphatidylethanolamine (O-16:1_20:4) levels	12	62657284	C	T	-1.21E-01	2.73E-02	1.13E-01	rs11174583	12_62657284_T_C	0.99	6639	9.96E-06	GCST90277276
Phosphatidylethanolamine (O-16:1_20:4) levels	12	130443132	A	G	-3.83E-01	8.35E-02	1.15E-02	rs75097472	12_130443132_G_A	0.88	6639	4.65E-06	GCST90277276
Phosphatidylethanolamine (O-16:1_20:4) levels	13	106475436	C	T	-1.32E-01	2.81E-02	1.19E-01	rs16968712	13_106475436_T_C	0.91	6639	2.63E-06	GCST90277276
Phosphatidylethanolamine (O-16:1_20:4) levels	15	58386313	T	C	1.12E-01	1.81E-02	3.38E-01	rs10468017	15_58386313_C_T	1.00	6639	7.41E-10	GCST90277276
Phosphatidylethanolamine (O-16:1_20:4) levels	15	58433640	G	A	9.30E-02	1.89E-02	3.14E-01	rs633695	15_58433640_A_G	1.00	6639	9.28E-07	GCST90277276
Phosphatidylethanolamine (O-16:1_20:4) levels	15	99707535	T	C	9.75E-02	2.14E-02	7.91E-01	rs325406	15_99707535_C_T	0.99	6639	5.19E-06	GCST90277276
Phosphatidylethanolamine (O-16:1_20:4) levels	16	14348932	T	C	-7.88E-02	1.77E-02	4.26E-01	rs60071820	16_14348932_C_T	0.99	6639	8.51E-06	GCST90277276
Phosphatidylethanolamine (O-16:1_20:4) levels	16	15051395	T	C	1.11E-01	1.90E-02	2.99E-01	rs55727637	16_15051395_C_T	1.00	6639	5.22E-09	GCST90277276
Phosphatidylethanolamine (O-16:1_20:4) levels	16	56960616	T	C	1.43E-01	1.92E-02	2.78E-01	rs17231506	16_56960616_C_T	1.00	6639	1.20E-13	GCST90277276
Phosphatidylethanolamine (O-16:1_20:4) levels	17	2780136	T	C	1.07E-01	2.41E-02	1.59E-01	rs12938196	17_2780136_C_T	0.96	6639	9.69E-06	GCST90277276
Phosphatidylethanolamine (O-16:1_20:4) levels	20	7847799	T	C	5.68E-01	1.28E-01	5.24E-03	rs146108252	20_7847799_C_T	0.82	6639	9.11E-06	GCST90277276
Phosphatidylethanolamine (O-16:1_20:4) levels	22	33757979	T	C	1.71E-01	3.84E-02	9.46E-01	rs483528	22_33757979_C_T	0.99	6639	9.00E-06	GCST90277276
Phosphatidylethanolamine (O-16:1_22:5) levels	1	5899241	T	A	9.06E-02	1.88E-02	2.59E-01	rs56270325	1_5899241_A_T	1.00	7174	1.49E-06	GCST90277277
Phosphatidylethanolamine (O-16:1_22:5) levels	1	55039974	T	G	-2.34E-01	4.66E-02	3.32E-02	rs11591147	1_55039974_G_T	1.00	7174	4.94E-07	GCST90277277
Phosphatidylethanolamine (O-16:1_22:5) levels	1	92970296	C	T	2.89E-01	5.76E-02	2.08E-02	rs148444916	1_92970296_T_C	0.99	7174	5.67E-07	GCST90277277
Phosphatidylethanolamine (O-16:1_22:5) levels	1	158505761	C	T	-1.73E-01	3.88E-02	4.84E-02	rs75023439	1_158505761_T_C	0.99	7174	8.49E-06	GCST90277277
Phosphatidylethanolamine (O-16:1_22:5) levels	2	21041028	A	G	8.38E-02	1.84E-02	2.85E-01	rs1367117	2_21041028_G_A	1.00	7174	5.33E-06	GCST90277277
Phosphatidylethanolamine (O-16:1_22:5) levels	3	186944804	G	T	1.86E-01	3.76E-02	9.48E-01	rs13060992	3_186944804_T_G	0.98	7174	7.57E-07	GCST90277277
Phosphatidylethanolamine (O-16:1_22:5) levels	4	110207431	T	C	-9.74E-02	2.12E-02	7.97E-01	rs5022521	4_110207431_C_T	0.94	7174	4.26E-06	GCST90277277
Phosphatidylethanolamine (O-16:1_22:5) levels	5	85099087	G	A	-1.07E-01	2.34E-02	1.58E-01	rs3936021	5_85099087_A_G	0.97	7174	4.96E-06	GCST90277277
Phosphatidylethanolamine (O-16:1_22:5) levels	5	159237696	A	G	8.12E-02	1.74E-02	3.47E-01	rs12522374	5_159237696_G_A	1.00	7174	3.11E-06	GCST90277277
Phosphatidylethanolamine (O-16:1_22:5) levels	6	32159700	A	C	9.67E-02	1.68E-02	5.83E-01	rs3134950	6_32159700_C_A	1.00	7174	9.96E-09	GCST90277277
Phosphatidylethanolamine (O-16:1_22:5) levels	6	123327335	G	A	1.44E-01	3.00E-02	8.22E-02	rs72974647	6_123327335_A_G	1.00	7174	1.61E-06	GCST90277277
Phosphatidylethanolamine (O-16:1_22:5) levels	7	17674637	G	C	8.41E-02	1.85E-02	7.19E-01	rs7780296	7_17674637_C_G	0.99	7174	5.57E-06	GCST90277277

Phosphatidylethanolamine (O-16:1_22:5) levels	7	154679558	G	A	-8.25E-02	1.86E-02	2.76E-01	rs3807226	7_154679558_A_G	0.98	7174	9.57E-06	GCST90277277
Phosphatidylethanolamine (O-16:1_22:5) levels	9	15296036	A	C	1.25E-01	2.61E-02	8.90E-01	rs643531	9_15296036_C_A	1.00	7174	1.91E-06	GCST90277277
Phosphatidylethanolamine (O-16:1_22:5) levels	9	69669802	C	A	9.07E-01	1.92E-01	9.98E-01	rs4744927	9_69669802_A_C	0.86	7174	2.35E-06	GCST90277277
Phosphatidylethanolamine (O-16:1_22:5) levels	9	80936784	G	A	1.01E-01	2.08E-02	1.98E-01	rs72733996	9_80936784_A_G	1.00	7174	1.23E-06	GCST90277277
Phosphatidylethanolamine (O-16:1_22:5) levels	9	136677616	G	C	-8.39E-02	1.77E-02	6.47E-01	rs2236514	9_136677616_C_G	0.95	7174	2.32E-06	GCST90277277
Phosphatidylethanolamine (O-16:1_22:5) levels	11	61802358	T	C	-1.49E-01	1.69E-02	4.07E-01	rs174546	11_61802358_C_T	1.00	7174	1.69E-18	GCST90277277
Phosphatidylethanolamine (O-16:1_22:5) levels	14	54570145	A	G	-1.42E-01	3.00E-02	9.16E-01	rs1187583	14_54570145_G_A	1.00	7174	2.41E-06	GCST90277277
Phosphatidylethanolamine (O-16:1_22:5) levels	15	58386521	C	T	-1.39E-01	1.69E-02	6.12E-01	rs261290	15_58386521_T_C	1.00	7174	2.37E-16	GCST90277277
Phosphatidylethanolamine (O-16:1_22:5) levels	15	58433640	G	A	9.11E-02	1.81E-02	3.14E-01	rs633695	15_58433640_A_G	1.00	7174	5.23E-07	GCST90277277
Phosphatidylethanolamine (O-16:1_22:5) levels	16	56960616	T	C	1.42E-01	1.85E-02	2.78E-01	rs17231506	16_56960616_C_T	1.00	7174	1.54E-14	GCST90277277
Phosphatidylethanolamine (O-16:1_22:5) levels	18	72301960	C	A	7.91E-02	1.74E-02	5.11E-01	rs9966610	18_72301960_A_C	0.92	7174	5.38E-06	GCST90277277
Phosphatidylethanolamine (O-16:1_22:5) levels	19	11082239	G	A	-1.58E-01	2.87E-02	9.21E-02	rs73015021	19_11082239_A_G	0.98	7174	3.49E-08	GCST90277277
Phosphatidylethanolamine (O-16:1_22:5) levels	22	40993330	A	G	-4.33E-01	9.45E-02	8.60E-03	rs78909142	22_40993330_G_A	0.94	7174	4.56E-06	GCST90277277
Phosphatidylethanolamine (O-18:1_18:2) levels	2	27508073	C	T	-1.08E-01	1.75E-02	6.51E-01	rs1260326	2_27508073_T_C	1.00	7172	6.12E-10	GCST90277278
Phosphatidylethanolamine (O-18:1_18:2) levels	2	167832629	T	C	1.19E-01	2.44E-02	1.44E-01	rs80227620	2_167832629_C_T	0.95	7172	1.19E-06	GCST90277278
Phosphatidylethanolamine (O-18:1_18:2) levels	4	17068686	C	A	1.09E-01	2.35E-02	1.46E-01	rs28524821	4_17068686_A_C	1.00	7172	3.98E-06	GCST90277278
Phosphatidylethanolamine (O-18:1_18:2) levels	4	162453799	G	A	7.58E-02	1.70E-02	5.54E-01	rs2597486	4_162453799_A_G	0.99	7172	8.12E-06	GCST90277278
Phosphatidylethanolamine (O-18:1_18:2) levels	4	180227646	A	G	-1.85E-01	4.10E-02	4.53E-02	rs116681793	4_180227646_G_A	0.96	7172	7.01E-06	GCST90277278
Phosphatidylethanolamine (O-18:1_18:2) levels	5	86296114	T	C	8.83E-02	1.97E-02	7.65E-01	rs4629629	5_86296114_C_T	1.00	7172	7.25E-06	GCST90277278
Phosphatidylethanolamine (O-18:1_18:2) levels	6	138148100	T	G	3.08E-01	6.61E-02	1.70E-02	rs113366516	6_138148100_G_T	0.93	7172	3.14E-06	GCST90277278
Phosphatidylethanolamine (O-18:1_18:2) levels	8	303655	T	C	1.86E-01	4.21E-02	4.21E-02	rs117726731	8_303655_C_T	0.97	7172	9.99E-06	GCST90277278
Phosphatidylethanolamine (O-18:1_18:2) levels	9	32796762	G	A	-1.10E-01	2.32E-02	1.54E-01	rs1854434	9_32796762_A_G	1.00	7172	2.15E-06	GCST90277278
Phosphatidylethanolamine (O-18:1_18:2) levels	9	123419309	C	T	7.89E-02	1.70E-02	5.10E-01	rs10760286	9_123419309_T_C	0.97	7172	3.32E-06	GCST90277278
Phosphatidylethanolamine (O-18:1_18:2) levels	10	92767403	G	A	-9.62E-02	2.03E-02	2.14E-01	rs35235479	10_92767403_A_G	1.00	7172	2.24E-06	GCST90277278
Phosphatidylethanolamine (O-18:1_18:2) levels	16	6538604	C	G	1.94E-01	4.30E-02	4.19E-02	rs62015468	16_6538604_G_C	0.92	7172	6.47E-06	GCST90277278
Phosphatidylethanolamine (O-18:1_18:2) levels	16	11833509	T	C	7.87E-02	1.70E-02	5.75E-01	rs11647818	16_11833509_C_T	1.00	7172	3.68E-06	GCST90277278
Phosphatidylethanolamine (O-18:1_18:2) levels	16	59856268	C	G	-2.41E-01	5.22E-02	2.89E-02	rs77007318	16_59856268_G_C	0.89	7172	3.88E-06	GCST90277278
Phosphatidylethanolamine (O-18:1_18:2) levels	16	71310208	G	A	-1.19E-01	2.64E-02	1.12E-01	rs34832413	16_71310208_A_G	0.99	7172	6.08E-06	GCST90277278
Phosphatidylethanolamine (O-18:1_18:2) levels	17	16458446	A	G	-3.34E-01	7.28E-02	1.35E-02	rs117571757	17_16458446_G_A	0.97	7172	4.44E-06	GCST90277278
Phosphatidylethanolamine (O-18:1_18:2) levels	18	63512537	C	T	-1.42E-01	3.07E-02	7.97E-02	rs62098276	18_63512537_T_C	0.99	7172	3.80E-06	GCST90277278
Phosphatidylethanolamine (O-18:1_18:2) levels	18	78257046	C	T	1.24E-01	2.77E-02	1.05E-01	rs12960635	18_78257046_T_C	0.96	7172	7.73E-06	GCST90277278
Phosphatidylethanolamine (O-18:1_20:4) levels	1	61714683	G	T	-3.43E-01	7.70E-02	1.32E-02	rs138107736	1_61714683_T_G	0.93	7076	8.58E-06	GCST90277279
Phosphatidylethanolamine (O-18:1_20:4) levels	1	158095972	T	C	-8.36E-02	1.78E-02	3.56E-01	rs12749808	1_158095972_C_T	0.96	7076	2.75E-06	GCST90277279
Phosphatidylethanolamine (O-18:1_20:4) levels	2	30022181	C	T	8.11E-02	1.77E-02	6.63E-01	rs12475699	2_30022181_T_C	1.00	7076	4.86E-06	GCST90277279
Phosphatidylethanolamine (O-18:1_20:4) levels	4	122641012	T	G	2.58E-01	5.52E-02	2.38E-02	rs78395462	4_122641012_G_T	0.98	7076	3.17E-06	GCST90277279
Phosphatidylethanolamine (O-18:1_20:4) levels	5	7954664	G	C	7.74E-02	1.72E-02	5.05E-01	rs162448	5_7954664_C_G	0.96	7076	6.82E-06	GCST90277279
Phosphatidylethanolamine (O-18:1_20:4) levels	6	78337820	T	C	7.40E-02	1.66E-02	4.76E-01	rs818276	6_78337820_C_T	1.00	7076	8.73E-06	GCST90277279
Phosphatidylethanolamine (O-18:1_20:4) levels	7	10826150	A	G	-2.20E-01	4.92E-02	3.08E-02	rs80201535	7_10826150_G_A	0.97	7076	7.45E-06	GCST90277279
Phosphatidylethanolamine (O-18:1_20:4) levels	8	101050430	T	C	-9.77E-02	2.13E-02	2.02E-01	rs2122922	8_101050430_C_T	0.95	7076	4.61E-06	GCST90277279
Phosphatidylethanolamine (O-18:1_20:4) levels	10	95941250	T	C	-3.52E-01	7.29E-02	1.36E-02	rs116911263	10_95941250_C_T	0.99	7076	1.47E-06	GCST90277279
Phosphatidylethanolamine (O-18:1_20:4) levels	11	1607718	A	G	1.20E-01	2.66E-02	1.13E-01	rs117085626	11_1607718_G_A	1.00	7076	6.77E-06	GCST90277279
Phosphatidylethanolamine (O-18:1_20:4) levels	11	4687754	G	A	-2.47E-01	5.52E-02	2.43E-02	rs10500613	11_4687754_A_G	0.98	7076	7.81E-06	GCST90277279
Phosphatidylethanolamine (O-18:1_20:4) levels	11	63193288	A	T	1.91E-01	4.32E-02	4.03E-02	rs2317091	11_63193288_T_A	0.99	7076	9.83E-06	GCST90277279
Phosphatidylethanolamine (O-18:1_20:4) levels	12	52522822	T	C	-9.24E-02	2.07E-02	2.13E-01	rs10783517	12_52522822_C_T	0.99	7076	7.89E-06	GCST90277279
Phosphatidylethanolamine (O-18:1_20:4) levels	14	97495861	AT	A	-1.05E-01	2.32E-02	1.63E-01	rs111290564	14_97495861_A_AT	0.94	7076	5.95E-06	GCST90277279
Phosphatidylethanolamine (O-18:1_20:4) levels	15	23752030	T	G	1.57E-01	3.48E-02	6.22E-02	rs78418403	15_23752030_G_T	0.96	7076	6.23E-06	GCST90277279
Phosphatidylethanolamine (O-18:1_20:4) levels	15	58387985	T	C	8.22E-02	1.71E-02	3.93E-01	rs7177289	15_58387985_C_T	1.00	7076	1.53E-06	GCST90277279
Phosphatidylethanolamine (O-18:1_20:4) levels	15	91344956	G	T	9.29E-02	1.73E-02	6.08E-01	rs6496787	15_91344956_T_G	0.99	7076	8.27E-08	GCST90277279
Phosphatidylethanolamine (O-18:1_20:4) levels	17	11337105	A	G	9.94E-02	2.23E-02	1.73E-01	rs12602611	17_11337105_G_A	0.98	7076	8.10E-06	GCST90277279
Phosphatidylethanolamine (O-18:1_20:4) levels	17	38380397	A	G	-4.96E-01	1.04E-01	7.22E-03	rs41488845	17_38380397_G_A	0.91	7076	2.04E-06	GCST90277279
Phosphatidylethanolamine (O-18:1_20:4) levels	19	55189474	C	T	9.05E-02	1.99E-02	2.45E-01	rs7248028	19_55189474_T_C	0.97	7076	5.47E-06	GCST90277279
Phosphatidylethanolamine (O-18:1_20:4) levels	20	24390991	A	G	2.68E-01	5.80E-02	2.16E-02	rs16986523	20_24390991_G_A	1.00	7076	3.78E-06	GCST90277279
Phosphatidylethanolamine (O-18:1_20:4) levels	22	36934040	C	T	-7.50E-02	1.69E-02	5.86E-01	rs2075937	22_36934040_T_C	1.00	7076	9.73E-06	GCST90277279
Phosphatidylethanolamine (O-18:1_20:4) levels	22	44080894	T	C	1.85E-01	4.12E-02	4.92E-02	rs13054328	22_44080894_C_T	0.88	7076	6.81E-06	GCST90277279
Phosphatidylethanolamine (O-18:2_18:1) levels	1	62497063	G	A	8.99E-02	1.89E-02	7.38E-01	rs1168036	1_62497063_A_G	1.00	7154	1.99E-06	GCST90277280
Phosphatidylethanolamine (O-18:2_18:1) levels	1	95020123	G	A	-7.69E-02	1.70E-02	3.79E-01	rs2391389	1_95020123_A_G	1.00	7154	5.98E-06	GCST90277280
Phosphatidylethanolamine (O-18:2_18:1) levels	1	236035687	C	T	8.49E-02	1.75E-02	3.38E-01	rs4659619	1_236035687_T_C	1.00	7154	1.21E-06	GCST90277280

Phosphatidylethanolamine (O-18:2_18:1) levels	2	37366100	C	T	-9.80E-02	2.19E-02	1.81E-01	rs11903018	2_37366100_T_C	0.99	7154	7.88E-06	GCST90277280
Phosphatidylethanolamine (O-18:2_18:1) levels	2	68074558	C	A	-1.93E-01	3.86E-02	5.16E-02	rs12052409	2_68074558_A_C	0.98	7154	5.57E-07	GCST90277280
Phosphatidylethanolamine (O-18:2_18:1) levels	4	137812361	A	C	1.11E-01	2.27E-02	1.63E-01	rs72723967	4_137812361_C_A	0.99	7154	1.18E-06	GCST90277280
Phosphatidylethanolamine (O-18:2_18:1) levels	4	169304745	A	G	1.03E-01	2.12E-02	8.06E-01	rs11723712	4_169304745_G_A	1.00	7154	1.11E-06	GCST90277280
Phosphatidylethanolamine (O-18:2_18:1) levels	4	188521987	T	C	1.00E-01	2.26E-02	1.67E-01	rs13119269	4_188521987_C_T	0.99	7154	9.47E-06	GCST90277280
Phosphatidylethanolamine (O-18:2_18:1) levels	5	983129	C	G	-1.03E-01	2.04E-02	2.40E-01	rs62330208	5_983129_G_C	0.91	7154	4.69E-07	GCST90277280
Phosphatidylethanolamine (O-18:2_18:1) levels	5	63116092	G	A	-3.56E-01	7.86E-02	1.20E-02	rs113053792	5_63116092_A_G	0.93	7154	5.97E-06	GCST90277280
Phosphatidylethanolamine (O-18:2_18:1) levels	5	151030574	A	G	-7.61E-02	1.72E-02	6.04E-01	rs3924	5_151030574_G_A	1.00	7154	9.52E-06	GCST90277280
Phosphatidylethanolamine (O-18:2_18:1) levels	6	18721099	G	C	5.49E-01	1.24E-01	5.13E-03	rs139644986	6_18721099_C_G	0.94	7154	9.93E-06	GCST90277280
Phosphatidylethanolamine (O-18:2_18:1) levels	6	32660651	A	C	-1.23E-01	2.71E-02	1.05E-01	rs2854275	6_32660651_C_A	0.99	7154	6.23E-06	GCST90277280
Phosphatidylethanolamine (O-18:2_18:1) levels	6	123327335	G	A	1.43E-01	3.01E-02	8.22E-02	rs72974647	6_123327335_A_G	1.00	7154	1.91E-06	GCST90277280
Phosphatidylethanolamine (O-18:2_18:1) levels	6	147174911	G	C	3.02E-01	6.54E-02	1.75E-02	rs117495065	6_147174911_C_G	0.91	7154	4.02E-06	GCST90277280
Phosphatidylethanolamine (O-18:2_18:1) levels	6	150183580	T	C	2.03E-01	4.47E-02	3.69E-02	rs141165989	6_150183580_C_T	0.98	7154	5.59E-06	GCST90277280
Phosphatidylethanolamine (O-18:2_18:1) levels	7	17925463	T	C	3.26E-01	7.30E-02	1.35E-02	rs76779498	7_17925463_C_T	0.96	7154	8.13E-06	GCST90277280
Phosphatidylethanolamine (O-18:2_18:1) levels	7	122869647	A	G	-1.02E-01	2.17E-02	1.85E-01	rs2214660	7_122869647_G_A	0.99	7154	2.56E-06	GCST90277280
Phosphatidylethanolamine (O-18:2_18:1) levels	7	153170994	A	G	-3.38E-01	7.38E-02	1.36E-02	rs113076792	7_153170994_G_A	0.95	7154	4.84E-06	GCST90277280
Phosphatidylethanolamine (O-18:2_18:1) levels	8	9324101	A	G	1.11E-01	2.34E-02	8.50E-01	rs2126263	8_9324101_G_A	1.00	7154	2.30E-06	GCST90277280
Phosphatidylethanolamine (O-18:2_18:1) levels	9	12222686	G	C	1.04E-01	2.28E-02	8.37E-01	rs10738267	9_12222686_C_G	0.99	7154	5.61E-06	GCST90277280
Phosphatidylethanolamine (O-18:2_18:1) levels	9	94130323	G	C	-7.99E-02	1.81E-02	3.21E-01	rs7046115	9_94130323_C_G	0.98	7154	9.93E-06	GCST90277280
Phosphatidylethanolamine (O-18:2_18:1) levels	9	132230557	A	G	-1.03E-01	2.32E-02	1.71E-01	rs2274855	9_132230557_G_A	0.89	7154	9.51E-06	GCST90277280
Phosphatidylethanolamine (O-18:2_18:1) levels	10	12379924	A	G	-2.15E-01	4.84E-02	3.36E-02	rs2724782	10_12379924_G_A	0.94	7154	8.99E-06	GCST90277280
Phosphatidylethanolamine (O-18:2_18:1) levels	11	58479658	C	T	-7.04E-01	1.58E-01	2.99E-03	rs118179558	11_58479658_T_C	0.92	7154	8.26E-06	GCST90277280
Phosphatidylethanolamine (O-18:2_18:1) levels	11	61855668	T	C	1.46E-01	1.69E-02	4.19E-01	rs174601	11_61855668_C_T	1.00	7154	6.00E-18	GCST90277280
Phosphatidylethanolamine (O-18:2_18:1) levels	11	107889276	A	G	-1.17E-01	2.39E-02	8.60E-01	rs10890773	11_107889276_G_A	1.00	7154	1.02E-06	GCST90277280
Phosphatidylethanolamine (O-18:2_18:1) levels	12	4779414	T	C	2.20E-01	4.92E-02	3.02E-02	rs143975147	12_4779414_C_T	0.98	7154	8.09E-06	GCST90277280
Phosphatidylethanolamine (O-18:2_18:1) levels	15	58386313	T	C	8.77E-02	1.76E-02	3.38E-01	rs10468017	15_58386313_C_T	1.00	7154	6.14E-07	GCST90277280
Phosphatidylethanolamine (O-18:2_18:1) levels	15	58433640	G	A	8.12E-02	1.82E-02	3.14E-01	rs633695	15_58433640_A_G	1.00	7154	8.31E-06	GCST90277280
Phosphatidylethanolamine (O-18:2_18:1) levels	16	6589736	C	G	-1.12E-01	2.44E-02	1.33E-01	rs76413658	16_6589736_G_C	0.99	7154	4.73E-06	GCST90277280
Phosphatidylethanolamine (O-18:2_18:1) levels	16	56956804	A	C	9.19E-02	1.86E-02	2.78E-01	rs247617	16_56956804_C_A	1.00	7154	7.77E-07	GCST90277280
Phosphatidylethanolamine (O-18:2_18:1) levels	17	44758756	A	C	1.29E-01	2.88E-02	9.22E-02	rs11870991	17_44758756_C_A	1.00	7154	8.31E-06	GCST90277280
Phosphatidylethanolamine (O-18:2_18:1) levels	18	14429629	G	A	9.27E-02	2.08E-02	2.49E-01	rs62080593	18_14429629_A_G	0.86	7154	8.02E-06	GCST90277280
Phosphatidylethanolamine (O-18:2_18:1) levels	18	49649558	C	T	5.94E-01	1.25E-01	4.85E-03	rs117528775	18_49649558_T_C	0.97	7154	2.25E-06	GCST90277280
Phosphatidylethanolamine (O-18:2_18:1) levels	19	47242784	G	A	7.93E-02	1.70E-02	4.96E-01	rs10853781	19_47242784_A_G	0.97	7154	3.32E-06	GCST90277280
Phosphatidylethanolamine (O-18:2_18:1) levels	20	819559	A	C	-2.72E-01	5.67E-02	2.33E-02	rs662846	20_819559_C_A	0.93	7154	1.66E-06	GCST90277280
Phosphatidylethanolamine (O-18:2_18:1) levels	21	31954469	A	G	-7.95E-02	1.79E-02	3.72E-01	rs2833577	21_31954469_G_A	0.95	7154	8.64E-06	GCST90277280
Phosphatidylethanolamine (O-18:2_18:1) levels	22	27828806	G	A	-1.05E-01	2.36E-02	1.50E-01	rs6005648	22_27828806_A_G	0.97	7154	8.65E-06	GCST90277280
Phosphatidylethanolamine (O-18:2_18:2) levels	1	230167404	T	C	9.03E-02	1.99E-02	7.77E-01	rs10779836	1_230167404_C_T	1.00	7174	6.13E-06	GCST90277281
Phosphatidylethanolamine (O-18:2_18:2) levels	2	27508073	C	T	-1.01E-01	1.75E-02	6.51E-01	rs1260326	2_27508073_T_C	1.00	7174	6.89E-09	GCST90277281
Phosphatidylethanolamine (O-18:2_18:2) levels	3	186944804	G	T	1.73E-01	3.77E-02	9.48E-01	rs13060992	3_186944804_T_G	0.98	7174	4.50E-06	GCST90277281
Phosphatidylethanolamine (O-18:2_18:2) levels	4	123415224	T	C	8.91E-02	1.96E-02	2.34E-01	rs12650992	4_123415224_C_T	1.00	7174	5.70E-06	GCST90277281
Phosphatidylethanolamine (O-18:2_18:2) levels	5	109218889	A	C	5.77E-01	1.27E-01	4.62E-03	rs114925849	5_109218889_C_A	0.94	7174	5.74E-06	GCST90277281
Phosphatidylethanolamine (O-18:2_18:2) levels	5	165822116	G	A	9.52E-02	2.08E-02	2.02E-01	rs28807772	5_165822116_A_G	1.00	7174	4.72E-06	GCST90277281
Phosphatidylethanolamine (O-18:2_18:2) levels	5	181070075	T	C	-1.21E-01	2.72E-02	1.31E-01	rs76546858	5_181070075_C_T	0.83	7174	7.80E-06	GCST90277281
Phosphatidylethanolamine (O-18:2_18:2) levels	6	32238762	T	C	-1.01E-01	2.08E-02	2.00E-01	rs3134937	6_32238762_C_T	0.99	7174	1.17E-06	GCST90277281
Phosphatidylethanolamine (O-18:2_18:2) levels	8	303655	T	C	1.86E-01	4.20E-02	4.21E-02	rs117726731	8_303655_C_T	0.97	7174	9.94E-06	GCST90277281
Phosphatidylethanolamine (O-18:2_18:2) levels	8	6228839	C	G	-9.05E-02	1.84E-02	7.03E-01	rs4538911	8_6228839_G_C	0.99	7174	8.54E-07	GCST90277281
Phosphatidylethanolamine (O-18:2_18:2) levels	8	129226534	G	A	7.72E-02	1.73E-02	6.19E-01	rs7004301	8_129226534_A_G	1.00	7174	7.95E-06	GCST90277281
Phosphatidylethanolamine (O-18:2_18:2) levels	10	127538724	T	G	-8.16E-02	1.84E-02	6.99E-01	rs7912885	10_127538724_G_T	0.98	7174	9.28E-06	GCST90277281
Phosphatidylethanolamine (O-18:2_18:2) levels	12	19869115	T	C	-1.70E-01	3.60E-02	6.19E-02	rs116997061	12_19869115_C_T	0.91	7174	2.35E-06	GCST90277281
Phosphatidylethanolamine (O-18:2_18:2) levels	12	47105688	T	C	-7.65E-02	1.68E-02	4.30E-01	rs1541361	12_47105688_C_T	1.00	7174	5.63E-06	GCST90277281
Phosphatidylethanolamine (O-18:2_18:2) levels	15	58391167	G	A	-9.78E-02	1.68E-02	5.73E-01	rs1532085	15_58391167_A_G	1.00	7174	6.25E-09	GCST90277281
Phosphatidylethanolamine (O-18:2_18:2) levels	15	58433640	G	A	9.03E-02	1.82E-02	3.14E-01	rs633695	15_58433640_A_G	1.00	7174	6.82E-07	GCST90277281
Phosphatidylethanolamine (O-18:2_18:2) levels	16	56956804	A	C	9.75E-02	1.86E-02	2.78E-01	rs247617	16_56956804_C_A	1.00	7174	1.56E-07	GCST90277281
Phosphatidylethanolamine (O-18:2_18:2) levels	16	71259459	G	C	-1.19E-01	2.63E-02	1.12E-01	rs1593135	16_71259459_C_G	1.00	7174	6.52E-06	GCST90277281
Phosphatidylethanolamine (O-18:2_18:2) levels	17	43849174	A	G	1.85E-01	3.85E-02	4.96E-02	rs77027238	17_43849174_G_A	0.97	7174	1.54E-06	GCST90277281
Phosphatidylethanolamine (O-18:2_18:2) levels	20	58326890	TC	T	-2.44E-01	5.34E-02	2.66E-02	rs201201195	20_58326890_T_TC	0.94	7174	5.14E-06	GCST90277281
Phosphatidylethanolamine (O-18:2_20:4) levels	1	16249217	T	C	-4.39E-01	9.75E-02	7.93E-03	rs187681244	1_16249217_C_T	0.91	7174	7.01E-06	GCST90277282

Phosphatidylethanolamine (O-18:2_20:4) levels	1	33445467	C	T	2.72E-01	6.11E-02	2.02E-02	rs75833961	1_33445467_T_C	0.94	7174	8.43E-06	GCST90277282
Phosphatidylethanolamine (O-18:2_20:4) levels	1	62437688	C	T	-1.27E-01	1.88E-02	2.61E-01	rs9436221	1_62437688_T_C	1.00	7174	1.82E-11	GCST90277282
Phosphatidylethanolamine (O-18:2_20:4) levels	1	189911359	A	T	-8.05E-02	1.79E-02	3.09E-01	rs6673582	1_189911359_T_A	1.00	7174	7.04E-06	GCST90277282
Phosphatidylethanolamine (O-18:2_20:4) levels	2	137186441	T	A	1.64E-01	3.51E-02	5.97E-02	rs13402814	2_137186441_A_T	1.00	7174	2.89E-06	GCST90277282
Phosphatidylethanolamine (O-18:2_20:4) levels	3	3947204	A	G	-6.95E-01	1.48E-01	3.87E-03	rs2629180	3_3947204_G_A	0.83	7174	2.56E-06	GCST90277282
Phosphatidylethanolamine (O-18:2_20:4) levels	3	10308480	AGTC	A	-3.03E-01	6.81E-02	1.54E-02	rs150352222	3_10308480_A_AGTC	0.98	7174	8.89E-06	GCST90277282
Phosphatidylethanolamine (O-18:2_20:4) levels	3	26848389	G	T	7.81E-02	1.76E-02	3.50E-01	rs1488201	3_26848389_T_G	0.99	7174	8.86E-06	GCST90277282
Phosphatidylethanolamine (O-18:2_20:4) levels	4	136284098	A	G	1.33E-01	2.81E-02	9.89E-02	rs11722429	4_136284098_G_A	0.98	7174	2.18E-06	GCST90277282
Phosphatidylethanolamine (O-18:2_20:4) levels	4	169277659	A	G	-1.35E-01	2.50E-02	1.30E-01	rs13150924	4_169277659_G_A	0.98	7174	7.20E-08	GCST90277282
Phosphatidylethanolamine (O-18:2_20:4) levels	5	983129	C	G	-8.99E-02	2.03E-02	2.40E-01	rs62330208	5_983129_G_C	0.91	7174	9.97E-06	GCST90277282
Phosphatidylethanolamine (O-18:2_20:4) levels	5	52415905	G	A	-8.09E-02	1.79E-02	6.79E-01	rs7717591	5_52415905_A_G	1.00	7174	6.26E-06	GCST90277282
Phosphatidylethanolamine (O-18:2_20:4) levels	5	96128576	T	C	2.47E-01	5.46E-02	2.51E-02	rs78882831	5_96128576_C_T	0.97	7174	6.35E-06	GCST90277282
Phosphatidylethanolamine (O-18:2_20:4) levels	5	165816602	A	T	9.33E-02	2.05E-02	2.11E-01	rs4415100	5_165816602_T_A	1.00	7174	5.24E-06	GCST90277282
Phosphatidylethanolamine (O-18:2_20:4) levels	6	124913409	A	G	7.16E-01	1.56E-01	3.20E-03	rs112569946	6_124913409_G_A	0.89	7174	4.34E-06	GCST90277282
Phosphatidylethanolamine (O-18:2_20:4) levels	9	13564220	G	A	1.95E-01	3.88E-02	4.83E-02	rs10961143	9_13564220_A_G	0.99	7174	4.88E-07	GCST90277282
Phosphatidylethanolamine (O-18:2_20:4) levels	10	57406164	A	G	-9.32E-02	2.08E-02	2.00E-01	rs4412689	10_57406164_G_A	1.00	7174	7.42E-06	GCST90277282
Phosphatidylethanolamine (O-18:2_20:4) levels	10	116647273	A	G	-9.00E-02	1.75E-02	6.48E-01	rs10787711	10_116647273_G_A	0.99	7174	2.74E-07	GCST90277282
Phosphatidylethanolamine (O-18:2_20:4) levels	11	6068510	A	G	-1.39E-01	3.09E-02	7.91E-02	rs6578729	11_6068510_G_A	1.00	7174	7.32E-06	GCST90277282
Phosphatidylethanolamine (O-18:2_20:4) levels	11	61770929	C	G	1.07E-01	1.71E-02	4.44E-01	rs174527	11_61770929_G_C	0.98	7174	3.51E-10	GCST90277282
Phosphatidylethanolamine (O-18:2_20:4) levels	11	61832870	C	A	-3.31E-01	1.66E-02	5.91E-01	rs174574	11_61832870_A_C	1.00	7174	5.66E-86	GCST90277282
Phosphatidylethanolamine (O-18:2_20:4) levels	11	62451557	G	A	-9.93E-02	1.96E-02	2.57E-01	rs7936002	11_62451557_A_G	0.93	7174	4.27E-07	GCST90277282
Phosphatidylethanolamine (O-18:2_20:4) levels	11	63135902	A	G	2.45E-01	5.29E-02	2.57E-02	rs56075928	11_63135902_G_A	0.99	7174	3.85E-06	GCST90277282
Phosphatidylethanolamine (O-18:2_20:4) levels	11	107889276	A	G	-1.26E-01	2.38E-02	8.60E-01	rs10890773	11_107889276_G_A	1.00	7174	1.42E-07	GCST90277282
Phosphatidylethanolamine (O-18:2_20:4) levels	11	116778201	C	G	-1.58E-01	2.32E-02	8.49E-01	rs964184	11_116778201_G_C	1.00	7174	9.56E-12	GCST90277282
Phosphatidylethanolamine (O-18:2_20:4) levels	12	29702911	A	G	-2.90E-01	6.44E-02	1.80E-02	rs150376922	12_29702911_G_A	0.96	7174	6.88E-06	GCST90277282
Phosphatidylethanolamine (O-18:2_20:4) levels	12	52311207	A	G	-9.94E-02	2.08E-02	2.03E-01	rs17126640	12_52311207_G_A	1.00	7174	1.89E-06	GCST90277282
Phosphatidylethanolamine (O-18:2_20:4) levels	12	99432175	A	G	2.54E-01	5.52E-02	2.49E-02	rs143066473	12_99432175_G_A	0.94	7174	4.29E-06	GCST90277282
Phosphatidylethanolamine (O-18:2_20:4) levels	12	110067189	C	A	1.96E-01	4.33E-02	3.79E-02	rs73205054	12_110067189_A_C	0.99	7174	6.17E-06	GCST90277282
Phosphatidylethanolamine (O-18:2_20:4) levels	15	58391167	G	A	-1.11E-01	1.68E-02	5.73E-01	rs1532085	15_58391167_A_G	1.00	7174	4.61E-11	GCST90277282
Phosphatidylethanolamine (O-18:2_20:4) levels	15	58431476	T	C	1.38E-01	1.93E-02	2.58E-01	rs1800588	15_58431476_C_T	0.99	7174	1.09E-12	GCST90277282
Phosphatidylethanolamine (O-18:2_20:4) levels	16	6626446	T	C	1.57E-01	3.27E-02	7.15E-02	rs1833172	16_6626446_C_T	0.96	7174	1.60E-06	GCST90277282
Phosphatidylethanolamine (O-18:2_20:4) levels	16	15043593	A	G	1.16E-01	1.77E-02	3.32E-01	rs62039480	16_15043593_G_A	1.00	7174	6.52E-11	GCST90277282
Phosphatidylethanolamine (O-18:2_20:4) levels	16	56960616	T	C	1.44E-01	1.85E-02	2.78E-01	rs17231506	16_56960616_C_T	1.00	7174	8.35E-15	GCST90277282
Phosphatidylethanolamine (O-18:2_20:4) levels	18	49649558	C	T	5.97E-01	1.25E-01	4.85E-03	rs117528775	18_49649558_T_C	0.97	7174	1.92E-06	GCST90277282
Phosphatidylethanolamine (O-18:2_20:4) levels	19	3325829	C	T	-1.59E-01	3.49E-02	6.74E-02	rs35925446	19_3325829_T_C	0.90	7174	5.28E-06	GCST90277282
Phosphatidylethanolamine (O-18:2_20:4) levels	22	27818698	T	C	-1.50E-01	3.29E-02	7.28E-02	rs16985573	22_27818698_C_T	0.95	7174	5.43E-06	GCST90277282
Phosphatidylinositol (16:0_18:1) levels	1	98597981	T	C	2.63E-01	5.89E-02	2.34E-02	rs114797690	1_98597981_C_T	0.87	7174	7.71E-06	GCST90277283
Phosphatidylinositol (16:0_18:1) levels	2	27508073	C	T	-1.15E-01	1.74E-02	6.51E-01	rs1260326	2_27508073_T_C	1.00	7174	4.02E-11	GCST90277283
Phosphatidylinositol (16:0_18:1) levels	3	76641189	G	A	2.09E-01	4.51E-02	3.61E-02	rs147507697	3_76641189_A_G	0.99	7174	3.46E-06	GCST90277283
Phosphatidylinositol (16:0_18:1) levels	3	117112654	C	T	-1.12E-01	2.28E-02	8.18E-01	rs1474236	3_117112654_T_C	0.90	7174	8.21E-07	GCST90277283
Phosphatidylinositol (16:0_18:1) levels	5	62191215	G	A	4.31E-01	9.10E-02	9.08E-03	rs79189815	5_62191215_A_G	0.95	7174	2.19E-06	GCST90277283
Phosphatidylinositol (16:0_18:1) levels	5	176924035	C	T	-2.82E-01	5.60E-02	2.36E-02	rs115079643	5_176924035_T_C	0.95	7174	4.60E-07	GCST90277283
Phosphatidylinositol (16:0_18:1) levels	6	123212261	T	C	7.66E-02	1.69E-02	4.93E-01	rs55841968	6_123212261_C_T	0.99	7174	5.99E-06	GCST90277283
Phosphatidylinositol (16:0_18:1) levels	6	155156506	G	T	-7.89E-02	1.76E-02	6.55E-01	rs9397785	6_155156506_T_G	1.00	7174	7.36E-06	GCST90277283
Phosphatidylinositol (16:0_18:1) levels	8	6245562	T	G	-8.17E-02	1.79E-02	6.80E-01	rs5025214	8_6245562_G_T	1.00	7174	5.20E-06	GCST90277283
Phosphatidylinositol (16:0_18:1) levels	9	137735506	A	G	-1.14E-01	2.58E-02	8.69E-01	rs3125786	9_137735506_G_A	0.93	7174	9.39E-06	GCST90277283
Phosphatidylinositol (16:0_18:1) levels	10	60834796	G	A	-1.39E-01	2.84E-02	9.01E-01	rs12255873	10_60834796_A_G	0.96	7174	9.11E-07	GCST90277283
Phosphatidylinositol (16:0_18:1) levels	10	112224000	T	C	-1.08E-01	2.41E-02	1.40E-01	rs60847460	10_112224000_C_T	0.99	7174	7.20E-06	GCST90277283
Phosphatidylinositol (16:0_18:1) levels	11	61853722	A	G	1.12E-01	1.76E-02	3.46E-01	rs174598	11_61853722_G_A	1.00	7174	2.24E-10	GCST90277283
Phosphatidylinositol (16:0_18:1) levels	12	120977490	A	G	-7.65E-02	1.68E-02	5.47E-01	rs7139079	12_120977490_G_A	1.00	7174	5.11E-06	GCST90277283
Phosphatidylinositol (16:0_18:1) levels	15	58431476	T	C	8.94E-02	1.94E-02	2.58E-01	rs1800588	15_58431476_C_T	0.99	7174	3.95E-06	GCST90277283
Phosphatidylinositol (16:0_18:1) levels	16	56956804	A	C	1.07E-01	1.85E-02	2.78E-01	rs247617	16_56956804_C_A	1.00	7174	9.57E-09	GCST90277283
Phosphatidylinositol (16:0_18:1) levels	17	27754096	T	A	-2.55E-01	5.55E-02	2.34E-02	rs192646452	17_27754096_A_T	0.96	7174	4.52E-06	GCST90277283
Phosphatidylinositol (16:0_18:1) levels	17	60163403	G	A	9.77E-02	2.18E-02	1.89E-01	rs345182	17_60163403_A_G	0.94	7174	7.79E-06	GCST90277283
Phosphatidylinositol (16:0_18:1) levels	18	62042253	A	G	-5.43E-01	1.17E-01	5.58E-03	rs138477772	18_62042253_G_A	0.96	7174	3.27E-06	GCST90277283
Phosphatidylinositol (16:0_18:1) levels	19	19347579	G	A	-1.89E-01	3.78E-02	5.38E-02	rs182611493	19_19347579_A_G	0.95	7174	6.28E-07	GCST90277283
Phosphatidylinositol (16:0_18:1) levels	19	24009974	T	C	-1.98E-01	4.37E-02	3.81E-02	rs144573714	19_24009974_C_T	0.98	7174	6.05E-06	GCST90277283

Phosphatidylinositol (16:0_18:1) levels	20	1141294	C	T	2.43E-01	5.16E-02	2.77E-02	rs111506410	20_1141294_T_C	0.95	7174	2.62E-06	GCST90277283
Phosphatidylinositol (16:0_18:1) levels	22	23430310	A	G	2.81E-01	6.32E-02	1.95E-02	rs57021772	22_23430310_G_A	0.93	7174	9.16E-06	GCST90277283
Phosphatidylinositol (16:0_18:2) levels	2	13150132	A	G	-1.98E-01	4.36E-02	4.29E-02	rs147707008	2_13150132_G_A	0.95	6882	5.91E-06	GCST90277284
Phosphatidylinositol (16:0_18:2) levels	3	8759154	C	T	8.45E-02	1.76E-02	3.92E-01	rs2268493	3_8759154_T_C	0.98	6882	1.60E-06	GCST90277284
Phosphatidylinositol (16:0_18:2) levels	5	137344967	A	G	1.95E-01	3.95E-02	4.97E-02	rs150608914	5_137344967_G_A	0.99	6882	8.25E-07	GCST90277284
Phosphatidylinositol (16:0_18:2) levels	6	18747694	T	C	1.17E-01	2.50E-02	1.29E-01	rs73728261	6_18747694_C_T	1.00	6882	3.11E-06	GCST90277284
Phosphatidylinositol (16:0_18:2) levels	6	51400810	A	G	7.89E-02	1.72E-02	4.48E-01	rs1409892	6_51400810_G_A	1.00	6882	4.54E-06	GCST90277284
Phosphatidylinositol (16:0_18:2) levels	7	134906346	T	C	9.22E-02	1.76E-02	4.17E-01	rs12707188	7_134906346_C_T	0.98	6882	1.66E-07	GCST90277284
Phosphatidylinositol (16:0_18:2) levels	10	19302248	C	T	1.10E-01	2.43E-02	8.46E-01	rs2499085	10_19302248_T_C	0.94	6882	6.30E-06	GCST90277284
Phosphatidylinositol (16:0_18:2) levels	10	63087988	T	C	-8.43E-02	1.79E-02	6.50E-01	rs10995430	10_63087988_C_T	0.99	6882	2.61E-06	GCST90277284
Phosphatidylinositol (16:0_18:2) levels	10	82089541	G	A	-4.27E-01	9.53E-02	9.62E-03	rs10490936	10_82089541_A_G	0.83	6882	7.47E-06	GCST90277284
Phosphatidylinositol (16:0_18:2) levels	10	114348244	G	A	1.06E-01	2.31E-02	1.83E-01	rs17715191	10_114348244_A_G	0.92	6882	4.77E-06	GCST90277284
Phosphatidylinositol (16:0_18:2) levels	11	61852357	A	C	-1.15E-01	1.73E-02	5.82E-01	rs174594	11_61852357_C_A	1.00	6882	3.58E-11	GCST90277284
Phosphatidylinositol (16:0_18:2) levels	22	44667863	T	C	8.49E-02	1.84E-02	6.78E-01	rs6007233	22_44667863_C_T	0.99	6882	4.06E-06	GCST90277284
Phosphatidylinositol (16:0_20:4) levels	1	62494579	C	T	9.26E-02	1.89E-02	7.33E-01	rs1168041	1_62494579_T_C	0.99	7135	9.29E-07	GCST90277285
Phosphatidylinositol (16:0_20:4) levels	2	27508073	C	T	-9.72E-02	1.75E-02	6.51E-01	rs1260326	2_27508073_T_C	1.00	7135	3.00E-08	GCST90277285
Phosphatidylinositol (16:0_20:4) levels	2	56194921	G	A	-2.85E-01	6.01E-02	2.03E-02	rs17268633	2_56194921_A_G	0.99	7135	2.07E-06	GCST90277285
Phosphatidylinositol (16:0_20:4) levels	4	26558575	A	G	5.95E-01	1.23E-01	5.53E-03	rs78230994	4_26558575_G_A	0.88	7135	1.25E-06	GCST90277285
Phosphatidylinositol (16:0_20:4) levels	4	176627941	C	T	-2.77E-01	5.73E-02	2.16E-02	rs142392117	4_176627941_T_C	0.97	7135	1.40E-06	GCST90277285
Phosphatidylinositol (16:0_20:4) levels	6	32203298	G	A	-9.70E-02	2.17E-02	1.77E-01	rs3132935	6_32203298_A_G	1.00	7135	7.80E-06	GCST90277285
Phosphatidylinositol (16:0_20:4) levels	6	99968458	T	G	-1.14E-01	2.48E-02	1.33E-01	rs34516410	6_99968458_G_T	0.99	7135	4.16E-06	GCST90277285
Phosphatidylinositol (16:0_20:4) levels	7	23532125	T	C	3.09E-01	6.74E-02	1.61E-02	rs12700446	7_23532125_C_T	0.98	7135	4.73E-06	GCST90277285
Phosphatidylinositol (16:0_20:4) levels	9	136677616	G	C	-9.07E-02	1.78E-02	6.47E-01	rs2236514	9_136677616_C_G	0.95	7135	3.85E-07	GCST90277285
Phosphatidylinositol (16:0_20:4) levels	10	51792352	A	T	-7.77E-01	1.67E-01	2.99E-03	rs148069564	10_51792352_T_A	0.88	7135	3.28E-06	GCST90277285
Phosphatidylinositol (16:0_20:4) levels	10	65395218	A	T	-8.49E-02	1.85E-02	2.74E-01	rs72800299	10_65395218_T_A	0.99	7135	4.69E-06	GCST90277285
Phosphatidylinositol (16:0_20:4) levels	10	90294727	C	T	-1.05E-01	2.37E-02	1.50E-01	rs12761749	10_90294727_T_C	0.99	7135	9.93E-06	GCST90277285
Phosphatidylinositol (16:0_20:4) levels	11	61851136	G	A	2.62E-01	1.68E-02	4.18E-01	rs174592	11_61851136_A_G	1.00	7135	4.96E-54	GCST90277285
Phosphatidylinositol (16:0_20:4) levels	11	63193288	A	T	2.18E-01	4.30E-02	4.03E-02	rs2317091	11_63193288_T_A	0.99	7135	4.11E-07	GCST90277285
Phosphatidylinositol (16:0_20:4) levels	11	67075266	A	G	-3.66E-01	7.97E-02	1.12E-02	rs1815763	11_67075266_G_A	0.98	7135	4.39E-06	GCST90277285
Phosphatidylinositol (16:0_20:4) levels	11	75745010	C	T	-1.10E-01	1.99E-02	2.32E-01	rs10160784	11_75745010_T_C	0.99	7135	3.61E-08	GCST90277285
Phosphatidylinositol (16:0_20:4) levels	11	107569619	G	A	-1.13E-01	2.35E-02	1.55E-01	rs79718024	11_107569619_A_G	0.97	7135	1.76E-06	GCST90277285
Phosphatidylinositol (16:0_20:4) levels	11	124146158	C	G	3.09E-01	6.89E-02	1.57E-02	rs17128436	11_124146158_G_C	0.98	7135	7.29E-06	GCST90277285
Phosphatidylinositol (16:0_20:4) levels	12	52311207	A	G	-9.82E-02	2.09E-02	2.03E-01	rs17126640	12_52311207_G_A	1.00	7135	2.70E-06	GCST90277285
Phosphatidylinositol (16:0_20:4) levels	12	120978819	G	C	7.54E-02	1.68E-02	4.70E-01	rs1169289	12_120978819_C_G	0.99	7135	7.32E-06	GCST90277285
Phosphatidylinositol (16:0_20:4) levels	14	89719965	G	A	-1.97E-01	4.11E-02	4.30E-02	rs113130033	14_89719965_A_G	0.98	7135	1.73E-06	GCST90277285
Phosphatidylinositol (16:0_20:4) levels	15	58391167	G	A	-9.38E-02	1.69E-02	5.73E-01	rs1532085	15_58391167_A_G	1.00	7135	2.69E-08	GCST90277285
Phosphatidylinositol (16:0_20:4) levels	15	58431476	T	C	1.07E-01	1.94E-02	2.58E-01	rs1800588	15_58431476_C_T	0.99	7135	3.85E-08	GCST90277285
Phosphatidylinositol (16:0_20:4) levels	16	6506441	C	G	-2.11E-01	4.77E-02	3.16E-02	rs1640963	16_6506441_G_C	0.98	7135	9.76E-06	GCST90277285
Phosphatidylinositol (16:0_20:4) levels	16	13890509	G	A	3.93E-01	8.27E-02	1.23E-02	rs113550786	16_13890509_A_G	0.85	7135	2.06E-06	GCST90277285
Phosphatidylinositol (16:0_20:4) levels	18	8971803	A	G	-2.60E-01	5.60E-02	2.33E-02	rs76711488	18_8971803_G_A	0.98	7135	3.60E-06	GCST90277285
Phosphatidylinositol (16:0_20:4) levels	18	26802732	T	C	3.89E-01	6.77E-02	1.77E-02	rs62081852	18_26802732_C_T	0.91	7135	9.35E-09	GCST90277285
Phosphatidylinositol (16:0_20:4) levels	19	19210016	G	C	-2.02E-01	3.87E-02	5.22E-02	rs150057262	19_19210016_C_G	0.94	7135	1.77E-07	GCST90277285
Phosphatidylinositol (16:0_20:4) levels	22	44572650	A	G	8.14E-02	1.84E-02	7.09E-01	rs138597	22_44572650_G_A	1.00	7135	9.82E-06	GCST90277285
Phosphatidylinositol (18:0_18:1) levels	1	62494579	C	T	8.93E-02	1.88E-02	7.33E-01	rs1168041	1_62494579_T_C	0.99	7174	2.10E-06	GCST90277286
Phosphatidylinositol (18:0_18:1) levels	2	27508073	C	T	-7.74E-02	1.75E-02	6.51E-01	rs1260326	2_27508073_T_C	1.00	7174	9.67E-06	GCST90277286
Phosphatidylinositol (18:0_18:1) levels	2	126127979	C	A	-3.07E-01	6.73E-02	1.71E-02	rs77990460	2_126127979_A_C	0.92	7174	5.35E-06	GCST90277286
Phosphatidylinositol (18:0_18:1) levels	5	114892988	A	G	6.78E-01	1.49E-01	3.33E-03	rs143838492	5_114892988_G_A	0.97	7174	5.19E-06	GCST90277286
Phosphatidylinositol (18:0_18:1) levels	5	120061007	G	A	3.80E-01	8.30E-02	1.02E-02	rs141883259	5_120061007_A_G	0.98	7174	4.89E-06	GCST90277286
Phosphatidylinositol (18:0_18:1) levels	6	156361355	A	G	-1.32E-01	2.91E-02	9.32E-02	rs151287892	6_156361355_G_A	0.95	7174	6.19E-06	GCST90277286
Phosphatidylinositol (18:0_18:1) levels	7	146970602	T	C	3.14E-01	6.90E-02	1.52E-02	rs372466687	7_146970602_C_T	0.98	7174	5.58E-06	GCST90277286
Phosphatidylinositol (18:0_18:1) levels	8	39183266	G	C	8.65E-02	1.84E-02	2.93E-01	rs10102672	8_39183266_C_G	1.00	7174	2.57E-06	GCST90277286
Phosphatidylinositol (18:0_18:1) levels	9	71015708	T	C	-4.02E-01	8.82E-02	9.43E-03	rs117372257	9_71015708_C_T	0.99	7174	5.23E-06	GCST90277286
Phosphatidylinositol (18:0_18:1) levels	10	90245408	T	C	7.92E-02	1.70E-02	5.05E-01	rs1410901	10_90245408_C_T	0.94	7174	3.24E-06	GCST90277286
Phosphatidylinositol (18:0_18:1) levels	11	61828092	T	C	2.57E-01	2.64E-02	1.13E-01	rs968567	11_61828092_C_T	1.00	7174	3.05E-22	GCST90277286
Phosphatidylinositol (18:0_18:1) levels	12	43610337	T	C	1.83E-01	3.98E-02	4.71E-02	rs138705401	12_43610337_C_T	0.98	7174	4.48E-06	GCST90277286
Phosphatidylinositol (18:0_18:1) levels	13	66958436	C	T	-2.00E-01	4.39E-02	3.70E-02	rs77419701	13_66958436_T_C	0.99	7174	5.36E-06	GCST90277286
Phosphatidylinositol (18:0_18:1) levels	13	93173799	C	G	8.71E-02	1.93E-02	2.55E-01	rs319552	13_93173799_G_C	0.96	7174	6.23E-06	GCST90277286

Phosphatidylinositol (18:0_18:1) levels	15	92337647	C	T	-8.39E-02	1.81E-02	6.74E-01	rs7173306	15_92337647_T_C	0.95	7174	3.78E-06	GCST90277286
Phosphatidylinositol (18:0_18:1) levels	16	15036737	G	A	-1.92E-01	1.76E-02	3.34E-01	rs6498540	16_15036737_A_G	0.99	7174	1.90E-27	GCST90277286
Phosphatidylinositol (18:0_18:1) levels	16	15773795	G	T	8.34E-02	1.84E-02	2.96E-01	rs56374730	16_15773795_T_G	0.99	7174	5.80E-06	GCST90277286
Phosphatidylinositol (18:0_18:1) levels	17	51561964	T	C	-2.34E-01	5.25E-02	2.75E-02	rs116917626	17_51561964_C_T	0.97	7174	8.10E-06	GCST90277286
Phosphatidylinositol (18:0_18:1) levels	19	19210016	G	C	-2.00E-01	3.87E-02	5.22E-02	rs150057262	19_19210016_C_G	0.94	7174	2.39E-07	GCST90277286
Phosphatidylinositol (18:0_18:1) levels	20	62310086	A	C	-7.73E-02	1.72E-02	3.99E-01	rs2379129	20_62310086_C_A	0.99	7174	6.76E-06	GCST90277286
Phosphatidylinositol (18:0_18:2) levels	1	4847659	T	G	1.67E-01	3.66E-02	5.56E-02	rs116643980	1_4847659_G_T	0.95	7174	4.84E-06	GCST90277287
Phosphatidylinositol (18:0_18:2) levels	1	201955621	C	G	8.91E-02	1.83E-02	2.91E-01	rs3820438	1_201955621_G_C	0.98	7174	1.15E-06	GCST90277287
Phosphatidylinositol (18:0_18:2) levels	3	186854300	C	T	-2.03E-01	4.59E-02	3.41E-02	rs17366743	3_186854300_T_C	1.00	7174	9.68E-06	GCST90277287
Phosphatidylinositol (18:0_18:2) levels	4	143352205	T	C	-2.31E-01	5.12E-02	2.68E-02	rs17017629	4_143352205_C_T	1.00	7174	6.78E-06	GCST90277287
Phosphatidylinositol (18:0_18:2) levels	4	181645150	T	C	-4.70E-01	1.02E-01	7.09E-03	rs116438525	4_181645150_C_T	0.95	7174	4.05E-06	GCST90277287
Phosphatidylinositol (18:0_18:2) levels	4	186464194	C	A	1.54E-01	3.48E-02	6.50E-02	rs149130695	4_186464194_A_C	0.95	7174	9.36E-06	GCST90277287
Phosphatidylinositol (18:0_18:2) levels	7	110566718	G	C	-1.47E-01	3.28E-02	9.32E-01	rs2966427	7_110566718_C_G	1.00	7174	7.78E-06	GCST90277287
Phosphatidylinositol (18:0_18:2) levels	7	156099813	T	C	9.25E-02	2.04E-02	2.21E-01	rs58160859	7_156099813_C_T	0.98	7174	5.72E-06	GCST90277287
Phosphatidylinositol (18:0_18:2) levels	8	8854096	A	T	-1.44E-01	2.96E-02	8.99E-02	rs1251003	8_8854096_T_A	0.96	7174	1.23E-06	GCST90277287
Phosphatidylinositol (18:0_18:2) levels	11	36469520	T	C	-7.76E-02	1.66E-02	4.56E-01	rs4756323	11_36469520_C_T	0.99	7174	3.22E-06	GCST90277287
Phosphatidylinositol (18:0_18:2) levels	11	47436878	CAT	C	-7.70E-02	1.73E-02	5.72E-01	rs371930929	11_47436878_C_CAT	0.96	7174	8.15E-06	GCST90277287
Phosphatidylinositol (18:0_18:2) levels	11	61490996	C	T	-1.39E-01	2.04E-02	7.83E-01	rs2943813	11_61490996_T_C	1.00	7174	9.96E-12	GCST90277287
Phosphatidylinositol (18:0_18:2) levels	11	61770929	C	G	-1.79E-01	1.70E-02	4.44E-01	rs174527	11_61770929_G_C	0.98	7174	8.38E-26	GCST90277287
Phosphatidylinositol (18:0_18:2) levels	11	61781553	A	G	-5.98E-01	1.55E-02	4.09E-01	rs174533	11_61781553_G_A	1.00	7174	6.29E-295	GCST90277287
Phosphatidylinositol (18:0_18:2) levels	11	61948885	A	G	-1.79E-01	3.88E-02	4.92E-02	rs74754540	11_61948885_G_A	0.99	7174	4.19E-06	GCST90277287
Phosphatidylinositol (18:0_18:2) levels	11	62314821	G	T	2.14E-01	2.96E-02	8.73E-02	rs113394924	11_62314821_T_G	0.99	7174	6.09E-13	GCST90277287
Phosphatidylinositol (18:0_18:2) levels	11	62435462	G	A	-1.08E-01	1.88E-02	3.02E-01	rs3018617	11_62435462_A_G	0.94	7174	8.62E-09	GCST90277287
Phosphatidylinositol (18:0_18:2) levels	11	75743976	A	C	-1.03E-01	1.98E-02	2.32E-01	rs499974	11_75743976_C_A	0.99	7174	2.26E-07	GCST90277287
Phosphatidylinositol (18:0_18:2) levels	12	2077875	G	A	-8.90E-02	2.00E-02	2.23E-01	rs11062107	12_2077875_A_G	0.99	7174	9.12E-06	GCST90277287
Phosphatidylinositol (18:0_18:2) levels	14	101397558	A	C	-2.72E-01	5.59E-02	2.33E-02	rs72709529	14_101397558_C_A	0.97	7174	1.13E-06	GCST90277287
Phosphatidylinositol (18:0_18:2) levels	16	1939264	T	G	2.46E-01	4.53E-02	3.59E-02	rs147549994	16_1939264_G_T	0.95	7174	5.51E-08	GCST90277287
Phosphatidylinositol (18:0_18:2) levels	16	15036737	G	A	-7.88E-02	1.77E-02	3.34E-01	rs6498540	16_15036737_A_G	0.99	7174	8.47E-06	GCST90277287
Phosphatidylinositol (18:0_18:2) levels	16	56966973	A	G	-9.64E-02	2.17E-02	1.79E-01	rs12720922	16_56966973_G_A	1.00	7174	9.09E-06	GCST90277287
Phosphatidylinositol (18:0_18:2) levels	16	87672930	C	A	1.58E-01	3.55E-02	5.96E-02	rs13329893	16_87672930_A_C	0.98	7174	9.16E-06	GCST90277287
Phosphatidylinositol (18:0_18:2) levels	17	10763022	G	A	-2.81E-01	5.97E-02	2.09E-02	rs141391095	17_10763022_A_G	0.93	7174	2.58E-06	GCST90277287
Phosphatidylinositol (18:0_18:2) levels	19	19269704	G	A	-2.26E-01	3.77E-02	5.35E-02	rs187429064	19_19269704_A_G	0.95	7174	2.04E-09	GCST90277287
Phosphatidylcholine (16:0_20:5) levels	1	8828055	T	C	-7.97E-02	1.75E-02	3.54E-01	rs4908782	1_8828055_C_T	0.99	7173	5.17E-06	GCST90277288
Phosphatidylcholine (16:0_20:5) levels	2	52123601	A	T	-2.83E-01	5.64E-02	2.25E-02	rs34822092	2_52123601_T_A	0.98	7173	5.21E-07	GCST90277288
Phosphatidylcholine (16:0_20:5) levels	2	239878629	A	G	-7.74E-02	1.74E-02	6.09E-01	rs3936196	2_239878629_G_A	0.95	7173	9.25E-06	GCST90277288
Phosphatidylcholine (16:0_20:5) levels	3	39249420	A	G	-9.91E-02	2.08E-02	2.06E-01	rs59839417	3_39249420_G_A	1.00	7173	2.02E-06	GCST90277288
Phosphatidylcholine (16:0_20:5) levels	3	114428196	C	T	-9.55E-02	2.15E-02	1.89E-01	rs2733412	3_114428196_T_C	0.99	7173	8.72E-06	GCST90277288
Phosphatidylcholine (16:0_20:5) levels	3	177015370	T	G	7.86E-02	1.77E-02	3.40E-01	rs10936925	3_177015370_G_T	1.00	7173	8.89E-06	GCST90277288
Phosphatidylcholine (16:0_20:5) levels	4	155800587	T	C	-9.16E-02	2.03E-02	2.28E-01	rs3796575	4_155800587_C_T	0.97	7173	6.46E-06	GCST90277288
Phosphatidylcholine (16:0_20:5) levels	5	115424684	T	C	-2.17E-01	4.89E-02	3.14E-02	rs146489454	5_115424684_C_T	0.97	7173	9.32E-06	GCST90277288
Phosphatidylcholine (16:0_20:5) levels	6	21142482	T	C	3.50E-01	7.43E-02	1.32E-02	rs113917790	6_21142482_C_T	0.99	7173	2.55E-06	GCST90277288
Phosphatidylcholine (16:0_20:5) levels	7	132693734	A	C	-8.62E-02	1.70E-02	4.09E-01	rs7780677	7_132693734_C_A	1.00	7173	4.33E-07	GCST90277288
Phosphatidylcholine (16:0_20:5) levels	11	42657198	C	T	1.08E-01	2.30E-02	1.55E-01	rs7947954	11_42657198_T_C	1.00	7173	2.80E-06	GCST90277288
Phosphatidylcholine (16:0_20:5) levels	11	61770929	C	G	-1.01E-01	1.71E-02	4.44E-01	rs174527	11_61770929_G_C	0.98	7173	3.21E-09	GCST90277288
Phosphatidylcholine (16:0_20:5) levels	11	61790354	C	T	-3.73E-01	1.65E-02	4.09E-01	rs102274	11_61790354_T_C	1.00	7173	4.34E-109	GCST90277288
Phosphatidylcholine (16:0_20:5) levels	11	61948885	A	G	-1.89E-01	3.89E-02	4.92E-02	rs74754540	11_61948885_G_A	0.99	7173	1.18E-06	GCST90277288
Phosphatidylcholine (16:0_20:5) levels	11	62314821	G	T	1.41E-01	2.97E-02	8.73E-02	rs113394924	11_62314821_T_G	0.99	7173	2.28E-06	GCST90277288
Phosphatidylcholine (16:0_20:5) levels	11	62435462	G	A	-9.35E-02	1.88E-02	3.02E-01	rs3018617	11_62435462_A_G	0.94	7173	7.08E-07	GCST90277288
Phosphatidylcholine (16:0_20:5) levels	11	75745089	T	C	-8.84E-02	1.99E-02	2.32E-01	rs531117	11_75745089_C_T	0.99	7173	8.73E-06	GCST90277288
Phosphatidylcholine (16:0_20:5) levels	11	87975724	G	A	1.15E-01	2.52E-02	1.31E-01	rs28569162	11_87975724_A_G	1.00	7173	4.68E-06	GCST90277288
Phosphatidylcholine (16:0_20:5) levels	11	103598670	G	A	1.09E-01	2.44E-02	8.61E-01	rs1481977	11_103598670_A_G	1.00	7173	7.66E-06	GCST90277288
Phosphatidylcholine (16:0_20:5) levels	12	101716327	G	A	-7.87E-02	1.68E-02	4.77E-01	rs2041149	12_101716327_A_G	1.00	7173	2.73E-06	GCST90277288
Phosphatidylcholine (16:0_20:5) levels	14	71270831	T	C	-2.04E-01	4.54E-02	3.68E-02	rs137945273	14_71270831_C_T	0.93	7173	7.22E-06	GCST90277288
Phosphatidylcholine (16:0_20:5) levels	14	104397326	A	G	-2.14E-01	4.82E-02	3.48E-02	rs80083325	14_104397326_G_A	0.90	7173	8.69E-06	GCST90277288
Phosphatidylcholine (16:0_20:5) levels	16	10434179	G	A	1.21E-01	2.27E-02	1.65E-01	rs62025936	16_10434179_A_G	0.99	7173	1.18E-07	GCST90277288
Phosphatidylcholine (16:0_20:5) levels	16	20568904	C	T	2.74E-01	6.04E-02	2.01E-02	rs61653779	16_20568904_T_C	1.00	7173	5.99E-06	GCST90277288
Phosphatidylcholine (16:0_20:5) levels	21	31127778	G	A	4.60E-01	1.03E-01	7.23E-03	rs2833287	21_31127778_A_G	0.95	7173	8.62E-06	GCST90277288

Phosphatidylcholine (16:0_22:4) levels	1	102472833	T	C	4.23E-01	9.52E-02	8.91E-03	rs75679298	1_102472833_C_T	0.92	6874	9.08E-06	GCST90277289
Phosphatidylcholine (16:0_22:4) levels	2	27508073	C	T	-8.03E-02	1.78E-02	6.51E-01	rs1260326	2_27508073_T_C	1.00	6874	6.72E-06	GCST90277289
Phosphatidylcholine (16:0_22:4) levels	2	30362772	T	C	4.21E-01	8.68E-02	1.00E-02	rs6713301	2_30362772_C_T	0.97	6874	1.25E-06	GCST90277289
Phosphatidylcholine (16:0_22:4) levels	2	101652331	T	G	-1.16E-01	1.98E-02	2.49E-01	rs61008604	2_101652331_G_T	0.98	6874	5.24E-09	GCST90277289
Phosphatidylcholine (16:0_22:4) levels	3	85098584	A	C	2.12E-01	4.02E-02	4.67E-02	rs4856561	3_85098584_C_A	1.00	6874	1.46E-07	GCST90277289
Phosphatidylcholine (16:0_22:4) levels	3	186727647	T	C	-8.59E-02	1.75E-02	3.58E-01	rs4686798	3_186727647_C_T	1.00	6874	9.65E-07	GCST90277289
Phosphatidylcholine (16:0_22:4) levels	4	14330096	G	T	5.65E-01	1.27E-01	4.90E-03	rs116522970	4_14330096_T_G	0.96	6874	8.98E-06	GCST90277289
Phosphatidylcholine (16:0_22:4) levels	4	83110085	C	A	1.15E-01	2.35E-02	8.31E-01	rs4693564	4_83110085_A_C	0.93	6874	9.19E-07	GCST90277289
Phosphatidylcholine (16:0_22:4) levels	4	136353788	C	T	2.00E-01	4.04E-02	4.92E-02	rs115448758	4_136353788_T_C	0.95	6874	7.11E-07	GCST90277289
Phosphatidylcholine (16:0_22:4) levels	5	83594473	T	C	-1.12E-01	2.31E-02	1.61E-01	rs35040911	5_83594473_C_T	1.00	6874	1.34E-06	GCST90277289
Phosphatidylcholine (16:0_22:4) levels	5	126004183	A	C	-1.04E-01	2.25E-02	1.72E-01	rs1393144	5_126004183_C_A	0.99	6874	3.99E-06	GCST90277289
Phosphatidylcholine (16:0_22:4) levels	6	148952703	A	G	1.82E-01	3.74E-02	5.57E-02	rs12528390	6_148952703_G_A	0.99	6874	1.22E-06	GCST90277289
Phosphatidylcholine (16:0_22:4) levels	7	43872241	G	T	8.15E-02	1.74E-02	5.96E-01	rs2730612	7_43872241_T_G	1.00	6874	2.94E-06	GCST90277289
Phosphatidylcholine (16:0_22:4) levels	8	84225835	C	T	-8.86E-02	1.89E-02	2.82E-01	rs7007401	8_84225835_T_C	1.00	6874	2.95E-06	GCST90277289
Phosphatidylcholine (16:0_22:4) levels	8	118116184	A	T	-2.33E-01	5.22E-02	2.84E-02	rs148506839	8_118116184_T_A	0.93	6874	8.18E-06	GCST90277289
Phosphatidylcholine (16:0_22:4) levels	9	6890384	T	C	-2.17E-01	4.74E-02	3.44E-02	rs140028072	9_6890384_C_T	0.98	6874	4.65E-06	GCST90277289
Phosphatidylcholine (16:0_22:4) levels	9	23713150	C	T	1.02E-01	2.13E-02	2.02E-01	rs1360225	9_23713150_T_C	1.00	6874	1.66E-06	GCST90277289
Phosphatidylcholine (16:0_22:4) levels	9	136677616	G	C	-9.20E-02	1.81E-02	6.47E-01	rs2236514	9_136677616_C_G	0.95	6874	4.04E-07	GCST90277289
Phosphatidylcholine (16:0_22:4) levels	10	130397790	T	C	-8.72E-02	1.95E-02	7.10E-01	rs10741214	10_130397790_C_T	0.94	6874	7.65E-06	GCST90277289
Phosphatidylcholine (16:0_22:4) levels	11	61744026	T	C	1.21E-01	2.51E-02	1.34E-01	rs3741252	11_61744026_C_T	0.99	6874	1.53E-06	GCST90277289
Phosphatidylcholine (16:0_22:4) levels	11	61806212	C	T	-2.90E-01	1.71E-02	4.06E-01	rs174551	11_61806212_T_C	1.00	6874	3.67E-63	GCST90277289
Phosphatidylcholine (16:0_22:4) levels	11	88318708	C	G	-7.87E-02	1.70E-02	5.48E-01	rs570929	11_88318708_G_C	1.00	6874	3.98E-06	GCST90277289
Phosphatidylcholine (16:0_22:4) levels	12	107709655	C	T	2.77E-01	5.57E-02	2.49E-02	rs192523409	12_107709655_T_C	0.98	6874	6.89E-07	GCST90277289
Phosphatidylcholine (16:0_22:4) levels	14	98833387	C	T	-1.31E-01	2.93E-02	9.60E-02	rs61979278	14_98833387_T_C	0.98	6874	7.79E-06	GCST90277289
Phosphatidylcholine (16:0_22:4) levels	14	100137997	G	A	-1.02E-01	2.29E-02	1.65E-01	rs10138425	14_100137997_A_G	1.00	6874	8.73E-06	GCST90277289
Phosphatidylcholine (16:0_22:4) levels	16	2352549	A	G	2.13E-01	4.52E-02	3.86E-02	rs77730345	16_2352549_G_A	0.95	6874	2.53E-06	GCST90277289
Phosphatidylcholine (16:0_22:4) levels	17	10024173	T	C	1.11E-01	2.46E-02	1.36E-01	rs72807394	17_10024173_C_T	0.99	6874	6.60E-06	GCST90277289
Phosphatidylcholine (16:0_22:4) levels	20	937096	T	C	1.23E-01	2.76E-02	1.19E-01	rs111622683	20_937096_C_T	0.89	6874	7.96E-06	GCST90277289
Phosphatidylcholine (16:0_22:5) levels	1	34932549	T	C	-3.41E-01	7.25E-02	1.53E-02	rs147057045	1_34932549_C_T	0.89	7172	2.63E-06	GCST90277290
Phosphatidylcholine (16:0_22:5) levels	1	212018649	A	G	8.33E-02	1.85E-02	7.16E-01	rs3009985	1_212018649_G_A	1.00	7172	6.56E-06	GCST90277290
Phosphatidylcholine (16:0_22:5) levels	2	27508073	C	T	-9.04E-02	1.74E-02	6.51E-01	rs1260326	2_27508073_T_C	1.00	7172	2.20E-07	GCST90277290
Phosphatidylcholine (16:0_22:5) levels	2	35106778	A	C	2.36E-01	4.94E-02	3.11E-02	rs62142428	2_35106778_C_A	0.95	7172	1.77E-06	GCST90277290
Phosphatidylcholine (16:0_22:5) levels	3	39250756	C	G	-9.35E-02	2.08E-02	2.06E-01	rs4016738	3_39250756_G_C	1.00	7172	7.04E-06	GCST90277290
Phosphatidylcholine (16:0_22:5) levels	4	7382480	A	G	1.03E-01	2.27E-02	1.78E-01	rs62280086	4_7382480_G_A	0.92	7172	5.38E-06	GCST90277290
Phosphatidylcholine (16:0_22:5) levels	4	30161140	T	G	8.08E-02	1.77E-02	3.73E-01	rs2613167	4_30161140_G_T	0.97	7172	4.83E-06	GCST90277290
Phosphatidylcholine (16:0_22:5) levels	5	134851174	T	C	-2.21E-01	4.91E-02	2.91E-02	rs139606388	5_134851174_C_T	0.99	7172	6.78E-06	GCST90277290
Phosphatidylcholine (16:0_22:5) levels	6	10999417	C	T	8.46E-02	1.68E-02	4.58E-01	rs9918362	6_10999417_T_C	1.00	7172	5.09E-07	GCST90277290
Phosphatidylcholine (16:0_22:5) levels	6	32139362	C	T	-8.36E-02	1.73E-02	6.31E-01	rs3096698	6_32139362_T_C	0.99	7172	1.39E-06	GCST90277290
Phosphatidylcholine (16:0_22:5) levels	6	160908659	C	T	1.12E-01	2.37E-02	1.42E-01	rs73019765	6_160908659_T_C	1.00	7172	2.31E-06	GCST90277290
Phosphatidylcholine (16:0_22:5) levels	7	50185795	A	G	8.07E-02	1.71E-02	4.02E-01	rs7805803	7_50185795_G_A	1.00	7172	2.33E-06	GCST90277290
Phosphatidylcholine (16:0_22:5) levels	8	25975855	A	G	1.17E-01	2.60E-02	1.21E-01	rs73225965	8_25975855_G_A	0.96	7172	6.97E-06	GCST90277290
Phosphatidylcholine (16:0_22:5) levels	8	93993037	C	T	1.50E-01	3.38E-02	6.89E-02	rs58967664	8_93993037_T_C	0.94	7172	9.44E-06	GCST90277290
Phosphatidylcholine (16:0_22:5) levels	9	136677616	G	C	-1.23E-01	1.77E-02	6.47E-01	rs2236514	9_136677616_C_G	0.95	7172	4.04E-12	GCST90277290
Phosphatidylcholine (16:0_22:5) levels	11	61481911	A	C	-1.09E-01	2.14E-02	8.03E-01	rs3019200	11_61481911_C_A	1.00	7172	3.77E-07	GCST90277290
Phosphatidylcholine (16:0_22:5) levels	11	61770929	C	G	-1.13E-01	1.70E-02	4.44E-01	rs174527	11_61770929_G_C	0.98	7172	3.69E-11	GCST90277290
Phosphatidylcholine (16:0_22:5) levels	11	61781553	A	G	-4.13E-01	1.63E-02	4.09E-01	rs174533	11_61781553_G_A	1.00	7172	5.08E-135	GCST90277290
Phosphatidylcholine (16:0_22:5) levels	11	62451557	G	A	8.90E-02	1.97E-02	2.57E-01	rs7936002	11_62451557_A_G	0.93	7172	6.17E-06	GCST90277290
Phosphatidylcholine (16:0_22:5) levels	11	68908029	T	C	-1.67E-01	2.86E-02	9.80E-02	rs508049	11_68908029_C_T	0.96	7172	5.62E-09	GCST90277290
Phosphatidylcholine (16:0_22:5) levels	12	78247210	T	G	3.92E-01	8.14E-02	1.10E-02	rs80197029	12_78247210_G_T	0.93	7172	1.48E-06	GCST90277290
Phosphatidylcholine (16:0_22:5) levels	12	120979061	T	C	2.08E-01	3.97E-02	4.57E-02	rs1800574	12_120979061_C_T	1.00	7172	1.51E-07	GCST90277290
Phosphatidylcholine (16:0_22:5) levels	15	71171261	C	T	-1.20E-01	2.70E-02	1.11E-01	rs11072257	15_71171261_T_C	0.98	7172	8.46E-06	GCST90277290
Phosphatidylcholine (16:0_22:5) levels	16	20568904	C	T	2.76E-01	6.02E-02	2.01E-02	rs61653779	16_20568904_T_C	1.00	7172	4.69E-06	GCST90277290
Phosphatidylcholine (16:0_22:5) levels	16	54140373	G	A	7.87E-02	1.74E-02	3.55E-01	rs12598390	16_54140373_A_G	1.00	7172	5.89E-06	GCST90277290
Phosphatidylcholine (16:0_22:5) levels	17	17471077	C	T	-7.48E-02	1.68E-02	5.01E-01	rs1242492	17_17471077_T_C	0.98	7172	8.58E-06	GCST90277290
Phosphatidylcholine (16:0_22:5) levels	18	52743009	C	T	-4.77E-01	8.91E-02	9.35E-03	rs147797702	18_52743009_T_C	0.96	7172	8.64E-08	GCST90277290
Phosphatidylcholine (16:0_22:5) levels	18	54253269	C	G	3.16E-01	6.73E-02	1.61E-02	rs146509239	18_54253269_G_C	0.97	7172	2.60E-06	GCST90277290
Phosphatidylcholine (16:0_22:5) levels	19	48224239	T	C	1.25E-01	2.75E-02	1.04E-01	rs56336136	19_48224239_C_T	0.97	7172	5.35E-06	GCST90277290

Phosphatidylcholine (16:0_22:5) levels	21	43971391	T	C	-2.46E-01	4.20E-02	3.91E-02	rs62229686	21_43971391_C_T	1.00	7172	4.54E-09	GCST90277290
Phosphatidylcholine (16:0_22:6) levels	2	158238814	T	C	-8.74E-02	1.76E-02	3.31E-01	rs57634784	2_158238814_C_T	0.99	7170	6.93E-07	GCST90277291
Phosphatidylcholine (16:0_22:6) levels	4	11829414	C	A	2.49E-01	5.58E-02	2.45E-02	rs76218130	4_11829414_A_C	0.92	7170	8.52E-06	GCST90277291
Phosphatidylcholine (16:0_22:6) levels	4	119529863	A	G	-9.57E-02	2.08E-02	2.04E-01	rs3775850	4_119529863_G_A	1.00	7170	4.12E-06	GCST90277291
Phosphatidylcholine (16:0_22:6) levels	5	54649287	G	A	-1.71E-01	3.82E-02	9.49E-01	rs6864719	5_54649287_A_G	0.99	7170	8.12E-06	GCST90277291
Phosphatidylcholine (16:0_22:6) levels	5	82984713	C	T	-3.40E-01	7.45E-02	1.29E-02	rs143699848	5_82984713_T_C	0.97	7170	5.15E-06	GCST90277291
Phosphatidylcholine (16:0_22:6) levels	5	153118411	C	T	-9.15E-01	1.96E-01	2.12E-03	rs77304558	5_153118411_T_C	0.93	7170	3.14E-06	GCST90277291
Phosphatidylcholine (16:0_22:6) levels	6	24353208	C	A	2.62E-01	5.03E-02	2.94E-02	rs10498720	6_24353208_A_C	0.98	7170	2.01E-07	GCST90277291
Phosphatidylcholine (16:0_22:6) levels	7	17680332	T	G	8.11E-02	1.68E-02	5.81E-01	rs2075083	7_17680332_G_T	0.99	7170	1.42E-06	GCST90277291
Phosphatidylcholine (16:0_22:6) levels	7	106395713	A	G	9.83E-02	2.20E-02	2.00E-01	rs73188528	7_106395713_G_A	0.91	7170	8.35E-06	GCST90277291
Phosphatidylcholine (16:0_22:6) levels	7	150780296	A	G	8.22E-02	1.83E-02	3.04E-01	rs17173596	7_150780296_G_A	1.00	7170	6.93E-06	GCST90277291
Phosphatidylcholine (16:0_22:6) levels	10	125205699	A	G	-2.43E-01	5.09E-02	3.17E-02	rs117398583	10_125205699_G_A	0.89	7170	1.88E-06	GCST90277291
Phosphatidylcholine (16:0_22:6) levels	10	126057886	C	A	-2.68E-01	5.91E-02	2.22E-02	rs117185962	10_126057886_A_C	0.94	7170	5.62E-06	GCST90277291
Phosphatidylcholine (16:0_22:6) levels	11	42654689	C	T	7.86E-02	1.69E-02	4.15E-01	rs61900787	11_42654689_T_C	1.00	7170	3.34E-06	GCST90277291
Phosphatidylcholine (16:0_22:6) levels	11	61800281	A	C	-1.27E-01	1.73E-02	3.80E-01	rs174544	11_61800281_C_A	1.00	7170	2.30E-13	GCST90277291
Phosphatidylcholine (16:0_22:6) levels	11	116778201	C	G	-1.22E-01	2.32E-02	8.49E-01	rs964184	11_116778201_G_C	1.00	7170	1.38E-07	GCST90277291
Phosphatidylcholine (16:0_22:6) levels	11	133501713	T	C	-1.19E-01	2.48E-02	1.36E-01	rs4937784	11_133501713_C_T	0.95	7170	1.56E-06	GCST90277291
Phosphatidylcholine (16:0_22:6) levels	13	107675829	C	T	8.26E-02	1.83E-02	2.87E-01	rs1935135	13_107675829_T_C	1.00	7170	6.42E-06	GCST90277291
Phosphatidylcholine (16:0_22:6) levels	14	72571809	T	C	-1.91E-01	3.82E-02	5.30E-02	rs10150075	14_72571809_C_T	0.95	7170	5.88E-07	GCST90277291
Phosphatidylcholine (16:0_22:6) levels	14	78360286	A	C	-2.76E-01	5.98E-02	2.02E-02	rs79508872	14_78360286_C_A	0.98	7170	4.14E-06	GCST90277291
Phosphatidylcholine (16:0_22:6) levels	15	64865848	A	G	-2.39E-01	5.36E-02	2.42E-02	rs77456635	15_64865848_G_A	1.00	7170	8.62E-06	GCST90277291
Phosphatidylcholine (16:0_22:6) levels	15	101196347	T	G	2.15E-01	4.11E-02	4.49E-02	rs117061362	15_101196347_G_T	0.93	7170	1.81E-07	GCST90277291
Phosphatidylcholine (16:0_22:6) levels	16	24973770	T	C	3.82E-01	8.42E-02	1.11E-02	rs74883996	16_24973770_C_T	0.89	7170	5.82E-06	GCST90277291
Phosphatidylcholine (16:0_22:6) levels	20	57590477	C	T	-2.47E-01	5.52E-02	2.55E-02	rs76744655	20_57590477_T_C	0.92	7170	7.72E-06	GCST90277291
Phosphatidylcholine (16:1_18:0) levels	2	6080522	C	G	-2.75E-01	5.72E-02	2.40E-02	rs2609122	2_6080522_G_C	1.00	6524	1.55E-06	GCST90277292
Phosphatidylcholine (16:1_18:0) levels	2	15232755	G	A	-4.49E-01	9.99E-02	8.90E-03	rs145028924	2_15232755_A_G	0.85	6524	6.87E-06	GCST90277292
Phosphatidylcholine (16:1_18:0) levels	2	27508073	C	T	-1.08E-01	1.83E-02	6.51E-01	rs1260326	2_27508073_T_C	1.00	6524	3.75E-09	GCST90277292
Phosphatidylcholine (16:1_18:0) levels	2	172180344	A	G	2.04E-01	4.54E-02	4.13E-02	rs114728714	2_172180344_G_A	0.94	6524	6.82E-06	GCST90277292
Phosphatidylcholine (16:1_18:0) levels	3	37451054	G	A	-9.80E-02	2.20E-02	2.01E-01	rs62239496	3_37451054_A_G	0.99	6524	8.56E-06	GCST90277292
Phosphatidylcholine (16:1_18:0) levels	4	17072136	A	C	1.14E-01	2.46E-02	1.45E-01	rs1533362	4_17072136_C_A	1.00	6524	3.89E-06	GCST90277292
Phosphatidylcholine (16:1_18:0) levels	4	131189906	A	G	-4.16E-01	8.38E-02	1.09E-02	rs76712623	4_131189906_G_A	0.98	6524	6.84E-07	GCST90277292
Phosphatidylcholine (16:1_18:0) levels	5	165822116	G	A	1.01E-01	2.18E-02	2.02E-01	rs28807772	5_165822116_A_G	1.00	6524	3.33E-06	GCST90277292
Phosphatidylcholine (16:1_18:0) levels	7	137280208	C	T	2.80E-01	6.24E-02	2.01E-02	rs78275150	7_137280208_T_C	0.97	6524	7.25E-06	GCST90277292
Phosphatidylcholine (16:1_18:0) levels	10	128061194	T	A	1.86E-01	4.08E-02	9.51E-01	rs4332435	10_128061194_A_T	0.98	6524	5.56E-06	GCST90277292
Phosphatidylcholine (16:1_18:0) levels	11	61814184	A	G	9.52E-02	1.90E-02	3.14E-01	rs174559	11_61814184_G_A	1.00	6524	5.39E-07	GCST90277292
Phosphatidylcholine (16:1_18:0) levels	12	105707476	C	G	-8.81E-02	1.94E-02	2.73E-01	rs11112689	12_105707476_G_C	1.00	6524	5.84E-06	GCST90277292
Phosphatidylcholine (16:1_18:0) levels	13	37372860	T	C	1.57E-01	3.46E-02	9.29E-01	rs7991657	13_37372860_C_T	1.00	6524	5.95E-06	GCST90277292
Phosphatidylcholine (16:1_18:0) levels	15	38611953	C	G	1.51E-01	3.33E-02	7.45E-02	rs10520096	15_38611953_G_C	0.98	6524	5.82E-06	GCST90277292
Phosphatidylcholine (16:1_18:0) levels	16	59856268	C	G	-2.79E-01	5.45E-02	2.89E-02	rs77007318	16_59856268_G_C	0.89	6524	3.19E-07	GCST90277292
Phosphatidylcholine (16:1_18:0) levels	16	78478626	C	A	-9.53E-02	1.99E-02	2.71E-01	rs9934078	16_78478626_A_C	0.99	6524	1.74E-06	GCST90277292
Phosphatidylcholine (16:1_18:0) levels	19	58583007	T	C	3.07E-01	6.10E-02	2.30E-02	rs76447935	19_58583007_C_T	0.98	6524	4.94E-07	GCST90277292
Phosphatidylcholine (16:1_18:0) levels	20	62310086	A	C	-8.67E-02	1.80E-02	3.99E-01	rs2379129	20_62310086_C_A	0.99	6524	1.44E-06	GCST90277292
Phosphatidylcholine (16:1_18:0) levels	21	14678032	T	C	1.99E-01	4.13E-02	4.70E-02	rs117402498	21_14678032_C_T	0.98	6524	1.59E-06	GCST90277292
Phosphatidylcholine (16:1_18:1) levels	1	211687024	C	G	-9.36E-02	2.05E-02	7.90E-01	rs701919	1_211687024_G_C	0.99	7166	5.04E-06	GCST90277293
Phosphatidylcholine (16:1_18:1) levels	2	27508073	C	T	-1.10E-01	1.75E-02	6.51E-01	rs1260326	2_27508073_T_C	1.00	7166	2.95E-10	GCST90277293
Phosphatidylcholine (16:1_18:1) levels	2	97756543	T	C	8.73E-02	1.84E-02	7.12E-01	rs5865	2_97756543_C_T	1.00	7166	2.13E-06	GCST90277293
Phosphatidylcholine (16:1_18:1) levels	2	137186441	T	A	1.69E-01	3.52E-02	5.97E-02	rs13402814	2_137186441_A_T	1.00	7166	1.68E-06	GCST90277293
Phosphatidylcholine (16:1_18:1) levels	2	169154895	A	G	-2.25E-01	4.94E-02	3.21E-02	rs80160469	2_169154895_G_A	0.90	7166	5.50E-06	GCST90277293
Phosphatidylcholine (16:1_18:1) levels	3	187394534	G	T	-9.02E-02	1.88E-02	2.92E-01	rs56316064	3_187394534_T_G	0.95	7166	1.58E-06	GCST90277293
Phosphatidylcholine (16:1_18:1) levels	5	20149566	C	A	-1.26E-01	2.81E-02	1.02E-01	rs74357965	5_20149566_A_C	0.96	7166	7.10E-06	GCST90277293
Phosphatidylcholine (16:1_18:1) levels	6	138148100	T	G	3.11E-01	6.61E-02	1.70E-02	rs113366516	6_138148100_G_T	0.93	7166	2.65E-06	GCST90277293
Phosphatidylcholine (16:1_18:1) levels	7	81204492	A	G	1.83E-01	4.11E-02	4.75E-02	rs75102979	7_81204492_G_A	0.90	7166	8.63E-06	GCST90277293
Phosphatidylcholine (16:1_18:1) levels	7	107240375	T	C	1.55E-01	3.25E-02	7.33E-02	rs145551604	7_107240375_C_T	0.97	7166	2.03E-06	GCST90277293
Phosphatidylcholine (16:1_18:1) levels	8	5187127	T	G	1.88E-01	4.15E-02	4.32E-02	rs141084476	8_5187127_G_T	0.97	7166	6.02E-06	GCST90277293
Phosphatidylcholine (16:1_18:1) levels	8	84225835	C	T	-8.22E-02	1.85E-02	2.82E-01	rs7007401	8_84225835_T_C	1.00	7166	9.16E-06	GCST90277293
Phosphatidylcholine (16:1_18:1) levels	10	62688203	T	C	1.96E-01	4.20E-02	4.26E-02	rs72834725	10_62688203_C_T	0.97	7166	2.92E-06	GCST90277293
Phosphatidylcholine (16:1_18:1) levels	10	81316583	A	G	-1.16E-01	2.48E-02	1.33E-01	rs11189347	10_81316583_G_A	0.98	7166	2.57E-06	GCST90277293

Phosphatidylcholine (16:1_18:1) levels	10	100315722	A	G	-1.76E-01	2.60E-02	1.21E-01	rs603424	10_100315722_G_A	1.00	7166	1.21E-11	GCST90277293
Phosphatidylcholine (16:1_18:1) levels	10	100600003	A	G	1.28E-01	2.42E-02	1.41E-01	rs4447106	10_100600003_G_A	0.98	7166	1.29E-07	GCST90277293
Phosphatidylcholine (16:1_18:1) levels	11	30070603	C	T	-7.23E-01	1.58E-01	2.95E-03	rs139935251	11_30070603_T_C	0.93	7166	4.78E-06	GCST90277293
Phosphatidylcholine (16:1_18:1) levels	11	61814292	C	T	1.76E-01	1.72E-02	3.83E-01	rs174560	11_61814292_T_C	1.00	7166	2.28E-24	GCST90277293
Phosphatidylcholine (16:1_18:1) levels	12	4996173	G	A	-7.55E-02	1.67E-02	4.40E-01	rs7968329	12_4996173_A_G	1.00	7166	6.45E-06	GCST90277293
Phosphatidylcholine (16:1_18:1) levels	12	19869115	T	C	-1.68E-01	3.60E-02	6.19E-02	rs116997061	12_19869115_C_T	0.91	7166	3.00E-06	GCST90277293
Phosphatidylcholine (16:1_18:1) levels	15	58391167	G	A	-9.26E-02	1.68E-02	5.73E-01	rs1532085	15_58391167_A_G	1.00	7166	3.79E-08	GCST90277293
Phosphatidylcholine (16:1_18:1) levels	15	58432643	A	G	9.38E-02	1.80E-02	3.37E-01	rs8033940	15_58432643_G_A	0.99	7166	2.02E-07	GCST90277293
Phosphatidylcholine (16:1_18:1) levels	16	6532848	T	G	-2.31E-01	5.09E-02	2.78E-02	rs117217513	16_6532848_G_T	0.96	7166	5.76E-06	GCST90277293
Phosphatidylcholine (16:1_18:1) levels	16	56953457	C	T	1.01E-01	1.86E-02	2.79E-01	rs56156922	16_56953457_T_C	1.00	7166	5.95E-08	GCST90277293
Phosphatidylcholine (16:1_18:1) levels	17	18969312	A	C	5.65E-01	1.24E-01	5.30E-03	rs185867215	17_18969312_C_A	0.87	7166	5.40E-06	GCST90277293
Phosphatidylcholine (16:1_18:1) levels	18	15033501	T	C	4.15E-01	7.37E-02	1.31E-02	rs146934967	18_15033501_C_T	0.99	7166	1.83E-08	GCST90277293
Phosphatidylcholine (16:1_18:1) levels	18	61427576	G	A	-8.76E-02	1.97E-02	2.53E-01	rs36106314	18_61427576_A_G	0.94	7166	9.22E-06	GCST90277293
Phosphatidylcholine (16:1_18:1) levels	19	19282905	A	G	-1.14E-01	2.25E-02	1.71E-01	rs8100204	19_19282905_G_A	0.97	7166	4.12E-07	GCST90277293
Phosphatidylcholine (16:1_18:2) levels	1	213668292	G	T	7.45E-02	1.67E-02	4.89E-01	rs12031139	1_213668292_T_G	0.99	7165	8.07E-06	GCST90277294
Phosphatidylcholine (16:1_18:2) levels	2	27508073	C	T	-9.85E-02	1.74E-02	6.51E-01	rs1260326	2_27508073_T_C	1.00	7165	1.66E-08	GCST90277294
Phosphatidylcholine (16:1_18:2) levels	2	137159225	A	G	1.57E-01	3.51E-02	5.96E-02	rs10201921	2_137159225_G_A	1.00	7165	8.15E-06	GCST90277294
Phosphatidylcholine (16:1_18:2) levels	3	26850125	G	A	9.60E-02	1.77E-02	3.35E-01	rs9839380	3_26850125_A_G	0.99	7165	6.37E-08	GCST90277294
Phosphatidylcholine (16:1_18:2) levels	4	169277659	A	G	-1.21E-01	2.50E-02	1.30E-01	rs13150924	4_169277659_G_A	0.98	7165	1.37E-06	GCST90277294
Phosphatidylcholine (16:1_18:2) levels	5	86303978	C	T	1.31E-01	2.84E-02	9.05E-01	rs6876235	5_86303978_T_C	1.00	7165	3.88E-06	GCST90277294
Phosphatidylcholine (16:1_18:2) levels	6	139585552	G	T	2.84E-01	6.23E-02	1.82E-02	rs184289628	6_139585552_T_G	0.97	7165	5.40E-06	GCST90277294
Phosphatidylcholine (16:1_18:2) levels	6	156383631	C	T	-2.20E-01	4.45E-02	3.72E-02	rs9480322	6_156383631_T_C	0.98	7165	8.16E-07	GCST90277294
Phosphatidylcholine (16:1_18:2) levels	7	6533456	G	T	8.60E-02	1.83E-02	3.08E-01	rs7783799	7_6533456_T_G	0.95	7165	2.63E-06	GCST90277294
Phosphatidylcholine (16:1_18:2) levels	7	10911524	G	T	-2.75E-01	6.09E-02	1.97E-02	rs77949981	7_10911524_T_G	0.97	7165	6.23E-06	GCST90277294
Phosphatidylcholine (16:1_18:2) levels	9	14382621	C	T	7.81E-02	1.76E-02	3.50E-01	rs7036554	9_14382621_T_C	0.97	7165	9.12E-06	GCST90277294
Phosphatidylcholine (16:1_18:2) levels	10	100315722	A	G	-1.61E-01	2.59E-02	1.21E-01	rs603424	10_100315722_G_A	1.00	7165	5.15E-10	GCST90277294
Phosphatidylcholine (16:1_18:2) levels	11	61770929	C	G	1.18E-01	1.70E-02	4.44E-01	rs174527	11_61770929_G_C	0.98	7165	4.23E-12	GCST90277294
Phosphatidylcholine (16:1_18:2) levels	11	61832870	C	A	-3.66E-01	1.65E-02	5.91E-01	rs174574	11_61832870_A_C	1.00	7165	2.11E-105	GCST90277294
Phosphatidylcholine (16:1_18:2) levels	11	62451557	G	A	-1.15E-01	1.96E-02	2.57E-01	rs7936002	11_62451557_A_G	0.93	7165	4.22E-09	GCST90277294
Phosphatidylcholine (16:1_18:2) levels	11	92398043	G	A	1.12E-01	2.47E-02	1.32E-01	rs11602429	11_92398043_A_G	0.99	7165	5.49E-06	GCST90277294
Phosphatidylcholine (16:1_18:2) levels	11	107889276	A	G	-1.09E-01	2.38E-02	8.60E-01	rs10890773	11_107889276_G_A	1.00	7165	4.82E-06	GCST90277294
Phosphatidylcholine (16:1_18:2) levels	15	58391167	G	A	-9.72E-02	1.68E-02	5.73E-01	rs1532085	15_58391167_A_G	1.00	7165	7.02E-09	GCST90277294
Phosphatidylcholine (16:1_18:2) levels	15	58431476	T	C	1.16E-01	1.93E-02	2.58E-01	rs1800588	15_58431476_C_T	0.99	7165	2.21E-09	GCST90277294
Phosphatidylcholine (16:1_18:2) levels	16	15080714	A	G	-8.78E-02	1.84E-02	2.81E-01	rs4122352	16_15080714_G_A	1.00	7165	1.96E-06	GCST90277294
Phosphatidylcholine (16:1_18:2) levels	16	56953457	C	T	1.14E-01	1.85E-02	2.79E-01	rs56156922	16_56953457_T_C	1.00	7165	8.30E-10	GCST90277294
Phosphatidylcholine (16:1_18:2) levels	16	60201094	G	A	-7.95E-02	1.73E-02	6.17E-01	rs216958	16_60201094_A_G	0.99	7165	4.47E-06	GCST90277294
Phosphatidylcholine (16:1_18:2) levels	18	15033501	T	C	4.43E-01	7.36E-02	1.31E-02	rs146934967	18_15033501_C_T	0.99	7165	1.88E-09	GCST90277294
Phosphatidylcholine (16:1_18:2) levels	18	71672897	G	C	1.04E-01	2.01E-02	2.32E-01	rs62104079	18_71672897_C_G	0.95	7165	2.34E-07	GCST90277294
Phosphatidylcholine (16:1_18:2) levels	19	19210016	G	C	-1.97E-01	3.86E-02	5.22E-02	rs150057262	19_19210016_C_G	0.94	7165	3.57E-07	GCST90277294
Phosphatidylcholine (16:1_18:2) levels	20	1014797	A	G	2.07E-01	4.63E-02	3.68E-02	rs78575494	20_1014797_G_A	0.91	7165	7.79E-06	GCST90277294
Phosphatidylcholine (16:1_20:4) levels	1	161649571	T	C	1.63E-01	3.48E-02	8.54E-02	rs2446624	1_161649571_C_T	0.94	5564	2.80E-06	GCST90277295
Phosphatidylcholine (16:1_20:4) levels	1	163867608	C	G	-3.06E-01	6.18E-02	2.55E-02	rs75878220	1_163867608_G_C	0.93	5564	7.32E-07	GCST90277295
Phosphatidylcholine (16:1_20:4) levels	2	61545122	G	A	9.53E-02	1.96E-02	6.20E-01	rs6545883	2_61545122_A_G	1.00	5564	1.17E-06	GCST90277295
Phosphatidylcholine (16:1_20:4) levels	3	43486710	A	C	-8.43E-02	1.90E-02	5.62E-01	rs9809733	3_43486710_C_A	0.99	5564	9.73E-06	GCST90277295
Phosphatidylcholine (16:1_20:4) levels	3	60495677	T	C	1.64E-01	3.67E-02	7.10E-02	rs2197750	3_60495677_C_T	0.98	5564	7.74E-06	GCST90277295
Phosphatidylcholine (16:1_20:4) levels	3	159910270	T	A	1.38E-01	2.92E-02	1.22E-01	rs35811605	3_159910270_A_T	0.99	5564	2.25E-06	GCST90277295
Phosphatidylcholine (16:1_20:4) levels	4	144171704	C	T	-1.03E-01	2.31E-02	7.69E-01	rs4539990	4_144171704_T_C	0.94	5564	9.31E-06	GCST90277295
Phosphatidylcholine (16:1_20:4) levels	4	176373256	G	T	1.88E-01	4.15E-02	9.34E-01	rs2085061	4_176373256_T_G	0.87	5564	6.19E-06	GCST90277295
Phosphatidylcholine (16:1_20:4) levels	6	168992667	A	G	9.31E-02	2.10E-02	7.23E-01	rs1757973	6_168992667_G_A	1.00	5564	9.54E-06	GCST90277295
Phosphatidylcholine (16:1_20:4) levels	7	89538067	T	C	6.05E-01	1.21E-01	7.06E-03	rs7787163	7_89538067_C_T	0.95	5564	5.65E-07	GCST90277295
Phosphatidylcholine (16:1_20:4) levels	7	150431359	A	G	2.69E-01	5.61E-02	3.11E-02	rs56317095	7_150431359_G_A	0.93	5564	1.62E-06	GCST90277295
Phosphatidylcholine (16:1_20:4) levels	11	61770929	C	G	-1.25E-01	1.94E-02	4.44E-01	rs174527	11_61770929_G_C	0.98	5564	1.09E-10	GCST90277295
Phosphatidylcholine (16:1_20:4) levels	11	61783884	C	T	-3.28E-01	1.92E-02	4.09E-01	rs174535	11_61783884_T_C	1.00	5564	5.33E-64	GCST90277295
Phosphatidylcholine (16:1_20:4) levels	11	75745535	T	A	-1.09E-01	2.25E-02	2.41E-01	rs695112	11_75745535_A_T	0.99	5564	1.33E-06	GCST90277295
Phosphatidylcholine (16:1_20:4) levels	11	84737810	G	T	1.69E-01	3.80E-02	6.74E-02	rs75781049	11_84737810_T_G	1.00	5564	9.13E-06	GCST90277295
Phosphatidylcholine (16:1_20:4) levels	11	124824361	T	C	1.10E-01	2.42E-02	1.92E-01	rs591931	11_124824361_C_T	0.98	5564	5.55E-06	GCST90277295
Phosphatidylcholine (16:1_20:4) levels	12	96871603	A	G	-2.26E-01	5.09E-02	3.77E-02	rs117505687	12_96871603_G_A	0.97	5564	9.36E-06	GCST90277295

Phosphatidylcholine (16:1_20:4) levels	12	130508437	C	G	-1.10E-01	2.33E-02	7.78E-01	rs10848122	12_130508437_G_C	0.98	5564	2.54E-06	GCST90277295
Phosphatidylcholine (16:1_20:4) levels	17	17971172	T	C	8.90E-02	1.91E-02	4.21E-01	rs8068175	17_17971172_C_T	0.99	5564	3.32E-06	GCST90277295
Phosphatidylcholine (16:1_20:4) levels	17	21397913	T	C	9.19E-02	1.91E-02	5.37E-01	rs11650558	17_21397913_C_T	0.98	5564	1.49E-06	GCST90277295
Phosphatidylcholine (16:1_20:4) levels	18	22904677	G	T	1.04E-01	2.21E-02	2.42E-01	rs11659363	18_22904677_T_G	1.00	5564	2.60E-06	GCST90277295
Phosphatidylcholine (16:1_20:4) levels	19	19282905	A	G	-1.26E-01	2.57E-02	1.71E-01	rs8100204	19_19282905_G_A	0.97	5564	9.87E-07	GCST90277295
Phosphatidylcholine (16:1_20:4) levels	19	54449569	T	C	2.03E-01	4.33E-02	5.26E-02	rs192436652	19_54449569_C_T	0.98	5564	2.80E-06	GCST90277295
Phosphatidylcholine (17:0_18:1) levels	1	7149275	G	A	8.32E-02	1.88E-02	2.90E-01	rs17030373	1_7149275_A_G	0.99	7073	9.60E-06	GCST90277296
Phosphatidylcholine (17:0_18:1) levels	1	247024993	A	T	5.92E-01	1.22E-01	5.19E-03	rs139070595	1_247024993_T_A	0.91	7073	1.35E-06	GCST90277296
Phosphatidylcholine (17:0_18:1) levels	2	134188357	C	G	-1.34E-01	3.00E-02	8.83E-02	rs1257173	2_134188357_G_C	0.97	7073	7.90E-06	GCST90277296
Phosphatidylcholine (17:0_18:1) levels	2	241739239	T	C	-1.14E-01	2.57E-02	1.29E-01	rs145967151	2_241739239_C_T	0.95	7073	9.92E-06	GCST90277296
Phosphatidylcholine (17:0_18:1) levels	4	110207431	T	C	9.63E-02	2.14E-02	7.97E-01	rs5022521	4_110207431_C_T	0.94	7073	6.55E-06	GCST90277296
Phosphatidylcholine (17:0_18:1) levels	4	130689035	A	C	-2.08E-01	4.51E-02	3.76E-02	rs77534789	4_130689035_C_A	0.96	7073	4.12E-06	GCST90277296
Phosphatidylcholine (17:0_18:1) levels	4	170196755	A	G	8.02E-02	1.69E-02	4.36E-01	rs4692808	4_170196755_G_A	1.00	7073	2.08E-06	GCST90277296
Phosphatidylcholine (17:0_18:1) levels	6	166616136	G	A	1.25E-01	2.45E-02	1.36E-01	rs1106933	6_166616136_A_G	1.00	7073	3.44E-07	GCST90277296
Phosphatidylcholine (17:0_18:1) levels	8	9324101	A	G	1.23E-01	2.36E-02	8.50E-01	rs2126263	8_9324101_G_A	1.00	7073	1.95E-07	GCST90277296
Phosphatidylcholine (17:0_18:1) levels	8	93920504	C	T	-3.18E-01	7.03E-02	1.56E-02	rs187552376	8_93920504_T_C	0.94	7073	6.09E-06	GCST90277296
Phosphatidylcholine (17:0_18:1) levels	9	137586795	G	T	1.53E-01	2.88E-02	1.03E-01	rs78308012	9_137586795_T_G	0.94	7073	1.11E-07	GCST90277296
Phosphatidylcholine (17:0_18:1) levels	12	14115139	T	A	2.96E-01	6.43E-02	1.84E-02	rs143506888	12_14115139_A_T	0.93	7073	4.13E-06	GCST90277296
Phosphatidylcholine (17:0_18:1) levels	13	78203799	T	G	-3.24E-01	7.14E-02	1.48E-02	rs76157703	13_78203799_G_T	0.97	7073	5.78E-06	GCST90277296
Phosphatidylcholine (17:0_18:1) levels	15	58387469	A	G	1.13E-01	1.71E-02	3.93E-01	rs7350789	15_58387469_G_A	1.00	7073	4.46E-11	GCST90277296
Phosphatidylcholine (17:0_18:1) levels	16	56960616	T	C	9.35E-02	1.87E-02	2.78E-01	rs17231506	16_56960616_C_T	1.00	7073	5.82E-07	GCST90277296
Phosphatidylcholine (17:0_18:1) levels	16	79172686	A	G	1.12E-01	2.50E-02	1.35E-01	rs12373089	16_79172686_G_A	0.98	7073	8.44E-06	GCST90277296
Phosphatidylcholine (17:0_18:1) levels	16	87644718	T	C	-7.54E-01	1.64E-01	2.67E-03	rs61737926	16_87644718_C_T	0.97	7073	4.10E-06	GCST90277296
Phosphatidylcholine (17:0_18:1) levels	18	44024688	T	C	-2.47E-01	5.36E-02	2.60E-02	rs141245990	18_44024688_C_T	0.98	7073	3.97E-06	GCST90277296
Phosphatidylcholine (17:0_18:1) levels	20	61233003	G	A	-9.30E-02	2.03E-02	2.23E-01	rs2003890	20_61233003_A_G	0.99	7073	4.57E-06	GCST90277296
Phosphatidylcholine (17:0_18:1) levels	20	61849802	A	G	6.46E-01	1.39E-01	4.15E-03	rs140419225	20_61849802_G_A	0.86	7073	3.40E-06	GCST90277296
Phosphatidylcholine (17:0_18:2) levels	1	62662654	G	A	8.61E-02	1.88E-02	7.38E-01	rs1168104	1_62662654_A_G	1.00	7170	4.96E-06	GCST90277297
Phosphatidylcholine (17:0_18:2) levels	1	99137108	T	C	2.26E-01	5.09E-02	2.84E-02	rs1253283	1_99137108_C_T	0.99	7170	9.36E-06	GCST90277297
Phosphatidylcholine (17:0_18:2) levels	1	211733797	C	T	-1.51E-01	3.24E-02	7.27E-02	rs72747028	1_211733797_T_C	0.98	7170	3.25E-06	GCST90277297
Phosphatidylcholine (17:0_18:2) levels	2	42156698	C	T	-1.25E-01	2.46E-02	1.32E-01	rs57518551	2_42156698_T_C	1.00	7170	3.79E-07	GCST90277297
Phosphatidylcholine (17:0_18:2) levels	4	113463133	C	T	-2.57E-01	5.27E-02	2.60E-02	rs192537901	4_113463133_T_C	0.97	7170	1.10E-06	GCST90277297
Phosphatidylcholine (17:0_18:2) levels	4	149246245	G	A	7.71E-02	1.68E-02	4.78E-01	rs7437408	4_149246245_A_G	1.00	7170	4.54E-06	GCST90277297
Phosphatidylcholine (17:0_18:2) levels	4	169401962	T	C	-1.00E-01	2.24E-02	1.66E-01	rs4478142	4_169401962_C_T	1.00	7170	7.67E-06	GCST90277297
Phosphatidylcholine (17:0_18:2) levels	5	3220235	A	C	1.43E-01	3.20E-02	7.78E-02	rs1609446	5_3220235_C_A	0.96	7170	7.97E-06	GCST90277297
Phosphatidylcholine (17:0_18:2) levels	5	40282763	T	C	-1.99E-01	4.28E-02	3.92E-02	rs115686032	5_40282763_C_T	0.99	7170	3.39E-06	GCST90277297
Phosphatidylcholine (17:0_18:2) levels	5	107005641	G	C	2.24E-01	4.98E-02	3.01E-02	rs111233850	5_107005641_C_G	0.99	7170	7.06E-06	GCST90277297
Phosphatidylcholine (17:0_18:2) levels	7	14912136	C	T	1.69E-01	3.65E-02	6.33E-02	rs73058910	7_14912136_T_C	0.89	7170	3.80E-06	GCST90277297
Phosphatidylcholine (17:0_18:2) levels	7	100927939	G	C	8.83E-02	1.91E-02	2.66E-01	rs34489224	7_100927939_C_G	0.98	7170	3.75E-06	GCST90277297
Phosphatidylcholine (17:0_18:2) levels	8	8532922	G	C	3.39E-01	7.35E-02	9.87E-01	rs4840981	8_8532922_C_G	1.00	7170	4.05E-06	GCST90277297
Phosphatidylcholine (17:0_18:2) levels	8	9327571	C	T	9.13E-02	1.91E-02	7.49E-01	rs2126260	8_9327571_T_C	1.00	7170	1.72E-06	GCST90277297
Phosphatidylcholine (17:0_18:2) levels	8	133696200	G	A	1.04E-01	2.21E-02	1.70E-01	rs12548274	8_133696200_A_G	0.98	7170	2.63E-06	GCST90277297
Phosphatidylcholine (17:0_18:2) levels	11	23815041	T	C	-1.32E-01	2.96E-02	8.91E-02	rs11027519	11_23815041_C_T	1.00	7170	8.04E-06	GCST90277297
Phosphatidylcholine (17:0_18:2) levels	11	61824890	G	A	2.59E-01	1.68E-02	4.08E-01	rs174566	11_61824890_A_G	1.00	7170	9.03E-53	GCST90277297
Phosphatidylcholine (17:0_18:2) levels	11	116778201	C	G	-1.54E-01	2.32E-02	8.49E-01	rs964184	11_116778201_G_C	1.00	7170	2.76E-11	GCST90277297
Phosphatidylcholine (17:0_18:2) levels	15	58416983	A	G	8.54E-02	1.68E-02	4.93E-01	rs422137	15_58416983_G_A	0.98	7170	3.66E-07	GCST90277297
Phosphatidylcholine (17:0_18:2) levels	15	58431476	T	C	1.15E-01	1.94E-02	2.58E-01	rs1800588	15_58431476_C_T	0.99	7170	3.31E-09	GCST90277297
Phosphatidylcholine (17:0_18:2) levels	15	73731833	T	C	-1.43E-01	3.18E-02	7.44E-02	rs141509040	15_73731833_C_T	0.99	7170	7.49E-06	GCST90277297
Phosphatidylcholine (17:0_18:2) levels	16	15036737	G	A	9.02E-02	1.77E-02	3.34E-01	rs6498540	16_15036737_A_G	0.99	7170	3.81E-07	GCST90277297
Phosphatidylcholine (17:0_18:2) levels	16	56960616	T	C	9.67E-02	1.85E-02	2.78E-01	rs17231506	16_56960616_C_T	1.00	7170	1.89E-07	GCST90277297
Phosphatidylcholine (17:0_18:2) levels	19	44938026	T	G	-1.28E-01	2.55E-02	1.27E-01	rs78592970	19_44938026_G_T	0.95	7170	5.18E-07	GCST90277297
Phosphatidylcholine (17:0_18:2) levels	22	20410911	C	T	2.25E-01	4.99E-02	2.91E-02	rs111314022	22_20410911_T_C	0.97	7170	6.54E-06	GCST90277297
Phosphatidylcholine (17:0_20:4) levels	1	4847659	T	G	1.69E-01	3.69E-02	5.56E-02	rs116643980	1_4847659_G_T	0.95	7106	4.65E-06	GCST90277298
Phosphatidylcholine (17:0_20:4) levels	1	27599934	G	A	2.70E-01	5.91E-02	2.25E-02	rs143309746	1_27599934_A_G	0.91	7106	4.94E-06	GCST90277298
Phosphatidylcholine (17:0_20:4) levels	1	62494579	C	T	8.84E-02	1.88E-02	7.33E-01	rs1168041	1_62494579_T_C	0.99	7106	2.75E-06	GCST90277298
Phosphatidylcholine (17:0_20:4) levels	1	236035822	T	A	8.51E-02	1.87E-02	2.62E-01	rs35788378	1_236035822_A_T	1.00	7106	5.52E-06	GCST90277298
Phosphatidylcholine (17:0_20:4) levels	2	142929648	A	G	-1.58E-01	3.47E-02	6.38E-02	rs190664666	2_142929648_G_A	0.99	7106	5.01E-06	GCST90277298
Phosphatidylcholine (17:0_20:4) levels	3	66040764	A	G	-2.48E-01	5.47E-02	2.48E-02	rs55725503	3_66040764_G_A	0.95	7106	5.71E-06	GCST90277298

Phosphatidylcholine (17:0_20:4) levels	5	38490169	G	A	9.88E-02	2.07E-02	2.08E-01	rs3110971	5_38490169_A_G	0.99	7106	1.78E-06	GCST90277298
Phosphatidylcholine (17:0_20:4) levels	5	132336964	C	T	8.44E-02	1.79E-02	3.23E-01	rs56399423	5_132336964_T_C	1.00	7106	2.49E-06	GCST90277298
Phosphatidylcholine (17:0_20:4) levels	6	158517411	A	C	-1.44E-01	3.11E-02	7.79E-02	rs142082962	6_158517411_C_A	0.99	7106	3.46E-06	GCST90277298
Phosphatidylcholine (17:0_20:4) levels	7	152050140	A	G	-1.03E-01	2.23E-02	1.81E-01	rs73161879	7_152050140_G_A	0.94	7106	3.77E-06	GCST90277298
Phosphatidylcholine (17:0_20:4) levels	8	85171256	T	C	-1.50E-01	3.24E-02	7.43E-02	rs143060591	8_85171256_C_T	0.97	7106	3.72E-06	GCST90277298
Phosphatidylcholine (17:0_20:4) levels	9	15293515	C	T	1.15E-01	2.42E-02	8.62E-01	rs585002	9_15293515_T_C	0.99	7106	2.33E-06	GCST90277298
Phosphatidylcholine (17:0_20:4) levels	9	137477951	A	G	1.31E-01	2.79E-02	1.10E-01	rs117580248	9_137477951_G_A	0.92	7106	2.78E-06	GCST90277298
Phosphatidylcholine (17:0_20:4) levels	11	61770929	C	G	-1.76E-01	1.71E-02	4.44E-01	rs174527	11_61770929_G_C	0.98	7106	1.14E-24	GCST90277298
Phosphatidylcholine (17:0_20:4) levels	11	61781553	A	G	-5.43E-01	1.59E-02	4.09E-01	rs174533	11_61781553_G_A	1.00	7106	9.69E-236	GCST90277298
Phosphatidylcholine (17:0_20:4) levels	11	61960675	T	C	-2.95E-01	5.26E-02	2.75E-02	rs57815521	11_61960675_C_T	0.99	7106	2.07E-08	GCST90277298
Phosphatidylcholine (17:0_20:4) levels	11	62195501	G	A	2.18E-01	3.47E-02	6.18E-02	rs140619100	11_62195501_A_G	0.99	7106	3.15E-10	GCST90277298
Phosphatidylcholine (17:0_20:4) levels	11	62267340	T	C	-1.88E-01	3.99E-02	4.80E-02	rs61896563	11_62267340_C_T	0.99	7106	2.47E-06	GCST90277298
Phosphatidylcholine (17:0_20:4) levels	11	62314821	G	T	1.94E-01	2.97E-02	8.73E-02	rs113394924	11_62314821_T_G	0.99	7106	8.01E-11	GCST90277298
Phosphatidylcholine (17:0_20:4) levels	11	75744328	T	A	-1.13E-01	1.99E-02	2.32E-01	rs600518	11_75744328_A_T	0.99	7106	1.42E-08	GCST90277298
Phosphatidylcholine (17:0_20:4) levels	12	88630462	C	T	-4.46E-01	8.83E-02	1.06E-02	rs74452899	12_88630462_T_C	0.85	7106	4.64E-07	GCST90277298
Phosphatidylcholine (17:0_20:4) levels	12	128751021	G	A	-9.68E-02	1.97E-02	2.63E-01	rs2398428	12_128751021_A_G	0.91	7106	8.96E-07	GCST90277298
Phosphatidylcholine (17:0_20:4) levels	13	37767109	T	C	8.82E-02	1.91E-02	2.74E-01	rs12429208	13_37767109_C_T	0.97	7106	3.76E-06	GCST90277298
Phosphatidylcholine (17:0_20:4) levels	14	81838369	A	G	-8.14E-02	1.81E-02	3.07E-01	rs11626405	14_81838369_G_A	1.00	7106	7.26E-06	GCST90277298
Phosphatidylcholine (17:0_20:4) levels	16	14611144	C	G	-8.56E-02	1.75E-02	3.61E-01	rs12928399	16_14611144_G_C	0.98	7106	1.08E-06	GCST90277298
Phosphatidylcholine (17:0_20:4) levels	18	44179195	C	T	9.99E-02	2.16E-02	1.84E-01	rs635196	18_44179195_T_C	1.00	7106	3.91E-06	GCST90277298
Phosphatidylcholine (17:0_20:4) levels	18	73095106	G	A	-9.21E-02	2.04E-02	7.87E-01	rs2469035	18_73095106_A_G	0.99	7106	6.69E-06	GCST90277298
Phosphatidylcholine (17:0_20:4) levels	19	19269704	G	A	-2.40E-01	3.80E-02	5.35E-02	rs187429064	19_19269704_A_G	0.95	7106	3.18E-10	GCST90277298
Phosphatidylcholine (17:0_20:4) levels	19	47696062	A	G	7.60E-02	1.70E-02	4.19E-01	rs11673120	19_47696062_G_A	0.98	7106	7.45E-06	GCST90277298
Phosphatidylcholine (17:0_20:4) levels	20	25397682	G	A	-7.75E-02	1.69E-02	5.36E-01	rs6083838	20_25397682_A_G	1.00	7106	4.51E-06	GCST90277298
Phosphatidylcholine (17:0_20:4) levels	21	20132472	A	G	1.45E-01	3.06E-02	9.14E-01	rs11088781	21_20132472_G_A	1.00	7106	2.27E-06	GCST90277298
Phosphatidylcholine (18:0_18:1) levels	1	94975736	T	C	-1.02E-01	1.76E-02	3.33E-01	rs2797620	1_94975736_C_T	1.00	7173	6.23E-09	GCST90277299
Phosphatidylcholine (18:0_18:1) levels	1	218198038	T	C	1.86E-01	3.80E-02	5.21E-02	rs34833702	1_218198038_C_T	0.98	7173	9.96E-07	GCST90277299
Phosphatidylcholine (18:0_18:1) levels	1	230188618	C	T	-1.28E-01	2.16E-02	1.80E-01	rs612577	1_230188618_T_C	1.00	7173	3.63E-09	GCST90277299
Phosphatidylcholine (18:0_18:1) levels	1	232142866	A	G	-1.07E-01	2.27E-02	1.62E-01	rs2183640	1_232142866_G_A	1.00	7173	2.30E-06	GCST90277299
Phosphatidylcholine (18:0_18:1) levels	2	97693419	T	C	8.36E-02	1.87E-02	7.23E-01	rs11123861	2_97693419_C_T	1.00	7173	8.22E-06	GCST90277299
Phosphatidylcholine (18:0_18:1) levels	3	155568069	C	T	-2.21E-01	4.80E-02	3.36E-02	rs535686	3_155568069_T_C	0.95	7173	4.00E-06	GCST90277299
Phosphatidylcholine (18:0_18:1) levels	3	180551409	T	G	-2.96E-01	6.59E-02	1.68E-02	rs115754811	3_180551409_G_T	0.98	7173	7.11E-06	GCST90277299
Phosphatidylcholine (18:0_18:1) levels	4	71877919	T	C	-8.17E-02	1.85E-02	2.77E-01	rs67835422	4_71877919_C_T	1.00	7173	9.60E-06	GCST90277299
Phosphatidylcholine (18:0_18:1) levels	5	86296114	T	C	9.39E-02	1.97E-02	7.65E-01	rs4629629	5_86296114_C_T	1.00	7173	1.83E-06	GCST90277299
Phosphatidylcholine (18:0_18:1) levels	6	11511662	A	G	-5.15E-01	1.15E-01	5.70E-03	rs140116915	6_11511662_G_A	0.92	7173	7.24E-06	GCST90277299
Phosphatidylcholine (18:0_18:1) levels	6	147266089	G	A	-1.53E-01	3.38E-02	6.69E-02	rs10457811	6_147266089_A_G	0.98	7173	6.19E-06	GCST90277299
Phosphatidylcholine (18:0_18:1) levels	7	50773538	A	G	2.28E-01	4.71E-02	3.37E-02	rs181751380	7_50773538_G_A	0.96	7173	1.34E-06	GCST90277299
Phosphatidylcholine (18:0_18:1) levels	8	9323885	G	A	1.07E-01	2.34E-02	8.50E-01	rs2169387	8_9323885_A_G	1.00	7173	4.82E-06	GCST90277299
Phosphatidylcholine (18:0_18:1) levels	9	111192133	A	G	-7.61E-02	1.71E-02	4.13E-01	rs12335533	9_111192133_G_A	1.00	7173	9.10E-06	GCST90277299
Phosphatidylcholine (18:0_18:1) levels	10	59020591	CT	C	1.61E-01	3.55E-02	6.56E-02	rs34677254	10_59020591_C_CT	0.90	7173	5.55E-06	GCST90277299
Phosphatidylcholine (18:0_18:1) levels	10	116636627	C	T	7.67E-02	1.67E-02	5.32E-01	rs11197775	10_116636627_T_C	1.00	7173	4.55E-06	GCST90277299
Phosphatidylcholine (18:0_18:1) levels	11	44029081	T	C	-7.42E-01	1.61E-01	2.86E-03	rs150862068	11_44029081_C_T	0.93	7173	3.97E-06	GCST90277299
Phosphatidylcholine (18:0_18:1) levels	11	91426863	C	T	4.21E-01	9.15E-02	8.81E-03	rs16916055	11_91426863_T_C	0.97	7173	4.28E-06	GCST90277299
Phosphatidylcholine (18:0_18:1) levels	12	34480655	T	C	1.38E-01	2.86E-02	9.72E-02	rs117207178	12_34480655_C_T	0.97	7173	1.38E-06	GCST90277299
Phosphatidylcholine (18:0_18:1) levels	12	79313979	C	T	-8.22E-02	1.85E-02	2.88E-01	rs11113650	12_79313979_T_C	0.99	7173	9.14E-06	GCST90277299
Phosphatidylcholine (18:0_18:1) levels	12	92131416	T	C	2.41E-01	4.73E-02	3.35E-02	rs73215839	12_92131416_C_T	0.96	7173	3.62E-07	GCST90277299
Phosphatidylcholine (18:0_18:1) levels	12	121025485	C	T	-8.26E-02	1.68E-02	4.90E-01	rs2708081	12_121025485_T_C	1.00	7173	8.38E-07	GCST90277299
Phosphatidylcholine (18:0_18:1) levels	15	58432593	A	T	8.56E-02	1.83E-02	3.11E-01	rs8034802	15_58432593_T_A	1.00	7173	2.94E-06	GCST90277299
Phosphatidylcholine (18:0_18:1) levels	16	32012667	T	A	3.02E-01	6.41E-02	2.06E-02	rs151331635	16_32012667_A_T	0.84	7173	2.43E-06	GCST90277299
Phosphatidylcholine (18:0_18:1) levels	16	70566761	C	T	-2.07E-01	4.45E-02	3.69E-02	rs77191869	16_70566761_T_C	0.98	7173	3.15E-06	GCST90277299
Phosphatidylcholine (18:0_18:1) levels	16	71310208	G	A	-1.18E-01	2.64E-02	1.12E-01	rs34832413	16_71310208_A_G	0.99	7173	7.68E-06	GCST90277299
Phosphatidylcholine (18:0_18:1) levels	17	31609275	T	C	-7.81E-02	1.77E-02	3.36E-01	rs1034642	17_31609275_C_T	0.99	7173	9.86E-06	GCST90277299
Phosphatidylcholine (18:0_18:1) levels	17	43849174	A	G	1.72E-01	3.85E-02	4.96E-02	rs77027238	17_43849174_G_A	0.97	7173	8.24E-06	GCST90277299
Phosphatidylcholine (18:0_18:1) levels	18	48681930	T	C	1.51E-01	3.42E-02	6.71E-02	rs113179719	18_48681930_C_T	0.94	7173	9.41E-06	GCST90277299
Phosphatidylcholine (18:0_18:1) levels	19	1991747	A	G	1.72E-01	3.71E-02	5.57E-02	rs7252513	19_1991747_G_A	0.98	7173	3.48E-06	GCST90277299
Phosphatidylcholine (18:0_18:1) levels	22	46721676	T	C	9.66E-02	2.16E-02	1.96E-01	rs5769111	22_46721676_C_T	0.94	7173	7.44E-06	GCST90277299
Phosphatidylcholine (18:0_18:2) levels	1	62633352	C	T	-1.01E-01	1.88E-02	2.62E-01	rs10889352	1_62633352_T_C	1.00	7174	8.49E-08	GCST90277300

Phosphatidylcholine (18:0_18:2) levels	1	95228712	G	A	2.00E-01	2.78E-02	9.02E-01	rs6678964	1_95228712_A_G	1.00	7174	6.84E-13	GCST90277300
Phosphatidylcholine (18:0_18:2) levels	1	189927375	T	C	-8.26E-02	1.85E-02	2.89E-01	rs12122878	1_189927375_C_T	0.98	7174	7.96E-06	GCST90277300
Phosphatidylcholine (18:0_18:2) levels	1	211735543	T	C	-1.73E-01	3.21E-02	7.34E-02	rs55917360	1_211735543_C_T	0.99	7174	6.90E-08	GCST90277300
Phosphatidylcholine (18:0_18:2) levels	1	212247960	G	A	-2.18E-01	4.56E-02	3.45E-02	rs150457719	1_212247960_A_G	0.98	7174	1.74E-06	GCST90277300
Phosphatidylcholine (18:0_18:2) levels	2	5749013	A	G	4.63E-01	1.02E-01	7.11E-03	rs12620952	2_5749013_G_A	0.98	7174	5.72E-06	GCST90277300
Phosphatidylcholine (18:0_18:2) levels	2	85365218	G	A	1.24E-01	2.70E-02	1.02E-01	rs72840078	2_85365218_A_G	0.99	7174	4.49E-06	GCST90277300
Phosphatidylcholine (18:0_18:2) levels	2	121313828	T	G	1.00E-01	2.26E-02	1.69E-01	rs77588674	2_121313828_G_T	0.95	7174	8.96E-06	GCST90277300
Phosphatidylcholine (18:0_18:2) levels	2	162918693	G	A	7.52E-02	1.68E-02	4.74E-01	rs6432738	2_162918693_A_G	0.97	7174	7.24E-06	GCST90277300
Phosphatidylcholine (18:0_18:2) levels	2	206629893	C	G	-2.70E-01	6.06E-02	1.91E-02	rs16838403	2_206629893_G_C	1.00	7174	8.74E-06	GCST90277300
Phosphatidylcholine (18:0_18:2) levels	4	14739231	G	A	1.33E-01	2.86E-02	9.93E-02	rs73126869	4_14739231_A_G	0.95	7174	3.48E-06	GCST90277300
Phosphatidylcholine (18:0_18:2) levels	4	110207431	T	C	9.38E-02	2.12E-02	7.97E-01	rs5022521	4_110207431_C_T	0.94	7174	9.45E-06	GCST90277300
Phosphatidylcholine (18:0_18:2) levels	5	86295297	T	G	8.76E-02	1.96E-02	7.64E-01	rs6878903	5_86295297_G_T	1.00	7174	8.02E-06	GCST90277300
Phosphatidylcholine (18:0_18:2) levels	5	138238036	A	G	9.40E-02	2.09E-02	1.92E-01	rs10040989	5_138238036_G_A	1.00	7174	6.86E-06	GCST90277300
Phosphatidylcholine (18:0_18:2) levels	6	46779152	A	G	-1.30E-01	2.88E-02	9.52E-02	rs12211498	6_46779152_G_A	0.98	7174	6.54E-06	GCST90277300
Phosphatidylcholine (18:0_18:2) levels	8	9325848	G	A	1.26E-01	2.34E-02	8.52E-01	rs9987289	8_9325848_A_G	1.00	7174	8.75E-08	GCST90277300
Phosphatidylcholine (18:0_18:2) levels	9	15298668	C	T	1.27E-01	2.73E-02	8.99E-01	rs675849	9_15298668_T_C	0.99	7174	3.50E-06	GCST90277300
Phosphatidylcholine (18:0_18:2) levels	9	80727726	C	T	-3.38E-01	7.23E-02	1.42E-02	rs192934615	9_80727726_T_C	0.96	7174	3.07E-06	GCST90277300
Phosphatidylcholine (18:0_18:2) levels	9	132230557	A	G	-1.05E-01	2.31E-02	1.71E-01	rs2274855	9_132230557_G_A	0.89	7174	5.43E-06	GCST90277300
Phosphatidylcholine (18:0_18:2) levels	11	43971474	A	G	7.73E-02	1.71E-02	6.17E-01	rs7932326	11_43971474_G_A	1.00	7174	6.29E-06	GCST90277300
Phosphatidylcholine (18:0_18:2) levels	11	61852357	A	C	-2.47E-01	1.67E-02	5.82E-01	rs174594	11_61852357_C_A	1.00	7174	8.13E-49	GCST90277300
Phosphatidylcholine (18:0_18:2) levels	11	107889276	A	G	-1.09E-01	2.38E-02	8.60E-01	rs10890773	11_107889276_G_A	1.00	7174	4.83E-06	GCST90277300
Phosphatidylcholine (18:0_18:2) levels	11	116778201	C	G	-1.28E-01	2.31E-02	8.49E-01	rs964184	11_116778201_G_C	1.00	7174	3.04E-08	GCST90277300
Phosphatidylcholine (18:0_18:2) levels	11	126095437	C	T	-1.65E-01	3.66E-02	5.50E-02	rs564216	11_126095437_T_C	0.98	7174	6.38E-06	GCST90277300
Phosphatidylcholine (18:0_18:2) levels	12	62017823	A	G	7.96E-02	1.68E-02	4.03E-01	rs1440724	12_62017823_G_A	1.00	7174	2.18E-06	GCST90277300
Phosphatidylcholine (18:0_18:2) levels	15	58416983	A	G	9.09E-02	1.67E-02	4.93E-01	rs422137	15_58416983_G_A	0.98	7174	5.70E-08	GCST90277300
Phosphatidylcholine (18:0_18:2) levels	15	58431476	T	C	1.35E-01	1.93E-02	2.58E-01	rs1800588	15_58431476_C_T	0.99	7174	2.52E-12	GCST90277300
Phosphatidylcholine (18:0_18:2) levels	16	15043593	A	G	9.57E-02	1.77E-02	3.32E-01	rs62039480	16_15043593_G_A	1.00	7174	6.94E-08	GCST90277300
Phosphatidylcholine (18:0_18:2) levels	16	56960616	T	C	1.07E-01	1.85E-02	2.78E-01	rs17231506	16_56960616_C_T	1.00	7174	6.78E-09	GCST90277300
Phosphatidylcholine (18:0_18:2) levels	17	44755436	A	G	1.11E-01	2.39E-02	1.39E-01	rs77008693	17_44755436_G_A	1.00	7174	3.40E-06	GCST90277300
Phosphatidylcholine (18:0_18:2) levels	18	13728480	T	C	-8.43E-02	1.75E-02	3.26E-01	rs34031934	18_13728480_C_T	1.00	7174	1.43E-06	GCST90277300
Phosphatidylcholine (18:0_18:2) levels	18	49649558	C	T	6.00E-01	1.25E-01	4.85E-03	rs117528775	18_49649558_T_C	0.97	7174	1.65E-06	GCST90277300
Phosphatidylcholine (18:0_18:2) levels	21	32410680	T	C	-1.64E-01	3.51E-02	6.40E-02	rs112121830	21_32410680_C_T	0.93	7174	3.10E-06	GCST90277300
Phosphatidylcholine (18:0_18:3) levels	1	91728966	A	G	-8.29E-02	1.76E-02	3.94E-01	rs10874911	1_91728966_G_A	0.99	6692	2.41E-06	GCST90277301
Phosphatidylcholine (18:0_18:3) levels	1	94975736	T	C	-9.71E-02	1.83E-02	3.33E-01	rs2797620	1_94975736_C_T	1.00	6692	1.07E-07	GCST90277301
Phosphatidylcholine (18:0_18:3) levels	1	230166915	G	A	9.12E-02	1.97E-02	7.47E-01	rs4846841	1_230166915_A_G	1.00	6692	3.79E-06	GCST90277301
Phosphatidylcholine (18:0_18:3) levels	2	27508073	C	T	-8.53E-02	1.81E-02	6.51E-01	rs1260326	2_27508073_T_C	1.00	6692	2.52E-06	GCST90277301
Phosphatidylcholine (18:0_18:3) levels	4	94538445	C	G	3.80E-01	8.33E-02	1.26E-02	rs79831249	4_94538445_G_C	0.86	6692	5.18E-06	GCST90277301
Phosphatidylcholine (18:0_18:3) levels	5	44740041	T	C	1.38E-01	2.99E-02	9.52E-02	rs75593273	5_44740041_C_T	0.93	6692	3.91E-06	GCST90277301
Phosphatidylcholine (18:0_18:3) levels	5	122510180	T	G	-9.00E-02	1.94E-02	2.81E-01	rs181264690	5_122510180_G_T	1.00	6692	3.53E-06	GCST90277301
Phosphatidylcholine (18:0_18:3) levels	5	176998593	A	C	-2.95E-01	5.93E-02	2.35E-02	rs183478874	5_176998593_C_A	0.95	6692	6.57E-07	GCST90277301
Phosphatidylcholine (18:0_18:3) levels	6	41878684	A	T	1.24E-01	2.71E-02	1.18E-01	rs72867166	6_41878684_T_A	0.99	6692	4.87E-06	GCST90277301
Phosphatidylcholine (18:0_18:3) levels	11	47350047	T	C	-1.25E-01	2.82E-02	1.05E-01	rs3729986	11_47350047_C_T	0.98	6692	8.87E-06	GCST90277301
Phosphatidylcholine (18:0_18:3) levels	11	130971030	A	T	-2.94E-01	6.63E-02	1.80E-02	rs11222413	11_130971030_T_A	0.96	6692	9.56E-06	GCST90277301
Phosphatidylcholine (18:0_18:3) levels	12	12204586	G	A	6.14E-01	1.35E-01	4.89E-03	rs116844659	12_12204586_A_G	0.91	6692	5.67E-06	GCST90277301
Phosphatidylcholine (18:0_18:3) levels	12	30829729	T	G	-4.48E-01	1.01E-01	7.53E-03	rs118094862	12_30829729_G_T	0.94	6692	9.08E-06	GCST90277301
Phosphatidylcholine (18:0_18:3) levels	12	92131416	T	C	2.69E-01	4.91E-02	3.35E-02	rs73215839	12_92131416_C_T	0.96	6692	4.33E-08	GCST90277301
Phosphatidylcholine (18:0_18:3) levels	12	120977490	A	G	-8.06E-02	1.74E-02	5.47E-01	rs7139079	12_120977490_G_A	1.00	6692	3.50E-06	GCST90277301
Phosphatidylcholine (18:0_18:3) levels	15	95071963	G	T	3.16E-01	7.11E-02	1.55E-02	rs16949722	15_95071963_T_G	0.96	6692	8.83E-06	GCST90277301
Phosphatidylcholine (18:0_18:3) levels	16	56951643	G	A	9.57E-02	2.16E-02	7.92E-01	rs12448528	16_56951643_A_G	0.97	6692	9.70E-06	GCST90277301
Phosphatidylcholine (18:0_18:3) levels	16	76816258	A	T	3.06E-01	6.43E-02	1.90E-02	rs4888557	16_76816258_T_A	0.98	6692	2.00E-06	GCST90277301
Phosphatidylcholine (18:0_18:3) levels	17	13574441	A	G	3.04E-01	6.71E-02	1.78E-02	rs143029300	17_13574441_G_A	0.93	6692	6.21E-06	GCST90277301
Phosphatidylcholine (18:0_18:3) levels	19	14102503	A	G	2.90E-01	6.32E-02	2.11E-02	rs62122127	19_14102503_G_A	0.92	6692	4.65E-06	GCST90277301
Phosphatidylcholine (18:0_18:3) levels	19	21959621	T	C	5.15E-01	1.15E-01	6.06E-03	rs145856648	19_21959621_C_T	0.94	6692	7.78E-06	GCST90277301
Phosphatidylcholine (18:0_18:3) levels	19	56525734	G	C	-9.70E-02	2.04E-02	2.34E-01	rs16987303	19_56525734_C_G	0.99	6692	2.11E-06	GCST90277301
Phosphatidylcholine (18:0_18:3) levels	21	33084026	T	C	8.10E-02	1.79E-02	6.23E-01	rs2834084	21_33084026_C_T	1.00	6692	6.43E-06	GCST90277301
Phosphatidylcholine (18:0_20:2) levels	1	214966966	A	G	-2.94E-01	6.51E-02	2.20E-02	rs111595890	1_214966966_G_A	0.97	5778	6.17E-06	GCST90277302
Phosphatidylcholine (18:0_20:2) levels	2	68444883	T	C	1.88E-01	4.26E-02	9.48E-01	rs6744368	2_68444883_C_T	0.98	5778	9.85E-06	GCST90277302

Phosphatidylcholine (18:0_20:2) levels	4	43912586	T	C	-8.84E-02	1.91E-02	6.21E-01	rs12513382	4_43912586_C_T	1.00	5778	3.68E-06	GCST90277302
Phosphatidylcholine (18:0_20:2) levels	5	86253956	A	G	-1.99E-01	4.44E-02	4.79E-02	rs112997491	5_86253956_G_A	1.00	5778	8.03E-06	GCST90277302
Phosphatidylcholine (18:0_20:2) levels	5	123138027	ACC	A	1.28E-01	2.62E-02	1.49E-01	rs112789380	5_123138027_A_ACC	1.00	5778	1.02E-06	GCST90277302
Phosphatidylcholine (18:0_20:2) levels	5	166671878	G	T	-8.97E-02	1.88E-02	5.93E-01	rs1422819	5_166671878_T_G	1.00	5778	1.97E-06	GCST90277302
Phosphatidylcholine (18:0_20:2) levels	8	5603995	T	C	-1.05E-01	2.36E-02	1.90E-01	rs67207258	8_5603995_C_T	0.99	5778	9.22E-06	GCST90277302
Phosphatidylcholine (18:0_20:2) levels	8	12981507	G	A	-2.90E-01	6.24E-02	2.38E-02	rs112483795	8_12981507_A_G	0.95	5778	3.37E-06	GCST90277302
Phosphatidylcholine (18:0_20:2) levels	11	61770929	C	G	1.02E-01	1.90E-02	4.44E-01	rs174527	11_61770929_G_C	0.98	5778	8.17E-08	GCST90277302
Phosphatidylcholine (18:0_20:2) levels	11	61803311	C	T	2.82E-01	1.86E-02	4.07E-01	rs174547	11_61803311_T_C	1.00	5778	4.05E-51	GCST90277302
Phosphatidylcholine (18:0_20:2) levels	12	90339893	G	A	8.70E-02	1.95E-02	3.48E-01	rs7959861	12_90339893_A_G	1.00	5778	8.23E-06	GCST90277302
Phosphatidylcholine (18:0_20:2) levels	12	120977490	A	G	-9.90E-02	1.87E-02	5.47E-01	rs7139079	12_120977490_G_A	1.00	5778	1.17E-07	GCST90277302
Phosphatidylcholine (18:0_20:2) levels	13	37429471	T	C	-1.23E-01	2.73E-02	1.38E-01	rs17238104	13_37429471_C_T	0.97	5778	7.01E-06	GCST90277302
Phosphatidylcholine (18:0_20:2) levels	13	73360255	G	T	4.27E-01	9.54E-02	1.01E-02	rs147220276	13_73360255_T_G	0.97	5778	7.84E-06	GCST90277302
Phosphatidylcholine (18:0_20:2) levels	14	24946787	A	T	-9.51E-02	2.06E-02	7.11E-01	rs9671254	14_24946787_T_A	0.99	5778	3.88E-06	GCST90277302
Phosphatidylcholine (18:0_20:2) levels	16	15045737	C	T	9.79E-02	1.88E-02	4.36E-01	rs34955778	16_15045737_T_C	0.99	5778	2.01E-07	GCST90277302
Phosphatidylcholine (18:0_20:2) levels	17	54741041	A	G	1.90E-01	3.97E-02	9.40E-01	rs8068923	17_54741041_G_A	0.99	5778	1.81E-06	GCST90277302
Phosphatidylcholine (18:0_20:2) levels	20	6660791	T	C	1.82E-01	4.06E-02	5.94E-02	rs8183765	20_6660791_C_T	0.92	5778	8.04E-06	GCST90277302
Phosphatidylcholine (18:0_20:2) levels	22	27828806	G	A	-1.23E-01	2.65E-02	1.50E-01	rs6005648	22_27828806_A_G	0.97	5778	3.25E-06	GCST90277302
Phosphatidylcholine (18:0_20:3) levels	1	74502464	C	T	2.27E-01	5.06E-02	2.92E-02	rs116259186	1_74502464_T_C	0.98	7169	7.49E-06	GCST90277303
Phosphatidylcholine (18:0_20:3) levels	1	94975736	T	C	-1.16E-01	1.75E-02	3.33E-01	rs2797620	1_94975736_C_T	1.00	7169	3.27E-11	GCST90277303
Phosphatidylcholine (18:0_20:3) levels	1	242606205	A	G	8.93E-02	1.99E-02	2.34E-01	rs11577288	1_242606205_G_A	0.98	7169	7.14E-06	GCST90277303
Phosphatidylcholine (18:0_20:3) levels	2	169055662	A	C	3.35E-01	7.44E-02	1.36E-02	rs13001426	2_169055662_C_A	0.92	7169	6.79E-06	GCST90277303
Phosphatidylcholine (18:0_20:3) levels	3	37451054	G	A	-9.44E-02	2.10E-02	2.01E-01	rs62239496	3_37451054_A_G	0.99	7169	7.30E-06	GCST90277303
Phosphatidylcholine (18:0_20:3) levels	3	148308352	C	A	-1.79E-01	3.94E-02	5.26E-02	rs114162005	3_148308352_A_C	0.89	7169	5.96E-06	GCST90277303
Phosphatidylcholine (18:0_20:3) levels	4	16403106	A	T	3.71E-01	8.28E-02	1.05E-02	rs139676091	4_16403106_T_A	0.97	7169	7.59E-06	GCST90277303
Phosphatidylcholine (18:0_20:3) levels	4	158046658	G	C	-1.27E-01	2.88E-02	9.59E-02	rs78009561	4_158046658_C_G	0.98	7169	9.94E-06	GCST90277303
Phosphatidylcholine (18:0_20:3) levels	5	16863519	G	A	-2.04E-01	4.39E-02	3.83E-02	rs140123208	5_16863519_A_G	0.97	7169	3.45E-06	GCST90277303
Phosphatidylcholine (18:0_20:3) levels	5	33853083	T	C	-1.97E-01	4.29E-02	4.09E-02	rs192117305	5_33853083_C_T	0.96	7169	4.38E-06	GCST90277303
Phosphatidylcholine (18:0_20:3) levels	5	168832803	G	T	1.43E-01	3.01E-02	8.56E-02	rs72827678	5_168832803_T_G	0.99	7169	2.15E-06	GCST90277303
Phosphatidylcholine (18:0_20:3) levels	6	32238762	T	C	-9.51E-02	2.08E-02	2.00E-01	rs3134937	6_32238762_C_T	0.99	7169	4.78E-06	GCST90277303
Phosphatidylcholine (18:0_20:3) levels	9	130575137	A	G	2.89E-01	6.14E-02	1.94E-02	rs151178379	9_130575137_G_A	0.99	7169	2.48E-06	GCST90277303
Phosphatidylcholine (18:0_20:3) levels	9	133667068	A	G	-2.99E-01	6.71E-02	1.64E-02	rs56205329	9_133667068_G_A	0.94	7169	8.55E-06	GCST90277303
Phosphatidylcholine (18:0_20:3) levels	11	61779596	A	G	2.36E-01	2.65E-02	1.12E-01	rs7943728	11_61779596_G_A	1.00	7169	6.87E-19	GCST90277303
Phosphatidylcholine (18:0_20:3) levels	11	124328766	A	G	3.64E-01	8.18E-02	1.14E-02	rs140588245	11_124328766_G_A	0.93	7169	8.52E-06	GCST90277303
Phosphatidylcholine (18:0_20:3) levels	12	6260820	C	A	2.10E-01	4.47E-02	3.81E-02	rs112534228	12_6260820_A_C	0.94	7169	2.78E-06	GCST90277303
Phosphatidylcholine (18:0_20:3) levels	12	120978847	C	A	7.75E-02	1.72E-02	3.77E-01	rs1169288	12_120978847_A_C	1.00	7169	6.39E-06	GCST90277303
Phosphatidylcholine (18:0_20:3) levels	14	33476504	A	T	-1.11E-01	2.23E-02	1.74E-01	rs11847009	14_33476504_T_A	0.99	7169	6.87E-07	GCST90277303
Phosphatidylcholine (18:0_20:3) levels	15	58368884	C	T	1.83E-01	3.87E-02	4.79E-02	rs59739041	15_58368884_T_C	0.99	7169	2.32E-06	GCST90277303
Phosphatidylcholine (18:0_20:3) levels	16	15036737	G	A	-1.99E-01	1.76E-02	3.34E-01	rs6498540	16_15036737_A_G	0.99	7169	2.09E-29	GCST90277303
Phosphatidylcholine (18:0_20:3) levels	16	15773795	G	T	8.85E-02	1.84E-02	2.96E-01	rs56374730	16_15773795_T_G	0.99	7169	1.44E-06	GCST90277303
Phosphatidylcholine (18:0_20:3) levels	16	78589971	C	G	1.52E-01	3.16E-02	7.83E-02	rs11648397	16_78589971_G_C	0.98	7169	1.59E-06	GCST90277303
Phosphatidylcholine (18:0_20:3) levels	17	51561964	T	C	-2.36E-01	5.25E-02	2.75E-02	rs116917626	17_51561964_C_T	0.97	7169	6.79E-06	GCST90277303
Phosphatidylcholine (18:0_20:3) levels	19	18294126	G	A	-8.84E-02	1.77E-02	6.66E-01	rs4808779	19_18294126_A_G	0.99	7169	5.93E-07	GCST90277303
Phosphatidylcholine (18:0_20:3) levels	20	32243354	C	G	-7.52E-02	1.68E-02	4.93E-01	rs6141277	20_32243354_G_C	1.00	7169	7.41E-06	GCST90277303
Phosphatidylcholine (18:0_20:3) levels	20	44413724	T	C	-1.84E-01	3.72E-02	5.24E-02	rs1800961	20_44413724_C_T	1.00	7169	7.41E-07	GCST90277303
Phosphatidylcholine (18:0_20:3) levels	20	62310086	A	C	-8.13E-02	1.71E-02	3.99E-01	rs2379129	20_62310086_C_A	0.99	7169	2.15E-06	GCST90277303
Phosphatidylcholine (18:0_20:4) levels	1	94975736	T	C	-1.01E-01	1.74E-02	3.33E-01	rs2797620	1_94975736_C_T	1.00	7174	6.11E-09	GCST90277304
Phosphatidylcholine (18:0_20:4) levels	3	64950744	C	T	-1.06E-01	2.30E-02	1.46E-01	rs938822	3_64950744_T_C	0.99	7174	4.66E-06	GCST90277304
Phosphatidylcholine (18:0_20:4) levels	5	38490169	G	A	9.57E-02	2.04E-02	2.08E-01	rs3110971	5_38490169_A_G	0.99	7174	2.72E-06	GCST90277304
Phosphatidylcholine (18:0_20:4) levels	5	132304843	G	A	7.92E-02	1.69E-02	4.20E-01	rs6860806	5_132304843_A_G	0.99	7174	2.78E-06	GCST90277304
Phosphatidylcholine (18:0_20:4) levels	6	42979275	C	G	7.62E-02	1.66E-02	4.58E-01	rs9462860	6_42979275_G_C	0.99	7174	4.66E-06	GCST90277304
Phosphatidylcholine (18:0_20:4) levels	10	36620136	T	C	-7.82E-02	1.68E-02	5.78E-01	rs2990536	10_36620136_C_T	1.00	7174	3.48E-06	GCST90277304
Phosphatidylcholine (18:0_20:4) levels	10	124924165	G	A	1.46E-01	3.20E-02	7.19E-02	rs61873274	10_124924165_A_G	0.98	7174	5.07E-06	GCST90277304
Phosphatidylcholine (18:0_20:4) levels	11	61012351	A	G	1.24E-01	2.47E-02	1.34E-01	rs12275418	11_61012351_G_A	1.00	7174	4.66E-07	GCST90277304
Phosphatidylcholine (18:0_20:4) levels	11	61481911	A	C	-1.63E-01	2.13E-02	8.03E-01	rs3019200	11_61481911_C_A	1.00	7174	2.57E-14	GCST90277304
Phosphatidylcholine (18:0_20:4) levels	11	61594967	G	A	-2.49E-01	4.47E-02	3.69E-02	rs79136768	11_61594967_A_G	0.98	7174	2.71E-08	GCST90277304
Phosphatidylcholine (18:0_20:4) levels	11	61744026	T	C	2.43E-01	2.45E-02	1.34E-01	rs3741252	11_61744026_C_T	0.99	7174	5.54E-23	GCST90277304
Phosphatidylcholine (18:0_20:4) levels	11	61776027	C	T	-6.66E-01	1.51E-02	4.14E-01	rs174528	11_61776027_T_C	1.00	7174	0.00E+00	GCST90277304

Phosphatidylcholine (18:0_20:4) levels	11	62056826	A	G	-3.46E-01	4.18E-02	4.33E-02	rs147981159	11_62056826_G_A	0.96	7174	1.54E-16	GCST90277304
Phosphatidylcholine (18:0_20:4) levels	11	62454004	A	G	1.49E-01	1.97E-02	2.56E-01	rs4382917	11_62454004_G_A	0.92	7174	4.68E-14	GCST90277304
Phosphatidylcholine (18:0_20:4) levels	11	75722099	A	G	-8.77E-02	1.68E-02	4.93E-01	rs1219539	11_75722099_G_A	0.99	7174	1.74E-07	GCST90277304
Phosphatidylcholine (18:0_20:4) levels	11	75744328	T	A	-1.01E-01	1.98E-02	2.32E-01	rs600518	11_75744328_A_T	0.99	7174	3.49E-07	GCST90277304
Phosphatidylcholine (18:0_20:4) levels	12	91627546	CA	C	1.26E-01	2.68E-02	1.07E-01	rs144719617	12_91627546_C_CA	1.00	7174	2.69E-06	GCST90277304
Phosphatidylcholine (18:0_20:4) levels	13	98690376	T	C	-7.53E-02	1.68E-02	4.65E-01	rs2802390	13_98690376_C_T	0.99	7174	7.51E-06	GCST90277304
Phosphatidylcholine (18:0_20:4) levels	14	83647468	A	G	-2.06E-01	4.63E-02	3.40E-02	rs139102782	14_83647468_G_A	0.96	7174	9.06E-06	GCST90277304
Phosphatidylcholine (18:0_20:4) levels	15	58431740	A	G	9.37E-02	1.90E-02	2.67E-01	rs2070895	15_58431740_G_A	0.98	7174	8.73E-07	GCST90277304
Phosphatidylcholine (18:0_20:4) levels	16	1939264	T	G	2.56E-01	4.50E-02	3.59E-02	rs147549994	16_1939264_G_T	0.95	7174	1.35E-08	GCST90277304
Phosphatidylcholine (18:0_20:4) levels	16	15033677	A	T	-9.66E-02	1.79E-02	3.15E-01	rs72789541	16_15033677_T_A	1.00	7174	6.36E-08	GCST90277304
Phosphatidylcholine (18:0_20:4) levels	16	56972678	T	C	-9.58E-02	2.16E-02	1.77E-01	rs7499892	16_56972678_C_T	1.00	7174	9.21E-06	GCST90277304
Phosphatidylcholine (18:0_20:4) levels	16	57423359	G	A	-1.03E-01	2.19E-02	1.72E-01	rs223841	16_57423359_A_G	1.00	7174	2.89E-06	GCST90277304
Phosphatidylcholine (18:0_20:4) levels	18	73092300	G	A	-1.02E-01	2.03E-02	7.89E-01	rs1477491	18_73092300_A_G	1.00	7174	5.06E-07	GCST90277304
Phosphatidylcholine (18:0_20:4) levels	19	7552489	C	G	-1.04E-01	2.16E-02	8.18E-01	rs793864	19_7552489_G_C	0.97	7174	1.50E-06	GCST90277304
Phosphatidylcholine (18:0_20:4) levels	19	15845766	A	G	-7.71E-02	1.74E-02	3.57E-01	rs73005469	19_15845766_G_A	0.99	7174	9.40E-06	GCST90277304
Phosphatidylcholine (18:0_20:4) levels	19	19269704	G	A	-2.02E-01	3.75E-02	5.35E-02	rs187429064	19_19269704_A_G	0.95	7174	7.42E-08	GCST90277304
Phosphatidylcholine (18:0_20:5) levels	1	8828055	T	C	-7.79E-02	1.75E-02	3.54E-01	rs4908782	1_8828055_C_T	0.99	7121	8.94E-06	GCST90277305
Phosphatidylcholine (18:0_20:5) levels	1	169897372	CTT	C	2.12E-01	4.19E-02	4.15E-02	rs138053477	1_169897372_C_CTT	1.00	7121	4.26E-07	GCST90277305
Phosphatidylcholine (18:0_20:5) levels	1	210394086	G	A	-7.56E-02	1.71E-02	4.27E-01	rs1076708	1_210394086_A_G	1.00	7121	9.92E-06	GCST90277305
Phosphatidylcholine (18:0_20:5) levels	3	139980152	G	A	-2.02E-01	4.30E-02	3.99E-02	rs35226034	3_139980152_A_G	0.98	7121	2.57E-06	GCST90277305
Phosphatidylcholine (18:0_20:5) levels	3	169305300	C	T	-2.28E-01	5.14E-02	2.78E-02	rs12487951	3_169305300_T_C	0.97	7121	9.17E-06	GCST90277305
Phosphatidylcholine (18:0_20:5) levels	3	176095628	C	T	1.68E-01	3.59E-02	6.35E-02	rs71629271	3_176095628_T_C	0.91	7121	2.97E-06	GCST90277305
Phosphatidylcholine (18:0_20:5) levels	4	37392086	G	A	-7.81E-02	1.67E-02	5.38E-01	rs2054498	4_37392086_A_G	1.00	7121	2.78E-06	GCST90277305
Phosphatidylcholine (18:0_20:5) levels	5	113783266	C	G	-7.94E-02	1.78E-02	3.87E-01	rs12656795	5_113783266_G_C	0.93	7121	8.21E-06	GCST90277305
Phosphatidylcholine (18:0_20:5) levels	5	174893516	A	G	2.64E-01	5.63E-02	2.67E-02	rs2431163	5_174893516_G_A	0.85	7121	2.81E-06	GCST90277305
Phosphatidylcholine (18:0_20:5) levels	6	11061917	T	C	1.19E-01	2.54E-02	1.23E-01	rs9380073	6_11061917_C_T	1.00	7121	2.65E-06	GCST90277305
Phosphatidylcholine (18:0_20:5) levels	6	67555567	T	G	-2.00E-01	4.51E-02	3.69E-02	rs187698886	6_67555567_G_T	0.97	7121	9.12E-06	GCST90277305
Phosphatidylcholine (18:0_20:5) levels	7	71849296	T	C	-7.68E-02	1.70E-02	4.35E-01	rs2023988	7_71849296_C_T	1.00	7121	6.28E-06	GCST90277305
Phosphatidylcholine (18:0_20:5) levels	7	132693734	A	C	-8.06E-02	1.71E-02	4.09E-01	rs7780677	7_132693734_C_A	1.00	7121	2.52E-06	GCST90277305
Phosphatidylcholine (18:0_20:5) levels	7	147506779	C	T	-5.28E-01	1.18E-01	5.52E-03	rs145259113	7_147506779_T_C	0.91	7121	7.30E-06	GCST90277305
Phosphatidylcholine (18:0_20:5) levels	9	16421864	CCT	C	-7.84E-02	1.68E-02	4.64E-01	rs5896690	9_16421864_C_CCT	0.99	7121	3.08E-06	GCST90277305
Phosphatidylcholine (18:0_20:5) levels	10	116536092	T	C	-4.41E-01	9.75E-02	8.57E-03	rs146438407	10_116536092_C_T	0.85	7121	6.21E-06	GCST90277305
Phosphatidylcholine (18:0_20:5) levels	11	61481911	A	C	-1.08E-01	2.15E-02	8.03E-01	rs3019200	11_61481911_C_A	1.00	7121	4.74E-07	GCST90277305
Phosphatidylcholine (18:0_20:5) levels	11	61770929	C	G	-1.16E-01	1.72E-02	4.44E-01	rs174527	11_61770929_G_C	0.98	7121	1.73E-11	GCST90277305
Phosphatidylcholine (18:0_20:5) levels	11	61790331	C	T	-4.20E-01	1.65E-02	4.10E-01	rs102275	11_61790331_T_C	1.00	7121	3.30E-137	GCST90277305
Phosphatidylcholine (18:0_20:5) levels	11	62451557	G	A	1.11E-01	1.97E-02	2.57E-01	rs7936002	11_62451557_A_G	0.93	7121	2.22E-08	GCST90277305
Phosphatidylcholine (18:0_20:5) levels	11	68908029	T	C	-1.41E-01	2.89E-02	9.80E-02	rs508049	11_68908029_C_T	0.96	7121	1.04E-06	GCST90277305
Phosphatidylcholine (18:0_20:5) levels	13	107595697	C	T	9.20E-02	2.01E-02	2.19E-01	rs9587408	13_107595697_T_C	1.00	7121	4.86E-06	GCST90277305
Phosphatidylcholine (18:0_20:5) levels	15	94356658	A	G	-7.65E-01	1.56E-01	3.53E-03	rs80116939	15_94356658_G_A	0.81	7121	1.00E-06	GCST90277305
Phosphatidylcholine (18:0_20:5) levels	16	10434179	G	A	1.23E-01	2.28E-02	1.65E-01	rs62025936	16_10434179_A_G	0.99	7121	6.27E-08	GCST90277305
Phosphatidylcholine (18:0_20:5) levels	16	20568904	C	T	2.76E-01	6.04E-02	2.01E-02	rs61653779	16_20568904_T_C	1.00	7121	5.09E-06	GCST90277305
Phosphatidylcholine (18:0_20:5) levels	18	71740925	A	T	7.54E-02	1.70E-02	5.81E-01	rs2135279	18_71740925_T_A	0.99	7121	9.45E-06	GCST90277305
Phosphatidylcholine (18:0_22:5) levels	1	94975736	T	C	-8.58E-02	1.81E-02	3.33E-01	rs2797620	1_94975736_C_T	1.00	6805	2.16E-06	GCST90277306
Phosphatidylcholine (18:0_22:5) levels	1	218198038	T	C	1.97E-01	3.90E-02	5.21E-02	rs34833702	1_218198038_C_T	0.98	6805	4.44E-07	GCST90277306
Phosphatidylcholine (18:0_22:5) levels	1	230188399	T	G	-9.72E-02	2.07E-02	2.14E-01	rs628035	1_230188399_G_T	1.00	6805	2.72E-06	GCST90277306
Phosphatidylcholine (18:0_22:5) levels	2	11621183	C	T	-2.27E-01	5.05E-02	9.68E-01	rs2304401	2_11621183_T_C	0.89	6805	7.43E-06	GCST90277306
Phosphatidylcholine (18:0_22:5) levels	2	27508073	C	T	-1.01E-01	1.79E-02	6.51E-01	rs1260326	2_27508073_T_C	1.00	6805	1.64E-08	GCST90277306
Phosphatidylcholine (18:0_22:5) levels	2	157711265	A	T	1.08E-01	2.14E-02	2.00E-01	rs1220131	2_157711265_T_A	1.00	6805	4.43E-07	GCST90277306
Phosphatidylcholine (18:0_22:5) levels	3	148282274	T	C	-2.03E-01	4.25E-02	4.86E-02	rs115247383	3_148282274_C_T	0.89	6805	1.84E-06	GCST90277306
Phosphatidylcholine (18:0_22:5) levels	4	30161140	T	G	8.29E-02	1.82E-02	3.73E-01	rs2613167	4_30161140_G_T	0.97	6805	5.19E-06	GCST90277306
Phosphatidylcholine (18:0_22:5) levels	5	52103978	C	T	7.84E-02	1.77E-02	3.78E-01	rs13182955	5_52103978_T_C	1.00	6805	9.35E-06	GCST90277306
Phosphatidylcholine (18:0_22:5) levels	5	170155391	G	A	7.90E-02	1.71E-02	5.06E-01	rs1465411	5_170155391_A_G	1.00	6805	3.70E-06	GCST90277306
Phosphatidylcholine (18:0_22:5) levels	6	10999417	C	T	8.18E-02	1.73E-02	4.58E-01	rs9918362	6_10999417_T_C	1.00	6805	2.24E-06	GCST90277306
Phosphatidylcholine (18:0_22:5) levels	6	110673493	C	T	-1.02E-01	1.94E-02	2.72E-01	rs9374192	6_110673493_T_C	1.00	6805	1.69E-07	GCST90277306
Phosphatidylcholine (18:0_22:5) levels	7	54771130	C	T	-3.29E-01	6.91E-02	1.59E-02	rs183410044	7_54771130_T_C	0.98	6805	1.90E-06	GCST90277306
Phosphatidylcholine (18:0_22:5) levels	7	136096416	C	G	-2.86E-01	6.34E-02	2.06E-02	rs143380352	7_136096416_G_C	0.89	6805	6.34E-06	GCST90277306
Phosphatidylcholine (18:0_22:5) levels	8	9326154	G	A	1.21E-01	2.40E-02	8.52E-01	rs4841133	8_9326154_A_G	1.00	6805	5.19E-07	GCST90277306

Phosphatidylcholine (18:0_22:5) levels	8	69019004	T	C	2.31E-01	4.94E-02	3.03E-02	rs113847854	8_69019004_C_T	0.98	6805	2.85E-06	GCST90277306
Phosphatidylcholine (18:0_22:5) levels	8	93993037	C	T	1.53E-01	3.46E-02	6.89E-02	rs58967664	8_93993037_T_C	0.94	6805	9.11E-06	GCST90277306
Phosphatidylcholine (18:0_22:5) levels	9	33767894	A	G	-7.89E-02	1.75E-02	3.83E-01	rs34415160	9_33767894_G_A	1.00	6805	6.71E-06	GCST90277306
Phosphatidylcholine (18:0_22:5) levels	9	136677616	G	C	-8.85E-02	1.83E-02	6.47E-01	rs2236514	9_136677616_C_G	0.95	6805	1.28E-06	GCST90277306
Phosphatidylcholine (18:0_22:5) levels	11	61770929	C	G	-1.00E-01	1.75E-02	4.44E-01	rs174527	11_61770929_G_C	0.98	6805	1.07E-08	GCST90277306
Phosphatidylcholine (18:0_22:5) levels	11	61790331	C	T	-2.85E-01	1.71E-02	4.10E-01	rs102275	11_61790331_T_C	1.00	6805	5.38E-61	GCST90277306
Phosphatidylcholine (18:0_22:5) levels	11	88482589	T	A	-1.27E-01	2.87E-02	9.83E-02	rs41352345	11_88482589_A_T	1.00	6805	9.19E-06	GCST90277306
Phosphatidylcholine (18:0_22:5) levels	12	70541856	G	A	-7.71E-02	1.71E-02	4.72E-01	rs11178277	12_70541856_A_G	1.00	6805	6.47E-06	GCST90277306
Phosphatidylcholine (18:0_22:5) levels	12	93538379	T	G	1.00E-01	2.10E-02	2.15E-01	rs7968201	12_93538379_G_T	0.99	6805	1.98E-06	GCST90277306
Phosphatidylcholine (18:0_22:5) levels	12	120978819	G	C	1.08E-01	1.72E-02	4.70E-01	rs1169289	12_120978819_C_G	0.99	6805	3.73E-10	GCST90277306
Phosphatidylcholine (18:0_22:5) levels	14	100895541	T	C	-9.56E-02	2.15E-02	2.23E-01	rs7146460	14_100895541_C_T	0.92	6805	8.96E-06	GCST90277306
Phosphatidylcholine (18:0_22:5) levels	15	70670258	C	A	-2.45E-01	5.53E-02	2.40E-02	rs55982271	15_70670258_A_C	0.98	6805	9.21E-06	GCST90277306
Phosphatidylcholine (18:0_22:5) levels	17	18192664	A	G	-1.05E-01	2.19E-02	8.14E-01	rs8068517	17_18192664_G_A	1.00	6805	1.74E-06	GCST90277306
Phosphatidylcholine (18:0_22:5) levels	18	52743009	C	T	-4.62E-01	9.18E-02	9.35E-03	rs147797702	18_52743009_T_C	0.96	6805	4.97E-07	GCST90277306
Phosphatidylcholine (18:0_22:5) levels	18	54154713	T	C	4.92E-01	1.02E-01	7.43E-03	rs117135394	18_54154713_C_T	0.93	6805	1.45E-06	GCST90277306
Phosphatidylcholine (18:0_22:6) levels	1	53711606	C	T	9.84E-02	2.21E-02	1.78E-01	rs7539908	1_53711606_T_C	1.00	7004	8.56E-06	GCST90277307
Phosphatidylcholine (18:0_22:6) levels	1	222093891	T	C	8.45E-02	1.77E-02	3.47E-01	rs7542307	1_222093891_C_T	1.00	7004	1.96E-06	GCST90277307
Phosphatidylcholine (18:0_22:6) levels	3	124802671	T	G	1.26E-01	2.71E-02	1.12E-01	rs2619330	3_124802671_G_T	0.99	7004	3.26E-06	GCST90277307
Phosphatidylcholine (18:0_22:6) levels	3	176189554	C	T	-8.96E-02	1.87E-02	7.07E-01	rs2067613	3_176189554_T_C	1.00	7004	1.67E-06	GCST90277307
Phosphatidylcholine (18:0_22:6) levels	4	147476374	A	G	-4.72E-01	1.00E-01	7.98E-03	rs62345669	4_147476374_G_A	0.89	7004	2.36E-06	GCST90277307
Phosphatidylcholine (18:0_22:6) levels	5	38486310	T	G	9.49E-02	2.08E-02	2.07E-01	rs3110235	5_38486310_G_T	0.99	7004	5.40E-06	GCST90277307
Phosphatidylcholine (18:0_22:6) levels	5	134851174	T	C	-2.44E-01	4.99E-02	2.91E-02	rs139606388	5_134851174_C_T	0.99	7004	1.04E-06	GCST90277307
Phosphatidylcholine (18:0_22:6) levels	6	42963486	A	G	1.02E-01	1.73E-02	3.93E-01	rs4987173	6_42963486_G_A	1.00	7004	3.48E-09	GCST90277307
Phosphatidylcholine (18:0_22:6) levels	7	155293878	T	C	-2.50E-01	5.54E-02	2.48E-02	rs9719746	7_155293878_C_T	0.96	7004	6.55E-06	GCST90277307
Phosphatidylcholine (18:0_22:6) levels	8	6096074	T	G	-1.92E-01	4.23E-02	4.20E-02	rs10503355	8_6096074_G_T	0.99	7004	5.43E-06	GCST90277307
Phosphatidylcholine (18:0_22:6) levels	8	9326154	G	A	1.06E-01	2.38E-02	8.52E-01	rs4841133	8_9326154_A_G	1.00	7004	8.17E-06	GCST90277307
Phosphatidylcholine (18:0_22:6) levels	8	64610547	T	C	1.01E-01	2.24E-02	8.28E-01	rs3779874	8_64610547_C_T	1.00	7004	6.12E-06	GCST90277307
Phosphatidylcholine (18:0_22:6) levels	9	874977	G	T	-1.33E-01	2.77E-02	1.03E-01	rs11792256	9_874977_T_G	0.99	7004	1.73E-06	GCST90277307
Phosphatidylcholine (18:0_22:6) levels	11	42654689	C	T	7.87E-02	1.71E-02	4.15E-01	rs61900787	11_42654689_T_C	1.00	7004	4.34E-06	GCST90277307
Phosphatidylcholine (18:0_22:6) levels	11	61779120	G	A	-1.67E-01	1.71E-02	4.14E-01	rs174530	11_61779120_A_G	1.00	7004	2.21E-22	GCST90277307
Phosphatidylcholine (18:0_22:6) levels	12	21263479	G	A	-8.04E-02	1.79E-02	6.47E-01	rs11045913	12_21263479_A_G	0.99	7004	7.32E-06	GCST90277307
Phosphatidylcholine (18:0_22:6) levels	12	46987694	A	G	-1.77E-01	3.84E-02	4.99E-02	rs143578075	12_46987694_G_A	1.00	7004	3.92E-06	GCST90277307
Phosphatidylcholine (18:0_22:6) levels	12	59019021	A	G	-1.16E-01	2.47E-02	1.37E-01	rs7974440	12_59019021_G_A	1.00	7004	2.57E-06	GCST90277307
Phosphatidylcholine (18:0_22:6) levels	14	72571809	T	C	-1.86E-01	3.88E-02	5.30E-02	rs10150075	14_72571809_C_T	0.95	7004	1.56E-06	GCST90277307
Phosphatidylcholine (18:0_22:6) levels	15	62205752	A	T	-8.28E-02	1.86E-02	2.96E-01	rs12050889	15_62205752_T_A	0.99	7004	8.75E-06	GCST90277307
Phosphatidylcholine (18:0_22:6) levels	15	101196347	T	G	1.89E-01	4.15E-02	4.49E-02	rs117061362	15_101196347_G_T	0.93	7004	5.13E-06	GCST90277307
Phosphatidylcholine (18:0_22:6) levels	17	18166314	T	G	1.11E-01	2.18E-02	1.81E-01	rs72827432	17_18166314_G_T	1.00	7004	3.38E-07	GCST90277307
Phosphatidylcholine (18:0_22:6) levels	20	43445166	G	C	-1.48E-01	3.27E-02	7.47E-02	rs6030870	20_43445166_C_G	0.98	7004	5.83E-06	GCST90277307
Phosphatidylcholine (18:0_22:6) levels	20	56297803	T	C	8.59E-02	1.90E-02	7.21E-01	rs2426606	20_56297803_C_T	1.00	7004	6.52E-06	GCST90277307
Phosphatidylcholine (18:1_18:1) levels	1	62633352	C	T	-1.12E-01	1.88E-02	2.62E-01	rs10889352	1_62633352_T_C	1.00	7174	2.61E-09	GCST90277308
Phosphatidylcholine (18:1_18:1) levels	1	111314654	C	T	-9.29E-02	2.01E-02	2.20E-01	rs41391244	1_111314654_T_C	1.00	7174	3.80E-06	GCST90277308
Phosphatidylcholine (18:1_18:1) levels	1	230167404	T	C	9.20E-02	2.00E-02	7.77E-01	rs10779836	1_230167404_C_T	1.00	7174	4.12E-06	GCST90277308
Phosphatidylcholine (18:1_18:1) levels	2	117641775	T	C	1.75E-01	3.84E-02	5.02E-02	rs4848460	2_117641775_C_T	0.96	7174	5.05E-06	GCST90277308
Phosphatidylcholine (18:1_18:1) levels	2	204579749	T	C	-2.27E-01	4.94E-02	3.15E-02	rs56057593	2_204579749_C_T	0.93	7174	4.30E-06	GCST90277308
Phosphatidylcholine (18:1_18:1) levels	4	29977517	T	C	5.82E-01	1.28E-01	4.67E-03	rs140268092	4_29977517_C_T	0.89	7174	5.51E-06	GCST90277308
Phosphatidylcholine (18:1_18:1) levels	4	38036537	T	C	-2.18E-01	4.57E-02	3.38E-02	rs73241068	4_38036537_C_T	1.00	7174	1.85E-06	GCST90277308
Phosphatidylcholine (18:1_18:1) levels	4	140097358	T	C	2.09E-01	4.59E-02	3.94E-02	rs79008894	4_140097358_C_T	0.86	7174	5.56E-06	GCST90277308
Phosphatidylcholine (18:1_18:1) levels	5	86717236	T	C	-3.94E-01	8.53E-02	9.82E-03	rs41441450	5_86717236_C_T	0.99	7174	3.92E-06	GCST90277308
Phosphatidylcholine (18:1_18:1) levels	5	151601186	C	G	-2.91E-01	6.55E-02	1.76E-02	rs113277188	5_151601186_G_C	0.93	7174	9.15E-06	GCST90277308
Phosphatidylcholine (18:1_18:1) levels	6	18685527	C	T	3.70E-01	6.89E-02	1.52E-02	rs144512980	6_18685527_T_C	0.99	7174	8.17E-08	GCST90277308
Phosphatidylcholine (18:1_18:1) levels	8	9324101	A	G	1.12E-01	2.34E-02	8.50E-01	rs2126263	8_9324101_G_A	1.00	7174	1.93E-06	GCST90277308
Phosphatidylcholine (18:1_18:1) levels	8	22702678	T	C	-7.67E-02	1.71E-02	4.30E-01	rs13278111	8_22702678_C_T	0.98	7174	7.00E-06	GCST90277308
Phosphatidylcholine (18:1_18:1) levels	11	61832870	C	A	-2.02E-01	1.69E-02	5.91E-01	rs174574	11_61832870_A_C	1.00	7174	1.28E-32	GCST90277308
Phosphatidylcholine (18:1_18:1) levels	11	116786845	T	C	-8.94E-02	1.96E-02	7.65E-01	rs3741298	11_116786845_C_T	1.00	7174	5.19E-06	GCST90277308
Phosphatidylcholine (18:1_18:1) levels	12	112553648	T	C	-1.25E-01	2.79E-02	1.00E-01	rs10850057	12_112553648_C_T	0.99	7174	8.27E-06	GCST90277308
Phosphatidylcholine (18:1_18:1) levels	12	121021637	G	C	-7.59E-02	1.68E-02	4.38E-01	rs7300883	12_121021637_C_G	1.00	7174	6.70E-06	GCST90277308
Phosphatidylcholine (18:1_18:1) levels	14	68688212	G	A	-2.67E-01	5.66E-02	9.76E-01	rs10145681	14_68688212_A_G	0.95	7174	2.32E-06	GCST90277308

Phosphatidylcholine (18:1_18:1) levels	15	58391167	G	A	-1.10E-01	1.68E-02	5.73E-01	rs1532085	15_58391167_A_G	1.00	7174	7.42E-11	GCST90277308
Phosphatidylcholine (18:1_18:1) levels	15	58431280	C	T	9.25E-02	1.92E-02	2.66E-01	rs1077834	15_58431280_T_C	0.98	7174	1.56E-06	GCST90277308
Phosphatidylcholine (18:1_18:1) levels	16	56960616	T	C	1.09E-01	1.86E-02	2.78E-01	rs17231506	16_56960616_C_T	1.00	7174	4.04E-09	GCST90277308
Phosphatidylcholine (18:1_18:1) levels	18	49695288	A	T	1.22E-01	2.71E-02	8.94E-01	rs2000827	18_49695288_T_A	0.97	7174	7.27E-06	GCST90277308
Phosphatidylcholine (18:1_18:1) levels	22	46945349	G	C	8.90E-02	1.89E-02	2.63E-01	rs136061	22_46945349_C_G	0.99	7174	2.41E-06	GCST90277308
Phosphatidylcholine (18:1_18:2) levels	1	62633352	C	T	-1.26E-01	1.88E-02	2.62E-01	rs10889352	1_62633352_T_C	1.00	7174	1.75E-11	GCST90277309
Phosphatidylcholine (18:1_18:2) levels	2	57690087	T	C	-8.87E-02	1.89E-02	2.65E-01	rs9789400	2_57690087_C_T	0.99	7174	2.74E-06	GCST90277309
Phosphatidylcholine (18:1_18:2) levels	4	145707221	A	G	-1.53E-01	3.40E-02	6.66E-02	rs28844909	4_145707221_G_A	0.95	7174	7.07E-06	GCST90277309
Phosphatidylcholine (18:1_18:2) levels	4	169311729	A	G	-1.01E-01	2.11E-02	1.96E-01	rs13128807	4_169311729_G_A	1.00	7174	1.81E-06	GCST90277309
Phosphatidylcholine (18:1_18:2) levels	5	96128576	T	C	2.43E-01	5.44E-02	2.51E-02	rs78882831	5_96128576_C_T	0.97	7174	8.43E-06	GCST90277309
Phosphatidylcholine (18:1_18:2) levels	6	18587903	T	C	5.80E-01	1.16E-01	5.39E-03	rs142689129	6_18587903_C_T	0.98	7174	6.42E-07	GCST90277309
Phosphatidylcholine (18:1_18:2) levels	6	29796264	T	C	4.57E-01	1.03E-01	7.34E-03	rs11757990	6_29796264_C_T	0.97	7174	9.55E-06	GCST90277309
Phosphatidylcholine (18:1_18:2) levels	7	5810475	C	G	-1.81E-01	3.92E-02	4.96E-02	rs147761802	7_5810475_G_C	0.95	7174	4.04E-06	GCST90277309
Phosphatidylcholine (18:1_18:2) levels	9	14382621	C	T	9.06E-02	1.75E-02	3.50E-01	rs7036554	9_14382621_T_C	0.97	7174	2.43E-07	GCST90277309
Phosphatidylcholine (18:1_18:2) levels	11	61770929	C	G	9.06E-02	1.71E-02	4.44E-01	rs174527	11_61770929_G_C	0.98	7174	1.11E-07	GCST90277309
Phosphatidylcholine (18:1_18:2) levels	11	61832870	C	A	-3.80E-01	1.64E-02	5.91E-01	rs174574	11_61832870_A_C	1.00	7174	2.79E-114	GCST90277309
Phosphatidylcholine (18:1_18:2) levels	11	62211023	G	A	-9.96E-02	2.09E-02	1.96E-01	rs118108980	11_62211023_A_G	1.00	7174	1.93E-06	GCST90277309
Phosphatidylcholine (18:1_18:2) levels	11	72735137	T	C	1.09E-01	2.43E-02	1.35E-01	rs4944732	11_72735137_C_T	0.99	7174	8.27E-06	GCST90277309
Phosphatidylcholine (18:1_18:2) levels	11	107869185	C	A	-1.07E-01	2.39E-02	8.59E-01	rs12798268	11_107869185_A_C	1.00	7174	7.19E-06	GCST90277309
Phosphatidylcholine (18:1_18:2) levels	11	116840252	G	A	-9.11E-02	1.73E-02	6.19E-01	rs613808	11_116840252_A_G	1.00	7174	1.43E-07	GCST90277309
Phosphatidylcholine (18:1_18:2) levels	11	126095437	C	T	-1.68E-01	3.66E-02	5.50E-02	rs564216	11_126095437_T_C	0.98	7174	4.51E-06	GCST90277309
Phosphatidylcholine (18:1_18:2) levels	15	58379566	T	G	-1.06E-01	1.70E-02	6.11E-01	rs1601935	15_58379566_G_T	0.99	7174	5.31E-10	GCST90277309
Phosphatidylcholine (18:1_18:2) levels	15	58431227	G	A	1.25E-01	1.91E-02	2.66E-01	rs1077835	15_58431227_A_G	0.98	7174	7.94E-11	GCST90277309
Phosphatidylcholine (18:1_18:2) levels	15	59198971	A	G	-1.31E-01	2.96E-02	9.13E-01	rs62004153	15_59198971_G_A	1.00	7174	9.20E-06	GCST90277309
Phosphatidylcholine (18:1_18:2) levels	16	15036737	G	A	7.93E-02	1.77E-02	3.34E-01	rs6498540	16_15036737_A_G	0.99	7174	7.68E-06	GCST90277309
Phosphatidylcholine (18:1_18:2) levels	16	56960616	T	C	1.20E-01	1.85E-02	2.78E-01	rs17231506	16_56960616_C_T	1.00	7174	1.06E-10	GCST90277309
Phosphatidylcholine (18:1_18:2) levels	18	13715335	G	A	-8.19E-02	1.75E-02	3.25E-01	rs948727	18_13715335_A_G	1.00	7174	2.78E-06	GCST90277309
Phosphatidylcholine (18:1_18:2) levels	20	55260213	C	T	-3.33E-01	7.21E-02	1.43E-02	rs6023985	20_55260213_T_C	0.91	7174	4.10E-06	GCST90277309
Phosphatidylcholine (18:1_18:2) levels	21	37563977	C	G	8.39E-02	1.67E-02	4.32E-01	rs2236605	21_37563977_G_C	0.99	7174	5.14E-07	GCST90277309
Phosphatidylcholine (18:1_18:3) levels	1	98597981	T	C	2.85E-01	6.22E-02	2.34E-02	rs114797690	1_98597981_C_T	0.87	6339	4.84E-06	GCST90277310
Phosphatidylcholine (18:1_18:3) levels	2	41556996	C	G	1.95E-01	4.29E-02	4.61E-02	rs149851240	2_41556996_G_C	0.98	6339	5.55E-06	GCST90277310
Phosphatidylcholine (18:1_18:3) levels	3	100870486	A	C	-9.25E-02	1.97E-02	2.72E-01	rs1116931	3_100870486_C_A	1.00	6339	2.78E-06	GCST90277310
Phosphatidylcholine (18:1_18:3) levels	4	5640434	G	A	8.80E-02	1.95E-02	6.45E-01	rs751841	4_5640434_A_G	0.90	6339	6.49E-06	GCST90277310
Phosphatidylcholine (18:1_18:3) levels	5	139952900	T	C	1.44E-01	3.25E-02	8.36E-02	rs17208010	5_139952900_C_T	0.96	6339	8.83E-06	GCST90277310
Phosphatidylcholine (18:1_18:3) levels	6	6411842	C	G	-8.38E-02	1.87E-02	6.64E-01	rs2774509	6_6411842_G_C	1.00	6339	7.22E-06	GCST90277310
Phosphatidylcholine (18:1_18:3) levels	6	97082368	G	C	-3.99E-01	8.21E-02	1.20E-02	rs144128487	6_97082368_C_G	0.97	6339	1.23E-06	GCST90277310
Phosphatidylcholine (18:1_18:3) levels	6	156378387	T	A	-2.04E-01	4.09E-02	4.97E-02	rs9480321	6_156378387_A_T	0.97	6339	6.16E-07	GCST90277310
Phosphatidylcholine (18:1_18:3) levels	11	5586584	A	G	8.83E-02	1.88E-02	3.37E-01	rs10769099	11_5586584_G_A	0.99	6339	2.73E-06	GCST90277310
Phosphatidylcholine (18:1_18:3) levels	11	61855668	T	C	8.01E-02	1.80E-02	4.19E-01	rs174601	11_61855668_C_T	1.00	6339	8.29E-06	GCST90277310
Phosphatidylcholine (18:1_18:3) levels	11	80166090	T	A	-4.18E-01	9.46E-02	8.91E-03	rs116910056	11_80166090_A_T	0.99	6339	9.84E-06	GCST90277310
Phosphatidylcholine (18:1_18:3) levels	12	96120840	A	G	-1.53E-01	3.43E-02	7.87E-02	rs148879093	12_96120840_G_A	0.91	6339	7.84E-06	GCST90277310
Phosphatidylcholine (18:1_18:3) levels	13	110209642	C	T	4.26E-01	9.55E-02	8.58E-03	rs9559745	13_110209642_T_C	0.95	6339	8.58E-06	GCST90277310
Phosphatidylcholine (18:1_18:3) levels	15	58416983	A	G	8.06E-02	1.79E-02	4.93E-01	rs422137	15_58416983_G_A	0.98	6339	6.73E-06	GCST90277310
Phosphatidylcholine (18:1_18:3) levels	15	58432643	A	G	9.06E-02	1.92E-02	3.37E-01	rs8033940	15_58432643_G_A	0.99	6339	2.32E-06	GCST90277310
Phosphatidylcholine (18:1_18:3) levels	16	56960616	T	C	8.90E-02	1.98E-02	2.78E-01	rs17231506	16_56960616_C_T	1.00	6339	6.76E-06	GCST90277310
Phosphatidylcholine (18:1_18:3) levels	16	75302415	A	G	-4.77E-01	1.03E-01	7.68E-03	rs140885981	16_75302415_G_A	0.89	6339	3.89E-06	GCST90277310
Phosphatidylcholine (18:1_18:3) levels	19	2561737	C	T	1.03E-01	2.33E-02	8.20E-01	rs2965212	19_2561737_T_C	0.99	6339	9.04E-06	GCST90277310
Phosphatidylcholine (18:1_20:2) levels	1	103864588	A	G	-3.73E-01	8.15E-02	1.38E-02	rs114139553	1_103864588_G_A	0.96	5852	4.63E-06	GCST90277311
Phosphatidylcholine (18:1_20:2) levels	1	217457286	T	C	1.78E-01	3.65E-02	7.37E-02	rs265114	1_217457286_C_T	0.96	5852	1.06E-06	GCST90277311
Phosphatidylcholine (18:1_20:2) levels	5	17302034	T	A	3.01E-01	6.68E-02	2.02E-02	rs17543793	5_17302034_A_T	0.94	5852	6.64E-06	GCST90277311
Phosphatidylcholine (18:1_20:2) levels	6	154419744	C	T	-1.39E-01	3.08E-02	9.71E-02	rs9384216	6_154419744_T_C	1.00	5852	7.05E-06	GCST90277311
Phosphatidylcholine (18:1_20:2) levels	7	6332316	G	A	1.02E-01	2.27E-02	2.05E-01	rs6944011	7_6332316_A_G	0.97	5852	7.50E-06	GCST90277311
Phosphatidylcholine (18:1_20:2) levels	7	10826150	A	G	-2.47E-01	5.35E-02	3.08E-02	rs80201535	7_10826150_G_A	0.97	5852	4.14E-06	GCST90277311
Phosphatidylcholine (18:1_20:2) levels	7	106989575	A	G	2.14E-01	4.69E-02	4.16E-02	rs116922907	7_106989575_G_A	0.98	5852	5.03E-06	GCST90277311
Phosphatidylcholine (18:1_20:2) levels	7	155435171	T	G	-2.75E-01	6.21E-02	2.51E-02	rs12113068	7_155435171_G_T	0.92	5852	9.34E-06	GCST90277311
Phosphatidylcholine (18:1_20:2) levels	8	9320758	T	A	1.22E-01	2.51E-02	8.37E-01	rs6984305	8_9320758_A_T	1.00	5852	1.30E-06	GCST90277311
Phosphatidylcholine (18:1_20:2) levels	9	78442148	C	T	-1.16E-01	2.14E-02	2.45E-01	rs7041283	9_78442148_T_C	1.00	5852	5.79E-08	GCST90277311

Phosphatidylcholine (18:1_20:2) levels	11	36094558	T	C	1.30E-01	2.78E-02	1.48E-01	rs35195855	11_36094558_C_T	0.87	5852	3.10E-06	GCST90277311
Phosphatidylcholine (18:1_20:2) levels	11	61770929	C	G	9.38E-02	1.89E-02	4.44E-01	rs174527	11_61770929_G_C	0.98	5852	7.37E-07	GCST90277311
Phosphatidylcholine (18:1_20:2) levels	11	61839170	G	A	4.31E-01	1.79E-02	4.10E-01	rs174580	11_61839170_A_G	1.00	5852	1.49E-122	GCST90277311
Phosphatidylcholine (18:1_20:2) levels	11	62060922	A	G	1.90E-01	2.90E-02	1.15E-01	rs12417747	11_62060922_G_A	0.99	5852	5.83E-11	GCST90277311
Phosphatidylcholine (18:1_20:2) levels	11	63036470	T	C	-1.57E-01	3.40E-02	8.11E-02	rs61893829	11_63036470_C_T	0.97	5852	3.96E-06	GCST90277311
Phosphatidylcholine (18:1_20:2) levels	11	75734293	G	A	-1.30E-01	2.22E-02	2.27E-01	rs656095	11_75734293_A_G	1.00	5852	4.24E-09	GCST90277311
Phosphatidylcholine (18:1_20:2) levels	12	54160565	A	G	-5.39E-01	1.03E-01	8.80E-03	rs74089903	12_54160565_G_A	0.96	5852	1.57E-07	GCST90277311
Phosphatidylcholine (18:1_20:2) levels	12	124836915	G	T	1.06E-01	2.15E-02	2.35E-01	rs10846753	12_124836915_T_G	1.00	5852	8.21E-07	GCST90277311
Phosphatidylcholine (18:1_20:2) levels	15	58431476	T	C	1.13E-01	2.15E-02	2.58E-01	rs1800588	15_58431476_C_T	0.99	5852	1.58E-07	GCST90277311
Phosphatidylcholine (18:1_20:2) levels	15	70406039	G	A	6.39E-01	1.35E-01	4.85E-03	rs117534325	15_70406039_A_G	0.95	5852	2.47E-06	GCST90277311
Phosphatidylcholine (18:1_20:2) levels	15	95538680	C	T	8.40E-02	1.89E-02	5.96E-01	rs7176661	15_95538680_T_C	0.99	5852	8.55E-06	GCST90277311
Phosphatidylcholine (18:1_20:2) levels	16	56976320	T	C	1.82E-01	3.53E-02	7.77E-02	rs56208677	16_56976320_C_T	0.96	5852	2.63E-07	GCST90277311
Phosphatidylcholine (18:1_20:2) levels	18	68219549	A	G	-3.06E-01	6.67E-02	1.93E-02	rs118150981	18_68219549_G_A	0.97	5852	4.54E-06	GCST90277311
Phosphatidylcholine (18:1_20:2) levels	20	40441104	G	T	1.51E-01	3.03E-02	1.12E-01	rs73292027	20_40441104_T_G	0.93	5852	6.31E-07	GCST90277311
Phosphatidylcholine (18:1_20:3) levels	1	62494430	G	A	-9.79E-02	1.84E-02	2.85E-01	rs10889335	1_62494430_A_G	1.00	7171	1.06E-07	GCST90277312
Phosphatidylcholine (18:1_20:3) levels	1	94975736	T	C	-9.25E-02	1.76E-02	3.33E-01	rs2797620	1_94975736_C_T	1.00	7171	1.49E-07	GCST90277312
Phosphatidylcholine (18:1_20:3) levels	2	10043013	G	C	-4.97E-01	1.07E-01	6.23E-03	rs79047909	2_10043013_C_G	0.96	7171	3.20E-06	GCST90277312
Phosphatidylcholine (18:1_20:3) levels	3	22971838	G	A	8.48E-02	1.68E-02	4.16E-01	rs822798	3_22971838_A_G	1.00	7171	4.93E-07	GCST90277312
Phosphatidylcholine (18:1_20:3) levels	4	115193660	C	A	-4.92E-01	1.05E-01	6.71E-03	rs182828998	4_115193660_A_C	0.98	7171	2.52E-06	GCST90277312
Phosphatidylcholine (18:1_20:3) levels	4	172429853	G	A	1.78E-01	3.94E-02	5.21E-02	rs116647148	4_172429853_A_G	0.91	7171	6.14E-06	GCST90277312
Phosphatidylcholine (18:1_20:3) levels	6	18489429	C	G	5.13E-01	1.14E-01	5.56E-03	rs12198590	6_18489429_G_C	0.98	7171	7.34E-06	GCST90277312
Phosphatidylcholine (18:1_20:3) levels	6	123327335	G	A	1.36E-01	3.01E-02	8.22E-02	rs72974647	6_123327335_A_G	1.00	7171	6.80E-06	GCST90277312
Phosphatidylcholine (18:1_20:3) levels	6	138148100	T	G	2.97E-01	6.61E-02	1.70E-02	rs113366516	6_138148100_G_T	0.93	7171	7.07E-06	GCST90277312
Phosphatidylcholine (18:1_20:3) levels	8	59010827	G	A	-1.37E-01	2.93E-02	9.01E-02	rs6985657	8_59010827_A_G	0.99	7171	2.77E-06	GCST90277312
Phosphatidylcholine (18:1_20:3) levels	8	84222419	G	C	-9.50E-02	1.91E-02	2.58E-01	rs10504795	8_84222419_C_G	1.00	7171	6.32E-07	GCST90277312
Phosphatidylcholine (18:1_20:3) levels	10	5578436	T	C	1.33E-01	2.86E-02	9.03E-01	rs7897746	10_5578436_C_T	0.99	7171	3.44E-06	GCST90277312
Phosphatidylcholine (18:1_20:3) levels	11	61788567	C	A	2.24E-01	2.64E-02	1.13E-01	rs61896141	11_61788567_A_C	1.00	7171	3.04E-17	GCST90277312
Phosphatidylcholine (18:1_20:3) levels	11	75745535	T	A	-9.97E-02	1.96E-02	2.41E-01	rs695112	11_75745535_A_T	0.99	7171	3.92E-07	GCST90277312
Phosphatidylcholine (18:1_20:3) levels	11	119000408	G	A	2.60E-01	5.16E-02	2.77E-02	rs75926193	11_119000408_A_G	0.93	7171	4.98E-07	GCST90277312
Phosphatidylcholine (18:1_20:3) levels	12	120977490	A	G	-8.30E-02	1.68E-02	5.47E-01	rs7139079	12_120977490_G_A	1.00	7171	7.63E-07	GCST90277312
Phosphatidylcholine (18:1_20:3) levels	14	95434157	C	T	1.94E-01	4.11E-02	4.42E-02	rs17092227	14_95434157_T_C	0.97	7171	2.53E-06	GCST90277312
Phosphatidylcholine (18:1_20:3) levels	15	58368884	C	T	1.73E-01	3.88E-02	4.79E-02	rs59739041	15_58368884_T_C	0.99	7171	8.86E-06	GCST90277312
Phosphatidylcholine (18:1_20:3) levels	15	58432643	A	G	9.52E-02	1.81E-02	3.37E-01	rs8033940	15_58432643_G_A	0.99	7171	1.38E-07	GCST90277312
Phosphatidylcholine (18:1_20:3) levels	16	15036737	G	A	-1.55E-01	1.77E-02	3.34E-01	rs6498540	16_15036737_A_G	0.99	7171	3.06E-18	GCST90277312
Phosphatidylcholine (18:1_20:3) levels	16	15773795	G	T	8.14E-02	1.84E-02	2.96E-01	rs56374730	16_15773795_T_G	0.99	7171	9.83E-06	GCST90277312
Phosphatidylcholine (18:1_20:3) levels	19	19117327	A	G	-9.35E-02	1.95E-02	7.53E-01	rs10423742	19_19117327_G_A	0.98	7171	1.71E-06	GCST90277312
Phosphatidylcholine (18:1_20:3) levels	19	19210016	G	C	-2.47E-01	3.86E-02	5.22E-02	rs150057262	19_19210016_C_G	0.94	7171	1.82E-10	GCST90277312
Phosphatidylcholine (18:1_20:3) levels	19	54173068	C	T	-9.22E-02	1.71E-02	5.93E-01	rs641738	19_54173068_T_C	0.97	7171	6.97E-08	GCST90277312
Phosphatidylcholine (18:1_20:3) levels	20	25397682	G	A	-7.93E-02	1.69E-02	5.36E-01	rs6083838	20_25397682_A_G	1.00	7171	2.60E-06	GCST90277312
Phosphatidylcholine (18:1_20:4) levels	1	62652525	C	A	-8.53E-02	1.88E-02	2.62E-01	rs10889353	1_62652525_A_C	1.00	7173	5.95E-06	GCST90277313
Phosphatidylcholine (18:1_20:4) levels	1	230143835	G	A	9.42E-02	1.96E-02	7.68E-01	rs4846905	1_230143835_A_G	1.00	7173	1.50E-06	GCST90277313
Phosphatidylcholine (18:1_20:4) levels	2	101570369	C	T	9.95E-02	2.01E-02	2.29E-01	rs79113397	2_101570369_T_C	0.99	7173	7.59E-07	GCST90277313
Phosphatidylcholine (18:1_20:4) levels	2	141078979	A	T	1.56E-01	3.50E-02	9.29E-01	rs1917701	2_141078979_T_A	0.83	7173	7.78E-06	GCST90277313
Phosphatidylcholine (18:1_20:4) levels	4	115324589	G	A	-4.58E-01	1.02E-01	7.09E-03	rs76582138	4_115324589_A_G	0.97	7173	6.56E-06	GCST90277313
Phosphatidylcholine (18:1_20:4) levels	4	140028398	C	T	-8.07E-02	1.68E-02	5.41E-01	rs6850591	4_140028398_T_C	1.00	7173	1.64E-06	GCST90277313
Phosphatidylcholine (18:1_20:4) levels	6	2822782	C	T	-4.25E-01	9.40E-02	8.67E-03	rs116814948	6_2822782_T_C	0.90	7173	6.20E-06	GCST90277313
Phosphatidylcholine (18:1_20:4) levels	7	39265042	G	C	-7.61E-02	1.71E-02	6.34E-01	rs4628166	7_39265042_C_G	1.00	7173	9.16E-06	GCST90277313
Phosphatidylcholine (18:1_20:4) levels	11	61490996	C	T	-1.30E-01	2.04E-02	7.83E-01	rs2943813	11_61490996_T_C	1.00	7173	2.26E-10	GCST90277313
Phosphatidylcholine (18:1_20:4) levels	11	61770929	C	G	-1.60E-01	1.70E-02	4.44E-01	rs174527	11_61770929_G_C	0.98	7173	5.01E-21	GCST90277313
Phosphatidylcholine (18:1_20:4) levels	11	61785208	T	G	-5.08E-01	1.60E-02	4.08E-01	rs174537	11_61785208_G_T	1.00	7173	5.48E-207	GCST90277313
Phosphatidylcholine (18:1_20:4) levels	11	62314821	G	T	1.87E-01	2.97E-02	8.73E-02	rs113394924	11_62314821_T_G	0.99	7173	3.52E-10	GCST90277313
Phosphatidylcholine (18:1_20:4) levels	11	62435462	G	A	-9.18E-02	1.88E-02	3.02E-01	rs3018617	11_62435462_A_G	0.94	7173	1.08E-06	GCST90277313
Phosphatidylcholine (18:1_20:4) levels	11	75723045	A	T	-7.72E-02	1.69E-02	4.85E-01	rs10793123	11_75723045_T_A	0.97	7173	5.11E-06	GCST90277313
Phosphatidylcholine (18:1_20:4) levels	11	75745089	T	C	-1.14E-01	1.98E-02	2.32E-01	rs531117	11_75745089_C_T	0.99	7173	9.96E-09	GCST90277313
Phosphatidylcholine (18:1_20:4) levels	12	76255614	T	C	-2.13E-01	4.81E-02	3.23E-02	rs113913626	12_76255614_C_T	0.94	7173	9.99E-06	GCST90277313
Phosphatidylcholine (18:1_20:4) levels	13	31500828	G	A	1.84E-01	4.04E-02	4.56E-02	rs73176681	13_31500828_A_G	0.98	7173	5.15E-06	GCST90277313
Phosphatidylcholine (18:1_20:4) levels	14	79220260	C	T	-2.61E-01	5.38E-02	2.62E-02	rs75976069	14_79220260_T_C	0.94	7173	1.31E-06	GCST90277313

Phosphatidylcholine (18:1_20:4) levels	14	94523321	T	C	-9.81E-02	1.86E-02	2.82E-01	rs61978272	14_94523321_C_T	0.98	7173	1.41E-07	GCST90277313
Phosphatidylcholine (18:1_20:4) levels	15	58431280	C	T	9.86E-02	1.92E-02	2.66E-01	rs1077834	15_58431280_T_C	0.98	7173	2.88E-07	GCST90277313
Phosphatidylcholine (18:1_20:4) levels	16	1939264	T	G	2.32E-01	4.54E-02	3.59E-02	rs147549994	16_1939264_G_T	0.95	7173	3.16E-07	GCST90277313
Phosphatidylcholine (18:1_20:4) levels	16	56954132	T	C	8.49E-02	1.84E-02	2.83E-01	rs173539	16_56954132_C_T	1.00	7173	4.26E-06	GCST90277313
Phosphatidylcholine (18:1_20:4) levels	16	78573129	CA	C	2.15E-01	4.85E-02	3.00E-02	rs113049169	16_78573129_C_CA	0.99	7173	9.26E-06	GCST90277313
Phosphatidylcholine (18:1_20:4) levels	18	52743009	C	T	-4.89E-01	8.90E-02	9.35E-03	rs147797702	18_52743009_T_C	0.96	7173	4.03E-08	GCST90277313
Phosphatidylcholine (18:1_20:4) levels	19	7552489	C	G	-1.02E-01	2.18E-02	8.18E-01	rs793864	19_7552489_G_C	0.97	7173	2.95E-06	GCST90277313
Phosphatidylcholine (18:1_20:4) levels	19	19269704	G	A	-2.76E-01	3.77E-02	5.35E-02	rs187429064	19_19269704_A_G	0.95	7173	2.68E-13	GCST90277313
Phosphatidylcholine (18:1_20:4) levels	19	19277691	T	A	-1.91E-01	3.44E-02	6.25E-02	rs8107974	19_19277691_A_T	1.00	7173	3.00E-08	GCST90277313
Phosphatidylcholine (18:1_20:4) levels	19	54173068	C	T	-8.52E-02	1.70E-02	5.93E-01	rs641738	19_54173068_T_C	0.97	7173	5.94E-07	GCST90277313
Phosphatidylcholine (18:1_20:4) levels	20	25397682	G	A	-7.45E-02	1.68E-02	5.36E-01	rs6083838	20_25397682_A_G	1.00	7173	9.53E-06	GCST90277313
Phosphatidylcholine (18:1_20:4) levels	20	36497804	A	G	-1.30E-01	2.87E-02	9.33E-02	rs73618588	20_36497804_G_A	0.99	7173	6.01E-06	GCST90277313
Phosphatidylcholine (18:2_18:2) levels	1	62670407	A	C	9.17E-02	1.89E-02	7.37E-01	rs1168127	1_62670407_C_A	1.00	7174	1.17E-06	GCST90277314
Phosphatidylcholine (18:2_18:2) levels	2	204695970	G	A	1.57E-01	3.28E-02	7.72E-02	rs148498852	2_204695970_A_G	0.93	7174	1.73E-06	GCST90277314
Phosphatidylcholine (18:2_18:2) levels	2	227805415	G	A	-2.97E-01	6.28E-02	1.96E-02	rs59256568	2_227805415_A_G	0.91	7174	2.34E-06	GCST90277314
Phosphatidylcholine (18:2_18:2) levels	3	10450834	T	C	8.64E-02	1.79E-02	3.20E-01	rs62238391	3_10450834_C_T	0.99	7174	1.38E-06	GCST90277314
Phosphatidylcholine (18:2_18:2) levels	3	80053426	C	A	-8.19E-02	1.76E-02	3.28E-01	rs13078306	3_80053426_A_C	1.00	7174	3.51E-06	GCST90277314
Phosphatidylcholine (18:2_18:2) levels	3	95414411	A	G	2.96E-01	6.38E-02	1.75E-02	rs56158036	3_95414411_G_A	1.00	7174	3.58E-06	GCST90277314
Phosphatidylcholine (18:2_18:2) levels	4	159948553	G	A	7.96E-02	1.68E-02	5.67E-01	rs4271965	4_159948553_A_G	1.00	7174	2.16E-06	GCST90277314
Phosphatidylcholine (18:2_18:2) levels	4	169277659	A	G	-1.12E-01	2.50E-02	1.30E-01	rs13150924	4_169277659_G_A	0.98	7174	8.25E-06	GCST90277314
Phosphatidylcholine (18:2_18:2) levels	4	185838932	T	G	4.22E-01	8.96E-02	1.00E-02	rs139598970	4_185838932_G_T	0.89	7174	2.54E-06	GCST90277314
Phosphatidylcholine (18:2_18:2) levels	5	52415905	G	A	-9.60E-02	1.79E-02	6.79E-01	rs7717591	5_52415905_A_G	1.00	7174	8.41E-08	GCST90277314
Phosphatidylcholine (18:2_18:2) levels	7	91299442	T	G	-8.18E-02	1.77E-02	3.39E-01	rs7798734	7_91299442_G_T	0.99	7174	3.70E-06	GCST90277314
Phosphatidylcholine (18:2_18:2) levels	7	120336033	G	A	1.58E-01	3.44E-02	6.95E-02	rs35958132	7_120336033_A_G	0.91	7174	4.47E-06	GCST90277314
Phosphatidylcholine (18:2_18:2) levels	10	93203138	T	C	3.62E-01	7.96E-02	1.18E-02	rs117017186	10_93203138_C_T	0.95	7174	5.33E-06	GCST90277314
Phosphatidylcholine (18:2_18:2) levels	11	61832870	C	A	-2.22E-01	1.69E-02	5.91E-01	rs174574	11_61832870_A_C	1.00	7174	4.16E-39	GCST90277314
Phosphatidylcholine (18:2_18:2) levels	11	107883610	G	C	-1.08E-01	2.38E-02	8.60E-01	rs7122803	11_107883610_C_G	1.00	7174	6.32E-06	GCST90277314
Phosphatidylcholine (18:2_18:2) levels	13	79574011	T	C	-8.39E-01	1.83E-01	2.53E-03	rs147002062	13_79574011_C_T	0.82	7174	4.77E-06	GCST90277314
Phosphatidylcholine (18:2_18:2) levels	15	58379566	T	G	-9.40E-02	1.71E-02	6.11E-01	rs1601935	15_58379566_G_T	0.99	7174	3.75E-08	GCST90277314
Phosphatidylcholine (18:2_18:2) levels	15	58431227	G	A	1.15E-01	1.92E-02	2.66E-01	rs1077835	15_58431227_A_G	0.98	7174	2.66E-09	GCST90277314
Phosphatidylcholine (18:2_18:2) levels	16	15036737	G	A	9.34E-02	1.77E-02	3.34E-01	rs6498540	16_15036737_A_G	0.99	7174	1.44E-07	GCST90277314
Phosphatidylcholine (18:2_18:2) levels	16	56954132	T	C	9.84E-02	1.85E-02	2.83E-01	rs173539	16_56954132_C_T	1.00	7174	1.01E-07	GCST90277314
Phosphatidylcholine (18:2_18:2) levels	17	34718046	T	C	-7.66E-02	1.71E-02	5.85E-01	rs11869356	17_34718046_C_T	0.99	7174	7.72E-06	GCST90277314
Phosphatidylcholine (18:2_18:2) levels	20	46799183	A	G	3.21E-01	6.54E-02	1.76E-02	rs117675848	20_46799183_G_A	0.95	7174	9.54E-07	GCST90277314
Phosphatidylcholine (18:2_18:2) levels	21	25877514	A	G	-7.68E-02	1.68E-02	4.18E-01	rs80315588	21_25877514_G_A	1.00	7174	5.15E-06	GCST90277314
Phosphatidylcholine (18:2_18:2) levels	21	37563977	C	G	7.58E-02	1.67E-02	4.32E-01	rs2236605	21_37563977_G_C	0.99	7174	6.19E-06	GCST90277314
Phosphatidylcholine (18:2_20:1) levels	3	38782852	G	A	-9.38E-02	2.12E-02	7.10E-01	rs4622847	3_38782852_A_G	0.99	5521	9.74E-06	GCST90277315
Phosphatidylcholine (18:2_20:1) levels	3	87312021	T	C	8.94E-02	1.92E-02	4.23E-01	rs56128146	3_87312021_C_T	1.00	5521	3.25E-06	GCST90277315
Phosphatidylcholine (18:2_20:1) levels	3	116723503	C	T	9.12E-02	1.97E-02	6.15E-01	rs17484	3_116723503_T_C	0.99	5521	3.72E-06	GCST90277315
Phosphatidylcholine (18:2_20:1) levels	4	14321016	A	G	8.59E-02	1.91E-02	4.25E-01	rs9994217	4_14321016_G_A	0.99	5521	7.26E-06	GCST90277315
Phosphatidylcholine (18:2_20:1) levels	4	74104898	A	C	2.66E-01	5.65E-02	3.03E-02	rs78098253	4_74104898_C_A	0.96	5521	2.53E-06	GCST90277315
Phosphatidylcholine (18:2_20:1) levels	4	85577702	C	T	3.60E-01	7.80E-02	1.78E-02	rs62315647	4_85577702_T_C	0.86	5521	3.96E-06	GCST90277315
Phosphatidylcholine (18:2_20:1) levels	5	33202141	A	C	-9.31E-02	2.10E-02	2.90E-01	rs11950850	5_33202141_C_A	1.00	5521	9.19E-06	GCST90277315
Phosphatidylcholine (18:2_20:1) levels	5	112580236	T	C	-1.58E-01	3.52E-02	8.05E-02	rs72795807	5_112580236_C_T	0.96	5521	7.34E-06	GCST90277315
Phosphatidylcholine (18:2_20:1) levels	6	138435656	T	A	-1.31E-01	2.78E-02	1.54E-01	rs9402974	6_138435656_A_T	0.91	5521	2.55E-06	GCST90277315
Phosphatidylcholine (18:2_20:1) levels	8	128749313	T	C	-9.67E-02	1.98E-02	3.74E-01	rs200921913	8_128749313_C_T	0.98	5521	1.12E-06	GCST90277315
Phosphatidylcholine (18:2_20:1) levels	10	70181497	T	A	8.45E-02	1.90E-02	4.39E-01	rs10740329	10_70181497_A_T	1.00	5521	8.42E-06	GCST90277315
Phosphatidylcholine (18:2_20:1) levels	11	61594967	G	A	2.41E-01	5.08E-02	3.69E-02	rs79136768	11_61594967_A_G	0.98	5521	2.05E-06	GCST90277315
Phosphatidylcholine (18:2_20:1) levels	11	61678244	C	A	-2.42E-01	4.53E-02	4.87E-02	rs182173873	11_61678244_A_C	0.97	5521	9.15E-08	GCST90277315
Phosphatidylcholine (18:2_20:1) levels	11	61854084	C	G	3.23E-01	1.87E-02	4.19E-01	rs174599	11_61854084_G_C	1.00	5521	3.92E-65	GCST90277315
Phosphatidylcholine (18:2_20:1) levels	11	64254349	T	C	1.51E-01	2.79E-02	1.30E-01	rs2302263	11_64254349_C_T	1.00	5521	6.10E-08	GCST90277315
Phosphatidylcholine (18:2_20:1) levels	11	97015272	A	G	2.77E-01	6.08E-02	2.95E-02	rs72956488	11_97015272_G_A	0.91	5521	5.44E-06	GCST90277315
Phosphatidylcholine (18:2_20:1) levels	12	31290266	T	C	2.54E-01	5.66E-02	3.01E-02	rs117210128	12_31290266_C_T	0.97	5521	7.49E-06	GCST90277315
Phosphatidylcholine (18:2_20:1) levels	17	36639734	T	C	-1.03E-01	2.29E-02	2.61E-01	rs211991	17_36639734_C_T	0.91	5521	6.83E-06	GCST90277315
Phosphatidylcholine (18:2_20:1) levels	17	68929782	T	C	-9.37E-02	1.93E-02	5.50E-01	rs4147976	17_68929782_C_T	0.98	5521	1.28E-06	GCST90277315
Phosphatidylcholine (18:2_20:3) levels	1	62679768	A	G	-9.24E-02	1.98E-02	2.62E-01	rs11208004	1_62679768_G_A	1.00	6615	3.11E-06	GCST90277316
Phosphatidylcholine (18:2_20:3) levels	1	87589957	T	G	1.64E-01	3.72E-02	5.65E-02	rs60417255	1_87589957_G_T	0.98	6615	9.89E-06	GCST90277316

Phosphatidylcholine (18:2_20:3) levels	1	91866179	A	T	-1.58E-01	3.52E-02	6.38E-02	rs2489189	1_91866179_T_A	1.00	6615	7.09E-06	GCST90277316
Phosphatidylcholine (18:2_20:3) levels	1	230167404	T	C	1.04E-01	2.08E-02	7.77E-01	rs10779836	1_230167404_C_T	1.00	6615	6.56E-07	GCST90277316
Phosphatidylcholine (18:2_20:3) levels	3	23041410	G	A	-2.00E-01	4.48E-02	3.81E-02	rs75662719	3_23041410_A_G	1.00	6615	8.25E-06	GCST90277316
Phosphatidylcholine (18:2_20:3) levels	4	43914879	C	A	-9.34E-02	1.85E-02	6.74E-01	rs2347672	4_43914879_A_C	1.00	6615	4.46E-07	GCST90277316
Phosphatidylcholine (18:2_20:3) levels	5	99176070	A	G	-5.83E-01	1.22E-01	4.93E-03	rs138827081	5_99176070_G_A	0.98	6615	1.97E-06	GCST90277316
Phosphatidylcholine (18:2_20:3) levels	6	29796264	T	C	4.71E-01	1.05E-01	7.34E-03	rs11757990	6_29796264_C_T	0.97	6615	7.54E-06	GCST90277316
Phosphatidylcholine (18:2_20:3) levels	6	98413921	C	G	-2.77E-01	6.10E-02	2.05E-02	rs146084531	6_98413921_G_C	0.99	6615	5.82E-06	GCST90277316
Phosphatidylcholine (18:2_20:3) levels	7	155488156	T	C	-1.54E-01	3.28E-02	8.37E-02	rs76833802	7_155488156_C_T	0.92	6615	2.58E-06	GCST90277316
Phosphatidylcholine (18:2_20:3) levels	10	58741730	C	T	-1.31E-01	2.94E-02	1.01E-01	rs35480480	10_58741730_T_C	0.96	6615	8.14E-06	GCST90277316
Phosphatidylcholine (18:2_20:3) levels	11	15133256	A	C	-3.37E-01	7.52E-02	1.40E-02	rs117663514	11_15133256_C_A	0.94	6615	7.48E-06	GCST90277316
Phosphatidylcholine (18:2_20:3) levels	11	61803876	G	C	2.10E-01	1.78E-02	3.84E-01	rs174548	11_61803876_C_G	1.00	6615	1.40E-31	GCST90277316
Phosphatidylcholine (18:2_20:3) levels	11	68952542	A	G	8.79E-02	1.79E-02	3.82E-01	rs10896384	11_68952542_G_A	1.00	6615	9.23E-07	GCST90277316
Phosphatidylcholine (18:2_20:3) levels	11	75745535	T	A	-1.09E-01	2.05E-02	2.41E-01	rs695112	11_75745535_A_T	0.99	6615	9.51E-08	GCST90277316
Phosphatidylcholine (18:2_20:3) levels	11	91426863	C	T	4.25E-01	9.42E-02	8.81E-03	rs16916055	11_91426863_T_C	0.97	6615	6.42E-06	GCST90277316
Phosphatidylcholine (18:2_20:3) levels	11	133528221	G	A	1.08E-01	2.40E-02	1.56E-01	rs4130016	11_133528221_A_G	1.00	6615	7.13E-06	GCST90277316
Phosphatidylcholine (18:2_20:3) levels	12	26133418	G	A	2.09E-01	4.42E-02	4.34E-02	rs75719904	12_26133418_A_G	0.98	6615	2.21E-06	GCST90277316
Phosphatidylcholine (18:2_20:3) levels	12	116425661	T	G	7.77E-02	1.72E-02	5.31E-01	rs10774859	12_116425661_G_T	1.00	6615	6.74E-06	GCST90277316
Phosphatidylcholine (18:2_20:3) levels	18	52821119	A	C	-1.05E-01	2.08E-02	2.32E-01	rs2096994	18_52821119_C_A	1.00	6615	4.59E-07	GCST90277316
Phosphatidylcholine (18:2_20:3) levels	19	19210016	G	C	-2.04E-01	4.06E-02	5.22E-02	rs150057262	19_19210016_C_G	0.94	6615	5.10E-07	GCST90277316
Phosphatidylcholine (18:2_20:4) levels	1	168675568	A	G	-8.23E-02	1.85E-02	7.04E-01	rs2419125	1_168675568_G_A	0.98	7049	8.59E-06	GCST90277317
Phosphatidylcholine (18:2_20:4) levels	1	202937186	T	C	-3.07E-01	6.87E-02	1.59E-02	rs114076093	1_202937186_C_T	0.95	7049	8.05E-06	GCST90277317
Phosphatidylcholine (18:2_20:4) levels	2	68091692	T	G	-1.81E-01	3.90E-02	5.13E-02	rs72830074	2_68091692_G_T	0.97	7049	3.53E-06	GCST90277317
Phosphatidylcholine (18:2_20:4) levels	3	76875178	C	T	-7.68E-02	1.70E-02	4.37E-01	rs10446372	3_76875178_T_C	0.99	7049	6.17E-06	GCST90277317
Phosphatidylcholine (18:2_20:4) levels	3	138128558	T	A	9.56E-02	1.88E-02	2.79E-01	rs34689885	3_138128558_A_T	1.00	7049	3.76E-07	GCST90277317
Phosphatidylcholine (18:2_20:4) levels	4	7394662	A	G	-1.73E-01	3.77E-02	5.63E-02	rs77985607	4_7394662_G_A	0.95	7049	4.18E-06	GCST90277317
Phosphatidylcholine (18:2_20:4) levels	4	37584805	T	C	7.93E-02	1.71E-02	5.06E-01	rs2973222	4_37584805_C_T	0.97	7049	3.39E-06	GCST90277317
Phosphatidylcholine (18:2_20:4) levels	4	173581856	G	A	2.17E-01	4.91E-02	3.20E-02	rs4695858	4_173581856_A_G	0.94	7049	9.96E-06	GCST90277317
Phosphatidylcholine (18:2_20:4) levels	6	104035109	C	G	-3.58E-01	8.04E-02	1.23E-02	rs117919959	6_104035109_G_C	0.91	7049	8.83E-06	GCST90277317
Phosphatidylcholine (18:2_20:4) levels	7	18606087	T	C	4.32E-01	9.30E-02	8.82E-03	rs117352906	7_18606087_C_T	0.92	7049	3.47E-06	GCST90277317
Phosphatidylcholine (18:2_20:4) levels	7	100924735	A	G	-8.19E-02	1.75E-02	6.08E-01	rs62482552	7_100924735_G_A	0.98	7049	2.81E-06	GCST90277317
Phosphatidylcholine (18:2_20:4) levels	7	153908913	T	C	-3.04E-01	5.74E-02	2.32E-02	rs139923737	7_153908913_C_T	0.94	7049	1.24E-07	GCST90277317
Phosphatidylcholine (18:2_20:4) levels	11	15423060	A	G	-1.22E-01	2.72E-02	8.90E-01	rs4237708	11_15423060_G_A	0.98	7049	7.26E-06	GCST90277317
Phosphatidylcholine (18:2_20:4) levels	11	43360418	G	T	2.35E-01	4.95E-02	3.05E-02	rs76780983	11_43360418_T_G	0.98	7049	2.11E-06	GCST90277317
Phosphatidylcholine (18:2_20:4) levels	11	61790354	C	T	-1.74E-01	1.71E-02	4.09E-01	rs102274	11_61790354_T_C	1.00	7049	2.66E-24	GCST90277317
Phosphatidylcholine (18:2_20:4) levels	11	75739531	C	T	-9.75E-02	1.97E-02	2.45E-01	rs673335	11_75739531_T_C	0.99	7049	7.43E-07	GCST90277317
Phosphatidylcholine (18:2_20:4) levels	13	40081407	G	A	-1.34E-01	2.63E-02	1.20E-01	rs71425785	13_40081407_A_G	0.95	7049	3.66E-07	GCST90277317
Phosphatidylcholine (18:2_20:4) levels	15	58379566	T	G	-8.16E-02	1.73E-02	6.11E-01	rs1601935	15_58379566_G_T	0.99	7049	2.32E-06	GCST90277317
Phosphatidylcholine (18:2_20:4) levels	15	58431280	C	T	1.23E-01	1.94E-02	2.66E-01	rs1077834	15_58431280_T_C	0.98	7049	2.67E-10	GCST90277317
Phosphatidylcholine (18:2_20:4) levels	15	89110533	G	A	-9.68E-02	1.74E-02	3.98E-01	rs1033155	15_89110533_A_G	1.00	7049	2.50E-08	GCST90277317
Phosphatidylcholine (18:2_20:4) levels	16	23549438	T	C	2.03E-01	4.26E-02	4.75E-02	rs75528309	16_23549438_C_T	0.85	7049	1.82E-06	GCST90277317
Phosphatidylcholine (18:2_20:4) levels	16	56954132	T	C	8.90E-02	1.87E-02	2.83E-01	rs173539	16_56954132_C_T	1.00	7049	1.92E-06	GCST90277317
Phosphatidylcholine (18:2_20:4) levels	16	81367012	A	G	-2.21E-01	4.46E-02	3.91E-02	rs79450037	16_81367012_G_A	0.98	7049	7.72E-07	GCST90277317
Phosphatidylcholine (18:2_20:4) levels	17	18105133	A	G	-7.42E-02	1.68E-02	5.02E-01	rs712265	17_18105133_G_A	1.00	7049	9.65E-06	GCST90277317
Phosphatidylcholine (18:2_20:4) levels	17	44595295	C	T	1.46E-01	3.25E-02	7.30E-02	rs9889746	17_44595295_T_C	0.98	7049	6.66E-06	GCST90277317
Phosphatidylcholine (18:2_20:4) levels	19	8084803	T	G	-7.69E-02	1.71E-02	4.40E-01	rs55942656	19_8084803_G_T	0.98	7049	6.88E-06	GCST90277317
Phosphatidylcholine (18:2_20:4) levels	19	19282905	A	G	-1.41E-01	2.27E-02	1.71E-01	rs8100204	19_19282905_G_A	0.97	7049	4.82E-10	GCST90277317
Phosphatidylcholine (18:2_20:4) levels	19	19760295	C	T	8.05E-02	1.70E-02	5.23E-01	rs248968	19_19760295_T_C	1.00	7049	2.38E-06	GCST90277317
Phosphatidylcholine (18:2_20:4) levels	19	20102422	T	C	-8.37E-02	1.79E-02	3.43E-01	rs836912	19_20102422_C_T	0.96	7049	3.12E-06	GCST90277317
Phosphatidylcholine (18:2_20:4) levels	19	54173495	C	T	-8.45E-02	1.72E-02	5.95E-01	rs8736	19_54173495_T_C	0.97	7049	9.99E-07	GCST90277317
Phosphatidylcholine (18:2_20:4) levels	21	18184728	T	C	-1.40E-01	2.89E-02	9.36E-02	rs8131052	21_18184728_C_T	0.99	7049	1.25E-06	GCST90277317
Phosphatidylcholine (O-16:0_16:0) levels	1	95674018	A	T	-1.49E-01	3.20E-02	8.13E-02	rs6675844	1_95674018_T_A	0.99	6605	3.50E-06	GCST90277318
Phosphatidylcholine (O-16:0_16:0) levels	1	155011063	T	C	8.04E-02	1.80E-02	4.00E-01	rs6686021	1_155011063_C_T	0.98	6605	7.97E-06	GCST90277318
Phosphatidylcholine (O-16:0_16:0) levels	2	49428827	C	T	2.81E-01	6.33E-02	1.97E-02	rs150651997	2_49428827_T_C	0.98	6605	9.15E-06	GCST90277318
Phosphatidylcholine (O-16:0_16:0) levels	2	227808434	G	C	-2.06E-01	4.44E-02	4.30E-02	rs148625143	2_227808434_C_G	0.93	6605	3.54E-06	GCST90277318
Phosphatidylcholine (O-16:0_16:0) levels	3	2923966	A	G	-8.44E-02	1.87E-02	3.23E-01	rs17586917	3_2923966_G_A	0.98	6605	6.69E-06	GCST90277318
Phosphatidylcholine (O-16:0_16:0) levels	4	144919778	C	A	-2.23E-01	4.56E-02	3.73E-02	rs115294982	4_144919778_A_C	0.99	6605	1.08E-06	GCST90277318
Phosphatidylcholine (O-16:0_16:0) levels	7	82989938	C	A	-1.89E-01	4.13E-02	4.80E-02	rs76494046	7_82989938_A_C	0.99	6605	4.97E-06	GCST90277318

Phosphatidylcholine (O-16:0_16:0) levels	7	97045603	T	C	2.53E-01	5.72E-02	2.61E-02	rs80134854	7_97045603_C_T	0.94	6605	9.65E-06	GCST90277318
Phosphatidylcholine (O-16:0_16:0) levels	8	71111290	G	A	9.99E-02	2.23E-02	1.94E-01	rs6987890	8_71111290_A_G	1.00	6605	7.34E-06	GCST90277318
Phosphatidylcholine (O-16:0_16:0) levels	9	81097537	A	G	-2.92E-01	6.40E-02	1.89E-02	rs72739734	9_81097537_G_A	0.97	6605	5.02E-06	GCST90277318
Phosphatidylcholine (O-16:0_16:0) levels	12	114634472	C	T	1.49E-01	3.28E-02	7.85E-02	rs112728288	12_114634472_T_C	0.98	6605	5.53E-06	GCST90277318
Phosphatidylcholine (O-16:0_16:0) levels	13	67207825	A	T	3.90E-01	8.57E-02	1.15E-02	rs17592511	13_67207825_T_A	0.89	6605	5.43E-06	GCST90277318
Phosphatidylcholine (O-16:0_16:0) levels	15	88328568	G	A	-1.30E-01	2.86E-02	1.11E-01	rs12440833	15_88328568_A_G	0.92	6605	5.88E-06	GCST90277318
Phosphatidylcholine (O-16:0_16:0) levels	15	94708362	A	C	8.26E-02	1.83E-02	6.70E-01	rs7170186	15_94708362_C_A	1.00	6605	6.71E-06	GCST90277318
Phosphatidylcholine (O-16:0_16:0) levels	16	10794552	C	G	-1.52E-01	3.41E-02	9.10E-02	rs74591482	16_10794552_G_C	0.80	6605	7.86E-06	GCST90277318
Phosphatidylcholine (O-16:0_16:0) levels	17	1884243	C	G	1.51E-01	3.39E-02	7.65E-02	rs17292238	17_1884243_G_C	0.91	6605	8.61E-06	GCST90277318
Phosphatidylcholine (O-16:0_16:0) levels	17	55740741	T	G	9.48E-02	2.11E-02	2.50E-01	rs1160027	17_55740741_G_T	0.89	6605	7.44E-06	GCST90277318
Phosphatidylcholine (O-16:0_16:0) levels	17	82135397	A	G	2.38E-01	4.82E-02	3.56E-02	rs75248478	17_82135397_G_A	0.95	6605	7.88E-07	GCST90277318
Phosphatidylcholine (O-16:0_16:0) levels	18	8859056	G	A	8.60E-02	1.86E-02	3.38E-01	rs34115485	18_8859056_A_G	1.00	6605	3.75E-06	GCST90277318
Phosphatidylcholine (O-16:0_16:0) levels	19	42262816	A	G	-7.04E-01	1.55E-01	3.75E-03	rs117091735	19_42262816_G_A	0.87	6605	5.89E-06	GCST90277318
Phosphatidylcholine (O-16:0_16:1) levels	2	47577655	C	G	1.50E-01	3.36E-02	9.01E-02	rs114541469	2_47577655_G_C	0.95	5658	8.33E-06	GCST90277319
Phosphatidylcholine (O-16:0_16:1) levels	2	70336870	A	C	-9.43E-02	1.95E-02	3.89E-01	rs13394815	2_70336870_C_A	0.97	5658	1.29E-06	GCST90277319
Phosphatidylcholine (O-16:0_16:1) levels	2	101572416	A	C	1.07E-01	2.14E-02	2.68E-01	rs77494629	2_101572416_C_A	0.99	5658	6.54E-07	GCST90277319
Phosphatidylcholine (O-16:0_16:1) levels	2	158513491	A	G	1.26E-01	2.80E-02	8.69E-01	rs2356187	2_158513491_G_A	1.00	5658	6.54E-06	GCST90277319
Phosphatidylcholine (O-16:0_16:1) levels	4	133202073	A	G	-1.62E-01	2.91E-02	1.24E-01	rs62311841	4_133202073_G_A	0.99	5658	2.88E-08	GCST90277319
Phosphatidylcholine (O-16:0_16:1) levels	5	79597004	G	A	1.94E-01	3.90E-02	9.34E-01	rs6453462	5_79597004_A_G	0.92	5658	6.61E-07	GCST90277319
Phosphatidylcholine (O-16:0_16:1) levels	7	131005149	A	T	1.44E-01	3.20E-02	9.99E-02	rs10248190	7_131005149_T_A	0.96	5658	7.48E-06	GCST90277319
Phosphatidylcholine (O-16:0_16:1) levels	8	51454913	A	G	2.10E-01	4.64E-02	4.36E-02	rs16916332	8_51454913_G_A	1.00	5658	6.24E-06	GCST90277319
Phosphatidylcholine (O-16:0_16:1) levels	9	31384831	C	T	2.02E-01	4.15E-02	5.84E-02	rs146085506	9_31384831_T_C	0.94	5658	1.20E-06	GCST90277319
Phosphatidylcholine (O-16:0_16:1) levels	9	69669802	C	A	9.53E-01	2.12E-01	9.98E-01	rs4744927	9_69669802_A_C	0.86	5658	7.11E-06	GCST90277319
Phosphatidylcholine (O-16:0_16:1) levels	10	100315722	A	G	-1.43E-01	2.94E-02	1.21E-01	rs603424	10_100315722_G_A	1.00	5658	1.10E-06	GCST90277319
Phosphatidylcholine (O-16:0_16:1) levels	11	45579256	A	G	-8.48E-02	1.87E-02	5.26E-01	rs728516	11_45579256_G_A	1.00	5658	5.97E-06	GCST90277319
Phosphatidylcholine (O-16:0_16:1) levels	11	105208627	G	A	-1.45E-01	3.07E-02	1.09E-01	rs72973984	11_105208627_A_G	0.98	5658	2.43E-06	GCST90277319
Phosphatidylcholine (O-16:0_16:1) levels	13	62695542	T	C	-5.21E-01	1.10E-01	7.46E-03	rs117421874	13_62695542_C_T	0.95	5658	2.36E-06	GCST90277319
Phosphatidylcholine (O-16:0_16:1) levels	14	62141305	G	A	-1.66E-01	3.58E-02	7.32E-02	rs28609532	14_62141305_A_G	1.00	5658	3.61E-06	GCST90277319
Phosphatidylcholine (O-16:0_16:1) levels	15	58431476	T	C	1.10E-01	2.18E-02	2.58E-01	rs1800588	15_58431476_C_T	0.99	5658	4.86E-07	GCST90277319
Phosphatidylcholine (O-16:0_16:1) levels	16	56960616	T	C	1.33E-01	2.08E-02	2.78E-01	rs17231506	16_56960616_C_T	1.00	5658	1.90E-10	GCST90277319
Phosphatidylcholine (O-16:0_18:1) levels	1	230180443	G	A	-8.24E-02	1.75E-02	3.74E-01	rs606587	1_230180443_A_G	1.00	6956	2.49E-06	GCST90277320
Phosphatidylcholine (O-16:0_18:1) levels	2	48551738	T	C	-1.11E-01	2.36E-02	1.50E-01	rs876969	2_48551738_C_T	1.00	6956	2.40E-06	GCST90277320
Phosphatidylcholine (O-16:0_18:1) levels	3	4949091	C	T	-8.00E-02	1.73E-02	4.35E-01	rs9682490	3_4949091_T_C	0.98	6956	3.82E-06	GCST90277320
Phosphatidylcholine (O-16:0_18:1) levels	4	137022381	G	C	3.93E-01	7.22E-02	1.43E-02	rs7677028	4_137022381_C_G	0.97	6956	5.29E-08	GCST90277320
Phosphatidylcholine (O-16:0_18:1) levels	5	115413799	C	G	7.91E-02	1.79E-02	5.81E-01	rs2963775	5_115413799_G_C	0.94	6956	9.87E-06	GCST90277320
Phosphatidylcholine (O-16:0_18:1) levels	6	31307500	G	A	1.12E-01	2.41E-02	1.45E-01	rs13194791	6_31307500_A_G	0.99	6956	3.55E-06	GCST90277320
Phosphatidylcholine (O-16:0_18:1) levels	8	2845634	G	C	1.54E-01	3.21E-02	7.69E-02	rs7845596	8_2845634_C_G	1.00	6956	1.59E-06	GCST90277320
Phosphatidylcholine (O-16:0_18:1) levels	8	31640951	A	G	8.71E-02	1.85E-02	2.99E-01	rs34861396	8_31640951_G_A	0.99	6956	2.52E-06	GCST90277320
Phosphatidylcholine (O-16:0_18:1) levels	8	118920650	T	C	8.87E-02	1.97E-02	2.48E-01	rs138038919	8_118920650_C_T	1.00	6956	7.10E-06	GCST90277320
Phosphatidylcholine (O-16:0_18:1) levels	9	26919609	G	T	9.22E-02	2.07E-02	2.08E-01	rs10120342	9_26919609_T_G	1.00	6956	8.42E-06	GCST90277320
Phosphatidylcholine (O-16:0_18:1) levels	11	12450600	C	T	1.89E-01	4.14E-02	4.91E-02	rs77211823	11_12450600_T_C	0.89	6956	4.83E-06	GCST90277320
Phosphatidylcholine (O-16:0_18:1) levels	11	35044759	T	G	-1.52E-01	3.29E-02	7.36E-02	rs72923423	11_35044759_G_T	0.99	6956	4.17E-06	GCST90277320
Phosphatidylcholine (O-16:0_18:1) levels	12	124844767	C	T	-7.74E-02	1.71E-02	4.54E-01	rs11057853	12_124844767_T_C	0.99	6956	6.20E-06	GCST90277320
Phosphatidylcholine (O-16:0_18:1) levels	13	60820833	A	C	1.43E-01	3.14E-02	8.17E-02	rs9538944	13_60820833_C_A	0.98	6956	5.62E-06	GCST90277320
Phosphatidylcholine (O-16:0_18:1) levels	15	58391167	G	A	-9.83E-02	1.71E-02	5.73E-01	rs1532085	15_58391167_A_G	1.00	6956	9.47E-09	GCST90277320
Phosphatidylcholine (O-16:0_18:1) levels	15	58431476	T	C	1.19E-01	1.96E-02	2.58E-01	rs1800588	15_58431476_C_T	0.99	6956	1.29E-09	GCST90277320
Phosphatidylcholine (O-16:0_18:1) levels	16	56960616	T	C	1.29E-01	1.88E-02	2.78E-01	rs17231506	16_56960616_C_T	1.00	6956	6.16E-12	GCST90277320
Phosphatidylcholine (O-16:0_18:1) levels	16	71063316	A	G	3.33E-01	7.20E-02	1.52E-02	rs117449635	16_71063316_G_A	0.92	6956	3.83E-06	GCST90277320
Phosphatidylcholine (O-16:0_18:1) levels	16	72797212	C	G	2.19E-01	4.77E-02	3.45E-02	rs79037957	16_72797212_G_C	0.95	6956	4.45E-06	GCST90277320
Phosphatidylcholine (O-16:0_18:1) levels	16	85344145	T	C	2.73E-01	5.93E-02	2.30E-02	rs113746682	16_85344145_C_T	0.91	6956	4.12E-06	GCST90277320
Phosphatidylcholine (O-16:0_18:1) levels	19	11082239	G	A	-1.37E-01	2.93E-02	9.21E-02	rs73015021	19_11082239_A_G	0.98	6956	2.92E-06	GCST90277320
Phosphatidylcholine (O-16:0_18:1) levels	20	11603340	A	G	-7.94E-02	1.74E-02	3.94E-01	rs7260891	20_11603340_G_A	0.99	6956	5.06E-06	GCST90277320
Phosphatidylcholine (O-16:0_18:1) levels	20	55260213	C	T	-3.26E-01	7.31E-02	1.43E-02	rs6023985	20_55260213_T_C	0.91	6956	8.23E-06	GCST90277320
Phosphatidylcholine (O-16:0_18:2) levels	2	207017121	T	C	8.48E-02	1.81E-02	3.29E-01	rs6741227	2_207017121_C_T	1.00	7158	2.78E-06	GCST90277321
Phosphatidylcholine (O-16:0_18:2) levels	3	112816210	A	G	1.06E-01	2.30E-02	1.59E-01	rs11918522	3_112816210_G_A	1.00	7158	4.10E-06	GCST90277321
Phosphatidylcholine (O-16:0_18:2) levels	3	128653644	A	G	-1.34E-01	2.97E-02	8.68E-02	rs6764912	3_128653644_G_A	0.98	7158	6.76E-06	GCST90277321
Phosphatidylcholine (O-16:0_18:2) levels	4	151270229	G	A	-1.26E-01	2.84E-02	9.98E-02	rs11936543	4_151270229_A_G	0.97	7158	8.95E-06	GCST90277321

Phosphatidylcholine (O-16:0_18:2) levels	5	96599571	C	G	-1.17E-01	2.48E-02	1.30E-01	rs17086339	5_96599571_G_C	0.99	7158	2.67E-06	GCST90277321
Phosphatidylcholine (O-16:0_18:2) levels	6	75047845	C	T	-1.08E-01	2.24E-02	8.34E-01	rs4579316	6_75047845_T_C	1.00	7158	1.66E-06	GCST90277321
Phosphatidylcholine (O-16:0_18:2) levels	6	107204231	A	G	5.66E-01	1.28E-01	4.47E-03	rs149918547	6_107204231_G_A	0.97	7158	9.97E-06	GCST90277321
Phosphatidylcholine (O-16:0_18:2) levels	7	10960253	G	C	-2.37E-01	5.37E-02	2.64E-02	rs79195215	7_10960253_C_G	0.96	7158	9.81E-06	GCST90277321
Phosphatidylcholine (O-16:0_18:2) levels	7	17693175	T	C	7.77E-02	1.73E-02	3.72E-01	rs2537611	7_17693175_C_T	1.00	7158	6.90E-06	GCST90277321
Phosphatidylcholine (O-16:0_18:2) levels	7	69316302	A	G	-1.32E-01	2.96E-02	9.63E-02	rs75863117	7_69316302_G_A	0.92	7158	8.85E-06	GCST90277321
Phosphatidylcholine (O-16:0_18:2) levels	7	117677562	T	C	6.76E-01	1.51E-01	3.98E-03	rs76888052	7_117677562_C_T	0.81	7158	8.00E-06	GCST90277321
Phosphatidylcholine (O-16:0_18:2) levels	8	22971029	T	C	-7.74E-02	1.75E-02	3.45E-01	rs11777584	8_22971029_C_T	0.99	7158	9.95E-06	GCST90277321
Phosphatidylcholine (O-16:0_18:2) levels	9	14779673	T	A	7.65E-02	1.68E-02	5.31E-01	rs923920	9_14779673_A_T	1.00	7158	5.10E-06	GCST90277321
Phosphatidylcholine (O-16:0_18:2) levels	11	61832870	C	A	-1.81E-01	1.70E-02	5.91E-01	rs174574	11_61832870_A_C	1.00	7158	1.55E-26	GCST90277321
Phosphatidylcholine (O-16:0_18:2) levels	12	76023289	T	A	-2.14E-01	4.72E-02	9.65E-01	rs6582326	12_76023289_A_T	0.93	7158	5.85E-06	GCST90277321
Phosphatidylcholine (O-16:0_18:2) levels	15	58391167	G	A	-8.60E-02	1.68E-02	5.73E-01	rs1532085	15_58391167_A_G	1.00	7158	3.40E-07	GCST90277321
Phosphatidylcholine (O-16:0_18:2) levels	16	14645161	T	A	-4.70E-01	1.03E-01	7.47E-03	rs181750334	16_14645161_A_T	0.91	7158	5.09E-06	GCST90277321
Phosphatidylcholine (O-16:0_18:2) levels	16	56960616	T	C	1.22E-01	1.86E-02	2.78E-01	rs17231506	16_56960616_C_T	1.00	7158	6.06E-11	GCST90277321
Phosphatidylcholine (O-16:0_18:2) levels	18	24042039	T	A	1.31E-01	2.95E-02	8.96E-02	rs78285907	18_24042039_A_T	0.98	7158	8.60E-06	GCST90277321
Phosphatidylcholine (O-16:0_18:2) levels	20	44186250	A	G	1.09E-01	2.37E-02	1.50E-01	rs75804573	20_44186250_G_A	0.98	7158	4.52E-06	GCST90277321
Phosphatidylcholine (O-16:0_18:2) levels	22	32427941	T	C	-3.21E-01	7.01E-02	1.51E-02	rs140661870	22_32427941_C_T	0.93	7158	4.84E-06	GCST90277321
Phosphatidylcholine (O-16:0_20:3) levels	2	71103693	T	C	9.09E-02	2.03E-02	2.52E-01	rs1833350	2_71103693_C_T	0.97	6709	7.62E-06	GCST90277322
Phosphatidylcholine (O-16:0_20:3) levels	3	134061016	A	G	-1.09E-01	2.33E-02	1.62E-01	rs75111872	3_134061016_G_A	1.00	6709	2.93E-06	GCST90277322
Phosphatidylcholine (O-16:0_20:3) levels	7	3168526	G	A	-8.72E-02	1.96E-02	7.25E-01	rs10951096	7_3168526_A_G	0.99	6709	8.90E-06	GCST90277322
Phosphatidylcholine (O-16:0_20:3) levels	7	28483085	A	C	3.11E-01	6.91E-02	1.67E-02	rs12671763	7_28483085_C_A	0.95	6709	6.85E-06	GCST90277322
Phosphatidylcholine (O-16:0_20:3) levels	7	29412521	G	A	8.08E-02	1.72E-02	5.19E-01	rs968742	7_29412521_A_G	1.00	6709	2.68E-06	GCST90277322
Phosphatidylcholine (O-16:0_20:3) levels	8	15196227	T	C	-1.01E-01	2.24E-02	1.80E-01	rs62501991	8_15196227_C_T	1.00	6709	7.35E-06	GCST90277322
Phosphatidylcholine (O-16:0_20:3) levels	9	420203	C	G	9.12E-02	2.04E-02	7.66E-01	rs2360706	9_420203_G_C	0.99	6709	7.69E-06	GCST90277322
Phosphatidylcholine (O-16:0_20:3) levels	10	124011070	A	G	5.29E-01	1.14E-01	6.95E-03	rs117435540	10_124011070_G_A	0.88	6709	3.69E-06	GCST90277322
Phosphatidylcholine (O-16:0_20:3) levels	11	61828092	T	C	2.71E-01	2.71E-02	1.13E-01	rs968567	11_61828092_C_T	1.00	6709	2.17E-23	GCST90277322
Phosphatidylcholine (O-16:0_20:3) levels	11	118930336	A	G	2.38E-01	5.34E-02	2.79E-02	rs75085081	11_118930336_G_A	0.94	6709	8.47E-06	GCST90277322
Phosphatidylcholine (O-16:0_20:3) levels	13	23426939	T	C	-1.36E-01	2.99E-02	9.51E-02	rs9552958	13_23426939_C_T	0.96	6709	5.66E-06	GCST90277322
Phosphatidylcholine (O-16:0_20:3) levels	13	52780240	C	A	-1.97E-01	4.33E-02	4.20E-02	rs79036443	13_52780240_A_C	0.96	6709	5.65E-06	GCST90277322
Phosphatidylcholine (O-16:0_20:3) levels	15	58431476	T	C	9.60E-02	2.00E-02	2.58E-01	rs1800588	15_58431476_C_T	0.99	6709	1.73E-06	GCST90277322
Phosphatidylcholine (O-16:0_20:3) levels	16	15036737	G	A	-1.54E-01	1.83E-02	3.34E-01	rs6498540	16_15036737_A_G	0.99	6709	5.73E-17	GCST90277322
Phosphatidylcholine (O-16:0_20:3) levels	19	44908822	T	C	2.25E-01	3.82E-02	5.31E-02	rs7412	19_44908822_C_T	1.00	6709	4.52E-09	GCST90277322
Phosphatidylcholine (O-16:0_20:3) levels	20	57822739	C	T	7.82E-02	1.77E-02	4.21E-01	rs6015087	20_57822739_T_C	0.98	6709	9.71E-06	GCST90277322
Phosphatidylcholine (O-16:0_20:4) levels	1	19722845	A	G	-4.98E-01	1.06E-01	7.35E-03	rs77277881	1_19722845_G_A	0.86	7174	2.89E-06	GCST90277323
Phosphatidylcholine (O-16:0_20:4) levels	1	161642443	C	T	1.86E-01	3.91E-02	5.04E-02	rs7554873	1_161642443_T_C	0.94	7174	2.13E-06	GCST90277323
Phosphatidylcholine (O-16:0_20:4) levels	1	245444784	T	C	-2.22E-01	5.00E-02	2.98E-02	rs115076819	1_245444784_C_T	0.96	7174	9.02E-06	GCST90277323
Phosphatidylcholine (O-16:0_20:4) levels	2	123584690	A	G	9.47E-02	1.71E-02	4.16E-01	rs6709442	2_123584690_G_A	1.00	7174	3.00E-08	GCST90277323
Phosphatidylcholine (O-16:0_20:4) levels	2	180057085	C	T	-8.13E-02	1.67E-02	4.68E-01	rs2678932	2_180057085_T_C	1.00	7174	1.08E-06	GCST90277323
Phosphatidylcholine (O-16:0_20:4) levels	2	205689820	A	G	8.18E-02	1.81E-02	6.86E-01	rs698909	2_205689820_G_A	1.00	7174	6.00E-06	GCST90277323
Phosphatidylcholine (O-16:0_20:4) levels	4	42283844	G	A	2.78E-01	6.19E-02	2.07E-02	rs114653320	4_42283844_A_G	0.91	7174	7.51E-06	GCST90277323
Phosphatidylcholine (O-16:0_20:4) levels	4	66818143	G	A	-4.25E-01	9.59E-02	8.14E-03	rs114373179	4_66818143_A_G	0.94	7174	9.46E-06	GCST90277323
Phosphatidylcholine (O-16:0_20:4) levels	5	154465674	A	G	1.07E-01	1.98E-02	2.29E-01	rs34881711	5_154465674_G_A	0.99	7174	6.84E-08	GCST90277323
Phosphatidylcholine (O-16:0_20:4) levels	7	80837985	G	C	1.77E-01	3.97E-02	4.73E-02	rs10487877	7_80837985_C_G	0.98	7174	8.71E-06	GCST90277323
Phosphatidylcholine (O-16:0_20:4) levels	7	110642820	A	G	2.98E-01	6.47E-02	1.68E-02	rs76798309	7_110642820_G_A	0.98	7174	4.36E-06	GCST90277323
Phosphatidylcholine (O-16:0_20:4) levels	8	8390548	A	G	8.30E-02	1.78E-02	3.34E-01	rs6601694	8_8390548_G_A	0.99	7174	3.04E-06	GCST90277323
Phosphatidylcholine (O-16:0_20:4) levels	8	66145639	G	A	-9.52E-02	2.10E-02	2.02E-01	rs75385811	8_66145639_A_G	0.99	7174	5.77E-06	GCST90277323
Phosphatidylcholine (O-16:0_20:4) levels	9	21616162	G	A	-1.30E-01	2.71E-02	1.12E-01	rs73422593	9_21616162_A_G	0.97	7174	1.68E-06	GCST90277323
Phosphatidylcholine (O-16:0_20:4) levels	9	80921135	T	C	-7.80E-02	1.71E-02	6.09E-01	rs10867645	9_80921135_C_T	1.00	7174	5.22E-06	GCST90277323
Phosphatidylcholine (O-16:0_20:4) levels	9	113637327	A	C	8.98E-02	1.93E-02	7.27E-01	rs658580	9_113637327_C_A	0.94	7174	3.18E-06	GCST90277323
Phosphatidylcholine (O-16:0_20:4) levels	11	35494674	G	C	-7.65E-02	1.73E-02	3.94E-01	rs642327	11_35494674_C_G	0.98	7174	9.68E-06	GCST90277323
Phosphatidylcholine (O-16:0_20:4) levels	11	61744026	T	C	1.39E-01	2.46E-02	1.34E-01	rs3741252	11_61744026_C_T	0.99	7174	1.70E-08	GCST90277323
Phosphatidylcholine (O-16:0_20:4) levels	11	61826344	T	C	-3.82E-01	1.65E-02	4.05E-01	rs174568	11_61826344_C_T	1.00	7174	1.16E-114	GCST90277323
Phosphatidylcholine (O-16:0_20:4) levels	11	62267882	A	T	2.02E-01	3.95E-02	9.52E-01	rs2903910	11_62267882_T_A	0.99	7174	3.17E-07	GCST90277323
Phosphatidylcholine (O-16:0_20:4) levels	11	103100479	A	G	1.97E-01	4.41E-02	3.71E-02	rs146605342	11_103100479_G_A	0.98	7174	8.18E-06	GCST90277323
Phosphatidylcholine (O-16:0_20:4) levels	12	61209189	A	G	-1.60E-01	3.28E-02	6.88E-02	rs11173828	12_61209189_G_A	1.00	7174	1.17E-06	GCST90277323
Phosphatidylcholine (O-16:0_20:4) levels	12	124844767	C	T	-8.81E-02	1.68E-02	4.54E-01	rs11057853	12_124844767_T_C	0.99	7174	1.69E-07	GCST90277323
Phosphatidylcholine (O-16:0_20:4) levels	13	40337326	C	T	9.25E-02	2.01E-02	2.14E-01	rs7982529	13_40337326_T_C	1.00	7174	4.30E-06	GCST90277323

Phosphatidylcholine (O-16:0_20:4) levels	14	41287117	T	G	-6.35E-01	1.32E-01	4.44E-03	rs79995028	14_41287117_G_T	0.89	7174	1.62E-06	GCST90277323
Phosphatidylcholine (O-16:0_20:4) levels	16	7546177	G	A	-7.80E-02	1.75E-02	6.50E-01	rs12928189	16_7546177_A_G	1.00	7174	8.41E-06	GCST90277323
Phosphatidylcholine (O-16:0_20:4) levels	16	56954132	T	C	8.86E-02	1.85E-02	2.83E-01	rs173539	16_56954132_C_T	1.00	7174	1.63E-06	GCST90277323
Phosphatidylcholine (O-16:0_20:4) levels	17	59341291	G	C	1.84E-01	3.42E-02	6.46E-02	rs145755646	17_59341291_C_G	0.99	7174	7.23E-08	GCST90277323
Phosphatidylcholine (O-16:0_20:4) levels	18	27258198	C	T	-2.00E-01	4.29E-02	4.16E-02	rs117215929	18_27258198_T_C	0.98	7174	3.27E-06	GCST90277323
Phosphatidylcholine (O-16:0_20:4) levels	21	25391715	T	A	8.14E-02	1.81E-02	3.14E-01	rs2829725	21_25391715_A_T	1.00	7174	7.18E-06	GCST90277323
Phosphatidylcholine (O-16:0_22:5) levels	1	16964634	G	C	-1.23E-01	2.63E-02	1.44E-01	rs55709923	1_16964634_C_G	0.98	5988	3.10E-06	GCST90277324
Phosphatidylcholine (O-16:0_22:5) levels	3	68831568	T	G	3.40E-01	7.59E-02	1.66E-02	rs187264161	3_68831568_G_T	0.90	5988	7.55E-06	GCST90277324
Phosphatidylcholine (O-16:0_22:5) levels	4	27344507	A	G	-3.40E-01	7.45E-02	1.72E-02	rs80261753	4_27344507_G_A	0.90	5988	5.12E-06	GCST90277324
Phosphatidylcholine (O-16:0_22:5) levels	5	76222760	A	T	-2.51E-01	5.22E-02	3.13E-02	rs145866244	5_76222760_T_A	0.96	5988	1.60E-06	GCST90277324
Phosphatidylcholine (O-16:0_22:5) levels	6	45929408	A	C	-6.73E-01	1.43E-01	4.19E-03	rs140792636	6_45929408_C_A	0.94	5988	2.47E-06	GCST90277324
Phosphatidylcholine (O-16:0_22:5) levels	6	162315657	G	A	-1.21E-01	2.74E-02	1.27E-01	rs9458511	6_162315657_A_G	1.00	5988	9.41E-06	GCST90277324
Phosphatidylcholine (O-16:0_22:5) levels	9	5604987	T	C	1.86E-01	4.10E-02	5.37E-02	rs79318457	9_5604987_C_T	0.93	5988	5.82E-06	GCST90277324
Phosphatidylcholine (O-16:0_22:5) levels	10	131373745	T	C	-2.32E-01	5.07E-02	3.26E-02	rs150885037	10_131373745_C_T	0.99	5988	5.08E-06	GCST90277324
Phosphatidylcholine (O-16:0_22:5) levels	11	61803876	G	C	-1.68E-01	1.89E-02	3.84E-01	rs174548	11_61803876_C_G	1.00	5988	7.57E-19	GCST90277324
Phosphatidylcholine (O-16:0_22:5) levels	11	134357507	G	A	8.80E-02	1.92E-02	6.44E-01	rs7103356	11_134357507_A_G	0.98	5988	4.83E-06	GCST90277324
Phosphatidylcholine (O-16:0_22:5) levels	15	58387188	C	G	-8.67E-02	1.80E-02	4.92E-01	rs56358871	15_58387188_G_C	1.00	5988	1.53E-06	GCST90277324
Phosphatidylcholine (O-16:0_22:5) levels	16	83721684	A	T	-2.94E-01	6.61E-02	2.16E-02	rs142015595	16_83721684_T_A	0.96	5988	8.76E-06	GCST90277324
Phosphatidylcholine (O-16:0_22:5) levels	18	76150476	T	C	1.68E-01	3.78E-02	6.18E-02	rs79157443	18_76150476_C_T	0.97	5988	8.43E-06	GCST90277324
Phosphatidylcholine (O-16:0_22:5) levels	19	55193090	G	A	-1.07E-01	2.33E-02	1.95E-01	rs10414438	19_55193090_A_G	0.98	5988	4.98E-06	GCST90277324
Phosphatidylcholine (O-16:0_22:5) levels	20	2539461	G	A	-8.39E-02	1.83E-02	5.36E-01	rs4815298	20_2539461_A_G	0.99	5988	4.88E-06	GCST90277324
Phosphatidylcholine (O-16:0_22:5) levels	20	18282575	T	G	-5.75E-01	1.16E-01	6.49E-03	rs118036933	20_18282575_G_T	0.95	5988	6.95E-07	GCST90277324
Phosphatidylcholine (O-16:0_22:5) levels	22	43089231	G	C	-2.44E-01	5.15E-02	3.46E-02	rs184099437	22_43089231_C_G	0.93	5988	2.24E-06	GCST90277324
Phosphatidylcholine (O-16:1_16:0) levels	2	178137573	G	T	-2.41E-01	5.39E-02	3.01E-02	rs74843639	2_178137573_T_G	0.99	5868	8.02E-06	GCST90277325
Phosphatidylcholine (O-16:1_16:0) levels	3	178540293	C	T	-2.18E-01	4.73E-02	3.92E-02	rs79425159	3_178540293_T_C	0.99	5868	4.26E-06	GCST90277325
Phosphatidylcholine (O-16:1_16:0) levels	4	160356985	T	A	-1.24E-01	2.73E-02	1.30E-01	rs13104164	4_160356985_A_T	0.99	5868	5.35E-06	GCST90277325
Phosphatidylcholine (O-16:1_16:0) levels	5	144386603	G	A	1.02E-01	2.15E-02	7.51E-01	rs1023679	5_144386603_A_G	1.00	5868	2.25E-06	GCST90277325
Phosphatidylcholine (O-16:1_16:0) levels	6	20072349	T	C	-8.87E-02	1.98E-02	6.68E-01	rs670210	6_20072349_C_T	0.97	5868	7.72E-06	GCST90277325
Phosphatidylcholine (O-16:1_16:0) levels	7	39384981	C	G	1.04E-01	2.29E-02	2.02E-01	rs73125516	7_39384981_G_C	1.00	5868	5.05E-06	GCST90277325
Phosphatidylcholine (O-16:1_16:0) levels	9	24151529	A	G	1.85E-01	4.10E-02	5.58E-02	rs75066420	9_24151529_G_A	0.95	5868	6.65E-06	GCST90277325
Phosphatidylcholine (O-16:1_16:0) levels	9	104899461	C	A	-1.08E-01	2.38E-02	1.85E-01	rs2740488	9_104899461_A_C	1.00	5868	5.18E-06	GCST90277325
Phosphatidylcholine (O-16:1_16:0) levels	10	76243822	G	T	9.35E-02	1.85E-02	5.49E-01	rs1247489	10_76243822_T_G	1.00	5868	4.76E-07	GCST90277325
Phosphatidylcholine (O-16:1_16:0) levels	10	133100099	T	C	2.72E-01	6.04E-02	2.54E-02	rs34160235	10_133100099_C_T	0.95	5868	6.76E-06	GCST90277325
Phosphatidylcholine (O-16:1_16:0) levels	12	69475924	T	C	2.17E-01	4.55E-02	4.09E-02	rs79367190	12_69475924_C_T	0.98	5868	1.85E-06	GCST90277325
Phosphatidylcholine (O-16:1_16:0) levels	12	95184329	A	G	4.65E-01	9.30E-02	1.03E-02	rs78682716	12_95184329_G_A	0.99	5868	5.82E-07	GCST90277325
Phosphatidylcholine (O-16:1_16:0) levels	13	36635371	C	T	3.06E-01	6.78E-02	2.10E-02	rs77351278	13_36635371_T_C	0.92	5868	6.71E-06	GCST90277325
Phosphatidylcholine (O-16:1_16:0) levels	14	67509105	C	A	-1.83E-01	1.83E-02	4.68E-01	rs1077989	14_67509105_A_C	1.00	5868	2.91E-23	GCST90277325
Phosphatidylcholine (O-16:1_16:0) levels	14	70230276	C	G	-2.00E-01	4.35E-02	5.29E-02	rs71423392	14_70230276_G_C	0.91	5868	4.18E-06	GCST90277325
Phosphatidylcholine (O-16:1_16:0) levels	15	40795879	A	G	4.23E-01	9.32E-02	1.06E-02	rs76014869	15_40795879_G_A	0.98	5868	5.88E-06	GCST90277325
Phosphatidylcholine (O-16:1_16:0) levels	15	88324326	C	A	-9.70E-02	2.12E-02	3.04E-01	rs11858902	15_88324326_A_C	0.89	5868	4.76E-06	GCST90277325
Phosphatidylcholine (O-16:1_16:0) levels	18	9003268	C	T	-1.07E-01	2.34E-02	1.95E-01	rs72942772	18_9003268_T_C	0.99	5868	5.25E-06	GCST90277325
Phosphatidylcholine (O-16:1_18:0) levels	3	150051286	G	A	-5.07E-01	1.14E-01	6.32E-03	rs115012576	3_150051286_A_G	0.95	6751	8.66E-06	GCST90277326
Phosphatidylcholine (O-16:1_18:0) levels	4	141036586	C	T	-1.77E-01	3.78E-02	5.60E-02	rs9997698	4_141036586_T_C	0.98	6751	2.73E-06	GCST90277326
Phosphatidylcholine (O-16:1_18:0) levels	6	109308440	C	T	-1.31E-01	2.88E-02	9.72E-02	rs74997551	6_109308440_T_C	1.00	6751	5.75E-06	GCST90277326
Phosphatidylcholine (O-16:1_18:0) levels	8	26457702	T	C	-4.02E-01	9.08E-02	9.60E-03	rs143594930	8_26457702_C_T	0.97	6751	9.56E-06	GCST90277326
Phosphatidylcholine (O-16:1_18:0) levels	8	77676794	G	T	-7.84E-02	1.74E-02	4.11E-01	rs1397106	8_77676794_T_G	1.00	6751	6.65E-06	GCST90277326
Phosphatidylcholine (O-16:1_18:0) levels	10	80809816	T	C	-4.52E-01	9.45E-02	8.80E-03	rs11187130	10_80809816_C_T	0.97	6751	1.78E-06	GCST90277326
Phosphatidylcholine (O-16:1_18:0) levels	10	94349017	C	T	-1.47E-01	2.92E-02	9.80E-02	rs1093316	10_94349017_T_C	0.99	6751	4.68E-07	GCST90277326
Phosphatidylcholine (O-16:1_18:0) levels	11	3204472	A	G	-8.65E-02	1.94E-02	2.59E-01	rs4565878	11_3204472_G_A	1.00	6751	8.27E-06	GCST90277326
Phosphatidylcholine (O-16:1_18:0) levels	11	36410769	A	G	-1.23E-01	2.57E-02	1.39E-01	rs12270739	11_36410769_G_A	0.94	6751	1.60E-06	GCST90277326
Phosphatidylcholine (O-16:1_18:0) levels	11	66971292	T	C	-1.34E-01	2.59E-02	1.30E-01	rs77512406	11_66971292_C_T	0.99	6751	2.65E-07	GCST90277326
Phosphatidylcholine (O-16:1_18:0) levels	12	69626170	T	C	2.21E-01	4.92E-02	3.20E-02	rs181154147	12_69626170_C_T	0.98	6751	7.02E-06	GCST90277326
Phosphatidylcholine (O-16:1_18:0) levels	13	102563170	A	G	4.39E-01	9.84E-02	8.06E-03	rs116883951	13_102563170_G_A	0.96	6751	8.38E-06	GCST90277326
Phosphatidylcholine (O-16:1_18:0) levels	14	48425602	A	G	7.95E-02	1.79E-02	6.30E-01	rs1440391	14_48425602_G_A	1.00	6751	9.17E-06	GCST90277326
Phosphatidylcholine (O-16:1_18:0) levels	14	85227560	A	G	-8.73E-02	1.87E-02	7.01E-01	rs1667515	14_85227560_G_A	1.00	6751	3.27E-06	GCST90277326
Phosphatidylcholine (O-16:1_18:0) levels	17	53585624	C	T	8.10E-02	1.77E-02	5.95E-01	rs1355105	17_53585624_T_C	0.98	6751	4.95E-06	GCST90277326
Phosphatidylcholine (O-16:1_18:0) levels	18	41193890	G	A	-8.57E-02	1.90E-02	2.82E-01	rs1941562	18_41193890_A_G	1.00	6751	6.51E-06	GCST90277326

Phosphatidylcholine (O-16:1_18:0) levels	22	22014247	C	T	1.72E-01	3.45E-02	8.17E-02	rs448313	22_22014247_T_C	0.84	6751	6.67E-07	GCST90277326
Phosphatidylcholine (O-16:1_18:1) levels	1	230167766	T	C	1.19E-01	1.89E-02	7.42E-01	rs6672758	1_230167766_C_T	1.00	7154	3.78E-10	GCST90277327
Phosphatidylcholine (O-16:1_18:1) levels	2	181590050	T	G	-1.26E-01	2.57E-02	1.20E-01	rs1441162	2_181590050_G_T	1.00	7154	8.95E-07	GCST90277327
Phosphatidylcholine (O-16:1_18:1) levels	3	30168901	A	G	-1.26E-01	2.84E-02	9.62E-02	rs116614673	3_30168901_G_A	0.99	7154	9.54E-06	GCST90277327
Phosphatidylcholine (O-16:1_18:1) levels	3	73177331	G	A	-2.04E-01	4.47E-02	3.82E-02	rs150860836	3_73177331_A_G	0.95	7154	5.22E-06	GCST90277327
Phosphatidylcholine (O-16:1_18:1) levels	4	137022381	G	C	3.28E-01	7.05E-02	1.43E-02	rs7677028	4_137022381_C_G	0.97	7154	3.28E-06	GCST90277327
Phosphatidylcholine (O-16:1_18:1) levels	4	150864359	A	T	-1.79E-01	3.62E-02	5.75E-02	rs116438463	4_150864359_T_A	0.98	7154	7.28E-07	GCST90277327
Phosphatidylcholine (O-16:1_18:1) levels	7	44312910	T	G	-4.28E-01	9.47E-02	8.31E-03	rs117331519	7_44312910_G_T	0.97	7154	6.37E-06	GCST90277327
Phosphatidylcholine (O-16:1_18:1) levels	7	104770675	G	A	-1.05E-01	2.33E-02	1.50E-01	rs35293298	7_104770675_A_G	0.99	7154	7.35E-06	GCST90277327
Phosphatidylcholine (O-16:1_18:1) levels	7	137203256	C	T	1.09E-01	2.28E-02	1.64E-01	rs73162481	7_137203256_T_C	0.96	7154	1.64E-06	GCST90277327
Phosphatidylcholine (O-16:1_18:1) levels	9	70909853	T	A	-4.53E-01	1.01E-01	7.07E-03	rs74629446	9_70909853_A_T	0.96	7154	8.26E-06	GCST90277327
Phosphatidylcholine (O-16:1_18:1) levels	9	131776174	A	G	7.74E-02	1.70E-02	3.98E-01	rs3012735	9_131776174_G_A	1.00	7154	5.58E-06	GCST90277327
Phosphatidylcholine (O-16:1_18:1) levels	10	110932051	T	C	1.93E-01	4.29E-02	4.31E-02	rs34040476	10_110932051_C_T	0.92	7154	6.59E-06	GCST90277327
Phosphatidylcholine (O-16:1_18:1) levels	11	30952568	A	C	1.32E-01	2.66E-02	1.12E-01	rs158633	11_30952568_C_A	1.00	7154	6.58E-07	GCST90277327
Phosphatidylcholine (O-16:1_18:1) levels	11	116778201	C	G	1.53E-01	2.32E-02	8.49E-01	rs964184	11_116778201_G_C	1.00	7154	4.01E-11	GCST90277327
Phosphatidylcholine (O-16:1_18:1) levels	11	131747965	A	G	2.74E-01	5.57E-02	2.47E-02	rs117722620	11_131747965_G_A	0.92	7154	9.31E-07	GCST90277327
Phosphatidylcholine (O-16:1_18:1) levels	12	68489126	C	T	-1.56E-01	3.05E-02	8.28E-02	rs12426108	12_68489126_T_C	0.98	7154	3.18E-07	GCST90277327
Phosphatidylcholine (O-16:1_18:1) levels	12	129441623	G	A	2.50E-01	5.62E-02	2.26E-02	rs60426605	12_129441623_A_G	1.00	7154	8.77E-06	GCST90277327
Phosphatidylcholine (O-16:1_18:1) levels	14	45836784	T	G	-2.08E-01	4.61E-02	3.41E-02	rs116948429	14_45836784_G_T	0.98	7154	6.36E-06	GCST90277327
Phosphatidylcholine (O-16:1_18:1) levels	14	67507335	A	C	-1.60E-01	3.06E-02	8.22E-02	rs116943761	14_67507335_C_A	0.99	7154	1.74E-07	GCST90277327
Phosphatidylcholine (O-16:1_18:1) levels	14	95480964	C	A	-4.43E-01	8.96E-02	1.01E-02	rs143615441	14_95480964_A_C	0.85	7154	7.69E-07	GCST90277327
Phosphatidylcholine (O-16:1_18:1) levels	15	58391167	G	A	-9.51E-02	1.68E-02	5.73E-01	rs1532085	15_58391167_A_G	1.00	7154	1.60E-08	GCST90277327
Phosphatidylcholine (O-16:1_18:1) levels	15	58431476	T	C	8.88E-02	1.94E-02	2.58E-01	rs1800588	15_58431476_C_T	0.99	7154	4.79E-06	GCST90277327
Phosphatidylcholine (O-16:1_18:1) levels	16	56960616	T	C	9.44E-02	1.86E-02	2.78E-01	rs17231506	16_56960616_C_T	1.00	7154	3.84E-07	GCST90277327
Phosphatidylcholine (O-16:1_18:1) levels	16	76864528	C	A	-9.84E-02	2.17E-02	8.14E-01	rs7190500	16_76864528_A_C	0.99	7154	5.62E-06	GCST90277327
Phosphatidylcholine (O-16:1_18:1) levels	18	369878	G	A	4.86E-01	1.05E-01	7.16E-03	rs62087966	18_369878_A_G	0.88	7154	3.47E-06	GCST90277327
Phosphatidylcholine (O-16:1_18:2) levels	1	230174822	T	C	-8.59E-02	1.71E-02	3.96E-01	rs627108	1_230174822_C_T	0.99	7096	5.20E-07	GCST90277328
Phosphatidylcholine (O-16:1_18:2) levels	2	181779530	C	A	6.53E-01	1.29E-01	5.10E-03	rs114517755	2_181779530_A_C	0.87	7096	4.31E-07	GCST90277328
Phosphatidylcholine (O-16:1_18:2) levels	2	205953957	T	C	8.49E-02	1.76E-02	3.56E-01	rs13403377	2_205953957_C_T	0.97	7096	1.36E-06	GCST90277328
Phosphatidylcholine (O-16:1_18:2) levels	2	214577935	T	C	2.52E-01	5.36E-02	2.62E-02	rs143533648	2_214577935_C_T	0.95	7096	2.71E-06	GCST90277328
Phosphatidylcholine (O-16:1_18:2) levels	3	112816210	A	G	1.09E-01	2.30E-02	1.59E-01	rs11918522	3_112816210_G_A	1.00	7096	2.08E-06	GCST90277328
Phosphatidylcholine (O-16:1_18:2) levels	3	129984722	A	G	-2.34E-01	4.98E-02	3.03E-02	rs2670896	3_129984722_G_A	1.00	7096	2.76E-06	GCST90277328
Phosphatidylcholine (O-16:1_18:2) levels	4	25796122	C	A	-3.57E-01	7.92E-02	1.33E-02	rs138542536	4_25796122_A_C	0.86	7096	6.61E-06	GCST90277328
Phosphatidylcholine (O-16:1_18:2) levels	5	149246504	A	G	-3.44E-01	7.45E-02	1.28E-02	rs61999264	5_149246504_G_A	0.99	7096	3.86E-06	GCST90277328
Phosphatidylcholine (O-16:1_18:2) levels	6	30094133	A	G	1.65E-01	3.64E-02	5.90E-02	rs17194174	6_30094133_G_A	1.00	7096	5.74E-06	GCST90277328
Phosphatidylcholine (O-16:1_18:2) levels	8	1742309	C	G	1.44E-01	3.12E-02	9.18E-01	rs2977197	8_1742309_G_C	0.97	7096	3.75E-06	GCST90277328
Phosphatidylcholine (O-16:1_18:2) levels	8	74189562	C	G	2.66E-01	5.83E-02	2.13E-02	rs151236898	8_74189562_G_C	0.97	7096	5.16E-06	GCST90277328
Phosphatidylcholine (O-16:1_18:2) levels	10	4699577	A	G	-2.12E-01	4.60E-02	3.57E-02	rs76103105	10_4699577_G_A	0.96	7096	4.23E-06	GCST90277328
Phosphatidylcholine (O-16:1_18:2) levels	11	15423060	A	G	-1.26E-01	2.70E-02	8.90E-01	rs4237708	11_15423060_G_A	0.98	7096	3.02E-06	GCST90277328
Phosphatidylcholine (O-16:1_18:2) levels	11	34810889	T	C	8.46E-02	1.91E-02	2.63E-01	rs9666651	11_34810889_C_T	0.99	7096	9.86E-06	GCST90277328
Phosphatidylcholine (O-16:1_18:2) levels	11	116728272	G	A	8.60E-02	1.80E-02	3.24E-01	rs180360	11_116728272_A_G	1.00	7096	1.69E-06	GCST90277328
Phosphatidylcholine (O-16:1_18:2) levels	12	23079509	C	T	-1.37E-01	2.99E-02	8.98E-02	rs77172221	12_23079509_T_C	0.97	7096	4.53E-06	GCST90277328
Phosphatidylcholine (O-16:1_18:2) levels	12	46480289	A	G	5.11E-01	1.11E-01	6.25E-03	rs117002628	12_46480289_G_A	0.91	7096	4.41E-06	GCST90277328
Phosphatidylcholine (O-16:1_18:2) levels	12	68489126	C	T	-1.48E-01	3.07E-02	8.28E-02	rs12426108	12_68489126_T_C	0.98	7096	1.52E-06	GCST90277328
Phosphatidylcholine (O-16:1_18:2) levels	12	94142171	G	A	-8.06E-02	1.77E-02	6.05E-01	rs10859677	12_94142171_A_G	0.96	7096	5.27E-06	GCST90277328
Phosphatidylcholine (O-16:1_18:2) levels	12	124792520	A	G	-2.07E-01	4.61E-02	3.56E-02	rs186412131	12_124792520_G_A	0.95	7096	6.79E-06	GCST90277328
Phosphatidylcholine (O-16:1_18:2) levels	15	58391167	G	A	-1.15E-01	1.69E-02	5.73E-01	rs1532085	15_58391167_A_G	1.00	7096	8.88E-12	GCST90277328
Phosphatidylcholine (O-16:1_18:2) levels	15	58431280	C	T	1.00E-01	1.93E-02	2.66E-01	rs1077834	15_58431280_T_C	0.98	7096	2.10E-07	GCST90277328
Phosphatidylcholine (O-16:1_18:2) levels	16	56960616	T	C	1.28E-01	1.86E-02	2.78E-01	rs17231506	16_56960616_C_T	1.00	7096	5.42E-12	GCST90277328
Phosphatidylcholine (O-16:1_18:2) levels	17	50256987	A	G	-3.61E-01	8.15E-02	1.16E-02	rs139013784	17_50256987_G_A	0.94	7096	9.33E-06	GCST90277328
Phosphatidylcholine (O-16:1_18:2) levels	18	369878	G	A	4.99E-01	1.05E-01	7.16E-03	rs62087966	18_369878_A_G	0.88	7096	1.89E-06	GCST90277328
Phosphatidylcholine (O-16:1_18:2) levels	21	45199268	A	G	-5.80E-01	1.31E-01	4.67E-03	rs77099066	21_45199268_G_A	0.82	7096	9.25E-06	GCST90277328
Phosphatidylcholine (O-16:1_20:3) levels	1	35503298	G	A	9.27E-02	2.08E-02	2.40E-01	rs1203148	1_35503298_A_G	0.93	6770	8.17E-06	GCST90277329
Phosphatidylcholine (O-16:1_20:3) levels	1	60908464	A	G	8.25E-02	1.78E-02	4.80E-01	rs686843	1_60908464_G_A	0.94	6770	3.83E-06	GCST90277329
Phosphatidylcholine (O-16:1_20:3) levels	2	215392258	C	T	-2.20E-01	4.07E-02	4.86E-02	rs11883812	2_215392258_T_C	0.98	6770	6.86E-08	GCST90277329
Phosphatidylcholine (O-16:1_20:3) levels	3	87569181	A	G	9.88E-02	1.98E-02	7.49E-01	rs9872756	3_87569181_G_A	0.99	6770	6.28E-07	GCST90277329
Phosphatidylcholine (O-16:1_20:3) levels	3	188687875	C	T	-7.84E-02	1.77E-02	3.90E-01	rs9880183	3_188687875_T_C	0.99	6770	9.86E-06	GCST90277329

Phosphatidylcholine (O-16:1_20:3) levels	4	37858054 T	C	1.19E-01	2.47E-02	1.44E-01 rs3795169	4_37858054_C_T	0.99	6770	1.51E-06 GCST90277329
Phosphatidylcholine (O-16:1_20:3) levels	4	56204082 G	A	9.19E-02	1.76E-02	5.88E-01 rs6817929	4_56204082_A_G	1.00	6770	1.90E-07 GCST90277329
Phosphatidylcholine (O-16:1_20:3) levels	4	95119717 C	G	-1.34E-01	2.74E-02	8.91E-01 rs6815866	4_95119717_G_C	0.99	6770	1.03E-06 GCST90277329
Phosphatidylcholine (O-16:1_20:3) levels	7	74470522 G	A	1.34E-01	2.95E-02	9.51E-02 rs58105955	7_74470522_A_G	0.96	6770	6.06E-06 GCST90277329
Phosphatidylcholine (O-16:1_20:3) levels	7	112024749 G	T	-1.02E-01	2.29E-02	1.72E-01 rs254868	7_112024749_T_G	0.98	6770	8.91E-06 GCST90277329
Phosphatidylcholine (O-16:1_20:3) levels	7	130154739 A	G	-8.73E-02	1.88E-02	3.13E-01 rs34455876	7_130154739_G_A	0.98	6770	3.40E-06 GCST90277329
Phosphatidylcholine (O-16:1_20:3) levels	9	16476973 A	G	9.93E-02	2.17E-02	1.90E-01 rs7874842	9_16476973_G_A	1.00	6770	5.08E-06 GCST90277329
Phosphatidylcholine (O-16:1_20:3) levels	11	61788567 C	A	2.20E-01	2.69E-02	1.13E-01 rs61896141	11_61788567_A_C	1.00	6770	3.61E-16 GCST90277329
Phosphatidylcholine (O-16:1_20:3) levels	12	7036085 T	C	1.52E-01	3.16E-02	7.98E-02 rs55665216	12_7036085_C_T	0.99	6770	1.59E-06 GCST90277329
Phosphatidylcholine (O-16:1_20:3) levels	12	28686870 T	C	-2.26E-01	4.67E-02	3.57E-02 rs35556451	12_28686870_C_T	1.00	6770	1.28E-06 GCST90277329
Phosphatidylcholine (O-16:1_20:3) levels	12	61219406 A	G	-1.79E-01	3.87E-02	5.19E-02 rs80320850	12_61219406_G_A	1.00	6770	3.99E-06 GCST90277329
Phosphatidylcholine (O-16:1_20:3) levels	13	27037828 G	T	7.59E-01	1.70E-01	2.93E-03 rs112516243	13_27037828_T_G	0.92	6770	8.27E-06 GCST90277329
Phosphatidylcholine (O-16:1_20:3) levels	13	85434789 G	T	1.14E-01	2.45E-02	1.47E-01 rs9602749	13_85434789_T_G	0.99	6770	3.30E-06 GCST90277329
Phosphatidylcholine (O-16:1_20:3) levels	14	56431229 T	C	-3.49E-01	7.60E-02	1.39E-02 rs56142013	14_56431229_C_T	0.92	6770	4.31E-06 GCST90277329
Phosphatidylcholine (O-16:1_20:3) levels	14	67509105 C	A	-1.21E-01	1.72E-02	4.68E-01 rs1077989	14_67509105_A_C	1.00	6770	1.70E-12 GCST90277329
Phosphatidylcholine (O-16:1_20:3) levels	15	58432643 A	G	8.93E-02	1.86E-02	3.37E-01 rs8033940	15_58432643_G_A	0.99	6770	1.62E-06 GCST90277329
Phosphatidylcholine (O-16:1_20:3) levels	16	15036737 G	A	-1.47E-01	1.83E-02	3.34E-01 rs6498540	16_15036737_A_G	0.99	6770	8.85E-16 GCST90277329
Phosphatidylcholine (O-16:1_20:3) levels	17	15541920 T	C	2.59E-01	5.54E-02	2.45E-02 rs144750125	17_15541920_C_T	0.99	6770	3.17E-06 GCST90277329
Phosphatidylcholine (O-16:1_20:3) levels	19	44821499 G	C	-2.14E-01	4.62E-02	3.76E-02 rs28399664	19_44821499_C_G	0.95	6770	3.68E-06 GCST90277329
Phosphatidylcholine (O-16:1_20:3) levels	20	44413724 T	C	-1.74E-01	3.85E-02	5.24E-02 rs1800961	20_44413724_C_T	1.00	6770	6.40E-06 GCST90277329
Phosphatidylcholine (O-16:1_20:4) levels	1	107442367 T	C	-4.58E-01	9.09E-02	1.07E-02 rs114325590	1_107442367_C_T	0.89	6025	4.70E-07 GCST90277330
Phosphatidylcholine (O-16:1_20:4) levels	2	56518805 G	A	-1.26E-01	2.82E-02	1.21E-01 rs17268903	2_56518805_A_G	0.97	6025	8.32E-06 GCST90277330
Phosphatidylcholine (O-16:1_20:4) levels	2	157798940 A	G	2.33E-01	5.09E-02	3.45E-02 rs56303140	2_157798940_G_A	0.94	6025	4.65E-06 GCST90277330
Phosphatidylcholine (O-16:1_20:4) levels	2	181604439 A	G	-1.29E-01	2.77E-02	1.20E-01 rs6752137	2_181604439_G_A	1.00	6025	3.46E-06 GCST90277330
Phosphatidylcholine (O-16:1_20:4) levels	2	217342965 A	G	-9.65E-02	2.15E-02	7.65E-01 rs5028238	2_217342965_G_A	0.99	6025	7.68E-06 GCST90277330
Phosphatidylcholine (O-16:1_20:4) levels	3	19181916 G	A	-2.99E-01	6.53E-02	2.15E-02 rs138018897	3_19181916_A_G	0.93	6025	4.74E-06 GCST90277330
Phosphatidylcholine (O-16:1_20:4) levels	3	153579259 A	C	8.16E-02	1.83E-02	5.34E-01 rs6776300	3_153579259_C_A	1.00	6025	8.20E-06 GCST90277330
Phosphatidylcholine (O-16:1_20:4) levels	7	80837985 G	C	2.09E-01	4.32E-02	4.73E-02 rs10487877	7_80837985_C_G	0.98	6025	1.29E-06 GCST90277330
Phosphatidylcholine (O-16:1_20:4) levels	8	9367590 G	C	-3.94E-01	8.73E-02	1.17E-02 rs74854572	8_9367590_C_G	0.90	6025	6.39E-06 GCST90277330
Phosphatidylcholine (O-16:1_20:4) levels	11	61770929 C	G	-1.21E-01	1.86E-02	4.44E-01 rs174527	11_61770929_G_C	0.98	6025	7.84E-11 GCST90277330
Phosphatidylcholine (O-16:1_20:4) levels	11	61783884 C	T	-4.04E-01	1.79E-02	4.09E-01 rs174535	11_61783884_T_C	1.00	6025	1.18E-107 GCST90277330
Phosphatidylcholine (O-16:1_20:4) levels	11	62058058 A	G	-1.34E-01	2.67E-02	1.37E-01 rs2015950	11_62058058_G_A	1.00	6025	5.32E-07 GCST90277330
Phosphatidylcholine (O-16:1_20:4) levels	11	116728272 G	A	8.81E-02	1.95E-02	3.24E-01 rs180360	11_116728272_A_G	1.00	6025	6.34E-06 GCST90277330
Phosphatidylcholine (O-16:1_20:4) levels	12	6976009 A	G	1.60E-01	3.28E-02	8.21E-02 rs12579775	12_6976009_G_A	0.99	6025	1.05E-06 GCST90277330
Phosphatidylcholine (O-16:1_20:4) levels	12	28633010 T	C	-2.22E-01	4.99E-02	3.57E-02 rs71452092	12_28633010_C_T	1.00	6025	8.89E-06 GCST90277330
Phosphatidylcholine (O-16:1_20:4) levels	12	124935624 T	G	-1.93E-01	4.26E-02	4.95E-02 rs7963111	12_124935624_G_T	0.93	6025	5.86E-06 GCST90277330
Phosphatidylcholine (O-16:1_20:4) levels	14	67471921 A	G	-9.00E-02	2.02E-02	2.99E-01 rs1316133	14_67471921_G_A	0.97	6025	8.71E-06 GCST90277330
Phosphatidylcholine (O-16:1_20:4) levels	14	67509105 C	A	-1.92E-01	1.81E-02	4.68E-01 rs1077989	14_67509105_A_C	1.00	6025	3.43E-26 GCST90277330
Phosphatidylcholine (O-16:1_20:4) levels	15	29614744 C	T	-1.73E-01	3.79E-02	6.11E-02 rs1037782	15_29614744_T_C	0.99	6025	5.26E-06 GCST90277330
Phosphatidylcholine (O-16:1_20:4) levels	15	58388755 C	T	-9.99E-02	1.83E-02	5.76E-01 rs2043085	15_58388755_T_C	1.00	6025	4.75E-08 GCST90277330
Phosphatidylcholine (O-16:1_20:4) levels	16	56959113 T	G	-1.04E-01	1.95E-02	3.30E-01 rs12920974	16_56959113_G_T	0.99	6025	1.16E-07 GCST90277330
Phosphatidylcholine (O-16:1_20:4) levels	17	15393870 G	A	1.18E-01	2.63E-02	1.48E-01 rs71366199	17_15393870_A_G	0.95	6025	6.50E-06 GCST90277330
Phosphatidylcholine (O-16:1_20:4) levels	19	7570522 A	G	1.05E-01	2.35E-02	1.99E-01 rs2431867	19_7570522_G_A	0.94	6025	7.79E-06 GCST90277330
Phosphatidylcholine (O-16:1_20:4) levels	19	18613744 T	C	-9.07E-02	1.98E-02	2.89E-01 rs73531625	19_18613744_C_T	0.99	6025	4.83E-06 GCST90277330
Phosphatidylcholine (O-16:1_20:4) levels	20	1463662 A	G	1.97E-01	4.29E-02	4.83E-02 rs79740876	20_1463662_G_A	0.97	6025	4.27E-06 GCST90277330
Phosphatidylcholine (O-16:2_18:0) levels	1	82430121 A	G	3.92E-01	8.80E-02	1.02E-02 rs141656454	1_82430121_G_A	0.93	6672	8.63E-06 GCST90277331
Phosphatidylcholine (O-16:2_18:0) levels	3	4383597 T	A	-9.39E-02	1.87E-02	3.29E-01 rs13433708	3_4383597_A_T	0.98	6672	5.61E-07 GCST90277331
Phosphatidylcholine (O-16:2_18:0) levels	5	158277324 G	A	-1.36E-01	2.79E-02	1.16E-01 rs1145605	5_158277324_A_G	0.95	6672	1.18E-06 GCST90277331
Phosphatidylcholine (O-16:2_18:0) levels	6	24525754 A	G	8.36E-01	1.76E-01	2.56E-03 rs184729252	6_24525754_G_A	0.88	6672	2.14E-06 GCST90277331
Phosphatidylcholine (O-16:2_18:0) levels	6	167990307 C	G	2.99E-01	6.51E-02	1.81E-02 rs6455474	6_167990307_G_C	0.97	6672	4.54E-06 GCST90277331
Phosphatidylcholine (O-16:2_18:0) levels	8	76625718 A	G	-1.43E-01	3.11E-02	8.76E-02 rs61703937	8_76625718_G_A	1.00	6672	4.26E-06 GCST90277331
Phosphatidylcholine (O-16:2_18:0) levels	9	68622132 G	A	8.19E-02	1.84E-02	3.42E-01 rs10869152	9_68622132_A_G	0.99	6672	8.30E-06 GCST90277331
Phosphatidylcholine (O-16:2_18:0) levels	9	81637892 T	C	-1.51E-01	3.15E-02	9.15E-01 rs3814533	9_81637892_C_T	0.96	6672	1.77E-06 GCST90277331
Phosphatidylcholine (O-16:2_18:0) levels	9	131354630 G	A	8.49E-02	1.84E-02	3.48E-01 rs35807440	9_131354630_A_G	0.98	6672	4.04E-06 GCST90277331
Phosphatidylcholine (O-16:2_18:0) levels	12	118561421 G	A	1.00E-01	2.21E-02	1.92E-01 rs74684016	12_118561421_A_G	0.99	6672	5.96E-06 GCST90277331
Phosphatidylcholine (O-16:2_18:0) levels	13	41576909 A	G	9.31E-02	2.07E-02	2.38E-01 rs55816615	13_41576909_G_A	0.97	6672	7.22E-06 GCST90277331
Phosphatidylcholine (O-16:2_18:0) levels	15	58391167 G	A	-1.74E-01	1.73E-02	5.73E-01 rs1532085	15_58391167_A_G	1.00	6672	1.26E-23 GCST90277331

Phosphatidylcholine (O-16:2_18:0) levels	15	58438299 T	C	-1.91E-01	2.02E-02	7.58E-01 rs588136	15_58438299_C_T	1.00	6672	4.89E-21 GCST90277331
Phosphatidylcholine (O-17:0_15:0) levels	1	30929301 C	T	-9.71E-02	1.96E-02	7.00E-01 rs4949186	1_30929301_T_C	0.99	6140	7.41E-07 GCST90277332
Phosphatidylcholine (O-17:0_15:0) levels	1	62121729 A	G	3.37E-01	6.89E-02	1.73E-02 rs145150348	1_62121729_G_A	0.96	6140	1.05E-06 GCST90277332
Phosphatidylcholine (O-17:0_15:0) levels	2	158921003 T	C	5.92E-01	1.22E-01	5.92E-03 rs144941484	2_158921003_C_T	0.91	6140	1.28E-06 GCST90277332
Phosphatidylcholine (O-17:0_15:0) levels	2	197373815 C	T	-2.82E-01	6.21E-02	2.37E-02 rs149897605	2_197373815_T_C	0.93	6140	5.62E-06 GCST90277332
Phosphatidylcholine (O-17:0_15:0) levels	2	238794542 T	C	-5.04E-01	1.10E-01	7.80E-03 rs150669352	2_238794542_C_T	0.84	6140	4.34E-06 GCST90277332
Phosphatidylcholine (O-17:0_15:0) levels	3	104655275 T	G	-1.71E-01	3.78E-02	6.13E-02 rs76913081	3_104655275_G_T	0.98	6140	6.58E-06 GCST90277332
Phosphatidylcholine (O-17:0_15:0) levels	3	173729514 T	A	-3.28E-01	7.17E-02	2.20E-02 rs74648736	3_173729514_A_T	0.81	6140	4.79E-06 GCST90277332
Phosphatidylcholine (O-17:0_15:0) levels	6	23380540 G	A	-8.39E-02	1.87E-02	6.08E-01 rs748301	6_23380540_A_G	0.96	6140	7.53E-06 GCST90277332
Phosphatidylcholine (O-17:0_15:0) levels	7	17648098 T	G	-2.38E-01	5.38E-02	2.96E-02 rs10225730	7_17648098_G_T	0.99	6140	9.76E-06 GCST90277332
Phosphatidylcholine (O-17:0_15:0) levels	7	41274867 T	C	-8.79E-02	1.88E-02	4.40E-01 rs417916	7_41274867_C_T	0.93	6140	3.09E-06 GCST90277332
Phosphatidylcholine (O-17:0_15:0) levels	7	81169417 T	C	2.85E-01	6.00E-02	2.61E-02 rs146967253	7_81169417_C_T	0.91	6140	2.05E-06 GCST90277332
Phosphatidylcholine (O-17:0_15:0) levels	11	95940105 T	A	-1.80E-01	3.86E-02	6.01E-02 rs72967793	11_95940105_A_T	0.97	6140	2.99E-06 GCST90277332
Phosphatidylcholine (O-17:0_15:0) levels	11	114838676 G	T	2.66E-01	5.97E-02	2.38E-02 rs141417792	11_114838676_T_G	0.97	6140	8.26E-06 GCST90277332
Phosphatidylcholine (O-17:0_15:0) levels	12	42408584 C	A	-2.61E-01	5.73E-02	2.71E-02 rs76292244	12_42408584_A_C	0.96	6140	5.52E-06 GCST90277332
Phosphatidylcholine (O-17:0_15:0) levels	13	42902737 G	A	4.70E-01	9.89E-02	9.38E-03 rs112686420	13_42902737_A_G	0.91	6140	2.02E-06 GCST90277332
Phosphatidylcholine (O-17:0_15:0) levels	16	29082111 G	A	8.17E-02	1.82E-02	4.24E-01 rs252277	16_29082111_A_G	0.99	6140	7.53E-06 GCST90277332
Phosphatidylcholine (O-17:0_15:0) levels	16	48346132 G	A	2.43E-01	5.49E-02	2.75E-02 rs116984749	16_48346132_A_G	0.98	6140	9.83E-06 GCST90277332
Phosphatidylcholine (O-17:0_15:0) levels	16	86938606 G	A	2.38E-01	5.26E-02	3.16E-02 rs7204112	16_86938606_A_G	0.96	6140	6.35E-06 GCST90277332
Phosphatidylcholine (O-17:0_15:0) levels	22	49966644 G	T	7.39E-01	1.60E-01	4.25E-03 rs139939351	22_49966644_T_G	0.86	6140	3.64E-06 GCST90277332
Phosphatidylcholine (O-17:0_17:1) levels	1	109494012 T	C	1.46E-01	3.28E-02	7.20E-02 rs115823613	1_109494012_C_T	1.00	6880	8.99E-06 GCST90277333
Phosphatidylcholine (O-17:0_17:1) levels	1	230188618 C	T	-1.14E-01	2.20E-02	1.80E-01 rs612577	1_230188618_T_C	1.00	6880	2.01E-07 GCST90277333
Phosphatidylcholine (O-17:0_17:1) levels	2	100856837 C	G	-1.83E-01	3.99E-02	4.73E-02 rs11691441	2_100856837_G_C	0.98	6880	4.67E-06 GCST90277333
Phosphatidylcholine (O-17:0_17:1) levels	2	113675313 G	T	-8.24E-02	1.80E-02	6.62E-01 rs7575011	2_113675313_T_G	1.00	6880	4.63E-06 GCST90277333
Phosphatidylcholine (O-17:0_17:1) levels	2	192167629 C	T	9.04E-02	2.02E-02	7.65E-01 rs7597998	2_192167629_T_C	0.98	6880	7.99E-06 GCST90277333
Phosphatidylcholine (O-17:0_17:1) levels	3	135812971 T	A	9.47E-02	2.08E-02	2.15E-01 rs72975671	3_135812971_A_T	0.99	6880	5.24E-06 GCST90277333
Phosphatidylcholine (O-17:0_17:1) levels	4	6203208 C	T	9.28E-02	2.03E-02	7.37E-01 rs11947730	4_6203208_T_C	0.92	6880	5.19E-06 GCST90277333
Phosphatidylcholine (O-17:0_17:1) levels	4	168219402 C	T	-9.98E-02	2.00E-02	2.41E-01 rs17611110	4_168219402_T_C	1.00	6880	5.86E-07 GCST90277333
Phosphatidylcholine (O-17:0_17:1) levels	5	3950508 T	C	2.27E-01	4.67E-02	3.60E-02 rs12186559	5_3950508_C_T	0.95	6880	1.19E-06 GCST90277333
Phosphatidylcholine (O-17:0_17:1) levels	6	123161385 TA	T	9.74E-02	2.09E-02	7.82E-01 rs78686158	6_123161385_T_TA	1.00	6880	3.16E-06 GCST90277333
Phosphatidylcholine (O-17:0_17:1) levels	6	136669145 C	T	-7.91E-02	1.79E-02	4.27E-01 rs3765259	6_136669145_T_C	0.93	6880	9.73E-06 GCST90277333
Phosphatidylcholine (O-17:0_17:1) levels	6	151101373 G	A	3.59E-01	7.97E-02	1.23E-02 rs112832771	6_151101373_A_G	0.92	6880	6.61E-06 GCST90277333
Phosphatidylcholine (O-17:0_17:1) levels	6	158732893 C	T	1.04E-01	2.11E-02	2.12E-01 rs17513056	6_158732893_T_C	0.99	6880	8.11E-07 GCST90277333
Phosphatidylcholine (O-17:0_17:1) levels	7	18311602 G	A	9.68E-02	2.06E-02	2.29E-01 rs79648513	7_18311602_A_G	0.95	6880	2.69E-06 GCST90277333
Phosphatidylcholine (O-17:0_17:1) levels	7	118070482 C	G	1.40E-01	3.06E-02	9.14E-01 rs38820	7_118070482_G_C	1.00	6880	4.96E-06 GCST90277333
Phosphatidylcholine (O-17:0_17:1) levels	8	4601984 A	T	-2.55E-01	5.69E-02	2.43E-02 rs2725087	8_4601984_T_A	0.98	6880	7.80E-06 GCST90277333
Phosphatidylcholine (O-17:0_17:1) levels	8	22926841 T	C	8.16E-02	1.81E-02	3.44E-01 rs7819125	8_22926841_C_T	0.99	6880	6.93E-06 GCST90277333
Phosphatidylcholine (O-17:0_17:1) levels	8	97974998 C	T	2.90E-01	6.52E-02	1.83E-02 rs4517092	8_97974998_T_C	0.98	6880	9.06E-06 GCST90277333
Phosphatidylcholine (O-17:0_17:1) levels	9	16670164 C	G	-3.85E-01	8.65E-02	1.04E-02 rs28403152	9_16670164_G_C	0.93	6880	8.72E-06 GCST90277333
Phosphatidylcholine (O-17:0_17:1) levels	10	97150097 A	C	1.66E-01	3.68E-02	9.41E-01 rs2784923	10_97150097_C_A	1.00	6880	6.83E-06 GCST90277333
Phosphatidylcholine (O-17:0_17:1) levels	11	61855668 T	C	8.91E-02	1.73E-02	4.19E-01 rs174601	11_61855668_C_T	1.00	6880	2.59E-07 GCST90277333
Phosphatidylcholine (O-17:0_17:1) levels	11	116732961 A	G	9.05E-02	2.02E-02	2.36E-01 rs1893460	11_116732961_G_A	1.00	6880	7.94E-06 GCST90277333
Phosphatidylcholine (O-17:0_17:1) levels	12	16652538 C	G	2.40E-01	5.30E-02	2.73E-02 rs151297234	12_16652538_G_C	0.99	6880	6.18E-06 GCST90277333
Phosphatidylcholine (O-17:0_17:1) levels	12	116703206 A	G	-9.43E-02	2.06E-02	2.23E-01 rs11068152	12_116703206_G_A	0.99	6880	4.89E-06 GCST90277333
Phosphatidylcholine (O-17:0_17:1) levels	13	27479289 T	C	-2.35E-01	5.22E-02	2.73E-02 rs61644965	13_27479289_C_T	1.00	6880	6.56E-06 GCST90277333
Phosphatidylcholine (O-17:0_17:1) levels	13	93646525 T	C	-8.07E-02	1.81E-02	3.30E-01 rs9516264	13_93646525_C_T	1.00	6880	8.59E-06 GCST90277333
Phosphatidylcholine (O-17:0_17:1) levels	14	56937249 A	C	7.66E-02	1.72E-02	4.67E-01 rs709981	14_56937249_C_A	0.99	6880	8.76E-06 GCST90277333
Phosphatidylcholine (O-17:0_17:1) levels	15	58386313 T	C	8.67E-02	1.80E-02	3.38E-01 rs10468017	15_58386313_C_T	1.00	6880	1.43E-06 GCST90277333
Phosphatidylcholine (O-17:0_17:1) levels	16	56956804 A	C	1.20E-01	1.89E-02	2.78E-01 rs247617	16_56956804_C_A	1.00	6880	2.69E-10 GCST90277333
Phosphatidylcholine (O-17:0_17:1) levels	16	79069483 A	G	-3.35E-01	7.55E-02	1.49E-02 rs75206639	16_79069483_G_A	0.89	6880	9.02E-06 GCST90277333
Phosphatidylcholine (O-17:0_17:1) levels	17	5010850 T	G	-1.08E-01	2.19E-02	1.94E-01 rs62072490	17_5010850_G_T	0.95	6880	8.69E-07 GCST90277333
Phosphatidylcholine (O-17:0_17:1) levels	17	66571589 A	C	8.16E-02	1.78E-02	3.82E-01 rs7223101	17_66571589_C_A	1.00	6880	4.39E-06 GCST90277333
Phosphatidylcholine (O-17:0_17:1) levels	19	45759621 C	T	-4.64E-01	1.01E-01	7.21E-03 rs150269921	19_45759621_T_C	0.99	6880	4.15E-06 GCST90277333
Phosphatidylcholine (O-17:0_17:1) levels	21	42137794 A	G	1.22E-01	2.59E-02	1.25E-01 rs72495602	21_42137794_G_A	1.00	6880	2.57E-06 GCST90277333
Phosphatidylcholine (O-18:0_14:0) levels	2	11801863 G	A	-1.18E-01	2.65E-02	1.40E-01 rs7566488	2_11801863_A_G	0.93	6462	7.91E-06 GCST90277334
Phosphatidylcholine (O-18:0_14:0) levels	3	127976198 C	A	-2.71E-01	5.87E-02	2.45E-02 rs77444850	3_127976198_A_C	0.94	6462	3.83E-06 GCST90277334
Phosphatidylcholine (O-18:0_14:0) levels	4	121735493 C	A	-3.21E-01	7.16E-02	1.69E-02 rs60635506	4_121735493_A_C	0.94	6462	7.54E-06 GCST90277334

Phosphatidylcholine (O-18:0_14:0) levels	4	158808075	T	C	3.08E-01	6.89E-02	1.73E-02	rs116304326	4_158808075_C_T	0.95	6462	7.93E-06	GCST90277334
Phosphatidylcholine (O-18:0_14:0) levels	5	174236186	G	A	-8.93E-02	2.01E-02	7.33E-01	rs10516113	5_174236186_A_G	1.00	6462	8.99E-06	GCST90277334
Phosphatidylcholine (O-18:0_14:0) levels	6	161187390	C	G	-1.20E-01	2.39E-02	8.37E-01	rs9365258	6_161187390_G_C	0.99	6462	5.76E-07	GCST90277334
Phosphatidylcholine (O-18:0_14:0) levels	10	58404019	T	C	-4.42E-01	9.01E-02	9.98E-03	rs117246644	10_58404019_C_T	0.97	6462	9.60E-07	GCST90277334
Phosphatidylcholine (O-18:0_14:0) levels	10	86168949	A	C	1.42E-01	3.04E-02	1.02E-01	rs72835302	10_86168949_C_A	0.92	6462	3.09E-06	GCST90277334
Phosphatidylcholine (O-18:0_14:0) levels	14	95941109	C	G	-4.87E-01	1.07E-01	7.65E-03	rs79139024	14_95941109_G_C	0.90	6462	4.96E-06	GCST90277334
Phosphatidylcholine (O-18:0_14:0) levels	15	76487930	T	C	-3.03E-01	6.81E-02	1.65E-02	rs111316958	15_76487930_C_T	1.00	6462	8.95E-06	GCST90277334
Phosphatidylcholine (O-18:0_14:0) levels	16	78809113	T	G	3.12E-01	6.32E-02	2.00E-02	rs146726414	16_78809113_G_T	0.97	6462	8.16E-07	GCST90277334
Phosphatidylcholine (O-18:0_14:0) levels	17	851904	G	A	-2.00E-01	4.48E-02	4.16E-02	rs73975572	17_851904_A_G	0.95	6462	7.98E-06	GCST90277334
Phosphatidylcholine (O-18:0_14:0) levels	17	16925824	T	C	-1.69E-01	3.54E-02	6.65E-02	rs72637380	17_16925824_C_T	0.99	6462	1.84E-06	GCST90277334
Phosphatidylcholine (O-18:0_14:0) levels	17	81307290	A	G	7.06E-01	1.50E-01	4.22E-03	rs116907632	17_81307290_G_A	0.85	6462	2.58E-06	GCST90277334
Phosphatidylcholine (O-18:0_14:0) levels	19	7899091	C	T	9.82E-02	2.05E-02	2.43E-01	rs2115108	19_7899091_T_C	0.99	6462	1.73E-06	GCST90277334
Phosphatidylcholine (O-18:0_14:0) levels	20	17196017	T	C	-1.74E-01	3.88E-02	5.27E-02	rs35728084	20_17196017_C_T	1.00	6462	7.53E-06	GCST90277334
Phosphatidylcholine (O-18:0_14:0) levels	21	38363308	G	A	9.62E-02	2.04E-02	2.52E-01	rs8129049	21_38363308_A_G	0.99	6462	2.40E-06	GCST90277334
Phosphatidylcholine (O-18:0_16:1) levels	1	65877338	A	G	1.14E-01	2.56E-02	1.55E-01	rs79014169	1_65877338_G_A	1.00	5959	9.29E-06	GCST90277335
Phosphatidylcholine (O-18:0_16:1) levels	1	117639417	T	G	2.09E-01	4.56E-02	4.59E-02	rs55969585	1_117639417_G_T	0.96	5959	4.75E-06	GCST90277335
Phosphatidylcholine (O-18:0_16:1) levels	2	229375003	C	T	-1.88E-01	3.70E-02	6.48E-02	rs149012657	2_229375003_T_C	0.97	5959	3.82E-07	GCST90277335
Phosphatidylcholine (O-18:0_16:1) levels	5	124738074	A	G	1.26E-01	2.50E-02	1.66E-01	rs4547920	5_124738074_G_A	0.98	5959	4.26E-07	GCST90277335
Phosphatidylcholine (O-18:0_16:1) levels	7	49798317	T	C	8.75E-02	1.87E-02	3.92E-01	rs10243115	7_49798317_C_T	1.00	5959	3.01E-06	GCST90277335
Phosphatidylcholine (O-18:0_16:1) levels	10	33061164	A	G	1.43E-01	3.13E-02	9.84E-02	rs11009204	10_33061164_G_A	0.99	5959	4.56E-06	GCST90277335
Phosphatidylcholine (O-18:0_16:1) levels	10	100315722	A	G	-1.81E-01	2.91E-02	1.21E-01	rs603424	10_100315722_G_A	1.00	5959	5.21E-10	GCST90277335
Phosphatidylcholine (O-18:0_16:1) levels	10	100348766	G	A	2.19E-01	4.88E-02	3.70E-02	rs639060	10_100348766_A_G	0.97	5959	7.25E-06	GCST90277335
Phosphatidylcholine (O-18:0_16:1) levels	11	96017012	T	C	1.12E-01	2.30E-02	2.05E-01	rs12575589	11_96017012_C_T	0.99	5959	1.17E-06	GCST90277335
Phosphatidylcholine (O-18:0_16:1) levels	12	94924181	G	A	-8.40E-02	1.85E-02	4.94E-01	rs10859795	12_94924181_A_G	1.00	5959	5.50E-06	GCST90277335
Phosphatidylcholine (O-18:0_16:1) levels	13	27642024	A	G	-3.44E-01	7.23E-02	1.69E-02	rs74644051	13_27642024_G_A	0.97	5959	2.04E-06	GCST90277335
Phosphatidylcholine (O-18:0_16:1) levels	14	99061905	G	A	-8.38E-02	1.86E-02	5.38E-01	rs1257588	14_99061905_A_G	0.98	5959	6.86E-06	GCST90277335
Phosphatidylcholine (O-18:0_16:1) levels	15	97398226	A	G	-1.17E-01	2.64E-02	1.46E-01	rs12903358	15_97398226_G_A	0.98	5959	9.03E-06	GCST90277335
Phosphatidylcholine (O-18:0_16:1) levels	16	88778601	T	C	-8.96E-02	1.86E-02	3.89E-01	rs2911458	16_88778601_C_T	1.00	5959	1.54E-06	GCST90277335
Phosphatidylcholine (O-18:0_16:1) levels	20	48803368	G	A	8.52E-02	1.88E-02	3.98E-01	rs13044736	20_48803368_A_G	1.00	5959	5.96E-06	GCST90277335
Phosphatidylcholine (O-18:0_20:4) levels	1	164956995	C	A	-7.75E-02	1.71E-02	5.38E-01	rs34737685	1_164956995_A_C	1.00	6956	5.63E-06	GCST90277336
Phosphatidylcholine (O-18:0_20:4) levels	2	66991489	A	G	-5.76E-01	1.11E-01	6.16E-03	rs138202451	2_66991489_G_A	0.93	6956	2.32E-07	GCST90277336
Phosphatidylcholine (O-18:0_20:4) levels	2	130169813	G	A	-2.61E-01	5.60E-02	2.43E-02	rs148462060	2_130169813_A_G	0.97	6956	3.30E-06	GCST90277336
Phosphatidylcholine (O-18:0_20:4) levels	2	163582301	G	T	-1.25E-01	2.84E-02	1.02E-01	rs79856406	2_163582301_T_G	0.96	6956	9.96E-06	GCST90277336
Phosphatidylcholine (O-18:0_20:4) levels	3	16036318	T	G	-1.98E-01	4.46E-02	3.68E-02	rs148840717	3_16036318_G_T	1.00	6956	8.98E-06	GCST90277336
Phosphatidylcholine (O-18:0_20:4) levels	5	106783090	G	A	8.27E-02	1.83E-02	3.24E-01	rs11954170	5_106783090_A_G	1.00	6956	6.07E-06	GCST90277336
Phosphatidylcholine (O-18:0_20:4) levels	6	72738363	G	A	-9.79E-02	2.21E-02	1.91E-01	rs4706511	6_72738363_A_G	0.96	6956	9.96E-06	GCST90277336
Phosphatidylcholine (O-18:0_20:4) levels	7	64330243	A	G	1.49E-01	2.84E-02	1.02E-01	rs143233558	7_64330243_G_A	0.98	6956	1.55E-07	GCST90277336
Phosphatidylcholine (O-18:0_20:4) levels	8	18172468	A	C	-8.22E-02	1.73E-02	3.90E-01	rs6992951	8_18172468_C_A	0.99	6956	2.05E-06	GCST90277336
Phosphatidylcholine (O-18:0_20:4) levels	9	79743146	A	G	1.82E-01	3.97E-02	4.98E-02	rs78711160	9_79743146_G_A	0.96	6956	4.51E-06	GCST90277336
Phosphatidylcholine (O-18:0_20:4) levels	11	61744026	T	C	1.50E-01	2.50E-02	1.34E-01	rs3741252	11_61744026_C_T	0.99	6956	1.99E-09	GCST90277336
Phosphatidylcholine (O-18:0_20:4) levels	11	61784455	C	A	-3.08E-01	1.69E-02	4.08E-01	rs174536	11_61784455_A_C	1.00	6956	2.28E-72	GCST90277336
Phosphatidylcholine (O-18:0_20:4) levels	11	120590088	G	A	-3.06E-01	6.72E-02	1.73E-02	rs117870830	11_120590088_A_G	0.89	6956	5.47E-06	GCST90277336
Phosphatidylcholine (O-18:0_20:4) levels	12	2231632	C	A	8.58E-02	1.76E-02	3.65E-01	rs34382810	12_2231632_A_C	0.99	6956	1.12E-06	GCST90277336
Phosphatidylcholine (O-18:0_20:4) levels	12	21873844	T	C	2.51E-01	5.52E-02	2.44E-02	rs73074943	12_21873844_C_T	0.98	6956	5.54E-06	GCST90277336
Phosphatidylcholine (O-18:0_20:4) levels	12	124844767	C	T	-7.75E-02	1.71E-02	4.54E-01	rs11057853	12_124844767_T_C	0.99	6956	6.11E-06	GCST90277336
Phosphatidylcholine (O-18:0_20:4) levels	13	51261013	T	A	1.20E-01	2.65E-02	1.18E-01	rs9591400	13_51261013_A_T	0.97	6956	5.67E-06	GCST90277336
Phosphatidylcholine (O-18:0_20:4) levels	15	58431476	T	C	1.03E-01	1.97E-02	2.58E-01	rs1800588	15_58431476_C_T	0.99	6956	1.56E-07	GCST90277336
Phosphatidylcholine (O-18:0_20:4) levels	16	83381927	C	T	1.25E-01	2.70E-02	8.88E-01	rs4782783	16_83381927_T_C	0.99	6956	3.64E-06	GCST90277336
Phosphatidylcholine (O-18:0_20:4) levels	17	4978481	G	C	-7.54E-02	1.69E-02	4.73E-01	rs376516	17_4978481_C_G	1.00	6956	8.56E-06	GCST90277336
Phosphatidylcholine (O-18:0_20:4) levels	17	59341291	G	C	1.60E-01	3.47E-02	6.46E-02	rs145755646	17_59341291_C_G	0.99	6956	4.21E-06	GCST90277336
Phosphatidylcholine (O-18:0_20:4) levels	19	44908822	T	C	1.94E-01	3.78E-02	5.31E-02	rs7412	19_44908822_C_T	1.00	6956	2.93E-07	GCST90277336
Phosphatidylcholine (O-18:1_16:0) levels	1	230180443	G	A	-7.86E-02	1.75E-02	3.74E-01	rs606587	1_230180443_A_G	1.00	6956	7.17E-06	GCST90277337
Phosphatidylcholine (O-18:1_16:0) levels	2	27780307	T	G	-1.08E-01	2.17E-02	1.86E-01	rs13030345	2_27780307_G_T	0.99	6956	6.73E-07	GCST90277337
Phosphatidylcholine (O-18:1_16:0) levels	2	181582854	G	A	-1.03E-01	2.19E-02	1.80E-01	rs60331765	2_181582854_A_G	0.99	6956	2.80E-06	GCST90277337
Phosphatidylcholine (O-18:1_16:0) levels	2	200469214	A	G	2.96E-01	6.69E-02	1.65E-02	rs142774676	2_200469214_G_A	0.98	6956	9.42E-06	GCST90277337
Phosphatidylcholine (O-18:1_16:0) levels	3	4949091	C	T	-8.62E-02	1.73E-02	4.35E-01	rs9682490	3_4949091_T_C	0.98	6956	6.24E-07	GCST90277337
Phosphatidylcholine (O-18:1_16:0) levels	4	25796122	C	A	-3.87E-01	7.94E-02	1.33E-02	rs138542536	4_25796122_A_C	0.86	6956	1.14E-06	GCST90277337

Phosphatidylcholine (O-18:1_16:0) levels	4	110407918 A	C	-1.57E-01	3.55E-02	6.32E-02 rs189970593	4_110407918_C_A	0.95	6956	9.50E-06 GCST90277337
Phosphatidylcholine (O-18:1_16:0) levels	4	124795272 C	T	1.59E-01	3.35E-02	7.10E-02 rs72683831	4_124795272_T_C	0.99	6956	2.23E-06 GCST90277337
Phosphatidylcholine (O-18:1_16:0) levels	4	137022381 G	C	3.82E-01	7.22E-02	1.43E-02 rs7677028	4_137022381_C_G	0.97	6956	1.30E-07 GCST90277337
Phosphatidylcholine (O-18:1_16:0) levels	5	82667322 A	G	1.25E-01	2.84E-02	1.01E-01 rs56868865	5_82667322_G_A	0.96	6956	9.97E-06 GCST90277337
Phosphatidylcholine (O-18:1_16:0) levels	5	115795209 T	C	-9.77E-02	2.16E-02	8.07E-01 rs258736	5_115795209_C_T	0.99	6956	6.54E-06 GCST90277337
Phosphatidylcholine (O-18:1_16:0) levels	6	31450839 A	G	-1.14E-01	2.40E-02	1.44E-01 rs7450458	6_31450839_G_A	1.00	6956	1.99E-06 GCST90277337
Phosphatidylcholine (O-18:1_16:0) levels	6	34475746 C	T	-1.44E-01	3.10E-02	8.14E-02 rs57493997	6_34475746_T_C	0.98	6956	3.57E-06 GCST90277337
Phosphatidylcholine (O-18:1_16:0) levels	6	111551279 C	T	-1.03E-01	2.30E-02	8.39E-01 rs6568686	6_111551279_T_C	0.99	6956	7.96E-06 GCST90277337
Phosphatidylcholine (O-18:1_16:0) levels	6	167330921 C	T	8.68E-02	1.95E-02	2.59E-01 rs6908815	6_167330921_T_C	0.99	6956	8.67E-06 GCST90277337
Phosphatidylcholine (O-18:1_16:0) levels	8	31640951 A	G	9.34E-02	1.85E-02	2.99E-01 rs34861396	8_31640951_G_A	0.99	6956	4.51E-07 GCST90277337
Phosphatidylcholine (O-18:1_16:0) levels	11	13766154 T	C	9.83E-02	2.12E-02	1.97E-01 rs1503311	11_13766154_C_T	1.00	6956	3.43E-06 GCST90277337
Phosphatidylcholine (O-18:1_16:0) levels	12	124853790 C	T	1.35E-01	2.64E-02	1.17E-01 rs78194510	12_124853790_T_C	0.97	6956	3.32E-07 GCST90277337
Phosphatidylcholine (O-18:1_16:0) levels	13	27424632 C	T	8.78E-02	1.94E-02	7.42E-01 rs1049302	13_27424632_T_C	0.99	6956	5.84E-06 GCST90277337
Phosphatidylcholine (O-18:1_16:0) levels	13	40354480 A	C	1.17E-01	2.36E-02	1.50E-01 rs7327319	13_40354480_C_A	1.00	6956	7.43E-07 GCST90277337
Phosphatidylcholine (O-18:1_16:0) levels	13	60824670 A	G	1.48E-01	3.25E-02	7.59E-02 rs7330980	13_60824670_G_A	0.99	6956	4.90E-06 GCST90277337
Phosphatidylcholine (O-18:1_16:0) levels	15	58382109 A	G	9.66E-02	1.71E-02	4.11E-01 rs2043082	15_58382109_G_A	1.00	6956	1.61E-08 GCST90277337
Phosphatidylcholine (O-18:1_16:0) levels	15	58431476 T	C	1.23E-01	1.96E-02	2.58E-01 rs1800588	15_58431476_C_T	0.99	6956	4.58E-10 GCST90277337
Phosphatidylcholine (O-18:1_16:0) levels	16	760921 T	C	-2.30E-01	4.90E-02	3.34E-02 rs118138240	16_760921_C_T	0.91	6956	2.89E-06 GCST90277337
Phosphatidylcholine (O-18:1_16:0) levels	16	14276763 A	C	1.58E-01	3.43E-02	6.73E-02 rs246168	16_14276763_C_A	0.99	6956	4.05E-06 GCST90277337
Phosphatidylcholine (O-18:1_16:0) levels	16	56960616 T	C	1.35E-01	1.88E-02	2.78E-01 rs17231506	16_56960616_C_T	1.00	6956	7.74E-13 GCST90277337
Phosphatidylcholine (O-18:1_16:0) levels	16	85344145 T	C	2.92E-01	5.92E-02	2.30E-02 rs113746682	16_85344145_C_T	0.91	6956	8.63E-07 GCST90277337
Phosphatidylcholine (O-18:1_16:0) levels	19	11082239 G	A	-1.47E-01	2.93E-02	9.21E-02 rs73015021	19_11082239_A_G	0.98	6956	5.36E-07 GCST90277337
Phosphatidylcholine (O-18:1_16:0) levels	19	28504762 C	T	1.29E-01	2.74E-02	1.11E-01 rs79674727	19_28504762_T_C	0.97	6956	2.72E-06 GCST90277337
Phosphatidylcholine (O-18:1_16:0) levels	20	61244195 G	C	2.60E-01	5.61E-02	2.64E-02 rs187861964	20_61244195_C_G	0.90	6956	3.54E-06 GCST90277337
Phosphatidylinositol (18:0_20:3) levels	1	11749358 T	C	-2.02E-01	4.57E-02	4.04E-02 rs17875991	1_11749358_C_T	0.93	6718	9.75E-06 GCST90277338
Phosphatidylinositol (18:0_20:3) levels	1	87571263 C	T	8.50E-02	1.89E-02	6.49E-01 rs4655970	1_87571263_T_C	0.90	6718	6.73E-06 GCST90277338
Phosphatidylinositol (18:0_20:3) levels	1	181004059 C	T	-2.13E-01	4.64E-02	3.62E-02 rs72709643	1_181004059_T_C	0.97	6718	4.74E-06 GCST90277338
Phosphatidylinositol (18:0_20:3) levels	1	183933905 A	G	-2.36E-01	5.19E-02	2.83E-02 rs111306887	1_183933905_G_A	0.99	6718	5.62E-06 GCST90277338
Phosphatidylinositol (18:0_20:3) levels	1	230180443 G	A	-9.07E-02	1.78E-02	3.74E-01 rs606587	1_230180443_A_G	1.00	6718	3.41E-07 GCST90277338
Phosphatidylinositol (18:0_20:3) levels	1	240595182 A	G	-9.05E-02	2.02E-02	2.43E-01 rs61833681	1_240595182_G_A	0.98	6718	7.76E-06 GCST90277338
Phosphatidylinositol (18:0_20:3) levels	3	112816210 A	G	1.27E-01	2.36E-02	1.59E-01 rs11918522	3_112816210_G_A	1.00	6718	7.40E-08 GCST90277338
Phosphatidylinositol (18:0_20:3) levels	3	182474482 G	A	1.34E-01	2.95E-02	1.10E-01 rs62294908	3_182474482_A_G	0.87	6718	5.72E-06 GCST90277338
Phosphatidylinositol (18:0_20:3) levels	4	104599304 C	G	1.07E-01	2.30E-02	1.73E-01 rs62331262	4_104599304_G_C	0.98	6718	3.11E-06 GCST90277338
Phosphatidylinositol (18:0_20:3) levels	4	173654939 G	T	3.03E-01	6.83E-02	1.75E-02 rs115600841	4_173654939_T_G	0.93	6718	9.46E-06 GCST90277338
Phosphatidylinositol (18:0_20:3) levels	5	96599571 C	G	-1.14E-01	2.56E-02	1.30E-01 rs17086339	5_96599571_G_C	0.99	6718	8.41E-06 GCST90277338
Phosphatidylinositol (18:0_20:3) levels	7	17693175 T	C	8.75E-02	1.79E-02	3.72E-01 rs2537611	7_17693175_C_T	1.00	6718	9.77E-07 GCST90277338
Phosphatidylinositol (18:0_20:3) levels	7	31320724 A	G	-2.23E-01	4.91E-02	3.32E-02 rs74864573	7_31320724_G_A	0.96	6718	5.58E-06 GCST90277338
Phosphatidylinositol (18:0_20:3) levels	7	109154252 A	C	-4.39E-01	9.83E-02	8.32E-03 rs117145701	7_109154252_C_A	0.89	6718	8.00E-06 GCST90277338
Phosphatidylinositol (18:0_20:3) levels	11	12425660 A	G	7.67E-02	1.73E-02	5.35E-01 rs11022354	11_12425660_G_A	1.00	6718	9.48E-06 GCST90277338
Phosphatidylinositol (18:0_20:3) levels	11	34887145 G	A	8.67E-02	1.84E-02	6.69E-01 rs2985394	11_34887145_A_G	1.00	6718	2.63E-06 GCST90277338
Phosphatidylinositol (18:0_20:3) levels	11	61806212 C	T	2.29E-01	1.74E-02	4.06E-01 rs174551	11_61806212_T_C	1.00	6718	3.59E-39 GCST90277338
Phosphatidylinositol (18:0_20:3) levels	11	97125581 A	G	2.01E-01	4.29E-02	4.44E-02 rs79040967	11_97125581_G_A	0.95	6718	2.75E-06 GCST90277338
Phosphatidylinositol (18:0_20:3) levels	15	45403250 T	G	-3.22E-01	7.28E-02	1.51E-02 rs147996419	15_45403250_G_T	0.94	6718	1.00E-05 GCST90277338
Phosphatidylinositol (18:0_20:3) levels	15	58379522 A	G	-9.68E-02	1.80E-02	6.45E-01 rs1601934	15_58379522_G_A	0.99	6718	8.40E-08 GCST90277338
Phosphatidylinositol (18:0_20:3) levels	15	58431476 T	C	9.02E-02	2.00E-02	2.58E-01 rs1800588	15_58431476_C_T	0.99	6718	6.89E-06 GCST90277338
Phosphatidylinositol (18:0_20:3) levels	16	56960616 T	C	9.54E-02	1.91E-02	2.78E-01 rs17231506	16_56960616_C_T	1.00	6718	6.14E-07 GCST90277338
Phosphatidylinositol (18:0_20:3) levels	19	2786104 A	T	1.97E-01	4.29E-02	4.81E-02 rs58828781	19_2786104_T_A	0.88	6718	4.42E-06 GCST90277338
Phosphatidylinositol (18:0_20:3) levels	20	21155899 C	A	5.52E-01	1.21E-01	5.33E-03 rs73133304	20_21155899_A_C	0.96	6718	4.81E-06 GCST90277338
Phosphatidylinositol (18:0_20:3) levels	20	55033469 C	G	-2.46E-01	5.41E-02	2.66E-02 rs2133619	20_55033469_G_C	0.97	6718	5.38E-06 GCST90277338
Phosphatidylinositol (18:0_20:4) levels	1	20394196 A	G	-1.13E-01	2.55E-02	1.28E-01 rs1925669	1_20394196_G_A	1.00	6836	9.76E-06 GCST90277339
Phosphatidylinositol (18:0_20:4) levels	1	212057557 A	G	-2.28E-01	5.06E-02	2.98E-02 rs149022879	1_212057557_G_A	0.95	6836	6.44E-06 GCST90277339
Phosphatidylinositol (18:0_20:4) levels	1	245438211 A	G	-1.30E-01	2.83E-02	1.08E-01 rs12239821	1_245438211_G_A	0.95	6836	4.62E-06 GCST90277339
Phosphatidylinositol (18:0_20:4) levels	3	22449600 G	A	9.98E-02	2.18E-02	1.96E-01 rs9843297	3_22449600_A_G	0.99	6836	4.75E-06 GCST90277339
Phosphatidylinositol (18:0_20:4) levels	3	25418634 C	T	6.28E-01	1.30E-01	5.16E-03 rs2170790	3_25418634_T_C	0.92	6836	1.33E-06 GCST90277339
Phosphatidylinositol (18:0_20:4) levels	5	9303860 C	T	1.07E-01	2.35E-02	1.55E-01 rs12659734	5_9303860_T_C	1.00	6836	5.21E-06 GCST90277339
Phosphatidylinositol (18:0_20:4) levels	7	18082952 T	C	8.27E-02	1.79E-02	5.40E-01 rs1540881	7_18082952_C_T	0.93	6836	3.73E-06 GCST90277339
Phosphatidylinositol (18:0_20:4) levels	7	158353254 T	C	-2.14E-01	4.63E-02	3.59E-02 rs62493656	7_158353254_C_T	0.97	6836	3.89E-06 GCST90277339

Phosphatidylinositol (18:0_20:4) levels	8	140933062 A	G	-2.06E-01	4.64E-02	3.47E-02	rs139594872	8_140933062_G_A	0.98	6836	9.50E-06	GCST90277339
Phosphatidylinositol (18:0_20:4) levels	9	70856641 T	C	2.23E-01	4.97E-02	3.01E-02	rs78183891	9_70856641_C_T	0.99	6836	7.43E-06	GCST90277339
Phosphatidylinositol (18:0_20:4) levels	11	2436436 A	G	1.03E-01	2.33E-02	1.96E-01	rs1978125	11_2436436_G_A	0.86	6836	9.23E-06	GCST90277339
Phosphatidylinositol (18:0_20:4) levels	11	61803876 G	C	2.59E-01	1.74E-02	3.84E-01	rs174548	11_61803876_C_G	1.00	6836	2.68E-49	GCST90277339
Phosphatidylinositol (18:0_20:4) levels	11	75841084 T	C	9.20E-02	1.79E-02	5.82E-01	rs11236557	11_75841084_C_T	0.95	6836	2.63E-07	GCST90277339
Phosphatidylinositol (18:0_20:4) levels	15	68561088 G	C	-2.30E-01	5.10E-02	3.16E-02	rs188004353	15_68561088_C_G	0.92	6836	6.36E-06	GCST90277339
Phosphatidylinositol (18:0_20:4) levels	16	15036737 G	A	-1.09E-01	1.82E-02	3.34E-01	rs6498540	16_15036737_A_G	0.99	6836	1.93E-09	GCST90277339
Phosphatidylinositol (18:0_20:4) levels	16	82643141 G	A	1.08E-01	2.31E-02	8.21E-01	rs12444786	16_82643141_A_G	0.92	6836	3.29E-06	GCST90277339
Phosphatidylinositol (18:0_20:4) levels	19	770714 T	C	-1.02E-01	2.23E-02	2.09E-01	rs62131325	19_770714_C_T	0.87	6836	5.29E-06	GCST90277339
Phosphatidylinositol (18:0_20:4) levels	19	33752643 G	A	-8.47E-02	1.87E-02	2.97E-01	rs71351740	19_33752643_A_G	0.99	6836	6.11E-06	GCST90277339
Phosphatidylinositol (18:0_20:4) levels	19	44908822 T	C	2.05E-01	3.83E-02	5.31E-02	rs7412	19_44908822_C_T	1.00	6836	9.45E-08	GCST90277339
Phosphatidylinositol (18:0_20:4) levels	20	35695325 C	G	-2.56E-01	5.58E-02	2.64E-02	rs111260004	20_35695325_G_C	0.92	6836	4.57E-06	GCST90277339
Phosphatidylinositol (18:1_18:1) levels	1	234273597 G	T	-7.66E-02	1.68E-02	5.24E-01	rs12760720	1_234273597_T_G	1.00	7173	5.43E-06	GCST90277340
Phosphatidylinositol (18:1_18:1) levels	2	123649367 C	T	7.56E-02	1.70E-02	4.28E-01	rs6721307	2_123649367_T_C	1.00	7173	8.63E-06	GCST90277340
Phosphatidylinositol (18:1_18:1) levels	3	124049075 G	T	-9.20E-02	2.05E-02	7.90E-01	rs2332690	3_124049075_T_G	1.00	7173	7.30E-06	GCST90277340
Phosphatidylinositol (18:1_18:1) levels	5	132461886 C	T	-7.57E-02	1.70E-02	5.76E-01	rs2522051	5_132461886_T_C	1.00	7173	8.47E-06	GCST90277340
Phosphatidylinositol (18:1_18:1) levels	5	154465674 A	G	9.00E-02	1.98E-02	2.29E-01	rs34881711	5_154465674_G_A	0.99	7173	5.67E-06	GCST90277340
Phosphatidylinositol (18:1_18:1) levels	6	2822782 C	T	-4.21E-01	9.42E-02	8.67E-03	rs116814948	6_2822782_T_C	0.90	7173	7.98E-06	GCST90277340
Phosphatidylinositol (18:1_18:1) levels	6	34451115 A	G	9.37E-02	1.95E-02	7.62E-01	rs4142412	6_34451115_G_A	1.00	7173	1.66E-06	GCST90277340
Phosphatidylinositol (18:1_18:1) levels	6	169319631 T	C	-1.17E-01	2.59E-02	1.22E-01	rs9800437	6_169319631_C_T	0.97	7173	5.97E-06	GCST90277340
Phosphatidylinositol (18:1_18:1) levels	8	55496716 G	A	2.77E-01	6.14E-02	1.90E-02	rs149490626	8_55496716_A_G	0.96	7173	6.41E-06	GCST90277340
Phosphatidylinositol (18:1_18:1) levels	9	1294441 T	G	7.70E-02	1.66E-02	5.06E-01	rs4618752	9_1294441_G_T	1.00	7173	3.57E-06	GCST90277340
Phosphatidylinositol (18:1_18:1) levels	9	15290014 G	A	1.14E-01	2.56E-02	8.81E-01	rs638491	9_15290014_A_G	1.00	7173	8.31E-06	GCST90277340
Phosphatidylinositol (18:1_18:1) levels	11	61770929 C	G	-9.04E-02	1.71E-02	4.44E-01	rs174527	11_61770929_G_C	0.98	7173	1.22E-07	GCST90277340
Phosphatidylinositol (18:1_18:1) levels	11	61784455 C	A	-2.84E-01	1.67E-02	4.08E-01	rs174536	11_61784455_A_C	1.00	7173	3.74E-63	GCST90277340
Phosphatidylinositol (18:1_18:1) levels	11	62406721 G	A	1.01E-01	2.12E-02	8.04E-01	rs3017103	11_62406721_A_G	0.99	7173	1.91E-06	GCST90277340
Phosphatidylinositol (18:1_18:1) levels	12	124844767 C	T	-9.40E-02	1.68E-02	4.54E-01	rs11057853	12_124844767_T_C	0.99	7173	2.45E-08	GCST90277340
Phosphatidylinositol (18:1_18:1) levels	13	42984710 C	T	-8.13E-02	1.79E-02	6.87E-01	rs4942185	13_42984710_T_C	1.00	7173	5.59E-06	GCST90277340
Phosphatidylinositol (18:1_18:1) levels	14	41287117 T	G	-6.04E-01	1.33E-01	4.44E-03	rs79995028	14_41287117_G_T	0.89	7173	5.32E-06	GCST90277340
Phosphatidylinositol (18:1_18:1) levels	14	61648205 A	G	1.88E-01	4.23E-02	4.27E-02	rs77199708	14_61648205_G_A	0.92	7173	9.19E-06	GCST90277340
Phosphatidylinositol (18:1_18:1) levels	15	58391167 G	A	-8.22E-02	1.68E-02	5.73E-01	rs1532085	15_58391167_A_G	1.00	7173	1.05E-06	GCST90277340
Phosphatidylinositol (18:1_18:1) levels	15	58431476 T	C	1.07E-01	1.94E-02	2.58E-01	rs1800588	15_58431476_C_T	0.99	7173	3.09E-08	GCST90277340
Phosphatidylinositol (18:1_18:1) levels	16	56954132 T	C	1.21E-01	1.85E-02	2.83E-01	rs173539	16_56954132_C_T	1.00	7173	6.68E-11	GCST90277340
Phosphatidylinositol (18:1_18:1) levels	18	27258198 C	T	-2.26E-01	4.30E-02	4.16E-02	rs117215929	18_27258198_T_C	0.98	7173	1.52E-07	GCST90277340
Phosphatidylinositol (18:1_18:1) levels	20	63507672 T	C	8.46E-02	1.91E-02	7.33E-01	rs6090437	20_63507672_C_T	0.99	7173	9.23E-06	GCST90277340
Phosphatidylinositol (18:1_18:2) levels	1	14405165 A	G	-2.21E-01	4.88E-02	3.26E-02	rs34446132	1_14405165_G_A	0.94	7158	6.08E-06	GCST90277341
Phosphatidylinositol (18:1_18:2) levels	1	49528668 T	G	-2.93E-01	6.23E-02	1.95E-02	rs185625951	1_49528668_G_T	0.98	7158	2.52E-06	GCST90277341
Phosphatidylinositol (18:1_18:2) levels	1	200136295 T	C	-1.61E-01	3.48E-02	6.12E-02	rs12123955	1_200136295_C_T	0.99	7158	3.59E-06	GCST90277341
Phosphatidylinositol (18:1_18:2) levels	1	216582264 A	C	-1.43E-01	3.21E-02	7.81E-02	rs11572749	1_216582264_C_A	0.94	7158	9.02E-06	GCST90277341
Phosphatidylinositol (18:1_18:2) levels	1	245514505 T	G	1.06E-01	2.23E-02	1.73E-01	rs76437425	1_245514505_G_T	0.97	7158	1.96E-06	GCST90277341
Phosphatidylinositol (18:1_18:2) levels	2	12892879 G	A	7.95E-01	1.79E-01	2.49E-03	rs114061227	2_12892879_A_G	0.90	7158	9.34E-06	GCST90277341
Phosphatidylinositol (18:1_18:2) levels	2	81754844 T	C	2.43E-01	5.00E-02	3.13E-02	rs7563526	2_81754844_C_T	0.90	7158	1.17E-06	GCST90277341
Phosphatidylinositol (18:1_18:2) levels	3	82448350 T	C	9.65E-02	2.13E-02	1.87E-01	rs55904113	3_82448350_C_T	1.00	7158	6.11E-06	GCST90277341
Phosphatidylinositol (18:1_18:2) levels	4	7350426 A	G	3.47E-01	6.45E-02	1.77E-02	rs142235374	4_7350426_G_A	0.96	7158	7.44E-08	GCST90277341
Phosphatidylinositol (18:1_18:2) levels	4	28252716 A	G	-4.49E-01	9.42E-02	9.08E-03	rs188033349	4_28252716_G_A	0.87	7158	1.88E-06	GCST90277341
Phosphatidylinositol (18:1_18:2) levels	4	78357277 T	C	-2.64E-01	5.92E-02	2.01E-02	rs77219173	4_78357277_C_T	0.99	7158	8.10E-06	GCST90277341
Phosphatidylinositol (18:1_18:2) levels	4	137022381 G	C	3.27E-01	7.07E-02	1.43E-02	rs7677028	4_137022381_C_G	0.97	7158	3.79E-06	GCST90277341
Phosphatidylinositol (18:1_18:2) levels	4	151270229 G	A	-1.29E-01	2.84E-02	9.98E-02	rs11936543	4_151270229_A_G	0.97	7158	5.25E-06	GCST90277341
Phosphatidylinositol (18:1_18:2) levels	6	74169888 G	A	-8.16E-02	1.78E-02	6.71E-01	rs9341454	6_74169888_A_G	1.00	7158	4.37E-06	GCST90277341
Phosphatidylinositol (18:1_18:2) levels	7	52644681 T	G	1.07E-01	2.34E-02	1.52E-01	rs75957793	7_52644681_G_T	1.00	7158	5.10E-06	GCST90277341
Phosphatidylinositol (18:1_18:2) levels	8	31250608 T	C	3.01E-01	6.25E-02	2.04E-02	rs149897686	8_31250608_C_T	0.90	7158	1.46E-06	GCST90277341
Phosphatidylinositol (18:1_18:2) levels	9	135771210 A	G	7.75E-02	1.73E-02	4.84E-01	rs7870035	9_135771210_G_A	0.93	7158	7.81E-06	GCST90277341
Phosphatidylinositol (18:1_18:2) levels	10	55141172 T	C	3.66E-01	8.01E-02	1.08E-02	rs7091107	10_55141172_C_T	1.00	7158	5.09E-06	GCST90277341
Phosphatidylinositol (18:1_18:2) levels	11	4976404 G	A	7.86E-02	1.70E-02	5.73E-01	rs10768424	11_4976404_A_G	1.00	7158	3.98E-06	GCST90277341
Phosphatidylinositol (18:1_18:2) levels	11	61824890 G	A	1.48E-01	1.70E-02	4.08E-01	rs174566	11_61824890_A_G	1.00	7158	4.33E-18	GCST90277341
Phosphatidylinositol (18:1_18:2) levels	11	100294047 T	C	-7.96E-01	1.65E-01	2.59E-03	rs113016188	11_100294047_C_T	0.96	7158	1.56E-06	GCST90277341
Phosphatidylinositol (18:1_18:2) levels	16	47545501 T	C	-3.25E-01	7.23E-02	1.37E-02	rs149389892	16_47545501_C_T	1.00	7158	7.22E-06	GCST90277341

Phosphatidylinositol (18:1_18:2) levels	16	56954132	T	C	1.09E-01	1.85E-02	2.83E-01	rs173539	16_56954132_C_T	1.00	7158	3.53E-09	GCST90277341
Phosphatidylinositol (18:1_18:2) levels	17	13886556	C	G	-2.10E-01	4.62E-02	3.40E-02	rs73263508	17_13886556_G_C	1.00	7158	5.60E-06	GCST90277341
Phosphatidylinositol (18:1_18:2) levels	18	49199328	A	G	-2.11E-01	4.67E-02	3.34E-02	rs146468726	18_49199328_G_A	0.98	7158	6.14E-06	GCST90277341
Phosphatidylinositol (18:1_18:2) levels	21	37607127	A	G	-7.83E-02	1.73E-02	6.21E-01	rs10775658	21_37607127_G_A	1.00	7158	6.06E-06	GCST90277341
Phosphatidylinositol (18:1_20:4) levels	1	181004059	C	T	-2.17E-01	4.65E-02	3.62E-02	rs72709643	1_181004059_T_C	0.97	6677	3.21E-06	GCST90277342
Phosphatidylinositol (18:1_20:4) levels	2	4201657	A	G	-2.16E-01	4.60E-02	3.73E-02	rs55975242	2_4201657_G_A	0.99	6677	2.58E-06	GCST90277342
Phosphatidylinositol (18:1_20:4) levels	2	4222758	ATT	AT	-2.09E-01	4.70E-02	3.77E-02	rs142598860	2_4222758_AT_ATT	0.98	6677	8.39E-06	GCST90277342
Phosphatidylinositol (18:1_20:4) levels	2	125728700	G	C	2.24E-01	4.96E-02	3.25E-02	rs148835629	2_125728700_C_G	0.97	6677	6.67E-06	GCST90277342
Phosphatidylinositol (18:1_20:4) levels	3	112839107	T	C	1.30E-01	2.38E-02	1.57E-01	rs7642984	3_112839107_C_T	1.00	6677	5.05E-08	GCST90277342
Phosphatidylinositol (18:1_20:4) levels	3	143232607	A	C	1.03E-01	2.26E-02	1.80E-01	rs56734149	3_143232607_C_A	1.00	6677	5.56E-06	GCST90277342
Phosphatidylinositol (18:1_20:4) levels	3	182320787	C	T	1.33E-01	2.93E-02	1.08E-01	rs73189920	3_182320787_T_C	0.91	6677	6.13E-06	GCST90277342
Phosphatidylinositol (18:1_20:4) levels	6	69804495	T	A	1.68E-01	3.76E-02	5.61E-02	rs79847391	6_69804495_A_T	0.99	6677	8.04E-06	GCST90277342
Phosphatidylinositol (18:1_20:4) levels	7	53119301	C	A	-2.37E-01	4.78E-02	3.40E-02	rs370796571	7_53119301_A_C	1.00	6677	7.72E-07	GCST90277342
Phosphatidylinositol (18:1_20:4) levels	8	77170491	G	A	-1.35E-01	3.00E-02	9.22E-02	rs1982433	8_77170491_A_G	1.00	6677	6.46E-06	GCST90277342
Phosphatidylinositol (18:1_20:4) levels	8	88464222	G	T	8.86E-02	1.98E-02	2.57E-01	rs3110417	8_88464222_T_G	1.00	6677	8.10E-06	GCST90277342
Phosphatidylinositol (18:1_20:4) levels	9	87561451	A	G	-8.87E-02	1.88E-02	2.99E-01	rs10868635	9_87561451_G_A	1.00	6677	2.59E-06	GCST90277342
Phosphatidylinositol (18:1_20:4) levels	10	119656173	A	G	9.73E-02	2.09E-02	2.29E-01	rs72840788	10_119656173_G_A	0.99	6677	3.16E-06	GCST90277342
Phosphatidylinositol (18:1_20:4) levels	11	35071655	A	G	-4.17E-01	8.95E-02	9.13E-03	rs117376487	11_35071655_G_A	1.00	6677	3.31E-06	GCST90277342
Phosphatidylinositol (18:1_20:4) levels	11	61820833	G	A	1.47E-01	1.75E-02	4.09E-01	rs174564	11_61820833_A_G	1.00	6677	7.47E-17	GCST90277342
Phosphatidylinositol (18:1_20:4) levels	14	73445684	C	A	8.76E-02	1.97E-02	2.58E-01	rs79713419	14_73445684_A_C	1.00	6677	9.10E-06	GCST90277342
Phosphatidylinositol (18:1_20:4) levels	15	58431280	C	T	8.95E-02	2.00E-02	2.66E-01	rs1077834	15_58431280_T_C	0.98	6677	7.47E-06	GCST90277342
Phosphatidylinositol (18:1_20:4) levels	15	59939767	A	G	7.94E-02	1.75E-02	5.39E-01	rs12441343	15_59939767_G_A	0.97	6677	5.58E-06	GCST90277342
Phosphatidylinositol (18:1_20:4) levels	17	66778240	G	C	1.75E-01	3.64E-02	6.09E-02	rs62621679	17_66778240_C_G	0.99	6677	1.61E-06	GCST90277342
Phosphatidylinositol (18:1_20:4) levels	19	2942174	T	C	-9.93E-02	2.24E-02	2.36E-01	rs77664718	19_2942174_C_T	0.83	6677	9.55E-06	GCST90277342
Sphingomyelin (d32:1) levels	1	43502581	C	A	2.34E-01	4.95E-02	3.06E-02	rs191784724	1_43502581_A_C	0.98	7153	2.38E-06	GCST90277343
Sphingomyelin (d32:1) levels	3	60237477	A	G	3.69E-01	7.84E-02	1.19E-02	rs114930909	3_60237477_G_A	0.95	7153	2.57E-06	GCST90277343
Sphingomyelin (d32:1) levels	4	3535970	T	C	1.58E-01	3.54E-02	6.36E-02	rs113370662	4_3535970_C_T	0.94	7153	8.15E-06	GCST90277343
Sphingomyelin (d32:1) levels	4	137022381	G	C	3.24E-01	7.06E-02	1.43E-02	rs7677028	4_137022381_C_G	0.97	7153	4.38E-06	GCST90277343
Sphingomyelin (d32:1) levels	5	38235392	T	C	-3.58E-01	7.94E-02	1.20E-02	rs149058290	5_38235392_C_T	0.92	7153	6.50E-06	GCST90277343
Sphingomyelin (d32:1) levels	5	154241592	G	T	1.95E-01	4.19E-02	4.17E-02	rs114401161	5_154241592_T_G	0.98	7153	3.14E-06	GCST90277343
Sphingomyelin (d32:1) levels	6	10147800	T	A	-7.75E-02	1.74E-02	6.36E-01	rs9366277	6_10147800_A_T	1.00	7153	8.27E-06	GCST90277343
Sphingomyelin (d32:1) levels	6	169316922	T	C	-1.75E-01	3.77E-02	5.43E-02	rs61448283	6_169316922_C_T	0.95	7153	3.69E-06	GCST90277343
Sphingomyelin (d32:1) levels	9	20097890	C	T	-3.44E-01	7.63E-02	1.27E-02	rs375094094	9_20097890_T_C	0.95	7153	6.53E-06	GCST90277343
Sphingomyelin (d32:1) levels	9	107072174	G	A	-8.10E-02	1.69E-02	5.50E-01	rs784655	9_107072174_A_G	1.00	7153	1.59E-06	GCST90277343
Sphingomyelin (d32:1) levels	11	732354	C	T	-1.75E-01	3.92E-02	4.92E-02	rs150046733	11_732354_T_C	0.97	7153	7.84E-06	GCST90277343
Sphingomyelin (d32:1) levels	11	61832870	C	A	-1.56E-01	1.70E-02	5.91E-01	rs174574	11_61832870_A_C	1.00	7153	6.55E-20	GCST90277343
Sphingomyelin (d32:1) levels	12	124967043	G	A	9.59E-02	1.87E-02	2.88E-01	rs35874447	12_124967043_A_G	0.96	7153	2.80E-07	GCST90277343
Sphingomyelin (d32:1) levels	14	77828999	A	G	1.12E-01	2.38E-02	1.45E-01	rs28489182	14_77828999_G_A	0.97	7153	2.46E-06	GCST90277343
Sphingomyelin (d32:1) levels	15	58379522	A	G	-8.74E-02	1.75E-02	6.45E-01	rs1601934	15_58379522_G_A	0.99	7153	5.69E-07	GCST90277343
Sphingomyelin (d32:1) levels	15	58431476	T	C	1.03E-01	1.94E-02	2.58E-01	rs1800588	15_58431476_C_T	0.99	7153	1.03E-07	GCST90277343
Sphingomyelin (d32:1) levels	16	56960616	T	C	1.17E-01	1.86E-02	2.78E-01	rs17231506	16_56960616_C_T	1.00	7153	2.96E-10	GCST90277343
Sphingomyelin (d32:1) levels	16	57284962	G	A	7.89E-02	1.74E-02	3.58E-01	rs12922657	16_57284962_A_G	0.97	7153	6.13E-06	GCST90277343
Sphingomyelin (d32:1) levels	21	30352436	C	T	-2.46E-01	5.48E-02	2.77E-02	rs140484966	21_30352436_T_C	0.89	7153	7.38E-06	GCST90277343
Sphingomyelin (d34:0) levels	1	47591044	A	G	-1.56E-01	3.21E-02	8.90E-02	rs12046045	1_47591044_G_A	0.99	6127	1.12E-06	GCST90277344
Sphingomyelin (d34:0) levels	1	164952845	A	G	-8.18E-02	1.82E-02	5.34E-01	rs1936090	1_164952845_G_A	1.00	6127	6.91E-06	GCST90277344
Sphingomyelin (d34:0) levels	1	201955621	C	G	8.84E-02	1.99E-02	2.91E-01	rs3820438	1_201955621_G_C	0.98	6127	9.13E-06	GCST90277344
Sphingomyelin (d34:0) levels	1	245190029	CG	C	-1.66E-01	3.62E-02	7.10E-02	rs36140849	1_245190029_C_CG	0.94	6127	4.62E-06	GCST90277344
Sphingomyelin (d34:0) levels	2	123610457	C	T	8.72E-02	1.84E-02	4.28E-01	rs13401553	2_123610457_T_C	1.00	6127	2.18E-06	GCST90277344
Sphingomyelin (d34:0) levels	2	191752537	C	A	-4.52E-01	9.60E-02	9.49E-03	rs6722606	2_191752537_A_C	0.99	6127	2.52E-06	GCST90277344
Sphingomyelin (d34:0) levels	4	179908526	T	A	-9.20E-02	2.08E-02	7.51E-01	rs7660654	4_179908526_A_T	1.00	6127	9.70E-06	GCST90277344
Sphingomyelin (d34:0) levels	7	41177033	C	T	5.17E-01	1.13E-01	6.57E-03	rs112718171	7_41177033_T_C	0.96	6127	5.32E-06	GCST90277344
Sphingomyelin (d34:0) levels	7	82556403	A	G	8.73E-02	1.81E-02	5.87E-01	rs2189991	7_82556403_G_A	1.00	6127	1.53E-06	GCST90277344
Sphingomyelin (d34:0) levels	7	130802167	G	A	-8.80E-02	1.88E-02	5.74E-01	rs6467318	7_130802167_A_G	0.92	6127	2.82E-06	GCST90277344
Sphingomyelin (d34:0) levels	8	76925244	A	C	-1.54E-01	3.44E-02	7.51E-02	rs73691232	8_76925244_C_A	0.99	6127	7.59E-06	GCST90277344
Sphingomyelin (d34:0) levels	9	32337256	G	T	3.40E-01	7.11E-02	1.78E-02	rs76375697	9_32337256_T_G	0.92	6127	1.77E-06	GCST90277344
Sphingomyelin (d34:0) levels	11	61785208	T	G	-1.76E-01	1.84E-02	4.08E-01	rs174537	11_61785208_G_T	1.00	6127	1.97E-21	GCST90277344
Sphingomyelin (d34:0) levels	11	89058340	A	G	-1.17E-01	2.46E-02	1.59E-01	rs7118021	11_89058340_G_A	0.99	6127	1.95E-06	GCST90277344

Sphingomyelin (d34:0) levels	13	66979217	A	G	-2.54E-01	4.75E-02	3.70E-02	rs113023745	13_66979217_G_A	0.99	6127	9.21E-08	GCST90277344
Sphingomyelin (d34:0) levels	14	67491655	C	A	-1.03E-01	1.81E-02	4.55E-01	rs1980615	14_67491655_A_C	1.00	6127	1.12E-08	GCST90277344
Sphingomyelin (d34:0) levels	15	43258624	A	G	2.50E-01	5.30E-02	3.23E-02	rs17778967	15_43258624_G_A	0.93	6127	2.46E-06	GCST90277344
Sphingomyelin (d34:0) levels	16	57439704	G	T	-2.00E-01	4.45E-02	4.13E-02	rs7203057	16_57439704_T_G	1.00	6127	6.77E-06	GCST90277344
Sphingomyelin (d34:0) levels	16	75555692	A	G	-1.24E-01	2.63E-02	1.34E-01	rs16975847	16_75555692_G_A	1.00	6127	2.76E-06	GCST90277344
Sphingomyelin (d34:0) levels	17	13263077	T	C	1.77E-01	3.90E-02	6.07E-02	rs145525514	17_13263077_C_T	0.94	6127	5.59E-06	GCST90277344
Sphingomyelin (d34:0) levels	17	17676043	T	C	-3.49E-01	7.57E-02	1.75E-02	rs141198865	17_17676043_C_T	0.85	6127	4.02E-06	GCST90277344
Sphingomyelin (d34:0) levels	18	27258198	C	T	-2.29E-01	4.67E-02	4.16E-02	rs117215929	18_27258198_T_C	0.98	6127	1.00E-06	GCST90277344
Sphingomyelin (d34:1) levels	1	15301756	G	T	1.71E-01	3.75E-02	7.15E-02	rs7550598	1_15301756_T_G	1.00	5462	5.20E-06	GCST90277345
Sphingomyelin (d34:1) levels	1	214395764	C	T	-1.53E-01	3.29E-02	9.57E-02	rs4129186	1_214395764_T_C	0.97	5462	3.37E-06	GCST90277345
Sphingomyelin (d34:1) levels	2	11679202	A	G	1.97E-01	4.22E-02	6.12E-02	rs140904588	2_11679202_G_A	0.89	5462	2.89E-06	GCST90277345
Sphingomyelin (d34:1) levels	2	27508073	C	T	-1.16E-01	1.99E-02	6.51E-01	rs1260326	2_27508073_T_C	1.00	5462	5.70E-09	GCST90277345
Sphingomyelin (d34:1) levels	5	163344530	T	C	1.73E-01	3.84E-02	6.76E-02	rs35319363	5_163344530_C_T	0.99	5462	6.97E-06	GCST90277345
Sphingomyelin (d34:1) levels	6	32363117	A	G	9.02E-02	2.01E-02	6.63E-01	rs6415128	6_32363117_G_A	1.00	5462	7.65E-06	GCST90277345
Sphingomyelin (d34:1) levels	6	169389898	C	T	1.22E-01	2.70E-02	8.51E-01	rs436509	6_169389898_T_C	0.99	5462	6.32E-06	GCST90277345
Sphingomyelin (d34:1) levels	7	43464229	G	A	9.22E-02	1.94E-02	3.90E-01	rs61696472	7_43464229_A_G	1.00	5462	1.99E-06	GCST90277345
Sphingomyelin (d34:1) levels	8	66311811	C	T	-5.04E-01	9.80E-02	9.96E-03	rs4269529	8_66311811_T_C	0.99	5462	2.76E-07	GCST90277345
Sphingomyelin (d34:1) levels	9	116846849	C	G	4.59E-01	9.77E-02	1.09E-02	rs117099105	9_116846849_G_C	0.96	5462	2.73E-06	GCST90277345
Sphingomyelin (d34:1) levels	9	123321244	T	C	-1.02E-01	2.29E-02	7.71E-01	rs1891637	9_123321244_C_T	0.97	5462	7.64E-06	GCST90277345
Sphingomyelin (d34:1) levels	10	63102554	G	T	-1.09E-01	2.44E-02	8.11E-01	rs4363488	10_63102554_T_G	1.00	5462	8.20E-06	GCST90277345
Sphingomyelin (d34:1) levels	11	61820833	G	A	3.08E-01	1.89E-02	4.09E-01	rs174564	11_61820833_A_G	1.00	5462	5.58E-58	GCST90277345
Sphingomyelin (d34:1) levels	11	62056158	C	T	1.18E-01	2.52E-02	1.73E-01	rs12226389	11_62056158_T_C	0.99	5462	3.11E-06	GCST90277345
Sphingomyelin (d34:1) levels	11	116943088	T	C	-1.46E-01	2.53E-02	8.29E-01	rs510988	11_116943088_C_T	1.00	5462	7.40E-09	GCST90277345
Sphingomyelin (d34:1) levels	15	57885393	T	C	9.56E-02	1.93E-02	5.30E-01	rs894342	15_57885393_C_T	0.99	5462	7.35E-07	GCST90277345
Sphingomyelin (d34:1) levels	15	58388755	C	T	-2.63E-01	1.88E-02	5.76E-01	rs2043085	15_58388755_T_C	1.00	5462	1.18E-43	GCST90277345
Sphingomyelin (d34:1) levels	15	58431280	C	T	3.16E-01	2.12E-02	2.66E-01	rs1077834	15_58431280_T_C	0.98	5462	1.42E-49	GCST90277345
Sphingomyelin (d34:1) levels	15	58729903	A	G	1.06E-01	2.11E-02	2.80E-01	rs544282	15_58729903_G_A	1.00	5462	5.18E-07	GCST90277345
Sphingomyelin (d34:1) levels	15	59032191	G	A	2.25E-01	2.85E-02	1.25E-01	rs34278620	15_59032191_A_G	1.00	5462	2.95E-15	GCST90277345
Sphingomyelin (d34:1) levels	15	74379820	G	A	-1.42E-01	2.73E-02	1.53E-01	rs7180275	15_74379820_A_G	0.99	5462	2.05E-07	GCST90277345
Sphingomyelin (d34:2) levels	1	7531062	C	A	8.46E-02	1.85E-02	6.55E-01	rs1193168	1_7531062_A_C	0.98	6666	4.64E-06	GCST90277346
Sphingomyelin (d34:2) levels	2	27508073	C	T	-8.62E-02	1.82E-02	6.51E-01	rs1260326	2_27508073_T_C	1.00	6666	2.11E-06	GCST90277346
Sphingomyelin (d34:2) levels	2	101740535	G	A	-1.21E-01	2.40E-02	1.51E-01	rs62155727	2_101740535_A_G	1.00	6666	4.42E-07	GCST90277346
Sphingomyelin (d34:2) levels	2	138745944	A	G	3.83E-01	8.20E-02	1.27E-02	rs79450270	2_138745944_G_A	0.90	6666	3.03E-06	GCST90277346
Sphingomyelin (d34:2) levels	3	8868967	C	T	-1.87E-01	4.20E-02	4.66E-02	rs74929979	3_8868967_T_C	0.98	6666	9.13E-06	GCST90277346
Sphingomyelin (d34:2) levels	3	141158645	G	C	-4.67E-01	9.67E-02	8.21E-03	rs115265221	3_141158645_C_G	0.97	6666	1.37E-06	GCST90277346
Sphingomyelin (d34:2) levels	4	100929253	T	G	3.13E-01	6.08E-02	2.16E-02	rs72679383	4_100929253_G_T	0.96	6666	2.65E-07	GCST90277346
Sphingomyelin (d34:2) levels	4	121541914	A	G	-8.30E-02	1.77E-02	4.17E-01	rs4370152	4_121541914_G_A	1.00	6666	2.70E-06	GCST90277346
Sphingomyelin (d34:2) levels	7	35570399	A	C	1.47E-01	3.28E-02	7.47E-02	rs76435838	7_35570399_C_A	1.00	6666	7.46E-06	GCST90277346
Sphingomyelin (d34:2) levels	8	138164717	C	T	-1.78E-01	3.74E-02	6.58E-02	rs75344234	8_138164717_T_C	0.87	6666	1.98E-06	GCST90277346
Sphingomyelin (d34:2) levels	9	137858500	A	T	1.45E-01	2.91E-02	1.09E-01	rs149460121	9_137858500_T_A	0.93	6666	5.91E-07	GCST90277346
Sphingomyelin (d34:2) levels	10	59368742	T	C	-2.54E-01	5.31E-02	3.04E-02	rs139848079	10_59368742_C_T	0.91	6666	1.66E-06	GCST90277346
Sphingomyelin (d34:2) levels	10	128061505	A	G	1.20E-01	2.57E-02	1.35E-01	rs9943332	10_128061505_G_A	0.99	6666	3.01E-06	GCST90277346
Sphingomyelin (d34:2) levels	11	98131421	T	G	1.35E-01	3.05E-02	8.87E-02	rs55864504	11_98131421_G_T	1.00	6666	9.93E-06	GCST90277346
Sphingomyelin (d34:2) levels	11	117050674	C	T	-1.34E-01	2.54E-02	8.66E-01	rs1815786	11_117050674_T_C	1.00	6666	1.43E-07	GCST90277346
Sphingomyelin (d34:2) levels	11	117828871	G	T	8.03E-02	1.74E-02	4.66E-01	rs10892181	11_117828871_T_G	0.98	6666	4.17E-06	GCST90277346
Sphingomyelin (d34:2) levels	12	60861641	T	C	1.67E-01	3.69E-02	5.92E-02	rs34733845	12_60861641_C_T	0.97	6666	5.96E-06	GCST90277346
Sphingomyelin (d34:2) levels	14	41753127	G	T	4.02E-01	9.06E-02	1.04E-02	rs192384799	14_41753127_T_G	1.00	6666	9.43E-06	GCST90277346
Sphingomyelin (d34:2) levels	14	57331615	C	T	1.48E-01	3.32E-02	7.83E-02	rs142259144	14_57331615_T_C	0.96	6666	8.74E-06	GCST90277346
Sphingomyelin (d34:2) levels	15	57616988	C	T	9.96E-02	1.75E-02	4.39E-01	rs1690333	15_57616988_T_C	1.00	6666	1.20E-08	GCST90277346
Sphingomyelin (d34:2) levels	15	58007401	A	G	1.61E-01	2.90E-02	1.04E-01	rs61999891	15_58007401_G_A	0.96	6666	3.10E-08	GCST90277346
Sphingomyelin (d34:2) levels	15	58388755	C	T	-3.72E-01	1.69E-02	5.76E-01	rs2043085	15_58388755_T_C	1.00	6666	3.62E-104	GCST90277346
Sphingomyelin (d34:2) levels	15	58431476	T	C	3.93E-01	1.94E-02	2.58E-01	rs1800588	15_58431476_C_T	0.99	6666	1.26E-88	GCST90277346
Sphingomyelin (d34:2) levels	15	59032191	G	A	2.13E-01	2.62E-02	1.25E-01	rs34278620	15_59032191_A_G	1.00	6666	4.39E-16	GCST90277346
Sphingomyelin (d34:2) levels	18	47243946	A	C	-1.18E-01	2.48E-02	8.53E-01	rs2684847	18_47243946_C_A	0.98	6666	2.02E-06	GCST90277346
Sphingomyelin (d36:1) levels	2	27508073	C	T	-1.22E-01	1.74E-02	6.51E-01	rs1260326	2_27508073_T_C	1.00	7128	2.57E-12	GCST90277347
Sphingomyelin (d36:1) levels	2	174696563	A	G	1.06E-01	2.30E-02	1.60E-01	rs4296402	2_174696563_G_A	0.98	7128	4.49E-06	GCST90277347
Sphingomyelin (d36:1) levels	4	4996200	T	C	1.00E-01	2.10E-02	2.14E-01	rs62291572	4_4996200_C_T	0.94	7128	1.96E-06	GCST90277347

Sphingomyelin (d36:1) levels	4	38137235	A	G	-1.21E-01	2.61E-02	1.14E-01	rs13110318	4_38137235_G_A	1.00	7128	3.74E-06 GCST90277347
Sphingomyelin (d36:1) levels	4	100929253	T	G	2.86E-01	5.85E-02	2.16E-02	rs72679383	4_100929253_G_T	0.96	7128	1.04E-06 GCST90277347
Sphingomyelin (d36:1) levels	4	115874677	T	C	-2.43E-01	5.47E-02	2.48E-02	rs149431055	4_115874677_C_T	0.97	7128	9.10E-06 GCST90277347
Sphingomyelin (d36:1) levels	5	86297284	C	T	9.72E-02	1.96E-02	7.64E-01	rs10069895	5_86297284_T_C	1.00	7128	7.18E-07 GCST90277347
Sphingomyelin (d36:1) levels	5	120061007	G	A	3.73E-01	8.26E-02	1.02E-02	rs141883259	5_120061007_A_G	0.98	7128	6.59E-06 GCST90277347
Sphingomyelin (d36:1) levels	6	31722232	A	G	8.50E-02	1.74E-02	3.78E-01	rs805292	6_31722232_G_A	1.00	7128	1.10E-06 GCST90277347
Sphingomyelin (d36:1) levels	6	94336592	T	A	-7.82E-02	1.70E-02	4.08E-01	rs419482	6_94336592_A_T	1.00	7128	4.12E-06 GCST90277347
Sphingomyelin (d36:1) levels	6	154681357	A	G	1.09E-01	2.30E-02	1.60E-01	rs62434228	6_154681357_G_A	0.99	7128	2.35E-06 GCST90277347
Sphingomyelin (d36:1) levels	7	146994417	C	T	-1.82E-01	4.03E-02	4.40E-02	rs10276778	7_146994417_T_C	1.00	7128	6.58E-06 GCST90277347
Sphingomyelin (d36:1) levels	8	12966162	C	T	1.05E-01	2.08E-02	2.06E-01	rs2946493	8_12966162_T_C	0.98	7128	4.35E-07 GCST90277347
Sphingomyelin (d36:1) levels	10	63110944	A	G	-1.04E-01	2.30E-02	8.45E-01	rs10761714	10_63110944_G_A	0.99	7128	6.47E-06 GCST90277347
Sphingomyelin (d36:1) levels	11	61770929	C	G	1.02E-01	1.71E-02	4.44E-01	rs174527	11_61770929_G_C	0.98	7128	2.73E-09 GCST90277347
Sphingomyelin (d36:1) levels	11	61825533	G	A	3.43E-01	1.66E-02	4.09E-01	rs174567	11_61825533_A_G	1.00	7128	1.25E-92 GCST90277347
Sphingomyelin (d36:1) levels	11	62205328	A	G	-2.13E-01	3.45E-02	6.18E-02	rs117900629	11_62205328_G_A	0.99	7128	6.25E-10 GCST90277347
Sphingomyelin (d36:1) levels	11	116778201	C	G	-1.54E-01	2.32E-02	8.49E-01	rs964184	11_116778201_G_C	1.00	7128	2.75E-11 GCST90277347
Sphingomyelin (d36:1) levels	12	17547405	C	T	-9.25E-02	2.08E-02	1.95E-01	rs35828755	12_17547405_T_C	0.99	7128	9.29E-06 GCST90277347
Sphingomyelin (d36:1) levels	12	60861641	T	C	1.65E-01	3.57E-02	5.92E-02	rs34733845	12_60861641_C_T	0.97	7128	3.86E-06 GCST90277347
Sphingomyelin (d36:1) levels	15	58244328	C	T	3.52E-01	5.36E-02	2.66E-02	rs138283783	15_58244328_T_C	0.96	7128	5.56E-11 GCST90277347
Sphingomyelin (d36:1) levels	15	58386313	T	C	2.98E-01	1.72E-02	3.38E-01	rs10468017	15_58386313_C_T	1.00	7128	9.03E-66 GCST90277347
Sphingomyelin (d36:1) levels	15	58433640	G	A	2.55E-01	1.80E-02	3.14E-01	rs633695	15_58433640_A_G	1.00	7128	5.56E-45 GCST90277347
Sphingomyelin (d36:1) levels	15	58506762	A	G	6.30E-01	7.04E-02	1.58E-02	rs117376818	15_58506762_G_A	0.90	7128	4.16E-19 GCST90277347
Sphingomyelin (d36:1) levels	15	58607410	C	T	3.55E-01	4.53E-02	3.50E-02	rs73424597	15_58607410_T_C	0.99	7128	5.88E-15 GCST90277347
Sphingomyelin (d36:1) levels	15	59047450	C	G	1.56E-01	3.38E-02	6.48E-02	rs112129861	15_59047450_G_C	1.00	7128	3.91E-06 GCST90277347
Sphingomyelin (d36:1) levels	15	59239619	C	T	2.80E-01	6.04E-02	2.23E-02	rs111543310	15_59239619_T_C	0.89	7128	3.55E-06 GCST90277347
Sphingomyelin (d36:1) levels	16	15056648	A	C	-1.13E-01	1.86E-02	2.74E-01	rs12928099	16_15056648_C_A	1.00	7128	1.50E-09 GCST90277347
Sphingomyelin (d36:1) levels	16	84499954	C	G	7.63E-02	1.68E-02	5.41E-01	rs449480	16_84499954_G_C	0.99	7128	5.89E-06 GCST90277347
Sphingomyelin (d36:1) levels	17	79394643	C	T	-8.08E-02	1.78E-02	6.84E-01	rs7226158	17_79394643_T_C	1.00	7128	6.06E-06 GCST90277347
Sphingomyelin (d36:1) levels	19	55803533	C	T	-1.11E-01	2.50E-02	1.33E-01	rs2840005	19_55803533_T_C	0.96	7128	8.44E-06 GCST90277347
Sphingomyelin (d36:2) levels	2	27508073	C	T	-8.75E-02	1.74E-02	6.51E-01	rs1260326	2_27508073_T_C	1.00	7164	5.43E-07 GCST90277348
Sphingomyelin (d36:2) levels	3	71834170	A	G	-8.19E-02	1.82E-02	3.01E-01	rs113887618	3_71834170_G_A	1.00	7164	6.81E-06 GCST90277348
Sphingomyelin (d36:2) levels	4	61547490	T	C	-1.88E-01	4.13E-02	4.24E-02	rs143607354	4_61547490_C_T	0.99	7164	5.48E-06 GCST90277348
Sphingomyelin (d36:2) levels	8	11624851	A	G	-2.60E-01	5.48E-02	2.51E-02	rs116866162	8_11624851_G_A	0.93	7164	2.07E-06 GCST90277348
Sphingomyelin (d36:2) levels	9	74092748	A	G	2.26E-01	4.88E-02	3.07E-02	rs62549032	9_74092748_G_A	0.95	7164	3.71E-06 GCST90277348
Sphingomyelin (d36:2) levels	9	120350676	T	C	2.21E-01	4.47E-02	3.92E-02	rs72753379	9_120350676_C_T	0.95	7164	8.09E-07 GCST90277348
Sphingomyelin (d36:2) levels	11	31584349	T	A	1.77E-01	3.54E-02	5.86E-02	rs11031419	11_31584349_A_T	0.99	7164	6.13E-07 GCST90277348
Sphingomyelin (d36:2) levels	11	61785208	T	G	-1.88E-01	1.69E-02	4.08E-01	rs174537	11_61785208_G_T	1.00	7164	1.33E-28 GCST90277348
Sphingomyelin (d36:2) levels	11	116834852	T	G	-1.49E-01	2.45E-02	8.63E-01	rs10750098	11_116834852_G_T	1.00	7164	1.09E-09 GCST90277348
Sphingomyelin (d36:2) levels	13	76058703	A	G	2.77E-01	6.23E-02	1.87E-02	rs77144832	13_76058703_G_A	0.96	7164	8.93E-06 GCST90277348
Sphingomyelin (d36:2) levels	15	58244328	C	T	3.65E-01	5.34E-02	2.66E-02	rs138283783	15_58244328_T_C	0.96	7164	9.22E-12 GCST90277348
Sphingomyelin (d36:2) levels	15	58386313	T	C	3.54E-01	1.71E-02	3.38E-01	rs10468017	15_58386313_C_T	1.00	7164	7.59E-93 GCST90277348
Sphingomyelin (d36:2) levels	15	58433640	G	A	2.88E-01	1.79E-02	3.14E-01	rs633695	15_58433640_A_G	1.00	7164	2.75E-57 GCST90277348
Sphingomyelin (d36:2) levels	15	58506762	A	G	6.64E-01	7.01E-02	1.58E-02	rs117376818	15_58506762_G_A	0.90	7164	3.43E-21 GCST90277348
Sphingomyelin (d36:2) levels	15	58576986	G	A	3.46E-01	4.56E-02	3.45E-02	rs73424577	15_58576986_A_G	0.99	7164	3.66E-14 GCST90277348
Sphingomyelin (d36:2) levels	15	59047450	C	G	1.74E-01	3.37E-02	6.48E-02	rs112129861	15_59047450_G_C	1.00	7164	2.51E-07 GCST90277348
Sphingomyelin (d36:2) levels	15	59239619	C	T	2.71E-01	6.01E-02	2.23E-02	rs111543310	15_59239619_T_C	0.89	7164	6.69E-06 GCST90277348
Sphingomyelin (d36:2) levels	18	47575930	G	A	2.15E-01	4.56E-02	3.81E-02	rs17798605	18_47575930_A_G	0.92	7164	2.43E-06 GCST90277348
Sphingomyelin (d36:2) levels	18	67212380	G	C	-2.27E-01	4.82E-02	3.18E-02	rs73964403	18_67212380_C_G	0.99	7164	2.55E-06 GCST90277348
Sphingomyelin (d36:2) levels	20	7958929	T	G	-1.02E-01	2.25E-02	1.70E-01	rs2876109	20_7958929_G_T	0.98	7164	6.32E-06 GCST90277348
Sphingomyelin (d38:1) levels	1	4046086	A	G	1.69E-01	3.57E-02	6.95E-02	rs10915321	1_4046086_G_A	0.85	7045	2.38E-06 GCST90277349
Sphingomyelin (d38:1) levels	2	11870210	T	C	1.84E-01	3.97E-02	5.16E-02	rs74899875	2_11870210_C_T	0.93	7045	3.60E-06 GCST90277349
Sphingomyelin (d38:1) levels	2	13358463	A	G	3.46E-01	7.18E-02	1.60E-02	rs78564380	2_13358463_G_A	0.88	7045	1.46E-06 GCST90277349
Sphingomyelin (d38:1) levels	2	27508073	C	T	-8.30E-02	1.76E-02	6.51E-01	rs1260326	2_27508073_T_C	1.00	7045	2.56E-06 GCST90277349
Sphingomyelin (d38:1) levels	3	118500064	T	G	-1.43E-01	3.03E-02	9.12E-01	rs2946819	3_118500064_G_T	0.99	7045	2.38E-06 GCST90277349
Sphingomyelin (d38:1) levels	3	133061273	G	A	-7.58E-02	1.69E-02	5.58E-01	rs9837048	3_133061273_A_G	1.00	7045	7.59E-06 GCST90277349
Sphingomyelin (d38:1) levels	5	5149248	T	A	-1.27E-01	2.78E-02	1.06E-01	rs16874868	5_5149248_A_T	1.00	7045	4.62E-06 GCST90277349
Sphingomyelin (d38:1) levels	6	67129565	T	A	-8.27E-02	1.82E-02	3.25E-01	rs2503980	6_67129565_A_T	1.00	7045	5.68E-06 GCST90277349
Sphingomyelin (d38:1) levels	6	85323731	T	G	7.89E-01	1.78E-01	2.48E-03	rs151000252	6_85323731_G_T	0.92	7045	9.22E-06 GCST90277349

Sphingomyelin (d38:1) levels	6	138010882	T	C	1.47E-01	3.28E-02	7.44E-02	rs112314129	6_138010882_C_T	0.95	7045	7.26E-06	GCST90277349
Sphingomyelin (d38:1) levels	7	43504908	G	A	9.15E-02	2.04E-02	2.14E-01	rs17151028	7_43504908_A_G	1.00	7045	7.40E-06	GCST90277349
Sphingomyelin (d38:1) levels	7	146504425	A	G	-4.22E-01	8.70E-02	1.02E-02	rs7779208	7_146504425_G_A	0.92	7045	1.22E-06	GCST90277349
Sphingomyelin (d38:1) levels	8	22695176	C	G	-7.64E-02	1.72E-02	4.32E-01	rs7009708	8_22695176_G_C	0.98	7045	9.35E-06	GCST90277349
Sphingomyelin (d38:1) levels	9	92627750	A	G	-4.76E-01	1.02E-01	7.05E-03	rs140764699	9_92627750_G_A	0.97	7045	3.08E-06	GCST90277349
Sphingomyelin (d38:1) levels	11	944466	G	A	-9.84E-02	2.17E-02	8.14E-01	rs10751670	11_944466_A_G	1.00	7045	5.99E-06	GCST90277349
Sphingomyelin (d38:1) levels	11	61803876	G	C	1.90E-01	1.73E-02	3.84E-01	rs174548	11_61803876_C_G	1.00	7045	1.12E-27	GCST90277349
Sphingomyelin (d38:1) levels	11	116888939	A	G	1.36E-01	2.23E-02	1.74E-01	rs17174502	11_116888939_G_A	0.99	7045	1.14E-09	GCST90277349
Sphingomyelin (d38:1) levels	15	57610033	A	C	8.73E-02	1.72E-02	4.18E-01	rs9920308	15_57610033_C_A	1.00	7045	4.26E-07	GCST90277349
Sphingomyelin (d38:1) levels	15	58391167	G	A	-2.07E-01	1.68E-02	5.73E-01	rs1532085	15_58391167_A_G	1.00	7045	3.29E-34	GCST90277349
Sphingomyelin (d38:1) levels	15	58431740	A	G	2.59E-01	1.92E-02	2.67E-01	rs2070895	15_58431740_G_A	0.98	7045	3.11E-41	GCST90277349
Sphingomyelin (d38:1) levels	15	58545523	A	G	1.35E-01	2.17E-02	1.91E-01	rs11633043	15_58545523_G_A	0.97	7045	4.34E-10	GCST90277349
Sphingomyelin (d38:1) levels	15	59047450	C	G	1.94E-01	3.40E-02	6.48E-02	rs112129861	15_59047450_G_C	1.00	7045	1.08E-08	GCST90277349
Sphingomyelin (d38:1) levels	17	65074311	A	G	8.06E-02	1.77E-02	3.98E-01	rs11651494	17_65074311_G_A	0.95	7045	5.58E-06	GCST90277349
Sphingomyelin (d38:1) levels	17	77018065	C	T	1.36E-01	2.85E-02	9.91E-02	rs72880096	17_77018065_T_C	1.00	7045	1.75E-06	GCST90277349
Sphingomyelin (d38:1) levels	20	45958142	T	G	-3.05E-01	6.83E-02	1.59E-02	rs139689930	20_45958142_G_T	0.98	7045	7.95E-06	GCST90277349
Sphingomyelin (d38:2) levels	1	66604062	A	G	1.85E-01	4.14E-02	5.81E-02	rs115261885	1_66604062_G_A	0.97	5430	7.78E-06	GCST90277350
Sphingomyelin (d38:2) levels	1	110525431	C	T	1.90E-01	3.99E-02	6.58E-02	rs78655391	1_110525431_T_C	0.96	5430	1.97E-06	GCST90277350
Sphingomyelin (d38:2) levels	1	110840034	T	C	-5.91E-01	1.33E-01	6.05E-03	rs78816612	1_110840034_C_T	0.87	5430	9.31E-06	GCST90277350
Sphingomyelin (d38:2) levels	2	42197717	A	G	2.73E-01	5.65E-02	2.94E-02	rs12997068	2_42197717_G_A	0.97	5430	1.36E-06	GCST90277350
Sphingomyelin (d38:2) levels	3	182462564	T	A	1.62E-01	3.49E-02	9.76E-02	rs62294904	3_182462564_A_T	0.87	5430	3.30E-06	GCST90277350
Sphingomyelin (d38:2) levels	4	28423082	C	T	-3.56E-01	7.94E-02	1.70E-02	rs35485513	4_28423082_T_C	0.93	5430	7.74E-06	GCST90277350
Sphingomyelin (d38:2) levels	5	73116953	A	T	9.08E-02	1.96E-02	4.03E-01	rs602307	5_73116953_T_A	0.99	5430	3.87E-06	GCST90277350
Sphingomyelin (d38:2) levels	6	10113293	G	A	-9.75E-02	2.16E-02	3.00E-01	rs10949554	6_10113293_A_G	0.93	5430	6.67E-06	GCST90277350
Sphingomyelin (d38:2) levels	8	54198654	G	C	-9.18E-02	1.95E-02	5.88E-01	rs311415	8_54198654_C_G	1.00	5430	2.65E-06	GCST90277350
Sphingomyelin (d38:2) levels	8	104150496	G	A	4.59E-01	1.04E-01	9.92E-03	rs71520868	8_104150496_A_G	0.95	5430	9.92E-06	GCST90277350
Sphingomyelin (d38:2) levels	8	121336542	T	C	-5.64E-01	1.26E-01	6.91E-03	rs111680982	8_121336542_C_T	0.86	5430	8.44E-06	GCST90277350
Sphingomyelin (d38:2) levels	11	61779120	G	A	1.49E-01	1.93E-02	4.14E-01	rs174530	11_61779120_A_G	1.00	5430	1.33E-14	GCST90277350
Sphingomyelin (d38:2) levels	13	27121538	C	T	5.64E-01	1.27E-01	6.24E-03	rs111761575	13_27121538_T_C	0.99	5430	8.76E-06	GCST90277350
Sphingomyelin (d38:2) levels	13	42237414	A	T	9.82E-02	1.98E-02	4.10E-01	rs9590678	13_42237414_T_A	0.97	5430	7.54E-07	GCST90277350
Sphingomyelin (d38:2) levels	15	69696241	T	C	2.73E-01	6.15E-02	2.72E-02	rs145863254	15_69696241_C_T	0.93	5430	9.08E-06	GCST90277350
Sphingomyelin (d38:2) levels	16	79902520	G	C	3.69E-01	8.13E-02	1.37E-02	rs75480840	16_79902520_C_G	0.96	5430	5.87E-06	GCST90277350
Sphingomyelin (d38:2) levels	19	55226813	A	G	-1.26E-01	2.77E-02	1.49E-01	rs3826884	19_55226813_G_A	0.99	5430	5.24E-06	GCST90277350
Sphingomyelin (d38:2) levels	22	38910264	C	G	-1.03E-01	2.09E-02	3.22E-01	rs5757363	22_38910264_G_C	0.96	5430	7.93E-07	GCST90277350
Sphingomyelin (d40:1) levels	1	119172555	T	C	-9.82E-02	2.21E-02	1.72E-01	rs143745495	1_119172555_C_T	1.00	7169	8.71E-06	GCST90277351
Sphingomyelin (d40:1) levels	1	163979236	C	T	-1.27E-01	2.76E-02	1.06E-01	rs10494405	1_163979236_T_C	0.97	7169	4.62E-06	GCST90277351
Sphingomyelin (d40:1) levels	1	201955621	C	G	8.99E-02	1.84E-02	2.91E-01	rs3820438	1_201955621_G_C	0.98	7169	1.03E-06	GCST90277351
Sphingomyelin (d40:1) levels	2	232520315	A	G	1.13E-01	2.27E-02	8.38E-01	rs2573210	2_232520315_G_A	0.98	7169	6.34E-07	GCST90277351
Sphingomyelin (d40:1) levels	4	52717255	C	T	2.59E-01	5.67E-02	2.23E-02	rs11725298	4_52717255_T_C	0.96	7169	4.96E-06	GCST90277351
Sphingomyelin (d40:1) levels	4	183919435	C	G	8.47E-02	1.72E-02	5.89E-01	rs13123798	4_183919435_G_C	0.93	7169	8.11E-07	GCST90277351
Sphingomyelin (d40:1) levels	5	74678000	T	C	9.66E-02	2.11E-02	1.93E-01	rs7445920	5_74678000_C_T	0.99	7169	4.82E-06	GCST90277351
Sphingomyelin (d40:1) levels	6	31347332	C	T	8.35E-02	1.71E-02	3.91E-01	rs9265944	6_31347332_T_C	0.99	7169	1.13E-06	GCST90277351
Sphingomyelin (d40:1) levels	7	8811155	T	C	8.50E-02	1.79E-02	3.19E-01	rs1034755	7_8811155_C_T	0.98	7169	2.13E-06	GCST90277351
Sphingomyelin (d40:1) levels	7	80837985	G	C	1.82E-01	3.98E-02	4.73E-02	rs10487877	7_80837985_C_G	0.98	7169	5.12E-06	GCST90277351
Sphingomyelin (d40:1) levels	7	94419222	G	T	-1.51E-01	3.10E-02	8.00E-02	rs10234951	7_94419222_T_G	0.99	7169	1.17E-06	GCST90277351
Sphingomyelin (d40:1) levels	7	138155111	A	T	-2.94E-01	6.08E-02	1.95E-02	rs79337987	7_138155111_T_A	0.97	7169	1.32E-06	GCST90277351
Sphingomyelin (d40:1) levels	8	1843472	G	T	-8.05E-02	1.71E-02	6.02E-01	rs11136432	8_1843472_T_G	0.98	7169	2.74E-06	GCST90277351
Sphingomyelin (d40:1) levels	8	8390548	A	G	8.58E-02	1.78E-02	3.34E-01	rs6601694	8_8390548_G_A	0.99	7169	1.47E-06	GCST90277351
Sphingomyelin (d40:1) levels	11	61856709	C	T	2.10E-01	1.69E-02	5.80E-01	rs97384	11_61856709_T_C	0.99	7169	4.34E-35	GCST90277351
Sphingomyelin (d40:1) levels	11	118311945	C	T	8.04E-02	1.78E-02	3.26E-01	rs2277289	11_118311945_T_C	1.00	7169	6.69E-06	GCST90277351
Sphingomyelin (d40:1) levels	12	121783011	A	G	1.46E-01	3.05E-02	8.30E-02	rs74677222	12_121783011_G_A	0.98	7169	1.79E-06	GCST90277351
Sphingomyelin (d40:1) levels	15	58431740	A	G	8.50E-02	1.92E-02	2.67E-01	rs2070895	15_58431740_G_A	0.98	7169	9.89E-06	GCST90277351
Sphingomyelin (d40:1) levels	16	56955918	A	G	-7.49E-02	1.68E-02	4.57E-01	rs12923459	16_56955918_G_A	0.99	7169	8.79E-06	GCST90277351
Sphingomyelin (d40:1) levels	18	4552061	G	T	-7.17E-01	1.51E-01	3.47E-03	rs9946720	18_4552061_T_G	0.89	7169	1.93E-06	GCST90277351
Sphingomyelin (d40:1) levels	18	77015662	T	C	-8.40E-02	1.86E-02	2.79E-01	rs7232502	18_77015662_C_T	0.98	7169	6.81E-06	GCST90277351
Sphingomyelin (d40:1) levels	19	44983921	T	C	-8.41E-02	1.71E-02	5.98E-01	rs204474	19_44983921_C_T	0.99	7169	9.15E-07	GCST90277351
Sphingomyelin (d40:1) levels	19	55226813	A	G	-1.79E-01	2.36E-02	1.49E-01	rs3826884	19_55226813_G_A	0.99	7169	3.56E-14	GCST90277351

Sphingomyelin (d40:1) levels	20	37995099	A	G	1.48E-01	3.33E-02	6.74E-02	rs78802873	20_37995099_G_A	1.00	7169	8.88E-06	GCST90277351
Sphingomyelin (d40:2) levels	1	39624750	G	A	-6.34E-01	1.27E-01	5.74E-03	rs41264497	1_39624750_A_G	0.97	5609	5.96E-07	GCST90277352
Sphingomyelin (d40:2) levels	1	98370123	T	C	-1.20E-01	2.66E-02	1.48E-01	rs55892660	1_98370123_C_T	0.99	5609	7.05E-06	GCST90277352
Sphingomyelin (d40:2) levels	2	62271952	T	G	2.43E-01	5.49E-02	3.08E-02	rs115002881	2_62271952_G_T	0.99	5609	9.44E-06	GCST90277352
Sphingomyelin (d40:2) levels	2	140761432	G	C	-5.27E-01	1.15E-01	6.66E-03	rs10187082	2_140761432_C_G	0.99	5609	4.40E-06	GCST90277352
Sphingomyelin (d40:2) levels	2	229625649	G	A	3.11E-01	6.80E-02	2.36E-02	rs72993205	2_229625649_A_G	0.86	5609	5.07E-06	GCST90277352
Sphingomyelin (d40:2) levels	3	60777864	C	T	1.54E-01	3.27E-02	9.21E-02	rs4974228	3_60777864_T_C	1.00	5609	2.43E-06	GCST90277352
Sphingomyelin (d40:2) levels	3	191170774	C	T	-1.04E-01	2.31E-02	2.09E-01	rs73199626	3_191170774_T_C	0.98	5609	6.31E-06	GCST90277352
Sphingomyelin (d40:2) levels	3	193209944	A	G	2.71E-01	5.99E-02	2.70E-02	rs115536895	3_193209944_G_A	0.97	5609	6.09E-06	GCST90277352
Sphingomyelin (d40:2) levels	5	32055261	C	G	1.81E-01	4.09E-02	6.21E-02	rs150942220	5_32055261_G_C	0.90	5609	9.64E-06	GCST90277352
Sphingomyelin (d40:2) levels	5	42470628	G	A	1.33E-01	2.92E-02	1.15E-01	rs2940919	5_42470628_A_G	1.00	5609	5.21E-06	GCST90277352
Sphingomyelin (d40:2) levels	5	107567832	C	G	5.79E-01	1.29E-01	5.41E-03	rs116250572	5_107567832_G_C	0.90	5609	6.85E-06	GCST90277352
Sphingomyelin (d40:2) levels	6	85404250	C	T	-8.73E-02	1.97E-02	3.85E-01	rs7761485	6_85404250_T_C	1.00	5609	9.05E-06	GCST90277352
Sphingomyelin (d40:2) levels	7	133098676	C	G	-3.09E-01	6.69E-02	2.12E-02	rs190716231	7_133098676_G_C	0.97	5609	3.97E-06	GCST90277352
Sphingomyelin (d40:2) levels	8	62982677	T	C	3.23E-01	7.22E-02	1.91E-02	rs76268189	8_62982677_C_T	0.94	5609	8.04E-06	GCST90277352
Sphingomyelin (d40:2) levels	9	114255513	T	C	9.34E-02	2.00E-02	3.74E-01	rs7028232	9_114255513_C_T	0.98	5609	2.95E-06	GCST90277352
Sphingomyelin (d40:2) levels	10	109515997	T	C	-3.91E-01	8.60E-02	1.17E-02	rs111698799	10_109515997_C_T	0.97	5609	5.47E-06	GCST90277352
Sphingomyelin (d40:2) levels	19	41215233	A	G	-3.13E-01	6.98E-02	1.95E-02	rs116937344	19_41215233_G_A	0.95	5609	7.36E-06	GCST90277352
Sphingomyelin (d42:2) levels	1	153820721	T	C	6.38E-01	1.43E-01	3.68E-03	rs182992622	1_153820721_C_T	0.93	6870	8.01E-06	GCST90277353
Sphingomyelin (d42:2) levels	1	206738030	A	G	-1.22E-01	2.53E-02	1.34E-01	rs76085699	1_206738030_G_A	0.98	6870	1.42E-06	GCST90277353
Sphingomyelin (d42:2) levels	1	240448048	T	G	-8.60E-02	1.89E-02	6.98E-01	rs10926263	1_240448048_G_T	0.98	6870	5.29E-06	GCST90277353
Sphingomyelin (d42:2) levels	1	242074452	T	C	-2.50E-01	5.48E-02	2.53E-02	rs149968069	1_242074452_C_T	0.95	6870	5.34E-06	GCST90277353
Sphingomyelin (d42:2) levels	2	204509075	G	A	3.34E-01	7.06E-02	1.57E-02	rs111703312	2_204509075_A_G	0.94	6870	2.19E-06	GCST90277353
Sphingomyelin (d42:2) levels	3	12470153	A	G	-7.78E-02	1.70E-02	4.73E-01	rs709166	3_12470153_G_A	1.00	6870	5.08E-06	GCST90277353
Sphingomyelin (d42:2) levels	3	181603334	G	A	-5.87E-01	1.24E-01	5.16E-03	rs187748847	3_181603334_A_G	0.93	6870	2.29E-06	GCST90277353
Sphingomyelin (d42:2) levels	4	18851049	A	G	-1.67E-01	3.73E-02	5.75E-02	rs4696957	4_18851049_G_A	0.94	6870	7.14E-06	GCST90277353
Sphingomyelin (d42:2) levels	4	74887304	A	C	-2.10E-01	4.36E-02	4.24E-02	rs55683433	4_74887304_C_A	0.94	6870	1.52E-06	GCST90277353
Sphingomyelin (d42:2) levels	4	92558738	G	C	1.51E-01	3.04E-02	8.80E-02	rs17019568	4_92558738_C_G	1.00	6870	7.10E-07	GCST90277353
Sphingomyelin (d42:2) levels	4	145707221	A	G	-1.67E-01	3.48E-02	6.66E-02	rs28844909	4_145707221_G_A	0.95	6870	1.69E-06	GCST90277353
Sphingomyelin (d42:2) levels	4	151270229	G	A	-1.43E-01	2.89E-02	9.98E-02	rs11936543	4_151270229_A_G	0.97	6870	7.70E-07	GCST90277353
Sphingomyelin (d42:2) levels	7	17697283	G	C	9.14E-02	2.05E-02	2.14E-01	rs17138200	7_17697283_C_G	1.00	6870	8.84E-06	GCST90277353
Sphingomyelin (d42:2) levels	7	81690761	C	T	9.84E-02	2.22E-02	1.84E-01	rs68103316	7_81690761_T_C	0.97	6870	9.14E-06	GCST90277353
Sphingomyelin (d42:2) levels	8	14334458	T	C	-1.20E-01	2.50E-02	1.39E-01	rs7003304	8_14334458_C_T	0.99	6870	1.54E-06	GCST90277353
Sphingomyelin (d42:2) levels	8	22936374	C	T	8.24E-02	1.85E-02	3.10E-01	rs9644026	8_22936374_T_C	1.00	6870	9.06E-06	GCST90277353
Sphingomyelin (d42:2) levels	8	114265886	C	T	2.47E-01	5.49E-02	2.56E-02	rs118048608	8_114265886_T_C	1.00	6870	6.92E-06	GCST90277353
Sphingomyelin (d42:2) levels	9	1911291	T	G	-1.07E-01	2.25E-02	1.95E-01	rs10811102	9_1911291_G_T	0.91	6870	2.28E-06	GCST90277353
Sphingomyelin (d42:2) levels	11	61803311	C	T	1.94E-01	1.73E-02	4.07E-01	rs174547	11_61803311_T_C	1.00	6870	3.60E-29	GCST90277353
Sphingomyelin (d42:2) levels	12	130607421	C	T	9.34E-02	1.73E-02	4.34E-01	rs10848158	12_130607421_T_C	0.97	6870	7.17E-08	GCST90277353
Sphingomyelin (d42:2) levels	15	58391167	G	A	-8.08E-02	1.72E-02	5.73E-01	rs1532085	15_58391167_A_G	1.00	6870	2.64E-06	GCST90277353
Sphingomyelin (d42:2) levels	15	58434545	C	G	-1.04E-01	2.00E-02	7.58E-01	rs261334	15_58434545_G_C	1.00	6870	1.91E-07	GCST90277353
Sphingomyelin (d42:2) levels	16	24297504	T	C	-8.05E-02	1.76E-02	4.23E-01	rs9652580	16_24297504_C_T	0.98	6870	5.02E-06	GCST90277353
Sphingomyelin (d42:2) levels	17	70147293	G	A	-1.29E-01	2.90E-02	9.46E-02	rs28415525	17_70147293_A_G	1.00	6870	8.81E-06	GCST90277353
Sphingomyelin (d42:2) levels	18	35300850	A	G	6.97E-01	1.43E-01	3.70E-03	rs117511403	18_35300850_G_A	0.99	6870	1.12E-06	GCST90277353
Sphingomyelin (d42:2) levels	19	44710429	T	C	1.02E-01	2.18E-02	1.87E-01	rs57684876	19_44710429_C_T	0.99	6870	2.64E-06	GCST90277353
Sphingomyelin (d42:2) levels	19	44921921	A	G	2.45E-01	5.09E-02	3.02E-02	rs190712692	19_44921921_G_A	0.96	6870	1.51E-06	GCST90277353
Sphingomyelin (d42:2) levels	20	45423037	T	C	1.57E-01	3.52E-02	6.60E-02	rs78605935	20_45423037_C_T	0.95	6870	8.09E-06	GCST90277353
Triacylglycerol (46:1) levels	1	35503298	G	A	9.07E-02	2.02E-02	2.40E-01	rs1203148	1_35503298_A_G	0.93	7172	7.34E-06	GCST90277354
Triacylglycerol (46:1) levels	1	107442367	T	C	-3.79E-01	8.54E-02	1.07E-02	rs114325590	1_107442367_C_T	0.89	7172	9.41E-06	GCST90277354
Triacylglycerol (46:1) levels	1	201955621	C	G	8.19E-02	1.84E-02	2.91E-01	rs3820438	1_201955621_G_C	0.98	7172	8.42E-06	GCST90277354
Triacylglycerol (46:1) levels	1	244982806	T	C	1.26E-01	2.51E-02	1.25E-01	rs72635955	1_244982806_C_T	1.00	7172	5.16E-07	GCST90277354
Triacylglycerol (46:1) levels	2	59638953	G	A	-1.46E-01	2.95E-02	8.93E-02	rs71420341	2_59638953_A_G	0.99	7172	7.54E-07	GCST90277354
Triacylglycerol (46:1) levels	2	123647385	C	T	8.00E-02	1.70E-02	4.29E-01	rs1513795	2_123647385_T_C	1.00	7172	2.47E-06	GCST90277354
Triacylglycerol (46:1) levels	4	52717255	C	T	2.56E-01	5.67E-02	2.23E-02	rs11725298	4_52717255_T_C	0.96	7172	6.50E-06	GCST90277354
Triacylglycerol (46:1) levels	4	170720084	A	G	1.22E-01	2.71E-02	1.14E-01	rs10520189	4_170720084_G_A	0.93	7172	7.55E-06	GCST90277354
Triacylglycerol (46:1) levels	7	94419222	G	T	-1.49E-01	3.11E-02	8.00E-02	rs10234951	7_94419222_T_G	0.99	7172	1.69E-06	GCST90277354
Triacylglycerol (46:1) levels	7	106360522	G	C	-3.61E-01	7.91E-02	9.87E-01	rs2705007	7_106360522_C_G	0.89	7172	5.12E-06	GCST90277354
Triacylglycerol (46:1) levels	8	8390548	A	G	8.75E-02	1.78E-02	3.34E-01	rs6601694	8_8390548_G_A	0.99	7172	8.85E-07	GCST90277354

Triacylglycerol (46:1) levels	8	54198249	T	C	-7.93E-02	1.76E-02	6.49E-01	rs311416	8_54198249_C_T	1.00	7172	6.58E-06	GCST90277354
Triacylglycerol (46:1) levels	8	69966863	A	G	1.05E-01	2.33E-02	1.60E-01	rs13269712	8_69966863_G_A	0.97	7172	6.82E-06	GCST90277354
Triacylglycerol (46:1) levels	9	14928596	C	T	7.50E-02	1.68E-02	5.51E-01	rs10756627	9_14928596_T_C	0.99	7172	8.04E-06	GCST90277354
Triacylglycerol (46:1) levels	9	130943915	T	C	2.91E-01	6.53E-02	1.81E-02	rs117002537	9_130943915_C_T	0.91	7172	8.52E-06	GCST90277354
Triacylglycerol (46:1) levels	11	61770929	C	G	-7.89E-02	1.71E-02	4.44E-01	rs174527	11_61770929_G_C	0.98	7172	3.97E-06	GCST90277354
Triacylglycerol (46:1) levels	11	61784455	C	A	-2.51E-01	1.68E-02	4.08E-01	rs174536	11_61784455_A_C	1.00	7172	1.09E-49	GCST90277354
Triacylglycerol (46:1) levels	11	118311945	C	T	9.11E-02	1.78E-02	3.26E-01	rs2277289	11_118311945_T_C	1.00	7172	3.31E-07	GCST90277354
Triacylglycerol (46:1) levels	12	2630538	A	G	2.55E-01	5.04E-02	3.39E-02	rs11062287	12_2630538_G_A	0.83	7172	4.45E-07	GCST90277354
Triacylglycerol (46:1) levels	12	76255614	T	C	-2.25E-01	4.82E-02	3.23E-02	rs113913626	12_76255614_C_T	0.94	7172	3.27E-06	GCST90277354
Triacylglycerol (46:1) levels	13	75596558	T	G	1.79E-01	3.89E-02	5.04E-02	rs17703316	13_75596558_G_T	0.97	7172	4.25E-06	GCST90277354
Triacylglycerol (46:1) levels	15	96625932	A	G	7.73E-02	1.72E-02	3.93E-01	rs4525452	15_96625932_G_A	0.98	7172	7.24E-06	GCST90277354
Triacylglycerol (46:1) levels	16	5924670	T	C	1.75E-01	3.77E-02	5.32E-02	rs117565387	16_5924670_C_T	0.96	7172	3.51E-06	GCST90277354
Triacylglycerol (46:1) levels	16	76463097	C	T	-1.40E-01	2.96E-02	8.70E-02	rs35427954	16_76463097_T_C	1.00	7172	2.30E-06	GCST90277354
Triacylglycerol (46:1) levels	18	27805383	ACT	A	4.02E-01	9.06E-02	8.45E-03	rs145926488	18_27805383_A_ACT	1.00	7172	9.11E-06	GCST90277354
Triacylglycerol (46:1) levels	18	44272134	G	T	-8.74E-02	1.93E-02	7.55E-01	rs1456608	18_44272134_T_G	1.00	7172	5.93E-06	GCST90277354
Triacylglycerol (46:1) levels	19	44923535	A	G	2.50E-01	5.01E-02	3.03E-02	rs141622900	19_44923535_G_A	0.96	7172	6.52E-07	GCST90277354
Triacylglycerol (46:1) levels	19	44987378	C	T	-9.53E-02	1.70E-02	5.95E-01	rs204468	19_44987378_T_C	1.00	7172	2.35E-08	GCST90277354
Triacylglycerol (46:1) levels	22	36714714	A	G	-1.12E-01	2.36E-02	1.46E-01	rs41376249	22_36714714_G_A	0.99	7172	2.08E-06	GCST90277354
Triacylglycerol (46:2) levels	1	9264154	T	C	1.61E-01	3.09E-02	8.43E-02	rs17368528	1_9264154_C_T	1.00	6717	2.04E-07	GCST90277355
Triacylglycerol (46:2) levels	1	167181238	A	G	2.09E-01	4.63E-02	3.97E-02	rs148727465	1_167181238_G_A	0.92	6717	6.67E-06	GCST90277355
Triacylglycerol (46:2) levels	1	224192928	T	C	1.38E-01	2.77E-02	1.12E-01	rs112243303	1_224192928_C_T	0.95	6717	5.86E-07	GCST90277355
Triacylglycerol (46:2) levels	1	241342846	G	C	-3.71E-01	8.35E-02	1.09E-02	rs116075306	1_241342846_C_G	0.93	6717	9.30E-06	GCST90277355
Triacylglycerol (46:2) levels	1	245122916	T	C	-2.61E-01	5.18E-02	2.91E-02	rs79878108	1_245122916_C_T	0.98	6717	5.08E-07	GCST90277355
Triacylglycerol (46:2) levels	2	12232463	G	A	1.18E-01	2.45E-02	1.51E-01	rs35253870	2_12232463_A_G	0.98	6717	1.39E-06	GCST90277355
Triacylglycerol (46:2) levels	2	14315428	C	T	-2.34E-01	5.16E-02	3.03E-02	rs112779958	2_14315428_T_C	0.96	6717	6.07E-06	GCST90277355
Triacylglycerol (46:2) levels	2	63521077	A	G	7.18E-01	1.52E-01	3.01E-03	rs192913427	2_63521077_G_A	0.96	6717	2.42E-06	GCST90277355
Triacylglycerol (46:2) levels	2	85141220	CAG	C	8.78E-02	1.93E-02	7.24E-01	rs199501971	2_85141220_C_CAG	0.99	6717	5.76E-06	GCST90277355
Triacylglycerol (46:2) levels	2	120398486	A	G	-9.80E-02	2.05E-02	2.29E-01	rs6749797	2_120398486_G_A	1.00	6717	1.74E-06	GCST90277355
Triacylglycerol (46:2) levels	2	218075695	T	C	1.02E-01	2.27E-02	1.86E-01	rs4674246	2_218075695_C_T	0.96	6717	7.63E-06	GCST90277355
Triacylglycerol (46:2) levels	3	236587	G	A	-8.97E-02	1.87E-02	6.93E-01	rs12496575	3_236587_A_G	1.00	6717	1.65E-06	GCST90277355
Triacylglycerol (46:2) levels	3	39235168	G	C	-1.72E-01	3.18E-02	8.15E-02	rs2271489	3_39235168_C_G	0.99	6717	6.67E-08	GCST90277355
Triacylglycerol (46:2) levels	5	141645323	TAGC	T	-8.84E-02	1.97E-02	2.56E-01	rs377255467	5_141645323_T_TAGC	1.00	6717	7.42E-06	GCST90277355
Triacylglycerol (46:2) levels	5	180049450	C	T	5.76E-01	1.30E-01	4.50E-03	rs116140603	5_180049450_T_C	0.97	6717	9.21E-06	GCST90277355
Triacylglycerol (46:2) levels	7	55161850	C	T	1.15E-01	2.53E-02	1.36E-01	rs3752651	7_55161850_T_C	0.99	6717	5.07E-06	GCST90277355
Triacylglycerol (46:2) levels	8	112417407	T	C	-1.19E-01	2.50E-02	1.39E-01	rs13257713	8_112417407_C_T	0.99	6717	2.06E-06	GCST90277355
Triacylglycerol (46:2) levels	9	2495519	G	A	4.89E-01	1.10E-01	6.47E-03	rs117049823	9_2495519_A_G	0.99	6717	8.55E-06	GCST90277355
Triacylglycerol (46:2) levels	9	102668496	A	G	5.19E-01	1.06E-01	6.89E-03	rs140484398	9_102668496_G_A	0.98	6717	1.02E-06	GCST90277355
Triacylglycerol (46:2) levels	11	732354	C	T	-1.81E-01	4.06E-02	4.92E-02	rs150046733	11_732354_T_C	0.97	6717	8.64E-06	GCST90277355
Triacylglycerol (46:2) levels	12	62005843	A	G	8.31E-02	1.85E-02	3.18E-01	rs11174276	12_62005843_G_A	0.99	6717	7.22E-06	GCST90277355
Triacylglycerol (46:2) levels	15	58431476	T	C	9.53E-02	2.00E-02	2.58E-01	rs1800588	15_58431476_C_T	0.99	6717	1.94E-06	GCST90277355
Triacylglycerol (46:2) levels	19	6934007	T	C	-2.07E-01	4.32E-02	4.27E-02	rs28606541	19_6934007_C_T	0.99	6717	1.71E-06	GCST90277355
Triacylglycerol (46:2) levels	19	44702607	C	T	1.06E-01	2.18E-02	1.89E-01	rs8108277	19_44702607_T_C	1.00	6717	1.13E-06	GCST90277355
Triacylglycerol (48:0) levels	2	216708722	T	C	-1.04E-01	2.20E-02	2.06E-01	rs34683829	2_216708722_C_T	0.98	6337	2.36E-06	GCST90277356
Triacylglycerol (48:0) levels	3	114457667	G	A	-2.71E-01	6.12E-02	2.25E-02	rs79087995	3_114457667_A_G	0.99	6337	9.55E-06	GCST90277356
Triacylglycerol (48:0) levels	4	151270229	G	A	-1.36E-01	3.02E-02	9.98E-02	rs11936543	4_151270229_A_G	0.97	6337	6.52E-06	GCST90277356
Triacylglycerol (48:0) levels	5	178795278	A	G	-5.64E-01	1.25E-01	5.74E-03	rs9686220	5_178795278_G_A	0.82	6337	6.50E-06	GCST90277356
Triacylglycerol (48:0) levels	8	139128322	T	A	-6.86E-01	1.44E-01	4.48E-03	rs7459809	8_139128322_A_T	0.88	6337	2.08E-06	GCST90277356
Triacylglycerol (48:0) levels	10	67993863	T	C	-1.07E-01	2.35E-02	1.90E-01	rs497849	10_67993863_C_T	0.92	6337	5.60E-06	GCST90277356
Triacylglycerol (48:0) levels	10	92455257	CTTGA	C	-1.09E-01	2.46E-02	1.54E-01	rs201503938	10_92455257_C_CTTGA	0.98	6337	9.62E-06	GCST90277356
Triacylglycerol (48:0) levels	10	119656173	A	G	9.49E-02	2.13E-02	2.29E-01	rs72840788	10_119656173_G_A	0.99	6337	8.98E-06	GCST90277356
Triacylglycerol (48:0) levels	11	15301207	C	G	1.53E-01	3.06E-02	9.86E-02	rs4603290	11_15301207_G_C	0.95	6337	5.94E-07	GCST90277356
Triacylglycerol (48:0) levels	11	61835765	T	C	1.77E-01	2.02E-02	2.58E-01	rs2727270	11_61835765_C_T	1.00	6337	2.43E-18	GCST90277356
Triacylglycerol (48:0) levels	12	109070443	T	C	5.40E-01	1.14E-01	7.07E-03	rs79998109	12_109070443_C_T	0.89	6337	2.24E-06	GCST90277356
Triacylglycerol (48:0) levels	13	108844720	AT	A	8.06E-02	1.81E-02	5.62E-01	rs76400690	13_108844720_A_AT	0.98	6337	9.06E-06	GCST90277356
Triacylglycerol (48:0) levels	16	14645161	T	A	-4.92E-01	1.10E-01	7.47E-03	rs181750334	16_14645161_A_T	0.91	6337	8.28E-06	GCST90277356
Triacylglycerol (48:0) levels	16	75515447	G	C	-8.50E-02	1.80E-02	4.14E-01	rs2550882	16_75515447_C_G	0.99	6337	2.36E-06	GCST90277356
Triacylglycerol (48:0) levels	17	4999663	A	G	-9.33E-02	2.01E-02	7.31E-01	rs238237	17_4999663_G_A	0.99	6337	3.70E-06	GCST90277356

Triacylglycerol (48:0) levels	17	76481609	T	C	-8.25E-02	1.83E-02	4.20E-01	rs11870168	17_76481609_C_T	0.98	6337	6.62E-06	GCST90277356
Triacylglycerol (48:0) levels	19	5868331	T	C	-2.59E-01	5.32E-02	3.13E-02	rs186111880	19_5868331_C_T	0.91	6337	1.16E-06	GCST90277356
Triacylglycerol (48:1) levels	1	119172555	T	C	-1.04E-01	2.21E-02	1.72E-01	rs143745495	1_119172555_C_T	1.00	7154	2.38E-06	GCST90277357
Triacylglycerol (48:1) levels	1	201955621	C	G	8.89E-02	1.84E-02	2.91E-01	rs3820438	1_201955621_G_C	0.98	7154	1.38E-06	GCST90277357
Triacylglycerol (48:1) levels	2	59638953	G	A	-1.38E-01	2.96E-02	8.93E-02	rs71420341	2_59638953_A_G	0.99	7154	3.15E-06	GCST90277357
Triacylglycerol (48:1) levels	2	106808276	G	T	-2.31E-01	5.11E-02	2.88E-02	rs72627458	2_106808276_T_G	0.97	7154	6.48E-06	GCST90277357
Triacylglycerol (48:1) levels	2	146766791	C	T	-2.47E-01	5.44E-02	2.49E-02	rs115069241	2_146766791_T_C	0.97	7154	5.54E-06	GCST90277357
Triacylglycerol (48:1) levels	3	5474437	G	A	-2.41E-01	5.39E-02	2.76E-02	rs2323249	3_5474437_A_G	0.90	7154	8.02E-06	GCST90277357
Triacylglycerol (48:1) levels	3	106127782	A	G	-8.03E-01	1.74E-01	2.75E-03	rs115623452	3_106127782_G_A	0.92	7154	4.14E-06	GCST90277357
Triacylglycerol (48:1) levels	3	132262400	T	C	-1.53E-01	3.28E-02	7.30E-02	rs61792775	3_132262400_C_T	0.97	7154	3.23E-06	GCST90277357
Triacylglycerol (48:1) levels	3	182336158	G	A	1.11E-01	2.51E-02	1.42E-01	rs55853808	3_182336158_A_G	0.92	7154	9.77E-06	GCST90277357
Triacylglycerol (48:1) levels	7	8811155	T	C	8.04E-02	1.80E-02	3.19E-01	rs1034755	7_8811155_C_T	0.98	7154	7.79E-06	GCST90277357
Triacylglycerol (48:1) levels	7	127303602	A	C	1.47E-01	3.22E-02	7.43E-02	rs17864186	7_127303602_C_A	0.98	7154	4.97E-06	GCST90277357
Triacylglycerol (48:1) levels	7	138155111	A	T	-2.76E-01	6.08E-02	1.95E-02	rs79337987	7_138155111_T_A	0.97	7154	5.74E-06	GCST90277357
Triacylglycerol (48:1) levels	8	8374175	G	A	9.25E-02	1.94E-02	2.47E-01	rs13280051	8_8374175_A_G	1.00	7154	1.88E-06	GCST90277357
Triacylglycerol (48:1) levels	8	69988547	A	G	1.03E-01	2.34E-02	1.56E-01	rs77754835	8_69988547_G_A	0.99	7154	9.77E-06	GCST90277357
Triacylglycerol (48:1) levels	9	81622002	C	A	7.89E-02	1.76E-02	6.00E-01	rs4877665	9_81622002_A_C	0.94	7154	7.61E-06	GCST90277357
Triacylglycerol (48:1) levels	10	36183817	T	C	2.67E-01	5.94E-02	2.31E-02	rs145522126	10_36183817_C_T	0.89	7154	6.81E-06	GCST90277357
Triacylglycerol (48:1) levels	11	56943668	A	G	1.02E-01	2.27E-02	1.66E-01	rs11522703	11_56943668_G_A	0.98	7154	7.85E-06	GCST90277357
Triacylglycerol (48:1) levels	11	61813163	T	C	-1.83E-01	1.72E-02	3.80E-01	rs174556	11_61813163_C_T	1.00	7154	4.16E-26	GCST90277357
Triacylglycerol (48:1) levels	13	34119843	A	G	-1.93E-01	4.33E-02	4.12E-02	rs9564073	13_34119843_G_A	0.94	7154	8.13E-06	GCST90277357
Triacylglycerol (48:1) levels	13	75596558	T	G	1.75E-01	3.90E-02	5.04E-02	rs17703316	13_75596558_G_T	0.97	7154	7.40E-06	GCST90277357
Triacylglycerol (48:1) levels	16	14645161	T	A	-5.37E-01	1.03E-01	7.47E-03	rs181750334	16_14645161_A_T	0.91	7154	1.91E-07	GCST90277357
Triacylglycerol (48:1) levels	16	75555515	A	C	-1.12E-01	2.48E-02	1.28E-01	rs80299509	16_75555515_C_A	1.00	7154	5.91E-06	GCST90277357
Triacylglycerol (48:1) levels	17	15393870	G	A	1.21E-01	2.41E-02	1.48E-01	rs71366199	17_15393870_A_G	0.95	7154	4.87E-07	GCST90277357
Triacylglycerol (48:1) levels	18	4552061	G	T	-7.18E-01	1.51E-01	3.47E-03	rs9946720	18_4552061_T_G	0.89	7154	1.91E-06	GCST90277357
Triacylglycerol (48:1) levels	18	46589554	C	T	1.19E-01	2.64E-02	1.14E-01	rs7228147	18_46589554_T_C	0.98	7154	6.68E-06	GCST90277357
Triacylglycerol (48:1) levels	19	44983921	T	C	-9.55E-02	1.71E-02	5.98E-01	rs204474	19_44983921_C_T	0.99	7154	2.58E-08	GCST90277357
Triacylglycerol (48:1) levels	20	47368051	C	T	-1.82E-01	4.04E-02	4.66E-02	rs141451859	20_47368051_T_C	0.95	7154	6.90E-06	GCST90277357
Triacylglycerol (48:2) levels	1	62710412	T	A	-8.55E-02	1.92E-02	2.49E-01	rs11208007	1_62710412_A_T	1.00	7157	8.47E-06	GCST90277358
Triacylglycerol (48:2) levels	2	10553201	A	G	1.03E-01	2.32E-02	8.40E-01	rs1918704	2_10553201_G_A	0.97	7157	9.15E-06	GCST90277358
Triacylglycerol (48:2) levels	2	45617902	A	T	-7.91E-02	1.74E-02	3.62E-01	rs10490343	2_45617902_T_A	1.00	7157	5.90E-06	GCST90277358
Triacylglycerol (48:2) levels	4	59111539	A	C	-7.78E-02	1.73E-02	3.78E-01	rs13113877	4_59111539_C_A	0.99	7157	7.13E-06	GCST90277358
Triacylglycerol (48:2) levels	4	80070812	G	A	-1.45E-01	3.25E-02	8.19E-02	rs79107687	4_80070812_A_G	0.89	7157	8.22E-06	GCST90277358
Triacylglycerol (48:2) levels	8	118730631	A	G	-1.19E-01	2.66E-02	8.82E-01	rs3103985	8_118730631_G_A	0.96	7157	8.20E-06	GCST90277358
Triacylglycerol (48:2) levels	10	52382881	A	G	1.68E-01	3.61E-02	6.00E-02	rs80310784	10_52382881_G_A	0.92	7157	3.44E-06	GCST90277358
Triacylglycerol (48:2) levels	10	62558401	T	C	2.43E-01	5.48E-02	2.46E-02	rs75678679	10_62558401_C_T	0.99	7157	9.53E-06	GCST90277358
Triacylglycerol (48:2) levels	11	61813163	T	C	9.97E-02	1.73E-02	3.80E-01	rs174556	11_61813163_C_T	1.00	7157	8.51E-09	GCST90277358
Triacylglycerol (48:2) levels	11	116778201	C	G	-1.23E-01	2.32E-02	8.49E-01	rs964184	11_116778201_G_C	1.00	7157	1.20E-07	GCST90277358
Triacylglycerol (48:2) levels	12	29000949	G	C	-2.93E-01	5.87E-02	2.17E-02	rs76902173	12_29000949_C_G	0.97	7157	5.81E-07	GCST90277358
Triacylglycerol (48:2) levels	13	73455183	T	C	-1.19E-01	2.50E-02	1.30E-01	rs9564991	13_73455183_C_T	1.00	7157	2.06E-06	GCST90277358
Triacylglycerol (48:2) levels	16	8941267	C	T	-7.73E-02	1.73E-02	4.06E-01	rs1471435	16_8941267_T_C	0.96	7157	7.98E-06	GCST90277358
Triacylglycerol (48:2) levels	16	51313598	G	C	7.34E-01	1.59E-01	3.15E-03	rs117226992	16_51313598_C_G	0.93	7157	3.78E-06	GCST90277358
Triacylglycerol (48:2) levels	18	48309616	C	T	7.60E-02	1.70E-02	4.12E-01	rs6507838	18_48309616_T_C	0.98	7157	7.44E-06	GCST90277358
Triacylglycerol (48:2) levels	18	49656294	G	A	9.95E-02	1.98E-02	7.57E-01	rs1540037	18_49656294_A_G	0.97	7157	5.07E-07	GCST90277358
Triacylglycerol (48:2) levels	19	54173068	C	T	-1.17E-01	1.71E-02	5.93E-01	rs641738	19_54173068_T_C	0.97	7157	9.74E-12	GCST90277358
Triacylglycerol (48:2) levels	22	47263264	T	C	1.65E-01	3.61E-02	5.95E-02	rs11704468	22_47263264_C_T	0.96	7157	5.05E-06	GCST90277358
Triacylglycerol (48:3) levels	1	62591465	C	T	-1.13E-01	1.91E-02	2.62E-01	rs1748199	1_62591465_T_C	1.00	6998	3.20E-09	GCST90277359
Triacylglycerol (48:3) levels	2	27375230	C	T	-8.90E-02	1.74E-02	6.20E-01	rs4665972	2_27375230_T_C	1.00	6998	3.19E-07	GCST90277359
Triacylglycerol (48:3) levels	2	36921090	A	T	2.22E-01	4.61E-02	3.63E-02	rs115506950	2_36921090_T_A	0.97	6998	1.46E-06	GCST90277359
Triacylglycerol (48:3) levels	4	108839211	C	T	1.24E-01	2.55E-02	1.28E-01	rs28675901	4_108839211_T_C	0.98	6998	1.11E-06	GCST90277359
Triacylglycerol (48:3) levels	5	77835346	A	G	-3.13E-01	6.83E-02	1.65E-02	rs73127058	5_77835346_G_A	0.94	6998	4.47E-06	GCST90277359
Triacylglycerol (48:3) levels	6	64177173	T	C	8.59E-02	1.75E-02	3.73E-01	rs9344829	6_64177173_C_T	0.99	6998	9.08E-07	GCST90277359
Triacylglycerol (48:3) levels	6	150841979	A	G	1.08E-01	2.15E-02	1.91E-01	rs17427389	6_150841979_G_A	1.00	6998	4.96E-07	GCST90277359
Triacylglycerol (48:3) levels	7	105583433	C	T	1.03E-01	2.16E-02	1.89E-01	rs34777947	7_105583433_T_C	0.97	6998	1.99E-06	GCST90277359
Triacylglycerol (48:3) levels	8	226933	T	C	2.44E-01	5.48E-02	2.41E-02	rs143705793	8_226933_C_T	0.98	6998	8.49E-06	GCST90277359
Triacylglycerol (48:3) levels	11	11262071	G	T	-7.76E-02	1.71E-02	4.20E-01	rs9666759	11_11262071_T_G	0.99	6998	5.86E-06	GCST90277359

Triacylglycerol (48:3) levels	11	61803910	A	G	1.64E-01	1.74E-02	3.82E-01	rs174549	11_61803910_G_A	1.00	6998	5.75E-21	GCST90277359
Triacylglycerol (48:3) levels	11	116739533	G	A	1.51E-01	2.85E-02	9.58E-02	rs10466588	11_116739533_A_G	1.00	6998	1.11E-07	GCST90277359
Triacylglycerol (48:3) levels	12	120994800	T	C	8.46E-02	1.76E-02	3.59E-01	rs2264782	12_120994800_C_T	1.00	6998	1.65E-06	GCST90277359
Triacylglycerol (48:3) levels	13	25953992	C	T	6.10E-01	1.36E-01	4.59E-03	rs184003290	13_25953992_T_C	0.88	6998	7.26E-06	GCST90277359
Triacylglycerol (48:3) levels	16	76627797	T	C	1.15E-01	2.46E-02	1.41E-01	rs57107736	16_76627797_C_T	0.99	6998	2.94E-06	GCST90277359
Triacylglycerol (48:3) levels	16	88985800	T	C	-1.85E-01	3.87E-02	5.05E-02	rs141824100	16_88985800_C_T	0.96	6998	1.93E-06	GCST90277359
Triacylglycerol (48:3) levels	18	362173	A	G	7.64E-02	1.72E-02	5.76E-01	rs514614	18_362173_G_A	1.00	6998	9.43E-06	GCST90277359
Triacylglycerol (48:3) levels	18	49592553	G	T	1.58E-01	2.90E-02	9.64E-02	rs3786247	18_49592553_T_G	1.00	6998	5.25E-08	GCST90277359
Triacylglycerol (48:3) levels	18	49634583	T	G	1.02E-01	2.19E-02	8.21E-01	rs7241918	18_49634583_G_T	1.00	6998	3.42E-06	GCST90277359
Triacylglycerol (48:3) levels	19	54038742	A	G	1.07E-01	2.29E-02	1.72E-01	rs11669648	19_54038742_G_A	0.95	6998	2.80E-06	GCST90277359
Triacylglycerol (48:3) levels	19	54173068	C	T	-2.42E-01	1.71E-02	5.93E-01	rs641738	19_54173068_T_C	0.97	6998	3.64E-45	GCST90277359
Triacylglycerol (48:3) levels	20	25320386	A	G	1.02E-01	1.91E-02	2.65E-01	rs2274890	20_25320386_G_A	1.00	6998	8.32E-08	GCST90277359
Triacylglycerol (48:3) levels	20	40537052	A	G	1.67E-01	3.44E-02	7.14E-02	rs117113213	20_40537052_G_A	0.91	6998	1.12E-06	GCST90277359
Triacylglycerol (49:1) levels	1	239331091	C	T	3.19E-01	7.17E-02	1.77E-02	rs111243657	1_239331091_T_C	0.98	5519	8.88E-06	GCST90277360
Triacylglycerol (49:1) levels	2	23397292	T	C	-3.97E-01	8.87E-02	1.22E-02	rs115609318	2_23397292_C_T	0.95	5519	7.63E-06	GCST90277360
Triacylglycerol (49:1) levels	2	27508073	C	T	-1.10E-01	1.99E-02	6.51E-01	rs1260326	2_27508073_T_C	1.00	5519	3.99E-08	GCST90277360
Triacylglycerol (49:1) levels	2	172180344	A	G	2.25E-01	4.94E-02	4.13E-02	rs114728714	2_172180344_G_A	0.94	5519	5.35E-06	GCST90277360
Triacylglycerol (49:1) levels	2	205962661	G	A	-9.97E-02	2.19E-02	7.54E-01	rs7562707	2_205962661_A_G	1.00	5519	5.51E-06	GCST90277360
Triacylglycerol (49:1) levels	3	27225555	G	C	9.12E-02	2.03E-02	3.40E-01	rs12494536	3_27225555_C_G	0.97	5519	7.09E-06	GCST90277360
Triacylglycerol (49:1) levels	3	108882807	C	A	-1.62E-01	3.21E-02	9.35E-02	rs73197087	3_108882807_A_C	0.98	5519	4.68E-07	GCST90277360
Triacylglycerol (49:1) levels	3	142941864	A	G	-1.11E-01	1.99E-02	3.64E-01	rs9653945	3_142941864_G_A	1.00	5519	2.72E-08	GCST90277360
Triacylglycerol (49:1) levels	5	1767659	G	A	-9.47E-02	2.10E-02	2.98E-01	rs6884150	5_1767659_A_G	0.98	5519	6.88E-06	GCST90277360
Triacylglycerol (49:1) levels	6	37305084	A	G	-3.98E-01	8.77E-02	1.26E-02	rs141039423	6_37305084_G_A	0.95	5519	5.75E-06	GCST90277360
Triacylglycerol (49:1) levels	6	116375468	G	C	2.87E-01	6.13E-02	2.61E-02	rs144018078	6_116375468_C_G	0.99	5519	3.03E-06	GCST90277360
Triacylglycerol (49:1) levels	7	67820	G	A	-9.79E-02	2.02E-02	5.93E-01	rs7782358	7_67820_A_G	0.93	5519	1.25E-06	GCST90277360
Triacylglycerol (49:1) levels	8	541839	A	G	2.40E-01	4.83E-02	4.00E-02	rs149878915	8_541839_G_A	0.99	5519	6.91E-07	GCST90277360
Triacylglycerol (49:1) levels	8	40568584	A	G	1.06E-01	2.40E-02	2.05E-01	rs12545907	8_40568584_G_A	0.98	5519	9.10E-06	GCST90277360
Triacylglycerol (49:1) levels	11	1669042	T	C	2.02E-01	4.42E-02	5.27E-02	rs112184984	11_1669042_C_T	0.96	5519	5.26E-06	GCST90277360
Triacylglycerol (49:1) levels	11	61785208	T	G	-1.36E-01	1.94E-02	4.08E-01	rs174537	11_61785208_G_T	1.00	5519	2.51E-12	GCST90277360
Triacylglycerol (49:1) levels	11	127595711	A	G	8.60E-01	1.83E-01	3.57E-03	rs145012974	11_127595711_G_A	0.87	5519	2.83E-06	GCST90277360
Triacylglycerol (49:1) levels	12	6138757	T	C	3.10E-01	6.60E-02	2.32E-02	rs76609522	12_6138757_C_T	0.91	5519	2.67E-06	GCST90277360
Triacylglycerol (49:1) levels	12	54071880	G	A	-9.61E-02	1.94E-02	4.00E-01	rs4759322	12_54071880_A_G	0.99	5519	7.70E-07	GCST90277360
Triacylglycerol (49:1) levels	16	31911781	A	G	3.11E-01	6.96E-02	2.02E-02	rs148590777	16_31911781_G_A	0.98	5519	8.04E-06	GCST90277360
Triacylglycerol (49:1) levels	17	52850260	C	A	-9.60E-02	1.94E-02	5.82E-01	rs12449710	17_52850260_A_C	1.00	5519	7.40E-07	GCST90277360
Triacylglycerol (49:1) levels	19	11074338	G	A	8.94E-02	2.01E-02	3.52E-01	rs11085758	19_11074338_A_G	0.98	5519	8.54E-06	GCST90277360
Triacylglycerol (49:1) levels	19	54173495	C	T	1.04E-01	1.96E-02	5.95E-01	rs8736	19_54173495_T_C	0.97	5519	1.17E-07	GCST90277360
Triacylglycerol (49:2) levels	1	62633352	C	T	-1.60E-01	1.88E-02	2.62E-01	rs10889352	1_62633352_T_C	1.00	7162	2.22E-17	GCST90277361
Triacylglycerol (49:2) levels	1	102480786	G	A	8.30E-02	1.75E-02	6.46E-01	rs1340414	1_102480786_A_G	1.00	7162	2.24E-06	GCST90277361
Triacylglycerol (49:2) levels	1	189927375	T	C	-8.56E-02	1.86E-02	2.89E-01	rs12122878	1_189927375_C_T	0.98	7162	3.98E-06	GCST90277361
Triacylglycerol (49:2) levels	1	230166915	G	A	9.09E-02	1.90E-02	7.47E-01	rs4846841	1_230166915_A_G	1.00	7162	1.85E-06	GCST90277361
Triacylglycerol (49:2) levels	2	45617902	A	T	-7.72E-02	1.74E-02	3.62E-01	rs10490343	2_45617902_T_A	1.00	7162	9.45E-06	GCST90277361
Triacylglycerol (49:2) levels	2	141967352	A	G	-2.10E-01	4.67E-02	3.39E-02	rs115510074	2_141967352_G_A	0.96	7162	7.06E-06	GCST90277361
Triacylglycerol (49:2) levels	3	142936448	G	A	1.60E-01	1.74E-02	6.32E-01	rs12638256	3_142936448_A_G	0.99	7162	4.88E-20	GCST90277361
Triacylglycerol (49:2) levels	4	27126056	G	A	-7.66E-02	1.73E-02	3.92E-01	rs6813524	4_27126056_A_G	0.96	7162	9.64E-06	GCST90277361
Triacylglycerol (49:2) levels	4	71877919	T	C	-8.83E-02	1.84E-02	2.77E-01	rs67835422	4_71877919_C_T	1.00	7162	1.72E-06	GCST90277361
Triacylglycerol (49:2) levels	5	5209937	G	A	8.23E-02	1.79E-02	3.28E-01	rs1560063	5_5209937_A_G	1.00	7162	4.51E-06	GCST90277361
Triacylglycerol (49:2) levels	5	176998593	A	C	-2.48E-01	5.60E-02	2.35E-02	rs183478874	5_176998593_C_A	0.95	7162	9.41E-06	GCST90277361
Triacylglycerol (49:2) levels	6	14803818	T	C	-1.90E-01	4.15E-02	4.42E-02	rs7742350	6_14803818_C_T	0.96	7162	4.85E-06	GCST90277361
Triacylglycerol (49:2) levels	8	58407830	G	A	-8.81E-02	1.72E-02	6.26E-01	rs11783515	8_58407830_A_G	1.00	7162	3.02E-07	GCST90277361
Triacylglycerol (49:2) levels	11	61813163	T	C	1.35E-01	1.73E-02	3.80E-01	rs174556	11_61813163_C_T	1.00	7162	5.98E-15	GCST90277361
Triacylglycerol (49:2) levels	11	107883610	G	C	-1.16E-01	2.39E-02	8.60E-01	rs7122803	11_107883610_C_G	1.00	7162	1.18E-06	GCST90277361
Triacylglycerol (49:2) levels	11	116778201	C	G	-1.95E-01	2.31E-02	8.49E-01	rs964184	11_116778201_G_C	1.00	7162	3.67E-17	GCST90277361
Triacylglycerol (49:2) levels	12	120994800	T	C	9.38E-02	1.74E-02	3.59E-01	rs2264782	12_120994800_C_T	1.00	7162	7.47E-08	GCST90277361
Triacylglycerol (49:2) levels	14	97495861	AT	A	-1.07E-01	2.32E-02	1.63E-01	rs111290564	14_97495861_A_AT	0.94	7162	3.85E-06	GCST90277361
Triacylglycerol (49:2) levels	15	99799692	C	T	-8.25E-02	1.85E-02	2.86E-01	rs12910401	15_99799692_T_C	0.99	7162	7.99E-06	GCST90277361
Triacylglycerol (49:2) levels	18	49579658	T	C	5.18E-01	1.16E-01	4.91E-03	rs149615216	18_49579658_C_T	1.00	7162	8.26E-06	GCST90277361
Triacylglycerol (49:2) levels	18	49656294	G	A	1.46E-01	1.97E-02	7.57E-01	rs1540037	18_49656294_A_G	0.97	7162	1.36E-13	GCST90277361

Triacylglycerol (49:2) levels	19	11220678	T	G	-1.67E-01	3.46E-02	6.24E-02	rs4804576	19_11220678_G_T	0.99	7162	1.40E-06	GCST90277361
Triacylglycerol (49:2) levels	19	54173068	C	T	-2.25E-01	1.69E-02	5.93E-01	rs641738	19_54173068_T_C	0.97	7162	7.56E-40	GCST90277361
Triacylglycerol (49:2) levels	21	18058617	T	C	-1.37E-01	3.09E-02	8.05E-02	rs204015	21_18058617_C_T	0.98	7162	8.93E-06	GCST90277361
Triacylglycerol (50:1) levels	1	62591465	C	T	-1.87E-01	1.87E-02	2.62E-01	rs1748199	1_62591465_T_C	1.00	7170	1.99E-23	GCST90277362
Triacylglycerol (50:1) levels	2	27519736	C	T	-9.09E-02	1.73E-02	6.46E-01	rs780093	2_27519736_T_C	1.00	7170	1.52E-07	GCST90277362
Triacylglycerol (50:1) levels	2	78784774	T	G	8.62E-02	1.69E-02	4.62E-01	rs7561644	2_78784774_G_T	0.99	7170	3.63E-07	GCST90277362
Triacylglycerol (50:1) levels	4	39404797	G	C	7.57E-02	1.69E-02	5.74E-01	rs7687811	4_39404797_C_G	1.00	7170	7.30E-06	GCST90277362
Triacylglycerol (50:1) levels	4	115592282	G	A	-2.84E-01	6.27E-02	1.84E-02	rs72676136	4_115592282_A_G	0.98	7170	6.00E-06	GCST90277362
Triacylglycerol (50:1) levels	4	133227651	T	G	-3.51E-01	7.88E-02	1.20E-02	rs146893850	4_133227651_G_T	0.93	7170	8.84E-06	GCST90277362
Triacylglycerol (50:1) levels	5	44280978	TAGTGTC	T	1.30E-01	2.91E-02	9.27E-02	rs144506691	5_44280978_T_TAGTGT	1.00	7170	7.30E-06	GCST90277362
Triacylglycerol (50:1) levels	6	10040346	C	T	6.38E-01	1.33E-01	4.56E-03	rs72830461	6_10040346_T_C	0.88	7170	1.56E-06	GCST90277362
Triacylglycerol (50:1) levels	6	32222613	G	T	-8.65E-02	1.77E-02	3.26E-01	rs915894	6_32222613_T_G	0.99	7170	1.07E-06	GCST90277362
Triacylglycerol (50:1) levels	6	160179246	C	T	7.38E-01	1.63E-01	2.78E-03	rs139001006	6_160179246_T_C	0.92	7170	6.26E-06	GCST90277362
Triacylglycerol (50:1) levels	7	9471070	G	A	8.06E-02	1.82E-02	6.98E-01	rs2713348	7_9471070_A_G	1.00	7170	9.26E-06	GCST90277362
Triacylglycerol (50:1) levels	7	44538023	A	G	9.44E-02	2.10E-02	1.97E-01	rs73107473	7_44538023_G_A	0.99	7170	6.83E-06	GCST90277362
Triacylglycerol (50:1) levels	7	105583433	C	T	1.02E-01	2.13E-02	1.89E-01	rs34777947	7_105583433_T_C	0.97	7170	1.74E-06	GCST90277362
Triacylglycerol (50:1) levels	7	135205323	T	C	-2.67E-01	5.83E-02	2.05E-02	rs112713750	7_135205323_C_T	1.00	7170	4.58E-06	GCST90277362
Triacylglycerol (50:1) levels	7	143395176	A	G	-1.32E-01	2.91E-02	8.96E-02	rs34372369	7_143395176_G_A	1.00	7170	5.79E-06	GCST90277362
Triacylglycerol (50:1) levels	8	58476006	C	T	-7.98E-02	1.70E-02	6.18E-01	rs2081687	8_58476006_T_C	1.00	7170	2.67E-06	GCST90277362
Triacylglycerol (50:1) levels	8	66293006	C	A	7.94E-02	1.80E-02	3.18E-01	rs11780222	8_66293006_A_C	1.00	7170	9.98E-06	GCST90277362
Triacylglycerol (50:1) levels	8	84010633	A	T	-2.63E-01	5.71E-02	2.20E-02	rs114488287	8_84010633_T_A	0.97	7170	4.11E-06	GCST90277362
Triacylglycerol (50:1) levels	11	61458997	G	T	3.11E-01	6.32E-02	1.79E-02	rs77470505	11_61458997_T_G	1.00	7170	8.70E-07	GCST90277362
Triacylglycerol (50:1) levels	11	61545254	T	G	3.08E-01	5.72E-02	2.40E-02	rs150684478	11_61545254_G_T	0.90	7170	7.46E-08	GCST90277362
Triacylglycerol (50:1) levels	11	61824890	G	A	2.92E-01	1.67E-02	4.08E-01	rs174566	11_61824890_A_G	1.00	7170	3.90E-67	GCST90277362
Triacylglycerol (50:1) levels	11	115593068	A	G	-7.78E-02	1.74E-02	6.35E-01	rs976947	11_115593068_G_A	1.00	7170	8.04E-06	GCST90277362
Triacylglycerol (50:1) levels	11	116778201	C	G	-2.39E-01	2.30E-02	8.49E-01	rs964184	11_116778201_G_C	1.00	7170	5.59E-25	GCST90277362
Triacylglycerol (50:1) levels	12	120982457	G	A	-1.10E-01	1.68E-02	5.77E-01	rs7979473	12_120982457_A_G	1.00	7170	7.38E-11	GCST90277362
Triacylglycerol (50:1) levels	13	25953992	C	T	6.08E-01	1.35E-01	4.59E-03	rs184003290	13_25953992_T_C	0.88	7170	7.25E-06	GCST90277362
Triacylglycerol (50:1) levels	13	30195598	A	G	-8.38E-02	1.84E-02	3.02E-01	rs9551866	13_30195598_G_A	0.97	7170	5.27E-06	GCST90277362
Triacylglycerol (50:1) levels	14	73791596	A	G	1.35E-01	3.05E-02	8.24E-02	rs12147624	14_73791596_G_A	1.00	7170	9.03E-06	GCST90277362
Triacylglycerol (50:1) levels	15	58432643	A	G	9.12E-02	1.80E-02	3.37E-01	rs8033940	15_58432643_G_A	0.99	7170	4.14E-07	GCST90277362
Triacylglycerol (50:1) levels	15	73927659	T	C	1.83E-01	3.86E-02	4.86E-02	rs41429348	15_73927659_C_T	0.99	7170	2.29E-06	GCST90277362
Triacylglycerol (50:1) levels	16	15043593	A	G	8.54E-02	1.77E-02	3.32E-01	rs62039480	16_15043593_G_A	1.00	7170	1.50E-06	GCST90277362
Triacylglycerol (50:1) levels	18	49579658	T	C	6.42E-01	1.16E-01	4.91E-03	rs149615216	18_49579658_C_T	1.00	7170	2.88E-08	GCST90277362
Triacylglycerol (50:1) levels	18	49656294	G	A	1.55E-01	1.97E-02	7.57E-01	rs1540037	18_49656294_A_G	0.97	7170	4.09E-15	GCST90277362
Triacylglycerol (50:1) levels	19	54038742	A	G	1.13E-01	2.25E-02	1.72E-01	rs11669648	19_54038742_G_A	0.95	7170	5.71E-07	GCST90277362
Triacylglycerol (50:1) levels	19	54173495	C	T	-3.69E-01	1.65E-02	5.95E-01	rs8736	19_54173495_T_C	0.97	7170	4.82E-107	GCST90277362
Triacylglycerol (50:1) levels	20	25230191	G	A	7.91E-02	1.67E-02	5.02E-01	rs6115094	20_25230191_A_G	1.00	7170	2.26E-06	GCST90277362
Triacylglycerol (50:1) levels	20	40537052	A	G	1.74E-01	3.39E-02	7.14E-02	rs117113213	20_40537052_G_A	0.91	7170	2.85E-07	GCST90277362
Triacylglycerol (50:2) levels	1	62662654	G	A	1.51E-01	1.94E-02	7.38E-01	rs1168104	1_62662654_A_G	1.00	6784	6.66E-15	GCST90277363
Triacylglycerol (50:2) levels	2	26361322	T	C	4.77E-01	1.06E-01	6.56E-03	rs7355249	2_26361322_C_T	0.99	6784	7.50E-06	GCST90277363
Triacylglycerol (50:2) levels	2	27508073	C	T	-9.00E-02	1.80E-02	6.51E-01	rs1260326	2_27508073_T_C	1.00	6784	5.42E-07	GCST90277363
Triacylglycerol (50:2) levels	4	4988836	A	G	-8.46E-02	1.78E-02	5.72E-01	rs6820980	4_4988836_G_A	0.95	6784	1.98E-06	GCST90277363
Triacylglycerol (50:2) levels	4	37896323	G	A	-2.57E-01	5.59E-02	2.58E-02	rs76457276	4_37896323_A_G	0.94	6784	4.21E-06	GCST90277363
Triacylglycerol (50:2) levels	4	78054777	C	T	-9.53E-02	2.05E-02	2.34E-01	rs67945876	4_78054777_T_C	0.98	6784	3.28E-06	GCST90277363
Triacylglycerol (50:2) levels	4	169287018	C	T	-1.04E-01	2.20E-02	1.89E-01	rs13147779	4_169287018_T_C	0.99	6784	2.03E-06	GCST90277363
Triacylglycerol (50:2) levels	6	156379168	C	T	-8.81E-02	1.88E-02	2.95E-01	rs2819082	6_156379168_T_C	0.98	6784	2.97E-06	GCST90277363
Triacylglycerol (50:2) levels	7	6295952	T	A	9.42E-02	2.05E-02	2.32E-01	rs7782961	7_6295952_A_T	0.97	6784	4.21E-06	GCST90277363
Triacylglycerol (50:2) levels	7	72363703	A	G	1.30E-01	2.81E-02	1.05E-01	rs11769084	7_72363703_G_A	0.99	6784	3.96E-06	GCST90277363
Triacylglycerol (50:2) levels	8	40712078	G	A	-8.22E-02	1.74E-02	5.76E-01	rs7845664	8_40712078_A_G	0.99	6784	2.26E-06	GCST90277363
Triacylglycerol (50:2) levels	8	58485873	G	A	-9.25E-02	2.00E-02	2.46E-01	rs28514538	8_58485873_A_G	1.00	6784	3.67E-06	GCST90277363
Triacylglycerol (50:2) levels	9	34672503	G	A	-1.98E-01	4.41E-02	3.95E-02	rs117761170	9_34672503_A_G	0.99	6784	7.29E-06	GCST90277363
Triacylglycerol (50:2) levels	9	81886311	C	T	-1.93E-01	4.29E-02	5.01E-02	rs17085148	9_81886311_T_C	0.82	6784	6.95E-06	GCST90277363
Triacylglycerol (50:2) levels	10	51855519	G	C	9.08E-02	1.90E-02	6.51E-01	rs1937711	10_51855519_C_G	0.89	6784	1.84E-06	GCST90277363
Triacylglycerol (50:2) levels	11	60782852	A	G	-9.57E-02	1.89E-02	3.06E-01	rs72914920	11_60782852_G_A	0.98	6784	3.98E-07	GCST90277363
Triacylglycerol (50:2) levels	11	61822009	G	A	3.49E-01	1.73E-02	3.84E-01	rs28456	11_61822009_A_G	1.00	6784	1.70E-88	GCST90277363
Triacylglycerol (50:2) levels	11	62056104	A	G	9.94E-02	1.83E-02	3.35E-01	rs10897211	11_62056104_G_A	0.99	6784	5.84E-08	GCST90277363

Triacylglycerol (50:2) levels	11	91426863	C	T	4.62E-01	9.46E-02	8.81E-03	rs16916055	11_91426863_T_C	0.97	6784	1.10E-06	GCST90277363
Triacylglycerol (50:2) levels	11	115593068	A	G	-8.09E-02	1.79E-02	6.35E-01	rs976947	11_115593068_G_A	1.00	6784	6.42E-06	GCST90277363
Triacylglycerol (50:2) levels	11	116778201	C	G	-1.62E-01	2.38E-02	8.49E-01	rs964184	11_116778201_G_C	1.00	6784	1.05E-11	GCST90277363
Triacylglycerol (50:2) levels	12	5886232	A	G	2.72E-01	6.10E-02	2.01E-02	rs59382088	12_5886232_G_A	0.99	6784	8.23E-06	GCST90277363
Triacylglycerol (50:2) levels	13	73071162	T	C	1.25E-01	2.80E-02	1.05E-01	rs9530145	13_73071162_C_T	1.00	6784	8.04E-06	GCST90277363
Triacylglycerol (50:2) levels	13	93173799	C	G	9.04E-02	1.98E-02	2.55E-01	rs319552	13_93173799_G_C	0.96	6784	5.02E-06	GCST90277363
Triacylglycerol (50:2) levels	15	58368884	C	T	1.76E-01	3.97E-02	4.79E-02	rs59739041	15_58368884_T_C	0.99	6784	8.81E-06	GCST90277363
Triacylglycerol (50:2) levels	16	15036737	G	A	-1.26E-01	1.82E-02	3.34E-01	rs6498540	16_15036737_A_G	0.99	6784	5.24E-12	GCST90277363
Triacylglycerol (50:2) levels	16	80253481	T	G	-9.05E-02	1.94E-02	2.68E-01	rs12598816	16_80253481_G_T	1.00	6784	3.12E-06	GCST90277363
Triacylglycerol (50:2) levels	16	84256448	T	C	-1.51E-01	3.36E-02	7.30E-02	rs80011585	16_84256448_C_T	0.95	6784	7.28E-06	GCST90277363
Triacylglycerol (50:2) levels	17	53197241	C	T	-1.83E-01	4.03E-02	4.93E-02	rs145740269	17_53197241_T_C	0.97	6784	6.00E-06	GCST90277363
Triacylglycerol (50:2) levels	18	49595788	C	A	1.47E-01	2.98E-02	9.34E-02	rs12458441	18_49595788_A_C	1.00	6784	8.74E-07	GCST90277363
Triacylglycerol (50:2) levels	18	68982283	G	T	3.21E-01	7.10E-02	1.57E-02	rs117006704	18_68982283_T_G	0.92	6784	6.03E-06	GCST90277363
Triacylglycerol (50:2) levels	19	18072285	T	C	-3.65E-01	7.72E-02	1.24E-02	rs117511121	19_18072285_C_T	1.00	6784	2.28E-06	GCST90277363
Triacylglycerol (50:2) levels	19	54173495	C	T	2.22E-01	1.74E-02	5.95E-01	rs8736	19_54173495_T_C	0.97	6784	4.35E-37	GCST90277363
Triacylglycerol (50:3) levels	1	18918184	T	C	1.14E-01	2.32E-02	1.52E-01	rs4912056	1_18918184_C_T	0.99	7174	1.02E-06	GCST90277364
Triacylglycerol (50:3) levels	1	62662654	G	A	1.94E-01	1.87E-02	7.38E-01	rs1168104	1_62662654_A_G	1.00	7174	6.30E-25	GCST90277364
Triacylglycerol (50:3) levels	1	239312130	T	C	3.03E-01	6.35E-02	1.74E-02	rs112944184	1_239312130_C_T	0.99	7174	1.92E-06	GCST90277364
Triacylglycerol (50:3) levels	2	27508073	C	T	-9.64E-02	1.74E-02	6.51E-01	rs1260326	2_27508073_T_C	1.00	7174	3.06E-08	GCST90277364
Triacylglycerol (50:3) levels	2	68915026	C	T	8.25E-02	1.66E-02	4.87E-01	rs4629190	2_68915026_T_C	0.99	7174	6.30E-07	GCST90277364
Triacylglycerol (50:3) levels	2	137161796	G	A	1.63E-01	3.50E-02	5.98E-02	rs11899922	2_137161796_A_G	1.00	7174	3.46E-06	GCST90277364
Triacylglycerol (50:3) levels	3	142933227	G	C	-9.41E-02	1.74E-02	6.31E-01	rs9848779	3_142933227_C_G	0.99	7174	6.90E-08	GCST90277364
Triacylglycerol (50:3) levels	3	171396003	A	G	-7.96E-02	1.67E-02	5.87E-01	rs13068826	3_171396003_G_A	1.00	7174	2.02E-06	GCST90277364
Triacylglycerol (50:3) levels	4	78054777	C	T	-9.42E-02	1.99E-02	2.34E-01	rs67945876	4_78054777_T_C	0.98	7174	2.11E-06	GCST90277364
Triacylglycerol (50:3) levels	5	35798066	T	C	-2.61E-01	5.81E-02	2.17E-02	rs116730430	5_35798066_C_T	0.97	7174	7.11E-06	GCST90277364
Triacylglycerol (50:3) levels	5	106005784	T	C	2.76E-01	6.23E-02	1.99E-02	rs186923696	5_106005784_C_T	0.90	7174	9.94E-06	GCST90277364
Triacylglycerol (50:3) levels	6	26710895	G	A	8.20E-02	1.76E-02	3.51E-01	rs62396086	6_26710895_A_G	1.00	7174	3.15E-06	GCST90277364
Triacylglycerol (50:3) levels	6	156391192	T	C	7.56E-02	1.66E-02	5.08E-01	rs12213531	6_156391192_C_T	1.00	7174	5.56E-06	GCST90277364
Triacylglycerol (50:3) levels	7	28245096	A	T	3.91E-01	8.74E-02	9.97E-03	rs111265437	7_28245096_T_A	0.90	7174	7.87E-06	GCST90277364
Triacylglycerol (50:3) levels	7	73597712	A	G	-1.29E-01	2.53E-02	1.22E-01	rs35332062	7_73597712_G_A	1.00	7174	4.08E-07	GCST90277364
Triacylglycerol (50:3) levels	7	80900239	A	G	-2.06E-01	4.59E-02	3.56E-02	rs187491194	7_80900239_G_A	0.94	7174	6.85E-06	GCST90277364
Triacylglycerol (50:3) levels	8	4623841	G	C	8.44E-02	1.72E-02	5.07E-01	rs2725021	8_4623841_C_G	0.94	7174	9.12E-07	GCST90277364
Triacylglycerol (50:3) levels	8	18402366	T	C	-9.22E-02	1.78E-02	6.80E-01	rs11780610	8_18402366_C_T	1.00	7174	2.25E-07	GCST90277364
Triacylglycerol (50:3) levels	9	69555144	C	T	3.51E-01	7.88E-02	1.21E-02	rs114051008	9_69555144_T_C	0.98	7174	8.37E-06	GCST90277364
Triacylglycerol (50:3) levels	10	112296190	C	G	-1.44E-01	3.07E-02	8.55E-02	rs61872308	10_112296190_G_C	0.92	7174	2.65E-06	GCST90277364
Triacylglycerol (50:3) levels	11	61781553	A	G	-2.49E-01	1.68E-02	4.09E-01	rs174533	11_61781553_G_A	1.00	7174	3.40E-49	GCST90277364
Triacylglycerol (50:3) levels	11	116778201	C	G	-2.67E-01	2.30E-02	8.49E-01	rs964184	11_116778201_G_C	1.00	7174	5.97E-31	GCST90277364
Triacylglycerol (50:3) levels	13	114124757	T	C	1.18E-01	2.66E-02	1.09E-01	rs2149773	13_114124757_C_T	0.99	7174	9.48E-06	GCST90277364
Triacylglycerol (50:3) levels	14	61547116	C	G	1.17E-01	2.47E-02	1.33E-01	rs10138449	14_61547116_G_C	0.98	7174	2.05E-06	GCST90277364
Triacylglycerol (50:3) levels	17	11562403	A	G	-1.34E-01	2.92E-02	9.05E-02	rs9910521	17_11562403_G_A	0.99	7174	4.48E-06	GCST90277364
Triacylglycerol (50:3) levels	18	22676206	T	G	7.49E-02	1.68E-02	5.54E-01	rs12052014	18_22676206_G_T	1.00	7174	8.65E-06	GCST90277364
Triacylglycerol (50:3) levels	18	49628409	C	T	1.88E-01	2.65E-02	1.14E-01	rs56339947	18_49628409_T_C	0.99	7174	1.36E-12	GCST90277364
Triacylglycerol (50:3) levels	18	49666690	A	T	7.88E-02	1.65E-02	4.99E-01	rs6507942	18_49666690_T_A	1.00	7174	1.98E-06	GCST90277364
Triacylglycerol (50:3) levels	19	11220266	G	A	-2.31E-01	3.93E-02	4.69E-02	rs17699030	19_11220266_A_G	0.98	7174	4.48E-09	GCST90277364
Triacylglycerol (50:3) levels	19	31666796	G	A	-1.20E-01	2.58E-02	8.74E-01	rs10423460	19_31666796_A_G	0.92	7174	3.22E-06	GCST90277364
Triacylglycerol (50:3) levels	19	53935764	T	C	-3.08E-01	6.56E-02	1.86E-02	rs144215405	19_53935764_C_T	0.87	7174	2.72E-06	GCST90277364
Triacylglycerol (50:3) levels	19	54173495	C	T	2.75E-01	1.67E-02	5.95E-01	rs8736	19_54173495_T_C	0.97	7174	1.26E-59	GCST90277364
Triacylglycerol (50:3) levels	20	36376425	C	T	7.81E-02	1.71E-02	6.30E-01	rs2425225	20_36376425_T_C	1.00	7174	4.87E-06	GCST90277364
Triacylglycerol (50:3) levels	21	34102305	C	T	1.55E-01	3.42E-02	6.39E-02	rs2032107	21_34102305_T_C	0.99	7174	6.31E-06	GCST90277364
Triacylglycerol (50:4) levels	1	62633352	C	T	-1.54E-01	1.88E-02	2.62E-01	rs10889352	1_62633352_T_C	1.00	7164	2.63E-16	GCST90277365
Triacylglycerol (50:4) levels	1	230174822	T	C	-8.46E-02	1.70E-02	3.96E-01	rs627108	1_230174822_C_T	0.99	7164	6.88E-07	GCST90277365
Triacylglycerol (50:4) levels	2	141712670	C	T	8.00E-02	1.71E-02	3.98E-01	rs72846591	2_141712670_T_C	1.00	7164	3.00E-06	GCST90277365
Triacylglycerol (50:4) levels	3	74470951	G	A	-8.60E-02	1.91E-02	2.58E-01	rs149540646	3_74470951_A_G	1.00	7164	6.60E-06	GCST90277365
Triacylglycerol (50:4) levels	4	25950031	A	G	1.95E-01	4.20E-02	4.62E-02	rs62409317	4_25950031_G_A	0.91	7164	3.65E-06	GCST90277365
Triacylglycerol (50:4) levels	8	58407830	G	A	-8.88E-02	1.72E-02	6.26E-01	rs11783515	8_58407830_A_G	1.00	7164	2.39E-07	GCST90277365
Triacylglycerol (50:4) levels	9	91769704	A	C	4.72E-01	1.04E-01	6.70E-03	rs145993592	9_91769704_C_A	0.94	7164	5.87E-06	GCST90277365
Triacylglycerol (50:4) levels	10	74877945	G	A	4.06E-01	8.90E-02	9.68E-03	rs78572804	10_74877945_A_G	0.92	7164	5.15E-06	GCST90277365

Triacylglycerol (50:4) levels	11	61826344	T	C	1.57E-01	1.70E-02	4.05E-01	rs174568	11_61826344_C_T	1.00	7164	3.37E-20	GCST90277365
Triacylglycerol (50:4) levels	11	116778201	C	G	-1.66E-01	2.32E-02	8.49E-01	rs964184	11_116778201_G_C	1.00	7164	7.65E-13	GCST90277365
Triacylglycerol (50:4) levels	13	22601154	A	G	8.94E-02	1.95E-02	2.38E-01	rs12428337	13_22601154_G_A	1.00	7164	4.56E-06	GCST90277365
Triacylglycerol (50:4) levels	13	95125494	G	A	-8.75E-02	1.96E-02	7.60E-01	rs1751064	13_95125494_A_G	1.00	7164	7.79E-06	GCST90277365
Triacylglycerol (50:4) levels	14	38415942	C	T	7.15E-01	1.39E-01	3.57E-03	rs116845732	14_38415942_T_C	0.89	7164	2.86E-07	GCST90277365
Triacylglycerol (50:4) levels	14	103616633	A	G	3.47E-01	7.53E-02	1.32E-02	rs186422572	14_103616633_G_A	0.94	7164	4.08E-06	GCST90277365
Triacylglycerol (50:4) levels	15	58386313	T	C	8.86E-02	1.76E-02	3.38E-01	rs10468017	15_58386313_C_T	1.00	7164	4.59E-07	GCST90277365
Triacylglycerol (50:4) levels	16	24297504	T	C	-8.05E-02	1.73E-02	4.23E-01	rs9652580	16_24297504_C_T	0.98	7164	3.30E-06	GCST90277365
Triacylglycerol (50:4) levels	18	49579658	T	C	5.25E-01	1.16E-01	4.91E-03	rs149615216	18_49579658_C_T	1.00	7164	6.17E-06	GCST90277365
Triacylglycerol (50:4) levels	18	49656294	G	A	1.65E-01	1.97E-02	7.57E-01	rs1540037	18_49656294_A_G	0.97	7164	7.66E-17	GCST90277365
Triacylglycerol (50:4) levels	18	69102156	G	A	1.21E-01	2.66E-02	1.21E-01	rs62095282	18_69102156_A_G	0.91	7164	5.08E-06	GCST90277365
Triacylglycerol (50:4) levels	19	11220678	T	G	-1.78E-01	3.45E-02	6.24E-02	rs4804576	19_11220678_G_T	0.99	7164	2.66E-07	GCST90277365
Triacylglycerol (50:4) levels	19	54142105	A	G	-1.22E-01	1.69E-02	5.93E-01	rs36634	19_54142105_G_A	1.00	7164	6.21E-13	GCST90277365
Triacylglycerol (50:4) levels	20	55260213	C	T	-3.21E-01	7.26E-02	1.43E-02	rs6023985	20_55260213_T_C	0.91	7164	9.62E-06	GCST90277365
Triacylglycerol (50:4) levels	21	36261009	G	T	1.48E-01	3.24E-02	7.62E-02	rs41418546	21_36261009_T_G	0.96	7164	4.75E-06	GCST90277365
Triacylglycerol (50:5) levels	1	62633352	C	T	-1.24E-01	1.99E-02	2.62E-01	rs10889352	1_62633352_T_C	1.00	6489	4.53E-10	GCST90277366
Triacylglycerol (50:5) levels	1	183589970	A	G	1.44E-01	3.10E-02	8.96E-02	rs10911365	1_183589970_G_A	0.98	6489	3.29E-06	GCST90277366
Triacylglycerol (50:5) levels	1	236637819	A	G	1.97E-01	4.11E-02	4.86E-02	rs11803708	1_236637819_G_A	1.00	6489	1.69E-06	GCST90277366
Triacylglycerol (50:5) levels	2	2513104	T	C	-1.70E-01	3.83E-02	5.80E-02	rs150683971	2_2513104_C_T	0.98	6489	9.18E-06	GCST90277366
Triacylglycerol (50:5) levels	2	42173936	A	C	-1.25E-01	2.62E-02	1.29E-01	rs79507869	2_42173936_C_A	1.00	6489	1.79E-06	GCST90277366
Triacylglycerol (50:5) levels	2	52445173	A	G	-4.66E-01	1.04E-01	7.60E-03	rs114782597	2_52445173_G_A	0.97	6489	8.06E-06	GCST90277366
Triacylglycerol (50:5) levels	2	117641775	T	C	1.87E-01	4.05E-02	5.02E-02	rs4848460	2_117641775_C_T	0.96	6489	3.94E-06	GCST90277366
Triacylglycerol (50:5) levels	3	62442977	C	G	-2.51E-01	5.63E-02	2.71E-02	rs72887719	3_62442977_G_C	0.92	6489	8.62E-06	GCST90277366
Triacylglycerol (50:5) levels	3	74310731	G	C	2.59E-01	5.46E-02	2.78E-02	rs73109128	3_74310731_C_G	1.00	6489	2.20E-06	GCST90277366
Triacylglycerol (50:5) levels	3	196572573	A	G	1.62E-01	3.55E-02	6.93E-02	rs75519325	3_196572573_G_A	0.93	6489	5.23E-06	GCST90277366
Triacylglycerol (50:5) levels	5	172222516	G	A	-1.40E-01	2.80E-02	1.09E-01	rs17074477	5_172222516_A_G	0.99	6489	6.51E-07	GCST90277366
Triacylglycerol (50:5) levels	8	99590810	T	C	1.34E-01	2.98E-02	9.91E-02	rs117234964	8_99590810_C_T	0.97	6489	7.08E-06	GCST90277366
Triacylglycerol (50:5) levels	11	61826344	T	C	1.61E-01	1.78E-02	4.05E-01	rs174568	11_61826344_C_T	1.00	6489	1.47E-19	GCST90277366
Triacylglycerol (50:5) levels	11	116778201	C	G	-1.23E-01	2.42E-02	8.49E-01	rs964184	11_116778201_G_C	1.00	6489	4.28E-07	GCST90277366
Triacylglycerol (50:5) levels	12	32548599	T	C	2.79E-01	5.65E-02	2.51E-02	rs144480172	12_32548599_C_T	0.96	6489	8.07E-07	GCST90277366
Triacylglycerol (50:5) levels	12	120915171	G	A	7.99E-02	1.76E-02	5.39E-01	rs2701185	12_120915171_A_G	1.00	6489	5.94E-06	GCST90277366
Triacylglycerol (50:5) levels	15	82771066	G	A	-4.17E-01	8.78E-02	1.07E-02	rs72753955	15_82771066_A_G	0.98	6489	2.09E-06	GCST90277366
Triacylglycerol (50:5) levels	16	5592867	A	C	8.24E-02	1.74E-02	5.17E-01	rs1544531	16_5592867_C_A	0.99	6489	2.37E-06	GCST90277366
Triacylglycerol (50:5) levels	16	24662577	A	G	-1.39E-01	3.04E-02	9.12E-02	rs12103149	16_24662577_G_A	1.00	6489	5.11E-06	GCST90277366
Triacylglycerol (50:5) levels	17	21216552	C	G	2.56E-01	5.67E-02	2.55E-02	rs12943438	17_21216552_G_C	0.96	6489	6.16E-06	GCST90277366
Triacylglycerol (50:5) levels	17	36639734	T	C	-9.51E-02	2.11E-02	2.61E-01	rs211991	17_36639734_C_T	0.91	6489	6.38E-06	GCST90277366
Triacylglycerol (50:5) levels	18	13582870	C	G	-1.68E-01	3.57E-02	6.65E-02	rs72884421	18_13582870_G_C	0.93	6489	2.40E-06	GCST90277366
Triacylglycerol (50:5) levels	18	49594230	A	G	1.54E-01	3.01E-02	9.62E-02	rs9953437	18_49594230_G_A	1.00	6489	2.91E-07	GCST90277366
Triacylglycerol (50:5) levels	19	11220266	G	A	-1.88E-01	4.19E-02	4.69E-02	rs17699030	19_11220266_A_G	0.98	6489	6.79E-06	GCST90277366
Triacylglycerol (50:5) levels	19	44858389	G	A	-1.41E-01	3.07E-02	9.08E-02	rs365653	19_44858389_A_G	0.99	6489	4.80E-06	GCST90277366
Triacylglycerol (50:5) levels	19	54173495	C	T	-1.31E-01	1.79E-02	5.95E-01	rs8736	19_54173495_T_C	0.97	6489	2.45E-13	GCST90277366
Triacylglycerol (51:1) levels	1	62670407	A	C	1.05E-01	2.10E-02	7.37E-01	rs1168127	1_62670407_C_A	1.00	5882	5.26E-07	GCST90277367
Triacylglycerol (51:1) levels	1	97483748	G	A	2.50E-01	5.57E-02	2.86E-02	rs117960039	1_97483748_A_G	0.94	5882	7.25E-06	GCST90277367
Triacylglycerol (51:1) levels	1	234028770	A	G	8.77E-02	1.83E-02	4.75E-01	rs4920209	1_234028770_G_A	1.00	5882	1.63E-06	GCST90277367
Triacylglycerol (51:1) levels	2	213973289	G	T	1.26E-01	2.80E-02	1.26E-01	rs12467458	2_213973289_T_G	0.98	5882	6.52E-06	GCST90277367
Triacylglycerol (51:1) levels	4	87407252	A	G	3.70E-01	7.85E-02	1.40E-02	rs140854080	4_87407252_G_A	0.99	5882	2.47E-06	GCST90277367
Triacylglycerol (51:1) levels	5	17631738	T	G	-1.62E-01	3.62E-02	9.27E-01	rs2379249	5_17631738_G_T	0.96	5882	8.22E-06	GCST90277367
Triacylglycerol (51:1) levels	5	174699125	T	C	1.93E-01	4.36E-02	4.87E-02	rs114918906	5_174699125_C_T	0.94	5882	9.38E-06	GCST90277367
Triacylglycerol (51:1) levels	6	94237667	C	A	-9.88E-02	2.07E-02	2.74E-01	rs55976852	6_94237667_A_C	0.99	5882	1.92E-06	GCST90277367
Triacylglycerol (51:1) levels	6	156379682	A	G	-9.24E-02	1.84E-02	4.86E-01	rs2819083	6_156379682_G_A	0.99	5882	4.92E-07	GCST90277367
Triacylglycerol (51:1) levels	8	100795002	T	C	1.94E-01	4.14E-02	5.21E-02	rs36061340	8_100795002_C_T	1.00	5882	2.94E-06	GCST90277367
Triacylglycerol (51:1) levels	9	31860328	G	T	1.36E-01	3.02E-02	1.10E-01	rs12554791	9_31860328_T_G	0.94	5882	6.69E-06	GCST90277367
Triacylglycerol (51:1) levels	9	133897690	C	T	9.60E-02	2.14E-02	2.76E-01	rs13291383	9_133897690_T_C	0.93	5882	7.48E-06	GCST90277367
Triacylglycerol (51:1) levels	10	6185479	A	G	-4.32E-01	9.74E-02	1.08E-02	rs74559802	10_6185479_G_A	0.81	5882	9.56E-06	GCST90277367
Triacylglycerol (51:1) levels	11	76222144	T	A	1.73E-01	3.76E-02	6.69E-02	rs670975	11_76222144_A_T	1.00	5882	4.20E-06	GCST90277367
Triacylglycerol (51:1) levels	11	116786845	T	C	-1.27E-01	2.16E-02	7.65E-01	rs3741298	11_116786845_C_T	1.00	5882	4.35E-09	GCST90277367
Triacylglycerol (51:1) levels	12	18171023	A	G	1.52E-01	3.20E-02	8.97E-02	rs61915733	12_18171023_G_A	0.97	5882	2.09E-06	GCST90277367

Triacylglycerol (51:1) levels	12	40642166	CT	C	-3.84E-01	8.41E-02	1.32E-02	rs200779799	12_40642166_C_CT	0.97	5882	4.97E-06	GCST90277367
Triacylglycerol (51:1) levels	13	31552770	A	G	-4.65E-01	1.05E-01	8.70E-03	rs111645166	13_31552770_G_A	0.92	5882	9.76E-06	GCST90277367
Triacylglycerol (51:1) levels	14	35052659	A	G	1.73E-01	3.89E-02	6.23E-02	rs74572381	14_35052659_G_A	1.00	5882	8.84E-06	GCST90277367
Triacylglycerol (51:1) levels	14	95031978	C	T	-4.82E-01	1.05E-01	8.15E-03	rs117598891	14_95031978_T_C	0.94	5882	4.91E-06	GCST90277367
Triacylglycerol (51:1) levels	15	63874899	GTA	G	-8.25E-02	1.86E-02	4.07E-01	rs34675318	15_63874899_G_GTA	1.00	5882	9.27E-06	GCST90277367
Triacylglycerol (51:1) levels	16	10136125	C	A	-9.59E-02	2.16E-02	7.53E-01	rs10775270	16_10136125_A_C	1.00	5882	9.48E-06	GCST90277367
Triacylglycerol (51:1) levels	16	73859072	G	A	-2.87E-01	4.98E-02	3.45E-02	rs117210444	16_73859072_A_G	0.97	5882	9.19E-09	GCST90277367
Triacylglycerol (51:1) levels	17	10103775	G	A	-8.72E-02	1.93E-02	4.37E-01	rs12938892	17_10103775_A_G	0.91	5882	6.54E-06	GCST90277367
Triacylglycerol (51:1) levels	18	49619347	A	G	1.52E-01	3.20E-02	9.37E-02	rs957142	18_49619347_G_A	0.99	5882	2.24E-06	GCST90277367
Triacylglycerol (51:1) levels	18	73825578	G	A	3.01E-01	6.49E-02	2.19E-02	rs75166839	18_73825578_A_G	0.93	5882	3.70E-06	GCST90277367
Triacylglycerol (51:1) levels	19	2402297	T	C	-3.02E-01	6.77E-02	2.14E-02	rs146290629	19_2402297_C_T	0.93	5882	8.49E-06	GCST90277367
Triacylglycerol (51:1) levels	19	52584863	C	A	-8.86E-02	1.94E-02	3.94E-01	rs599859	19_52584863_A_C	0.96	5882	4.86E-06	GCST90277367
Triacylglycerol (51:1) levels	22	24485813	T	C	8.44E-02	1.86E-02	5.45E-01	rs4822502	22_24485813_C_T	1.00	5882	5.54E-06	GCST90277367
Triacylglycerol (51:2) levels	1	55039974	T	G	-2.33E-01	4.65E-02	3.32E-02	rs11591147	1_55039974_G_T	1.00	7174	5.44E-07	GCST90277368
Triacylglycerol (51:2) levels	1	230244207	G	A	-1.88E-01	4.23E-02	4.28E-02	rs16851199	1_230244207_A_G	0.94	7174	9.37E-06	GCST90277368
Triacylglycerol (51:2) levels	2	47216823	T	C	-1.20E-01	2.62E-02	1.13E-01	rs12622488	2_47216823_C_T	0.98	7174	5.02E-06	GCST90277368
Triacylglycerol (51:2) levels	2	57531720	C	G	-8.63E-02	1.90E-02	7.21E-01	rs10192465	2_57531720_G_C	0.94	7174	5.41E-06	GCST90277368
Triacylglycerol (51:2) levels	2	124364198	T	A	-1.51E-01	3.27E-02	6.99E-02	rs2553631	2_124364198_A_T	1.00	7174	4.24E-06	GCST90277368
Triacylglycerol (51:2) levels	2	209438053	C	G	7.43E-02	1.65E-02	5.01E-01	rs7594469	2_209438053_G_C	1.00	7174	7.05E-06	GCST90277368
Triacylglycerol (51:2) levels	3	4819419	C	G	7.85E-02	1.66E-02	5.25E-01	rs6768493	3_4819419_G_C	0.99	7174	2.27E-06	GCST90277368
Triacylglycerol (51:2) levels	3	186868271	T	C	1.80E-01	3.75E-02	5.20E-02	rs6444176	3_186868271_C_T	0.96	7174	1.59E-06	GCST90277368
Triacylglycerol (51:2) levels	4	4501634	T	C	4.06E-01	7.92E-02	1.19E-02	rs75418091	4_4501634_C_T	0.94	7174	3.03E-07	GCST90277368
Triacylglycerol (51:2) levels	4	60937521	A	G	-8.96E-02	1.94E-02	7.54E-01	rs2604631	4_60937521_G_A	1.00	7174	4.03E-06	GCST90277368
Triacylglycerol (51:2) levels	6	169320808	T	C	7.89E-02	1.68E-02	4.65E-01	rs12192157	6_169320808_C_T	0.98	7174	2.78E-06	GCST90277368
Triacylglycerol (51:2) levels	9	5837780	A	G	2.46E-01	4.87E-02	3.12E-02	rs10975312	9_5837780_G_A	0.98	7174	4.55E-07	GCST90277368
Triacylglycerol (51:2) levels	9	6764828	A	G	-1.04E-01	2.25E-02	1.65E-01	rs58001441	9_6764828_G_A	1.00	7174	4.21E-06	GCST90277368
Triacylglycerol (51:2) levels	9	28896200	G	A	1.30E-01	2.92E-02	9.22E-02	rs10968815	9_28896200_A_G	0.99	7174	8.82E-06	GCST90277368
Triacylglycerol (51:2) levels	10	14269684	C	A	9.96E-02	2.23E-02	1.70E-01	rs1218344	10_14269684_A_C	1.00	7174	7.92E-06	GCST90277368
Triacylglycerol (51:2) levels	11	124076588	G	A	8.16E-02	1.67E-02	4.83E-01	rs3741111	11_124076588_A_G	0.99	7174	1.02E-06	GCST90277368
Triacylglycerol (51:2) levels	12	79722154	T	C	-2.21E-01	4.86E-02	3.29E-02	rs61927425	12_79722154_C_T	0.89	7174	5.53E-06	GCST90277368
Triacylglycerol (51:2) levels	12	106336256	T	C	-9.45E-02	2.11E-02	1.97E-01	rs10717199	12_106336256_C_T	0.99	7174	7.30E-06	GCST90277368
Triacylglycerol (51:2) levels	14	22820083	G	C	2.82E-01	6.26E-02	2.01E-02	rs61977690	14_22820083_C_G	0.89	7174	7.03E-06	GCST90277368
Triacylglycerol (51:2) levels	14	63768838	T	G	5.13E-01	2.44E-02	1.25E-01	rs7157785	14_63768838_G_T	1.00	7174	3.08E-95	GCST90277368
Triacylglycerol (51:2) levels	14	65115063	G	A	-3.48E-01	7.17E-02	9.86E-01	rs8017356	14_65115063_A_G	0.99	7174	1.29E-06	GCST90277368
Triacylglycerol (51:2) levels	15	58386313	T	C	8.35E-02	1.75E-02	3.38E-01	rs10468017	15_58386313_C_T	1.00	7174	1.82E-06	GCST90277368
Triacylglycerol (51:2) levels	15	93611333	C	G	8.15E-02	1.78E-02	3.32E-01	rs72649550	15_93611333_G_C	0.98	7174	4.73E-06	GCST90277368
Triacylglycerol (51:2) levels	16	55539036	G	A	-5.84E-01	1.27E-01	4.50E-03	rs117510063	16_55539036_A_G	0.94	7174	4.59E-06	GCST90277368
Triacylglycerol (51:2) levels	16	72072066	C	T	-9.55E-02	1.98E-02	2.28E-01	rs217184	16_72072066_T_C	0.99	7174	1.49E-06	GCST90277368
Triacylglycerol (51:2) levels	17	4764677	A	C	-2.56E-01	5.37E-02	2.58E-02	rs75679663	17_4764677_C_A	0.98	7174	1.86E-06	GCST90277368
Triacylglycerol (51:2) levels	17	5384095	A	G	-1.75E-01	3.12E-02	7.46E-02	rs3026120	17_5384095_G_A	1.00	7174	2.14E-08	GCST90277368
Triacylglycerol (51:2) levels	19	11079868	T	C	-1.53E-01	2.81E-02	9.46E-02	rs118068660	19_11079868_C_T	1.00	7174	5.34E-08	GCST90277368
Triacylglycerol (51:2) levels	19	19684751	A	C	-8.00E-02	1.74E-02	3.94E-01	rs7247433	19_19684751_C_A	0.96	7174	4.21E-06	GCST90277368
Triacylglycerol (51:2) levels	20	12982070	G	A	-1.10E-01	1.76E-02	6.61E-01	rs364585	20_12982070_A_G	1.00	7174	4.03E-10	GCST90277368
Triacylglycerol (51:2) levels	20	33730205	A	G	-3.28E-01	7.01E-02	1.49E-02	rs138440819	20_33730205_G_A	0.93	7174	2.89E-06	GCST90277368
Triacylglycerol (51:2) levels	21	34990612	T	C	1.19E-01	2.64E-02	1.15E-01	rs76596580	21_34990612_C_T	0.96	7174	6.91E-06	GCST90277368
Triacylglycerol (51:3) levels	1	55039974	T	G	-2.56E-01	5.28E-02	3.32E-02	rs11591147	1_55039974_G_T	1.00	6207	1.33E-06	GCST90277369
Triacylglycerol (51:3) levels	1	198625465	T	C	1.78E-01	3.65E-02	9.34E-01	rs6683774	1_198625465_C_T	0.97	6207	1.17E-06	GCST90277369
Triacylglycerol (51:3) levels	1	222184468	G	A	1.19E-01	2.52E-02	1.50E-01	rs17497891	1_222184468_A_G	0.99	6207	2.51E-06	GCST90277369
Triacylglycerol (51:3) levels	2	21055901	T	G	9.78E-02	1.98E-02	2.97E-01	rs934198	2_21055901_G_T	1.00	6207	8.40E-07	GCST90277369
Triacylglycerol (51:3) levels	2	75724208	C	G	-3.00E-01	6.69E-02	1.97E-02	rs114372832	2_75724208_G_C	0.99	6207	7.69E-06	GCST90277369
Triacylglycerol (51:3) levels	3	113313552	C	T	-3.99E-01	8.36E-02	1.24E-02	rs55811539	3_113313552_T_C	0.99	6207	1.83E-06	GCST90277369
Triacylglycerol (51:3) levels	4	72876701	A	G	2.62E-01	5.10E-02	3.16E-02	rs112585713	4_72876701_G_A	0.98	6207	2.96E-07	GCST90277369
Triacylglycerol (51:3) levels	4	74006728	G	A	2.27E-01	5.04E-02	3.33E-02	rs16850360	4_74006728_A_G	0.99	6207	6.51E-06	GCST90277369
Triacylglycerol (51:3) levels	7	11194365	G	A	-3.77E-01	8.51E-02	1.16E-02	rs140560087	7_11194365_A_G	0.93	6207	9.31E-06	GCST90277369
Triacylglycerol (51:3) levels	8	122227701	T	G	-8.70E-02	1.93E-02	3.28E-01	rs7814780	8_122227701_G_T	1.00	6207	6.75E-06	GCST90277369
Triacylglycerol (51:3) levels	8	142178314	A	C	-2.69E-01	5.90E-02	2.56E-02	rs35275531	8_142178314_C_A	0.90	6207	5.38E-06	GCST90277369
Triacylglycerol (51:3) levels	9	80858018	G	T	9.00E-02	2.01E-02	2.71E-01	rs1324162	9_80858018_T_G	1.00	6207	7.81E-06	GCST90277369

Triacylglycerol (51:3) levels	9	98537277	C	A	-9.79E-02	2.11E-02	2.42E-01	rs2808569	9_98537277_A_C	1.00	6207	3.51E-06	GCST90277369
Triacylglycerol (51:3) levels	10	106229406	T	C	-1.82E-01	3.90E-02	5.50E-02	rs80130206	10_106229406_C_T	0.99	6207	2.99E-06	GCST90277369
Triacylglycerol (51:3) levels	11	59731240	A	G	-6.45E-01	1.46E-01	3.74E-03	rs113312978	11_59731240_G_A	0.89	6207	9.79E-06	GCST90277369
Triacylglycerol (51:3) levels	11	61783884	C	T	-9.76E-02	1.84E-02	4.09E-01	rs174535	11_61783884_T_C	1.00	6207	1.14E-07	GCST90277369
Triacylglycerol (51:3) levels	14	63769473	C	T	-3.27E-01	2.71E-02	1.25E-01	rs12880341	14_63769473_T_C	1.00	6207	3.94E-33	GCST90277369
Triacylglycerol (51:3) levels	15	57322347	T	C	-9.12E-02	1.87E-02	6.18E-01	rs11631073	15_57322347_C_T	0.99	6207	1.11E-06	GCST90277369
Triacylglycerol (51:3) levels	17	3769209	A	T	-5.60E-01	1.01E-01	8.82E-03	rs55985238	17_3769209_T_A	0.90	6207	2.72E-08	GCST90277369
Triacylglycerol (51:3) levels	17	4764677	A	C	-6.89E-01	6.23E-02	2.58E-02	rs75679663	17_4764677_C_A	0.98	6207	3.31E-28	GCST90277369
Triacylglycerol (51:3) levels	17	5384095	A	G	-2.07E-01	3.42E-02	7.46E-02	rs3026120	17_5384095_G_A	1.00	6207	1.44E-09	GCST90277369
Triacylglycerol (51:3) levels	17	80990283	A	C	8.48E-02	1.81E-02	5.69E-01	rs8071514	17_80990283_C_A	1.00	6207	2.80E-06	GCST90277369
Triacylglycerol (51:3) levels	19	11082239	G	A	-1.53E-01	3.16E-02	9.21E-02	rs73015021	19_11082239_A_G	0.98	6207	1.32E-06	GCST90277369
Triacylglycerol (51:3) levels	19	48629610	C	G	-2.41E-01	5.30E-02	3.05E-02	rs61751862	19_48629610_G_C	1.00	6207	5.67E-06	GCST90277369
Triacylglycerol (51:3) levels	20	11012603	G	A	-8.23E-02	1.83E-02	3.97E-01	rs1073042	20_11012603_A_G	0.99	6207	6.87E-06	GCST90277369
Triacylglycerol (51:3) levels	21	43371358	C	T	9.00E-02	1.91E-02	3.40E-01	rs595587	21_43371358_T_C	0.99	6207	2.56E-06	GCST90277369
Triacylglycerol (51:3) levels	22	19694202	A	G	-3.01E-01	6.79E-02	1.98E-02	rs1989780	22_19694202_G_A	0.91	6207	9.27E-06	GCST90277369
Triacylglycerol (51:4) levels	1	55039974	T	G	-4.19E-01	4.64E-02	3.32E-02	rs11591147	1_55039974_G_T	1.00	7174	2.32E-19	GCST90277370
Triacylglycerol (51:4) levels	1	180552628	T	G	8.16E-02	1.74E-02	6.27E-01	rs1934410	1_180552628_G_T	1.00	7174	2.86E-06	GCST90277370
Triacylglycerol (51:4) levels	2	21050618	T	G	1.00E-01	1.85E-02	2.96E-01	rs7575840	2_21050618_G_T	1.00	7174	5.50E-08	GCST90277370
Triacylglycerol (51:4) levels	2	138816573	C	T	8.42E-02	1.90E-02	7.38E-01	rs4954960	2_138816573_T_C	0.99	7174	9.90E-06	GCST90277370
Triacylglycerol (51:4) levels	2	181902451	G	C	5.98E-01	1.25E-01	5.41E-03	rs34087767	2_181902451_C_G	0.87	7174	1.78E-06	GCST90277370
Triacylglycerol (51:4) levels	4	72899760	A	G	2.88E-01	4.42E-02	3.78E-02	rs74915447	4_72899760_G_A	0.97	7174	7.50E-11	GCST90277370
Triacylglycerol (51:4) levels	4	73124451	T	C	2.82E-01	4.48E-02	3.81E-02	rs114483871	4_73124451_C_T	0.93	7174	3.24E-10	GCST90277370
Triacylglycerol (51:4) levels	4	74505174	T	C	3.77E-01	5.07E-02	2.81E-02	rs116302332	4_74505174_C_T	0.96	7174	1.28E-13	GCST90277370
Triacylglycerol (51:4) levels	4	162543049	G	A	1.26E-01	2.46E-02	1.35E-01	rs35577358	4_162543049_A_G	0.99	7174	2.84E-07	GCST90277370
Triacylglycerol (51:4) levels	5	126552164	G	A	-1.01E-01	2.14E-02	1.83E-01	rs2306619	5_126552164_A_G	1.00	7174	2.14E-06	GCST90277370
Triacylglycerol (51:4) levels	8	9315848	A	G	7.99E-02	1.68E-02	5.12E-01	rs7012814	8_9315848_G_A	0.99	7174	2.09E-06	GCST90277370
Triacylglycerol (51:4) levels	9	5527351	G	A	3.43E-01	7.05E-02	1.49E-02	rs78143047	9_5527351_A_G	0.94	7174	1.19E-06	GCST90277370
Triacylglycerol (51:4) levels	9	114372665	C	T	8.42E-02	1.67E-02	4.81E-01	rs7032795	9_114372665_T_C	1.00	7174	4.47E-07	GCST90277370
Triacylglycerol (51:4) levels	9	133276354	T	C	-8.90E-02	2.00E-02	7.78E-01	rs600038	9_133276354_C_T	1.00	7174	8.70E-06	GCST90277370
Triacylglycerol (51:4) levels	10	52220949	GT	G	7.99E-02	1.69E-02	5.79E-01	rs11404918	10_52220949_G_GT	0.99	7174	2.38E-06	GCST90277370
Triacylglycerol (51:4) levels	10	71241186	G	A	-7.61E-02	1.72E-02	3.74E-01	rs2894147	10_71241186_A_G	1.00	7174	9.47E-06	GCST90277370
Triacylglycerol (51:4) levels	11	61796827	T	G	-7.49E-02	1.68E-02	4.36E-01	rs4246215	11_61796827_G_T	0.99	7174	8.47E-06	GCST90277370
Triacylglycerol (51:4) levels	11	103250744	C	A	-9.27E-02	2.09E-02	2.00E-01	rs3912622	11_103250744_A_C	1.00	7174	9.57E-06	GCST90277370
Triacylglycerol (51:4) levels	12	52892255	A	G	-3.34E-01	6.94E-02	1.44E-02	rs117279275	12_52892255_G_A	1.00	7174	1.52E-06	GCST90277370
Triacylglycerol (51:4) levels	13	47201342	T	C	-2.44E-01	5.30E-02	2.72E-02	rs191640693	13_47201342_C_T	0.95	7174	4.13E-06	GCST90277370
Triacylglycerol (51:4) levels	14	69889451	A	C	1.42E-01	3.11E-02	7.89E-02	rs722814	14_69889451_C_A	0.99	7174	4.74E-06	GCST90277370
Triacylglycerol (51:4) levels	14	86739725	C	T	7.71E-02	1.67E-02	5.63E-01	rs56108088	14_86739725_T_C	0.99	7174	4.11E-06	GCST90277370
Triacylglycerol (51:4) levels	15	58387469	A	G	1.10E-01	1.69E-02	3.93E-01	rs7350789	15_58387469_G_A	1.00	7174	6.71E-11	GCST90277370
Triacylglycerol (51:4) levels	16	51029992	G	A	2.19E-01	4.52E-02	4.08E-02	rs141286087	16_51029992_A_G	0.86	7174	1.31E-06	GCST90277370
Triacylglycerol (51:4) levels	16	76342662	T	C	-4.99E-01	1.05E-01	7.15E-03	rs72794954	16_76342662_C_T	0.93	7174	1.93E-06	GCST90277370
Triacylglycerol (51:4) levels	17	4764677	A	C	-3.04E-01	5.37E-02	2.58E-02	rs75679663	17_4764677_C_A	0.98	7174	1.50E-08	GCST90277370
Triacylglycerol (51:4) levels	17	5384095	A	G	-1.62E-01	3.12E-02	7.46E-02	rs3026120	17_5384095_G_A	1.00	7174	2.01E-07	GCST90277370
Triacylglycerol (51:4) levels	19	11079868	T	C	-2.44E-01	2.80E-02	9.46E-02	rs118068660	19_11079868_C_T	1.00	7174	4.10E-18	GCST90277370
Triacylglycerol (51:4) levels	19	41030717	G	A	-7.68E-02	1.73E-02	6.30E-01	rs7257703	19_41030717_A_G	0.99	7174	9.08E-06	GCST90277370
Triacylglycerol (51:4) levels	19	44908822	T	C	-1.99E-01	3.72E-02	5.31E-02	rs7412	19_44908822_C_T	1.00	7174	9.41E-08	GCST90277370
Triacylglycerol (51:4) levels	20	44413724	T	C	-1.89E-01	3.72E-02	5.24E-02	rs1800961	20_44413724_C_T	1.00	7174	3.93E-07	GCST90277370
Triacylglycerol (51:4) levels	21	38573905	A	G	1.67E-01	3.58E-02	5.97E-02	rs2836524	21_38573905_G_A	0.96	7174	2.90E-06	GCST90277370
Triacylglycerol (52:2) levels	1	44173695	G	C	2.67E-01	5.88E-02	2.02E-02	rs56163454	1_44173695_C_G	0.99	7174	5.58E-06	GCST90277371
Triacylglycerol (52:2) levels	1	55039974	T	G	-2.46E-01	4.65E-02	3.32E-02	rs11591147	1_55039974_G_T	1.00	7174	1.27E-07	GCST90277371
Triacylglycerol (52:2) levels	3	186868495	G	C	-9.53E-02	2.11E-02	8.01E-01	rs9835223	3_186868495_C_G	0.96	7174	6.54E-06	GCST90277371
Triacylglycerol (52:2) levels	4	10615596	T	C	3.71E-01	8.33E-02	1.05E-02	rs2531191	4_10615596_C_T	0.95	7174	8.23E-06	GCST90277371
Triacylglycerol (52:2) levels	4	72899760	A	G	1.96E-01	4.43E-02	3.78E-02	rs74915447	4_72899760_G_A	0.97	7174	9.99E-06	GCST90277371
Triacylglycerol (52:2) levels	4	74505174	T	C	2.98E-01	5.08E-02	2.81E-02	rs116302332	4_74505174_C_T	0.96	7174	4.74E-09	GCST90277371
Triacylglycerol (52:2) levels	5	66284563	G	C	-9.19E-02	2.04E-02	2.26E-01	rs10471676	5_66284563_C_G	0.92	7174	6.75E-06	GCST90277371
Triacylglycerol (52:2) levels	5	147384423	T	C	7.93E-01	1.76E-01	2.70E-03	rs146136464	5_147384423_C_T	0.87	7174	6.79E-06	GCST90277371
Triacylglycerol (52:2) levels	6	67724705	T	A	-6.56E-01	1.40E-01	3.91E-03	rs62412280	6_67724705_A_T	0.90	7174	2.97E-06	GCST90277371
Triacylglycerol (52:2) levels	7	89538067	T	C	4.77E-01	1.03E-01	7.06E-03	rs7787163	7_89538067_C_T	0.95	7174	3.33E-06	GCST90277371

Triacylglycerol (52:2) levels	7	110130812	C	T	6.96E-01	1.54E-01	3.46E-03	rs1404846	7_110130812_T_C	0.92	7174	6.24E-06 GCST90277371
Triacylglycerol (52:2) levels	8	9315848	A	G	8.66E-02	1.68E-02	5.12E-01	rs7012814	8_9315848_G_A	0.99	7174	2.67E-07 GCST90277371
Triacylglycerol (52:2) levels	9	5837780	A	G	2.37E-01	4.87E-02	3.12E-02	rs10975312	9_5837780_G_A	0.98	7174	1.18E-06 GCST90277371
Triacylglycerol (52:2) levels	9	80862163	G	C	9.20E-02	2.07E-02	2.07E-01	rs10867632	9_80862163_C_G	0.99	7174	8.84E-06 GCST90277371
Triacylglycerol (52:2) levels	10	70837164	A	G	-2.17E-01	3.83E-02	4.98E-02	rs113127305	10_70837164_G_A	1.00	7174	1.46E-08 GCST90277371
Triacylglycerol (52:2) levels	11	61815236	C	T	-1.70E-01	1.72E-02	3.81E-01	rs174561	11_61815236_T_C	1.00	7174	7.26E-23 GCST90277371
Triacylglycerol (52:2) levels	11	103250744	C	A	-9.29E-02	2.09E-02	2.00E-01	rs3912622	11_103250744_A_C	1.00	7174	9.05E-06 GCST90277371
Triacylglycerol (52:2) levels	12	124846478	C	T	1.10E-01	2.31E-02	1.50E-01	rs117009181	12_124846478_T_C	0.98	7174	2.03E-06 GCST90277371
Triacylglycerol (52:2) levels	13	33753876	C	T	1.71E-01	3.67E-02	5.37E-02	rs111507730	13_33753876_T_C	1.00	7174	3.33E-06 GCST90277371
Triacylglycerol (52:2) levels	14	20017956	T	C	-2.14E-01	4.60E-02	3.35E-02	rs17276940	14_20017956_C_T	0.99	7174	3.48E-06 GCST90277371
Triacylglycerol (52:2) levels	14	99309314	A	G	-1.05E-01	2.22E-02	1.76E-01	rs1033030	14_99309314_G_A	0.96	7174	2.16E-06 GCST90277371
Triacylglycerol (52:2) levels	15	58391167	G	A	-1.09E-01	1.67E-02	5.73E-01	rs1532085	15_58391167_A_G	1.00	7174	7.57E-11 GCST90277371
Triacylglycerol (52:2) levels	15	80029927	A	C	-9.62E-02	2.04E-02	2.11E-01	rs939971	15_80029927_C_A	1.00	7174	2.43E-06 GCST90277371
Triacylglycerol (52:2) levels	17	4482208	T	C	8.53E-02	1.86E-02	3.26E-01	rs12952341	17_4482208_C_T	0.90	7174	4.84E-06 GCST90277371
Triacylglycerol (52:2) levels	19	11078596	C	T	-1.86E-01	2.81E-02	9.43E-02	rs113722226	19_11078596_T_C	1.00	7174	3.70E-11 GCST90277371
Triacylglycerol (52:2) levels	19	19684751	A	C	-7.77E-02	1.74E-02	3.94E-01	rs7247433	19_19684751_C_A	0.96	7174	7.82E-06 GCST90277371
Triacylglycerol (52:2) levels	19	48629610	C	G	2.53E-01	4.79E-02	3.05E-02	rs61751862	19_48629610_G_C	1.00	7174	1.39E-07 GCST90277371
Triacylglycerol (52:2) levels	20	44413724	T	C	-1.68E-01	3.72E-02	5.24E-02	rs1800961	20_44413724_C_T	1.00	7174	5.97E-06 GCST90277371
Triacylglycerol (52:3) levels	1	55039974	T	G	-2.59E-01	4.66E-02	3.32E-02	rs11591147	1_55039974_G_T	1.00	7174	2.96E-08 GCST90277372
Triacylglycerol (52:3) levels	2	50050811	G	T	-1.17E-01	2.40E-02	1.38E-01	rs1452773	2_50050811_T_G	0.99	7174	1.10E-06 GCST90277372
Triacylglycerol (52:3) levels	2	60982893	A	G	2.29E-01	4.79E-02	3.26E-02	rs12464353	2_60982893_G_A	0.99	7174	1.74E-06 GCST90277372
Triacylglycerol (52:3) levels	2	241457278	T	G	8.11E-02	1.69E-02	4.68E-01	rs886813	2_241457278_G_T	0.98	7174	1.61E-06 GCST90277372
Triacylglycerol (52:3) levels	3	194176577	C	A	-9.00E-02	2.02E-02	2.41E-01	rs73073606	3_194176577_A_C	0.94	7174	8.25E-06 GCST90277372
Triacylglycerol (52:3) levels	4	72876701	A	G	2.82E-01	4.80E-02	3.16E-02	rs112585713	4_72876701_G_A	0.98	7174	4.41E-09 GCST90277372
Triacylglycerol (52:3) levels	4	73424911	T	C	2.34E-01	4.09E-02	4.51E-02	rs79146711	4_73424911_C_T	0.94	7174	1.02E-08 GCST90277372
Triacylglycerol (52:3) levels	4	73947510	C	A	3.39E-01	5.29E-02	2.53E-02	rs182695896	4_73947510_A_C	0.96	7174	1.58E-10 GCST90277372
Triacylglycerol (52:3) levels	4	110405753	G	A	-7.87E-02	1.72E-02	4.16E-01	rs6829637	4_110405753_A_G	0.97	7174	4.77E-06 GCST90277372
Triacylglycerol (52:3) levels	5	90249218	G	A	-4.45E-01	9.46E-02	8.23E-03	rs114839471	5_90249218_A_G	0.96	7174	2.59E-06 GCST90277372
Triacylglycerol (52:3) levels	5	174239481	T	C	-7.53E-02	1.67E-02	5.10E-01	rs884532	5_174239481_C_T	1.00	7174	6.95E-06 GCST90277372
Triacylglycerol (52:3) levels	6	2365034	G	A	-1.13E-01	2.53E-02	1.26E-01	rs4144237	6_2365034_A_G	0.98	7174	8.50E-06 GCST90277372
Triacylglycerol (52:3) levels	6	13187371	C	T	1.67E-01	3.67E-02	5.74E-02	rs17664981	6_13187371_T_C	0.96	7174	5.34E-06 GCST90277372
Triacylglycerol (52:3) levels	6	160682773	A	T	1.61E-01	3.63E-02	5.74E-02	rs374071816	6_160682773_T_A	0.98	7174	9.69E-06 GCST90277372
Triacylglycerol (52:3) levels	8	4789612	A	G	1.26E-01	2.64E-02	1.12E-01	rs12675806	8_4789612_G_A	1.00	7174	1.90E-06 GCST90277372
Triacylglycerol (52:3) levels	8	29518250	C	T	-1.40E-01	2.77E-02	1.01E-01	rs6558122	8_29518250_T_C	0.99	7174	4.58E-07 GCST90277372
Triacylglycerol (52:3) levels	8	71073538	G	T	1.14E-01	2.56E-02	1.26E-01	rs11779209	8_71073538_T_G	0.97	7174	9.39E-06 GCST90277372
Triacylglycerol (52:3) levels	9	15305380	C	G	1.14E-01	2.37E-02	8.56E-01	rs581080	9_15305380_G_C	1.00	7174	1.47E-06 GCST90277372
Triacylglycerol (52:3) levels	11	4966841	A	G	6.09E-01	1.38E-01	3.87E-03	rs117442205	11_4966841_G_A	0.93	7174	9.71E-06 GCST90277372
Triacylglycerol (52:3) levels	11	48198260	T	G	9.16E-02	2.01E-02	7.83E-01	rs10838826	11_48198260_G_T	1.00	7174	5.13E-06 GCST90277372
Triacylglycerol (52:3) levels	11	61796827	T	G	-7.48E-02	1.68E-02	4.36E-01	rs4246215	11_61796827_G_T	0.99	7174	8.88E-06 GCST90277372
Triacylglycerol (52:3) levels	12	109256177	T	C	-7.66E-02	1.69E-02	4.23E-01	rs3742023	12_109256177_C_T	1.00	7174	5.63E-06 GCST90277372
Triacylglycerol (52:3) levels	12	121000508	T	C	-8.53E-02	1.74E-02	3.60E-01	rs1169306	12_121000508_C_T	1.00	7174	9.27E-07 GCST90277372
Triacylglycerol (52:3) levels	14	22831454	A	G	-7.71E-02	1.74E-02	3.60E-01	rs4981443	14_22831454_G_A	1.00	7174	9.63E-06 GCST90277372
Triacylglycerol (52:3) levels	14	63768838	T	G	1.36E-01	2.51E-02	1.25E-01	rs7157785	14_63768838_G_T	1.00	7174	6.56E-08 GCST90277372
Triacylglycerol (52:3) levels	15	68380799	A	G	1.93E-01	4.24E-02	4.49E-02	rs138524673	15_68380799_G_A	0.92	7174	5.75E-06 GCST90277372
Triacylglycerol (52:3) levels	17	3547599	G	T	-4.16E-01	8.40E-02	1.01E-02	rs186039163	17_3547599_T_G	0.97	7174	7.74E-07 GCST90277372
Triacylglycerol (52:3) levels	17	4764677	A	C	-3.25E-01	5.37E-02	2.58E-02	rs75679663	17_4764677_C_A	0.98	7174	1.42E-09 GCST90277372
Triacylglycerol (52:3) levels	17	5065669	T	C	-1.71E-01	3.41E-02	6.20E-02	rs117643293	17_5065669_C_T	0.99	7174	5.50E-07 GCST90277372
Triacylglycerol (52:3) levels	17	77755768	T	C	2.86E-01	6.35E-02	1.93E-02	rs72898481	17_77755768_C_T	0.91	7174	6.72E-06 GCST90277372
Triacylglycerol (52:3) levels	19	8209156	C	G	1.95E-01	1.70E-02	3.99E-01	rs2336171	19_8209156_G_C	0.99	7174	2.94E-30 GCST90277372
Triacylglycerol (52:3) levels	19	8236164	T	C	1.45E-01	1.90E-02	7.21E-01	rs7248003	19_8236164_C_T	0.94	7174	2.69E-14 GCST90277372
Triacylglycerol (52:3) levels	19	8607097	C	T	-1.08E-01	1.93E-02	2.61E-01	rs12462619	19_8607097_T_C	0.97	7174	2.32E-08 GCST90277372
Triacylglycerol (52:3) levels	20	12982070	G	A	-1.09E-01	1.76E-02	6.61E-01	rs364585	20_12982070_A_G	1.00	7174	5.55E-10 GCST90277372
Triacylglycerol (52:3) levels	20	51460945	C	T	1.29E-01	2.50E-02	1.34E-01	rs4809845	20_51460945_T_C	0.97	7174	2.41E-07 GCST90277372
Triacylglycerol (52:3) levels	22	17172477	T	C	-8.81E-02	1.93E-02	2.57E-01	rs8139868	22_17172477_C_T	0.99	7174	5.04E-06 GCST90277372
Triacylglycerol (52:4) levels	2	50050811	G	T	-1.09E-01	2.40E-02	1.38E-01	rs1452773	2_50050811_T_G	0.99	7173	5.98E-06 GCST90277373
Triacylglycerol (52:4) levels	2	60982893	A	G	2.21E-01	4.79E-02	3.26E-02	rs12464353	2_60982893_G_A	0.99	7173	4.04E-06 GCST90277373
Triacylglycerol (52:4) levels	2	64114191	G	T	-5.04E-01	1.13E-01	5.98E-03	rs13005731	2_64114191_T_G	0.90	7173	7.86E-06 GCST90277373

Triacylglycerol (52:4) levels	2	203425314	C	T	-1.48E-01	2.91E-02	9.10E-02	rs78999781	2_203425314_T_C	1.00	7173	3.95E-07	GCST90277373
Triacylglycerol (52:4) levels	3	19323998	A	G	-3.61E-01	8.08E-02	1.09E-02	rs146465816	3_19323998_G_A	0.96	7173	8.07E-06	GCST90277373
Triacylglycerol (52:4) levels	3	186868271	T	C	1.75E-01	3.76E-02	5.20E-02	rs6444176	3_186868271_C_T	0.96	7173	3.22E-06	GCST90277373
Triacylglycerol (52:4) levels	4	72803111	A	G	2.43E-01	5.38E-02	2.56E-02	rs77645768	4_72803111_G_A	0.97	7173	6.45E-06	GCST90277373
Triacylglycerol (52:4) levels	4	73947510	C	A	3.03E-01	5.30E-02	2.53E-02	rs182695896	4_73947510_A_C	0.96	7173	1.12E-08	GCST90277373
Triacylglycerol (52:4) levels	4	113100013	A	G	-7.65E-02	1.70E-02	3.96E-01	rs313950	4_113100013_G_A	1.00	7173	7.32E-06	GCST90277373
Triacylglycerol (52:4) levels	5	35225964	A	G	-2.88E-01	6.45E-02	1.68E-02	rs146253188	5_35225964_G_A	1.00	7173	8.07E-06	GCST90277373
Triacylglycerol (52:4) levels	5	130859001	A	G	-7.95E-02	1.73E-02	3.54E-01	rs7700432	5_130859001_G_A	1.00	7173	4.55E-06	GCST90277373
Triacylglycerol (52:4) levels	6	10952103	A	G	-9.64E-02	2.16E-02	1.82E-01	rs12207488	6_10952103_G_A	1.00	7173	8.21E-06	GCST90277373
Triacylglycerol (52:4) levels	6	67518953	TCA	T	7.70E-01	1.59E-01	9.97E-01	rs10650176	6_67518953_T_TCA	0.85	7173	1.40E-06	GCST90277373
Triacylglycerol (52:4) levels	6	98098087	G	T	-1.90E-01	3.99E-02	4.73E-02	rs117546336	6_98098087_T_G	0.96	7173	2.00E-06	GCST90277373
Triacylglycerol (52:4) levels	9	15305380	C	G	1.08E-01	2.37E-02	8.56E-01	rs581080	9_15305380_G_C	1.00	7173	5.20E-06	GCST90277373
Triacylglycerol (52:4) levels	10	70899547	A	G	-1.88E-01	4.23E-02	4.19E-02	rs7920200	10_70899547_G_A	0.96	7173	8.75E-06	GCST90277373
Triacylglycerol (52:4) levels	11	61800281	A	C	-1.78E-01	1.72E-02	3.80E-01	rs174544	11_61800281_C_A	1.00	7173	5.30E-25	GCST90277373
Triacylglycerol (52:4) levels	12	120985856	G	A	1.01E-01	1.69E-02	5.79E-01	rs9738226	12_120985856_A_G	1.00	7173	2.37E-09	GCST90277373
Triacylglycerol (52:4) levels	14	20017956	T	C	-2.19E-01	4.61E-02	3.35E-02	rs17276940	14_20017956_C_T	0.99	7173	2.12E-06	GCST90277373
Triacylglycerol (52:4) levels	18	13203131	A	G	3.82E-01	8.34E-02	1.05E-02	rs72879665	18_13203131_G_A	0.94	7173	4.73E-06	GCST90277373
Triacylglycerol (52:4) levels	19	8222034	C	T	1.81E-01	1.77E-02	6.75E-01	rs7253584	19_8222034_T_C	0.99	7173	2.48E-24	GCST90277373
Triacylglycerol (52:4) levels	19	8602475	T	C	-1.03E-01	1.93E-02	2.67E-01	rs7247304	19_8602475_C_T	0.96	7173	9.07E-08	GCST90277373
Triacylglycerol (52:4) levels	20	37965215	T	C	1.53E-01	3.46E-02	6.22E-02	rs68137603	20_37965215_C_T	1.00	7173	9.80E-06	GCST90277373
Triacylglycerol (52:4) levels	22	48612019	A	G	4.01E-01	8.72E-02	1.06E-02	rs148860058	22_48612019_G_A	0.88	7173	4.37E-06	GCST90277373
Triacylglycerol (52:5) levels	1	8738706	T	C	3.34E-01	7.19E-02	1.40E-02	rs76174849	1_8738706_C_T	0.97	7174	3.44E-06	GCST90277374
Triacylglycerol (52:5) levels	1	55039974	T	G	-3.01E-01	4.65E-02	3.32E-02	rs11591147	1_55039974_G_T	1.00	7174	1.07E-10	GCST90277374
Triacylglycerol (52:5) levels	2	50050811	G	T	-1.10E-01	2.39E-02	1.38E-01	rs1452773	2_50050811_T_G	0.99	7174	4.26E-06	GCST90277374
Triacylglycerol (52:5) levels	2	146363675	GT	G	3.03E-01	6.38E-02	1.76E-02	rs200949196	2_146363675_G_GT	0.97	7174	2.03E-06	GCST90277374
Triacylglycerol (52:5) levels	2	157097431	G	C	-1.78E-01	3.95E-02	5.12E-02	rs188346987	2_157097431_C_G	0.91	7174	6.67E-06	GCST90277374
Triacylglycerol (52:5) levels	3	105974935	A	C	-2.43E-01	5.39E-02	2.60E-02	rs73193962	3_105974935_C_A	0.95	7174	6.41E-06	GCST90277374
Triacylglycerol (52:5) levels	3	194176577	C	A	-9.65E-02	2.01E-02	2.41E-01	rs73073606	3_194176577_A_C	0.94	7174	1.68E-06	GCST90277374
Triacylglycerol (52:5) levels	4	72803111	A	G	3.04E-01	5.36E-02	2.56E-02	rs77645768	4_72803111_G_A	0.97	7174	1.53E-08	GCST90277374
Triacylglycerol (52:5) levels	4	73124451	T	C	2.68E-01	4.48E-02	3.81E-02	rs114483871	4_73124451_C_T	0.93	7174	2.19E-09	GCST90277374
Triacylglycerol (52:5) levels	4	73947510	C	A	3.71E-01	5.28E-02	2.53E-02	rs182695896	4_73947510_A_C	0.96	7174	2.53E-12	GCST90277374
Triacylglycerol (52:5) levels	4	114762666	AATATAT	A	-7.56E-02	1.69E-02	4.06E-01	rs146127446	4_114762666_A_AATAT	0.99	7174	8.13E-06	GCST90277374
Triacylglycerol (52:5) levels	6	160682773	A	T	1.90E-01	3.63E-02	5.74E-02	rs374071816	6_160682773_T_A	0.98	7174	1.62E-07	GCST90277374
Triacylglycerol (52:5) levels	8	4789612	A	G	1.21E-01	2.64E-02	1.12E-01	rs12675806	8_4789612_G_A	1.00	7174	4.82E-06	GCST90277374
Triacylglycerol (52:5) levels	8	23959870	T	C	3.59E-01	7.86E-02	1.32E-02	rs147426822	8_23959870_C_T	0.88	7174	4.97E-06	GCST90277374
Triacylglycerol (52:5) levels	8	53151614	G	A	2.94E-01	6.55E-02	1.98E-02	rs79231873	8_53151614_A_G	0.83	7174	7.37E-06	GCST90277374
Triacylglycerol (52:5) levels	8	125480355	G	T	9.26E-02	1.94E-02	2.41E-01	rs13258507	8_125480355_T_G	1.00	7174	1.92E-06	GCST90277374
Triacylglycerol (52:5) levels	9	104975112	T	C	-9.19E-02	1.97E-02	2.33E-01	rs715119	9_104975112_C_T	1.00	7174	3.11E-06	GCST90277374
Triacylglycerol (52:5) levels	10	101716501	A	T	-8.30E-02	1.78E-02	6.69E-01	rs7478167	10_101716501_T_A	0.99	7174	3.36E-06	GCST90277374
Triacylglycerol (52:5) levels	11	95614411	G	C	2.30E-01	5.11E-02	3.07E-02	rs75740927	11_95614411_C_G	0.89	7174	7.25E-06	GCST90277374
Triacylglycerol (52:5) levels	11	124076588	G	A	8.64E-02	1.67E-02	4.83E-01	rs3741111	11_124076588_A_G	0.99	7174	2.19E-07	GCST90277374
Triacylglycerol (52:5) levels	12	61250942	A	C	2.58E-01	5.74E-02	2.22E-02	rs118070067	12_61250942_C_A	0.93	7174	7.31E-06	GCST90277374
Triacylglycerol (52:5) levels	12	67513820	A	C	-2.25E-01	4.78E-02	3.50E-02	rs7970512	12_67513820_C_A	0.87	7174	2.46E-06	GCST90277374
Triacylglycerol (52:5) levels	12	101922985	C	T	1.83E-01	3.92E-02	4.82E-02	rs117146578	12_101922985_T_C	1.00	7174	3.27E-06	GCST90277374
Triacylglycerol (52:5) levels	14	63768838	T	G	3.70E-01	2.48E-02	1.25E-01	rs7157785	14_63768838_G_T	1.00	7174	1.11E-49	GCST90277374
Triacylglycerol (52:5) levels	15	69995976	G	A	7.71E-02	1.74E-02	3.73E-01	rs7172051	15_69995976_A_G	0.97	7174	9.32E-06	GCST90277374
Triacylglycerol (52:5) levels	15	78071433	A	G	8.20E-02	1.82E-02	3.05E-01	rs2867985	15_78071433_G_A	1.00	7174	6.90E-06	GCST90277374
Triacylglycerol (52:5) levels	17	3547599	G	T	-3.81E-01	8.40E-02	1.01E-02	rs186039163	17_3547599_T_G	0.97	7174	5.68E-06	GCST90277374
Triacylglycerol (52:5) levels	17	4764677	A	C	-4.74E-01	5.35E-02	2.58E-02	rs75679663	17_4764677_C_A	0.98	7174	9.06E-19	GCST90277374
Triacylglycerol (52:5) levels	17	5065669	T	C	-2.43E-01	3.39E-02	6.20E-02	rs117643293	17_5065669_C_T	0.99	7174	8.25E-13	GCST90277374
Triacylglycerol (52:5) levels	18	71848979	C	T	-3.94E-01	8.19E-02	1.13E-02	rs144440646	18_71848979_T_C	0.94	7174	1.54E-06	GCST90277374
Triacylglycerol (52:5) levels	19	8206837	A	G	2.17E-01	1.69E-02	3.99E-01	rs7258249	19_8206837_G_A	1.00	7174	2.81E-37	GCST90277374
Triacylglycerol (52:5) levels	19	8236164	T	C	1.09E-01	1.91E-02	7.21E-01	rs7248003	19_8236164_C_T	0.94	7174	9.84E-09	GCST90277374
Triacylglycerol (52:5) levels	19	8607097	C	T	-9.63E-02	1.93E-02	2.61E-01	rs12462619	19_8607097_T_C	0.97	7174	6.04E-07	GCST90277374
Triacylglycerol (52:5) levels	19	19684751	A	C	-8.52E-02	1.74E-02	3.94E-01	rs7247433	19_19684751_C_A	0.96	7174	9.72E-07	GCST90277374
Triacylglycerol (52:5) levels	19	44908822	T	C	-1.76E-01	3.72E-02	5.31E-02	rs7412	19_44908822_C_T	1.00	7174	2.28E-06	GCST90277374
Triacylglycerol (52:5) levels	20	12982070	G	A	-1.64E-01	1.75E-02	6.61E-01	rs364585	20_12982070_A_G	1.00	7174	7.39E-21	GCST90277374

Triacylglycerol (52:5) levels	20	41303716	G	T	7.63E-02	1.68E-02	4.74E-01	rs12480916	20_41303716_T_G	1.00	7174	5.52E-06	GCST90277374
Triacylglycerol (52:5) levels	21	34990612	T	C	1.21E-01	2.64E-02	1.15E-01	rs76596580	21_34990612_C_T	0.96	7174	4.58E-06	GCST90277374
Triacylglycerol (52:6) levels	1	55039974	T	G	-2.19E-01	4.66E-02	3.32E-02	rs11591147	1_55039974_G_T	1.00	7150	2.84E-06	GCST90277375
Triacylglycerol (52:6) levels	2	46494121	G	A	-4.20E-01	9.37E-02	8.15E-03	rs116411822	2_46494121_A_G	0.97	7150	7.29E-06	GCST90277375
Triacylglycerol (52:6) levels	2	210749914	T	G	9.05E-02	1.90E-02	2.68E-01	rs10206976	2_210749914_G_T	1.00	7150	1.88E-06	GCST90277375
Triacylglycerol (52:6) levels	3	184623650	G	A	-1.04E-01	2.21E-02	1.80E-01	rs3936109	3_184623650_A_G	0.98	7150	2.72E-06	GCST90277375
Triacylglycerol (52:6) levels	4	72803111	A	G	2.68E-01	5.38E-02	2.56E-02	rs77645768	4_72803111_G_A	0.97	7150	6.16E-07	GCST90277375
Triacylglycerol (52:6) levels	4	73124451	T	C	2.20E-01	4.49E-02	3.81E-02	rs114483871	4_73124451_C_T	0.93	7150	9.38E-07	GCST90277375
Triacylglycerol (52:6) levels	4	73947510	C	A	3.26E-01	5.30E-02	2.53E-02	rs182695896	4_73947510_A_C	0.96	7150	8.57E-10	GCST90277375
Triacylglycerol (52:6) levels	6	48788044	A	T	1.97E-01	3.96E-02	4.57E-02	rs114717988	6_48788044_T_A	0.99	7150	6.37E-07	GCST90277375
Triacylglycerol (52:6) levels	9	15305380	C	G	1.15E-01	2.37E-02	8.56E-01	rs581080	9_15305380_G_C	1.00	7150	1.19E-06	GCST90277375
Triacylglycerol (52:6) levels	10	14418295	G	A	-4.59E-01	1.00E-01	7.87E-03	rs672959	10_14418295_A_G	0.89	7150	4.67E-06	GCST90277375
Triacylglycerol (52:6) levels	10	70459848	T	C	5.21E-01	1.00E-01	7.71E-03	rs139150589	10_70459848_C_T	0.91	7150	1.87E-07	GCST90277375
Triacylglycerol (52:6) levels	10	122833455	A	G	-1.08E-01	2.29E-02	1.59E-01	rs72839716	10_122833455_G_A	0.98	7150	2.53E-06	GCST90277375
Triacylglycerol (52:6) levels	11	61813163	T	C	-1.44E-01	1.72E-02	3.80E-01	rs174556	11_61813163_C_T	1.00	7150	6.46E-17	GCST90277375
Triacylglycerol (52:6) levels	11	69517944	T	C	8.17E-02	1.84E-02	2.84E-01	rs657315	11_69517944_C_T	1.00	7150	8.75E-06	GCST90277375
Triacylglycerol (52:6) levels	12	12341682	C	G	7.26E-01	1.60E-01	3.10E-03	rs75038600	12_12341682_G_C	0.85	7150	5.94E-06	GCST90277375
Triacylglycerol (52:6) levels	12	121000508	T	C	-1.21E-01	1.74E-02	3.60E-01	rs1169306	12_121000508_C_T	1.00	7150	4.14E-12	GCST90277375
Triacylglycerol (52:6) levels	13	62988645	G	A	5.27E-01	1.07E-01	6.17E-03	rs75098294	13_62988645_A_G	0.95	7150	9.06E-07	GCST90277375
Triacylglycerol (52:6) levels	14	20017956	T	C	-2.45E-01	4.61E-02	3.35E-02	rs17276940	14_20017956_C_T	0.99	7150	1.02E-07	GCST90277375
Triacylglycerol (52:6) levels	15	59274186	T	C	3.83E-01	7.97E-02	1.23E-02	rs113118892	15_59274186_C_T	0.91	7150	1.58E-06	GCST90277375
Triacylglycerol (52:6) levels	16	12147559	A	G	-2.56E-01	5.75E-02	2.19E-02	rs146723536	16_12147559_G_A	0.96	7150	8.40E-06	GCST90277375
Triacylglycerol (52:6) levels	16	76342662	T	C	-4.90E-01	1.05E-01	7.15E-03	rs72794954	16_76342662_C_T	0.93	7150	3.02E-06	GCST90277375
Triacylglycerol (52:6) levels	18	67530560	G	A	-3.03E-01	6.78E-02	1.86E-02	rs142118024	18_67530560_A_G	0.84	7150	8.02E-06	GCST90277375
Triacylglycerol (52:6) levels	19	8209156	C	G	2.61E-01	1.69E-02	3.99E-01	rs2336171	19_8209156_G_C	0.99	7150	5.70E-53	GCST90277375
Triacylglycerol (52:6) levels	19	8236164	T	C	1.52E-01	1.91E-02	7.21E-01	rs7248003	19_8236164_C_T	0.94	7150	2.09E-15	GCST90277375
Triacylglycerol (52:6) levels	19	8268177	C	T	-1.09E-01	2.02E-02	2.29E-01	rs250507	19_8268177_T_C	0.97	7150	7.49E-08	GCST90277375
Triacylglycerol (52:6) levels	19	8341496	T	C	-9.46E-02	1.82E-02	3.09E-01	rs10405061	19_8341496_C_T	0.99	7150	2.01E-07	GCST90277375
Triacylglycerol (52:6) levels	19	8607097	C	T	-1.31E-01	1.93E-02	2.61E-01	rs12462619	19_8607097_T_C	0.97	7150	1.44E-11	GCST90277375
Triacylglycerol (52:6) levels	19	8628548	A	C	-8.67E-02	1.81E-02	6.91E-01	rs4804319	19_8628548_C_A	0.97	7150	1.74E-06	GCST90277375
Triacylglycerol (52:6) levels	19	48629610	C	G	2.30E-01	4.81E-02	3.05E-02	rs61751862	19_48629610_G_C	1.00	7150	1.70E-06	GCST90277375
Triacylglycerol (52:6) levels	21	29991071	T	C	5.45E-01	1.22E-01	5.13E-03	rs142551539	21_29991071_C_T	0.95	7150	7.56E-06	GCST90277375
Triacylglycerol (52:6) levels	22	48622225	G	C	3.39E-01	7.35E-02	1.44E-02	rs148683988	22_48622225_C_G	0.92	7150	4.04E-06	GCST90277375
Triacylglycerol (53:2) levels	1	8738706	T	C	3.67E-01	7.19E-02	1.40E-02	rs76174849	1_8738706_C_T	0.97	7174	3.41E-07	GCST90277376
Triacylglycerol (53:2) levels	1	31012510	T	C	-9.86E-02	1.99E-02	7.73E-01	rs4949190	1_31012510_C_T	0.99	7174	7.78E-07	GCST90277376
Triacylglycerol (53:2) levels	1	55039974	T	G	-3.32E-01	4.65E-02	3.32E-02	rs11591147	1_55039974_G_T	1.00	7174	1.05E-12	GCST90277376
Triacylglycerol (53:2) levels	1	155912046	G	C	2.52E-01	5.63E-02	2.35E-02	rs80019356	1_155912046_C_G	0.99	7174	7.92E-06	GCST90277376
Triacylglycerol (53:2) levels	1	236024983	G	C	8.23E-02	1.76E-02	3.27E-01	rs10927285	1_236024983_C_G	0.99	7174	2.86E-06	GCST90277376
Triacylglycerol (53:2) levels	2	79000117	C	A	-1.06E-01	2.16E-02	8.16E-01	rs283819	2_79000117_A_C	0.99	7174	1.01E-06	GCST90277376
Triacylglycerol (53:2) levels	2	138816573	C	T	9.06E-02	1.90E-02	7.38E-01	rs4954960	2_138816573_T_C	0.99	7174	1.90E-06	GCST90277376
Triacylglycerol (53:2) levels	2	157097431	G	C	-1.76E-01	3.95E-02	5.12E-02	rs188346987	2_157097431_C_G	0.91	7174	8.76E-06	GCST90277376
Triacylglycerol (53:2) levels	3	186868495	G	C	-1.04E-01	2.11E-02	8.01E-01	rs9835223	3_186868495_C_G	0.96	7174	9.29E-07	GCST90277376
Triacylglycerol (53:2) levels	4	72375030	A	G	3.34E-01	5.08E-02	2.96E-02	rs80101718	4_72375030_G_A	0.93	7174	4.97E-11	GCST90277376
Triacylglycerol (53:2) levels	4	72803111	A	G	3.20E-01	5.36E-02	2.56E-02	rs77645768	4_72803111_G_A	0.97	7174	2.56E-09	GCST90277376
Triacylglycerol (53:2) levels	4	73947510	C	A	3.77E-01	5.28E-02	2.53E-02	rs182695896	4_73947510_A_C	0.96	7174	1.11E-12	GCST90277376
Triacylglycerol (53:2) levels	4	168898979	T	C	2.25E-01	4.79E-02	3.76E-02	rs77030061	4_168898979_C_T	0.83	7174	2.68E-06	GCST90277376
Triacylglycerol (53:2) levels	5	101638460	G	A	1.41E-01	3.18E-02	7.47E-02	rs66626762	5_101638460_A_G	0.99	7174	9.67E-06	GCST90277376
Triacylglycerol (53:2) levels	6	160682773	A	T	1.75E-01	3.63E-02	5.74E-02	rs374071816	6_160682773_T_A	0.98	7174	1.38E-06	GCST90277376
Triacylglycerol (53:2) levels	8	4075730	T	G	-9.28E-02	2.01E-02	2.21E-01	rs2552134	8_4075730_G_T	0.99	7174	3.88E-06	GCST90277376
Triacylglycerol (53:2) levels	8	93048045	G	A	-1.57E-01	3.45E-02	9.38E-01	rs279967	8_93048045_A_G	0.98	7174	5.45E-06	GCST90277376
Triacylglycerol (53:2) levels	9	104888931	C	T	-1.23E-01	2.52E-02	1.25E-01	rs2275544	9_104888931_T_C	1.00	7174	1.13E-06	GCST90277376
Triacylglycerol (53:2) levels	9	133274295	T	A	9.33E-02	2.08E-02	1.99E-01	rs115478735	9_133274295_A_T	1.00	7174	7.41E-06	GCST90277376
Triacylglycerol (53:2) levels	10	101716501	A	T	-8.06E-02	1.78E-02	6.69E-01	rs7478167	10_101716501_T_A	0.99	7174	6.39E-06	GCST90277376
Triacylglycerol (53:2) levels	10	124492922	G	A	7.54E-02	1.67E-02	5.11E-01	rs4962634	10_124492922_A_G	0.99	7174	6.80E-06	GCST90277376
Triacylglycerol (53:2) levels	11	22327737	A	G	2.11E-01	4.74E-02	3.31E-02	rs138255545	11_22327737_G_A	0.97	7174	8.58E-06	GCST90277376
Triacylglycerol (53:2) levels	11	46848527	T	C	1.62E-01	3.42E-02	6.32E-02	rs113318071	11_46848527_C_T	0.97	7174	2.33E-06	GCST90277376
Triacylglycerol (53:2) levels	11	48806055	A	G	1.56E-01	3.38E-02	7.02E-02	rs8189230	11_48806055_G_A	0.93	7174	3.88E-06	GCST90277376

Triacylglycerol (53:2) levels	12	30557703 T	G	1.47E-01	3.24E-02	6.97E-02 rs11050950	12_30557703_G_T	0.98	7174	6.29E-06 GCST90277376
Triacylglycerol (53:2) levels	12	67513820 A	C	-2.29E-01	4.78E-02	3.50E-02 rs7970512	12_67513820_C_A	0.87	7174	1.65E-06 GCST90277376
Triacylglycerol (53:2) levels	12	101922985 C	T	1.79E-01	3.92E-02	4.82E-02 rs117146578	12_101922985_T_C	1.00	7174	5.15E-06 GCST90277376
Triacylglycerol (53:2) levels	12	112753113 C	T	8.73E-02	1.75E-02	6.65E-01 rs4767003	12_112753113_T_C	1.00	7174	6.52E-07 GCST90277376
Triacylglycerol (53:2) levels	13	96476320 A	G	1.77E-01	3.98E-02	4.50E-02 rs34359547	13_96476320_G_A	0.99	7174	9.05E-06 GCST90277376
Triacylglycerol (53:2) levels	13	103929015 A	T	8.29E-01	1.85E-01	2.23E-03 rs145797759	13_103929015_T_A	0.94	7174	7.39E-06 GCST90277376
Triacylglycerol (53:2) levels	15	59274186 T	C	3.59E-01	7.93E-02	1.23E-02 rs113118892	15_59274186_C_T	0.91	7174	6.26E-06 GCST90277376
Triacylglycerol (53:2) levels	17	3547599 G	T	-3.99E-01	8.40E-02	1.01E-02 rs186039163	17_3547599_T_G	0.97	7174	2.09E-06 GCST90277376
Triacylglycerol (53:2) levels	17	4498679 A	G	-1.37E-01	1.94E-02	2.53E-01 rs76790474	17_4498679_G_A	0.94	7174	1.64E-12 GCST90277376
Triacylglycerol (53:2) levels	17	4764677 A	C	-7.74E-01	5.30E-02	2.58E-02 rs75679663	17_4764677_C_A	0.98	7174	1.26E-47 GCST90277376
Triacylglycerol (53:2) levels	17	4779345 T	C	-5.62E-01	8.74E-02	9.06E-03 rs192913735	17_4779345_C_T	0.98	7174	1.36E-10 GCST90277376
Triacylglycerol (53:2) levels	17	5065669 T	C	-3.25E-01	3.39E-02	6.20E-02 rs117643293	17_5065669_C_T	0.99	7174	1.08E-21 GCST90277376
Triacylglycerol (53:2) levels	17	81050837 A	G	7.88E-02	1.71E-02	3.96E-01 rs8067235	17_81050837_G_A	1.00	7174	4.10E-06 GCST90277376
Triacylglycerol (53:2) levels	19	11079868 T	C	-1.43E-01	2.81E-02	9.46E-02 rs118068660	19_11079868_C_T	1.00	7174	3.46E-07 GCST90277376
Triacylglycerol (53:2) levels	19	44908822 T	C	-2.00E-01	3.71E-02	5.31E-02 rs7412	19_44908822_C_T	1.00	7174	7.36E-08 GCST90277376
Triacylglycerol (53:2) levels	19	44963068 C	T	-7.97E-02	1.67E-02	5.01E-01 rs111997200	19_44963068_T_C	0.99	7174	1.99E-06 GCST90277376
Triacylglycerol (53:2) levels	20	4931633 A	G	2.41E-01	5.36E-02	2.55E-02 rs56671692	20_4931633_G_A	0.97	7174	7.09E-06 GCST90277376
Triacylglycerol (53:2) levels	20	41301845 C	G	8.87E-02	1.68E-02	4.75E-01 rs59596298	20_41301845_G_C	1.00	7174	1.27E-07 GCST90277376
Triacylglycerol (53:2) levels	20	44413724 T	C	-1.78E-01	3.72E-02	5.24E-02 rs1800961	20_44413724_C_T	1.00	7174	1.71E-06 GCST90277376
Triacylglycerol (53:2) levels	21	38913660 T	G	4.03E-01	8.96E-02	1.01E-02 rs140202522	21_38913660_G_T	0.88	7174	6.98E-06 GCST90277376
Triacylglycerol (53:3) levels	1	8809221 C	A	3.43E-01	7.58E-02	1.26E-02 rs79984432	1_8809221_A_C	0.97	7174	5.94E-06 GCST90277377
Triacylglycerol (53:3) levels	1	55039974 T	G	-2.59E-01	4.66E-02	3.32E-02 rs11591147	1_55039974_G_T	1.00	7174	2.73E-08 GCST90277377
Triacylglycerol (53:3) levels	1	164890921 A	C	-8.94E-02	2.02E-02	7.82E-01 rs1237196	1_164890921_C_A	1.00	7174	9.98E-06 GCST90277377
Triacylglycerol (53:3) levels	1	180274389 G	A	7.62E-02	1.69E-02	5.78E-01 rs7536561	1_180274389_A_G	1.00	7174	6.94E-06 GCST90277377
Triacylglycerol (53:3) levels	1	192592415 G	A	8.35E-02	1.79E-02	3.33E-01 rs2760512	1_192592415_A_G	1.00	7174	3.31E-06 GCST90277377
Triacylglycerol (53:3) levels	1	230244207 G	A	-1.89E-01	4.24E-02	4.28E-02 rs16851199	1_230244207_A_G	0.94	7174	8.33E-06 GCST90277377
Triacylglycerol (53:3) levels	2	50201990 A	T	-1.31E-01	2.64E-02	1.11E-01 rs10490233	2_50201990_T_A	1.00	7174	7.65E-07 GCST90277377
Triacylglycerol (53:3) levels	2	128299827 A	G	-3.52E-01	7.73E-02	1.24E-02 rs148316008	2_128299827_G_A	0.97	7174	5.30E-06 GCST90277377
Triacylglycerol (53:3) levels	2	210741300 T	C	9.57E-02	1.90E-02	2.68E-01 rs56298236	2_210741300_C_T	1.00	7174	4.61E-07 GCST90277377
Triacylglycerol (53:3) levels	3	20089961 G	A	4.68E-01	1.02E-01	7.05E-03 rs115880518	3_20089961_A_G	0.97	7174	4.20E-06 GCST90277377
Triacylglycerol (53:3) levels	3	50264152 G	A	-8.12E-02	1.77E-02	3.25E-01 rs9839427	3_50264152_A_G	0.99	7174	4.75E-06 GCST90277377
Triacylglycerol (53:3) levels	3	186868495 G	C	-1.05E-01	2.11E-02	8.01E-01 rs9835223	3_186868495_C_G	0.96	7174	6.33E-07 GCST90277377
Triacylglycerol (53:3) levels	3	194176577 C	A	-9.48E-02	2.02E-02	2.41E-01 rs73073606	3_194176577_A_C	0.94	7174	2.59E-06 GCST90277377
Triacylglycerol (53:3) levels	4	72803111 A	G	3.04E-01	5.37E-02	2.56E-02 rs77645768	4_72803111_G_A	0.97	7174	1.55E-08 GCST90277377
Triacylglycerol (53:3) levels	4	73124451 T	C	2.36E-01	4.49E-02	3.81E-02 rs114483871	4_73124451_C_T	0.93	7174	1.41E-07 GCST90277377
Triacylglycerol (53:3) levels	4	73947510 C	A	3.53E-01	5.29E-02	2.53E-02 rs182695896	4_73947510_A_C	0.96	7174	2.77E-11 GCST90277377
Triacylglycerol (53:3) levels	4	168864851 C	T	2.14E-01	4.65E-02	3.88E-02 rs79402526	4_168864851_T_C	0.85	7174	4.43E-06 GCST90277377
Triacylglycerol (53:3) levels	6	151920771 G	A	3.80E-01	8.16E-02	1.11E-02 rs187144380	6_151920771_A_G	0.94	7174	3.36E-06 GCST90277377
Triacylglycerol (53:3) levels	9	5454500 T	A	2.94E-01	6.37E-02	1.76E-02 rs73641617	9_5454500_A_T	0.98	7174	3.84E-06 GCST90277377
Triacylglycerol (53:3) levels	10	101716501 A	T	-8.16E-02	1.79E-02	6.69E-01 rs7478167	10_101716501_T_A	0.99	7174	5.02E-06 GCST90277377
Triacylglycerol (53:3) levels	11	56943668 A	G	1.00E-01	2.26E-02	1.66E-01 rs11522703	11_56943668_G_A	0.98	7174	8.88E-06 GCST90277377
Triacylglycerol (53:3) levels	11	61815236 C	T	-1.21E-01	1.72E-02	3.81E-01 rs174561	11_61815236_T_C	1.00	7174	2.13E-12 GCST90277377
Triacylglycerol (53:3) levels	11	125920827 C	T	8.17E-02	1.79E-02	6.79E-01 rs646488	11_125920827_T_C	0.99	7174	5.12E-06 GCST90277377
Triacylglycerol (53:3) levels	12	31794260 A	G	7.88E-02	1.76E-02	6.56E-01 rs1797734	12_31794260_G_A	0.99	7174	7.57E-06 GCST90277377
Triacylglycerol (53:3) levels	12	45576938 A	G	1.07E-01	2.42E-02	1.44E-01 rs17604972	12_45576938_G_A	0.96	7174	9.37E-06 GCST90277377
Triacylglycerol (53:3) levels	14	20017746 A	T	-1.65E-01	3.61E-02	5.67E-02 rs17276933	14_20017746_T_A	1.00	7174	4.64E-06 GCST90277377
Triacylglycerol (53:3) levels	14	56720725 T	C	-2.37E-01	5.28E-02	2.78E-02 rs146855398	14_56720725_C_T	0.92	7174	7.06E-06 GCST90277377
Triacylglycerol (53:3) levels	14	63768838 T	G	3.37E-01	2.49E-02	1.25E-01 rs7157785	14_63768838_G_T	1.00	7174	2.10E-41 GCST90277377
Triacylglycerol (53:3) levels	15	31376503 A	G	9.81E-02	2.17E-02	1.83E-01 rs12595641	15_31376503_G_A	0.98	7174	6.00E-06 GCST90277377
Triacylglycerol (53:3) levels	15	59274186 T	C	3.62E-01	7.94E-02	1.23E-02 rs113118892	15_59274186_C_T	0.91	7174	5.09E-06 GCST90277377
Triacylglycerol (53:3) levels	17	4764677 A	C	-2.77E-01	5.37E-02	2.58E-02 rs75679663	17_4764677_C_A	0.98	7174	2.59E-07 GCST90277377
Triacylglycerol (53:3) levels	17	5384095 A	G	-1.88E-01	3.12E-02	7.46E-02 rs3026120	17_5384095_G_A	1.00	7174	1.61E-09 GCST90277377
Triacylglycerol (53:3) levels	19	11078596 C	T	-1.41E-01	2.82E-02	9.43E-02 rs113722226	19_11078596_T_C	1.00	7174	5.58E-07 GCST90277377
Triacylglycerol (53:3) levels	19	19684751 A	C	-7.77E-02	1.74E-02	3.94E-01 rs7247433	19_19684751_C_A	0.96	7174	7.99E-06 GCST90277377
Triacylglycerol (53:3) levels	19	48629610 C	G	2.38E-01	4.80E-02	3.05E-02 rs61751862	19_48629610_G_C	1.00	7174	7.20E-07 GCST90277377
Triacylglycerol (53:3) levels	20	12982070 G	A	-1.22E-01	1.76E-02	6.61E-01 rs364585	20_12982070_A_G	1.00	7174	4.33E-12 GCST90277377
Triacylglycerol (53:4) levels	1	54369167 T	C	-1.67E-01	3.53E-02	6.10E-02 rs17110517	1_54369167_C_T	0.98	7174	2.26E-06 GCST90277378

Triacylglycerol (53:4) levels	1	55039974	T	G	-3.62E-01	4.65E-02	3.32E-02	rs11591147	1_55039974_G_T	1.00	7174	8.29E-15	GCST90277378
Triacylglycerol (53:4) levels	1	107036673	C	A	-8.15E-02	1.83E-02	2.79E-01	rs17018185	1_107036673_A_C	1.00	7174	8.28E-06	GCST90277378
Triacylglycerol (53:4) levels	1	180277126	T	C	9.66E-02	2.11E-02	7.95E-01	rs6680361	1_180277126_C_T	0.99	7174	4.53E-06	GCST90277378
Triacylglycerol (53:4) levels	2	21041028	A	G	9.95E-02	1.84E-02	2.85E-01	rs1367117	2_21041028_G_A	1.00	7174	6.88E-08	GCST90277378
Triacylglycerol (53:4) levels	2	135153852	C	T	9.00E-02	1.77E-02	3.26E-01	rs17261772	2_135153852_T_C	1.00	7174	3.94E-07	GCST90277378
Triacylglycerol (53:4) levels	2	241431336	T	G	7.82E-02	1.69E-02	4.13E-01	rs59916403	2_241431336_G_T	1.00	7174	3.85E-06	GCST90277378
Triacylglycerol (53:4) levels	3	19330366	G	A	-8.17E-02	1.82E-02	3.00E-01	rs2034007	3_19330366_A_G	0.99	7174	7.52E-06	GCST90277378
Triacylglycerol (53:4) levels	3	103515604	C	T	-6.80E-01	1.46E-01	3.81E-03	rs62274367	3_103515604_T_C	0.88	7174	3.03E-06	GCST90277378
Triacylglycerol (53:4) levels	3	153576128	A	G	7.48E-02	1.67E-02	5.34E-01	rs2177333	3_153576128_G_A	1.00	7174	7.76E-06	GCST90277378
Triacylglycerol (53:4) levels	4	16520100	G	T	9.46E-02	1.88E-02	3.12E-01	rs6843498	4_16520100_T_G	0.92	7174	5.00E-07	GCST90277378
Triacylglycerol (53:4) levels	4	72876701	A	G	2.88E-01	4.79E-02	3.16E-02	rs112585713	4_72876701_G_A	0.98	7174	1.86E-09	GCST90277378
Triacylglycerol (53:4) levels	4	73124451	T	C	2.79E-01	4.48E-02	3.81E-02	rs114483871	4_73124451_C_T	0.93	7174	4.78E-10	GCST90277378
Triacylglycerol (53:4) levels	4	73947510	C	A	3.82E-01	5.28E-02	2.53E-02	rs182695896	4_73947510_A_C	0.96	7174	5.36E-13	GCST90277378
Triacylglycerol (53:4) levels	5	90870095	T	C	2.94E-01	6.33E-02	1.81E-02	rs115350073	5_90870095_C_T	0.97	7174	3.56E-06	GCST90277378
Triacylglycerol (53:4) levels	5	108641417	C	A	-1.02E-01	2.19E-02	1.76E-01	rs67718253	5_108641417_A_C	0.99	7174	3.65E-06	GCST90277378
Triacylglycerol (53:4) levels	6	14104359	G	A	-1.11E-01	2.42E-02	1.33E-01	rs12530474	6_14104359_A_G	1.00	7174	4.56E-06	GCST90277378
Triacylglycerol (53:4) levels	7	73612048	T	C	1.32E-01	2.39E-02	1.41E-01	rs17145750	7_73612048_C_T	1.00	7174	3.07E-08	GCST90277378
Triacylglycerol (53:4) levels	7	151941718	C	G	2.57E-01	5.81E-02	9.78E-01	rs10252648	7_151941718_G_C	0.99	7174	9.95E-06	GCST90277378
Triacylglycerol (53:4) levels	8	46957856	G	A	2.36E-01	5.33E-02	2.65E-02	rs75444922	8_46957856_A_G	0.94	7174	9.45E-06	GCST90277378
Triacylglycerol (53:4) levels	9	104906960	C	T	-1.56E-01	3.37E-02	6.62E-02	rs4100654	9_104906960_T_C	0.97	7174	3.76E-06	GCST90277378
Triacylglycerol (53:4) levels	10	45824374	G	C	5.64E-01	1.23E-01	5.25E-03	rs184845520	10_45824374_C_G	0.87	7174	4.49E-06	GCST90277378
Triacylglycerol (53:4) levels	10	128742890	C	A	3.44E-01	7.59E-02	1.39E-02	rs139474488	10_128742890_A_C	0.87	7174	6.01E-06	GCST90277378
Triacylglycerol (53:4) levels	11	101739572	C	G	2.27E-01	4.48E-02	3.70E-02	rs116893213	11_101739572_G_C	0.98	7174	4.17E-07	GCST90277378
Triacylglycerol (53:4) levels	11	103250744	C	A	-1.09E-01	2.09E-02	2.00E-01	rs3912622	11_103250744_A_C	1.00	7174	2.00E-07	GCST90277378
Triacylglycerol (53:4) levels	11	116658307	T	C	9.28E-02	2.06E-02	2.05E-01	rs7104343	11_116658307_C_T	1.00	7174	6.92E-06	GCST90277378
Triacylglycerol (53:4) levels	12	112765162	T	C	7.93E-02	1.66E-02	4.22E-01	rs11610836	12_112765162_C_T	1.00	7174	1.75E-06	GCST90277378
Triacylglycerol (53:4) levels	14	20017746	A	T	-1.71E-01	3.61E-02	5.67E-02	rs17276933	14_20017746_T_A	1.00	7174	2.11E-06	GCST90277378
Triacylglycerol (53:4) levels	15	38953699	A	G	-1.04E-01	2.30E-02	8.40E-01	rs4283208	15_38953699_G_A	0.98	7174	6.55E-06	GCST90277378
Triacylglycerol (53:4) levels	15	58424058	G	A	-9.17E-02	1.89E-02	6.88E-01	rs515081	15_58424058_A_G	0.89	7174	1.31E-06	GCST90277378
Triacylglycerol (53:4) levels	17	3531540	A	G	-3.34E-01	7.46E-02	1.33E-02	rs116919723	17_3531540_G_A	0.94	7174	7.56E-06	GCST90277378
Triacylglycerol (53:4) levels	17	4764677	A	C	-5.38E-01	5.34E-02	2.58E-02	rs75679663	17_4764677_C_A	0.98	7174	1.09E-23	GCST90277378
Triacylglycerol (53:4) levels	17	5464230	C	T	-2.19E-01	3.45E-02	5.96E-02	rs146331166	17_5464230_T_C	0.99	7174	2.56E-10	GCST90277378
Triacylglycerol (53:4) levels	17	80964698	A	C	7.73E-02	1.70E-02	4.34E-01	rs3751934	17_80964698_C_A	0.98	7174	5.32E-06	GCST90277378
Triacylglycerol (53:4) levels	19	11079868	T	C	-2.19E-01	2.81E-02	9.46E-02	rs118068660	19_11079868_C_T	1.00	7174	6.77E-15	GCST90277378
Triacylglycerol (53:4) levels	19	19684751	A	C	-7.91E-02	1.74E-02	3.94E-01	rs7247433	19_19684751_C_A	0.96	7174	5.43E-06	GCST90277378
Triacylglycerol (53:4) levels	19	44908822	T	C	-2.11E-01	3.72E-02	5.31E-02	rs7412	19_44908822_C_T	1.00	7174	1.45E-08	GCST90277378
Triacylglycerol (53:4) levels	19	44954120	A	G	-8.03E-02	1.68E-02	4.98E-01	rs73047641	19_44954120_G_A	0.99	7174	1.73E-06	GCST90277378
Triacylglycerol (54:3) levels	1	184416563	G	C	4.06E-01	9.03E-02	1.10E-02	rs78056622	1_184416563_C_G	0.91	6070	6.91E-06	GCST90277379
Triacylglycerol (54:3) levels	2	27508073	C	T	-1.01E-01	1.89E-02	6.51E-01	rs1260326	2_27508073_T_C	1.00	6070	8.99E-08	GCST90277379
Triacylglycerol (54:3) levels	2	136406475	C	T	7.88E-01	1.78E-01	9.97E-01	rs478562	2_136406475_T_C	0.86	6070	9.57E-06	GCST90277379
Triacylglycerol (54:3) levels	2	215338573	G	A	1.00E-01	2.19E-02	2.23E-01	rs16853823	2_215338573_A_G	1.00	6070	4.54E-06	GCST90277379
Triacylglycerol (54:3) levels	3	72260449	T	G	-9.73E-02	2.15E-02	2.36E-01	rs9837572	3_72260449_G_T	0.99	6070	6.10E-06	GCST90277379
Triacylglycerol (54:3) levels	4	135989116	G	A	8.88E-02	2.00E-02	3.00E-01	rs4318703	4_135989116_A_G	1.00	6070	8.71E-06	GCST90277379
Triacylglycerol (54:3) levels	8	40367808	A	G	-4.02E-01	8.49E-02	1.31E-02	rs117591451	8_40367808_G_A	0.92	6070	2.27E-06	GCST90277379
Triacylglycerol (54:3) levels	8	58158358	T	A	1.86E-01	4.05E-02	5.21E-02	rs16923137	8_58158358_A_T	0.98	6070	4.37E-06	GCST90277379
Triacylglycerol (54:3) levels	9	2770506	C	T	2.63E-01	5.41E-02	2.99E-02	rs117730598	9_2770506_T_C	0.96	6070	1.16E-06	GCST90277379
Triacylglycerol (54:3) levels	9	132470502	G	A	2.79E-01	5.93E-02	2.59E-02	rs111568723	9_132470502_A_G	0.95	6070	2.63E-06	GCST90277379
Triacylglycerol (54:3) levels	11	110379615	T	C	-1.17E-01	2.42E-02	1.69E-01	rs12225049	11_110379615_C_T	0.99	6070	1.44E-06	GCST90277379
Triacylglycerol (54:3) levels	12	81672829	T	C	1.13E-01	2.33E-02	1.89E-01	rs12301917	12_81672829_C_T	0.99	6070	1.16E-06	GCST90277379
Triacylglycerol (54:3) levels	14	29818391	G	A	-2.16E-01	4.85E-02	3.72E-02	rs73259585	14_29818391_A_G	0.98	6070	8.36E-06	GCST90277379
Triacylglycerol (54:3) levels	14	92187131	G	A	-1.33E-01	2.90E-02	1.16E-01	rs11160050	14_92187131_A_G	0.94	6070	4.42E-06	GCST90277379
Triacylglycerol (54:3) levels	16	30484331	A	G	-1.31E-01	2.83E-02	1.25E-01	rs9796793	16_30484331_G_A	0.95	6070	3.81E-06	GCST90277379
Triacylglycerol (54:3) levels	16	55542249	C	A	1.07E-01	2.34E-02	1.95E-01	rs10521320	16_55542249_A_C	0.95	6070	5.44E-06	GCST90277379
Triacylglycerol (54:3) levels	17	53571810	G	A	-9.12E-02	1.87E-02	6.14E-01	rs1553368	17_53571810_A_G	1.00	6070	1.17E-06	GCST90277379
Triacylglycerol (54:3) levels	17	77816410	T	C	2.73E-01	5.76E-02	2.64E-02	rs35840156	17_77816410_C_T	0.94	6070	2.14E-06	GCST90277379
Triacylglycerol (54:3) levels	18	30887893	G	C	-2.02E-01	4.22E-02	5.09E-02	rs10502551	18_30887893_C_G	0.96	6070	1.83E-06	GCST90277379
Triacylglycerol (54:3) levels	19	55802466	C	T	-1.95E-01	3.90E-02	6.03E-02	rs79152531	19_55802466_T_C	0.97	6070	5.89E-07	GCST90277379

Triacylglycerol (54:3) levels	20	13847007	T	C	-8.19E-02	1.79E-02	4.65E-01	rs998182	20_13847007_C_T	1.00	6070	5.07E-06	GCST90277379
Triacylglycerol (54:3) levels	21	40708914	C	G	9.25E-02	2.03E-02	2.77E-01	rs2205135	21_40708914_G_C	0.99	6070	5.49E-06	GCST90277379
Triacylglycerol (54:4) levels	1	184416563	G	C	4.72E-01	9.60E-02	1.10E-02	rs78056622	1_184416563_C_G	0.91	5448	8.72E-07	GCST90277380
Triacylglycerol (54:4) levels	2	27508073	C	T	-1.14E-01	2.00E-02	6.51E-01	rs1260326	2_27508073_T_C	1.00	5448	1.35E-08	GCST90277380
Triacylglycerol (54:4) levels	2	238572357	G	C	-8.74E-02	1.95E-02	4.52E-01	rs7560313	2_238572357_C_G	0.99	5448	7.53E-06	GCST90277380
Triacylglycerol (54:4) levels	4	62386287	C	G	-1.43E-01	2.96E-02	1.20E-01	rs778957	4_62386287_G_C	0.99	5448	1.30E-06	GCST90277380
Triacylglycerol (54:4) levels	6	30972348	T	C	-1.42E-01	3.17E-02	1.03E-01	rs17189819	6_30972348_C_T	1.00	5448	7.30E-06	GCST90277380
Triacylglycerol (54:4) levels	6	33985737	A	G	2.98E-01	6.60E-02	2.35E-02	rs141935215	6_33985737_G_A	0.92	5448	6.61E-06	GCST90277380
Triacylglycerol (54:4) levels	8	40367808	A	G	-4.08E-01	9.07E-02	1.31E-02	rs117591451	8_40367808_G_A	0.92	5448	7.07E-06	GCST90277380
Triacylglycerol (54:4) levels	8	56711024	T	C	-1.15E-01	2.46E-02	8.17E-01	rs6474079	8_56711024_C_T	0.98	5448	2.88E-06	GCST90277380
Triacylglycerol (54:4) levels	8	58330047	G	A	2.25E-01	4.41E-02	4.81E-02	rs191616475	8_58330047_A_G	1.00	5448	3.64E-07	GCST90277380
Triacylglycerol (54:4) levels	9	2283431	C	T	-2.28E-01	5.06E-02	3.89E-02	rs140796055	9_2283431_T_C	0.98	5448	6.76E-06	GCST90277380
Triacylglycerol (54:4) levels	9	99655889	T	A	-4.77E-01	1.06E-01	8.74E-03	rs117529982	9_99655889_A_T	0.94	5448	6.46E-06	GCST90277380
Triacylglycerol (54:4) levels	9	112114209	A	G	8.30E-01	1.83E-01	3.23E-03	rs116923150	9_112114209_G_A	0.90	5448	5.75E-06	GCST90277380
Triacylglycerol (54:4) levels	11	26783827	A	G	9.13E-02	1.91E-02	4.61E-01	rs2084594	11_26783827_G_A	1.00	5448	1.79E-06	GCST90277380
Triacylglycerol (54:4) levels	11	110379615	T	C	-1.18E-01	2.57E-02	1.69E-01	rs12225049	11_110379615_C_T	0.99	5448	4.24E-06	GCST90277380
Triacylglycerol (54:4) levels	12	8608014	G	A	-1.18E-01	2.65E-02	1.63E-01	rs11613792	12_8608014_A_G	0.93	5448	8.83E-06	GCST90277380
Triacylglycerol (54:4) levels	12	81672829	T	C	1.10E-01	2.46E-02	1.89E-01	rs12301917	12_81672829_C_T	0.99	5448	7.62E-06	GCST90277380
Triacylglycerol (54:4) levels	13	33335601	T	C	1.05E-01	2.31E-02	2.20E-01	rs7323268	13_33335601_C_T	1.00	5448	5.24E-06	GCST90277380
Triacylglycerol (54:4) levels	13	76166574	C	T	1.30E-01	2.79E-02	1.36E-01	rs73226143	13_76166574_T_C	1.00	5448	3.52E-06	GCST90277380
Triacylglycerol (54:4) levels	16	30484331	A	G	-1.50E-01	2.98E-02	1.25E-01	rs9796793	16_30484331_G_A	0.95	5448	4.57E-07	GCST90277380
Triacylglycerol (54:4) levels	17	6764031	T	C	1.48E-01	3.25E-02	1.03E-01	rs6502975	17_6764031_C_T	0.98	5448	5.20E-06	GCST90277380
Triacylglycerol (54:4) levels	17	53554728	A	T	-9.45E-02	1.96E-02	4.12E-01	rs6504835	17_53554728_T_A	1.00	5448	1.53E-06	GCST90277380
Triacylglycerol (54:4) levels	18	30887893	G	C	-2.29E-01	4.49E-02	5.09E-02	rs10502551	18_30887893_C_G	0.96	5448	3.43E-07	GCST90277380
Triacylglycerol (54:4) levels	19	15080523	T	C	9.27E-02	2.01E-02	6.03E-01	rs2018873	19_15080523_C_T	0.94	5448	4.28E-06	GCST90277380
Triacylglycerol (54:4) levels	19	32508986	T	C	1.54E-01	3.44E-02	8.60E-02	rs35305910	19_32508986_C_T	0.95	5448	7.60E-06	GCST90277380
Triacylglycerol (54:4) levels	19	55802466	C	T	-1.91E-01	4.16E-02	6.03E-02	rs79152531	19_55802466_T_C	0.97	5448	4.47E-06	GCST90277380
Triacylglycerol (54:5) levels	1	6029418	T	G	-3.52E-01	7.59E-02	1.74E-02	rs10218823	1_6029418_G_T	0.93	5463	3.63E-06	GCST90277381
Triacylglycerol (54:5) levels	2	210779037	G	A	1.33E-01	3.00E-02	1.20E-01	rs969816	2_210779037_A_G	1.00	5463	9.09E-06	GCST90277381
Triacylglycerol (54:5) levels	3	179092711	C	T	-1.07E-01	2.32E-02	7.85E-01	rs4955801	3_179092711_T_C	1.00	5463	4.09E-06	GCST90277381
Triacylglycerol (54:5) levels	4	34767471	G	A	4.24E-01	9.49E-02	1.06E-02	rs140828820	4_34767471_A_G	0.93	5463	7.93E-06	GCST90277381
Triacylglycerol (54:5) levels	4	186453244	A	G	-1.41E-01	2.97E-02	1.18E-01	rs62348866	4_186453244_G_A	0.99	5463	2.01E-06	GCST90277381
Triacylglycerol (54:5) levels	5	149703644	C	T	-1.04E-01	2.33E-02	2.18E-01	rs7721676	5_149703644_T_C	1.00	5463	8.45E-06	GCST90277381
Triacylglycerol (54:5) levels	7	31677697	G	T	1.63E-01	3.57E-02	8.00E-02	rs79159286	7_31677697_T_G	0.97	5463	5.20E-06	GCST90277381
Triacylglycerol (54:5) levels	8	11538263	A	T	-4.75E-01	1.07E-01	9.26E-03	rs117945317	8_11538263_T_A	0.86	5463	9.91E-06	GCST90277381
Triacylglycerol (54:5) levels	9	2640492	G	A	-1.03E-01	2.22E-02	7.52E-01	rs2242104	9_2640492_A_G	0.99	5463	4.16E-06	GCST90277381
Triacylglycerol (54:5) levels	9	135992678	G	C	-3.31E-01	7.24E-02	1.90E-02	rs117264209	9_135992678_C_G	0.96	5463	4.84E-06	GCST90277381
Triacylglycerol (54:5) levels	10	48593367	C	T	5.18E-01	1.16E-01	7.44E-03	rs73302224	10_48593367_T_C	0.98	5463	7.88E-06	GCST90277381
Triacylglycerol (54:5) levels	12	81637055	C	A	1.14E-01	2.55E-02	1.72E-01	rs60243822	12_81637055_A_C	0.99	5463	7.93E-06	GCST90277381
Triacylglycerol (54:5) levels	12	101725095	T	G	-3.23E-01	6.97E-02	9.79E-01	rs222507	12_101725095_G_T	0.93	5463	3.68E-06	GCST90277381
Triacylglycerol (54:5) levels	14	36678136	C	G	-8.91E-02	1.96E-02	6.15E-01	rs10141087	14_36678136_G_C	1.00	5463	5.80E-06	GCST90277381
Triacylglycerol (54:5) levels	17	3420961	T	C	9.10E-02	1.91E-02	5.09E-01	rs769432	17_3420961_C_T	1.00	5463	2.04E-06	GCST90277381
Triacylglycerol (54:5) levels	17	53554728	A	T	-8.69E-02	1.96E-02	4.12E-01	rs6504835	17_53554728_T_A	1.00	5463	9.96E-06	GCST90277381
Triacylglycerol (54:5) levels	18	79524930	G	C	8.85E-02	1.98E-02	5.22E-01	rs3894049	18_79524930_C_G	0.92	5463	8.06E-06	GCST90277381
Triacylglycerol (54:5) levels	19	9009145	A	T	3.05E-01	6.64E-02	2.27E-02	rs10425025	19_9009145_T_A	0.91	5463	4.55E-06	GCST90277381
Triacylglycerol (54:5) levels	19	49524378	T	C	2.96E-01	6.31E-02	2.57E-02	rs76375876	19_49524378_C_T	0.91	5463	2.78E-06	GCST90277381
Triacylglycerol (54:5) levels	20	15445391	T	C	9.59E-02	1.95E-02	3.89E-01	rs2180528	20_15445391_C_T	1.00	5463	9.31E-07	GCST90277381
Triacylglycerol (54:5) levels	22	37593713	A	G	2.41E-01	5.12E-02	3.72E-02	rs62235973	22_37593713_G_A	0.97	5463	2.54E-06	GCST90277381
Triacylglycerol (54:6) levels	1	77362636	G	A	7.95E-02	1.76E-02	6.24E-01	rs12565526	1_77362636_A_G	1.00	7019	6.17E-06	GCST90277382
Triacylglycerol (54:6) levels	1	102847102	G	T	1.26E-01	2.83E-02	1.04E-01	rs79416109	1_102847102_T_G	0.95	7019	8.66E-06	GCST90277382
Triacylglycerol (54:6) levels	1	227429818	T	C	-9.68E-02	2.16E-02	1.90E-01	rs6664147	1_227429818_C_T	1.00	7019	7.94E-06	GCST90277382
Triacylglycerol (54:6) levels	2	27508073	C	T	-1.19E-01	1.76E-02	6.51E-01	rs1260326	2_27508073_T_C	1.00	7019	1.89E-11	GCST90277382
Triacylglycerol (54:6) levels	2	62122935	G	A	-2.20E-01	4.94E-02	3.00E-02	rs67093838	2_62122935_A_G	0.97	7019	8.76E-06	GCST90277382
Triacylglycerol (54:6) levels	2	163555501	A	G	1.66E-01	3.66E-02	5.85E-02	rs17192812	2_163555501_G_A	0.95	7019	6.03E-06	GCST90277382
Triacylglycerol (54:6) levels	2	170225142	C	G	1.58E-01	3.54E-02	9.37E-01	rs6721083	2_170225142_G_C	0.95	7019	8.31E-06	GCST90277382
Triacylglycerol (54:6) levels	2	226227689	A	T	7.84E-02	1.76E-02	6.31E-01	rs1515104	2_226227689_T_A	1.00	7019	8.22E-06	GCST90277382
Triacylglycerol (54:6) levels	3	104035543	G	T	-8.03E-02	1.80E-02	3.37E-01	rs6804331	3_104035543_T_G	0.99	7019	8.25E-06	GCST90277382

Triacylglycerol (54:6) levels	3	171488096	T	C	-4.40E-01	9.69E-02	7.55E-03	rs147463852	3_171488096_C_T	1.00	7019	5.62E-06	GCST90277382
Triacylglycerol (54:6) levels	5	149703644	C	T	-9.08E-02	2.04E-02	2.18E-01	rs7721676	5_149703644_T_C	1.00	7019	8.92E-06	GCST90277382
Triacylglycerol (54:6) levels	8	2825696	C	T	-4.14E-01	8.99E-02	9.63E-03	rs75178403	8_2825696_T_C	0.88	7019	4.19E-06	GCST90277382
Triacylglycerol (54:6) levels	8	58158358	T	A	1.72E-01	3.78E-02	5.21E-02	rs16923137	8_58158358_A_T	0.98	7019	5.08E-06	GCST90277382
Triacylglycerol (54:6) levels	9	2770506	C	T	2.57E-01	5.09E-02	2.99E-02	rs117730598	9_2770506_T_C	0.96	7019	4.40E-07	GCST90277382
Triacylglycerol (54:6) levels	9	120350676	T	C	2.07E-01	4.53E-02	3.92E-02	rs72753379	9_120350676_C_T	0.95	7019	4.90E-06	GCST90277382
Triacylglycerol (54:6) levels	11	116778201	C	G	-1.29E-01	2.34E-02	8.49E-01	rs964184	11_116778201_G_C	1.00	7019	3.48E-08	GCST90277382
Triacylglycerol (54:6) levels	12	52243392	T	C	1.73E-01	3.88E-02	5.26E-02	rs56082136	12_52243392_C_T	0.93	7019	8.47E-06	GCST90277382
Triacylglycerol (54:6) levels	12	105758014	A	G	-9.52E-02	1.94E-02	2.42E-01	rs10861498	12_105758014_G_A	0.99	7019	9.10E-07	GCST90277382
Triacylglycerol (54:6) levels	12	131622906	C	G	1.41E-01	3.17E-02	8.43E-02	rs117387197	12_131622906_G_C	0.93	7019	9.39E-06	GCST90277382
Triacylglycerol (54:6) levels	13	60832603	A	G	8.83E-02	1.86E-02	7.18E-01	rs3127050	13_60832603_G_A	1.00	7019	2.18E-06	GCST90277382
Triacylglycerol (54:6) levels	14	36679967	A	C	-8.49E-02	1.75E-02	6.23E-01	rs1884216	14_36679967_C_A	1.00	7019	1.18E-06	GCST90277382
Triacylglycerol (54:6) levels	15	39999889	C	A	-2.02E-01	4.26E-02	3.94E-02	rs16970164	15_39999889_A_C	0.99	7019	2.04E-06	GCST90277382
Triacylglycerol (54:6) levels	19	55802466	C	T	-1.88E-01	3.60E-02	6.03E-02	rs79152531	19_55802466_T_C	0.97	7019	1.80E-07	GCST90277382
Triacylglycerol (54:6) levels	20	13891447	T	C	-7.65E-02	1.73E-02	6.39E-01	rs6079205	20_13891447_C_T	1.00	7019	9.42E-06	GCST90277382
Triacylglycerol (54:7) levels	1	227429818	T	C	-9.59E-02	2.16E-02	1.90E-01	rs6664147	1_227429818_C_T	1.00	7071	8.72E-06	GCST90277383
Triacylglycerol (54:7) levels	2	27508073	C	T	-1.38E-01	1.75E-02	6.51E-01	rs1260326	2_27508073_T_C	1.00	7071	4.46E-15	GCST90277383
Triacylglycerol (54:7) levels	2	163558771	T	G	1.72E-01	3.64E-02	5.91E-02	rs10084264	2_163558771_G_T	0.95	7071	2.26E-06	GCST90277383
Triacylglycerol (54:7) levels	3	104035543	G	T	-8.13E-02	1.79E-02	3.37E-01	rs6804331	3_104035543_T_G	0.99	7071	5.87E-06	GCST90277383
Triacylglycerol (54:7) levels	6	23919006	G	A	-6.10E-01	1.37E-01	4.04E-03	rs79934453	6_23919006_A_G	0.91	7071	8.65E-06	GCST90277383
Triacylglycerol (54:7) levels	7	30277547	T	A	8.29E-02	1.85E-02	6.63E-01	rs173848	7_30277547_A_T	0.93	7071	7.34E-06	GCST90277383
Triacylglycerol (54:7) levels	8	40367808	A	G	-3.60E-01	7.76E-02	1.31E-02	rs117591451	8_40367808_G_A	0.92	7071	3.43E-06	GCST90277383
Triacylglycerol (54:7) levels	8	58330047	G	A	1.89E-01	3.88E-02	4.81E-02	rs191616475	8_58330047_A_G	1.00	7071	1.15E-06	GCST90277383
Triacylglycerol (54:7) levels	8	85466785	A	G	2.82E-01	6.30E-02	2.02E-02	rs138672261	8_85466785_G_A	0.93	7071	7.50E-06	GCST90277383
Triacylglycerol (54:7) levels	9	2770506	C	T	2.36E-01	5.05E-02	2.99E-02	rs117730598	9_2770506_T_C	0.96	7071	2.97E-06	GCST90277383
Triacylglycerol (54:7) levels	9	33656919	C	A	8.32E-02	1.88E-02	7.25E-01	rs1832326	9_33656919_A_C	1.00	7071	9.60E-06	GCST90277383
Triacylglycerol (54:7) levels	9	80090365	A	G	1.02E-01	2.21E-02	1.79E-01	rs72738698	9_80090365_G_A	0.99	7071	4.16E-06	GCST90277383
Triacylglycerol (54:7) levels	9	120350676	T	C	2.08E-01	4.51E-02	3.92E-02	rs72753379	9_120350676_C_T	0.95	7071	3.94E-06	GCST90277383
Triacylglycerol (54:7) levels	11	116778201	C	G	-1.43E-01	2.33E-02	8.49E-01	rs964184	11_116778201_G_C	1.00	7071	9.60E-10	GCST90277383
Triacylglycerol (54:7) levels	12	52243392	T	C	1.74E-01	3.86E-02	5.26E-02	rs56082136	12_52243392_C_T	0.93	7071	6.20E-06	GCST90277383
Triacylglycerol (54:7) levels	12	105758014	A	G	-8.80E-02	1.93E-02	2.42E-01	rs10861498	12_105758014_G_A	0.99	7071	5.21E-06	GCST90277383
Triacylglycerol (54:7) levels	13	60832603	A	G	8.85E-02	1.86E-02	7.18E-01	rs3127050	13_60832603_G_A	1.00	7071	1.87E-06	GCST90277383
Triacylglycerol (54:7) levels	18	23684659	C	G	2.38E-01	4.98E-02	3.07E-02	rs113777805	18_23684659_G_C	0.97	7071	1.78E-06	GCST90277383
Triacylglycerol (54:7) levels	18	30887893	G	C	-1.85E-01	3.89E-02	5.09E-02	rs10502551	18_30887893_C_G	0.96	7071	2.01E-06	GCST90277383
Triacylglycerol (54:7) levels	19	34971287	G	A	-8.02E-02	1.67E-02	5.33E-01	rs2546043	19_34971287_A_G	0.99	7071	1.66E-06	GCST90277383
Triacylglycerol (54:7) levels	19	43181186	T	C	3.00E-01	6.76E-02	1.76E-02	rs117882070	19_43181186_C_T	0.94	7071	9.40E-06	GCST90277383
Triacylglycerol (54:7) levels	19	55802466	C	T	-1.94E-01	3.60E-02	6.03E-02	rs79152531	19_55802466_T_C	0.97	7071	6.80E-08	GCST90277383
Triacylglycerol (54:7) levels	20	56580207	T	C	-1.80E-01	4.04E-02	4.64E-02	rs13042732	20_56580207_C_T	0.96	7071	8.96E-06	GCST90277383
Triacylglycerol (54:7) levels	22	23971765	G	A	1.71E-01	3.58E-02	6.00E-02	rs79213832	22_23971765_A_G	0.98	7071	1.68E-06	GCST90277383
Triacylglycerol (56:3) levels	2	21002409	T	C	8.98E-02	1.90E-02	7.28E-01	rs1042034	2_21002409_C_T	1.00	6855	2.44E-06	GCST90277384
Triacylglycerol (56:3) levels	2	27508073	C	T	-1.42E-01	1.78E-02	6.51E-01	rs1260326	2_27508073_T_C	1.00	6855	1.98E-15	GCST90277384
Triacylglycerol (56:3) levels	2	36390910	G	A	-1.11E-01	2.47E-02	1.35E-01	rs111430300	2_36390910_A_G	1.00	6855	6.78E-06	GCST90277384
Triacylglycerol (56:3) levels	3	72260449	T	G	-9.25E-02	2.02E-02	2.36E-01	rs9837572	3_72260449_G_T	0.99	6855	4.82E-06	GCST90277384
Triacylglycerol (56:3) levels	4	44884393	T	C	8.25E-02	1.82E-02	3.35E-01	rs62410903	4_44884393_C_T	1.00	6855	5.78E-06	GCST90277384
Triacylglycerol (56:3) levels	8	40367808	A	G	-3.87E-01	7.78E-02	1.31E-02	rs117591451	8_40367808_G_A	0.92	6855	6.57E-07	GCST90277384
Triacylglycerol (56:3) levels	8	58330047	G	A	2.05E-01	3.95E-02	4.81E-02	rs191616475	8_58330047_A_G	1.00	6855	2.08E-07	GCST90277384
Triacylglycerol (56:3) levels	9	2770506	C	T	2.38E-01	5.11E-02	2.99E-02	rs117730598	9_2770506_T_C	0.96	6855	3.37E-06	GCST90277384
Triacylglycerol (56:3) levels	9	80090365	A	G	1.09E-01	2.24E-02	1.79E-01	rs72738698	9_80090365_G_A	0.99	6855	1.25E-06	GCST90277384
Triacylglycerol (56:3) levels	11	7455964	G	A	1.40E-01	3.05E-02	8.65E-02	rs72846885	11_7455964_A_G	0.98	6855	4.92E-06	GCST90277384
Triacylglycerol (56:3) levels	11	38161116	G	A	1.46E-01	3.22E-02	9.17E-01	rs820903	11_38161116_A_G	0.95	6855	5.39E-06	GCST90277384
Triacylglycerol (56:3) levels	11	116778201	C	G	-1.63E-01	2.37E-02	8.49E-01	rs964184	11_116778201_G_C	1.00	6855	6.67E-12	GCST90277384
Triacylglycerol (56:3) levels	12	52243392	T	C	1.89E-01	3.93E-02	5.26E-02	rs56082136	12_52243392_C_T	0.93	6855	1.49E-06	GCST90277384
Triacylglycerol (56:3) levels	12	105758014	A	G	-9.14E-02	1.96E-02	2.42E-01	rs10861498	12_105758014_G_A	0.99	6855	3.26E-06	GCST90277384
Triacylglycerol (56:3) levels	13	21074873	T	C	-1.41E-01	3.16E-02	8.08E-02	rs77745982	13_21074873_C_T	1.00	6855	8.49E-06	GCST90277384
Triacylglycerol (56:3) levels	13	25886443	T	C	-5.61E-01	1.24E-01	5.33E-03	rs112578388	13_25886443_C_T	0.86	6855	6.36E-06	GCST90277384
Triacylglycerol (56:3) levels	14	93951456	A	C	7.60E-02	1.72E-02	5.78E-01	rs4353433	14_93951456_C_A	1.00	6855	9.85E-06	GCST90277384
Triacylglycerol (56:3) levels	15	57470542	A	G	-1.38E-01	3.12E-02	8.62E-02	rs2280065	15_57470542_G_A	0.93	6855	9.58E-06	GCST90277384

Triacylglycerol (56:3) levels	16	30484331	A	G	-1.26E-01	2.63E-02	1.25E-01	rs9796793	16_30484331_G_A	0.95	6855	1.80E-06	GCST90277384
Triacylglycerol (56:3) levels	16	84544387	T	C	-1.09E-01	2.46E-02	1.44E-01	rs8047793	16_84544387_C_T	1.00	6855	9.34E-06	GCST90277384
Triacylglycerol (56:3) levels	18	23684659	C	G	2.49E-01	5.05E-02	3.07E-02	rs113777805	18_23684659_G_C	0.97	6855	8.55E-07	GCST90277384
Triacylglycerol (56:3) levels	18	30887893	G	C	-1.99E-01	3.96E-02	5.09E-02	rs10502551	18_30887893_C_G	0.96	6855	4.90E-07	GCST90277384
Triacylglycerol (56:3) levels	19	19269704	G	A	-1.88E-01	3.89E-02	5.35E-02	rs187429064	19_19269704_A_G	0.95	6855	1.44E-06	GCST90277384
Triacylglycerol (56:3) levels	19	34971287	G	A	-8.24E-02	1.70E-02	5.33E-01	rs2546043	19_34971287_A_G	0.99	6855	1.24E-06	GCST90277384
Triacylglycerol (56:3) levels	19	55802466	C	T	-1.74E-01	3.67E-02	6.03E-02	rs79152531	19_55802466_T_C	0.97	6855	2.28E-06	GCST90277384
Triacylglycerol (56:3) levels	20	56580207	T	C	-1.83E-01	4.12E-02	4.64E-02	rs13042732	20_56580207_C_T	0.96	6855	9.32E-06	GCST90277384
Triacylglycerol (56:3) levels	22	23971765	G	A	1.60E-01	3.62E-02	6.00E-02	rs79213832	22_23971765_A_G	0.98	6855	9.53E-06	GCST90277384
Triacylglycerol (56:4) levels	1	6096167	T	C	-4.97E-01	1.10E-01	7.03E-03	rs80278227	1_6096167_C_T	0.96	5843	6.05E-06	GCST90277385
Triacylglycerol (56:4) levels	1	230712956	T	C	-1.05E-01	2.35E-02	1.96E-01	rs2004776	1_230712956_C_T	1.00	5843	8.53E-06	GCST90277385
Triacylglycerol (56:4) levels	1	241221572	T	C	8.50E-02	1.91E-02	3.65E-01	rs6429254	1_241221572_C_T	1.00	5843	8.53E-06	GCST90277385
Triacylglycerol (56:4) levels	2	12497377	C	T	1.50E-01	3.37E-02	8.77E-02	rs34379959	2_12497377_T_C	0.96	5843	9.49E-06	GCST90277385
Triacylglycerol (56:4) levels	2	27508073	C	T	-9.44E-02	1.93E-02	6.51E-01	rs1260326	2_27508073_T_C	1.00	5843	1.02E-06	GCST90277385
Triacylglycerol (56:4) levels	2	241698370	A	G	2.31E-01	5.06E-02	3.58E-02	rs149371861	2_241698370_G_A	0.98	5843	5.13E-06	GCST90277385
Triacylglycerol (56:4) levels	4	35018175	G	T	-1.30E-01	2.88E-02	1.18E-01	rs73216924	4_35018175_T_G	1.00	5843	6.80E-06	GCST90277385
Triacylglycerol (56:4) levels	4	182736762	T	G	1.90E-01	4.29E-02	5.15E-02	rs71620944	4_182736762_G_T	0.96	5843	9.58E-06	GCST90277385
Triacylglycerol (56:4) levels	5	108626779	G	C	-3.13E-01	6.93E-02	1.81E-02	rs79725259	5_108626779_C_G	0.97	5843	6.43E-06	GCST90277385
Triacylglycerol (56:4) levels	5	149735907	T	C	1.83E-01	4.04E-02	5.59E-02	rs112902435	5_149735907_C_T	0.98	5843	5.99E-06	GCST90277385
Triacylglycerol (56:4) levels	6	28292472	A	T	-1.02E-01	2.21E-02	2.21E-01	rs1233660	6_28292472_T_A	1.00	5843	4.45E-06	GCST90277385
Triacylglycerol (56:4) levels	7	73615107	T	C	-1.30E-01	2.84E-02	1.22E-01	rs33951980	7_73615107_C_T	1.00	5843	4.91E-06	GCST90277385
Triacylglycerol (56:4) levels	8	40367808	A	G	-4.08E-01	8.54E-02	1.31E-02	rs117591451	8_40367808_G_A	0.92	5843	1.87E-06	GCST90277385
Triacylglycerol (56:4) levels	8	85466785	A	G	3.08E-01	6.87E-02	2.02E-02	rs138672261	8_85466785_G_A	0.93	5843	7.60E-06	GCST90277385
Triacylglycerol (56:4) levels	9	2770506	C	T	2.80E-01	5.53E-02	2.99E-02	rs117730598	9_2770506_T_C	0.96	5843	4.31E-07	GCST90277385
Triacylglycerol (56:4) levels	9	27742537	A	T	8.90E-02	1.98E-02	3.75E-01	rs10812660	9_27742537_T_A	0.93	5843	7.02E-06	GCST90277385
Triacylglycerol (56:4) levels	9	120350676	T	C	2.32E-01	4.96E-02	3.92E-02	rs72753379	9_120350676_C_T	0.95	5843	2.96E-06	GCST90277385
Triacylglycerol (56:4) levels	11	116778201	C	G	-1.31E-01	2.53E-02	8.49E-01	rs964184	11_116778201_G_C	1.00	5843	2.24E-07	GCST90277385
Triacylglycerol (56:4) levels	12	83707933	T	A	6.27E-01	1.34E-01	5.21E-03	rs146238333	12_83707933_A_T	0.89	5843	2.75E-06	GCST90277385
Triacylglycerol (56:4) levels	12	105758014	A	G	-9.99E-02	2.14E-02	2.42E-01	rs10861498	12_105758014_G_A	0.99	5843	3.17E-06	GCST90277385
Triacylglycerol (56:4) levels	13	49437013	T	C	-1.02E-01	2.30E-02	2.06E-01	rs2031531	13_49437013_C_T	0.98	5843	9.14E-06	GCST90277385
Triacylglycerol (56:4) levels	14	29818391	G	A	-2.27E-01	5.01E-02	3.72E-02	rs73259585	14_29818391_A_G	0.98	5843	5.85E-06	GCST90277385
Triacylglycerol (56:4) levels	15	39999889	C	A	-2.11E-01	4.77E-02	3.94E-02	rs16970164	15_39999889_A_C	0.99	5843	9.81E-06	GCST90277385
Triacylglycerol (56:4) levels	16	6818646	T	A	-8.49E-02	1.86E-02	5.10E-01	rs8055088	16_6818646_A_T	1.00	5843	4.97E-06	GCST90277385
Triacylglycerol (56:4) levels	18	23684659	C	G	2.44E-01	5.36E-02	3.07E-02	rs113777805	18_23684659_G_C	0.97	5843	5.40E-06	GCST90277385
Triacylglycerol (56:4) levels	19	44908822	T	C	1.99E-01	4.15E-02	5.31E-02	rs7412	19_44908822_C_T	1.00	5843	1.63E-06	GCST90277385
Triacylglycerol (56:5) levels	2	27508073	C	T	-1.20E-01	1.89E-02	6.51E-01	rs1260326	2_27508073_T_C	1.00	6076	2.23E-10	GCST90277386
Triacylglycerol (56:5) levels	4	64367871	A	G	-1.01E-01	2.19E-02	2.15E-01	rs6846577	4_64367871_G_A	1.00	6076	4.27E-06	GCST90277386
Triacylglycerol (56:5) levels	5	114892988	A	G	7.58E-01	1.59E-01	3.33E-03	rs143838492	5_114892988_G_A	0.97	6076	1.88E-06	GCST90277386
Triacylglycerol (56:5) levels	6	50783096	G	A	-5.16E-01	1.12E-01	6.40E-03	rs145055632	6_50783096_A_G	0.99	6076	4.36E-06	GCST90277386
Triacylglycerol (56:5) levels	6	107854775	C	T	1.14E-01	2.48E-02	1.65E-01	rs71556492	6_107854775_T_C	0.98	6076	4.51E-06	GCST90277386
Triacylglycerol (56:5) levels	8	133451589	G	T	4.63E-01	1.01E-01	9.92E-01	rs2736863	8_133451589_T_G	0.96	6076	4.81E-06	GCST90277386
Triacylglycerol (56:5) levels	9	2770506	C	T	2.73E-01	5.45E-02	2.99E-02	rs117730598	9_2770506_T_C	0.96	6076	5.54E-07	GCST90277386
Triacylglycerol (56:5) levels	11	116778201	C	G	-1.55E-01	2.49E-02	8.49E-01	rs964184	11_116778201_G_C	1.00	6076	5.55E-10	GCST90277386
Triacylglycerol (56:5) levels	11	129348709	G	A	-8.49E-02	1.83E-02	4.67E-01	rs4937417	11_129348709_A_G	1.00	6076	3.39E-06	GCST90277386
Triacylglycerol (56:5) levels	12	105758014	A	G	-9.96E-02	2.10E-02	2.42E-01	rs10861498	12_105758014_G_A	0.99	6076	2.27E-06	GCST90277386
Triacylglycerol (56:5) levels	15	59517420	T	G	-1.36E-01	2.87E-02	1.16E-01	rs76411296	15_59517420_G_T	0.98	6076	2.28E-06	GCST90277386
Triacylglycerol (56:5) levels	17	12222568	C	T	-1.64E-01	3.70E-02	6.54E-02	rs10468471	17_12222568_T_C	0.98	6076	9.83E-06	GCST90277386
Triacylglycerol (56:5) levels	17	66539562	C	T	8.48E-02	1.85E-02	4.28E-01	rs1810258	17_66539562_T_C	1.00	6076	4.49E-06	GCST90277386
Triacylglycerol (56:5) levels	18	23684659	C	G	2.33E-01	5.24E-02	3.07E-02	rs113777805	18_23684659_G_C	0.97	6076	9.18E-06	GCST90277386
Triacylglycerol (56:5) levels	18	30887893	G	C	-1.99E-01	4.24E-02	5.09E-02	rs10502551	18_30887893_C_G	0.96	6076	2.76E-06	GCST90277386
Triacylglycerol (56:5) levels	19	19269704	G	A	-2.20E-01	4.11E-02	5.35E-02	rs187429064	19_19269704_A_G	0.95	6076	8.65E-08	GCST90277386
Triacylglycerol (56:5) levels	19	30685856	A	T	-2.97E-01	5.93E-02	2.57E-02	rs140283718	19_30685856_T_A	0.92	6076	5.75E-07	GCST90277386
Triacylglycerol (56:6) levels	1	102847102	G	T	1.33E-01	2.81E-02	1.04E-01	rs79416109	1_102847102_T_G	0.95	7143	2.05E-06	GCST90277387
Triacylglycerol (56:6) levels	1	196028959	T	C	3.37E-01	7.56E-02	1.38E-02	rs78275146	1_196028959_C_T	0.92	7143	8.42E-06	GCST90277387
Triacylglycerol (56:6) levels	1	230598232	T	C	1.48E-01	3.01E-02	9.25E-02	rs55857911	1_230598232_C_T	0.92	7143	9.79E-07	GCST90277387
Triacylglycerol (56:6) levels	2	27508073	C	T	-1.09E-01	1.75E-02	6.51E-01	rs1260326	2_27508073_T_C	1.00	7143	4.18E-10	GCST90277387
Triacylglycerol (56:6) levels	2	226218695	G	A	8.34E-02	1.74E-02	6.32E-01	rs1399627	2_226218695_A_G	1.00	7143	1.76E-06	GCST90277387

Triacylglycerol (56:6) levels	3	104035543	G	T	-7.92E-02	1.78E-02	3.37E-01	rs6804331	3_104035543_T_G	0.99	7143	9.07E-06	GCST90277387
Triacylglycerol (56:6) levels	3	122660865	T	C	-2.34E-01	5.20E-02	2.71E-02	rs144960920	3_122660865_C_T	0.99	7143	6.84E-06	GCST90277387
Triacylglycerol (56:6) levels	5	22651038	A	T	7.77E-02	1.73E-02	3.50E-01	rs268983	5_22651038_T_A	1.00	7143	7.56E-06	GCST90277387
Triacylglycerol (56:6) levels	5	114892988	A	G	6.73E-01	1.49E-01	3.33E-03	rs143838492	5_114892988_G_A	0.97	7143	6.07E-06	GCST90277387
Triacylglycerol (56:6) levels	5	151110256	T	C	1.01E-01	2.26E-02	1.66E-01	rs9324675	5_151110256_C_T	0.99	7143	8.16E-06	GCST90277387
Triacylglycerol (56:6) levels	6	23919006	G	A	-6.16E-01	1.37E-01	4.04E-03	rs79934453	6_23919006_A_G	0.91	7143	7.04E-06	GCST90277387
Triacylglycerol (56:6) levels	6	32168770	G	T	9.98E-02	1.69E-02	5.84E-01	rs1061808	6_32168770_T_G	1.00	7143	3.91E-09	GCST90277387
Triacylglycerol (56:6) levels	6	66453262	C	A	-5.72E-01	1.28E-01	4.89E-03	rs113739902	6_66453262_A_C	0.92	7143	7.97E-06	GCST90277387
Triacylglycerol (56:6) levels	8	2825696	C	T	-3.98E-01	8.98E-02	9.63E-03	rs75178403	8_2825696_T_C	0.88	7143	9.44E-06	GCST90277387
Triacylglycerol (56:6) levels	9	2770506	C	T	2.35E-01	5.04E-02	2.99E-02	rs117730598	9_2770506_T_C	0.96	7143	2.99E-06	GCST90277387
Triacylglycerol (56:6) levels	9	120350676	T	C	2.04E-01	4.48E-02	3.92E-02	rs72753379	9_120350676_C_T	0.95	7143	5.18E-06	GCST90277387
Triacylglycerol (56:6) levels	9	132455588	A	C	7.87E-02	1.76E-02	3.40E-01	rs480021	9_132455588_C_A	1.00	7143	7.35E-06	GCST90277387
Triacylglycerol (56:6) levels	9	136257994	G	A	-2.40E-01	4.86E-02	3.60E-02	rs112122274	9_136257994_A_G	0.85	7143	7.85E-07	GCST90277387
Triacylglycerol (56:6) levels	11	116778201	C	G	-1.62E-01	2.32E-02	8.49E-01	rs964184	11_116778201_G_C	1.00	7143	3.12E-12	GCST90277387
Triacylglycerol (56:6) levels	11	132660922	A	G	6.75E-01	1.41E-01	4.06E-03	rs61906420	11_132660922_G_A	0.85	7143	1.64E-06	GCST90277387
Triacylglycerol (56:6) levels	12	105758014	A	G	-8.94E-02	1.92E-02	2.42E-01	rs10861498	12_105758014_G_A	0.99	7143	3.20E-06	GCST90277387
Triacylglycerol (56:6) levels	14	36679967	A	C	-8.23E-02	1.73E-02	6.23E-01	rs1884216	14_36679967_C_A	1.00	7143	2.03E-06	GCST90277387
Triacylglycerol (56:6) levels	15	39994335	C	T	-2.02E-01	4.14E-02	4.19E-02	rs75931581	15_39994335_T_C	0.99	7143	1.13E-06	GCST90277387
Triacylglycerol (56:6) levels	16	6791691	C	A	-1.77E-01	3.84E-02	5.08E-02	rs62016118	16_6791691_A_C	0.97	7143	4.08E-06	GCST90277387
Triacylglycerol (56:6) levels	17	78119940	G	A	2.14E-01	4.79E-02	3.25E-02	rs187952596	17_78119940_A_G	0.94	7143	8.37E-06	GCST90277387
Triacylglycerol (56:6) levels	18	5149977	G	C	-7.84E-02	1.74E-02	6.51E-01	rs447117	18_5149977_C_G	1.00	7143	6.56E-06	GCST90277387
Triacylglycerol (56:7) levels	1	102847102	G	T	1.28E-01	2.80E-02	1.04E-01	rs79416109	1_102847102_T_G	0.95	7172	5.23E-06	GCST90277388
Triacylglycerol (56:7) levels	1	196028959	T	C	3.40E-01	7.53E-02	1.38E-02	rs78275146	1_196028959_C_T	0.92	7172	6.59E-06	GCST90277388
Triacylglycerol (56:7) levels	2	27508073	C	T	-1.39E-01	1.74E-02	6.51E-01	rs1260326	2_27508073_T_C	1.00	7172	1.53E-15	GCST90277388
Triacylglycerol (56:7) levels	2	36390910	G	A	-1.12E-01	2.42E-02	1.35E-01	rs111430300	2_36390910_A_G	1.00	7172	3.36E-06	GCST90277388
Triacylglycerol (56:7) levels	4	81524180	G	T	-8.09E-02	1.82E-02	3.06E-01	rs10213073	4_81524180_T_G	1.00	7172	9.29E-06	GCST90277388
Triacylglycerol (56:7) levels	5	114892988	A	G	7.23E-01	1.48E-01	3.33E-03	rs143838492	5_114892988_G_A	0.97	7172	1.15E-06	GCST90277388
Triacylglycerol (56:7) levels	6	23919006	G	A	-6.43E-01	1.37E-01	4.04E-03	rs79934453	6_23919006_A_G	0.91	7172	2.72E-06	GCST90277388
Triacylglycerol (56:7) levels	8	19967156	T	C	-8.64E-02	1.89E-02	2.61E-01	rs15285	8_19967156_C_T	1.00	7172	5.17E-06	GCST90277388
Triacylglycerol (56:7) levels	8	59089983	T	C	9.27E-02	2.08E-02	1.98E-01	rs1010313	8_59089983_C_T	1.00	7172	8.23E-06	GCST90277388
Triacylglycerol (56:7) levels	9	24032283	T	C	1.62E-01	3.60E-02	5.82E-02	rs4615671	9_24032283_C_T	0.95	7172	7.13E-06	GCST90277388
Triacylglycerol (56:7) levels	9	120350676	T	C	2.16E-01	4.47E-02	3.92E-02	rs72753379	9_120350676_C_T	0.95	7172	1.42E-06	GCST90277388
Triacylglycerol (56:7) levels	9	136257994	G	A	-2.19E-01	4.85E-02	3.60E-02	rs112122274	9_136257994_A_G	0.85	7172	6.84E-06	GCST90277388
Triacylglycerol (56:7) levels	10	62375635	CA	C	-2.60E-01	5.63E-02	2.27E-02	rs35162925	10_62375635_C_CA	1.00	7172	3.94E-06	GCST90277388
Triacylglycerol (56:7) levels	11	116778201	C	G	-1.84E-01	2.31E-02	8.49E-01	rs964184	11_116778201_G_C	1.00	7172	2.10E-15	GCST90277388
Triacylglycerol (56:7) levels	12	105758014	A	G	-8.46E-02	1.91E-02	2.42E-01	rs10861498	12_105758014_G_A	0.99	7172	9.89E-06	GCST90277388
Triacylglycerol (56:7) levels	15	92583935	T	G	8.89E-02	2.00E-02	2.26E-01	rs17704001	15_92583935_G_T	1.00	7172	9.24E-06	GCST90277388
Triacylglycerol (56:7) levels	19	19269704	G	A	-1.72E-01	3.79E-02	5.35E-02	rs187429064	19_19269704_A_G	0.95	7172	5.47E-06	GCST90277388
Triacylglycerol (56:7) levels	20	13842673	G	C	-7.84E-02	1.71E-02	6.40E-01	rs6033833	20_13842673_C_G	1.00	7172	4.48E-06	GCST90277388
Triacylglycerol (56:7) levels	22	23971765	G	A	1.64E-01	3.56E-02	6.00E-02	rs79213832	22_23971765_A_G	0.98	7172	4.31E-06	GCST90277388
Triacylglycerol (56:8) levels	1	227027789	C	T	-1.15E-01	2.56E-02	1.23E-01	rs12120691	1_227027789_T_C	0.99	7161	6.66E-06	GCST90277389
Triacylglycerol (56:8) levels	2	21002409	T	C	9.86E-02	1.86E-02	7.28E-01	rs1042034	2_21002409_C_T	1.00	7161	1.25E-07	GCST90277389
Triacylglycerol (56:8) levels	2	27508073	C	T	-1.59E-01	1.74E-02	6.51E-01	rs1260326	2_27508073_T_C	1.00	7161	7.41E-20	GCST90277389
Triacylglycerol (56:8) levels	2	36390910	G	A	-1.11E-01	2.42E-02	1.35E-01	rs111430300	2_36390910_A_G	1.00	7161	4.66E-06	GCST90277389
Triacylglycerol (56:8) levels	3	72433728	G	A	3.59E-01	8.09E-02	1.20E-02	rs79771052	3_72433728_A_G	0.91	7161	8.91E-06	GCST90277389
Triacylglycerol (56:8) levels	3	87298641	A	G	-1.86E-01	3.92E-02	4.88E-02	rs116011373	3_87298641_G_A	0.96	7161	2.25E-06	GCST90277389
Triacylglycerol (56:8) levels	4	120830005	C	G	-6.43E-01	1.36E-01	4.41E-03	rs139499638	4_120830005_G_C	0.92	7161	2.35E-06	GCST90277389
Triacylglycerol (56:8) levels	5	114892988	A	G	6.76E-01	1.48E-01	3.33E-03	rs143838492	5_114892988_G_A	0.97	7161	5.34E-06	GCST90277389
Triacylglycerol (56:8) levels	6	23919006	G	A	-6.37E-01	1.37E-01	4.04E-03	rs79934453	6_23919006_A_G	0.91	7161	3.41E-06	GCST90277389
Triacylglycerol (56:8) levels	6	49123122	A	C	-4.40E-01	9.57E-02	8.14E-03	rs79954170	6_49123122_C_A	0.96	7161	4.32E-06	GCST90277389
Triacylglycerol (56:8) levels	6	64177173	T	C	8.01E-02	1.72E-02	3.73E-01	rs9344829	6_64177173_C_T	0.99	7161	3.39E-06	GCST90277389
Triacylglycerol (56:8) levels	7	73603327	T	C	-1.03E-01	2.23E-02	1.67E-01	rs35368205	7_73603327_C_T	1.00	7161	4.03E-06	GCST90277389
Triacylglycerol (56:8) levels	8	19970337	A	C	-1.01E-01	1.85E-02	2.79E-01	rs10105606	8_19970337_C_A	1.00	7161	5.10E-08	GCST90277389
Triacylglycerol (56:8) levels	8	59082215	C	A	9.87E-02	2.09E-02	1.97E-01	rs1428264	8_59082215_A_C	1.00	7161	2.24E-06	GCST90277389
Triacylglycerol (56:8) levels	9	80090365	A	G	1.04E-01	2.19E-02	1.79E-01	rs72738698	9_80090365_G_A	0.99	7161	1.95E-06	GCST90277389
Triacylglycerol (56:8) levels	9	120350676	T	C	2.08E-01	4.47E-02	3.92E-02	rs72753379	9_120350676_C_T	0.95	7161	3.22E-06	GCST90277389
Triacylglycerol (56:8) levels	9	130572514	A	C	2.94E-01	6.64E-02	1.71E-02	rs12344868	9_130572514_C_A	0.99	7161	9.56E-06	GCST90277389

Triacylglycerol (56:8) levels	11	3228978	G	A	-1.01E-01	2.24E-02	8.32E-01	rs10741876	11_3228978_A_G	0.99	7161	6.10E-06	GCST90277389
Triacylglycerol (56:8) levels	11	38161116	G	A	1.40E-01	3.13E-02	9.17E-01	rs820903	11_38161116_A_G	0.95	7161	7.51E-06	GCST90277389
Triacylglycerol (56:8) levels	11	116778201	C	G	-2.13E-01	2.31E-02	8.49E-01	rs964184	11_116778201_G_C	1.00	7161	4.05E-20	GCST90277389
Triacylglycerol (56:8) levels	14	76971123	A	G	1.21E-01	2.74E-02	1.05E-01	rs111526079	14_76971123_G_A	0.96	7161	9.51E-06	GCST90277389
Triacylglycerol (56:8) levels	14	92538920	G	A	2.11E-01	4.64E-02	3.57E-02	rs72697234	14_92538920_A_G	0.93	7161	5.49E-06	GCST90277389
Triacylglycerol (56:8) levels	14	95566739	A	G	-7.51E-02	1.65E-02	4.87E-01	rs11849573	14_95566739_G_A	1.00	7161	5.58E-06	GCST90277389
Triacylglycerol (56:8) levels	15	92583935	T	G	8.96E-02	2.00E-02	2.26E-01	rs17704001	15_92583935_G_T	1.00	7161	7.71E-06	GCST90277389
Triacylglycerol (56:8) levels	17	1623049	C	T	9.59E-02	2.14E-02	1.96E-01	rs35986054	17_1623049_T_C	0.98	7161	7.59E-06	GCST90277389
Triacylglycerol (56:8) levels	18	23684659	C	G	2.19E-01	4.90E-02	3.07E-02	rs113777805	18_23684659_G_C	0.97	7161	8.15E-06	GCST90277389
Triacylglycerol (56:8) levels	18	30887893	G	C	-1.70E-01	3.85E-02	5.09E-02	rs10502551	18_30887893_C_G	0.96	7161	9.80E-06	GCST90277389
Triacylglycerol (56:8) levels	19	19269704	G	A	-2.35E-01	3.78E-02	5.35E-02	rs187429064	19_19269704_A_G	0.95	7161	5.20E-10	GCST90277389
Triacylglycerol (56:8) levels	19	55802466	C	T	-1.62E-01	3.57E-02	6.03E-02	rs79152531	19_55802466_T_C	0.97	7161	5.94E-06	GCST90277389
Triacylglycerol (56:8) levels	22	23971765	G	A	1.66E-01	3.56E-02	6.00E-02	rs79213832	22_23971765_A_G	0.98	7161	3.16E-06	GCST90277389
Triacylglycerol (58:7) levels	1	227027789	C	T	-1.18E-01	2.56E-02	1.23E-01	rs12120691	1_227027789_T_C	0.99	7166	3.71E-06	GCST90277390
Triacylglycerol (58:7) levels	2	21002409	T	C	9.32E-02	1.86E-02	7.28E-01	rs1042034	2_21002409_C_T	1.00	7166	5.96E-07	GCST90277390
Triacylglycerol (58:7) levels	2	27508073	C	T	-1.69E-01	1.74E-02	6.51E-01	rs1260326	2_27508073_T_C	1.00	7166	3.53E-22	GCST90277390
Triacylglycerol (58:7) levels	3	11749984	G	A	8.14E-02	1.77E-02	3.40E-01	rs301555	3_11749984_A_G	0.98	7166	4.36E-06	GCST90277390
Triacylglycerol (58:7) levels	3	87180483	T	G	-1.84E-01	3.92E-02	4.98E-02	rs116497138	3_87180483_G_T	0.94	7166	2.68E-06	GCST90277390
Triacylglycerol (58:7) levels	3	151540066	A	G	2.30E-01	5.12E-02	2.79E-02	rs116635711	3_151540066_G_A	0.99	7166	7.33E-06	GCST90277390
Triacylglycerol (58:7) levels	5	157774262	C	T	1.16E-01	2.58E-02	1.19E-01	rs17054866	5_157774262_T_C	0.99	7166	6.86E-06	GCST90277390
Triacylglycerol (58:7) levels	6	64177173	T	C	7.70E-02	1.72E-02	3.73E-01	rs9344829	6_64177173_C_T	0.99	7166	7.85E-06	GCST90277390
Triacylglycerol (58:7) levels	8	764450	G	A	-9.96E-02	2.11E-02	2.00E-01	rs11783556	8_764450_A_G	0.99	7166	2.35E-06	GCST90277390
Triacylglycerol (58:7) levels	8	19970337	A	C	-9.73E-02	1.85E-02	2.79E-01	rs10105606	8_19970337_C_A	1.00	7166	1.54E-07	GCST90277390
Triacylglycerol (58:7) levels	8	58330047	G	A	1.85E-01	3.86E-02	4.81E-02	rs191616475	8_58330047_A_G	1.00	7166	1.61E-06	GCST90277390
Triacylglycerol (58:7) levels	9	2770506	C	T	2.36E-01	5.02E-02	2.99E-02	rs117730598	9_2770506_T_C	0.96	7166	2.61E-06	GCST90277390
Triacylglycerol (58:7) levels	9	33656919	C	A	8.56E-02	1.86E-02	7.25E-01	rs1832326	9_33656919_A_C	1.00	7166	4.51E-06	GCST90277390
Triacylglycerol (58:7) levels	9	80090365	A	G	1.05E-01	2.19E-02	1.79E-01	rs72738698	9_80090365_G_A	0.99	7166	1.44E-06	GCST90277390
Triacylglycerol (58:7) levels	9	120284301	G	A	-2.14E-01	4.70E-02	9.64E-01	rs759126	9_120284301_A_G	0.92	7166	5.56E-06	GCST90277390
Triacylglycerol (58:7) levels	9	132462939	ATGGT	A	1.85E-01	3.91E-02	4.81E-02	rs371163839	9_132462939_A_ATGGT	0.98	7166	2.12E-06	GCST90277390
Triacylglycerol (58:7) levels	10	7871139	C	T	4.16E-01	8.80E-02	9.45E-03	rs190411877	10_7871139_T_C	0.94	7166	2.38E-06	GCST90277390
Triacylglycerol (58:7) levels	11	7455964	G	A	1.35E-01	2.99E-02	8.65E-02	rs72846885	11_7455964_A_G	0.98	7166	6.30E-06	GCST90277390
Triacylglycerol (58:7) levels	11	38161116	G	A	1.40E-01	3.13E-02	9.17E-01	rs820903	11_38161116_A_G	0.95	7166	7.48E-06	GCST90277390
Triacylglycerol (58:7) levels	11	116778201	C	G	-2.03E-01	2.31E-02	8.49E-01	rs964184	11_116778201_G_C	1.00	7166	2.01E-18	GCST90277390
Triacylglycerol (58:7) levels	14	92538920	G	A	2.07E-01	4.64E-02	3.57E-02	rs72697234	14_92538920_A_G	0.93	7166	7.93E-06	GCST90277390
Triacylglycerol (58:7) levels	15	61669154	A	G	7.44E-02	1.66E-02	5.25E-01	rs2249682	15_61669154_G_A	1.00	7166	7.64E-06	GCST90277390
Triacylglycerol (58:7) levels	17	49154037	GCT	G	1.21E-01	2.53E-02	1.21E-01	rs61156392	17_49154037_G_GCT	0.98	7166	1.70E-06	GCST90277390
Triacylglycerol (58:7) levels	18	23684659	C	G	2.21E-01	4.90E-02	3.07E-02	rs113777805	18_23684659_G_C	0.97	7166	6.34E-06	GCST90277390
Triacylglycerol (58:7) levels	18	30887893	G	C	-1.72E-01	3.86E-02	5.09E-02	rs10502551	18_30887893_C_G	0.96	7166	8.00E-06	GCST90277390
Triacylglycerol (58:7) levels	18	69122883	T	C	-2.41E-01	5.44E-02	9.76E-01	rs4453607	18_69122883_C_T	0.98	7166	9.71E-06	GCST90277390
Triacylglycerol (58:7) levels	18	79647516	A	G	7.79E-02	1.69E-02	5.45E-01	rs668013	18_79647516_G_A	0.98	7166	4.34E-06	GCST90277390
Triacylglycerol (58:7) levels	19	19269704	G	A	-2.47E-01	3.78E-02	5.35E-02	rs187429064	19_19269704_A_G	0.95	7166	7.59E-11	GCST90277390
Triacylglycerol (58:7) levels	19	34971287	G	A	-7.61E-02	1.66E-02	5.33E-01	rs2546043	19_34971287_A_G	0.99	7166	4.65E-06	GCST90277390
Triacylglycerol (58:7) levels	19	55802466	C	T	-1.77E-01	3.57E-02	6.03E-02	rs79152531	19_55802466_T_C	0.97	7166	7.55E-07	GCST90277390
Triacylglycerol (58:7) levels	22	23971765	G	A	1.68E-01	3.56E-02	6.00E-02	rs79213832	22_23971765_A_G	0.98	7166	2.30E-06	GCST90277390
Triacylglycerol (58:8) levels	1	25641071	C	T	1.05E-01	2.36E-02	2.11E-01	rs3767910	1_25641071_T_C	0.99	5491	9.28E-06	GCST90277391
Triacylglycerol (58:8) levels	1	63377301	A	G	-9.72E-02	2.19E-02	2.67E-01	rs11208199	1_63377301_G_A	0.97	5491	9.03E-06	GCST90277391
Triacylglycerol (58:8) levels	2	27508073	C	T	-1.57E-01	1.98E-02	6.51E-01	rs1260326	2_27508073_T_C	1.00	5491	2.77E-15	GCST90277391
Triacylglycerol (58:8) levels	4	28034565	T	G	3.04E-01	6.79E-02	2.16E-02	rs115914883	4_28034565_G_T	0.96	5491	7.61E-06	GCST90277391
Triacylglycerol (58:8) levels	5	108951913	T	A	1.08E-01	2.29E-02	2.20E-01	rs3906419	5_108951913_A_T	1.00	5491	2.28E-06	GCST90277391
Triacylglycerol (58:8) levels	5	157845455	A	G	1.01E-01	1.91E-02	4.42E-01	rs254668	5_157845455_G_A	1.00	5491	1.28E-07	GCST90277391
Triacylglycerol (58:8) levels	5	176875666	G	C	-4.22E-01	9.20E-02	1.23E-02	rs187087759	5_176875666_C_G	0.86	5491	4.65E-06	GCST90277391
Triacylglycerol (58:8) levels	7	71777939	C	G	5.26E-01	1.18E-01	7.15E-03	rs78571096	7_71777939_G_C	0.87	5491	7.87E-06	GCST90277391
Triacylglycerol (58:8) levels	8	3605480	A	G	-6.10E-01	1.37E-01	5.81E-03	rs117906663	8_3605480_G_A	0.89	5491	8.78E-06	GCST90277391
Triacylglycerol (58:8) levels	8	9820239	G	C	-8.37E-01	1.84E-01	2.68E-03	rs143248091	8_9820239_C_G	0.99	5491	5.73E-06	GCST90277391
Triacylglycerol (58:8) levels	8	125495147	A	C	-9.96E-02	2.18E-02	7.43E-01	rs2954038	8_125495147_C_A	1.00	5491	5.22E-06	GCST90277391
Triacylglycerol (58:8) levels	8	139794453	C	T	-2.03E-01	4.36E-02	5.08E-02	rs67080320	8_139794453_T_C	1.00	5491	3.25E-06	GCST90277391
Triacylglycerol (58:8) levels	9	2770506	C	T	2.66E-01	5.60E-02	2.99E-02	rs117730598	9_2770506_T_C	0.96	5491	2.10E-06	GCST90277391

Triacylglycerol (58:8) levels	10	12582085	T	C	5.73E-01	1.26E-01	6.25E-03	rs11257904	10_12582085_C_T	0.97	5491	5.45E-06	GCST90277391
Triacylglycerol (58:8) levels	11	21740959	C	T	-2.22E-01	4.50E-02	5.05E-02	rs150889723	11_21740959_T_C	0.93	5491	7.81E-07	GCST90277391
Triacylglycerol (58:8) levels	11	78029938	T	C	-3.05E-01	6.79E-02	2.12E-02	rs181240184	11_78029938_C_T	0.97	5491	7.15E-06	GCST90277391
Triacylglycerol (58:8) levels	11	116778201	C	G	-1.84E-01	2.58E-02	8.49E-01	rs964184	11_116778201_G_C	1.00	5491	1.16E-12	GCST90277391
Triacylglycerol (58:8) levels	13	25886443	T	C	-6.72E-01	1.48E-01	5.33E-03	rs112578388	13_25886443_C_T	0.86	5491	5.82E-06	GCST90277391
Triacylglycerol (58:8) levels	16	27125614	A	G	4.95E-01	1.10E-01	7.67E-03	rs116876951	16_27125614_G_A	0.99	5491	7.37E-06	GCST90277391
Triacylglycerol (58:8) levels	16	70692892	A	C	8.85E-02	1.93E-02	5.36E-01	rs2278983	16_70692892_C_A	0.98	5491	4.76E-06	GCST90277391
Triacylglycerol (58:8) levels	18	44974706	G	A	1.48E-01	3.15E-02	1.04E-01	rs7241605	18_44974706_A_G	0.99	5491	2.82E-06	GCST90277391
Triacylglycerol (58:8) levels	19	3058543	T	C	1.54E-01	3.46E-02	9.35E-02	rs62125455	19_3058543_C_T	0.89	5491	8.72E-06	GCST90277391
Triacylglycerol (58:8) levels	19	19269704	G	A	-2.69E-01	4.45E-02	5.35E-02	rs187429064	19_19269704_A_G	0.95	5491	1.62E-09	GCST90277391
Triacylglycerol (58:8) levels	19	34974283	C	A	-9.45E-02	1.96E-02	6.21E-01	rs888860	19_34974283_A_C	1.00	5491	1.39E-06	GCST90277391
Triacylglycerol (58:8) levels	19	38522685	C	T	1.00E-01	2.16E-02	2.60E-01	rs2915945	19_38522685_T_C	0.99	5491	3.90E-06	GCST90277391
Triacylglycerol (58:8) levels	22	19759510	G	C	8.49E-01	1.63E-01	4.23E-03	rs72646948	22_19759510_C_G	0.85	5491	1.85E-07	GCST90277391
Diacylglycerol (18:1_18:3) levels	1	111242194	A	G	-1.10E-01	2.38E-02	1.98E-01	rs11102221	1_111242194_G_A	1.00	5495	3.73E-06	GCST90277392
Diacylglycerol (18:1_18:3) levels	2	27508073	C	T	-1.02E-01	1.99E-02	6.51E-01	rs1260326	2_27508073_T_C	1.00	5495	2.96E-07	GCST90277392
Diacylglycerol (18:1_18:3) levels	2	49039715	C	T	2.14E-01	4.84E-02	4.16E-02	rs12613782	2_49039715_T_C	1.00	5495	9.64E-06	GCST90277392
Diacylglycerol (18:1_18:3) levels	2	62122935	G	A	-2.51E-01	5.68E-02	3.00E-02	rs67093838	2_62122935_A_G	0.97	5495	9.88E-06	GCST90277392
Diacylglycerol (18:1_18:3) levels	4	34767471	G	A	4.51E-01	9.52E-02	1.06E-02	rs140828820	4_34767471_A_G	0.93	5495	2.18E-06	GCST90277392
Diacylglycerol (18:1_18:3) levels	4	62360675	C	T	-1.17E-01	2.41E-02	1.99E-01	rs2169099	4_62360675_T_C	0.99	5495	1.21E-06	GCST90277392
Diacylglycerol (18:1_18:3) levels	5	133108436	A	G	-1.97E-01	4.42E-02	5.08E-02	rs72801474	5_133108436_G_A	0.96	5495	8.77E-06	GCST90277392
Diacylglycerol (18:1_18:3) levels	7	73440219	T	C	-1.47E-01	3.26E-02	9.53E-02	rs71556711	7_73440219_C_T	0.99	5495	6.93E-06	GCST90277392
Diacylglycerol (18:1_18:3) levels	9	2640492	G	A	-1.01E-01	2.21E-02	7.52E-01	rs2242104	9_2640492_A_G	0.99	5495	5.25E-06	GCST90277392
Diacylglycerol (18:1_18:3) levels	9	33414022	G	A	-8.85E-02	2.00E-02	5.99E-01	rs832231	9_33414022_A_G	0.96	5495	9.78E-06	GCST90277392
Diacylglycerol (18:1_18:3) levels	9	71635471	C	A	-1.02E-01	2.06E-02	6.47E-01	rs1928423	9_71635471_A_C	0.95	5495	8.56E-07	GCST90277392
Diacylglycerol (18:1_18:3) levels	9	113789023	A	G	-9.67E-02	2.04E-02	6.77E-01	rs4979284	9_113789023_G_A	0.99	5495	2.13E-06	GCST90277392
Diacylglycerol (18:1_18:3) levels	9	132470502	G	A	3.38E-01	6.21E-02	2.59E-02	rs111568723	9_132470502_A_G	0.95	5495	5.37E-08	GCST90277392
Diacylglycerol (18:1_18:3) levels	10	85979448	A	G	-8.77E-02	1.92E-02	4.70E-01	rs4448624	10_85979448_G_A	0.98	5495	4.82E-06	GCST90277392
Diacylglycerol (18:1_18:3) levels	10	132832356	G	C	1.80E-01	3.90E-02	6.51E-02	rs7076657	10_132832356_C_G	0.97	5495	4.21E-06	GCST90277392
Diacylglycerol (18:1_18:3) levels	11	26777276	A	T	8.47E-02	1.90E-02	4.28E-01	rs10767577	11_26777276_T_A	0.99	5495	8.81E-06	GCST90277392
Diacylglycerol (18:1_18:3) levels	11	96250298	T	C	-1.73E-01	3.84E-02	7.01E-02	rs143186626	11_96250298_C_T	0.97	5495	6.47E-06	GCST90277392
Diacylglycerol (18:1_18:3) levels	11	116778201	C	G	-1.40E-01	2.62E-02	8.49E-01	rs964184	11_116778201_G_C	1.00	5495	9.62E-08	GCST90277392
Diacylglycerol (18:1_18:3) levels	12	83707933	T	A	6.21E-01	1.34E-01	5.21E-03	rs146238333	12_83707933_A_T	0.89	5495	3.60E-06	GCST90277392
Diacylglycerol (18:1_18:3) levels	17	51439207	A	G	-1.12E-01	2.24E-02	2.37E-01	rs34496841	17_51439207_G_A	0.99	5495	5.15E-07	GCST90277392
Diacylglycerol (18:1_18:3) levels	22	45070829	A	G	1.74E-01	3.78E-02	7.90E-02	rs116845319	22_45070829_G_A	0.90	5495	4.11E-06	GCST90277392
Lysophosphatidylcholine (16:0) levels	1	27100305	C	T	1.60E-01	3.62E-02	9.41E-01	rs4266911	1_27100305_T_C	0.94	7149	9.76E-06	GCST90277393
Lysophosphatidylcholine (16:0) levels	1	103243167	T	C	1.47E-01	3.22E-02	7.72E-02	rs144241030	1_103243167_C_T	0.94	7149	5.05E-06	GCST90277393
Lysophosphatidylcholine (16:0) levels	2	27508073	C	T	-1.28E-01	1.74E-02	6.51E-01	rs1260326	2_27508073_T_C	1.00	7149	2.46E-13	GCST90277393
Lysophosphatidylcholine (16:0) levels	2	128427075	G	A	1.63E-01	3.21E-02	7.45E-02	rs76950187	2_128427075_A_G	0.99	7149	3.65E-07	GCST90277393
Lysophosphatidylcholine (16:0) levels	2	170225142	C	G	1.61E-01	3.50E-02	9.37E-01	rs6721083	2_170225142_G_C	0.95	7149	4.43E-06	GCST90277393
Lysophosphatidylcholine (16:0) levels	3	134977489	T	C	-1.16E-01	2.64E-02	1.19E-01	rs17282355	3_134977489_C_T	0.96	7149	1.00E-05	GCST90277393
Lysophosphatidylcholine (16:0) levels	4	120830005	C	G	-6.17E-01	1.36E-01	4.41E-03	rs139499638	4_120830005_G_C	0.92	7149	6.00E-06	GCST90277393
Lysophosphatidylcholine (16:0) levels	4	182501447	G	T	7.47E-02	1.68E-02	5.16E-01	rs28437809	4_182501447_T_G	0.99	7149	8.29E-06	GCST90277393
Lysophosphatidylcholine (16:0) levels	5	114892988	A	G	6.81E-01	1.48E-01	3.33E-03	rs143838492	5_114892988_G_A	0.97	7149	4.53E-06	GCST90277393
Lysophosphatidylcholine (16:0) levels	6	2839825	T	C	-7.58E-02	1.71E-02	4.68E-01	rs316343	6_2839825_C_T	0.98	7149	9.37E-06	GCST90277393
Lysophosphatidylcholine (16:0) levels	7	73615107	T	C	-1.36E-01	2.55E-02	1.22E-01	rs33951980	7_73615107_C_T	1.00	7149	9.09E-08	GCST90277393
Lysophosphatidylcholine (16:0) levels	9	2770506	C	T	2.31E-01	5.04E-02	2.99E-02	rs117730598	9_2770506_T_C	0.96	7149	4.35E-06	GCST90277393
Lysophosphatidylcholine (16:0) levels	9	120350676	T	C	2.07E-01	4.48E-02	3.92E-02	rs72753379	9_120350676_C_T	0.95	7149	3.94E-06	GCST90277393
Lysophosphatidylcholine (16:0) levels	9	136001733	A	G	-8.84E-02	1.98E-02	2.40E-01	rs28368829	9_136001733_G_A	0.99	7149	8.07E-06	GCST90277393
Lysophosphatidylcholine (16:0) levels	9	136257994	G	A	-2.21E-01	4.87E-02	3.60E-02	rs112122274	9_136257994_A_G	0.85	7149	5.64E-06	GCST90277393
Lysophosphatidylcholine (16:0) levels	11	61813163	T	C	8.45E-02	1.73E-02	3.80E-01	rs174556	11_61813163_C_T	1.00	7149	1.08E-06	GCST90277393
Lysophosphatidylcholine (16:0) levels	11	116778201	C	G	-2.12E-01	2.31E-02	8.49E-01	rs964184	11_116778201_G_C	1.00	7149	5.60E-20	GCST90277393
Lysophosphatidylcholine (16:0) levels	13	21038010	T	C	-1.35E-01	3.05E-02	8.45E-02	rs75573052	13_21038010_C_T	0.99	7149	9.70E-06	GCST90277393
Lysophosphatidylcholine (16:0) levels	14	29823442	G	A	-2.27E-01	4.47E-02	3.72E-02	rs10147474	14_29823442_A_G	0.98	7149	3.98E-07	GCST90277393
Lysophosphatidylcholine (16:0) levels	15	93154154	A	C	-7.96E-02	1.68E-02	4.18E-01	rs62043594	15_93154154_C_A	1.00	7149	2.16E-06	GCST90277393
Lysophosphatidylcholine (16:0) levels	19	44908822	T	C	1.68E-01	3.74E-02	5.31E-02	rs7412	19_44908822_C_T	1.00	7149	7.41E-06	GCST90277393
Lysophosphatidylcholine (18:0) levels	1	230148628	T	C	-8.82E-02	1.89E-02	7.40E-01	rs6692319	1_230148628_C_T	1.00	7119	2.93E-06	GCST90277394
Lysophosphatidylcholine (18:0) levels	2	21002409	T	C	1.07E-01	1.87E-02	7.28E-01	rs1042034	2_21002409_C_T	1.00	7119	1.01E-08	GCST90277394

Lysophosphatidylcholine (18:0) levels	2	27508073	C	T	-1.38E-01	1.75E-02	6.51E-01	rs1260326	2_27508073_T_C	1.00	7119	3.28E-15	GCST90277394
Lysophosphatidylcholine (18:0) levels	3	1895465	G	A	7.53E-02	1.68E-02	5.60E-01	rs12635725	3_1895465_A_G	0.99	7119	7.13E-06	GCST90277394
Lysophosphatidylcholine (18:0) levels	3	87298641	A	G	-1.91E-01	3.94E-02	4.88E-02	rs116011373	3_87298641_G_A	0.96	7119	1.29E-06	GCST90277394
Lysophosphatidylcholine (18:0) levels	3	148395586	G	A	-7.99E-02	1.70E-02	4.83E-01	rs35397574	3_148395586_A_G	0.98	7119	2.72E-06	GCST90277394
Lysophosphatidylcholine (18:0) levels	3	177023858	C	A	2.52E-01	5.63E-02	2.19E-02	rs139278484	3_177023858_A_C	1.00	7119	7.54E-06	GCST90277394
Lysophosphatidylcholine (18:0) levels	4	107886862	T	C	-1.48E-01	3.22E-02	8.04E-02	rs11728165	4_107886862_C_T	0.93	7119	4.08E-06	GCST90277394
Lysophosphatidylcholine (18:0) levels	4	120830005	C	G	-6.59E-01	1.36E-01	4.41E-03	rs139499638	4_120830005_G_C	0.92	7119	1.28E-06	GCST90277394
Lysophosphatidylcholine (18:0) levels	4	163552148	G	A	3.02E-01	6.65E-02	1.62E-02	rs138427786	4_163552148_A_G	0.97	7119	5.76E-06	GCST90277394
Lysophosphatidylcholine (18:0) levels	6	49493994	G	A	-4.48E-01	9.52E-02	8.35E-03	rs146131769	6_49493994_A_G	0.97	7119	2.58E-06	GCST90277394
Lysophosphatidylcholine (18:0) levels	7	6295952	T	A	8.98E-02	2.01E-02	2.32E-01	rs7782961	7_6295952_A_T	0.97	7119	7.66E-06	GCST90277394
Lysophosphatidylcholine (18:0) levels	7	73597712	A	G	-1.51E-01	2.55E-02	1.22E-01	rs35332062	7_73597712_G_A	1.00	7119	3.41E-09	GCST90277394
Lysophosphatidylcholine (18:0) levels	8	19967156	T	C	-1.06E-01	1.90E-02	2.61E-01	rs15285	8_19967156_C_T	1.00	7119	2.62E-08	GCST90277394
Lysophosphatidylcholine (18:0) levels	8	59088496	C	T	9.49E-02	2.13E-02	1.91E-01	rs7846649	8_59088496_T_C	0.99	7119	8.14E-06	GCST90277394
Lysophosphatidylcholine (18:0) levels	9	2770506	C	T	2.43E-01	5.05E-02	2.99E-02	rs117730598	9_2770506_T_C	0.96	7119	1.60E-06	GCST90277394
Lysophosphatidylcholine (18:0) levels	9	132470502	G	A	2.42E-01	5.47E-02	2.59E-02	rs111568723	9_132470502_A_G	0.95	7119	9.58E-06	GCST90277394
Lysophosphatidylcholine (18:0) levels	11	26777276	A	T	7.78E-02	1.68E-02	4.28E-01	rs10767577	11_26777276_T_A	0.99	7119	3.68E-06	GCST90277394
Lysophosphatidylcholine (18:0) levels	11	116778201	C	G	-2.49E-01	2.31E-02	8.49E-01	rs964184	11_116778201_G_C	1.00	7119	5.67E-27	GCST90277394
Lysophosphatidylcholine (18:0) levels	12	20960665	T	C	4.66E-01	1.02E-01	7.25E-03	rs117428564	12_20960665_C_T	0.97	7119	5.38E-06	GCST90277394
Lysophosphatidylcholine (18:0) levels	12	96850670	C	A	-3.73E-01	7.99E-02	1.12E-02	rs139500046	12_96850670_A_C	0.99	7119	3.04E-06	GCST90277394
Lysophosphatidylcholine (18:0) levels	12	108520819	A	G	1.43E-01	3.17E-02	7.60E-02	rs1047974	12_108520819_G_A	1.00	7119	6.08E-06	GCST90277394
Lysophosphatidylcholine (18:0) levels	14	29823442	G	A	-2.05E-01	4.49E-02	3.72E-02	rs10147474	14_29823442_A_G	0.98	7119	4.99E-06	GCST90277394
Lysophosphatidylcholine (18:0) levels	14	76971123	A	G	1.25E-01	2.74E-02	1.05E-01	rs111526079	14_76971123_G_A	0.96	7119	5.14E-06	GCST90277394
Lysophosphatidylcholine (18:0) levels	17	66543378	A	T	7.73E-02	1.70E-02	4.32E-01	rs4791050	17_66543378_T_A	0.99	7119	5.36E-06	GCST90277394
Lysophosphatidylcholine (18:0) levels	19	19269704	G	A	-2.26E-01	3.80E-02	5.35E-02	rs187429064	19_19269704_A_G	0.95	7119	3.17E-09	GCST90277394
Lysophosphatidylcholine (18:0) levels	19	19547663	T	G	-1.48E-01	3.35E-02	6.58E-02	rs16996148	19_19547663_G_T	1.00	7119	9.94E-06	GCST90277394
Lysophosphatidylcholine (18:0) levels	19	44913574	G	T	1.51E-01	3.13E-02	7.73E-02	rs390082	19_44913574_T_G	1.00	7119	1.51E-06	GCST90277394
Lysophosphatidylcholine (18:1) levels	1	170382059	C	G	1.01E-01	2.24E-02	2.50E-01	rs2151252	1_170382059_G_C	1.00	5237	7.12E-06	GCST90277395
Lysophosphatidylcholine (18:1) levels	2	27508073	C	T	-1.35E-01	2.03E-02	6.51E-01	rs1260326	2_27508073_T_C	1.00	5237	3.13E-11	GCST90277395
Lysophosphatidylcholine (18:1) levels	3	55269076	G	A	3.38E-01	6.95E-02	1.98E-02	rs149992851	3_55269076_A_G	0.98	5237	1.17E-06	GCST90277395
Lysophosphatidylcholine (18:1) levels	3	66778832	G	A	-1.27E-01	2.67E-02	1.64E-01	rs966039	3_66778832_A_G	0.98	5237	2.09E-06	GCST90277395
Lysophosphatidylcholine (18:1) levels	3	188269372	C	T	1.63E-01	3.61E-02	7.75E-02	rs76308170	3_188269372_T_C	0.97	5237	6.45E-06	GCST90277395
Lysophosphatidylcholine (18:1) levels	4	4996200	T	C	1.19E-01	2.44E-02	2.14E-01	rs62291572	4_4996200_C_T	0.94	5237	1.05E-06	GCST90277395
Lysophosphatidylcholine (18:1) levels	4	86536048	T	C	2.62E-01	5.68E-02	3.01E-02	rs71605604	4_86536048_C_T	0.97	5237	4.16E-06	GCST90277395
Lysophosphatidylcholine (18:1) levels	6	31297960	G	A	9.53E-02	1.95E-02	5.05E-01	rs2853923	6_31297960_A_G	1.00	5237	1.03E-06	GCST90277395
Lysophosphatidylcholine (18:1) levels	6	164722387	C	A	1.08E-01	2.41E-02	7.94E-01	rs4709886	6_164722387_A_C	1.00	5237	7.67E-06	GCST90277395
Lysophosphatidylcholine (18:1) levels	8	4633421	C	T	-2.00E-01	4.21E-02	5.90E-02	rs11782705	8_4633421_T_C	0.98	5237	2.02E-06	GCST90277395
Lysophosphatidylcholine (18:1) levels	9	112114209	A	G	9.55E-01	1.92E-01	3.23E-03	rs116923150	9_112114209_G_A	0.90	5237	6.84E-07	GCST90277395
Lysophosphatidylcholine (18:1) levels	10	17398917	T	G	-7.78E-01	1.74E-01	3.05E-03	rs10159864	10_17398917_G_T	0.97	5237	7.89E-06	GCST90277395
Lysophosphatidylcholine (18:1) levels	10	71191707	C	T	-5.47E-01	1.19E-01	7.85E-03	rs182295482	10_71191707_T_C	0.86	5237	4.54E-06	GCST90277395
Lysophosphatidylcholine (18:1) levels	11	116778201	C	G	-2.40E-01	2.62E-02	8.49E-01	rs964184	11_116778201_G_C	1.00	5237	6.65E-20	GCST90277395
Lysophosphatidylcholine (18:1) levels	11	117173887	G	C	-1.90E-01	4.22E-02	5.84E-02	rs7112577	11_117173887_C_G	1.00	5237	7.28E-06	GCST90277395
Lysophosphatidylcholine (18:1) levels	13	24038710	T	A	-9.71E-01	1.97E-01	2.36E-03	rs112421492	13_24038710_A_T	0.92	5237	8.22E-07	GCST90277395
Lysophosphatidylcholine (18:1) levels	13	105939568	G	A	1.10E-01	2.38E-02	2.09E-01	rs17514333	13_105939568_A_G	1.00	5237	3.41E-06	GCST90277395
Lysophosphatidylcholine (18:1) levels	15	59004639	C	T	-3.28E-01	7.06E-02	1.93E-02	rs62002515	15_59004639_T_C	0.99	5237	3.46E-06	GCST90277395
Lysophosphatidylcholine (18:1) levels	19	13789855	A	G	-1.25E-01	2.66E-02	1.71E-01	rs62111901	19_13789855_G_A	0.95	5237	2.57E-06	GCST90277395
Lysophosphatidylcholine (18:1) levels	19	33235567	A	G	-1.34E-01	2.96E-02	1.34E-01	rs78327363	19_33235567_G_A	0.93	5237	6.34E-06	GCST90277395
Lysophosphatidylcholine (18:1) levels	19	44913484	T	C	1.15E-01	2.29E-02	2.42E-01	rs438811	19_44913484_C_T	1.00	5237	5.09E-07	GCST90277395
Lysophosphatidylcholine (18:2) levels	1	103243167	T	C	1.50E-01	3.22E-02	7.72E-02	rs144241030	1_103243167_C_T	0.94	7168	3.26E-06	GCST90277396
Lysophosphatidylcholine (18:2) levels	1	196028959	T	C	3.50E-01	7.53E-02	1.38E-02	rs78275146	1_196028959_C_T	0.92	7168	3.49E-06	GCST90277396
Lysophosphatidylcholine (18:2) levels	1	220806736	A	C	1.08E-01	2.16E-02	1.85E-01	rs34621709	1_220806736_C_A	1.00	7168	5.87E-07	GCST90277396
Lysophosphatidylcholine (18:2) levels	2	21002409	T	C	9.03E-02	1.86E-02	7.28E-01	rs1042034	2_21002409_C_T	1.00	7168	1.28E-06	GCST90277396
Lysophosphatidylcholine (18:2) levels	2	27508073	C	T	-1.22E-01	1.74E-02	6.51E-01	rs1260326	2_27508073_T_C	1.00	7168	2.79E-12	GCST90277396
Lysophosphatidylcholine (18:2) levels	2	36549412	T	G	-2.37E-01	5.09E-02	2.77E-02	rs3770813	2_36549412_G_T	1.00	7168	3.47E-06	GCST90277396
Lysophosphatidylcholine (18:2) levels	2	104244849	A	T	-2.61E-01	5.65E-02	2.27E-02	rs147987331	2_104244849_T_A	0.97	7168	3.96E-06	GCST90277396
Lysophosphatidylcholine (18:2) levels	2	128427075	G	A	1.54E-01	3.20E-02	7.45E-02	rs76950187	2_128427075_A_G	0.99	7168	1.54E-06	GCST90277396
Lysophosphatidylcholine (18:2) levels	2	212010621	G	A	-8.41E-02	1.79E-02	6.76E-01	rs10169217	2_212010621_A_G	1.00	7168	2.59E-06	GCST90277396
Lysophosphatidylcholine (18:2) levels	4	4996200	T	C	9.54E-02	2.10E-02	2.14E-01	rs62291572	4_4996200_C_T	0.94	7168	5.90E-06	GCST90277396

Lysophosphatidylcholine (18:2) levels	5	6523892	G	A	7.44E-02	1.67E-02	4.25E-01	rs501474	5_6523892_A_G	1.00	7168	8.81E-06	GCST90277396
Lysophosphatidylcholine (18:2) levels	5	83807154	A	G	2.38E-01	5.23E-02	2.62E-02	rs150741556	5_83807154_G_A	0.98	7168	5.63E-06	GCST90277396
Lysophosphatidylcholine (18:2) levels	6	23919006	G	A	-6.44E-01	1.37E-01	4.04E-03	rs79934453	6_23919006_A_G	0.91	7168	2.67E-06	GCST90277396
Lysophosphatidylcholine (18:2) levels	6	49123122	A	C	-4.43E-01	9.57E-02	8.14E-03	rs79954170	6_49123122_C_A	0.96	7168	3.85E-06	GCST90277396
Lysophosphatidylcholine (18:2) levels	7	73597712	A	G	-1.41E-01	2.54E-02	1.22E-01	rs35332062	7_73597712_G_A	1.00	7168	3.06E-08	GCST90277396
Lysophosphatidylcholine (18:2) levels	8	19967156	T	C	-1.06E-01	1.89E-02	2.61E-01	rs15285	8_19967156_C_T	1.00	7168	2.15E-08	GCST90277396
Lysophosphatidylcholine (18:2) levels	8	59089983	T	C	9.65E-02	2.08E-02	1.98E-01	rs1010313	8_59089983_C_T	1.00	7168	3.42E-06	GCST90277396
Lysophosphatidylcholine (18:2) levels	8	125495066	C	T	-8.70E-02	1.91E-02	7.43E-01	rs2980888	8_125495066_T_C	1.00	7168	5.32E-06	GCST90277396
Lysophosphatidylcholine (18:2) levels	9	120350676	T	C	2.06E-01	4.47E-02	3.92E-02	rs72753379	9_120350676_C_T	0.95	7168	4.08E-06	GCST90277396
Lysophosphatidylcholine (18:2) levels	9	136257994	G	A	-2.39E-01	4.85E-02	3.60E-02	rs112122274	9_136257994_A_G	0.85	7168	8.47E-07	GCST90277396
Lysophosphatidylcholine (18:2) levels	10	62375635	CA	C	-2.57E-01	5.63E-02	2.27E-02	rs35162925	10_62375635_C_CA	1.00	7168	5.24E-06	GCST90277396
Lysophosphatidylcholine (18:2) levels	10	131254190	C	T	-1.76E-01	3.96E-02	4.73E-02	rs11017845	10_131254190_T_C	0.99	7168	9.65E-06	GCST90277396
Lysophosphatidylcholine (18:2) levels	11	116778201	C	G	-2.48E-01	2.30E-02	8.49E-01	rs964184	11_116778201_G_C	1.00	7168	7.83E-27	GCST90277396
Lysophosphatidylcholine (18:2) levels	14	29823442	G	A	-2.16E-01	4.47E-02	3.72E-02	rs10147474	14_29823442_A_G	0.98	7168	1.32E-06	GCST90277396
Lysophosphatidylcholine (20:4) levels	1	8199214	C	T	-8.18E-02	1.85E-02	2.83E-01	rs55724405	1_8199214_T_C	1.00	7173	9.56E-06	GCST90277397
Lysophosphatidylcholine (20:4) levels	1	220806736	A	C	1.12E-01	2.15E-02	1.85E-01	rs34621709	1_220806736_C_A	1.00	7173	2.27E-07	GCST90277397
Lysophosphatidylcholine (20:4) levels	1	230156121	C	T	-1.14E-01	1.96E-02	7.69E-01	rs2352723	1_230156121_T_C	1.00	7173	5.93E-09	GCST90277397
Lysophosphatidylcholine (20:4) levels	2	21002409	T	C	1.10E-01	1.86E-02	7.28E-01	rs1042034	2_21002409_C_T	1.00	7173	3.90E-09	GCST90277397
Lysophosphatidylcholine (20:4) levels	2	27508073	C	T	-1.37E-01	1.74E-02	6.51E-01	rs1260326	2_27508073_T_C	1.00	7173	4.16E-15	GCST90277397
Lysophosphatidylcholine (20:4) levels	2	104244849	A	T	-2.60E-01	5.65E-02	2.27E-02	rs147987331	2_104244849_T_A	0.97	7173	4.27E-06	GCST90277397
Lysophosphatidylcholine (20:4) levels	3	87298641	A	G	-1.76E-01	3.92E-02	4.88E-02	rs116011373	3_87298641_G_A	0.96	7173	7.27E-06	GCST90277397
Lysophosphatidylcholine (20:4) levels	3	171536246	A	G	-4.99E-01	1.07E-01	7.31E-03	rs57500102	3_171536246_G_A	0.92	7173	3.10E-06	GCST90277397
Lysophosphatidylcholine (20:4) levels	3	189123018	A	G	2.09E-01	4.63E-02	3.45E-02	rs76078494	3_189123018_G_A	0.96	7173	6.73E-06	GCST90277397
Lysophosphatidylcholine (20:4) levels	4	55360008	T	C	7.48E-02	1.68E-02	4.67E-01	rs6554273	4_55360008_C_T	0.99	7173	8.50E-06	GCST90277397
Lysophosphatidylcholine (20:4) levels	4	113004554	C	G	-8.10E-02	1.72E-02	6.13E-01	rs9997195	4_113004554_G_C	1.00	7173	2.61E-06	GCST90277397
Lysophosphatidylcholine (20:4) levels	4	141550641	A	G	-1.35E-01	3.04E-02	8.41E-02	rs144313866	4_141550641_G_A	0.96	7173	8.80E-06	GCST90277397
Lysophosphatidylcholine (20:4) levels	6	49123122	A	C	-5.11E-01	9.56E-02	8.14E-03	rs79954170	6_49123122_C_A	0.96	7173	9.54E-08	GCST90277397
Lysophosphatidylcholine (20:4) levels	6	154916245	C	T	1.57E-01	3.36E-02	6.82E-02	rs144072769	6_154916245_T_C	0.96	7173	2.84E-06	GCST90277397
Lysophosphatidylcholine (20:4) levels	7	73597712	A	G	-1.60E-01	2.54E-02	1.22E-01	rs35332062	7_73597712_G_A	1.00	7173	3.11E-10	GCST90277397
Lysophosphatidylcholine (20:4) levels	8	19970337	A	C	-1.29E-01	1.85E-02	2.79E-01	rs10105606	8_19970337_C_A	1.00	7173	2.84E-12	GCST90277397
Lysophosphatidylcholine (20:4) levels	8	59089983	T	C	1.03E-01	2.07E-02	1.98E-01	rs1010313	8_59089983_C_T	1.00	7173	6.95E-07	GCST90277397
Lysophosphatidylcholine (20:4) levels	8	125495066	C	T	-8.86E-02	1.91E-02	7.43E-01	rs2980888	8_125495066_T_C	1.00	7173	3.51E-06	GCST90277397
Lysophosphatidylcholine (20:4) levels	10	59062519	A	G	-4.57E-01	1.03E-01	8.06E-03	rs16913024	10_59062519_G_A	0.85	7173	9.81E-06	GCST90277397
Lysophosphatidylcholine (20:4) levels	10	112142696	T	C	-8.09E-02	1.82E-02	2.97E-01	rs72836628	10_112142696_C_T	1.00	7173	9.11E-06	GCST90277397
Lysophosphatidylcholine (20:4) levels	11	61814292	C	T	7.68E-02	1.73E-02	3.83E-01	rs174560	11_61814292_T_C	1.00	7173	8.73E-06	GCST90277397
Lysophosphatidylcholine (20:4) levels	11	116618319	G	A	1.12E-01	2.26E-02	1.66E-01	rs12365864	11_116618319_A_G	0.97	7173	6.71E-07	GCST90277397
Lysophosphatidylcholine (20:4) levels	11	116778201	C	G	-2.99E-01	2.29E-02	8.49E-01	rs964184	11_116778201_G_C	1.00	7173	2.31E-38	GCST90277397
Lysophosphatidylcholine (20:4) levels	13	21038010	T	C	-1.38E-01	3.04E-02	8.45E-02	rs75573052	13_21038010_C_T	0.99	7173	5.67E-06	GCST90277397
Lysophosphatidylcholine (20:4) levels	14	95218464	A	G	-8.35E-02	1.87E-02	2.75E-01	rs61702480	14_95218464_G_A	0.99	7173	8.15E-06	GCST90277397
Lysophosphatidylcholine (20:4) levels	17	1623049	C	T	9.47E-02	2.14E-02	1.96E-01	rs35986054	17_1623049_T_C	0.98	7173	9.56E-06	GCST90277397
Lysophosphatidylcholine (20:4) levels	17	72177274	C	G	-2.16E-01	4.73E-02	3.46E-02	rs75237020	17_72177274_G_C	0.90	7173	4.96E-06	GCST90277397
Lysophosphatidylcholine (20:4) levels	19	8364439	A	G	-2.41E-01	5.14E-02	2.59E-02	rs116843064	19_8364439_G_A	1.00	7173	2.70E-06	GCST90277397
Lysophosphatidylcholine (20:4) levels	19	19269704	G	A	-1.85E-01	3.78E-02	5.35E-02	rs187429064	19_19269704_A_G	0.95	7173	9.80E-07	GCST90277397
Lysophosphatidylcholine (20:4) levels	22	32729795	T	C	1.77E-01	3.79E-02	5.12E-02	rs117950471	22_32729795_C_T	0.97	7173	3.24E-06	GCST90277397
Lysophosphatidylethanolamine (18:0) levels	1	220806736	A	C	1.10E-01	2.15E-02	1.85E-01	rs34621709	1_220806736_C_A	1.00	7174	3.33E-07	GCST90277398
Lysophosphatidylethanolamine (18:0) levels	1	230167404	T	C	-1.17E-01	1.99E-02	7.77E-01	rs10779836	1_230167404_C_T	1.00	7174	4.90E-09	GCST90277398
Lysophosphatidylethanolamine (18:0) levels	2	21002409	T	C	1.06E-01	1.86E-02	7.28E-01	rs1042034	2_21002409_C_T	1.00	7174	1.28E-08	GCST90277398
Lysophosphatidylethanolamine (18:0) levels	2	27508073	C	T	-1.39E-01	1.74E-02	6.51E-01	rs1260326	2_27508073_T_C	1.00	7174	1.80E-15	GCST90277398
Lysophosphatidylethanolamine (18:0) levels	2	212061574	C	T	2.57E-01	5.81E-02	2.18E-02	rs76330538	2_212061574_T_C	0.98	7174	9.76E-06	GCST90277398
Lysophosphatidylethanolamine (18:0) levels	3	87180483	T	G	-1.83E-01	3.91E-02	4.98E-02	rs116497138	3_87180483_G_T	0.94	7174	3.01E-06	GCST90277398
Lysophosphatidylethanolamine (18:0) levels	4	99807593	T	C	-2.20E-01	4.96E-02	2.96E-02	rs56376607	4_99807593_C_T	0.96	7174	9.24E-06	GCST90277398
Lysophosphatidylethanolamine (18:0) levels	6	49123122	A	C	-4.66E-01	9.57E-02	8.14E-03	rs79954170	6_49123122_C_A	0.96	7174	1.15E-06	GCST90277398
Lysophosphatidylethanolamine (18:0) levels	6	64177173	T	C	7.84E-02	1.72E-02	3.73E-01	rs9344829	6_64177173_C_T	0.99	7174	5.25E-06	GCST90277398
Lysophosphatidylethanolamine (18:0) levels	6	154916245	C	T	1.63E-01	3.36E-02	6.82E-02	rs144072769	6_154916245_T_C	0.96	7174	1.17E-06	GCST90277398
Lysophosphatidylethanolamine (18:0) levels	7	73598455	G	A	-1.41E-01	2.54E-02	1.22E-01	rs79624003	7_73598455_A_G	1.00	7174	2.70E-08	GCST90277398
Lysophosphatidylethanolamine (18:0) levels	8	764450	G	A	-9.53E-02	2.11E-02	2.00E-01	rs11783556	8_764450_A_G	0.99	7174	6.06E-06	GCST90277398
Lysophosphatidylethanolamine (18:0) levels	8	19970337	A	C	-1.25E-01	1.85E-02	2.79E-01	rs10105606	8_19970337_C_A	1.00	7174	1.44E-11	GCST90277398

Lysophosphatidylethanolamine (18:0) levels	8	59090254	A	G	9.88E-02	2.08E-02	1.97E-01	rs13259370	8_59090254_G_A	1.00	7174	2.11E-06	GCST90277398
Lysophosphatidylethanolamine (18:0) levels	8	97226039	G	A	-7.44E-02	1.66E-02	5.31E-01	rs13261274	8_97226039_A_G	1.00	7174	7.82E-06	GCST90277398
Lysophosphatidylethanolamine (18:0) levels	10	101966464	C	A	1.60E-01	3.62E-02	5.48E-02	rs142146849	10_101966464_A_C	1.00	7174	9.83E-06	GCST90277398
Lysophosphatidylethanolamine (18:0) levels	11	45826596	A	G	-2.61E-01	5.76E-02	2.31E-02	rs72900657	11_45826596_G_A	0.92	7174	6.06E-06	GCST90277398
Lysophosphatidylethanolamine (18:0) levels	11	116618319	G	A	1.05E-01	2.26E-02	1.66E-01	rs12365864	11_116618319_A_G	0.97	7174	3.23E-06	GCST90277398
Lysophosphatidylethanolamine (18:0) levels	11	116778201	C	G	-2.84E-01	2.30E-02	8.49E-01	rs964184	11_116778201_G_C	1.00	7174	6.53E-35	GCST90277398
Lysophosphatidylethanolamine (18:0) levels	12	129796667	A	G	-8.16E-02	1.80E-02	6.83E-01	rs10773708	12_129796667_G_A	1.00	7174	6.20E-06	GCST90277398
Lysophosphatidylethanolamine (18:0) levels	14	95218464	A	G	-8.30E-02	1.87E-02	2.75E-01	rs61702480	14_95218464_G_A	0.99	7174	9.36E-06	GCST90277398
Lysophosphatidylethanolamine (18:0) levels	17	1615646	G	A	8.84E-02	1.85E-02	3.02E-01	rs56051325	17_1615646_A_G	0.97	7174	1.72E-06	GCST90277398
Lysophosphatidylethanolamine (18:0) levels	17	49154037	GCT	G	1.14E-01	2.52E-02	1.21E-01	rs61156392	17_49154037_G_GCT	0.98	7174	6.83E-06	GCST90277398
Lysophosphatidylethanolamine (18:0) levels	19	19269704	G	A	-2.06E-01	3.78E-02	5.35E-02	rs187429064	19_19269704_A_G	0.95	7174	4.86E-08	GCST90277398
Lysophosphatidylethanolamine (18:0) levels	19	33135815	C	G	1.39E-01	3.11E-02	7.80E-02	rs113672231	19_33135815_G_C	0.98	7174	7.46E-06	GCST90277398
Lysophosphatidylethanolamine (18:0) levels	19	44892962	T	C	9.50E-02	2.01E-02	2.23E-01	rs157582	19_44892962_C_T	1.00	7174	2.29E-06	GCST90277398
Lysophosphatidylethanolamine (18:1) levels	1	220805303	T	C	1.10E-01	2.34E-02	1.52E-01	rs17596144	1_220805303_C_T	0.99	7172	2.60E-06	GCST90277399
Lysophosphatidylethanolamine (18:1) levels	1	230167404	T	C	-9.43E-02	1.99E-02	7.77E-01	rs10779836	1_230167404_C_T	1.00	7172	2.30E-06	GCST90277399
Lysophosphatidylethanolamine (18:1) levels	2	21002409	T	C	9.04E-02	1.86E-02	7.28E-01	rs1042034	2_21002409_C_T	1.00	7172	1.22E-06	GCST90277399
Lysophosphatidylethanolamine (18:1) levels	2	27508073	C	T	-1.54E-01	1.74E-02	6.51E-01	rs1260326	2_27508073_T_C	1.00	7172	1.01E-18	GCST90277399
Lysophosphatidylethanolamine (18:1) levels	2	42239140	G	C	-8.24E-02	1.85E-02	2.84E-01	rs6753694	2_42239140_C_G	1.00	7172	8.14E-06	GCST90277399
Lysophosphatidylethanolamine (18:1) levels	3	11749984	G	A	8.15E-02	1.77E-02	3.40E-01	rs301555	3_11749984_A_G	0.98	7172	4.18E-06	GCST90277399
Lysophosphatidylethanolamine (18:1) levels	3	87180483	T	G	-1.74E-01	3.91E-02	4.98E-02	rs116497138	3_87180483_G_T	0.94	7172	8.43E-06	GCST90277399
Lysophosphatidylethanolamine (18:1) levels	6	64177173	T	C	8.00E-02	1.72E-02	3.73E-01	rs9344829	6_64177173_C_T	0.99	7172	3.41E-06	GCST90277399
Lysophosphatidylethanolamine (18:1) levels	6	154916245	C	T	1.57E-01	3.36E-02	6.82E-02	rs144072769	6_154916245_T_C	0.96	7172	2.86E-06	GCST90277399
Lysophosphatidylethanolamine (18:1) levels	7	73615107	T	C	-1.13E-01	2.54E-02	1.22E-01	rs33951980	7_73615107_C_T	1.00	7172	8.70E-06	GCST90277399
Lysophosphatidylethanolamine (18:1) levels	8	764450	G	A	-9.84E-02	2.11E-02	2.00E-01	rs11783556	8_764450_A_G	0.99	7172	3.05E-06	GCST90277399
Lysophosphatidylethanolamine (18:1) levels	8	19970337	A	C	-1.12E-01	1.85E-02	2.79E-01	rs10105606	8_19970337_C_A	1.00	7172	1.58E-09	GCST90277399
Lysophosphatidylethanolamine (18:1) levels	8	59090254	A	G	9.31E-02	2.08E-02	1.97E-01	rs13259370	8_59090254_G_A	1.00	7172	7.84E-06	GCST90277399
Lysophosphatidylethanolamine (18:1) levels	8	100382190	G	T	8.43E-02	1.90E-02	2.68E-01	rs4529433	8_100382190_T_G	0.99	7172	9.02E-06	GCST90277399
Lysophosphatidylethanolamine (18:1) levels	9	132470502	G	A	2.41E-01	5.45E-02	2.59E-02	rs111568723	9_132470502_A_G	0.95	7172	1.00E-05	GCST90277399
Lysophosphatidylethanolamine (18:1) levels	10	7871139	C	T	4.28E-01	8.80E-02	9.45E-03	rs190411877	10_7871139_T_C	0.94	7172	1.15E-06	GCST90277399
Lysophosphatidylethanolamine (18:1) levels	10	101966464	C	A	1.71E-01	3.62E-02	5.48E-02	rs142146849	10_101966464_A_C	1.00	7172	2.46E-06	GCST90277399
Lysophosphatidylethanolamine (18:1) levels	11	45826596	A	G	-2.73E-01	5.76E-02	2.31E-02	rs72900657	11_45826596_G_A	0.92	7172	2.21E-06	GCST90277399
Lysophosphatidylethanolamine (18:1) levels	11	116778201	C	G	-2.51E-01	2.30E-02	8.49E-01	rs964184	11_116778201_G_C	1.00	7172	1.59E-27	GCST90277399
Lysophosphatidylethanolamine (18:1) levels	13	50548374	T	C	8.60E-02	1.93E-02	2.50E-01	rs7996305	13_50548374_C_T	1.00	7172	7.99E-06	GCST90277399
Lysophosphatidylethanolamine (18:1) levels	17	1623049	C	T	1.00E-01	2.14E-02	1.96E-01	rs35986054	17_1623049_T_C	0.98	7172	2.77E-06	GCST90277399
Lysophosphatidylethanolamine (18:1) levels	17	49154037	GCT	G	1.19E-01	2.53E-02	1.21E-01	rs61156392	17_49154037_G_GCT	0.98	7172	2.54E-06	GCST90277399
Lysophosphatidylethanolamine (18:1) levels	17	79406653	G	A	-1.21E-01	2.68E-02	1.28E-01	rs75560495	17_79406653_A_G	0.88	7172	6.03E-06	GCST90277399
Lysophosphatidylethanolamine (18:1) levels	19	17375221	A	G	1.78E-01	3.97E-02	4.90E-02	rs143467207	19_17375221_G_A	0.96	7172	7.42E-06	GCST90277399
Lysophosphatidylethanolamine (18:1) levels	19	19269704	G	A	-2.41E-01	3.78E-02	5.35E-02	rs187429064	19_19269704_A_G	0.95	7172	1.76E-10	GCST90277399
Lysophosphatidylethanolamine (18:1) levels	19	33135815	C	G	1.42E-01	3.11E-02	7.80E-02	rs113672231	19_33135815_G_C	0.98	7172	4.95E-06	GCST90277399
Lysophosphatidylethanolamine (18:1) levels	19	44892962	T	C	9.40E-02	2.01E-02	2.23E-01	rs157582	19_44892962_C_T	1.00	7172	2.92E-06	GCST90277399
Lysophosphatidylethanolamine (18:2) levels	1	237378917	T	C	-3.06E-01	6.44E-02	1.99E-02	rs78095833	1_237378917_C_T	0.90	6554	2.01E-06	GCST90277400
Lysophosphatidylethanolamine (18:2) levels	2	27508073	C	T	-1.32E-01	1.82E-02	6.51E-01	rs1260326	2_27508073_T_C	1.00	6554	4.04E-13	GCST90277400
Lysophosphatidylethanolamine (18:2) levels	2	45482958	T	C	-1.04E-01	2.31E-02	8.26E-01	rs6752909	2_45482958_C_T	1.00	6554	7.19E-06	GCST90277400
Lysophosphatidylethanolamine (18:2) levels	2	193027049	A	C	4.13E-01	9.19E-02	9.62E-03	rs114314612	2_193027049_C_A	0.93	6554	6.97E-06	GCST90277400
Lysophosphatidylethanolamine (18:2) levels	3	37387931	G	A	-8.71E-02	1.82E-02	3.65E-01	rs4508726	3_37387931_A_G	1.00	6554	1.77E-06	GCST90277400
Lysophosphatidylethanolamine (18:2) levels	3	133895761	T	C	1.12E-01	2.34E-02	1.73E-01	rs117518401	3_133895761_C_T	0.98	6554	1.70E-06	GCST90277400
Lysophosphatidylethanolamine (18:2) levels	4	179015062	C	T	-3.13E-01	6.52E-02	1.96E-02	rs78178542	4_179015062_T_C	0.92	6554	1.59E-06	GCST90277400
Lysophosphatidylethanolamine (18:2) levels	4	180460689	A	G	8.13E-02	1.73E-02	4.59E-01	rs1017216	4_180460689_G_A	1.00	6554	2.73E-06	GCST90277400
Lysophosphatidylethanolamine (18:2) levels	7	71530474	A	G	4.28E-01	9.25E-02	9.28E-03	rs56257715	7_71530474_G_A	0.91	6554	3.70E-06	GCST90277400
Lysophosphatidylethanolamine (18:2) levels	8	19970337	A	C	-9.48E-02	1.93E-02	2.79E-01	rs10105606	8_19970337_C_A	1.00	6554	9.51E-07	GCST90277400
Lysophosphatidylethanolamine (18:2) levels	8	64695903	G	A	9.80E-02	2.15E-02	7.92E-01	rs7815372	8_64695903_A_G	1.00	6554	5.52E-06	GCST90277400
Lysophosphatidylethanolamine (18:2) levels	9	79867479	T	C	-2.64E-01	5.87E-02	2.23E-02	rs117082735	9_79867479_C_T	0.98	6554	6.72E-06	GCST90277400
Lysophosphatidylethanolamine (18:2) levels	10	94617201	T	A	-3.84E-01	8.57E-02	1.03E-02	rs140620869	10_94617201_A_T	0.99	6554	7.67E-06	GCST90277400
Lysophosphatidylethanolamine (18:2) levels	11	61790354	C	T	-1.32E-01	1.78E-02	4.09E-01	rs102274	11_61790354_T_C	1.00	6554	1.00E-13	GCST90277400
Lysophosphatidylethanolamine (18:2) levels	11	89627134	C	G	-1.90E-01	4.01E-02	4.98E-02	rs61905503	11_89627134_G_C	0.99	6554	2.23E-06	GCST90277400
Lysophosphatidylethanolamine (18:2) levels	11	116778201	C	G	-2.03E-01	2.41E-02	8.49E-01	rs964184	11_116778201_G_C	1.00	6554	3.70E-17	GCST90277400
Lysophosphatidylethanolamine (18:2) levels	13	25886443	T	C	-5.76E-01	1.26E-01	5.33E-03	rs112578388	13_25886443_C_T	0.86	6554	5.22E-06	GCST90277400

Lysophosphatidylethanolamine (18:2) levels	13	51065337	A	T	-1.28E-01	2.65E-02	1.24E-01	rs9591384	13_51065337_T_A	1.00	6554	1.25E-06	GCST90277400
Lysophosphatidylethanolamine (18:2) levels	17	49154037	GCT	G	1.20E-01	2.64E-02	1.21E-01	rs61156392	17_49154037_G_GCT	0.98	6554	6.02E-06	GCST90277400
Lysophosphatidylethanolamine (18:2) levels	18	79621226	G	A	-8.15E-02	1.81E-02	3.73E-01	rs7232381	18_79621226_A_G	0.99	6554	6.68E-06	GCST90277400
Lysophosphatidylethanolamine (18:2) levels	19	17375221	A	G	1.85E-01	4.17E-02	4.90E-02	rs143467207	19_17375221_G_A	0.96	6554	9.39E-06	GCST90277400
Lysophosphatidylethanolamine (18:2) levels	19	19347579	G	A	-2.75E-01	4.00E-02	5.38E-02	rs182611493	19_19347579_A_G	0.95	6554	6.08E-12	GCST90277400
Lysophosphatidylethanolamine (18:2) levels	19	34971620	G	A	-7.98E-02	1.73E-02	5.33E-01	rs2546044	19_34971620_A_G	0.99	6554	4.10E-06	GCST90277400
Lysophosphatidylethanolamine (18:2) levels	19	44912383	A	G	1.58E-01	3.24E-02	7.74E-02	rs445925	19_44912383_G_A	1.00	6554	1.15E-06	GCST90277400
Phosphatidylcholine (14:0_16:0) levels	1	27100305	C	T	1.65E-01	3.67E-02	9.41E-01	rs4266911	1_27100305_T_C	0.94	6996	6.96E-06	GCST90277401
Phosphatidylcholine (14:0_16:0) levels	1	220806736	A	C	1.13E-01	2.18E-02	1.85E-01	rs34621709	1_220806736_C_A	1.00	6996	2.40E-07	GCST90277401
Phosphatidylcholine (14:0_16:0) levels	2	21002409	T	C	8.37E-02	1.89E-02	7.28E-01	rs1042034	2_21002409_C_T	1.00	6996	9.40E-06	GCST90277401
Phosphatidylcholine (14:0_16:0) levels	2	27508073	C	T	-1.19E-01	1.76E-02	6.51E-01	rs1260326	2_27508073_T_C	1.00	6996	1.73E-11	GCST90277401
Phosphatidylcholine (14:0_16:0) levels	2	128427075	G	A	1.57E-01	3.23E-02	7.45E-02	rs76950187	2_128427075_A_G	0.99	6996	1.17E-06	GCST90277401
Phosphatidylcholine (14:0_16:0) levels	3	1895465	G	A	7.58E-02	1.69E-02	5.60E-01	rs12635725	3_1895465_A_G	0.99	6996	7.41E-06	GCST90277401
Phosphatidylcholine (14:0_16:0) levels	3	87180483	T	G	-1.84E-01	3.97E-02	4.98E-02	rs116497138	3_87180483_G_T	0.94	6996	3.54E-06	GCST90277401
Phosphatidylcholine (14:0_16:0) levels	3	134995496	G	A	-8.34E-02	1.87E-02	2.84E-01	rs11926107	3_134995496_A_G	1.00	6996	8.32E-06	GCST90277401
Phosphatidylcholine (14:0_16:0) levels	3	168112642	C	T	-2.22E-01	4.99E-02	3.27E-02	rs111241638	3_168112642_T_C	0.91	6996	8.97E-06	GCST90277401
Phosphatidylcholine (14:0_16:0) levels	3	177504180	G	C	9.06E-02	2.03E-02	2.25E-01	rs55870249	3_177504180_C_G	1.00	6996	7.99E-06	GCST90277401
Phosphatidylcholine (14:0_16:0) levels	4	4996200	T	C	1.03E-01	2.12E-02	2.14E-01	rs62291572	4_4996200_C_T	0.94	6996	1.20E-06	GCST90277401
Phosphatidylcholine (14:0_16:0) levels	4	110201006	G	A	9.18E-02	1.93E-02	2.70E-01	rs72679249	4_110201006_A_G	0.97	6996	2.06E-06	GCST90277401
Phosphatidylcholine (14:0_16:0) levels	5	83807154	A	G	2.35E-01	5.27E-02	2.62E-02	rs150741556	5_83807154_G_A	0.98	6996	8.45E-06	GCST90277401
Phosphatidylcholine (14:0_16:0) levels	7	1998126	A	G	-8.84E-02	1.81E-02	3.20E-01	rs34021847	7_1998126_G_A	0.99	6996	1.03E-06	GCST90277401
Phosphatidylcholine (14:0_16:0) levels	7	73597712	A	G	-1.34E-01	2.59E-02	1.22E-01	rs35332062	7_73597712_G_A	1.00	6996	2.21E-07	GCST90277401
Phosphatidylcholine (14:0_16:0) levels	8	19967156	T	C	-9.72E-02	1.92E-02	2.61E-01	rs15285	8_19967156_C_T	1.00	6996	4.10E-07	GCST90277401
Phosphatidylcholine (14:0_16:0) levels	11	61813163	T	C	9.52E-02	1.75E-02	3.80E-01	rs174556	11_61813163_C_T	1.00	6996	5.17E-08	GCST90277401
Phosphatidylcholine (14:0_16:0) levels	11	116778201	C	G	-2.33E-01	2.33E-02	8.49E-01	rs964184	11_116778201_G_C	1.00	6996	2.38E-23	GCST90277401
Phosphatidylcholine (14:0_16:0) levels	13	21038010	T	C	-1.42E-01	3.09E-02	8.45E-02	rs75573052	13_21038010_C_T	0.99	6996	4.79E-06	GCST90277401
Phosphatidylcholine (14:0_16:0) levels	14	29823442	G	A	-2.19E-01	4.54E-02	3.72E-02	rs10147474	14_29823442_A_G	0.98	6996	1.54E-06	GCST90277401
Phosphatidylcholine (14:0_16:0) levels	15	93154154	A	C	-9.06E-02	1.70E-02	4.18E-01	rs62043594	15_93154154_C_A	1.00	6996	9.63E-08	GCST90277401
Phosphatidylcholine (14:0_16:0) levels	19	44913574	G	T	1.44E-01	3.16E-02	7.73E-02	rs390082	19_44913574_T_G	1.00	6996	5.53E-06	GCST90277401
Phosphatidylcholine (14:0_18:1) levels	1	150322476	G	A	-1.14E-01	2.44E-02	1.37E-01	rs11584070	1_150322476_A_G	1.00	7113	2.83E-06	GCST90277402
Phosphatidylcholine (14:0_18:1) levels	1	220806736	A	C	1.24E-01	2.16E-02	1.85E-01	rs34621709	1_220806736_C_A	1.00	7113	9.47E-09	GCST90277402
Phosphatidylcholine (14:0_18:1) levels	1	230148628	T	C	-9.52E-02	1.88E-02	7.40E-01	rs6692319	1_230148628_C_T	1.00	7113	4.41E-07	GCST90277402
Phosphatidylcholine (14:0_18:1) levels	2	21002409	T	C	9.71E-02	1.87E-02	7.28E-01	rs1042034	2_21002409_C_T	1.00	7113	2.12E-07	GCST90277402
Phosphatidylcholine (14:0_18:1) levels	2	27508073	C	T	-1.37E-01	1.75E-02	6.51E-01	rs1260326	2_27508073_T_C	1.00	7113	4.37E-15	GCST90277402
Phosphatidylcholine (14:0_18:1) levels	2	128427075	G	A	1.48E-01	3.21E-02	7.45E-02	rs76950187	2_128427075_A_G	0.99	7113	4.23E-06	GCST90277402
Phosphatidylcholine (14:0_18:1) levels	3	1895465	G	A	8.07E-02	1.67E-02	5.60E-01	rs12635725	3_1895465_A_G	0.99	7113	1.46E-06	GCST90277402
Phosphatidylcholine (14:0_18:1) levels	3	15332543	T	C	-1.19E-01	2.63E-02	1.12E-01	rs28450659	3_15332543_C_T	0.99	7113	6.66E-06	GCST90277402
Phosphatidylcholine (14:0_18:1) levels	3	87298641	A	G	-1.92E-01	3.93E-02	4.88E-02	rs116011373	3_87298641_G_A	0.96	7113	1.02E-06	GCST90277402
Phosphatidylcholine (14:0_18:1) levels	4	5003811	A	G	9.93E-02	2.11E-02	2.14E-01	rs62291574	4_5003811_G_A	0.94	7113	2.61E-06	GCST90277402
Phosphatidylcholine (14:0_18:1) levels	5	6523892	G	A	7.66E-02	1.68E-02	4.25E-01	rs501474	5_6523892_A_G	1.00	7113	5.14E-06	GCST90277402
Phosphatidylcholine (14:0_18:1) levels	5	83807154	A	G	2.58E-01	5.25E-02	2.62E-02	rs150741556	5_83807154_G_A	0.98	7113	9.24E-07	GCST90277402
Phosphatidylcholine (14:0_18:1) levels	7	73597712	A	G	-1.48E-01	2.55E-02	1.22E-01	rs35332062	7_73597712_G_A	1.00	7113	7.43E-09	GCST90277402
Phosphatidylcholine (14:0_18:1) levels	8	19967156	T	C	-1.11E-01	1.90E-02	2.61E-01	rs15285	8_19967156_C_T	1.00	7113	4.62E-09	GCST90277402
Phosphatidylcholine (14:0_18:1) levels	9	23713682	C	T	9.24E-02	2.08E-02	2.03E-01	rs3829088	9_23713682_T_C	1.00	7113	9.16E-06	GCST90277402
Phosphatidylcholine (14:0_18:1) levels	9	132470502	G	A	2.50E-01	5.46E-02	2.59E-02	rs111568723	9_132470502_A_G	0.95	7113	4.67E-06	GCST90277402
Phosphatidylcholine (14:0_18:1) levels	11	61814292	C	T	1.04E-01	1.73E-02	3.83E-01	rs174560	11_61814292_T_C	1.00	7113	1.96E-09	GCST90277402
Phosphatidylcholine (14:0_18:1) levels	11	116778201	C	G	-2.83E-01	2.30E-02	8.49E-01	rs964184	11_116778201_G_C	1.00	7113	2.83E-34	GCST90277402
Phosphatidylcholine (14:0_18:1) levels	12	26757135	C	A	-2.91E-01	6.46E-02	1.70E-02	rs138622921	12_26757135_A_C	0.99	7113	6.86E-06	GCST90277402
Phosphatidylcholine (14:0_18:1) levels	12	96850670	C	A	-3.81E-01	7.94E-02	1.12E-02	rs139500046	12_96850670_A_C	0.99	7113	1.56E-06	GCST90277402
Phosphatidylcholine (14:0_18:1) levels	12	129791836	A	G	-7.87E-02	1.75E-02	6.35E-01	rs935833	12_129791836_G_A	0.99	7113	7.19E-06	GCST90277402
Phosphatidylcholine (14:0_18:1) levels	13	21038020	T	C	-1.50E-01	3.04E-02	8.46E-02	rs1003966	13_21038020_C_T	0.99	7113	9.16E-07	GCST90277402
Phosphatidylcholine (14:0_18:1) levels	14	29823442	G	A	-2.11E-01	4.49E-02	3.72E-02	rs10147474	14_29823442_A_G	0.98	7113	2.67E-06	GCST90277402
Phosphatidylcholine (14:0_18:1) levels	16	4104550	C	T	7.74E-02	1.67E-02	4.43E-01	rs12448223	16_4104550_T_C	1.00	7113	3.77E-06	GCST90277402
Phosphatidylcholine (14:0_18:1) levels	16	71133811	T	C	-8.63E-02	1.92E-02	2.54E-01	rs1989995	16_71133811_C_T	0.99	7113	6.83E-06	GCST90277402
Phosphatidylcholine (14:0_18:2) levels	1	4317851	T	C	-2.62E-01	5.85E-02	9.75E-01	rs351596	1_4317851_C_T	0.92	6479	7.43E-06	GCST90277403
Phosphatidylcholine (14:0_18:2) levels	1	109657509	T	C	4.20E-01	9.36E-02	8.94E-03	rs116014876	1_109657509_C_T	0.97	6479	7.33E-06	GCST90277403
Phosphatidylcholine (14:0_18:2) levels	1	220806736	A	C	1.02E-01	2.25E-02	1.85E-01	rs34621709	1_220806736_C_A	1.00	6479	5.63E-06	GCST90277403

Phosphatidylcholine (14:0_18:2) levels	2	27508073	C	T	-1.36E-01	1.83E-02	6.51E-01	rs1260326	2_27508073_T_C	1.00	6479	1.14E-13	GCST90277403
Phosphatidylcholine (14:0_18:2) levels	2	141741698	A	C	-8.71E-02	1.79E-02	4.29E-01	rs1949011	2_141741698_C_A	0.97	6479	1.19E-06	GCST90277403
Phosphatidylcholine (14:0_18:2) levels	3	1895465	G	A	7.95E-02	1.76E-02	5.60E-01	rs12635725	3_1895465_A_G	0.99	6479	6.57E-06	GCST90277403
Phosphatidylcholine (14:0_18:2) levels	3	42816309	A	C	2.18E-01	4.67E-02	3.79E-02	rs79235240	3_42816309_C_A	0.98	6479	3.14E-06	GCST90277403
Phosphatidylcholine (14:0_18:2) levels	3	190557173	T	A	7.76E-01	1.59E-01	3.39E-03	rs138987797	3_190557173_A_T	0.93	6479	1.04E-06	GCST90277403
Phosphatidylcholine (14:0_18:2) levels	4	4999649	C	T	1.00E-01	2.21E-02	2.14E-01	rs62291573	4_4999649_T_C	0.94	6479	6.00E-06	GCST90277403
Phosphatidylcholine (14:0_18:2) levels	4	27061504	T	A	-7.65E-01	1.70E-01	3.12E-03	rs144500033	4_27061504_A_T	0.81	6479	7.15E-06	GCST90277403
Phosphatidylcholine (14:0_18:2) levels	4	180456342	T	C	7.74E-02	1.74E-02	4.63E-01	rs2546584	4_180456342_C_T	1.00	6479	9.37E-06	GCST90277403
Phosphatidylcholine (14:0_18:2) levels	5	66234813	A	G	-2.55E-01	5.68E-02	2.74E-02	rs55672000	5_66234813_G_A	0.91	6479	7.10E-06	GCST90277403
Phosphatidylcholine (14:0_18:2) levels	5	128702919	C	T	2.97E-01	6.62E-02	9.83E-01	rs247164	5_128702919_T_C	0.99	6479	7.48E-06	GCST90277403
Phosphatidylcholine (14:0_18:2) levels	6	31202352	A	T	1.10E-01	2.41E-02	1.59E-01	rs28362364	6_31202352_T_A	1.00	6479	5.48E-06	GCST90277403
Phosphatidylcholine (14:0_18:2) levels	7	73440219	T	C	-1.41E-01	3.00E-02	9.53E-02	rs71556711	7_73440219_C_T	0.99	6479	2.40E-06	GCST90277403
Phosphatidylcholine (14:0_18:2) levels	8	771605	C	G	2.33E-01	5.12E-02	3.20E-02	rs117829392	8_771605_G_C	0.96	6479	5.50E-06	GCST90277403
Phosphatidylcholine (14:0_18:2) levels	8	19970337	A	C	-1.12E-01	1.96E-02	2.79E-01	rs10105606	8_19970337_C_A	1.00	6479	1.29E-08	GCST90277403
Phosphatidylcholine (14:0_18:2) levels	8	59088496	C	T	1.26E-01	2.23E-02	1.91E-01	rs7846649	8_59088496_T_C	0.99	6479	1.58E-08	GCST90277403
Phosphatidylcholine (14:0_18:2) levels	9	1147365	T	C	-2.36E-01	5.24E-02	3.15E-02	rs13288401	9_1147365_C_T	0.91	6479	6.82E-06	GCST90277403
Phosphatidylcholine (14:0_18:2) levels	9	23713682	C	T	9.73E-02	2.20E-02	2.03E-01	rs3829088	9_23713682_T_C	1.00	6479	9.59E-06	GCST90277403
Phosphatidylcholine (14:0_18:2) levels	9	120256064	G	A	-2.02E-01	4.46E-02	9.55E-01	rs4837740	9_120256064_A_G	0.92	6479	6.30E-06	GCST90277403
Phosphatidylcholine (14:0_18:2) levels	11	38197193	A	G	-1.49E-01	3.21E-02	8.70E-02	rs36174561	11_38197193_G_A	0.96	6479	3.64E-06	GCST90277403
Phosphatidylcholine (14:0_18:2) levels	11	116778201	C	G	-2.74E-01	2.40E-02	8.49E-01	rs964184	11_116778201_G_C	1.00	6479	5.39E-30	GCST90277403
Phosphatidylcholine (14:0_18:2) levels	12	78389910	C	T	1.16E-01	2.41E-02	1.57E-01	rs2370511	12_78389910_T_C	1.00	6479	1.44E-06	GCST90277403
Phosphatidylcholine (14:0_18:2) levels	12	96850670	C	A	-4.02E-01	8.34E-02	1.12E-02	rs139500046	12_96850670_A_C	0.99	6479	1.52E-06	GCST90277403
Phosphatidylcholine (14:0_18:2) levels	13	34561042	G	A	8.65E-02	1.93E-02	3.34E-01	rs17081481	13_34561042_A_G	0.90	6479	7.33E-06	GCST90277403
Phosphatidylcholine (14:0_18:2) levels	14	97793198	A	G	1.04E-01	2.31E-02	1.90E-01	rs7152096	14_97793198_G_A	0.94	6479	7.83E-06	GCST90277403
Phosphatidylcholine (14:0_18:2) levels	16	73556815	A	G	-1.39E-01	2.89E-02	1.10E-01	rs825692	16_73556815_G_A	0.93	6479	1.65E-06	GCST90277403
Phosphatidylcholine (14:0_18:2) levels	17	1624088	T	G	1.04E-01	2.26E-02	1.96E-01	rs35832030	17_1624088_G_T	0.99	6479	4.30E-06	GCST90277403
Phosphatidylcholine (14:0_18:2) levels	17	41828494	A	G	-1.77E-01	3.86E-02	5.71E-02	rs117922651	17_41828494_G_A	1.00	6479	4.79E-06	GCST90277403
Phosphatidylcholine (14:0_18:2) levels	19	19282905	A	G	-1.14E-01	2.37E-02	1.71E-01	rs8100204	19_19282905_G_A	0.97	6479	1.43E-06	GCST90277403
Phosphatidylcholine (14:0_18:2) levels	19	41198399	C	T	7.85E-02	1.77E-02	5.77E-01	rs338593	19_41198399_T_C	1.00	6479	9.36E-06	GCST90277403
Phosphatidylcholine (14:0_18:2) levels	19	44892962	T	C	9.89E-02	2.11E-02	2.23E-01	rs157582	19_44892962_C_T	1.00	6479	3.00E-06	GCST90277403
Phosphatidylcholine (14:0_18:2) levels	20	59301745	A	G	3.16E-01	6.84E-02	1.95E-02	rs11570257	20_59301745_G_A	0.91	6479	4.00E-06	GCST90277403
Phosphatidylcholine (15:0_18:1) levels	1	2352403	T	G	-7.91E-02	1.68E-02	4.51E-01	rs2643912	1_2352403_G_T	1.00	7162	2.67E-06	GCST90277404
Phosphatidylcholine (15:0_18:1) levels	1	220800221	T	C	-1.17E-01	1.82E-02	6.93E-01	rs2642442	1_220800221_C_T	0.99	7162	1.42E-10	GCST90277404
Phosphatidylcholine (15:0_18:1) levels	1	230161390	T	C	-7.78E-02	1.69E-02	5.67E-01	rs2281721	1_230161390_C_T	1.00	7162	4.41E-06	GCST90277404
Phosphatidylcholine (15:0_18:1) levels	2	21002409	T	C	9.46E-02	1.86E-02	7.28E-01	rs1042034	2_21002409_C_T	1.00	7162	3.97E-07	GCST90277404
Phosphatidylcholine (15:0_18:1) levels	2	27508073	C	T	-9.49E-02	1.75E-02	6.51E-01	rs1260326	2_27508073_T_C	1.00	7162	5.61E-08	GCST90277404
Phosphatidylcholine (15:0_18:1) levels	2	104244849	A	T	-2.52E-01	5.66E-02	2.27E-02	rs147987331	2_104244849_T_A	0.97	7162	8.45E-06	GCST90277404
Phosphatidylcholine (15:0_18:1) levels	2	128427075	G	A	1.50E-01	3.20E-02	7.45E-02	rs76950187	2_128427075_A_G	0.99	7162	2.79E-06	GCST90277404
Phosphatidylcholine (15:0_18:1) levels	2	212010621	G	A	-8.26E-02	1.79E-02	6.76E-01	rs10169217	2_212010621_A_G	1.00	7162	3.95E-06	GCST90277404
Phosphatidylcholine (15:0_18:1) levels	3	1898506	T	G	7.77E-02	1.74E-02	3.49E-01	rs7638534	3_1898506_G_T	1.00	7162	8.59E-06	GCST90277404
Phosphatidylcholine (15:0_18:1) levels	4	4996200	T	C	1.10E-01	2.11E-02	2.14E-01	rs62291572	4_4996200_C_T	0.94	7162	1.63E-07	GCST90277404
Phosphatidylcholine (15:0_18:1) levels	4	81683582	G	C	-1.26E-01	2.67E-02	1.15E-01	rs3934642	4_81683582_C_G	0.96	7162	2.55E-06	GCST90277404
Phosphatidylcholine (15:0_18:1) levels	5	114571608	A	T	2.46E-01	5.52E-02	2.38E-02	rs78540742	5_114571608_T_A	0.97	7162	8.35E-06	GCST90277404
Phosphatidylcholine (15:0_18:1) levels	6	154880792	G	C	1.50E-01	3.21E-02	7.40E-02	rs141116593	6_154880792_C_G	0.99	7162	3.15E-06	GCST90277404
Phosphatidylcholine (15:0_18:1) levels	7	73606007	G	C	-1.20E-01	2.55E-02	1.21E-01	rs3812316	7_73606007_C_G	1.00	7162	2.41E-06	GCST90277404
Phosphatidylcholine (15:0_18:1) levels	7	155371896	A	G	-1.72E-01	3.81E-02	5.39E-02	rs13237172	7_155371896_G_A	0.95	7162	6.67E-06	GCST90277404
Phosphatidylcholine (15:0_18:1) levels	8	2896369	C	T	-3.74E-01	8.02E-02	1.18E-02	rs144679576	8_2896369_T_C	0.94	7162	3.18E-06	GCST90277404
Phosphatidylcholine (15:0_18:1) levels	8	19970337	A	C	-1.13E-01	1.85E-02	2.79E-01	rs10105606	8_19970337_C_A	1.00	7162	9.69E-10	GCST90277404
Phosphatidylcholine (15:0_18:1) levels	8	125495066	C	T	-8.76E-02	1.91E-02	7.43E-01	rs2980888	8_125495066_T_C	1.00	7162	4.60E-06	GCST90277404
Phosphatidylcholine (15:0_18:1) levels	9	136257994	G	A	-2.17E-01	4.86E-02	3.60E-02	rs112122274	9_136257994_A_G	0.85	7162	7.89E-06	GCST90277404
Phosphatidylcholine (15:0_18:1) levels	10	88675523	T	G	1.18E-01	2.56E-02	1.23E-01	rs17333900	10_88675523_G_T	0.99	7162	3.84E-06	GCST90277404
Phosphatidylcholine (15:0_18:1) levels	10	112142696	T	C	-8.72E-02	1.82E-02	2.97E-01	rs72836628	10_112142696_C_T	1.00	7162	1.79E-06	GCST90277404
Phosphatidylcholine (15:0_18:1) levels	11	61814292	C	T	1.33E-01	1.72E-02	3.83E-01	rs174560	11_61814292_T_C	1.00	7162	1.13E-14	GCST90277404
Phosphatidylcholine (15:0_18:1) levels	11	65951162	C	T	9.01E-02	2.01E-02	2.29E-01	rs56252769	11_65951162_T_C	0.99	7162	7.50E-06	GCST90277404
Phosphatidylcholine (15:0_18:1) levels	11	116618319	G	A	1.16E-01	2.27E-02	1.66E-01	rs12365864	11_116618319_A_G	0.97	7162	3.12E-07	GCST90277404
Phosphatidylcholine (15:0_18:1) levels	11	116778201	C	G	-2.86E-01	2.30E-02	8.49E-01	rs964184	11_116778201_G_C	1.00	7162	3.91E-35	GCST90277404
Phosphatidylcholine (15:0_18:1) levels	12	10312813	C	A	1.82E-01	4.08E-02	4.55E-02	rs112465335	12_10312813_A_C	0.93	7162	7.99E-06	GCST90277404

Phosphatidylcholine (15:0_18:1) levels	12	20732159	G	A	-4.08E-01	9.05E-02	9.58E-03	rs112576575	12_20732159_A_G	0.91	7162	6.74E-06	GCST90277404
Phosphatidylcholine (15:0_18:1) levels	12	117079732	C	G	9.43E-02	2.11E-02	1.92E-01	rs11068322	12_117079732_G_C	0.99	7162	7.76E-06	GCST90277404
Phosphatidylcholine (15:0_18:1) levels	13	20972374	G	C	9.83E-02	2.17E-02	1.82E-01	rs9578336	13_20972374_C_G	0.97	7162	5.84E-06	GCST90277404
Phosphatidylcholine (15:0_18:1) levels	14	58138012	C	T	3.50E-01	6.60E-02	1.73E-02	rs137871178	14_58138012_T_C	0.96	7162	1.15E-07	GCST90277404
Phosphatidylcholine (15:0_18:2) levels	1	2352403	T	G	-7.46E-02	1.68E-02	4.51E-01	rs2643912	1_2352403_G_T	1.00	7165	9.42E-06	GCST90277405
Phosphatidylcholine (15:0_18:2) levels	1	4317851	T	C	-2.47E-01	5.57E-02	9.75E-01	rs351596	1_4317851_C_T	0.92	7165	9.46E-06	GCST90277405
Phosphatidylcholine (15:0_18:2) levels	1	74445900	A	G	1.84E-01	4.06E-02	4.69E-02	rs59017319	1_74445900_G_A	0.92	7165	5.56E-06	GCST90277405
Phosphatidylcholine (15:0_18:2) levels	1	220800221	T	C	-1.20E-01	1.82E-02	6.93E-01	rs2642442	1_220800221_C_T	0.99	7165	4.79E-11	GCST90277405
Phosphatidylcholine (15:0_18:2) levels	1	230157290	G	A	-9.33E-02	1.76E-02	6.57E-01	rs10779833	1_230157290_A_G	0.97	7165	1.21E-07	GCST90277405
Phosphatidylcholine (15:0_18:2) levels	2	21002409	T	C	1.02E-01	1.86E-02	7.28E-01	rs1042034	2_21002409_C_T	1.00	7165	4.38E-08	GCST90277405
Phosphatidylcholine (15:0_18:2) levels	2	27508073	C	T	-1.05E-01	1.74E-02	6.51E-01	rs1260326	2_27508073_T_C	1.00	7165	1.95E-09	GCST90277405
Phosphatidylcholine (15:0_18:2) levels	2	79833869	C	T	-1.69E-01	3.67E-02	9.43E-01	rs2941755	2_79833869_T_C	0.97	7165	4.22E-06	GCST90277405
Phosphatidylcholine (15:0_18:2) levels	4	4999649	C	T	9.80E-02	2.10E-02	2.14E-01	rs62291573	4_4999649_T_C	0.94	7165	3.24E-06	GCST90277405
Phosphatidylcholine (15:0_18:2) levels	5	107893061	C	A	9.82E-02	2.16E-02	1.80E-01	rs17160706	5_107893061_A_C	0.98	7165	5.77E-06	GCST90277405
Phosphatidylcholine (15:0_18:2) levels	6	154916245	C	T	1.67E-01	3.36E-02	6.82E-02	rs144072769	6_154916245_T_C	0.96	7165	6.72E-07	GCST90277405
Phosphatidylcholine (15:0_18:2) levels	6	165581086	T	C	-1.96E-01	4.29E-02	4.38E-02	rs73022136	6_165581086_C_T	0.92	7165	4.96E-06	GCST90277405
Phosphatidylcholine (15:0_18:2) levels	7	44922206	T	C	-2.68E-01	5.91E-02	2.10E-02	rs78433420	7_44922206_C_T	0.97	7165	5.58E-06	GCST90277405
Phosphatidylcholine (15:0_18:2) levels	7	73606007	G	C	-1.24E-01	2.55E-02	1.21E-01	rs3812316	7_73606007_C_G	1.00	7165	1.09E-06	GCST90277405
Phosphatidylcholine (15:0_18:2) levels	8	19970337	A	C	-1.24E-01	1.85E-02	2.79E-01	rs10105606	8_19970337_C_A	1.00	7165	2.21E-11	GCST90277405
Phosphatidylcholine (15:0_18:2) levels	10	112142696	T	C	-8.98E-02	1.82E-02	2.97E-01	rs72836628	10_112142696_C_T	1.00	7165	8.55E-07	GCST90277405
Phosphatidylcholine (15:0_18:2) levels	11	40018396	A	G	-1.49E-01	3.34E-02	7.15E-02	rs189281327	11_40018396_G_A	0.92	7165	7.91E-06	GCST90277405
Phosphatidylcholine (15:0_18:2) levels	11	61858798	G	C	9.83E-02	1.74E-02	3.72E-01	rs174604	11_61858798_C_G	0.99	7165	1.71E-08	GCST90277405
Phosphatidylcholine (15:0_18:2) levels	11	116618319	G	A	1.22E-01	2.26E-02	1.66E-01	rs12365864	11_116618319_A_G	0.97	7165	7.80E-08	GCST90277405
Phosphatidylcholine (15:0_18:2) levels	11	116778201	C	G	-3.01E-01	2.29E-02	8.49E-01	rs964184	11_116778201_G_C	1.00	7165	9.12E-39	GCST90277405
Phosphatidylcholine (15:0_18:2) levels	12	129796667	A	G	-8.86E-02	1.81E-02	6.83E-01	rs10773708	12_129796667_G_A	1.00	7165	9.43E-07	GCST90277405
Phosphatidylcholine (15:0_18:2) levels	14	58138012	C	T	3.37E-01	6.59E-02	1.73E-02	rs137871178	14_58138012_T_C	0.96	7165	3.40E-07	GCST90277405
Phosphatidylcholine (15:0_18:2) levels	14	95218464	A	G	-8.34E-02	1.87E-02	2.75E-01	rs61702480	14_95218464_G_A	0.99	7165	8.61E-06	GCST90277405
Phosphatidylcholine (15:0_18:2) levels	17	1615646	G	A	8.54E-02	1.85E-02	3.02E-01	rs56051325	17_1615646_A_G	0.97	7165	3.91E-06	GCST90277405
Phosphatidylcholine (15:0_18:2) levels	19	8364439	A	G	-2.37E-01	5.14E-02	2.59E-02	rs116843064	19_8364439_G_A	1.00	7165	4.18E-06	GCST90277405
Phosphatidylcholine (15:0_18:2) levels	19	33148516	G	C	1.68E-01	3.66E-02	5.49E-02	rs115215688	19_33148516_C_G	0.97	7165	4.31E-06	GCST90277405
Phosphatidylcholine (15:0_18:2) levels	19	44913484	T	C	9.06E-02	1.96E-02	2.42E-01	rs438811	19_44913484_C_T	1.00	7165	3.66E-06	GCST90277405
Phosphatidylcholine (16:0_16:0) levels	1	4317851	T	C	-2.52E-01	5.57E-02	9.75E-01	rs351596	1_4317851_C_T	0.92	7146	5.89E-06	GCST90277406
Phosphatidylcholine (16:0_16:0) levels	1	74445900	A	G	1.87E-01	4.06E-02	4.69E-02	rs59017319	1_74445900_G_A	0.92	7146	4.20E-06	GCST90277406
Phosphatidylcholine (16:0_16:0) levels	1	220805303	T	C	1.30E-01	2.34E-02	1.52E-01	rs17596144	1_220805303_C_T	0.99	7146	2.84E-08	GCST90277406
Phosphatidylcholine (16:0_16:0) levels	1	230156121	C	T	-9.70E-02	1.96E-02	7.69E-01	rs2352723	1_230156121_T_C	1.00	7146	7.46E-07	GCST90277406
Phosphatidylcholine (16:0_16:0) levels	2	21002409	T	C	9.21E-02	1.86E-02	7.28E-01	rs1042034	2_21002409_C_T	1.00	7146	8.05E-07	GCST90277406
Phosphatidylcholine (16:0_16:0) levels	2	27508073	C	T	-1.22E-01	1.74E-02	6.51E-01	rs1260326	2_27508073_T_C	1.00	7146	3.24E-12	GCST90277406
Phosphatidylcholine (16:0_16:0) levels	2	87205327	T	C	4.79E-01	1.06E-01	6.65E-03	rs115614772	2_87205327_C_T	0.99	7146	6.30E-06	GCST90277406
Phosphatidylcholine (16:0_16:0) levels	3	11749984	G	A	7.88E-02	1.77E-02	3.40E-01	rs301555	3_11749984_A_G	0.98	7146	8.98E-06	GCST90277406
Phosphatidylcholine (16:0_16:0) levels	3	148163041	C	T	-4.51E-01	9.49E-02	8.76E-03	rs1456674	3_148163041_T_C	0.89	7146	2.10E-06	GCST90277406
Phosphatidylcholine (16:0_16:0) levels	5	107893061	C	A	9.68E-02	2.17E-02	1.80E-01	rs17160706	5_107893061_A_C	0.98	7146	8.00E-06	GCST90277406
Phosphatidylcholine (16:0_16:0) levels	5	118341860	G	A	9.03E-02	1.88E-02	7.39E-01	rs13178172	5_118341860_A_G	1.00	7146	1.53E-06	GCST90277406
Phosphatidylcholine (16:0_16:0) levels	6	98110337	T	A	3.57E-01	7.85E-02	1.18E-02	rs147151674	6_98110337_A_T	0.95	7146	5.61E-06	GCST90277406
Phosphatidylcholine (16:0_16:0) levels	6	154916245	C	T	1.64E-01	3.36E-02	6.82E-02	rs144072769	6_154916245_T_C	0.96	7146	1.03E-06	GCST90277406
Phosphatidylcholine (16:0_16:0) levels	7	73608416	A	G	-1.22E-01	2.55E-02	1.21E-01	rs13246993	7_73608416_G_A	1.00	7146	1.57E-06	GCST90277406
Phosphatidylcholine (16:0_16:0) levels	8	16479793	T	C	-2.30E-01	5.14E-02	3.00E-02	rs139245678	8_16479793_C_T	0.91	7146	7.90E-06	GCST90277406
Phosphatidylcholine (16:0_16:0) levels	8	19970337	A	C	-1.16E-01	1.85E-02	2.79E-01	rs10105606	8_19970337_C_A	1.00	7146	4.47E-10	GCST90277406
Phosphatidylcholine (16:0_16:0) levels	9	33414691	G	A	-7.61E-02	1.71E-02	4.38E-01	rs664178	9_33414691_A_G	0.96	7146	8.74E-06	GCST90277406
Phosphatidylcholine (16:0_16:0) levels	11	116618319	G	A	1.13E-01	2.27E-02	1.66E-01	rs12365864	11_116618319_A_G	0.97	7146	6.92E-07	GCST90277406
Phosphatidylcholine (16:0_16:0) levels	11	116778201	C	G	-2.68E-01	2.30E-02	8.49E-01	rs964184	11_116778201_G_C	1.00	7146	5.22E-31	GCST90277406
Phosphatidylcholine (16:0_16:0) levels	12	129796667	A	G	-8.71E-02	1.81E-02	6.83E-01	rs10773708	12_129796667_G_A	1.00	7146	1.43E-06	GCST90277406
Phosphatidylcholine (16:0_16:0) levels	13	20972374	G	C	9.72E-02	2.17E-02	1.82E-01	rs9578336	13_20972374_C_G	0.97	7146	7.48E-06	GCST90277406
Phosphatidylcholine (16:0_16:0) levels	13	89358643	A	G	-2.61E-01	5.77E-02	2.26E-02	rs79380657	13_89358643_G_A	0.95	7146	5.97E-06	GCST90277406
Phosphatidylcholine (16:0_16:0) levels	14	21794664	G	A	-1.50E-01	3.37E-02	6.71E-02	rs72671922	14_21794664_A_G	0.98	7146	9.57E-06	GCST90277406
Phosphatidylcholine (16:0_16:0) levels	14	58138012	C	T	2.98E-01	6.61E-02	1.73E-02	rs137871178	14_58138012_T_C	0.96	7146	6.67E-06	GCST90277406
Phosphatidylcholine (16:0_16:0) levels	17	1615646	G	A	9.13E-02	1.85E-02	3.02E-01	rs56051325	17_1615646_A_G	0.97	7146	8.23E-07	GCST90277406
Phosphatidylcholine (16:0_16:0) levels	19	33147680	G	C	1.91E-01	3.93E-02	4.80E-02	rs74994260	19_33147680_C_G	0.98	7146	1.28E-06	GCST90277406

Phosphatidylcholine (16:0_16:0) levels	19	44913484	T	C	9.21E-02	1.96E-02	2.42E-01	rs438811	19_44913484_C_T	1.00	7146	2.59E-06	GCST90277406
Phosphatidylcholine (16:0_16:1) levels	1	187918746	T	A	-8.33E-02	1.82E-02	3.15E-01	rs12405870	1_187918746_A_T	0.98	7066	5.13E-06	GCST90277407
Phosphatidylcholine (16:0_16:1) levels	1	220805303	T	C	1.10E-01	2.36E-02	1.52E-01	rs17596144	1_220805303_C_T	0.99	7066	3.22E-06	GCST90277407
Phosphatidylcholine (16:0_16:1) levels	1	228344494	T	G	7.58E-02	1.69E-02	5.90E-01	rs417237	1_228344494_G_T	1.00	7066	7.77E-06	GCST90277407
Phosphatidylcholine (16:0_16:1) levels	2	27508073	C	T	-1.12E-01	1.76E-02	6.51E-01	rs1260326	2_27508073_T_C	1.00	7066	1.83E-10	GCST90277407
Phosphatidylcholine (16:0_16:1) levels	2	204066660	G	A	6.17E-01	1.29E-01	4.64E-03	rs114561185	2_204066660_A_G	0.91	7066	1.70E-06	GCST90277407
Phosphatidylcholine (16:0_16:1) levels	3	148163041	C	T	-4.37E-01	9.58E-02	8.76E-03	rs1456674	3_148163041_T_C	0.89	7066	5.16E-06	GCST90277407
Phosphatidylcholine (16:0_16:1) levels	4	163303980	C	A	8.08E-02	1.69E-02	5.46E-01	rs4057791	4_163303980_A_C	1.00	7066	1.72E-06	GCST90277407
Phosphatidylcholine (16:0_16:1) levels	5	118341860	G	A	9.14E-02	1.89E-02	7.39E-01	rs13178172	5_118341860_A_G	1.00	7066	1.30E-06	GCST90277407
Phosphatidylcholine (16:0_16:1) levels	6	64177173	T	C	7.75E-02	1.73E-02	3.73E-01	rs9344829	6_64177173_C_T	0.99	7066	8.10E-06	GCST90277407
Phosphatidylcholine (16:0_16:1) levels	6	164617090	G	A	-3.51E-01	7.79E-02	1.23E-02	rs181609235	6_164617090_A_G	0.97	7066	6.54E-06	GCST90277407
Phosphatidylcholine (16:0_16:1) levels	8	19956018	G	A	2.94E-01	5.74E-02	2.27E-02	rs268	8_19956018_A_G	0.97	7066	3.09E-07	GCST90277407
Phosphatidylcholine (16:0_16:1) levels	9	31005163	A	G	4.67E-01	1.03E-01	7.05E-03	rs140647506	9_31005163_G_A	0.94	7066	5.83E-06	GCST90277407
Phosphatidylcholine (16:0_16:1) levels	10	7871139	C	T	4.32E-01	8.86E-02	9.45E-03	rs190411877	10_7871139_T_C	0.94	7066	1.08E-06	GCST90277407
Phosphatidylcholine (16:0_16:1) levels	11	61811991	G	A	-9.83E-02	1.71E-02	4.06E-01	rs174554	11_61811991_A_G	1.00	7066	9.75E-09	GCST90277407
Phosphatidylcholine (16:0_16:1) levels	11	116778201	C	G	-2.18E-01	2.32E-02	8.49E-01	rs964184	11_116778201_G_C	1.00	7066	6.27E-21	GCST90277407
Phosphatidylcholine (16:0_16:1) levels	17	1615646	G	A	9.21E-02	1.86E-02	3.02E-01	rs56051325	17_1615646_A_G	0.97	7066	8.01E-07	GCST90277407
Phosphatidylcholine (16:0_16:1) levels	17	78966282	T	C	9.55E-02	2.10E-02	7.93E-01	rs6501273	17_78966282_C_T	0.98	7066	5.34E-06	GCST90277407
Phosphatidylcholine (16:0_16:1) levels	19	19347579	G	A	-1.95E-01	3.79E-02	5.38E-02	rs182611493	19_19347579_A_G	0.95	7066	2.97E-07	GCST90277407
Phosphatidylcholine (16:0_16:1) levels	19	44912383	A	G	1.55E-01	3.13E-02	7.74E-02	rs445925	19_44912383_G_A	1.00	7066	7.83E-07	GCST90277407
Phosphatidylcholine (16:0_16:1) levels	20	46633119	A	C	1.22E-01	2.71E-02	1.11E-01	rs8123587	20_46633119_C_A	0.98	7066	6.47E-06	GCST90277407
Phosphatidylcholine (16:0_17:1) levels	1	109791954	A	T	8.87E-02	1.91E-02	3.27E-01	rs4970780	1_109791954_T_A	0.89	6828	3.47E-06	GCST90277408
Phosphatidylcholine (16:0_17:1) levels	1	217318646	G	A	2.65E-01	5.68E-02	2.35E-02	rs76481684	1_217318646_A_G	0.98	6828	3.18E-06	GCST90277408
Phosphatidylcholine (16:0_17:1) levels	1	228344494	T	G	8.06E-02	1.72E-02	5.90E-01	rs417237	1_228344494_G_T	1.00	6828	3.02E-06	GCST90277408
Phosphatidylcholine (16:0_17:1) levels	1	230959661	A	G	-1.58E-01	3.35E-02	7.08E-02	rs111853669	1_230959661_G_A	0.98	6828	2.58E-06	GCST90277408
Phosphatidylcholine (16:0_17:1) levels	1	246000079	C	T	9.39E-02	1.85E-02	6.97E-01	rs12120498	1_246000079_T_C	0.99	6828	4.10E-07	GCST90277408
Phosphatidylcholine (16:0_17:1) levels	2	27508073	C	T	-1.03E-01	1.78E-02	6.51E-01	rs1260326	2_27508073_T_C	1.00	6828	8.72E-09	GCST90277408
Phosphatidylcholine (16:0_17:1) levels	3	20815484	C	T	-7.86E-02	1.74E-02	4.24E-01	rs4858271	3_20815484_T_C	1.00	6828	6.08E-06	GCST90277408
Phosphatidylcholine (16:0_17:1) levels	3	122621089	T	C	-3.19E-01	7.01E-02	1.52E-02	rs73192177	3_122621089_C_T	0.96	6828	5.59E-06	GCST90277408
Phosphatidylcholine (16:0_17:1) levels	4	185010479	T	G	-5.85E-01	1.31E-01	4.53E-03	rs138383428	4_185010479_G_T	0.87	6828	8.67E-06	GCST90277408
Phosphatidylcholine (16:0_17:1) levels	6	129737300	G	A	-1.88E-01	4.19E-02	4.68E-02	rs12210453	6_129737300_A_G	0.94	6828	7.46E-06	GCST90277408
Phosphatidylcholine (16:0_17:1) levels	7	4768282	A	G	7.92E-01	1.72E-01	3.09E-03	rs377037955	7_4768282_G_A	0.84	6828	3.97E-06	GCST90277408
Phosphatidylcholine (16:0_17:1) levels	7	71293164	T	C	-9.78E-02	2.17E-02	1.96E-01	rs74516326	7_71293164_C_T	0.98	6828	6.58E-06	GCST90277408
Phosphatidylcholine (16:0_17:1) levels	8	5570990	G	C	-3.65E-01	8.26E-02	1.05E-02	rs73660137	8_5570990_C_G	0.99	6828	9.79E-06	GCST90277408
Phosphatidylcholine (16:0_17:1) levels	8	20012504	G	T	-9.16E-02	1.96E-02	2.53E-01	rs34942551	8_20012504_T_G	1.00	6828	3.11E-06	GCST90277408
Phosphatidylcholine (16:0_17:1) levels	9	1496121	G	A	-1.67E-01	3.61E-02	6.05E-02	rs62534834	9_1496121_A_G	0.98	6828	3.72E-06	GCST90277408
Phosphatidylcholine (16:0_17:1) levels	9	31005163	A	G	4.64E-01	1.03E-01	7.05E-03	rs140647506	9_31005163_G_A	0.94	6828	6.41E-06	GCST90277408
Phosphatidylcholine (16:0_17:1) levels	10	7871139	C	T	4.23E-01	8.88E-02	9.45E-03	rs190411877	10_7871139_T_C	0.94	6828	1.91E-06	GCST90277408
Phosphatidylcholine (16:0_17:1) levels	10	57923245	A	G	8.94E-02	1.94E-02	2.69E-01	rs2192081	10_57923245_G_A	1.00	6828	4.07E-06	GCST90277408
Phosphatidylcholine (16:0_17:1) levels	10	94386362	T	C	-3.95E-01	8.86E-02	9.13E-03	rs184609620	10_94386362_C_T	1.00	6828	8.31E-06	GCST90277408
Phosphatidylcholine (16:0_17:1) levels	11	61843278	A	G	-1.26E-01	1.74E-02	4.10E-01	rs174584	11_61843278_G_A	1.00	6828	5.09E-13	GCST90277408
Phosphatidylcholine (16:0_17:1) levels	11	62438816	T	C	-8.51E-02	1.81E-02	3.22E-01	rs9645690	11_62438816_C_T	1.00	6828	2.74E-06	GCST90277408
Phosphatidylcholine (16:0_17:1) levels	11	116778201	C	G	-1.74E-01	2.36E-02	8.49E-01	rs964184	11_116778201_G_C	1.00	6828	1.70E-13	GCST90277408
Phosphatidylcholine (16:0_17:1) levels	14	104290084	C	G	1.22E-01	2.63E-02	1.35E-01	rs56870907	14_104290084_G_C	0.90	6828	3.61E-06	GCST90277408
Phosphatidylcholine (16:0_17:1) levels	17	1623049	C	T	9.73E-02	2.19E-02	1.96E-01	rs35986054	17_1623049_T_C	0.98	6828	9.38E-06	GCST90277408
Phosphatidylcholine (16:0_17:1) levels	19	19347579	G	A	-2.27E-01	3.88E-02	5.38E-02	rs182611493	19_19347579_A_G	0.95	6828	5.05E-09	GCST90277408
Phosphatidylcholine (16:0_17:1) levels	19	19547663	T	G	-1.61E-01	3.45E-02	6.58E-02	rs16996148	19_19547663_G_T	1.00	6828	3.16E-06	GCST90277408
Phosphatidylcholine (16:0_18:0) levels	1	27886295	T	C	1.46E-01	3.01E-02	1.00E-01	rs11399	1_27886295_C_T	0.99	6172	1.19E-06	GCST90277409
Phosphatidylcholine (16:0_18:0) levels	1	99527521	T	C	-1.27E-01	2.67E-02	8.66E-01	rs4908327	1_99527521_C_T	0.98	6172	2.03E-06	GCST90277409
Phosphatidylcholine (16:0_18:0) levels	1	162720456	C	T	9.84E-02	2.20E-02	7.94E-01	rs1539733	1_162720456_T_C	0.99	6172	7.49E-06	GCST90277409
Phosphatidylcholine (16:0_18:0) levels	1	220805303	T	C	1.62E-01	2.51E-02	1.52E-01	rs17596144	1_220805303_C_T	0.99	6172	1.11E-10	GCST90277409
Phosphatidylcholine (16:0_18:0) levels	2	212010621	G	A	-9.92E-02	1.92E-02	6.76E-01	rs10169217	2_212010621_A_G	1.00	6172	2.63E-07	GCST90277409
Phosphatidylcholine (16:0_18:0) levels	3	103282265	A	G	1.19E-01	2.57E-02	1.48E-01	rs9851701	3_103282265_G_A	0.97	6172	3.58E-06	GCST90277409
Phosphatidylcholine (16:0_18:0) levels	4	4996200	T	C	1.08E-01	2.25E-02	2.14E-01	rs62291572	4_4996200_C_T	0.94	6172	1.86E-06	GCST90277409
Phosphatidylcholine (16:0_18:0) levels	5	109602550	T	G	-8.88E-02	1.94E-02	3.02E-01	rs10058653	5_109602550_G_T	1.00	6172	4.77E-06	GCST90277409
Phosphatidylcholine (16:0_18:0) levels	6	64441454	A	G	1.36E-01	2.72E-02	1.28E-01	rs75008187	6_64441454_G_A	1.00	6172	6.10E-07	GCST90277409
Phosphatidylcholine (16:0_18:0) levels	8	125472396	C	G	-1.01E-01	2.06E-02	7.43E-01	rs2980862	8_125472396_G_C	1.00	6172	8.68E-07	GCST90277409

Phosphatidylcholine (16:0_18:0) levels	9	122973735	T	C	3.87E-01	8.45E-02	1.43E-02	rs145737110	9_122973735_C_T	0.86	6172	4.77E-06	GCST90277409
Phosphatidylcholine (16:0_18:0) levels	9	132470502	G	A	2.87E-01	5.88E-02	2.59E-02	rs111568723	9_132470502_A_G	0.95	6172	1.05E-06	GCST90277409
Phosphatidylcholine (16:0_18:0) levels	11	61814292	C	T	1.70E-01	1.84E-02	3.83E-01	rs174560	11_61814292_T_C	1.00	6172	3.95E-20	GCST90277409
Phosphatidylcholine (16:0_18:0) levels	11	65863732	A	G	2.08E-01	4.44E-02	4.44E-02	rs149199066	11_65863732_G_A	0.98	6172	2.92E-06	GCST90277409
Phosphatidylcholine (16:0_18:0) levels	11	79066210	A	G	-2.44E-01	5.29E-02	3.22E-02	rs116846412	11_79066210_G_A	0.91	6172	3.92E-06	GCST90277409
Phosphatidylcholine (16:0_18:0) levels	11	116778201	C	G	-2.38E-01	2.45E-02	8.49E-01	rs964184	11_116778201_G_C	1.00	6172	4.19E-22	GCST90277409
Phosphatidylcholine (16:0_18:0) levels	11	128757016	A	G	-2.38E-01	5.09E-02	3.38E-02	rs117786044	11_128757016_G_A	0.95	6172	2.83E-06	GCST90277409
Phosphatidylcholine (16:0_18:0) levels	13	22883681	A	G	1.19E-01	2.70E-02	1.29E-01	rs58355654	13_22883681_G_A	0.99	6172	9.76E-06	GCST90277409
Phosphatidylcholine (16:0_18:0) levels	16	6791691	C	A	-1.85E-01	4.19E-02	5.08E-02	rs62016118	16_6791691_A_C	0.97	6172	9.92E-06	GCST90277409
Phosphatidylcholine (16:0_18:0) levels	16	23267355	A	G	-2.51E-01	5.51E-02	2.88E-02	rs117819583	16_23267355_G_A	0.92	6172	5.16E-06	GCST90277409
Phosphatidylcholine (16:0_18:0) levels	16	62982329	A	C	-8.50E-01	1.78E-01	2.49E-03	rs181023072	16_62982329_C_A	0.97	6172	1.72E-06	GCST90277409
Phosphatidylcholine (16:0_18:0) levels	18	72273093	G	A	8.57E-02	1.88E-02	3.46E-01	rs11151739	18_72273093_A_G	1.00	6172	5.08E-06	GCST90277409
Phosphatidylcholine (16:0_18:0) levels	19	44908822	T	C	2.20E-01	3.99E-02	5.31E-02	rs7412	19_44908822_C_T	1.00	6172	3.91E-08	GCST90277409
Phosphatidylcholine (16:0_18:0) levels	21	24358586	G	A	-2.02E-01	4.56E-02	4.11E-02	rs138845231	21_24358586_A_G	0.96	6172	9.89E-06	GCST90277409
Phosphatidylcholine (16:0_18:1) levels	1	220806736	A	C	1.48E-01	2.38E-02	1.85E-01	rs34621709	1_220806736_C_A	1.00	5820	5.58E-10	GCST90277410
Phosphatidylcholine (16:0_18:1) levels	2	212010621	G	A	-9.49E-02	1.97E-02	6.76E-01	rs10169217	2_212010621_A_G	1.00	5820	1.53E-06	GCST90277410
Phosphatidylcholine (16:0_18:1) levels	4	4996200	T	C	1.10E-01	2.32E-02	2.14E-01	rs62291572	4_4996200_C_T	0.94	5820	2.19E-06	GCST90277410
Phosphatidylcholine (16:0_18:1) levels	4	95245119	T	C	-2.35E-01	5.28E-02	3.44E-02	rs35120448	4_95245119_C_T	0.90	5820	9.11E-06	GCST90277410
Phosphatidylcholine (16:0_18:1) levels	4	142175079	C	A	-3.36E-01	7.61E-02	1.54E-02	rs75533654	4_142175079_A_C	0.93	5820	9.90E-06	GCST90277410
Phosphatidylcholine (16:0_18:1) levels	6	154880792	G	C	1.61E-01	3.52E-02	7.40E-02	rs141116593	6_154880792_C_G	0.99	5820	4.83E-06	GCST90277410
Phosphatidylcholine (16:0_18:1) levels	7	115692953	A	G	1.92E-01	4.35E-02	5.21E-02	rs62475137	7_115692953_G_A	0.90	5820	9.80E-06	GCST90277410
Phosphatidylcholine (16:0_18:1) levels	8	6766077	G	T	8.54E-02	1.90E-02	5.51E-01	rs2978946	8_6766077_T_G	0.95	5820	7.07E-06	GCST90277410
Phosphatidylcholine (16:0_18:1) levels	8	56921257	G	T	2.03E-01	4.20E-02	5.16E-02	rs117883640	8_56921257_T_G	0.96	5820	1.35E-06	GCST90277410
Phosphatidylcholine (16:0_18:1) levels	9	23714477	T	A	1.09E-01	2.30E-02	2.02E-01	rs10966037	9_23714477_A_T	1.00	5820	1.94E-06	GCST90277410
Phosphatidylcholine (16:0_18:1) levels	11	43542682	A	G	-1.16E-01	2.34E-02	2.02E-01	rs10430989	11_43542682_G_A	0.97	5820	6.63E-07	GCST90277410
Phosphatidylcholine (16:0_18:1) levels	11	61851136	G	A	1.36E-01	1.87E-02	4.18E-01	rs174592	11_61851136_A_G	1.00	5820	4.89E-13	GCST90277410
Phosphatidylcholine (16:0_18:1) levels	11	116778201	C	G	-2.42E-01	2.53E-02	8.49E-01	rs964184	11_116778201_G_C	1.00	5820	2.14E-21	GCST90277410
Phosphatidylcholine (16:0_18:1) levels	12	72431183	C	T	-9.27E-02	1.97E-02	3.48E-01	rs36094040	12_72431183_T_C	0.99	5820	2.67E-06	GCST90277410
Phosphatidylcholine (16:0_18:1) levels	12	129794726	T	C	1.09E-01	2.24E-02	2.16E-01	rs61942065	12_129794726_C_T	1.00	5820	1.15E-06	GCST90277410
Phosphatidylcholine (16:0_18:1) levels	15	45435268	GA	G	2.54E-01	5.50E-02	3.33E-02	rs146995542	15_45435268_G_GA	0.93	5820	3.87E-06	GCST90277410
Phosphatidylcholine (16:0_18:1) levels	16	4104550	C	T	8.17E-02	1.85E-02	4.43E-01	rs12448223	16_4104550_T_C	1.00	5820	1.00E-05	GCST90277410
Phosphatidylcholine (16:0_18:1) levels	19	44913574	G	T	1.73E-01	3.44E-02	7.73E-02	rs390082	19_44913574_T_G	1.00	5820	5.27E-07	GCST90277410
Phosphatidylcholine (16:0_18:1) levels	20	983640	T	C	-3.91E-01	8.59E-02	1.30E-02	rs73082153	20_983640_C_T	0.93	5820	5.27E-06	GCST90277410
Phosphatidylcholine (16:0_18:1) levels	22	43932163	C	T	9.89E-02	1.90E-02	3.91E-01	rs1883350	22_43932163_T_C	0.99	5820	2.12E-07	GCST90277410
Phosphatidylcholine (16:0_18:2) levels	1	182723688	C	G	-2.37E-01	5.19E-02	3.52E-02	rs182009884	1_182723688_G_C	0.97	5490	4.98E-06	GCST90277411
Phosphatidylcholine (16:0_18:2) levels	1	220806736	A	C	1.25E-01	2.46E-02	1.85E-01	rs34621709	1_220806736_C_A	1.00	5490	3.71E-07	GCST90277411
Phosphatidylcholine (16:0_18:2) levels	2	212010621	G	A	-1.04E-01	2.03E-02	6.76E-01	rs10169217	2_212010621_A_G	1.00	5490	3.51E-07	GCST90277411
Phosphatidylcholine (16:0_18:2) levels	3	6179893	A	G	6.99E-01	1.49E-01	5.17E-03	rs149473233	3_6179893_G_A	0.89	5490	2.64E-06	GCST90277411
Phosphatidylcholine (16:0_18:2) levels	3	87531960	A	G	1.52E-01	3.38E-02	9.13E-01	rs11127989	3_87531960_G_A	1.00	5490	7.06E-06	GCST90277411
Phosphatidylcholine (16:0_18:2) levels	3	189075054	G	A	1.71E-01	3.42E-02	8.82E-02	rs9682946	3_189075054_A_G	0.98	5490	5.66E-07	GCST90277411
Phosphatidylcholine (16:0_18:2) levels	4	27694435	A	G	3.58E-01	7.72E-02	1.54E-02	rs78081886	4_27694435_G_A	0.96	5490	3.52E-06	GCST90277411
Phosphatidylcholine (16:0_18:2) levels	4	32591008	T	A	1.05E-01	2.24E-02	2.78E-01	rs73125564	4_32591008_A_T	0.90	5490	2.68E-06	GCST90277411
Phosphatidylcholine (16:0_18:2) levels	4	38490214	T	C	-1.02E-01	2.31E-02	2.34E-01	rs4073968	4_38490214_C_T	0.96	5490	9.99E-06	GCST90277411
Phosphatidylcholine (16:0_18:2) levels	4	99524202	T	C	2.85E-01	6.21E-02	2.45E-02	rs9997979	4_99524202_C_T	0.98	5490	4.44E-06	GCST90277411
Phosphatidylcholine (16:0_18:2) levels	6	109778182	T	C	-3.25E-01	7.35E-02	1.78E-02	rs75768973	6_109778182_C_T	0.99	5490	9.80E-06	GCST90277411
Phosphatidylcholine (16:0_18:2) levels	6	163939775	A	G	9.08E-02	2.02E-02	4.50E-01	rs12193589	6_163939775_G_A	0.90	5490	7.08E-06	GCST90277411
Phosphatidylcholine (16:0_18:2) levels	8	20005513	T	C	-1.33E-01	2.12E-02	2.80E-01	rs1441764	8_20005513_C_T	1.00	5490	3.60E-10	GCST90277411
Phosphatidylcholine (16:0_18:2) levels	8	107215216	TGG	T	-6.56E-01	1.45E-01	4.66E-03	rs199629362	8_107215216_T_TGG	0.96	5490	5.83E-06	GCST90277411
Phosphatidylcholine (16:0_18:2) levels	9	114264016	T	C	-1.27E-01	2.79E-02	1.38E-01	rs34527930	9_114264016_C_T	0.99	5490	5.54E-06	GCST90277411
Phosphatidylcholine (16:0_18:2) levels	10	112611346	T	G	2.42E-01	5.34E-02	9.64E-01	rs2038844	10_112611346_G_T	0.92	5490	6.10E-06	GCST90277411
Phosphatidylcholine (16:0_18:2) levels	11	4653826	A	G	8.78E-02	1.95E-02	4.22E-01	rs1472230	11_4653826_G_A	1.00	5490	6.60E-06	GCST90277411
Phosphatidylcholine (16:0_18:2) levels	11	61856709	C	T	1.54E-01	1.95E-02	5.80E-01	rs97384	11_61856709_T_C	0.99	5490	3.45E-15	GCST90277411
Phosphatidylcholine (16:0_18:2) levels	11	116778201	C	G	-2.10E-01	2.58E-02	8.49E-01	rs964184	11_116778201_G_C	1.00	5490	4.58E-16	GCST90277411
Phosphatidylcholine (16:0_18:2) levels	13	83982225	G	A	-1.51E-01	3.05E-02	1.15E-01	rs61960432	13_83982225_A_G	0.97	5490	8.22E-07	GCST90277411
Phosphatidylcholine (16:0_18:2) levels	14	95282764	A	G	-9.21E-02	2.00E-02	6.50E-01	rs11623520	14_95282764_G_A	1.00	5490	4.02E-06	GCST90277411
Phosphatidylcholine (16:0_18:2) levels	17	78155616	C	T	1.21E-01	2.68E-02	1.54E-01	rs60102597	17_78155616_T_C	0.95	5490	6.74E-06	GCST90277411
Phosphatidylcholine (16:0_18:2) levels	19	20106360	T	C	-2.18E-01	4.73E-02	4.53E-02	rs4608457	19_20106360_C_T	0.97	5490	3.98E-06	GCST90277411

Phosphatidylcholine (16:0_18:2) levels	19	44913574	G	T	1.78E-01	3.48E-02	7.73E-02	rs390082	19_44913574_T_G	1.00	5490	3.18E-07	GCST90277411
Phosphatidylcholine (16:0_18:2) levels	22	43928847	G	C	1.45E-01	2.27E-02	2.24E-01	rs738409	22_43928847_C_G	1.00	5490	1.65E-10	GCST90277411
Phosphatidylcholine (16:0_18:3) levels	1	220805303	T	C	1.11E-01	2.34E-02	1.52E-01	rs17596144	1_220805303_C_T	0.99	7151	2.05E-06	GCST90277412
Phosphatidylcholine (16:0_18:3) levels	2	20934251	C	T	-8.79E-02	1.83E-02	2.85E-01	rs56296027	2_20934251_T_C	1.00	7151	1.60E-06	GCST90277412
Phosphatidylcholine (16:0_18:3) levels	2	27508073	C	T	-1.18E-01	1.74E-02	6.51E-01	rs1260326	2_27508073_T_C	1.00	7151	1.60E-11	GCST90277412
Phosphatidylcholine (16:0_18:3) levels	2	56529283	G	A	3.48E-01	7.58E-02	1.27E-02	rs114971431	2_56529283_A_G	0.97	7151	4.45E-06	GCST90277412
Phosphatidylcholine (16:0_18:3) levels	3	37455234	G	A	-8.66E-02	1.70E-02	4.02E-01	rs197730	3_37455234_A_G	1.00	7151	3.38E-07	GCST90277412
Phosphatidylcholine (16:0_18:3) levels	3	176326805	T	C	-2.48E-01	5.20E-02	2.74E-02	rs187665388	3_176326805_C_T	0.97	7151	1.93E-06	GCST90277412
Phosphatidylcholine (16:0_18:3) levels	6	32139362	C	T	-7.68E-02	1.73E-02	6.31E-01	rs3096698	6_32139362_T_C	0.99	7151	9.21E-06	GCST90277412
Phosphatidylcholine (16:0_18:3) levels	6	44048675	C	T	4.02E-01	8.88E-02	9.91E-03	rs115007623	6_44048675_T_C	0.90	7151	5.98E-06	GCST90277412
Phosphatidylcholine (16:0_18:3) levels	6	168930674	G	A	-9.53E-02	1.96E-02	2.41E-01	rs4277987	6_168930674_A_G	0.98	7151	1.14E-06	GCST90277412
Phosphatidylcholine (16:0_18:3) levels	7	73615107	T	C	-1.28E-01	2.54E-02	1.22E-01	rs33951980	7_73615107_C_T	1.00	7151	4.81E-07	GCST90277412
Phosphatidylcholine (16:0_18:3) levels	7	86663619	G	T	8.33E-02	1.88E-02	2.64E-01	rs1089588	7_86663619_T_G	1.00	7151	9.81E-06	GCST90277412
Phosphatidylcholine (16:0_18:3) levels	8	19970337	A	C	-1.30E-01	1.85E-02	2.79E-01	rs10105606	8_19970337_C_A	1.00	7151	2.19E-12	GCST90277412
Phosphatidylcholine (16:0_18:3) levels	8	125469835	G	A	-8.04E-02	1.68E-02	5.38E-01	rs2954021	8_125469835_A_G	1.00	7151	1.66E-06	GCST90277412
Phosphatidylcholine (16:0_18:3) levels	8	130143197	A	G	-7.98E-02	1.75E-02	6.37E-01	rs7846376	8_130143197_G_A	1.00	7151	5.53E-06	GCST90277412
Phosphatidylcholine (16:0_18:3) levels	9	71634286	T	C	-1.37E-01	2.95E-02	9.49E-02	rs7863337	9_71634286_C_T	0.95	7151	3.65E-06	GCST90277412
Phosphatidylcholine (16:0_18:3) levels	9	76634477	CAGCCAAT	C	1.69E-01	3.66E-02	5.81E-02	rs142908175	9_76634477_C_CAGCCA	0.94	7151	4.02E-06	GCST90277412
Phosphatidylcholine (16:0_18:3) levels	9	120268566	C	T	-2.19E-01	4.61E-02	9.62E-01	rs10739560	9_120268566_T_C	0.92	7151	2.07E-06	GCST90277412
Phosphatidylcholine (16:0_18:3) levels	10	74276153	T	C	-3.79E-01	8.27E-02	1.03E-02	rs56013388	10_74276153_C_T	0.97	7151	4.59E-06	GCST90277412
Phosphatidylcholine (16:0_18:3) levels	11	61770929	C	G	-7.79E-02	1.71E-02	4.44E-01	rs174527	11_61770929_G_C	0.98	7151	5.30E-06	GCST90277412
Phosphatidylcholine (16:0_18:3) levels	11	61781553	A	G	-2.33E-01	1.68E-02	4.09E-01	rs174533	11_61781553_G_A	1.00	7151	8.17E-43	GCST90277412
Phosphatidylcholine (16:0_18:3) levels	11	116778201	C	G	-2.41E-01	2.31E-02	8.49E-01	rs964184	11_116778201_G_C	1.00	7151	2.07E-25	GCST90277412
Phosphatidylcholine (16:0_18:3) levels	13	20972374	G	C	1.00E-01	2.17E-02	1.82E-01	rs9578336	13_20972374_C_G	0.97	7151	4.07E-06	GCST90277412
Phosphatidylcholine (16:0_18:3) levels	16	82427963	C	T	2.80E-01	6.20E-02	1.90E-02	rs187032204	16_82427963_T_C	0.97	7151	6.51E-06	GCST90277412
Phosphatidylcholine (16:0_18:3) levels	16	85292036	G	C	-1.04E-01	2.34E-02	1.55E-01	rs2968430	16_85292036_C_G	0.96	7151	8.42E-06	GCST90277412
Phosphatidylcholine (16:0_18:3) levels	19	19269704	G	A	-2.97E-01	3.79E-02	5.35E-02	rs187429064	19_19269704_A_G	0.95	7151	5.28E-15	GCST90277412
Phosphatidylcholine (16:0_18:3) levels	19	19547663	T	G	-1.71E-01	3.34E-02	6.58E-02	rs16996148	19_19547663_G_T	1.00	7151	3.15E-07	GCST90277412
Phosphatidylcholine (16:0_18:3) levels	19	44912383	A	G	1.94E-01	3.11E-02	7.74E-02	rs445925	19_44912383_G_A	1.00	7151	4.64E-10	GCST90277412
Phosphatidylcholine (16:0_18:3) levels	20	15006201	C	A	5.20E-01	1.16E-01	5.39E-03	rs114160087	20_15006201_A_C	0.94	7151	7.95E-06	GCST90277412
Phosphatidylcholine (16:0_18:3) levels	22	43928847	G	C	1.78E-01	2.00E-02	2.24E-01	rs738409	22_43928847_C_G	1.00	7151	5.90E-19	GCST90277412
Phosphatidylcholine (16:0_20:1) levels	1	72049213	T	C	1.24E-01	2.66E-02	1.14E-01	rs6699175	1_72049213_C_T	0.96	7148	3.27E-06	GCST90277413
Phosphatidylcholine (16:0_20:1) levels	1	109650768	G	A	8.04E-02	1.69E-02	5.95E-01	rs592586	1_109650768_A_G	0.99	7148	2.10E-06	GCST90277413
Phosphatidylcholine (16:0_20:1) levels	2	27508073	C	T	-9.86E-02	1.75E-02	6.51E-01	rs1260326	2_27508073_T_C	1.00	7148	1.79E-08	GCST90277413
Phosphatidylcholine (16:0_20:1) levels	2	74005215	A	G	-1.27E-01	2.82E-02	1.04E-01	rs7608044	2_74005215_G_A	0.95	7148	7.11E-06	GCST90277413
Phosphatidylcholine (16:0_20:1) levels	3	72073972	A	G	-2.28E-01	5.12E-02	2.88E-02	rs73095198	3_72073972_G_A	0.99	7148	8.52E-06	GCST90277413
Phosphatidylcholine (16:0_20:1) levels	3	166723434	C	A	-7.57E-02	1.68E-02	5.25E-01	rs595400	3_166723434_A_C	1.00	7148	6.59E-06	GCST90277413
Phosphatidylcholine (16:0_20:1) levels	3	196197114	C	T	1.64E-01	3.53E-02	6.09E-02	rs112214356	3_196197114_T_C	0.99	7148	3.46E-06	GCST90277413
Phosphatidylcholine (16:0_20:1) levels	4	75629568	A	G	-1.64E-01	3.69E-02	5.37E-02	rs80329613	4_75629568_G_A	0.99	7148	8.55E-06	GCST90277413
Phosphatidylcholine (16:0_20:1) levels	4	155035692	G	T	7.56E-02	1.66E-02	5.05E-01	rs1902483	4_155035692_T_G	1.00	7148	5.63E-06	GCST90277413
Phosphatidylcholine (16:0_20:1) levels	5	57685337	A	T	2.96E-01	6.58E-02	1.66E-02	rs72767411	5_57685337_T_A	0.96	7148	6.92E-06	GCST90277413
Phosphatidylcholine (16:0_20:1) levels	6	44048675	C	T	4.03E-01	8.93E-02	9.91E-03	rs115007623	6_44048675_T_C	0.90	7148	6.41E-06	GCST90277413
Phosphatidylcholine (16:0_20:1) levels	6	105463186	G	C	-1.72E-01	3.73E-02	6.05E-02	rs62419698	6_105463186_C_G	0.89	7148	3.81E-06	GCST90277413
Phosphatidylcholine (16:0_20:1) levels	6	134421023	A	G	1.45E-01	3.15E-02	8.06E-02	rs6904245	6_134421023_G_A	0.98	7148	4.28E-06	GCST90277413
Phosphatidylcholine (16:0_20:1) levels	8	19967357	A	G	-1.26E-01	1.92E-02	2.53E-01	rs3916027	8_19967357_G_A	1.00	7148	6.22E-11	GCST90277413
Phosphatidylcholine (16:0_20:1) levels	8	64694359	A	G	9.53E-02	2.11E-02	8.05E-01	rs7002672	8_64694359_G_A	1.00	7148	6.36E-06	GCST90277413
Phosphatidylcholine (16:0_20:1) levels	9	130364541	C	T	1.56E-01	3.25E-02	7.46E-02	rs75402627	9_130364541_T_C	0.98	7148	1.59E-06	GCST90277413
Phosphatidylcholine (16:0_20:1) levels	11	61811991	G	A	-1.61E-01	1.70E-02	4.06E-01	rs174554	11_61811991_A_G	1.00	7148	2.72E-21	GCST90277413
Phosphatidylcholine (16:0_20:1) levels	11	68794860	T	C	-1.21E-01	2.35E-02	1.56E-01	rs2229738	11_68794860_C_T	0.94	7148	2.81E-07	GCST90277413
Phosphatidylcholine (16:0_20:1) levels	11	116778201	C	G	-2.16E-01	2.31E-02	8.49E-01	rs964184	11_116778201_G_C	1.00	7148	1.24E-20	GCST90277413
Phosphatidylcholine (16:0_20:1) levels	12	129053394	C	T	7.93E-02	1.74E-02	5.64E-01	rs513272	12_129053394_T_C	0.95	7148	4.97E-06	GCST90277413
Phosphatidylcholine (16:0_20:1) levels	13	55008165	C	T	-1.33E-01	2.84E-02	9.69E-02	rs72623355	13_55008165_T_C	0.98	7148	2.64E-06	GCST90277413
Phosphatidylcholine (16:0_20:1) levels	14	36233601	G	C	1.22E-01	2.71E-02	1.14E-01	rs7156531	14_36233601_C_G	0.95	7148	6.40E-06	GCST90277413
Phosphatidylcholine (16:0_20:1) levels	14	74300719	T	C	-7.36E-01	1.63E-01	2.92E-03	rs148132478	14_74300719_C_T	0.89	7148	6.59E-06	GCST90277413
Phosphatidylcholine (16:0_20:1) levels	14	95049182	G	A	9.33E-02	2.11E-02	7.96E-01	rs10149017	14_95049182_A_G	0.99	7148	9.44E-06	GCST90277413
Phosphatidylcholine (16:0_20:1) levels	14	97502998	A	G	-1.10E-01	2.42E-02	8.62E-01	rs2626606	14_97502998_G_A	0.98	7148	5.80E-06	GCST90277413
Phosphatidylcholine (16:0_20:1) levels	15	59864454	G	A	-7.61E-02	1.69E-02	5.79E-01	rs7170012	15_59864454_A_G	1.00	7148	7.03E-06	GCST90277413

Phosphatidylcholine (16:0_20:1) levels	17	16960373 G	C	3.58E-01	8.10E-02	1.16E-02 rs34001747	17_16960373_C_G	0.90	7148	1.00E-05 GCST90277413
Phosphatidylcholine (16:0_20:1) levels	19	19347579 G	A	-2.71E-01	3.79E-02	5.38E-02 rs182611493	19_19347579_A_G	0.95	7148	8.42E-13 GCST90277413
Phosphatidylcholine (16:0_20:1) levels	19	19547663 T	G	-1.51E-01	3.36E-02	6.58E-02 rs16996148	19_19547663_G_T	1.00	7148	7.18E-06 GCST90277413
Phosphatidylcholine (16:0_20:1) levels	19	21770359 T	C	7.93E-02	1.72E-02	6.05E-01 rs57706029	19_21770359_C_T	0.99	7148	3.93E-06 GCST90277413
Phosphatidylcholine (16:0_20:1) levels	19	44912921 T	G	1.05E-01	1.96E-02	2.42E-01 rs483082	19_44912921_G_T	1.00	7148	7.96E-08 GCST90277413
Phosphatidylcholine (16:0_20:1) levels	22	28083968 A	G	1.33E-01	2.88E-02	9.43E-02 rs150578762	22_28083968_G_A	0.99	7148	4.11E-06 GCST90277413
Phosphatidylcholine (16:0_20:1) levels	22	43932163 C	T	8.79E-02	1.72E-02	3.91E-01 rs1883350	22_43932163_T_C	0.99	7148	3.37E-07 GCST90277413
Phosphatidylcholine (16:0_20:2) levels	1	109650768 G	A	8.17E-02	1.74E-02	5.95E-01 rs592586	1_109650768_A_G	0.99	6800	2.57E-06 GCST90277414
Phosphatidylcholine (16:0_20:2) levels	1	237378917 T	C	-2.85E-01	6.37E-02	1.99E-02 rs78095833	1_237378917_C_T	0.90	6800	8.05E-06 GCST90277414
Phosphatidylcholine (16:0_20:2) levels	2	27508073 C	T	-8.57E-02	1.80E-02	6.51E-01 rs1260326	2_27508073_T_C	1.00	6800	1.90E-06 GCST90277414
Phosphatidylcholine (16:0_20:2) levels	2	40100036 C	G	-1.70E-01	3.75E-02	5.62E-02 rs11892100	2_40100036_G_C	0.98	6800	5.67E-06 GCST90277414
Phosphatidylcholine (16:0_20:2) levels	3	35239965 A	G	4.99E-01	1.08E-01	6.81E-03 rs62258666	3_35239965_G_A	0.94	6800	3.69E-06 GCST90277414
Phosphatidylcholine (16:0_20:2) levels	3	166672944 A	G	-8.14E-02	1.73E-02	4.50E-01 rs7621165	3_166672944_G_A	1.00	6800	2.75E-06 GCST90277414
Phosphatidylcholine (16:0_20:2) levels	4	124768047 C	G	9.28E-02	2.03E-02	2.40E-01 rs2647110	4_124768047_G_C	0.99	6800	4.68E-06 GCST90277414
Phosphatidylcholine (16:0_20:2) levels	6	44395493 T	A	1.44E-01	3.23E-02	7.87E-02 rs4711782	6_44395493_A_T	1.00	6800	8.44E-06 GCST90277414
Phosphatidylcholine (16:0_20:2) levels	6	105463186 G	C	-1.84E-01	3.83E-02	6.05E-02 rs62419698	6_105463186_C_G	0.89	6800	1.62E-06 GCST90277414
Phosphatidylcholine (16:0_20:2) levels	6	137580166 C	T	-1.20E-01	2.64E-02	1.20E-01 rs13205789	6_137580166_T_C	0.99	6800	5.38E-06 GCST90277414
Phosphatidylcholine (16:0_20:2) levels	7	104552272 G	C	9.22E-02	1.92E-02	2.66E-01 rs10480656	7_104552272_C_G	0.99	6800	1.63E-06 GCST90277414
Phosphatidylcholine (16:0_20:2) levels	7	144901272 G	C	6.86E-01	1.52E-01	3.35E-03 rs141329532	7_144901272_C_G	0.89	6800	7.02E-06 GCST90277414
Phosphatidylcholine (16:0_20:2) levels	7	152575262 T	C	-9.70E-02	2.15E-02	2.01E-01 rs4726178	7_152575262_C_T	1.00	6800	6.81E-06 GCST90277414
Phosphatidylcholine (16:0_20:2) levels	8	19998749 G	T	-1.11E-01	1.92E-02	2.71E-01 rs2165558	8_19998749_T_G	1.00	6800	6.70E-09 GCST90277414
Phosphatidylcholine (16:0_20:2) levels	8	64695903 G	A	9.91E-02	2.11E-02	7.92E-01 rs7815372	8_64695903_A_G	1.00	6800	2.68E-06 GCST90277414
Phosphatidylcholine (16:0_20:2) levels	8	92429509 A	G	-7.92E-02	1.77E-02	3.68E-01 rs57588467	8_92429509_G_A	1.00	6800	7.59E-06 GCST90277414
Phosphatidylcholine (16:0_20:2) levels	9	70932129 A	G	-1.93E-01	4.27E-02	4.29E-02 rs11142645	9_70932129_G_A	0.97	6800	6.61E-06 GCST90277414
Phosphatidylcholine (16:0_20:2) levels	9	130374888 T	C	-1.47E-01	3.19E-02	9.20E-01 rs7031579	9_130374888_C_T	1.00	6800	4.18E-06 GCST90277414
Phosphatidylcholine (16:0_20:2) levels	11	61779120 G	A	-1.41E-01	1.75E-02	4.14E-01 rs174530	11_61779120_A_G	1.00	6800	9.01E-16 GCST90277414
Phosphatidylcholine (16:0_20:2) levels	11	68794860 T	C	-1.37E-01	2.41E-02	1.56E-01 rs2229738	11_68794860_C_T	0.94	6800	1.48E-08 GCST90277414
Phosphatidylcholine (16:0_20:2) levels	11	116778201 C	G	-1.94E-01	2.38E-02	8.49E-01 rs964184	11_116778201_G_C	1.00	6800	4.73E-16 GCST90277414
Phosphatidylcholine (16:0_20:2) levels	13	55008165 C	T	-1.32E-01	2.93E-02	9.69E-02 rs72623355	13_55008165_T_C	0.98	6800	6.70E-06 GCST90277414
Phosphatidylcholine (16:0_20:2) levels	13	73092472 C	A	-1.61E-01	3.48E-02	6.44E-02 rs61967369	13_73092472_A_C	1.00	6800	3.71E-06 GCST90277414
Phosphatidylcholine (16:0_20:2) levels	13	107454127 A	G	-2.92E-01	6.52E-02	1.82E-02 rs60543421	13_107454127_G_A	0.99	6800	7.35E-06 GCST90277414
Phosphatidylcholine (16:0_20:2) levels	14	78906415 A	T	-6.10E-01	1.20E-01	5.52E-03 rs61994001	14_78906415_T_A	0.99	6800	4.12E-07 GCST90277414
Phosphatidylcholine (16:0_20:2) levels	15	75426328 T	G	-1.03E-01	2.21E-02	1.88E-01 rs8028182	15_75426328_G_T	1.00	6800	3.45E-06 GCST90277414
Phosphatidylcholine (16:0_20:2) levels	15	82134529 G	A	-2.12E-01	4.64E-02	3.66E-02 rs117564891	15_82134529_A_G	0.98	6800	4.80E-06 GCST90277414
Phosphatidylcholine (16:0_20:2) levels	17	49540307 G	A	1.05E-01	2.27E-02	1.82E-01 rs11649869	17_49540307_A_G	0.96	6800	3.75E-06 GCST90277414
Phosphatidylcholine (16:0_20:2) levels	19	19347579 G	A	-2.59E-01	3.93E-02	5.38E-02 rs182611493	19_19347579_A_G	0.95	6800	5.15E-11 GCST90277414
Phosphatidylcholine (16:0_20:2) levels	19	19547663 T	G	-1.53E-01	3.45E-02	6.58E-02 rs16996148	19_19547663_G_T	1.00	6800	9.22E-06 GCST90277414
Phosphatidylcholine (16:0_20:2) levels	19	21770359 T	C	7.98E-02	1.76E-02	6.05E-01 rs57706029	19_21770359_C_T	0.99	6800	5.72E-06 GCST90277414
Phosphatidylcholine (16:0_20:2) levels	19	44913484 T	C	1.08E-01	2.01E-02	2.42E-01 rs438811	19_44913484_C_T	1.00	6800	8.66E-08 GCST90277414
Phosphatidylcholine (16:0_20:3) levels	1	109650768 G	A	9.12E-02	1.95E-02	5.95E-01 rs592586	1_109650768_A_G	0.99	5403	2.91E-06 GCST90277415
Phosphatidylcholine (16:0_20:3) levels	1	220806736 A	C	1.11E-01	2.50E-02	1.85E-01 rs34621709	1_220806736_C_A	1.00	5403	8.13E-06 GCST90277415
Phosphatidylcholine (16:0_20:3) levels	2	27508073 C	T	-1.19E-01	2.00E-02	6.51E-01 rs1260326	2_27508073_T_C	1.00	5403	2.66E-09 GCST90277415
Phosphatidylcholine (16:0_20:3) levels	6	112137404 C	T	1.08E-01	2.42E-02	1.95E-01 rs7764213	6_112137404_T_C	1.00	5403	7.83E-06 GCST90277415
Phosphatidylcholine (16:0_20:3) levels	6	138930517 T	G	-4.54E-01	1.01E-01	8.80E-03 rs117926062	6_138930517_G_T	0.97	5403	7.65E-06 GCST90277415
Phosphatidylcholine (16:0_20:3) levels	7	19617082 A	G	1.58E-01	3.33E-02	9.00E-01 rs12700064	7_19617082_G_A	0.93	5403	2.12E-06 GCST90277415
Phosphatidylcholine (16:0_20:3) levels	8	19967156 T	C	-1.23E-01	2.19E-02	2.61E-01 rs15285	8_19967156_C_T	1.00	5403	1.99E-08 GCST90277415
Phosphatidylcholine (16:0_20:3) levels	10	120774281 G	A	9.91E-02	2.08E-02	6.73E-01 rs3011337	10_120774281_A_G	0.98	5403	1.85E-06 GCST90277415
Phosphatidylcholine (16:0_20:3) levels	11	61790331 C	T	-1.59E-01	1.96E-02	4.10E-01 rs102275	11_61790331_T_C	1.00	5403	5.95E-16 GCST90277415
Phosphatidylcholine (16:0_20:3) levels	11	88458623 A	G	-1.22E-01	2.75E-02	1.41E-01 rs145956593	11_88458623_G_A	0.99	5403	8.97E-06 GCST90277415
Phosphatidylcholine (16:0_20:3) levels	11	116778201 C	G	-2.10E-01	2.63E-02	8.49E-01 rs964184	11_116778201_G_C	1.00	5403	1.38E-15 GCST90277415
Phosphatidylcholine (16:0_20:3) levels	12	31313616 T	G	1.80E-01	3.90E-02	9.31E-01 rs1258712	12_31313616_G_T	0.96	5403	4.08E-06 GCST90277415
Phosphatidylcholine (16:0_20:3) levels	12	43836589 G	A	1.25E-01	2.76E-02	1.38E-01 rs75214296	12_43836589_A_G	0.97	5403	5.94E-06 GCST90277415
Phosphatidylcholine (16:0_20:3) levels	14	76516128 T	G	-2.63E-01	5.32E-02	3.53E-02 rs141765432	14_76516128_G_T	0.93	5403	8.36E-07 GCST90277415
Phosphatidylcholine (16:0_20:3) levels	18	78936828 T	C	-2.82E-01	6.27E-02	2.39E-02 rs67076780	18_78936828_C_T	0.98	5403	6.79E-06 GCST90277415
Phosphatidylcholine (16:0_20:3) levels	19	19269704 G	A	-2.16E-01	4.49E-02	5.35E-02 rs187429064	19_19269704_A_G	0.95	5403	1.60E-06 GCST90277415
Phosphatidylcholine (16:0_20:3) levels	19	21652047 A	G	1.38E-01	3.02E-02	1.21E-01 rs111473971	19_21652047_G_A	0.95	5403	5.11E-06 GCST90277415
Phosphatidylcholine (16:0_20:3) levels	19	44912383 A	G	2.21E-01	3.54E-02	7.74E-02 rs445925	19_44912383_G_A	1.00	5403	5.08E-10 GCST90277415

Phosphatidylcholine (16:0_20:3) levels	20	13843242	C	T	8.79E-02	1.98E-02	3.54E-01	rs62209231	20_13843242_T_C	1.00	5403	9.08E-06	GCST90277415
Phosphatidylcholine (16:0_20:3) levels	20	44634395	A	G	-1.77E-01	3.68E-02	9.15E-01	rs371927	20_44634395_G_A	0.87	5403	1.50E-06	GCST90277415
Phosphatidylcholine (16:0_20:3) levels	22	28083968	A	G	1.55E-01	3.29E-02	9.43E-02	rs150578762	22_28083968_G_A	0.99	5403	2.52E-06	GCST90277415
Phosphatidylcholine (16:0_20:3) levels	22	43932163	C	T	1.14E-01	1.97E-02	3.91E-01	rs1883350	22_43932163_T_C	0.99	5403	6.13E-09	GCST90277415
Phosphatidylcholine (16:0_20:4) levels	1	109649355	A	C	9.29E-02	1.93E-02	7.05E-01	rs506912	1_109649355_C_A	0.99	6384	1.46E-06	GCST90277416
Phosphatidylcholine (16:0_20:4) levels	1	237429307	A	C	1.16E-01	2.59E-02	8.60E-01	rs6429013	1_237429307_C_A	0.99	6384	6.87E-06	GCST90277416
Phosphatidylcholine (16:0_20:4) levels	2	29060519	G	C	1.26E-01	2.80E-02	1.15E-01	rs12476545	2_29060519_C_G	0.98	6384	6.61E-06	GCST90277416
Phosphatidylcholine (16:0_20:4) levels	2	67277529	A	G	2.80E-01	6.21E-02	2.17E-02	rs79294676	2_67277529_G_A	0.98	6384	6.81E-06	GCST90277416
Phosphatidylcholine (16:0_20:4) levels	3	2139069	A	G	4.95E-01	1.07E-01	7.37E-03	rs116095614	3_2139069_G_A	0.94	6384	3.78E-06	GCST90277416
Phosphatidylcholine (16:0_20:4) levels	3	65548155	G	T	5.05E-01	1.07E-01	6.99E-03	rs67872791	3_65548155_T_G	0.99	6384	2.40E-06	GCST90277416
Phosphatidylcholine (16:0_20:4) levels	4	75860202	T	G	-2.06E-01	4.03E-02	5.02E-02	rs62321577	4_75860202_G_T	0.98	6384	3.13E-07	GCST90277416
Phosphatidylcholine (16:0_20:4) levels	6	105463186	G	C	-1.78E-01	3.94E-02	6.05E-02	rs62419698	6_105463186_C_G	0.89	6384	6.66E-06	GCST90277416
Phosphatidylcholine (16:0_20:4) levels	6	137598572	G	A	-1.24E-01	2.72E-02	1.20E-01	rs34718616	6_137598572_A_G	0.99	6384	5.68E-06	GCST90277416
Phosphatidylcholine (16:0_20:4) levels	6	162733959	A	G	-2.84E-01	6.41E-02	2.00E-02	rs151093994	6_162733959_G_A	0.95	6384	9.38E-06	GCST90277416
Phosphatidylcholine (16:0_20:4) levels	7	144901272	G	C	7.11E-01	1.60E-01	3.35E-03	rs141329532	7_144901272_C_G	0.89	6384	9.39E-06	GCST90277416
Phosphatidylcholine (16:0_20:4) levels	7	152575262	T	C	-1.06E-01	2.21E-02	2.01E-01	rs4726178	7_152575262_C_T	1.00	6384	1.54E-06	GCST90277416
Phosphatidylcholine (16:0_20:4) levels	8	19967357	A	G	-1.08E-01	2.04E-02	2.53E-01	rs3916027	8_19967357_G_A	1.00	6384	1.32E-07	GCST90277416
Phosphatidylcholine (16:0_20:4) levels	8	92462316	T	C	-8.73E-02	1.96E-02	2.88E-01	rs11776355	8_92462316_C_T	0.99	6384	8.51E-06	GCST90277416
Phosphatidylcholine (16:0_20:4) levels	9	19072292	A	C	-9.28E-02	1.92E-02	3.22E-01	rs112792028	9_19072292_C_A	0.98	6384	1.34E-06	GCST90277416
Phosphatidylcholine (16:0_20:4) levels	9	70920558	T	C	-1.83E-01	4.14E-02	4.87E-02	rs10521440	9_70920558_C_T	0.98	6384	9.72E-06	GCST90277416
Phosphatidylcholine (16:0_20:4) levels	9	126866252	G	C	-4.35E-01	9.75E-02	9.02E-03	rs143453636	9_126866252_C_G	0.95	6384	8.52E-06	GCST90277416
Phosphatidylcholine (16:0_20:4) levels	11	17897155	T	G	-6.78E-01	1.53E-01	3.22E-03	rs138578034	11_17897155_G_T	0.94	6384	9.37E-06	GCST90277416
Phosphatidylcholine (16:0_20:4) levels	11	61800281	A	C	-9.64E-02	1.84E-02	3.80E-01	rs174544	11_61800281_C_A	1.00	6384	1.74E-07	GCST90277416
Phosphatidylcholine (16:0_20:4) levels	11	68794860	T	C	-1.19E-01	2.50E-02	1.56E-01	rs2229738	11_68794860_C_T	0.94	6384	1.92E-06	GCST90277416
Phosphatidylcholine (16:0_20:4) levels	11	116778201	C	G	-1.71E-01	2.44E-02	8.49E-01	rs964184	11_116778201_G_C	1.00	6384	2.38E-12	GCST90277416
Phosphatidylcholine (16:0_20:4) levels	13	21648634	G	A	-2.80E-01	6.33E-02	2.13E-02	rs7336059	13_21648634_A_G	0.92	6384	9.87E-06	GCST90277416
Phosphatidylcholine (16:0_20:4) levels	13	73092472	C	A	-1.73E-01	3.60E-02	6.44E-02	rs61967369	13_73092472_A_C	1.00	6384	1.55E-06	GCST90277416
Phosphatidylcholine (16:0_20:4) levels	14	36553367	T	C	-1.14E-01	2.51E-02	1.40E-01	rs17765709	14_36553367_C_T	0.99	6384	6.02E-06	GCST90277416
Phosphatidylcholine (16:0_20:4) levels	15	58387979	C	T	8.13E-02	1.80E-02	3.95E-01	rs261291	15_58387979_T_C	1.00	6384	6.05E-06	GCST90277416
Phosphatidylcholine (16:0_20:4) levels	19	19347579	G	A	-2.44E-01	4.07E-02	5.38E-02	rs182611493	19_19347579_A_G	0.95	6384	2.40E-09	GCST90277416
Phosphatidylcholine (16:0_20:4) levels	19	44912383	A	G	1.59E-01	3.28E-02	7.74E-02	rs445925	19_44912383_G_A	1.00	6384	1.36E-06	GCST90277416
Phosphatidylcholine (16:0_20:4) levels	19	55460024	G	A	-8.91E-02	1.87E-02	3.93E-01	rs11880266	19_55460024_A_G	0.93	6384	2.00E-06	GCST90277416

Supplementary table 2

Used SNP Data

exposure	outcome	SNP	effect_allele.exposure	other_allele.exposure	effect_allele.outcome	other_allele.outcome	beta.exposure	beta.outcome	eaf.exposure	eaf.outcome	palindromic	ambiguous	chr.outcome	pos.outcome	se.outcome	pval.outcome	mr_keep.outcome	chr.exposure	pos.exposure	se.exposure	info.exposure	samplesize.exposure	pval.exposure	mr_keep.exposure	mr_keep	samplesize.outcome	F statistics
Sterol ester (27:1/14:0) levels	Parkinson's disease	rs10812660	A	T	A	T	7.90E-02	2.06E-02	3.75E-01	3.26E-01	TRUE	FALSE	9	27742535	2.93E-02	3.18E-01	TRUE	9	27742537	1.79E-02	0.93	7174	9.81E-06	TRUE	TRUE	482,730	19.58
Sterol ester (27:1/14:0) levels	Parkinson's disease	rs11608	A	G	A	G	8.85E-02	4.76E-02	7.23E-01	3.02E-01	FALSE	FALSE	2	27435374	1.84E-02	4.84E-03	TRUE	2	27212506	1.88E-02	1.00	7174	2.53E-06	TRUE	TRUE	482,730	22.18
Sterol ester (27:1/14:0) levels	Parkinson's disease	rs116778355	T	A	T	A	-3.80E-01	-9.20E-03	1.25E-02	2.18E-02	TRUE	FALSE	2	225816642	8.62E-02	3.85E-02	TRUE	2	224951925	7.77E-02	0.93	7174	1.00E-06	TRUE	TRUE	482,730	23.97
Sterol ester (27:1/14:0) levels	Parkinson's disease	rs12787616	C	T	C	T	7.71E-02	1.56E-02	3.73E-01	6.19E-01	FALSE	FALSE	11	26030069	2.32E-02	2.99E-01	TRUE	11	26008522	1.74E-02	0.99	7174	9.98E-06	TRUE	TRUE	482,730	19.54
Sterol ester (27:1/14:0) levels	Parkinson's disease	rs1696359	C	T	C	T	-7.96E-02	5.20E-03	5.85E-01	3.51E-01	FALSE	FALSE	12	121388559	1.86E-02	1.07E-01	TRUE	12	120950756	1.70E-02	1.00	7174	2.84E-06	TRUE	TRUE	482,730	21.95
Sterol ester (27:1/14:0) levels	Parkinson's disease	rs2479959	A	G	A	G	-8.03E-02	3.31E-02	3.00E-01	7.56E-01	FALSE	FALSE	13	111970433	2.62E-02	6.86E-01	TRUE	13	111318086	1.82E-02	1.00	7174	9.95E-06	TRUE	TRUE	482,730	19.55
Sterol ester (27:1/14:0) levels	Parkinson's disease	rs41289170	A	G	A	G	7.99E-01	3.42E-01	2.54E-03	9.85E-01	FALSE	FALSE	1	73256817	1.09E-01	8.80E-04	TRUE	1	72791134	1.79E-01	0.92	7174	8.30E-06	TRUE	TRUE	482,730	19.90
Sterol ester (27:1/14:0) levels	Parkinson's disease	rs4246215	T	G	T	G	-8.05E-02	-5.30E-03	4.36E-01	6.58E-01	FALSE	FALSE	11	61564299	1.79E-02	1.14E-01	TRUE	11	61796827	1.69E-02	0.99	7174	1.80E-06	TRUE	TRUE	482,730	22.83
Sterol ester (27:1/14:0) levels	Parkinson's disease	rs55764039	C	G	C	G	-1.81E-01	3.85E-02	5.27E-02	2.63E-02	TRUE	FALSE	7	17959690	7.21E-02	2.26E-01	TRUE	7	17920067	3.75E-02	0.99	7174	1.41E-06	TRUE	TRUE	482,730	23.30
Sterol ester (27:1/14:0) levels	Parkinson's disease	rs7198817	A	C	A	C	7.63E-02	-2.13E-02	5.78E-01	3.66E-01	FALSE	FALSE	16	11945778	1.81E-02	6.21E-01	TRUE	16	11851921	1.70E-02	1.00	7174	7.55E-06	TRUE	TRUE	482,730	20.08
Sterol ester (27:1/14:0) levels	Parkinson's disease	rs75922719	C	T	C	T	4.81E-01	-2.51E-01	6.84E-03	9.89E-01	FALSE	FALSE	15	94449395	1.66E-01	8.79E-01	TRUE	15	93906166	1.03E-01	0.95	7174	2.79E-06	TRUE	TRUE	482,730	21.99
Sterol ester (27:1/14:0) levels	Parkinson's disease	rs7957768	T	G	T	G	-8.68E-02	2.79E-02	5.21E-01	5.96E-01	FALSE	FALSE	12	102148319	1.87E-02	8.65E-01	TRUE	12	101754541	1.67E-02	1.00	7174	2.21E-07	TRUE	TRUE	482,730	26.88
Sterol ester (27:1/14:0) levels	Parkinson's disease	rs80195215	G	A	G	A	-1.87E-01	4.20E-03	4.66E-02	9.32E-01	FALSE	FALSE	10	123893275	4.72E-02	3.24E-02	TRUE	10	122133760	4.10E-02	0.94	7174	5.46E-06	TRUE	TRUE	482,730	20.70
Sterol ester (27:1/15:0) levels	Parkinson's disease	rs113844909	C	G	C	G	-3.30E-01	-2.12E-02	1.70E-02	2.71E-02	TRUE	FALSE	4	26518555	7.57E-02	1.08E-01	TRUE	4	26516933	7.03E-02	0.95	6428	2.77E-06	TRUE	TRUE	482,730	22.01
Sterol ester (27:1/15:0) levels	Parkinson's disease	rs115686032	T	C	T	C	-2.32E-01	6.00E-04	3.92E-02	9.82E-01	FALSE	FALSE	5	40282865	7.88E-02	2.83E-03	TRUE	5	40282763	4.57E-02	0.99	6428	3.88E-07	TRUE	TRUE	482,730	25.81
Sterol ester (27:1/15:0) levels	Parkinson's disease	rs12589429	T	C	T	C	-1.03E-01	-2.73E-02	1.82E-01	8.07E-01	FALSE	FALSE	14	48699122	2.49E-02	5.65E-01	TRUE	14	48229919	2.31E-02	0.99	6428	8.08E-06	TRUE	TRUE	482,730	19.95
Sterol ester (27:1/15:0) levels	Parkinson's disease	rs139857433	T	A	T	A	-4.60E-01	1.66E-01	8.71E-03	1.26E-02	TRUE	FALSE	2	98970522	1.13E-01	8.48E-01	TRUE	2	98354059	1.00E-01	0.92	6428	4.49E-06	TRUE	TRUE	482,730	21.08
Sterol ester (27:1/15:0) levels	Parkinson's disease	rs146236963	C	T	C	T	-3.27E-01	1.49E-01	1.65E-02	9.85E-01	FALSE	FALSE	7	103821281	1.01E-01	8.46E-01	TRUE	7	104180833	7.13E-02	0.98	6428	4.49E-06	TRUE	TRUE	482,730	21.08
Sterol ester (27:1/15:0) levels	Parkinson's disease	rs150680623	G	T	G	T	3.32E-01	4.70E-03	1.42E-02	9.67E-01	FALSE	FALSE	5	62891162	6.36E-02	2.61E-02	TRUE	5	63595335	7.38E-02	0.99	6428	7.08E-06	TRUE	TRUE	482,730	20.20
Sterol ester (27:1/15:0) levels	Parkinson's disease	rs17014799	C	T	C	T	4.62E-01	1.61E-01	7.74E-03	9.88E-01	FALSE	FALSE	1	209749541	1.07E-01	8.73E-01	TRUE	1	209576196	1.02E-01	1.00	6428	6.37E-06	TRUE	TRUE	482,730	20.41
Sterol ester (27:1/15:0) levels	Parkinson's disease	rs1999811	T	C	T	C	-8.55E-02	4.80E-03	6.91E-01	4.29E-01	FALSE	FALSE	1	31464327	2.27E-02	8.05E-02	TRUE	1	30991480	1.91E-02	1.00	6428	7.28E-06	TRUE	TRUE	482,730	20.15
Sterol ester (27:1/15:0) levels	Parkinson's disease	rs2100040	A	G	A	G	9.15E-02	1.51E-02	2.59E-01	7.52E-01	FALSE	FALSE	5	65134980	2.59E-02	2.53E-01	TRUE	5	65839152	2.01E-02	1.00	6428	5.57E-06	TRUE	TRUE	482,730	20.66
Sterol ester (27:1/15:0) levels	Parkinson's disease	rs2237478	T	C	T	C	-1.05E-01	-1.83E-02	1.98E-01	7.74E-01	FALSE	FALSE	7	50745794	2.36E-02	3.60E-01	TRUE	7	50678097	2.23E-02	1.00	6428	2.55E-06	TRUE	TRUE	482,730	22.17
Sterol ester (27:1/15:0) levels	Parkinson's disease	rs4246215	T	G	T	G	-8.40E-02	-5.30E-03	4.36E-01	6.58E-01	FALSE	FALSE	11	61564299	1.79E-02	1.14E-01	TRUE	11	61796827	1.78E-02	0.99	6428	2.52E-06	TRUE	TRUE	482,730	22.19
Sterol ester (27:1/15:0) levels	Parkinson's disease	rs55662279	T	C	T	C	-7.80E-01	7.91E-02	3.54E-03	9.79E-01	FALSE	FALSE	5	175204768	9.02E-02	4.19E-01	TRUE	5	175777765	1.66E-01	0.80	6428	2.72E-06	TRUE	TRUE	482,730	22.04
Sterol ester (27:1/15:0) levels	Parkinson's disease	rs61859797	T	C	T	C	1.09E-01	-5.57E-02	1.78E-01	8.76E-01	FALSE	FALSE	10	131259483	3.45E-02	9.73E-01	TRUE	10	129461219	2.32E-02	1.00	6428	2.63E-06	TRUE	TRUE	482,730	22.11
Sterol ester (27:1/15:0) levels	Parkinson's disease	rs77201364	A	G	A	G	3.67E-01	-1.89E-01	1.28E-02	9.94E-01	FALSE	FALSE	17	14567821	2.44E-01	3.57E-01	TRUE	17	14664504	8.16E-02	0.92	6428	6.93E-06	TRUE	TRUE	482,730	20.24
Sterol ester (27:1/15:0) levels	Parkinson's disease	rs930917	G	A	G	A	8.18E-02	2.37E-02	3.73E-01	7.09E-01	FALSE	FALSE	3	39889596	2.47E-02	4.70E-01	TRUE	3	39848105	1.82E-02	1.00	6428	7.39E-06	TRUE	TRUE	482,730	20.12
Sterol ester (27:1/15:0) levels	Parkinson's disease	rs985679	G	A	G	A	7.96E-02	2.55E-02	5.31E-01	4.90E-01	FALSE	FALSE	8	139453936	2.24E-02	5.96E-01	TRUE	8	138441693	1.76E-02	0.99	6428	6.64E-06	TRUE	TRUE	482,730	20.33
Sterol ester (27:1/16:0) levels	Parkinson's disease	rs10078182	C	T	C	T	2.68E-01	7.95E-02	2.18E-02	9.21E-01	FALSE	FALSE	5	118111042	4.34E-02	3.35E-02	TRUE	5	118775347	5.87E-02	0.95	7174	5.22E-06	TRUE	TRUE	482,730	20.79
Sterol ester (27:1/16:0) levels	Parkinson's disease	rs10795530	G	A	G	A	-7.61E-02	9.40E-03	5.80E-01	4.52E-01	FALSE	FALSE	10	7204878	2.40E-02	1.57E-01	TRUE	10	7162916	1.69E-02	1.00	7174	7.08E-06	TRUE	TRUE	482,730	20.20
Sterol ester (27:1/16:0) levels	Parkinson's disease	rs10841310	T	G	T	G	-9.60E-02	-8.70E-03	2.46E-01	7.56E-01	FALSE	FALSE	12	19830896	2.63E-02	1.30E-01	TRUE	12	19677962	1.94E-02	0.98	7174	7.87E-07	TRUE	TRUE	482,730	24.43
Sterol ester (27:1/16:0) levels	Parkinson's disease	rs10860778	T	C	T	C	-8.50E-02	2.58E-02	4.91E-01	5.82E-01	FALSE	FALSE	12	102108585	1.85E-02	7.86E-01	TRUE	12	101714807	1.67E-02	1.00	7174	3.85E-07	TRUE	TRUE	482,730	25.81
Sterol ester (27:1/16:0) levels	Parkinson's disease	rs11591147	T	G	T	G	-3.90E-01	-3.77E-02	3.32E-02	9.82E-01	FALSE	FALSE	1	55505647	6.83E-02	2.36E-01	TRUE	1	55039974	4.64E-02	1.00	7174	5.36E-17	TRUE	TRUE	482,730	70.55
Sterol ester (27:1/16:0) levels	Parkinson's disease	rs11604424	T	C	T	C	-1.05E-01	3.94E-02	7.53E-01	2.12E-01	FALSE	FALSE	11	116651115	2.06E-02	2.79E-02	TRUE	11	116780399	1.92E-02	1.00	7174	5.06E-08	TRUE	TRUE	482,730	29.76
Sterol ester (27:1/16:0) levels	Parkinson's disease	rs117010856	T	C	T	C	7.51E-01	-6.80E-02	2.76E-03	9.86E-01	FALSE	FALSE	10	53838961	1.01E-01	3.00E-01	TRUE	10	52079201	1.65E-01	0.86	7174	5.80E-06	TRUE	TRUE	482,730	20.58
Sterol ester (27:1/16:0) levels	Parkinson's disease	rs13085650	C	T	C	T	-8.70E-02	5.80E-03	2.52E-01	7.54E-01	FALSE	FALSE	3	76949295	2.65E-02	8.28E-02	TRUE	3	76900144	1.94E-02	0.99	7174	7.31E-06	TRUE	TRUE	482,730	20.14
Sterol ester (27:1/16:0) levels	Parkinson's disease	rs1336770	G	A	G	A	7.96E-02	-3.44E-02	5.86E-01	5.36E-01	FALSE	FALSE	6	124824016	2.24E-02	9.02E-01	TRUE	6	124502870	1.71E-02	0.99	7174	3.07E-06	TRUE	TRUE	482,730	21.80
Sterol ester (27:1/16:0) levels	Parkinson's disease	rs1367117	A	G	A	G	1.09E-01	6.00E-03	2.85E-01	6.89E-01	FALSE	FALSE	2	21263900	1.81E-02	1.31E-01	TRUE	2	21041028	1.84E-02	1.00	7174	2.74E-09	TRUE	TRUE	482,730	35.45
Sterol ester (27:1/16:0) levels	Parkinson's disease	rs138270540	C	A	C	A	3.30E-01	1.01E-01	2.81E-02	9.76E-01	FALSE	FALSE	4	75353427	8.13E-02	6.69E-01	TRUE	4	74487710	5.07E-02	0.96	7174	8.36E-11	TRUE	TRUE	482,730	42.29
Sterol ester (27:1/16:0) levels	Parkinson's disease	rs141836086	T	C	T	C	2.23E-01	7.45E-02	3.34E-02	9.83E-01	FALSE	FALSE	6	152971269	8.18E-02	4.41E-01	TRUE	6	152650134	4.63E-02	1.00	7174	1.48E-06	TRUE	TRUE	482,730	23.21
Sterol ester (27:1/16:0) levels	Parkinson's disease	rs174547	C	T	C	T	-2.06E-01	-3.30E-03	4.07E-01	6.72E-01	FALSE	FALSE	11	61570783	1.79E-02	6.93E-02	TRUE	11	61803311	1.68E-02	1.00	7174	4.77E-34	TRUE	TRUE	482,730	149.53
Sterol ester (27:1/16:0) levels	Parkinson's disease	rs2236514	G	C	G	C	-8.58E-02	2.80E-03	6.47E-01	6.35E-01	TRUE	FALSE	9	139572068	2.00E-02	5.10E-02	TRUE	9	136677616	1.77E-02	0.95	7174	1.38E-06	TRUE	TRUE	482,730	23.35
Sterol ester (27:1/16:0) levels	Parkinson's disease	rs28627829	G	T	G	T	-1.19E-01	3.25E-02	1.34E-01	8.74E-01	FALSE	FALSE	4	165560959	3.53E-02	4.46E-01	TRUE	4	164639807	2.47E-02	0.96	7174	1.63E-06	TRUE	TRUE	482,730	23.03
Sterol ester (27:1/16:0) levels	Parkinson's disease	rs3005923	A	G	A	G	-2.86E-01	-5.27E-02	2.76E-02	9.83E-01	FALSE	FALSE	1	56801542	1.69E-01	1.22E-01	TRUE	1	56335870	5.29E-02	0.92	7174	6.58E-08	TRUE	TRUE	482,730	29.24
Sterol ester (27:1/16:0) levels	Parkinson's disease	rs3017103	G	A	G	A	9.43E-02	1.33E-02	8.04E-01	1.78E-01	FALSE	FALSE	11	62174193	2.88E-02	1.90E-01	TRUE	11	62406721	2.11E-02	0.99	7174	8.15E-06	TRUE	TRUE	482,730	19.93
Sterol ester (27:1/16:0) levels	Parkinson's disease	rs538047	A	G	A	G	-7.35E-02	1.90E-03	4.63E-01	4.64E-01	FALSE	FALSE	11	88047189	1.96E-02	3.49E-02	TRUE	11	88314021	1.66E-02	1.00	7174	9.75E-06	TRUE	TRUE	482,730	19.59
Sterol ester (27:1/16:0) levels	Parkinson's disease	rs56767222	C	T	C	T	6.67E-01	-6.54E-02	3.36E-03	9.88E-01	FALSE	FALSE	3	3384570	1.47E-01	1.84E-01	TRUE	3	3342886	1.42E-01	0.99	7174	2.84E-06	TRUE	TRUE	482,730	21.95
Sterol ester (27:1/16:0) levels	Parkinson's disease	rs7167665	C	T	C	T	8.61E-02	2.37E-02	5.38E-01	4.99E-01	FALSE	FALSE	15	53750574	2.24E-02	5.35E-01	TRUE	15	53458377	1.67E-02	0.99	7174	2.37E-07	TRUE	TRUE	482,730	26.75
Sterol ester (27:1/16:0) levels	Parkinson's disease	rs7487904	G	C	G	C	1.09E-01	-1.47E-02	1.47E-01	9.63E-02	TRUE	FALSE	12	53624954	2.90E-02	2.13E-01	TRUE	12	53231170	2.37E-02	1.00	7174	3.94E-06	TRUE	TRUE	482,730	21.33
Sterol ester (27:1/16:0) levels	Parkinson's disease	rs74915447	A	G	A	G	2.47E-01	5.94E-02	3.78E-02	9.71E-01	FALSE	FALSE	4	73765477	7.20E-02	3.88E-01	TRUE	4	72899760	4.42E-02	0.97	7174	2.55E-08	TRUE	TRUE	482,730	31.09
Sterol ester (27:1/16:0) levels	Parkinson's disease	rs7517801	A	G	A	G	-2.56E-01	7.67E-02	2.24E-02	9.45E-01	FALSE	FALSE	1	154015682	4.28E-02	3.66E-02	TRUE	1	154043206	5.67E-02	1.00	7174	6.62E-06	TRUE	TRUE	482,730	20.33
Sterol ester (27:1/16:0) levels	Parkinson's disease	rs79146711	T	C	T	C	2.24E-01	-6.90E-03	4.51E-02	9.76E-01	FALSE	FALSE	4	74290628	8.45E-02	2.92E-02	TRUE	4	73424911	4.08E-02	0.94	7174	4.45E-08	TRUE	TRUE	482,730	30.01
Sterol ester (27:1/16:0) levels	Parkinson's disease	rs7932326	A	G	A	G	7.88E-02	1.61E-02	6.17E-01	4.05E-01	FALSE	FALSE	11	43993024	2.26E-02	3.21E-01	TRUE	11	43971474	1.71E-02	1.00	7174	4.13E-06	TRUE	TRUE	482,730	21.24
Sterol ester (27:1/16:0) levels	Parkinson's disease	rs9788072	G	A	G	A	1.70E-01	5.90E-03	5.63E-02	9.24E-01	FALSE	FALSE	12	4905214	4.47E-02	4.82E-02	TRUE	12	4796048	3.65E-02	0.95	7174	3.41E-06	TRUE	TRUE	482,730	21.61
Sterol ester (27:1/16:0) levels	Parkinson's disease	rs9924448	T	C	T	C	-8.45E-02	-1.70E-02	5.57E-01	4.76E-01	FALSE	FALSE	16	73383143	2.72E-02	2.73E-01	TRUE	16	73349244	1.70E-02	0.97	7174	7.15E-07	TRUE	TRUE	482,730	24.62
Sterol ester (27:1/16:1) levels	Parkinson's disease	rs10760286	C	T	C	T	8.28E-02	2.43E-02	5.10E-01	4.54E-01	FALSE	FALSE	9	126181588	2.11E-02	6.04E-01	TRUE	9	123419309	1.70E-02	0.97	7172	1.06E-06	TRUE	TRUE	482,730	23.85
Sterol ester (27:1/16:1) levels	Parkinson's disease	rs10812660	A	T	A	T	8.01E-02	2.06E-02	3.75E-01	3.26E-01	TRUE	FALSE	9	27742535	2.93E-02	3.18E-01	TRUE	9	27742537	1.78E-02	0.93	7172	7.26E-06	TRUE	TRUE	482,730	20.15
Sterol ester (27:1/16:1) levels	Parkinson's disease	rs11119973	A	C	A	C	1.11E-01	1.38E-02	1.53E-01	8.87E-01	FALSE	FALSE	1	212734714	3.15E-02	1.79E-01	TRUE	1	212561372	2.32E-02	1.00	7172	1.84E-06	TRUE	TRUE	482,730	22.79
Sterol ester (27:1/16:1) levels	Parkinson's disease	rs111656006	T	C	T	C	1.85E-01	1.87E-02	4.83E-02	9.11E-01	FALSE	FALSE	1	38711354	4.01E-02	1.94E-01	TRUE	1	38245682	4.00E-02	0.97	7172	3.82E-06	TRUE	TRUE	482,730	21.38
Sterol ester (27:1/16:1) levels	Parkinson's disease	rs113130033	G	A	G	A	-1.94E-01	-4.14E-02	4.30E-02	9.36E-01	FALSE	FALSE	14	90186309	4.77E-02	4.15E-01	TRUE	14	89719965	4.10E-02	0.98	7172	2.22E-06	TRUE	TRUE	482,730	22.43
Sterol ester (27:1/16:1) levels	Parkinson's disease	rs116522970	G	T	G	T	5.58E-01	3.65E-02	4.90E-03	9.83E-01	FALSE	FALSE	4	14331720	8.87E-02	1.67E-01	TRUE	4	14330096	1.24E-01	0.96	7172	7.01E-06	TRUE	TRUE	482,730	20.22
Sterol ester (27:1/16:1) levels	Parkinson's disease	rs116917626	T	C	T	C	-2.59E-01	1.84E-02	2.75E-02	9.86E-01	FALSE	FALSE	17	49639325	1.53E-01	4.36E-02	TRUE	17	51561964	5.25E-02	0.97	7172	7.87E-07	TRUE	TRUE	482,730	24.43
Sterol ester (27:1/16:1) levels	Parkinson's disease	rs1260326	C	T	C	T	-9.04E-02	-6.78E-02	6.51E-01	4.20E-01	FALSE	FALSE	2	27730940	1.72E-02	4.04E-05	TRUE	2	27508073	1.75E-02	1.00	7172	2.33E-07	TRUE	TRUE	482,730	26.79
Sterol ester (27:1/16:1) levels	Parkinson's disease	rs142738732	A	T	A	T	-1.66E-01	3.21E-02	6.34E-02	2.00E-02	TRUE	FALSE	1	1265106	8.43E-02	1.53E-01	TRUE	1	1329726	3.50E-02	0.96	7172	2.20E-06	TRUE	TRUE	482,730	22.45
Sterol ester (27:1/16:1) levels	Parkinson's disease	rs150352030	C	A	C	A	-6.45E-01	1.32E-01	3.78E-03	9.92E-01	FALSE	FALSE	17	33005262	1.48E-01	4.28E-01	TRUE	17	34678243	1.42E-01	0.95	7172	5.67E-06	TRUE	TRUE	482,730	20.63
Sterol ester (27:1/16:1) levels	Parkinson's disease	rs17603855	T	C	T	C	1.69E-01	-7.20E-03	5.75E-02	9.09E-01	FALSE	FALSE	7	137871779	4.05E-02	6.64E-02	TRUE	7	138187033	3.57E-02	0.97	7172	2.13E-06	TRUE	TRUE	482,730	22.51
Sterol ester (27:1/16:1) levels	Parkinson's disease	rs201714080	A	C	A	C	-1.88E-01	-2.31E-01	5.24E-02	9.82E-01	FALSE	FALSE	16	71129688	1.05E-01	1.36E-02	TRUE	16	71095785	3.81E-02	0.97	7172	7.60E-07	TRUE	TRUE	482,730	24.50
Sterol ester (27:1/16:1) levels	Parkinson's disease	rs2464190	C	T	C	T	7.64E-02	-1.42E-02	4.57E-01	5.75E-01	FALSE	FALSE	12	121415390	1.72E-02	3.90E-01	TRUE	12	120977587	1.68E-02	0.99	7172	5.25E-06	TRUE	TRUE	482,730	20.77
Sterol ester (27:1/16:1) levels	Parkinson's disease	rs274673	C	T	C	T	1.86E-01	4.90E-02	9.52E-01	2.74E-02	FALSE	FALSE	5	6743423	7.33E-02	2.97E-01	TRUE	5	6743310	3.93E-02	0.97	7172	2.24E-06	TRUE	TRUE	482,730	22.41
Sterol ester (27:1/16:1) levels	Parkinson's disease	rs4447106	A	G	A	G	1.18E-01	2.09E-02	1.41E-01	8.83E-01	FALSE	FALSE	10	102359760	2.92E-02	3.23E-01	TRUE	10	100600003	2.42E-02	0.98	7172	1.01E-06	TRUE	TRUE	482,730	23.94
Sterol ester (27:1/16:1) levels	Parkinson's disease	rs5022521	T	C	T	C	-1.08E-01	1.02E-02	7.97E-01	1.41E-01	FALSE	FALSE	4	111128587	4.02E-02	9.69E-02	TRUE	4	110207431	2.12E-02	0.94	7172	3.39E-07	TRUE	TRUE	482,730	26.06
Sterol ester (27:1/16:1) levels	Parkinson's disease	rs603424	A	G	A	G	-1.56E-01	6.90E-03	1.21E-01	8.02E-01	FALSE	FALSE	10	102075479	2.49E-02	1.07E-01	TRUE	10	100315722	2.60E-02	1.00	7172	2.07E-09	TRUE	TRUE	482,730	36.00
Sterol ester (27:1/16:1) levels	Parkinson's disease	rs7313803	T	C	T	C	8.73E-02	3.60E-03	2.60E-01	7.65E-01	FALSE	FALSE	12	88384450	2.65E-02	4.92E-02	TRUE	12	87990673	1.94E-02	0.95	7172	6.97E-06	TRUE	TRUE	482,730	20.23
Sterol ester (27:1/16:1) levels	Parkinson's disease	rs7355269	C	T	C	T	2.43E-01	-1.67E-02	2.61E-02	9.76E-01	FALSE	FALSE	2	62300074	5.86E-02	1.10E-01	TRUE	2	62072939	5.29E-02	0.99	7172	4.35E-06	TRUE	TRUE	482,730	21.14
Sterol ester (27:1/16:1) levels	Parkinson's disease	rs80227620	T	C	T	C	1.17E-01	-3.70E-03	1.44E-01	8.51E-01	FALSE	FALSE	2	168689139	4.02E-02	3.26E-02	TRUE	2	167832629	2.44E-02	0.95	7172	1.73E-06	TRUE	TRUE	482,730	22.91
Sterol ester (27:1/16:1) levels	Parkinson's disease	rs855500	C	T	C	T	7.62E-02	-1.77E-02	5.82E-01	3.78E-01	FALSE	FALSE	9	33614214	2.00E-02	4.26E-01	TRUE	9	33614216	1.69E-02	1.00	7172	6.24E-06	TRUE	TRUE	482,730	20.44
Sterol ester (27:1/17:0) levels	Parkinson's disease	rs1002079	G	C	G	C	-1.42E-01	5.98E-02	1.01E-01	1.07E-01	TRUE	FALSE	16	76498997	2.83E-02	1.73E-02	TRUE	16	76465100	2.80E-02	0.98	7047	3.89E-07	TRUE	TRUE	482,730	25.80
Sterol ester (27:1/17:0) levels	Parkinson's disease	rs10241012	G	A	G	A	1.18E-01	-5.80E-03	1.23E-01	8.82E-01	FALSE	FALSE	7	103975232	3.48E-02	6.20E-02	TRUE	7	104334784	2.59E-02	1.00	7047	4.98E-06	TRUE	TRUE	482,730	20.88
Sterol ester (27:1/17:0) levels	Parkinson's disease	rs10775274	C	T	C	T	1.70E-01	2.14E-01	9.38E-01	2.47E-02	FALSE	FALSE	16	25223796	8.62E-02	6.48E-03	TRUE	16	25212475	3.58E-02	0.94	7047	1.93E-06	TRUE	TRUE	482,730	22.70
Sterol ester (27:1/17:0) levels	Parkinson's disease	rs10881589	T	C	T	C	7.87E-02	3.64E-02	4.33E-01	6.61E-01	FALSE	FALSE	9	137135457	3.59E-02	5.07E-01	TRUE	9	134243611	1.76E-02	0.92	7047	8.37E-06	TRUE	TRUE	482,730	19.88
Sterol ester (27:1/17:0) levels	Parkinson's disease	rs111566713	T	C	T	C	-2.38E-01	3.66E-02	2.49E-02	9.72E-01	FALSE	FALSE	9	88796735	7.34E-02	2.09E-01	TRUE	9	86181820	5.35E-02	0.98	7047	8.77E-06	TRUE	TRUE	482,730	19.79
Sterol ester (27:1/17:0) levels	Parkinson's disease	rs11265455	G	A	G	A	1.19E-01	-4.00E-04	1.41E-01	8.49E-01	FALSE	FALSE	1	160620151	3.34E-02	4.23E-03	TRUE	1	160650361	2.56E-02	0.90	7047	3.30E-06	TRUE	TRUE	482,730	21.67
Sterol ester (27:1/17:0) levels	Parkinson's disease	rs113505726	A	G	A	G	-1.92E-01	3.60E-03	4.18E-02	9.58E-01	FALSE	FALSE	12	54432285	5.77E-02	2.21E-02	TRUE	12	54038501	4.22E-02	0.97	7047	5.11E-06	TRUE	TRUE	482,730	20.83
Sterol ester (27:1/17:0) levels	Parkinson's disease	rs116859210	G	A	G	A	3.51E-01	-5.00E-02	1.23E-02	9.76E-01	FALSE	FALSE	12	47129520	6.70E-02	3.42E-01	TRUE	12	46735737	7.69E-02	1.00	7047	5.04E-06	TRUE	TRUE	482,730	20.85
Sterol ester (27:1/17:0) levels	Parkinson's disease	rs12296850	G	A	G	A	1.73E-01	-7.28E-02	5.43E-02	9.34E-01	FALSE	FALSE	12	100820085	4.39E-02	4.86E-02	TRUE	12	100426307	3.76E-02	1.00	7047	4.26E-06	TRUE	TRUE	482,730	21.18
Sterol ester (27:1/17:0) levels	Parkinson's disease	rs12735602	A	C	A	C	7.59E-02	-2.91E-02	5.41E-01	4.40E-01	FALSE	FALSE	1	210494069	2.25E-02	7.11E-01	TRUE	1	210320724	1.71E-02	1.00	7047	9.09E-06	TRUE	TRUE	482,730	19.72
Sterol ester (27:1/17:0) levels	Parkinson's disease	rs12921107	C	T	C	T	-1.25E-01	2.63E-02	1.42E-01	8.26E-01	FALSE	FALSE	16	342437	2.27E-02	6.09E-01	TRUE	16	292437	2.42E-02	0.99	7047	2.38E-07	TRUE	TRUE	482,730	26.75
Sterol ester (27:1/17:0) levels	Parkinson's disease	rs13125535	G	T	G	T	8.61E-02	-7.90E-03	5.98E-01	3.92E-01	FALSE	FALSE	4	12265411	2.28E-02	1.37E-01	TRUE	4	12263787	1.72E-02	1.00	7047	5.61E-07	TRUE	TRUE	482,730	25.09
Sterol ester (27:1/17:0) levels	Parkinson's disease	rs140015624	C	T	C	T	3.23E-01	3.57E-02	1.58E-02	9.89E-01	FALSE	FALSE	11	71024702	1.32E-01	1.04E-01	TRUE	11	71313656	6.90E-02	0.93	7047	2.96E-06	TRUE	TRUE	482,730	21.88
Sterol ester (27:1/17:0) levels	Parkinson's disease	rs17791425	A	G	A	G	1.80E-01	-1.18E-01	5.00E-02	9.45E-01	FALSE	FALSE	6	84970341	4.91E-02	8.22E-03	TRUE	6	84260623	3.94E-02	0.97	7047	4.90E-06	TRUE	TRUE	482,730	20.91
Sterol ester (27:1/17:0) levels	Parkinson's disease	rs1868072	C	T	C	T	-8.29E-02	-8.30E-03	7.05E-01	2.22E-01	FALSE	FALSE	2	30403706	2.77E-02	1.17E-01	TRUE	2	30180840	1.86E-02	0.98	7047	8.47E-06	TRUE	TRUE	482,730	19.85
Sterol ester (27:1/17:0) levels	Parkinson's disease	rs2280696	T	A	T	A	2.04E-01	-3.97E-02	1.26E-01	6.97E-02	TRUE	FALSE	12	53512756	3.64E-02	5.60E-01	TRUE	12	53118972	2.52E-02	0.99	7047	6.37E-16	TRUE	TRUE	482,730	65.62
Sterol ester (27:1/17:0) levels	Parkinson's disease	rs2358779	A	G	A	G	-2.43E-01	-4.73E-02	9.71E-01	4.39E-02	FALSE	FALSE	16	31861962	4.80E-02	4.89E-01	TRUE	16	31850641	5.32E-02	0.91	7047	5.03E-06	TRUE	TRUE	482,730	20.86
Sterol ester (27:1/17:0) levels	Parkinson's disease	rs2382335	C	T	C	T	1.02E-01	-6.24E-02	8.35E-01	1.24E-01	FALSE	FALSE	9	12243520	3.40E-02	3.32E-02	TRUE	9	12243520	2.27E-02	1.00	7047	7.21E-06	TRUE	TRUE	482,730	20.16
Sterol ester (27:1/17:0) levels	Parkinson's disease	rs4246215	T	G	T	G	-8.46E-02	-5.30E-03	4.36E-01	6.58E-01	FALSE	FALSE	11	61564299	1.79E-02	1.14E-01	TRUE	11	61796827	1.70E-02	0.99	7047	6.70E-07	TRUE	TRUE	482,730	24.74
Sterol ester (27:1/17:0) levels	Parkinson's disease	rs4503592	G	T	G	T	-8.96E-02	-4.50E-03	2.22E-01	8.44E-01	FALSE	FALSE	12	9007161	2.75E-02	6.04E-02	TRUE	12	8854565	2.02E-02	0.99	7047	9.63E-06	TRUE	TRUE	482,730	19.61
Sterol ester (27:1/17:0) levels	Parkinson's disease	rs72945021	A	T	A	T	-9.97E-02	-5.58E-02	1.83E-01	9.86E-02	TRUE	FALSE	2	212530898	3.82E-02	8.40E-01	TRUE	2	211666173	2.18E-02	0.99	7047	5.01E-06	TRUE	TRUE	482,730	20.86
Sterol ester (27:1/17:0) levels	Parkinson's disease	rs77943189	G	A	G	A	3.36E-01	2.34E-02	1.60E-02	9.78E-01	FALSE	FALSE	6	122605322	1.06E-01	8.37E-02	TRUE	6	122284176	7.07E-02	0.91	7047	2.05E-06	TRUE	TRUE	482,730	22.59
Sterol ester (27:1/17:0) levels	Parkinson's disease	rs79528439	A	T	A	T	-1.32E-01	-2.56E-02	1.18E-01	1.08E-01	TRUE	FALSE	15	49001197	3.06E-02	3.94E-01	TRUE	15	48709000	2.66E-02	0.97	7047	7.60E-07	TRUE	TRUE	482,730	24.50
Sterol ester (27:1/17:0) levels	Parkinson's disease	rs79568254	A	T	A	T	-3.10E-01	-7.25E-02	1.49E-02	1.46E-02	TRUE	FALSE	4	137569283	1.00E-01	3.29E-01	TRUE	4	136648128	6.92E-02	0.98	7047	7.56E-06	TRUE	TRUE	482,730	20.07
Sterol ester (27:1/17:0) levels	Parkinson's disease	rs9827613	G	A	G	A	-7.54E-02	1.43E-02	4.61E-01	5.63E-01	FALSE	FALSE	3	11875444	2.47E-02	2.51E-01	TRUE	3	11833970	1.69E-02	0.99	7047	8.51E-06	TRUE	TRUE	482,730	19.85
Sterol ester (27:1/17:1) levels	Parkinson's disease	rs10746106	G	A	G	A	-8.45E-02	1.11E-02	2.72E-01	7.11E-01	FALSE	FALSE	12	79711360	2.49E-02	1.83E-01	TRUE	12	79317580	1.88E-02	0.99	7166	7.24E-06	TRUE	TRUE	482,730	20.16
Sterol ester (27:1/17:1) levels	Parkinson's disease	rs10812660	A	T	A	T	9.32E-02	2.06E-02	3.75E-01	3.26E-01	TRUE	FALSE	9	27742535	2.93E-02	3.18E-01	TRUE	9	27742537	1.79E-02	0.93	7166	1.91E-07	TRUE	TRUE	482,730	27.17
Sterol ester (27:1/17:1) levels	Parkinson's disease	rs11614909	A	C	A	C	-9.72E-02	-5.68E-02	1.86E-01	8.84E-01	FALSE	FALSE	12	69329696	3.53E-02	9.68E-01	TRUE	12	68935916	2.16E-02	0.99	7166	7.04E-06	TRUE	TRUE	482,730	20.21
Sterol ester (27:1/17:1) levels	Parkinson's disease	rs116778355	T	A	T	A	-3.57E-01	-9.20E-03	1.25E-02	2.18E-02	TRUE	FALSE	2	225816642	8.62E-02	3.85E-02	TRUE	2	224951925	7.79E-02	0.93	7166	4.61E-06	TRUE	TRUE	482,730	21.03
Sterol ester (27:1/17:1) levels	Parkinson's disease	rs117691424	A	G	A	G	-6.34E-01	5.18E-02	3.79E-03	9.92E-01	FALSE	FALSE	17	33030753	1.41E-01	1.47E-01	TRUE	17	34703734	1.42E-01	0.96	7166	8.36E-06	TRUE	TRUE	482,730	19.88
Sterol ester (27:1/17:1) levels	Parkinson's disease	rs217184	C	T	C	T	-9.29E-02	-4.18E-02	2.28E-01	8.02E-01	FALSE	FALSE	16	72105965	2.27E-02	3.28E-02	TRUE	16	72072066	1.99E-02	0.99	7166	3.24E-06	TRUE	TRUE	482,730	21.70
Sterol ester (27:1/17:1) levels	Parkinson's disease	rs6469013	G	A	G	A	8.22E-02	-1.40E-02	2.99E-01	6.86E-01	FALSE	FALSE	8	106746435	2.02E-02	3.10E-01	TRUE	8	105734207	1.83E-02	0.99	7166	7.16E-06	TRUE	TRUE	482,730	20.18
Sterol ester (27:1/17:1) levels	Parkinson's disease	rs7151696	T	C	T	C	7.80E-02	1.75E-02	5.70E-01	3.76E-01	FALSE	FALSE	14	23271437	2.22E-02	3.67E-01	TRUE	14	22802228	1.70E-02	1.00	7166	4.31E-06	TRUE	TRUE	482,730	21.15

Sterol ester (27:1/17:1) levels	Parkinson's disease	rs72723967	A	C	A	C	1.03E-01	9.20E-03	1.63E-01	8.16E-01	FALSE	FALSE	4	138733515	2.85E-02	1.26E-01	TRUE	4	137812361	2.28E-02	0.99	7166	5.81E-06	TRUE	TRUE	482,730	20.58
Sterol ester (27:1/17:1) levels	Parkinson's disease	rs77392374	C	T	C	T	-2.43E-01	8.91E-02	2.68E-02	9.73E-01	FALSE	FALSE	10	73046421	7.81E-02	5.96E-01	TRUE	10	71286664	5.33E-02	0.93	7166	5.17E-06	TRUE	TRUE	482,730	20.80
Sterol ester (27:1/17:1) levels	Parkinson's disease	rs79101108	A	G	A	G	2.18E-01	-1.00E-02	3.07E-02	9.57E-01	FALSE	FALSE	5	105203727	5.69E-02	6.51E-02	TRUE	5	105868026	4.91E-02	0.98	7166	9.05E-06	TRUE	TRUE	482,730	19.73
Sterol ester (27:1/18:0) levels	Parkinson's disease	rs10735853	C	T	C	T	-7.90E-02	-3.62E-02	4.82E-01	4.87E-01	FALSE	FALSE	12	54318146	1.96E-02	3.24E-02	TRUE	12	53924362	1.68E-02	1.00	7157	2.61E-06	TRUE	TRUE	482,730	22.11
Sterol ester (27:1/18:0) levels	Parkinson's disease	rs11130797	A	G	A	G	8.35E-02	2.96E-02	3.00E-01	7.21E-01	FALSE	FALSE	3	60914079	2.34E-02	6.85E-01	TRUE	3	60928407	1.82E-02	1.00	7157	4.54E-06	TRUE	TRUE	482,730	21.05
Sterol ester (27:1/18:0) levels	Parkinson's disease	rs11154240	C	T	C	T	9.41E-02	9.30E-03	7.83E-01	1.98E-01	FALSE	FALSE	6	124778360	2.77E-02	1.33E-01	TRUE	6	124457214	2.02E-02	1.00	7157	3.12E-06	TRUE	TRUE	482,730	21.77
Sterol ester (27:1/18:0) levels	Parkinson's disease	rs113270487	A	G	A	G	1.75E-01	1.26E-02	5.25E-02	9.31E-01	FALSE	FALSE	1	5393123	4.72E-02	1.03E-01	TRUE	1	5333063	3.83E-02	0.95	7157	5.00E-06	TRUE	TRUE	482,730	20.87
Sterol ester (27:1/18:0) levels	Parkinson's disease	rs11646327	G	A	G	A	9.33E-02	-3.00E-02	2.55E-01	7.01E-01	FALSE	FALSE	16	29128438	2.96E-02	5.06E-01	TRUE	16	29117117	1.92E-02	0.99	7157	1.24E-06	TRUE	TRUE	482,730	23.55
Sterol ester (27:1/18:0) levels	Parkinson's disease	rs13405349	T	G	T	G	-1.01E-01	4.17E-02	1.85E-01	8.95E-01	FALSE	FALSE	2	212524248	3.70E-02	5.85E-01	TRUE	2	211659523	2.16E-02	0.99	7157	3.35E-06	TRUE	TRUE	482,730	21.64
Sterol ester (27:1/18:0) levels	Parkinson's disease	rs141162511	T	C	T	C	2.67E-01	-1.48E-01	1.98E-02	9.90E-01	FALSE	FALSE	2	173626165	1.60E-01	4.48E-01	TRUE	2	172761437	5.99E-02	0.99	7157	8.21E-06	TRUE	TRUE	482,730	19.92
Sterol ester (27:1/18:0) levels	Parkinson's disease	rs144944992	T	C	T	C	4.95E-01	1.77E-02	9.43E-03	9.87E-01	FALSE	FALSE	1	164759815	1.18E-01	5.51E-02	TRUE	1	164790578	8.97E-02	0.96	7157	3.58E-08	TRUE	TRUE	482,730	30.43
Sterol ester (27:1/18:0) levels	Parkinson's disease	rs17181296	A	G	A	G	1.22E-01	7.50E-03	1.14E-01	9.48E-01	FALSE	FALSE	6	154438244	5.79E-02	4.72E-02	TRUE	6	154117109	2.70E-02	0.96	7157	6.76E-06	TRUE	TRUE	482,730	20.29
Sterol ester (27:1/18:0) levels	Parkinson's disease	rs17725246	C	T	C	T	9.71E-02	-3.92E-02	3.39E-01	6.89E-01	FALSE	FALSE	7	44581986	2.32E-02	4.55E-02	TRUE	7	44542387	1.77E-02	1.00	7157	4.14E-08	TRUE	TRUE	482,730	30.15
Sterol ester (27:1/18:0) levels	Parkinson's disease	rs2280696	T	A	T	A	2.54E-01	-3.97E-02	1.26E-01	6.97E-02	TRUE	FALSE	12	53512756	3.64E-02	5.60E-01	TRUE	12	53118972	2.49E-02	0.99	7157	3.69E-24	TRUE	TRUE	482,730	103.53
Sterol ester (27:1/18:0) levels	Parkinson's disease	rs30743	C	T	C	T	-9.51E-02	1.33E-02	7.63E-01	2.36E-01	FALSE	FALSE	5	135332571	2.76E-02	2.00E-01	TRUE	5	135996882	2.04E-02	0.92	7157	2.99E-06	TRUE	TRUE	482,730	21.86
Sterol ester (27:1/18:0) levels	Parkinson's disease	rs366590	A	G	A	G	8.36E-02	-4.80E-03	2.88E-01	6.88E-01	FALSE	FALSE	11	16872440	2.06E-02	8.77E-02	TRUE	11	16850893	1.87E-02	0.98	7157	7.83E-06	TRUE	TRUE	482,730	20.01
Sterol ester (27:1/18:0) levels	Parkinson's disease	rs3741298	T	C	T	C	-1.09E-01	4.06E-02	7.65E-01	1.98E-01	FALSE	FALSE	11	116657561	2.12E-02	2.77E-02	TRUE	11	116786845	1.96E-02	1.00	7157	3.09E-08	TRUE	TRUE	482,730	30.72
Sterol ester (27:1/18:0) levels	Parkinson's disease	rs4246215	T	G	T	G	-1.11E-01	-5.30E-03	4.36E-01	6.58E-01	FALSE	FALSE	11	61564299	1.79E-02	1.14E-01	TRUE	11	61796827	1.69E-02	0.99	7157	4.47E-11	TRUE	TRUE	482,730	43.53
Sterol ester (27:1/18:0) levels	Parkinson's disease	rs55721138	T	C	T	C	-1.81E-01	6.59E-02	5.32E-02	9.64E-01	FALSE	FALSE	3	13624799	6.49E-02	5.09E-01	TRUE	3	13583299	3.76E-02	0.96	7157	1.53E-06	TRUE	TRUE	482,730	23.15
Sterol ester (27:1/18:0) levels	Parkinson's disease	rs56228609	T	C	T	C	-1.04E-01	8.20E-03	2.76E-01	6.95E-01	FALSE	FALSE	16	56987765	1.83E-02	1.83E-01	TRUE	16	56953853	1.87E-02	1.00	7157	2.73E-08	TRUE	TRUE	482,730	30.96
Sterol ester (27:1/18:0) levels	Parkinson's disease	rs7130086	A	C	A	C	-1.03E-01	-2.40E-03	1.60E-01	8.09E-01	FALSE	FALSE	11	48239182	2.20E-02	4.01E-02	TRUE	11	48217630	2.25E-02	1.00	7157	4.68E-06	TRUE	TRUE	482,730	21.00
Sterol ester (27:1/18:0) levels	Parkinson's disease	rs7425339	A	T	A	T	7.87E-02	-2.66E-02	6.23E-01	7.15E-01	TRUE	FALSE	2	163656912	2.49E-02	5.46E-01	TRUE	2	162800402	1.74E-02	1.00	7157	6.17E-06	TRUE	TRUE	482,730	20.46
Sterol ester (27:1/18:1) levels	Parkinson's disease	rs10078182	C	T	C	T	2.68E-01	7.95E-02	2.18E-02	9.21E-01	FALSE	FALSE	5	118111042	4.34E-02	3.35E-02	TRUE	5	118775347	5.87E-02	0.95	7174	4.93E-06	TRUE	TRUE	482,730	20.90
Sterol ester (27:1/18:1) levels	Parkinson's disease	rs1061808	G	T	G	T	-8.14E-02	-2.62E-02	5.84E-01	3.18E-01	FALSE	FALSE	6	32136547	2.05E-02	6.96E-01	TRUE	6	32168770	1.69E-02	1.00	7174	1.47E-06	TRUE	TRUE	482,730	23.22
Sterol ester (27:1/18:1) levels	Parkinson's disease	rs10812660	A	T	A	T	7.99E-02	2.06E-02	3.75E-01	3.26E-01	TRUE	FALSE	9	27742535	2.93E-02	3.18E-01	TRUE	9	27742537	1.78E-02	0.93	7174	7.44E-06	TRUE	TRUE	482,730	20.11
Sterol ester (27:1/18:1) levels	Parkinson's disease	rs10841310	T	G	T	G	-1.06E-01	-8.70E-03	2.46E-01	7.56E-01	FALSE	FALSE	12	19830896	2.63E-02	1.30E-01	TRUE	12	19677962	1.94E-02	0.98	7174	5.70E-08	TRUE	TRUE	482,730	29.52
Sterol ester (27:1/18:1) levels	Parkinson's disease	rs10860778	T	C	T	C	-8.15E-02	2.58E-02	4.91E-01	5.82E-01	FALSE	FALSE	12	102108585	1.85E-02	7.86E-01	TRUE	12	101714807	1.67E-02	1.00	7174	1.16E-06	TRUE	TRUE	482,730	23.69
Sterol ester (27:1/18:1) levels	Parkinson's disease	rs114483871	T	C	T	C	2.34E-01	-2.94E-02	3.81E-02	9.77E-01	FALSE	FALSE	4	73990168	8.47E-02	1.38E-01	TRUE	4	73124451	4.49E-02	0.93	7174	1.85E-07	TRUE	TRUE	482,730	27.23
Sterol ester (27:1/18:1) levels	Parkinson's disease	rs115419570	T	C	T	C	2.95E-01	3.21E-02	2.04E-02	9.79E-01	FALSE	FALSE	2	157378956	8.38E-02	1.54E-01	TRUE	2	156522444	5.97E-02	0.98	7174	7.75E-07	TRUE	TRUE	482,730	24.46
Sterol ester (27:1/18:1) levels	Parkinson's disease	rs11588501	G	C	G	C	2.42E-01	-3.52E-02	2.45E-02	2.05E-02	TRUE	FALSE	1	56998623	8.42E-02	1.70E-01	TRUE	1	56532951	5.46E-02	0.97	7174	9.06E-06	TRUE	TRUE	482,730	19.73
Sterol ester (27:1/18:1) levels	Parkinson's disease	rs11591147	T	G	T	G	-2.98E-01	-3.77E-02	3.32E-02	9.82E-01	FALSE	FALSE	1	55505647	6.83E-02	2.36E-01	TRUE	1	55039974	4.66E-02	1.00	7174	1.63E-10	TRUE	TRUE	482,730	40.98
Sterol ester (27:1/18:1) levels	Parkinson's disease	rs11687710	C	T	C	T	8.95E-02	2.70E-03	2.57E-01	6.88E-01	FALSE	FALSE	2	21371068	1.92E-02	5.23E-02	TRUE	2	21148196	1.92E-02	1.00	7174	3.09E-06	TRUE	TRUE	482,730	21.80
Sterol ester (27:1/18:1) levels	Parkinson's disease	rs12127700	G	A	G	A	7.91E-02	-1.95E-02	4.55E-01	5.81E-01	FALSE	FALSE	1	38731439	2.54E-02	3.54E-01	TRUE	1	38265767	1.78E-02	0.88	7174	9.36E-06	TRUE	TRUE	482,730	19.67
Sterol ester (27:1/18:1) levels	Parkinson's disease	rs151274583	A	C	A	C	2.88E-01	-2.61E-02	1.76E-02	9.81E-01	FALSE	FALSE	9	119712968	8.67E-02	1.17E-01	TRUE	9	116950689	6.30E-02	0.95	7174	4.99E-06	TRUE	TRUE	482,730	20.87
Sterol ester (27:1/18:1) levels	Parkinson's disease	rs182695896	C	A	C	A	3.38E-01	1.53E-01	2.53E-02	9.84E-01	FALSE	FALSE	4	74813227	1.83E-01	3.94E-01	TRUE	4	73947510	5.30E-02	0.96	7174	1.78E-10	TRUE	TRUE	482,730	40.81
Sterol ester (27:1/18:1) levels	Parkinson's disease	rs41441450	T	C	T	C	-3.84E-01	8.84E-02	9.82E-03	9.87E-01	FALSE	FALSE	5	86013053	1.02E-01	4.15E-01	TRUE	5	86717236	8.52E-02	0.99	7174	6.70E-06	TRUE	TRUE	482,730	20.30
Sterol ester (27:1/18:1) levels	Parkinson's disease	rs4845593	C	T	C	T	-2.90E-01	7.16E-02	2.22E-02	9.43E-01	FALSE	FALSE	1	154050499	4.23E-02	4.53E-02	TRUE	1	154078023	5.71E-02	1.00	7174	3.80E-07	TRUE	TRUE	482,730	25.84
Sterol ester (27:1/18:1) levels	Parkinson's disease	rs4906111	C	T	C	T	-2.46E-01	-6.97E-02	2.43E-02	9.22E-01	FALSE	FALSE	14	101859654	4.21E-02	4.89E-02	TRUE	14	101393317	5.41E-02	1.00	7174	5.51E-06	TRUE	TRUE	482,730	20.68
Sterol ester (27:1/18:1) levels	Parkinson's disease	rs701081	G	A	G	A	-8.36E-02	2.75E-02	6.03E-01	4.14E-01	FALSE	FALSE	12	125147969	2.28E-02	6.43E-01	TRUE	12	124663423	1.71E-02	0.99	7174	1.09E-06	TRUE	TRUE	482,730	23.80
Sterol ester (27:1/18:1) levels	Parkinson's disease	rs73176681	G	A	G	A	1.91E-01	3.50E-02	4.56E-02	9.65E-01	FALSE	FALSE	13	32074965	6.22E-02	2.41E-01	TRUE	13	31500828	4.04E-02	0.98	7174	2.33E-06	TRUE	TRUE	482,730	22.34
Sterol ester (27:1/18:1) levels	Parkinson's disease	rs7487904	G	C	G	C	1.28E-01	-1.47E-02	1.47E-01	9.63E-02	TRUE	FALSE	12	53624954	2.90E-02	2.13E-01	TRUE	12	53231170	2.37E-02	1.00	7174	7.01E-08	TRUE	TRUE	482,730	29.12
Sterol ester (27:1/18:1) levels	Parkinson's disease	rs74915447	A	G	A	G	2.63E-01	5.94E-02	3.78E-02	9.71E-01	FALSE	FALSE	4	73765477	7.20E-02	3.88E-01	TRUE	4	72899760	4.43E-02	0.97	7174	2.80E-09	TRUE	TRUE	482,730	35.41
Sterol ester (27:1/18:1) levels	Parkinson's disease	rs7804103	A	T	A	T	-1.59E-01	-2.67E-02	6.17E-02	7.83E-02	TRUE	FALSE	7	19980419	3.33E-02	3.74E-01	TRUE	7	19940796	3.48E-02	1.00	7174	5.05E-06	TRUE	TRUE	482,730	20.85
Sterol ester (27:1/18:1) levels	Parkinson's disease	rs7932326	A	G	A	G	7.72E-02	1.61E-02	6.17E-01	4.05E-01	FALSE	FALSE	11	43993024	2.26E-02	3.21E-01	TRUE	11	43971474	1.71E-02	1.00	7174	6.56E-06	TRUE	TRUE	482,730	20.35
Sterol ester (27:1/18:1) levels	Parkinson's disease	rs9924448	T	C	T	C	-7.74E-02	-1.70E-02	5.57E-01	4.76E-01	FALSE	FALSE	16	73383143	2.72E-02	2.73E-01	TRUE	16	73349244	1.71E-02	0.97	7174	5.93E-06	TRUE	TRUE	482,730	20.54
Sterol ester (27:1/18:2) levels	Parkinson's disease	rs10796911	C	G	C	G	1.17E-01	-5.53E-02	8.65E-01	8.46E-01	TRUE	FALSE	1	38088567	2.99E-02	3.22E-02	TRUE	1	37622895	2.46E-02	1.00	7174	2.06E-06	TRUE	TRUE	482,730	22.57
Sterol ester (27:1/18:2) levels	Parkinson's disease	rs11591147	T	G	T	G	-3.58E-01	-3.77E-02	3.32E-02	9.82E-01	FALSE	FALSE	1	55505647	6.83E-02	2.36E-01	TRUE	1	55039974	4.66E-02	1.00	7174	1.69E-14	TRUE	TRUE	482,730	59.11
Sterol ester (27:1/18:2) levels	Parkinson's disease	rs11604424	T	C	T	C	-9.73E-02	3.94E-02	7.53E-01	2.12E-01	FALSE	FALSE	11	116651115	2.06E-02	2.79E-02	TRUE	11	116780399	1.92E-02	1.00	7174	4.37E-07	TRUE	TRUE	482,730	25.57
Sterol ester (27:1/18:2) levels	Parkinson's disease	rs116302332	T	C	T	C	3.46E-01	1.00E-01	2.81E-02	9.75E-01	FALSE	FALSE	4	75370891	8.11E-02	6.62E-01	TRUE	4	74505174	5.09E-02	0.96	7174	1.17E-11	TRUE	TRUE	482,730	46.16
Sterol ester (27:1/18:2) levels	Parkinson's disease	rs117806675	A	G	A	G	-4.63E-01	-1.71E-01	7.54E-03	9.87E-01	FALSE	FALSE	10	5426504	1.08E-01	9.46E-01	TRUE	10	5384541	9.83E-02	0.98	7174	2.58E-06	TRUE	TRUE	482,730	22.14
Sterol ester (27:1/18:2) levels	Parkinson's disease	rs144064722	G	A	G	A	2.39E-01	-1.15E-01	3.24E-02	9.77E-01	FALSE	FALSE	4	73406173	7.75E-02	8.62E-01	TRUE	4	72540456	4.75E-02	0.98	7174	4.81E-07	TRUE	TRUE	482,730	25.38
Sterol ester (27:1/18:2) levels	Parkinson's disease	rs144863685	C	A	C	A	5.00E-01	-1.16E-01	6.10E-03	9.83E-01	FALSE	FALSE	16	88835245	8.35E-02	7.82E-01	TRUE	16	88768837	1.10E-01	0.91	7174	6.12E-06	TRUE	TRUE	482,730	20.48
Sterol ester (27:1/18:2) levels	Parkinson's disease	rs17395333	G	A	G	A	1.04E-01	3.00E-03	2.78E-01	6.89E-01	FALSE	FALSE	2	21323369	1.91E-02	5.74E-02	TRUE	2	21100497	1.87E-02	1.00	7174	3.06E-08	TRUE	TRUE	482,730	30.74
Sterol ester (27:1/18:2) levels	Parkinson's disease	rs2521811	C	T	C	T	-8.26E-02	6.85E-02	7.14E-01	3.09E-01	FALSE	FALSE	17	43934044	2.35E-02	1.78E-03	TRUE	17	45856678	1.84E-02	0.98	7174	7.51E-06	TRUE	TRUE	482,730	20.09
Sterol ester (27:1/18:2) levels	Parkinson's disease	rs2524299	T	A	T	A	1.39E-01	-2.85E-02	2.59E-01	1.26E-01	TRUE	FALSE	11	61604782	2.56E-02	5.76E-01	TRUE	11	61837310	1.90E-02	1.00	7174	3.25E-13	TRUE	TRUE	482,730	53.25
Sterol ester (27:1/18:2) levels	Parkinson's disease	rs61796597	T	C	T	C	-2.09E-01	1.57E-02	3.76E-02	9.57E-01	FALSE	FALSE	4	36229816	5.58E-02	1.09E-01	TRUE	4	36228194	4.46E-02	0.96	7174	2.97E-06	TRUE	TRUE	482,730	21.87
Sterol ester (27:1/18:2) levels	Parkinson's disease	rs61941338	T	C	T	C	2.24E-01	3.64E-02	3.22E-02	9.57E-01	FALSE	FALSE	12	112815622	6.04E-02	2.62E-01	TRUE	12	112377818	4.91E-02	0.94	7174	5.44E-06	TRUE	TRUE	482,730	20.71
Sterol ester (27:1/18:2) levels	Parkinson's disease	rs7012814	A	G	A	G	7.86E-02	-3.76E-02	5.12E-01	5.23E-01	FALSE	FALSE	8	9173358	2.54E-02	8.59E-01	TRUE	8	9315848	1.69E-02	0.99	7174	3.22E-06	TRUE	TRUE	482,730	21.72
Sterol ester (27:1/18:2) levels	Parkinson's disease	rs73327386	C	T	C	T	-6.38E-01	2.27E-02	3.72E-03	9.85E-01	FALSE	FALSE	5	171910795	1.09E-01	7.84E-02	TRUE	5	172483791	1.38E-01	0.96	7174	3.66E-06	TRUE	TRUE	482,730	21.47
Sterol ester (27:1/18:2) levels	Parkinson's disease	rs79146711	T	C	T	C	2.84E-01	-6.90E-03	4.51E-02	9.76E-01	FALSE	FALSE	4	74290628	8.45E-02	2.92E-02	TRUE	4	73424911	4.09E-02	0.94	7174	4.02E-12	TRUE	TRUE	482,730	48.28
Sterol ester (27:1/18:2) levels	Parkinson's disease	rs7932326	A	G	A	G	7.57E-02	1.61E-02	6.17E-01	4.05E-01	FALSE	FALSE	11	43993024	2.26E-02	3.21E-01	TRUE	11	43971474	1.71E-02	1.00	7174	9.93E-06	TRUE	TRUE	482,730	19.55
Sterol ester (27:1/18:2) levels	Parkinson's disease	rs80339463	T	C	T	C	3.25E-01	4.17E-02	1.45E-02	9.58E-01	FALSE	FALSE	12	19921605	5.96E-02	3.15E-01	TRUE	12	19768671	7.26E-02	0.98	7174	7.90E-06	TRUE	TRUE	482,730	19.99
Sterol ester (27:1/18:2) levels	Parkinson's disease	rs9330455	A	C	A	C	1.06E-01	-1.35E-02	8.28E-01	2.05E-01	FALSE	FALSE	9	92517624	3.19E-02	1.72E-01	TRUE	9	89816177	2.31E-02	0.92	7174	4.30E-06	TRUE	TRUE	482,730	21.16
Sterol ester (27:1/18:2) levels	Parkinson's disease	rs9405846	G	A	G	A	7.76E-02	-1.24E-02	5.85E-01	3.84E-01	FALSE	FALSE	6	5542407	2.33E-02	2.27E-01	TRUE	6	5542174	1.69E-02	0.99	7174	4.63E-06	TRUE	TRUE	482,730	21.02
Sterol ester (27:1/18:3) levels	Parkinson's disease	rs113130033	G	A	G	A	-2.17E-01	-4.14E-02	4.30E-02	9.36E-01	FALSE	FALSE	14	90186309	4.77E-02	4.15E-01	TRUE	14	89719965	4.10E-02	0.98	7174	1.22E-07	TRUE	TRUE	482,730	28.05
Sterol ester (27:1/18:3) levels	Parkinson's disease	rs11591147	T	G	T	G	-2.25E-01	-3.77E-02	3.32E-02	9.82E-01	FALSE	FALSE	1	55505647	6.83E-02	2.36E-01	TRUE	1	55039974	4.67E-02	1.00	7174	1.47E-06	TRUE	TRUE	482,730	23.23
Sterol ester (27:1/18:3) levels	Parkinson's disease	rs12199598	T	C	T	C	5.01E-01	-7.33E-02	5.84E-03	9.82E-01	FALSE	FALSE	6	95163440	9.01E-02	3.81E-01	TRUE	6	94453722	1.13E-01	0.95	7174	8.72E-06	TRUE	TRUE	482,730	19.80
Sterol ester (27:1/18:3) levels	Parkinson's disease	rs1260326	C	T	C	T	-1.05E-01	-6.78E-02	6.51E-01	4.20E-01	FALSE	FALSE	2	27730940	1.72E-02	4.04E-05	TRUE	2	27508073	1.75E-02	1.00	7174	1.64E-09	TRUE	TRUE	482,730	36.45
Sterol ester (27:1/18:3) levels	Parkinson's disease	rs137900618	G	T	G	T	6.05E-01	1.07E-01	4.45E-03	9.94E-01	FALSE	FALSE	2	137397881	2.00E-01	2.27E-01	TRUE	2	136640311	1.32E-01	0.97	7174	5.00E-06	TRUE	TRUE	482,730	20.87
Sterol ester (27:1/18:3) levels	Parkinson's disease	rs146997505	G	A	G	A	2.97E-01	1.38E-02	1.67E-02	9.73E-01	FALSE	FALSE	13	51784733	6.96E-02	7.44E-02	TRUE	13	51210597	6.62E-02	0.96	7174	7.42E-06	TRUE	TRUE	482,730	20.11
Sterol ester (27:1/18:3) levels	Parkinson's disease	rs174584	A	G	A	G	-1.83E-01	-8.90E-03	4.10E-01	6.56E-01	FALSE	FALSE	11	61610750	1.77E-02	2.10E-01	TRUE	11	61843278	1.69E-02	1.00	7174	4.02E-27	TRUE	TRUE	482,730	117.28
Sterol ester (27:1/18:3) levels	Parkinson's disease	rs1980102	A	C	A	C	8.11E-02	-1.30E-03	3.75E-01	6.35E-01	FALSE	FALSE	15	33618942	2.32E-02	1.98E-02	TRUE	15	33326741	1.71E-02	1.00	7174	2.27E-06	TRUE	TRUE	482,730	22.39
Sterol ester (27:1/18:3) levels	Parkinson's disease	rs2255531	A	G	A	G	9.34E-02	-1.31E-02	3.95E-01	6.41E-01	FALSE	FALSE	12	121414915	1.76E-02	3.40E-01	TRUE	12	120977112	1.70E-02	1.00	7174	4.25E-08	TRUE	TRUE	482,730	30.10
Sterol ester (27:1/18:3) levels	Parkinson's disease	rs319552	C	G	C	G	8.52E-02	-6.00E-03	2.55E-01	2.82E-01	TRUE	FALSE	13	93826052	3.07E-02	7.26E-02	TRUE	13	93173799	1.93E-02	0.96	7174	9.76E-06	TRUE	TRUE	482,730	19.58
Sterol ester (27:1/18:3) levels	Parkinson's disease	rs34500711	T	C	T	C	2.10E-01	-3.48E-02	3.81E-02	9.56E-01	FALSE	FALSE	9	132864264	5.89E-02	2.56E-01	TRUE	9	130101985	4.36E-02	0.96	7174	1.59E-06	TRUE	TRUE	482,730	23.08
Sterol ester (27:1/18:3) levels	Parkinson's disease	rs62294915	C	T	C	T	1.88E-01	-4.46E-02	4.83E-02	9.64E-01	FALSE	FALSE	3	182227125	8.78E-02	2.14E-01	TRUE	3	182509337	4.22E-02	0.88	7174	8.97E-06	TRUE	TRUE	482,730	19.75
Sterol ester (27:1/18:3) levels	Parkinson's disease	rs7355269	C	T	C	T	2.59E-01	-1.67E-02	2.61E-02	9.76E-01	FALSE	FALSE	2	62300074	5.86E-02	1.10E-01	TRUE	2	62072939	5.29E-02	0.99	7174	9.93E-07	TRUE	TRUE	482,730	23.98
Sterol ester (27:1/20:2) levels	Parkinson's disease	rs10826704	T	C	T	C	-1.73E-01	-1.70E-03	6.75E-02	8.93E-01	FALSE	FALSE	10	30098732	3.67E-02	1.66E-02	TRUE	10	29809803	3.81E-02	0.96	5848	5.50E-06	TRUE	TRUE	482,730	20.69
Sterol ester (27:1/20:2) levels	Parkinson's disease	rs114838148	T	C	T	C	2.47E-01	2.68E-02	3.14E-02	9.11E-01	FALSE	FALSE	5	180076403	4.15E-02	2.85E-01	TRUE	5	180649403	5.53E-02	0.94	5848	7.99E-06	TRUE	TRUE	482,730	19.97
Sterol ester (27:1/20:2) levels	Parkinson's disease	rs12527805	C	T	C	T	1.31E-01	7.97E-02	1.19E-01	8.41E-01	FALSE	FALSE	6	138944712	3.90E-02	2.05E-02	TRUE	6	138623575	2.89E-02	0.96	5848	6.29E-06	TRUE	TRUE	482,730	20.43
Sterol ester (27:1/20:2) levels	Parkinson's disease	rs13121156	C	T	C	T	-8.72E-02	-1.25E-02	3.20E-01	6.98E-01	FALSE	FALSE	4	97918159	2.44E-02	2.16E-01	TRUE	4	96997008	1.97E-02	1.00	5848	9.25E-06	TRUE	TRUE	482,730	19.69
Sterol ester (27:1/20:2) levels	Parkinson's disease	rs1404999	T	C	T	C	-1.02E-01	-1.57E-02	3.14E-01	6.85E-01	FALSE	FALSE	7	39121465	2.39E-02	2.92E-01	TRUE	7	39081865	2.01E-02	1.00	5848	3.99E-07	TRUE	TRUE	482,730	25.75
Sterol ester (27:1/20:2) levels	Parkinson's disease	rs182695896	C	A	C	A	2.86E-01	1.53E-01	2.53E-02	9.84E-01	FALSE	FALSE	4	74813227	1.83E-01	3.94E-01	TRUE	4	73947510	5.94E-02	0.96	5848	1.52E-06	TRUE	TRUE	482,730	23.16
Sterol ester (27:1/20:2) levels	Parkinson's disease	rs1903973	A	G	A	G	8.64E-02	2.62E-02	5.56E-01	4.77E-01	FALSE	FALSE	10	54865648	1.71E-02	9.03E-01	TRUE	10	53105888	1.84E-02	1.00	5848	2.62E-06	TRUE	TRUE	482,730	22.12
Sterol ester (27:1/20:2) levels	Parkinson's disease	rs2244334	C	T	C	T	-1.91E-01	-4.14E-02	5.46E-02	9.45E-01	FALSE	FALSE	10	71695290	5.41E-02	3.53E-01	TRUE	10	69935534	4.26E-02	0.90	5848	7.39E-06	TRUE	TRUE	482,730	20.12
Sterol ester (27:1/20:2) levels	Parkinson's disease	rs4245791	T	C	T	C	-2.56E-01	-2.80E-03	7.73E-01	3.24E-01	FALSE	FALSE	2	44074431	1.80E-02	5.64E-02	TRUE	2	43847292	2.17E-02	1.00	5848	9.13E-32	TRUE	TRUE	482,730	139.18
Sterol ester (27:1/20:2) levels	Parkinson's disease	rs4778971	G	A	G	A	-9.02E-02	6.15E-02	6.09E-01	4.03E-01	FALSE	FALSE	15	79472522	2.31E-02	3.88E-03	TRUE	15	79180180	1.97E-02	0.94	5848	4.61E-06	TRUE	TRUE	482,730	21.03
Sterol ester (27:1/20:2) levels	Parkinson's disease	rs55837134	A	G	A	G	9.94E-02	7.00E-04	2.39E-01	7.75E-01	FALSE	FALSE	7	44571657	2.67E-02	9.22E-03	TRUE	7	44532058	2.18E-02	0.98	5848	4.95E-06	TRUE	TRUE	482,730	20.89
Sterol ester (27:1/20:2) levels	Parkinson's disease	rs56276984	T	C	T	C	-1.68E-01	-6.70E-02	6.92E-02	9.26E-01	FALSE	FALSE	8	23405794	3.27E-02	2.02E-02	TRUE	8	23548281	3.62E-02	1.00	5848	3.57E-06	TRUE	TRUE	482,730	21.52
Sterol ester (27:1/20:2) levels	Parkinson's disease	rs6490297	C	T	C	T	-9.42E-02	2.77E-02	3.13E-01	7.38E-01	FALSE	FALSE	12	121055159	2.20E-02	6.80E-01	TRUE	12	120617356	1.98E-02	0.99	5848	2.04E-06	TRUE	TRUE	482,730	22.60
Sterol ester (27:1/20:2) levels	Parkinson's disease	rs6709904	G	A	G	A	-2.11E-01	1.00E-03	9.98E-02	8.86E-01	FALSE	FALSE	2	44080324	3.03E-02	1.18E-02	TRUE	2	43853185	3.19E-02	0.99	5848	3.77E-11	TRUE	TRUE	482,730	43.89
Sterol ester (27:1/20:2) levels	Parkinson's disease	rs6755057	C	G	C	G	-1.80E-01	2.45E-02	9.35E-01	9.14E-01	TRUE	FALSE	2	25658098	4.05E-02	2.64E-01	TRUE	2	25435229	3.72E-02	0.99	5848	1.33E-06	TRUE	TRUE	482,730	23.43
Sterol ester (27:1/20:2) levels	Parkinson's disease	rs700320	T	C	T	C	-9.56E-02	-4.56E-02	2.69E-01	7.39E-01	FALSE	FALSE	7	147471122	2.50E-02	3.41E-02	TRUE	7	147774030	2.10E-02	1.00	5848	5.31E-06	TRUE	TRUE	482,730	20.76
Sterol ester (27:1/20:2) levels	Parkinson's disease	rs7202845	C	A	C	A	9.87E-02	-1.47E-02	6.70E-01	4.06E-01	FALSE	FALSE	16	26728889	2.28E-02	2.83E-01	TRUE	16	26717568	1.98E-02	0.99	5848	6.41E-07	TRUE	TRUE	482,730	24.84
Sterol ester (27:1/20:2) levels	Parkinson's disease	rs74726976	T	C	T	C	2.27E-01	6.16E-02	3.36E-02	9.70E-01	FALSE	FALSE	5	90250534	5.41E-02	5.93E-01	TRUE	5	90954717	5.13E-02	0.99	5848	9.64E-06	TRUE	TRUE	482,730	19.61
Sterol ester (27:1/20:2) levels	Parkinson's disease	rs75823353	G	A	G	A	1.69E-01	-2.65E-02	7.29E-02	9.53E-01	FALSE	FALSE	3	7529319	5.64E-02	1.95E-01	TRUE	3	7487632	3.57E-02	0.98	5848	2.39E-06	TRUE	TRUE	482,730	22.30
Sterol ester (27:1/20:2) levels	Parkinson's disease	rs77590808	A	G	A	G	-3.88E-01	-1.84E-02	1.20E-02	9.86E-01	FALSE	FALSE	2	165972435	9.42E-02	7.32E-02	TRUE	2	165115925	8.74E-02	0.98	5848	9.32E-06	TRUE	TRUE	482,730	19.68
Sterol ester (27:1/20:2) levels	Parkinson's disease	rs7853989	C	G	C	G	-1.33E-01	2.84E-02	1.56E-01	9.70E-02	TRUE	FALSE	9	136131592	2.87E-02	4.90E-01	TRUE	9	133256205	2.58E-02	1.00	5848	2.41E-07	TRUE	TRUE	482,730	26.73
Sterol ester (27:1/20:2) levels	Parkinson's disease	rs7933	G	A	G	A	1.06E-01	9.40E-03	3.29E-01	6.35E-01	FALSE	FALSE	2	43452183	2.02E-02	1.92E-01	TRUE	2	43225044	1.97E-02	0.99	5848	9.14E-08	TRUE	TRUE	482,730	28.62
Sterol ester (27:1/20:2) levels	Parkinson's disease	rs79849855	C	T	C	T	-3.11E-01	-1.07E-01	1.96E-02	9.80E-01	FALSE	FALSE	2	61048359	7.13E-02	8.71E-01	TRUE	2	60821224	6.75E-02	0.97	5848	4.07E-06	TRUE	TRUE	482,730	21.27
Sterol ester (27:1/20:2) levels	Parkinson's disease	rs9504426	G	C	G	C	-9.23E-02	-3.24E-02	3.78E-01	3.26E-01	TRUE	FALSE	6	5531242	2.40E-02	7.49E-01	TRUE	6	5531009	1.90E-02	0.99	5848	1.16E-06	TRUE	TRUE	482,730	23.68
Sterol ester (27:1/20:3) levels	Parkinson's disease	rs111575978	A	G	A	G	2.89E-01	4.71E-02	2.13E-02	9.84E-01	FALSE	FALSE	14	47163150	1.02E-01	1.92E-01	TRUE	14	46693947	5.90E-02	0.96	7171	9.70E-07	TRUE	TRUE	482,730	24.03
Sterol ester (27:1/20:3) levels	Parkinson's disease	rs112478356	C	G	C	G	3.16E-01	8.04E-02	1.72E-02	4.33E-02	TRUE	FALSE	2	174552785	5.90E-02	7.63E-01	TRUE	2	173688057	6.68E-02	0.90	7171	2.29E-06	TRUE	TRUE	482,730	22.36
Sterol ester (27:1/20:3) levels	Parkinson's disease	rs1135999	G	A	G	A	-2.26E-01	2.26E-02	3.15E-01	6.99E-01	FALSE	FALSE	16	15131962	1.91E-02	6.26E-01	TRUE	16	15038105	1.78E-02	1.00	7171	1.90E-36	TRUE	TRUE	482,730	160.75
Sterol ester (27:1/20:3) levels	Parkinson's disease	rs116917626	T	C	T	C	-2.38E-01	1.84E-02	2.75E-02	9.86E-01	FALSE	FALSE	17	49639325	1.53E-01	4.36E-02	TRUE	17	51561964	5.25E-02	0.97	7171	5.66E-06	TRUE	TRUE	482,730	20.63
Sterol ester (27:1/20:3) levels	Parkinson's disease	rs117079226	A	G	A	G	3.76E-01	3.31E-02	1.15E-02	9.89E-01	FALSE	FALSE	9	112466763	1.25E-01	1.02E-01	TRUE	9	109704483	7.79E-02	0.98	7171	1.47E-06	TRUE	TRUE	482,730	23.23
Sterol ester (27:1/20:3) levels	Parkinson's disease	rs117754141	G	C	G	C	2.91E-01	3.14E-02	2.26E-02	3.05E-02	TRUE	FALSE	6	109472816	7.18E-02	1.79E-01	TRUE	6	109151613	5.98E-02	0.92	7171	1.12E-06	TRUE	TRUE	482,730	23.75
Sterol ester (27:1/20:3) levels	Parkinson's disease	rs1428498	G	A	G	A	-8.12E-02	1.22E-02	3.51E-01	7.25E-01	FALSE	FALSE	5	38698659	2.51E-02	2.02E-01	TRUE	5	38698557	1.72E-02	1.00	7171	2.48E-06	TRUE	TRUE	482,730	22.22
Sterol ester (27:1/20:3) levels	Parkinson's disease	rs182695896	C	A	C	A	2.85E-01	1.53E-01	2.53E-02	9.84E-01	FALSE	FALSE	4	74813227	1.83E-01	3.94E-01	TRUE	4	73947510	5.31E-02	0.96	7171	8.01E-08	TRUE	TRUE	482,730	28.86
Sterol ester (27:1/20:3) levels	Parkinson's disease	rs2352991	G	A	G	A	-8.89E-02	1.97E-02	7.35E-01	2.66E-01	FALSE	FALSE	14	95749854	2.55E-02	3.58E-01	TRUE	14	95283517	1.92E-02	0.98	7171	3.61E-06	TRUE	TRUE	482,730	21.49
Sterol ester (27:1/20:3) levels	Parkinson's disease	rs56374730	G	T	G	T	1.12E-01	-3.00E-02	2.96E-01	7.43E-01	FALSE	FALSE	16	15867652	2.56E-02	6.19E-01	TRUE	16	15773795	1.84E-02	0.99	7171	1.15E-09	TRUE	TRUE	482,730	37.16
Sterol ester (27:1/20:3) levels	Parkinson's disease	rs624605	T	G	T	G	-8.41E-02	1.08E-02	4.44E-01	6.02E-01	FALSE	FALSE	11	75754620	2.37E-02	1.87E-01	TRUE	11	76043576	1.74E-02	0.94	7171	1.35E-06	TRUE	TRUE	482,730	23.39
Sterol ester (27:1/20:3) levels	Parkinson's disease	rs685850	G	A	G	A	8.34E-02	2.54E-02	5.57E-01	4.95E-01	FALSE	FALSE	15	53751702	2.24E-02	5.88E-01	TRUE	15	53459505	1.68E-02	0.99	7171	7.01E-07	TRUE	TRUE	482,730	24.65
Sterol ester (27:1/20:3) levels	Parkinson's disease	rs7036889	C	A	C	A	-1.34E-01	2.19E-02	9.08E-02	9.16E-01	FALSE	FALSE	9	135211532	3.11E-02	3.17E-01	TRUE	9	132336145	2.95E-02	0.99	7171	5.37E-06	TRUE	TRUE	482,730	20.73
Sterol ester (27:1/20:3) levels	Parkinson's disease	rs72660594	C	T	C	T	-2.45E-01	-1.36E-01	4.20E-02	9.82E-01	FALSE	FALSE	1	55636240	9.22E-02	8.54E-01	TRUE	1	55170567	4.17E-02	0.99	7171	4.38E-09	TRUE	TRUE	482,730	34.53
Sterol ester (27:1/20:3) levels	Parkinson's disease	rs77645768	A	G	A	G	2.72E-01	5.69E-02	2.56E-02	9.74E-01	FALSE	FALSE	4	73668828	7.75E-02	3.34E-01	TRUE	4	72803111	5.38E-02	0.97	7171	4.39E-07	TRUE	TRUE	482,730	25.56

Sterol ester (27:1/20:3) levels	Parkinson's disease	rs79047909	G	C	G	C	-5.16E-01	6.88E-02	6.23E-03	9.10E-03	TRUE	FALSE	2	10183140	1.38E-01	2.08E-01	TRUE	2	10043013	1.07E-01	0.96	7171	1.38E-06	TRUE	TRUE	482,730	23.35
Sterol ester (27:1/20:3) levels	Parkinson's disease	rs79146711	T	C	T	C	1.86E-01	-6.90E-03	4.51E-02	9.76E-01	FALSE	FALSE	4	74290628	8.45E-02	2.92E-02	TRUE	4	73424911	4.10E-02	0.94	7171	5.45E-06	TRUE	TRUE	482,730	20.70
Sterol ester (27:1/20:3) levels	Parkinson's disease	rs79771052	G	A	G	A	3.92E-01	5.30E-03	1.20E-02	9.83E-01	FALSE	FALSE	3	72482879	9.20E-02	2.02E-02	TRUE	3	72433728	8.07E-02	0.91	7171	1.25E-06	TRUE	TRUE	482,730	23.54
Sterol ester (27:1/20:3) levels	Parkinson's disease	rs80325645	C	T	C	T	-4.65E-01	-5.00E-03	7.79E-03	9.83E-01	FALSE	FALSE	9	134767770	9.44E-02	1.87E-02	TRUE	9	131892383	9.66E-02	0.96	7171	1.53E-06	TRUE	TRUE	482,730	23.15
Sterol ester (27:1/20:3) levels	Parkinson's disease	rs9310455	A	C	A	C	-1.52E-01	-6.20E-03	6.68E-02	9.51E-01	FALSE	FALSE	3	14686153	4.17E-02	5.48E-02	TRUE	3	14644646	3.36E-02	1.00	7171	5.99E-06	TRUE	TRUE	482,730	20.52
Sterol ester (27:1/20:4) levels	Parkinson's disease	rs11022403	T	C	T	C	8.45E-02	3.08E-02	3.04E-01	7.78E-01	FALSE	FALSE	11	12563989	3.30E-02	4.55E-01	TRUE	11	12542442	1.83E-02	0.96	7174	4.15E-06	TRUE	TRUE	482,730	21.23
Sterol ester (27:1/20:4) levels	Parkinson's disease	rs113248417	G	A	G	A	-2.68E-01	3.50E-02	3.69E-02	9.74E-01	FALSE	FALSE	11	61826934	7.13E-02	2.05E-01	TRUE	11	62059462	4.49E-02	0.98	7174	2.59E-09	TRUE	TRUE	482,730	35.55
Sterol ester (27:1/20:4) levels	Parkinson's disease	rs1135999	G	A	G	A	-1.01E-01	2.26E-02	3.15E-01	6.99E-01	FALSE	FALSE	16	15131962	1.91E-02	6.26E-01	TRUE	16	15038105	1.78E-02	1.00	7174	1.23E-08	TRUE	TRUE	482,730	32.52
Sterol ester (27:1/20:4) levels	Parkinson's disease	rs113603971	C	T	C	T	-1.61E-01	-4.23E-02	5.75E-02	9.72E-01	FALSE	FALSE	10	13850836	7.63E-02	2.37E-01	TRUE	10	13808836	3.63E-02	0.95	7174	9.89E-06	TRUE	TRUE	482,730	19.56
Sterol ester (27:1/20:4) levels	Parkinson's disease	rs11591147	T	G	T	G	-2.97E-01	-3.77E-02	3.32E-02	9.82E-01	FALSE	FALSE	1	55505647	6.83E-02	2.36E-01	TRUE	1	55039974	4.62E-02	1.00	7174	1.30E-10	TRUE	TRUE	482,730	41.43
Sterol ester (27:1/20:4) levels	Parkinson's disease	rs117084735	T	C	T	C	3.20E-01	-1.80E-03	1.38E-02	9.84E-01	FALSE	FALSE	14	99289369	9.35E-02	6.78E-03	TRUE	14	98823032	7.09E-02	0.99	7174	6.51E-06	TRUE	TRUE	482,730	20.36
Sterol ester (27:1/20:4) levels	Parkinson's disease	rs117186386	C	T	C	T	1.78E-01	3.42E-02	5.99E-02	9.41E-01	FALSE	FALSE	16	87710120	4.87E-02	3.16E-01	TRUE	16	87676514	3.53E-02	0.98	7174	4.60E-07	TRUE	TRUE	482,730	25.47
Sterol ester (27:1/20:4) levels	Parkinson's disease	rs117825513	T	C	T	C	3.85E-01	-9.12E-02	1.10E-02	9.81E-01	FALSE	FALSE	9	22932647	8.91E-02	5.14E-01	TRUE	9	22932648	8.54E-02	0.96	7174	6.64E-06	TRUE	TRUE	482,730	20.32
Sterol ester (27:1/20:4) levels	Parkinson's disease	rs147981159	A	G	A	G	-3.27E-01	2.71E-02	4.33E-02	9.64E-01	FALSE	FALSE	11	61824298	6.29E-02	1.76E-01	TRUE	11	62056826	4.18E-02	0.96	7174	6.76E-15	TRUE	TRUE	482,730	60.92
Sterol ester (27:1/20:4) levels	Parkinson's disease	rs174528	C	T	C	T	-6.48E-01	-6.00E-03	4.14E-01	6.34E-01	FALSE	FALSE	11	61543499	1.79E-02	1.33E-01	TRUE	11	61776027	1.52E-02	1.00	7174	1.00E-200	TRUE	TRUE	482,730	1820.36
Sterol ester (27:1/20:4) levels	Parkinson's disease	rs182695896	C	A	C	A	2.96E-01	1.53E-01	2.53E-02	9.84E-01	FALSE	FALSE	4	74813227	1.83E-01	3.94E-01	TRUE	4	73947510	5.25E-02	0.96	7174	1.82E-08	TRUE	TRUE	482,730	31.75
Sterol ester (27:1/20:4) levels	Parkinson's disease	rs186913978	T	G	T	G	-4.14E-01	6.93E-02	1.20E-02	9.77E-01	FALSE	FALSE	2	17328531	8.26E-02	3.96E-01	TRUE	2	17147264	7.73E-02	0.95	7174	8.60E-08	TRUE	TRUE	482,730	28.73
Sterol ester (27:1/20:4) levels	Parkinson's disease	rs2229738	T	C	T	C	-1.53E-01	5.00E-04	1.56E-01	9.25E-01	FALSE	FALSE	11	68562328	3.29E-02	4.94E-03	TRUE	11	68794860	2.34E-02	0.94	7174	7.01E-11	TRUE	TRUE	482,730	42.64
Sterol ester (27:1/20:4) levels	Parkinson's disease	rs223841	G	A	G	A	-1.01E-01	-1.10E-03	1.72E-01	8.16E-01	FALSE	FALSE	16	57457271	2.54E-02	1.54E-02	TRUE	16	57423359	2.19E-02	1.00	7174	4.23E-06	TRUE	TRUE	482,730	21.19
Sterol ester (27:1/20:4) levels	Parkinson's disease	rs3741252	T	C	T	C	2.12E-01	-2.38E-02	1.34E-01	9.32E-01	FALSE	FALSE	11	61511498	4.03E-02	2.56E-01	TRUE	11	61744026	2.45E-02	0.99	7174	6.46E-18	TRUE	TRUE	482,730	74.77
Sterol ester (27:1/20:4) levels	Parkinson's disease	rs3767941	T	C	T	C	1.05E-01	-1.65E-02	1.66E-01	8.25E-01	FALSE	FALSE	1	41290235	2.88E-02	2.47E-01	TRUE	1	40824563	2.21E-02	0.99	7174	2.01E-06	TRUE	TRUE	482,730	22.62
Sterol ester (27:1/20:4) levels	Parkinson's disease	rs3820438	C	G	C	G	8.44E-02	5.55E-02	2.91E-01	2.27E-01	TRUE	FALSE	1	201924749	3.28E-02	4.53E-02	TRUE	1	201955621	1.82E-02	0.98	7174	3.54E-06	TRUE	TRUE	482,730	21.53
Sterol ester (27:1/20:4) levels	Parkinson's disease	rs4382917	A	G	A	G	1.46E-01	-6.22E-02	2.56E-01	7.72E-01	FALSE	FALSE	11	62221476	3.28E-02	2.90E-02	TRUE	11	62454004	1.97E-02	0.92	7174	1.24E-13	TRUE	TRUE	482,730	55.16
Sterol ester (27:1/20:4) levels	Parkinson's disease	rs6812370	A	G	A	G	-3.44E-01	1.05E-01	1.66E-02	9.88E-01	FALSE	FALSE	4	174947684	1.21E-01	4.15E-01	TRUE	4	174026533	6.56E-02	1.00	7174	1.55E-07	TRUE	TRUE	482,730	27.58
Sterol ester (27:1/20:4) levels	Parkinson's disease	rs6860806	G	A	G	A	7.57E-02	1.51E-02	4.20E-01	4.48E-01	FALSE	FALSE	5	131640536	1.73E-02	4.19E-01	TRUE	5	132304843	1.69E-02	0.99	7174	7.76E-06	TRUE	TRUE	482,730	20.02
Sterol ester (27:1/20:4) levels	Parkinson's disease	rs74915447	A	G	A	G	1.96E-01	5.94E-02	3.78E-02	9.71E-01	FALSE	FALSE	4	73765477	7.20E-02	3.88E-01	TRUE	4	72899760	4.39E-02	0.97	7174	7.81E-06	TRUE	TRUE	482,730	20.01
Sterol ester (27:1/20:4) levels	Parkinson's disease	rs7837272	C	G	C	G	8.90E-01	-6.81E-02	2.07E-03	9.80E-03	TRUE	FALSE	8	22050965	1.76E-01	1.56E-01	TRUE	8	22193452	1.94E-01	0.90	7174	4.47E-06	TRUE	TRUE	482,730	21.09
Sterol ester (27:1/20:4) levels	Parkinson's disease	rs79136768	G	A	G	A	-2.20E-01	-8.48E-02	3.69E-02	9.78E-01	FALSE	FALSE	11	61362439	6.47E-02	7.20E-01	TRUE	11	61594967	4.47E-02	0.98	7174	9.10E-07	TRUE	TRUE	482,730	24.15
Sterol ester (27:1/20:5) levels	Parkinson's disease	rs10860778	T	C	T	C	-8.58E-02	2.58E-02	4.91E-01	5.82E-01	FALSE	FALSE	12	102108585	1.85E-02	7.86E-01	TRUE	12	101714807	1.68E-02	1.00	7174	3.23E-07	TRUE	TRUE	482,730	26.16
Sterol ester (27:1/20:5) levels	Parkinson's disease	rs11986253	C	G	C	G	-1.06E-01	-8.53E-02	1.75E-01	2.14E-01	TRUE	FALSE	8	133409490	2.87E-02	1.48E-03	TRUE	8	132397243	2.26E-02	0.96	7174	3.00E-06	TRUE	TRUE	482,730	21.85
Sterol ester (27:1/20:5) levels	Parkinson's disease	rs12515163	G	A	G	A	-8.20E-02	1.04E-02	4.00E-01	6.30E-01	FALSE	FALSE	5	113119700	2.67E-02	1.57E-01	TRUE	5	113784003	1.76E-02	0.93	7174	3.21E-06	TRUE	TRUE	482,730	21.72
Sterol ester (27:1/20:5) levels	Parkinson's disease	rs143678376	A	C	A	C	1.52E-01	1.03E-01	7.77E-02	9.64E-01	FALSE	FALSE	5	84815387	1.50E-01	3.07E-01	TRUE	5	85519569	3.13E-02	0.98	7174	1.12E-06	TRUE	TRUE	482,730	23.75
Sterol ester (27:1/20:5) levels	Parkinson's disease	rs1481977	G	A	G	A	1.13E-01	9.40E-03	8.61E-01	1.51E-01	FALSE	FALSE	11	103469398	3.13E-02	1.18E-01	TRUE	11	103598670	2.44E-02	1.00	7174	3.79E-06	TRUE	TRUE	482,730	21.40
Sterol ester (27:1/20:5) levels	Parkinson's disease	rs174581	A	G	A	G	-4.11E-01	-7.50E-03	4.10E-01	6.58E-01	FALSE	FALSE	11	61606683	1.77E-02	1.73E-01	TRUE	11	61839211	1.64E-02	1.00	7174	3.56E-133	TRUE	TRUE	482,730	629.25
Sterol ester (27:1/20:5) levels	Parkinson's disease	rs17548303	G	C	G	C	8.57E-02	-2.74E-02	2.62E-01	2.49E-01	TRUE	FALSE	15	53740000	2.58E-02	5.40E-01	TRUE	15	53447803	1.89E-02	0.99	7174	6.25E-06	TRUE	TRUE	482,730	20.44
Sterol ester (27:1/20:5) levels	Parkinson's disease	rs2054498	G	A	G	A	-8.53E-02	2.37E-02	5.38E-01	4.63E-01	FALSE	FALSE	4	37393708	2.23E-02	5.41E-01	TRUE	4	37392086	1.66E-02	1.00	7174	2.88E-07	TRUE	TRUE	482,730	26.38
Sterol ester (27:1/20:5) levels	Parkinson's disease	rs2625222	C	T	C	T	-1.25E-01	-2.39E-02	1.07E-01	9.06E-01	FALSE	FALSE	1	204903958	3.39E-02	3.18E-01	TRUE	1	204934830	2.71E-02	0.99	7174	3.84E-06	TRUE	TRUE	482,730	21.37
Sterol ester (27:1/20:5) levels	Parkinson's disease	rs3019200	A	C	A	C	-1.01E-01	-3.10E-03	8.03E-01	1.90E-01	FALSE	FALSE	11	61249383	2.40E-02	4.77E-02	TRUE	11	61481911	2.14E-02	1.00	7174	2.37E-06	TRUE	TRUE	482,730	22.30
Sterol ester (27:1/20:5) levels	Parkinson's disease	rs34343838	A	G	A	G	1.24E-01	-2.61E-02	1.20E-01	9.07E-01	FALSE	FALSE	1	169648105	3.23E-02	3.78E-01	TRUE	1	169678964	2.57E-02	0.99	7174	1.33E-06	TRUE	TRUE	482,730	23.42
Sterol ester (27:1/20:5) levels	Parkinson's disease	rs4490551	A	G	A	G	-9.35E-02	5.40E-02	2.05E-01	8.11E-01	FALSE	FALSE	5	23468145	2.93E-02	3.27E-02	TRUE	5	23468036	2.11E-02	0.96	7174	9.25E-06	TRUE	TRUE	482,730	19.69
Sterol ester (27:1/20:5) levels	Parkinson's disease	rs4908782	T	C	T	C	-7.93E-02	1.20E-02	3.54E-01	6.89E-01	FALSE	FALSE	1	8888114	2.40E-02	2.10E-01	TRUE	1	8828055	1.75E-02	0.99	7174	5.59E-06	TRUE	TRUE	482,730	20.65
Sterol ester (27:1/20:5) levels	Parkinson's disease	rs508049	T	C	T	C	-1.58E-01	1.46E-02	9.80E-02	9.44E-01	FALSE	FALSE	11	68675497	4.30E-02	1.34E-01	TRUE	11	68908029	2.87E-02	0.96	7174	3.35E-08	TRUE	TRUE	482,730	30.56
Sterol ester (27:1/20:5) levels	Parkinson's disease	rs62025936	G	A	G	A	1.07E-01	2.69E-02	1.65E-01	8.40E-01	FALSE	FALSE	16	10528036	3.04E-02	4.25E-01	TRUE	16	10434179	2.27E-02	0.99	7174	2.75E-06	TRUE	TRUE	482,730	22.01
Sterol ester (27:1/20:5) levels	Parkinson's disease	rs71629271	C	T	C	T	1.60E-01	4.35E-02	6.35E-02	9.46E-01	FALSE	FALSE	3	175813416	5.60E-02	3.60E-01	TRUE	3	176095628	3.58E-02	0.91	7174	8.36E-06	TRUE	TRUE	482,730	19.88
Sterol ester (27:1/20:5) levels	Parkinson's disease	rs75897070	C	G	C	G	1.69E-01	-4.55E-01	5.14E-02	3.19E-02	TRUE	FALSE	8	2934815	2.07E-01	1.39E-02	TRUE	8	3077293	3.80E-02	0.97	7174	8.62E-06	TRUE	TRUE	482,730	19.82
Sterol ester (27:1/20:5) levels	Parkinson's disease	rs7780677	A	C	A	C	-7.65E-02	2.17E-02	4.09E-01	6.73E-01	FALSE	FALSE	7	132378493	2.38E-02	4.41E-01	TRUE	7	132693734	1.70E-02	1.00	7174	7.21E-06	TRUE	TRUE	482,730	20.16
Sterol ester (27:1/20:5) levels	Parkinson's disease	rs7936002	G	A	G	A	1.10E-01	-5.27E-02	2.57E-01	7.70E-01	FALSE	FALSE	11	62219029	3.25E-02	9.78E-01	TRUE	11	62451557	1.97E-02	0.93	7174	2.53E-08	TRUE	TRUE	482,730	31.10
Sterol ester (27:1/20:5) levels	Parkinson's disease	rs7947954	C	T	C	T	1.11E-01	-2.94E-02	1.55E-01	8.79E-01	FALSE	FALSE	11	42678748	3.44E-02	4.07E-01	TRUE	11	42657198	2.30E-02	1.00	7174	1.55E-06	TRUE	TRUE	482,730	23.13
Sterol ester (27:1/20:5) levels	Parkinson's disease	rs980814	G	T	G	T	7.91E-02	-3.77E-02	4.55E-01	5.09E-01	FALSE	FALSE	6	156761338	2.79E-02	7.51E-01	TRUE	6	156440204	1.70E-02	0.96	7174	3.55E-06	TRUE	TRUE	482,730	21.53
Sterol ester (27:1/22:6) levels	Parkinson's disease	rs10150075	T	C	T	C	-2.05E-01	9.26E-02	5.30E-02	9.00E-01	FALSE	FALSE	14	73038517	3.80E-02	7.41E-03	TRUE	14	72571809	3.82E-02	0.95	7173	8.96E-08	TRUE	TRUE	482,730	28.64
Sterol ester (27:1/22:6) levels	Parkinson's disease	rs111751059	T	C	T	C	-1.64E-01	3.41E-02	5.52E-02	9.21E-01	FALSE	FALSE	14	20478934	4.11E-02	3.91E-01	TRUE	14	20010775	3.66E-02	0.99	7173	7.18E-06	TRUE	TRUE	482,730	20.17
Sterol ester (27:1/22:6) levels	Parkinson's disease	rs113651041	T	C	T	C	7.63E-02	-5.00E-04	3.85E-01	5.99E-01	FALSE	FALSE	3	185260229	1.80E-02	1.02E-02	TRUE	3	185542441	1.73E-02	1.00	7173	9.97E-06	TRUE	TRUE	482,730	19.54
Sterol ester (27:1/22:6) levels	Parkinson's disease	rs115647337	T	G	T	G	-1.98E-01	-7.35E-02	4.12E-02	9.79E-01	FALSE	FALSE	4	92257245	8.59E-02	4.07E-01	TRUE	4	91336094	4.25E-02	0.97	7173	3.24E-06	TRUE	TRUE	482,730	21.70
Sterol ester (27:1/22:6) levels	Parkinson's disease	rs11591147	T	G	T	G	-2.38E-01	-3.77E-02	3.32E-02	9.82E-01	FALSE	FALSE	1	55505647	6.83E-02	2.36E-01	TRUE	1	55039974	4.69E-02	1.00	7173	3.90E-07	TRUE	TRUE	482,730	25.79
Sterol ester (27:1/22:6) levels	Parkinson's disease	rs11633878	A	G	A	G	-9.75E-02	-3.73E-02	7.19E-01	3.01E-01	FALSE	FALSE	15	93782603	2.74E-02	7.62E-01	TRUE	15	93239374	2.05E-02	0.83	7173	1.97E-06	TRUE	TRUE	482,730	22.66
Sterol ester (27:1/22:6) levels	Parkinson's disease	rs116794590	C	T	C	T	-2.48E-01	5.71E-02	2.55E-02	9.66E-01	FALSE	FALSE	4	13025959	6.39E-02	4.30E-01	TRUE	4	13024335	5.33E-02	0.98	7173	3.28E-06	TRUE	TRUE	482,730	21.68
Sterol ester (27:1/22:6) levels	Parkinson's disease	rs117061362	T	G	T	G	1.99E-01	1.04E-01	4.49E-02	9.81E-01	FALSE	FALSE	15	101736552	1.03E-01	5.07E-01	TRUE	15	101196347	4.11E-02	0.93	7173	1.26E-06	TRUE	TRUE	482,730	23.53
Sterol ester (27:1/22:6) levels	Parkinson's disease	rs149229417	A	C	A	C	3.18E-01	-6.19E-02	1.71E-02	9.73E-01	FALSE	FALSE	3	68715206	7.57E-02	3.84E-01	TRUE	3	68666055	6.60E-02	0.95	7173	1.44E-06	TRUE	TRUE	482,730	23.26
Sterol ester (27:1/22:6) levels	Parkinson's disease	rs17110517	T	C	T	C	-1.75E-01	-3.06E-02	6.10E-02	9.48E-01	FALSE	FALSE	1	54834840	5.36E-02	2.46E-01	TRUE	1	54369167	3.54E-02	0.98	7173	7.85E-07	TRUE	TRUE	482,730	24.44
Sterol ester (27:1/22:6) levels	Parkinson's disease	rs2075083	T	G	T	G	7.45E-02	1.10E-02	5.81E-01	5.06E-01	FALSE	FALSE	7	17719956	2.61E-02	1.72E-01	TRUE	7	17680332	1.68E-02	0.99	7173	9.42E-06	TRUE	TRUE	482,730	19.65
Sterol ester (27:1/22:6) levels	Parkinson's disease	rs2229738	T	C	T	C	-1.14E-01	5.00E-04	1.56E-01	9.25E-01	FALSE	FALSE	11	68562328	3.29E-02	4.94E-03	TRUE	11	68794860	2.34E-02	0.94	7173	1.28E-06	TRUE	TRUE	482,730	23.49
Sterol ester (27:1/22:6) levels	Parkinson's disease	rs72657952	G	C	G	C	2.45E-01	6.00E-04	2.43E-02	2.48E-02	TRUE	FALSE	4	88822167	7.44E-02	2.66E-03	TRUE	4	87901015	5.48E-02	0.97	7173	8.06E-06	TRUE	TRUE	482,730	19.95
Sterol ester (27:1/22:6) levels	Parkinson's disease	rs72852391	T	C	T	C	2.13E-01	-1.17E-01	3.91E-02	9.73E-01	FALSE	FALSE	17	76962777	7.80E-02	8.76E-01	TRUE	17	78966695	4.50E-02	0.91	7173	2.18E-06	TRUE	TRUE	482,730	22.46
Sterol ester (27:1/22:6) levels	Parkinson's disease	rs73769662	A	G	A	G	-1.30E-01	-2.31E-02	8.88E-02	8.69E-01	FALSE	FALSE	6	120367239	3.28E-02	3.18E-01	TRUE	6	120046093	2.93E-02	1.00	7173	9.33E-06	TRUE	TRUE	482,730	19.67
Sterol ester (27:1/22:6) levels	Parkinson's disease	rs75101588	C	T	C	T	2.59E-01	7.50E-03	3.00E-02	8.97E-01	FALSE	FALSE	6	24387361	3.71E-02	7.56E-02	TRUE	6	24387133	4.99E-02	0.98	7173	2.23E-07	TRUE	TRUE	482,730	26.87
Sterol ester (27:1/22:6) levels	Parkinson's disease	rs77456635	A	G	A	G	-2.68E-01	1.32E-02	2.42E-02	9.69E-01	FALSE	FALSE	15	65158047	5.11E-02	9.87E-02	TRUE	15	64865848	5.36E-02	1.00	7173	5.68E-07	TRUE	TRUE	482,730	25.06
Sterol ester (27:1/22:6) levels	Parkinson's disease	rs77708260	A	G	A	G	2.45E-01	1.54E-01	2.45E-02	9.86E-01	FALSE	FALSE	1	119892600	1.02E-01	8.85E-01	TRUE	1	119349977	5.47E-02	0.99	7173	7.41E-06	TRUE	TRUE	482,730	20.11
Sterol ester (27:1/22:6) levels	Parkinson's disease	rs9559135	G	A	G	A	8.55E-02	4.02E-02	2.84E-01	6.89E-01	FALSE	FALSE	13	108329616	2.43E-02	4.90E-02	TRUE	13	107677268	1.83E-02	1.00	7173	3.17E-06	TRUE	TRUE	482,730	21.74
Ceramide (d40:1) levels	Parkinson's disease	rs10025139	C	T	C	T	8.99E-02	-5.20E-03	2.22E-01	8.00E-01	FALSE	FALSE	4	36313238	2.27E-02	8.70E-02	TRUE	4	36311616	2.00E-02	0.99	7117	7.10E-06	TRUE	TRUE	482,730	20.19
Ceramide (d40:1) levels	Parkinson's disease	rs11064535	A	G	A	G	-1.59E-01	-6.66E-02	5.85E-02	9.37E-01	FALSE	FALSE	12	221020	4.95E-02	7.47E-01	TRUE	12	111854	3.59E-02	0.97	7117	9.98E-06	TRUE	TRUE	482,730	19.54
Ceramide (d40:1) levels	Parkinson's disease	rs114910162	T	C	T	C	1.95E-01	1.25E-02	4.95E-02	9.40E-01	FALSE	FALSE	2	142624932	4.94E-02	9.69E-02	TRUE	2	141867363	3.99E-02	0.95	7117	9.74E-07	TRUE	TRUE	482,730	24.02
Ceramide (d40:1) levels	Parkinson's disease	rs117873718	C	T	C	T	-2.23E-01	8.33E-02	3.26E-02	9.80E-01	FALSE	FALSE	12	67878891	9.45E-02	4.22E-01	TRUE	12	67485111	4.83E-02	0.92	7117	4.03E-06	TRUE	TRUE	482,730	21.28
Ceramide (d40:1) levels	Parkinson's disease	rs11982486	C	T	C	T	8.94E-02	-2.29E-02	2.41E-01	6.52E-01	FALSE	FALSE	7	95055598	1.88E-02	6.51E-01	TRUE	7	95426286	1.95E-02	1.00	7117	4.77E-06	TRUE	TRUE	482,730	20.96
Ceramide (d40:1) levels	Parkinson's disease	rs12366015	G	A	G	A	-8.81E-02	4.53E-02	7.17E-01	2.13E-01	FALSE	FALSE	11	116990851	2.13E-02	1.67E-02	TRUE	11	117120135	1.86E-02	1.00	7117	2.25E-06	TRUE	TRUE	482,730	22.40
Ceramide (d40:1) levels	Parkinson's disease	rs1260326	C	T	C	T	-9.95E-02	-6.78E-02	6.51E-01	4.20E-01	FALSE	FALSE	2	27730940	1.72E-02	4.04E-05	TRUE	2	27508073	1.75E-02	1.00	7117	1.40E-08	TRUE	TRUE	482,730	32.26
Ceramide (d40:1) levels	Parkinson's disease	rs1346910	T	C	T	C	-1.03E-01	-1.55E-02	8.23E-01	1.95E-01	FALSE	FALSE	9	74075645	2.41E-02	2.83E-01	TRUE	9	71460729	2.21E-02	1.00	7117	3.46E-06	TRUE	TRUE	482,730	21.58
Ceramide (d40:1) levels	Parkinson's disease	rs2255744	G	A	G	A	-8.83E-02	4.33E-02	7.68E-01	1.81E-01	FALSE	FALSE	7	47408029	2.90E-02	8.71E-01	TRUE	7	47368432	1.99E-02	1.00	7117	9.31E-06	TRUE	TRUE	482,730	19.68
Ceramide (d40:1) levels	Parkinson's disease	rs28505920	A	T	A	T	1.23E-01	-9.53E-02	1.71E-01	1.73E-01	TRUE	FALSE	8	119280966	3.16E-02	1.28E-03	TRUE	8	118268727	2.23E-02	0.96	7117	3.58E-08	TRUE	TRUE	482,730	30.43
Ceramide (d40:1) levels	Parkinson's disease	rs4704352	A	G	A	G	8.87E-02	-1.50E-03	7.73E-01	2.24E-01	FALSE	FALSE	5	75968518	2.30E-02	2.27E-02	TRUE	5	76672693	1.99E-02	1.00	7117	8.80E-06	TRUE	TRUE	482,730	19.78
Ceramide (d40:1) levels	Parkinson's disease	rs56143221	A	G	A	G	-1.11E-01	3.20E-02	1.44E-01	9.09E-01	FALSE	FALSE	10	75491213	4.20E-02	3.50E-01	TRUE	10	73731455	2.41E-02	0.98	7117	4.14E-06	TRUE	TRUE	482,730	21.23
Ceramide (d40:1) levels	Parkinson's disease	rs6535831	T	G	T	G	1.14E-01	-1.22E-02	8.61E-01	1.20E-01	FALSE	FALSE	4	152917734	2.85E-02	1.75E-01	TRUE	4	151996582	2.44E-02	0.99	7117	2.85E-06	TRUE	TRUE	482,730	21.95
Ceramide (d40:1) levels	Parkinson's disease	rs7157785	T	G	T	G	1.48E-01	-3.20E-02	1.25E-01	8.32E-01	FALSE	FALSE	14	64235556	2.25E-02	8.11E-01	TRUE	14	63768838	2.52E-02	1.00	7117	5.10E-09	TRUE	TRUE	482,730	34.24
Ceramide (d40:1) levels	Parkinson's disease	rs78573518	G	A	G	A	-4.79E-01	6.35E-02	6.78E-03	9.70E-01	FALSE	FALSE	2	201244090	6.95E-02	4.42E-01	TRUE	2	200379367	1.08E-01	0.92	7117	9.82E-06	TRUE	TRUE	482,730	19.57
Ceramide (d40:1) levels	Parkinson's disease	rs865934	G	T	G	T	-2.83E-01	7.09E-02	1.78E-02	9.89E-01	FALSE	FALSE	14	98523595	1.12E-01	2.79E-01	TRUE	14	98057258	6.34E-02	1.00	7117	8.11E-06	TRUE	TRUE	482,730	19.94
Ceramide (d40:2) levels	Parkinson's disease	rs10869023	T	C	T	C	1.60E-01	-1.60E-02	8.25E-02	9.38E-01	FALSE	FALSE	9	74059731	4.70E-02	1.35E-01	TRUE	9	71444815	3.55E-02	0.99	5287	6.84E-06	TRUE	TRUE	482,730	20.28
Ceramide (d40:2) levels	Parkinson's disease	rs111745969	A	G	A	G	-1.35E-01	-3.22E-02	1.35E-01	9.08E-01	FALSE	FALSE	15	93110222	4.03E-02	3.73E-01	TRUE	15	92566992	2.87E-02	0.97	5287	2.59E-06	TRUE	TRUE	482,730	22.15
Ceramide (d40:2) levels	Parkinson's disease	rs114316759	A	G	A	G	-1.98E-01	1.18E-01	5.94E-02	9.66E-01	FALSE	FALSE	2	132896835	1.23E-01	4.69E-01	TRUE	2	132139262	4.46E-02	0.86	5287	8.85E-06	TRUE	TRUE	482,730	19.78
Ceramide (d40:2) levels	Parkinson's disease	rs115833524	T	C	T	C	2.31E-01	4.10E-03	4.18E-02	9.81E-01	FALSE	FALSE	2	168784668	8.96E-02	1.61E-02	TRUE	2	167928158	4.94E-02	0.95	5287	2.92E-06	TRUE	TRUE	482,730	21.91
Ceramide (d40:2) levels	Parkinson's disease	rs117064201	A	C	A	C	-3.02E-01	9.38E-02	2.23E-02	9.84E-01	FALSE	FALSE	9	78886090	1.00E-01	4.56E-01	TRUE	9	76271174	6.74E-02	0.93	5287	7.66E-06	TRUE	TRUE	482,730	20.06
Ceramide (d40:2) levels	Parkinson's disease	rs117671667	T	C	T	C	2.67E-01	-3.46E-02	3.41E-02	9.56E-01	FALSE	FALSE	11	36096740	5.99E-02	2.49E-01	TRUE	11	36075190	5.71E-02	0.88	5287	2.83E-06	TRUE	TRUE	482,730	21.97
Ceramide (d40:2) levels	Parkinson's disease	rs12878001	G	T	G	T	2.16E-01	-3.26E-02	1.24E-01	8.32E-01	FALSE	FALSE	14	64239629	2.26E-02	8.27E-01	TRUE	14	63772911	2.87E-02	1.00	5287	5.67E-14	TRUE	TRUE	482,730	56.77
Ceramide (d40:2) levels	Parkinson's disease	rs139456652	A	G	A	G	2.14E-01	2.10E-03	5.88E-02	9.37E-01	FALSE	FALSE	11	6321932	4.93E-02	1.50E-02	TRUE	11	6300702	4.32E-02	0.88	5287	8.01E-07	TRUE	TRUE	482,730	24.41
Ceramide (d40:2) levels	Parkinson's disease	rs147060788	C	T	C	T	2.49E-01	-4.60E-03	3.41E-02	9.85E-01	FALSE	FALSE	2	241269243	8.89E-02	1.81E-02	TRUE	2	240329826	5.33E-02	0.98	5287	3.08E-06	TRUE	TRUE	482,730	21.81
Ceramide (d40:2) levels	Parkinson's disease	rs17748108	C	T	C	T	1.45E-01	-4.00E-04	1.23E-01	8.28E-01	FALSE	FALSE	13	77408426	3.55E-02	3.93E-03	TRUE	13	76834291	3.02E-02	0.96	5287	1.72E-06	TRUE	TRUE	482,730	22.94
Ceramide (d40:2) levels	Parkinson's disease	rs61834063	G	A	G	A	-1.13E-01	9.30E-03	2.09E-01	7.60E-01	FALSE	FALSE	1	241040805	2.60E-02	1.42E-01	TRUE	1	240877505	2.38E-02	0.99	5287	2.10E-06	TRUE	TRUE	482,730	22.55
Ceramide (d40:2) levels	Parkinson's disease	rs629047	G	A	G	A	-8.95E-02	-2.24E-02	4.34E-01	5.17E-01	FALSE	FALSE	11	97588674	2.28E-02	4.87E-01	TRUE	11	97717674	1.97E-02	0.98	5287	5.44E-06	TRUE	TRUE	482,730	20.71
Ceramide (d40:2) levels	Parkinson's disease	rs6537481	G	A	G	A	-1.14E-01	1.92E-02	1.89E-01	7.54E-01	FALSE	FALSE	4	148396094	2.18E-02	4.23E-01	TRUE	4	147474942	2.53E-02	1.00	5287	6.59E-06	TRUE	TRUE	482,730	20.35
Ceramide (d40:2) levels	Parkinson's disease	rs6559102	A	G	A	G	-4.18E-01	6.90E-03	1.31E-02	9.69E-01	FALSE	FALSE	8	6024268	6.97E-02	3.57E-02	TRUE	8	6166747	8.81E-02	0.96	5287	2.12E-06	TRUE	TRUE	482,730	22.53
Ceramide (d40:2) levels	Parkinson's disease	rs79320828	A	C	A	C	4.25E-01	1.55E-01	1.26E-02	9.82E-01	FALSE	FALSE	7	150988223	9.58E-02	9.75E-01	TRUE	7	151291137	9.50E-02	0.92	5287	7.88E-06	TRUE	TRUE	482,730	19.99
Ceramide (d40:2) levels	Parkinson's disease	rs964184	C	G	C	G	-1.40E-01	-5.68E-02	8.49E-01	8.63E-01	TRUE	FALSE	11	116648917	2.43E-02	9.71E-03	TRUE	11	116778201	2.67E-02	1.00	5287	1.64E-07	TRUE	TRUE	482,730	27.48
Ceramide (d42:1) levels	Parkinson's disease	rs10033592	G	T	G	T	-8.21E-02	-1.40E-02	6.55E-01	3.59E-01	FALSE	FALSE	4	40239404	2.36E-02	2.57E-01	TRUE	4	40237784	1.77E-02	0.98	7174	3.36E-06	TRUE	TRUE	482,730	21.63
Ceramide (d42:1) levels	Parkinson's disease	rs10201832	T	A	T	A	2.62E-01	7.38E-02	2.14E-02	1.34E-02	TRUE	FALSE	2	62269298	7.98E-02	4.50E-01	TRUE	2	62042163	5.84E-02	0.99	7174	7.31E-06	TRUE	TRUE	482,730	20.14
Ceramide (d42:1) levels	Parkinson's disease	rs11105228	A	G	A	G	-8.60E-02	-2.11E-02	3.37E-01	7.11E-01	FALSE	FALSE	12	89653689	2.48E-02	4.03E-01	TRUE	12	89259912	1.76E-02	1.00	7174	1.13E-06	TRUE	TRUE	482,730	23.74
Ceramide (d42:1) levels	Parkinson's disease	rs11130463	C	A	C	A	1.15E-01	3.11E-02	8.81E-01	1.34E-01	FALSE	FALSE	3	55227014	3.31E-02	4.60E-01	TRUE	3	55192986	2.59E-02	0.99	7174	8.35E-06	TRUE	TRUE	482,730	19.89
Ceramide (d42:1) levels	Parkinson's disease	rs112631157	T	C	T	C	-5.38E-01	-9.65E-02	5.97E-03	9.83E-01	FALSE	FALSE	8	17489841	1.11E-01	4.14E-01	TRUE	8	17632332	1.20E-01	0.82	7174	7.37E-06	TRUE	TRUE	482,730	20.12
Ceramide (d42:1) levels	Parkinson's disease	rs114822153	A	G	A	G	2.37E-01	5.42E-02	2.96E-02	9.67E-01	FALSE	FALSE	4	73238544	7.11E-02	3.51E-01	TRUE	4	72372827	5.10E-02	0.93	7174	3.21E-06	TRUE	TRUE	482,730	21.72
Ceramide (d42:1) levels	Parkinson's disease	rs114910162	T	C	T	C	1.86E-01	1.25E-02	4.95E-02	9.40E-01	FALSE	FALSE	2	142624932	4.94E-02	9.69E-02	TRUE	2	141867363	3.97E-02	0.95	7174	2.99E-06	TRUE	TRUE	482,730	21.85
Ceramide (d42:1) levels	Parkinson's disease	rs117888980	G	T	G	T	-2.94E-01	2.44E-01	1.86E-02	9.80E-01	FALSE	FALSE	9	109783890	9.86E-02	6.59E-03	TRUE	9	107021609	6.36E-02	0.97	7174	3.74E-06	TRUE	TRUE	482,730	21.43
Ceramide (d42:1) levels	Parkinson's disease	rs1260326	C	T	C	T	-8.88E-02	-6.78E-02	6.51E-01	4.20E-01	FALSE	FALSE	2	27730940	1.72E-02	4.04E-05	TRUE	2	27508073	1.74E-02	1.00	7174	3.64E-07	TRUE	TRUE	482,730	25.93
Ceramide (d42:1) levels	Parkinson's disease	rs186176314	C	T	C	T	2.69E-01	1.84E-01	2.25E-02	9.87E-01	FALSE	FALSE	5	16573109	1.40E-01	7.21E-01	TRUE	5	16573000	5.77E-02	0.96	7174	3.13E-06	TRUE	TRUE	482,730	21.77
Ceramide (d42:1) levels	Parkinson's disease	rs28505920	A	T	A	T	1.01E-01	-9.53E-02	1.71E-01	1.73E-01	TRUE	FALSE	8	119280966	3.16E-02	1.28E-03	TRUE	8	118268727	2.23E-02	0.96	7174	5.57E-06	TRUE	TRUE	482,730	20.66
Ceramide (d42:1) levels	Parkinson's disease	rs56368930	C	G	C	G	3.80E-01	-1.80E-03	1.24E-02	1.35E-02	TRUE	FALSE	14	33539312	1.76E-01	3.62E-03	TRUE	14	33070106	8.27E-02	0.83	7174	4.44E-06	TRUE	TRUE	482,730	21.10
Ceramide (d42:1) levels	Parkinson's disease	rs57099041	T	C	T	C	1.54E-01	4.99E-02	6.41E-02	9.70E-01	FALSE	FALSE	4	176828525	6.33E-02	3.67E-01	TRUE	4	175907374	3.40E-02	1.00	7174	5.50E-06	TRUE	TRUE	482,730	20.69
Ceramide (d42:1) levels	Parkinson's disease	rs6977389	A	G	A	G	-7.78E-02	4.83E-02	5.60E-01	5.41E-01	FALSE	FALSE	7	95019355	1.68E-02	2.02E-03	TRUE	7	95390043	1.69E-02	1.00	7174	4.10E-06	TRUE	TRUE	482,730	21.25
Ceramide (d42:1) levels	Parkinson's disease	rs7123454	A	C	A	C	-1.11E-01	3.19E-02	8.11E-01	1.26E-01	FALSE	FALSE	11	116704178	2.58E-02	6.66E-01	TRUE	11	116833462	2.16E-02	0.99	7174	2.87E-07	TRUE	TRUE	482,730	26.38
Ceramide (d42:1) levels	Parkinson's disease	rs72941422	C	T	C	T	8.38E-02	-2.08E-02	2.77E-01	7.01E-01	FALSE	FALSE	4	132362364	2.45E-02	4.01E-01	TRUE	4	131441209	1.86E-02	1.00	7174	6.55E-06	TRUE	TRUE	482,730	20.35

Ceramide (d42:1) levels	Parkinson's disease	rs78573518	G	A	G	A	-5.00E-01	6.35E-02	6.78E-03	9.70E-01	FALSE	FALSE	2	201244090	6.95E-02	4.42E-01	TRUE	2	200379367	1.08E-01	0.92	7174	3.38E-06	TRUE	TRUE	482,730	21.62
Ceramide (d42:1) levels	Parkinson's disease	rs7970512	A	C	A	C	-2.12E-01	7.19E-02	3.50E-02	9.81E-01	FALSE	FALSE	12	67907600	1.01E-01	3.23E-01	TRUE	12	67513820	4.78E-02	0.87	7174	9.54E-06	TRUE	TRUE	482,730	19.63
Ceramide (d42:2) levels	Parkinson's disease	rs1042034	T	C	T	C	1.03E-01	-5.30E-03	7.28E-01	2.18E-01	FALSE	FALSE	2	21225281	2.03E-02	9.96E-02	TRUE	2	21002409	1.86E-02	1.00	7173	3.54E-08	TRUE	TRUE	482,730	30.45
Ceramide (d42:2) levels	Parkinson's disease	rs11507232	A	G	A	G	1.27E-01	2.34E-02	9.50E-02	9.17E-01	FALSE	FALSE	9	74085486	3.71E-02	2.77E-01	TRUE	9	71470570	2.86E-02	0.99	7173	9.99E-06	TRUE	TRUE	482,730	19.54
Ceramide (d42:2) levels	Parkinson's disease	rs116327692	T	C	T	C	3.47E-01	1.14E-01	1.43E-02	9.88E-01	FALSE	FALSE	2	138122162	8.43E-02	7.54E-01	TRUE	2	137364592	7.15E-02	0.97	7173	1.28E-06	TRUE	TRUE	482,730	23.49
Ceramide (d42:2) levels	Parkinson's disease	rs1169306	T	C	T	C	-9.83E-02	2.60E-03	3.60E-01	6.36E-01	FALSE	FALSE	12	121438311	1.75E-02	5.42E-02	TRUE	12	121000508	1.74E-02	1.00	7173	1.51E-08	TRUE	TRUE	482,730	32.11
Ceramide (d42:2) levels	Parkinson's disease	rs11713732	T	C	T	C	1.12E-01	-3.38E-02	1.60E-01	8.38E-01	FALSE	FALSE	3	54547617	3.33E-02	5.08E-01	TRUE	3	54513590	2.37E-02	0.92	7173	2.12E-06	TRUE	TRUE	482,730	22.52
Ceramide (d42:2) levels	Parkinson's disease	rs117704733	T	C	T	C	1.37E-01	-5.29E-02	8.76E-02	9.30E-01	FALSE	FALSE	17	35859400	4.01E-02	7.28E-01	TRUE	17	37499294	2.97E-02	0.97	7173	4.39E-06	TRUE	TRUE	482,730	21.12
Ceramide (d42:2) levels	Parkinson's disease	rs12356523	A	G	A	G	9.80E-02	2.89E-02	1.91E-01	8.25E-01	FALSE	FALSE	10	132149615	2.87E-02	5.03E-01	TRUE	10	130351351	2.11E-02	0.99	7173	3.31E-06	TRUE	TRUE	482,730	21.66
Ceramide (d42:2) levels	Parkinson's disease	rs1260326	C	T	C	T	-9.48E-02	-6.78E-02	6.51E-01	4.20E-01	FALSE	FALSE	2	27730940	1.72E-02	4.04E-05	TRUE	2	27508073	1.74E-02	1.00	7173	5.58E-08	TRUE	TRUE	482,730	29.56
Ceramide (d42:2) levels	Parkinson's disease	rs12763964	C	T	C	T	-1.32E-01	9.70E-03	2.06E-01	7.64E-01	FALSE	FALSE	10	72602891	2.15E-02	1.86E-01	TRUE	10	70843134	2.07E-02	1.00	7173	1.88E-10	TRUE	TRUE	482,730	40.70
Ceramide (d42:2) levels	Parkinson's disease	rs142985829	T	C	T	C	3.62E-01	1.30E-01	1.21E-02	9.85E-01	FALSE	FALSE	3	102370319	9.89E-02	7.20E-01	TRUE	3	102651475	7.91E-02	0.93	7173	4.79E-06	TRUE	TRUE	482,730	20.95
Ceramide (d42:2) levels	Parkinson's disease	rs1572595	T	C	T	C	-8.38E-02	8.10E-03	3.82E-01	5.73E-01	FALSE	FALSE	10	8448605	2.33E-02	1.38E-01	TRUE	10	8406642	1.73E-02	0.98	7173	1.24E-06	TRUE	TRUE	482,730	23.56
Ceramide (d42:2) levels	Parkinson's disease	rs28505920	A	T	A	T	1.17E-01	-9.53E-02	1.71E-01	1.73E-01	TRUE	FALSE	8	119280966	3.16E-02	1.28E-03	TRUE	8	118268727	2.22E-02	0.96	7173	1.66E-07	TRUE	TRUE	482,730	27.45
Ceramide (d42:2) levels	Parkinson's disease	rs61836115	G	T	G	T	1.23E-01	1.94E-02	1.05E-01	8.72E-01	FALSE	FALSE	10	14315932	3.32E-02	2.53E-01	TRUE	10	14273933	2.75E-02	1.00	7173	8.54E-06	TRUE	TRUE	482,730	19.84
Ceramide (d42:2) levels	Parkinson's disease	rs6503695	C	T	C	T	8.38E-02	1.26E-02	3.14E-01	6.68E-01	FALSE	FALSE	17	40499533	1.90E-02	2.93E-01	TRUE	17	42347515	1.80E-02	1.00	7173	3.28E-06	TRUE	TRUE	482,730	21.68
Ceramide (d42:2) levels	Parkinson's disease	rs6535831	T	G	T	G	1.21E-01	-1.22E-02	8.61E-01	1.20E-01	FALSE	FALSE	4	152917734	2.85E-02	1.75E-01	TRUE	4	151996582	2.43E-02	0.99	7173	6.38E-07	TRUE	TRUE	482,730	24.84
Ceramide (d42:2) levels	Parkinson's disease	rs6837202	A	G	A	G	-2.09E-01	5.41E-02	3.34E-02	9.40E-01	FALSE	FALSE	4	169246153	3.82E-02	8.04E-01	TRUE	4	168325002	4.61E-02	1.00	7173	6.22E-06	TRUE	TRUE	482,730	20.45
Ceramide (d42:2) levels	Parkinson's disease	rs7493416	C	T	C	T	8.15E-02	-1.40E-02	6.91E-01	3.09E-01	FALSE	FALSE	14	64355690	2.09E-02	2.97E-01	TRUE	14	63888972	1.82E-02	1.00	7173	7.57E-06	TRUE	TRUE	482,730	20.07
Ceramide (d42:2) levels	Parkinson's disease	rs7769878	G	C	G	C	1.16E-01	1.70E-02	1.34E-01	1.01E-01	TRUE	FALSE	6	55975462	4.00E-02	1.74E-01	TRUE	6	56110664	2.54E-02	0.93	7173	5.19E-06	TRUE	TRUE	482,730	20.80
Ceramide (d42:2) levels	Parkinson's disease	rs779714	T	C	T	C	1.21E-01	3.28E-02	8.70E-01	1.49E-01	FALSE	FALSE	3	7509984	3.33E-02	4.88E-01	TRUE	3	7468297	2.62E-02	0.88	7173	4.03E-06	TRUE	TRUE	482,730	21.28
Ceramide (d42:2) levels	Parkinson's disease	rs9839625	A	C	A	C	-8.67E-02	8.80E-03	2.60E-01	6.95E-01	FALSE	FALSE	3	31288740	2.90E-02	1.19E-01	TRUE	3	31247248	1.91E-02	0.98	7173	5.88E-06	TRUE	TRUE	482,730	20.55
Cholesterol levels	Parkinson's disease	rs10812660	A	T	A	T	8.60E-02	2.06E-02	3.75E-01	3.26E-01	TRUE	FALSE	9	27742535	2.93E-02	3.18E-01	TRUE	9	27742537	1.79E-02	0.93	7166	1.57E-06	TRUE	TRUE	482,730	23.10
Cholesterol levels	Parkinson's disease	rs112411704	G	A	G	A	3.03E-01	2.09E-02	1.75E-02	9.79E-01	FALSE	FALSE	9	2958367	8.64E-02	9.24E-02	TRUE	9	2958367	6.60E-02	0.91	7166	4.51E-06	TRUE	TRUE	482,730	21.07
Cholesterol levels	Parkinson's disease	rs11591147	T	G	T	G	-2.14E-01	-3.77E-02	3.32E-02	9.82E-01	FALSE	FALSE	1	55505647	6.83E-02	2.36E-01	TRUE	1	55039974	4.70E-02	1.00	7166	5.51E-06	TRUE	TRUE	482,730	20.68
Cholesterol levels	Parkinson's disease	rs11730766	T	C	T	C	2.53E-01	5.35E-02	2.87E-02	9.66E-01	FALSE	FALSE	4	73232228	6.95E-02	3.55E-01	TRUE	4	72366511	5.16E-02	0.94	7166	9.67E-07	TRUE	TRUE	482,730	24.03
Cholesterol levels	Parkinson's disease	rs11781607	A	G	A	G	-8.07E-02	1.38E-02	3.22E-01	7.09E-01	FALSE	FALSE	8	11502408	2.13E-02	2.87E-01	TRUE	8	11644899	1.81E-02	1.00	7166	7.89E-06	TRUE	TRUE	482,730	19.99
Cholesterol levels	Parkinson's disease	rs11858279	C	T	C	T	8.77E-02	-1.50E-02	3.03E-01	7.04E-01	FALSE	FALSE	15	58712973	2.17E-02	3.09E-01	TRUE	15	58420774	1.82E-02	0.98	7166	1.43E-06	TRUE	TRUE	482,730	23.27
Cholesterol levels	Parkinson's disease	rs12366015	G	A	G	A	-8.24E-02	4.53E-02	7.17E-01	2.13E-01	FALSE	FALSE	11	116990851	2.13E-02	1.67E-02	TRUE	11	117120135	1.86E-02	1.00	7166	9.91E-06	TRUE	TRUE	482,730	19.56
Cholesterol levels	Parkinson's disease	rs12552499	G	A	G	A	-1.89E-01	7.80E-03	4.74E-02	8.77E-01	FALSE	FALSE	9	123897495	2.78E-02	1.08E-01	TRUE	9	121135217	4.01E-02	0.99	7166	2.50E-06	TRUE	TRUE	482,730	22.20
Cholesterol levels	Parkinson's disease	rs12562141	C	T	C	T	-1.39E-01	-9.90E-03	8.32E-02	9.09E-01	FALSE	FALSE	1	181921568	3.40E-02	1.13E-01	TRUE	1	181952433	3.03E-02	1.00	7166	4.52E-06	TRUE	TRUE	482,730	21.06
Cholesterol levels	Parkinson's disease	rs1259751	T	C	T	C	2.38E-01	1.71E-02	2.78E-02	9.01E-01	FALSE	FALSE	12	31975635	5.94E-02	1.12E-01	TRUE	12	31822701	5.15E-02	0.98	7166	4.02E-06	TRUE	TRUE	482,730	21.29
Cholesterol levels	Parkinson's disease	rs1271671	A	G	A	G	2.35E-01	-4.40E-03	2.79E-02	9.64E-01	FALSE	FALSE	12	31963829	6.04E-02	2.63E-02	TRUE	12	31810895	5.13E-02	0.98	7166	4.70E-06	TRUE	TRUE	482,730	20.99
Cholesterol levels	Parkinson's disease	rs143214193	A	G	A	G	-4.95E-01	-2.01E-02	7.64E-03	9.81E-01	FALSE	FALSE	3	194685164	9.33E-02	8.11E-02	TRUE	3	194964435	1.09E-01	0.85	7166	5.35E-06	TRUE	TRUE	482,730	20.74
Cholesterol levels	Parkinson's disease	rs16963800	C	T	C	T	1.10E-01	4.32E-02	1.60E-01	8.82E-01	FALSE	FALSE	16	14670767	3.53E-02	6.54E-01	TRUE	16	14576910	2.28E-02	0.99	7166	1.36E-06	TRUE	TRUE	482,730	23.38
Cholesterol levels	Parkinson's disease	rs182695896	C	A	C	A	2.68E-01	1.53E-01	2.53E-02	9.84E-01	FALSE	FALSE	4	74813227	1.83E-01	3.94E-01	TRUE	4	73947510	5.33E-02	0.96	7166	5.02E-07	TRUE	TRUE	482,730	25.30
Cholesterol levels	Parkinson's disease	rs2237478	T	C	T	C	-9.93E-02	-1.83E-02	1.98E-01	7.74E-01	FALSE	FALSE	7	50745794	2.36E-02	3.60E-01	TRUE	7	50678097	2.11E-02	1.00	7166	2.52E-06	TRUE	TRUE	482,730	22.19
Cholesterol levels	Parkinson's disease	rs223841	G	A	G	A	-9.82E-02	-1.10E-03	1.72E-01	8.16E-01	FALSE	FALSE	16	57457271	2.54E-02	1.54E-02	TRUE	16	57423359	2.22E-02	1.00	7166	9.81E-06	TRUE	TRUE	482,730	19.57
Cholesterol levels	Parkinson's disease	rs2879630	A	G	A	G	-1.01E-01	-1.70E-03	1.89E-01	8.15E-01	FALSE	FALSE	10	53678344	2.86E-02	2.11E-02	TRUE	10	51918584	2.12E-02	1.00	7166	1.80E-06	TRUE	TRUE	482,730	22.83
Cholesterol levels	Parkinson's disease	rs34462361	G	A	G	A	8.54E-02	-1.75E-02	2.56E-01	7.69E-01	FALSE	FALSE	2	216376236	2.62E-02	2.98E-01	TRUE	2	215511513	1.91E-02	1.00	7166	8.05E-06	TRUE	TRUE	482,730	19.95
Cholesterol levels	Parkinson's disease	rs669552	G	A	G	A	-1.08E-01	1.05E-01	1.56E-01	8.80E-01	FALSE	FALSE	3	172053664	3.53E-02	1.43E-03	TRUE	3	172335874	2.33E-02	0.99	7166	3.40E-06	TRUE	TRUE	482,730	21.61
Cholesterol levels	Parkinson's disease	rs75059546	A	C	A	C	-2.78E-01	-6.92E-02	2.01E-02	9.75E-01	FALSE	FALSE	5	125526395	7.86E-02	4.21E-01	TRUE	5	126190702	6.10E-02	0.95	7166	5.45E-06	TRUE	TRUE	482,730	20.70
Cholesterol levels	Parkinson's disease	rs75611814	C	T	C	T	1.24E-01	-5.60E-03	1.03E-01	9.35E-01	FALSE	FALSE	13	23724479	3.87E-02	5.30E-02	TRUE	13	23150340	2.74E-02	0.99	7166	5.82E-06	TRUE	TRUE	482,730	20.58
Cholesterol levels	Parkinson's disease	rs77645768	A	G	A	G	2.42E-01	5.69E-02	2.56E-02	9.74E-01	FALSE	FALSE	4	73668828	7.75E-02	3.34E-01	TRUE	4	72803111	5.39E-02	0.97	7166	7.16E-06	TRUE	TRUE	482,730	20.18
Cholesterol levels	Parkinson's disease	rs78721266	G	A	G	A	1.91E-01	-5.07E-02	4.40E-02	9.78E-01	FALSE	FALSE	9	98975938	7.77E-02	2.89E-01	TRUE	9	96213656	4.13E-02	0.99	7166	3.90E-06	TRUE	TRUE	482,730	21.35
Cholesterol levels	Parkinson's disease	rs7909144	G	A	G	A	7.61E-02	3.97E-02	5.93E-01	3.88E-01	FALSE	FALSE	10	54000704	2.28E-02	4.08E-02	TRUE	10	52240944	1.70E-02	1.00	7166	7.66E-06	TRUE	TRUE	482,730	20.05
Diacylglycerol (16:0_18:1) levels	Parkinson's disease	rs111568723	G	A	G	A	2.59E-01	-1.20E-01	2.59E-02	9.47E-01	FALSE	FALSE	9	135345889	5.14E-02	9.73E-03	TRUE	9	132470502	5.83E-02	0.95	6293	8.97E-06	TRUE	TRUE	482,730	19.75
Diacylglycerol (16:0_18:1) levels	Parkinson's disease	rs115473344	A	G	A	G	3.74E-01	8.57E-02	1.13E-02	9.86E-01	FALSE	FALSE	5	119443538	9.88E-02	4.14E-01	TRUE	5	120107843	8.42E-02	0.98	6293	8.89E-06	TRUE	TRUE	482,730	19.77
Diacylglycerol (16:0_18:1) levels	Parkinson's disease	rs117614422	G	C	G	C	-1.73E-01	-8.42E-02	6.50E-02	5.54E-02	TRUE	FALSE	15	40544531	4.91E-02	4.32E-02	TRUE	15	40252330	3.79E-02	0.93	6293	4.70E-06	TRUE	TRUE	482,730	20.99
Diacylglycerol (16:0_18:1) levels	Parkinson's disease	rs11775636	A	G	A	G	-1.22E-01	-2.10E-03	1.58E-01	8.27E-01	FALSE	FALSE	8	143833850	2.50E-02	3.05E-02	TRUE	8	142752432	2.42E-02	1.00	6293	4.60E-07	TRUE	TRUE	482,730	25.48
Diacylglycerol (16:0_18:1) levels	Parkinson's disease	rs117905902	G	A	G	A	1.91E-01	5.70E-03	4.82E-02	9.63E-01	FALSE	FALSE	7	76312481	6.13E-02	3.33E-02	TRUE	7	76683164	4.25E-02	0.98	6293	7.21E-06	TRUE	TRUE	482,730	20.17
Diacylglycerol (16:0_18:1) levels	Parkinson's disease	rs118099052	T	A	T	A	3.79E-01	4.64E-02	1.46E-02	1.71E-02	TRUE	FALSE	12	44856413	9.82E-02	1.96E-01	TRUE	12	44462630	7.63E-02	0.93	6293	7.22E-07	TRUE	TRUE	482,730	24.60
Diacylglycerol (16:0_18:1) levels	Parkinson's disease	rs1260326	C	T	C	T	-9.93E-02	-6.78E-02	6.51E-01	4.20E-01	FALSE	FALSE	2	27730940	1.72E-02	4.04E-05	TRUE	2	27508073	1.86E-02	1.00	6293	9.81E-08	TRUE	TRUE	482,730	28.47
Diacylglycerol (16:0_18:1) levels	Parkinson's disease	rs17033194	C	G	C	G	1.02E-01	-4.49E-02	2.13E-01	2.16E-01	TRUE	FALSE	3	10640211	2.79E-02	9.70E-01	TRUE	3	10598526	2.25E-02	0.96	6293	5.69E-06	TRUE	TRUE	482,730	20.62
Diacylglycerol (16:0_18:1) levels	Parkinson's disease	rs180999317	A	G	A	G	-6.54E-01	1.66E-02	4.75E-03	9.83E-01	FALSE	FALSE	7	157148144	1.02E-01	6.02E-02	TRUE	7	157355450	1.37E-01	0.86	6293	2.04E-06	TRUE	TRUE	482,730	22.60
Diacylglycerol (16:0_18:1) levels	Parkinson's disease	rs28689929	T	C	T	C	-8.18E-02	-1.62E-02	3.78E-01	6.37E-01	FALSE	FALSE	15	93697861	2.33E-02	3.13E-01	TRUE	15	93154632	1.83E-02	0.99	6293	8.05E-06	TRUE	TRUE	482,730	19.96
Diacylglycerol (16:0_18:1) levels	Parkinson's disease	rs36094040	C	T	C	T	-8.71E-02	-2.90E-03	3.48E-01	5.96E-01	FALSE	FALSE	12	72824963	2.33E-02	4.53E-02	TRUE	12	72431183	1.90E-02	0.99	6293	4.64E-06	TRUE	TRUE	482,730	21.02
Diacylglycerol (16:0_18:1) levels	Parkinson's disease	rs55857911	T	C	T	C	1.48E-01	-1.25E-02	9.25E-02	8.70E-01	FALSE	FALSE	1	230733978	3.62E-02	1.37E-01	TRUE	1	230598232	3.20E-02	0.92	6293	3.66E-06	TRUE	TRUE	482,730	21.47
Diacylglycerol (16:0_18:1) levels	Parkinson's disease	rs72753379	T	C	T	C	2.31E-01	2.30E-02	3.92E-02	9.80E-01	FALSE	FALSE	9	123112954	9.08E-02	9.67E-02	TRUE	9	120350676	4.77E-02	0.95	6293	1.28E-06	TRUE	TRUE	482,730	23.50
Diacylglycerol (16:0_18:1) levels	Parkinson's disease	rs76606887	C	T	C	T	2.18E-01	1.98E-01	3.53E-02	9.78E-01	FALSE	FALSE	16	78630198	1.95E-01	5.11E-01	TRUE	16	78596301	4.90E-02	0.98	6293	8.68E-06	TRUE	TRUE	482,730	19.81
Diacylglycerol (16:0_18:1) levels	Parkinson's disease	rs78056622	G	C	G	C	4.17E-01	1.20E-01	1.10E-02	1.60E-02	TRUE	FALSE	1	184385697	1.07E-01	5.83E-01	TRUE	1	184416563	9.16E-02	0.91	6293	5.51E-06	TRUE	TRUE	482,730	20.68
Diacylglycerol (16:0_18:1) levels	Parkinson's disease	rs93	C	T	C	T	8.17E-02	4.40E-03	6.56E-01	3.60E-01	FALSE	FALSE	7	24441525	2.49E-02	6.60E-02	TRUE	7	24401906	1.85E-02	1.00	6293	9.99E-06	TRUE	TRUE	482,730	19.54
Diacylglycerol (16:0_18:1) levels	Parkinson's disease	rs964184	C	G	C	G	-1.86E-01	-5.68E-02	8.49E-01	8.63E-01	TRUE	FALSE	11	116648917	2.43E-02	9.71E-03	TRUE	11	116778201	2.46E-02	1.00	6293	4.04E-14	TRUE	TRUE	482,730	57.41
Diacylglycerol (16:0_18:1) levels	Parkinson's disease	rs9796793	A	G	A	G	-1.23E-01	-6.30E-02	1.25E-01	9.58E-01	FALSE	FALSE	16	30495652	5.46E-02	6.04E-01	TRUE	16	30484331	2.76E-02	0.95	6293	7.82E-06	TRUE	TRUE	482,730	20.01
Diacylglycerol (16:0_18:2) levels	Parkinson's disease	rs1032956	C	T	C	T	8.28E-02	-1.90E-03	4.51E-01	4.80E-01	FALSE	FALSE	15	98231080	2.27E-02	2.97E-02	TRUE	15	97687850	1.85E-02	0.98	6075	7.88E-06	TRUE	TRUE	482,730	20.00
Diacylglycerol (16:0_18:2) levels	Parkinson's disease	rs11182480	C	T	C	T	3.41E-01	-1.11E-02	1.78E-02	9.66E-01	FALSE	FALSE	12	44780362	6.60E-02	6.24E-02	TRUE	12	44386579	7.12E-02	0.93	6075	1.78E-06	TRUE	TRUE	482,730	22.87
Diacylglycerol (16:0_18:2) levels	Parkinson's disease	rs111934503	C	T	C	T	6.69E-01	1.96E-01	4.57E-03	9.88E-01	FALSE	FALSE	8	131555954	1.62E-01	6.48E-01	TRUE	8	130543708	1.40E-01	0.92	6075	1.66E-06	TRUE	TRUE	482,730	22.99
Diacylglycerol (16:0_18:2) levels	Parkinson's disease	rs112122274	G	A	G	A	-2.53E-01	4.76E-02	3.60E-02	9.49E-01	FALSE	FALSE	9	139149840	6.87E-02	3.11E-01	TRUE	9	136257994	5.27E-02	0.85	6075	1.68E-06	TRUE	TRUE	482,730	22.97
Diacylglycerol (16:0_18:2) levels	Parkinson's disease	rs11756450	A	C	A	C	9.35E-02	5.00E-03	3.24E-01	7.09E-01	FALSE	FALSE	6	65119695	1.88E-02	1.02E-01	TRUE	6	64409802	1.95E-02	0.99	6075	1.61E-06	TRUE	TRUE	482,730	23.06
Diacylglycerol (16:0_18:2) levels	Parkinson's disease	rs1260326	C	T	C	T	-1.02E-01	-6.78E-02	6.51E-01	4.20E-01	FALSE	FALSE	2	27730940	1.72E-02	4.04E-05	TRUE	2	27508073	1.89E-02	1.00	6075	6.21E-08	TRUE	TRUE	482,730	29.37
Diacylglycerol (16:0_18:2) levels	Parkinson's disease	rs140314539	A	G	A	G	3.08E-01	1.49E-01	2.14E-02	9.85E-01	FALSE	FALSE	2	221080090	2.24E-01	2.96E-01	TRUE	2	220215369	6.38E-02	0.98	6075	1.38E-06	TRUE	TRUE	482,730	23.36
Diacylglycerol (16:0_18:2) levels	Parkinson's disease	rs148163011	G	C	G	C	-2.81E-01	-7.32E-02	2.42E-02	2.10E-02	TRUE	FALSE	9	73894566	7.80E-02	4.58E-01	TRUE	9	71279650	6.00E-02	0.97	6075	2.83E-06	TRUE	TRUE	482,730	21.97
Diacylglycerol (16:0_18:2) levels	Parkinson's disease	rs149992851	G	A	G	A	2.93E-01	-1.26E-01	1.98E-02	9.87E-01	FALSE	FALSE	3	55303104	1.02E-01	6.65E-01	TRUE	3	55269076	6.54E-02	0.98	6075	7.72E-06	TRUE	TRUE	482,730	20.04
Diacylglycerol (16:0_18:2) levels	Parkinson's disease	rs17145750	T	C	T	C	-1.34E-01	-4.00E-04	1.41E-01	8.50E-01	FALSE	FALSE	7	73026378	2.45E-02	6.30E-03	TRUE	7	73612048	2.61E-02	1.00	6075	3.14E-07	TRUE	TRUE	482,730	26.22
Diacylglycerol (16:0_18:2) levels	Parkinson's disease	rs2004776	T	C	T	C	-1.08E-01	2.69E-02	1.96E-01	7.61E-01	FALSE	FALSE	1	230848702	2.05E-02	7.19E-01	TRUE	1	230712956	2.29E-02	1.00	6075	2.83E-06	TRUE	TRUE	482,730	21.97
Diacylglycerol (16:0_18:2) levels	Parkinson's disease	rs2868424	A	T	A	T	1.11E-01	-3.33E-02	1.65E-01	1.72E-01	TRUE	FALSE	16	11678289	3.08E-02	5.53E-01	TRUE	16	11584433	2.49E-02	0.97	6075	8.47E-06	TRUE	TRUE	482,730	19.86
Diacylglycerol (16:0_18:2) levels	Parkinson's disease	rs34606692	T	C	T	C	-1.14E-01	-7.60E-03	1.96E-01	7.57E-01	FALSE	FALSE	5	149135269	2.55E-02	1.17E-01	TRUE	5	149755706	2.30E-02	1.00	6075	7.38E-07	TRUE	TRUE	482,730	24.56
Diacylglycerol (16:0_18:2) levels	Parkinson's disease	rs34792804	G	T	G	T	-1.73E-01	2.50E-02	5.96E-02	9.30E-01	FALSE	FALSE	9	8556190	4.59E-02	2.31E-01	TRUE	9	8556190	3.89E-02	0.97	6075	8.70E-06	TRUE	TRUE	482,730	19.81
Diacylglycerol (16:0_18:2) levels	Parkinson's disease	rs35986054	C	T	C	T	1.07E-01	-1.07E-02	1.96E-01	8.03E-01	FALSE	FALSE	17	1526343	2.45E-02	1.79E-01	TRUE	17	1623049	2.32E-02	0.98	6075	4.30E-06	TRUE	TRUE	482,730	21.16
Diacylglycerol (16:0_18:2) levels	Parkinson's disease	rs4262724	A	C	A	C	9.17E-02	1.64E-02	7.17E-01	3.25E-01	FALSE	FALSE	11	23936766	2.37E-02	3.09E-01	TRUE	11	23915220	2.03E-02	1.00	6075	6.46E-06	TRUE	TRUE	482,730	20.38
Diacylglycerol (16:0_18:2) levels	Parkinson's disease	rs57747810	A	C	A	C	-4.04E-01	1.09E-01	1.17E-02	9.80E-01	FALSE	FALSE	12	20077695	9.14E-02	6.30E-01	TRUE	12	19924761	8.44E-02	0.95	6075	1.70E-06	TRUE	TRUE	482,730	22.95
Diacylglycerol (16:0_18:2) levels	Parkinson's disease	rs6687291	A	G	A	G	9.81E-02	2.74E-02	3.20E-01	6.45E-01	FALSE	FALSE	1	230722027	2.42E-02	5.90E-01	TRUE	1	230586281	1.98E-02	0.97	6075	7.54E-07	TRUE	TRUE	482,730	24.52
Diacylglycerol (16:0_18:2) levels	Parkinson's disease	rs6810359	T	G	T	G	-1.92E-01	5.55E-02	5.28E-02	9.20E-01	FALSE	FALSE	3	188510214	4.33E-02	6.98E-01	TRUE	3	188792426	4.15E-02	0.95	6075	3.61E-06	TRUE	TRUE	482,730	21.50
Diacylglycerol (16:0_18:2) levels	Parkinson's disease	rs7165355	C	G	C	G	-1.07E-01	-1.19E-02	1.92E-01	2.22E-01	TRUE	FALSE	15	38967537	2.30E-02	2.18E-01	TRUE	15	38675336	2.32E-02	1.00	6075	3.92E-06	TRUE	TRUE	482,730	21.34
Diacylglycerol (16:0_18:2) levels	Parkinson's disease	rs7569544	A	C	A	C	-9.13E-02	2.81E-02	2.65E-01	7.58E-01	FALSE	FALSE	2	42517516	2.57E-02	5.62E-01	TRUE	2	42290376	2.06E-02	1.00	6075	9.64E-06	TRUE	TRUE	482,730	19.61
Diacylglycerol (16:0_18:2) levels	Parkinson's disease	rs758398	T	C	T	C	-1.16E-01	-9.20E-03	1.83E-01	7.26E-01	FALSE	FALSE	6	28320674	1.94E-02	1.95E-01	TRUE	6	28352897	2.34E-02	1.00	6075	7.42E-07	TRUE	TRUE	482,730	24.55
Diacylglycerol (16:0_18:2) levels	Parkinson's disease	rs76587848	T	G	T	G	1.78E-01	-4.20E-03	5.80E-02	9.01E-01	FALSE	FALSE	1	186818943	4.04E-02	3.77E-02	TRUE	1	186849811	4.02E-02	0.93	6075	9.36E-06	TRUE	TRUE	482,730	19.67
Diacylglycerol (16:0_18:2) levels	Parkinson's disease	rs78504564	T	C	T	C	-6.30E-01	9.96E-02	4.92E-03	9.83E-01	FALSE	FALSE	2	43180242	9.05E-02	5.67E-01	TRUE	2	42953102	1.34E-01	0.95	6075	2.49E-06	TRUE	TRUE	482,730	22.22
Diacylglycerol (16:0_18:2) levels	Parkinson's disease	rs964184	C	G	C	G	-2.25E-01	-5.68E-02	8.49E-01	8.63E-01	TRUE	FALSE	11	116648917	2.43E-02	9.71E-03	TRUE	11	116778201	2.48E-02	1.00	6075	1.64E-19	TRUE	TRUE	482,730	82.18
Diacylglycerol (16:1_18:1) levels	Parkinson's disease	rs10022344	T	C	T	C	1.06E-01	7.97E-02	1.81E-01	9.06E-01	FALSE	FALSE	4	125864319	3.96E-02	2.21E-02	TRUE	4	124943164	2.37E-02	0.98	6215	8.41E-06	TRUE	TRUE	482,730	19.87
Diacylglycerol (16:1_18:1) levels	Parkinson's disease	rs11182480	C	T	C	T	3.45E-01	-1.11E-02	1.78E-02	9.66E-01	FALSE	FALSE	12	44780362	6.60E-02	6.24E-02	TRUE	12	44386579	6.96E-02	0.93	6215	7.16E-07	TRUE	TRUE	482,730	24.62
Diacylglycerol (16:1_18:1) levels	Parkinson's disease	rs112122274	G	A	G	A	-2.60E-01	4.76E-02	3.60E-02	9.49E-01	FALSE	FALSE	9	139149840	6.87E-02	3.11E-01	TRUE	9	136257994	5.25E-02	0.85	6215	7.58E-07	TRUE	TRUE	482,730	24.51
Diacylglycerol (16:1_18:1) levels	Parkinson's disease	rs11775636	A	G	A	G	-1.13E-01	-2.10E-03	1.58E-01	8.27E-01	FALSE	FALSE	8	143833850	2.50E-02	3.05E-02	TRUE	8	142752432	2.43E-02	1.00	6215	3.05E-06	TRUE	TRUE	482,730	21.82
Diacylglycerol (16:1_18:1) levels	Parkinson's disease	rs12050879	A	G	A	G	-2.78E-01	-4.75E-02	2.09E-02	9.88E-01	FALSE	FALSE	15	40275933	1.50E-01	1.24E-01	TRUE	15	39983732	6.22E-02	1.00	6215	7.99E-06	TRUE	TRUE	482,730	19.97
Diacylglycerol (16:1_18:1) levels	Parkinson's disease	rs1260326	C	T	C	T	-1.12E-01	-6.78E-02	6.51E-01	4.20E-01	FALSE	FALSE	2	27730940	1.72E-02	4.04E-05	TRUE	2	27508073	1.87E-02	1.00	6215	2.15E-09	TRUE	TRUE	482,730	35.93
Diacylglycerol (16:1_18:1) levels	Parkinson's disease	rs143808674	G	A	G	A	-1.86E-01	-2.87E-02	4.80E-02	9.48E-01	FALSE	FALSE	1	82137339	5.17E-02	2.37E-01	TRUE	1	81671654	4.19E-02	0.99	6215	8.76E-06	TRUE	TRUE	482,730	19.80
Diacylglycerol (16:1_18:1) levels	Parkinson's disease	rs2182380	G	A	G	A	2.61E-01	8.54E-02	3.06E-02	9.58E-01	FALSE	FALSE	6	43868612	5.97E-02	8.18E-01	TRUE	6	43900875	5.44E-02	0.92	6215	1.65E-06	TRUE	TRUE	482,730	23.01
Diacylglycerol (16:1_18:1) levels	Parkinson's disease	rs2524075	T	C	T	C	-1.07E-01	-4.53E-02	2.08E-01	6.98E-01	FALSE	FALSE	6	31243859	2.14E-02	1.71E-02	TRUE	6	31276082	2.18E-02	1.00	6215	9.22E-07	TRUE	TRUE	482,730	24.13
Diacylglycerol (16:1_18:1) levels	Parkinson's disease	rs3829088	C	T	C	T	1.02E-01	2.59E-02	2.03E-01	8.53E-01	FALSE	FALSE	9	23713680	3.15E-02	3.86E-01	TRUE	9	23713682	2.24E-02	1.00	6215	5.65E-06	TRUE	TRUE	482,730	20.64
Diacylglycerol (16:1_18:1) levels	Parkinson's disease	rs4419068	A	G	A	G	-9.31E-02	2.00E-04	2.37E-01	7.42E-01	FALSE	FALSE	16	71343709	1.94E-02	3.40E-03	TRUE	16	71309806	2.11E-02	1.00	6215	9.89E-06	TRUE	TRUE	482,730	19.56
Diacylglycerol (16:1_18:1) levels	Parkinson's disease	rs77134026	A	G	A	G	2.58E-01	-1.49E-02	3.06E-02	9.77E-01	FALSE	FALSE	7	36534736	8.36E-02	6.65E-02	TRUE	7	36495127	5.53E-02	0.85	6215	3.20E-06	TRUE	TRUE	482,730	21.73
Diacylglycerol (16:1_18:1) levels	Parkinson's disease	rs7955732	T	G	T	G	-8.86E-02	8.90E-03	4.23E-01	5.66E-01	FALSE	FALSE	12	72724995	2.26E-02	1.58E-01	TRUE	12	72331215	1.83E-02	1.00	6215	1.24E-06	TRUE	TRUE	482,730	23.55
Diacylglycerol (16:1_18:1) levels	Parkinson's disease	rs908150	C	T	C	T	8.07E-02	2.77E-02	4.28E-01	5.83E-01	FALSE	FALSE	17	64536433	2.26E-02	6.55E-01	TRUE	17	66540315	1.82E-02	1.00	6215	9.56E-06	TRUE	TRUE	482,730	19.63
Diacylglycerol (16:1_18:1) levels	Parkinson's disease	rs964184	C	G	C	G	-1.64E-01	-5.68E-02	8.49E-01	8.63E-01	TRUE	FALSE	11	116648917	2.43E-02	9.71E-03	TRUE	11	116778201	2.47E-02	1.00	6215	3.17E-11	TRUE	TRUE	482,730	44.23
Diacylglycerol (18:1_18:1) levels	Parkinson's disease	rs10169217	G	A	G	A	-9.83E-02	-2.56E-02	6.76E-01	3.13E-01	FALSE	FALSE	2	212875346	2.37E-02	5.51E-01	TRUE	2	212010621	1.82E-02	1.00	6884	7.18E-08	TRUE	TRUE	482,730	29.08
Diacylglycerol (18:1_18:1) levels	Parkinson's disease	rs1042034	T	C	T	C	1.04E-01	-5.30E-03	7.28E-01	2.18E-01	FALSE	FALSE	2	21225281	2.03E-02	9.96E-02	TRUE	2	21002409	1.90E-02	1.00	6884	4.97E-08	TRUE	TRUE	482,730	29.79
Diacylglycerol (18:1_18:1) levels	Parkinson's disease	rs1060743	G	A	G	A	-8.35E-02	-9.20E-03	2.91E-01	6.99E-01	FALSE	FALSE	2	127826533	1.82E-02	2.13E-01	TRUE	2	127068957	1.88E-02	1.00	6884	8.86E-06	TRUE	TRUE	482,730	19.77
Diacylglycerol (18:1_18:1) levels	Parkinson's disease	rs116011373	A	G	A	G	-1.86E-01	-2.23E-02	4.88E-02	9.73E-01	FALSE	FALSE	3	87347791	8.48E-02	1.01E-01	TRUE	3	87298641	3.98E-02	0.96	6884	3.06E-06	TRUE	TRUE	482,730	21.82
Diacylglycerol (18:1_18:1) levels	Parkinson's disease	rs116796527	T	C	T	C	-3.37E-01	-1.78E-01	1.43E-02	9.87E-01	FALSE	FALSE	4	63210698	1.09E-01	9.93E-01	TRUE	4	62344980	7.18E-02	0.99	6884	2.68E-06	TRUE	TRUE	482,730	22.07
Diacylglycerol (18:1_18:1) levels	Parkinson's disease	rs12365864	G	A	G	A	1.05E-01	-2.95E-02	1.66E-01	7.86E-01	FALSE	FALSE	11	116489036	2.73E-02	5.55E-01	TRUE	11	116618319	2.31E-02	0.97	6884	5.55E-06	TRUE	TRUE	482,730	20.67
Diacylglycerol (18:1_18:1) levels	Parkinson's disease	rs1260326	C	T	C	T	-1.23E-01	-6.78E-02	6.51E-01	4.20E-01	FALSE	FALSE	2	27730940	1.72E-02	4.04E-05	TRUE	2	27508073	1.78E-02	1.00	6884	5.88E-12	TRUE	TRUE	482,730	47.53
Diacylglycerol (18:1_18:1) levels	Parkinson's disease	rs137871178	C	T	C	T	2.99E-01	-1.60E-03	1.73E-02	9.89E-01	FALSE	FALSE	14	58604730	1.19E-01	4.80E-03	TRUE	14	58138012	6.71E-02	0.96	6884	8.42E-06	TRUE	TRUE	482,730	19.87
Diacylglycerol (18:1_18:1) levels	Parkinson's disease	rs139500046	C	A	C	A	-3.93E-01	-4.41E-02	1.12E-02	9.83E-01	FALSE	FALSE	12	97244448	8.65E-02	2.15E-01	TRUE	12	96850670	8.07E-02	0.99	6884	1.13E-06	TRUE	TRUE	482,730	23.73
Diacylglycerol (18:1_18:1) levels	Parkinson's disease	rs17596144	T	C	T	C	1.31E-01	-2.90E-03	1.52E-01	8.43E-01	FALSE	FALSE	1	220978645	2.58E-02	4.12E-02	TRUE	1	220805303	2.39E-02	0.99	6884	4.26E-08	TRUE	TRUE	482,730	30.09
Diacylglycerol (18:1_18:1) levels	Parkinson's disease	rs268	G	A	G	A	2.81E-01	1.59E-02	2.27E-02	9.83E-01	FALSE	FALSE	8	19813529	6.69E-02	9.03E-02	TRUE	8	19956018	5.80E-02	0.97	6884	1.26E-06	TRUE	TRUE	482,730	23.52
Diacylglycerol (18:1_18:1) levels	Parkinson's disease	rs2954021	G	A	G	A	-7.58E-02	-1.31E-02	5.38E-01	4.96E-01	FALSE	FALSE	8	126482077	1.69E-02	3.58E-01	TRUE	8	125469835	1.71E-02	1.00	6884	9.05E-06	TRUE	TRUE	482,730	19.73

Diacylglycerol (18:1_18:1) levels	Parkinson's disease	rs35332062	A	G	A	G	-1.44E-01	-4.60E-03	1.22E-01	8.81E-01	FALSE	FALSE	7	73012042	2.72E-02	6.20E-02	TRUE	7	73597712	2.59E-02	1.00	6884	3.11E-08	TRUE	TRUE	482,730	30.70
Diacylglycerol (18:1_18:1) levels	Parkinson's disease	rs36094040	C	T	C	T	-8.29E-02	-2.90E-03	3.48E-01	5.96E-01	FALSE	FALSE	12	72824963	2.33E-02	4.53E-02	TRUE	12	72431183	1.81E-02	0.99	6884	4.96E-06	TRUE	TRUE	482,730	20.88
Diacylglycerol (18:1_18:1) levels	Parkinson's disease	rs62046303	C	T	C	T	3.25E-01	1.59E-02	1.74E-02	9.43E-01	FALSE	FALSE	16	82051998	5.19E-02	1.19E-01	TRUE	16	82018393	7.03E-02	0.90	6884	3.80E-06	TRUE	TRUE	482,730	21.40
Diacylglycerol (18:1_18:1) levels	Parkinson's disease	rs62291573	C	T	C	T	1.07E-01	-2.81E-02	2.14E-01	7.47E-01	FALSE	FALSE	4	5001376	2.75E-02	5.13E-01	TRUE	4	4999649	2.15E-02	0.94	6884	6.91E-07	TRUE	TRUE	482,730	24.68
Diacylglycerol (18:1_18:1) levels	Parkinson's disease	rs76950187	G	A	G	A	1.60E-01	6.08E-02	7.45E-02	9.52E-01	FALSE	FALSE	2	129184649	5.53E-02	5.65E-01	TRUE	2	128427075	3.26E-02	0.99	6884	9.58E-07	TRUE	TRUE	482,730	24.05
Diacylglycerol (18:1_18:1) levels	Parkinson's disease	rs7782961	T	A	T	A	9.46E-02	5.86E-02	2.32E-01	1.82E-01	TRUE	FALSE	7	6335583	2.64E-02	1.32E-02	TRUE	7	6295952	2.04E-02	0.97	6884	3.51E-06	TRUE	TRUE	482,730	21.55
Diacylglycerol (18:1_18:1) levels	Parkinson's disease	rs964184	C	G	C	G	-2.64E-01	-5.68E-02	8.49E-01	8.63E-01	TRUE	FALSE	11	116648917	2.43E-02	9.71E-03	TRUE	11	116778201	2.34E-02	1.00	6884	3.74E-29	TRUE	TRUE	482,730	126.77
Diacylglycerol (18:1_18:1) levels	Parkinson's disease	rs9865586	G	A	G	A	-1.07E-01	-4.00E-03	1.65E-01	8.77E-01	FALSE	FALSE	3	131734843	2.72E-02	5.36E-02	TRUE	3	132015999	2.26E-02	0.99	6884	2.33E-06	TRUE	TRUE	482,730	22.33
Diacylglycerol (18:1_18:2) levels	Parkinson's disease	rs1042034	T	C	T	C	9.34E-02	-5.30E-03	7.28E-01	2.18E-01	FALSE	FALSE	2	21225281	2.03E-02	9.96E-02	TRUE	2	21002409	1.95E-02	1.00	6613	1.65E-06	TRUE	TRUE	482,730	23.00
Diacylglycerol (18:1_18:2) levels	Parkinson's disease	rs10773708	A	G	A	G	-8.71E-02	3.91E-02	6.83E-01	2.93E-01	FALSE	FALSE	12	130281212	2.62E-02	8.67E-01	TRUE	12	129796667	1.88E-02	1.00	6613	3.71E-06	TRUE	TRUE	482,730	21.44
Diacylglycerol (18:1_18:2) levels	Parkinson's disease	rs114444157	C	T	C	T	1.49E-01	-3.10E-03	7.76E-02	9.49E-01	FALSE	FALSE	3	175898079	5.83E-02	1.88E-02	TRUE	3	176180291	3.33E-02	0.95	6613	7.23E-06	TRUE	TRUE	482,730	20.16
Diacylglycerol (18:1_18:2) levels	Parkinson's disease	rs116011373	A	G	A	G	-1.82E-01	-2.23E-02	4.88E-02	9.73E-01	FALSE	FALSE	3	87347791	8.48E-02	1.01E-01	TRUE	3	87298641	4.07E-02	0.96	6613	7.38E-06	TRUE	TRUE	482,730	20.12
Diacylglycerol (18:1_18:2) levels	Parkinson's disease	rs11684166	A	G	A	G	1.06E-01	-4.58E-02	1.77E-01	8.39E-01	FALSE	FALSE	2	111817641	2.48E-02	3.24E-02	TRUE	2	111060064	2.29E-02	0.99	6613	3.49E-06	TRUE	TRUE	482,730	21.56
Diacylglycerol (18:1_18:2) levels	Parkinson's disease	rs117416257	T	C	T	C	-6.04E-01	-2.70E-03	4.57E-03	9.86E-01	FALSE	FALSE	10	5835054	8.36E-02	1.14E-02	TRUE	10	5793091	1.31E-01	0.99	6613	3.83E-06	TRUE	TRUE	482,730	21.38
Diacylglycerol (18:1_18:2) levels	Parkinson's disease	rs1233660	A	T	A	T	-9.77E-02	-2.80E-03	2.21E-01	3.51E-01	TRUE	FALSE	6	28260249	1.81E-02	5.72E-02	TRUE	6	28292472	2.09E-02	1.00	6613	3.00E-06	TRUE	TRUE	482,730	21.85
Diacylglycerol (18:1_18:2) levels	Parkinson's disease	rs12365864	G	A	G	A	1.24E-01	-2.95E-02	1.66E-01	7.86E-01	FALSE	FALSE	11	116489036	2.73E-02	5.55E-01	TRUE	11	116618319	2.35E-02	0.97	6613	1.58E-07	TRUE	TRUE	482,730	27.54
Diacylglycerol (18:1_18:2) levels	Parkinson's disease	rs1260326	C	T	C	T	-1.29E-01	-6.78E-02	6.51E-01	4.20E-01	FALSE	FALSE	2	27730940	1.72E-02	4.04E-05	TRUE	2	27508073	1.81E-02	1.00	6613	9.98E-13	TRUE	TRUE	482,730	51.05
Diacylglycerol (18:1_18:2) levels	Parkinson's disease	rs138578034	T	G	T	G	-6.89E-01	1.54E-01	3.22E-03	9.93E-01	FALSE	FALSE	11	17918702	1.95E-01	3.66E-01	TRUE	11	17897155	1.54E-01	0.94	6613	7.80E-06	TRUE	TRUE	482,730	20.02
Diacylglycerol (18:1_18:2) levels	Parkinson's disease	rs141116593	G	C	G	C	1.55E-01	9.30E-02	7.40E-02	2.71E-02	TRUE	FALSE	6	155201926	1.44E-01	2.85E-01	TRUE	6	154880792	3.31E-02	0.99	6613	2.82E-06	TRUE	TRUE	482,730	21.97
Diacylglycerol (18:1_18:2) levels	Parkinson's disease	rs142868058	T	C	T	C	-5.59E-01	1.27E-02	5.02E-03	9.83E-01	FALSE	FALSE	11	62484531	7.44E-02	6.33E-02	TRUE	11	62717059	1.25E-01	0.97	6613	8.45E-06	TRUE	TRUE	482,730	19.86
Diacylglycerol (18:1_18:2) levels	Parkinson's disease	rs17007519	T	C	T	C	-3.01E-01	7.70E-03	2.19E-02	9.71E-01	FALSE	FALSE	4	142593901	7.15E-02	3.92E-02	TRUE	4	141672748	6.04E-02	0.96	6613	6.24E-07	TRUE	TRUE	482,730	24.88
Diacylglycerol (18:1_18:2) levels	Parkinson's disease	rs181215046	G	T	G	T	-4.77E-01	2.03E-01	7.47E-03	9.85E-01	FALSE	FALSE	11	32552482	1.39E-01	8.42E-01	TRUE	11	32530936	1.05E-01	0.96	6613	5.32E-06	TRUE	TRUE	482,730	20.75
Diacylglycerol (18:1_18:2) levels	Parkinson's disease	rs2352723	C	T	C	T	-1.12E-01	-2.69E-02	7.69E-01	1.85E-01	FALSE	FALSE	1	230291868	2.61E-02	5.20E-01	TRUE	1	230156121	2.04E-02	1.00	6613	3.76E-08	TRUE	TRUE	482,730	30.34
Diacylglycerol (18:1_18:2) levels	Parkinson's disease	rs268	G	A	G	A	3.36E-01	1.59E-02	2.27E-02	9.83E-01	FALSE	FALSE	8	19813529	6.69E-02	9.03E-02	TRUE	8	19956018	5.82E-02	0.97	6613	8.26E-09	TRUE	TRUE	482,730	33.30
Diacylglycerol (18:1_18:2) levels	Parkinson's disease	rs4691907	T	G	T	G	7.83E-02	-7.70E-03	5.32E-01	3.70E-01	FALSE	FALSE	4	164223658	2.06E-02	1.49E-01	TRUE	4	163302506	1.75E-02	1.00	6613	7.60E-06	TRUE	TRUE	482,730	20.07
Diacylglycerol (18:1_18:2) levels	Parkinson's disease	rs55788915	A	C	A	C	-2.51E-01	-3.83E-02	3.10E-02	9.71E-01	FALSE	FALSE	16	23278377	7.15E-02	2.28E-01	TRUE	16	23267056	5.28E-02	0.93	6613	1.92E-06	TRUE	TRUE	482,730	22.71
Diacylglycerol (18:1_18:2) levels	Parkinson's disease	rs57500102	A	G	A	G	-4.99E-01	8.37E-02	7.31E-03	9.81E-01	FALSE	FALSE	3	171254035	8.57E-02	4.83E-01	TRUE	3	171536246	1.11E-01	0.92	6613	7.12E-06	TRUE	TRUE	482,730	20.19
Diacylglycerol (18:1_18:2) levels	Parkinson's disease	rs62291573	C	T	C	T	1.00E-01	-2.81E-02	2.14E-01	7.47E-01	FALSE	FALSE	4	5001376	2.75E-02	5.13E-01	TRUE	4	4999649	2.19E-02	0.94	6613	4.75E-06	TRUE	TRUE	482,730	20.97
Diacylglycerol (18:1_18:2) levels	Parkinson's disease	rs72694990	A	G	A	G	-1.21E-01	4.74E-02	1.29E-01	8.77E-01	FALSE	FALSE	1	150223780	3.11E-02	8.94E-01	TRUE	1	150251390	2.61E-02	0.99	6613	4.00E-06	TRUE	TRUE	482,730	21.30
Diacylglycerol (18:1_18:2) levels	Parkinson's disease	rs72836628	T	C	T	C	-9.02E-02	3.49E-02	2.97E-01	7.34E-01	FALSE	FALSE	10	113902454	1.99E-02	3.97E-02	TRUE	10	112142696	1.90E-02	1.00	6613	2.11E-06	TRUE	TRUE	482,730	22.53
Diacylglycerol (18:1_18:2) levels	Parkinson's disease	rs75008187	A	G	A	G	1.29E-01	1.58E-02	1.28E-01	8.85E-01	FALSE	FALSE	6	65151347	3.11E-02	2.14E-01	TRUE	6	64441454	2.63E-02	1.00	6613	9.80E-07	TRUE	TRUE	482,730	24.01
Diacylglycerol (18:1_18:2) levels	Parkinson's disease	rs75082203	A	G	A	G	3.49E-01	-5.00E-02	1.29E-02	9.85E-01	FALSE	FALSE	10	90366743	8.30E-02	2.62E-01	TRUE	10	88606986	7.84E-02	0.98	6613	8.40E-06	TRUE	TRUE	482,730	19.87
Diacylglycerol (18:1_18:2) levels	Parkinson's disease	rs75596627	A	G	A	G	1.28E-01	9.50E-03	1.14E-01	9.26E-01	FALSE	FALSE	8	43505645	4.87E-02	7.32E-02	TRUE	8	43650502	2.79E-02	0.95	6613	4.62E-06	TRUE	TRUE	482,730	21.02
Diacylglycerol (18:1_18:2) levels	Parkinson's disease	rs7745829	G	A	G	A	-1.12E-01	-4.26E-02	1.47E-01	9.29E-01	FALSE	FALSE	6	14146403	4.43E-02	4.73E-01	TRUE	6	14146172	2.47E-02	0.99	6613	5.66E-06	TRUE	TRUE	482,730	20.63
Diacylglycerol (18:1_18:2) levels	Parkinson's disease	rs7782961	T	A	T	A	9.18E-02	5.86E-02	2.32E-01	1.82E-01	TRUE	FALSE	7	6335583	2.64E-02	1.32E-02	TRUE	7	6295952	2.08E-02	0.97	6613	9.94E-06	TRUE	TRUE	482,730	19.55
Diacylglycerol (18:1_18:2) levels	Parkinson's disease	rs79624003	G	A	G	A	-1.72E-01	-5.80E-03	1.22E-01	8.81E-01	FALSE	FALSE	7	73012785	2.71E-02	8.04E-02	TRUE	7	73598455	2.65E-02	1.00	6613	8.98E-11	TRUE	TRUE	482,730	42.17
Diacylglycerol (18:1_18:2) levels	Parkinson's disease	rs964184	C	G	C	G	-2.91E-01	-5.68E-02	8.49E-01	8.63E-01	TRUE	FALSE	11	116648917	2.43E-02	9.71E-03	TRUE	11	116778201	2.37E-02	1.00	6613	3.26E-34	TRUE	TRUE	482,730	150.43
Diacylglycerol (18:1_18:2) levels	Parkinson's disease	rs9973939	G	A	G	A	-8.19E-02	-4.53E-02	6.60E-01	3.78E-01	FALSE	FALSE	2	212943534	2.30E-02	2.44E-02	TRUE	2	212078809	1.84E-02	1.00	6613	8.26E-06	TRUE	TRUE	482,730	19.91
Diacylglycerol (18:1_18:3) levels	Parkinson's disease	rs10749053	C	T	C	T	-1.42E-01	-2.15E-02	8.74E-01	1.43E-01	FALSE	FALSE	10	112576695	3.42E-02	2.75E-01	TRUE	10	110816937	2.76E-02	0.92	6572	2.57E-07	TRUE	TRUE	482,730	26.60
Diacylglycerol (18:1_18:3) levels	Parkinson's disease	rs112075774	A	G	A	G	-1.65E-01	-2.37E-02	7.70E-02	9.31E-01	FALSE	FALSE	16	23274991	4.77E-02	2.08E-01	TRUE	16	23263670	3.39E-02	0.93	6572	1.21E-06	TRUE	TRUE	482,730	23.61
Diacylglycerol (18:1_18:3) levels	Parkinson's disease	rs112374014	A	G	A	G	3.18E-01	-1.18E-01	1.61E-02	9.90E-01	FALSE	FALSE	5	177830017	1.58E-01	3.41E-01	TRUE	5	178403016	7.09E-02	0.96	6572	7.33E-06	TRUE	TRUE	482,730	20.14
Diacylglycerol (18:1_18:3) levels	Parkinson's disease	rs114441702	G	A	G	A	-2.57E-01	-5.83E-02	2.65E-02	9.60E-01	FALSE	FALSE	4	142893320	6.30E-02	4.50E-01	TRUE	4	141972167	5.61E-02	0.93	6572	4.52E-06	TRUE	TRUE	482,730	21.06
Diacylglycerol (18:1_18:3) levels	Parkinson's disease	rs116011373	A	G	A	G	-1.91E-01	-2.23E-02	4.88E-02	9.73E-01	FALSE	FALSE	3	87347791	8.48E-02	1.01E-01	TRUE	3	87298641	4.09E-02	0.96	6572	3.06E-06	TRUE	TRUE	482,730	21.82
Diacylglycerol (18:1_18:3) levels	Parkinson's disease	rs117518401	T	C	T	C	1.12E-01	3.10E-02	1.73E-01	8.68E-01	FALSE	FALSE	3	133614605	3.42E-02	4.37E-01	TRUE	3	133895761	2.32E-02	0.98	6572	1.48E-06	TRUE	TRUE	482,730	23.22
Diacylglycerol (18:1_18:3) levels	Parkinson's disease	rs1260326	C	T	C	T	-8.98E-02	-6.78E-02	6.51E-01	4.20E-01	FALSE	FALSE	2	27730940	1.72E-02	4.04E-05	TRUE	2	27508073	1.82E-02	1.00	6572	8.83E-07	TRUE	TRUE	482,730	24.21
Diacylglycerol (18:1_18:3) levels	Parkinson's disease	rs13237829	A	G	A	G	-8.08E-02	3.40E-03	5.03E-01	5.34E-01	FALSE	FALSE	7	73874881	2.39E-02	5.18E-02	TRUE	7	74460551	1.73E-02	1.00	6572	3.03E-06	TRUE	TRUE	482,730	21.83
Diacylglycerol (18:1_18:3) levels	Parkinson's disease	rs141217537	C	T	C	T	2.19E-01	-1.27E-01	3.25E-02	9.74E-01	FALSE	FALSE	1	186210950	7.60E-02	4.72E-02	TRUE	1	186241818	4.86E-02	0.96	6572	6.59E-06	TRUE	TRUE	482,730	20.34
Diacylglycerol (18:1_18:3) levels	Parkinson's disease	rs180761622	T	C	T	C	2.35E-01	8.98E-02	3.31E-02	9.83E-01	FALSE	FALSE	8	32311895	1.99E-01	1.85E-01	TRUE	8	32454379	4.91E-02	1.00	6572	1.73E-06	TRUE	TRUE	482,730	22.91
Diacylglycerol (18:1_18:3) levels	Parkinson's disease	rs268	G	A	G	A	2.81E-01	1.59E-02	2.27E-02	9.83E-01	FALSE	FALSE	8	19813529	6.69E-02	9.03E-02	TRUE	8	19956018	5.94E-02	0.97	6572	2.39E-06	TRUE	TRUE	482,730	22.29
Diacylglycerol (18:1_18:3) levels	Parkinson's disease	rs55865364	G	A	G	A	2.31E-01	-7.15E-02	3.18E-02	9.74E-01	FALSE	FALSE	1	196901329	6.73E-02	5.40E-01	TRUE	1	196932199	5.02E-02	0.97	6572	4.22E-06	TRUE	TRUE	482,730	21.20
Diacylglycerol (18:1_18:3) levels	Parkinson's disease	rs61950149	C	T	C	T	1.01E-01	-1.45E-02	1.85E-01	8.84E-01	FALSE	FALSE	13	21543502	2.74E-02	2.25E-01	TRUE	13	20969363	2.26E-02	0.97	6572	7.99E-06	TRUE	TRUE	482,730	19.97
Diacylglycerol (18:1_18:3) levels	Parkinson's disease	rs6583035	C	T	C	T	-1.12E-01	-3.80E-02	1.62E-01	7.15E-01	FALSE	FALSE	1	108186083	2.45E-02	9.16E-01	TRUE	1	107643461	2.37E-02	1.00	6572	2.46E-06	TRUE	TRUE	482,730	22.23
Diacylglycerol (18:1_18:3) levels	Parkinson's disease	rs6958772	C	G	C	G	9.56E-02	-1.44E-02	2.44E-01	2.85E-01	TRUE	FALSE	7	10702253	2.48E-02	2.52E-01	TRUE	7	10662626	2.02E-02	1.00	6572	2.27E-06	TRUE	TRUE	482,730	22.39
Diacylglycerol (18:1_18:3) levels	Parkinson's disease	rs75224949	T	C	T	C	-1.89E-01	-1.45E-01	4.93E-02	9.67E-01	FALSE	FALSE	10	24453171	6.80E-02	1.63E-02	TRUE	10	24164242	4.21E-02	0.91	6572	7.27E-06	TRUE	TRUE	482,730	20.15
Diacylglycerol (18:1_18:3) levels	Parkinson's disease	rs7599400	A	C	A	C	4.90E-01	-2.50E-03	7.95E-03	9.78E-01	FALSE	FALSE	2	36542577	8.50E-02	1.02E-02	TRUE	2	36315434	9.60E-02	0.99	6572	3.46E-07	TRUE	TRUE	482,730	26.02
Diacylglycerol (18:1_18:3) levels	Parkinson's disease	rs77442856	A	G	A	G	-1.23E-01	7.00E-04	1.22E-01	8.72E-01	FALSE	FALSE	5	152785080	3.41E-02	7.14E-03	TRUE	5	153405520	2.68E-02	0.98	6572	4.21E-06	TRUE	TRUE	482,730	21.20
Diacylglycerol (18:1_18:3) levels	Parkinson's disease	rs964184	C	G	C	G	-1.71E-01	-5.68E-02	8.49E-01	8.63E-01	TRUE	FALSE	11	116648917	2.43E-02	9.71E-03	TRUE	11	116778201	2.40E-02	1.00	6572	1.43E-12	TRUE	TRUE	482,730	50.34
Lysophosphatidylcholine (16:0) levels	Parkinson's disease	rs11051913	C	T	C	T	-8.79E-02	-2.70E-03	2.50E-01	7.98E-01	FALSE	FALSE	12	32455030	2.82E-02	3.49E-02	TRUE	12	32302096	1.94E-02	0.99	7174	6.29E-06	TRUE	TRUE	482,730	20.43
Lysophosphatidylcholine (16:0) levels	Parkinson's disease	rs117001547	G	C	G	C	2.77E-01	1.17E-01	1.97E-02	1.98E-02	TRUE	FALSE	8	101072213	8.77E-02	7.39E-01	TRUE	8	100059985	6.21E-02	0.95	7174	8.34E-06	TRUE	TRUE	482,730	19.89
Lysophosphatidylcholine (16:0) levels	Parkinson's disease	rs117646116	T	C	T	C	1.61E-01	9.02E-02	5.76E-02	9.70E-01	FALSE	FALSE	10	21092416	7.29E-02	6.65E-01	TRUE	10	20803487	3.61E-02	0.98	7174	8.59E-06	TRUE	TRUE	482,730	19.83
Lysophosphatidylcholine (16:0) levels	Parkinson's disease	rs12116819	A	G	A	G	-2.46E-01	-8.35E-02	2.96E-02	9.28E-01	FALSE	FALSE	1	163443904	4.45E-02	3.03E-02	TRUE	1	163474114	5.08E-02	0.94	7174	1.24E-06	TRUE	TRUE	482,730	23.55
Lysophosphatidylcholine (16:0) levels	Parkinson's disease	rs1260326	C	T	C	T	-9.90E-02	-6.78E-02	6.51E-01	4.20E-01	FALSE	FALSE	2	27730940	1.72E-02	4.04E-05	TRUE	2	27508073	1.75E-02	1.00	7174	1.56E-08	TRUE	TRUE	482,730	32.05
Lysophosphatidylcholine (16:0) levels	Parkinson's disease	rs12926135	A	C	A	C	-8.01E-02	3.86E-02	3.77E-01	6.13E-01	FALSE	FALSE	16	23634750	2.94E-02	7.23E-01	TRUE	16	23623429	1.80E-02	0.92	7174	9.11E-06	TRUE	TRUE	482,730	19.72
Lysophosphatidylcholine (16:0) levels	Parkinson's disease	rs149105878	C	A	C	A	-7.33E-01	9.22E-02	3.08E-03	9.87E-01	FALSE	FALSE	12	18004333	1.24E-01	3.40E-01	TRUE	12	17851399	1.63E-01	0.83	7174	7.12E-06	TRUE	TRUE	482,730	20.19
Lysophosphatidylcholine (16:0) levels	Parkinson's disease	rs17029364	G	A	G	A	-9.74E-02	-7.62E-02	1.89E-01	8.52E-01	FALSE	FALSE	4	153893072	2.58E-02	1.57E-03	TRUE	4	152971920	2.16E-02	0.99	7174	6.68E-06	TRUE	TRUE	482,730	20.31
Lysophosphatidylcholine (16:0) levels	Parkinson's disease	rs17477122	G	A	G	A	-1.10E-01	5.61E-02	1.42E-01	8.55E-01	FALSE	FALSE	12	26772060	2.87E-02	2.53E-02	TRUE	12	26619127	2.41E-02	1.00	7174	4.77E-06	TRUE	TRUE	482,730	20.96
Lysophosphatidylcholine (16:0) levels	Parkinson's disease	rs181353915	G	A	G	A	2.90E-01	1.71E-02	1.65E-02	9.94E-01	FALSE	FALSE	17	47475878	2.16E-01	2.82E-02	TRUE	17	49398516	6.54E-02	0.97	7174	9.48E-06	TRUE	TRUE	482,730	19.64
Lysophosphatidylcholine (16:0) levels	Parkinson's disease	rs191695863	A	C	A	C	-5.46E-01	2.62E-01	5.67E-03	9.91E-01	FALSE	FALSE	11	40288986	1.98E-01	7.29E-01	TRUE	11	40267436	1.19E-01	0.88	7174	4.41E-06	TRUE	TRUE	482,730	21.11
Lysophosphatidylcholine (16:0) levels	Parkinson's disease	rs2691773	T	C	T	C	7.50E-02	2.22E-02	5.56E-01	4.05E-01	FALSE	FALSE	2	156456278	2.26E-02	4.86E-01	TRUE	2	155599766	1.68E-02	1.00	7174	8.10E-06	TRUE	TRUE	482,730	19.94
Lysophosphatidylcholine (16:0) levels	Parkinson's disease	rs2714309	A	G	A	G	7.97E-02	-3.26E-02	3.91E-01	6.71E-01	FALSE	FALSE	6	7298287	1.95E-02	4.73E-02	TRUE	6	7298054	1.72E-02	1.00	7174	3.69E-06	TRUE	TRUE	482,730	21.45
Lysophosphatidylcholine (16:0) levels	Parkinson's disease	rs34782816	T	C	T	C	-9.41E-02	1.06E-02	2.03E-01	8.46E-01	FALSE	FALSE	3	120019326	2.95E-02	1.44E-01	TRUE	3	120300479	2.07E-02	0.99	7174	5.65E-06	TRUE	TRUE	482,730	20.63
Lysophosphatidylcholine (16:0) levels	Parkinson's disease	rs4955144	A	C	A	C	8.25E-02	8.60E-03	7.08E-01	2.99E-01	FALSE	FALSE	3	32395216	2.45E-02	1.39E-01	TRUE	3	32353724	1.82E-02	0.99	7174	5.91E-06	TRUE	TRUE	482,730	20.55
Lysophosphatidylcholine (16:0) levels	Parkinson's disease	rs571344	C	T	C	T	8.18E-02	-1.18E-02	6.30E-01	3.56E-01	FALSE	FALSE	1	98964063	2.77E-02	1.74E-01	TRUE	1	98498507	1.78E-02	0.97	7174	4.23E-06	TRUE	TRUE	482,730	21.19
Lysophosphatidylcholine (16:0) levels	Parkinson's disease	rs62231613	A	G	A	G	-4.42E-01	-1.47E-01	8.73E-03	9.77E-01	FALSE	FALSE	3	4804677	7.99E-02	3.30E-02	TRUE	3	4762993	9.31E-02	0.91	7174	2.10E-06	TRUE	TRUE	482,730	22.53
Lysophosphatidylcholine (16:0) levels	Parkinson's disease	rs7168957	T	C	T	C	-1.69E-01	-7.40E-03	9.37E-01	2.40E-02	FALSE	FALSE	15	61890531	6.97E-02	3.82E-02	TRUE	15	61598332	3.43E-02	1.00	7174	9.02E-07	TRUE	TRUE	482,730	24.17
Lysophosphatidylcholine (16:0) levels	Parkinson's disease	rs73200277	T	C	T	C	2.84E-01	7.91E-02	2.17E-02	9.80E-01	FALSE	FALSE	8	17983719	8.55E-02	4.50E-01	TRUE	8	18126210	5.90E-02	0.95	7174	1.45E-06	TRUE	TRUE	482,730	23.25
Lysophosphatidylcholine (16:0) levels	Parkinson's disease	rs73693512	A	G	A	G	2.38E-01	2.20E-03	2.64E-02	9.58E-01	FALSE	FALSE	8	80743307	5.56E-02	1.38E-02	TRUE	8	79831072	5.28E-02	0.99	7174	6.97E-06	TRUE	TRUE	482,730	20.23
Lysophosphatidylcholine (16:0) levels	Parkinson's disease	rs7529794	T	G	T	G	1.34E-01	-2.50E-02	2.50E-01	7.52E-01	FALSE	FALSE	1	40403370	2.57E-02	4.81E-01	TRUE	1	39937698	1.93E-02	0.99	7174	4.32E-12	TRUE	TRUE	482,730	48.14
Lysophosphatidylcholine (16:0) levels	Parkinson's disease	rs75563920	C	T	C	T	-1.26E-01	-2.25E-02	1.17E-01	9.36E-01	FALSE	FALSE	4	81153335	4.68E-02	2.01E-01	TRUE	4	80232181	2.59E-02	1.00	7174	1.29E-06	TRUE	TRUE	482,730	23.48
Lysophosphatidylcholine (16:0) levels	Parkinson's disease	rs75668753	G	A	G	A	-2.97E-01	-5.29E-02	2.11E-02	9.75E-01	FALSE	FALSE	5	117193293	7.47E-02	3.20E-01	TRUE	5	117857598	5.92E-02	0.96	7174	5.49E-07	TRUE	TRUE	482,730	25.13
Lysophosphatidylcholine (16:0) levels	Parkinson's disease	rs76309137	T	C	T	C	-3.40E-01	-1.37E-01	1.27E-02	9.78E-01	FALSE	FALSE	7	95202788	7.96E-02	4.22E-02	TRUE	7	95573476	7.58E-02	0.99	7174	7.34E-06	TRUE	TRUE	482,730	20.13
Lysophosphatidylcholine (16:0) levels	Parkinson's disease	rs76530357	T	G	T	G	2.56E-01	-9.45E-02	2.45E-02	9.84E-01	FALSE	FALSE	3	77057587	1.44E-01	2.91E-01	TRUE	3	77008436	5.45E-02	0.96	7174	2.70E-06	TRUE	TRUE	482,730	22.05
Lysophosphatidylcholine (16:0) levels	Parkinson's disease	rs77116141	C	T	C	T	1.10E-01	-4.18E-02	1.37E-01	7.87E-01	FALSE	FALSE	13	111772414	2.76E-02	8.87E-01	TRUE	13	111120067	2.44E-02	1.00	7174	5.99E-06	TRUE	TRUE	482,730	20.52
Lysophosphatidylcholine (18:0) levels	Parkinson's disease	rs10095602	T	C	T	C	1.76E-01	5.12E-02	5.18E-02	9.54E-01	FALSE	FALSE	8	54581599	5.42E-02	4.63E-01	TRUE	8	53669039	3.80E-02	0.99	7173	3.79E-06	TRUE	TRUE	482,730	21.40
Lysophosphatidylcholine (18:0) levels	Parkinson's disease	rs1018070	A	T	A	T	1.77E-01	-6.56E-02	6.11E-02	4.92E-02	TRUE	FALSE	8	18273627	4.59E-02	8.14E-01	TRUE	8	18416117	3.55E-02	0.99	7173	5.83E-07	TRUE	TRUE	482,730	25.01
Lysophosphatidylcholine (18:0) levels	Parkinson's disease	rs1146461	A	G	A	G	-7.99E-02	-9.90E-03	4.63E-01	4.17E-01	FALSE	FALSE	1	95408492	2.28E-02	1.77E-01	TRUE	1	94942936	1.66E-02	1.00	7173	1.57E-06	TRUE	TRUE	482,730	23.10
Lysophosphatidylcholine (18:0) levels	Parkinson's disease	rs11582724	T	C	T	C	-7.83E-02	3.95E-02	5.75E-01	4.40E-01	FALSE	FALSE	1	201101864	1.84E-02	1.59E-02	TRUE	1	201132736	1.71E-02	0.98	7173	4.64E-06	TRUE	TRUE	482,730	21.01
Lysophosphatidylcholine (18:0) levels	Parkinson's disease	rs117140365	C	G	C	G	-3.29E-01	9.79E-02	1.60E-02	2.39E-02	TRUE	FALSE	10	76620921	8.33E-02	6.20E-01	TRUE	10	74861163	6.72E-02	0.96	7173	9.83E-07	TRUE	TRUE	482,730	24.00
Lysophosphatidylcholine (18:0) levels	Parkinson's disease	rs11780085	T	A	T	A	4.52E-01	-2.68E-01	8.10E-03	1.28E-02	TRUE	FALSE	8	11537613	1.34E-01	2.29E-02	TRUE	8	11680104	1.02E-01	0.86	7173	8.92E-06	TRUE	TRUE	482,730	19.76
Lysophosphatidylcholine (18:0) levels	Parkinson's disease	rs145366219	G	A	G	A	-1.58E-01	6.62E-02	6.68E-02	8.91E-01	FALSE	FALSE	9	33013632	3.63E-02	3.41E-02	TRUE	9	33013634	3.37E-02	1.00	7173	2.68E-06	TRUE	TRUE	482,730	22.07
Lysophosphatidylcholine (18:0) levels	Parkinson's disease	rs56121911	G	A	G	A	1.31E-01	-2.77E-02	9.68E-02	9.28E-01	FALSE	FALSE	3	45292618	3.30E-02	3.97E-01	TRUE	3	45251126	2.86E-02	1.00	7173	5.24E-06	TRUE	TRUE	482,730	20.78
Lysophosphatidylcholine (18:0) levels	Parkinson's disease	rs59634655	T	G	T	G	-1.08E-01	5.70E-03	1.63E-01	8.22E-01	FALSE	FALSE	16	78491577	2.93E-02	7.32E-02	TRUE	16	78457680	2.29E-02	0.99	7173	2.46E-06	TRUE	TRUE	482,730	22.23
Lysophosphatidylcholine (18:0) levels	Parkinson's disease	rs7048143	T	C	T	C	-8.77E-02	1.19E-02	2.47E-01	7.75E-01	FALSE	FALSE	9	94569160	3.33E-02	1.43E-01	TRUE	9	91806878	1.98E-02	0.96	7173	9.79E-06	TRUE	TRUE	482,730	19.58
Lysophosphatidylcholine (18:0) levels	Parkinson's disease	rs73040346	C	T	C	T	-2.17E-01	2.75E-02	3.12E-02	9.52E-01	FALSE	FALSE	2	179691793	4.05E-02	3.03E-01	TRUE	2	178827066	4.86E-02	1.00	7173	8.28E-06	TRUE	TRUE	482,730	19.90
Lysophosphatidylcholine (18:0) levels	Parkinson's disease	rs7529794	T	G	T	G	1.01E-01	-2.50E-02	2.50E-01	7.52E-01	FALSE	FALSE	1	40403370	2.57E-02	4.81E-01	TRUE	1	39937698	1.94E-02	0.99	7173	1.65E-07	TRUE	TRUE	482,730	27.46
Lysophosphatidylcholine (18:0) levels	Parkinson's disease	rs75668753	G	A	G	A	-2.68E-01	-5.29E-02	2.11E-02	9.75E-01	FALSE	FALSE	5	117193293	7.47E-02	3.20E-01	TRUE	5	117857598	5.92E-02	0.96	7173	6.12E-06	TRUE	TRUE	482,730	20.48
Lysophosphatidylcholine (18:0) levels	Parkinson's disease	rs76179782	A	C	A	C	1.99E-01	-1.08E-01	4.43E-02	8.98E-01	FALSE	FALSE	10	72218069	4.90E-02	1.41E-02	TRUE	10	70458313	4.38E-02	0.85	7173	5.97E-06	TRUE	TRUE	482,730	20.53
Lysophosphatidylcholine (18:0) levels	Parkinson's disease	rs76309137	T	C	T	C	-3.51E-01	-1.37E-01	1.27E-02	9.78E-01	FALSE	FALSE	7	95202788	7.96E-02	4.22E-02	TRUE	7	95573476	7.57E-02	0.99	7173	3.63E-06	TRUE	TRUE	482,730	21.48
Lysophosphatidylcholine (18:0) levels	Parkinson's disease	rs76530357	T	G	T	G	2.66E-01	-9.45E-02	2.45E-02	9.84E-01	FALSE	FALSE	3	77057587	1.44E-01	2.91E-01	TRUE	3	77008436	5.45E-02	0.96	7173	1.05E-06	TRUE	TRUE	482,730	23.87
Lysophosphatidylcholine (18:0) levels	Parkinson's disease	rs77751297	T	G	T	G	1.13E-01	-1.44E-02	1.65E-01	8.43E-01	FALSE	FALSE	12	96247258	2.57E-02	2.40E-01	TRUE	12	95853480	2.26E-02	0.99	7173	6.41E-07	TRUE	TRUE	482,730	24.83
Lysophosphatidylcholine (18:0) levels	Parkinson's disease	rs78392466	T	A	T	A	-2.09E-01	2.17E-01	3.77E-02	1.92E-02	TRUE	FALSE	6	101530843	8.64E-02	6.05E-03	TRUE	6	101082967	4.39E-02	0.99	7173	1.98E-06	TRUE	TRUE	482,730	22.65
Lysophosphatidylcholine (18:0) levels	Parkinson's disease	rs79713567	T	C	T	C	-1.87E-01	-6.50E-03	5.04E-02	9.59E-01	FALSE	FALSE	14	37310796	6.26E-02	3.78E-02	TRUE	14	36841591	4.08E-02	0.87	7173	4.75E-06	TRUE	TRUE	482,730	20.96
Lysophosphatidylcholine (18:0) levels	Parkinson's disease	rs797821	C	T	C	T	-7.54E-02	3.53E-02	5.61E-01	3.71E-01	FALSE	FALSE	7	83590852	2.24E-02	9.41E-01	TRUE	7	83961536	1.68E-02	1.00	7173	7.21E-06	TRUE	TRUE	482,730	20.17
Lysophosphatidylcholine (18:1) levels	Parkinson's disease	rs11212840	C	T	C	T	-7.97E-02	1.25E-02	3.17E-01	6.18E-01	FALSE	FALSE	11	108876418	2.33E-02	2.28E-01	TRUE	11	109005691	1.79E-02	0.99	7174	8.07E-06	TRUE	TRUE	482,730	19.95
Lysophosphatidylcholine (18:1) levels	Parkinson's disease	rs115972339	G	A	G	A	-2.11E-01	-6.92E-02	3.50E-02	9.77E-01	FALSE	FALSE	4	78006847	8.09E-02	4.06E-01	TRUE	4	77085694	4.62E-02	0.98	7174	5.16E-06	TRUE	TRUE	482,730	20.81
Lysophosphatidylcholine (18:1) levels	Parkinson's disease	rs11720139	G	C	G	C	7.84E-02	-8.60E-03	3.84E-01	4.06E-01	TRUE	FALSE	3	47036756	1.75E-02	2.07E-01	TRUE	3	46995266	1.71E-02	1.00	7174	4.97E-06	TRUE	TRUE	482,730	20.88
Lysophosphatidylcholine (18:1) levels	Parkinson's disease	rs11727825	A	G	A	G	9.29E-02	2.24E-02	2.63E-01	8.21E-01	FALSE	FALSE	4	24135056	3.06E-02	3.33E-01	TRUE	4	24133433	1.93E-02	0.97	7174	1.43E-06	TRUE	TRUE	482,730	23.28
Lysophosphatidylcholine (18:1) levels	Parkinson's disease	rs142604671	T	C	T	C	1.47E-01	1.99E-02	9.29E-02	9.26E-01	FALSE	FALSE	17	59629860	5.25E-02	1.52E-01	TRUE	17	61552499	3.07E-02	0.88	7174	1.56E-06	TRUE	TRUE	482,730	23.10
Lysophosphatidylcholine (18:1) levels	Parkinson's disease	rs146435288	C	A	C	A	1.53E-01	4.80E-03	7.02E-02	9.36E-01	FALSE	FALSE	5	2070088	4.63E-02	3.73E-02	TRUE	5	2069974	3.34E-02	0.97	7174	4.37E-06	TRUE	TRUE	482,730	21.13
Lysophosphatidylcholine (18:1) levels	Parkinson's disease	rs2714309	A	G	A	G	7.96E-02	-3.26E-02	3.91E-01	6.71E-01	FALSE	FALSE	6	7298287	1.95E-02	4.73E-02	TRUE	6	7298054	1.72E-02	1.00	7174	3.76E-06	TRUE	TRUE	482,730	21.42
Lysophosphatidylcholine (18:1) levels	Parkinson's disease	rs35583283	C	G	C	G	1.02E-01	-5.94E-02	1.93E-01	1.57E-01	TRUE	FALSE	8	18254509	2.53E-02	9.44E-03	TRUE	8	18396999	2.13E-02	1.00	7174	1.70E-06	TRUE	TRUE	482,730	22.95
Lysophosphatidylcholine (18:1) levels	Parkinson's disease	rs55908410	C	T	C	T	-3.14E-01	-7.58E-02	1.51E-02	9.56E-01	FALSE	FALSE	17	59791813	5.92E-02	6.98E-01	TRUE	17	61714452	6.99E-02	0.95	7174	7.15E-06	TRUE	TRUE	482,730	20.18
Lysophosphatidylcholine (18:1) levels	Parkinson's disease	rs6455722	G	T	G	T	-8.11E-02	1.99E-02	3.35E-01	7.17E-01	FALSE	FALSE	6	161761089	2.60E-02	3.52E-01	TRUE	6	161340057	1.76E-02	0.99	7174	4.26E-06	TRUE	TRUE	482,730	21.18
Lysophosphatidylcholine (18:1) levels	Parkinson's disease	rs6539823	C	T	C	T	7.91E-02	6.80E-03	6.50E-01	2.46E-01	FALSE	FALSE	12	84560908	2.12E-02	1.25E-01	TRUE	12	84167129	1.75E-02	1.00	7174	6.07E-06	TRUE	TRUE	482,730	20.50
Lysophosphatidylcholine (18:1) levels	Parkinson's disease	rs71379351	C	T	C	T	-8.07E-02	2.13E-02	4.83E-01	5.42E-01	FALSE	FALSE	17	47348140	1.86E-02	6.00E-01	TRUE	17	49270778	1.68E-02	1.00	7174	1.54E-06	TRUE	TRUE	482,730	23.13
Lysophosphatidylcholine (18:1) levels	Parkinson's disease	rs72845685	C	G	C	G	9.53E-02	-1.93E-02	1.90E-01	1.82E-01	TRUE	FALSE	17	66492500	2.37E-02	3.82E-01	TRUE	17	68496359	2.11E-02	1.00	7174	6.52E-06	TRUE	TRUE	482,730	20.36

Lysophosphatidylcholine (18:1) levels	Parkinson's disease	rs7529794	T	G	T	G	1.26E-01	-2.50E-02	2.50E-01	7.52E-01	FALSE	FALSE	1	40403370	2.57E-02	4.81E-01	TRUE	1	39937698	1.93E-02	0.99	7174	8.15E-11	TRUE	TRUE	482,730	42.35
Lysophosphatidylcholine (18:1) levels	Parkinson's disease	rs76881617	T	C	T	C	1.72E-01	8.95E-02	4.91E-02	9.44E-01	FALSE	FALSE	2	27479543	3.92E-02	1.12E-02	TRUE	2	27256675	3.84E-02	0.99	7174	7.48E-06	TRUE	TRUE	482,730	20.09
Lysophosphatidylcholine (18:1) levels	Parkinson's disease	rs7935110	G	C	G	C	8.23E-02	3.10E-03	6.33E-01	6.58E-01	TRUE	FALSE	11	41108575	2.35E-02	4.80E-02	TRUE	11	41087025	1.72E-02	1.00	7174	1.70E-06	TRUE	TRUE	482,730	22.94
Lysophosphatidylcholine (18:1) levels	Parkinson's disease	rs9527967	G	A	G	A	-8.64E-02	1.58E-02	2.77E-01	7.13E-01	FALSE	FALSE	13	59898697	2.57E-02	2.69E-01	TRUE	13	59324563	1.89E-02	0.98	7174	5.07E-06	TRUE	TRUE	482,730	20.84
Lysophosphatidylcholine (18:1) levels	Parkinson's disease	rs9691107	C	T	C	T	-7.57E-02	1.87E-02	4.36E-01	6.05E-01	FALSE	FALSE	7	100517687	1.79E-02	5.27E-01	TRUE	7	100920067	1.69E-02	0.99	7174	7.83E-06	TRUE	TRUE	482,730	20.01
Lysophosphatidylcholine (18:2) levels	Parkinson's disease	rs114796420	A	G	A	G	2.75E-01	1.82E-02	1.91E-02	9.70E-01	FALSE	FALSE	3	121997996	5.93E-02	1.20E-01	TRUE	3	122279149	6.14E-02	0.99	7174	7.90E-06	TRUE	TRUE	482,730	19.99
Lysophosphatidylcholine (18:2) levels	Parkinson's disease	rs115972339	G	A	G	A	-2.25E-01	-6.92E-02	3.50E-02	9.77E-01	FALSE	FALSE	4	78006847	8.09E-02	4.06E-01	TRUE	4	77085694	4.61E-02	0.98	7174	1.04E-06	TRUE	TRUE	482,730	23.89
Lysophosphatidylcholine (18:2) levels	Parkinson's disease	rs139683825	G	A	G	A	-2.09E-01	-2.59E-02	3.84E-02	9.71E-01	FALSE	FALSE	11	30358058	7.27E-02	1.42E-01	TRUE	11	30336511	4.44E-02	0.98	7174	2.52E-06	TRUE	TRUE	482,730	22.18
Lysophosphatidylcholine (18:2) levels	Parkinson's disease	rs149768482	T	C	T	C	1.98E-01	2.40E-02	6.71E-02	9.76E-01	FALSE	FALSE	2	205552235	1.03E-01	8.82E-02	TRUE	2	204687512	3.50E-02	0.93	7174	1.46E-08	TRUE	TRUE	482,730	32.18
Lysophosphatidylcholine (18:2) levels	Parkinson's disease	rs150917153	T	C	T	C	1.76E-01	-7.60E-03	5.51E-02	9.66E-01	FALSE	FALSE	4	183196547	6.46E-02	4.26E-02	TRUE	4	182275394	3.65E-02	0.98	7174	1.45E-06	TRUE	TRUE	482,730	23.24
Lysophosphatidylcholine (18:2) levels	Parkinson's disease	rs174560	C	T	C	T	1.31E-01	-1.90E-03	3.83E-01	7.01E-01	FALSE	FALSE	11	61581764	1.84E-02	3.82E-02	TRUE	11	61814292	1.72E-02	1.00	7174	3.22E-14	TRUE	TRUE	482,730	57.82
Lysophosphatidylcholine (18:2) levels	Parkinson's disease	rs2610042	G	A	G	A	2.74E-01	-2.34E-02	9.77E-01	2.17E-02	FALSE	FALSE	8	57421947	8.17E-02	1.11E-01	TRUE	8	56509388	5.64E-02	0.99	7174	1.19E-06	TRUE	TRUE	482,730	23.64
Lysophosphatidylcholine (18:2) levels	Parkinson's disease	rs414383	T	A	T	A	-8.12E-02	-4.36E-02	6.71E-01	6.97E-01	TRUE	FALSE	17	29949767	2.45E-02	3.76E-02	TRUE	17	31622748	1.77E-02	0.99	7174	4.37E-06	TRUE	TRUE	482,730	21.13
Lysophosphatidylcholine (18:2) levels	Parkinson's disease	rs59319733	A	G	A	G	-8.54E-02	-2.20E-03	3.39E-01	7.00E-01	FALSE	FALSE	1	246856690	2.14E-02	3.68E-02	TRUE	1	246693388	1.76E-02	1.00	7174	1.20E-06	TRUE	TRUE	482,730	23.62
Lysophosphatidylcholine (18:2) levels	Parkinson's disease	rs6685742	A	G	A	G	8.80E-02	6.59E-02	2.57E-01	6.94E-01	FALSE	FALSE	1	171674774	1.82E-02	1.47E-04	TRUE	1	171705634	1.94E-02	1.00	7174	5.73E-06	TRUE	TRUE	482,730	20.61
Lysophosphatidylcholine (18:2) levels	Parkinson's disease	rs71379351	C	T	C	T	-7.66E-02	2.13E-02	4.83E-01	5.42E-01	FALSE	FALSE	17	47348140	1.86E-02	6.00E-01	TRUE	17	49270778	1.68E-02	1.00	7174	4.95E-06	TRUE	TRUE	482,730	20.89
Lysophosphatidylcholine (18:2) levels	Parkinson's disease	rs72707125	G	A	G	A	2.22E-01	-3.97E-02	3.01E-02	9.53E-01	FALSE	FALSE	4	185760834	5.46E-02	3.31E-01	TRUE	4	184839680	4.97E-02	0.94	7174	7.93E-06	TRUE	TRUE	482,730	19.98
Lysophosphatidylcholine (18:2) levels	Parkinson's disease	rs73218874	G	A	G	A	-2.07E-01	1.68E-01	3.81E-02	9.70E-01	FALSE	FALSE	3	110441743	8.00E-02	1.80E-02	TRUE	3	110722896	4.69E-02	0.87	7174	9.92E-06	TRUE	TRUE	482,730	19.55
Lysophosphatidylcholine (18:2) levels	Parkinson's disease	rs7529794	T	G	T	G	1.17E-01	-2.50E-02	2.50E-01	7.52E-01	FALSE	FALSE	1	40403370	2.57E-02	4.81E-01	TRUE	1	39937698	1.93E-02	0.99	7174	1.76E-09	TRUE	TRUE	482,730	36.31
Lysophosphatidylcholine (18:2) levels	Parkinson's disease	rs8069148	C	A	C	A	8.94E-02	-6.30E-03	2.52E-01	7.53E-01	FALSE	FALSE	17	66487837	2.21E-02	1.10E-01	TRUE	17	68491696	1.92E-02	1.00	7174	3.14E-06	TRUE	TRUE	482,730	21.76
Lysophosphatidylcholine (20:4) levels	Parkinson's disease	rs10733608	T	G	T	G	8.28E-02	-1.03E-02	4.85E-01	5.16E-01	FALSE	FALSE	9	117148430	1.71E-02	2.62E-01	TRUE	9	114386150	1.78E-02	1.00	6235	3.34E-06	TRUE	TRUE	482,730	21.65
Lysophosphatidylcholine (20:4) levels	Parkinson's disease	rs10884272	A	G	A	G	-8.34E-02	-1.15E-02	3.65E-01	5.25E-01	FALSE	FALSE	10	107980605	2.25E-02	2.16E-01	TRUE	10	106220847	1.85E-02	0.99	6235	6.46E-06	TRUE	TRUE	482,730	20.38
Lysophosphatidylcholine (20:4) levels	Parkinson's disease	rs12905466	T	C	T	C	-1.02E-01	3.96E-02	2.04E-01	7.65E-01	FALSE	FALSE	15	71772886	2.64E-02	8.76E-01	TRUE	15	71480547	2.24E-02	0.99	6235	5.01E-06	TRUE	TRUE	482,730	20.87
Lysophosphatidylcholine (20:4) levels	Parkinson's disease	rs142999489	G	C	G	C	-1.94E-01	-2.23E-02	5.62E-02	3.00E-02	TRUE	FALSE	7	95603352	7.08E-02	1.23E-01	TRUE	7	95974040	3.98E-02	0.97	6235	1.09E-06	TRUE	TRUE	482,730	23.80
Lysophosphatidylcholine (20:4) levels	Parkinson's disease	rs150158785	A	G	A	G	-1.48E-01	4.96E-02	8.01E-02	9.39E-01	FALSE	FALSE	17	47547873	4.88E-02	5.09E-01	TRUE	17	49470511	3.29E-02	0.97	6235	7.51E-06	TRUE	TRUE	482,730	20.09
Lysophosphatidylcholine (20:4) levels	Parkinson's disease	rs191895023	A	C	A	C	8.05E-01	4.97E-02	2.36E-03	9.92E-01	FALSE	FALSE	7	2329617	1.97E-01	9.68E-02	TRUE	7	2289982	1.77E-01	0.89	6235	5.63E-06	TRUE	TRUE	482,730	20.65
Lysophosphatidylcholine (20:4) levels	Parkinson's disease	rs3741252	T	C	T	C	1.66E-01	-2.38E-02	1.34E-01	9.32E-01	FALSE	FALSE	11	61511498	4.03E-02	2.56E-01	TRUE	11	61744026	2.63E-02	0.99	6235	3.34E-10	TRUE	TRUE	482,730	39.59
Lysophosphatidylcholine (20:4) levels	Parkinson's disease	rs4708901	A	G	A	G	-9.87E-02	-9.70E-03	2.15E-01	8.09E-01	FALSE	FALSE	6	161692473	2.84E-02	1.34E-01	TRUE	6	161271441	2.17E-02	0.99	6235	5.37E-06	TRUE	TRUE	482,730	20.74
Lysophosphatidylcholine (20:4) levels	Parkinson's disease	rs529446	C	T	C	T	9.72E-02	1.80E-03	7.84E-01	2.43E-01	FALSE	FALSE	12	5221319	2.60E-02	2.42E-02	TRUE	12	5112153	2.17E-02	1.00	6235	7.85E-06	TRUE	TRUE	482,730	20.01
Lysophosphatidylcholine (20:4) levels	Parkinson's disease	rs600626	G	A	G	A	-1.21E-01	1.27E-02	2.32E-01	8.34E-01	FALSE	FALSE	11	75455309	2.83E-02	1.85E-01	TRUE	11	75744264	2.12E-02	0.99	6235	1.23E-08	TRUE	TRUE	482,730	32.52
Lysophosphatidylcholine (20:4) levels	Parkinson's disease	rs62511275	G	C	G	C	2.20E-01	1.75E-02	4.20E-02	5.41E-02	TRUE	FALSE	8	117163869	5.70E-02	1.20E-01	TRUE	8	116151644	4.64E-02	0.89	6235	2.08E-06	TRUE	TRUE	482,730	22.56
Lysophosphatidylcholine (20:4) levels	Parkinson's disease	rs6564238	G	C	G	C	8.56E-02	-5.02E-02	3.29E-01	2.74E-01	TRUE	FALSE	16	75253680	2.56E-02	2.49E-02	TRUE	16	75219782	1.91E-02	0.99	6235	7.56E-06	TRUE	TRUE	482,730	20.08
Lysophosphatidylcholine (20:4) levels	Parkinson's disease	rs7179330	T	A	T	A	3.13E-01	9.95E-02	9.80E-01	9.84E-01	TRUE	FALSE	15	24082764	9.29E-02	5.47E-01	TRUE	15	23837617	6.43E-02	0.97	6235	1.15E-06	TRUE	TRUE	482,730	23.71
Lysophosphatidylcholine (20:4) levels	Parkinson's disease	rs76469829	T	C	T	C	-2.16E-01	-3.20E-03	3.71E-02	9.66E-01	FALSE	FALSE	1	30434878	6.63E-02	1.70E-02	TRUE	1	29962031	4.76E-02	0.95	6235	5.69E-06	TRUE	TRUE	482,730	20.62
Lysophosphatidylcholine (20:4) levels	Parkinson's disease	rs76513766	A	G	A	G	3.78E-01	1.60E-03	1.17E-02	9.55E-01	FALSE	FALSE	1	172099894	4.89E-02	1.18E-02	TRUE	1	172130754	8.42E-02	0.99	6235	7.01E-06	TRUE	TRUE	482,730	20.22
Lysophosphatidylcholine (20:4) levels	Parkinson's disease	rs7936002	G	A	G	A	1.34E-01	-5.27E-02	2.57E-01	7.70E-01	FALSE	FALSE	11	62219029	3.25E-02	9.78E-01	TRUE	11	62451557	2.09E-02	0.93	6235	1.65E-10	TRUE	TRUE	482,730	40.98
Lysophosphatidylcholine (20:4) levels	Parkinson's disease	rs9967725	T	A	T	A	-1.01E-01	-2.40E-02	3.50E-01	3.00E-01	TRUE	FALSE	2	56609599	2.47E-02	4.78E-01	TRUE	2	56382464	1.87E-02	1.00	6235	6.79E-08	TRUE	TRUE	482,730	29.19
Lysophosphatidylethanolamine (18:0) levels	Parkinson's disease	rs116987919	G	C	G	C	3.40E-01	-1.34E-02	1.63E-02	2.77E-02	TRUE	FALSE	7	70463610	7.08E-02	7.04E-02	TRUE	7	70998624	7.27E-02	0.97	6258	2.98E-06	TRUE	TRUE	482,730	21.87
Lysophosphatidylethanolamine (18:0) levels	Parkinson's disease	rs12196051	A	G	A	G	-2.92E-01	-3.30E-02	2.44E-02	9.31E-01	FALSE	FALSE	6	143213699	4.47E-02	3.37E-01	TRUE	6	142892562	5.92E-02	0.95	6258	8.50E-07	TRUE	TRUE	482,730	24.29
Lysophosphatidylethanolamine (18:0) levels	Parkinson's disease	rs1260326	C	T	C	T	-9.43E-02	-6.78E-02	6.51E-01	4.20E-01	FALSE	FALSE	2	27730940	1.72E-02	4.04E-05	TRUE	2	27508073	1.88E-02	1.00	6258	5.12E-07	TRUE	TRUE	482,730	25.27
Lysophosphatidylethanolamine (18:0) levels	Parkinson's disease	rs12726330	A	G	A	G	-2.32E-01	-6.81E-01	4.29E-02	9.80E-01	FALSE	FALSE	1	155108167	6.28E-02	1.05E-27	TRUE	1	155135691	4.50E-02	1.00	6258	2.54E-07	TRUE	TRUE	482,730	26.62
Lysophosphatidylethanolamine (18:0) levels	Parkinson's disease	rs149182005	C	T	C	T	2.77E-01	3.56E-02	2.43E-02	9.73E-01	FALSE	FALSE	6	159978505	7.57E-02	1.95E-01	TRUE	6	159557473	6.07E-02	0.93	6258	5.17E-06	TRUE	TRUE	482,730	20.81
Lysophosphatidylethanolamine (18:0) levels	Parkinson's disease	rs16918167	T	G	T	G	-1.19E-01	-3.40E-03	1.51E-01	7.65E-01	FALSE	FALSE	9	101815339	2.23E-02	5.57E-02	TRUE	9	99053057	2.50E-02	1.00	6258	1.96E-06	TRUE	TRUE	482,730	22.67
Lysophosphatidylethanolamine (18:0) levels	Parkinson's disease	rs1800588	T	C	T	C	1.09E-01	-3.30E-03	2.58E-01	7.75E-01	FALSE	FALSE	15	58723675	2.12E-02	5.73E-02	TRUE	15	58431476	2.08E-02	0.99	6258	1.76E-07	TRUE	TRUE	482,730	27.35
Lysophosphatidylethanolamine (18:0) levels	Parkinson's disease	rs3135062	G	C	G	C	1.04E-01	-3.36E-02	1.91E-01	2.70E-01	TRUE	FALSE	4	3287459	2.13E-02	9.40E-01	TRUE	4	3285732	2.29E-02	0.99	6258	5.91E-06	TRUE	TRUE	482,730	20.55
Lysophosphatidylethanolamine (18:0) levels	Parkinson's disease	rs35152003	A	T	A	T	-9.69E-02	1.30E-03	6.62E-01	6.60E-01	TRUE	FALSE	8	18291401	2.28E-02	1.96E-02	TRUE	8	18433891	1.91E-02	0.97	6258	4.05E-07	TRUE	TRUE	482,730	25.72
Lysophosphatidylethanolamine (18:0) levels	Parkinson's disease	rs4663191	G	A	G	A	9.72E-02	-2.71E-02	7.46E-01	2.65E-01	FALSE	FALSE	2	236174758	3.10E-02	4.19E-01	TRUE	2	235266114	2.07E-02	0.97	6258	2.64E-06	TRUE	TRUE	482,730	22.10
Lysophosphatidylethanolamine (18:0) levels	Parkinson's disease	rs62411861	T	C	T	C	-7.38E-01	3.22E-01	3.39E-03	9.91E-01	FALSE	FALSE	6	49158656	1.96E-01	9.97E-01	TRUE	6	49191020	1.64E-01	0.94	6258	6.88E-06	TRUE	TRUE	482,730	20.26
Lysophosphatidylethanolamine (18:0) levels	Parkinson's disease	rs72757442	T	C	T	C	2.17E-01	-2.81E-02	4.89E-02	9.43E-01	FALSE	FALSE	5	54067240	5.29E-02	2.25E-01	TRUE	5	54771412	4.33E-02	0.90	6258	5.83E-07	TRUE	TRUE	482,730	25.02
Lysophosphatidylethanolamine (18:0) levels	Parkinson's disease	rs7529794	T	G	T	G	1.51E-01	-2.50E-02	2.50E-01	7.52E-01	FALSE	FALSE	1	40403370	2.57E-02	4.81E-01	TRUE	1	39937698	2.07E-02	0.99	6258	3.79E-13	TRUE	TRUE	482,730	52.97
Lysophosphatidylethanolamine (18:0) levels	Parkinson's disease	rs758186	T	C	T	C	8.08E-02	-7.00E-03	5.93E-01	3.63E-01	FALSE	FALSE	17	12500658	2.09E-02	1.33E-01	TRUE	17	12597341	1.80E-02	0.99	6258	7.19E-06	TRUE	TRUE	482,730	20.17
Lysophosphatidylethanolamine (18:0) levels	Parkinson's disease	rs79577600	T	G	T	G	1.46E-01	2.84E-02	9.58E-02	9.25E-01	FALSE	FALSE	17	48043662	4.54E-02	2.75E-01	TRUE	17	49966298	3.12E-02	0.95	6258	2.89E-06	TRUE	TRUE	482,730	21.93
Lysophosphatidylethanolamine (18:1) levels	Parkinson's disease	rs10015043	G	A	G	A	8.86E-02	6.10E-03	4.63E-01	5.64E-01	FALSE	FALSE	4	169485237	2.26E-02	1.04E-01	TRUE	4	168564086	1.98E-02	1.00	5172	7.91E-06	TRUE	TRUE	482,730	20.00
Lysophosphatidylethanolamine (18:1) levels	Parkinson's disease	rs115554476	T	C	T	C	-3.55E-01	1.22E-01	1.48E-02	9.72E-01	FALSE	FALSE	5	4129194	7.34E-02	4.81E-02	TRUE	5	4129081	7.99E-02	0.96	5172	9.06E-06	TRUE	TRUE	482,730	19.74
Lysophosphatidylethanolamine (18:1) levels	Parkinson's disease	rs116537941	G	A	G	A	-1.98E-01	-6.30E-03	5.41E-02	9.78E-01	FALSE	FALSE	1	230259882	8.92E-02	2.50E-02	TRUE	1	230124135	4.40E-02	0.95	5172	6.84E-06	TRUE	TRUE	482,730	20.28
Lysophosphatidylethanolamine (18:1) levels	Parkinson's disease	rs117398262	A	C	A	C	2.66E-01	1.07E-01	2.87E-02	9.76E-01	FALSE	FALSE	15	38703787	7.90E-02	7.57E-01	TRUE	15	38411586	5.91E-02	0.98	5172	6.72E-06	TRUE	TRUE	482,730	20.31
Lysophosphatidylethanolamine (18:1) levels	Parkinson's disease	rs11917269	T	G	T	G	1.09E-01	3.45E-02	2.77E-01	7.46E-01	FALSE	FALSE	3	50481035	2.01E-02	4.30E-02	TRUE	3	50443604	2.18E-02	1.00	5172	6.75E-07	TRUE	TRUE	482,730	24.75
Lysophosphatidylethanolamine (18:1) levels	Parkinson's disease	rs149492888	A	G	A	G	-5.25E-01	-1.27E-02	9.18E-03	9.88E-01	FALSE	FALSE	5	98528653	1.14E-01	4.02E-02	TRUE	5	99192949	1.13E-01	0.93	5172	3.40E-06	TRUE	TRUE	482,730	21.62
Lysophosphatidylethanolamine (18:1) levels	Parkinson's disease	rs151000252	T	G	T	G	8.73E-01	-1.57E-01	2.48E-03	9.92E-01	FALSE	FALSE	6	86033449	1.75E-01	4.33E-01	TRUE	6	85323731	1.95E-01	0.92	5172	7.49E-06	TRUE	TRUE	482,730	20.10
Lysophosphatidylethanolamine (18:1) levels	Parkinson's disease	rs2296637	T	C	T	C	-1.45E-01	7.32E-02	1.09E-01	9.04E-01	FALSE	FALSE	10	129160101	3.78E-02	2.64E-02	TRUE	10	127361837	3.23E-02	0.97	5172	7.14E-06	TRUE	TRUE	482,730	20.19
Lysophosphatidylethanolamine (18:1) levels	Parkinson's disease	rs2585266	T	C	T	C	-2.41E-01	1.21E-02	4.43E-02	9.14E-01	FALSE	FALSE	17	5213391	4.41E-02	1.06E-01	TRUE	17	5310096	4.87E-02	0.96	5172	7.67E-07	TRUE	TRUE	482,730	24.50
Lysophosphatidylethanolamine (18:1) levels	Parkinson's disease	rs6434115	A	G	A	G	9.33E-02	1.92E-02	6.13E-01	3.47E-01	FALSE	FALSE	2	153462347	2.41E-02	3.71E-01	TRUE	2	152605833	2.07E-02	0.95	5172	6.92E-06	TRUE	TRUE	482,730	20.25
Lysophosphatidylethanolamine (18:1) levels	Parkinson's disease	rs6768813	C	T	C	T	1.10E-01	-3.01E-02	6.29E-01	4.62E-01	FALSE	FALSE	3	142665991	2.23E-02	7.50E-01	TRUE	3	142947149	2.04E-02	0.99	5172	7.74E-08	TRUE	TRUE	482,730	28.95
Lysophosphatidylethanolamine (18:1) levels	Parkinson's disease	rs75388820	A	G	A	G	2.28E-01	8.46E-02	4.68E-02	9.46E-01	FALSE	FALSE	2	27325141	4.39E-02	2.70E-02	TRUE	2	27102273	4.69E-02	0.97	5172	1.19E-06	TRUE	TRUE	482,730	23.65
Lysophosphatidylethanolamine (18:1) levels	Parkinson's disease	rs7776911	A	G	A	G	-1.37E-01	-1.77E-02	8.41E-01	1.42E-01	FALSE	FALSE	7	50884453	3.23E-02	2.35E-01	TRUE	7	50816756	2.68E-02	1.00	5172	3.22E-07	TRUE	TRUE	482,730	26.18
Lysophosphatidylethanolamine (18:1) levels	Parkinson's disease	rs9826895	A	G	A	G	-1.03E-01	1.64E-02	2.72E-01	7.46E-01	FALSE	FALSE	3	23822938	2.59E-02	2.78E-01	TRUE	3	23781447	2.20E-02	0.99	5172	2.95E-06	TRUE	TRUE	482,730	21.90
Lysophosphatidylethanolamine (18:2) levels	Parkinson's disease	rs11063393	G	A	G	A	-1.34E-01	2.44E-02	1.02E-01	8.27E-01	FALSE	FALSE	12	5001323	3.68E-02	2.95E-01	TRUE	12	4892157	2.87E-02	0.96	6905	3.22E-06	TRUE	TRUE	482,730	21.72
Lysophosphatidylethanolamine (18:2) levels	Parkinson's disease	rs111877698	C	A	C	A	4.23E-01	-8.54E-02	1.26E-02	9.83E-01	FALSE	FALSE	1	201449886	1.01E-01	4.03E-01	TRUE	1	201480758	8.67E-02	0.82	6905	1.10E-06	TRUE	TRUE	482,730	23.79
Lysophosphatidylethanolamine (18:2) levels	Parkinson's disease	rs114899411	A	G	A	G	-1.89E-01	3.55E-02	5.75E-02	9.78E-01	FALSE	FALSE	4	152953100	8.04E-02	1.81E-01	TRUE	4	152031948	3.75E-02	0.99	6905	5.08E-07	TRUE	TRUE	482,730	25.28
Lysophosphatidylethanolamine (18:2) levels	Parkinson's disease	rs115359603	G	A	G	A	-3.02E-01	2.49E-02	1.73E-02	9.84E-01	FALSE	FALSE	5	21867938	1.04E-01	9.12E-02	TRUE	5	21867829	6.63E-02	0.94	6905	5.26E-06	TRUE	TRUE	482,730	20.77
Lysophosphatidylethanolamine (18:2) levels	Parkinson's disease	rs1260326	C	T	C	T	-1.15E-01	-6.78E-02	6.51E-01	4.20E-01	FALSE	FALSE	2	27730940	1.72E-02	4.04E-05	TRUE	2	27508073	1.78E-02	1.00	6905	1.12E-10	TRUE	TRUE	482,730	41.72
Lysophosphatidylethanolamine (18:2) levels	Parkinson's disease	rs1338449	C	T	C	T	8.22E-02	-1.75E-02	3.49E-01	6.12E-01	FALSE	FALSE	1	218351323	1.78E-02	4.88E-01	TRUE	1	218177981	1.80E-02	1.00	6905	4.98E-06	TRUE	TRUE	482,730	20.88
Lysophosphatidylethanolamine (18:2) levels	Parkinson's disease	rs1442407	T	G	T	G	-1.16E-01	6.89E-02	1.79E-01	8.46E-01	FALSE	FALSE	8	3091584	2.76E-02	6.27E-03	TRUE	8	3234062	2.21E-02	1.00	6905	1.60E-07	TRUE	TRUE	482,730	27.52
Lysophosphatidylethanolamine (18:2) levels	Parkinson's disease	rs146330404	C	G	C	G	5.60E-01	-9.60E-03	5.95E-03	1.80E-02	TRUE	FALSE	8	2390619	9.54E-02	3.64E-02	TRUE	8	2533493	1.15E-01	0.89	6905	1.14E-06	TRUE	TRUE	482,730	23.71
Lysophosphatidylethanolamine (18:2) levels	Parkinson's disease	rs174560	C	T	C	T	3.65E-01	-1.90E-03	3.83E-01	7.01E-01	FALSE	FALSE	11	61581764	1.84E-02	3.82E-02	TRUE	11	61814292	1.71E-02	1.00	6905	7.02E-98	TRUE	TRUE	482,730	455.25
Lysophosphatidylethanolamine (18:2) levels	Parkinson's disease	rs1792909	A	G	A	G	9.02E-02	2.18E-02	3.87E-01	4.86E-01	FALSE	FALSE	11	61937545	1.77E-02	6.66E-01	TRUE	11	62170073	1.75E-02	1.00	6905	2.60E-07	TRUE	TRUE	482,730	26.58
Lysophosphatidylethanolamine (18:2) levels	Parkinson's disease	rs188404615	C	T	C	T	5.44E-01	1.51E-01	6.81E-03	9.91E-01	FALSE	FALSE	12	100113071	1.83E-01	3.88E-01	TRUE	12	99719293	1.04E-01	0.95	6905	1.53E-07	TRUE	TRUE	482,730	27.60
Lysophosphatidylethanolamine (18:2) levels	Parkinson's disease	rs2236950	A	C	A	C	-8.73E-02	-3.21E-02	2.65E-01	8.26E-01	FALSE	FALSE	3	50420554	2.29E-02	7.91E-01	TRUE	3	50383123	1.92E-02	0.99	6905	5.47E-06	TRUE	TRUE	482,730	20.70
Lysophosphatidylethanolamine (18:2) levels	Parkinson's disease	rs2290053	T	C	T	C	7.62E-02	2.99E-02	4.97E-01	5.10E-01	FALSE	FALSE	3	125833260	2.10E-02	8.11E-01	TRUE	3	126114417	1.70E-02	1.00	6905	7.24E-06	TRUE	TRUE	482,730	20.16
Lysophosphatidylethanolamine (18:2) levels	Parkinson's disease	rs326048	G	A	G	A	-8.96E-02	-9.90E-03	2.85E-01	7.70E-01	FALSE	FALSE	5	996172	2.99E-02	1.30E-01	TRUE	5	996057	1.95E-02	0.93	6905	4.60E-06	TRUE	TRUE	482,730	21.03
Lysophosphatidylethanolamine (18:2) levels	Parkinson's disease	rs3741259	T	C	T	C	1.39E-01	-2.16E-02	1.16E-01	9.09E-01	FALSE	FALSE	11	61282350	3.85E-02	2.40E-01	TRUE	11	61514878	2.66E-02	0.99	6905	1.86E-07	TRUE	TRUE	482,730	27.22
Lysophosphatidylethanolamine (18:2) levels	Parkinson's disease	rs4726320	A	T	A	T	1.28E-01	-4.00E-04	8.95E-01	8.70E-01	TRUE	FALSE	7	153338749	3.44E-02	3.84E-03	TRUE	7	153641664	2.83E-02	0.96	6905	5.80E-06	TRUE	TRUE	482,730	20.58
Lysophosphatidylethanolamine (18:2) levels	Parkinson's disease	rs6413913	A	G	A	G	-8.79E-02	-8.10E-03	3.09E-01	6.95E-01	FALSE	FALSE	1	189878594	2.43E-02	1.32E-01	TRUE	1	189909464	1.83E-02	1.00	6905	1.50E-06	TRUE	TRUE	482,730	23.19
Lysophosphatidylethanolamine (18:2) levels	Parkinson's disease	rs71508993	T	C	T	C	2.45E-01	6.83E-02	2.55E-02	9.30E-01	FALSE	FALSE	10	65956838	4.45E-02	9.03E-01	TRUE	10	64197077	5.40E-02	0.98	6905	5.81E-06	TRUE	TRUE	482,730	20.58
Lysophosphatidylethanolamine (18:2) levels	Parkinson's disease	rs7776911	A	G	A	G	-1.04E-01	-1.77E-02	8.41E-01	1.42E-01	FALSE	FALSE	7	50884453	3.23E-02	2.35E-01	TRUE	7	50816756	2.34E-02	1.00	6905	9.36E-06	TRUE	TRUE	482,730	19.67
Lysophosphatidylethanolamine (18:2) levels	Parkinson's disease	rs791631	C	G	C	G	-7.98E-02	-2.65E-02	6.28E-01	6.02E-01	TRUE	FALSE	5	173101915	2.30E-02	6.02E-01	TRUE	5	173674912	1.75E-02	0.99	6905	5.58E-06	TRUE	TRUE	482,730	20.66
Lysophosphatidylethanolamine (18:2) levels	Parkinson's disease	rs79211442	C	T	C	T	1.11E-01	3.09E-02	1.36E-01	9.05E-01	FALSE	FALSE	6	22879454	4.64E-02	2.97E-01	TRUE	6	22879225	2.49E-02	0.99	6905	9.23E-06	TRUE	TRUE	482,730	19.69
Lysophosphatidylethanolamine (18:2) levels	Parkinson's disease	rs8034802	A	T	A	T	8.81E-02	-1.41E-02	3.11E-01	2.96E-01	TRUE	FALSE	15	58724792	2.32E-02	2.65E-01	TRUE	15	58432593	1.86E-02	1.00	6905	2.31E-06	TRUE	TRUE	482,730	22.35
Phosphatidylcholine (14:0_16:0) levels	Parkinson's disease	rs148426771	G	T	G	T	-2.12E-01	5.61E-02	4.93E-02	9.64E-01	FALSE	FALSE	5	21444211	7.15E-02	3.64E-01	TRUE	5	21444102	4.71E-02	0.89	5497	6.49E-06	TRUE	TRUE	482,730	20.38
Phosphatidylcholine (14:0_16:0) levels	Parkinson's disease	rs1553262	T	C	T	C	5.17E-01	-2.78E-01	7.52E-03	9.88E-01	FALSE	FALSE	15	98169179	1.05E-01	3.99E-03	TRUE	15	97625949	1.15E-01	0.94	5497	6.57E-06	TRUE	TRUE	482,730	20.36
Phosphatidylcholine (14:0_16:0) levels	Parkinson's disease	rs28416404	C	T	C	T	-1.66E-01	-2.74E-02	7.93E-02	9.12E-01	FALSE	FALSE	15	58023255	4.09E-02	2.99E-01	TRUE	15	57731057	3.54E-02	0.98	5497	2.84E-06	TRUE	TRUE	482,730	21.97
Phosphatidylcholine (14:0_16:0) levels	Parkinson's disease	rs55654962	A	G	A	G	1.34E-01	1.61E-02	1.09E-01	8.60E-01	FALSE	FALSE	8	27700609	3.37E-02	1.98E-01	TRUE	8	27843092	3.01E-02	0.99	5497	8.32E-06	TRUE	TRUE	482,730	19.90
Phosphatidylcholine (14:0_16:0) levels	Parkinson's disease	rs60984271	T	A	T	A	1.03E-01	7.90E-03	2.20E-01	1.81E-01	TRUE	FALSE	7	102349124	2.95E-02	1.04E-01	TRUE	7	102708677	2.30E-02	0.99	5497	8.34E-06	TRUE	TRUE	482,730	19.90
Phosphatidylcholine (14:0_16:0) levels	Parkinson's disease	rs7075480	A	G	A	G	5.27E-01	7.39E-02	7.37E-03	9.75E-01	FALSE	FALSE	10	100176627	5.94E-02	6.72E-01	TRUE	10	98416870	1.17E-01	0.99	5497	7.18E-06	TRUE	TRUE	482,730	20.19
Phosphatidylcholine (14:0_16:0) levels	Parkinson's disease	rs72666927	T	C	T	C	-2.29E-01	-5.99E-02	3.96E-02	9.59E-01	FALSE	FALSE	4	94117626	6.06E-02	4.91E-01	TRUE	4	93196475	5.11E-02	0.96	5497	7.34E-06	TRUE	TRUE	482,730	20.14
Phosphatidylcholine (14:0_16:0) levels	Parkinson's disease	rs72764354	A	G	A	G	2.10E-01	-4.99E-02	4.52E-02	9.25E-01	FALSE	FALSE	9	129789234	4.26E-02	6.18E-01	TRUE	9	127026955	4.63E-02	0.99	5497	5.86E-06	TRUE	TRUE	482,730	20.58
Phosphatidylcholine (14:0_16:0) levels	Parkinson's disease	rs74939791	G	T	G	T	2.70E-01	-5.27E-02	2.98E-02	9.60E-01	FALSE	FALSE	8	83844031	5.88E-02	4.31E-01	TRUE	8	82931796	5.67E-02	0.98	5497	2.03E-06	TRUE	TRUE	482,730	22.62
Phosphatidylcholine (14:0_16:0) levels	Parkinson's disease	rs77191266	T	C	T	C	2.42E-01	1.37E-01	3.67E-02	9.81E-01	FALSE	FALSE	6	87333899	9.07E-02	8.78E-01	TRUE	6	86624181	5.26E-02	0.95	5497	4.43E-06	TRUE	TRUE	482,730	21.11
Phosphatidylcholine (14:0_16:0) levels	Parkinson's disease	rs9507859	T	C	T	C	1.11E-01	-5.60E-03	7.65E-01	2.61E-01	FALSE	FALSE	13	27802798	2.54E-02	8.34E-02	TRUE	13	27228661	2.23E-02	1.00	5497	7.13E-07	TRUE	TRUE	482,730	24.63
Phosphatidylcholine (14:0_18:1) levels	Parkinson's disease	rs113668788	A	G	A	G	1.35E-01	5.35E-02	1.08E-01	9.65E-01	FALSE	FALSE	9	140605676	7.44E-02	3.26E-01	TRUE	9	137711224	2.82E-02	0.93	7064	1.81E-06	TRUE	TRUE	482,730	22.82
Phosphatidylcholine (14:0_18:1) levels	Parkinson's disease	rs12446515	T	C	T	C	9.71E-02	1.26E-02	2.79E-01	6.87E-01	FALSE	FALSE	16	56987015	1.82E-02	3.11E-01	TRUE	16	56953103	1.87E-02	1.00	7064	2.30E-07	TRUE	TRUE	482,730	26.82
Phosphatidylcholine (14:0_18:1) levels	Parkinson's disease	rs1260326	C	T	C	T	-1.05E-01	-6.78E-02	6.51E-01	4.20E-01	FALSE	FALSE	2	27730940	1.72E-02	4.04E-05	TRUE	2	27508073	1.76E-02	1.00	7064	2.39E-09	TRUE	TRUE	482,730	35.72
Phosphatidylcholine (14:0_18:1) levels	Parkinson's disease	rs2946493	C	T	C	T	9.87E-02	-2.73E-02	2.06E-01	8.07E-01	FALSE	FALSE	8	12823671	2.60E-02	5.32E-01	TRUE	8	12966162	2.11E-02	0.98	7064	2.91E-06	TRUE	TRUE	482,730	21.91
Phosphatidylcholine (14:0_18:1) levels	Parkinson's disease	rs3816689	T	C	T	C	-1.21E-01	3.66E-02	1.11E-01	8.53E-01	FALSE	FALSE	17	4383790	3.14E-02	6.12E-01	TRUE	17	4480495	2.69E-02	0.99	7064	7.13E-06	TRUE	TRUE	482,730	20.19
Phosphatidylcholine (14:0_18:1) levels	Parkinson's disease	rs4564909	G	A	G	A	-8.06E-02	-4.20E-03	6.53E-01	3.11E-01	FALSE	FALSE	3	137866433	2.00E-02	7.82E-02	TRUE	3	138147591	1.78E-02	1.00	7064	5.94E-06	TRUE	TRUE	482,730	20.54
Phosphatidylcholine (14:0_18:1) levels	Parkinson's disease	rs4846905	G	A	G	A	8.80E-02	-2.83E-02	7.68E-01	1.86E-01	FALSE	FALSE	1	230279582	2.86E-02	4.91E-01	TRUE	1	230143835	1.98E-02	1.00	7064	8.68E-06	TRUE	TRUE	482,730	19.81
Phosphatidylcholine (14:0_18:1) levels	Parkinson's disease	rs62189362	C	T	C	T	1.24E-01	-5.30E-02	1.08E-01	8.36E-01	FALSE	FALSE	2	228265954	2.92E-02	3.48E-02	TRUE	2	227401238	2.71E-02	0.99	7064	4.45E-06	TRUE	TRUE	482,730	21.09
Phosphatidylcholine (14:0_18:1) levels	Parkinson's disease	rs6531968	G	T	G	T	-7.86E-02	-1.32E-02	5.44E-01	5.92E-01	FALSE	FALSE	4	88199484	1.87E-02	3.17E-01	TRUE	4	87278332	1.70E-02	1.00	7064	3.79E-06	TRUE	TRUE	482,730	21.40
Phosphatidylcholine (14:0_18:1) levels	Parkinson's disease	rs7484827	G	A	G	A	9.03E-02	3.10E-03	2.41E-01	7.04E-01	FALSE	FALSE	12	48123563	2.02E-02	5.64E-02	TRUE	12	47729780	1.96E-02	1.00	7064	4.21E-06	TRUE	TRUE	482,730	21.20
Phosphatidylcholine (14:0_18:1) levels	Parkinson's disease	rs78414457	T	G	T	G	1.55E-01	-5.40E-02	7.44E-02	9.39E-01	FALSE	FALSE	13	37955257	5.01E-02	5.52E-01	TRUE	13	37381120	3.28E-02	0.96	7064	2.43E-06	TRUE	TRUE	482,730	22.25
Phosphatidylcholine (14:0_18:2) levels	Parkinson's disease	rs116105185	C	T	C	T	2.22E-01	4.14E-02	3.04E-02	9.63E-01	FALSE	FALSE	1	63866459	5.60E-02	3.38E-01	TRUE	1	63400788	5.03E-02	0.96	6866	9.97E-06	TRUE	TRUE	482,730	19.54
Phosphatidylcholine (14:0_18:2) levels	Parkinson's disease	rs11853492	T	C	T	C	-8.34E-02	-2.00E-04	6.60E-01	3.78E-01	FALSE	FALSE	15	97682752	2.44E-02	2.70E-03	TRUE	15	97139522	1.86E-02	0.93	6866	7.13E-06	TRUE	TRUE	482,730	20.19
Phosphatidylcholine (14:0_18:2) levels	Parkinson's disease	rs12113381	T	C	T	C	1.23E-01	1.69E-02	1.20E-01	8.49E-01	FALSE	FALSE	7	1143127	3.12E-02	2.30E-01	TRUE	7	1103491	2.64E-02	1.00	6866	3.24E-06	TRUE	TRUE	482,730	21.70
Phosphatidylcholine (14:0_18:2) levels	Parkinson's disease	rs12137781	A	G	A	G	-1.70E-01	-2.68E-02	5.90E-02	9.19E-01	FALSE	FALSE	1	79824075	4.40E-02	2.66E-01	TRUE	1	79358390	3.68E-02	0.96	6866	3.83E-06	TRUE	TRUE	482,730	21.38
Phosphatidylcholine (14:0_18:2) levels	Parkinson's disease	rs1262295	G	A	G	A	1.17E-01	2.39E-02	8.64E-01	1.30E-01	FALSE	FALSE	1	201377438	2.97E-02	3.75E-01	TRUE	1	201408310	2.53E-02	0.98	6866	3.86E-06	TRUE	TRUE	482,730	21.37
Phosphatidylcholine (14:0_18:2) levels	Parkinson's disease	rs13418209	T	G	T	G	7.99E-02	1.06E-02	3.64E-01	6.92E-01	FALSE	FALSE	2	164727379	1.88E-02	2.42E-01	TRUE	2	163870869	1.78E-02	0.99	6866	7.65E-06	TRUE	TRUE	482,730	20.05

Phosphatidylcholine (14:0_18:2) levels	Parkinson's disease	rs144154975	A	C	A	C	2.48E-01	-2.38E-01	2.54E-02	9.87E-01	FALSE	FALSE	13	66324148	1.03E-01	1.02E-02	TRUE	13	65750016	5.50E-02	0.98	6866	6.56E-06	TRUE	TRUE	482,730	20.35
Phosphatidylcholine (14:0_18:2) levels	Parkinson's disease	rs174548	G	C	G	C	1.84E-01	2.00E-03	3.84E-01	3.00E-01	TRUE	FALSE	11	61571348	1.83E-02	3.90E-02	TRUE	11	61803876	1.75E-02	1.00	6866	1.15E-25	TRUE	TRUE	482,730	110.58
Phosphatidylcholine (14:0_18:2) levels	Parkinson's disease	rs4665972	C	T	C	T	-1.15E-01	-7.12E-02	6.20E-01	4.20E-01	FALSE	FALSE	2	27598097	1.79E-02	3.48E-05	TRUE	2	27375230	1.75E-02	1.00	6866	6.24E-11	TRUE	TRUE	482,730	42.88
Phosphatidylcholine (14:0_18:2) levels	Parkinson's disease	rs4683767	C	G	C	G	-9.70E-02	-5.60E-03	1.94E-01	2.40E-01	TRUE	FALSE	3	139593906	2.24E-02	9.45E-02	TRUE	3	139875064	2.15E-02	1.00	6866	6.52E-06	TRUE	TRUE	482,730	20.36
Phosphatidylcholine (14:0_18:2) levels	Parkinson's disease	rs4952487	T	A	T	A	-7.90E-02	6.60E-03	3.66E-01	2.51E-01	TRUE	FALSE	2	41759578	2.47E-02	1.02E-01	TRUE	2	41532438	1.77E-02	1.00	6866	8.30E-06	TRUE	TRUE	482,730	19.90
Phosphatidylcholine (14:0_18:2) levels	Parkinson's disease	rs55771840	T	C	T	C	1.10E-01	-5.60E-03	1.40E-01	8.77E-01	FALSE	FALSE	17	77451373	3.79E-02	5.47E-02	TRUE	17	79455291	2.47E-02	0.98	6866	8.48E-06	TRUE	TRUE	482,730	19.86
Phosphatidylcholine (14:0_18:2) levels	Parkinson's disease	rs74920877	A	G	A	G	2.20E-01	4.11E-02	3.06E-02	9.75E-01	FALSE	FALSE	6	8749268	7.29E-02	2.42E-01	TRUE	6	8749035	4.94E-02	0.98	6866	8.64E-06	TRUE	TRUE	482,730	19.82
Phosphatidylcholine (14:0_18:2) levels	Parkinson's disease	rs78880935	C	T	C	T	2.77E-01	7.56E-02	2.03E-02	9.79E-01	FALSE	FALSE	3	68329070	8.57E-02	4.23E-01	TRUE	3	68279920	6.26E-02	0.93	6866	9.75E-06	TRUE	TRUE	482,730	19.59
Phosphatidylcholine (14:0_18:2) levels	Parkinson's disease	rs8015793	C	G	C	G	8.76E-02	-2.28E-02	2.74E-01	2.89E-01	TRUE	FALSE	14	95937661	2.65E-02	4.09E-01	TRUE	14	95471324	1.97E-02	0.93	6866	8.53E-06	TRUE	TRUE	482,730	19.84
Phosphatidylcholine (14:0_18:2) levels	Parkinson's disease	rs950668	A	G	A	G	-8.12E-02	-1.36E-02	4.83E-01	5.26E-01	FALSE	FALSE	15	71921839	2.90E-02	1.94E-01	TRUE	15	71629500	1.77E-02	0.91	6866	4.50E-06	TRUE	TRUE	482,730	21.07
Phosphatidylcholine (14:0_18:2) levels	Parkinson's disease	rs9663572	G	C	G	C	-1.25E-01	-6.99E-02	1.10E-01	1.83E-01	TRUE	FALSE	10	42411056	4.65E-02	8.77E-01	TRUE	10	41829153	2.80E-02	0.96	6866	8.13E-06	TRUE	TRUE	482,730	19.94
Phosphatidylcholine (15:0_18:1) levels	Parkinson's disease	rs1044043	C	A	C	A	-9.80E-02	-3.78E-02	8.08E-01	2.08E-01	FALSE	FALSE	6	32793981	2.53E-02	8.70E-01	TRUE	6	32826204	2.20E-02	1.00	6468	8.88E-06	TRUE	TRUE	482,730	19.77
Phosphatidylcholine (15:0_18:1) levels	Parkinson's disease	rs10917404	T	C	T	C	-9.80E-02	3.42E-02	2.27E-01	7.59E-01	FALSE	FALSE	1	23965829	2.22E-02	9.10E-01	TRUE	1	23639339	2.11E-02	1.00	6468	3.32E-06	TRUE	TRUE	482,730	21.66
Phosphatidylcholine (15:0_18:1) levels	Parkinson's disease	rs11075022	C	T	C	T	8.57E-02	-2.38E-02	5.70E-01	3.55E-01	FALSE	FALSE	16	11921263	1.84E-02	7.07E-01	TRUE	16	11827406	1.78E-02	1.00	6468	1.60E-06	TRUE	TRUE	482,730	23.07
Phosphatidylcholine (15:0_18:1) levels	Parkinson's disease	rs117207178	T	C	T	C	1.34E-01	-2.70E-02	9.72E-02	8.93E-01	FALSE	FALSE	12	34633590	4.07E-02	2.96E-01	TRUE	12	34480655	3.01E-02	0.97	6468	8.39E-06	TRUE	TRUE	482,730	19.88
Phosphatidylcholine (15:0_18:1) levels	Parkinson's disease	rs1260326	C	T	C	T	-9.74E-02	-6.78E-02	6.51E-01	4.20E-01	FALSE	FALSE	2	27730940	1.72E-02	4.04E-05	TRUE	2	27508073	1.84E-02	1.00	6468	1.22E-07	TRUE	TRUE	482,730	28.04
Phosphatidylcholine (15:0_18:1) levels	Parkinson's disease	rs12926159	A	G	A	G	-4.31E-01	-9.54E-02	1.28E-02	9.82E-01	FALSE	FALSE	16	88801500	7.80E-02	6.54E-01	TRUE	16	88735092	7.96E-02	0.96	6468	6.10E-08	TRUE	TRUE	482,730	29.40
Phosphatidylcholine (15:0_18:1) levels	Parkinson's disease	rs13161129	A	G	A	G	-1.26E-01	0.00E+00	1.14E-01	8.55E-01	FALSE	FALSE	5	132830294	3.27E-02	1.30E-04	TRUE	5	133494602	2.80E-02	0.96	6468	6.63E-06	TRUE	TRUE	482,730	20.32
Phosphatidylcholine (15:0_18:1) levels	Parkinson's disease	rs138445434	A	G	A	G	-2.47E-01	-1.09E-01	2.65E-02	9.85E-01	FALSE	FALSE	16	19436938	9.39E-02	6.10E-01	TRUE	16	19425616	5.52E-02	0.97	6468	7.74E-06	TRUE	TRUE	482,730	20.04
Phosphatidylcholine (15:0_18:1) levels	Parkinson's disease	rs142477396	A	G	A	G	-1.07E-01	2.91E-02	1.75E-01	8.03E-01	FALSE	FALSE	6	21056443	2.79E-02	5.26E-01	TRUE	6	21056212	2.31E-02	0.99	6468	3.77E-06	TRUE	TRUE	482,730	21.41
Phosphatidylcholine (15:0_18:1) levels	Parkinson's disease	rs41441450	T	C	T	C	-4.08E-01	8.84E-02	9.82E-03	9.87E-01	FALSE	FALSE	5	86013053	1.02E-01	4.15E-01	TRUE	5	86717236	8.94E-02	0.99	6468	5.08E-06	TRUE	TRUE	482,730	20.84
Phosphatidylcholine (15:0_18:1) levels	Parkinson's disease	rs4238010	A	G	A	G	-1.15E-01	2.10E-02	8.66E-01	1.20E-01	FALSE	FALSE	12	4118317	2.69E-02	3.61E-01	TRUE	12	4009151	2.59E-02	1.00	6468	9.44E-06	TRUE	TRUE	482,730	19.65
Phosphatidylcholine (15:0_18:1) levels	Parkinson's disease	rs581080	C	G	C	G	1.17E-01	3.05E-02	8.56E-01	8.10E-01	TRUE	FALSE	9	15305378	2.15E-02	8.05E-01	TRUE	9	15305380	2.49E-02	1.00	6468	2.75E-06	TRUE	TRUE	482,730	22.02
Phosphatidylcholine (15:0_18:1) levels	Parkinson's disease	rs6866162	C	T	C	T	1.34E-01	2.18E-02	1.27E-01	8.72E-01	FALSE	FALSE	5	173755653	3.62E-02	2.62E-01	TRUE	5	174328650	2.77E-02	0.92	6468	1.34E-06	TRUE	TRUE	482,730	23.41
Phosphatidylcholine (15:0_18:1) levels	Parkinson's disease	rs72815248	T	C	T	C	-1.30E-01	-2.83E-02	1.22E-01	9.16E-01	FALSE	FALSE	16	54493872	4.22E-02	2.99E-01	TRUE	16	54459960	2.71E-02	0.96	6468	1.66E-06	TRUE	TRUE	482,730	23.00
Phosphatidylcholine (15:0_18:1) levels	Parkinson's disease	rs75922719	C	T	C	T	4.76E-01	-2.51E-01	6.84E-03	9.89E-01	FALSE	FALSE	15	94449395	1.66E-01	8.79E-01	TRUE	15	93906166	1.07E-01	0.95	6468	8.01E-06	TRUE	TRUE	482,730	19.97
Phosphatidylcholine (15:0_18:1) levels	Parkinson's disease	rs77392374	C	T	C	T	-2.52E-01	8.91E-02	2.68E-02	9.73E-01	FALSE	FALSE	10	73046421	7.81E-02	5.96E-01	TRUE	10	71286664	5.67E-02	0.93	6468	9.28E-06	TRUE	TRUE	482,730	19.68
Phosphatidylcholine (15:0_18:2) levels	Parkinson's disease	rs10468017	T	C	T	C	1.12E-01	2.75E-02	3.38E-01	7.16E-01	FALSE	FALSE	15	58678512	1.87E-02	8.48E-01	TRUE	15	58386313	1.81E-02	1.00	6639	7.41E-10	TRUE	TRUE	482,730	38.02
Phosphatidylcholine (15:0_18:2) levels	Parkinson's disease	rs11174583	C	T	C	T	-1.21E-01	1.87E-02	1.13E-01	8.15E-01	FALSE	FALSE	12	63051064	2.88E-02	2.86E-01	TRUE	12	62657284	2.73E-02	0.99	6639	9.96E-06	TRUE	TRUE	482,730	19.55
Phosphatidylcholine (15:0_18:2) levels	Parkinson's disease	rs114498622	C	A	C	A	3.47E-01	1.03E-01	1.56E-02	9.85E-01	FALSE	FALSE	2	214562803	1.45E-01	3.23E-01	TRUE	2	213698079	7.55E-02	0.88	6639	4.32E-06	TRUE	TRUE	482,730	21.15
Phosphatidylcholine (15:0_18:2) levels	Parkinson's disease	rs115852609	A	G	A	G	2.55E-01	3.73E-02	2.35E-02	9.75E-01	FALSE	FALSE	2	157145460	7.37E-02	2.13E-01	TRUE	2	156288948	5.73E-02	0.99	6639	8.90E-06	TRUE	TRUE	482,730	19.76
Phosphatidylcholine (15:0_18:2) levels	Parkinson's disease	rs1168036	G	A	G	A	1.07E-01	-3.10E-03	7.38E-01	3.28E-01	FALSE	FALSE	1	62962734	1.80E-02	6.38E-02	TRUE	1	62497063	1.96E-02	1.00	6639	4.78E-08	TRUE	TRUE	482,730	29.87
Phosphatidylcholine (15:0_18:2) levels	Parkinson's disease	rs11722429	A	G	A	G	1.32E-01	4.32E-02	9.89E-02	9.47E-01	FALSE	FALSE	4	137205253	5.46E-02	3.68E-01	TRUE	4	136284098	2.92E-02	0.98	6639	5.94E-06	TRUE	TRUE	482,730	20.54
Phosphatidylcholine (15:0_18:2) levels	Parkinson's disease	rs12113214	A	T	A	T	9.31E-02	1.07E-02	3.23E-01	2.81E-01	TRUE	FALSE	7	6569774	2.29E-02	1.93E-01	TRUE	7	6530143	1.88E-02	0.95	6639	7.34E-07	TRUE	TRUE	482,730	24.57
Phosphatidylcholine (15:0_18:2) levels	Parkinson's disease	rs12938196	T	C	T	C	1.07E-01	7.50E-03	1.59E-01	8.42E-01	FALSE	FALSE	17	2683430	3.90E-02	7.18E-02	TRUE	17	2780136	2.41E-02	0.96	6639	9.69E-06	TRUE	TRUE	482,730	19.60
Phosphatidylcholine (15:0_18:2) levels	Parkinson's disease	rs13063467	A	C	A	C	2.12E-01	-7.91E-02	3.50E-02	9.64E-01	FALSE	FALSE	3	11207775	6.04E-02	7.21E-01	TRUE	3	11166089	4.77E-02	1.00	6639	9.06E-06	TRUE	TRUE	482,730	19.73
Phosphatidylcholine (15:0_18:2) levels	Parkinson's disease	rs13101957	A	C	A	C	-1.22E-01	-9.70E-03	1.30E-01	8.24E-01	FALSE	FALSE	4	170199286	2.99E-02	1.27E-01	TRUE	4	169278135	2.60E-02	0.98	6639	2.89E-06	TRUE	TRUE	482,730	21.92
Phosphatidylcholine (15:0_18:2) levels	Parkinson's disease	rs13281002	A	G	A	G	-2.62E-01	-1.19E-01	2.68E-02	9.70E-01	FALSE	FALSE	8	66573447	6.80E-02	4.07E-02	TRUE	8	65661212	5.33E-02	0.96	6639	9.59E-07	TRUE	TRUE	482,730	24.05
Phosphatidylcholine (15:0_18:2) levels	Parkinson's disease	rs139075653	C	T	C	T	6.46E-01	5.04E-01	4.42E-03	9.94E-01	FALSE	FALSE	6	114530533	2.23E-01	1.20E-02	TRUE	6	114209369	1.38E-01	0.94	6639	3.04E-06	TRUE	TRUE	482,730	21.83
Phosphatidylcholine (15:0_18:2) levels	Parkinson's disease	rs1488201	G	T	G	T	8.47E-02	-2.14E-02	3.50E-01	6.94E-01	FALSE	FALSE	3	26889880	2.43E-02	4.21E-01	TRUE	3	26848389	1.83E-02	0.99	6639	3.60E-06	TRUE	TRUE	482,730	21.50
Phosphatidylcholine (15:0_18:2) levels	Parkinson's disease	rs16968712	C	T	C	T	-1.32E-01	-9.20E-03	1.19E-01	8.09E-01	FALSE	FALSE	13	107127784	2.99E-02	1.20E-01	TRUE	13	106475436	2.81E-02	0.91	6639	2.63E-06	TRUE	TRUE	482,730	22.11
Phosphatidylcholine (15:0_18:2) levels	Parkinson's disease	rs17231506	T	C	T	C	1.43E-01	1.36E-02	2.78E-01	6.87E-01	FALSE	FALSE	16	56994528	1.81E-02	3.45E-01	TRUE	16	56960616	1.92E-02	1.00	6639	1.20E-13	TRUE	TRUE	482,730	55.23
Phosphatidylcholine (15:0_18:2) levels	Parkinson's disease	rs174566	G	A	G	A	3.17E-01	-5.70E-03	4.08E-01	6.64E-01	FALSE	FALSE	11	61592362	1.78E-02	1.24E-01	TRUE	11	61824890	1.73E-02	1.00	6639	1.46E-73	TRUE	TRUE	482,730	337.47
Phosphatidylcholine (15:0_18:2) levels	Parkinson's disease	rs17614174	C	T	C	T	-1.87E-01	4.55E-02	5.24E-02	8.96E-01	FALSE	FALSE	5	16862419	3.89E-02	6.16E-01	TRUE	5	16862310	4.05E-02	0.92	6639	3.75E-06	TRUE	TRUE	482,730	21.42
Phosphatidylcholine (15:0_18:2) levels	Parkinson's disease	rs184289628	G	T	G	T	2.99E-01	1.15E-01	1.82E-02	9.90E-01	FALSE	FALSE	6	139906689	1.23E-01	4.58E-01	TRUE	6	139585552	6.45E-02	0.97	6639	3.67E-06	TRUE	TRUE	482,730	21.46
Phosphatidylcholine (15:0_18:2) levels	Parkinson's disease	rs1911704	C	G	C	G	-8.35E-02	1.20E-02	3.49E-01	2.50E-01	TRUE	FALSE	2	41698973	2.49E-02	2.00E-01	TRUE	2	41471833	1.82E-02	1.00	6639	4.65E-06	TRUE	TRUE	482,730	21.01
Phosphatidylcholine (15:0_18:2) levels	Parkinson's disease	rs325406	T	C	T	C	9.75E-02	8.00E-03	7.91E-01	2.73E-01	FALSE	FALSE	15	100247740	2.15E-02	1.49E-01	TRUE	15	99707535	2.14E-02	0.99	6639	5.19E-06	TRUE	TRUE	482,730	20.80
Phosphatidylcholine (15:0_18:2) levels	Parkinson's disease	rs35960238	G	A	G	A	-1.31E-01	5.76E-02	1.27E-01	8.94E-01	FALSE	FALSE	10	26620556	3.66E-02	9.37E-01	TRUE	10	26331627	2.61E-02	0.99	6639	5.37E-07	TRUE	TRUE	482,730	25.17
Phosphatidylcholine (15:0_18:2) levels	Parkinson's disease	rs4335452	A	G	A	G	-1.03E-01	1.95E-02	8.00E-01	2.54E-01	FALSE	FALSE	10	5914799	2.61E-02	3.42E-01	TRUE	10	5872836	2.24E-02	0.95	6639	3.88E-06	TRUE	TRUE	482,730	21.36
Phosphatidylcholine (15:0_18:2) levels	Parkinson's disease	rs4382917	A	G	A	G	-1.01E-01	-6.22E-02	2.56E-01	7.72E-01	FALSE	FALSE	11	62221476	3.28E-02	2.90E-02	TRUE	11	62454004	2.06E-02	0.92	6639	9.84E-07	TRUE	TRUE	482,730	24.00
Phosphatidylcholine (15:0_18:2) levels	Parkinson's disease	rs551412	T	A	T	A	8.84E-02	9.70E-03	2.86E-01	2.69E-01	TRUE	FALSE	9	135326175	2.18E-02	1.82E-01	TRUE	9	132450788	1.93E-02	0.99	6639	4.64E-06	TRUE	TRUE	482,730	21.01
Phosphatidylcholine (15:0_18:2) levels	Parkinson's disease	rs55727637	T	C	T	C	1.11E-01	1.10E-02	2.99E-01	6.91E-01	FALSE	FALSE	16	15145252	1.83E-02	2.62E-01	TRUE	16	15051395	1.90E-02	1.00	6639	5.22E-09	TRUE	TRUE	482,730	34.19
Phosphatidylcholine (15:0_18:2) levels	Parkinson's disease	rs56144603	A	G	A	G	2.47E-01	1.73E-02	3.15E-02	9.35E-01	FALSE	FALSE	6	73930603	3.65E-02	1.97E-01	TRUE	6	73220880	5.13E-02	0.99	6639	1.42E-06	TRUE	TRUE	482,730	23.29
Phosphatidylcholine (15:0_18:2) levels	Parkinson's disease	rs60071820	T	C	T	C	-7.88E-02	1.46E-02	4.26E-01	5.86E-01	FALSE	FALSE	16	14442789	2.16E-02	3.01E-01	TRUE	16	14348932	1.77E-02	0.99	6639	8.51E-06	TRUE	TRUE	482,730	19.85
Phosphatidylcholine (15:0_18:2) levels	Parkinson's disease	rs633695	G	A	G	A	9.30E-02	2.32E-02	3.14E-01	6.47E-01	FALSE	FALSE	15	58725839	2.19E-02	5.39E-01	TRUE	15	58433640	1.89E-02	1.00	6639	9.28E-07	TRUE	TRUE	482,730	24.12
Phosphatidylcholine (15:0_18:2) levels	Parkinson's disease	rs639956	G	A	G	A	-7.79E-02	4.38E-02	5.61E-01	3.74E-01	FALSE	FALSE	11	79351151	2.31E-02	2.90E-02	TRUE	11	79640107	1.74E-02	1.00	6639	8.06E-06	TRUE	TRUE	482,730	19.95
Phosphatidylcholine (15:0_18:2) levels	Parkinson's disease	rs6558177	G	A	G	A	-8.05E-02	-3.41E-02	6.22E-01	3.81E-01	FALSE	FALSE	8	22558515	2.25E-02	8.86E-01	TRUE	8	22701002	1.80E-02	0.99	6639	8.36E-06	TRUE	TRUE	482,730	19.88
Phosphatidylcholine (15:0_18:2) levels	Parkinson's disease	rs75097472	A	G	A	G	-3.83E-01	-2.58E-02	1.15E-02	9.69E-01	FALSE	FALSE	12	130927677	6.91E-02	1.49E-01	TRUE	12	130443132	8.35E-02	0.88	6639	4.65E-06	TRUE	TRUE	482,730	21.01
Phosphatidylcholine (15:0_18:2) levels	Parkinson's disease	rs76916318	C	T	C	T	2.93E-01	1.19E-01	1.84E-02	9.87E-01	FALSE	FALSE	10	44846111	9.95E-02	6.32E-01	TRUE	10	44350663	6.46E-02	0.99	6639	5.76E-06	TRUE	TRUE	482,730	20.60
Phosphatidylcholine (15:0_18:2) levels	Parkinson's disease	rs77404354	T	C	T	C	-4.78E-01	7.39E-02	8.45E-03	9.77E-01	FALSE	FALSE	8	65553596	8.57E-02	4.10E-01	TRUE	8	64641039	1.00E-01	0.90	6639	1.84E-06	TRUE	TRUE	482,730	22.79
Phosphatidylcholine (15:0_18:2) levels	Parkinson's disease	rs7920171	A	G	A	G	9.46E-02	-4.70E-03	2.08E-01	7.54E-01	FALSE	FALSE	10	64854103	2.27E-02	7.79E-02	TRUE	10	63094343	2.13E-02	1.00	6639	8.96E-06	TRUE	TRUE	482,730	19.75
Phosphatidylcholine (15:0_18:2) levels	Parkinson's disease	rs964184	C	G	C	G	-1.41E-01	-5.68E-02	8.49E-01	8.63E-01	TRUE	FALSE	11	116648917	2.43E-02	9.71E-03	TRUE	11	116778201	2.41E-02	1.00	6639	5.34E-09	TRUE	TRUE	482,730	34.15
Phosphatidylcholine (16:0_16:0) levels	Parkinson's disease	rs11591147	T	G	T	G	-2.34E-01	-3.77E-02	3.32E-02	9.82E-01	FALSE	FALSE	1	55505647	6.83E-02	2.36E-01	TRUE	1	55039974	4.66E-02	1.00	7174	4.94E-07	TRUE	TRUE	482,730	25.33
Phosphatidylcholine (16:0_16:0) levels	Parkinson's disease	rs1187583	A	G	A	G	-1.42E-01	6.30E-03	9.16E-01	8.79E-02	FALSE	FALSE	14	55036863	3.38E-02	6.96E-02	TRUE	14	54570145	3.00E-02	1.00	7174	2.41E-06	TRUE	TRUE	482,730	22.28
Phosphatidylcholine (16:0_16:0) levels	Parkinson's disease	rs12522374	A	G	A	G	8.12E-02	1.17E-02	3.47E-01	6.99E-01	FALSE	FALSE	5	158664704	2.09E-02	2.40E-01	TRUE	5	159237696	1.74E-02	1.00	7174	3.11E-06	TRUE	TRUE	482,730	21.78
Phosphatidylcholine (16:0_16:0) levels	Parkinson's disease	rs1367117	A	G	A	G	8.38E-02	6.00E-03	2.85E-01	6.89E-01	FALSE	FALSE	2	21263900	1.81E-02	1.31E-01	TRUE	2	21041028	1.84E-02	1.00	7174	5.33E-06	TRUE	TRUE	482,730	20.75
Phosphatidylcholine (16:0_16:0) levels	Parkinson's disease	rs148444916	C	T	C	T	2.89E-01	1.92E-01	2.08E-02	9.90E-01	FALSE	FALSE	1	93435853	1.55E-01	6.68E-01	TRUE	1	92970296	5.76E-02	0.99	7174	5.67E-07	TRUE	TRUE	482,730	25.07
Phosphatidylcholine (16:0_16:0) levels	Parkinson's disease	rs17231506	T	C	T	C	1.42E-01	1.36E-02	2.78E-01	6.87E-01	FALSE	FALSE	16	56994528	1.81E-02	3.45E-01	TRUE	16	56960616	1.85E-02	1.00	7174	1.54E-14	TRUE	TRUE	482,730	59.28
Phosphatidylcholine (16:0_16:0) levels	Parkinson's disease	rs174546	T	C	T	C	-1.49E-01	-2.90E-03	4.07E-01	6.72E-01	FALSE	FALSE	11	61569830	1.79E-02	5.91E-02	TRUE	11	61802358	1.69E-02	1.00	7174	1.69E-18	TRUE	TRUE	482,730	77.44
Phosphatidylcholine (16:0_16:0) levels	Parkinson's disease	rs2236514	G	C	G	C	-8.39E-02	2.80E-03	6.47E-01	6.35E-01	TRUE	FALSE	9	139572068	2.00E-02	5.10E-02	TRUE	9	136677616	1.77E-02	0.95	7174	2.32E-06	TRUE	TRUE	482,730	22.35
Phosphatidylcholine (16:0_16:0) levels	Parkinson's disease	rs261290	C	T	C	T	-1.39E-01	-1.69E-02	6.12E-01	3.36E-01	FALSE	FALSE	15	58678720	1.80E-02	4.58E-01	TRUE	15	58386521	1.69E-02	1.00	7174	2.37E-16	TRUE	TRUE	482,730	67.59
Phosphatidylcholine (16:0_16:0) levels	Parkinson's disease	rs3134950	A	C	A	C	9.67E-02	-3.04E-02	5.83E-01	3.15E-01	FALSE	FALSE	6	32127477	2.17E-02	7.93E-01	TRUE	6	32159700	1.68E-02	1.00	7174	9.96E-09	TRUE	TRUE	482,730	32.93
Phosphatidylcholine (16:0_16:0) levels	Parkinson's disease	rs3807226	G	A	G	A	-8.25E-02	4.40E-03	2.76E-01	7.01E-01	FALSE	FALSE	7	154471268	2.50E-02	6.48E-02	TRUE	7	154679558	1.86E-02	0.98	7174	9.57E-06	TRUE	TRUE	482,730	19.62
Phosphatidylcholine (16:0_16:0) levels	Parkinson's disease	rs3936021	G	A	G	A	-1.07E-01	-3.90E-03	1.58E-01	8.79E-01	FALSE	FALSE	5	84394905	4.21E-02	3.30E-02	TRUE	5	85099087	2.34E-02	0.97	7174	4.96E-06	TRUE	TRUE	482,730	20.88
Phosphatidylcholine (16:0_16:0) levels	Parkinson's disease	rs4744927	C	A	C	A	9.07E-01	2.19E-01	9.98E-01	8.30E-03	FALSE	FALSE	9	72284718	2.03E-01	5.54E-01	TRUE	9	69669802	1.92E-01	0.86	7174	2.35E-06	TRUE	TRUE	482,730	22.33
Phosphatidylcholine (16:0_16:0) levels	Parkinson's disease	rs5022521	T	C	T	C	-9.74E-02	1.02E-02	7.97E-01	1.41E-01	FALSE	FALSE	4	111128587	4.02E-02	9.69E-02	TRUE	4	110207431	2.12E-02	0.94	7174	4.26E-06	TRUE	TRUE	482,730	21.17
Phosphatidylcholine (16:0_16:0) levels	Parkinson's disease	rs56270325	T	A	T	A	9.06E-02	9.80E-03	2.59E-01	2.33E-01	TRUE	FALSE	1	5959301	2.64E-02	1.48E-01	TRUE	1	5899241	1.88E-02	1.00	7174	1.49E-06	TRUE	TRUE	482,730	23.20
Phosphatidylcholine (16:0_16:0) levels	Parkinson's disease	rs633695	G	A	G	A	9.11E-02	2.32E-02	3.14E-01	6.47E-01	FALSE	FALSE	15	58725839	2.19E-02	5.39E-01	TRUE	15	58433640	1.81E-02	1.00	7174	5.23E-07	TRUE	TRUE	482,730	25.22
Phosphatidylcholine (16:0_16:0) levels	Parkinson's disease	rs643531	A	C	A	C	1.25E-01	-3.13E-02	8.90E-01	1.37E-01	FALSE	FALSE	9	15296034	2.48E-02	6.85E-01	TRUE	9	15296036	2.61E-02	1.00	7174	1.91E-06	TRUE	TRUE	482,730	22.72
Phosphatidylcholine (16:0_16:0) levels	Parkinson's disease	rs72733996	G	A	G	A	1.01E-01	2.46E-02	1.98E-01	7.52E-01	FALSE	FALSE	9	83551699	2.59E-02	4.66E-01	TRUE	9	80936784	2.08E-02	1.00	7174	1.23E-06	TRUE	TRUE	482,730	23.57
Phosphatidylcholine (16:0_16:0) levels	Parkinson's disease	rs72974647	G	A	G	A	1.44E-01	4.59E-02	8.22E-02	9.34E-01	FALSE	FALSE	6	123648480	3.92E-02	6.18E-01	TRUE	6	123327335	3.00E-02	1.00	7174	1.61E-06	TRUE	TRUE	482,730	23.05
Phosphatidylcholine (16:0_16:0) levels	Parkinson's disease	rs75023439	C	T	C	T	-1.73E-01	1.55E-02	4.84E-02	9.36E-01	FALSE	FALSE	1	158475551	3.76E-02	1.68E-01	TRUE	1	158505761	3.88E-02	0.99	7174	8.49E-06	TRUE	TRUE	482,730	19.85
Phosphatidylcholine (16:0_16:0) levels	Parkinson's disease	rs7780296	G	C	G	C	8.41E-02	-7.30E-03	7.19E-01	6.43E-01	TRUE	FALSE	7	17714261	2.33E-02	1.22E-01	TRUE	7	17674637	1.85E-02	0.99	7174	5.57E-06	TRUE	TRUE	482,730	20.66
Phosphatidylcholine (16:0_16:1) levels	Parkinson's disease	rs10760286	C	T	C	T	7.89E-02	2.43E-02	5.10E-01	4.54E-01	FALSE	FALSE	9	126181588	2.11E-02	6.04E-01	TRUE	9	123419309	1.70E-02	0.97	7172	3.32E-06	TRUE	TRUE	482,730	21.66
Phosphatidylcholine (16:0_16:1) levels	Parkinson's disease	rs113366516	T	G	T	G	3.08E-01	-9.44E-02	1.70E-02	9.91E-01	FALSE	FALSE	6	138469237	1.90E-01	2.08E-01	TRUE	6	138148100	6.61E-02	0.93	7172	3.14E-06	TRUE	TRUE	482,730	21.76
Phosphatidylcholine (16:0_16:1) levels	Parkinson's disease	rs116681793	A	G	A	G	-1.85E-01	-1.02E-02	4.53E-02	9.80E-01	FALSE	FALSE	4	181148799	8.67E-02	4.28E-02	TRUE	4	180227646	4.10E-02	0.96	7172	7.01E-06	TRUE	TRUE	482,730	20.22
Phosphatidylcholine (16:0_16:1) levels	Parkinson's disease	rs117571757	A	G	A	G	-3.34E-01	-6.85E-02	1.35E-02	9.89E-01	FALSE	FALSE	17	16361760	1.36E-01	2.12E-01	TRUE	17	16458446	7.28E-02	0.97	7172	4.44E-06	TRUE	TRUE	482,730	21.10
Phosphatidylcholine (16:0_16:1) levels	Parkinson's disease	rs1260326	C	T	C	T	-1.08E-01	-6.78E-02	6.51E-01	4.20E-01	FALSE	FALSE	2	27730940	1.72E-02	4.04E-05	TRUE	2	27508073	1.75E-02	1.00	7172	6.12E-10	TRUE	TRUE	482,730	38.38
Phosphatidylcholine (16:0_16:1) levels	Parkinson's disease	rs1854434	G	A	G	A	-1.10E-01	1.70E-03	1.54E-01	8.51E-01	FALSE	FALSE	9	32796760	3.11E-02	1.99E-02	TRUE	9	32796762	2.32E-02	1.00	7172	2.15E-06	TRUE	TRUE	482,730	22.50
Phosphatidylcholine (16:0_16:1) levels	Parkinson's disease	rs2597486	G	A	G	A	7.58E-02	-3.40E-02	5.54E-01	4.40E-01	FALSE	FALSE	4	163374951	2.27E-02	8.75E-01	TRUE	4	162453799	1.70E-02	0.99	7172	8.12E-06	TRUE	TRUE	482,730	19.94
Phosphatidylcholine (16:0_16:1) levels	Parkinson's disease	rs28524821	C	A	C	A	1.09E-01	1.67E-02	1.46E-01	8.73E-01	FALSE	FALSE	4	17070309	3.34E-02	2.10E-01	TRUE	4	17068686	2.35E-02	1.00	7172	3.98E-06	TRUE	TRUE	482,730	21.31
Phosphatidylcholine (16:0_16:1) levels	Parkinson's disease	rs34832413	G	A	G	A	-1.19E-01	-5.45E-02	1.12E-01	9.26E-01	FALSE	FALSE	16	71344111	3.53E-02	9.11E-01	TRUE	16	71310208	2.64E-02	0.99	7172	6.08E-06	TRUE	TRUE	482,730	20.49
Phosphatidylcholine (16:0_16:1) levels	Parkinson's disease	rs35235479	G	A	G	A	-9.62E-02	4.90E-03	2.14E-01	8.15E-01	FALSE	FALSE	10	94527160	2.53E-02	7.27E-02	TRUE	10	92767403	2.03E-02	1.00	7172	2.24E-06	TRUE	TRUE	482,730	22.42
Phosphatidylcholine (16:0_16:1) levels	Parkinson's disease	rs4629629	T	C	T	C	8.83E-02	5.43E-02	7.65E-01	1.45E-01	FALSE	FALSE	5	85591932	3.39E-02	9.61E-01	TRUE	5	86296114	1.97E-02	1.00	7172	7.25E-06	TRUE	TRUE	482,730	20.16
Phosphatidylcholine (16:0_16:1) levels	Parkinson's disease	rs62015468	C	G	C	G	1.94E-01	-6.20E-03	4.19E-02	1.01E-01	TRUE	FALSE	16	6588605	4.07E-02	5.59E-02	TRUE	16	6538604	4.30E-02	0.92	7172	6.47E-06	TRUE	TRUE	482,730	20.37
Phosphatidylcholine (16:0_16:1) levels	Parkinson's disease	rs77007318	C	G	C	G	-2.41E-01	2.65E-02	2.89E-02	2.25E-02	TRUE	FALSE	16	59890172	8.92E-02	1.16E-01	TRUE	16	59856268	5.22E-02	0.89	7172	3.88E-06	TRUE	TRUE	482,730	21.36
Phosphatidylcholine (16:0_16:1) levels	Parkinson's disease	rs80227620	T	C	T	C	1.19E-01	-3.70E-03	1.44E-01	8.51E-01	FALSE	FALSE	2	168689139	4.02E-02	3.26E-02	TRUE	2	167832629	2.44E-02	0.95	7172	1.19E-06	TRUE	TRUE	482,730	23.63
Phosphatidylcholine (16:0_17:1) levels	Parkinson's disease	rs10500613	G	A	G	A	-2.47E-01	1.00E-01	2.43E-02	9.80E-01	FALSE	FALSE	11	4708984	6.84E-02	8.48E-01	TRUE	11	4687754	5.52E-02	0.98	7076	7.81E-06	TRUE	TRUE	482,730	20.01
Phosphatidylcholine (16:0_17:1) levels	Parkinson's disease	rs10783517	T	C	T	C	-9.24E-02	5.70E-03	2.13E-01	8.48E-01	FALSE	FALSE	12	52916606	2.58E-02	8.27E-02	TRUE	12	52522822	2.07E-02	0.99	7076	7.89E-06	TRUE	TRUE	482,730	20.00
Phosphatidylcholine (16:0_17:1) levels	Parkinson's disease	rs116911263	T	C	T	C	-3.52E-01	-1.32E-02	1.36E-02	9.87E-01	FALSE	FALSE	10	97701007	9.68E-02	4.99E-02	TRUE	10	95941250	7.29E-02	0.99	7076	1.47E-06	TRUE	TRUE	482,730	23.23
Phosphatidylcholine (16:0_17:1) levels	Parkinson's disease	rs117085626	A	G	A	G	1.20E-01	2.00E-03	1.13E-01	8.73E-01	FALSE	FALSE	11	1628948	2.68E-02	2.65E-02	TRUE	11	1607718	2.66E-02	1.00	7076	6.77E-06	TRUE	TRUE	482,730	20.29
Phosphatidylcholine (16:0_17:1) levels	Parkinson's disease	rs12475699	C	T	C	T	8.11E-02	-3.11E-02	6.63E-01	4.24E-01	FALSE	FALSE	2	30245047	2.27E-02	7.70E-01	TRUE	2	30022181	1.77E-02	1.00	7076	4.86E-06	TRUE	TRUE	482,730	20.93
Phosphatidylcholine (16:0_17:1) levels	Parkinson's disease	rs12602611	A	G	A	G	9.94E-02	-2.14E-02	1.73E-01	7.83E-01	FALSE	FALSE	17	11240422	2.78E-02	3.56E-01	TRUE	17	11337105	2.23E-02	0.98	7076	8.10E-06	TRUE	TRUE	482,730	19.94
Phosphatidylcholine (16:0_17:1) levels	Parkinson's disease	rs12749808	T	C	T	C	-8.36E-02	-1.33E-02	3.56E-01	6.50E-01	FALSE	FALSE	1	158065762	1.95E-02	3.05E-01	TRUE	1	158095972	1.78E-02	0.96	7076	2.75E-06	TRUE	TRUE	482,730	22.01
Phosphatidylcholine (16:0_17:1) levels	Parkinson's disease	rs138107736	G	T	G	T	-3.43E-01	1.69E-01	1.32E-02	9.88E-01	FALSE	FALSE	1	62180355	1.58E-01	5.45E-01	TRUE	1	61714683	7.70E-02	0.93	7076	8.58E-06	TRUE	TRUE	482,730	19.83
Phosphatidylcholine (16:0_17:1) levels	Parkinson's disease	rs2122922	T	C	T	C	-9.77E-02	4.75E-02	2.02E-01	7.22E-01	FALSE	FALSE	8	102062658	3.03E-02	9.32E-01	TRUE	8	101050430	2.13E-02	0.95	7076	4.61E-06	TRUE	TRUE	482,730	21.03
Phosphatidylcholine (16:0_17:1) levels	Parkinson's disease	rs2317091	A	T	A	T	1.91E-01	-3.11E-02	4.03E-02	3.74E-02	TRUE	FALSE	11	62960760	4.96E-02	2.75E-01	TRUE	11	63193288	4.32E-02	0.99	7076	9.83E-06	TRUE	TRUE	482,730	19.57
Phosphatidylcholine (16:0_17:1) levels	Parkinson's disease	rs41488845	A	G	A	G	-4.96E-01	-5.59E-02	7.22E-03	9.87E-01	FALSE	FALSE	17	36536223	1.19E-01	1.95E-01	TRUE	17	38380397	1.04E-01	0.91	7076	2.04E-06	TRUE	TRUE	482,730	22.59
Phosphatidylcholine (16:0_17:1) levels	Parkinson's disease	rs7177289	T	C	T	C	8.22E-02	1.76E-02	3.93E-01	6.60E-01	FALSE	FALSE	15	58680184	1.78E-02	4.91E-01	TRUE	15	58387985	1.71E-02	1.00	7076	1.53E-06	TRUE	TRUE	482,730	23.15
Phosphatidylcholine (16:0_17:1) levels	Parkinson's disease	rs78395462	T	G	T	G	2.58E-01	-2.10E-03	2.38E-02	9.85E-01	FALSE	FALSE	4	123562167	9.38E-02	7.67E-03	TRUE	4	122641012	5.52E-02	0.98	7076	3.17E-06	TRUE	TRUE	482,730	21.74
Phosphatidylcholine (16:0_17:1) levels	Parkinson's disease	rs78418403	T	G	T	G	1.57E-01	5.17E-02	6.22E-02	9.48E-01	FALSE	FALSE	15	23997177	5.33E-02	4.78E-01	TRUE	15	23752030	3.48E-02	0.96	7076	6.23E-06	TRUE	TRUE	482,730	20.45
Phosphatidylcholine (16:0_17:1) levels	Parkinson's disease	rs80201535	A	G	A	G	-2.20E-01	-2.03E-01	3.08E-02	9.85E-01	FALSE	FALSE	7	10865777	1.01E-01	2.21E-02	TRUE	7	10826150	4.92E-02	0.97	7076	7.45E-06	TRUE	TRUE	482,730	20.11
Phosphatidylcholine (16:0_17:1) levels	Parkinson's disease	rs818276	T	C	T	C	7.40E-02	-1.30E-02	4.76E-01	3.94E-01	FALSE	FALSE	6	79047537	2.29E-02	2.44E-01	TRUE	6	78337820	1.66E-02	1.00	7076	8.73E-06	TRUE	TRUE	482,730	19.80
Phosphatidylcholine (16:0_18:0) levels	Parkinson's disease	rs10468017	T	C	T	C	8.77E-02	2.75E-02	3.38E-01	7.16E-01	FALSE	FALSE	15	58678512	1.87E-02	8.48E-01	TRUE	15	58386313	1.76E-02	1.00	7154	6.14E-07	TRUE	TRUE	482,730	24.91
Phosphatidylcholine (16:0_18:0) levels	Parkinson's disease	rs10738267	G	C	G	C	1.04E-01	6.28E-02	8.37E-01	8.74E-01	TRUE	FALSE	9	12222686	3.40E-02	3.24E-02	TRUE	9	12222686	2.28E-02	0.99	7154	5.61E-06	TRUE	TRUE	482,730	20.65
Phosphatidylcholine (16:0_18:0) levels	Parkinson's disease	rs10890773	A	G	A	G	-1.17E-01	5.70E-02	8.60E-01	1.45E-01	FALSE	FALSE	11	107760002	3.20E-02	3.74E-02	TRUE	11	107889276	2.39E-02	1.00	7154	1.02E-06	TRUE	TRUE	482,730	23.93

Phosphatidylcholine (16:0_18:0) levels	Parkinson's disease	rs113053792	G	A	G	A	-3.56E-01	9.88E-02	1.20E-02	9.85E-01	FALSE	FALSE	5	62411919	1.03E-01	4.74E-01	TRUE	5	63116092	7.86E-02	0.93	7154	5.97E-06	TRUE	TRUE	482,730	20.53
Phosphatidylcholine (16:0_18:0) levels	Parkinson's disease	rs113076792	A	G	A	G	-3.38E-01	4.53E-02	1.36E-02	9.63E-01	FALSE	FALSE	7	152868079	6.12E-02	3.38E-01	TRUE	7	153170994	7.38E-02	0.95	7154	4.84E-06	TRUE	TRUE	482,730	20.93
Phosphatidylcholine (16:0_18:0) levels	Parkinson's disease	rs1168036	G	A	G	A	8.99E-02	-3.10E-03	7.38E-01	3.28E-01	FALSE	FALSE	1	62962734	1.80E-02	6.38E-02	TRUE	1	62497063	1.89E-02	1.00	7154	1.99E-06	TRUE	TRUE	482,730	22.64
Phosphatidylcholine (16:0_18:0) levels	Parkinson's disease	rs11723712	A	G	A	G	1.03E-01	9.50E-03	8.06E-01	2.52E-01	FALSE	FALSE	4	170225896	2.56E-02	1.49E-01	TRUE	4	169304745	2.12E-02	1.00	7154	1.11E-06	TRUE	TRUE	482,730	23.77
Phosphatidylcholine (16:0_18:0) levels	Parkinson's disease	rs117495065	G	C	G	C	3.02E-01	-1.61E-01	1.75E-02	1.66E-02	TRUE	FALSE	6	147496047	1.07E-01	8.81E-01	TRUE	6	147174911	6.54E-02	0.91	7154	4.02E-06	TRUE	TRUE	482,730	21.29
Phosphatidylcholine (16:0_18:0) levels	Parkinson's disease	rs118179558	C	T	C	T	-7.04E-01	7.78E-02	2.99E-03	9.88E-01	FALSE	FALSE	11	58247131	1.21E-01	2.83E-01	TRUE	11	58479658	1.58E-01	0.92	7154	8.26E-06	TRUE	TRUE	482,730	19.90
Phosphatidylcholine (16:0_18:0) levels	Parkinson's disease	rs11870991	A	C	A	C	1.29E-01	-2.65E-02	9.22E-02	8.66E-01	FALSE	FALSE	17	42836124	2.81E-02	4.61E-01	TRUE	17	44758756	2.88E-02	1.00	7154	8.31E-06	TRUE	TRUE	482,730	19.89
Phosphatidylcholine (16:0_18:0) levels	Parkinson's disease	rs11903018	C	T	C	T	-9.80E-02	-3.88E-02	1.81E-01	7.77E-01	FALSE	FALSE	2	37593243	2.29E-02	4.51E-02	TRUE	2	37366100	2.19E-02	0.99	7154	7.88E-06	TRUE	TRUE	482,730	19.99
Phosphatidylcholine (16:0_18:0) levels	Parkinson's disease	rs12052409	C	A	C	A	-1.93E-01	-2.05E-02	5.16E-02	9.42E-01	FALSE	FALSE	2	68301690	4.14E-02	2.07E-01	TRUE	2	68074558	3.86E-02	0.98	7154	5.57E-07	TRUE	TRUE	482,730	25.10
Phosphatidylcholine (16:0_18:0) levels	Parkinson's disease	rs13119269	T	C	T	C	1.00E-01	-2.80E-03	1.67E-01	8.12E-01	FALSE	FALSE	4	189443141	2.87E-02	3.55E-02	TRUE	4	188521987	2.26E-02	0.99	7154	9.47E-06	TRUE	TRUE	482,730	19.64
Phosphatidylcholine (16:0_18:0) levels	Parkinson's disease	rs139644986	G	C	G	C	5.49E-01	6.58E-02	5.13E-03	1.44E-02	TRUE	FALSE	6	18721330	1.38E-01	1.99E-01	TRUE	6	18721099	1.24E-01	0.94	7154	9.93E-06	TRUE	TRUE	482,730	19.55
Phosphatidylcholine (16:0_18:0) levels	Parkinson's disease	rs141165989	T	C	T	C	2.03E-01	1.25E-01	3.69E-02	9.80E-01	FALSE	FALSE	6	150504716	8.81E-02	8.08E-01	TRUE	6	150183580	4.47E-02	0.98	7154	5.59E-06	TRUE	TRUE	482,730	20.65
Phosphatidylcholine (16:0_18:0) levels	Parkinson's disease	rs143975147	T	C	T	C	2.20E-01	5.04E-02	3.02E-02	9.81E-01	FALSE	FALSE	12	4888580	8.72E-02	2.49E-01	TRUE	12	4779414	4.92E-02	0.98	7154	8.09E-06	TRUE	TRUE	482,730	19.94
Phosphatidylcholine (16:0_18:0) levels	Parkinson's disease	rs174601	T	C	T	C	1.46E-01	-3.70E-03	4.19E-01	6.38E-01	FALSE	FALSE	11	61623140	1.79E-02	7.75E-02	TRUE	11	61855668	1.69E-02	1.00	7154	6.00E-18	TRUE	TRUE	482,730	74.91
Phosphatidylcholine (16:0_18:0) levels	Parkinson's disease	rs2126263	A	G	A	G	1.11E-01	-6.82E-02	8.50E-01	9.57E-02	FALSE	FALSE	8	9181611	3.04E-02	1.24E-02	TRUE	8	9324101	2.34E-02	1.00	7154	2.30E-06	TRUE	TRUE	482,730	22.36
Phosphatidylcholine (16:0_18:0) levels	Parkinson's disease	rs2214660	A	G	A	G	-1.02E-01	5.63E-02	1.85E-01	8.63E-01	FALSE	FALSE	7	122509701	3.26E-02	4.21E-02	TRUE	7	122869647	2.17E-02	0.99	7154	2.56E-06	TRUE	TRUE	482,730	22.16
Phosphatidylcholine (16:0_18:0) levels	Parkinson's disease	rs2274855	A	G	A	G	-1.03E-01	-1.29E-02	1.71E-01	8.35E-01	FALSE	FALSE	9	135105944	3.32E-02	1.57E-01	TRUE	9	132230557	2.32E-02	0.89	7154	9.51E-06	TRUE	TRUE	482,730	19.63
Phosphatidylcholine (16:0_18:0) levels	Parkinson's disease	rs2391389	G	A	G	A	-7.69E-02	4.60E-03	3.79E-01	5.37E-01	FALSE	FALSE	1	95485679	2.25E-02	7.59E-02	TRUE	1	95020123	1.70E-02	1.00	7154	5.98E-06	TRUE	TRUE	482,730	20.52
Phosphatidylcholine (16:0_18:0) levels	Parkinson's disease	rs247617	A	C	A	C	9.19E-02	1.47E-02	2.78E-01	6.86E-01	FALSE	FALSE	16	56990716	1.80E-02	3.80E-01	TRUE	16	56956804	1.86E-02	1.00	7154	7.77E-07	TRUE	TRUE	482,730	24.46
Phosphatidylcholine (16:0_18:0) levels	Parkinson's disease	rs2724782	A	G	A	G	-2.15E-01	2.06E-02	3.36E-02	9.10E-01	FALSE	FALSE	10	12421923	4.08E-02	2.12E-01	TRUE	10	12379924	4.84E-02	0.94	7154	8.99E-06	TRUE	TRUE	482,730	19.74
Phosphatidylcholine (16:0_18:0) levels	Parkinson's disease	rs2854275	A	C	A	C	-1.23E-01	-8.70E-03	1.05E-01	8.73E-01	FALSE	FALSE	6	32628428	3.41E-02	9.71E-02	TRUE	6	32660651	2.71E-02	0.99	7154	6.23E-06	TRUE	TRUE	482,730	20.45
Phosphatidylcholine (16:0_18:0) levels	Parkinson's disease	rs3924	A	G	A	G	-7.61E-02	3.00E-02	6.04E-01	3.66E-01	FALSE	FALSE	5	150410135	2.06E-02	8.41E-01	TRUE	5	151030574	1.72E-02	1.00	7154	9.52E-06	TRUE	TRUE	482,730	19.63
Phosphatidylcholine (16:0_18:0) levels	Parkinson's disease	rs4659619	C	T	C	T	8.49E-02	1.44E-02	3.38E-01	6.75E-01	FALSE	FALSE	1	236198987	1.97E-02	3.34E-01	TRUE	1	236035687	1.75E-02	1.00	7154	1.21E-06	TRUE	TRUE	482,730	23.61
Phosphatidylcholine (16:0_18:0) levels	Parkinson's disease	rs62330208	C	G	C	G	-1.03E-01	8.90E-03	2.40E-01	1.82E-01	TRUE	FALSE	5	983244	3.81E-02	8.86E-02	TRUE	5	983129	2.04E-02	0.91	7154	4.69E-07	TRUE	TRUE	482,730	25.43
Phosphatidylcholine (16:0_18:0) levels	Parkinson's disease	rs633695	G	A	G	A	8.12E-02	2.32E-02	3.14E-01	6.47E-01	FALSE	FALSE	15	58725839	2.19E-02	5.39E-01	TRUE	15	58433640	1.82E-02	1.00	7154	8.31E-06	TRUE	TRUE	482,730	19.89
Phosphatidylcholine (16:0_18:0) levels	Parkinson's disease	rs7046115	G	C	G	C	-7.99E-02	1.84E-02	3.21E-01	3.87E-01	TRUE	FALSE	9	96892605	2.31E-02	3.70E-01	TRUE	9	94130323	1.81E-02	0.98	7154	9.93E-06	TRUE	TRUE	482,730	19.55
Phosphatidylcholine (16:0_18:0) levels	Parkinson's disease	rs72723967	A	C	A	C	1.11E-01	9.20E-03	1.63E-01	8.16E-01	FALSE	FALSE	4	138733515	2.85E-02	1.26E-01	TRUE	4	137812361	2.27E-02	0.99	7154	1.18E-06	TRUE	TRUE	482,730	23.65
Phosphatidylcholine (16:0_18:0) levels	Parkinson's disease	rs72974647	G	A	G	A	1.43E-01	4.59E-02	8.22E-02	9.34E-01	FALSE	FALSE	6	123648480	3.92E-02	6.18E-01	TRUE	6	123327335	3.01E-02	1.00	7154	1.91E-06	TRUE	TRUE	482,730	22.71
Phosphatidylcholine (16:0_18:0) levels	Parkinson's disease	rs76413658	C	G	C	G	-1.12E-01	9.00E-03	1.33E-01	7.11E-02	TRUE	FALSE	16	6639737	4.43E-02	7.61E-02	TRUE	16	6589736	2.44E-02	0.99	7154	4.73E-06	TRUE	TRUE	482,730	20.98
Phosphatidylcholine (16:0_18:0) levels	Parkinson's disease	rs76779498	T	C	T	C	3.26E-01	-7.10E-03	1.35E-02	9.84E-01	FALSE	FALSE	7	17965086	9.35E-02	2.71E-02	TRUE	7	17925463	7.30E-02	0.96	7154	8.13E-06	TRUE	TRUE	482,730	19.94
Phosphatidylcholine (16:0_18:1) levels	Parkinson's disease	rs10779836	T	C	T	C	9.03E-02	-4.40E-03	7.77E-01	1.96E-01	FALSE	FALSE	1	230303150	2.40E-02	6.80E-02	TRUE	1	230167404	1.99E-02	1.00	7174	6.13E-06	TRUE	TRUE	482,730	20.48
Phosphatidylcholine (16:0_18:1) levels	Parkinson's disease	rs114925849	A	C	A	C	5.77E-01	-7.54E-02	4.62E-03	9.83E-01	FALSE	FALSE	5	108554590	9.57E-02	3.66E-01	TRUE	5	109218889	1.27E-01	0.94	7174	5.74E-06	TRUE	TRUE	482,730	20.60
Phosphatidylcholine (16:0_18:1) levels	Parkinson's disease	rs116997061	T	C	T	C	-1.70E-01	8.14E-02	6.19E-02	9.50E-01	FALSE	FALSE	12	20022049	5.65E-02	8.25E-01	TRUE	12	19869115	3.60E-02	0.91	7174	2.35E-06	TRUE	TRUE	482,730	22.32
Phosphatidylcholine (16:0_18:1) levels	Parkinson's disease	rs1260326	C	T	C	T	-1.01E-01	-6.78E-02	6.51E-01	4.20E-01	FALSE	FALSE	2	27730940	1.72E-02	4.04E-05	TRUE	2	27508073	1.75E-02	1.00	7174	6.89E-09	TRUE	TRUE	482,730	33.64
Phosphatidylcholine (16:0_18:1) levels	Parkinson's disease	rs12650992	T	C	T	C	8.91E-02	2.03E-02	2.34E-01	7.78E-01	FALSE	FALSE	4	124336379	2.66E-02	3.52E-01	TRUE	4	123415224	1.96E-02	1.00	7174	5.70E-06	TRUE	TRUE	482,730	20.61
Phosphatidylcholine (16:0_18:1) levels	Parkinson's disease	rs1541361	T	C	T	C	-7.65E-02	3.00E-03	4.30E-01	5.79E-01	FALSE	FALSE	12	47499471	2.25E-02	4.85E-02	TRUE	12	47105688	1.68E-02	1.00	7174	5.63E-06	TRUE	TRUE	482,730	20.64
Phosphatidylcholine (16:0_18:1) levels	Parkinson's disease	rs1593135	G	C	G	C	-1.19E-01	6.39E-02	1.12E-01	7.17E-02	TRUE	FALSE	16	71293362	3.69E-02	4.17E-02	TRUE	16	71259459	2.63E-02	1.00	7174	6.52E-06	TRUE	TRUE	482,730	20.36
Phosphatidylcholine (16:0_18:1) levels	Parkinson's disease	rs247617	A	C	A	C	9.75E-02	1.47E-02	2.78E-01	6.86E-01	FALSE	FALSE	16	56990716	1.80E-02	3.80E-01	TRUE	16	56956804	1.86E-02	1.00	7174	1.56E-07	TRUE	TRUE	482,730	27.57
Phosphatidylcholine (16:0_18:1) levels	Parkinson's disease	rs28807772	G	A	G	A	9.52E-02	-2.01E-02	2.02E-01	7.80E-01	FALSE	FALSE	5	165249121	2.24E-02	4.31E-01	TRUE	5	165822116	2.08E-02	1.00	7174	4.72E-06	TRUE	TRUE	482,730	20.98
Phosphatidylcholine (16:0_18:1) levels	Parkinson's disease	rs3134937	T	C	T	C	-1.01E-01	-3.09E-02	2.00E-01	7.15E-01	FALSE	FALSE	6	32206539	2.18E-02	8.05E-01	TRUE	6	32238762	2.08E-02	0.99	7174	1.17E-06	TRUE	TRUE	482,730	23.66
Phosphatidylcholine (16:0_18:1) levels	Parkinson's disease	rs4538911	C	G	C	G	-9.05E-02	1.26E-02	7.03E-01	6.41E-01	TRUE	FALSE	8	6086360	2.36E-02	2.27E-01	TRUE	8	6228839	1.84E-02	0.99	7174	8.54E-07	TRUE	TRUE	482,730	24.27
Phosphatidylcholine (16:0_18:1) levels	Parkinson's disease	rs633695	G	A	G	A	9.03E-02	2.32E-02	3.14E-01	6.47E-01	FALSE	FALSE	15	58725839	2.19E-02	5.39E-01	TRUE	15	58433640	1.82E-02	1.00	7174	6.82E-07	TRUE	TRUE	482,730	24.71
Phosphatidylcholine (16:0_18:1) levels	Parkinson's disease	rs7004301	G	A	G	A	7.72E-02	1.13E-02	6.19E-01	4.62E-01	FALSE	FALSE	8	130238780	2.22E-02	2.15E-01	TRUE	8	129226534	1.73E-02	1.00	7174	7.95E-06	TRUE	TRUE	482,730	19.98
Phosphatidylcholine (16:0_18:1) levels	Parkinson's disease	rs76546858	T	C	T	C	-1.23E-01	-1.77E-02	1.31E-01	9.15E-01	FALSE	FALSE	5	180497075	6.04E-02	1.14E-01	TRUE	5	181070075	2.72E-02	0.83	7174	7.80E-06	TRUE	TRUE	482,730	20.01
Phosphatidylcholine (16:0_18:1) levels	Parkinson's disease	rs77027238	A	G	A	G	1.85E-01	1.22E-02	4.96E-02	9.75E-01	FALSE	FALSE	17	41926542	6.82E-02	6.65E-02	TRUE	17	43849174	3.85E-02	0.97	7174	1.54E-06	TRUE	TRUE	482,730	23.14
Phosphatidylcholine (16:0_18:1) levels	Parkinson's disease	rs7912885	T	G	T	G	-8.16E-02	1.32E-02	6.99E-01	2.84E-01	FALSE	FALSE	10	129336988	2.49E-02	2.24E-01	TRUE	10	127538724	1.84E-02	0.98	7174	9.28E-06	TRUE	TRUE	482,730	19.68
Phosphatidylcholine (16:0_18:2) levels	Parkinson's disease	rs10787711	A	G	A	G	-9.00E-02	1.81E-02	6.48E-01	4.18E-01	FALSE	FALSE	10	118406784	2.05E-02	4.26E-01	TRUE	10	116647273	1.75E-02	0.99	7174	2.74E-07	TRUE	TRUE	482,730	26.47
Phosphatidylcholine (16:0_18:2) levels	Parkinson's disease	rs10890773	A	G	A	G	-1.26E-01	5.70E-02	8.60E-01	1.45E-01	FALSE	FALSE	11	107760002	3.20E-02	3.74E-02	TRUE	11	107889276	2.38E-02	1.00	7174	1.42E-07	TRUE	TRUE	482,730	27.75
Phosphatidylcholine (16:0_18:2) levels	Parkinson's disease	rs10961143	G	A	G	A	1.95E-01	3.91E-02	4.83E-02	9.58E-01	FALSE	FALSE	9	13564219	5.63E-02	3.12E-01	TRUE	9	13564220	3.88E-02	0.99	7174	4.88E-07	TRUE	TRUE	482,730	25.36
Phosphatidylcholine (16:0_18:2) levels	Parkinson's disease	rs112569946	A	G	A	G	7.16E-01	6.32E-02	3.20E-03	9.87E-01	FALSE	FALSE	6	125234555	1.09E-01	2.50E-01	TRUE	6	124913409	1.56E-01	0.89	7174	4.34E-06	TRUE	TRUE	482,730	21.14
Phosphatidylcholine (16:0_18:2) levels	Parkinson's disease	rs11722429	A	G	A	G	1.33E-01	4.32E-02	9.89E-02	9.47E-01	FALSE	FALSE	4	137205253	5.46E-02	3.68E-01	TRUE	4	136284098	2.81E-02	0.98	7174	2.18E-06	TRUE	TRUE	482,730	22.47
Phosphatidylcholine (16:0_18:2) levels	Parkinson's disease	rs13150924	A	G	A	G	-1.35E-01	-1.06E-02	1.30E-01	8.24E-01	FALSE	FALSE	4	170198810	2.99E-02	1.41E-01	TRUE	4	169277659	2.50E-02	0.98	7174	7.20E-08	TRUE	TRUE	482,730	29.07
Phosphatidylcholine (16:0_18:2) levels	Parkinson's disease	rs13402814	T	A	T	A	1.64E-01	9.00E-04	5.97E-02	7.48E-02	TRUE	FALSE	2	137944011	3.30E-02	9.22E-03	TRUE	2	137186441	3.51E-02	1.00	7174	2.89E-06	TRUE	TRUE	482,730	21.92
Phosphatidylcholine (16:0_18:2) levels	Parkinson's disease	rs143066473	A	G	A	G	2.54E-01	-2.94E-01	2.49E-02	9.86E-01	FALSE	FALSE	12	99825953	2.37E-01	6.67E-01	TRUE	12	99432175	5.52E-02	0.94	7174	4.29E-06	TRUE	TRUE	482,730	21.16
Phosphatidylcholine (16:0_18:2) levels	Parkinson's disease	rs1488201	G	T	G	T	7.81E-02	-2.14E-02	3.50E-01	6.94E-01	FALSE	FALSE	3	26889880	2.43E-02	4.21E-01	TRUE	3	26848389	1.76E-02	0.99	7174	8.86E-06	TRUE	TRUE	482,730	19.77
Phosphatidylcholine (16:0_18:2) levels	Parkinson's disease	rs150376922	A	G	A	G	-2.90E-01	4.52E-02	1.80E-02	9.85E-01	FALSE	FALSE	12	29855844	1.02E-01	1.82E-01	TRUE	12	29702911	6.44E-02	0.96	7174	6.88E-06	TRUE	TRUE	482,730	20.25
Phosphatidylcholine (16:0_18:2) levels	Parkinson's disease	rs17126640	A	G	A	G	-9.94E-02	3.37E-02	2.03E-01	8.80E-01	FALSE	FALSE	12	52704991	2.81E-02	6.37E-01	TRUE	12	52311207	2.08E-02	1.00	7174	1.89E-06	TRUE	TRUE	482,730	22.74
Phosphatidylcholine (16:0_18:2) levels	Parkinson's disease	rs17231506	T	C	T	C	1.44E-01	1.36E-02	2.78E-01	6.87E-01	FALSE	FALSE	16	56994528	1.81E-02	3.45E-01	TRUE	16	56960616	1.85E-02	1.00	7174	8.35E-15	TRUE	TRUE	482,730	60.50
Phosphatidylcholine (16:0_18:2) levels	Parkinson's disease	rs174574	C	A	C	A	-3.31E-01	6.60E-03	5.91E-01	3.39E-01	FALSE	FALSE	11	61600342	1.77E-02	1.48E-01	TRUE	11	61832870	1.66E-02	1.00	7174	5.66E-86	TRUE	TRUE	482,730	396.75
Phosphatidylcholine (16:0_18:2) levels	Parkinson's disease	rs1800588	T	C	T	C	1.38E-01	-3.30E-03	2.58E-01	7.75E-01	FALSE	FALSE	15	58723675	2.12E-02	5.73E-02	TRUE	15	58431476	1.93E-02	0.99	7174	1.09E-12	TRUE	TRUE	482,730	50.85
Phosphatidylcholine (16:0_18:2) levels	Parkinson's disease	rs187681244	T	C	T	C	-4.39E-01	-4.99E-02	7.93E-03	9.92E-01	FALSE	FALSE	1	16575712	2.22E-01	8.49E-02	TRUE	1	16249217	9.75E-02	0.91	7174	7.01E-06	TRUE	TRUE	482,730	20.22
Phosphatidylcholine (16:0_18:2) levels	Parkinson's disease	rs2629180	A	G	A	G	-6.95E-01	5.64E-02	3.87E-03	9.80E-01	FALSE	FALSE	3	3988888	9.49E-02	2.58E-01	TRUE	3	3947204	1.48E-01	0.83	7174	2.56E-06	TRUE	TRUE	482,730	22.15
Phosphatidylcholine (16:0_18:2) levels	Parkinson's disease	rs4412689	A	G	A	G	-9.32E-02	5.70E-02	2.00E-01	7.68E-01	FALSE	FALSE	10	59165924	2.65E-02	1.57E-02	TRUE	10	57406164	2.08E-02	1.00	7174	7.42E-06	TRUE	TRUE	482,730	20.11
Phosphatidylcholine (16:0_18:2) levels	Parkinson's disease	rs4415100	A	T	A	T	9.33E-02	1.87E-02	2.11E-01	2.41E-01	TRUE	FALSE	5	165243607	2.20E-02	4.01E-01	TRUE	5	165816602	2.05E-02	1.00	7174	5.24E-06	TRUE	TRUE	482,730	20.78
Phosphatidylcholine (16:0_18:2) levels	Parkinson's disease	rs56075928	A	G	A	G	2.45E-01	1.10E-01	2.57E-02	9.90E-01	FALSE	FALSE	11	62903374	1.18E-01	4.55E-01	TRUE	11	63135902	5.29E-02	0.99	7174	3.85E-06	TRUE	TRUE	482,730	21.37
Phosphatidylcholine (16:0_18:2) levels	Parkinson's disease	rs62039480	A	G	A	G	1.16E-01	1.69E-02	3.32E-01	6.66E-01	FALSE	FALSE	16	15137450	1.83E-02	4.52E-01	TRUE	16	15043593	1.77E-02	1.00	7174	6.52E-11	TRUE	TRUE	482,730	42.79
Phosphatidylcholine (16:0_18:2) levels	Parkinson's disease	rs62330208	C	G	C	G	-8.99E-02	8.90E-03	2.40E-01	1.82E-01	TRUE	FALSE	5	983244	3.81E-02	8.86E-02	TRUE	5	983129	2.03E-02	0.91	7174	9.97E-06	TRUE	TRUE	482,730	19.54
Phosphatidylcholine (16:0_18:2) levels	Parkinson's disease	rs6578729	A	G	A	G	-1.39E-01	2.36E-02	7.91E-02	8.66E-01	FALSE	FALSE	11	6089740	2.76E-02	4.06E-01	TRUE	11	6068510	3.09E-02	1.00	7174	7.32E-06	TRUE	TRUE	482,730	20.14
Phosphatidylcholine (16:0_18:2) levels	Parkinson's disease	rs6673582	A	T	A	T	-8.05E-02	8.60E-03	3.09E-01	3.05E-01	TRUE	FALSE	1	189880489	2.43E-02	1.40E-01	TRUE	1	189911359	1.79E-02	1.00	7174	7.04E-06	TRUE	TRUE	482,730	20.21
Phosphatidylcholine (16:0_18:2) levels	Parkinson's disease	rs73205054	C	A	C	A	1.96E-01	-7.83E-02	3.79E-02	9.70E-01	FALSE	FALSE	12	110504994	5.76E-02	7.60E-01	TRUE	12	110067189	4.33E-02	0.99	7174	6.17E-06	TRUE	TRUE	482,730	20.46
Phosphatidylcholine (16:0_18:2) levels	Parkinson's disease	rs75833961	C	T	C	T	2.72E-01	1.58E-01	2.02E-02	9.80E-01	FALSE	FALSE	1	33911068	8.56E-02	3.24E-02	TRUE	1	33445467	6.11E-02	0.94	7174	8.43E-06	TRUE	TRUE	482,730	19.87
Phosphatidylcholine (16:0_18:2) levels	Parkinson's disease	rs7717591	G	A	G	A	-8.09E-02	-1.38E-02	6.79E-01	3.91E-01	FALSE	FALSE	5	51711739	2.28E-02	2.62E-01	TRUE	5	52415905	1.79E-02	1.00	7174	6.26E-06	TRUE	TRUE	482,730	20.44
Phosphatidylcholine (16:0_18:2) levels	Parkinson's disease	rs78882831	T	C	T	C	2.47E-01	2.05E-01	2.51E-02	9.81E-01	FALSE	FALSE	5	95464280	9.18E-02	1.27E-02	TRUE	5	96128576	5.46E-02	0.97	7174	6.35E-06	TRUE	TRUE	482,730	20.41
Phosphatidylcholine (16:0_18:2) levels	Parkinson's disease	rs7936002	G	A	G	A	-9.93E-02	-5.27E-02	2.57E-01	7.70E-01	FALSE	FALSE	11	62219029	3.25E-02	9.78E-01	TRUE	11	62451557	1.96E-02	0.93	7174	4.82E-07	TRUE	TRUE	482,730	25.61
Phosphatidylcholine (16:0_18:2) levels	Parkinson's disease	rs9436221	C	T	C	T	-1.27E-01	3.60E-03	2.61E-01	6.80E-01	FALSE	FALSE	1	62903359	1.88E-02	7.09E-02	TRUE	1	62437688	1.88E-02	1.00	7174	1.82E-11	TRUE	TRUE	482,730	45.30
Phosphatidylcholine (16:0_18:2) levels	Parkinson's disease	rs964184	C	G	C	G	-1.58E-01	-5.68E-02	8.49E-01	8.63E-01	TRUE	FALSE	11	116648917	2.43E-02	9.71E-03	TRUE	11	116778201	2.32E-02	1.00	7174	9.56E-12	TRUE	TRUE	482,730	46.57
Phosphatidylcholine (16:0_18:3) levels	Parkinson's disease	rs114797690	T	C	T	C	2.63E-01	2.25E-02	2.34E-02	9.58E-01	FALSE	FALSE	1	99063537	6.22E-02	1.44E-01	TRUE	1	98597981	5.89E-02	0.87	7174	7.71E-06	TRUE	TRUE	482,730	20.04
Phosphatidylcholine (16:0_18:3) levels	Parkinson's disease	rs115079643	C	T	C	T	-2.82E-01	-6.61E-02	2.36E-02	9.72E-01	FALSE	FALSE	5	176351036	8.53E-02	3.58E-01	TRUE	5	176924035	5.60E-02	0.95	7174	4.60E-07	TRUE	TRUE	482,730	25.47
Phosphatidylcholine (16:0_18:3) levels	Parkinson's disease	rs12255873	G	A	G	A	-1.39E-01	2.78E-02	9.01E-01	1.03E-01	FALSE	FALSE	10	62594554	3.94E-02	3.19E-01	TRUE	10	60834796	2.84E-02	0.97	7174	9.11E-07	TRUE	TRUE	482,730	24.15
Phosphatidylcholine (16:0_18:3) levels	Parkinson's disease	rs1260326	C	T	C	T	-1.15E-01	-6.78E-02	6.51E-01	4.20E-01	FALSE	FALSE	2	27730940	1.72E-02	4.04E-05	TRUE	2	27508073	1.74E-02	1.00	7174	4.02E-11	TRUE	TRUE	482,730	43.74
Phosphatidylcholine (16:0_18:3) levels	Parkinson's disease	rs1474236	C	T	C	T	-1.12E-01	-1.48E-02	8.18E-01	1.68E-01	FALSE	FALSE	3	116831501	3.90E-02	1.52E-01	TRUE	3	117112654	2.28E-02	0.90	7174	8.21E-07	TRUE	TRUE	482,730	24.35
Phosphatidylcholine (16:0_18:3) levels	Parkinson's disease	rs147507697	G	A	G	A	2.09E-01	-3.39E-02	3.61E-02	9.83E-01	FALSE	FALSE	3	76690340	8.76E-02	1.56E-01	TRUE	3	76641189	4.51E-02	0.99	7174	3.46E-06	TRUE	TRUE	482,730	21.57
Phosphatidylcholine (16:0_18:3) levels	Parkinson's disease	rs174598	A	G	A	G	1.12E-01	-1.46E-02	3.46E-01	6.90E-01	FALSE	FALSE	11	61621194	1.87E-02	3.61E-01	TRUE	11	61853722	1.76E-02	1.00	7174	2.24E-10	TRUE	TRUE	482,730	40.36
Phosphatidylcholine (16:0_18:3) levels	Parkinson's disease	rs1800588	T	C	T	C	8.94E-02	-3.30E-03	2.58E-01	7.75E-01	FALSE	FALSE	15	58723675	2.12E-02	5.73E-02	TRUE	15	58431476	1.94E-02	1.00	7174	3.95E-06	TRUE	TRUE	482,730	21.32
Phosphatidylcholine (16:0_18:3) levels	Parkinson's disease	rs192646452	T	A	T	A	-2.55E-01	-4.89E-02	2.34E-02	2.40E-02	TRUE	FALSE	17	26081122	7.90E-02	2.71E-01	TRUE	17	27754096	5.55E-02	0.96	7174	4.52E-06	TRUE	TRUE	482,730	21.06
Phosphatidylcholine (16:0_18:3) levels	Parkinson's disease	rs247617	A	C	A	C	1.07E-01	1.47E-02	2.78E-01	6.86E-01	FALSE	FALSE	16	56990716	1.80E-02	3.80E-01	TRUE	16	56956804	1.85E-02	1.00	7174	9.57E-09	TRUE	TRUE	482,730	33.01
Phosphatidylcholine (16:0_18:3) levels	Parkinson's disease	rs3125786	A	G	A	G	-1.14E-01	-1.68E-02	8.69E-01	4.69E-02	FALSE	FALSE	9	140629958	6.33E-02	1.02E-01	TRUE	9	137735506	2.58E-02	0.93	7174	9.39E-06	TRUE	TRUE	482,730	19.66
Phosphatidylcholine (16:0_18:3) levels	Parkinson's disease	rs345182	G	A	G	A	9.77E-02	3.54E-02	1.89E-01	7.98E-01	FALSE	FALSE	17	58240764	2.29E-02	9.10E-01	TRUE	17	60163403	2.18E-02	0.94	7174	7.79E-06	TRUE	TRUE	482,730	20.02
Phosphatidylcholine (16:0_18:3) levels	Parkinson's disease	rs5025214	T	G	T	G	-8.17E-02	-2.70E-02	6.80E-01	3.76E-01	FALSE	FALSE	8	6103083	2.31E-02	6.17E-01	TRUE	8	6245562	1.79E-02	1.00	7174	5.20E-06	TRUE	TRUE	482,730	20.79
Phosphatidylcholine (16:0_18:3) levels	Parkinson's disease	rs55841968	T	G	T	C	7.66E-02	5.80E-02	4.93E-01	4.33E-01	FALSE	FALSE	6	123533406	2.30E-02	5.84E-03	TRUE	6	123212261	1.69E-02	0.99	7174	5.99E-06	TRUE	TRUE	482,730	20.52
Phosphatidylcholine (16:0_18:3) levels	Parkinson's disease	rs60847460	T	C	T	C	-1.08E-01	1.95E-02	1.40E-01	8.55E-01	FALSE	FALSE	10	113983758	3.17E-02	2.69E-01	TRUE	10	112224000	2.41E-02	0.99	7174	7.20E-06	TRUE	TRUE	482,730	20.16
Phosphatidylcholine (16:0_18:3) levels	Parkinson's disease	rs7139079	A	G	A	G	-7.65E-02	1.53E-02	5.47E-01	4.21E-01	FALSE	FALSE	12	121415293	1.78E-02	4.09E-01	TRUE	12	120977490	1.68E-02	1.00	7174	5.11E-06	TRUE	TRUE	482,730	20.82
Phosphatidylcholine (16:0_18:3) levels	Parkinson's disease	rs79189815	G	A	G	A	4.31E-01	-5.17E-01	9.08E-03	9.96E-01	FALSE	FALSE	5	61487042	3.15E-01	1.00E+00	TRUE	5	62191215	9.10E-02	0.95	7174	2.19E-06	TRUE	TRUE	482,730	22.46
Phosphatidylcholine (16:0_18:3) levels	Parkinson's disease	rs9397785	G	T	G	T	-7.89E-02	4.60E-03	6.55E-01	3.05E-01	FALSE	FALSE	6	155477640	2.44E-02	7.08E-02	TRUE	6	155156506	1.76E-02	1.00	7174	7.36E-06	TRUE	TRUE	482,730	20.12
Phosphatidylcholine (16:0_20:1) levels	Parkinson's disease	rs10490936	G	A	G	A	-4.27E-01	-1.63E-02	9.62E-03	9.84E-01	FALSE	FALSE	10	83849297	1.07E-01	5.61E-02	TRUE	10	82089541	9.53E-02	0.83	6882	7.47E-06	TRUE	TRUE	482,730	20.10
Phosphatidylcholine (16:0_20:1) levels	Parkinson's disease	rs10995430	T	C	T	C	-8.43E-02	1.20E-03	6.50E-01	3.87E-01	FALSE	FALSE	10	64847748	2.02E-02	1.12E-01	TRUE	10	63087988	1.79E-02	0.99	6882	2.61E-06	TRUE	TRUE	482,730	22.12
Phosphatidylcholine (16:0_20:1) levels	Parkinson's disease	rs12707188	T	C	T	C	9.22E-02	1.05E-02	4.17E-01	6.46E-01	FALSE	FALSE	7	134591097	1.92E-02	2.34E-01	TRUE	7	134906346	1.76E-02	0.98	6882	1.66E-07	TRUE	TRUE	482,730	27.45
Phosphatidylcholine (16:0_20:1) levels	Parkinson's disease	rs1409892	A	G	A	G	7.89E-02	1.20E-02	4.48E-01	5.75E-01	FALSE	FALSE	6	51265608	1.74E-02	3.11E-01	TRUE	6	51400810	1.72E-02	1.00	6882	4.54E-06	TRUE	TRUE	482,730	21.06
Phosphatidylcholine (16:0_20:1) levels	Parkinson's disease	rs147707008	A	G	A	G	-1.98E-01	4.26E-02	4.29E-02	9.80E-01	FALSE	FALSE	2	13290257	2.05E-01	7.82E-02	TRUE	2	13150132	4.36E-02	0.95	6882	5.91E-06	TRUE	TRUE	482,730	20.55
Phosphatidylcholine (16:0_20:1) levels	Parkinson's disease	rs150608914	A	G	A	G	1.95E-01	-8.28E-02	4.97E-02	9.80E-01	FALSE	FALSE	5	136680656	8.78E-02	4.61E-01	TRUE	5	137344967	3.95E-02	0.99	6882	8.25E-07	TRUE	TRUE	482,730	24.34
Phosphatidylcholine (16:0_20:1) levels	Parkinson's disease	rs174594	A	C	A	C	-1.15E-01	9.90E-03	5.82E-01	3.63E-01	FALSE	FALSE	11	61619829	1.78E-02	2.39E-01	TRUE	11	61852357	1.73E-02	1.00	6882	3.58E-11	TRUE	TRUE	482,730	43.97
Phosphatidylcholine (16:0_20:1) levels	Parkinson's disease	rs17715191	G	A	G	A	1.06E-01	-2.56E-02	1.83E-01	8.26E-01	FALSE	FALSE	10	116108003	3.60E-02	3.21E-01	TRUE	10	114348244	2.31E-02	0.92	6882	4.77E-06	TRUE	TRUE	482,730	20.96
Phosphatidylcholine (16:0_20:1) levels	Parkinson's disease	rs2268493	C	T	C	T	8.45E-02	-1.82E-02	3.92E-01	7.06E-01	FALSE	FALSE	3	8800840	2.54E-02	3.24E-01	TRUE	3	8759154	1.76E-02	0.98	6882	1.60E-06	TRUE	TRUE	482,730	23.06
Phosphatidylcholine (16:0_20:1) levels	Parkinson's disease	rs2499085	C	T	C	T	1.10E-01	1.45E-02	8.46E-01	1.38E-01	FALSE	FALSE	10	19591177	4.45E-02	1.28E-01	TRUE	10	19302248	2.43E-02	0.94	6882	6.30E-06	TRUE	TRUE	482,730	20.42
Phosphatidylcholine (16:0_20:1) levels	Parkinson's disease	rs73728261	T	C	T	C	1.17E-01	2.79E-02	1.29E-01	9.02E-01	FALSE	FALSE	6	18747925	4.01E-02	3.12E-01	TRUE	6	18747694	2.50E-02	1.00	6882	3.11E-06	TRUE	TRUE	482,730	21.78
Phosphatidylcholine (16:0_20:2) levels	Parkinson's disease	rs10160784	C	T	C	T	-1.10E-01	1.60E-02	2.32E-01	8.33E-01	FALSE	FALSE	11	75456055	2.82E-02	2.44E-01	TRUE	11	75745010	1.99E-02	0.99	7135	3.61E-08	TRUE	TRUE	482,730	30.42
Phosphatidylcholine (16:0_20:2) levels	Parkinson's disease	rs113130033	G	A	G	A	-1.97E-01	-4.14E-02	4.30E-02	9.36E-01	FALSE	FALSE	14	90186309	4.77E-02	4.15E-01	TRUE	14	89719965	4.11E-02	0.98	7135	1.73E-06	TRUE	TRUE	482,730	22.91
Phosphatidylcholine (16:0_20:2) levels	Parkinson's disease	rs113550786	G	A	G	A	3.93E-01	-6.48E-02	1.23E-02	9.72E-01	FALSE	FALSE	16	13984366	9.55E-02	3.03E-01	TRUE	16	13890509	8.27E-02	0.85	7135	2.06E-06	TRUE	TRUE	482,730	22.57
Phosphatidylcholine (16:0_20:2) levels	Parkinson's disease	rs1168041	C	T	C	T	9.26E-02	-1.92E-02	7.33E-01	3.18E-01	FALSE	FALSE	1	62960250	2.05E-02	4.57E-01	TRUE	1	62494579	1.89E-02	0.99	7135	9.29E-07	TRUE	TRUE	482,730	24.11
Phosphatidylcholine (16:0_20:2) levels	Parkinson's disease	rs1260326	C	T	C	T	-9.72E-02	-6.78E-02	6.51E-01	4.20E-01	FALSE	FALSE	2	27730940	1.72E-02	4.04E-05	TRUE	2	27508073	1.75E-02	1.00	7135	3.00E-08	TRUE	TRUE	482,730	30.78
Phosphatidylcholine (16:0_20:2) levels	Parkinson's disease	rs12700446	T	C	T	C	3.09E-01	4.72E-02	1.61E-02	9.66E-01	FALSE	FALSE	7	23571744	5.50E-02	4.08E-01	TRUE	7	23532125	6.74E-02	0.98	7135	4.73E-06	TRUE	TRUE	482,730	20.97
Phosphatidylcholine (16:0_20:2) levels	Parkinson's disease	rs12761749	C	T	C	T	-1.05E-01	1.16E-02	1.50E-01	7.14E-01	FALSE	FALSE	10	92054484	2.49E-02	1.93E-01	TRUE	10	90294727	2.37E-02	0.99	7135	9.93E-06	TRUE	TRUE	482,730	19.55
Phosphatidylcholine (16:0_20:2) levels	Parkinson's disease	rs142392117	C	T	C	T	-2.77E-01	-2.17E-02	2.16E-02	9.83E-01	FALSE	FALSE	4	177549092	9.10E-02	9.09E-02	TRUE	4	176627941	5.73E-02	0.97	7135	1.40E-06	TRUE	TRUE	482,730	23.33
Phosphatidylcholine (16:0_20:2) levels	Parkinson's disease	rs148069564	T	A	T	A	-7.77E-01	1.06E-01	2.99E-03	2.56E-02	TRUE	FALSE	10	53552112	7.69E-02	7.76E-01	TRUE	10	51792352	1.67E-01	0.88	7135	3.28E-06	TRUE	TRUE	482,730	21.68
Phosphatidylcholine (16:0_20:2) levels	Parkinson's disease	rs17126640	A	G	A	G	-9.82E-02	3.37E-02	2.03E-01	8.80E-01	FALSE	FALSE	12	52704991	2.81E-02	6.37E-01	TRUE	12	52311207	2.09E-02	1.00	7135	2.70E-06	TRUE	TRUE	482,730	22.05
Phosphatidylcholine (16:0_20:2) levels	Parkinson's disease	rs17128436	G	G	C	G	3.09E-01	-1.00E-04	1.57E-02	3.20E-02	TRUE	FALSE	11	124016865	4.96E-02	9.13E-04	TRUE	11	124146158	6.89E-02	0.98	7135	7.29E-06	TRUE	TRUE	482,730	20.14

Phosphatidylcholine (16:0_20:2) levels	Parkinson's disease	rs17268633	G	A	G	A	-2.85E-01	-1.75E-02	2.03E-02	9.62E-01	FALSE	FALSE	2	56422056	5.87E-02	1.16E-01	TRUE	2	56194921	6.01E-02	0.99	7135	2.07E-06	TRUE	TRUE	482,730	22.57
Phosphatidylcholine (16:0_20:2) levels	Parkinson's disease	rs174592	G	A	G	A	2.62E-01	-6.80E-03	4.18E-01	6.42E-01	FALSE	FALSE	11	61618608	1.78E-02	1.54E-01	TRUE	11	61851136	1.68E-02	1.00	7135	4.96E-54	TRUE	TRUE	482,730	243.59
Phosphatidylcholine (16:0_20:2) levels	Parkinson's disease	rs1800588	T	C	T	C	1.07E-01	-3.30E-03	2.58E-01	7.75E-01	FALSE	FALSE	15	58723675	2.12E-02	5.73E-02	TRUE	15	58431476	1.94E-02	0.99	7135	3.85E-08	TRUE	TRUE	482,730	30.29
Phosphatidylcholine (16:0_20:2) levels	Parkinson's disease	rs1815763	A	G	A	G	-3.66E-01	1.90E-03	1.12E-02	9.78E-01	FALSE	FALSE	11	66842737	6.15E-02	1.06E-02	TRUE	11	67075266	7.97E-02	0.98	7135	4.39E-06	TRUE	TRUE	482,730	21.12
Phosphatidylcholine (16:0_20:2) levels	Parkinson's disease	rs2236514	G	C	G	C	-9.07E-02	2.80E-03	6.47E-01	6.35E-01	TRUE	FALSE	9	139572068	2.00E-02	5.10E-02	TRUE	9	136677616	1.78E-02	0.95	7135	3.85E-07	TRUE	TRUE	482,730	25.81
Phosphatidylcholine (16:0_20:2) levels	Parkinson's disease	rs2317091	A	T	A	T	2.18E-01	-3.11E-02	4.03E-02	3.74E-02	TRUE	FALSE	11	62960760	4.96E-02	2.75E-01	TRUE	11	63193288	4.30E-02	0.99	7135	4.11E-07	TRUE	TRUE	482,730	25.69
Phosphatidylcholine (16:0_20:2) levels	Parkinson's disease	rs3132935	G	A	G	A	-9.70E-02	-3.75E-02	1.77E-01	8.21E-01	FALSE	FALSE	6	32171075	2.57E-02	8.41E-01	TRUE	6	32203298	2.17E-02	1.00	7135	7.80E-06	TRUE	TRUE	482,730	20.02
Phosphatidylcholine (16:0_20:2) levels	Parkinson's disease	rs34516410	T	G	T	G	-1.14E-01	4.20E-03	1.33E-01	8.10E-01	FALSE	FALSE	6	100416334	2.84E-02	5.36E-02	TRUE	6	99968458	2.48E-02	0.99	7135	4.16E-06	TRUE	TRUE	482,730	21.22
Phosphatidylcholine (16:0_20:2) levels	Parkinson's disease	rs72800299	A	T	A	T	-8.49E-02	-3.81E-02	2.74E-01	2.38E-01	TRUE	FALSE	10	67154976	2.66E-02	8.18E-01	TRUE	10	65395218	1.85E-02	0.99	7135	4.69E-06	TRUE	TRUE	482,730	20.99
Phosphatidylcholine (16:0_20:2) levels	Parkinson's disease	rs78230994	A	G	A	G	5.95E-01	4.49E-02	5.53E-03	9.79E-01	FALSE	FALSE	4	26560197	8.92E-02	2.12E-01	TRUE	4	26558575	1.23E-01	0.88	7135	1.25E-06	TRUE	TRUE	482,730	23.54
Phosphatidylcholine (16:0_20:2) levels	Parkinson's disease	rs79718024	G	A	G	A	-1.13E-01	-6.50E-03	1.55E-01	8.70E-01	FALSE	FALSE	11	107440345	3.46E-02	7.03E-02	TRUE	11	107569619	2.35E-02	0.97	7135	1.76E-06	TRUE	TRUE	482,730	22.88
Phosphatidylcholine (16:0_20:3) levels	Parkinson's disease	rs10102672	G	C	G	C	8.65E-02	-2.01E-02	2.93E-01	2.64E-01	TRUE	FALSE	8	39040785	2.26E-02	4.27E-01	TRUE	8	39183266	1.84E-02	1.00	7174	2.57E-06	TRUE	TRUE	482,730	22.15
Phosphatidylcholine (16:0_20:3) levels	Parkinson's disease	rs1168041	C	T	C	T	8.93E-02	-1.92E-02	7.33E-01	3.18E-01	FALSE	FALSE	1	62960250	2.05E-02	4.57E-01	TRUE	1	62494579	1.88E-02	0.99	7174	2.10E-06	TRUE	TRUE	482,730	22.54
Phosphatidylcholine (16:0_20:3) levels	Parkinson's disease	rs116917626	T	C	T	C	-2.34E-01	1.84E-02	2.75E-02	9.86E-01	FALSE	FALSE	17	49639325	1.53E-01	4.36E-02	TRUE	17	51561964	5.25E-02	0.97	7174	8.10E-06	TRUE	TRUE	482,730	19.94
Phosphatidylcholine (16:0_20:3) levels	Parkinson's disease	rs117372257	T	C	T	C	-4.02E-01	7.09E-02	9.43E-03	9.87E-01	FALSE	FALSE	9	73630624	1.03E-01	3.09E-01	TRUE	9	71015708	8.82E-02	0.99	7174	5.23E-06	TRUE	TRUE	482,730	20.78
Phosphatidylcholine (16:0_20:3) levels	Parkinson's disease	rs1260326	C	T	C	T	-7.74E-02	-6.78E-02	6.51E-01	4.20E-01	FALSE	FALSE	2	27730940	1.72E-02	4.04E-05	TRUE	2	27508073	1.75E-02	1.00	7174	9.67E-06	TRUE	TRUE	482,730	19.60
Phosphatidylcholine (16:0_20:3) levels	Parkinson's disease	rs138705401	T	C	T	C	1.83E-01	-6.06E-02	4.71E-02	9.53E-01	FALSE	FALSE	12	44004140	5.26E-02	6.04E-01	TRUE	12	43610337	3.98E-02	0.98	7174	4.48E-06	TRUE	TRUE	482,730	21.08
Phosphatidylcholine (16:0_20:3) levels	Parkinson's disease	rs1410901	T	C	T	C	7.92E-02	1.33E-02	5.05E-01	5.43E-01	FALSE	FALSE	10	92005165	2.78E-02	1.98E-01	TRUE	10	90245408	1.70E-02	0.94	7174	3.24E-06	TRUE	TRUE	482,730	21.70
Phosphatidylcholine (16:0_20:3) levels	Parkinson's disease	rs141883259	G	A	G	A	3.80E-01	1.48E-01	1.02E-02	9.87E-01	FALSE	FALSE	5	119396702	1.01E-01	8.40E-01	TRUE	5	120061007	8.30E-02	0.98	7174	4.89E-06	TRUE	TRUE	482,730	20.91
Phosphatidylcholine (16:0_20:3) levels	Parkinson's disease	rs143838492	A	G	A	G	6.78E-01	1.82E-01	3.33E-03	9.90E-01	FALSE	FALSE	5	114228685	1.54E-01	6.27E-01	TRUE	5	114892988	1.49E-01	0.97	7174	5.19E-06	TRUE	TRUE	482,730	20.80
Phosphatidylcholine (16:0_20:3) levels	Parkinson's disease	rs319552	C	G	C	G	8.71E-02	-6.00E-03	2.55E-01	2.82E-01	TRUE	FALSE	13	93826052	3.07E-02	7.26E-02	TRUE	13	93173799	1.93E-02	0.96	7174	6.23E-06	TRUE	TRUE	482,730	20.45
Phosphatidylcholine (16:0_20:3) levels	Parkinson's disease	rs56374730	G	T	G	T	8.34E-02	-3.00E-02	2.96E-01	7.43E-01	FALSE	FALSE	16	15867652	2.56E-02	6.19E-01	TRUE	16	15773795	1.84E-02	0.99	7174	5.80E-06	TRUE	TRUE	482,730	20.58
Phosphatidylcholine (16:0_20:3) levels	Parkinson's disease	rs6498540	G	A	G	A	-1.92E-01	1.82E-02	3.34E-01	6.94E-01	FALSE	FALSE	16	15130594	1.84E-02	4.93E-01	TRUE	16	15036737	1.76E-02	0.99	7174	1.90E-27	TRUE	TRUE	482,730	118.79
Phosphatidylcholine (16:0_20:3) levels	Parkinson's disease	rs77419701	C	T	C	T	-2.00E-01	3.23E-02	3.70E-02	9.62E-01	FALSE	FALSE	13	67532568	6.08E-02	2.25E-01	TRUE	13	66958436	4.39E-02	0.99	7174	5.36E-06	TRUE	TRUE	482,730	20.73
Phosphatidylcholine (16:0_20:3) levels	Parkinson's disease	rs77990460	C	A	C	A	-3.07E-01	4.68E-02	1.71E-02	9.84E-01	FALSE	FALSE	2	126885556	1.08E-01	1.76E-01	TRUE	2	126127979	6.73E-02	0.92	7174	5.35E-06	TRUE	TRUE	482,730	20.74
Phosphatidylcholine (16:0_20:3) levels	Parkinson's disease	rs968567	T	C	T	C	2.57E-01	-3.41E-02	1.13E-01	8.31E-01	FALSE	FALSE	11	61595564	2.24E-02	8.94E-01	TRUE	11	61828092	2.64E-02	1.00	7174	3.05E-22	TRUE	TRUE	482,730	94.69
Phosphatidylcholine (16:0_20:4) levels	Parkinson's disease	rs11062107	G	A	G	A	-8.90E-02	7.20E-03	2.23E-01	8.27E-01	FALSE	FALSE	12	2187041	3.03E-02	8.99E-02	TRUE	12	2077875	2.00E-02	0.99	7174	9.12E-06	TRUE	TRUE	482,730	19.71
Phosphatidylcholine (16:0_20:4) levels	Parkinson's disease	rs113394924	G	T	G	T	2.14E-01	-6.22E-02	8.73E-02	9.66E-01	FALSE	FALSE	11	62082293	5.88E-02	5.37E-01	TRUE	11	62314821	2.96E-02	0.99	7174	6.09E-13	TRUE	TRUE	482,730	52.01
Phosphatidylcholine (16:0_20:4) levels	Parkinson's disease	rs116438525	T	C	T	C	-4.70E-01	-1.74E-01	7.09E-03	9.90E-01	FALSE	FALSE	4	182566303	1.45E-01	6.35E-01	TRUE	4	181645150	1.02E-01	0.95	7174	4.05E-06	TRUE	TRUE	482,730	21.27
Phosphatidylcholine (16:0_20:4) levels	Parkinson's disease	rs116643980	T	G	T	G	1.67E-01	1.84E-01	5.56E-02	9.83E-01	FALSE	FALSE	1	4907719	1.05E-01	4.04E-02	TRUE	1	4847659	3.66E-02	0.95	7174	4.84E-06	TRUE	TRUE	482,730	20.93
Phosphatidylcholine (16:0_20:4) levels	Parkinson's disease	rs12720922	A	G	A	G	-9.64E-02	8.60E-03	1.79E-01	8.19E-01	FALSE	FALSE	16	57000885	2.23E-02	1.56E-01	TRUE	16	56966973	2.17E-02	1.00	7174	9.09E-06	TRUE	TRUE	482,730	19.72
Phosphatidylcholine (16:0_20:4) levels	Parkinson's disease	rs13329893	C	A	C	A	1.58E-01	1.62E-02	5.96E-02	9.39E-01	FALSE	FALSE	16	87706536	4.80E-02	1.33E-01	TRUE	16	87672930	3.55E-02	0.98	7174	9.16E-06	TRUE	TRUE	482,730	19.71
Phosphatidylcholine (16:0_20:4) levels	Parkinson's disease	rs141391095	G	A	G	A	-2.81E-01	-1.68E-02	2.09E-02	9.66E-01	FALSE	FALSE	17	10666339	6.66E-02	9.67E-02	TRUE	17	10763022	5.97E-02	0.93	7174	2.58E-06	TRUE	TRUE	482,730	22.14
Phosphatidylcholine (16:0_20:4) levels	Parkinson's disease	rs147549994	T	G	T	G	2.46E-01	-1.38E-02	3.59E-02	9.86E-01	FALSE	FALSE	16	1989265	1.01E-01	4.97E-02	TRUE	16	1939264	4.53E-02	0.95	7174	5.51E-08	TRUE	TRUE	482,730	29.59
Phosphatidylcholine (16:0_20:4) levels	Parkinson's disease	rs149130695	C	A	C	A	1.54E-01	-4.20E-03	6.50E-02	9.30E-01	FALSE	FALSE	4	187385348	5.64E-02	2.64E-02	TRUE	4	186464194	3.48E-02	0.95	7174	9.36E-06	TRUE	TRUE	482,730	19.66
Phosphatidylcholine (16:0_20:4) levels	Parkinson's disease	rs17017629	T	C	T	C	-2.31E-01	-1.34E-01	2.68E-02	9.56E-01	FALSE	FALSE	4	144273358	5.40E-02	6.47E-03	TRUE	4	143352205	5.12E-02	1.00	7174	6.78E-06	TRUE	TRUE	482,730	20.29
Phosphatidylcholine (16:0_20:4) levels	Parkinson's disease	rs17366743	C	T	C	T	-2.03E-01	-5.63E-02	3.41E-02	9.69E-01	FALSE	FALSE	3	186572089	5.48E-02	5.18E-01	TRUE	3	186854300	4.59E-02	1.00	7174	9.68E-06	TRUE	TRUE	482,730	19.60
Phosphatidylcholine (16:0_20:4) levels	Parkinson's disease	rs174533	A	G	A	G	-5.98E-01	-9.00E-04	4.09E-01	6.69E-01	FALSE	FALSE	11	61549025	1.79E-02	1.84E-02	TRUE	11	61781553	1.55E-02	1.00	7174	1.00E-200	TRUE	TRUE	482,730	1481.30
Phosphatidylcholine (16:0_20:4) levels	Parkinson's disease	rs2943813	C	T	C	T	-1.39E-01	-4.50E-03	7.83E-01	2.16E-01	FALSE	FALSE	11	61258468	2.30E-02	7.25E-02	TRUE	11	61490996	2.04E-02	1.00	7174	9.96E-12	TRUE	TRUE	482,730	46.48
Phosphatidylcholine (16:0_20:4) levels	Parkinson's disease	rs2966427	G	C	G	C	-1.47E-01	4.63E-02	9.32E-01	8.98E-01	TRUE	FALSE	7	110206775	3.71E-02	6.72E-01	TRUE	7	110566718	3.28E-02	1.00	7174	7.78E-06	TRUE	TRUE	482,730	20.02
Phosphatidylcholine (16:0_20:4) levels	Parkinson's disease	rs3018617	G	A	G	A	-1.08E-01	3.21E-02	3.02E-01	6.78E-01	FALSE	FALSE	11	62202934	3.28E-02	4.84E-01	TRUE	11	62435462	1.88E-02	0.94	7174	8.62E-09	TRUE	TRUE	482,730	33.21
Phosphatidylcholine (16:0_20:4) levels	Parkinson's disease	rs3820438	C	G	C	G	8.91E-02	5.55E-02	2.91E-01	2.27E-01	TRUE	FALSE	1	201924749	3.28E-02	4.53E-02	TRUE	1	201955621	1.83E-02	0.98	7174	1.15E-06	TRUE	TRUE	482,730	23.71
Phosphatidylcholine (16:0_20:4) levels	Parkinson's disease	rs4756323	T	C	T	C	-7.76E-02	3.80E-02	4.56E-01	5.36E-01	FALSE	FALSE	11	36491070	2.26E-02	4.63E-02	TRUE	11	36469520	1.66E-02	0.99	7174	3.22E-06	TRUE	TRUE	482,730	21.72
Phosphatidylcholine (16:0_20:4) levels	Parkinson's disease	rs499974	A	C	A	C	-1.03E-01	1.23E-02	2.32E-01	8.34E-01	FALSE	FALSE	11	75455021	2.83E-02	1.79E-01	TRUE	11	75743976	1.98E-02	0.99	7174	2.26E-07	TRUE	TRUE	482,730	26.85
Phosphatidylcholine (16:0_20:4) levels	Parkinson's disease	rs58160859	T	C	T	C	9.25E-02	-6.10E-03	2.21E-01	7.77E-01	FALSE	FALSE	7	155892507	2.75E-02	8.39E-02	TRUE	7	156099813	2.04E-02	0.98	7174	5.72E-06	TRUE	TRUE	482,730	20.61
Phosphatidylcholine (16:0_20:4) levels	Parkinson's disease	rs6498540	G	A	G	A	-7.88E-02	1.82E-02	3.34E-01	6.94E-01	FALSE	FALSE	16	15130594	1.84E-02	4.93E-01	TRUE	16	15036737	1.77E-02	0.99	7174	8.47E-06	TRUE	TRUE	482,730	19.86
Phosphatidylcholine (16:0_20:4) levels	Parkinson's disease	rs72709529	A	C	A	C	-2.72E-01	-6.63E-02	2.33E-02	9.23E-01	FALSE	FALSE	14	101863895	4.25E-02	9.25E-01	TRUE	14	101397558	5.59E-02	0.97	7174	1.13E-06	TRUE	TRUE	482,730	23.74
Phosphatidylcholine (16:0_20:4) levels	Parkinson's disease	rs74754540	A	G	A	G	-1.79E-01	-2.79E-02	4.92E-02	9.73E-01	FALSE	FALSE	11	61716357	6.82E-02	1.66E-01	TRUE	11	61948885	3.88E-02	0.99	7174	4.19E-06	TRUE	TRUE	482,730	21.21
Phosphatidylcholine (16:0_20:5) levels	Parkinson's disease	rs102274	C	T	C	T	-3.73E-01	-3.90E-03	4.09E-01	6.70E-01	FALSE	FALSE	11	61557826	1.79E-02	8.34E-02	TRUE	11	61790354	1.65E-02	1.00	7173	4.34E-109	TRUE	TRUE	482,730	509.87
Phosphatidylcholine (16:0_20:5) levels	Parkinson's disease	rs10936925	T	G	T	G	7.86E-02	-5.03E-02	3.40E-01	7.00E-01	FALSE	FALSE	3	176733158	2.42E-02	1.88E-02	TRUE	3	177015370	1.77E-02	1.00	7173	8.89E-06	TRUE	TRUE	482,730	19.76
Phosphatidylcholine (16:0_20:5) levels	Parkinson's disease	rs113394924	G	T	G	T	1.41E-01	-6.22E-02	8.73E-02	9.66E-01	FALSE	FALSE	11	62082293	5.88E-02	5.37E-01	TRUE	11	62314821	2.97E-02	0.99	7173	2.28E-06	TRUE	TRUE	482,730	22.38
Phosphatidylcholine (16:0_20:5) levels	Parkinson's disease	rs137945273	T	C	T	C	-2.04E-01	-2.38E-02	3.68E-02	9.83E-01	FALSE	FALSE	14	71737548	1.91E-01	4.52E-02	TRUE	14	71270831	4.54E-02	0.93	7173	7.22E-06	TRUE	TRUE	482,730	20.16
Phosphatidylcholine (16:0_20:5) levels	Parkinson's disease	rs146489454	T	C	T	C	-2.17E-01	3.65E-02	3.14E-02	9.87E-01	FALSE	FALSE	5	114760381	1.11E-01	1.29E-01	TRUE	5	115424684	4.89E-02	0.97	7173	9.32E-06	TRUE	TRUE	482,730	19.67
Phosphatidylcholine (16:0_20:5) levels	Parkinson's disease	rs1481977	G	A	G	A	1.09E-01	9.40E-03	8.61E-01	1.51E-01	FALSE	FALSE	11	103469398	3.13E-02	1.18E-01	TRUE	11	103598670	2.44E-02	1.00	7173	7.66E-06	TRUE	TRUE	482,730	20.05
Phosphatidylcholine (16:0_20:5) levels	Parkinson's disease	rs2041149	G	A	G	A	-7.87E-02	3.27E-02	4.77E-01	6.04E-01	FALSE	FALSE	12	102110105	1.86E-02	3.94E-02	TRUE	12	101716327	1.68E-02	1.00	7173	2.73E-06	TRUE	TRUE	482,730	22.03
Phosphatidylcholine (16:0_20:5) levels	Parkinson's disease	rs2733412	C	T	C	T	-9.55E-02	-2.21E-02	1.89E-01	8.03E-01	FALSE	FALSE	3	114147043	2.38E-02	4.52E-01	TRUE	3	114428196	2.15E-02	0.99	7173	8.72E-06	TRUE	TRUE	482,730	19.80
Phosphatidylcholine (16:0_20:5) levels	Parkinson's disease	rs28569162	G	A	G	A	1.15E-01	-3.15E-02	1.31E-01	8.23E-01	FALSE	FALSE	11	87686616	2.87E-02	5.65E-01	TRUE	11	87975724	2.52E-02	1.00	7173	4.68E-06	TRUE	TRUE	482,730	20.99
Phosphatidylcholine (16:0_20:5) levels	Parkinson's disease	rs3018617	G	A	G	A	-9.35E-02	3.21E-02	3.02E-01	6.78E-01	FALSE	FALSE	11	62202934	3.28E-02	4.84E-01	TRUE	11	62435462	1.88E-02	0.94	7173	7.08E-07	TRUE	TRUE	482,730	24.63
Phosphatidylcholine (16:0_20:5) levels	Parkinson's disease	rs34822092	A	T	A	T	-2.83E-01	-6.78E-02	2.25E-02	3.35E-02	TRUE	FALSE	2	52350739	6.41E-02	5.38E-01	TRUE	2	52123601	5.64E-02	0.98	7173	5.21E-07	TRUE	TRUE	482,730	25.23
Phosphatidylcholine (16:0_20:5) levels	Parkinson's disease	rs3796575	T	C	T	C	-9.16E-02	-5.10E-03	2.28E-01	6.89E-01	FALSE	FALSE	4	156721739	2.42E-02	7.89E-02	TRUE	4	155800587	2.03E-02	0.97	7173	6.46E-06	TRUE	TRUE	482,730	20.38
Phosphatidylcholine (16:0_20:5) levels	Parkinson's disease	rs3936196	A	G	A	G	-7.74E-02	1.12E-02	6.09E-01	4.02E-01	FALSE	FALSE	2	240818046	2.90E-02	1.55E-01	TRUE	2	239878629	1.74E-02	0.95	7173	9.25E-06	TRUE	TRUE	482,730	19.69
Phosphatidylcholine (16:0_20:5) levels	Parkinson's disease	rs4908782	T	C	T	C	-7.97E-02	1.20E-02	3.54E-01	6.89E-01	FALSE	FALSE	1	8888114	2.40E-02	2.10E-01	TRUE	1	8828055	1.75E-02	0.99	7173	5.17E-06	TRUE	TRUE	482,730	20.80
Phosphatidylcholine (16:0_20:5) levels	Parkinson's disease	rs531117	T	C	T	C	-8.84E-02	1.42E-02	2.32E-01	8.33E-01	FALSE	FALSE	11	75456134	2.83E-02	2.11E-01	TRUE	11	75745089	1.99E-02	0.99	7173	8.73E-06	TRUE	TRUE	482,730	19.80
Phosphatidylcholine (16:0_20:5) levels	Parkinson's disease	rs59839417	A	G	A	G	-9.91E-02	-2.32E-02	2.06E-01	7.86E-01	FALSE	FALSE	3	39290911	2.13E-02	5.57E-01	TRUE	3	39249420	2.08E-02	1.00	7173	2.02E-06	TRUE	TRUE	482,730	22.61
Phosphatidylcholine (16:0_20:5) levels	Parkinson's disease	rs61653779	C	T	C	T	2.74E-01	-5.22E-02	2.01E-02	9.74E-01	FALSE	FALSE	16	20580226	5.54E-02	4.61E-01	TRUE	16	20568904	6.04E-02	1.00	7173	5.99E-06	TRUE	TRUE	482,730	20.52
Phosphatidylcholine (16:0_20:5) levels	Parkinson's disease	rs62025936	G	A	G	A	1.21E-01	2.69E-02	1.65E-01	8.40E-01	FALSE	FALSE	16	10528036	3.04E-02	4.25E-01	TRUE	16	10434179	2.27E-02	0.99	7173	1.18E-07	TRUE	TRUE	482,730	28.10
Phosphatidylcholine (16:0_20:5) levels	Parkinson's disease	rs74754540	A	G	A	G	-1.89E-01	-2.79E-02	4.92E-02	9.73E-01	FALSE	FALSE	11	61716357	6.82E-02	1.66E-01	TRUE	11	61948885	3.89E-02	0.99	7173	1.18E-06	TRUE	TRUE	482,730	23.65
Phosphatidylcholine (16:0_20:5) levels	Parkinson's disease	rs7780677	A	C	A	C	-8.62E-02	2.17E-02	4.09E-01	6.73E-01	FALSE	FALSE	7	132378493	2.38E-02	4.41E-01	TRUE	7	132693734	1.70E-02	1.00	7173	4.33E-07	TRUE	TRUE	482,730	25.59
Phosphatidylcholine (16:0_20:5) levels	Parkinson's disease	rs7947954	C	T	C	T	1.08E-01	-2.94E-02	1.55E-01	8.79E-01	FALSE	FALSE	11	42678748	3.44E-02	4.07E-01	TRUE	11	42657198	2.30E-02	1.00	7173	2.80E-06	TRUE	TRUE	482,730	21.98
Phosphatidylcholine (16:0_20:5) levels	Parkinson's disease	rs80083325	A	G	A	G	-2.14E-01	-3.54E-02	3.48E-02	9.48E-01	FALSE	FALSE	14	104863663	5.83E-02	2.65E-01	TRUE	14	104397326	4.82E-02	0.90	7173	8.69E-06	TRUE	TRUE	482,730	19.81
Phosphatidylcholine (16:0_22:4) levels	Parkinson's disease	rs10138425	G	A	G	A	-1.02E-01	-8.17E-02	1.65E-01	8.40E-01	FALSE	FALSE	14	100604334	2.96E-02	2.89E-03	TRUE	14	100137997	2.29E-02	1.00	6874	8.73E-06	TRUE	TRUE	482,730	19.80
Phosphatidylcholine (16:0_22:4) levels	Parkinson's disease	rs115448758	C	T	C	T	2.00E-01	-1.35E-01	4.92E-02	9.71E-01	FALSE	FALSE	4	137274943	7.44E-02	3.44E-02	TRUE	4	136353788	4.04E-02	0.95	6874	7.11E-07	TRUE	TRUE	482,730	24.63
Phosphatidylcholine (16:0_22:4) levels	Parkinson's disease	rs116522970	G	T	G	T	5.65E-01	3.65E-02	4.90E-03	9.83E-01	FALSE	FALSE	4	14331720	8.87E-02	1.67E-01	TRUE	4	14330096	1.27E-01	0.96	6874	8.98E-06	TRUE	TRUE	482,730	19.75
Phosphatidylcholine (16:0_22:4) levels	Parkinson's disease	rs12528390	A	G	A	G	1.82E-01	-2.15E-02	5.57E-02	9.42E-01	FALSE	FALSE	6	149273839	4.80E-02	1.85E-01	TRUE	6	148952703	3.74E-02	0.99	6874	1.22E-06	TRUE	TRUE	482,730	23.59
Phosphatidylcholine (16:0_22:4) levels	Parkinson's disease	rs1260326	C	T	C	T	-8.03E-02	-6.78E-02	6.51E-01	4.20E-01	FALSE	FALSE	2	27730940	1.72E-02	4.04E-05	TRUE	2	27508073	1.78E-02	1.00	6874	6.72E-06	TRUE	TRUE	482,730	20.30
Phosphatidylcholine (16:0_22:4) levels	Parkinson's disease	rs1360225	C	T	C	T	1.02E-01	2.58E-02	2.02E-01	8.53E-01	FALSE	FALSE	9	23713148	3.15E-02	3.84E-01	TRUE	9	23713150	2.13E-02	1.00	6874	1.66E-06	TRUE	TRUE	482,730	23.00
Phosphatidylcholine (16:0_22:4) levels	Parkinson's disease	rs1393144	A	C	A	C	-1.04E-01	3.62E-02	1.72E-01	8.00E-01	FALSE	FALSE	5	125339876	2.81E-02	7.05E-01	TRUE	5	126004183	2.25E-02	0.99	6874	3.99E-06	TRUE	TRUE	482,730	21.30
Phosphatidylcholine (16:0_22:4) levels	Parkinson's disease	rs140028072	T	C	T	C	-2.17E-01	-8.47E-02	3.44E-02	9.84E-01	FALSE	FALSE	9	6890384	9.08E-02	4.55E-01	TRUE	9	6890384	4.74E-02	0.98	6874	4.65E-06	TRUE	TRUE	482,730	21.01
Phosphatidylcholine (16:0_22:4) levels	Parkinson's disease	rs148506839	A	T	A	T	-2.33E-01	9.21E-02	2.84E-02	3.79E-02	TRUE	FALSE	8	119128423	6.65E-02	7.79E-01	TRUE	8	118116184	5.22E-02	0.93	6874	8.18E-06	TRUE	TRUE	482,730	19.92
Phosphatidylcholine (16:0_22:4) levels	Parkinson's disease	rs192523409	C	T	C	T	2.77E-01	1.36E-01	2.49E-02	9.88E-01	FALSE	FALSE	12	108103432	1.53E-01	4.25E-01	TRUE	12	107709655	5.57E-02	0.98	6874	6.89E-07	TRUE	TRUE	482,730	24.69
Phosphatidylcholine (16:0_22:4) levels	Parkinson's disease	rs2236514	G	C	G	C	-9.20E-02	2.80E-03	6.47E-01	6.35E-01	TRUE	FALSE	9	139572068	2.00E-02	5.10E-02	TRUE	9	136677616	1.81E-02	0.95	6874	4.04E-07	TRUE	TRUE	482,730	25.72
Phosphatidylcholine (16:0_22:4) levels	Parkinson's disease	rs2730612	G	T	G	T	8.15E-02	3.15E-02	5.96E-01	4.03E-01	FALSE	FALSE	7	43911840	1.81E-02	4.09E-02	TRUE	7	43872241	1.74E-02	1.00	6874	2.94E-06	TRUE	TRUE	482,730	21.89
Phosphatidylcholine (16:0_22:4) levels	Parkinson's disease	rs35040911	T	C	T	C	-1.12E-01	-2.88E-02	1.61E-01	8.72E-01	FALSE	FALSE	5	82890292	2.84E-02	5.09E-01	TRUE	5	83594473	2.31E-02	1.00	6874	1.34E-06	TRUE	TRUE	482,730	23.40
Phosphatidylcholine (16:0_22:4) levels	Parkinson's disease	rs3741252	T	C	T	C	1.21E-01	-2.38E-02	1.34E-01	9.32E-01	FALSE	FALSE	11	61511498	4.03E-02	2.56E-01	TRUE	11	61744026	2.51E-02	0.99	6874	1.53E-06	TRUE	TRUE	482,730	23.16
Phosphatidylcholine (16:0_22:4) levels	Parkinson's disease	rs4686798	T	C	T	C	-8.59E-02	9.30E-03	3.58E-01	6.07E-01	FALSE	FALSE	3	186445436	1.74E-02	2.28E-01	TRUE	3	186727647	1.75E-02	1.00	6874	9.65E-07	TRUE	TRUE	482,730	24.04
Phosphatidylcholine (16:0_22:4) levels	Parkinson's disease	rs4693564	C	A	C	A	1.15E-01	6.40E-03	8.31E-01	1.37E-01	FALSE	FALSE	4	84031238	3.43E-02	6.99E-02	TRUE	4	83110085	2.35E-02	0.93	6874	9.19E-07	TRUE	TRUE	482,730	24.13
Phosphatidylcholine (16:0_22:4) levels	Parkinson's disease	rs4856561	A	C	A	C	2.12E-01	-3.35E-02	4.67E-02	9.55E-01	FALSE	FALSE	3	85147735	5.25E-02	2.81E-01	TRUE	3	85098584	4.02E-02	1.00	6874	1.46E-07	TRUE	TRUE	482,730	27.70
Phosphatidylcholine (16:0_22:4) levels	Parkinson's disease	rs61008604	T	G	T	G	-1.16E-01	-1.83E-02	2.49E-01	7.27E-01	FALSE	FALSE	2	102268793	2.51E-02	3.32E-01	TRUE	2	101652331	1.98E-02	0.98	6874	5.24E-09	TRUE	TRUE	482,730	34.18
Phosphatidylcholine (16:0_22:4) levels	Parkinson's disease	rs61979278	C	T	C	T	-1.31E-01	3.34E-02	9.60E-02	9.30E-01	FALSE	FALSE	14	99299724	4.69E-02	3.22E-01	TRUE	14	98833387	2.93E-02	0.98	6874	7.79E-06	TRUE	TRUE	482,730	20.02
Phosphatidylcholine (16:0_22:4) levels	Parkinson's disease	rs6713301	T	C	T	C	4.21E-01	3.10E-02	1.00E-02	9.66E-01	FALSE	FALSE	2	30585638	6.33E-02	2.04E-01	TRUE	2	30362772	8.68E-02	0.97	6874	1.25E-06	TRUE	TRUE	482,730	23.53
Phosphatidylcholine (16:0_22:4) levels	Parkinson's disease	rs7007401	C	T	C	T	-8.86E-02	-4.20E-03	2.82E-01	8.00E-01	FALSE	FALSE	8	85138070	2.78E-02	5.56E-02	TRUE	8	84225835	1.89E-02	1.00	6874	2.95E-06	TRUE	TRUE	482,730	21.89
Phosphatidylcholine (16:0_22:4) levels	Parkinson's disease	rs72807394	T	C	T	C	1.11E-01	-1.77E-02	1.36E-01	8.46E-01	FALSE	FALSE	17	9927490	3.13E-02	2.43E-01	TRUE	17	10024173	2.46E-02	0.99	6874	6.60E-06	TRUE	TRUE	482,730	20.34
Phosphatidylcholine (16:0_22:4) levels	Parkinson's disease	rs75679298	T	C	T	C	4.23E-01	2.46E-01	8.91E-03	9.92E-01	FALSE	FALSE	1	102938389	2.02E-01	6.52E-01	TRUE	1	102472833	9.52E-02	0.92	6874	9.08E-06	TRUE	TRUE	482,730	19.73
Phosphatidylcholine (16:0_22:4) levels	Parkinson's disease	rs77730345	A	G	A	G	2.13E-01	6.32E-02	3.86E-02	9.80E-01	FALSE	FALSE	16	2402550	8.45E-02	3.43E-01	TRUE	16	2352549	4.52E-02	0.95	6874	2.53E-06	TRUE	TRUE	482,730	22.18
Phosphatidylcholine (16:0_22:5) levels	Parkinson's disease	rs11072257	C	T	C	T	-1.20E-01	6.17E-02	1.11E-01	9.27E-01	FALSE	FALSE	15	71463600	4.54E-02	7.60E-01	TRUE	15	71171261	2.70E-02	0.98	7172	8.46E-06	TRUE	TRUE	482,730	19.86
Phosphatidylcholine (16:0_22:5) levels	Parkinson's disease	rs1242492	C	T	C	T	-7.48E-02	-2.29E-02	5.01E-01	4.93E-01	FALSE	FALSE	17	17374391	1.93E-02	6.27E-01	TRUE	17	17471077	1.68E-02	0.98	7172	8.58E-06	TRUE	TRUE	482,730	19.83
Phosphatidylcholine (16:0_22:5) levels	Parkinson's disease	rs12598390	G	A	G	A	7.87E-02	-2.20E-02	3.55E-01	6.43E-01	FALSE	FALSE	16	54174285	2.33E-02	4.62E-01	TRUE	16	54140373	1.74E-02	1.00	7172	5.89E-06	TRUE	TRUE	482,730	20.55
Phosphatidylcholine (16:0_22:5) levels	Parkinson's disease	rs1260326	C	T	C	T	-9.04E-02	-6.78E-02	6.51E-01	4.20E-01	FALSE	FALSE	2	27730940	1.72E-02	4.04E-05	TRUE	2	27508073	1.74E-02	1.00	7172	2.20E-07	TRUE	TRUE	482,730	26.90
Phosphatidylcholine (16:0_22:5) levels	Parkinson's disease	rs139606388	T	C	T	C	-2.21E-01	5.00E-02	2.91E-02	9.81E-01	FALSE	FALSE	5	134186864	6.87E-02	3.31E-01	TRUE	5	134851174	4.91E-02	0.99	7172	6.78E-06	TRUE	TRUE	482,730	20.28
Phosphatidylcholine (16:0_22:5) levels	Parkinson's disease	rs147057045	T	C	T	C	-3.41E-01	-6.00E-04	1.53E-02	9.87E-01	FALSE	FALSE	1	35398150	1.81E-01	1.22E-03	TRUE	1	34932549	7.25E-02	0.89	7172	2.63E-06	TRUE	TRUE	482,730	22.10
Phosphatidylcholine (16:0_22:5) levels	Parkinson's disease	rs174533	A	G	A	G	-4.13E-01	-9.00E-04	4.09E-01	6.69E-01	FALSE	FALSE	11	61549025	1.79E-02	1.84E-02	TRUE	11	61781553	1.63E-02	1.00	7172	5.08E-135	TRUE	TRUE	482,730	638.42
Phosphatidylcholine (16:0_22:5) levels	Parkinson's disease	rs1800574	T	C	T	C	2.08E-01	-8.60E-03	4.57E-02	9.69E-01	FALSE	FALSE	12	121416864	4.97E-02	6.40E-02	TRUE	12	120979061	3.97E-02	1.00	7172	1.51E-07	TRUE	TRUE	482,730	27.63
Phosphatidylcholine (16:0_22:5) levels	Parkinson's disease	rs2236514	G	C	G	C	-1.23E-01	2.80E-03	6.47E-01	6.35E-01	TRUE	FALSE	9	139572068	2.00E-02	5.10E-02	TRUE	9	136677616	1.77E-02	0.95	7172	4.04E-12	TRUE	TRUE	482,730	48.26
Phosphatidylcholine (16:0_22:5) levels	Parkinson's disease	rs2613167	T	G	T	G	8.08E-02	-7.59E-02	3.73E-01	6.47E-01	FALSE	FALSE	4	30162762	2.84E-02	3.76E-03	TRUE	4	30161140	1.77E-02	0.97	7172	4.83E-06	TRUE	TRUE	482,730	20.93
Phosphatidylcholine (16:0_22:5) levels	Parkinson's disease	rs3009985	A	G	A	G	8.33E-02	2.45E-02	7.16E-01	3.18E-01	FALSE	FALSE	1	212191991	1.93E-02	6.93E-01	TRUE	1	212018649	1.85E-02	1.00	7172	6.56E-06	TRUE	TRUE	482,730	20.35
Phosphatidylcholine (16:0_22:5) levels	Parkinson's disease	rs3019200	A	C	A	C	-1.09E-01	-3.10E-03	8.03E-01	1.90E-01	FALSE	FALSE	11	61249383	2.40E-02	4.77E-02	TRUE	11	61481911	2.14E-02	1.00	7172	3.77E-07	TRUE	TRUE	482,730	25.85
Phosphatidylcholine (16:0_22:5) levels	Parkinson's disease	rs3096698	C	T	C	T	-8.36E-02	-2.36E-02	6.31E-01	3.22E-01	FALSE	FALSE	6	32107139	2.04E-02	6.06E-01	TRUE	6	32139362	1.73E-02	0.99	7172	1.39E-06	TRUE	TRUE	482,730	23.34
Phosphatidylcholine (16:0_22:5) levels	Parkinson's disease	rs4016738	C	G	C	G	-9.35E-02	2.35E-02	2.06E-01	2.15E-01	TRUE	FALSE	3	39292247	2.13E-02	5.68E-01	TRUE	3	39250756	2.08E-02	1.00	7172	7.04E-06	TRUE	TRUE	482,730	20.21
Phosphatidylcholine (16:0_22:5) levels	Parkinson's disease	rs508049	T	C	T	C	-1.67E-01	1.46E-02	9.80E-02	9.44E-01	FALSE	FALSE	11	68675497	4.30E-02	1.34E-01	TRUE	11	68908029	2.86E-02	0.96	7172	5.62E-09	TRUE	TRUE	482,730	34.05
Phosphatidylcholine (16:0_22:5) levels	Parkinson's disease	rs58967664	C	T	C	T	1.50E-01	2.51E-02	6.89E-02	9.49E-01	FALSE	FALSE	8	95005265	5.45E-02	1.91E-01	TRUE	8	93993037	3.38E-02	0.94	7172	9.44E-06	TRUE	TRUE	482,730	19.65
Phosphatidylcholine (16:0_22:5) levels	Parkinson's disease	rs61653779	C	T	C	T	2.76E-01	-5.22E-02	2.01E-02	9.74E-01	FALSE	FALSE	16	20580226	5.54E-02	4.61E-01	TRUE	16	20568904	6.02E-02	1.00	7172	4.69E-06	TRUE	TRUE	482,730	20.99
Phosphatidylcholine (16:0_22:5) levels	Parkinson's disease	rs62142428	A	C	A	C	2.36E-01	-9.50E-03	3.11E-02	9.51E-01	FALSE	FALSE	2	35331844	5.52E-02	6.39E-02	TRUE	2	35106778	4.94E-02	0.95	7172	1.77E-06	TRUE	TRUE	482,730	22.87
Phosphatidylcholine (16:0_22:5) levels	Parkinson's disease	rs62280086	A	G	A	G	1.03E-01	1.73E-02	1.78E-01	7.89E-01	FALSE	FALSE	4	7384207	2.98E-02	2.51E-01	TRUE	4	7382480	2.27E-02	0.92	7172	5.38E-06	TRUE	TRUE	482,730	20.73
Phosphatidylcholine (16:0_22:5) levels	Parkinson's disease	rs73019765	C	T	C	T	1.12E-01	-1.10E-03	1.42E-01	8.46E-01	FALSE	FALSE	6	161329691	3.05E-02	1.24E-02	TRUE	6	160908659	2.37E-02	1.00	7172	2.31E-06	TRUE	TRUE	482,730	22.35
Phosphatidylcholine (16:0_22:5) levels	Parkinson's disease	rs73225965	A	G	A	G	1.17E-01	5.49E-02	1.21E-01	8.84E-01	FALSE	FALSE	8	25833371	3.65E-02	8.77E-01	TRUE	8	25975855	2.60E-02	0.96	7172	6.97E-06	TRUE	TRUE	482,730	20.23
Phosphatidylcholine (16:0_22:5) levels	Parkinson's disease	rs7805803	A	G	A	G	8.07E-02	-4.10E-03	4.02E-01	5.30E-01	FALSE	FALSE	7	50225391	1.69E-02	9.19E-02	TRUE	7	50185795	1.71E-02	1.00	7172	2.33E-06	TRUE	TRUE	482,730	22.34

Phosphatidylcholine (16:0_22:5) levels	Parkinson's disease	rs7936002	G	A	G	A	8.90E-02	-5.27E-02	2.57E-01	7.70E-01	FALSE	FALSE	FALSE	11	62219029	3.25E-02	9.78E-01	TRUE	11	62451557	1.97E-02	0.93	7172	6.17E-06	TRUE	TRUE	482,730	20.46
Phosphatidylcholine (16:0_22:5) levels	Parkinson's disease	rs80197029	T	G	T	G	3.92E-01	1.94E-01	1.10E-02	9.91E-01	FALSE	FALSE	FALSE	12	78640990	1.82E-01	5.43E-01	TRUE	12	78247210	8.14E-02	0.93	7172	1.48E-06	TRUE	TRUE	482,730	23.21
Phosphatidylcholine (16:0_22:5) levels	Parkinson's disease	rs9918362	C	T	C	T	8.46E-02	5.00E-03	4.58E-01	5.62E-01	FALSE	FALSE	FALSE	6	10999650	1.69E-02	1.15E-01	TRUE	6	10999417	1.68E-02	1.00	7172	5.09E-07	TRUE	TRUE	482,730	25.27
Phosphatidylcholine (16:0_22:6) levels	Parkinson's disease	rs10150075	T	C	T	C	-1.91E-01	9.26E-02	5.30E-02	9.00E-01	FALSE	FALSE	FALSE	14	73038517	3.80E-02	7.41E-03	TRUE	14	72571809	3.82E-02	0.95	7170	5.88E-07	TRUE	TRUE	482,730	24.99
Phosphatidylcholine (16:0_22:6) levels	Parkinson's disease	rs10498720	C	A	C	A	2.62E-01	-3.68E-02	2.94E-02	9.68E-01	FALSE	FALSE	FALSE	6	24353436	6.00E-02	2.68E-01	TRUE	6	24353208	5.03E-02	0.98	7170	2.01E-07	TRUE	TRUE	482,730	27.07
Phosphatidylcholine (16:0_22:6) levels	Parkinson's disease	rs117061362	T	G	T	G	2.15E-01	1.04E-01	4.49E-02	9.81E-01	FALSE	FALSE	FALSE	15	101736552	1.03E-01	5.07E-01	TRUE	15	101196347	4.11E-02	0.93	7170	1.81E-07	TRUE	TRUE	482,730	27.28
Phosphatidylcholine (16:0_22:6) levels	Parkinson's disease	rs117185962	C	A	C	A	-2.68E-01	-5.52E-02	2.22E-02	9.67E-01	FALSE	FALSE	FALSE	10	127746455	6.56E-02	3.98E-01	TRUE	10	126057886	5.91E-02	0.94	7170	5.62E-06	TRUE	TRUE	482,730	20.64
Phosphatidylcholine (16:0_22:6) levels	Parkinson's disease	rs117398583	A	G	A	G	-2.43E-01	-1.37E-01	3.17E-02	9.83E-01	FALSE	FALSE	FALSE	10	126894268	9.90E-02	7.75E-01	TRUE	10	125205699	5.09E-02	0.89	7170	1.88E-06	TRUE	TRUE	482,730	22.75
Phosphatidylcholine (16:0_22:6) levels	Parkinson's disease	rs143699848	C	T	C	T	-3.40E-01	-2.51E-02	1.29E-02	9.82E-01	FALSE	FALSE	FALSE	5	82280532	9.28E-02	1.04E-01	TRUE	5	82984713	7.45E-02	0.97	7170	5.15E-06	TRUE	TRUE	482,730	20.81
Phosphatidylcholine (16:0_22:6) levels	Parkinson's disease	rs17173596	A	G	A	G	8.22E-02	-2.92E-02	3.04E-01	6.39E-01	FALSE	FALSE	FALSE	7	150477384	1.90E-02	9.04E-01	TRUE	7	150780296	1.83E-02	1.00	7170	6.93E-06	TRUE	TRUE	482,730	20.24
Phosphatidylcholine (16:0_22:6) levels	Parkinson's disease	rs174544	A	C	A	C	-1.27E-01	-9.00E-04	3.80E-01	7.09E-01	FALSE	FALSE	FALSE	11	61567753	1.85E-02	1.67E-02	TRUE	11	61800281	1.73E-02	1.00	7170	2.30E-13	TRUE	TRUE	482,730	53.93
Phosphatidylcholine (16:0_22:6) levels	Parkinson's disease	rs1935135	C	T	C	T	8.26E-02	4.15E-02	2.87E-01	6.79E-01	FALSE	FALSE	FALSE	13	108328177	2.58E-02	9.69E-01	TRUE	13	107675829	1.83E-02	1.00	7170	6.42E-06	TRUE	TRUE	482,730	20.39
Phosphatidylcholine (16:0_22:6) levels	Parkinson's disease	rs2075083	T	G	T	G	8.11E-02	1.10E-02	5.81E-01	5.06E-01	FALSE	FALSE	FALSE	7	17719956	2.61E-02	1.72E-01	TRUE	7	17680332	1.68E-02	0.99	7170	1.42E-06	TRUE	TRUE	482,730	23.29
Phosphatidylcholine (16:0_22:6) levels	Parkinson's disease	rs3775850	A	G	A	G	-9.57E-02	6.07E-02	2.04E-01	8.24E-01	FALSE	FALSE	FALSE	4	120451018	2.38E-02	5.38E-03	TRUE	4	119529863	2.08E-02	1.00	7170	4.12E-06	TRUE	TRUE	482,730	21.24
Phosphatidylcholine (16:0_22:6) levels	Parkinson's disease	rs4937784	T	C	T	C	-1.19E-01	-4.60E-02	1.36E-01	8.36E-01	FALSE	FALSE	FALSE	11	133371608	3.08E-02	8.68E-01	TRUE	11	133501713	2.48E-02	0.95	7170	1.56E-06	TRUE	TRUE	482,730	23.12
Phosphatidylcholine (16:0_22:6) levels	Parkinson's disease	rs57634784	T	C	T	C	-8.74E-02	1.99E-02	3.31E-01	6.15E-01	FALSE	FALSE	FALSE	2	159095326	1.91E-02	5.28E-01	TRUE	2	158238814	1.76E-02	0.99	7170	6.93E-07	TRUE	TRUE	482,730	24.68
Phosphatidylcholine (16:0_22:6) levels	Parkinson's disease	rs61900787	C	T	C	T	7.86E-02	-2.50E-02	4.15E-01	6.86E-01	FALSE	FALSE	FALSE	11	42676239	2.40E-02	5.26E-01	TRUE	11	42654689	1.69E-02	1.00	7170	3.34E-06	TRUE	TRUE	482,730	21.65
Phosphatidylcholine (16:0_22:6) levels	Parkinson's disease	rs6864719	G	A	G	A	-1.71E-01	-3.59E-02	9.49E-01	9.97E-02	FALSE	FALSE	FALSE	5	53945116	3.78E-02	4.66E-01	TRUE	5	54649287	3.82E-02	0.99	7170	8.12E-06	TRUE	TRUE	482,730	19.94
Phosphatidylcholine (16:0_22:6) levels	Parkinson's disease	rs73188528	A	G	A	G	9.83E-02	2.72E-02	2.00E-01	8.02E-01	FALSE	FALSE	FALSE	7	106036159	3.13E-02	4.15E-01	TRUE	7	106395713	2.20E-02	0.91	7170	8.35E-06	TRUE	TRUE	482,730	19.88
Phosphatidylcholine (16:0_22:6) levels	Parkinson's disease	rs74883996	T	C	T	C	3.82E-01	1.44E-01	1.11E-02	9.86E-01	FALSE	FALSE	FALSE	16	24985091	1.10E-01	7.15E-01	TRUE	16	24973770	8.42E-02	0.89	7170	5.82E-06	TRUE	TRUE	482,730	20.58
Phosphatidylcholine (16:0_22:6) levels	Parkinson's disease	rs76218130	C	A	C	A	2.49E-01	-5.80E-02	2.45E-02	9.49E-01	FALSE	FALSE	FALSE	4	11831038	5.33E-02	5.57E-01	TRUE	4	11829414	5.58E-02	0.92	7170	8.52E-06	TRUE	TRUE	482,730	19.85
Phosphatidylcholine (16:0_22:6) levels	Parkinson's disease	rs77304558	C	T	C	T	-9.15E-01	2.31E-01	2.12E-03	9.82E-01	FALSE	FALSE	FALSE	5	152497971	9.20E-02	6.00E-03	TRUE	5	153118411	1.96E-01	0.93	7170	3.14E-06	TRUE	TRUE	482,730	21.76
Phosphatidylcholine (16:0_22:6) levels	Parkinson's disease	rs77456635	A	G	A	G	-2.39E-01	1.32E-02	2.42E-02	9.69E-01	FALSE	FALSE	FALSE	15	65158047	5.11E-02	9.87E-02	TRUE	15	64865848	5.36E-02	1.00	7170	8.62E-06	TRUE	TRUE	482,730	19.82
Phosphatidylcholine (16:0_22:6) levels	Parkinson's disease	rs79508872	A	C	A	C	-2.76E-01	-3.69E-02	2.02E-02	9.55E-01	FALSE	FALSE	FALSE	14	78826629	5.59E-02	2.94E-01	TRUE	14	78360286	5.98E-02	0.98	7170	4.14E-06	TRUE	TRUE	482,730	21.23
Phosphatidylcholine (16:0_22:6) levels	Parkinson's disease	rs964184	C	G	C	G	-1.22E-01	-5.68E-02	8.49E-01	8.63E-01	TRUE	FALSE	FALSE	11	116648917	2.43E-02	9.71E-03	TRUE	11	116778201	2.32E-02	1.00	7170	1.38E-07	TRUE	TRUE	482,730	27.80
Phosphatidylcholine (16:1_18:0) levels	Parkinson's disease	rs10520096	C	G	C	G	1.51E-01	-3.46E-02	7.45E-02	1.16E-01	TRUE	FALSE	FALSE	15	38904154	2.98E-02	6.10E-01	TRUE	15	38611953	3.33E-02	0.98	6524	5.82E-06	TRUE	TRUE	482,730	20.58
Phosphatidylcholine (16:1_18:0) levels	Parkinson's disease	rs11112689	C	G	C	G	-8.81E-02	1.46E-02	2.73E-01	2.85E-01	TRUE	FALSE	FALSE	12	106101254	2.45E-02	2.58E-01	TRUE	12	105707476	1.94E-02	1.00	6524	5.84E-06	TRUE	TRUE	482,730	20.57
Phosphatidylcholine (16:1_18:0) levels	Parkinson's disease	rs114728714	A	G	A	G	2.04E-01	1.92E-02	4.13E-02	9.77E-01	FALSE	FALSE	FALSE	2	173045072	8.72E-02	8.32E-02	TRUE	2	172180344	4.54E-02	0.94	6524	6.82E-06	TRUE	TRUE	482,730	20.27
Phosphatidylcholine (16:1_18:0) levels	Parkinson's disease	rs1260326	C	T	C	T	-1.08E-01	-6.78E-02	6.51E-01	4.20E-01	FALSE	FALSE	FALSE	2	27730940	1.72E-02	4.04E-05	TRUE	2	27508073	1.83E-02	1.00	6524	3.75E-09	TRUE	TRUE	482,730	34.84
Phosphatidylcholine (16:1_18:0) levels	Parkinson's disease	rs145028924	G	A	G	A	-4.49E-01	7.76E-02	8.90E-03	9.79E-01	FALSE	FALSE	FALSE	2	15372879	9.48E-02	3.84E-01	TRUE	2	15232755	9.99E-02	0.85	6524	6.87E-06	TRUE	TRUE	482,730	20.26
Phosphatidylcholine (16:1_18:0) levels	Parkinson's disease	rs1533362	A	C	A	C	1.14E-01	1.30E-02	1.45E-01	8.73E-01	FALSE	FALSE	FALSE	4	17073759	3.34E-02	1.57E-01	TRUE	4	17072136	2.46E-02	1.00	6524	3.89E-06	TRUE	TRUE	482,730	21.36
Phosphatidylcholine (16:1_18:0) levels	Parkinson's disease	rs174559	A	G	A	G	9.52E-02	-1.84E-02	3.14E-01	7.39E-01	FALSE	FALSE	FALSE	11	61581656	2.00E-02	4.47E-01	TRUE	11	61814184	1.90E-02	1.00	6524	5.39E-07	TRUE	TRUE	482,730	25.17
Phosphatidylcholine (16:1_18:0) levels	Parkinson's disease	rs28807772	G	A	G	A	1.01E-01	-2.01E-02	2.02E-01	7.80E-01	FALSE	FALSE	FALSE	5	165249121	2.24E-02	4.31E-01	TRUE	5	165822116	2.18E-02	1.00	6524	3.33E-06	TRUE	TRUE	482,730	21.65
Phosphatidylcholine (16:1_18:0) levels	Parkinson's disease	rs4332435	T	A	T	A	1.86E-01	2.40E-03	9.51E-01	9.39E-01	TRUE	FALSE	FALSE	10	129859458	3.90E-02	2.16E-02	TRUE	10	128061194	4.08E-02	0.98	6524	5.56E-06	TRUE	TRUE	482,730	20.67
Phosphatidylcholine (16:1_18:0) levels	Parkinson's disease	rs62239496	G	A	G	A	-9.80E-02	-3.60E-03	2.01E-01	7.18E-01	FALSE	FALSE	FALSE	3	37492545	2.19E-02	6.11E-02	TRUE	3	37451054	2.20E-02	0.99	6524	8.56E-06	TRUE	TRUE	482,730	19.84
Phosphatidylcholine (16:1_18:0) levels	Parkinson's disease	rs76712623	A	G	A	G	-4.16E-01	2.94E-02	1.09E-02	9.83E-01	FALSE	FALSE	FALSE	4	132111061	8.83E-02	1.31E-01	TRUE	4	131189906	8.38E-02	0.98	6524	6.84E-07	TRUE	TRUE	482,730	24.71
Phosphatidylcholine (16:1_18:0) levels	Parkinson's disease	rs77007318	C	G	C	G	-2.79E-01	2.65E-02	2.89E-02	2.25E-02	TRUE	FALSE	FALSE	16	59890172	8.92E-02	1.16E-01	TRUE	16	59856268	5.45E-02	0.89	6524	3.19E-07	TRUE	TRUE	482,730	26.18
Phosphatidylcholine (16:1_18:0) levels	Parkinson's disease	rs78275150	C	T	C	T	2.80E-01	-2.22E-01	2.01E-02	9.81E-01	FALSE	FALSE	FALSE	7	136964955	8.36E-02	4.00E-03	TRUE	7	137280208	6.24E-02	0.97	6524	7.25E-06	TRUE	TRUE	482,730	20.16
Phosphatidylcholine (16:1_18:0) levels	Parkinson's disease	rs7991657	T	C	T	C	1.57E-01	3.02E-02	9.29E-01	6.36E-02	FALSE	FALSE	FALSE	13	37946997	4.67E-02	2.86E-01	TRUE	13	37372860	3.46E-02	1.00	6524	5.95E-06	TRUE	TRUE	482,730	20.54
Phosphatidylcholine (16:1_18:0) levels	Parkinson's disease	rs9934078	C	A	C	A	-9.53E-02	1.10E-02	2.71E-01	7.10E-01	FALSE	FALSE	FALSE	16	78512523	2.53E-02	1.78E-01	TRUE	16	78478626	1.99E-02	0.99	6524	1.74E-06	TRUE	TRUE	482,730	22.90
Phosphatidylcholine (16:1_18:1) levels	Parkinson's disease	rs11189347	A	G	A	G	-1.16E-01	3.84E-02	1.33E-01	8.51E-01	FALSE	FALSE	FALSE	10	83076339	3.22E-02	6.32E-01	TRUE	10	81316583	2.48E-02	0.98	7166	2.57E-06	TRUE	TRUE	482,730	22.15
Phosphatidylcholine (16:1_18:1) levels	Parkinson's disease	rs113366516	T	G	T	G	3.11E-01	-9.44E-02	1.70E-02	9.91E-01	FALSE	FALSE	FALSE	6	138469237	1.90E-01	2.08E-01	TRUE	6	138148100	6.61E-02	0.93	7166	2.65E-06	TRUE	TRUE	482,730	22.09
Phosphatidylcholine (16:1_18:1) levels	Parkinson's disease	rs116997061	T	C	T	C	-1.68E-01	8.14E-02	6.19E-02	9.50E-01	FALSE	FALSE	FALSE	12	20022049	5.65E-02	8.25E-01	TRUE	12	19869115	3.60E-02	0.91	7166	3.00E-06	TRUE	TRUE	482,730	21.85
Phosphatidylcholine (16:1_18:1) levels	Parkinson's disease	rs117217513	T	G	T	G	-2.31E-01	5.61E-02	2.78E-02	9.86E-01	FALSE	FALSE	FALSE	16	6582849	2.09E-01	1.03E-01	TRUE	16	6532848	5.09E-02	0.96	7166	5.76E-06	TRUE	TRUE	482,730	20.60
Phosphatidylcholine (16:1_18:1) levels	Parkinson's disease	rs1260326	C	T	C	T	-1.10E-01	-6.78E-02	6.51E-01	4.20E-01	FALSE	FALSE	FALSE	2	27730940	1.72E-02	4.04E-05	TRUE	2	27508073	1.75E-02	1.00	7166	2.95E-10	TRUE	TRUE	482,730	39.81
Phosphatidylcholine (16:1_18:1) levels	Parkinson's disease	rs13402814	T	A	T	A	1.69E-01	9.00E-04	5.97E-02	7.48E-02	TRUE	FALSE	FALSE	2	137944011	3.30E-02	9.22E-03	TRUE	2	137186441	3.52E-02	1.00	7166	1.68E-06	TRUE	TRUE	482,730	22.97
Phosphatidylcholine (16:1_18:1) levels	Parkinson's disease	rs139935251	C	T	C	T	-7.23E-01	3.48E-01	2.95E-03	9.91E-01	FALSE	FALSE	FALSE	11	30092150	1.99E-01	4.06E-02	TRUE	11	30070603	1.58E-01	0.93	7166	4.78E-06	TRUE	TRUE	482,730	20.95
Phosphatidylcholine (16:1_18:1) levels	Parkinson's disease	rs141084476	T	G	T	G	1.88E-01	-3.58E-02	4.32E-02	9.73E-01	FALSE	FALSE	FALSE	8	5044649	2.04E-01	6.51E-02	TRUE	8	5187127	4.15E-02	0.97	7166	6.02E-06	TRUE	TRUE	482,730	20.51
Phosphatidylcholine (16:1_18:1) levels	Parkinson's disease	rs145551604	T	C	T	C	1.55E-01	2.48E-02	7.33E-02	9.46E-01	FALSE	FALSE	FALSE	7	106880820	5.37E-02	1.91E-01	TRUE	7	107240375	3.25E-02	0.97	7166	2.03E-06	TRUE	TRUE	482,730	22.60
Phosphatidylcholine (16:1_18:1) levels	Parkinson's disease	rs174560	C	T	C	T	1.76E-01	-1.90E-03	3.83E-01	7.01E-01	FALSE	FALSE	FALSE	11	61581764	1.84E-02	3.82E-02	TRUE	11	61814292	1.72E-02	1.00	7166	2.28E-24	TRUE	TRUE	482,730	104.52
Phosphatidylcholine (16:1_18:1) levels	Parkinson's disease	rs185867215	A	C	A	C	5.65E-01	-2.52E-01	5.30E-03	9.94E-01	FALSE	FALSE	FALSE	17	18872625	3.56E-01	3.19E-01	TRUE	17	18969312	1.24E-01	0.87	7166	5.40E-06	TRUE	TRUE	482,730	20.72
Phosphatidylcholine (16:1_18:1) levels	Parkinson's disease	rs4447106	A	G	A	G	1.28E-01	2.09E-02	1.41E-01	8.83E-01	FALSE	FALSE	FALSE	10	102359760	2.92E-02	3.23E-01	TRUE	10	100600003	2.42E-02	0.98	7166	1.29E-07	TRUE	TRUE	482,730	27.93
Phosphatidylcholine (16:1_18:1) levels	Parkinson's disease	rs56156922	C	T	C	T	1.01E-01	1.46E-02	2.79E-01	6.86E-01	FALSE	FALSE	FALSE	16	56987369	1.82E-02	3.75E-01	TRUE	16	56953457	1.86E-02	1.00	7166	5.95E-08	TRUE	TRUE	482,730	29.44
Phosphatidylcholine (16:1_18:1) levels	Parkinson's disease	rs56316064	G	T	G	T	-9.02E-02	-1.40E-03	2.92E-01	7.04E-01	FALSE	FALSE	FALSE	3	187112322	2.25E-02	2.23E-02	TRUE	3	187394534	1.88E-02	0.95	7166	1.58E-06	TRUE	TRUE	482,730	23.09
Phosphatidylcholine (16:1_18:1) levels	Parkinson's disease	rs5865	T	C	T	C	8.73E-02	7.80E-03	7.12E-01	3.47E-01	FALSE	FALSE	FALSE	2	98373006	1.93E-02	1.64E-01	TRUE	2	97756543	1.84E-02	1.00	7166	2.13E-06	TRUE	TRUE	482,730	22.51
Phosphatidylcholine (16:1_18:1) levels	Parkinson's disease	rs603424	A	G	A	G	-1.76E-01	6.90E-03	1.21E-01	8.02E-01	FALSE	FALSE	FALSE	10	102075479	2.49E-02	1.07E-01	TRUE	10	100315722	2.60E-02	1.00	7166	1.21E-11	TRUE	TRUE	482,730	46.10
Phosphatidylcholine (16:1_18:1) levels	Parkinson's disease	rs7007401	C	T	C	T	-8.22E-02	-4.20E-03	2.82E-01	8.00E-01	FALSE	FALSE	FALSE	8	85138070	2.78E-02	5.56E-02	TRUE	8	84225835	1.85E-02	1.00	7166	9.16E-06	TRUE	TRUE	482,730	19.70
Phosphatidylcholine (16:1_18:1) levels	Parkinson's disease	rs701919	C	G	C	G	-9.36E-02	-9.30E-03	7.90E-01	6.75E-01	TRUE	FALSE	FALSE	1	211860366	2.07E-02	1.86E-01	TRUE	1	211687024	2.05E-02	0.99	7166	5.04E-06	TRUE	TRUE	482,730	20.85
Phosphatidylcholine (16:1_18:1) levels	Parkinson's disease	rs74357965	C	A	C	A	-1.26E-01	1.90E-03	1.02E-01	8.97E-01	FALSE	FALSE	FALSE	5	20149675	4.04E-02	1.68E-02	TRUE	5	20149566	2.81E-02	0.96	7166	7.10E-06	TRUE	TRUE	482,730	20.19
Phosphatidylcholine (16:1_18:1) levels	Parkinson's disease	rs75102979	A	G	A	G	1.83E-01	-4.88E-02	4.75E-02	9.46E-01	FALSE	FALSE	FALSE	7	80833808	6.81E-02	3.25E-01	TRUE	7	81204492	4.11E-02	0.90	7166	8.63E-06	TRUE	TRUE	482,730	19.82
Phosphatidylcholine (16:1_18:1) levels	Parkinson's disease	rs7968329	G	A	G	A	-7.55E-02	4.59E-02	4.40E-01	5.92E-01	FALSE	FALSE	FALSE	12	5105339	2.27E-02	2.16E-02	TRUE	12	4996173	1.67E-02	1.00	7166	6.45E-06	TRUE	TRUE	482,730	20.38
Phosphatidylcholine (16:1_18:1) levels	Parkinson's disease	rs80160469	A	G	A	G	-2.25E-01	2.96E-02	3.21E-02	9.61E-01	FALSE	FALSE	FALSE	2	170011405	5.25E-02	2.42E-01	TRUE	2	169154895	4.94E-02	0.90	7166	5.50E-06	TRUE	TRUE	482,730	20.68
Phosphatidylcholine (16:1_18:2) levels	Parkinson's disease	rs10201921	A	G	A	G	1.57E-01	-1.20E-03	5.96E-02	9.26E-01	FALSE	FALSE	FALSE	2	137916795	3.32E-02	1.27E-02	TRUE	2	137159225	3.51E-02	1.00	7165	8.15E-06	TRUE	TRUE	482,730	19.93
Phosphatidylcholine (16:1_18:2) levels	Parkinson's disease	rs10890773	A	G	A	G	-1.09E-01	5.70E-02	8.60E-01	1.45E-01	FALSE	FALSE	FALSE	11	107760002	3.20E-02	3.74E-02	TRUE	11	107889276	2.38E-02	1.00	7165	4.82E-06	TRUE	TRUE	482,730	20.94
Phosphatidylcholine (16:1_18:2) levels	Parkinson's disease	rs11602429	G	A	G	A	1.12E-01	-2.75E-02	1.32E-01	8.69E-01	FALSE	FALSE	FALSE	11	92131209	3.30E-02	3.94E-01	TRUE	11	92398043	2.47E-02	0.99	7165	5.49E-06	TRUE	TRUE	482,730	20.69
Phosphatidylcholine (16:1_18:2) levels	Parkinson's disease	rs12031139	G	T	G	T	7.45E-02	-1.25E-02	4.89E-01	5.18E-01	FALSE	FALSE	FALSE	1	213841635	2.29E-02	2.33E-01	TRUE	1	213668292	1.67E-02	0.99	7165	8.07E-06	TRUE	TRUE	482,730	19.95
Phosphatidylcholine (16:1_18:2) levels	Parkinson's disease	rs1260326	C	T	C	T	-9.85E-02	-6.78E-02	6.51E-01	4.20E-01	FALSE	FALSE	FALSE	2	27730940	1.72E-02	4.04E-05	TRUE	2	27508073	1.74E-02	1.00	7165	1.66E-08	TRUE	TRUE	482,730	31.92
Phosphatidylcholine (16:1_18:2) levels	Parkinson's disease	rs13150924	A	G	A	G	-1.21E-01	-1.06E-02	1.30E-01	8.24E-01	FALSE	FALSE	FALSE	4	170198810	2.99E-02	1.41E-01	TRUE	4	169277659	2.50E-02	0.98	7165	1.37E-06	TRUE	TRUE	482,730	23.36
Phosphatidylcholine (16:1_18:2) levels	Parkinson's disease	rs174574	C	A	C	A	-3.66E-01	6.60E-03	5.91E-01	3.39E-01	FALSE	FALSE	FALSE	11	61600342	1.77E-02	1.48E-01	TRUE	11	61832870	1.65E-02	1.00	7165	2.11E-105	TRUE	TRUE	482,730	491.51
Phosphatidylcholine (16:1_18:2) levels	Parkinson's disease	rs1800588	T	C	T	C	1.16E-01	-3.30E-03	2.58E-01	7.75E-01	FALSE	FALSE	FALSE	15	58723675	2.12E-02	5.73E-02	TRUE	15	58431476	1.93E-02	0.99	7165	2.21E-09	TRUE	TRUE	482,730	35.86
Phosphatidylcholine (16:1_18:2) levels	Parkinson's disease	rs184289628	G	T	G	T	2.84E-01	1.15E-01	1.82E-02	9.90E-01	FALSE	FALSE	FALSE	6	139906689	1.23E-01	4.58E-01	TRUE	6	139585552	6.23E-02	0.97	7165	5.40E-06	TRUE	TRUE	482,730	20.72
Phosphatidylcholine (16:1_18:2) levels	Parkinson's disease	rs216958	G	A	G	A	-7.95E-02	2.24E-02	6.17E-01	3.65E-01	FALSE	FALSE	FALSE	16	60234998	2.34E-02	4.68E-01	TRUE	16	60201094	1.73E-02	0.99	7165	4.47E-06	TRUE	TRUE	482,730	21.08
Phosphatidylcholine (16:1_18:2) levels	Parkinson's disease	rs56156922	C	T	C	T	1.14E-01	1.46E-02	2.79E-01	6.86E-01	FALSE	FALSE	FALSE	16	56987369	1.82E-02	3.75E-01	TRUE	16	56953457	1.85E-02	1.00	7165	8.30E-10	TRUE	TRUE	482,730	37.78
Phosphatidylcholine (16:1_18:2) levels	Parkinson's disease	rs603424	A	G	A	G	-1.61E-01	6.90E-03	1.21E-01	8.02E-01	FALSE	FALSE	FALSE	10	102075479	2.49E-02	1.07E-01	TRUE	10	100315722	2.59E-02	1.00	7165	5.15E-10	TRUE	TRUE	482,730	38.72
Phosphatidylcholine (16:1_18:2) levels	Parkinson's disease	rs6876235	C	T	C	T	1.31E-01	9.54E-02	9.05E-01	5.97E-02	FALSE	FALSE	FALSE	5	85599796	4.66E-02	2.03E-02	TRUE	5	86303978	2.84E-02	1.00	7165	3.88E-06	TRUE	TRUE	482,730	21.36
Phosphatidylcholine (16:1_18:2) levels	Parkinson's disease	rs7036554	C	T	C	T	7.81E-02	-3.60E-03	3.50E-01	6.32E-01	FALSE	FALSE	FALSE	9	14382620	2.36E-02	5.54E-02	TRUE	9	14382621	1.76E-02	0.97	7165	9.12E-06	TRUE	TRUE	482,730	19.71
Phosphatidylcholine (16:1_18:2) levels	Parkinson's disease	rs7783799	G	T	G	T	8.60E-02	8.90E-03	3.08E-01	7.26E-01	FALSE	FALSE	FALSE	7	6573087	2.71E-02	1.29E-01	TRUE	7	6533456	1.83E-02	0.95	7165	2.63E-06	TRUE	TRUE	482,730	22.10
Phosphatidylcholine (16:1_18:2) levels	Parkinson's disease	rs77949981	G	T	G	T	-2.75E-01	-6.40E-03	1.97E-02	9.47E-01	FALSE	FALSE	FALSE	7	10951151	4.60E-02	5.12E-02	TRUE	7	10911524	6.09E-02	0.97	7165	6.23E-06	TRUE	TRUE	482,730	20.44
Phosphatidylcholine (16:1_18:2) levels	Parkinson's disease	rs7936002	G	A	G	A	-1.15E-01	-5.27E-02	2.57E-01	7.70E-01	FALSE	FALSE	FALSE	11	62219029	3.25E-02	9.78E-01	TRUE	11	62451557	1.96E-02	0.93	7165	4.22E-09	TRUE	TRUE	482,730	34.60
Phosphatidylcholine (16:1_18:2) levels	Parkinson's disease	rs9480322	C	T	C	T	-2.20E-01	1.56E-01	3.72E-02	9.76E-01	FALSE	FALSE	FALSE	6	156704765	8.03E-02	2.64E-02	TRUE	6	156383631	4.45E-02	0.98	7165	8.16E-07	TRUE	TRUE	482,730	24.36
Phosphatidylcholine (16:1_18:2) levels	Parkinson's disease	rs9839380	G	A	G	A	9.60E-02	-2.13E-02	3.35E-01	7.05E-01	FALSE	FALSE	FALSE	3	26891616	2.44E-02	4.18E-01	TRUE	3	26850125	1.77E-02	0.99	7165	6.37E-08	TRUE	TRUE	482,730	29.31
Phosphatidylcholine (16:1_20:4) levels	Parkinson's disease	rs10848122	C	G	C	G	-1.10E-01	-2.92E-02	7.78E-01	7.55E-01	TRUE	FALSE	FALSE	12	130992982	2.67E-02	5.63E-01	TRUE	12	130508437	2.33E-02	0.98	5564	2.54E-06	TRUE	TRUE	482,730	22.18
Phosphatidylcholine (16:1_20:4) levels	Parkinson's disease	rs11650558	T	C	T	C	9.19E-02	-2.11E-02	5.37E-01	4.57E-01	FALSE	FALSE	FALSE	17	21301225	3.02E-02	3.14E-01	TRUE	17	21397913	1.91E-02	0.98	5564	1.49E-06	TRUE	TRUE	482,730	23.21
Phosphatidylcholine (16:1_20:4) levels	Parkinson's disease	rs117505687	A	G	A	G	-2.26E-01	2.17E-02	3.77E-02	9.56E-01	FALSE	FALSE	FALSE	12	97265381	5.87E-02	1.48E-01	TRUE	12	96871603	5.09E-02	0.97	5564	9.36E-06	TRUE	TRUE	482,730	19.67
Phosphatidylcholine (16:1_20:4) levels	Parkinson's disease	rs174535	C	T	C	T	-3.28E-01	-2.60E-03	4.09E-01	6.66E-01	FALSE	FALSE	FALSE	11	61551356	1.78E-02	5.31E-02	TRUE	11	61783884	1.92E-02	1.00	5564	5.33E-64	TRUE	TRUE	482,730	292.68
Phosphatidylcholine (16:1_20:4) levels	Parkinson's disease	rs1757973	A	G	A	G	9.31E-02	4.42E-02	7.23E-01	2.79E-01	FALSE	FALSE	FALSE	6	169392762	2.47E-02	3.68E-02	TRUE	6	168992667	2.10E-02	1.00	5564	9.54E-06	TRUE	TRUE	482,730	19.64
Phosphatidylcholine (16:1_20:4) levels	Parkinson's disease	rs2085061	G	T	G	T	1.88E-01	4.30E-02	9.34E-01	3.93E-02	FALSE	FALSE	FALSE	4	177294407	8.10E-02	2.25E-01	TRUE	4	176373256	4.15E-02	0.87	5564	6.19E-06	TRUE	TRUE	482,730	20.47
Phosphatidylcholine (16:1_20:4) levels	Parkinson's disease	rs2197750	T	C	T	C	1.64E-01	-1.29E-01	7.10E-02	9.62E-01	FALSE	FALSE	FALSE	3	60481410	5.96E-02	1.50E-02	TRUE	3	60495677	3.67E-02	0.98	5564	7.74E-06	TRUE	TRUE	482,730	20.04
Phosphatidylcholine (16:1_20:4) levels	Parkinson's disease	rs35811605	T	A	T	A	1.38E-01	3.50E-02	1.22E-01	1.45E-01	TRUE	FALSE	FALSE	3	159628059	3.20E-02	5.62E-01	TRUE	3	159910270	2.92E-02	0.99	5564	2.25E-06	TRUE	TRUE	482,730	22.41
Phosphatidylcholine (16:1_20:4) levels	Parkinson's disease	rs4539990	C	T	C	T	-1.03E-01	-7.50E-03	7.69E-01	2.19E-01	FALSE	FALSE	FALSE	4	145092857	3.45E-02	8.14E-02	TRUE	4	144171704	2.31E-02	0.94	5564	9.31E-06	TRUE	TRUE	482,730	19.68
Phosphatidylcholine (16:1_20:4) levels	Parkinson's disease	rs56317095	A	G	A	G	2.69E-01	-1.02E-02	3.11E-02	9.65E-01	FALSE	FALSE	FALSE	7	150128447	6.58E-02	5.69E-02	TRUE	7	150431359	5.61E-02	0.93	5564	1.62E-06	TRUE	TRUE	482,730	23.05
Phosphatidylcholine (16:1_20:4) levels	Parkinson's disease	rs591931	T	C	T	C	1.10E-01	1.84E-02	1.92E-01	7.99E-01	FALSE	FALSE	FALSE	11	124694257	3.22E-02	2.46E-01	TRUE	11	124824361	2.42E-02	0.98	5564	5.55E-06	TRUE	TRUE	482,730	20.68
Phosphatidylcholine (16:1_20:4) levels	Parkinson's disease	rs6545883	G	A	G	A	9.53E-02	5.39E-02	6.20E-01	3.88E-01	FALSE	FALSE	FALSE	2	61772257	1.72E-02	8.63E-04	TRUE	2	61545122	1.96E-02	1.00	5564	1.17E-06	TRUE	TRUE	482,730	23.68
Phosphatidylcholine (16:1_20:4) levels	Parkinson's disease	rs75781049	G	T	G	T	1.69E-01	2.43E-02	6.74E-02	9.07E-01	FALSE	FALSE	FALSE	11	84448853	3.81E-02	2.81E-01	TRUE	11	84737810	3.80E-02	1.00	5564	9.13E-06	TRUE	TRUE	482,730	19.72
Phosphatidylcholine (16:1_20:4) levels	Parkinson's disease	rs75878220	C	G	C	G	-3.06E-01	-1.09E-01	2.55E-02	3.59E-02	TRUE	FALSE	FALSE	1	163836845	6.81E-02	9.65E-01	TRUE	1	163867608	6.18E-02	0.93	5564	7.32E-07	TRUE	TRUE	482,730	24.58
Phosphatidylcholine (16:1_20:4) levels	Parkinson's disease	rs8068175	T	C	T	C	8.90E-02	3.74E-02	4.21E-01	6.55E-01	FALSE	FALSE	FALSE	17	17874486	1.77E-02	1.73E-02	TRUE	17	17971172	1.91E-02	0.99	5564	3.32E-06	TRUE	TRUE	482,730	21.67
Phosphatidylcholine (17:0_18:1) levels	Parkinson's disease	rs1106933	G	A	G	A	1.25E-01	-2.35E-02	1.36E-01	8.79E-01	FALSE	FALSE	FALSE	6	167029624	3.47E-02	3.02E-01	TRUE	6	166616136	2.45E-02	1.00	7073	3.44E-07	TRUE	TRUE	482,730	26.03
Phosphatidylcholine (17:0_18:1) levels	Parkinson's disease	rs1257173	C	G	C	G	-1.34E-01	-1.51E-02	8.83E-02	6.14E-02	TRUE	FALSE	FALSE	2	134945928	5.04E-02	1.17E-01	TRUE	2	134188357	3.00E-02	0.97	7073	7.90E-06	TRUE	TRUE	482,730	19.99
Phosphatidylcholine (17:0_18:1) levels	Parkinson's disease	rs139070595	A	T	A	T	5.92E-01	8.18E-02	5.19E-03	1.62E-02	TRUE	FALSE	FALSE	1	247188295	9.60E-02	4.04E-01	TRUE	1	247024993	1.22E-01	0.91	7073	1.35E-06	TRUE	TRUE	482,730	23.39
Phosphatidylcholine (17:0_18:1) levels	Parkinson's disease	rs143506888	T	A	T	A	2.96E-01	-2.43E-01	1.84E-02	1.23E-02	TRUE	FALSE	FALSE	12	14268073	1.62E-01	8.78E-01	TRUE	12	14115139	6.43E-02	0.93	7073	4.13E-06	TRUE	TRUE	482,730	21.24
Phosphatidylcholine (17:0_18:1) levels	Parkinson's disease	rs145967151	T	C	T	C	-1.14E-01	3.17E-02	1.29E-01	8.75E-01	FALSE	FALSE	FALSE	2	242678654	3.55E-02	4.29E-01	TRUE	2	241739239	2.57E-02	0.95	7073	9.92E-06	TRUE	TRUE	482,730	19.55
Phosphatidylcholine (17:0_18:1) levels	Parkinson's disease	rs17231506	T	C	T	C	9.35E-02	1.36E-02	2.78E-01	6.87E-01	FALSE	FALSE	FALSE	16	56994528	1.81E-02	3.45E-01	TRUE	16	56960616	1.87E-02	1.00	7073	5.82E-07	TRUE	TRUE	482,730	25.02
Phosphatidylcholine (17:0_18:1) levels	Parkinson's disease	rs2126263	A	G	A	G	1.23E-01	-6.82E-02	8.50E-01	9.57E-02	FALSE	FALSE	FALSE	8	9181611	3.04E-02	1.24E-02	TRUE	8	9324101	2.36E-02	1.00	7073	1.95E-07	TRUE	TRUE	482,730	27.13
Phosphatidylcholine (17:0_18:1) levels	Parkinson's disease	rs4692808	A	G	A	G	8.02E-02	2.28E-02	4.36E-01	6.23E-01	FALSE	FALSE	FALSE	4	171117906	1.86E-02	6.56E-01	TRUE	4	170196755	1.69E-02	1.00	7073	2.08E-06	TRUE	TRUE	482,730	22.56
Phosphatidylcholine (17:0_18:1) levels	Parkinson's disease	rs5022521	T	C	T	C	9.63E-02	1.02E-02	7.97E-01	1.41E-01	FALSE	FALSE	FALSE	4	111128587	4.02E-02	9.69E-02	TRUE	4	110207431	2.14E-02	0.94	7073	6.55E-06	TRUE	TRUE	482,730	20.35
Phosphatidylcholine (17:0_18:1) levels	Parkinson's disease	rs61737926	T	C	T	C	-7.54E-01	-2.00E-02	2.67E-03	9.83E-01	FALSE	FALSE	FALSE	16	87678324	1.01E-01	7.40E-02	TRUE	16	87644718	1.64E-01	0.97	7073	4.10E-06	TRUE	TRUE	482,730	21.25
Phosphatidylcholine (17:0_18:1) levels	Parkinson's disease	rs7350789	A	G	A	G	1.13E-01	1.67E-02	3.93E-01	6.61E-01	FALSE	FALSE	FALSE	15	58679668	1.79E-02	4.57E-01	TRUE	15	58387469	1.71E-02	1.00	7073	4.46E-11	TRUE	TRUE	482,730	43.53
Phosphatidylcholine (17:0_18:1) levels	Parkinson's disease	rs76157703	T	G	T	G	-3.24E-01	-7.90E-02	1.48E-02	9.69E-01	FALSE	FALSE	FALSE	13	78777934	6.81E-02	6.09E-01	TRUE	13	78203799	7.14E-02	0.97	7073	5.78E-06	TRUE	TRUE	482,730	20.59
Phosphatidylcholine (17:0_18:1) levels	Parkinson's disease	rs77534789	A	C	A	C	-2.08E-01	-1.28E-01	3.76E-02	9.82E-01	FALSE	FALSE	FALSE	4	131610190	9.15E-02	7.90E-01	TRUE	4	130689035	4.51E-02	0.96	7073	4.12E-06	TRUE	TRUE	482,730	21.24
Phosphatidylcholine (17:0_18:1) levels	Parkinson's disease	rs78308012	G	T	G	T	1.53E-01	6.70E-02	1.03E-01	9.73E-01	FALSE	FALSE	FALSE	9	140481247	9.70E-02	3.10E-01	TRUE	9	137586795	2.88E-02	0.94	7073	1.11E-07	TRUE	TRUE	482,730	28.23
Phosphatidylcholine (17:0_18:2) levels	Parkinson's disease	rs11027519	T	C	T	C	-1.32E-01	2.40E-02	8.91E-02	9.02E-01	FALSE	FALSE	FALSE	11	23836587	3.74E-02	2.83E-01	TRUE	11	23815041	2.96E-02	1.00	7170	8.04E-06	TRUE	TRUE	482,730	19.96
Phosphatidylcholine (17:0_18:2) levels	Parkinson's disease	rs111233850	G	C	G	C	2.24E-01	-1.10E-02	3.01E-02	3.26E-02	TRUE	FALSE	FALSE	5	106341342	6.50E-02	6.25E-02	TRUE	5	107005641	4.98E-02	0.99	7170	7.06E-06	TRUE	TRUE	482,730	20.20
Phosphatidylcholine (17:0_18:2) levels	Parkinson's disease	rs115686032	T	C	T	C	-1.99E-01	6.00E-04	3.92E-02	9.82E-01	FALSE	FALSE	FALSE	5	40282865	7.88E-02	2.83E-03	TRUE	5	40282763	4.28E-02	0.99	7170	3.39E-06	TRUE	TRUE	482,730	21.61
Phosphatidylcholine (17:0_18:2) levels	Parkinson's disease	rs1253283	T	C	T	C	2.26E-01	3.75E-02	2.84E-02	9.44E-01	FALSE	FALSE	FALSE	1	99602664	4.87E-02	3.56E-01	TRUE	1	99137108	5.09E-02	0.99	7170	9.36E-06	TRUE	TRUE	482,730	19.67
Phosphatidylcholine (17:0_18:2) levels	Parkinson's disease	rs12548274	G	A	G	A	1.04E-01	2.22E-02	1.70E-01	8.98E-01	FALSE	FALSE	FALSE	8	134708443	3.84E-02	2.50E-01	TRUE	8	133696200	2.21E-02	0.98	7170	2.63E-06	TRUE	TRUE	482,730	22.10
Phosphatidylcholine (17:0_18:2) levels	Parkinson's disease	rs141509040	T	C	T	C	-1.43E-01	3.47E-02	7.44E-02	9.55E-01	FALSE	FALSE	FALSE	15	74024174	5.86E-02	2.57E-01	TRUE	15	73731833	3.18E-02	0.99	7170	7.49E-06	TRUE	TRUE	482,730	20.09

Phosphatidylcholine (17:0_18:2) levels	Parkinson's disease	rs1609446	A	C	A	C	1.43E-01	-1.91E-02	7.78E-02	8.81E-01	FALSE	FALSE	5	3220349	3.05E-02	2.74E-01	TRUE	5	3220235	3.20E-02	0.96	7170	7.97E-06	TRUE	TRUE	482,730	19.97
Phosphatidylcholine (17:0_18:2) levels	Parkinson's disease	rs17231506	T	C	T	C	9.67E-02	1.36E-02	2.78E-01	6.87E-01	FALSE	FALSE	16	56994528	1.81E-02	3.45E-01	TRUE	16	56960616	1.85E-02	1.00	7170	1.89E-07	TRUE	TRUE	482,730	27.19
Phosphatidylcholine (17:0_18:2) levels	Parkinson's disease	rs174566	G	A	G	A	2.59E-01	-5.70E-03	4.08E-01	6.64E-01	FALSE	FALSE	11	61592362	1.78E-02	1.24E-01	TRUE	11	61824890	1.68E-02	1.00	7170	9.03E-53	TRUE	TRUE	482,730	237.60
Phosphatidylcholine (17:0_18:2) levels	Parkinson's disease	rs1800588	T	C	T	C	1.15E-01	-3.30E-03	2.58E-01	7.75E-01	FALSE	FALSE	15	58723675	2.12E-02	5.73E-02	TRUE	15	58431476	1.94E-02	0.99	7170	3.31E-09	TRUE	TRUE	482,730	35.08
Phosphatidylcholine (17:0_18:2) levels	Parkinson's disease	rs192537901	C	T	C	T	-2.57E-01	1.24E-01	2.60E-02	9.86E-01	FALSE	FALSE	4	114384289	1.35E-01	4.48E-01	TRUE	4	113463133	5.27E-02	0.97	7170	1.10E-06	TRUE	TRUE	482,730	23.79
Phosphatidylcholine (17:0_18:2) levels	Parkinson's disease	rs2126260	C	T	C	T	9.13E-02	-9.70E-03	7.49E-01	2.03E-01	FALSE	FALSE	8	9185081	2.29E-02	1.73E-01	TRUE	8	9327571	1.91E-02	1.00	7170	1.72E-06	TRUE	TRUE	482,730	22.92
Phosphatidylcholine (17:0_18:2) levels	Parkinson's disease	rs4478142	T	C	T	C	-1.00E-01	3.76E-02	1.66E-01	7.65E-01	FALSE	FALSE	4	170323113	2.28E-02	4.96E-02	TRUE	4	169401962	2.24E-02	1.00	7170	7.67E-06	TRUE	TRUE	482,730	20.04
Phosphatidylcholine (17:0_18:2) levels	Parkinson's disease	rs4840981	G	C	G	C	3.39E-01	-1.13E-01	9.87E-01	9.82E-01	TRUE	FALSE	8	8390432	8.47E-02	7.40E-01	TRUE	8	8532922	7.35E-02	1.00	7170	4.05E-06	TRUE	TRUE	482,730	21.27
Phosphatidylcholine (17:0_18:2) levels	Parkinson's disease	rs57518551	C	T	C	T	-1.25E-01	8.50E-03	1.32E-01	8.90E-01	FALSE	FALSE	2	42383838	3.69E-02	8.77E-02	TRUE	2	42156698	2.46E-02	1.00	7170	3.79E-07	TRUE	TRUE	482,730	25.85
Phosphatidylcholine (17:0_18:2) levels	Parkinson's disease	rs6498540	G	A	G	A	9.02E-02	1.82E-02	3.34E-01	6.94E-01	FALSE	FALSE	16	15130594	1.84E-02	4.93E-01	TRUE	16	15036737	1.77E-02	0.99	7170	3.81E-07	TRUE	TRUE	482,730	25.83
Phosphatidylcholine (17:0_18:2) levels	Parkinson's disease	rs72747028	C	T	C	T	-1.51E-01	7.83E-02	7.27E-02	9.41E-01	FALSE	FALSE	1	211907139	4.91E-02	9.56E-01	TRUE	1	211733797	3.24E-02	0.98	7170	3.25E-06	TRUE	TRUE	482,730	21.70
Phosphatidylcholine (17:0_18:2) levels	Parkinson's disease	rs73058910	C	T	C	T	1.69E-01	-5.91E-02	6.33E-02	9.56E-01	FALSE	FALSE	7	14951761	6.05E-02	4.83E-01	TRUE	7	14912136	3.65E-02	0.89	7170	3.80E-06	TRUE	TRUE	482,730	21.40
Phosphatidylcholine (17:0_18:2) levels	Parkinson's disease	rs7437408	G	A	G	A	7.71E-02	-1.70E-02	4.78E-01	4.94E-01	FALSE	FALSE	4	150167397	2.22E-02	3.52E-01	TRUE	4	149246245	1.68E-02	1.00	7170	4.54E-06	TRUE	TRUE	482,730	21.05
Phosphatidylcholine (17:0_18:2) levels	Parkinson's disease	rs964184	C	G	C	G	-1.54E-01	-5.68E-02	8.49E-01	8.63E-01	TRUE	FALSE	11	116648917	2.43E-02	9.71E-03	TRUE	11	116778201	2.32E-02	1.00	7170	2.76E-11	TRUE	TRUE	482,730	44.48
Phosphatidylcholine (18:0_18:1) levels	Parkinson's disease	rs113394924	G	T	G	T	1.94E-01	-6.22E-02	8.73E-02	9.66E-01	FALSE	FALSE	11	62082293	5.88E-02	5.37E-01	TRUE	11	62314821	2.97E-02	0.99	7106	8.01E-11	TRUE	TRUE	482,730	42.39
Phosphatidylcholine (18:0_18:1) levels	Parkinson's disease	rs11626405	A	G	A	G	-8.14E-02	-5.90E-02	3.07E-01	7.61E-01	FALSE	FALSE	14	82304713	2.61E-02	1.19E-02	TRUE	14	81838369	1.81E-02	1.00	7106	7.26E-06	TRUE	TRUE	482,730	20.15
Phosphatidylcholine (18:0_18:1) levels	Parkinson's disease	rs116643980	T	G	T	G	1.69E-01	1.84E-01	5.56E-02	9.83E-01	FALSE	FALSE	1	4907719	1.05E-01	4.04E-02	TRUE	1	4847659	3.69E-02	0.95	7106	4.65E-06	TRUE	TRUE	482,730	21.01
Phosphatidylcholine (18:0_18:1) levels	Parkinson's disease	rs1168041	C	T	C	T	8.84E-02	-1.92E-02	7.33E-01	3.18E-01	FALSE	FALSE	1	62960250	2.05E-02	4.57E-01	TRUE	1	62494579	1.88E-02	0.99	7106	2.75E-06	TRUE	TRUE	482,730	22.02
Phosphatidylcholine (18:0_18:1) levels	Parkinson's disease	rs117580248	A	G	A	G	1.31E-01	-2.04E-01	1.10E-01	9.73E-01	FALSE	FALSE	9	140372403	9.93E-02	1.99E-02	TRUE	9	137477951	2.79E-02	0.92	7106	2.78E-06	TRUE	TRUE	482,730	22.00
Phosphatidylcholine (18:0_18:1) levels	Parkinson's disease	rs12429208	T	C	T	C	8.82E-02	2.20E-03	2.74E-01	6.45E-01	FALSE	FALSE	13	38341246	2.39E-02	3.28E-02	TRUE	13	37767109	1.91E-02	0.97	7106	3.76E-06	TRUE	TRUE	482,730	21.41
Phosphatidylcholine (18:0_18:1) levels	Parkinson's disease	rs12928399	C	G	C	G	-8.56E-02	4.18E-02	3.61E-01	3.71E-01	TRUE	FALSE	16	14705001	2.37E-02	3.89E-02	TRUE	16	14611144	1.75E-02	0.98	7106	1.08E-06	TRUE	TRUE	482,730	23.82
Phosphatidylcholine (18:0_18:1) levels	Parkinson's disease	rs142082962	A	C	A	C	-1.44E-01	-1.38E-02	7.79E-02	9.36E-01	FALSE	FALSE	6	158938443	4.59E-02	1.17E-01	TRUE	6	158517411	3.11E-02	0.99	7106	3.46E-06	TRUE	TRUE	482,730	21.57
Phosphatidylcholine (18:0_18:1) levels	Parkinson's disease	rs143060591	T	C	T	C	-1.50E-01	5.28E-02	7.43E-02	9.60E-01	FALSE	FALSE	8	86083491	1.65E-01	1.26E-01	TRUE	8	85171256	3.24E-02	0.97	7106	3.72E-06	TRUE	TRUE	482,730	21.44
Phosphatidylcholine (18:0_18:1) levels	Parkinson's disease	rs143309746	G	A	G	A	2.70E-01	-4.99E-02	2.25E-02	9.89E-01	FALSE	FALSE	1	27926445	1.94E-01	9.85E-02	TRUE	1	27599934	5.91E-02	0.91	7106	4.94E-06	TRUE	TRUE	482,730	20.89
Phosphatidylcholine (18:0_18:1) levels	Parkinson's disease	rs174533	A	G	A	G	-5.43E-01	-9.00E-04	4.09E-01	6.69E-01	FALSE	FALSE	11	61549025	1.79E-02	1.84E-02	TRUE	11	61781553	1.59E-02	1.00	7106	1.00E-200	TRUE	TRUE	482,730	1159.90
Phosphatidylcholine (18:0_18:1) levels	Parkinson's disease	rs190664666	A	G	A	G	-1.58E-01	3.36E-02	6.38E-02	9.74E-01	FALSE	FALSE	2	143687217	8.12E-02	1.68E-01	TRUE	2	142929648	3.47E-02	0.99	7106	5.01E-06	TRUE	TRUE	482,730	20.87
Phosphatidylcholine (18:0_18:1) levels	Parkinson's disease	rs2398428	G	A	G	A	-9.68E-02	-1.27E-02	2.63E-01	7.41E-01	FALSE	FALSE	12	129235566	2.67E-02	1.97E-01	TRUE	12	128751021	1.97E-02	0.91	7106	8.96E-07	TRUE	TRUE	482,730	24.18
Phosphatidylcholine (18:0_18:1) levels	Parkinson's disease	rs3110971	G	A	G	A	9.88E-02	-4.70E-03	2.08E-01	7.95E-01	FALSE	FALSE	5	38490271	2.11E-02	8.35E-02	TRUE	5	38490169	2.07E-02	0.99	7106	1.78E-06	TRUE	TRUE	482,730	22.86
Phosphatidylcholine (18:0_18:1) levels	Parkinson's disease	rs35788378	T	A	T	A	8.51E-02	-1.32E-02	2.62E-01	2.58E-01	TRUE	FALSE	1	236199122	2.16E-02	2.66E-01	TRUE	1	236035822	1.87E-02	1.00	7106	5.52E-06	TRUE	TRUE	482,730	20.68
Phosphatidylcholine (18:0_18:1) levels	Parkinson's disease	rs55725503	A	G	A	G	-2.48E-01	8.10E-03	2.48E-02	9.62E-01	FALSE	FALSE	3	66026439	6.15E-02	4.78E-02	TRUE	3	66040764	5.47E-02	0.95	7106	5.71E-06	TRUE	TRUE	482,730	20.61
Phosphatidylcholine (18:0_18:1) levels	Parkinson's disease	rs56399423	C	T	C	T	8.44E-02	2.90E-03	3.23E-01	5.74E-01	FALSE	FALSE	5	131672657	1.71E-02	6.19E-02	TRUE	5	132336964	1.79E-02	1.00	7106	2.49E-06	TRUE	TRUE	482,730	22.21
Phosphatidylcholine (18:0_18:1) levels	Parkinson's disease	rs57815521	T	C	T	C	-2.95E-01	-1.10E-01	2.75E-02	9.89E-01	FALSE	FALSE	11	61728147	1.07E-01	5.15E-01	TRUE	11	61960675	5.26E-02	0.99	7106	2.07E-08	TRUE	TRUE	482,730	31.50
Phosphatidylcholine (18:0_18:1) levels	Parkinson's disease	rs585002	C	T	C	T	1.15E-01	-3.05E-02	8.62E-01	1.93E-01	FALSE	FALSE	9	15293513	2.22E-02	7.70E-01	TRUE	9	15293515	2.42E-02	0.99	7106	2.33E-06	TRUE	TRUE	482,730	22.33
Phosphatidylcholine (18:0_18:1) levels	Parkinson's disease	rs600518	T	A	T	A	-1.13E-01	-1.27E-02	2.32E-01	1.66E-01	TRUE	FALSE	11	75455373	2.83E-02	1.85E-01	TRUE	11	75744328	1.99E-02	0.99	7106	1.42E-08	TRUE	TRUE	482,730	32.23
Phosphatidylcholine (18:0_18:1) levels	Parkinson's disease	rs61896563	T	C	T	C	-1.88E-01	2.39E-02	4.80E-02	9.60E-01	FALSE	FALSE	11	62034812	5.07E-02	1.96E-01	TRUE	11	62267340	3.99E-02	0.99	7106	2.47E-06	TRUE	TRUE	482,730	22.22
Phosphatidylcholine (18:0_18:1) levels	Parkinson's disease	rs73161879	A	G	A	G	-1.03E-01	-4.42E-02	1.81E-01	6.91E-01	FALSE	FALSE	7	151747225	3.36E-02	7.25E-01	TRUE	7	152050140	2.23E-02	0.94	7106	3.77E-06	TRUE	TRUE	482,730	21.41
Phosphatidylcholine (18:0_18:1) levels	Parkinson's disease	rs74452899	C	T	C	T	-4.46E-01	1.15E-01	1.06E-02	9.80E-01	FALSE	FALSE	12	89024239	1.15E-01	5.01E-01	TRUE	12	88630462	8.83E-02	0.85	7106	4.64E-07	TRUE	TRUE	482,730	25.45
Phosphatidylcholine (18:0_18:2) levels	Parkinson's disease	rs10040989	A	G	A	G	9.40E-02	3.23E-02	1.92E-01	8.76E-01	FALSE	FALSE	5	137573725	2.84E-02	5.93E-01	TRUE	5	138238036	2.09E-02	1.00	7174	6.86E-06	TRUE	TRUE	482,730	20.26
Phosphatidylcholine (18:0_18:2) levels	Parkinson's disease	rs10889352	C	T	C	T	-1.01E-01	4.00E-04	2.62E-01	6.75E-01	FALSE	FALSE	1	63099023	1.80E-02	6.92E-03	TRUE	1	62633352	1.88E-02	1.00	7174	8.49E-08	TRUE	TRUE	482,730	28.75
Phosphatidylcholine (18:0_18:2) levels	Parkinson's disease	rs10890773	A	G	A	G	-1.09E-01	5.70E-02	8.60E-01	1.45E-01	FALSE	FALSE	11	107760002	3.20E-02	3.74E-02	TRUE	11	107889276	2.38E-02	1.00	7174	4.83E-06	TRUE	TRUE	482,730	20.93
Phosphatidylcholine (18:0_18:2) levels	Parkinson's disease	rs12122878	T	C	T	C	-8.26E-02	-2.16E-02	2.89E-01	7.35E-01	FALSE	FALSE	1	189896505	3.04E-02	3.21E-01	TRUE	1	189927375	1.85E-02	0.98	7174	7.96E-06	TRUE	TRUE	482,730	19.98
Phosphatidylcholine (18:0_18:2) levels	Parkinson's disease	rs12211498	A	G	A	G	-1.30E-01	-5.42E-02	9.52E-02	8.95E-01	FALSE	FALSE	6	46746889	3.04E-02	3.73E-02	TRUE	6	46779152	2.88E-02	0.98	7174	6.54E-06	TRUE	TRUE	482,730	20.35
Phosphatidylcholine (18:0_18:2) levels	Parkinson's disease	rs12620952	A	G	A	G	4.63E-01	-2.25E-02	7.11E-03	9.74E-01	FALSE	FALSE	2	5889145	7.38E-02	1.19E-01	TRUE	2	5749013	1.02E-01	0.98	7174	5.72E-06	TRUE	TRUE	482,730	20.61
Phosphatidylcholine (18:0_18:2) levels	Parkinson's disease	rs150457719	G	A	G	A	-2.18E-01	-4.55E-02	3.45E-02	9.85E-01	FALSE	FALSE	1	212421302	1.02E-01	1.83E-01	TRUE	1	212247960	4.56E-02	0.98	7174	1.74E-06	TRUE	TRUE	482,730	22.89
Phosphatidylcholine (18:0_18:2) levels	Parkinson's disease	rs16838403	C	G	C	G	-2.70E-01	7.89E-02	1.91E-02	2.77E-02	TRUE	FALSE	2	207494617	5.88E-02	7.46E-01	TRUE	2	206629893	6.06E-02	1.00	7174	8.74E-06	TRUE	TRUE	482,730	19.80
Phosphatidylcholine (18:0_18:2) levels	Parkinson's disease	rs17231506	T	C	T	C	1.07E-01	1.36E-02	2.78E-01	6.87E-01	FALSE	FALSE	16	56994528	1.81E-02	3.45E-01	TRUE	16	56960616	1.85E-02	1.00	7174	6.78E-09	TRUE	TRUE	482,730	33.68
Phosphatidylcholine (18:0_18:2) levels	Parkinson's disease	rs174594	A	C	A	C	-2.47E-01	9.90E-03	5.82E-01	3.63E-01	FALSE	FALSE	11	61619829	1.78E-02	2.39E-01	TRUE	11	61852357	1.67E-02	1.00	7174	8.13E-49	TRUE	TRUE	482,730	218.91
Phosphatidylcholine (18:0_18:2) levels	Parkinson's disease	rs1800588	T	C	T	C	1.35E-01	-3.30E-03	2.58E-01	7.75E-01	FALSE	FALSE	15	58723675	2.12E-02	5.73E-02	TRUE	15	58431476	1.93E-02	0.99	7174	2.52E-12	TRUE	TRUE	482,730	49.20
Phosphatidylcholine (18:0_18:2) levels	Parkinson's disease	rs2274855	A	G	A	G	-1.05E-01	-1.29E-02	1.71E-01	8.35E-01	FALSE	FALSE	9	135105944	3.32E-02	1.57E-01	TRUE	9	132230557	2.31E-02	0.89	7174	5.43E-06	TRUE	TRUE	482,730	20.71
Phosphatidylcholine (18:0_18:2) levels	Parkinson's disease	rs5022521	T	C	T	C	9.38E-02	1.02E-02	7.97E-01	1.41E-01	FALSE	FALSE	4	111128587	4.02E-02	9.69E-02	TRUE	4	110207431	2.12E-02	0.94	7174	9.45E-06	TRUE	TRUE	482,730	19.65
Phosphatidylcholine (18:0_18:2) levels	Parkinson's disease	rs55917360	T	C	T	C	-1.73E-01	9.71E-02	7.34E-02	9.37E-01	FALSE	FALSE	1	211908885	4.77E-02	2.09E-02	TRUE	1	211735543	3.21E-02	0.99	7174	6.90E-08	TRUE	TRUE	482,730	29.15
Phosphatidylcholine (18:0_18:2) levels	Parkinson's disease	rs564216	C	T	C	T	-1.65E-01	-4.50E-02	5.50E-02	9.28E-01	FALSE	FALSE	11	125965332	3.75E-02	6.39E-01	TRUE	11	126095437	3.66E-02	0.98	7174	6.38E-06	TRUE	TRUE	482,730	20.40
Phosphatidylcholine (18:0_18:2) levels	Parkinson's disease	rs62039480	A	G	A	G	9.57E-02	1.69E-02	3.32E-01	6.66E-01	FALSE	FALSE	16	15137450	1.83E-02	4.52E-01	TRUE	16	15043593	1.77E-02	1.00	7174	6.94E-08	TRUE	TRUE	482,730	29.14
Phosphatidylcholine (18:0_18:2) levels	Parkinson's disease	rs6432738	G	A	G	A	7.52E-02	-1.70E-03	4.74E-01	4.57E-01	FALSE	FALSE	2	163775203	2.07E-02	2.99E-02	TRUE	2	162918693	1.68E-02	0.97	7174	7.24E-06	TRUE	TRUE	482,730	20.16
Phosphatidylcholine (18:0_18:2) levels	Parkinson's disease	rs6678964	G	A	G	A	2.00E-01	-3.46E-02	9.02E-01	8.77E-02	FALSE	FALSE	1	95694268	3.13E-02	5.69E-01	TRUE	1	95228712	2.78E-02	1.00	7174	6.84E-13	TRUE	TRUE	482,730	51.78
Phosphatidylcholine (18:0_18:2) levels	Parkinson's disease	rs675849	C	T	C	T	1.27E-01	-3.21E-02	8.99E-01	1.39E-01	FALSE	FALSE	9	15298666	2.51E-02	6.99E-01	TRUE	9	15298668	2.73E-02	0.99	7174	3.50E-06	TRUE	TRUE	482,730	21.55
Phosphatidylcholine (18:0_18:2) levels	Parkinson's disease	rs6878903	T	G	T	G	8.76E-02	3.60E-02	7.64E-01	1.43E-01	FALSE	FALSE	5	85591115	3.17E-02	5.91E-01	TRUE	5	86295297	1.96E-02	1.00	7174	8.02E-06	TRUE	TRUE	482,730	19.96
Phosphatidylcholine (18:0_18:2) levels	Parkinson's disease	rs73126869	G	A	G	A	1.33E-01	-3.92E-02	9.93E-02	8.72E-01	FALSE	FALSE	4	14740855	2.87E-02	7.66E-01	TRUE	4	14739231	2.86E-02	0.95	7174	3.48E-06	TRUE	TRUE	482,730	21.57
Phosphatidylcholine (18:0_18:2) levels	Parkinson's disease	rs77008693	A	G	A	G	1.11E-01	-3.98E-02	1.39E-01	8.10E-01	FALSE	FALSE	17	42832804	2.22E-02	3.65E-02	TRUE	17	44755436	2.39E-02	1.00	7174	3.40E-06	TRUE	TRUE	482,730	21.61
Phosphatidylcholine (18:0_18:2) levels	Parkinson's disease	rs77588674	T	G	T	G	1.00E-01	-3.45E-02	1.69E-01	8.69E-01	FALSE	FALSE	2	122071404	4.32E-02	3.73E-01	TRUE	2	121313828	2.26E-02	0.95	7174	8.96E-06	TRUE	TRUE	482,730	19.75
Phosphatidylcholine (18:0_18:2) levels	Parkinson's disease	rs7932326	A	G	A	G	7.73E-02	1.61E-02	6.17E-01	4.05E-01	FALSE	FALSE	11	43993024	2.26E-02	3.21E-01	TRUE	11	43971474	1.71E-02	1.00	7174	6.29E-06	TRUE	TRUE	482,730	20.43
Phosphatidylcholine (18:0_18:2) levels	Parkinson's disease	rs964184	C	G	C	G	-1.28E-01	-5.68E-02	8.49E-01	8.63E-01	TRUE	FALSE	11	116648917	2.43E-02	9.71E-03	TRUE	11	116778201	2.31E-02	1.00	7174	3.04E-08	TRUE	TRUE	482,730	30.75
Phosphatidylcholine (18:0_18:2) levels	Parkinson's disease	rs9987289	G	A	G	A	1.26E-01	-6.15E-02	8.52E-01	8.85E-02	FALSE	FALSE	8	9183358	3.10E-02	2.36E-02	TRUE	8	9325848	2.34E-02	1.00	7174	8.75E-08	TRUE	TRUE	482,730	28.69
Phosphatidylcholine (18:0_18:3) levels	Parkinson's disease	rs10874911	A	G	A	G	-8.29E-02	-5.20E-03	3.94E-01	4.66E-01	FALSE	FALSE	1	92194523	2.23E-02	8.79E-02	TRUE	1	91728966	1.76E-02	0.99	6692	2.41E-06	TRUE	TRUE	482,730	22.27
Phosphatidylcholine (18:0_18:3) levels	Parkinson's disease	rs11222413	A	T	A	T	-2.94E-01	3.50E-03	1.80E-02	2.85E-02	TRUE	FALSE	11	130840925	6.88E-02	1.82E-02	TRUE	11	130971030	6.63E-02	0.96	6692	9.56E-06	TRUE	TRUE	482,730	19.63
Phosphatidylcholine (18:0_18:3) levels	Parkinson's disease	rs116844659	G	A	G	A	6.14E-01	1.18E-02	4.89E-03	9.80E-01	FALSE	FALSE	12	12357520	8.22E-02	5.25E-02	TRUE	12	12204586	1.35E-01	0.91	6692	5.67E-06	TRUE	TRUE	482,730	20.63
Phosphatidylcholine (18:0_18:3) levels	Parkinson's disease	rs118094862	T	G	T	G	-4.48E-01	4.16E-02	7.53E-03	9.87E-01	FALSE	FALSE	12	30982663	1.26E-01	1.31E-01	TRUE	12	30829729	1.01E-01	0.94	6692	9.08E-06	TRUE	TRUE	482,730	19.72
Phosphatidylcholine (18:0_18:3) levels	Parkinson's disease	rs12448528	G	A	G	A	9.57E-02	2.16E-02	7.92E-01	2.16E-01	FALSE	FALSE	16	56985555	2.25E-02	4.70E-01	TRUE	16	56951643	2.16E-02	0.97	6692	9.70E-06	TRUE	TRUE	482,730	19.60
Phosphatidylcholine (18:0_18:3) levels	Parkinson's disease	rs1260326	C	T	C	T	-8.53E-02	-6.78E-02	6.51E-01	4.20E-01	FALSE	FALSE	2	27730940	1.72E-02	4.04E-05	TRUE	2	27508073	1.81E-02	1.00	6692	2.52E-06	TRUE	TRUE	482,730	22.19
Phosphatidylcholine (18:0_18:3) levels	Parkinson's disease	rs143029300	A	G	A	G	3.04E-01	3.45E-02	1.78E-02	9.81E-01	FALSE	FALSE	17	13477758	9.02E-02	1.53E-01	TRUE	17	13574441	6.71E-02	0.93	6692	6.21E-06	TRUE	TRUE	482,730	20.46
Phosphatidylcholine (18:0_18:3) levels	Parkinson's disease	rs16949722	G	T	G	T	3.16E-01	-1.86E-02	1.55E-02	9.59E-01	FALSE	FALSE	15	95615192	5.91E-02	1.23E-01	TRUE	15	95071963	7.11E-02	0.96	6692	8.83E-06	TRUE	TRUE	482,730	19.78
Phosphatidylcholine (18:0_18:3) levels	Parkinson's disease	rs183478874	A	C	A	C	-2.95E-01	-9.29E-02	2.35E-02	9.69E-01	FALSE	FALSE	5	176425594	7.33E-02	6.88E-01	TRUE	5	176998593	5.93E-02	0.95	6692	6.57E-07	TRUE	TRUE	482,730	24.78
Phosphatidylcholine (18:0_18:3) levels	Parkinson's disease	rs2797620	T	C	T	C	-9.71E-02	1.39E-02	3.33E-01	6.30E-01	FALSE	FALSE	1	95441292	2.33E-02	2.59E-01	TRUE	1	94975736	1.83E-02	1.00	6692	1.07E-07	TRUE	TRUE	482,730	28.31
Phosphatidylcholine (18:0_18:3) levels	Parkinson's disease	rs3729986	T	C	T	C	-1.25E-01	5.62E-02	1.05E-01	9.03E-01	FALSE	FALSE	11	47371598	2.90E-02	2.63E-02	TRUE	11	47350047	2.82E-02	0.98	6692	8.87E-06	TRUE	TRUE	482,730	19.77
Phosphatidylcholine (18:0_18:3) levels	Parkinson's disease	rs4846841	G	A	G	A	9.12E-02	7.00E-04	7.47E-01	2.07E-01	FALSE	FALSE	1	230302661	2.34E-02	1.06E-02	TRUE	1	230166915	1.97E-02	1.00	6692	3.79E-06	TRUE	TRUE	482,730	21.40
Phosphatidylcholine (18:0_18:3) levels	Parkinson's disease	rs4888557	A	T	A	T	3.06E-01	-8.48E-02	1.90E-02	6.09E-02	TRUE	FALSE	16	76850155	4.85E-02	4.02E-02	TRUE	16	76816258	6.43E-02	0.98	6692	2.00E-06	TRUE	TRUE	482,730	22.63
Phosphatidylcholine (18:0_18:3) levels	Parkinson's disease	rs7139079	A	G	A	G	-8.06E-02	1.53E-02	5.47E-01	4.21E-01	FALSE	FALSE	12	121415293	1.78E-02	4.09E-01	TRUE	12	120977490	1.74E-02	1.00	6692	3.50E-06	TRUE	TRUE	482,730	21.56
Phosphatidylcholine (18:0_18:3) levels	Parkinson's disease	rs72867166	A	T	A	T	1.24E-01	-1.04E-02	1.18E-01	9.12E-02	TRUE	FALSE	6	41846422	3.40E-02	1.20E-01	TRUE	6	41878684	2.71E-02	0.99	6692	4.87E-06	TRUE	TRUE	482,730	20.92
Phosphatidylcholine (18:0_18:3) levels	Parkinson's disease	rs73215839	T	C	T	C	2.69E-01	1.91E-02	3.35E-02	9.40E-01	FALSE	FALSE	12	92525192	5.02E-02	1.53E-01	TRUE	12	92131416	4.91E-02	0.96	6692	4.33E-08	TRUE	TRUE	482,730	30.06
Phosphatidylcholine (18:0_18:3) levels	Parkinson's disease	rs75593273	T	C	T	C	1.38E-01	-7.93E-02	9.52E-02	9.58E-01	FALSE	FALSE	5	44740143	7.97E-02	4.95E-01	TRUE	5	44740041	2.99E-02	0.93	6692	3.91E-06	TRUE	TRUE	482,730	21.34
Phosphatidylcholine (18:0_18:3) levels	Parkinson's disease	rs79831249	C	G	C	G	3.80E-01	-1.25E-01	1.26E-02	1.32E-02	TRUE	FALSE	4	95459596	1.09E-01	5.99E-01	TRUE	4	94538445	8.33E-02	0.86	6692	5.18E-06	TRUE	TRUE	482,730	20.80
Phosphatidylcholine (18:0_20:2) levels	Parkinson's disease	rs111595890	A	G	A	G	-2.94E-01	3.48E-02	2.20E-02	9.72E-01	FALSE	FALSE	1	215140309	7.08E-02	2.06E-01	TRUE	1	214966966	6.51E-02	0.97	5778	6.17E-06	TRUE	TRUE	482,730	20.47
Phosphatidylcholine (18:0_20:2) levels	Parkinson's disease	rs112483795	G	A	G	A	-2.90E-01	4.30E-02	2.38E-02	9.65E-01	FALSE	FALSE	8	12839016	6.46E-02	2.96E-01	TRUE	8	12981507	6.24E-02	0.95	5778	3.37E-06	TRUE	TRUE	482,730	21.64
Phosphatidylcholine (18:0_20:2) levels	Parkinson's disease	rs12513382	T	C	T	C	-8.84E-02	-4.90E-03	6.21E-01	5.32E-01	FALSE	FALSE	4	43914603	1.72E-02	1.10E-01	TRUE	4	43912586	1.91E-02	1.00	5778	3.68E-06	TRUE	TRUE	482,730	21.46
Phosphatidylcholine (18:0_20:2) levels	Parkinson's disease	rs1422819	G	T	G	T	-8.97E-02	4.32E-02	5.93E-01	5.15E-01	FALSE	FALSE	5	166098883	2.23E-02	2.64E-02	TRUE	5	166671878	1.88E-02	1.00	5778	1.97E-06	TRUE	TRUE	482,730	22.66
Phosphatidylcholine (18:0_20:2) levels	Parkinson's disease	rs147220276	G	T	G	T	4.27E-01	1.79E-02	1.01E-02	9.80E-01	FALSE	FALSE	13	73934392	8.89E-02	7.56E-02	TRUE	13	73360255	9.54E-02	0.97	5778	7.84E-06	TRUE	TRUE	482,730	20.01
Phosphatidylcholine (18:0_20:2) levels	Parkinson's disease	rs17238104	T	C	T	C	-1.23E-01	1.62E-02	1.38E-01	8.69E-01	FALSE	FALSE	13	38003608	3.42E-02	1.96E-01	TRUE	13	37429471	2.73E-02	0.97	5778	7.01E-06	TRUE	TRUE	482,730	20.23
Phosphatidylcholine (18:0_20:2) levels	Parkinson's disease	rs174547	C	T	C	T	2.82E-01	-3.30E-03	4.07E-01	6.72E-01	FALSE	FALSE	11	61570783	1.79E-02	6.93E-02	TRUE	11	61803311	1.86E-02	1.00	5778	4.05E-51	TRUE	TRUE	482,730	230.65
Phosphatidylcholine (18:0_20:2) levels	Parkinson's disease	rs34955778	C	T	C	T	9.79E-02	5.20E-03	4.36E-01	5.86E-01	FALSE	FALSE	16	15139594	1.78E-02	1.15E-01	TRUE	16	15045737	1.88E-02	0.99	5778	2.01E-07	TRUE	TRUE	482,730	27.09
Phosphatidylcholine (18:0_20:2) levels	Parkinson's disease	rs67207258	T	C	T	C	-1.05E-01	-3.00E-02	1.90E-01	7.74E-01	FALSE	FALSE	8	5461517	2.73E-02	5.66E-01	TRUE	8	5603995	2.36E-02	0.99	5778	9.22E-06	TRUE	TRUE	482,730	19.70
Phosphatidylcholine (18:0_20:2) levels	Parkinson's disease	rs6744368	T	C	T	C	1.88E-01	-8.00E-04	9.48E-01	6.60E-02	FALSE	FALSE	2	68672015	3.80E-02	7.58E-03	TRUE	2	68444883	4.26E-02	0.98	5778	9.85E-06	TRUE	TRUE	482,730	19.57
Phosphatidylcholine (18:0_20:2) levels	Parkinson's disease	rs7139079	A	G	A	G	-9.90E-02	1.53E-02	5.47E-01	4.21E-01	FALSE	FALSE	12	121415293	1.78E-02	4.09E-01	TRUE	12	120977490	1.87E-02	1.00	5778	1.17E-07	TRUE	TRUE	482,730	28.14
Phosphatidylcholine (18:0_20:2) levels	Parkinson's disease	rs7959861	G	A	G	A	8.70E-02	1.53E-02	3.48E-01	6.09E-01	FALSE	FALSE	12	90733670	2.30E-02	2.95E-01	TRUE	12	90339893	1.95E-02	1.00	5778	8.23E-06	TRUE	TRUE	482,730	19.92
Phosphatidylcholine (18:0_20:2) levels	Parkinson's disease	rs8068923	A	G	A	G	1.90E-01	4.97E-02	9.40E-01	7.55E-02	FALSE	FALSE	17	52818402	4.25E-02	6.15E-01	TRUE	17	54741041	3.97E-02	0.99	5778	1.81E-06	TRUE	TRUE	482,730	22.83
Phosphatidylcholine (18:0_20:2) levels	Parkinson's disease	rs9671254	A	T	A	T	-9.51E-02	-2.76E-02	7.11E-01	7.61E-01	TRUE	FALSE	14	25415993	2.57E-02	5.50E-01	TRUE	14	24946787	2.06E-02	0.99	5778	3.88E-06	TRUE	TRUE	482,730	21.36
Phosphatidylcholine (18:0_20:3) levels	Parkinson's disease	rs114162005	C	A	C	A	-1.79E-01	1.11E-02	5.26E-02	9.65E-01	FALSE	FALSE	3	148026139	7.25E-02	5.65E-02	TRUE	3	148308352	3.94E-02	0.89	7169	5.96E-06	TRUE	TRUE	482,730	20.53
Phosphatidylcholine (18:0_20:3) levels	Parkinson's disease	rs11577288	A	G	A	G	8.93E-02	-6.50E-03	2.34E-01	7.94E-01	FALSE	FALSE	1	242769507	2.27E-02	1.11E-01	TRUE	1	242606205	1.99E-02	0.98	7169	7.14E-06	TRUE	TRUE	482,730	20.18
Phosphatidylcholine (18:0_20:3) levels	Parkinson's disease	rs116259186	C	T	C	T	2.27E-01	-2.29E-02	2.92E-02	9.51E-01	FALSE	FALSE	1	74968148	4.58E-02	2.09E-01	TRUE	1	74502464	5.06E-02	0.98	7169	7.49E-06	TRUE	TRUE	482,730	20.09
Phosphatidylcholine (18:0_20:3) levels	Parkinson's disease	rs11648397	C	G	C	G	1.52E-01	1.85E-02	7.83E-02	7.21E-02	TRUE	FALSE	16	78623868	4.37E-02	1.72E-01	TRUE	16	78558971	3.16E-02	0.98	7169	1.59E-06	TRUE	TRUE	482,730	23.07
Phosphatidylcholine (18:0_20:3) levels	Parkinson's disease	rs116917626	T	C	T	C	-2.36E-01	1.84E-02	2.75E-02	9.86E-01	FALSE	FALSE	17	49639325	1.53E-01	4.36E-02	TRUE	17	51561964	5.25E-02	0.97	7169	6.79E-06	TRUE	TRUE	482,730	20.28
Phosphatidylcholine (18:0_20:3) levels	Parkinson's disease	rs1169288	C	A	C	A	7.75E-02	2.30E-03	3.77E-01	6.72E-01	FALSE	FALSE	12	121416650	1.79E-02	4.77E-02	TRUE	12	120978847	1.72E-02	1.00	7169	6.39E-06	TRUE	TRUE	482,730	20.40
Phosphatidylcholine (18:0_20:3) levels	Parkinson's disease	rs11847009	A	T	A	T	-1.11E-01	-7.80E-03	1.74E-01	2.45E-01	TRUE	FALSE	14	33945710	2.61E-02	1.15E-01	TRUE	14	33476504	2.23E-02	0.99	7169	6.87E-07	TRUE	TRUE	482,730	24.69
Phosphatidylcholine (18:0_20:3) levels	Parkinson's disease	rs13001426	A	C	A	C	3.35E-01	8.92E-02	1.36E-02	9.87E-01	FALSE	FALSE	2	169912172	1.18E-01	3.47E-01	TRUE	2	169055662	7.44E-02	0.92	7169	6.79E-06	TRUE	TRUE	482,730	20.28
Phosphatidylcholine (18:0_20:3) levels	Parkinson's disease	rs140123208	G	A	G	A	-2.04E-01	-6.83E-02	3.83E-02	9.85E-01	FALSE	FALSE	5	16863628	1.53E-01	1.84E-01	TRUE	5	16863519	4.39E-02	0.97	7169	3.45E-06	TRUE	TRUE	482,730	21.58
Phosphatidylcholine (18:0_20:3) levels	Parkinson's disease	rs140588245	A	G	A	G	3.64E-01	2.84E-02	1.14E-02	9.88E-01	FALSE	FALSE	11	124198662	8.60E-02	1.30E-01	TRUE	11	124328766	8.18E-02	0.93	7169	8.52E-06	TRUE	TRUE	482,730	19.84
Phosphatidylcholine (18:0_20:3) levels	Parkinson's disease	rs151178379	A	G	A	G	2.89E-01	-1.60E-01	1.94E-02	9.82E-01	FALSE	FALSE	9	133450524	8.81E-02	3.51E-02	TRUE	9	130575137	6.14E-02	0.99	7169	2.48E-06	TRUE	TRUE	482,730	22.21
Phosphatidylcholine (18:0_20:3) levels	Parkinson's disease	rs192117305	T	C	T	C	-1.97E-01	2.07E-01	4.09E-02	9.82E-01	FALSE	FALSE	5	33853188	1.98E-01	5.29E-01	TRUE	5	33853083	4.29E-02	0.96	7169	4.38E-06	TRUE	TRUE	482,730	21.12
Phosphatidylcholine (18:0_20:3) levels	Parkinson's disease	rs2797620	T	C	T	C	-1.16E-01	1.39E-02	3.33E-01	6.30E-01	FALSE	FALSE	1	95441292	2.33E-02	2.59E-01	TRUE	1	94975736	1.75E-02	1.00	7169	3.27E-11	TRUE	TRUE	482,730	44.14
Phosphatidylcholine (18:0_20:3) levels	Parkinson's disease	rs3134937	T	C	T	C	-9.51E-02	-3.09E-02	2.00E-01	7.15E-01	FALSE	FALSE	6	32206539	2.18E-02	8.05E-01	TRUE	6	32238762	2.08E-02	0.99	7169	4.78E-06	TRUE	TRUE	482,730	20.95
Phosphatidylcholine (18:0_20:3) levels	Parkinson's disease	rs56374730	G	T	G	T	8.85E-02	-3.00E-02	2.96E-01	7.43E-01	FALSE	FALSE	16	15867652	2.56E-02	6.19E-01	TRUE	16	15773795	1.84E-02	0.99	7169	1.44E-06	TRUE	TRUE	482,730	23.26
Phosphatidylcholine (18:0_20:3) levels	Parkinson's disease	rs59739041	C	T	C	T	1.83E-01	2.12E-02	4.79E-02	9.50E-01	FALSE	FALSE	15	58661083	5.23E-02	1.64E-01	TRUE	15	58368884	3.87E-02	0.99	7169	2.32E-06	TRUE	TRUE	482,730	22.34
Phosphatidylcholine (18:0_20:3) levels	Parkinson's disease	rs62239496	G	A	G	A	-9.44E-02	-3.60E-03	2.01E-01	7.18E-01	FALSE	FALSE	3	37492545	2.19E-02	6.11E-02	TRUE	3	37451054	2.10E-02	0.99	7169	7.30E-06	TRUE	TRUE	482,730	20.14
Phosphatidylcholine (18:0_20:3) levels	Parkinson's disease	rs6498540	G	A	G	A	-1.99E-01	1.82E-02	3.34E-01	6.94E-01	FALSE	FALSE	16	15130594	1.84E-02	4.93E-01	TRUE	16	15036737	1.76E-02	0.99	7169	2.09E-29	TRUE	TRUE	482,730	127.89
Phosphatidylcholine (18:0_20:3) levels	Parkinson's disease	rs72827678	G	T	G	T	1.43E-01	3.36E-02	8.56E-02	9.25E-01	FALSE	FALSE	5	168259808	4.35E-02	3.56E-01	TRUE	5	168832803	3.01E-02	0.99	7169	2.15E-06	TRUE	TRUE	482,730	22.49
Phosphatidylcholine (18:0_20:3) levels	Parkinson's disease	rs78009561	G	C	G	C	-1.27E-01	-3.70E-02	9.59E-02	8.54E-02	TRUE	FALSE	4	158967810	4.12E-02	4.33E-01	TRUE	4	158046658	2.88E-02	0.98	7169	9.94E-06	TRUE	TRUE	482,730	19.55
Phosphatidylcholine (18:0_20:3) levels	Parkinson's disease	rs7943728	A	G	A	G	2.36E-01	-3.17E-02	1.12E-01	8.28E-01	FALSE	FALSE	11	61547068	2.27E-02	7.88E-01	TRUE	11	61779596	2.65E-02	1.00	7169	6.87E-19	TRUE	TRUE	482,730	79.24

Phosphatidylcholine (18:0_20:4) levels	Parkinson's disease	rs1219539	A	G	A	G	-8.77E-02	1.79E-02	4.93E-01	4.78E-01	FALSE	FALSE	11	75433144	1.71E-02	5.32E-01	TRUE	11	75722099	1.68E-02	0.99	7174	1.74E-07	TRUE	TRUE	482,730	27.35
Phosphatidylcholine (18:0_20:4) levels	Parkinson's disease	rs12275418	A	G	A	G	1.24E-01	4.33E-02	1.34E-01	8.09E-01	FALSE	FALSE	11	60779823	2.25E-02	2.71E-02	TRUE	11	61012351	2.47E-02	1.00	7174	4.66E-07	TRUE	TRUE	482,730	25.44
Phosphatidylcholine (18:0_20:4) levels	Parkinson's disease	rs139102782	A	G	A	G	-2.06E-01	1.28E-01	3.40E-02	9.84E-01	FALSE	FALSE	14	84113812	1.28E-01	4.98E-01	TRUE	14	83647468	4.63E-02	0.96	7174	9.06E-06	TRUE	TRUE	482,730	19.73
Phosphatidylcholine (18:0_20:4) levels	Parkinson's disease	rs147549994	T	G	T	G	2.56E-01	-1.38E-02	3.59E-02	9.86E-01	FALSE	FALSE	16	1989265	1.01E-01	4.97E-02	TRUE	16	1939264	4.50E-02	0.95	7174	1.35E-08	TRUE	TRUE	482,730	32.32
Phosphatidylcholine (18:0_20:4) levels	Parkinson's disease	rs147981159	A	G	A	G	-3.46E-01	2.71E-02	4.33E-02	9.64E-01	FALSE	FALSE	11	61824298	6.29E-02	1.76E-01	TRUE	11	62056826	4.18E-02	0.96	7174	1.54E-16	TRUE	TRUE	482,730	68.44
Phosphatidylcholine (18:0_20:4) levels	Parkinson's disease	rs174528	C	T	C	T	-6.66E-01	-6.00E-03	4.14E-01	6.34E-01	FALSE	FALSE	11	61543499	1.79E-02	1.33E-01	TRUE	11	61776027	1.51E-02	1.00	7174	1.00E-200	TRUE	TRUE	482,730	1946.15
Phosphatidylcholine (18:0_20:4) levels	Parkinson's disease	rs2070895	A	G	A	G	9.37E-02	-4.20E-03	2.67E-01	7.58E-01	FALSE	FALSE	15	58723939	2.23E-02	7.08E-02	TRUE	15	58431740	1.90E-02	0.98	7174	8.73E-07	TRUE	TRUE	482,730	24.23
Phosphatidylcholine (18:0_20:4) levels	Parkinson's disease	rs223841	G	A	G	A	-1.03E-01	-1.10E-03	1.72E-01	8.16E-01	FALSE	FALSE	16	57457271	2.54E-02	1.54E-02	TRUE	16	57423359	2.19E-02	1.00	7174	2.89E-06	TRUE	TRUE	482,730	21.93
Phosphatidylcholine (18:0_20:4) levels	Parkinson's disease	rs2797620	T	C	T	C	-1.01E-01	1.39E-02	3.33E-01	6.30E-01	FALSE	FALSE	1	95441292	2.33E-02	2.59E-01	TRUE	1	94975736	1.74E-02	1.00	7174	6.11E-09	TRUE	TRUE	482,730	33.88
Phosphatidylcholine (18:0_20:4) levels	Parkinson's disease	rs2802390	T	C	T	C	-7.53E-02	-2.00E-04	4.65E-01	4.74E-01	FALSE	FALSE	13	99342630	2.26E-02	3.18E-03	TRUE	13	98690376	1.68E-02	0.99	7174	7.51E-06	TRUE	TRUE	482,730	20.09
Phosphatidylcholine (18:0_20:4) levels	Parkinson's disease	rs2990536	T	C	T	C	-7.82E-02	-1.87E-02	5.78E-01	3.40E-01	FALSE	FALSE	10	36909064	2.35E-02	3.70E-01	TRUE	10	36620136	1.68E-02	1.00	7174	3.48E-06	TRUE	TRUE	482,730	21.57
Phosphatidylcholine (18:0_20:4) levels	Parkinson's disease	rs3019200	A	C	A	C	-1.63E-01	-3.10E-03	8.03E-01	1.90E-01	FALSE	FALSE	11	61249383	2.40E-02	4.77E-02	TRUE	11	61481911	2.13E-02	1.00	7174	2.57E-14	TRUE	TRUE	482,730	58.27
Phosphatidylcholine (18:0_20:4) levels	Parkinson's disease	rs3110971	G	A	G	A	9.57E-02	-4.70E-03	2.08E-01	7.95E-01	FALSE	FALSE	5	38490271	2.11E-02	8.35E-02	TRUE	5	38490169	2.04E-02	0.99	7174	2.72E-06	TRUE	TRUE	482,730	22.04
Phosphatidylcholine (18:0_20:4) levels	Parkinson's disease	rs3741252	T	C	T	C	2.43E-01	-2.38E-02	1.34E-01	9.32E-01	FALSE	FALSE	11	61511498	4.03E-02	2.56E-01	TRUE	11	61744026	2.45E-02	0.99	7174	5.54E-23	TRUE	TRUE	482,730	98.11
Phosphatidylcholine (18:0_20:4) levels	Parkinson's disease	rs4382917	A	G	A	G	1.49E-01	-6.22E-02	2.56E-01	7.72E-01	FALSE	FALSE	11	62221476	3.28E-02	2.90E-02	TRUE	11	62454004	1.97E-02	0.92	7174	4.68E-14	TRUE	TRUE	482,730	57.08
Phosphatidylcholine (18:0_20:4) levels	Parkinson's disease	rs600518	T	A	T	A	-1.01E-01	-1.27E-02	2.32E-01	1.66E-01	TRUE	FALSE	11	75455373	2.83E-02	1.85E-01	TRUE	11	75744328	1.98E-02	0.99	7174	3.49E-07	TRUE	TRUE	482,730	26.00
Phosphatidylcholine (18:0_20:4) levels	Parkinson's disease	rs61873274	G	A	G	A	1.46E-01	-1.66E-02	7.19E-02	9.29E-01	FALSE	FALSE	10	126612734	3.89E-02	1.74E-01	TRUE	10	124924165	3.20E-02	0.98	7174	5.07E-06	TRUE	TRUE	482,730	20.84
Phosphatidylcholine (18:0_20:4) levels	Parkinson's disease	rs6860806	G	A	G	A	7.92E-02	1.51E-02	4.20E-01	4.48E-01	FALSE	FALSE	5	131640536	1.73E-02	4.19E-01	TRUE	5	132304843	1.69E-02	0.99	7174	2.78E-06	TRUE	TRUE	482,730	22.00
Phosphatidylcholine (18:0_20:4) levels	Parkinson's disease	rs7499892	T	C	T	C	-9.58E-02	1.27E-02	1.77E-01	8.22E-01	FALSE	FALSE	16	57006590	2.36E-02	2.30E-01	TRUE	16	56972678	2.16E-02	1.00	7174	9.21E-06	TRUE	TRUE	482,730	19.70
Phosphatidylcholine (18:0_20:4) levels	Parkinson's disease	rs79136768	G	A	G	A	-2.49E-01	-8.48E-02	3.69E-02	9.78E-01	FALSE	FALSE	11	61362439	6.47E-02	7.20E-01	TRUE	11	61594967	4.47E-02	0.98	7174	2.71E-08	TRUE	TRUE	482,730	30.98
Phosphatidylcholine (18:0_20:4) levels	Parkinson's disease	rs938822	C	T	C	T	-1.06E-01	4.20E-03	1.46E-01	8.78E-01	FALSE	FALSE	3	64936419	3.42E-02	4.48E-02	TRUE	3	64950744	2.30E-02	0.99	7174	4.66E-06	TRUE	TRUE	482,730	21.00
Phosphatidylcholine (18:0_20:5) levels	Parkinson's disease	rs102275	C	T	C	T	-4.20E-01	-9.10E-03	4.10E-01	6.60E-01	FALSE	FALSE	11	61557803	1.78E-02	2.14E-01	TRUE	11	61790331	1.65E-02	1.00	7121	3.30E-137	TRUE	TRUE	482,730	649.93
Phosphatidylcholine (18:0_20:5) levels	Parkinson's disease	rs1076708	G	A	G	A	-7.56E-02	-2.37E-02	4.27E-01	5.91E-01	FALSE	FALSE	1	210567430	2.27E-02	5.28E-01	TRUE	1	210394086	1.71E-02	1.00	7121	9.92E-06	TRUE	TRUE	482,730	19.55
Phosphatidylcholine (18:0_20:5) levels	Parkinson's disease	rs12487951	C	T	C	T	-2.28E-01	-2.72E-02	2.78E-02	9.48E-01	FALSE	FALSE	3	169023088	5.07E-02	2.28E-01	TRUE	3	169305300	5.14E-02	0.97	7121	9.17E-06	TRUE	TRUE	482,730	19.70
Phosphatidylcholine (18:0_20:5) levels	Parkinson's disease	rs12656795	C	G	C	G	-7.94E-02	9.20E-03	3.87E-01	3.61E-01	TRUE	FALSE	5	113118963	2.47E-02	1.49E-01	TRUE	5	113783266	1.78E-02	0.93	7121	8.21E-06	TRUE	TRUE	482,730	19.91
Phosphatidylcholine (18:0_20:5) levels	Parkinson's disease	rs145259113	C	T	C	T	-5.28E-01	-1.38E-01	5.52E-03	9.89E-01	FALSE	FALSE	7	147203871	1.16E-01	6.32E-01	TRUE	7	147506779	1.18E-01	0.91	7121	7.30E-06	TRUE	TRUE	482,730	20.14
Phosphatidylcholine (18:0_20:5) levels	Parkinson's disease	rs146438407	T	C	T	C	-4.41E-01	2.72E-02	8.57E-03	9.83E-01	FALSE	FALSE	10	118295604	1.08E-01	9.65E-02	TRUE	10	116536092	9.75E-02	0.85	7121	6.21E-06	TRUE	TRUE	482,730	20.45
Phosphatidylcholine (18:0_20:5) levels	Parkinson's disease	rs187698886	T	G	T	G	-2.00E-01	6.77E-02	3.69E-02	9.77E-01	FALSE	FALSE	6	68265460	1.78E-01	1.52E-01	TRUE	6	67555567	4.51E-02	0.97	7121	9.12E-06	TRUE	TRUE	482,730	19.72
Phosphatidylcholine (18:0_20:5) levels	Parkinson's disease	rs2023988	T	C	T	C	-7.68E-02	-7.10E-03	4.35E-01	5.65E-01	FALSE	FALSE	7	71314281	2.64E-02	1.03E-01	TRUE	7	71849296	1.70E-02	1.00	7121	6.28E-06	TRUE	TRUE	482,730	20.43
Phosphatidylcholine (18:0_20:5) levels	Parkinson's disease	rs2054498	G	A	G	A	-7.81E-02	2.37E-02	5.38E-01	4.63E-01	FALSE	FALSE	4	37393708	2.23E-02	5.41E-01	TRUE	4	37392086	1.67E-02	1.00	7121	2.78E-06	TRUE	TRUE	482,730	21.99
Phosphatidylcholine (18:0_20:5) levels	Parkinson's disease	rs2431163	A	G	A	G	2.64E-01	-3.68E-02	2.67E-02	9.79E-01	FALSE	FALSE	5	174320519	1.55E-01	9.01E-02	TRUE	5	174893516	5.63E-02	0.85	7121	2.81E-06	TRUE	TRUE	482,730	21.98
Phosphatidylcholine (18:0_20:5) levels	Parkinson's disease	rs3019200	A	C	A	C	-1.08E-01	-3.10E-03	8.03E-01	1.90E-01	FALSE	FALSE	11	61249383	2.40E-02	4.77E-02	TRUE	11	61481911	2.15E-02	1.00	7121	4.74E-07	TRUE	TRUE	482,730	25.41
Phosphatidylcholine (18:0_20:5) levels	Parkinson's disease	rs35226034	G	A	G	A	-2.02E-01	3.95E-02	3.99E-02	9.64E-01	FALSE	FALSE	3	139698994	6.21E-02	2.80E-01	TRUE	3	139980152	4.30E-02	0.98	7121	2.57E-06	TRUE	TRUE	482,730	22.15
Phosphatidylcholine (18:0_20:5) levels	Parkinson's disease	rs4908782	T	C	T	C	-7.79E-02	1.20E-02	3.54E-01	6.89E-01	FALSE	FALSE	1	8888114	2.40E-02	2.10E-01	TRUE	1	8828055	1.75E-02	0.99	7121	8.94E-06	TRUE	TRUE	482,730	19.75
Phosphatidylcholine (18:0_20:5) levels	Parkinson's disease	rs508049	T	C	T	C	-1.41E-01	1.46E-02	9.80E-02	9.44E-01	FALSE	FALSE	11	68675497	4.30E-02	1.34E-01	TRUE	11	68908029	2.89E-02	0.96	7121	1.04E-06	TRUE	TRUE	482,730	23.89
Phosphatidylcholine (18:0_20:5) levels	Parkinson's disease	rs61653779	C	T	C	T	2.76E-01	-5.22E-02	2.01E-02	9.74E-01	FALSE	FALSE	16	20580226	5.54E-02	4.61E-01	TRUE	16	20568904	6.04E-02	1.00	7121	5.09E-06	TRUE	TRUE	482,730	20.83
Phosphatidylcholine (18:0_20:5) levels	Parkinson's disease	rs62025936	G	A	G	A	1.23E-01	2.69E-02	1.65E-01	8.40E-01	FALSE	FALSE	16	10528036	3.04E-02	4.25E-01	TRUE	16	10434179	2.28E-02	0.99	7121	6.27E-08	TRUE	TRUE	482,730	29.34
Phosphatidylcholine (18:0_20:5) levels	Parkinson's disease	rs71629271	C	T	C	T	1.68E-01	4.35E-02	6.35E-02	9.46E-01	FALSE	FALSE	3	175813416	5.60E-02	3.60E-01	TRUE	3	176095628	3.59E-02	0.91	7121	2.97E-06	TRUE	TRUE	482,730	21.87
Phosphatidylcholine (18:0_20:5) levels	Parkinson's disease	rs7780677	A	C	A	C	-8.06E-02	2.17E-02	4.09E-01	6.73E-01	FALSE	FALSE	7	132378493	2.38E-02	4.41E-01	TRUE	7	132693734	1.71E-02	1.00	7121	2.52E-06	TRUE	TRUE	482,730	22.18
Phosphatidylcholine (18:0_20:5) levels	Parkinson's disease	rs7936002	G	A	G	A	1.11E-01	-5.27E-02	2.57E-01	7.70E-01	FALSE	FALSE	11	62219029	3.25E-02	9.78E-01	TRUE	11	62451557	1.97E-02	0.93	7121	2.22E-08	TRUE	TRUE	482,730	31.36
Phosphatidylcholine (18:0_20:5) levels	Parkinson's disease	rs80116939	A	G	A	G	-7.65E-01	-1.51E-02	3.53E-03	9.86E-01	FALSE	FALSE	15	94899887	1.15E-01	4.83E-02	TRUE	15	94356658	1.56E-01	0.81	7121	1.00E-06	TRUE	TRUE	482,730	23.97
Phosphatidylcholine (18:0_20:5) levels	Parkinson's disease	rs9380073	T	C	T	C	1.19E-01	-2.80E-02	1.23E-01	7.55E-01	FALSE	FALSE	6	11062150	1.99E-02	7.98E-01	TRUE	6	11061917	2.54E-02	1.00	7121	2.65E-06	TRUE	TRUE	482,730	22.09
Phosphatidylcholine (18:0_20:5) levels	Parkinson's disease	rs9587408	C	T	C	T	9.20E-02	-1.77E-02	2.19E-01	7.31E-01	FALSE	FALSE	13	108248045	2.38E-02	3.40E-01	TRUE	13	107595697	2.01E-02	1.00	7121	4.86E-06	TRUE	TRUE	482,730	20.92
Phosphatidylcholine (18:0_22:5) levels	Parkinson's disease	rs102275	C	T	C	T	-2.85E-01	-9.10E-03	4.10E-01	6.60E-01	FALSE	FALSE	11	61557803	1.78E-02	2.14E-01	TRUE	11	61790331	1.71E-02	1.00	6805	5.38E-61	TRUE	TRUE	482,730	276.97
Phosphatidylcholine (18:0_22:5) levels	Parkinson's disease	rs11178277	G	A	G	A	-7.71E-02	-3.09E-02	4.72E-01	6.08E-01	FALSE	FALSE	12	70935636	2.27E-02	7.61E-01	TRUE	12	70541856	1.71E-02	1.00	6805	6.47E-06	TRUE	TRUE	482,730	20.38
Phosphatidylcholine (18:0_22:5) levels	Parkinson's disease	rs113847854	T	C	T	C	2.31E-01	-8.97E-02	3.03E-02	9.71E-01	FALSE	FALSE	8	69931239	6.87E-02	7.16E-01	TRUE	8	69019004	4.94E-02	0.98	6805	2.85E-06	TRUE	TRUE	482,730	21.95
Phosphatidylcholine (18:0_22:5) levels	Parkinson's disease	rs115247383	T	C	T	C	-2.03E-01	-3.74E-02	4.86E-02	9.70E-01	FALSE	FALSE	3	148000061	7.74E-02	2.01E-01	TRUE	3	148282274	4.25E-02	0.89	6805	1.84E-06	TRUE	TRUE	482,730	22.80
Phosphatidylcholine (18:0_22:5) levels	Parkinson's disease	rs1220131	A	T	A	T	1.08E-01	6.75E-02	2.00E-01	2.43E-01	TRUE	FALSE	2	158567777	2.62E-02	4.99E-03	TRUE	2	157711265	2.14E-02	1.00	6805	4.43E-07	TRUE	TRUE	482,730	25.54
Phosphatidylcholine (18:0_22:5) levels	Parkinson's disease	rs1260326	C	T	C	T	-1.01E-01	-6.78E-02	6.51E-01	4.20E-01	FALSE	FALSE	2	27730940	1.72E-02	4.04E-05	TRUE	2	27508073	1.79E-02	1.00	6805	1.64E-08	TRUE	TRUE	482,730	31.95
Phosphatidylcholine (18:0_22:5) levels	Parkinson's disease	rs13182955	C	T	C	T	7.84E-02	-1.00E-04	3.78E-01	6.50E-01	FALSE	FALSE	5	51399812	1.93E-02	1.22E-03	TRUE	5	52103978	1.77E-02	1.00	6805	9.35E-06	TRUE	TRUE	482,730	19.67
Phosphatidylcholine (18:0_22:5) levels	Parkinson's disease	rs1465411	G	A	G	A	7.90E-02	2.94E-02	5.06E-01	3.33E-01	FALSE	FALSE	5	169582395	2.38E-02	6.63E-01	TRUE	5	170155391	1.71E-02	1.00	6805	3.70E-06	TRUE	TRUE	482,730	21.45
Phosphatidylcholine (18:0_22:5) levels	Parkinson's disease	rs183410044	C	T	C	T	-3.29E-01	1.37E-02	1.59E-02	9.83E-01	FALSE	FALSE	7	54838823	1.00E-01	4.99E-02	TRUE	7	54771130	6.91E-02	0.98	6805	1.90E-06	TRUE	TRUE	482,730	22.73
Phosphatidylcholine (18:0_22:5) levels	Parkinson's disease	rs2236514	G	C	G	C	-8.85E-02	2.80E-03	6.47E-01	6.35E-01	TRUE	FALSE	9	139572068	2.00E-02	5.10E-02	TRUE	9	136677616	1.83E-02	0.95	6805	1.28E-06	TRUE	TRUE	482,730	23.49
Phosphatidylcholine (18:0_22:5) levels	Parkinson's disease	rs2304401	C	T	C	T	-2.27E-01	-3.65E-02	9.68E-01	5.58E-02	FALSE	FALSE	2	11761309	6.76E-02	2.30E-01	TRUE	2	11621183	5.05E-02	0.89	6805	7.43E-06	TRUE	TRUE	482,730	20.11
Phosphatidylcholine (18:0_22:5) levels	Parkinson's disease	rs2613167	T	G	T	G	8.29E-02	-7.59E-02	3.73E-01	6.47E-01	FALSE	FALSE	4	30162762	2.84E-02	3.76E-03	TRUE	4	30161140	1.82E-02	0.97	6805	5.19E-06	TRUE	TRUE	482,730	20.80
Phosphatidylcholine (18:0_22:5) levels	Parkinson's disease	rs2797620	T	C	T	C	-8.58E-02	1.39E-02	3.33E-01	6.30E-01	FALSE	FALSE	1	95441292	2.33E-02	2.59E-01	TRUE	1	94975736	1.81E-02	1.00	6805	2.16E-06	TRUE	TRUE	482,730	22.48
Phosphatidylcholine (18:0_22:5) levels	Parkinson's disease	rs34833702	T	C	T	C	1.97E-01	-1.16E-02	5.21E-02	9.45E-01	FALSE	FALSE	1	218371380	4.99E-02	8.83E-02	TRUE	1	218198038	3.90E-02	0.98	6805	4.44E-07	TRUE	TRUE	482,730	25.54
Phosphatidylcholine (18:0_22:5) levels	Parkinson's disease	rs41352345	T	A	T	A	-1.27E-01	-8.50E-03	9.83E-02	1.55E-01	TRUE	FALSE	11	88215757	3.12E-02	1.05E-01	TRUE	11	88482589	2.87E-02	1.00	6805	9.19E-06	TRUE	TRUE	482,730	19.70
Phosphatidylcholine (18:0_22:5) levels	Parkinson's disease	rs4841133	G	A	G	A	1.21E-01	-6.05E-02	8.52E-01	8.86E-02	FALSE	FALSE	8	9183664	3.10E-02	2.55E-02	TRUE	8	9326154	2.40E-02	1.00	6805	5.19E-07	TRUE	TRUE	482,730	25.24
Phosphatidylcholine (18:0_22:5) levels	Parkinson's disease	rs55982271	C	A	C	A	-2.45E-01	2.17E-01	2.40E-02	9.84E-01	FALSE	FALSE	15	70962597	9.59E-02	1.18E-02	TRUE	15	70670258	5.53E-02	0.98	6805	9.21E-06	TRUE	TRUE	482,730	19.70
Phosphatidylcholine (18:0_22:5) levels	Parkinson's disease	rs58967664	C	T	C	T	1.53E-01	2.51E-02	6.89E-02	9.49E-01	FALSE	FALSE	8	95005265	5.45E-02	1.91E-01	TRUE	8	93993037	3.46E-02	0.94	6805	9.11E-06	TRUE	TRUE	482,730	19.72
Phosphatidylcholine (18:0_22:5) levels	Parkinson's disease	rs628035	T	G	T	G	-9.72E-02	7.00E-03	2.14E-01	8.41E-01	FALSE	FALSE	1	230324145	2.74E-02	9.73E-02	TRUE	1	230188399	2.07E-02	1.00	6805	2.72E-06	TRUE	TRUE	482,730	22.04
Phosphatidylcholine (18:0_22:5) levels	Parkinson's disease	rs7146460	T	C	T	C	-9.56E-02	6.04E-02	2.23E-01	8.27E-01	FALSE	FALSE	14	101361878	3.86E-02	9.30E-01	TRUE	14	100895541	2.15E-02	0.92	6805	8.96E-06	TRUE	TRUE	482,730	19.75
Phosphatidylcholine (18:0_22:5) levels	Parkinson's disease	rs7968201	T	G	T	G	1.00E-01	2.20E-02	2.15E-01	7.23E-01	FALSE	FALSE	12	93932155	2.50E-02	4.22E-01	TRUE	12	93538379	2.10E-02	0.99	6805	1.98E-06	TRUE	TRUE	482,730	22.65
Phosphatidylcholine (18:0_22:5) levels	Parkinson's disease	rs8068517	A	G	A	G	-1.05E-01	-1.87E-02	8.14E-01	1.13E-01	FALSE	FALSE	17	18095978	2.68E-02	3.15E-01	TRUE	17	18192664	2.19E-02	1.00	6805	1.74E-06	TRUE	TRUE	482,730	22.91
Phosphatidylcholine (18:0_22:5) levels	Parkinson's disease	rs9374192	C	T	C	T	-1.02E-01	-2.53E-02	2.72E-01	8.42E-01	FALSE	FALSE	6	110994696	2.67E-02	4.66E-01	TRUE	6	110673493	1.94E-02	1.00	6805	1.69E-07	TRUE	TRUE	482,730	27.41
Phosphatidylcholine (18:0_22:5) levels	Parkinson's disease	rs9918362	C	T	C	T	8.18E-02	5.00E-03	4.58E-01	5.62E-01	FALSE	FALSE	6	10999650	1.69E-02	1.15E-01	TRUE	6	10999417	1.73E-02	1.00	6805	2.24E-06	TRUE	TRUE	482,730	22.42
Phosphatidylcholine (18:0_22:6) levels	Parkinson's disease	rs10150075	T	C	T	C	-1.86E-01	9.26E-02	5.30E-02	9.00E-01	FALSE	FALSE	14	73038517	3.80E-02	7.41E-03	TRUE	14	72571809	3.88E-02	0.95	7004	1.56E-06	TRUE	TRUE	482,730	23.12
Phosphatidylcholine (18:0_22:6) levels	Parkinson's disease	rs10503355	T	G	T	G	-1.92E-01	1.33E-01	4.20E-02	9.71E-01	FALSE	FALSE	8	5953596	6.77E-02	2.49E-02	TRUE	8	6096074	4.23E-02	0.99	7004	5.43E-06	TRUE	TRUE	482,730	20.71
Phosphatidylcholine (18:0_22:6) levels	Parkinson's disease	rs11045913	G	A	G	A	-8.04E-02	1.41E-02	6.47E-01	4.36E-01	FALSE	FALSE	12	21416413	2.17E-02	2.87E-01	TRUE	12	21263479	1.79E-02	0.99	7004	7.32E-06	TRUE	TRUE	482,730	20.14
Phosphatidylcholine (18:0_22:6) levels	Parkinson's disease	rs117061362	T	G	T	G	1.89E-01	1.04E-01	4.49E-02	9.81E-01	FALSE	FALSE	15	101736552	1.03E-01	5.07E-01	TRUE	15	101196347	4.15E-02	0.93	7004	5.13E-06	TRUE	TRUE	482,730	20.82
Phosphatidylcholine (18:0_22:6) levels	Parkinson's disease	rs11792256	G	T	G	T	-1.33E-01	-6.40E-03	1.03E-01	8.68E-01	FALSE	FALSE	9	874977	3.34E-02	7.18E-02	TRUE	9	874977	2.77E-02	0.99	7004	1.73E-06	TRUE	TRUE	482,730	22.91
Phosphatidylcholine (18:0_22:6) levels	Parkinson's disease	rs12050889	A	T	A	T	-8.28E-02	3.49E-02	2.96E-01	3.60E-01	TRUE	FALSE	15	62497951	1.97E-02	3.82E-02	TRUE	15	62205752	1.86E-02	0.99	7004	8.75E-06	TRUE	TRUE	482,730	19.79
Phosphatidylcholine (18:0_22:6) levels	Parkinson's disease	rs139606388	T	C	T	C	-2.44E-01	5.00E-02	2.91E-02	9.81E-01	FALSE	FALSE	5	134186864	6.87E-02	3.31E-01	TRUE	5	134851174	4.99E-02	0.99	7004	1.04E-06	TRUE	TRUE	482,730	23.90
Phosphatidylcholine (18:0_22:6) levels	Parkinson's disease	rs174530	G	A	G	A	-1.67E-01	-1.80E-03	4.14E-01	6.44E-01	FALSE	FALSE	11	61546592	1.79E-02	3.58E-02	TRUE	11	61779120	1.71E-02	1.00	7004	2.21E-22	TRUE	TRUE	482,730	95.35
Phosphatidylcholine (18:0_22:6) levels	Parkinson's disease	rs2067613	C	T	C	T	-8.96E-02	1.67E-02	7.07E-01	3.37E-01	FALSE	FALSE	3	175907342	2.72E-02	2.69E-01	TRUE	3	176189554	1.87E-02	1.00	7004	1.67E-06	TRUE	TRUE	482,730	22.98
Phosphatidylcholine (18:0_22:6) levels	Parkinson's disease	rs3110235	T	G	T	G	9.49E-02	-5.10E-03	2.07E-01	7.95E-01	FALSE	FALSE	5	38486412	2.11E-02	9.21E-02	TRUE	5	38486310	2.08E-02	0.99	7004	5.40E-06	TRUE	TRUE	482,730	20.72
Phosphatidylcholine (18:0_22:6) levels	Parkinson's disease	rs3779874	T	C	T	C	1.01E-01	-1.57E-02	8.28E-01	2.58E-01	FALSE	FALSE	8	65523104	2.00E-02	3.65E-01	TRUE	8	64610547	2.24E-02	1.00	7004	6.12E-06	TRUE	TRUE	482,730	20.48
Phosphatidylcholine (18:0_22:6) levels	Parkinson's disease	rs4841133	G	A	G	A	1.06E-01	-6.05E-02	8.52E-01	8.86E-02	FALSE	FALSE	8	9183664	3.10E-02	2.55E-02	TRUE	8	9326154	2.38E-02	1.00	7004	8.17E-06	TRUE	TRUE	482,730	19.93
Phosphatidylcholine (18:0_22:6) levels	Parkinson's disease	rs4987173	A	G	A	G	1.02E-01	-4.10E-02	3.93E-01	5.44E-01	FALSE	FALSE	6	42931224	1.68E-02	7.33E-03	TRUE	6	42963486	1.73E-02	1.00	7004	3.48E-09	TRUE	TRUE	482,730	34.98
Phosphatidylcholine (18:0_22:6) levels	Parkinson's disease	rs61900787	C	T	C	T	7.87E-02	-2.50E-02	4.15E-01	6.86E-01	FALSE	FALSE	11	42676239	2.40E-02	5.26E-01	TRUE	11	42654689	1.71E-02	1.00	7004	4.34E-06	TRUE	TRUE	482,730	21.14
Phosphatidylcholine (18:0_22:6) levels	Parkinson's disease	rs62345669	A	G	A	G	-4.72E-01	-2.47E-02	7.98E-03	9.77E-01	FALSE	FALSE	4	148397526	9.34E-02	1.02E-01	TRUE	4	147476374	1.00E-01	0.89	7004	2.36E-06	TRUE	TRUE	482,730	22.31
Phosphatidylcholine (18:0_22:6) levels	Parkinson's disease	rs72827432	T	G	T	G	1.11E-01	2.14E-02	1.81E-01	8.89E-01	FALSE	FALSE	17	18069628	2.69E-02	3.70E-01	TRUE	17	18166314	2.18E-02	1.00	7004	3.38E-07	TRUE	TRUE	482,730	26.07
Phosphatidylcholine (18:0_22:6) levels	Parkinson's disease	rs7539908	C	T	C	T	9.84E-02	-2.37E-02	1.78E-01	7.85E-01	FALSE	FALSE	1	54177279	2.43E-02	4.81E-01	TRUE	1	53711606	2.21E-02	1.00	7004	8.56E-06	TRUE	TRUE	482,730	19.84
Phosphatidylcholine (18:0_22:6) levels	Parkinson's disease	rs7974440	A	G	A	G	-1.16E-01	2.27E-02	1.37E-01	8.60E-01	FALSE	FALSE	12	59412802	3.48E-02	2.88E-01	TRUE	12	59019021	2.47E-02	1.00	7004	2.57E-06	TRUE	TRUE	482,730	22.15
Phosphatidylcholine (18:0_22:6) levels	Parkinson's disease	rs9719746	T	C	T	C	-2.50E-01	1.00E-02	2.48E-02	9.74E-01	FALSE	FALSE	7	155085588	7.22E-02	5.07E-02	TRUE	7	155293878	5.54E-02	0.96	7004	6.55E-06	TRUE	TRUE	482,730	20.35
Phosphatidylcholine (18:1_18:1) levels	Parkinson's disease	rs10145681	G	A	G	A	-2.67E-01	5.40E-02	9.76E-01	3.15E-02	FALSE	FALSE	14	69154929	6.54E-02	3.88E-01	TRUE	14	68688212	5.66E-02	0.95	7174	2.32E-06	TRUE	TRUE	482,730	22.35
Phosphatidylcholine (18:1_18:1) levels	Parkinson's disease	rs1077834	C	T	C	T	9.25E-02	-3.20E-03	2.66E-01	7.60E-01	FALSE	FALSE	15	58723479	2.20E-02	5.35E-02	TRUE	15	58431280	1.92E-02	0.98	7174	1.56E-06	TRUE	TRUE	482,730	23.11
Phosphatidylcholine (18:1_18:1) levels	Parkinson's disease	rs10779836	T	C	T	C	9.20E-02	-4.40E-03	7.77E-01	1.96E-01	FALSE	FALSE	1	230303150	2.40E-02	6.80E-02	TRUE	1	230167404	2.00E-02	1.00	7174	4.12E-06	TRUE	TRUE	482,730	21.24
Phosphatidylcholine (18:1_18:1) levels	Parkinson's disease	rs10850057	T	C	T	C	-1.25E-01	9.62E-02	1.00E-01	9.20E-01	FALSE	FALSE	12	112991452	3.65E-02	4.20E-03	TRUE	12	112553648	2.79E-02	0.99	7174	8.27E-06	TRUE	TRUE	482,730	19.90
Phosphatidylcholine (18:1_18:1) levels	Parkinson's disease	rs10889352	C	T	C	T	-1.12E-01	4.00E-04	2.62E-01	6.75E-01	FALSE	FALSE	1	63099023	1.80E-02	6.92E-03	TRUE	1	62633352	1.88E-02	1.00	7174	2.61E-09	TRUE	TRUE	482,730	35.54
Phosphatidylcholine (18:1_18:1) levels	Parkinson's disease	rs113277188	C	G	C	G	-2.91E-01	-7.14E-02	1.76E-02	3.65E-02	TRUE	FALSE	5	150980747	6.59E-02	5.55E-01	TRUE	5	151601186	6.55E-02	0.93	7174	9.15E-06	TRUE	TRUE	482,730	19.71
Phosphatidylcholine (18:1_18:1) levels	Parkinson's disease	rs140268092	T	C	T	C	5.82E-01	1.49E-01	4.67E-03	9.88E-01	FALSE	FALSE	4	29979139	1.62E-01	4.47E-01	TRUE	4	29977517	1.28E-01	0.89	7174	5.51E-06	TRUE	TRUE	482,730	20.68
Phosphatidylcholine (18:1_18:1) levels	Parkinson's disease	rs144512980	C	T	C	T	3.70E-01	4.28E-02	1.52E-02	9.83E-01	FALSE	FALSE	6	18685758	9.43E-02	1.87E-01	TRUE	6	18685527	6.89E-02	0.99	7174	8.17E-08	TRUE	TRUE	482,730	28.82
Phosphatidylcholine (18:1_18:1) levels	Parkinson's disease	rs17231506	T	C	T	C	1.09E-01	1.36E-02	2.78E-01	6.87E-01	FALSE	FALSE	16	56994528	1.81E-02	3.45E-01	TRUE	16	56960616	1.86E-02	1.00	7174	4.04E-09	TRUE	TRUE	482,730	34.69
Phosphatidylcholine (18:1_18:1) levels	Parkinson's disease	rs174574	C	A	C	A	-2.02E-01	6.60E-03	5.91E-01	3.39E-01	FALSE	FALSE	11	61600342	1.77E-02	1.48E-01	TRUE	11	61832870	1.69E-02	1.00	7174	1.28E-32	TRUE	TRUE	482,730	142.86
Phosphatidylcholine (18:1_18:1) levels	Parkinson's disease	rs2126263	A	G	A	G	1.12E-01	-6.82E-02	8.50E-01	9.57E-02	FALSE	FALSE	8	9181611	3.04E-02	1.24E-02	TRUE	8	9324101	2.34E-02	1.00	7174	1.93E-06	TRUE	TRUE	482,730	22.70
Phosphatidylcholine (18:1_18:1) levels	Parkinson's disease	rs3741298	T	C	T	C	-8.94E-02	4.06E-02	7.65E-01	1.98E-01	FALSE	FALSE	11	116657561	2.12E-02	2.77E-02	TRUE	11	116786845	1.96E-02	1.00	7174	5.19E-06	TRUE	TRUE	482,730	20.80
Phosphatidylcholine (18:1_18:1) levels	Parkinson's disease	rs41391244	C	T	C	T	-9.29E-02	1.70E-02	2.20E-01	7.46E-01	FALSE	FALSE	1	111857276	1.92E-02	4.27E-01	TRUE	1	111314654	2.01E-02	1.00	7174	3.80E-06	TRUE	TRUE	482,730	21.39
Phosphatidylcholine (18:1_18:1) levels	Parkinson's disease	rs41441450	T	C	T	C	-3.94E-01	8.84E-02	9.82E-03	9.87E-01	FALSE	FALSE	5	86013053	1.02E-01	4.15E-01	TRUE	5	86717236	8.53E-02	0.99	7174	3.92E-06	TRUE	TRUE	482,730	21.33
Phosphatidylcholine (18:1_18:1) levels	Parkinson's disease	rs4848460	T	C	T	C	1.75E-01	-9.50E-03	5.02E-02	9.27E-01	FALSE	FALSE	2	118399351	4.47E-02	7.99E-02	TRUE	2	117641775	3.84E-02	0.96	7174	5.05E-06	TRUE	TRUE	482,730	20.85
Phosphatidylcholine (18:1_18:1) levels	Parkinson's disease	rs56057593	T	C	T	C	-2.27E-01	-5.67E-02	3.15E-02	9.75E-01	FALSE	FALSE	2	205444472	7.65E-02	3.39E-01	TRUE	2	204579749	4.94E-02	0.93	7174	4.30E-06	TRUE	TRUE	482,730	21.16
Phosphatidylcholine (18:1_18:1) levels	Parkinson's disease	rs73241068	T	C	T	C	-2.18E-01	6.03E-02	3.38E-02	9.59E-01	FALSE	FALSE	4	38038158	5.00E-02	6.42E-01	TRUE	4	38036537	4.57E-02	1.00	7174	1.85E-06	TRUE	TRUE	482,730	22.78
Phosphatidylcholine (18:1_18:1) levels	Parkinson's disease	rs79008894	T	C	T	C	2.09E-01	2.10E-03	3.94E-02	9.50E-01	FALSE	FALSE	4	141018512	6.65E-02	1.09E-02	TRUE	4	140097358	4.59E-02	0.86	7174	5.56E-06	TRUE	TRUE	482,730	20.66
Phosphatidylcholine (18:1_18:2) levels	Parkinson's disease	rs1077835	G	A	G	A	1.25E-01	-3.00E-03	2.66E-01	7.57E-01	FALSE	FALSE	15	58723426	2.21E-02	4.92E-02	TRUE	15	58431227	1.91E-02	0.98	7174	7.94E-11	TRUE	TRUE	482,730	42.40
Phosphatidylcholine (18:1_18:2) levels	Parkinson's disease	rs10889352	C	T	C	T	-1.26E-01	4.00E-04	2.62E-01	6.75E-01	FALSE	FALSE	1	63099023	1.80E-02	6.92E-03	TRUE	1	62633352	1.88E-02	1.00	7174	1.75E-11	TRUE	TRUE	482,730	45.38
Phosphatidylcholine (18:1_18:2) levels	Parkinson's disease	rs11757990	T	C	T	C	4.57E-01	-1.28E-01	7.34E-03	9.74E-01	FALSE	FALSE	6	29764041	6.94E-02	3.25E-02	TRUE	6	29796264	1.03E-01	0.97	7174	9.55E-06	TRUE	TRUE	482,730	19.62
Phosphatidylcholine (18:1_18:2) levels	Parkinson's disease	rs118108980	G	A	G	A	-9.96E-02	-7.80E-02	1.96E-01	9.32E-01	FALSE	FALSE	11	61978495	3.86E-02	2.17E-02	TRUE	11	62211023	2.09E-02	1.00	7174	1.93E-06	TRUE	TRUE	482,730	22.70
Phosphatidylcholine (18:1_18:2) levels	Parkinson's disease	rs12798268	C	A	C	A	-1.07E-01	4.91E-02	8.59E-01	1.59E-01	FALSE	FALSE	11	107739911	3.07E-02	9.59E-01	TRUE	11	107869185	2.39E-02	1.00	7174	7.19E-06	TRUE	TRUE	482,730	20.17
Phosphatidylcholine (18:1_18:2) levels	Parkinson's disease	rs13128807	A	G	A	G	-1.01E-01	-1.11E-02	1.96E-01	7.50E-01	FALSE	FALSE	4	170232880	2.54E-02	1.78E-01	TRUE	4	169311729	2.11E-02	1.00	7174	1.81E-06	TRUE	TRUE	482,730	22.82
Phosphatidylcholine (18:1_18:2) levels	Parkinson's disease	rs142689129	T	C	T	C	5.80E-01	8.70E-03	5.39E-03	9.84E-01	FALSE	FALSE	6	18588134	9.57E-02	3.27E-02	TRUE	6	18587903	1.16E-01	0.98	7174	6.42E-07	TRUE	TRUE	482,730	24.82
Phosphatidylcholine (18:1_18:2) levels	Parkinson's disease	rs147761802	C	G	C	G	-1.81E-01	-1.60E-02	4.96E-02	5.42E-02	TRUE	FALSE	7	5850106	5.11E-02	1.23E-01	TRUE	7	5810475	3.92E-02	0.95	7174	4.04E-06	TRUE	TRUE	482,730	21.28
Phosphatidylcholine (18:1_18:2) levels	Parkinson's disease	rs1601935	T	G	T	G	-1.06E-01	-3.28E-02	6.11E-01	3.41E-01	FALSE	FALSE	15	58671765	1.86E-02	3.89E-02	TRUE	15	58379566	1.70E-02	0.99	7174	5.31E-10	TRUE	TRUE	482,730	38.67
Phosphatidylcholine (18:1_18:2) levels	Parkinson's disease	rs17231506	T	C	T	C	1.20E-01	1.36E-02	2.78E-01	6.87E-01	FALSE	FALSE	16	56994528	1.81E-02	3.45E-01	TRUE	16	56960616	1.85E-02	1.00	7174	1.06E-10	TRUE	TRUE	482,730	41.83
Phosphatidylcholine (18:1_18:2) levels	Parkinson's disease	rs174574	C	A	C	A	-3.80E-01	6.60E-03	5.91E-01	3.39E-01	FALSE	FALSE	11	61600342	1.77E-02	1.48E-01	TRUE	11	61832870	1.64E-02	1.00	7174	2.79E-114	TRUE	TRUE	482,730	535.27
Phosphatidylcholine (18:1_18:2) levels	Parkinson's disease	rs28844909	A	G	A	G	-1.53E-01	-4.91E-02	6.66E-02	9.73E-01	FALSE	FALSE	4	146628373	8.04E-02	2.67E-01	TRUE	4	145707221	3.40E-02	0.95	7174	7.07E-06	TRUE	TRUE	482,730	20.20

Phosphatidylcholine (18:1_18:2) levels	Parkinson's disease	rs4944732	T	C	T	C	1.09E-01	4.00E-03	1.35E-01	7.93E-01	FALSE	FALSE	11	72446182	2.43E-02	6.03E-02	TRUE	11	72735137	2.43E-02	0.99	7174	8.27E-06	TRUE	TRUE	482,730	19.90
Phosphatidylcholine (18:1_18:2) levels	Parkinson's disease	rs564216	C	T	C	T	-1.68E-01	-4.50E-02	5.50E-02	9.28E-01	FALSE	FALSE	11	125965332	3.75E-02	6.39E-01	TRUE	11	126095437	3.66E-02	0.98	7174	4.51E-06	TRUE	TRUE	482,730	21.06
Phosphatidylcholine (18:1_18:2) levels	Parkinson's disease	rs613808	G	A	G	A	-9.11E-02	5.14E-02	6.19E-01	2.94E-01	FALSE	FALSE	11	116710968	1.90E-02	3.41E-03	TRUE	11	116840252	1.73E-02	1.00	7174	1.43E-07	TRUE	TRUE	482,730	27.74
Phosphatidylcholine (18:1_18:2) levels	Parkinson's disease	rs62004153	A	G	A	G	-1.31E-01	1.96E-01	9.13E-01	8.49E-02	FALSE	FALSE	15	59491170	1.64E-01	6.37E-01	TRUE	15	59198971	2.96E-02	1.00	7174	9.20E-06	TRUE	TRUE	482,730	19.70
Phosphatidylcholine (18:1_18:2) levels	Parkinson's disease	rs6498540	G	A	G	A	7.93E-02	1.82E-02	3.34E-01	6.94E-01	FALSE	FALSE	16	15130594	1.84E-02	4.93E-01	TRUE	16	15036737	1.77E-02	0.99	7174	7.68E-06	TRUE	TRUE	482,730	20.04
Phosphatidylcholine (18:1_18:2) levels	Parkinson's disease	rs7036554	C	T	C	T	9.06E-02	-3.60E-03	3.50E-01	6.32E-01	FALSE	FALSE	9	14382620	2.36E-02	5.54E-02	TRUE	9	14382621	1.75E-02	0.97	7174	2.43E-07	TRUE	TRUE	482,730	26.70
Phosphatidylcholine (18:1_18:2) levels	Parkinson's disease	rs78882831	T	C	T	C	2.43E-01	2.05E-01	2.51E-02	9.81E-01	FALSE	FALSE	5	95464280	9.18E-02	1.27E-02	TRUE	5	96128576	5.44E-02	0.97	7174	8.43E-06	TRUE	TRUE	482,730	19.87
Phosphatidylcholine (18:1_18:2) levels	Parkinson's disease	rs9789400	T	C	T	C	-8.87E-02	1.40E-03	2.65E-01	6.15E-01	FALSE	FALSE	2	57917222	2.02E-02	2.54E-02	TRUE	2	57690087	1.89E-02	0.99	7174	2.74E-06	TRUE	TRUE	482,730	22.03
Phosphatidylcholine (18:1_18:3) levels	Parkinson's disease	rs10769099	A	G	A	G	8.83E-02	2.20E-03	3.37E-01	6.87E-01	FALSE	FALSE	11	5607814	1.83E-02	4.42E-02	TRUE	11	5586584	1.88E-02	0.99	6339	2.73E-06	TRUE	TRUE	482,730	22.03
Phosphatidylcholine (18:1_18:3) levels	Parkinson's disease	rs1116931	A	C	A	C	-9.25E-02	-2.99E-02	2.72E-01	6.95E-01	FALSE	FALSE	3	100589330	2.40E-02	6.72E-01	TRUE	3	100870486	1.97E-02	1.00	6339	2.78E-06	TRUE	TRUE	482,730	22.00
Phosphatidylcholine (18:1_18:3) levels	Parkinson's disease	rs114797690	T	C	T	C	2.85E-01	2.25E-02	2.34E-02	9.58E-01	FALSE	FALSE	1	99063537	6.22E-02	1.44E-01	TRUE	1	98597981	6.22E-02	0.87	6339	4.84E-06	TRUE	TRUE	482,730	20.93
Phosphatidylcholine (18:1_18:3) levels	Parkinson's disease	rs116910056	T	A	T	A	-4.18E-01	-5.34E-02	8.91E-03	1.86E-02	TRUE	FALSE	11	79877134	8.70E-02	2.68E-01	TRUE	11	80166090	9.46E-02	0.99	6339	9.84E-06	TRUE	TRUE	482,730	19.57
Phosphatidylcholine (18:1_18:3) levels	Parkinson's disease	rs140885981	A	G	A	G	-4.77E-01	1.05E-01	7.68E-03	9.87E-01	FALSE	FALSE	16	75336313	1.54E-01	3.04E-01	TRUE	16	75302415	1.03E-01	0.89	6339	3.89E-06	TRUE	TRUE	482,730	21.36
Phosphatidylcholine (18:1_18:3) levels	Parkinson's disease	rs144128487	G	C	G	C	-3.99E-01	-1.40E-01	1.20E-02	1.83E-02	TRUE	FALSE	6	97530244	9.38E-02	8.67E-01	TRUE	6	97082368	8.21E-02	0.97	6339	1.23E-06	TRUE	TRUE	482,730	23.57
Phosphatidylcholine (18:1_18:3) levels	Parkinson's disease	rs148879093	A	G	A	G	-1.53E-01	-1.14E-01	7.87E-02	9.63E-01	FALSE	FALSE	12	96514618	7.12E-02	9.65E-01	TRUE	12	96120840	3.43E-02	0.91	6339	7.84E-06	TRUE	TRUE	482,730	20.01
Phosphatidylcholine (18:1_18:3) levels	Parkinson's disease	rs149851240	C	G	C	G	1.95E-01	2.34E-02	4.61E-02	2.87E-02	TRUE	FALSE	2	41784136	7.30E-02	1.26E-01	TRUE	2	41556996	4.29E-02	0.98	6339	5.55E-06	TRUE	TRUE	482,730	20.67
Phosphatidylcholine (18:1_18:3) levels	Parkinson's disease	rs17208010	T	C	T	C	1.44E-01	5.42E-02	8.36E-02	9.17E-01	FALSE	FALSE	5	139332485	3.78E-02	8.18E-01	TRUE	5	139952900	3.25E-02	0.96	6339	8.83E-06	TRUE	TRUE	482,730	19.78
Phosphatidylcholine (18:1_18:3) levels	Parkinson's disease	rs17231506	T	C	T	C	8.90E-02	1.36E-02	2.78E-01	6.87E-01	FALSE	FALSE	16	56994528	1.81E-02	3.45E-01	TRUE	16	56960616	1.98E-02	1.00	6339	6.76E-06	TRUE	TRUE	482,730	20.29
Phosphatidylcholine (18:1_18:3) levels	Parkinson's disease	rs174601	T	C	T	C	8.01E-02	-3.70E-03	4.19E-01	6.38E-01	FALSE	FALSE	11	61623140	1.79E-02	7.75E-02	TRUE	11	61855668	1.80E-02	1.00	6339	8.29E-06	TRUE	TRUE	482,730	19.90
Phosphatidylcholine (18:1_18:3) levels	Parkinson's disease	rs2774509	C	G	C	G	-8.38E-02	-3.69E-02	6.64E-01	7.01E-01	TRUE	FALSE	6	6412075	2.44E-02	8.85E-01	TRUE	6	6411842	1.87E-02	1.00	6339	7.22E-06	TRUE	TRUE	482,730	20.16
Phosphatidylcholine (18:1_18:3) levels	Parkinson's disease	rs9480321	T	A	T	A	-2.04E-01	2.13E-02	4.97E-02	4.84E-02	TRUE	FALSE	6	156699521	5.53E-02	1.55E-01	TRUE	6	156378387	4.09E-02	0.97	6339	6.16E-07	TRUE	TRUE	482,730	24.91
Phosphatidylcholine (18:1_18:3) levels	Parkinson's disease	rs9559745	C	T	C	T	4.26E-01	4.29E-02	8.58E-03	9.78E-01	FALSE	FALSE	13	110861989	8.17E-02	2.22E-01	TRUE	13	110209642	9.55E-02	0.95	6339	8.58E-06	TRUE	TRUE	482,730	19.84
Phosphatidylcholine (18:1_20:2) levels	Parkinson's disease	rs10846753	G	T	G	T	1.06E-01	2.38E-02	2.35E-01	7.72E-01	FALSE	FALSE	12	125321461	2.19E-02	5.55E-01	TRUE	12	124836915	2.15E-02	1.00	5852	8.21E-07	TRUE	TRUE	482,730	24.36
Phosphatidylcholine (18:1_20:2) levels	Parkinson's disease	rs114139553	A	G	A	G	-3.73E-01	3.79E-02	1.38E-02	9.65E-01	FALSE	FALSE	1	104407210	7.03E-02	2.29E-01	TRUE	1	103864588	8.15E-02	0.96	5852	4.63E-06	TRUE	TRUE	482,730	21.02
Phosphatidylcholine (18:1_20:2) levels	Parkinson's disease	rs116922907	A	G	A	G	2.14E-01	-5.93E-02	4.16E-02	9.44E-01	FALSE	FALSE	7	106630020	4.26E-02	7.86E-01	TRUE	7	106989575	4.69E-02	0.98	5852	5.03E-06	TRUE	TRUE	482,730	20.86
Phosphatidylcholine (18:1_20:2) levels	Parkinson's disease	rs117534325	G	A	G	A	6.39E-01	-9.84E-02	4.85E-03	9.88E-01	FALSE	FALSE	15	70698378	1.45E-01	3.02E-01	TRUE	15	70406039	1.35E-01	0.95	5852	2.47E-06	TRUE	TRUE	482,730	22.23
Phosphatidylcholine (18:1_20:2) levels	Parkinson's disease	rs12113068	T	G	T	G	-2.75E-01	2.23E-02	2.51E-02	9.50E-01	FALSE	FALSE	7	155227866	5.51E-02	1.64E-01	TRUE	7	155435171	6.21E-02	0.92	5852	9.34E-06	TRUE	TRUE	482,730	19.68
Phosphatidylcholine (18:1_20:2) levels	Parkinson's disease	rs12417747	A	G	A	G	1.90E-01	-1.65E-02	1.15E-01	8.54E-01	FALSE	FALSE	11	61828394	2.86E-02	2.49E-01	TRUE	11	62060922	2.90E-02	0.99	5852	5.83E-11	TRUE	TRUE	482,730	43.02
Phosphatidylcholine (18:1_20:2) levels	Parkinson's disease	rs174580	G	A	G	A	4.31E-01	-7.10E-03	4.10E-01	6.58E-01	FALSE	FALSE	11	61606642	1.77E-02	1.61E-01	TRUE	11	61839170	1.79E-02	1.00	5852	1.49E-122	TRUE	TRUE	482,730	580.89
Phosphatidylcholine (18:1_20:2) levels	Parkinson's disease	rs17543793	T	A	T	A	3.01E-01	7.90E-03	2.02E-02	1.45E-02	TRUE	FALSE	5	17302143	1.31E-01	2.14E-02	TRUE	5	17302034	6.68E-02	0.94	5852	6.64E-06	TRUE	TRUE	482,730	20.33
Phosphatidylcholine (18:1_20:2) levels	Parkinson's disease	rs1800588	T	C	T	C	1.13E-01	-3.30E-03	2.58E-01	7.75E-01	FALSE	FALSE	15	58723675	2.12E-02	5.73E-02	TRUE	15	58431476	2.15E-02	0.99	5852	1.58E-07	TRUE	TRUE	482,730	27.55
Phosphatidylcholine (18:1_20:2) levels	Parkinson's disease	rs265114	T	C	T	C	1.78E-01	3.69E-02	7.37E-02	8.92E-01	FALSE	FALSE	1	217630628	3.75E-02	4.88E-01	TRUE	1	217457286	3.65E-02	0.96	5852	1.06E-06	TRUE	TRUE	482,730	23.86
Phosphatidylcholine (18:1_20:2) levels	Parkinson's disease	rs35195855	T	C	T	C	1.30E-01	-4.73E-02	1.48E-01	8.08E-01	FALSE	FALSE	11	36116108	3.14E-02	8.78E-01	TRUE	11	36094558	2.78E-02	0.87	5852	3.10E-06	TRUE	TRUE	482,730	21.79
Phosphatidylcholine (18:1_20:2) levels	Parkinson's disease	rs56208677	T	C	T	C	1.82E-01	1.66E-02	7.77E-02	9.36E-01	FALSE	FALSE	16	57010232	4.08E-02	1.65E-01	TRUE	16	56976320	3.53E-02	0.96	5852	2.63E-07	TRUE	TRUE	482,730	26.56
Phosphatidylcholine (18:1_20:2) levels	Parkinson's disease	rs61893829	T	C	T	C	-1.57E-01	1.95E-02	8.11E-02	9.54E-01	FALSE	FALSE	11	62803942	5.09E-02	1.53E-01	TRUE	11	63036470	3.40E-02	0.97	5852	3.96E-06	TRUE	TRUE	482,730	21.31
Phosphatidylcholine (18:1_20:2) levels	Parkinson's disease	rs656095	G	A	G	A	-1.30E-01	2.22E-02	2.27E-01	8.25E-01	FALSE	FALSE	11	75445338	2.79E-02	3.70E-01	TRUE	11	75734293	2.22E-02	1.00	5852	4.24E-09	TRUE	TRUE	482,730	34.61
Phosphatidylcholine (18:1_20:2) levels	Parkinson's disease	rs6944011	G	A	G	A	1.02E-01	-7.00E-02	2.05E-01	7.75E-01	FALSE	FALSE	7	6371947	2.65E-02	4.13E-03	TRUE	7	6332316	2.27E-02	0.97	5852	7.50E-06	TRUE	TRUE	482,730	20.09
Phosphatidylcholine (18:1_20:2) levels	Parkinson's disease	rs6984305	T	A	T	A	1.22E-01	5.12E-02	8.37E-01	8.93E-01	TRUE	FALSE	8	9178268	2.86E-02	3.67E-02	TRUE	8	9320758	2.51E-02	1.00	5852	1.30E-06	TRUE	TRUE	482,730	23.47
Phosphatidylcholine (18:1_20:2) levels	Parkinson's disease	rs7041283	C	T	C	T	-1.16E-01	-5.30E-03	2.45E-01	6.69E-01	FALSE	FALSE	9	81057064	2.38E-02	8.32E-02	TRUE	9	78442148	2.14E-02	1.00	5852	5.79E-08	TRUE	TRUE	482,730	29.50
Phosphatidylcholine (18:1_20:2) levels	Parkinson's disease	rs7176661	C	T	C	T	8.40E-02	1.12E-02	5.96E-01	4.62E-01	FALSE	FALSE	15	96081909	2.25E-02	2.10E-01	TRUE	15	95538680	1.89E-02	0.99	5852	8.55E-06	TRUE	TRUE	482,730	19.84
Phosphatidylcholine (18:1_20:2) levels	Parkinson's disease	rs74089903	A	G	A	G	-5.39E-01	1.16E-01	8.80E-03	9.77E-01	FALSE	FALSE	12	54554349	7.73E-02	8.68E-01	TRUE	12	54160565	1.03E-01	0.96	5852	1.57E-07	TRUE	TRUE	482,730	27.56
Phosphatidylcholine (18:1_20:2) levels	Parkinson's disease	rs80201535	A	G	A	G	-2.47E-01	-2.03E-01	3.08E-02	9.85E-01	FALSE	FALSE	7	10865777	1.01E-01	2.21E-02	TRUE	7	10826150	5.35E-02	0.97	5852	4.14E-06	TRUE	TRUE	482,730	21.24
Phosphatidylcholine (18:1_20:2) levels	Parkinson's disease	rs9384216	C	T	C	T	-1.39E-01	-1.71E-02	9.71E-02	9.28E-01	FALSE	FALSE	6	154740878	4.34E-02	1.59E-01	TRUE	6	154419744	3.08E-02	1.00	5852	7.05E-06	TRUE	TRUE	482,730	20.22
Phosphatidylcholine (18:1_20:3) levels	Parkinson's disease	rs10504795	G	C	G	C	-9.50E-02	6.00E-04	2.58E-01	1.66E-01	TRUE	FALSE	8	85134654	3.02E-02	6.56E-03	TRUE	8	84222419	1.91E-02	1.00	7171	6.32E-07	TRUE	TRUE	482,730	24.85
Phosphatidylcholine (18:1_20:3) levels	Parkinson's disease	rs10889335	G	A	G	A	-9.79E-02	3.10E-03	2.85E-01	6.69E-01	FALSE	FALSE	1	62960101	1.80E-02	6.33E-02	TRUE	1	62494430	1.84E-02	1.00	7171	1.06E-07	TRUE	TRUE	482,730	28.32
Phosphatidylcholine (18:1_20:3) levels	Parkinson's disease	rs113366516	T	G	T	G	2.97E-01	-9.44E-02	1.70E-02	9.91E-01	FALSE	FALSE	6	138469237	1.90E-01	2.08E-01	TRUE	6	138148100	6.61E-02	0.93	7171	7.07E-06	TRUE	TRUE	482,730	20.20
Phosphatidylcholine (18:1_20:3) levels	Parkinson's disease	rs116647148	G	A	G	A	1.78E-01	-2.20E-02	5.21E-02	9.44E-01	FALSE	FALSE	4	173351004	5.53E-02	1.60E-01	TRUE	4	172429853	3.94E-02	0.91	7171	6.14E-06	TRUE	TRUE	482,730	20.47
Phosphatidylcholine (18:1_20:3) levels	Parkinson's disease	rs12198590	C	G	C	G	5.13E-01	-4.76E-02	5.56E-03	1.64E-02	TRUE	FALSE	6	18489660	9.68E-02	2.05E-01	TRUE	6	18489429	1.14E-01	0.98	7171	7.34E-06	TRUE	TRUE	482,730	20.13
Phosphatidylcholine (18:1_20:3) levels	Parkinson's disease	rs17092227	C	T	C	T	1.94E-01	-4.60E-02	4.42E-02	9.73E-01	FALSE	FALSE	14	95900494	6.97E-02	2.93E-01	TRUE	14	95434157	4.11E-02	0.97	7171	2.53E-06	TRUE	TRUE	482,730	22.18
Phosphatidylcholine (18:1_20:3) levels	Parkinson's disease	rs182828998	C	A	C	A	-4.92E-01	3.73E-02	6.71E-03	9.84E-01	FALSE	FALSE	4	116114816	9.57E-02	1.57E-01	TRUE	4	115193660	1.05E-01	0.98	7171	2.52E-06	TRUE	TRUE	482,730	22.19
Phosphatidylcholine (18:1_20:3) levels	Parkinson's disease	rs2797620	T	C	T	C	-9.25E-02	1.39E-02	3.33E-01	6.30E-01	FALSE	FALSE	1	95441292	2.33E-02	2.59E-01	TRUE	1	94975736	1.76E-02	1.00	7171	1.49E-07	TRUE	TRUE	482,730	27.65
Phosphatidylcholine (18:1_20:3) levels	Parkinson's disease	rs56374730	G	T	G	T	8.14E-02	-3.00E-02	2.96E-01	7.43E-01	FALSE	FALSE	16	15867652	2.56E-02	6.19E-01	TRUE	16	15773795	1.84E-02	0.99	7171	9.83E-06	TRUE	TRUE	482,730	19.57
Phosphatidylcholine (18:1_20:3) levels	Parkinson's disease	rs59739041	C	T	C	T	1.73E-01	2.12E-02	4.79E-02	9.50E-01	FALSE	FALSE	15	58661083	5.23E-02	1.64E-01	TRUE	15	58368884	3.88E-02	0.99	7171	8.86E-06	TRUE	TRUE	482,730	19.77
Phosphatidylcholine (18:1_20:3) levels	Parkinson's disease	rs61896141	C	A	C	A	2.24E-01	-3.28E-02	1.13E-01	8.31E-01	FALSE	FALSE	11	61556039	2.26E-02	8.35E-01	TRUE	11	61788567	2.64E-02	1.00	7171	3.04E-17	TRUE	TRUE	482,730	71.67
Phosphatidylcholine (18:1_20:3) levels	Parkinson's disease	rs6498540	G	A	G	A	-1.55E-01	1.82E-02	3.34E-01	6.94E-01	FALSE	FALSE	16	15130594	1.84E-02	4.93E-01	TRUE	16	15036737	1.77E-02	0.99	7171	3.06E-18	TRUE	TRUE	482,730	76.26
Phosphatidylcholine (18:1_20:3) levels	Parkinson's disease	rs6985657	G	A	G	A	-1.37E-01	1.69E-02	9.01E-02	9.21E-01	FALSE	FALSE	8	59923386	4.21E-02	1.62E-01	TRUE	8	59010827	2.93E-02	0.99	7171	2.77E-06	TRUE	TRUE	482,730	22.00
Phosphatidylcholine (18:1_20:3) levels	Parkinson's disease	rs7139079	A	G	A	G	-8.30E-02	1.53E-02	5.47E-01	4.21E-01	FALSE	FALSE	12	121415293	1.78E-02	4.09E-01	TRUE	12	120977490	1.68E-02	1.00	7171	7.63E-07	TRUE	TRUE	482,730	24.49
Phosphatidylcholine (18:1_20:3) levels	Parkinson's disease	rs72974647	G	A	G	A	1.36E-01	4.59E-02	8.22E-02	9.34E-01	FALSE	FALSE	6	123648480	3.92E-02	6.18E-01	TRUE	6	123327335	3.01E-02	1.00	7171	6.80E-06	TRUE	TRUE	482,730	20.28
Phosphatidylcholine (18:1_20:3) levels	Parkinson's disease	rs75926193	G	A	G	A	2.60E-01	4.33E-02	2.77E-02	9.82E-01	FALSE	FALSE	11	118871118	9.76E-02	1.82E-01	TRUE	11	119000408	5.16E-02	0.93	7171	4.98E-07	TRUE	TRUE	482,730	25.32
Phosphatidylcholine (18:1_20:3) levels	Parkinson's disease	rs7897746	T	C	T	C	1.33E-01	1.47E-02	9.03E-01	1.32E-01	FALSE	FALSE	10	5620399	3.61E-02	1.64E-01	TRUE	10	5578436	2.86E-02	0.99	7171	3.44E-06	TRUE	TRUE	482,730	21.59
Phosphatidylcholine (18:1_20:3) levels	Parkinson's disease	rs79047909	G	C	G	C	-4.97E-01	6.88E-02	6.23E-03	9.10E-03	TRUE	FALSE	2	10183140	1.38E-01	2.08E-01	TRUE	2	10043013	1.07E-01	0.96	7171	3.20E-06	TRUE	TRUE	482,730	21.72
Phosphatidylcholine (18:1_20:3) levels	Parkinson's disease	rs822798	G	A	G	A	8.48E-02	3.87E-02	4.16E-01	5.63E-01	FALSE	FALSE	3	23013329	2.25E-02	4.27E-02	TRUE	3	22971838	1.68E-02	1.00	7171	4.93E-07	TRUE	TRUE	482,730	25.34
Phosphatidylcholine (18:1_20:4) levels	Parkinson's disease	rs1077834	C	T	C	T	9.86E-02	-3.20E-03	2.66E-01	7.60E-01	FALSE	FALSE	15	58723479	2.20E-02	5.35E-02	TRUE	15	58431280	1.92E-02	0.98	7173	2.88E-07	TRUE	TRUE	482,730	26.38
Phosphatidylcholine (18:1_20:4) levels	Parkinson's disease	rs10889353	C	A	C	A	-8.53E-02	-5.00E-04	2.62E-01	6.75E-01	FALSE	FALSE	1	63118196	1.80E-02	9.57E-03	TRUE	1	62652525	1.88E-02	1.00	7173	5.95E-06	TRUE	TRUE	482,730	20.54
Phosphatidylcholine (18:1_20:4) levels	Parkinson's disease	rs113394924	G	T	G	T	1.87E-01	-6.22E-02	8.73E-02	9.66E-01	FALSE	FALSE	11	62082293	5.88E-02	5.37E-01	TRUE	11	62314821	2.97E-02	0.99	7173	3.52E-10	TRUE	TRUE	482,730	39.47
Phosphatidylcholine (18:1_20:4) levels	Parkinson's disease	rs113913626	T	C	T	C	-2.13E-01	-1.93E-01	3.23E-02	9.82E-01	FALSE	FALSE	12	76649394	8.81E-02	1.42E-02	TRUE	12	76255614	4.81E-02	0.94	7173	9.99E-06	TRUE	TRUE	482,730	19.54
Phosphatidylcholine (18:1_20:4) levels	Parkinson's disease	rs116814948	C	T	C	T	-4.25E-01	-1.26E-01	8.67E-03	9.75E-01	FALSE	FALSE	6	2823016	8.35E-02	8.77E-01	TRUE	6	2822782	9.40E-02	0.90	7173	6.20E-06	TRUE	TRUE	482,730	20.45
Phosphatidylcholine (18:1_20:4) levels	Parkinson's disease	rs147549994	T	G	T	G	2.32E-01	-1.38E-02	3.59E-02	9.86E-01	FALSE	FALSE	16	1989265	1.01E-01	4.97E-02	TRUE	16	1939264	4.54E-02	0.95	7173	3.16E-07	TRUE	TRUE	482,730	26.20
Phosphatidylcholine (18:1_20:4) levels	Parkinson's disease	rs174537	T	G	T	G	-5.08E-01	-2.50E-03	4.08E-01	6.71E-01	FALSE	FALSE	11	61552680	1.79E-02	5.23E-02	TRUE	11	61785208	1.60E-02	1.00	7173	1.00E-200	TRUE	TRUE	482,730	1007.10
Phosphatidylcholine (18:1_20:4) levels	Parkinson's disease	rs1917701	A	T	A	T	1.56E-01	-2.66E-02	9.29E-01	9.54E-01	TRUE	FALSE	2	141836548	5.28E-02	2.12E-01	TRUE	2	141078979	3.50E-02	0.83	7173	7.78E-06	TRUE	TRUE	482,730	20.02
Phosphatidylcholine (18:1_20:4) levels	Parkinson's disease	rs2943813	C	T	C	T	-1.30E-01	-4.50E-03	7.83E-01	2.16E-01	FALSE	FALSE	11	61258468	2.30E-02	7.25E-02	TRUE	11	61490996	2.04E-02	1.00	7173	2.26E-10	TRUE	TRUE	482,730	40.33
Phosphatidylcholine (18:1_20:4) levels	Parkinson's disease	rs3018617	G	A	G	A	-9.18E-02	3.21E-02	3.02E-01	6.78E-01	FALSE	FALSE	11	62202934	3.28E-02	4.84E-01	TRUE	11	62435462	1.88E-02	0.94	7173	1.08E-06	TRUE	TRUE	482,730	23.81
Phosphatidylcholine (18:1_20:4) levels	Parkinson's disease	rs4628166	G	C	G	C	-7.61E-02	-3.56E-02	6.34E-01	6.96E-01	TRUE	FALSE	7	39304641	2.41E-02	8.54E-01	TRUE	7	39265042	1.71E-02	1.00	7173	9.16E-06	TRUE	TRUE	482,730	19.70
Phosphatidylcholine (18:1_20:4) levels	Parkinson's disease	rs4846905	G	A	G	A	9.42E-02	-2.83E-02	7.68E-01	1.86E-01	FALSE	FALSE	1	230279582	2.86E-02	4.91E-01	TRUE	1	230143835	1.96E-02	1.00	7173	1.50E-06	TRUE	TRUE	482,730	23.19
Phosphatidylcholine (18:1_20:4) levels	Parkinson's disease	rs531117	T	C	T	C	-1.14E-01	1.42E-02	2.32E-01	8.33E-01	FALSE	FALSE	11	75456134	2.83E-02	2.11E-01	TRUE	11	75745089	1.98E-02	0.99	7173	9.96E-09	TRUE	TRUE	482,730	32.93
Phosphatidylcholine (18:1_20:4) levels	Parkinson's disease	rs61978272	T	C	T	C	-9.81E-02	-1.19E-02	2.82E-01	7.45E-01	FALSE	FALSE	14	94989658	2.65E-02	1.85E-01	TRUE	14	94523321	1.86E-02	0.98	7173	1.41E-07	TRUE	TRUE	482,730	27.76
Phosphatidylcholine (18:1_20:4) levels	Parkinson's disease	rs6850591	C	T	C	T	-8.07E-02	-2.44E-02	5.41E-01	4.52E-01	FALSE	FALSE	4	140949552	2.25E-02	5.57E-01	TRUE	4	140028398	1.68E-02	1.00	7173	1.64E-06	TRUE	TRUE	482,730	23.01
Phosphatidylcholine (18:1_20:4) levels	Parkinson's disease	rs73176681	G	A	G	A	1.84E-01	3.50E-02	4.56E-02	9.65E-01	FALSE	FALSE	13	32074965	6.22E-02	2.41E-01	TRUE	13	31500828	4.04E-02	0.98	7173	5.15E-06	TRUE	TRUE	482,730	20.81
Phosphatidylcholine (18:1_20:4) levels	Parkinson's disease	rs75976069	C	T	C	T	-2.61E-01	1.14E-01	2.62E-02	9.79E-01	FALSE	FALSE	14	79686603	8.59E-02	7.37E-01	TRUE	14	79220260	5.38E-02	0.94	7173	1.31E-06	TRUE	TRUE	482,730	23.45
Phosphatidylcholine (18:1_20:4) levels	Parkinson's disease	rs76582138	G	A	G	A	-4.58E-01	1.06E-01	7.09E-03	9.82E-01	FALSE	FALSE	4	116245745	9.32E-02	5.91E-01	TRUE	4	115324589	1.02E-01	0.97	7173	6.56E-06	TRUE	TRUE	482,730	20.35
Phosphatidylcholine (18:1_20:4) levels	Parkinson's disease	rs79113397	C	T	C	T	9.95E-02	-6.40E-03	2.29E-01	8.86E-01	FALSE	FALSE	2	102186831	3.56E-02	6.67E-02	TRUE	2	101570369	2.01E-02	0.99	7173	7.59E-07	TRUE	TRUE	482,730	24.50
Phosphatidylcholine (18:2_18:2) levels	Parkinson's disease	rs1077835	G	A	G	A	1.15E-01	-3.00E-03	2.66E-01	7.57E-01	FALSE	FALSE	15	58723426	2.21E-02	4.92E-02	TRUE	15	58431227	1.92E-02	0.98	7174	2.66E-09	TRUE	TRUE	482,730	35.50
Phosphatidylcholine (18:2_18:2) levels	Parkinson's disease	rs117017186	T	C	T	C	3.62E-01	-9.97E-02	1.18E-02	9.85E-01	FALSE	FALSE	10	94962895	1.01E-01	4.88E-01	TRUE	10	93203138	7.96E-02	0.95	7174	5.33E-06	TRUE	TRUE	482,730	20.75
Phosphatidylcholine (18:2_18:2) levels	Parkinson's disease	rs11869356	T	C	T	C	-7.66E-02	-3.13E-02	5.85E-01	4.53E-01	FALSE	FALSE	17	33045065	2.26E-02	7.81E-01	TRUE	17	34718046	1.71E-02	0.99	7174	7.72E-06	TRUE	TRUE	482,730	20.03
Phosphatidylcholine (18:2_18:2) levels	Parkinson's disease	rs13078306	C	A	C	A	-8.19E-02	-2.10E-03	3.28E-01	6.37E-01	FALSE	FALSE	3	80102576	2.32E-02	3.31E-02	TRUE	3	80053426	1.76E-02	1.00	7174	3.51E-06	TRUE	TRUE	482,730	21.55
Phosphatidylcholine (18:2_18:2) levels	Parkinson's disease	rs13150924	A	G	A	G	-1.12E-01	-1.06E-02	1.30E-01	8.24E-01	FALSE	FALSE	4	170198810	2.99E-02	1.41E-01	TRUE	4	169277659	2.50E-02	0.98	7174	8.25E-06	TRUE	TRUE	482,730	19.91
Phosphatidylcholine (18:2_18:2) levels	Parkinson's disease	rs139598970	T	G	T	G	4.22E-01	4.61E-02	1.00E-02	9.87E-01	FALSE	FALSE	4	186760086	1.04E-01	1.82E-01	TRUE	4	185838932	8.96E-02	0.89	7174	2.54E-06	TRUE	TRUE	482,730	22.17
Phosphatidylcholine (18:2_18:2) levels	Parkinson's disease	rs148498852	G	A	G	A	1.57E-01	8.60E-03	7.72E-02	9.63E-01	FALSE	FALSE	2	205560693	7.22E-02	4.31E-02	TRUE	2	204695970	3.28E-02	0.93	7174	1.73E-06	TRUE	TRUE	482,730	22.92
Phosphatidylcholine (18:2_18:2) levels	Parkinson's disease	rs1601935	T	G	T	G	-9.40E-02	-3.28E-02	6.11E-01	3.41E-01	FALSE	FALSE	15	58671765	1.86E-02	3.89E-02	TRUE	15	58379566	1.71E-02	0.99	7174	3.75E-08	TRUE	TRUE	482,730	30.34
Phosphatidylcholine (18:2_18:2) levels	Parkinson's disease	rs174574	C	A	C	A	-2.22E-01	6.60E-03	5.91E-01	3.39E-01	FALSE	FALSE	11	61600342	1.77E-02	1.48E-01	TRUE	11	61832870	1.69E-02	1.00	7174	4.16E-39	TRUE	TRUE	482,730	173.20
Phosphatidylcholine (18:2_18:2) levels	Parkinson's disease	rs35958132	G	A	G	A	1.58E-01	-1.24E-01	6.95E-02	9.56E-01	FALSE	FALSE	7	119976087	6.61E-02	3.03E-02	TRUE	7	120336033	3.44E-02	0.91	7174	4.47E-06	TRUE	TRUE	482,730	21.08
Phosphatidylcholine (18:2_18:2) levels	Parkinson's disease	rs4271965	G	A	G	A	7.96E-02	-5.00E-03	5.67E-01	3.88E-01	FALSE	FALSE	4	160869705	2.29E-02	8.27E-02	TRUE	4	159948553	1.68E-02	1.00	7174	2.16E-06	TRUE	TRUE	482,730	22.48
Phosphatidylcholine (18:2_18:2) levels	Parkinson's disease	rs56158036	A	G	A	G	2.96E-01	-6.23E-02	1.75E-02	9.80E-01	FALSE	FALSE	3	95133255	7.24E-02	4.10E-01	TRUE	3	95414411	6.38E-02	1.00	7174	3.58E-06	TRUE	TRUE	482,730	21.51
Phosphatidylcholine (18:2_18:2) levels	Parkinson's disease	rs59256568	G	A	G	A	-2.97E-01	-2.66E-02	1.96E-02	9.59E-01	FALSE	FALSE	2	228670131	6.18E-02	1.76E-01	TRUE	2	227805415	6.28E-02	0.91	7174	2.34E-06	TRUE	TRUE	482,730	22.33
Phosphatidylcholine (18:2_18:2) levels	Parkinson's disease	rs62238391	T	C	T	C	8.64E-02	-9.00E-03	3.20E-01	6.55E-01	FALSE	FALSE	3	10492518	2.37E-02	1.52E-01	TRUE	3	10450834	1.79E-02	0.99	7174	1.38E-06	TRUE	TRUE	482,730	23.34
Phosphatidylcholine (18:2_18:2) levels	Parkinson's disease	rs6498540	G	A	G	A	9.34E-02	1.82E-02	3.34E-01	6.94E-01	FALSE	FALSE	16	15130594	1.84E-02	4.93E-01	TRUE	16	15036737	1.77E-02	0.99	7174	1.44E-07	TRUE	TRUE	482,730	27.73
Phosphatidylcholine (18:2_18:2) levels	Parkinson's disease	rs7122803	G	C	G	C	-1.08E-01	-6.31E-02	8.60E-01	8.56E-01	TRUE	FALSE	11	107754336	3.20E-02	2.43E-02	TRUE	11	107883610	2.38E-02	1.00	7174	6.32E-06	TRUE	TRUE	482,730	20.42
Phosphatidylcholine (18:2_18:2) levels	Parkinson's disease	rs7717591	G	A	G	A	-9.60E-02	-1.38E-02	6.79E-01	3.91E-01	FALSE	FALSE	5	51711739	2.28E-02	2.62E-01	TRUE	5	52415905	1.79E-02	1.00	7174	8.41E-08	TRUE	TRUE	482,730	28.77
Phosphatidylcholine (18:2_18:2) levels	Parkinson's disease	rs7798734	T	G	T	G	-8.18E-02	5.07E-02	3.39E-01	6.96E-01	FALSE	FALSE	7	90928757	2.45E-02	1.93E-02	TRUE	7	91299442	1.77E-02	0.99	7174	3.70E-06	TRUE	TRUE	482,730	21.44
Phosphatidylcholine (18:2_20:1) levels	Parkinson's disease	rs117210128	T	C	T	C	2.54E-01	4.01E-02	3.01E-02	9.70E-01	FALSE	FALSE	12	31443200	7.05E-02	2.45E-01	TRUE	12	31290266	5.66E-02	0.97	5521	7.49E-06	TRUE	TRUE	482,730	20.10
Phosphatidylcholine (18:2_20:1) levels	Parkinson's disease	rs174599	C	G	C	G	3.23E-01	6.70E-03	4.19E-01	3.61E-01	TRUE	FALSE	11	61621556	1.78E-02	1.49E-01	TRUE	11	61854084	1.87E-02	1.00	5521	3.92E-65	TRUE	TRUE	482,730	298.28
Phosphatidylcholine (18:2_20:1) levels	Parkinson's disease	rs17484	C	T	C	T	9.12E-02	-1.09E-02	6.15E-01	3.01E-01	FALSE	FALSE	3	116442350	2.47E-02	1.81E-01	TRUE	3	116723503	1.97E-02	0.99	5521	3.72E-06	TRUE	TRUE	482,730	21.45
Phosphatidylcholine (18:2_20:1) levels	Parkinson's disease	rs182173873	C	A	C	A	-2.42E-01	3.28E-02	4.87E-02	9.81E-01	FALSE	FALSE	11	61445716	1.92E-01	6.33E-02	TRUE	11	61678244	4.53E-02	0.97	5521	9.15E-08	TRUE	TRUE	482,730	28.62
Phosphatidylcholine (18:2_20:1) levels	Parkinson's disease	rs2302263	T	C	T	C	1.51E-01	-1.00E-03	1.30E-01	9.10E-01	FALSE	FALSE	11	64021821	3.12E-02	1.12E-02	TRUE	11	64254349	2.79E-02	1.00	5521	6.10E-08	TRUE	TRUE	482,730	29.41
Phosphatidylcholine (18:2_20:1) levels	Parkinson's disease	rs4147976	T	C	T	C	-9.37E-02	4.00E-03	5.50E-01	4.30E-01	FALSE	FALSE	17	66925923	2.73E-02	5.35E-02	TRUE	17	68929782	1.93E-02	0.98	5521	1.28E-06	TRUE	TRUE	482,730	23.51
Phosphatidylcholine (18:2_20:1) levels	Parkinson's disease	rs4622847	G	A	G	A	-9.38E-02	-1.16E-02	7.10E-01	2.91E-01	FALSE	FALSE	3	38824343	1.88E-02	2.69E-01	TRUE	3	38782852	2.12E-02	0.99	5521	9.74E-06	TRUE	TRUE	482,730	19.60
Phosphatidylcholine (18:2_20:1) levels	Parkinson's disease	rs56128146	T	C	T	C	8.94E-02	3.40E-02	4.23E-01	5.68E-01	FALSE	FALSE	3	87361171	2.02E-02	4.62E-02	TRUE	3	87312021	1.92E-02	1.00	5521	3.25E-06	TRUE	TRUE	482,730	21.70
Phosphatidylcholine (18:2_20:1) levels	Parkinson's disease	rs62315647	C	T	C	T	3.60E-01	5.56E-02	1.78E-02	9.71E-01	FALSE	FALSE	4	86498855	8.05E-02	3.10E-01	TRUE	4	85577702	7.80E-02	0.86	5521	3.96E-06	TRUE	TRUE	482,730	21.33
Phosphatidylcholine (18:2_20:1) levels	Parkinson's disease	rs72795807	T	C	T	C	-1.58E-01	-2.89E-02	8.05E-02	8.87E-01	FALSE	FALSE	5	111915933	4.50E-02	2.83E-01	TRUE	5	112580236	3.52E-02	0.96	5521	7.34E-06	TRUE	TRUE	482,730	20.14
Phosphatidylcholine (18:2_20:1) levels	Parkinson's disease	rs72956488	A	G	A	G	2.77E-01	6.24E-02	2.95E-02	9.55E-01	FALSE	FALSE	11	96886272	5.94E-02	5.32E-01	TRUE	11	97015272	6.08E-02	0.91	5521	5.44E-06	TRUE	TRUE	482,730	20.71
Phosphatidylcholine (18:2_20:1) levels	Parkinson's disease	rs78098253	A	C	A	C	2.66E-01	-3.39E-02	3.03E-02	9.72E-01	FALSE	FALSE	4	74970615	7.01E-02	2.02E-01	TRUE	4	74104898	5.65E-02	0.96	5521	2.53E-06	TRUE	TRUE	482,730	22.19
Phosphatidylcholine (18:2_20:1) levels	Parkinson's disease	rs79136768	G	A	G	A	2.41E-01	-8.48E-02	3.69E-02	9.78E-01	FALSE	FALSE	11	61362439	6.47E-02	7.20E-01	TRUE	11	61594967	5.08E-02	0.98	5521	2.05E-06	TRUE	TRUE	482,730	22.59
Phosphatidylcholine (18:2_20:1) levels	Parkinson's disease	rs9402974	T	A	T	A	-1.31E-01	2.99E-02	1.54E-01	1.02E-01	TRUE	FALSE	6	138756793	4.46E-02	2.98E-01	TRUE	6	138435656	2.78E-02	0.91	5521	2.55E-06	TRUE	TRUE	482,730	22.17
Phosphatidylcholine (18:2_20:1) levels	Parkinson's disease	rs9994217	A	G	A	G	8.59E-02	1.91E-02	4.25E-01	4.98E-01	FALSE	FALSE	4	14322640	2.24E-02	4.03E-01	TRUE	4	14321016	1.91E-02	0.99	5521	7.26E-06	TRUE	TRUE	482,730	20.16
Phosphatidylcholine (18:2_20:3) levels	Parkinson's disease	rs10774859	T	G	T	G	7.77E-02	4.87E-02	5.31E-01	4.19E-01	FALSE	FALSE	12	116863466	2.27E-02	1.60E-02	TRUE	12	116425661	1.72E-02	1.00	6615	6.74E-06	TRUE	TRUE	482,730	20.30
Phosphatidylcholine (18:2_20:3) levels	Parkinson's disease	rs10779836	T	C	T	C	1.04E-01	-4.40E-03	7.77E-01	1.96E-01	FALSE	FALSE	1	230303150	2.40E-02	6.80E-02	TRUE	1	230167404	2.08E-02	1.00	6615	6.56E-07	TRUE	TRUE	482,730	24.79

Phosphatidylcholine (18:2_20:3) levels	Parkinson's disease	rs11208004	A	G	A	G	-9.24E-02	1.10E-03	2.62E-01	6.78E-01	FALSE	FALSE	1	63145439	1.83E-02	2.16E-02	TRUE	1	62679768	1.98E-02	1.00	6615	3.11E-06	TRUE	TRUE	482,730	21.78
Phosphatidylcholine (18:2_20:3) levels	Parkinson's disease	rs11757990	T	C	T	C	4.71E-01	-1.28E-01	7.34E-03	9.74E-01	FALSE	FALSE	6	29764041	6.94E-02	3.25E-02	TRUE	6	29796264	1.05E-01	0.97	6615	7.54E-06	TRUE	TRUE	482,730	20.08
Phosphatidylcholine (18:2_20:3) levels	Parkinson's disease	rs117663514	A	C	A	C	-3.37E-01	8.47E-02	1.40E-02	9.79E-01	FALSE	FALSE	11	15154802	8.61E-02	4.88E-01	TRUE	11	15133256	7.52E-02	0.94	6615	7.48E-06	TRUE	TRUE	482,730	20.10
Phosphatidylcholine (18:2_20:3) levels	Parkinson's disease	rs138827081	A	G	A	G	-5.83E-01	-5.17E-02	4.93E-03	9.86E-01	FALSE	FALSE	5	98511774	9.92E-02	2.20E-01	TRUE	5	99176070	1.22E-01	0.98	6615	1.97E-06	TRUE	TRUE	482,730	22.67
Phosphatidylcholine (18:2_20:3) levels	Parkinson's disease	rs146084531	C	G	C	G	-2.77E-01	1.09E-01	2.05E-02	1.08E-02	TRUE	FALSE	6	98861797	1.98E-01	2.36E-01	TRUE	6	98413921	6.10E-02	0.99	6615	5.82E-06	TRUE	TRUE	482,730	20.58
Phosphatidylcholine (18:2_20:3) levels	Parkinson's disease	rs16916055	C	T	C	T	4.25E-01	8.78E-02	8.81E-03	9.88E-01	FALSE	FALSE	11	91160029	1.27E-01	3.11E-01	TRUE	11	91426863	9.42E-02	0.97	6615	6.42E-06	TRUE	TRUE	482,730	20.39
Phosphatidylcholine (18:2_20:3) levels	Parkinson's disease	rs174548	G	C	G	C	2.10E-01	2.00E-03	3.84E-01	3.00E-01	TRUE	FALSE	11	61571348	1.83E-02	3.90E-02	TRUE	11	61803876	1.78E-02	1.00	6615	1.40E-31	TRUE	TRUE	482,730	138.13
Phosphatidylcholine (18:2_20:3) levels	Parkinson's disease	rs2347672	C	A	C	A	-9.34E-02	-1.10E-02	6.74E-01	4.49E-01	FALSE	FALSE	4	43916896	1.73E-02	2.78E-01	TRUE	4	43914879	1.85E-02	1.00	6615	4.46E-07	TRUE	TRUE	482,730	25.53
Phosphatidylcholine (18:2_20:3) levels	Parkinson's disease	rs2489189	A	T	A	T	-1.58E-01	1.70E-03	6.38E-02	6.10E-02	TRUE	FALSE	1	92331736	3.67E-02	1.66E-02	TRUE	1	91866179	3.52E-02	1.00	6615	7.09E-06	TRUE	TRUE	482,730	20.20
Phosphatidylcholine (18:2_20:3) levels	Parkinson's disease	rs35480480	C	T	C	T	-1.31E-01	2.17E-02	1.01E-01	9.32E-01	FALSE	FALSE	10	60501490	4.93E-02	1.81E-01	TRUE	10	58741730	2.94E-02	0.96	6615	8.14E-06	TRUE	TRUE	482,730	19.94
Phosphatidylcholine (18:2_20:3) levels	Parkinson's disease	rs4130016	G	A	G	A	1.08E-01	-4.09E-02	1.56E-01	8.26E-01	FALSE	FALSE	11	133398116	2.93E-02	7.90E-01	TRUE	11	133528221	2.40E-02	1.00	6615	7.13E-06	TRUE	TRUE	482,730	20.19
Phosphatidylcholine (18:2_20:3) levels	Parkinson's disease	rs60417255	T	G	T	G	1.64E-01	1.66E-02	5.65E-02	9.61E-01	FALSE	FALSE	1	88055640	6.05E-02	1.06E-01	TRUE	1	87589957	3.72E-02	0.98	6615	9.89E-06	TRUE	TRUE	482,730	19.56
Phosphatidylcholine (18:2_20:3) levels	Parkinson's disease	rs75662719	G	A	G	A	-2.00E-01	-1.30E-01	3.81E-02	9.83E-01	FALSE	FALSE	3	23082901	9.01E-02	8.24E-01	TRUE	3	23041410	4.48E-02	1.00	6615	8.25E-06	TRUE	TRUE	482,730	19.91
Phosphatidylcholine (18:2_20:3) levels	Parkinson's disease	rs75719904	G	A	G	A	2.09E-01	1.44E-02	4.34E-02	9.80E-01	FALSE	FALSE	12	26286351	8.81E-02	6.06E-02	TRUE	12	26133418	4.42E-02	0.98	6615	2.21E-06	TRUE	TRUE	482,730	22.44
Phosphatidylcholine (18:2_20:3) levels	Parkinson's disease	rs76833802	T	C	T	C	-1.54E-01	1.64E-02	8.37E-02	8.82E-01	FALSE	FALSE	7	155280851	3.70E-02	1.82E-01	TRUE	7	155488156	3.28E-02	0.92	6615	2.58E-06	TRUE	TRUE	482,730	22.14
Phosphatidylcholine (18:2_20:4) levels	Parkinson's disease	rs102274	C	T	C	T	-1.74E-01	-3.90E-03	4.09E-01	6.70E-01	FALSE	FALSE	11	61557826	1.79E-02	8.34E-02	TRUE	11	61790354	1.71E-02	1.00	7049	2.66E-24	TRUE	TRUE	482,730	104.21
Phosphatidylcholine (18:2_20:4) levels	Parkinson's disease	rs1033155	G	A	G	A	-9.68E-02	1.40E-03	3.98E-01	5.73E-01	FALSE	FALSE	15	89653764	2.01E-02	2.41E-02	TRUE	15	89110533	1.74E-02	1.00	7049	2.50E-08	TRUE	TRUE	482,730	31.13
Phosphatidylcholine (18:2_20:4) levels	Parkinson's disease	rs10446372	C	T	C	T	-7.68E-02	2.28E-02	4.37E-01	5.59E-01	FALSE	FALSE	3	76924329	2.30E-02	4.95E-01	TRUE	3	76875178	1.70E-02	0.99	7049	6.17E-06	TRUE	TRUE	482,730	20.47
Phosphatidylcholine (18:2_20:4) levels	Parkinson's disease	rs1077834	C	T	C	T	1.23E-01	-3.20E-03	2.66E-01	7.60E-01	FALSE	FALSE	15	58723479	2.20E-02	5.35E-02	TRUE	15	58431280	1.94E-02	0.98	7049	2.67E-10	TRUE	TRUE	482,730	40.01
Phosphatidylcholine (18:2_20:4) levels	Parkinson's disease	rs114076093	T	C	T	C	-3.07E-01	1.14E-01	1.59E-02	9.79E-01	FALSE	FALSE	1	202906314	9.20E-02	6.65E-01	TRUE	1	202937186	6.87E-02	0.95	7049	8.05E-06	TRUE	TRUE	482,730	19.96
Phosphatidylcholine (18:2_20:4) levels	Parkinson's disease	rs117352906	T	C	T	C	4.32E-01	-4.81E-02	8.82E-03	9.78E-01	FALSE	FALSE	7	18645710	8.56E-02	2.41E-01	TRUE	7	18606087	9.30E-02	0.92	7049	3.47E-06	TRUE	TRUE	482,730	21.57
Phosphatidylcholine (18:2_20:4) levels	Parkinson's disease	rs117919959	C	G	C	G	-3.58E-01	1.57E-02	1.23E-02	1.73E-02	TRUE	FALSE	6	104482984	9.40E-02	6.20E-02	TRUE	6	104035109	8.04E-02	0.91	7049	8.83E-06	TRUE	TRUE	482,730	19.78
Phosphatidylcholine (18:2_20:4) levels	Parkinson's disease	rs139923737	T	C	T	C	-3.04E-01	1.19E-01	2.32E-02	9.77E-01	FALSE	FALSE	7	153605998	8.25E-02	8.28E-01	TRUE	7	153908913	5.74E-02	0.94	7049	1.24E-07	TRUE	TRUE	482,730	28.01
Phosphatidylcholine (18:2_20:4) levels	Parkinson's disease	rs1601935	T	G	T	G	-8.16E-02	-3.28E-02	6.11E-01	3.41E-01	FALSE	FALSE	15	58671765	1.86E-02	3.89E-02	TRUE	15	58379566	1.73E-02	0.99	7049	2.32E-06	TRUE	TRUE	482,730	22.35
Phosphatidylcholine (18:2_20:4) levels	Parkinson's disease	rs2419125	A	G	A	G	-8.23E-02	4.00E-03	7.04E-01	2.62E-01	FALSE	FALSE	1	168644806	2.57E-02	5.68E-02	TRUE	1	168675568	1.85E-02	0.98	7049	8.59E-06	TRUE	TRUE	482,730	19.83
Phosphatidylcholine (18:2_20:4) levels	Parkinson's disease	rs2973222	T	C	T	C	7.93E-02	-2.87E-02	5.06E-01	4.40E-01	FALSE	FALSE	4	37586427	2.73E-02	5.32E-01	TRUE	4	37584805	1.71E-02	0.97	7049	3.39E-06	TRUE	TRUE	482,730	21.61
Phosphatidylcholine (18:2_20:4) levels	Parkinson's disease	rs34689885	T	A	T	A	9.56E-02	-3.80E-03	2.79E-01	2.53E-01	TRUE	FALSE	3	137847400	2.13E-02	6.63E-02	TRUE	3	138128558	1.88E-02	1.00	7049	3.76E-07	TRUE	TRUE	482,730	25.86
Phosphatidylcholine (18:2_20:4) levels	Parkinson's disease	rs4695858	G	A	G	A	2.17E-01	-5.44E-02	3.20E-02	9.65E-01	FALSE	FALSE	4	174503007	6.60E-02	3.87E-01	TRUE	4	173581856	4.91E-02	0.94	7049	9.96E-06	TRUE	TRUE	482,730	19.55
Phosphatidylcholine (18:2_20:4) levels	Parkinson's disease	rs62482552	A	G	A	G	-8.19E-02	1.69E-02	6.08E-01	4.41E-01	FALSE	FALSE	7	100522355	1.98E-02	4.04E-01	TRUE	7	100924735	1.75E-02	0.98	7049	2.81E-06	TRUE	TRUE	482,730	21.97
Phosphatidylcholine (18:2_20:4) levels	Parkinson's disease	rs673335	C	T	C	T	-9.75E-02	9.70E-03	2.45E-01	8.31E-01	FALSE	FALSE	11	75450576	2.81E-02	1.37E-01	TRUE	11	75739531	1.97E-02	0.99	7049	7.43E-07	TRUE	TRUE	482,730	24.55
Phosphatidylcholine (18:2_20:4) levels	Parkinson's disease	rs712265	A	G	A	G	-7.42E-02	-3.39E-02	5.02E-01	4.16E-01	FALSE	FALSE	17	18008447	1.71E-02	2.37E-02	TRUE	17	18105133	1.68E-02	1.00	7049	9.65E-06	TRUE	TRUE	482,730	19.61
Phosphatidylcholine (18:2_20:4) levels	Parkinson's disease	rs72830074	T	G	T	G	-1.81E-01	-2.25E-02	5.13E-02	9.46E-01	FALSE	FALSE	2	68318824	4.30E-02	2.21E-01	TRUE	2	68091692	3.90E-02	0.97	7049	3.53E-06	TRUE	TRUE	482,730	21.54
Phosphatidylcholine (18:2_20:4) levels	Parkinson's disease	rs75528309	T	C	T	C	2.03E-01	3.05E-01	4.75E-02	9.77E-01	FALSE	FALSE	16	23560759	1.34E-01	1.15E-02	TRUE	16	23549438	4.26E-02	0.85	7049	1.82E-06	TRUE	TRUE	482,730	22.81
Phosphatidylcholine (18:2_20:4) levels	Parkinson's disease	rs76780983	G	T	G	T	2.35E-01	1.44E-02	3.05E-02	9.77E-01	FALSE	FALSE	11	43381968	8.04E-02	6.68E-02	TRUE	11	43360418	4.95E-02	0.98	7049	2.11E-06	TRUE	TRUE	482,730	22.53
Phosphatidylcholine (18:2_20:4) levels	Parkinson's disease	rs77985607	A	G	A	G	-1.73E-01	6.07E-02	5.63E-02	9.56E-01	FALSE	FALSE	4	7396389	5.59E-02	5.57E-01	TRUE	4	7394662	3.77E-02	0.95	7049	4.18E-06	TRUE	TRUE	482,730	21.21
Phosphatidylcholine (18:2_20:4) levels	Parkinson's disease	rs79450037	A	G	A	G	-2.21E-01	4.60E-02	3.91E-02	9.72E-01	FALSE	FALSE	16	81400617	6.82E-02	3.01E-01	TRUE	16	81367012	4.46E-02	0.98	7049	7.72E-07	TRUE	TRUE	482,730	24.47
Phosphatidylcholine (18:2_20:4) levels	Parkinson's disease	rs9889746	C	T	C	T	1.46E-01	-7.91E-02	7.30E-02	8.68E-01	FALSE	FALSE	17	42672663	2.99E-02	4.08E-03	TRUE	17	44595295	3.25E-02	0.98	7049	6.66E-06	TRUE	TRUE	482,730	20.32
Phosphatidylcholine (O-16:0_16:0) levels	Parkinson's disease	rs112728288	C	T	C	T	1.49E-01	2.84E-02	7.85E-02	8.77E-01	FALSE	FALSE	12	115072277	3.56E-02	3.70E-01	TRUE	12	114634472	3.28E-02	0.98	6605	5.53E-06	TRUE	TRUE	482,730	20.68
Phosphatidylcholine (O-16:0_16:0) levels	Parkinson's disease	rs115294982	C	A	C	A	-2.23E-01	-5.55E-02	3.73E-02	9.87E-01	FALSE	FALSE	4	145840930	1.14E-01	2.03E-01	TRUE	4	144919778	4.56E-02	0.99	6605	1.08E-06	TRUE	TRUE	482,730	23.82
Phosphatidylcholine (O-16:0_16:0) levels	Parkinson's disease	rs1160027	T	G	T	G	9.48E-02	-2.69E-02	2.50E-01	7.62E-01	FALSE	FALSE	17	53818102	3.60E-02	3.43E-01	TRUE	17	55740741	2.11E-02	0.89	6605	7.44E-06	TRUE	TRUE	482,730	20.11
Phosphatidylcholine (O-16:0_16:0) levels	Parkinson's disease	rs12440833	G	A	G	A	-1.30E-01	-5.30E-03	1.11E-01	9.40E-01	FALSE	FALSE	15	88871799	5.84E-02	3.27E-02	TRUE	15	88328568	2.86E-02	0.92	6605	5.88E-06	TRUE	TRUE	482,730	20.56
Phosphatidylcholine (O-16:0_16:0) levels	Parkinson's disease	rs148625143	G	C	G	C	-2.06E-01	7.40E-03	4.30E-02	6.11E-02	TRUE	FALSE	2	228673150	5.01E-02	5.45E-02	TRUE	2	227808434	4.44E-02	0.93	6605	3.54E-06	TRUE	TRUE	482,730	21.53
Phosphatidylcholine (O-16:0_16:0) levels	Parkinson's disease	rs150651997	C	T	C	T	2.81E-01	3.18E-02	1.97E-02	9.64E-01	FALSE	FALSE	2	49655965	6.26E-02	2.14E-01	TRUE	2	49428827	6.33E-02	0.98	6605	9.15E-06	TRUE	TRUE	482,730	19.71
Phosphatidylcholine (O-16:0_16:0) levels	Parkinson's disease	rs17292238	C	G	C	G	1.51E-01	8.93E-02	7.65E-02	8.01E-02	TRUE	FALSE	17	1787537	4.43E-02	2.19E-02	TRUE	17	1884243	3.39E-02	0.91	6605	8.61E-06	TRUE	TRUE	482,730	19.83
Phosphatidylcholine (O-16:0_16:0) levels	Parkinson's disease	rs17586917	A	G	A	G	-8.44E-02	6.24E-02	3.23E-01	6.76E-01	FALSE	FALSE	3	2965650	2.86E-02	1.46E-02	TRUE	3	2923966	1.87E-02	0.98	6605	6.69E-06	TRUE	TRUE	482,730	20.31
Phosphatidylcholine (O-16:0_16:0) levels	Parkinson's disease	rs17592511	A	T	A	T	3.90E-01	-1.73E-02	1.15E-02	2.44E-02	TRUE	FALSE	13	67781957	7.99E-02	8.14E-02	TRUE	13	67207825	8.57E-02	0.89	6605	5.43E-06	TRUE	TRUE	482,730	20.71
Phosphatidylcholine (O-16:0_16:0) levels	Parkinson's disease	rs6675844	A	T	A	T	-1.49E-01	2.27E-02	8.13E-02	9.38E-02	TRUE	FALSE	1	96139574	3.82E-02	2.57E-01	TRUE	1	95674018	3.20E-02	0.99	6605	3.50E-06	TRUE	TRUE	482,730	21.56
Phosphatidylcholine (O-16:0_16:0) levels	Parkinson's disease	rs6686021	T	C	T	C	8.04E-02	-2.40E-03	4.00E-01	5.88E-01	FALSE	FALSE	1	154983539	2.09E-02	4.16E-02	TRUE	1	155011063	1.80E-02	0.98	6605	7.97E-06	TRUE	TRUE	482,730	19.97
Phosphatidylcholine (O-16:0_16:0) levels	Parkinson's disease	rs6987890	G	A	G	A	9.99E-02	-5.20E-03	1.94E-01	8.52E-01	FALSE	FALSE	8	72023525	3.21E-02	6.03E-02	TRUE	8	71111290	2.23E-02	1.00	6605	7.34E-06	TRUE	TRUE	482,730	20.13
Phosphatidylcholine (O-16:0_16:0) levels	Parkinson's disease	rs7170186	A	C	A	C	8.26E-02	-3.03E-02	6.70E-01	3.31E-01	FALSE	FALSE	15	95251591	2.00E-02	8.87E-01	TRUE	15	94708362	1.83E-02	1.00	6605	6.71E-06	TRUE	TRUE	482,730	20.30
Phosphatidylcholine (O-16:0_16:0) levels	Parkinson's disease	rs72739734	A	G	A	G	-2.92E-01	2.27E-02	1.89E-02	9.64E-01	FALSE	FALSE	9	83712452	6.22E-02	1.46E-01	TRUE	9	81097537	6.40E-02	0.97	6605	5.02E-06	TRUE	TRUE	482,730	20.86
Phosphatidylcholine (O-16:0_16:0) levels	Parkinson's disease	rs74591482	C	G	C	G	-1.52E-01	-5.01E-02	9.10E-02	8.63E-02	TRUE	FALSE	16	10888409	5.16E-02	4.79E-01	TRUE	16	10794552	3.41E-02	0.80	6605	7.86E-06	TRUE	TRUE	482,730	20.00
Phosphatidylcholine (O-16:0_16:0) levels	Parkinson's disease	rs76494046	C	A	C	A	-1.89E-01	-1.23E-01	4.80E-02	9.39E-01	FALSE	FALSE	7	82619254	4.64E-02	4.09E-03	TRUE	7	82989938	4.13E-02	0.99	6605	4.97E-06	TRUE	TRUE	482,730	20.88
Phosphatidylcholine (O-16:0_16:0) levels	Parkinson's disease	rs80134854	T	C	T	C	2.53E-01	3.12E-01	2.61E-02	9.87E-01	FALSE	FALSE	7	96674915	1.69E-01	3.26E-02	TRUE	7	97045603	5.72E-02	0.94	6605	9.65E-06	TRUE	TRUE	482,730	19.61
Phosphatidylcholine (O-16:0_16:1) levels	Parkinson's disease	rs10248190	A	T	A	T	1.44E-01	3.70E-03	9.99E-02	1.46E-01	TRUE	FALSE	7	130689908	3.50E-02	3.83E-02	TRUE	7	131005149	3.20E-02	0.96	5658	7.48E-06	TRUE	TRUE	482,730	20.10
Phosphatidylcholine (O-16:0_16:1) levels	Parkinson's disease	rs114541469	C	G	C	G	1.50E-01	-4.41E-02	9.01E-02	5.97E-02	TRUE	FALSE	2	47804794	5.11E-02	4.11E-01	TRUE	2	47577655	3.36E-02	0.95	5658	8.33E-06	TRUE	TRUE	482,730	19.89
Phosphatidylcholine (O-16:0_16:1) levels	Parkinson's disease	rs13394815	A	C	A	C	-9.43E-02	2.45E-02	3.89E-01	7.30E-01	FALSE	FALSE	2	70564002	2.52E-02	4.81E-01	TRUE	2	70336870	1.95E-02	0.97	5658	1.29E-06	TRUE	TRUE	482,730	23.48
Phosphatidylcholine (O-16:0_16:1) levels	Parkinson's disease	rs146085506	C	T	C	T	2.02E-01	1.42E-01	5.84E-02	9.69E-01	FALSE	FALSE	9	31384829	7.85E-02	3.57E-02	TRUE	9	31384831	4.15E-02	0.94	5658	1.20E-06	TRUE	TRUE	482,730	23.63
Phosphatidylcholine (O-16:0_16:1) levels	Parkinson's disease	rs16916332	A	G	A	G	2.10E-01	1.53E-01	4.36E-02	9.67E-01	FALSE	FALSE	8	52367473	5.87E-02	4.51E-03	TRUE	8	51454913	4.64E-02	1.00	5658	6.24E-06	TRUE	TRUE	482,730	20.45
Phosphatidylcholine (O-16:0_16:1) levels	Parkinson's disease	rs17231506	T	C	T	C	1.33E-01	1.36E-02	2.78E-01	6.87E-01	FALSE	FALSE	16	56994528	1.81E-02	3.45E-01	TRUE	16	56960616	2.08E-02	1.00	5658	1.90E-10	TRUE	TRUE	482,730	40.72
Phosphatidylcholine (O-16:0_16:1) levels	Parkinson's disease	rs1800588	T	C	T	C	1.10E-01	-3.30E-03	2.58E-01	7.75E-01	FALSE	FALSE	15	58723675	2.12E-02	5.73E-02	TRUE	15	58431476	2.18E-02	0.99	5658	4.86E-07	TRUE	TRUE	482,730	25.37
Phosphatidylcholine (O-16:0_16:1) levels	Parkinson's disease	rs2356187	A	G	A	G	1.26E-01	-9.00E-04	8.69E-01	7.69E-02	FALSE	FALSE	2	159370003	4.25E-02	7.71E-03	TRUE	2	158513491	2.80E-02	1.00	5658	6.54E-06	TRUE	TRUE	482,730	20.36
Phosphatidylcholine (O-16:0_16:1) levels	Parkinson's disease	rs28609532	G	A	G	A	-1.66E-01	5.49E-02	7.32E-02	8.86E-01	FALSE	FALSE	14	62608023	3.54E-02	9.16E-01	TRUE	14	62141305	3.58E-02	1.00	5658	3.61E-06	TRUE	TRUE	482,730	21.50
Phosphatidylcholine (O-16:0_16:1) levels	Parkinson's disease	rs4744927	C	A	C	A	9.53E-01	2.19E-01	9.98E-01	8.30E-03	FALSE	FALSE	9	72284718	2.03E-01	5.54E-01	TRUE	9	69669802	2.12E-01	0.86	5658	7.11E-06	TRUE	TRUE	482,730	20.19
Phosphatidylcholine (O-16:0_16:1) levels	Parkinson's disease	rs603424	A	G	A	G	-1.43E-01	6.90E-03	1.21E-01	8.02E-01	FALSE	FALSE	10	102075479	2.49E-02	1.07E-01	TRUE	10	100315722	2.94E-02	1.00	5658	1.10E-06	TRUE	TRUE	482,730	23.79
Phosphatidylcholine (O-16:0_16:1) levels	Parkinson's disease	rs62311841	A	G	A	G	-1.62E-01	-2.02E-02	1.24E-01	8.14E-01	FALSE	FALSE	4	134123228	2.85E-02	3.19E-01	TRUE	4	133202073	2.91E-02	0.99	5658	2.88E-08	TRUE	TRUE	482,730	30.87
Phosphatidylcholine (O-16:0_16:1) levels	Parkinson's disease	rs728516	A	G	A	G	-8.48E-02	-4.27E-02	5.26E-01	3.80E-01	FALSE	FALSE	11	45600806	2.30E-02	3.17E-02	TRUE	11	45579256	1.87E-02	1.00	5658	5.97E-06	TRUE	TRUE	482,730	20.54
Phosphatidylcholine (O-16:0_16:1) levels	Parkinson's disease	rs72973984	G	A	G	A	-1.45E-01	6.84E-02	1.09E-01	9.14E-01	FALSE	FALSE	11	105079354	4.32E-02	9.46E-01	TRUE	11	105208627	3.07E-02	0.98	5658	2.43E-06	TRUE	TRUE	482,730	22.26
Phosphatidylcholine (O-16:0_16:1) levels	Parkinson's disease	rs77494629	A	C	A	C	1.07E-01	-2.57E-02	2.68E-01	8.80E-01	FALSE	FALSE	2	102188878	3.49E-02	3.35E-01	TRUE	2	101572416	2.14E-02	0.99	5658	6.54E-07	TRUE	TRUE	482,730	24.80
Phosphatidylcholine (O-16:0_18:1) levels	Parkinson's disease	rs10120342	G	T	G	T	9.22E-02	1.21E-02	2.08E-01	7.19E-01	FALSE	FALSE	9	26919607	2.43E-02	2.09E-01	TRUE	9	26919609	2.07E-02	1.00	6956	8.42E-06	TRUE	TRUE	482,730	19.87
Phosphatidylcholine (O-16:0_18:1) levels	Parkinson's disease	rs11057853	C	T	C	T	-7.74E-02	-1.98E-02	4.54E-01	5.57E-01	FALSE	FALSE	12	125329313	2.29E-02	4.13E-01	TRUE	12	124844767	1.71E-02	0.99	6956	6.20E-06	TRUE	TRUE	482,730	20.45
Phosphatidylcholine (O-16:0_18:1) levels	Parkinson's disease	rs113746682	T	C	T	C	2.73E-01	-5.36E-02	2.30E-02	9.68E-01	FALSE	FALSE	16	85377751	7.09E-02	3.48E-01	TRUE	16	85344145	5.93E-02	0.91	6956	4.12E-06	TRUE	TRUE	482,730	21.24
Phosphatidylcholine (O-16:0_18:1) levels	Parkinson's disease	rs117449635	A	G	A	G	3.33E-01	-6.47E-02	1.52E-02	9.80E-01	FALSE	FALSE	16	71097219	1.13E-01	2.46E-01	TRUE	16	71063316	7.20E-02	0.92	6956	3.83E-06	TRUE	TRUE	482,730	21.38
Phosphatidylcholine (O-16:0_18:1) levels	Parkinson's disease	rs138038919	T	C	T	C	8.87E-02	-2.89E-02	2.48E-01	8.02E-01	FALSE	FALSE	8	119932889	2.74E-02	5.35E-01	TRUE	8	118920650	1.97E-02	1.00	6956	7.10E-06	TRUE	TRUE	482,730	20.19
Phosphatidylcholine (O-16:0_18:1) levels	Parkinson's disease	rs17231506	T	C	T	C	1.29E-01	1.36E-02	2.78E-01	6.87E-01	FALSE	FALSE	16	56994528	1.81E-02	3.45E-01	TRUE	16	56960616	1.88E-02	1.00	6956	6.16E-12	TRUE	TRUE	482,730	47.44
Phosphatidylcholine (O-16:0_18:1) levels	Parkinson's disease	rs1800588	T	C	T	C	1.19E-01	-3.30E-03	2.58E-01	7.75E-01	FALSE	FALSE	15	58723675	2.12E-02	5.73E-02	TRUE	15	58431476	1.96E-02	0.99	6956	1.29E-09	TRUE	TRUE	482,730	36.93
Phosphatidylcholine (O-16:0_18:1) levels	Parkinson's disease	rs34861396	A	G	A	G	8.71E-02	2.29E-02	2.99E-01	6.38E-01	FALSE	FALSE	8	31498467	2.76E-02	3.92E-01	TRUE	8	31640951	1.85E-02	0.99	6956	2.52E-06	TRUE	TRUE	482,730	22.19
Phosphatidylcholine (O-16:0_18:1) levels	Parkinson's disease	rs606587	G	A	G	A	-8.24E-02	1.27E-02	3.74E-01	6.75E-01	FALSE	FALSE	1	230316189	2.04E-02	2.72E-01	TRUE	1	230180443	1.75E-02	1.00	6956	2.49E-06	TRUE	TRUE	482,730	22.21
Phosphatidylcholine (O-16:0_18:1) levels	Parkinson's disease	rs72923423	T	G	T	G	-1.52E-01	-6.30E-03	7.36E-02	9.30E-01	FALSE	FALSE	11	35066306	3.96E-02	5.84E-02	TRUE	11	35044759	3.29E-02	0.99	6956	4.17E-06	TRUE	TRUE	482,730	21.22
Phosphatidylcholine (O-16:0_18:1) levels	Parkinson's disease	rs7677028	G	C	G	C	3.93E-01	2.67E-02	1.43E-02	2.61E-02	TRUE	FALSE	4	137943535	7.37E-02	1.44E-01	TRUE	4	137022381	7.22E-02	0.97	6956	5.29E-08	TRUE	TRUE	482,730	29.67
Phosphatidylcholine (O-16:0_18:1) levels	Parkinson's disease	rs77211823	C	T	C	T	1.89E-01	2.17E-02	4.91E-02	8.98E-01	FALSE	FALSE	11	12472147	3.96E-02	2.34E-01	TRUE	11	12450600	4.14E-02	0.89	6956	4.83E-06	TRUE	TRUE	482,730	20.93
Phosphatidylcholine (O-16:0_18:1) levels	Parkinson's disease	rs7845596	G	C	G	C	1.54E-01	-2.87E-02	7.69E-02	1.33E-01	TRUE	FALSE	8	2703156	3.32E-02	4.12E-01	TRUE	8	2845634	3.21E-02	1.00	6956	1.59E-06	TRUE	TRUE	482,730	23.07
Phosphatidylcholine (O-16:0_18:1) levels	Parkinson's disease	rs79037957	C	G	C	G	2.19E-01	8.00E-04	3.45E-02	1.44E-02	TRUE	FALSE	16	72831111	7.60E-02	3.88E-03	TRUE	16	72797212	4.77E-02	0.95	6956	4.45E-06	TRUE	TRUE	482,730	21.09
Phosphatidylcholine (O-16:0_18:1) levels	Parkinson's disease	rs876969	T	C	T	C	-1.11E-01	-7.30E-03	1.50E-01	7.55E-01	FALSE	FALSE	2	48778877	2.23E-02	1.29E-01	TRUE	2	48551738	2.36E-02	1.00	6956	2.40E-06	TRUE	TRUE	482,730	22.28
Phosphatidylcholine (O-16:0_18:1) levels	Parkinson's disease	rs9538944	A	C	A	C	1.43E-01	1.57E-02	8.17E-02	8.57E-01	FALSE	FALSE	13	61394967	3.18E-02	2.06E-01	TRUE	13	60820833	3.14E-02	0.98	6956	5.62E-06	TRUE	TRUE	482,730	20.64
Phosphatidylcholine (O-16:0_18:1) levels	Parkinson's disease	rs9682490	C	T	C	T	-8.00E-02	-2.14E-02	4.35E-01	5.64E-01	FALSE	FALSE	3	4990776	2.26E-02	4.66E-01	TRUE	3	4949091	1.73E-02	0.98	6956	3.82E-06	TRUE	TRUE	482,730	21.38
Phosphatidylcholine (O-16:0_18:2) levels	Parkinson's disease	rs11777584	T	C	T	C	-7.74E-02	-3.72E-02	3.45E-01	7.15E-01	FALSE	FALSE	8	22828542	2.47E-02	8.78E-01	TRUE	8	22971029	1.75E-02	0.99	7158	9.95E-06	TRUE	TRUE	482,730	19.55
Phosphatidylcholine (O-16:0_18:2) levels	Parkinson's disease	rs11918522	A	G	A	G	1.06E-01	-3.09E-02	1.59E-01	8.27E-01	FALSE	FALSE	3	112535057	2.25E-02	7.70E-01	TRUE	3	112816210	2.30E-02	1.00	7158	4.10E-06	TRUE	TRUE	482,730	21.25
Phosphatidylcholine (O-16:0_18:2) levels	Parkinson's disease	rs11936543	G	A	G	A	-1.26E-01	5.67E-02	9.98E-02	9.62E-01	FALSE	FALSE	4	152191381	7.37E-02	3.54E-01	TRUE	4	151270229	2.84E-02	0.97	7158	8.95E-06	TRUE	TRUE	482,730	19.75
Phosphatidylcholine (O-16:0_18:2) levels	Parkinson's disease	rs149918547	A	G	A	G	5.66E-01	-3.63E-02	4.47E-03	9.89E-01	FALSE	FALSE	6	107525435	1.05E-01	1.36E-01	TRUE	6	107204231	1.28E-01	0.97	7158	9.97E-06	TRUE	TRUE	482,730	19.54
Phosphatidylcholine (O-16:0_18:2) levels	Parkinson's disease	rs17086339	C	G	C	G	-1.17E-01	2.51E-02	1.30E-01	7.06E-02	TRUE	FALSE	5	95935275	4.57E-02	2.35E-01	TRUE	5	96599571	2.48E-02	0.99	7158	2.67E-06	TRUE	TRUE	482,730	22.07
Phosphatidylcholine (O-16:0_18:2) levels	Parkinson's disease	rs17231506	T	C	T	C	1.22E-01	1.36E-02	2.78E-01	6.87E-01	FALSE	FALSE	16	56994528	1.81E-02	3.45E-01	TRUE	16	56960616	1.86E-02	1.00	7158	6.06E-11	TRUE	TRUE	482,730	42.93
Phosphatidylcholine (O-16:0_18:2) levels	Parkinson's disease	rs174574	C	A	C	A	-1.81E-01	6.60E-03	5.91E-01	3.39E-01	FALSE	FALSE	11	61600342	1.77E-02	1.48E-01	TRUE	11	61832870	1.70E-02	1.00	7158	1.55E-26	TRUE	TRUE	482,730	114.57
Phosphatidylcholine (O-16:0_18:2) levels	Parkinson's disease	rs181750334	T	A	T	A	-4.70E-01	3.30E-02	7.47E-03	1.37E-02	TRUE	FALSE	16	14739018	1.04E-01	1.24E-01	TRUE	16	14645161	1.03E-01	0.91	7158	5.09E-06	TRUE	TRUE	482,730	20.83
Phosphatidylcholine (O-16:0_18:2) levels	Parkinson's disease	rs2537611	T	C	T	C	7.77E-02	2.01E-02	3.72E-01	6.72E-01	FALSE	FALSE	7	17732799	2.36E-02	4.04E-01	TRUE	7	17693175	1.73E-02	1.00	7158	6.90E-06	TRUE	TRUE	482,730	20.25
Phosphatidylcholine (O-16:0_18:2) levels	Parkinson's disease	rs4579316	C	T	C	T	-1.08E-01	-5.70E-03	8.34E-01	1.65E-01	FALSE	FALSE	6	75757561	3.04E-02	7.04E-02	TRUE	6	75047845	2.24E-02	1.00	7158	1.66E-06	TRUE	TRUE	482,730	22.99
Phosphatidylcholine (O-16:0_18:2) levels	Parkinson's disease	rs6582326	T	A	T	A	-2.14E-01	-6.73E-02	9.65E-01	8.96E-01	TRUE	FALSE	12	76417069	4.66E-02	8.28E-01	TRUE	12	76023289	4.72E-02	0.93	7158	5.85E-06	TRUE	TRUE	482,730	20.57
Phosphatidylcholine (O-16:0_18:2) levels	Parkinson's disease	rs6741227	T	C	T	C	8.48E-02	-2.50E-02	3.29E-01	7.22E-01	FALSE	FALSE	2	207881845	2.49E-02	4.99E-01	TRUE	2	207017121	1.81E-02	1.00	7158	2.78E-06	TRUE	TRUE	482,730	22.00
Phosphatidylcholine (O-16:0_18:2) levels	Parkinson's disease	rs6764912	A	G	A	G	-1.34E-01	9.29E-02	8.68E-02	9.36E-01	FALSE	FALSE	3	128372487	4.87E-02	2.82E-02	TRUE	3	128653644	2.97E-02	0.98	7158	6.76E-06	TRUE	TRUE	482,730	20.29
Phosphatidylcholine (O-16:0_18:2) levels	Parkinson's disease	rs75863117	A	G	A	G	-1.32E-01	1.20E-02	9.63E-02	8.84E-01	FALSE	FALSE	7	68781288	3.93E-02	1.19E-01	TRUE	7	69316302	2.96E-02	0.92	7158	8.85E-06	TRUE	TRUE	482,730	19.77
Phosphatidylcholine (O-16:0_18:2) levels	Parkinson's disease	rs76888052	T	C	T	C	6.76E-01	3.52E-02	3.98E-03	9.80E-01	FALSE	FALSE	7	117317616	9.45E-02	1.49E-01	TRUE	7	117677562	1.51E-01	0.81	7158	8.00E-06	TRUE	TRUE	482,730	19.97
Phosphatidylcholine (O-16:0_18:2) levels	Parkinson's disease	rs79195215	G	C	G	C	-2.37E-01	-4.61E-02	2.64E-02	4.26E-02	TRUE	FALSE	7	10999880	5.85E-02	3.66E-01	TRUE	7	10960253	5.37E-02	0.96	7158	9.81E-06	TRUE	TRUE	482,730	19.57
Phosphatidylcholine (O-16:0_20:3) levels	Parkinson's disease	rs10951096	G	A	G	A	-8.72E-02	1.09E-02	7.25E-01	2.29E-01	FALSE	FALSE	7	3208159	2.67E-02	1.65E-01	TRUE	7	3168526	1.96E-02	0.99	6709	8.90E-06	TRUE	TRUE	482,730	19.76
Phosphatidylcholine (O-16:0_20:3) levels	Parkinson's disease	rs117435540	A	G	A	G	5.29E-01	1.38E-01	6.95E-03	9.74E-01	FALSE	FALSE	10	125770586	8.11E-02	4.43E-02	TRUE	10	124011070	1.14E-01	0.88	6709	3.69E-06	TRUE	TRUE	482,730	21.45
Phosphatidylcholine (O-16:0_20:3) levels	Parkinson's disease	rs12671763	A	C	A	C	3.11E-01	-3.06E-02	1.67E-02	9.70E-01	FALSE	FALSE	7	28522703	7.00E-02	1.79E-01	TRUE	7	28483085	6.91E-02	0.95	6709	6.85E-06	TRUE	TRUE	482,730	20.27
Phosphatidylcholine (O-16:0_20:3) levels	Parkinson's disease	rs1800588	T	C	T	C	9.60E-02	-3.30E-03	2.58E-01	7.75E-01	FALSE	FALSE	15	58723675	2.12E-02	5.73E-02	TRUE	15	58431476	2.00E-02	0.99	6709	1.73E-06	TRUE	TRUE	482,730	22.92
Phosphatidylcholine (O-16:0_20:3) levels	Parkinson's disease	rs1833350	T	C	T	C	9.09E-02	-1.89E-02	2.52E-01	7.05E-01	FALSE	FALSE	2	71330823	2.00E-02	4.65E-01	TRUE	2	71103693	2.03E-02	0.97	6709	7.62E-06	TRUE	TRUE	482,730	20.06
Phosphatidylcholine (O-16:0_20:3) levels	Parkinson's disease	rs2360706	C	G	C	G	9.12E-02	-2.01E-02	7.66E-01	7.98E-01	TRUE	FALSE	9	420203	2.80E-02	3.24E-01	TRUE	9	420203	2.04E-02	0.99	6709	7.69E-06	TRUE	TRUE	482,730	20.04
Phosphatidylcholine (O-16:0_20:3) levels	Parkinson's disease	rs62501991	T	C	T	C	-1.01E-01	2.40E-03	1.80E-01	7.85E-01	FALSE	FALSE	8	15053736	2.73E-02	3.17E-02	TRUE	8	15196227	2.24E-02	1.00	6709	7.35E-06	TRUE	TRUE	482,730	20.13
Phosphatidylcholine (O-16:0_20:3) levels	Parkinson's disease	rs6498540	G	A	G	A	-1.54E-01	1.82E-02	3.34E-01	6.94E-01	FALSE	FALSE	16	15130594	1.84E-02	4.93E-01	TRUE	16	15036737	1.83E-02	0.99	6709	5.73E-17	TRUE	TRUE	482,730	70.44
Phosphatidylcholine (O-16:0_20:3) levels	Parkinson's disease	rs75085081	A	G	A	G	2.38E-01	-1.08E-02	2.79E-02	9.78E-01	FALSE	FALSE	11	118801045	7.96E-02	4.98E-02	TRUE	11	118930336	5.34E-02	0.94	6709	8.47E-06	TRUE	TRUE	482,730	19.86
Phosphatidylcholine (O-16:0_20:3) levels	Parkinson's disease	rs75111872	A	G	A	G	-1.09E-01	-1.79E-02	1.62E-01	7.93E-01	FALSE	FALSE	3	133779860	2.74E-02	2.88E-01	TRUE	3	134061016	2.33E-02	1.00	6709	2.93E-06	TRUE	TRUE	482,730	21.90
Phosphatidylcholine (O-16:0_20:3) levels	Parkinson's disease	rs79036443	C	A	C	A	-1.97E-01	1.09E-01	4.20E-02	9.62E-01	FALSE	FALSE	13	53354375	6.38E-02	4.45E-02	TRUE	13	52780240	4.33E-02	0.96	6709	5.65E-06	TRUE	TRUE	482,730	20.63
Phosphatidylcholine (O-16:0_20:3) levels	Parkinson's disease	rs9552958	T	C	T	C	-1.36E-01	4.56E-02	9.51E-02	8.95E-01	FALSE	FALSE	13	24001078	3.25E-02	7.97E-01	TRUE	13	23426939	2.99E-02	0.96	6709	5.66E-06	TRUE	TRUE	482,730	20.63
Phosphatidylcholine (O-16:0_20:3) levels	Parkinson's disease	rs968567	T	C	T	C	2.71E-01	-3.41E-02	1.13E-01	8.31E-01	FALSE	FALSE	11	61595564	2.24E-02	8.94E-01	TRUE	11	61828092	2.71E-02	1.00	6709	2.17E-23	TRUE	TRUE	482,730	100.05
Phosphatidylcholine (O-16:0_20:3) levels	Parkinson's disease	rs968742	G	A	G	A	8.08E-02	2.40E-02	5.19E-01	5.01E-01	FALSE	FALSE	7	29452137	1.91E-02	6.81E-01	TRUE	7	29412521	1.72E-02	1.00	6709	2.68E-06	TRUE	TRUE	482,730	22.07

Phosphatidylcholine (O-16:0_20:4) levels	Parkinson's disease	rs10487877	G	C	G	C	1.77E-01	1.13E-01	4.73E-02	1.84E-02	TRUE	FALSE	FALSE	7	80467301	1.35E-01	3.93E-01	TRUE	7	80837985	3.97E-02	0.98	7174	8.71E-06	TRUE	TRUE	482,730	19.80
Phosphatidylcholine (O-16:0_20:4) levels	Parkinson's disease	rs10867645	T	C	T	C	-7.80E-02	-1.82E-02	6.09E-01	3.94E-01	FALSE	FALSE	FALSE	9	83536050	2.29E-02	3.71E-01	TRUE	9	80921135	1.71E-02	1.00	7174	5.22E-06	TRUE	TRUE	482,730	20.79
Phosphatidylcholine (O-16:0_20:4) levels	Parkinson's disease	rs11057853	C	T	C	T	-8.81E-02	-1.98E-02	4.54E-01	5.57E-01	FALSE	FALSE	FALSE	12	125329313	2.29E-02	4.13E-01	TRUE	12	124844767	1.68E-02	0.99	7174	1.69E-07	TRUE	TRUE	482,730	27.41
Phosphatidylcholine (O-16:0_20:4) levels	Parkinson's disease	rs11173828	A	G	A	G	-1.60E-01	6.83E-02	6.88E-02	9.52E-01	FALSE	FALSE	FALSE	12	61602970	5.39E-02	6.89E-01	TRUE	12	61209189	3.28E-02	1.00	7174	1.17E-06	TRUE	TRUE	482,730	23.66
Phosphatidylcholine (O-16:0_20:4) levels	Parkinson's disease	rs114373179	G	A	G	A	-4.25E-01	-1.00E-04	8.14E-03	9.84E-01	FALSE	FALSE	FALSE	4	67683861	1.38E-01	1.74E-04	TRUE	4	66818143	9.59E-02	0.94	7174	9.46E-06	TRUE	TRUE	482,730	19.64
Phosphatidylcholine (O-16:0_20:4) levels	Parkinson's disease	rs114653320	G	A	G	A	2.78E-01	9.18E-02	2.07E-02	9.79E-01	FALSE	FALSE	FALSE	4	42285861	9.12E-02	5.03E-01	TRUE	4	42283844	6.19E-02	0.91	7174	7.51E-06	TRUE	TRUE	482,730	20.08
Phosphatidylcholine (O-16:0_20:4) levels	Parkinson's disease	rs115076819	T	C	T	C	-2.22E-01	-7.01E-02	2.98E-02	9.51E-01	FALSE	FALSE	FALSE	1	245608086	5.22E-02	7.48E-01	TRUE	1	245444784	5.00E-02	0.96	7174	9.02E-06	TRUE	TRUE	482,730	19.74
Phosphatidylcholine (O-16:0_20:4) levels	Parkinson's disease	rs12928189	G	A	G	A	-7.80E-02	2.94E-02	6.50E-01	2.49E-01	FALSE	FALSE	FALSE	16	7596179	2.10E-02	7.93E-01	TRUE	16	7546177	1.75E-02	1.00	7174	8.41E-06	TRUE	TRUE	482,730	19.87
Phosphatidylcholine (O-16:0_20:4) levels	Parkinson's disease	rs145755646	G	C	G	C	1.84E-01	2.59E-02	6.46E-02	4.82E-02	TRUE	FALSE	FALSE	17	57418652	5.40E-02	1.99E-01	TRUE	17	59341291	3.42E-02	0.99	7174	7.23E-08	TRUE	TRUE	482,730	29.06
Phosphatidylcholine (O-16:0_20:4) levels	Parkinson's disease	rs174568	T	C	T	C	-3.82E-01	-4.40E-03	4.05E-01	6.73E-01	FALSE	FALSE	FALSE	11	61593816	1.79E-02	9.37E-02	TRUE	11	61826344	1.65E-02	1.00	7174	1.16E-114	TRUE	TRUE	482,730	537.15
Phosphatidylcholine (O-16:0_20:4) levels	Parkinson's disease	rs2903910	A	T	A	T	2.02E-01	3.50E-03	9.52E-01	9.56E-01	TRUE	FALSE	FALSE	11	62035354	4.89E-02	2.54E-02	TRUE	11	62267882	3.95E-02	0.99	7174	3.17E-07	TRUE	TRUE	482,730	26.19
Phosphatidylcholine (O-16:0_20:4) levels	Parkinson's disease	rs34881711	A	G	A	G	1.07E-01	-1.03E-02	2.29E-01	7.90E-01	FALSE	FALSE	FALSE	5	153845234	2.38E-02	1.77E-01	TRUE	5	154465674	1.98E-02	0.99	7174	6.84E-08	TRUE	TRUE	482,730	29.17
Phosphatidylcholine (O-16:0_20:4) levels	Parkinson's disease	rs3741252	T	C	T	C	1.39E-01	-2.38E-02	1.34E-01	9.32E-01	FALSE	FALSE	FALSE	11	61511498	4.03E-02	2.56E-01	TRUE	11	61744026	2.46E-02	0.99	7174	1.70E-08	TRUE	TRUE	482,730	31.88
Phosphatidylcholine (O-16:0_20:4) levels	Parkinson's disease	rs642327	G	C	G	C	-7.65E-02	1.33E-02	3.94E-01	3.83E-01	TRUE	FALSE	FALSE	11	35516222	2.33E-02	2.45E-01	TRUE	11	35494674	1.73E-02	0.98	7174	9.68E-06	TRUE	TRUE	482,730	19.60
Phosphatidylcholine (O-16:0_20:4) levels	Parkinson's disease	rs658580	A	C	A	C	8.98E-02	1.00E-03	7.27E-01	3.12E-01	FALSE	FALSE	FALSE	9	116399607	2.51E-02	1.43E-02	TRUE	9	113637327	1.93E-02	0.94	7174	3.18E-06	TRUE	TRUE	482,730	21.74
Phosphatidylcholine (O-16:0_20:4) levels	Parkinson's disease	rs6601694	A	G	A	G	8.30E-02	-2.82E-02	3.34E-01	7.30E-01	FALSE	FALSE	FALSE	8	8248058	2.21E-02	6.94E-01	TRUE	8	8390548	1.78E-02	0.99	7174	3.04E-06	TRUE	TRUE	482,730	21.82
Phosphatidylcholine (O-16:0_20:4) levels	Parkinson's disease	rs6709442	A	G	A	G	9.47E-02	-2.24E-02	4.16E-01	5.49E-01	FALSE	FALSE	FALSE	2	124342266	2.23E-02	5.03E-01	TRUE	2	123584690	1.71E-02	1.00	7174	3.00E-08	TRUE	TRUE	482,730	30.77
Phosphatidylcholine (O-16:0_20:4) levels	Parkinson's disease	rs698909	A	G	A	G	8.18E-02	2.19E-02	6.86E-01	3.36E-01	FALSE	FALSE	FALSE	2	206554544	2.35E-02	4.54E-01	TRUE	2	205689820	1.81E-02	1.00	7174	6.00E-06	TRUE	TRUE	482,730	20.52
Phosphatidylcholine (O-16:0_20:4) levels	Parkinson's disease	rs73422593	G	A	G	A	-1.30E-01	2.40E-02	1.12E-01	8.96E-01	FALSE	FALSE	FALSE	9	21616161	3.79E-02	2.78E-01	TRUE	9	21616162	2.71E-02	0.97	7174	1.68E-06	TRUE	TRUE	482,730	22.96
Phosphatidylcholine (O-16:0_20:4) levels	Parkinson's disease	rs75385811	G	A	G	A	-9.52E-02	2.20E-03	2.02E-01	7.71E-01	FALSE	FALSE	FALSE	8	67057874	3.17E-02	2.46E-02	TRUE	8	66145639	2.10E-02	0.99	7174	5.77E-06	TRUE	TRUE	482,730	20.59
Phosphatidylcholine (O-16:0_20:4) levels	Parkinson's disease	rs7554873	C	T	C	T	1.86E-01	1.09E-02	5.04E-02	9.44E-01	FALSE	FALSE	FALSE	1	161612233	4.46E-02	9.31E-02	TRUE	1	161642443	3.91E-02	0.94	7174	2.13E-06	TRUE	TRUE	482,730	22.51
Phosphatidylcholine (O-16:0_20:4) levels	Parkinson's disease	rs76798309	A	G	A	G	2.98E-01	1.34E-01	1.68E-02	9.70E-01	FALSE	FALSE	FALSE	7	110282876	6.89E-02	2.60E-02	TRUE	7	110642820	6.47E-02	0.98	7174	4.36E-06	TRUE	TRUE	482,730	21.13
Phosphatidylcholine (O-16:0_20:4) levels	Parkinson's disease	rs77277881	A	G	A	G	-4.98E-01	2.17E-01	7.35E-03	9.84E-01	FALSE	FALSE	FALSE	1	20049338	1.12E-01	2.58E-02	TRUE	1	19722845	1.06E-01	0.86	7174	2.89E-06	TRUE	TRUE	482,730	21.92
Phosphatidylcholine (O-16:0_20:4) levels	Parkinson's disease	rs7982529	C	T	C	T	9.25E-02	1.97E-02	2.14E-01	7.70E-01	FALSE	FALSE	FALSE	13	40911463	2.67E-02	3.37E-01	TRUE	13	40337326	2.01E-02	1.00	7174	4.30E-06	TRUE	TRUE	482,730	21.16
Phosphatidylcholine (O-16:0_22:5) levels	Parkinson's disease	rs140792636	A	C	A	C	-6.73E-01	1.92E-01	4.19E-03	9.86E-01	FALSE	FALSE	FALSE	6	45897145	1.26E-01	8.98E-01	TRUE	6	45929408	1.43E-01	0.94	5988	2.47E-06	TRUE	TRUE	482,730	22.23
Phosphatidylcholine (O-16:0_22:5) levels	Parkinson's disease	rs142015595	A	T	A	T	-2.94E-01	4.91E-02	2.16E-02	3.16E-02	TRUE	FALSE	FALSE	16	83755289	6.67E-02	3.36E-01	TRUE	16	83721684	6.61E-02	0.96	5988	8.76E-06	TRUE	TRUE	482,730	19.80
Phosphatidylcholine (O-16:0_22:5) levels	Parkinson's disease	rs145866244	A	T	A	T	-2.51E-01	-1.84E-01	3.13E-02	1.91E-02	TRUE	FALSE	FALSE	5	75518585	9.06E-02	2.12E-02	TRUE	5	76222760	5.22E-02	0.96	5988	1.60E-06	TRUE	TRUE	482,730	23.07
Phosphatidylcholine (O-16:0_22:5) levels	Parkinson's disease	rs150885037	T	C	T	C	-2.32E-01	5.53E-02	3.26E-02	9.35E-01	FALSE	FALSE	FALSE	10	133172008	4.80E-02	6.04E-01	TRUE	10	131373745	5.07E-02	0.99	5988	5.08E-06	TRUE	TRUE	482,730	20.85
Phosphatidylcholine (O-16:0_22:5) levels	Parkinson's disease	rs174548	G	C	G	C	-1.68E-01	2.00E-03	3.84E-01	3.00E-01	TRUE	FALSE	FALSE	11	61571348	1.83E-02	3.90E-02	TRUE	11	61803876	1.89E-02	1.00	5988	7.57E-19	TRUE	TRUE	482,730	79.15
Phosphatidylcholine (O-16:0_22:5) levels	Parkinson's disease	rs55709923	G	C	G	C	-1.23E-01	5.26E-02	1.44E-01	1.03E-01	TRUE	FALSE	FALSE	1	17291129	3.90E-02	7.52E-01	TRUE	1	16964634	2.63E-02	0.98	5988	3.10E-06	TRUE	TRUE	482,730	21.80
Phosphatidylcholine (O-16:0_22:5) levels	Parkinson's disease	rs7103356	G	A	G	A	8.80E-02	1.02E-02	6.44E-01	3.93E-01	FALSE	FALSE	FALSE	11	134227401	2.36E-02	1.76E-01	TRUE	11	134357507	1.92E-02	0.98	5988	4.83E-06	TRUE	TRUE	482,730	20.94
Phosphatidylcholine (O-16:0_22:5) levels	Parkinson's disease	rs79318457	T	C	T	C	1.86E-01	-7.79E-02	5.37E-02	9.23E-01	FALSE	FALSE	FALSE	9	5604987	4.47E-02	4.07E-02	TRUE	9	5604987	4.10E-02	0.93	5988	5.82E-06	TRUE	TRUE	482,730	20.58
Phosphatidylcholine (O-16:0_22:5) levels	Parkinson's disease	rs80261753	A	G	A	G	-3.40E-01	6.18E-02	1.72E-02	9.76E-01	FALSE	FALSE	FALSE	4	27346129	8.26E-02	3.42E-01	TRUE	4	27344507	7.45E-02	0.90	5988	5.12E-06	TRUE	TRUE	482,730	20.83
Phosphatidylcholine (O-16:0_22:5) levels	Parkinson's disease	rs9458511	G	A	G	A	-1.21E-01	2.68E-02	1.27E-01	8.36E-01	FALSE	FALSE	FALSE	6	162736689	3.22E-02	3.92E-01	TRUE	6	162315657	2.74E-02	1.00	5988	9.41E-06	TRUE	TRUE	482,730	19.66
Phosphatidylcholine (O-16:1_16:0) levels	Parkinson's disease	rs1023679	G	A	G	A	1.02E-01	-1.14E-02	7.51E-01	2.78E-01	FALSE	FALSE	FALSE	5	143766166	2.48E-02	1.91E-01	TRUE	5	144386603	2.15E-02	1.00	5868	2.25E-06	TRUE	TRUE	482,730	22.41
Phosphatidylcholine (O-16:1_16:0) levels	Parkinson's disease	rs1077989	C	A	C	A	-1.83E-01	-2.34E-02	4.68E-01	5.22E-01	FALSE	FALSE	FALSE	14	67975822	1.67E-02	7.91E-01	TRUE	14	67509105	1.83E-02	1.00	5868	2.91E-23	TRUE	TRUE	482,730	99.57
Phosphatidylcholine (O-16:1_16:0) levels	Parkinson's disease	rs11858902	C	A	C	A	-9.70E-02	2.66E-02	3.04E-01	7.47E-01	FALSE	FALSE	FALSE	15	88867557	2.67E-02	4.95E-01	TRUE	15	88324326	2.12E-02	0.89	5868	4.76E-06	TRUE	TRUE	482,730	20.97
Phosphatidylcholine (O-16:1_16:0) levels	Parkinson's disease	rs1247489	G	T	G	T	9.35E-02	-1.29E-02	5.49E-01	3.73E-01	FALSE	FALSE	FALSE	10	78003580	2.32E-02	2.39E-01	TRUE	10	76243822	1.85E-02	1.00	5868	4.76E-07	TRUE	TRUE	482,730	25.42
Phosphatidylcholine (O-16:1_16:0) levels	Parkinson's disease	rs13104164	T	A	T	A	-1.24E-01	-3.39E-02	1.30E-01	1.19E-01	TRUE	FALSE	FALSE	4	161278137	3.55E-02	4.69E-01	TRUE	4	160356985	2.73E-02	0.99	5868	5.35E-06	TRUE	TRUE	482,730	20.75
Phosphatidylcholine (O-16:1_16:0) levels	Parkinson's disease	rs2740488	C	A	C	A	-1.08E-01	-1.10E-03	1.85E-01	7.27E-01	FALSE	FALSE	FALSE	9	107661742	1.89E-02	2.14E-02	TRUE	9	104899461	2.38E-02	1.00	5868	5.18E-06	TRUE	TRUE	482,730	20.80
Phosphatidylcholine (O-16:1_16:0) levels	Parkinson's disease	rs34160235	T	C	T	C	2.72E-01	-3.94E-02	2.54E-02	9.81E-01	FALSE	FALSE	FALSE	10	134913603	8.75E-02	1.86E-01	TRUE	10	133100099	6.04E-02	0.95	5868	6.76E-06	TRUE	TRUE	482,730	20.29
Phosphatidylcholine (O-16:1_16:0) levels	Parkinson's disease	rs670210	T	C	T	C	-8.87E-02	5.22E-02	6.68E-01	3.16E-01	FALSE	FALSE	FALSE	6	20072580	2.43E-02	1.59E-02	TRUE	6	20072349	1.98E-02	0.97	5868	7.72E-06	TRUE	TRUE	482,730	20.04
Phosphatidylcholine (O-16:1_16:0) levels	Parkinson's disease	rs71423392	C	G	C	G	-2.00E-01	-6.04E-02	5.29E-02	5.22E-02	TRUE	FALSE	FALSE	14	70696993	6.38E-02	4.64E-01	TRUE	14	70230276	4.35E-02	0.91	5868	4.18E-06	TRUE	TRUE	482,730	21.22
Phosphatidylcholine (O-16:1_16:0) levels	Parkinson's disease	rs73125516	C	G	C	G	1.04E-01	-1.47E-02	2.02E-01	1.61E-01	TRUE	FALSE	FALSE	7	39424580	3.02E-02	2.03E-01	TRUE	7	39384981	2.29E-02	1.00	5868	5.05E-06	TRUE	TRUE	482,730	20.86
Phosphatidylcholine (O-16:1_16:0) levels	Parkinson's disease	rs74843639	G	T	G	T	-2.41E-01	1.37E-02	3.01E-02	9.77E-01	FALSE	FALSE	FALSE	2	179002300	7.04E-02	7.30E-02	TRUE	2	178137573	5.39E-02	0.99	5868	8.02E-06	TRUE	TRUE	482,730	19.97
Phosphatidylcholine (O-16:1_16:0) levels	Parkinson's disease	rs75066420	A	G	A	G	1.85E-01	-1.54E-02	5.58E-02	9.63E-01	FALSE	FALSE	FALSE	9	24151527	6.16E-02	9.53E-02	TRUE	9	24151529	4.10E-02	0.95	5868	6.65E-06	TRUE	TRUE	482,730	20.33
Phosphatidylcholine (O-16:1_16:0) levels	Parkinson's disease	rs76014869	A	G	A	G	4.23E-01	7.47E-02	1.06E-02	9.76E-01	FALSE	FALSE	FALSE	15	41088077	6.32E-02	6.25E-01	TRUE	15	40795879	9.32E-02	0.98	5868	5.88E-06	TRUE	TRUE	482,730	20.56
Phosphatidylcholine (O-16:1_16:0) levels	Parkinson's disease	rs77351278	C	T	C	T	3.06E-01	-4.31E-02	2.10E-02	9.54E-01	FALSE	FALSE	FALSE	13	37209508	5.40E-02	3.72E-01	TRUE	13	36635371	6.78E-02	0.92	5868	6.71E-06	TRUE	TRUE	482,730	20.31
Phosphatidylcholine (O-16:1_16:0) levels	Parkinson's disease	rs78682716	A	G	A	G	4.65E-01	3.09E-02	1.03E-02	9.60E-01	FALSE	FALSE	FALSE	12	95578105	4.77E-02	2.86E-01	TRUE	12	95184329	9.30E-02	0.99	5868	5.82E-07	TRUE	TRUE	482,730	25.03
Phosphatidylcholine (O-16:1_16:0) levels	Parkinson's disease	rs79425159	C	T	C	T	-2.18E-01	-3.25E-02	3.92E-02	9.69E-01	FALSE	FALSE	FALSE	3	178258081	6.77E-02	2.00E-01	TRUE	3	178540293	4.73E-02	0.99	5868	4.26E-06	TRUE	TRUE	482,730	21.18
Phosphatidylcholine (O-16:1_18:0) levels	Parkinson's disease	rs1093316	C	T	C	T	-1.47E-01	5.09E-02	9.80E-02	8.34E-01	FALSE	FALSE	FALSE	10	96108774	2.46E-02	1.93E-02	TRUE	10	94349017	2.92E-02	0.99	6751	4.68E-07	TRUE	TRUE	482,730	25.44
Phosphatidylcholine (O-16:1_18:0) levels	Parkinson's disease	rs11187130	T	C	T	C	-4.52E-01	1.64E-02	8.80E-03	9.80E-01	FALSE	FALSE	FALSE	10	82569572	8.53E-02	7.17E-02	TRUE	10	80809816	9.45E-02	0.97	6751	1.78E-06	TRUE	TRUE	482,730	22.86
Phosphatidylcholine (O-16:1_18:0) levels	Parkinson's disease	rs115012576	G	A	G	A	-5.07E-01	-2.13E-01	6.32E-03	9.88E-01	FALSE	FALSE	FALSE	3	149769073	1.61E-01	7.30E-01	TRUE	3	150051286	1.14E-01	0.95	6751	8.66E-06	TRUE	TRUE	482,730	19.82
Phosphatidylcholine (O-16:1_18:0) levels	Parkinson's disease	rs116883951	A	G	A	G	4.39E-01	-7.90E-02	8.06E-03	9.61E-01	FALSE	FALSE	FALSE	13	103215520	5.76E-02	7.70E-01	TRUE	13	102563170	9.84E-02	0.96	6751	8.38E-06	TRUE	TRUE	482,730	19.88
Phosphatidylcholine (O-16:1_18:0) levels	Parkinson's disease	rs12270739	A	G	A	G	-1.23E-01	-8.70E-03	1.39E-01	8.85E-01	FALSE	FALSE	FALSE	11	36432319	4.56E-02	7.09E-02	TRUE	11	36410769	2.57E-02	0.94	6751	1.60E-06	TRUE	TRUE	482,730	23.06
Phosphatidylcholine (O-16:1_18:0) levels	Parkinson's disease	rs1355105	C	T	C	T	8.10E-02	1.50E-03	5.95E-01	4.46E-01	FALSE	FALSE	FALSE	17	51662985	2.66E-02	1.95E-02	TRUE	17	53585624	1.77E-02	0.98	6751	4.95E-06	TRUE	TRUE	482,730	20.89
Phosphatidylcholine (O-16:1_18:0) levels	Parkinson's disease	rs1397106	G	T	G	T	-7.84E-02	4.48E-02	4.11E-01	6.34E-01	FALSE	FALSE	FALSE	8	78589030	1.96E-02	1.11E-02	TRUE	8	77676794	1.74E-02	1.00	6751	6.65E-06	TRUE	TRUE	482,730	20.32
Phosphatidylcholine (O-16:1_18:0) levels	Parkinson's disease	rs143594930	T	C	T	C	-4.02E-01	-3.94E-02	9.60E-03	9.89E-01	FALSE	FALSE	FALSE	8	26315218	1.11E-01	1.41E-01	TRUE	8	26457702	9.08E-02	0.97	6751	9.56E-06	TRUE	TRUE	482,730	19.63
Phosphatidylcholine (O-16:1_18:0) levels	Parkinson's disease	rs1440391	A	G	A	G	7.95E-02	2.25E-02	6.30E-01	3.02E-01	FALSE	FALSE	FALSE	14	48894805	2.40E-02	4.57E-01	TRUE	14	48425602	1.79E-02	1.00	6751	9.17E-06	TRUE	TRUE	482,730	19.71
Phosphatidylcholine (O-16:1_18:0) levels	Parkinson's disease	rs1667515	A	G	A	G	-8.73E-02	2.30E-03	7.01E-01	3.72E-01	FALSE	FALSE	FALSE	14	85693904	2.31E-02	3.61E-02	TRUE	14	85227560	1.87E-02	1.00	6751	3.27E-06	TRUE	TRUE	482,730	21.69
Phosphatidylcholine (O-16:1_18:0) levels	Parkinson's disease	rs181154147	T	C	T	C	2.21E-01	-8.78E-02	3.20E-02	9.78E-01	FALSE	FALSE	FALSE	12	70019950	8.34E-02	5.33E-01	TRUE	12	69626170	4.92E-02	0.98	6751	7.02E-06	TRUE	TRUE	482,730	20.22
Phosphatidylcholine (O-16:1_18:0) levels	Parkinson's disease	rs4565878	A	G	A	G	-8.65E-02	-5.68E-02	2.59E-01	7.34E-01	FALSE	FALSE	FALSE	11	3225702	2.56E-02	1.33E-02	TRUE	11	3204472	1.94E-02	1.00	6751	8.27E-06	TRUE	TRUE	482,730	19.90
Phosphatidylcholine (O-16:1_18:0) levels	Parkinson's disease	rs77512406	T	C	T	C	-1.34E-01	-3.50E-03	1.30E-01	9.40E-01	FALSE	FALSE	FALSE	11	66738763	4.27E-02	2.96E-02	TRUE	11	66971292	2.59E-02	0.99	6751	2.65E-07	TRUE	TRUE	482,730	26.54
Phosphatidylcholine (O-16:1_18:0) levels	Parkinson's disease	rs9997698	C	T	C	T	-1.77E-01	-5.29E-02	5.60E-02	9.11E-01	FALSE	FALSE	FALSE	4	141957740	3.97E-02	7.38E-01	TRUE	4	141036586	3.78E-02	0.98	6751	2.73E-06	TRUE	TRUE	482,730	22.03
Phosphatidylcholine (O-16:1_18:1) levels	Parkinson's disease	rs116438463	A	T	A	T	-1.79E-01	-1.70E-01	5.75E-02	1.80E-02	TRUE	FALSE	FALSE	4	151785511	1.08E-01	9.39E-01	TRUE	4	150864359	3.62E-02	0.98	7154	7.28E-07	TRUE	TRUE	482,730	24.58
Phosphatidylcholine (O-16:1_18:1) levels	Parkinson's disease	rs116614673	A	G	A	G	-1.26E-01	1.80E-02	9.62E-02	9.46E-01	FALSE	FALSE	FALSE	3	30210392	5.11E-02	1.40E-01	TRUE	3	30168901	2.84E-02	0.99	7154	9.54E-06	TRUE	TRUE	482,730	19.63
Phosphatidylcholine (O-16:1_18:1) levels	Parkinson's disease	rs116943761	A	C	A	C	-1.60E-01	-1.10E-03	8.22E-02	9.30E-01	FALSE	FALSE	FALSE	14	67974052	3.46E-02	1.14E-02	TRUE	14	67507335	3.06E-02	0.99	7154	1.74E-07	TRUE	TRUE	482,730	27.36
Phosphatidylcholine (O-16:1_18:1) levels	Parkinson's disease	rs116948429	T	G	T	G	-2.08E-01	-1.12E-01	3.41E-02	9.84E-01	FALSE	FALSE	FALSE	14	46305987	1.01E-01	5.75E-01	TRUE	14	45836784	4.61E-02	0.98	7154	6.36E-06	TRUE	TRUE	482,730	20.41
Phosphatidylcholine (O-16:1_18:1) levels	Parkinson's disease	rs117331519	T	G	T	G	-4.28E-01	-4.64E-02	8.31E-03	9.80E-01	FALSE	FALSE	FALSE	7	44352509	8.32E-02	2.39E-01	TRUE	7	44312910	9.47E-02	0.97	7154	6.37E-06	TRUE	TRUE	482,730	20.40
Phosphatidylcholine (O-16:1_18:1) levels	Parkinson's disease	rs117722620	A	G	A	G	2.74E-01	-3.74E-02	2.47E-02	9.67E-01	FALSE	FALSE	FALSE	11	131617859	6.90E-02	2.31E-01	TRUE	11	131747965	5.57E-02	0.92	7154	9.31E-07	TRUE	TRUE	482,730	24.11
Phosphatidylcholine (O-16:1_18:1) levels	Parkinson's disease	rs12426108	C	T	C	T	-1.56E-01	-8.40E-03	8.28E-02	9.69E-01	FALSE	FALSE	FALSE	12	68882906	6.05E-02	5.08E-02	TRUE	12	68489126	3.05E-02	0.98	7154	3.18E-07	TRUE	TRUE	482,730	26.19
Phosphatidylcholine (O-16:1_18:1) levels	Parkinson's disease	rs143615441	C	A	C	A	-4.43E-01	4.38E-02	1.01E-02	9.86E-01	FALSE	FALSE	FALSE	14	95947301	1.21E-01	1.44E-01	TRUE	14	95480964	8.96E-02	0.85	7154	7.69E-07	TRUE	TRUE	482,730	24.48
Phosphatidylcholine (O-16:1_18:1) levels	Parkinson's disease	rs1441162	T	G	T	G	-1.26E-01	-1.01E-02	1.20E-01	9.19E-01	FALSE	FALSE	FALSE	2	182454777	3.92E-02	9.91E-02	TRUE	2	181590050	2.57E-02	1.00	7154	8.95E-07	TRUE	TRUE	482,730	24.18
Phosphatidylcholine (O-16:1_18:1) levels	Parkinson's disease	rs150860836	G	A	G	A	-2.04E-01	-3.79E-02	3.82E-02	9.85E-01	FALSE	FALSE	FALSE	3	73226482	1.78E-01	8.03E-02	TRUE	3	73177331	4.47E-02	0.95	7154	5.22E-06	TRUE	TRUE	482,730	20.78
Phosphatidylcholine (O-16:1_18:1) levels	Parkinson's disease	rs17231506	T	C	T	C	9.44E-02	1.36E-02	2.78E-01	6.87E-01	FALSE	FALSE	FALSE	16	56994528	1.81E-02	3.45E-01	TRUE	16	56960616	1.86E-02	1.00	7154	3.84E-07	TRUE	TRUE	482,730	25.82
Phosphatidylcholine (O-16:1_18:1) levels	Parkinson's disease	rs1800588	T	C	T	C	8.88E-02	-3.30E-03	2.58E-01	7.75E-01	FALSE	FALSE	FALSE	15	58723675	2.12E-02	5.73E-02	TRUE	15	58431476	1.94E-02	0.99	7154	4.79E-06	TRUE	TRUE	482,730	20.95
Phosphatidylcholine (O-16:1_18:1) levels	Parkinson's disease	rs3012735	A	G	A	G	7.74E-02	-5.50E-03	3.98E-01	5.28E-01	FALSE	FALSE	FALSE	9	134651561	2.23E-02	9.47E-02	TRUE	9	131776174	1.70E-02	1.00	7154	5.58E-06	TRUE	TRUE	482,730	20.66
Phosphatidylcholine (O-16:1_18:1) levels	Parkinson's disease	rs34040476	T	C	T	C	1.93E-01	-1.02E-01	4.31E-02	9.63E-01	FALSE	FALSE	FALSE	10	112691809	6.69E-02	8.96E-01	TRUE	10	110932051	4.29E-02	0.92	7154	6.59E-06	TRUE	TRUE	482,730	20.34
Phosphatidylcholine (O-16:1_18:1) levels	Parkinson's disease	rs35293298	G	A	G	A	-1.05E-01	-1.10E-02	1.50E-01	8.13E-01	FALSE	FALSE	FALSE	7	104411122	2.89E-02	1.53E-01	TRUE	7	104770675	2.33E-02	0.99	7154	7.35E-06	TRUE	TRUE	482,730	20.13
Phosphatidylcholine (O-16:1_18:1) levels	Parkinson's disease	rs60426605	G	A	G	A	2.50E-01	-2.16E-02	2.26E-02	9.61E-01	FALSE	FALSE	FALSE	12	129926168	5.80E-02	1.49E-01	TRUE	12	129441623	5.62E-02	1.00	7154	8.77E-06	TRUE	TRUE	482,730	19.79
Phosphatidylcholine (O-16:1_18:1) levels	Parkinson's disease	rs6672758	T	C	T	C	1.19E-01	-2.10E-03	7.42E-01	2.04E-01	FALSE	FALSE	FALSE	1	230303512	2.37E-02	3.19E-02	TRUE	1	230167766	1.89E-02	1.00	7154	3.78E-10	TRUE	TRUE	482,730	39.33
Phosphatidylcholine (O-16:1_18:1) levels	Parkinson's disease	rs7190500	C	A	C	A	-9.84E-02	-1.46E-02	8.14E-01	1.13E-01	FALSE	FALSE	FALSE	16	76898425	3.59E-02	1.64E-01	TRUE	16	76864528	2.17E-02	0.99	7154	5.62E-06	TRUE	TRUE	482,730	20.64
Phosphatidylcholine (O-16:1_18:1) levels	Parkinson's disease	rs73162481	C	T	C	T	1.09E-01	-3.90E-03	1.64E-01	7.86E-01	FALSE	FALSE	FALSE	7	136888003	2.84E-02	5.01E-02	TRUE	7	137203256	2.28E-02	0.96	7154	1.64E-06	TRUE	TRUE	482,730	23.01
Phosphatidylcholine (O-16:1_18:1) levels	Parkinson's disease	rs74629446	T	A	T	A	-4.53E-01	1.96E-01	7.07E-03	1.10E-02	TRUE	FALSE	FALSE	9	73524769	1.59E-01	6.66E-01	TRUE	9	70909853	1.01E-01	0.96	7154	8.26E-06	TRUE	TRUE	482,730	19.90
Phosphatidylcholine (O-16:1_18:1) levels	Parkinson's disease	rs7677028	G	C	G	C	3.28E-01	2.67E-02	1.43E-02	2.61E-02	TRUE	FALSE	FALSE	4	137943535	7.37E-02	1.44E-01	TRUE	4	137022381	7.05E-02	0.97	7154	3.28E-06	TRUE	TRUE	482,730	21.68
Phosphatidylcholine (O-16:1_18:1) levels	Parkinson's disease	rs964184	C	G	C	G	1.53E-01	-5.68E-02	8.49E-01	8.63E-01	TRUE	FALSE	FALSE	11	116648917	2.43E-02	9.71E-03	TRUE	11	116778201	2.32E-02	1.00	7154	4.01E-11	TRUE	TRUE	482,730	43.74
Phosphatidylcholine (O-16:1_18:2) levels	Parkinson's disease	rs1077834	C	T	C	T	1.00E-01	-3.20E-03	2.66E-01	7.60E-01	FALSE	FALSE	FALSE	15	58723479	2.20E-02	5.35E-02	TRUE	15	58431280	1.93E-02	0.98	7096	2.10E-07	TRUE	TRUE	482,730	26.99
Phosphatidylcholine (O-16:1_18:2) levels	Parkinson's disease	rs10859677	G	A	G	A	-8.06E-02	4.20E-03	6.05E-01	3.88E-01	FALSE	FALSE	FALSE	12	94535947	2.34E-02	6.68E-02	TRUE	12	94142171	1.77E-02	0.96	7096	5.27E-06	TRUE	TRUE	482,730	20.77
Phosphatidylcholine (O-16:1_18:2) levels	Parkinson's disease	rs114517755	C	A	C	A	6.53E-01	-1.31E-01	5.10E-03	9.82E-01	FALSE	FALSE	FALSE	2	182644257	9.51E-02	7.73E-01	TRUE	2	181779530	1.29E-01	0.87	7096	4.31E-07	TRUE	TRUE	482,730	25.59
Phosphatidylcholine (O-16:1_18:2) levels	Parkinson's disease	rs117002628	A	G	A	G	5.11E-01	1.69E-01	6.25E-03	9.88E-01	FALSE	FALSE	FALSE	12	46874072	1.92E-01	4.24E-01	TRUE	12	46480289	1.11E-01	0.91	7096	4.41E-06	TRUE	TRUE	482,730	21.11
Phosphatidylcholine (O-16:1_18:2) levels	Parkinson's disease	rs11918522	A	G	A	G	1.09E-01	-3.09E-02	1.59E-01	8.27E-01	FALSE	FALSE	FALSE	3	112535057	2.25E-02	7.70E-01	TRUE	3	112816210	2.30E-02	1.00	7096	2.08E-06	TRUE	TRUE	482,730	22.56
Phosphatidylcholine (O-16:1_18:2) levels	Parkinson's disease	rs12426108	C	T	C	T	-1.48E-01	-8.40E-03	8.28E-02	9.69E-01	FALSE	FALSE	FALSE	12	68882906	6.05E-02	5.08E-02	TRUE	12	68489126	3.07E-02	0.98	7096	1.52E-06	TRUE	TRUE	482,730	23.16
Phosphatidylcholine (O-16:1_18:2) levels	Parkinson's disease	rs138542536	C	A	C	A	-3.57E-01	-1.28E-01	1.33E-02	9.88E-01	FALSE	FALSE	FALSE	4	25797744	1.68E-01	3.48E-01	TRUE	4	25796122	7.92E-02	0.86	7096	6.61E-06	TRUE	TRUE	482,730	20.33
Phosphatidylcholine (O-16:1_18:2) levels	Parkinson's disease	rs139013784	A	G	A	G	-3.61E-01	-9.26E-02	1.16E-02	9.89E-01	FALSE	FALSE	FALSE	17	48334348	1.78E-01	2.20E-01	TRUE	17	50256987	8.15E-02	0.94	7096	9.33E-06	TRUE	TRUE	482,730	19.67
Phosphatidylcholine (O-16:1_18:2) levels	Parkinson's disease	rs143533648	T	C	T	C	2.52E-01	-3.09E-02	2.62E-02	9.87E-01	FALSE	FALSE	FALSE	2	215442659	1.62E-01	7.12E-02	TRUE	2	214577935	5.36E-02	0.95	7096	2.71E-06	TRUE	TRUE	482,730	22.04
Phosphatidylcholine (O-16:1_18:2) levels	Parkinson's disease	rs17194174	A	G	A	G	1.65E-01	-2.70E-03	5.90E-02	8.14E-01	FALSE	FALSE	FALSE	6	30061910	2.52E-02	3.80E-02	TRUE	6	30094133	3.64E-02	1.00	7096	5.74E-06	TRUE	TRUE	482,730	20.60
Phosphatidylcholine (O-16:1_18:2) levels	Parkinson's disease	rs17231506	T	C	T	C	1.28E-01	1.36E-02	2.78E-01	6.87E-01	FALSE	FALSE	FALSE	16	56994528	1.81E-02	3.45E-01	TRUE	16	56960616	1.86E-02	1.00	7096	5.42E-12	TRUE	TRUE	482,730	47.69
Phosphatidylcholine (O-16:1_18:2) levels	Parkinson's disease	rs180360	G	A	G	A	8.60E-02	4.95E-02	3.24E-01	7.22E-01	FALSE	FALSE	FALSE	11	116598988	2.12E-02	9.77E-03	TRUE	11	116728272	1.80E-02	1.00	7096	1.69E-06	TRUE	TRUE	482,730	22.95
Phosphatidylcholine (O-16:1_18:2) levels	Parkinson's disease	rs186412131	A	G	A	G	-2.07E-01	-3.16E-01	3.56E-02	9.78E-01	FALSE	FALSE	FALSE	12	125277066	2.13E-01	8.60E-01	TRUE	12	124792520	4.61E-02	0.95	7096	6.79E-06	TRUE	TRUE	482,730	20.28
Phosphatidylcholine (O-16:1_18:2) levels	Parkinson's disease	rs2670896	A	G	A	G	-2.34E-01	2.67E-02	3.03E-02	9.62E-01	FALSE	FALSE	FALSE	3	129703565	5.61E-02	1.98E-01	TRUE	3	129984722	4.98E-02	1.00	7096	2.76E-06	TRUE	TRUE	482,730	22.01
Phosphatidylcholine (O-16:1_18:2) levels	Parkinson's disease	rs61999264	A	G	A	G	-3.44E-01	-2.15E-02	1.28E-02	9.80E-01	FALSE	FALSE	FALSE	5	148626067	8.43E-02	9.73E-02	TRUE	5	149246504	7.45E-02	0.99	7096	3.86E-06	TRUE	TRUE	482,730	21.36
Phosphatidylcholine (O-16:1_18:2) levels	Parkinson's disease	rs627108	T	C	T	C	-8.59E-02	7.60E-03	3.96E-01	6.49E-01	FALSE	FALSE	FALSE	1	230310568	1.98E-02	1.55E-01	TRUE	1	230174822	1.71E-02	0.99	7096	5.20E-07	TRUE	TRUE	482,730	25.23
Phosphatidylcholine (O-16:1_18:2) levels	Parkinson's disease	rs76103105	A	G	A	G	-2.12E-01	3.06E-02	3.57E-02	9.55E-01	FALSE	FALSE	FALSE	10	4741769	6.48E-02	1.96E-01	TRUE	10	4699577	4.60E-02	0.96	7096	4.23E-06	TRUE	TRUE	482,730	21.19
Phosphatidylcholine (O-16:1_18:2) levels	Parkinson's disease	rs77172221	C	T	C	T	-1.37E-01	-7.10E-03	8.98E-02	9.42E-01	FALSE	FALSE	FALSE	12	23232443	5.06E-02	5.16E-02	TRUE	12	23079509	2.99E-02	0.97	7096	4.53E-06	TRUE	TRUE	482,730	21.06
Phosphatidylcholine (O-16:1_18:2) levels	Parkinson's disease	rs9666651	T	C	T	C	8.46E-02	-2.34E-02	2.63E-01	7.37E-01	FALSE	FALSE	FALSE	11	34832436	2.10E-02	5.76E-01	TRUE	11	34810889	1.91E-02	0.99	7096	9.86E-06	TRUE	TRUE	482,730	19.57
Phosphatidylcholine (O-16:1_20:3) levels	Parkinson's disease	rs1077989	C	A	C	A	-1.21E-01	-2.34E-02	4.68E-01	5.22E-01	FALSE	FALSE	FALSE	14	67975822	1.67E-02	7.91E-01	TRUE	14	67509105	1.72E-02	1.00	6770	1.70E-12	TRUE	TRUE	482,730	49.98
Phosphatidylcholine (O-16:1_20:3) levels	Parkinson's disease	rs112516243	G	T	G	T	7.59E-01	-4.31E-02	2.93E-03	9.86E-01	FALSE	FALSE	FALSE	13	27611965	1.04E-01	1.69E-01	TRUE	13	27037828	1.70E-01	0.92	6770	8.27E-06	TRUE	TRUE	482,730	19.90
Phosphatidylcholine (O-16:1_20:3) levels	Parkinson's disease	rs11883812	C	T	C	T	-2.20E-01	7.60E-03	4.86E-02	9.05E-01	FALSE	FALSE	FALSE	2	216256981	3.15E-02	9.14E-02	TRUE	2	215392258	4.07E-02	0.98	6770	6.86E-08	TRUE	TRUE	482,730	29.17
Phosphatidylcholine (O-16:1_20:3) levels	Parkinson's disease	rs1203148	G	A	G	A	9.27E-02	-5.07E-02	2.40E-01	7.90E-01	FALSE	FALSE	FALSE	1	35968899	2.72E-02	3.12E-02	TRUE	1	35503298	2.08E-02	0.93	6770	8.17E-06	TRUE	TRUE	482,730	19.93
Phosphatidylcholine (O-16:1_20:3) levels	Parkinson's disease	rs144750125	T	C	T	C	2.59E-01	9.05E-02	2.45E-02	9.83E-01	FALSE	FALSE	FALSE	17	15445234	8.01E-02	5.87E-01	TRUE	17	15541920	5.54E-02	0.99	6770	3.17E-06	TRUE	TRUE	482,730	21.75
Phosphatidylcholine (O-16:1_20:3) levels	Parkinson's disease	rs254868	G	T	G	T	-1.02E-01	4.63E-02	1.72E-01	7.95E-01	FALSE	FALSE	FALSE	7	111664804	2.81E-02	4.97E-02	TRUE	7	112024749	2.29E-02	0.98	6770	8.91E-06	TRUE	TRUE	482,730	19.76
Phosphatidylcholine (O-16:1_20:3) levels	Parkinson's disease	rs34455876	A	G	A	G	-8.73E-02	-1.18E-02	3.13E-01	5.97E-01	FALSE	FALSE	FALSE	7	129794579	2.02E-02	2.52E-01	TRUE	7	130154739	1.88E-02	0.98	6770	3.40E-06	TRUE	TRUE	482,730	21.61
Phosphatidylcholine (O-16:1_20:3) levels	Parkinson's disease	rs35556451	T	C	T	C	-2.26E-01	-5.02E-02	3.57E-02	9.63E-01	FALSE	FALSE	FALSE	12	28839803	6.00E-02	3.95E-01	TRUE	12	28686870	4.67E-02	1.00	6770	1.28E-06	TRUE	TRUE	482,730	23.49
Phosphatidylcholine (O-16:1_20:3) levels	Parkinson's disease	rs3795169	T	C	T	C	1.19E-01	5.66E-02	1.44E-01	8.62E-01	FALSE	FALSE	FALSE	4	37859675	3.28E-02	4.22E-02	TRUE	4	37858054	2.47E-02	0.99	6770	1.51E-06	TRUE	TRUE	482,730	23.17
Phosphatidylcholine (O-16:1_20:3) levels	Parkinson's disease	rs55665216	T	C	T	C	1.52E-01	-3.22E-02	7.98E-02	9.10E-01	FALSE	FALSE	FALSE	12	7143389	3.14E-02	5.17E-01	TRUE	12	7036085	3.16E-02	0.99	6770	1.59E-06	TRUE	TRUE	482,730	23.07
Phosphatidylcholine (O-16:1_20:3) levels	Parkinson's disease	rs56142013	T	C	T	C	-3.49E-01	3.97E-02	1.39E-02	9.80E-01	FALSE	FALSE	FALSE	14	56897947	8.96E-02	1.82E-01	TRUE	14	56431229	7.60E-02	0.92	6770	4.31E-06	TRUE	TRUE	482,730	21.15

Phosphatidylcholine (O-16:1_20:3) levels	Parkinson's disease	rs58105955	G	A	G	A	1.34E-01	-4.09E-02	9.51E-02	8.83E-01	FALSE	FALSE	7	73884852	3.87E-02	5.38E-01	TRUE	7	74470522	2.95E-02	0.96	6770	6.06E-06	TRUE	TRUE	482,730	20.50
Phosphatidylcholine (O-16:1_20:3) levels	Parkinson's disease	rs61896141	C	A	C	A	2.20E-01	-3.28E-02	1.13E-01	8.31E-01	FALSE	FALSE	11	61556039	2.26E-02	8.35E-01	TRUE	11	61788567	2.69E-02	1.00	6770	3.61E-16	TRUE	TRUE	482,730	66.77
Phosphatidylcholine (O-16:1_20:3) levels	Parkinson's disease	rs6498540	G	A	G	A	-1.47E-01	1.82E-02	3.34E-01	6.94E-01	FALSE	FALSE	16	15130594	1.84E-02	4.93E-01	TRUE	16	15036737	1.83E-02	0.99	6770	8.85E-16	TRUE	TRUE	482,730	64.98
Phosphatidylcholine (O-16:1_20:3) levels	Parkinson's disease	rs6815866	C	G	C	G	-1.34E-01	9.93E-02	8.91E-01	9.46E-01	TRUE	FALSE	4	96040868	5.28E-02	3.00E-02	TRUE	4	95119717	2.74E-02	0.99	6770	1.03E-06	TRUE	TRUE	482,730	23.91
Phosphatidylcholine (O-16:1_20:3) levels	Parkinson's disease	rs6817929	G	A	G	A	9.19E-02	2.20E-02	5.88E-01	5.69E-01	FALSE	FALSE	4	57070248	2.25E-02	4.86E-01	TRUE	4	56204082	1.76E-02	1.00	6770	1.90E-07	TRUE	TRUE	482,730	27.19
Phosphatidylcholine (O-16:1_20:3) levels	Parkinson's disease	rs686843	A	G	A	G	8.25E-02	-3.20E-03	4.80E-01	5.15E-01	FALSE	FALSE	1	61374136	2.36E-02	4.91E-02	TRUE	1	60908464	1.78E-02	0.94	6770	3.83E-06	TRUE	TRUE	482,730	21.38
Phosphatidylcholine (O-16:1_20:3) levels	Parkinson's disease	rs7874842	A	G	A	G	9.93E-02	-5.09E-02	1.90E-01	8.35E-01	FALSE	FALSE	9	16476971	3.01E-02	4.54E-02	TRUE	9	16476973	2.17E-02	1.00	6770	5.08E-06	TRUE	TRUE	482,730	20.84
Phosphatidylcholine (O-16:1_20:3) levels	Parkinson's disease	rs80320850	A	G	A	G	-1.79E-01	4.04E-02	5.19E-02	9.73E-01	FALSE	FALSE	12	61613187	8.45E-02	1.99E-01	TRUE	12	61219406	3.87E-02	1.00	6770	3.99E-06	TRUE	TRUE	482,730	21.30
Phosphatidylcholine (O-16:1_20:3) levels	Parkinson's disease	rs9602749	G	T	G	T	1.14E-01	1.01E-01	1.47E-01	8.87E-01	FALSE	FALSE	13	86008924	3.54E-02	2.11E-03	TRUE	13	85434789	2.45E-02	0.99	6770	3.30E-06	TRUE	TRUE	482,730	21.67
Phosphatidylcholine (O-16:1_20:3) levels	Parkinson's disease	rs9872756	A	G	A	G	9.88E-02	2.44E-02	7.49E-01	2.41E-01	FALSE	FALSE	3	87618331	2.62E-02	4.53E-01	TRUE	3	87569181	1.98E-02	0.99	6770	6.28E-07	TRUE	TRUE	482,730	24.87
Phosphatidylcholine (O-16:1_20:4) levels	Parkinson's disease	rs1037782	C	T	C	T	-1.73E-01	-5.34E-02	6.11E-02	8.90E-01	FALSE	FALSE	15	29906948	3.56E-02	8.75E-01	TRUE	15	29614744	3.79E-02	0.99	6025	5.26E-06	TRUE	TRUE	482,730	20.78
Phosphatidylcholine (O-16:1_20:4) levels	Parkinson's disease	rs10487877	G	C	G	C	2.09E-01	1.13E-01	4.73E-02	1.84E-02	TRUE	FALSE	7	80467301	1.35E-01	3.93E-01	TRUE	7	80837985	4.32E-02	0.98	6025	1.29E-06	TRUE	TRUE	482,730	23.48
Phosphatidylcholine (O-16:1_20:4) levels	Parkinson's disease	rs1077989	C	A	C	A	-1.92E-01	-2.34E-02	4.68E-01	5.22E-01	FALSE	FALSE	14	67975822	1.67E-02	7.91E-01	TRUE	14	67509105	1.81E-02	1.00	6025	3.43E-26	TRUE	TRUE	482,730	113.13
Phosphatidylcholine (O-16:1_20:4) levels	Parkinson's disease	rs12579775	A	G	A	G	1.60E-01	-2.25E-02	8.21E-02	9.05E-01	FALSE	FALSE	12	7085171	3.07E-02	3.33E-01	TRUE	12	6976009	3.28E-02	0.99	6025	1.05E-06	TRUE	TRUE	482,730	23.89
Phosphatidylcholine (O-16:1_20:4) levels	Parkinson's disease	rs12920974	T	G	T	G	-1.04E-01	-2.37E-02	3.30E-01	7.16E-01	FALSE	FALSE	16	56993025	1.95E-02	6.51E-01	TRUE	16	56959113	1.95E-02	0.99	6025	1.16E-07	TRUE	TRUE	482,730	28.16
Phosphatidylcholine (O-16:1_20:4) levels	Parkinson's disease	rs1316133	A	G	A	G	-9.00E-02	1.71E-02	2.99E-01	6.58E-01	FALSE	FALSE	14	67938638	2.15E-02	3.71E-01	TRUE	14	67471921	2.02E-02	0.97	6025	8.71E-06	TRUE	TRUE	482,730	19.81
Phosphatidylcholine (O-16:1_20:4) levels	Parkinson's disease	rs138018897	G	A	G	A	-2.99E-01	-7.10E-02	2.15E-02	9.87E-01	FALSE	FALSE	3	19223408	1.26E-01	2.43E-01	TRUE	3	19181916	6.53E-02	0.93	6025	4.74E-06	TRUE	TRUE	482,730	20.98
Phosphatidylcholine (O-16:1_20:4) levels	Parkinson's disease	rs17268903	G	A	G	A	-1.26E-01	8.10E-03	1.21E-01	9.32E-01	FALSE	FALSE	2	56745940	4.87E-02	6.13E-02	TRUE	2	56518805	2.82E-02	0.97	6025	8.32E-06	TRUE	TRUE	482,730	19.90
Phosphatidylcholine (O-16:1_20:4) levels	Parkinson's disease	rs174535	C	T	C	T	-4.04E-01	-2.60E-03	4.09E-01	6.66E-01	FALSE	FALSE	11	61551356	1.78E-02	5.31E-02	TRUE	11	61783884	1.79E-02	1.00	6025	1.18E-107	TRUE	TRUE	482,730	505.91
Phosphatidylcholine (O-16:1_20:4) levels	Parkinson's disease	rs180360	G	A	G	A	8.81E-02	4.95E-02	3.24E-01	7.22E-01	FALSE	FALSE	11	116598988	2.12E-02	9.77E-03	TRUE	11	116728272	1.95E-02	1.00	6025	6.34E-06	TRUE	TRUE	482,730	20.42
Phosphatidylcholine (O-16:1_20:4) levels	Parkinson's disease	rs2015950	A	G	A	G	-1.34E-01	-2.93E-02	1.37E-01	8.35E-01	FALSE	FALSE	11	61825530	2.71E-02	5.56E-01	TRUE	11	62058058	2.67E-02	1.00	6025	5.32E-07	TRUE	TRUE	482,730	25.20
Phosphatidylcholine (O-16:1_20:4) levels	Parkinson's disease	rs2043085	C	T	C	T	-9.99E-02	-2.04E-02	5.76E-01	3.79E-01	FALSE	FALSE	15	58680954	1.75E-02	6.16E-01	TRUE	15	58388755	1.83E-02	1.00	6025	4.75E-08	TRUE	TRUE	482,730	29.89
Phosphatidylcholine (O-16:1_20:4) levels	Parkinson's disease	rs5028238	A	G	A	G	-9.65E-02	3.90E-02	7.65E-01	2.44E-01	FALSE	FALSE	2	218207688	2.64E-02	8.57E-01	TRUE	2	217342965	2.15E-02	0.99	6025	7.68E-06	TRUE	TRUE	482,730	20.05
Phosphatidylcholine (O-16:1_20:4) levels	Parkinson's disease	rs56303140	A	G	A	G	2.33E-01	-2.84E-02	3.45E-02	9.67E-01	FALSE	FALSE	2	158655452	6.78E-02	1.71E-01	TRUE	2	157798940	5.09E-02	0.94	6025	4.65E-06	TRUE	TRUE	482,730	21.01
Phosphatidylcholine (O-16:1_20:4) levels	Parkinson's disease	rs6752137	A	G	A	G	-1.29E-01	-8.00E-03	1.20E-01	9.19E-01	FALSE	FALSE	2	182469166	3.93E-02	7.60E-02	TRUE	2	181604439	2.77E-02	1.00	6025	3.46E-06	TRUE	TRUE	482,730	21.58
Phosphatidylcholine (O-16:1_20:4) levels	Parkinson's disease	rs6776300	A	C	A	C	8.16E-02	1.73E-02	5.34E-01	4.35E-01	FALSE	FALSE	3	153297048	2.23E-02	3.57E-01	TRUE	3	153579259	1.83E-02	1.00	6025	8.20E-06	TRUE	TRUE	482,730	19.92
Phosphatidylcholine (O-16:1_20:4) levels	Parkinson's disease	rs71366199	G	A	G	A	1.18E-01	-2.11E-02	1.48E-01	8.89E-01	FALSE	FALSE	17	15297187	3.83E-02	2.35E-01	TRUE	17	15393870	2.63E-02	0.95	6025	6.50E-06	TRUE	TRUE	482,730	20.37
Phosphatidylcholine (O-16:1_20:4) levels	Parkinson's disease	rs74854572	G	C	G	C	-3.94E-01	-1.62E-01	1.17E-02	1.14E-02	TRUE	FALSE	8	9225100	1.36E-01	6.31E-01	TRUE	8	9367590	8.73E-02	0.90	6025	6.39E-06	TRUE	TRUE	482,730	20.40
Phosphatidylcholine (O-16:1_20:4) levels	Parkinson's disease	rs7963111	T	G	T	G	-1.93E-01	-3.51E-02	4.95E-02	9.59E-01	FALSE	FALSE	12	125420170	5.08E-02	3.10E-01	TRUE	12	124935624	4.26E-02	0.93	6025	5.86E-06	TRUE	TRUE	482,730	20.57
Phosphatidylcholine (O-16:2_18:0) levels	Parkinson's disease	rs10869152	G	A	G	A	8.19E-02	5.02E-02	3.42E-01	6.56E-01	FALSE	FALSE	9	71237048	2.87E-02	4.01E-02	TRUE	9	68622132	1.84E-02	0.99	6672	8.30E-06	TRUE	TRUE	482,730	19.90
Phosphatidylcholine (O-16:2_18:0) levels	Parkinson's disease	rs1145605	G	A	G	A	-1.36E-01	-6.06E-02	1.16E-01	8.93E-01	FALSE	FALSE	5	157704332	3.69E-02	1.00E+00	TRUE	5	158277324	2.79E-02	0.95	6672	1.18E-06	TRUE	TRUE	482,730	23.65
Phosphatidylcholine (O-16:2_18:0) levels	Parkinson's disease	rs13433708	T	A	T	A	-9.39E-02	2.04E-02	3.29E-01	3.07E-01	TRUE	FALSE	3	4425281	2.45E-02	3.91E-01	TRUE	3	4383597	1.87E-02	0.98	6672	5.61E-07	TRUE	TRUE	482,730	25.09
Phosphatidylcholine (O-16:2_18:0) levels	Parkinson's disease	rs141656454	A	G	A	G	3.92E-01	4.31E-01	1.02E-02	9.90E-01	FALSE	FALSE	1	82895804	1.82E-01	8.92E-03	TRUE	1	82430121	8.80E-02	0.93	6672	8.63E-06	TRUE	TRUE	482,730	19.82
Phosphatidylcholine (O-16:2_18:0) levels	Parkinson's disease	rs184729252	A	G	A	G	8.36E-01	-3.84E-02	2.56E-03	9.88E-01	FALSE	FALSE	6	24525982	1.68E-01	8.66E-02	TRUE	6	24525754	1.76E-01	0.88	6672	2.14E-06	TRUE	TRUE	482,730	22.51
Phosphatidylcholine (O-16:2_18:0) levels	Parkinson's disease	rs3814533	T	C	T	C	-1.51E-01	1.83E-02	9.15E-01	9.78E-02	FALSE	FALSE	9	84252807	3.78E-02	2.02E-01	TRUE	9	81637892	3.15E-02	0.96	6672	1.77E-06	TRUE	TRUE	482,730	22.87
Phosphatidylcholine (O-16:2_18:0) levels	Parkinson's disease	rs55816615	A	G	A	G	9.31E-02	1.93E-02	2.38E-01	7.22E-01	FALSE	FALSE	13	42151045	2.53E-02	3.50E-01	TRUE	13	41576909	2.07E-02	0.97	6672	7.22E-06	TRUE	TRUE	482,730	20.17
Phosphatidylcholine (O-16:2_18:0) levels	Parkinson's disease	rs588136	T	C	T	C	-1.91E-01	-2.45E-02	7.58E-01	3.25E-01	FALSE	FALSE	15	58730498	2.28E-02	5.48E-01	TRUE	15	58438299	2.02E-02	1.00	6672	4.89E-21	TRUE	TRUE	482,730	89.18
Phosphatidylcholine (O-16:2_18:0) levels	Parkinson's disease	rs61703937	A	G	A	G	-1.43E-01	-8.39E-02	8.76E-02	9.07E-01	FALSE	FALSE	8	77537953	3.83E-02	1.42E-02	TRUE	8	76625718	3.11E-02	1.00	6672	4.26E-06	TRUE	TRUE	482,730	21.17
Phosphatidylcholine (O-16:2_18:0) levels	Parkinson's disease	rs6455474	C	G	C	G	2.99E-01	3.30E-02	1.81E-02	2.73E-02	TRUE	FALSE	6	168390987	7.06E-02	1.93E-01	TRUE	6	167990307	6.51E-02	0.97	6672	4.54E-06	TRUE	TRUE	482,730	21.06
Phosphatidylcholine (O-16:2_18:0) levels	Parkinson's disease	rs74684016	G	A	G	A	1.00E-01	-2.40E-03	1.92E-01	7.84E-01	FALSE	FALSE	12	118999226	2.74E-02	3.20E-02	TRUE	12	118561421	2.21E-02	0.99	6672	5.96E-06	TRUE	TRUE	482,730	20.53
Phosphatidylcholine (O-17:0_15:0) levels	Parkinson's disease	rs10225730	T	G	T	G	-2.38E-01	-4.94E-02	2.96E-02	9.59E-01	FALSE	FALSE	7	17687722	5.63E-02	4.20E-01	TRUE	7	17648098	5.38E-02	0.99	6140	9.76E-06	TRUE	TRUE	482,730	19.59
Phosphatidylcholine (O-17:0_15:0) levels	Parkinson's disease	rs112686420	G	A	G	A	4.70E-01	-8.25E-02	9.38E-03	9.86E-01	FALSE	FALSE	13	43476873	1.63E-01	2.13E-01	TRUE	13	42902737	9.89E-02	0.91	6140	2.02E-06	TRUE	TRUE	482,730	22.62
Phosphatidylcholine (O-17:0_15:0) levels	Parkinson's disease	rs116984749	G	A	G	A	2.43E-01	-4.00E-02	2.75E-02	9.66E-01	FALSE	FALSE	16	48380043	6.59E-02	2.64E-01	TRUE	16	48346132	5.49E-02	0.98	6140	9.83E-06	TRUE	TRUE	482,730	19.57
Phosphatidylcholine (O-17:0_15:0) levels	Parkinson's disease	rs141417792	G	T	G	T	2.66E-01	1.53E-01	2.38E-02	9.80E-01	FALSE	FALSE	11	114709398	8.21E-02	3.11E-02	TRUE	11	114838676	5.97E-02	0.97	6140	8.26E-06	TRUE	TRUE	482,730	19.91
Phosphatidylcholine (O-17:0_15:0) levels	Parkinson's disease	rs144941484	T	C	T	C	5.92E-01	2.63E-01	5.92E-03	9.89E-01	FALSE	FALSE	2	159777515	1.66E-01	9.43E-01	TRUE	2	158921003	1.22E-01	0.91	6140	1.28E-06	TRUE	TRUE	482,730	23.49
Phosphatidylcholine (O-17:0_15:0) levels	Parkinson's disease	rs145150348	A	G	A	G	3.37E-01	-3.11E-02	1.73E-02	9.71E-01	FALSE	FALSE	1	62587401	7.03E-02	1.82E-01	TRUE	1	62121729	6.89E-02	0.96	6140	1.05E-06	TRUE	TRUE	482,730	23.88
Phosphatidylcholine (O-17:0_15:0) levels	Parkinson's disease	rs146967253	T	C	T	C	2.85E-01	-2.10E-03	2.61E-02	9.75E-01	FALSE	FALSE	7	80798733	8.22E-02	9.00E-03	TRUE	7	81169417	6.00E-02	0.91	6140	2.05E-06	TRUE	TRUE	482,730	22.59
Phosphatidylcholine (O-17:0_15:0) levels	Parkinson's disease	rs149897605	C	T	C	T	-2.82E-01	9.10E-03	2.37E-02	9.86E-01	FALSE	FALSE	2	198238539	1.17E-01	2.77E-02	TRUE	2	197373815	6.21E-02	0.93	6140	5.62E-06	TRUE	TRUE	482,730	20.65
Phosphatidylcholine (O-17:0_15:0) levels	Parkinson's disease	rs417916	T	C	T	C	-8.79E-02	3.14E-02	4.40E-01	5.33E-01	FALSE	FALSE	7	41314465	2.37E-02	7.35E-01	TRUE	7	41274867	1.88E-02	0.93	6140	3.09E-06	TRUE	TRUE	482,730	21.80
Phosphatidylcholine (O-17:0_15:0) levels	Parkinson's disease	rs4949186	C	T	C	T	-9.71E-02	4.60E-03	7.00E-01	4.21E-01	FALSE	FALSE	1	31402148	2.29E-02	7.45E-02	TRUE	1	30929301	1.96E-02	0.99	6140	7.41E-07	TRUE	TRUE	482,730	24.55
Phosphatidylcholine (O-17:0_15:0) levels	Parkinson's disease	rs7204112	G	A	G	A	2.38E-01	3.65E-02	3.16E-02	9.49E-01	FALSE	FALSE	16	86972212	5.27E-02	3.11E-01	TRUE	16	86938606	5.26E-02	0.96	6140	6.35E-06	TRUE	TRUE	482,730	20.41
Phosphatidylcholine (O-17:0_15:0) levels	Parkinson's disease	rs72967793	T	A	T	A	-1.80E-01	3.54E-02	6.01E-02	7.19E-02	TRUE	FALSE	11	95673269	4.69E-02	3.47E-01	TRUE	11	95940105	3.86E-02	0.97	6140	2.99E-06	TRUE	TRUE	482,730	21.86
Phosphatidylcholine (O-17:0_15:0) levels	Parkinson's disease	rs74648736	T	A	T	A	-3.28E-01	9.95E-02	2.20E-02	2.94E-02	TRUE	FALSE	3	173447304	7.52E-02	7.31E-01	TRUE	3	173729514	7.17E-02	0.81	6140	4.79E-06	TRUE	TRUE	482,730	20.95
Phosphatidylcholine (O-17:0_15:0) levels	Parkinson's disease	rs748301	G	A	G	A	-8.39E-02	-3.40E-03	6.08E-01	4.28E-01	FALSE	FALSE	6	23380768	2.33E-02	5.38E-02	TRUE	6	23380540	1.87E-02	0.96	6140	7.53E-06	TRUE	TRUE	482,730	20.09
Phosphatidylcholine (O-17:0_15:0) levels	Parkinson's disease	rs76292244	C	A	C	A	-2.61E-01	6.19E-02	2.71E-02	9.84E-01	FALSE	FALSE	12	42802386	9.67E-02	2.82E-01	TRUE	12	42408584	5.73E-02	0.96	6140	5.52E-06	TRUE	TRUE	482,730	20.68
Phosphatidylcholine (O-17:0_15:0) levels	Parkinson's disease	rs76913081	T	G	T	G	-1.71E-01	4.94E-02	6.13E-02	8.93E-01	FALSE	FALSE	3	104374119	3.70E-02	7.38E-01	TRUE	3	104655275	3.78E-02	0.98	6140	6.58E-06	TRUE	TRUE	482,730	20.35
Phosphatidylcholine (O-17:0_17:1) levels	Parkinson's disease	rs10468017	T	C	T	C	8.67E-02	2.75E-02	3.38E-01	7.16E-01	FALSE	FALSE	15	58678512	1.87E-02	8.48E-01	TRUE	15	58386313	1.80E-02	1.00	6880	1.43E-06	TRUE	TRUE	482,730	23.28
Phosphatidylcholine (O-17:0_17:1) levels	Parkinson's disease	rs11068152	A	G	A	G	-9.43E-02	2.98E-02	2.23E-01	8.22E-01	FALSE	FALSE	12	117141011	2.94E-02	5.09E-01	TRUE	12	116703206	2.06E-02	0.99	6880	4.89E-06	TRUE	TRUE	482,730	20.91
Phosphatidylcholine (O-17:0_17:1) levels	Parkinson's disease	rs112832771	G	A	G	A	3.59E-01	8.88E-02	1.23E-02	9.62E-01	FALSE	FALSE	6	151422509	6.37E-02	7.87E-01	TRUE	6	151101373	7.97E-02	0.92	6880	6.61E-06	TRUE	TRUE	482,730	20.33
Phosphatidylcholine (O-17:0_17:1) levels	Parkinson's disease	rs11691441	C	G	C	G	-1.83E-01	2.80E-03	4.73E-02	8.08E-02	TRUE	FALSE	2	101473299	3.54E-02	2.84E-02	TRUE	2	100856837	3.99E-02	0.98	6880	4.67E-06	TRUE	TRUE	482,730	21.00
Phosphatidylcholine (O-17:0_17:1) levels	Parkinson's disease	rs11947730	C	T	C	T	9.28E-02	3.72E-02	7.37E-01	2.00E-01	FALSE	FALSE	4	6204935	2.98E-02	6.76E-01	TRUE	4	6203208	2.03E-02	0.92	6880	5.19E-06	TRUE	TRUE	482,730	20.79
Phosphatidylcholine (O-17:0_17:1) levels	Parkinson's disease	rs12186559	T	C	T	C	2.27E-01	1.37E-01	3.60E-02	9.66E-01	FALSE	FALSE	5	3950622	6.85E-02	2.27E-02	TRUE	5	3950508	4.67E-02	0.95	6880	1.19E-06	TRUE	TRUE	482,730	23.62
Phosphatidylcholine (O-17:0_17:1) levels	Parkinson's disease	rs151297234	C	G	C	G	2.40E-01	-1.55E-02	2.73E-02	4.03E-02	TRUE	FALSE	12	16805472	6.02E-02	9.88E-02	TRUE	12	16652538	5.30E-02	0.99	6880	6.18E-06	TRUE	TRUE	482,730	20.46
Phosphatidylcholine (O-17:0_17:1) levels	Parkinson's disease	rs174601	T	C	T	C	8.91E-02	-3.70E-03	4.19E-01	6.38E-01	FALSE	FALSE	11	61623140	1.79E-02	7.75E-02	TRUE	11	61855668	1.73E-02	1.00	6880	2.59E-07	TRUE	TRUE	482,730	26.59
Phosphatidylcholine (O-17:0_17:1) levels	Parkinson's disease	rs17513056	C	T	C	T	1.04E-01	-1.88E-02	2.12E-01	7.88E-01	FALSE	FALSE	6	159153925	2.73E-02	3.08E-01	TRUE	6	158732893	2.11E-02	0.99	6880	8.11E-07	TRUE	TRUE	482,730	24.37
Phosphatidylcholine (O-17:0_17:1) levels	Parkinson's disease	rs17611110	C	T	C	T	-9.98E-02	-4.57E-02	2.41E-01	6.63E-01	FALSE	FALSE	4	169140553	1.91E-02	8.36E-03	TRUE	4	168219402	2.00E-02	1.00	6880	5.86E-07	TRUE	TRUE	482,730	25.00
Phosphatidylcholine (O-17:0_17:1) levels	Parkinson's disease	rs1893460	A	G	A	G	9.05E-02	1.20E-02	2.36E-01	8.19E-01	FALSE	FALSE	11	116603677	2.24E-02	2.28E-01	TRUE	11	116732961	2.02E-02	1.00	6880	7.94E-06	TRUE	TRUE	482,730	19.98
Phosphatidylcholine (O-17:0_17:1) levels	Parkinson's disease	rs247617	A	C	A	C	1.20E-01	1.47E-02	2.78E-01	6.86E-01	FALSE	FALSE	16	56990716	1.80E-02	3.80E-01	TRUE	16	56956804	1.89E-02	1.00	6880	2.69E-10	TRUE	TRUE	482,730	40.01
Phosphatidylcholine (O-17:0_17:1) levels	Parkinson's disease	rs2725087	A	T	A	T	-2.55E-01	4.11E-02	2.43E-02	1.87E-02	TRUE	FALSE	8	4459506	8.42E-02	2.04E-01	TRUE	8	4601984	5.69E-02	0.98	6880	7.80E-06	TRUE	TRUE	482,730	20.02
Phosphatidylcholine (O-17:0_17:1) levels	Parkinson's disease	rs2784923	A	C	A	C	1.66E-01	5.50E-03	9.41E-01	8.20E-02	FALSE	FALSE	10	98909854	4.13E-02	4.86E-02	TRUE	10	97150097	3.68E-02	1.00	6880	6.83E-06	TRUE	TRUE	482,730	20.27
Phosphatidylcholine (O-17:0_17:1) levels	Parkinson's disease	rs28403152	C	G	C	G	-3.85E-01	1.76E-01	1.04E-02	2.52E-02	TRUE	FALSE	9	16670162	7.92E-02	1.30E-02	TRUE	9	16670164	8.65E-02	0.93	6880	8.72E-06	TRUE	TRUE	482,730	19.80
Phosphatidylcholine (O-17:0_17:1) levels	Parkinson's disease	rs3765259	C	T	C	T	-7.91E-02	-3.20E-03	4.27E-01	4.94E-01	FALSE	FALSE	6	136990283	2.38E-02	4.85E-02	TRUE	6	136669145	1.79E-02	0.93	6880	9.73E-06	TRUE	TRUE	482,730	19.59
Phosphatidylcholine (O-17:0_17:1) levels	Parkinson's disease	rs38820	C	G	C	G	1.40E-01	-5.29E-02	9.14E-01	8.48E-01	TRUE	FALSE	7	117710536	3.06E-02	4.19E-02	TRUE	7	118070482	3.06E-02	1.00	6880	4.96E-06	TRUE	TRUE	482,730	20.89
Phosphatidylcholine (O-17:0_17:1) levels	Parkinson's disease	rs4517092	C	T	C	T	2.90E-01	-2.55E-02	1.83E-02	9.73E-01	FALSE	FALSE	8	98987226	7.25E-02	1.40E-01	TRUE	8	97974998	6.52E-02	0.98	6880	9.06E-06	TRUE	TRUE	482,730	19.73
Phosphatidylcholine (O-17:0_17:1) levels	Parkinson's disease	rs612577	C	T	C	T	-1.14E-01	1.87E-02	1.80E-01	8.53E-01	FALSE	FALSE	1	230324364	2.85E-02	2.91E-01	TRUE	1	230188618	2.20E-02	1.00	6880	2.01E-07	TRUE	TRUE	482,730	27.08
Phosphatidylcholine (O-17:0_17:1) levels	Parkinson's disease	rs61644965	T	C	T	C	-2.35E-01	5.01E-02	2.73E-02	9.83E-01	FALSE	FALSE	13	28053426	8.79E-02	2.45E-01	TRUE	13	27479289	5.22E-02	1.00	6880	6.56E-06	TRUE	TRUE	482,730	20.35
Phosphatidylcholine (O-17:0_17:1) levels	Parkinson's disease	rs62072490	T	G	T	G	-1.08E-01	4.00E-03	1.94E-01	6.61E-01	FALSE	FALSE	17	4914145	2.48E-02	5.90E-02	TRUE	17	5010850	2.19E-02	0.95	6880	8.69E-07	TRUE	TRUE	482,730	24.24
Phosphatidylcholine (O-17:0_17:1) levels	Parkinson's disease	rs709981	A	C	A	C	7.66E-02	5.00E-03	4.67E-01	5.18E-01	FALSE	FALSE	14	57403967	2.10E-02	9.15E-02	TRUE	14	56937249	1.72E-02	0.99	6880	8.76E-06	TRUE	TRUE	482,730	19.79
Phosphatidylcholine (O-17:0_17:1) levels	Parkinson's disease	rs72975671	T	A	T	A	9.47E-02	-7.53E-02	2.15E-01	1.93E-01	TRUE	FALSE	3	135531813	2.25E-02	4.09E-04	TRUE	3	135812971	2.08E-02	0.99	6880	5.24E-06	TRUE	TRUE	482,730	20.78
Phosphatidylcholine (O-17:0_17:1) levels	Parkinson's disease	rs75206639	A	G	A	G	-3.35E-01	8.17E-02	1.49E-02	9.75E-01	FALSE	FALSE	16	79103380	8.23E-02	4.94E-01	TRUE	16	79069483	7.55E-02	0.89	6880	9.02E-06	TRUE	TRUE	482,730	19.73
Phosphatidylcholine (O-17:0_17:1) levels	Parkinson's disease	rs7575011	G	T	G	T	-8.24E-02	2.54E-02	6.62E-01	3.17E-01	FALSE	FALSE	2	114432890	2.38E-02	5.42E-01	TRUE	2	113675313	1.80E-02	1.00	6880	4.63E-06	TRUE	TRUE	482,730	21.02
Phosphatidylcholine (O-17:0_17:1) levels	Parkinson's disease	rs7597998	C	T	C	T	9.04E-02	-9.40E-03	7.65E-01	3.74E-01	FALSE	FALSE	2	193032355	2.75E-02	1.35E-01	TRUE	2	192167629	2.02E-02	0.98	6880	7.99E-06	TRUE	TRUE	482,730	19.97
Phosphatidylcholine (O-17:0_17:1) levels	Parkinson's disease	rs7819125	T	C	T	C	8.16E-02	2.14E-02	3.44E-01	6.20E-01	FALSE	FALSE	8	22784354	2.50E-02	4.08E-01	TRUE	8	22926841	1.81E-02	0.99	6880	6.93E-06	TRUE	TRUE	482,730	20.24
Phosphatidylcholine (O-17:0_17:1) levels	Parkinson's disease	rs79648513	G	A	G	A	9.68E-02	5.37E-02	2.29E-01	7.96E-01	FALSE	FALSE	7	18351225	2.89E-02	3.16E-02	TRUE	7	18311602	2.06E-02	0.95	6880	2.69E-06	TRUE	TRUE	482,730	22.06
Phosphatidylcholine (O-17:0_17:1) levels	Parkinson's disease	rs9516264	T	C	T	C	-8.07E-02	-3.03E-02	3.30E-01	6.97E-01	FALSE	FALSE	13	94298778	2.42E-02	6.79E-01	TRUE	13	93646525	1.81E-02	1.00	6880	8.59E-06	TRUE	TRUE	482,730	19.83
Phosphatidylcholine (O-18:0_14:0) levels	Parkinson's disease	rs10516113	G	A	G	A	-8.93E-02	1.79E-02	7.33E-01	3.20E-01	FALSE	FALSE	5	173663189	2.39E-02	3.42E-01	TRUE	5	174236186	2.01E-02	1.00	6462	8.99E-06	TRUE	TRUE	482,730	19.74
Phosphatidylcholine (O-18:0_14:0) levels	Parkinson's disease	rs111316958	T	C	T	C	-3.03E-01	-7.62E-02	1.65E-02	9.75E-01	FALSE	FALSE	15	76780271	6.30E-02	6.45E-01	TRUE	15	76487930	6.81E-02	1.00	6462	8.95E-06	TRUE	TRUE	482,730	19.75
Phosphatidylcholine (O-18:0_14:0) levels	Parkinson's disease	rs116304326	T	C	T	C	3.08E-01	-3.44E-02	1.73E-02	9.83E-01	FALSE	FALSE	4	159729227	9.68E-02	1.41E-01	TRUE	4	158808075	6.89E-02	0.95	6462	7.93E-06	TRUE	TRUE	482,730	19.99
Phosphatidylcholine (O-18:0_14:0) levels	Parkinson's disease	rs117246644	T	C	T	C	-4.42E-01	-7.87E-02	9.98E-03	9.78E-01	FALSE	FALSE	10	60163779	7.66E-02	5.16E-01	TRUE	10	58404019	9.01E-02	0.97	6462	9.60E-07	TRUE	TRUE	482,730	24.05
Phosphatidylcholine (O-18:0_14:0) levels	Parkinson's disease	rs146726414	T	G	T	G	3.12E-01	-3.78E-02	2.00E-02	9.89E-01	FALSE	FALSE	16	78843010	1.20E-01	1.23E-01	TRUE	16	78809113	6.32E-02	0.97	6462	8.16E-07	TRUE	TRUE	482,730	24.37
Phosphatidylcholine (O-18:0_14:0) levels	Parkinson's disease	rs60635506	C	A	C	A	-3.21E-01	1.33E-01	1.69E-02	9.61E-01	FALSE	FALSE	4	122656648	5.98E-02	1.28E-02	TRUE	4	121735493	7.16E-02	0.94	6462	7.54E-06	TRUE	TRUE	482,730	20.08
Phosphatidylcholine (O-18:0_14:0) levels	Parkinson's disease	rs72637380	T	C	T	C	-1.69E-01	5.80E-03	6.65E-02	9.46E-01	FALSE	FALSE	17	16829138	5.34E-02	3.91E-02	TRUE	17	16925824	3.54E-02	0.99	6462	1.84E-06	TRUE	TRUE	482,730	22.80
Phosphatidylcholine (O-18:0_14:0) levels	Parkinson's disease	rs72835302	A	C	A	C	1.42E-01	1.30E-03	1.02E-01	8.90E-01	FALSE	FALSE	10	87928706	4.79E-02	9.44E-03	TRUE	10	86168949	3.04E-02	0.92	6462	3.09E-06	TRUE	TRUE	482,730	21.80
Phosphatidylcholine (O-18:0_14:0) levels	Parkinson's disease	rs73975572	G	A	G	A	-2.00E-01	1.17E-01	4.16E-02	9.49E-01	FALSE	FALSE	17	755144	5.97E-02	2.55E-02	TRUE	17	851904	4.48E-02	0.95	6462	7.98E-06	TRUE	TRUE	482,730	19.97
Phosphatidylcholine (O-18:0_14:0) levels	Parkinson's disease	rs7566488	G	A	G	A	-1.18E-01	-1.71E-02	1.40E-01	7.92E-01	FALSE	FALSE	2	11941989	2.99E-02	2.45E-01	TRUE	2	11801863	2.65E-02	0.93	6462	7.91E-06	TRUE	TRUE	482,730	19.99
Phosphatidylcholine (O-18:0_14:0) levels	Parkinson's disease	rs77444850	C	A	C	A	-2.71E-01	1.88E-02	2.45E-02	9.65E-01	FALSE	FALSE	3	127695041	6.65E-02	1.10E-01	TRUE	3	127976198	5.87E-02	0.94	6462	3.83E-06	TRUE	TRUE	482,730	21.38
Phosphatidylcholine (O-18:0_14:0) levels	Parkinson's disease	rs79139024	C	G	C	G	-4.87E-01	1.23E-01	7.65E-03	6.60E-03	TRUE	FALSE	14	96407446	2.50E-01	2.05E-01	TRUE	14	95941109	1.07E-01	0.90	6462	4.96E-06	TRUE	TRUE	482,730	20.89
Phosphatidylcholine (O-18:0_14:0) levels	Parkinson's disease	rs9365258	C	G	C	G	-1.20E-01	-3.80E-03	8.37E-01	8.64E-01	TRUE	FALSE	6	161608422	3.25E-02	4.21E-02	TRUE	6	161187390	2.39E-02	0.99	6462	5.76E-07	TRUE	TRUE	482,730	25.04
Phosphatidylcholine (O-18:0_16:1) levels	Parkinson's disease	rs10243115	T	C	T	C	8.75E-02	1.84E-02	3.92E-01	6.37E-01	FALSE	FALSE	7	49837913	1.89E-02	4.81E-01	TRUE	7	49798317	1.87E-02	1.00	5959	3.01E-06	TRUE	TRUE	482,730	21.85
Phosphatidylcholine (O-18:0_16:1) levels	Parkinson's disease	rs10859795	G	A	G	A	-8.40E-02	-1.86E-02	4.94E-01	4.25E-01	FALSE	FALSE	12	95317957	1.92E-02	4.77E-01	TRUE	12	94924181	1.85E-02	1.00	5959	5.50E-06	TRUE	TRUE	482,730	20.69
Phosphatidylcholine (O-18:0_16:1) levels	Parkinson's disease	rs11009204	A	G	A	G	1.43E-01	-6.60E-03	9.84E-02	8.83E-01	FALSE	FALSE	10	33350092	3.38E-02	7.36E-02	TRUE	10	33061164	3.13E-02	0.99	5959	4.56E-06	TRUE	TRUE	482,730	21.05
Phosphatidylcholine (O-18:0_16:1) levels	Parkinson's disease	rs12575589	T	C	T	C	1.12E-01	-2.47E-02	2.05E-01	8.72E-01	FALSE	FALSE	11	95750175	3.38E-02	3.33E-01	TRUE	11	96017012	2.30E-02	0.99	5959	1.17E-06	TRUE	TRUE	482,730	23.67
Phosphatidylcholine (O-18:0_16:1) levels	Parkinson's disease	rs1257588	G	A	G	A	-8.38E-02	1.50E-02	5.38E-01	4.04E-01	FALSE	FALSE	14	99528242	2.29E-02	2.90E-01	TRUE	14	99061905	1.86E-02	0.98	5959	6.86E-06	TRUE	TRUE	482,730	20.26
Phosphatidylcholine (O-18:0_16:1) levels	Parkinson's disease	rs12903358	A	G	A	G	-1.17E-01	-1.77E-02	1.46E-01	9.08E-01	FALSE	FALSE	15	97941456	3.87E-02	1.88E-01	TRUE	15	97398226	2.64E-02	0.98	5959	9.03E-06	TRUE	TRUE	482,730	19.74
Phosphatidylcholine (O-18:0_16:1) levels	Parkinson's disease	rs149012657	C	T	C	T	-1.88E-01	5.94E-02	6.48E-02	9.74E-01	FALSE	FALSE	2	230239719	1.38E-01	1.76E-01	TRUE	2	229375003	3.70E-02	0.97	5959	3.82E-07	TRUE	TRUE	482,730	25.84
Phosphatidylcholine (O-18:0_16:1) levels	Parkinson's disease	rs2911458	T	C	T	C	-8.96E-02	-3.49E-02	3.89E-01	6.10E-01	FALSE	FALSE	16	88845009	1.99E-02	3.97E-02	TRUE	16	88778601	1.86E-02	1.00	5959	1.54E-06	TRUE	TRUE	482,730	23.14
Phosphatidylcholine (O-18:0_16:1) levels	Parkinson's disease	rs4547920	A	G	A	G	1.26E-01	5.63E-02	1.66E-01	8.31E-01	FALSE	FALSE	5	124073767	3.03E-02	3.16E-02	TRUE	5	124738074	2.50E-02	0.98	5959	4.26E-07	TRUE	TRUE	482,730	25.63
Phosphatidylcholine (O-18:0_16:1) levels	Parkinson's disease	rs55969585	T	G	T	G	2.09E-01	-3.70E-02	4.59E-02	9.24E-01	FALSE	FALSE	1	118182039	4.26E-02	4.14E-01	TRUE	1	117639417	4.56E-02	0.96	5959	4.75E-06	TRUE	TRUE	482,730	20.98
Phosphatidylcholine (O-18:0_16:1) levels	Parkinson's disease	rs603424	A	G	A	G	-1.81E-01	6.90E-03	1.21E-01	8.02E-01	FALSE	FALSE	10	102075479	2.49E-02	1.07E-01	TRUE	10	100315722	2.91E-02	1.00	5959	5.21E-10	TRUE	TRUE	482,730	38.71
Phosphatidylcholine (O-18:0_16:1) levels	Parkinson's disease	rs639060	G	A	G	A	2.19E-01	9.17E-02	3.70E-02	9.85E-01	FALSE	FALSE	10	102108523	1.83E-01	2.10E-01	TRUE	10	100348766	4.88E-02	0.97	5959	7.25E-06	TRUE	TRUE	482,730	20.16
Phosphatidylcholine (O-18:0_16:1) levels	Parkinson's disease	rs74644051	A	G	A	G	-3.44E-01	1.30E-02	1.69E-02	9.66E-01	FALSE	FALSE	13	28216161	6.48E-02	7.50E-02	TRUE	13	27642024	7.23E-02	0.97	5959	2.04E-06	TRUE	TRUE	482,730	22.61
Phosphatidylcholine (O-18:0_20:4) levels	Parkinson's disease	rs11057853	C	T	C	T	-7.75E-02	-1.98E-02	4.54E-01	5.57E-01	FALSE	FALSE	12	125329313	2.29E-02	4.13E-01	TRUE	12	124844767	1.71E-02	0.99	6956	6.11E-06	TRUE	TRUE	482,730	20.48
Phosphatidylcholine (O-18:0_20:4) levels	Parkinson's disease	rs117870830	G	A	G	A	-3.06E-01	4.16E-02	1.73E-02	9.78E-01	FALSE	FALSE	11	120460797	8.60E-02	2.02E-01	TRUE	11	120590088	6.72E-02	0.89	6956	5.47E-06	TRUE	TRUE	482,730	20.69
Phosphatidylcholine (O-18:0_20:4) levels	Parkinson's disease	rs11954170	G	A	G	A	8.27E-02	2.48E-02	3.24E-01	6.66E-01	FALSE	FALSE	5	106118791	2.37E-02	5.32E-01	TRUE	5	106783090	1.83E-02	1.00	6956	6.07E-06	TRUE	TRUE	482,730	20.50
Phosphatidylcholine (O-18:0_20:4) levels	Parkinson's disease	rs138202451	A	G	A	G	-5.76E-01	-3.83E-02	6.16E-03	9.85E-01	FALSE	FALSE	2	67218621	9.57E-02	1.62E-01	TRUE	2	66991489	1.11E-01	0.93	6956	2.32E-07	TRUE	TRUE	482,730	26.79
Phosphatidylcholine (O-18:0_20:4) levels	Parkinson's disease	rs143233558	A	G	A	G	1.49E-01	5.62E-02	1.02E-01	9.02E-01	FALSE	FALSE	7	63790621	3.47E-02	9.77E-01	TRUE	7	64330243	2.84E-02	0.98	6956	1.55E-07	TRUE	TRUE	482,730	27.59

Phosphatidylcholine (O-18:0_20:4) levels	Parkinson's disease	rs145755646	G	C	G	C	1.60E-01	2.59E-02	6.46E-02	4.82E-02	TRUE	FALSE	17	57418652	5.40E-02	1.99E-01	TRUE	17	59341291	3.47E-02	0.99	6956	4.21E-06	TRUE	TRUE	482,730	21.20
Phosphatidylcholine (O-18:0_20:4) levels	Parkinson's disease	rs148462060	G	A	G	A	-2.61E-01	-1.50E-01	2.43E-02	9.89E-01	FALSE	FALSE	2	130927386	1.87E-01	3.74E-01	TRUE	2	130169813	5.60E-02	0.97	6956	3.30E-06	TRUE	TRUE	482,730	21.67
Phosphatidylcholine (O-18:0_20:4) levels	Parkinson's disease	rs148840717	T	G	T	G	-1.98E-01	-7.62E-02	3.68E-02	9.73E-01	FALSE	FALSE	3	16077825	6.98E-02	5.61E-01	TRUE	3	16036318	4.46E-02	1.00	6956	8.98E-06	TRUE	TRUE	482,730	19.75
Phosphatidylcholine (O-18:0_20:4) levels	Parkinson's disease	rs174536	C	A	C	A	-3.08E-01	-2.00E-03	4.08E-01	6.68E-01	FALSE	FALSE	11	61551927	1.78E-02	4.16E-02	TRUE	11	61784455	1.69E-02	1.00	6956	2.28E-72	TRUE	TRUE	482,730	331.28
Phosphatidylcholine (O-18:0_20:4) levels	Parkinson's disease	rs1800588	T	C	T	C	1.03E-01	-3.30E-03	2.58E-01	7.75E-01	FALSE	FALSE	15	58723675	2.12E-02	5.73E-02	TRUE	15	58431476	1.97E-02	0.99	6956	1.56E-07	TRUE	TRUE	482,730	27.57
Phosphatidylcholine (O-18:0_20:4) levels	Parkinson's disease	rs34382810	C	A	C	A	8.58E-02	-1.00E-04	3.65E-01	5.78E-01	FALSE	FALSE	12	2340798	1.87E-02	9.57E-04	TRUE	12	2231632	1.76E-02	0.99	6956	1.12E-06	TRUE	TRUE	482,730	23.76
Phosphatidylcholine (O-18:0_20:4) levels	Parkinson's disease	rs34737685	C	A	C	A	-7.75E-02	-2.05E-02	5.38E-01	5.17E-01	FALSE	FALSE	1	164926232	1.87E-02	5.62E-01	TRUE	1	164956995	1.71E-02	1.00	6956	5.63E-06	TRUE	TRUE	482,730	20.64
Phosphatidylcholine (O-18:0_20:4) levels	Parkinson's disease	rs3741252	T	C	T	C	1.50E-01	-2.38E-02	1.34E-01	9.32E-01	FALSE	FALSE	11	61511498	4.03E-02	2.56E-01	TRUE	11	61744026	2.50E-02	0.99	6956	1.99E-09	TRUE	TRUE	482,730	36.07
Phosphatidylcholine (O-18:0_20:4) levels	Parkinson's disease	rs4782783	C	T	C	T	1.25E-01	-4.05E-02	8.88E-01	1.06E-01	FALSE	FALSE	16	83415532	3.71E-02	5.59E-01	TRUE	16	83381927	2.70E-02	0.99	6956	3.64E-06	TRUE	TRUE	482,730	21.48
Phosphatidylcholine (O-18:0_20:4) levels	Parkinson's disease	rs6992951	A	C	A	C	-8.22E-02	-1.73E-02	3.90E-01	5.90E-01	FALSE	FALSE	8	18029977	2.26E-02	3.53E-01	TRUE	8	18172468	1.73E-02	0.99	6956	2.05E-06	TRUE	TRUE	482,730	22.59
Phosphatidylcholine (O-18:0_20:4) levels	Parkinson's disease	rs73074943	T	C	T	C	2.51E-01	4.23E-02	2.44E-02	9.80E-01	FALSE	FALSE	12	22026778	8.57E-02	2.07E-01	TRUE	12	21873844	5.52E-02	0.98	6956	5.54E-06	TRUE	TRUE	482,730	20.67
Phosphatidylcholine (O-18:0_20:4) levels	Parkinson's disease	rs78711160	A	G	A	G	1.82E-01	2.50E-02	4.98E-02	9.79E-01	FALSE	FALSE	9	82358061	1.37E-01	6.79E-02	TRUE	9	79743146	3.97E-02	0.96	6956	4.51E-06	TRUE	TRUE	482,730	21.07
Phosphatidylcholine (O-18:0_20:4) levels	Parkinson's disease	rs79856406	G	T	G	T	-1.25E-01	-6.56E-02	1.02E-01	9.22E-01	FALSE	FALSE	2	164438811	4.23E-02	9.18E-01	TRUE	2	163582301	2.84E-02	0.96	6956	9.96E-06	TRUE	TRUE	482,730	19.55
Phosphatidylcholine (O-18:0_20:4) levels	Parkinson's disease	rs9591400	T	A	T	A	1.20E-01	4.63E-02	1.18E-01	1.15E-01	TRUE	FALSE	13	51835149	3.02E-02	9.01E-01	TRUE	13	51261013	2.65E-02	0.97	6956	5.67E-06	TRUE	TRUE	482,730	20.63
Phosphatidylcholine (O-18:1_16:0) levels	Parkinson's disease	rs1049302	C	T	C	T	8.78E-02	-3.25E-02	7.42E-01	2.16E-01	FALSE	FALSE	13	27998769	2.25E-02	8.30E-01	TRUE	13	27424632	1.94E-02	0.99	6956	5.84E-06	TRUE	TRUE	482,730	20.57
Phosphatidylcholine (O-18:1_16:0) levels	Parkinson's disease	rs113746682	T	C	T	C	2.92E-01	-5.36E-02	2.30E-02	9.68E-01	FALSE	FALSE	16	85377751	7.09E-02	3.48E-01	TRUE	16	85344145	5.92E-02	0.91	6956	8.63E-07	TRUE	TRUE	482,730	24.25
Phosphatidylcholine (O-18:1_16:0) levels	Parkinson's disease	rs118138240	T	C	T	C	-2.30E-01	4.23E-02	3.34E-02	9.60E-01	FALSE	FALSE	16	810921	5.29E-02	3.72E-01	TRUE	16	760921	4.90E-02	0.91	6956	2.89E-06	TRUE	TRUE	482,730	21.92
Phosphatidylcholine (O-18:1_16:0) levels	Parkinson's disease	rs13030345	T	G	T	G	-1.08E-01	4.39E-02	1.86E-01	8.04E-01	FALSE	FALSE	2	28003174	2.26E-02	2.60E-02	TRUE	2	27780307	2.17E-02	0.99	6956	6.73E-07	TRUE	TRUE	482,730	24.73
Phosphatidylcholine (O-18:1_16:0) levels	Parkinson's disease	rs138542536	C	A	C	A	-3.87E-01	-1.28E-01	1.33E-02	9.88E-01	FALSE	FALSE	4	25797744	1.68E-01	3.48E-01	TRUE	4	25796122	7.94E-02	0.86	6956	1.14E-06	TRUE	TRUE	482,730	23.73
Phosphatidylcholine (O-18:1_16:0) levels	Parkinson's disease	rs142774676	A	G	A	G	2.96E-01	-1.16E-02	1.65E-02	9.69E-01	FALSE	FALSE	2	201333937	5.89E-02	7.34E-02	TRUE	2	200469214	6.69E-02	0.98	6956	9.42E-06	TRUE	TRUE	482,730	19.65
Phosphatidylcholine (O-18:1_16:0) levels	Parkinson's disease	rs1503311	T	C	T	C	9.83E-02	5.27E-02	1.97E-01	8.60E-01	FALSE	FALSE	11	13787701	3.24E-02	9.85E-01	TRUE	11	13766154	2.12E-02	1.00	6956	3.43E-06	TRUE	TRUE	482,730	21.59
Phosphatidylcholine (O-18:1_16:0) levels	Parkinson's disease	rs17231506	T	C	T	C	1.35E-01	1.36E-02	2.78E-01	6.87E-01	FALSE	FALSE	16	56994528	1.81E-02	3.45E-01	TRUE	16	56960616	1.88E-02	1.00	6956	7.74E-13	TRUE	TRUE	482,730	51.54
Phosphatidylcholine (O-18:1_16:0) levels	Parkinson's disease	rs1800588	T	C	T	C	1.23E-01	-3.30E-03	2.58E-01	7.75E-01	FALSE	FALSE	15	58723675	2.12E-02	5.73E-02	TRUE	15	58431476	1.96E-02	0.99	6956	4.58E-10	TRUE	TRUE	482,730	38.96
Phosphatidylcholine (O-18:1_16:0) levels	Parkinson's disease	rs2043082	A	G	A	G	9.66E-02	2.15E-02	4.11E-01	6.56E-01	FALSE	FALSE	15	58674308	1.79E-02	6.37E-01	TRUE	15	58382109	1.71E-02	1.00	6956	1.61E-08	TRUE	TRUE	482,730	31.98
Phosphatidylcholine (O-18:1_16:0) levels	Parkinson's disease	rs246168	A	C	A	C	1.58E-01	2.50E-02	6.73E-02	8.95E-01	FALSE	FALSE	16	14370620	3.37E-02	3.38E-01	TRUE	16	14276763	3.43E-02	0.99	6956	4.05E-06	TRUE	TRUE	482,730	21.27
Phosphatidylcholine (O-18:1_16:0) levels	Parkinson's disease	rs258736	T	C	T	C	-9.77E-02	-3.47E-02	8.07E-01	1.50E-01	FALSE	FALSE	5	115130906	3.17E-02	5.64E-01	TRUE	5	115795209	2.16E-02	0.99	6956	6.54E-06	TRUE	TRUE	482,730	20.35
Phosphatidylcholine (O-18:1_16:0) levels	Parkinson's disease	rs34861396	A	G	A	G	9.34E-02	2.29E-02	2.99E-01	6.38E-01	FALSE	FALSE	8	31498467	2.76E-02	3.92E-01	TRUE	8	31640951	1.85E-02	0.99	6956	4.51E-07	TRUE	TRUE	482,730	25.51
Phosphatidylcholine (O-18:1_16:0) levels	Parkinson's disease	rs56868865	A	G	A	G	1.25E-01	4.02E-02	1.01E-01	8.86E-01	FALSE	FALSE	5	81963141	3.65E-02	5.67E-01	TRUE	5	82667322	2.84E-02	0.96	6956	9.97E-06	TRUE	TRUE	482,730	19.54
Phosphatidylcholine (O-18:1_16:0) levels	Parkinson's disease	rs57493997	C	T	C	T	-1.44E-01	-1.60E-03	8.14E-02	9.06E-01	FALSE	FALSE	6	34443523	3.26E-02	1.69E-02	TRUE	6	34475746	3.10E-02	0.98	6956	3.57E-06	TRUE	TRUE	482,730	21.52
Phosphatidylcholine (O-18:1_16:0) levels	Parkinson's disease	rs60331765	G	A	G	A	-1.03E-01	-2.92E-02	1.80E-01	8.38E-01	FALSE	FALSE	2	182447581	2.71E-02	5.52E-01	TRUE	2	181582854	2.19E-02	0.99	6956	2.80E-06	TRUE	TRUE	482,730	21.99
Phosphatidylcholine (O-18:1_16:0) levels	Parkinson's disease	rs606587	G	A	G	A	-7.86E-02	1.27E-02	3.74E-01	6.75E-01	FALSE	FALSE	1	230316189	2.04E-02	2.72E-01	TRUE	1	230180443	1.75E-02	1.00	6956	7.17E-06	TRUE	TRUE	482,730	20.18
Phosphatidylcholine (O-18:1_16:0) levels	Parkinson's disease	rs6568686	C	T	C	T	-1.03E-01	2.86E-02	8.39E-01	2.39E-01	FALSE	FALSE	6	111872482	1.99E-02	8.25E-01	TRUE	6	111551279	2.30E-02	0.99	6956	7.96E-06	TRUE	TRUE	482,730	19.98
Phosphatidylcholine (O-18:1_16:0) levels	Parkinson's disease	rs6908815	C	T	C	T	8.68E-02	1.85E-02	2.59E-01	7.35E-01	FALSE	FALSE	6	167744409	2.07E-02	4.31E-01	TRUE	6	167330921	1.95E-02	0.99	6956	8.67E-06	TRUE	TRUE	482,730	19.81
Phosphatidylcholine (O-18:1_16:0) levels	Parkinson's disease	rs72683831	C	T	C	T	1.59E-01	-1.04E-02	7.10E-02	9.22E-01	FALSE	FALSE	4	125716427	4.22E-02	9.39E-02	TRUE	4	124795272	3.35E-02	0.99	6956	2.23E-06	TRUE	TRUE	482,730	22.42
Phosphatidylcholine (O-18:1_16:0) levels	Parkinson's disease	rs7327319	A	C	A	C	1.17E-01	-3.74E-02	1.50E-01	8.04E-01	FALSE	FALSE	13	40928617	3.05E-02	6.57E-01	TRUE	13	40354480	2.36E-02	1.00	6956	7.43E-07	TRUE	TRUE	482,730	24.54
Phosphatidylcholine (O-18:1_16:0) levels	Parkinson's disease	rs7330980	A	G	A	G	1.48E-01	9.50E-03	7.59E-02	8.65E-01	FALSE	FALSE	13	61398804	3.24E-02	1.14E-01	TRUE	13	60824670	3.25E-02	0.99	6956	4.90E-06	TRUE	TRUE	482,730	20.91
Phosphatidylcholine (O-18:1_16:0) levels	Parkinson's disease	rs7450458	A	G	A	G	-1.14E-01	1.29E-02	1.44E-01	9.34E-01	FALSE	FALSE	6	31418616	3.87E-02	1.32E-01	TRUE	6	31450839	2.40E-02	1.00	6956	1.99E-06	TRUE	TRUE	482,730	22.64
Phosphatidylcholine (O-18:1_16:0) levels	Parkinson's disease	rs7677028	G	C	G	C	3.82E-01	2.67E-02	1.43E-02	2.61E-02	TRUE	FALSE	4	137943535	7.37E-02	1.44E-01	TRUE	4	137022381	7.22E-02	0.97	6956	1.30E-07	TRUE	TRUE	482,730	27.92
Phosphatidylcholine (O-18:1_16:0) levels	Parkinson's disease	rs78194510	C	T	C	T	1.35E-01	-1.81E-02	1.17E-01	9.08E-01	FALSE	FALSE	12	125338336	3.99E-02	1.86E-01	TRUE	12	124853790	2.64E-02	0.97	6956	3.32E-07	TRUE	TRUE	482,730	26.10
Phosphatidylcholine (O-18:1_16:0) levels	Parkinson's disease	rs9682490	C	T	C	T	-8.62E-02	-2.14E-02	4.35E-01	5.64E-01	FALSE	FALSE	3	4990776	2.26E-02	4.66E-01	TRUE	3	4949091	1.73E-02	0.98	6956	6.24E-07	TRUE	TRUE	482,730	24.88
Phosphatidylcholine (O-18:1_18:2) levels	Parkinson's disease	rs11022354	A	G	A	G	7.67E-02	-1.70E-02	5.35E-01	4.33E-01	FALSE	FALSE	11	12447207	2.24E-02	3.48E-01	TRUE	11	12425660	1.73E-02	1.00	6718	9.48E-06	TRUE	TRUE	482,730	19.64
Phosphatidylcholine (O-18:1_18:2) levels	Parkinson's disease	rs111306887	A	G	A	G	-2.36E-01	2.50E-02	2.83E-02	9.77E-01	FALSE	FALSE	1	183903039	7.49E-02	1.31E-01	TRUE	1	183933905	5.19E-02	0.99	6718	5.62E-06	TRUE	TRUE	482,730	20.65
Phosphatidylcholine (O-18:1_18:2) levels	Parkinson's disease	rs115600841	G	T	G	T	3.03E-01	-1.51E-01	1.75E-02	9.47E-01	FALSE	FALSE	4	174576090	5.14E-02	1.65E-03	TRUE	4	173654939	6.83E-02	0.93	6718	9.46E-06	TRUE	TRUE	482,730	19.65
Phosphatidylcholine (O-18:1_18:2) levels	Parkinson's disease	rs117145701	A	C	A	C	-4.39E-01	-7.58E-02	8.32E-03	9.80E-01	FALSE	FALSE	7	108794309	8.91E-02	4.04E-01	TRUE	7	109154252	9.83E-02	0.89	6718	8.00E-06	TRUE	TRUE	482,730	19.97
Phosphatidylcholine (O-18:1_18:2) levels	Parkinson's disease	rs11918522	A	G	A	G	1.27E-01	-3.09E-02	1.59E-01	8.27E-01	FALSE	FALSE	3	112535057	2.25E-02	7.70E-01	TRUE	3	112816210	2.36E-02	1.00	6718	7.40E-08	TRUE	TRUE	482,730	29.02
Phosphatidylcholine (O-18:1_18:2) levels	Parkinson's disease	rs147996419	T	G	T	G	-3.22E-01	-1.76E-01	1.51E-02	9.88E-01	FALSE	FALSE	15	45695448	1.84E-01	4.71E-01	TRUE	15	45403250	7.28E-02	0.94	6718	1.00E-05	TRUE	TRUE	482,730	19.54
Phosphatidylcholine (O-18:1_18:2) levels	Parkinson's disease	rs17086339	C	G	C	G	-1.14E-01	2.51E-02	1.30E-01	7.06E-02	TRUE	FALSE	5	95935275	4.57E-02	2.35E-01	TRUE	5	96599571	2.56E-02	0.99	6718	8.41E-06	TRUE	TRUE	482,730	19.87
Phosphatidylcholine (O-18:1_18:2) levels	Parkinson's disease	rs17231506	T	C	T	C	9.54E-02	1.36E-02	2.78E-01	6.87E-01	FALSE	FALSE	16	56994528	1.81E-02	3.45E-01	TRUE	16	56960616	1.91E-02	1.00	6718	6.14E-07	TRUE	TRUE	482,730	24.91
Phosphatidylcholine (O-18:1_18:2) levels	Parkinson's disease	rs17875991	T	C	T	C	-2.02E-01	1.59E-01	4.04E-02	9.82E-01	FALSE	FALSE	1	11809415	1.44E-01	5.69E-01	TRUE	1	11749358	4.57E-02	0.93	6718	9.75E-06	TRUE	TRUE	482,730	19.59
Phosphatidylcholine (O-18:1_18:2) levels	Parkinson's disease	rs1800588	T	C	T	C	9.02E-02	-3.30E-03	2.58E-01	7.75E-01	FALSE	FALSE	15	58723675	2.12E-02	5.73E-02	TRUE	15	58431476	2.00E-02	0.99	6718	6.89E-06	TRUE	TRUE	482,730	20.25
Phosphatidylcholine (O-18:1_18:2) levels	Parkinson's disease	rs2537611	T	C	T	C	8.75E-02	2.01E-02	3.72E-01	6.72E-01	FALSE	FALSE	7	17732799	2.36E-02	4.04E-01	TRUE	7	17693175	1.79E-02	1.00	6718	9.77E-07	TRUE	TRUE	482,730	24.02
Phosphatidylcholine (O-18:1_18:2) levels	Parkinson's disease	rs2985394	G	A	G	A	8.67E-02	-5.96E-02	6.69E-01	3.32E-01	FALSE	FALSE	11	34908692	2.19E-02	3.25E-03	TRUE	11	34887145	1.84E-02	1.00	6718	2.63E-06	TRUE	TRUE	482,730	22.10
Phosphatidylcholine (O-18:1_18:2) levels	Parkinson's disease	rs4655970	C	T	C	T	8.50E-02	2.54E-02	6.49E-01	3.67E-01	FALSE	FALSE	1	88036946	3.13E-02	3.79E-01	TRUE	1	87571263	1.89E-02	0.90	6718	6.73E-06	TRUE	TRUE	482,730	20.30
Phosphatidylcholine (O-18:1_18:2) levels	Parkinson's disease	rs606587	G	A	G	A	-9.07E-02	1.27E-02	3.74E-01	6.75E-01	FALSE	FALSE	1	230316189	2.04E-02	2.72E-01	TRUE	1	230180443	1.78E-02	1.00	6718	3.41E-07	TRUE	TRUE	482,730	26.05
Phosphatidylcholine (O-18:1_18:2) levels	Parkinson's disease	rs61833681	A	G	A	G	-9.05E-02	-3.30E-02	2.43E-01	6.92E-01	FALSE	FALSE	1	240758482	2.49E-02	7.31E-01	TRUE	1	240595182	2.02E-02	0.98	6718	7.76E-06	TRUE	TRUE	482,730	20.03
Phosphatidylcholine (O-18:1_18:2) levels	Parkinson's disease	rs62294908	G	A	G	A	1.34E-01	-8.90E-03	1.10E-01	9.03E-01	FALSE	FALSE	3	182192270	4.20E-02	7.97E-02	TRUE	3	182474482	2.95E-02	0.87	6718	5.72E-06	TRUE	TRUE	482,730	20.61
Phosphatidylcholine (O-18:1_18:2) levels	Parkinson's disease	rs62331262	C	G	C	G	1.07E-01	-1.42E-02	1.73E-01	2.32E-01	TRUE	FALSE	4	105520461	2.69E-02	2.24E-01	TRUE	4	104599304	2.30E-02	0.98	6718	3.11E-06	TRUE	TRUE	482,730	21.78
Phosphatidylcholine (O-18:1_18:2) levels	Parkinson's disease	rs72709643	C	T	C	T	-2.13E-01	-1.55E-02	3.62E-02	9.54E-01	FALSE	FALSE	1	180973195	4.81E-02	1.26E-01	TRUE	1	181004059	4.64E-02	0.97	6718	4.74E-06	TRUE	TRUE	482,730	20.97
Phosphatidylcholine (O-18:1_18:2) levels	Parkinson's disease	rs74864573	A	G	A	G	-2.23E-01	-9.10E-02	3.32E-02	9.79E-01	FALSE	FALSE	7	31360338	8.04E-02	5.89E-01	TRUE	7	31320724	4.91E-02	0.96	6718	5.58E-06	TRUE	TRUE	482,730	20.66
Phosphatidylcholine (O-18:1_18:2) levels	Parkinson's disease	rs79040967	A	G	A	G	2.01E-01	1.26E-01	4.44E-02	9.75E-01	FALSE	FALSE	11	96996581	8.17E-02	9.09E-01	TRUE	11	97125581	4.29E-02	0.95	6718	2.75E-06	TRUE	TRUE	482,730	22.02
Phosphatidylcholine (O-18:1_20:3) levels	Parkinson's disease	rs11236557	T	C	T	C	9.20E-02	-2.12E-02	5.82E-01	3.68E-01	FALSE	FALSE	11	75552129	2.41E-02	4.20E-01	TRUE	11	75841084	1.79E-02	0.95	6836	2.63E-07	TRUE	TRUE	482,730	26.56
Phosphatidylcholine (O-18:1_20:3) levels	Parkinson's disease	rs12239821	A	G	A	G	-1.30E-01	-3.72E-02	1.08E-01	8.83E-01	FALSE	FALSE	1	245601513	3.54E-02	5.32E-01	TRUE	1	245438211	2.83E-02	0.95	6836	4.62E-06	TRUE	TRUE	482,730	21.02
Phosphatidylcholine (O-18:1_20:3) levels	Parkinson's disease	rs12444786	G	A	G	A	1.08E-01	5.28E-02	8.21E-01	1.74E-01	FALSE	FALSE	16	82676746	3.64E-02	8.33E-01	TRUE	16	82643141	2.31E-02	0.92	6836	3.29E-06	TRUE	TRUE	482,730	21.68
Phosphatidylcholine (O-18:1_20:3) levels	Parkinson's disease	rs12659734	C	T	C	T	1.07E-01	-2.22E-02	1.55E-01	8.71E-01	FALSE	FALSE	5	9303972	2.88E-02	3.57E-01	TRUE	5	9303860	2.35E-02	1.00	6836	5.21E-06	TRUE	TRUE	482,730	20.79
Phosphatidylcholine (O-18:1_20:3) levels	Parkinson's disease	rs139594872	A	G	A	G	-2.06E-01	1.30E-01	3.47E-02	9.84E-01	FALSE	FALSE	8	141943161	1.15E-01	5.85E-01	TRUE	8	140933062	4.64E-02	0.98	6836	9.50E-06	TRUE	TRUE	482,730	19.64
Phosphatidylcholine (O-18:1_20:3) levels	Parkinson's disease	rs149022879	A	G	A	G	-2.28E-01	-1.87E-02	2.98E-02	9.87E-01	FALSE	FALSE	1	212230899	1.45E-01	4.69E-02	TRUE	1	212057557	5.06E-02	0.95	6836	6.44E-06	TRUE	TRUE	482,730	20.38
Phosphatidylcholine (O-18:1_20:3) levels	Parkinson's disease	rs1540881	T	C	T	C	8.27E-02	-1.02E-02	5.40E-01	4.21E-01	FALSE	FALSE	7	18122575	2.38E-02	1.75E-01	TRUE	7	18082952	1.79E-02	0.93	6836	3.73E-06	TRUE	TRUE	482,730	21.43
Phosphatidylcholine (O-18:1_20:3) levels	Parkinson's disease	rs174548	G	C	G	C	2.59E-01	2.00E-03	3.84E-01	3.00E-01	TRUE	FALSE	11	61571348	1.83E-02	3.90E-02	TRUE	11	61803876	1.74E-02	1.00	6836	2.68E-49	TRUE	TRUE	482,730	221.33
Phosphatidylcholine (O-18:1_20:3) levels	Parkinson's disease	rs188004353	G	C	G	C	-2.30E-01	-1.74E-01	3.16E-02	1.47E-02	TRUE	FALSE	15	68853427	1.44E-01	6.42E-01	TRUE	15	68561088	5.10E-02	0.92	6836	6.36E-06	TRUE	TRUE	482,730	20.41
Phosphatidylcholine (O-18:1_20:3) levels	Parkinson's disease	rs1925669	A	G	A	G	-1.13E-01	8.10E-03	1.28E-01	8.67E-01	FALSE	FALSE	1	20720689	2.61E-02	1.21E-01	TRUE	1	20394196	2.55E-02	1.00	6836	9.76E-06	TRUE	TRUE	482,730	19.58
Phosphatidylcholine (O-18:1_20:3) levels	Parkinson's disease	rs1978125	A	G	A	G	1.03E-01	5.86E-02	1.96E-01	8.80E-01	FALSE	FALSE	11	2457666	3.40E-02	4.24E-02	TRUE	11	2436436	2.33E-02	0.86	6836	9.23E-06	TRUE	TRUE	482,730	19.69
Phosphatidylcholine (O-18:1_20:3) levels	Parkinson's disease	rs2170790	C	T	C	T	6.28E-01	-1.14E-01	5.16E-03	9.90E-01	FALSE	FALSE	3	25460125	1.83E-01	2.74E-01	TRUE	3	25418634	1.30E-01	0.92	6836	1.33E-06	TRUE	TRUE	482,730	23.41
Phosphatidylcholine (O-18:1_20:3) levels	Parkinson's disease	rs62493656	T	C	T	C	-2.14E-01	-8.55E-02	3.59E-02	9.71E-01	FALSE	FALSE	7	158145946	7.08E-02	6.45E-01	TRUE	7	158353254	4.63E-02	0.97	6836	3.89E-06	TRUE	TRUE	482,730	21.35
Phosphatidylcholine (O-18:1_20:3) levels	Parkinson's disease	rs6498540	G	A	G	A	-1.09E-01	1.82E-02	3.34E-01	6.94E-01	FALSE	FALSE	16	15130594	1.84E-02	4.93E-01	TRUE	16	15036737	1.82E-02	0.99	6836	1.93E-09	TRUE	TRUE	482,730	36.14
Phosphatidylcholine (O-18:1_20:3) levels	Parkinson's disease	rs78183891	T	C	T	C	2.23E-01	-7.54E-02	3.01E-02	9.76E-01	FALSE	FALSE	9	73471557	7.35E-02	5.16E-01	TRUE	9	70856641	4.97E-02	0.99	6836	7.43E-06	TRUE	TRUE	482,730	20.11
Phosphatidylcholine (O-18:1_20:3) levels	Parkinson's disease	rs9843297	G	A	G	A	9.98E-02	9.90E-03	1.96E-01	8.16E-01	FALSE	FALSE	3	22491091	2.95E-02	1.32E-01	TRUE	3	22449600	2.18E-02	0.99	6836	4.75E-06	TRUE	TRUE	482,730	20.97
Phosphatidylcholine (O-18:1_20:4) levels	Parkinson's disease	rs11057853	C	T	C	T	-9.40E-02	-1.98E-02	4.54E-01	5.57E-01	FALSE	FALSE	12	125329313	2.29E-02	4.13E-01	TRUE	12	124844767	1.68E-02	0.99	7173	2.45E-08	TRUE	TRUE	482,730	31.16
Phosphatidylcholine (O-18:1_20:4) levels	Parkinson's disease	rs116814948	C	T	C	T	-4.21E-01	-1.26E-01	8.67E-03	9.75E-01	FALSE	FALSE	6	2823016	8.35E-02	8.77E-01	TRUE	6	2822782	9.42E-02	0.90	7173	7.98E-06	TRUE	TRUE	482,730	19.97
Phosphatidylcholine (O-18:1_20:4) levels	Parkinson's disease	rs12760720	G	T	G	T	-7.66E-02	2.42E-02	5.24E-01	5.15E-01	FALSE	FALSE	1	234409343	2.25E-02	5.48E-01	TRUE	1	234273597	1.68E-02	1.00	7173	5.43E-06	TRUE	TRUE	482,730	20.71
Phosphatidylcholine (O-18:1_20:4) levels	Parkinson's disease	rs149490626	G	A	G	A	2.77E-01	-1.04E-01	1.90E-02	9.80E-01	FALSE	FALSE	8	56409275	8.82E-02	6.20E-01	TRUE	8	55496716	6.14E-02	0.96	7173	6.41E-06	TRUE	TRUE	482,730	20.39
Phosphatidylcholine (O-18:1_20:4) levels	Parkinson's disease	rs174536	C	A	C	A	-2.84E-01	-2.00E-03	4.08E-01	6.68E-01	FALSE	FALSE	11	61551927	1.78E-02	4.16E-02	TRUE	11	61784455	1.67E-02	1.00	7173	3.74E-63	TRUE	TRUE	482,730	286.96
Phosphatidylcholine (O-18:1_20:4) levels	Parkinson's disease	rs1800588	T	C	T	C	1.07E-01	-3.30E-03	2.58E-01	7.75E-01	FALSE	FALSE	15	58723675	2.12E-02	5.73E-02	TRUE	15	58431476	1.94E-02	0.99	7173	3.09E-08	TRUE	TRUE	482,730	30.71
Phosphatidylcholine (O-18:1_20:4) levels	Parkinson's disease	rs2332690	G	T	G	T	-9.20E-02	-3.56E-02	7.90E-01	1.71E-01	FALSE	FALSE	3	123767922	2.96E-02	6.42E-01	TRUE	3	124049075	2.05E-02	1.00	7173	7.30E-06	TRUE	TRUE	482,730	20.14
Phosphatidylcholine (O-18:1_20:4) levels	Parkinson's disease	rs2522051	C	T	C	T	-7.57E-02	-1.81E-02	5.76E-01	5.46E-01	FALSE	FALSE	5	131797578	1.69E-02	5.46E-01	TRUE	5	132461886	1.70E-02	1.00	7173	8.47E-06	TRUE	TRUE	482,730	19.86
Phosphatidylcholine (O-18:1_20:4) levels	Parkinson's disease	rs3017103	G	A	G	A	1.01E-01	1.33E-02	8.04E-01	1.78E-01	FALSE	FALSE	11	62174193	2.88E-02	1.90E-01	TRUE	11	62406721	2.12E-02	0.99	7173	1.91E-06	TRUE	TRUE	482,730	22.72
Phosphatidylcholine (O-18:1_20:4) levels	Parkinson's disease	rs34881711	A	G	A	G	9.00E-02	-1.03E-02	2.29E-01	7.90E-01	FALSE	FALSE	5	153845234	2.38E-02	1.77E-01	TRUE	5	154465674	1.98E-02	0.99	7173	5.67E-06	TRUE	TRUE	482,730	20.62
Phosphatidylcholine (O-18:1_20:4) levels	Parkinson's disease	rs4142412	A	G	A	G	9.37E-02	-7.10E-03	7.62E-01	2.49E-01	FALSE	FALSE	6	34418892	2.22E-02	1.25E-01	TRUE	6	34451115	1.95E-02	1.00	7173	1.66E-06	TRUE	TRUE	482,730	23.00
Phosphatidylcholine (O-18:1_20:4) levels	Parkinson's disease	rs4618752	T	G	T	G	7.70E-02	-3.03E-02	5.06E-01	6.14E-01	FALSE	FALSE	9	1294441	2.28E-02	7.35E-01	TRUE	9	1294441	1.66E-02	1.00	7173	3.57E-06	TRUE	TRUE	482,730	21.52
Phosphatidylcholine (O-18:1_20:4) levels	Parkinson's disease	rs4942185	C	T	C	T	-8.13E-02	-5.00E-03	6.87E-01	3.33E-01	FALSE	FALSE	13	43558846	2.02E-02	9.37E-02	TRUE	13	42984710	1.79E-02	1.00	7173	5.59E-06	TRUE	TRUE	482,730	20.65
Phosphatidylcholine (O-18:1_20:4) levels	Parkinson's disease	rs638491	G	A	G	A	1.14E-01	-2.08E-02	8.81E-01	1.73E-01	FALSE	FALSE	9	15290012	2.37E-02	4.22E-01	TRUE	9	15290014	2.56E-02	1.00	7173	8.31E-06	TRUE	TRUE	482,730	19.89
Phosphatidylcholine (O-18:1_20:4) levels	Parkinson's disease	rs6721307	C	T	C	T	7.56E-02	-2.52E-02	4.28E-01	4.83E-01	FALSE	FALSE	2	124406943	2.22E-02	5.90E-01	TRUE	2	123649367	1.70E-02	1.00	7173	8.63E-06	TRUE	TRUE	482,730	19.82
Phosphatidylcholine (O-18:1_20:4) levels	Parkinson's disease	rs77199708	A	G	A	G	1.88E-01	7.01E-02	4.27E-02	9.43E-01	FALSE	FALSE	14	62114923	6.56E-02	5.45E-01	TRUE	14	61648205	4.23E-02	0.92	7173	9.19E-06	TRUE	TRUE	482,730	19.70
Phosphatidylcholine (O-18:1_20:4) levels	Parkinson's disease	rs9800437	T	C	T	C	-1.17E-01	-8.46E-02	1.22E-01	9.56E-01	FALSE	FALSE	6	169719726	6.73E-02	6.80E-01	TRUE	6	169319631	2.59E-02	0.97	7173	5.97E-06	TRUE	TRUE	482,730	20.53
Phosphatidylcholine (O-18:2_16:0) levels	Parkinson's disease	rs10768424	G	A	G	A	7.86E-02	3.52E-02	5.73E-01	3.92E-01	FALSE	FALSE	11	4997634	1.92E-02	3.34E-02	TRUE	11	4976404	1.70E-02	1.00	7158	3.98E-06	TRUE	TRUE	482,730	21.31
Phosphatidylcholine (O-18:2_16:0) levels	Parkinson's disease	rs113016188	T	C	T	C	-7.96E-01	-1.72E-02	2.59E-03	9.85E-01	FALSE	FALSE	11	100164779	9.81E-02	6.49E-02	TRUE	11	100294047	1.65E-01	0.96	7158	1.56E-06	TRUE	TRUE	482,730	23.11
Phosphatidylcholine (O-18:2_16:0) levels	Parkinson's disease	rs114061227	G	A	G	A	7.95E-01	-6.94E-02	2.49E-03	9.93E-01	FALSE	FALSE	2	13033005	2.12E-01	1.29E-01	TRUE	2	12892879	1.79E-01	0.90	7158	9.34E-06	TRUE	TRUE	482,730	19.67
Phosphatidylcholine (O-18:2_16:0) levels	Parkinson's disease	rs11936543	G	A	G	A	-1.29E-01	5.67E-02	9.98E-02	9.62E-01	FALSE	FALSE	4	152191381	7.37E-02	3.54E-01	TRUE	4	151270229	2.84E-02	0.97	7158	5.25E-06	TRUE	TRUE	482,730	20.77
Phosphatidylcholine (O-18:2_16:0) levels	Parkinson's disease	rs12123955	T	C	T	C	-1.61E-01	-6.43E-02	6.12E-02	9.10E-01	FALSE	FALSE	1	200105423	3.97E-02	9.78E-01	TRUE	1	200136295	3.48E-02	0.99	7158	3.59E-06	TRUE	TRUE	482,730	21.50
Phosphatidylcholine (O-18:2_16:0) levels	Parkinson's disease	rs142235374	A	G	A	G	3.47E-01	-3.73E-02	1.77E-02	9.71E-01	FALSE	FALSE	4	7352153	6.88E-02	2.31E-01	TRUE	4	7350426	6.45E-02	0.96	7158	7.44E-08	TRUE	TRUE	482,730	29.01
Phosphatidylcholine (O-18:2_16:0) levels	Parkinson's disease	rs149389892	T	C	T	C	-3.25E-01	2.34E-02	1.37E-02	9.60E-01	FALSE	FALSE	16	47579412	4.48E-02	2.21E-01	TRUE	16	47545501	7.23E-02	1.00	7158	7.22E-06	TRUE	TRUE	482,730	20.16
Phosphatidylcholine (O-18:2_16:0) levels	Parkinson's disease	rs149897686	T	C	T	C	3.01E-01	2.35E-01	2.04E-02	9.87E-01	FALSE	FALSE	8	31108124	1.45E-01	9.78E-01	TRUE	8	31250608	6.25E-02	0.90	7158	1.46E-06	TRUE	TRUE	482,730	23.24
Phosphatidylcholine (O-18:2_16:0) levels	Parkinson's disease	rs174566	G	A	G	A	1.48E-01	-5.70E-03	4.08E-01	6.64E-01	FALSE	FALSE	11	61592362	1.78E-02	1.24E-01	TRUE	11	61824890	1.70E-02	1.00	7158	4.33E-18	TRUE	TRUE	482,730	75.56
Phosphatidylcholine (O-18:2_16:0) levels	Parkinson's disease	rs185625951	T	G	T	G	-2.93E-01	-1.23E-02	1.95E-02	9.87E-01	FALSE	FALSE	1	49994340	1.11E-01	4.01E-02	TRUE	1	49528668	6.23E-02	0.98	7158	2.52E-06	TRUE	TRUE	482,730	22.18
Phosphatidylcholine (O-18:2_16:0) levels	Parkinson's disease	rs188033349	A	G	A	G	-4.49E-01	6.13E-02	9.08E-03	9.89E-01	FALSE	FALSE	4	28254338	1.86E-01	1.30E-01	TRUE	4	28252716	9.42E-02	0.87	7158	1.88E-06	TRUE	TRUE	482,730	22.75
Phosphatidylcholine (O-18:2_16:0) levels	Parkinson's disease	rs34446132	A	G	A	G	-2.21E-01	6.22E-02	3.26E-02	9.58E-01	FALSE	FALSE	1	14731661	5.90E-02	5.36E-01	TRUE	1	14405165	4.88E-02	0.94	7158	6.08E-06	TRUE	TRUE	482,730	20.49
Phosphatidylcholine (O-18:2_16:0) levels	Parkinson's disease	rs55904113	T	C	T	C	9.65E-02	-4.21E-02	1.87E-01	8.50E-01	FALSE	FALSE	3	82497501	3.12E-02	7.52E-01	TRUE	3	82448350	2.13E-02	1.00	7158	6.11E-06	TRUE	TRUE	482,730	20.48
Phosphatidylcholine (O-18:2_16:0) levels	Parkinson's disease	rs7091107	T	C	T	C	3.66E-01	2.46E-02	1.08E-02	9.74E-01	FALSE	FALSE	10	56900932	6.93E-02	1.41E-01	TRUE	10	55141172	8.01E-02	1.00	7158	5.09E-06	TRUE	TRUE	482,730	20.83
Phosphatidylcholine (O-18:2_16:0) levels	Parkinson's disease	rs73263508	C	G	C	G	-2.10E-01	1.94E-01	3.40E-02	1.57E-02	TRUE	FALSE	17	13789873	1.06E-01	3.37E-02	TRUE	17	13886556	4.62E-02	1.00	7158	5.60E-06	TRUE	TRUE	482,730	20.65
Phosphatidylcholine (O-18:2_16:0) levels	Parkinson's disease	rs7563526	T	C	T	C	2.43E-01	-9.27E-02	3.13E-02	9.71E-01	FALSE	FALSE	2	81981968	6.99E-02	7.33E-01	TRUE	2	81754844	5.00E-02	0.90	7158	1.17E-06	TRUE	TRUE	482,730	23.67
Phosphatidylcholine (O-18:2_16:0) levels	Parkinson's disease	rs76437425	T	G	T	G	1.06E-01	3.00E-03	1.73E-01	7.96E-01	FALSE	FALSE	1	245677807	2.86E-02	3.85E-02	TRUE	1	245514505	2.23E-02	0.97	7158	1.96E-06	TRUE	TRUE	482,730	22.67
Phosphatidylcholine (O-18:2_16:0) levels	Parkinson's disease	rs7677028	G	C	G	C	3.27E-01	2.67E-02	1.43E-02	2.61E-02	TRUE	FALSE	4	137943535	7.37E-02	1.44E-01	TRUE	4	137022381	7.07E-02	0.97	7158	3.79E-06	TRUE	TRUE	482,730	21.40
Phosphatidylcholine (O-18:2_16:0) levels	Parkinson's disease	rs77219173	T	C	T	C	-2.64E-01	2.03E-02	2.01E-02	9.86E-01	FALSE	FALSE	4	79278431	8.90E-02	8.64E-02	TRUE	4	78357277	5.92E-02	0.99	7158	8.10E-06	TRUE	TRUE	482,730	19.94
Phosphatidylcholine (O-18:2_16:0) levels	Parkinson's disease	rs9341454	G	A	G	A	-8.16E-02	7.70E-03	6.71E-01	3.51E-01	FALSE	FALSE	6	74879604	2.35E-02	1.29E-01	TRUE	6	74169888	1.78E-02	1.00	7158	4.37E-06	TRUE	TRUE	482,730	21.13
Phosphatidylcholine (O-18:2_18:1) levels	Parkinson's disease	rs1077834	C	T	C	T	8.95E-02	-3.20E-03	2.66E-01	7.60E-01	FALSE	FALSE	15	58723479	2.20E-02	5.35E-02	TRUE	15	58431280	2.00E-02	0.98	6677	7.47E-06	TRUE	TRUE	482,730	20.10
Phosphatidylcholine (O-18:2_18:1) levels	Parkinson's disease	rs117376487	A	G	A	G	-4.17E-01	-1.34E-01	9.13E-03	9.82E-01	FALSE	FALSE	11	35093202	9.02E-02	8.65E-01	TRUE	11	35071655	8.95E-02	1.00	6677	3.31E-06	TRUE	TRUE	482,730	21.66
Phosphatidylcholine (O-18:2_18:1) levels	Parkinson's disease	rs12441343	A	G	A	G	7.94E-02	-1.70E-02	5.39E-01	4.41E-01	FALSE	FALSE	15	60231966	2.36E-02	3.26E-01	TRUE	15	59939767	1.75E-02	0.97	6677	5.58E-06	TRUE	TRUE	482,730	20.66

Phosphatidylcholine (O-18:2_18:1) levels	Parkinson's disease	rs174564	G	A	G	A	1.47E-01	-4.60E-03	4.09E-01	6.66E-01	FALSE	FALSE	11	61588305	1.78E-02	9.84E-02	TRUE	11	61820833	1.75E-02	1.00	6677	7.47E-17	TRUE	TRUE	482,730	69.91
Phosphatidylcholine (O-18:2_18:1) levels	Parkinson's disease	rs1982433	G	A	G	A	-1.35E-01	1.70E-03	9.22E-02	9.23E-01	FALSE	FALSE	8	78082727	4.17E-02	1.41E-02	TRUE	8	77170491	3.00E-02	1.00	6677	6.46E-06	TRUE	TRUE	482,730	20.38
Phosphatidylcholine (O-18:2_18:1) levels	Parkinson's disease	rs55975242	A	G	A	G	-2.16E-01	-1.40E-02	3.73E-02	9.14E-01	FALSE	FALSE	2	4249247	4.11E-02	1.34E-01	TRUE	2	4201657	4.60E-02	0.99	6677	2.58E-06	TRUE	TRUE	482,730	22.15
Phosphatidylcholine (O-18:2_18:1) levels	Parkinson's disease	rs56734149	A	C	A	C	1.03E-01	8.20E-03	1.80E-01	8.03E-01	FALSE	FALSE	3	142951449	2.79E-02	1.14E-01	TRUE	3	143232607	2.26E-02	1.00	6677	5.56E-06	TRUE	TRUE	482,730	20.67
Phosphatidylcholine (O-18:2_18:1) levels	Parkinson's disease	rs62621679	G	C	G	C	1.75E-01	-1.40E-02	6.09E-02	3.79E-02	TRUE	FALSE	17	64774358	6.16E-02	8.62E-02	TRUE	17	66778240	3.64E-02	0.99	6677	1.61E-06	TRUE	TRUE	482,730	23.04
Phosphatidylcholine (O-18:2_18:1) levels	Parkinson's disease	rs72709643	C	T	C	T	-2.17E-01	-1.55E-02	3.62E-02	9.54E-01	FALSE	FALSE	1	180973195	4.81E-02	1.26E-01	TRUE	1	181004059	4.65E-02	0.97	6677	3.21E-06	TRUE	TRUE	482,730	21.72
Phosphatidylcholine (O-18:2_18:1) levels	Parkinson's disease	rs72840788	A	G	A	G	9.73E-02	-9.10E-02	2.29E-01	7.84E-01	FALSE	FALSE	10	121415685	2.06E-02	4.99E-06	TRUE	10	119656173	2.09E-02	0.99	6677	3.16E-06	TRUE	TRUE	482,730	21.75
Phosphatidylcholine (O-18:2_18:1) levels	Parkinson's disease	rs73189920	C	T	C	T	1.33E-01	-2.39E-02	1.08E-01	8.98E-01	FALSE	FALSE	3	182038575	4.06E-02	2.55E-01	TRUE	3	182320787	2.93E-02	0.91	6677	6.13E-06	TRUE	TRUE	482,730	20.48
Phosphatidylcholine (O-18:2_18:1) levels	Parkinson's disease	rs7642984	T	C	T	C	1.30E-01	-4.33E-02	1.57E-01	8.52E-01	FALSE	FALSE	3	112557954	2.40E-02	3.56E-02	TRUE	3	112839107	2.38E-02	1.00	6677	5.05E-08	TRUE	TRUE	482,730	29.76
Phosphatidylcholine (O-18:2_18:1) levels	Parkinson's disease	rs79713419	C	A	C	A	8.76E-02	-6.33E-02	2.58E-01	8.34E-01	FALSE	FALSE	14	73912392	2.50E-02	5.67E-03	TRUE	14	73445684	1.97E-02	1.00	6677	9.10E-06	TRUE	TRUE	482,730	19.72
Phosphatidylcholine (O-18:2_18:1) levels	Parkinson's disease	rs79847391	T	A	T	A	1.68E-01	7.12E-02	5.61E-02	4.43E-02	TRUE	FALSE	6	70514387	5.51E-02	7.08E-01	TRUE	6	69804495	3.76E-02	0.99	6677	8.04E-06	TRUE	TRUE	482,730	19.96
Phosphatidylcholine (O-18:2_18:2) levels	Parkinson's disease	rs113370662	T	C	T	C	1.58E-01	7.66E-02	6.36E-02	9.68E-01	FALSE	FALSE	4	3537697	6.22E-02	6.62E-01	TRUE	4	3535970	3.54E-02	0.94	7153	8.15E-06	TRUE	TRUE	482,730	19.93
Phosphatidylcholine (O-18:2_18:2) levels	Parkinson's disease	rs114401161	G	T	G	T	1.95E-01	-1.07E-02	4.17E-02	9.74E-01	FALSE	FALSE	5	153621152	7.49E-02	5.26E-02	TRUE	5	154241592	4.19E-02	0.98	7153	3.14E-06	TRUE	TRUE	482,730	21.76
Phosphatidylcholine (O-18:2_18:2) levels	Parkinson's disease	rs114930909	A	G	A	G	3.69E-01	-3.44E-02	1.19E-02	9.80E-01	FALSE	FALSE	3	60223205	8.39E-02	1.66E-01	TRUE	3	60237477	7.84E-02	0.95	7153	2.57E-06	TRUE	TRUE	482,730	22.14
Phosphatidylcholine (O-18:2_18:2) levels	Parkinson's disease	rs12922657	G	A	G	A	7.89E-02	-1.60E-03	3.58E-01	6.46E-01	FALSE	FALSE	16	57318874	2.36E-02	2.42E-02	TRUE	16	57284962	1.74E-02	0.97	7153	6.13E-06	TRUE	TRUE	482,730	20.48
Phosphatidylcholine (O-18:2_18:2) levels	Parkinson's disease	rs149058290	T	C	T	C	-3.58E-01	-6.17E-02	1.20E-02	9.91E-01	FALSE	FALSE	5	38235494	2.03E-01	1.19E-01	TRUE	5	38235392	7.94E-02	0.92	7153	6.50E-06	TRUE	TRUE	482,730	20.37
Phosphatidylcholine (O-18:2_18:2) levels	Parkinson's disease	rs150046733	C	T	C	T	-1.75E-01	2.44E-02	4.92E-02	9.46E-01	FALSE	FALSE	11	732354	4.31E-02	2.43E-01	TRUE	11	732354	3.92E-02	0.97	7153	7.84E-06	TRUE	TRUE	482,730	20.00
Phosphatidylcholine (O-18:2_18:2) levels	Parkinson's disease	rs17231506	T	C	T	C	1.17E-01	1.36E-02	2.78E-01	6.87E-01	FALSE	FALSE	16	56994528	1.81E-02	3.45E-01	TRUE	16	56960616	1.86E-02	1.00	7153	2.96E-10	TRUE	TRUE	482,730	39.81
Phosphatidylcholine (O-18:2_18:2) levels	Parkinson's disease	rs174574	C	A	C	A	-1.56E-01	6.60E-03	5.91E-01	3.39E-01	FALSE	FALSE	11	61600342	1.77E-02	1.48E-01	TRUE	11	61832870	1.70E-02	1.00	7153	6.55E-20	TRUE	TRUE	482,730	83.94
Phosphatidylcholine (O-18:2_18:2) levels	Parkinson's disease	rs1800588	T	C	T	C	1.03E-01	-3.30E-03	2.58E-01	7.75E-01	FALSE	FALSE	15	58723675	2.12E-02	5.73E-02	TRUE	15	58431476	1.94E-02	0.99	7153	1.03E-07	TRUE	TRUE	482,730	28.37
Phosphatidylcholine (O-18:2_18:2) levels	Parkinson's disease	rs28489182	A	G	A	G	1.12E-01	-4.58E-02	1.45E-01	8.61E-01	FALSE	FALSE	14	78295342	3.23E-02	8.05E-01	TRUE	14	77828999	2.38E-02	0.97	7153	2.46E-06	TRUE	TRUE	482,730	22.23
Phosphatidylcholine (O-18:2_18:2) levels	Parkinson's disease	rs35874447	G	A	G	A	9.59E-02	-5.10E-02	2.88E-01	7.77E-01	FALSE	FALSE	12	125451589	2.31E-02	1.36E-02	TRUE	12	124967043	1.87E-02	0.96	7153	2.80E-07	TRUE	TRUE	482,730	26.43
Phosphatidylcholine (O-18:2_18:2) levels	Parkinson's disease	rs61448283	T	C	T	C	-1.75E-01	-7.38E-02	5.43E-02	9.71E-01	FALSE	FALSE	6	169717017	7.40E-02	4.97E-01	TRUE	6	169316922	3.77E-02	0.95	7153	3.69E-06	TRUE	TRUE	482,730	21.45
Phosphatidylcholine (O-18:2_18:2) levels	Parkinson's disease	rs7677028	G	C	G	C	3.24E-01	2.67E-02	1.43E-02	2.61E-02	TRUE	FALSE	4	137943535	7.37E-02	1.44E-01	TRUE	4	137022381	7.06E-02	0.97	7153	4.38E-06	TRUE	TRUE	482,730	21.12
Phosphatidylcholine (O-18:2_18:2) levels	Parkinson's disease	rs784655	G	A	G	A	-8.10E-02	-8.70E-03	5.50E-01	3.97E-01	FALSE	FALSE	9	109834455	2.27E-02	1.53E-01	TRUE	9	107072174	1.69E-02	1.00	7153	1.59E-06	TRUE	TRUE	482,730	23.07
Phosphatidylcholine (O-18:2_18:2) levels	Parkinson's disease	rs9366277	T	A	T	A	-7.75E-02	5.60E-03	6.36E-01	6.33E-01	TRUE	FALSE	6	10148033	1.91E-02	1.14E-01	TRUE	6	10147800	1.74E-02	1.00	7153	8.27E-06	TRUE	TRUE	482,730	19.90
Phosphatidylcholine (O-18:2_20:4) levels	Parkinson's disease	rs112718171	C	T	C	T	5.17E-01	-8.94E-02	6.57E-03	9.86E-01	FALSE	FALSE	7	41216631	1.19E-01	3.43E-01	TRUE	7	41177033	1.13E-01	0.96	6127	5.32E-06	TRUE	TRUE	482,730	20.75
Phosphatidylcholine (O-18:2_20:4) levels	Parkinson's disease	rs113023745	A	G	A	G	-2.54E-01	3.21E-02	3.70E-02	9.61E-01	FALSE	FALSE	13	67553349	5.97E-02	2.28E-01	TRUE	13	66979217	4.75E-02	0.99	6127	9.21E-08	TRUE	TRUE	482,730	28.60
Phosphatidylcholine (O-18:2_20:4) levels	Parkinson's disease	rs13401553	C	T	C	T	8.72E-02	-2.46E-02	4.28E-01	4.85E-01	FALSE	FALSE	2	124368033	2.22E-02	5.73E-01	TRUE	2	123610457	1.84E-02	1.00	6127	2.18E-06	TRUE	TRUE	482,730	22.47
Phosphatidylcholine (O-18:2_20:4) levels	Parkinson's disease	rs141198865	T	C	T	C	-3.49E-01	6.64E-02	1.75E-02	9.79E-01	FALSE	FALSE	17	17579357	9.56E-02	3.12E-01	TRUE	17	17676043	7.57E-02	0.85	6127	4.02E-06	TRUE	TRUE	482,730	21.29
Phosphatidylcholine (O-18:2_20:4) levels	Parkinson's disease	rs145525514	T	C	T	C	1.77E-01	2.90E-02	6.07E-02	9.62E-01	FALSE	FALSE	17	13166394	6.81E-02	1.74E-01	TRUE	17	13263077	3.90E-02	0.94	6127	5.59E-06	TRUE	TRUE	482,730	20.66
Phosphatidylcholine (O-18:2_20:4) levels	Parkinson's disease	rs16975847	A	G	A	G	-1.24E-01	-6.39E-02	1.34E-01	9.11E-01	FALSE	FALSE	16	75589590	3.19E-02	2.26E-02	TRUE	16	75555692	2.63E-02	1.00	6127	2.76E-06	TRUE	TRUE	482,730	22.01
Phosphatidylcholine (O-18:2_20:4) levels	Parkinson's disease	rs174537	T	G	T	G	-1.76E-01	-2.50E-03	4.08E-01	6.71E-01	FALSE	FALSE	11	61552680	1.79E-02	5.23E-02	TRUE	11	61785208	1.84E-02	1.00	6127	1.97E-21	TRUE	TRUE	482,730	91.04
Phosphatidylcholine (O-18:2_20:4) levels	Parkinson's disease	rs17778967	A	G	A	G	2.50E-01	-1.73E-02	3.23E-02	9.70E-01	FALSE	FALSE	15	43550822	8.86E-02	7.32E-02	TRUE	15	43258624	5.30E-02	0.93	6127	2.46E-06	TRUE	TRUE	482,730	22.24
Phosphatidylcholine (O-18:2_20:4) levels	Parkinson's disease	rs1936090	A	G	A	G	-8.18E-02	-2.06E-02	5.34E-01	5.24E-01	FALSE	FALSE	1	164922082	1.86E-02	5.71E-01	TRUE	1	164952845	1.82E-02	1.00	6127	6.91E-06	TRUE	TRUE	482,730	20.25
Phosphatidylcholine (O-18:2_20:4) levels	Parkinson's disease	rs1980615	C	A	C	A	-1.03E-01	-2.78E-02	4.55E-01	5.51E-01	FALSE	FALSE	14	67958372	1.73E-02	9.66E-01	TRUE	14	67491655	1.81E-02	1.00	6127	1.12E-08	TRUE	TRUE	482,730	32.72
Phosphatidylcholine (O-18:2_20:4) levels	Parkinson's disease	rs2189991	A	G	A	G	8.73E-02	-8.90E-03	5.87E-01	3.67E-01	FALSE	FALSE	7	82185719	2.31E-02	1.54E-01	TRUE	7	82556403	1.81E-02	1.00	6127	1.53E-06	TRUE	TRUE	482,730	23.15
Phosphatidylcholine (O-18:2_20:4) levels	Parkinson's disease	rs3820438	C	G	C	G	8.84E-02	5.55E-02	2.91E-01	2.27E-01	TRUE	FALSE	1	201924749	3.28E-02	4.53E-02	TRUE	1	201955621	1.99E-02	0.98	6127	9.13E-06	TRUE	TRUE	482,730	19.72
Phosphatidylcholine (O-18:2_20:4) levels	Parkinson's disease	rs6467318	G	A	G	A	-8.80E-02	5.41E-02	5.74E-01	3.97E-01	FALSE	FALSE	7	130486926	3.37E-02	9.64E-01	TRUE	7	130802167	1.88E-02	0.92	6127	2.82E-06	TRUE	TRUE	482,730	21.98
Phosphatidylcholine (O-18:2_20:4) levels	Parkinson's disease	rs6722606	C	A	C	A	-4.52E-01	-1.48E-02	9.49E-03	9.70E-01	FALSE	FALSE	2	192617263	6.68E-02	8.36E-02	TRUE	2	191752537	9.60E-02	0.99	6127	2.52E-06	TRUE	TRUE	482,730	22.19
Phosphatidylcholine (O-18:2_20:4) levels	Parkinson's disease	rs7118021	A	G	A	G	-1.17E-01	-1.11E-02	1.59E-01	7.67E-01	FALSE	FALSE	11	88791508	2.35E-02	1.96E-01	TRUE	11	89058340	2.46E-02	0.99	6127	1.95E-06	TRUE	TRUE	482,730	22.68
Phosphatidylcholine (O-18:2_20:4) levels	Parkinson's disease	rs7203057	G	T	G	T	-2.00E-01	-7.50E-03	4.13E-02	9.34E-01	FALSE	FALSE	16	57473616	4.51E-02	6.11E-02	TRUE	16	57439704	4.45E-02	1.00	6127	6.77E-06	TRUE	TRUE	482,730	20.29
Phosphatidylcholine (O-18:2_20:4) levels	Parkinson's disease	rs76375697	G	T	G	T	3.40E-01	4.80E-03	1.78E-02	9.78E-01	FALSE	FALSE	9	32337254	7.88E-02	2.14E-02	TRUE	9	32337256	7.11E-02	0.92	6127	1.77E-06	TRUE	TRUE	482,730	22.88
Phosphatidylcholine (O-18:2_20:4) levels	Parkinson's disease	rs7660654	T	A	T	A	-9.20E-02	1.83E-02	7.51E-01	7.23E-01	TRUE	FALSE	4	180829679	2.20E-02	3.93E-01	TRUE	4	179908526	2.08E-02	1.00	6127	9.70E-06	TRUE	TRUE	482,730	19.60
Phosphatidylethanolamine (16:0_18:2) levels	Parkinson's disease	rs1077834	C	T	C	T	3.16E-01	-3.20E-03	2.66E-01	7.60E-01	FALSE	FALSE	15	58723479	2.20E-02	5.35E-02	TRUE	15	58431280	2.12E-02	0.98	5462	1.42E-49	TRUE	TRUE	482,730	223.53
Phosphatidylethanolamine (16:0_18:2) levels	Parkinson's disease	rs117099105	C	G	C	G	4.59E-01	2.98E-02	1.09E-02	1.86E-02	TRUE	FALSE	9	119609128	8.99E-02	1.31E-01	TRUE	9	116846849	9.77E-02	0.96	5462	2.73E-06	TRUE	TRUE	482,730	22.04
Phosphatidylethanolamine (16:0_18:2) levels	Parkinson's disease	rs1260326	C	T	C	T	-1.16E-01	-6.78E-02	6.51E-01	4.20E-01	FALSE	FALSE	2	27730940	1.72E-02	4.04E-05	TRUE	2	27508073	1.99E-02	1.00	5462	5.70E-09	TRUE	TRUE	482,730	34.04
Phosphatidylethanolamine (16:0_18:2) levels	Parkinson's disease	rs140904588	A	G	A	G	1.97E-01	-1.00E-03	6.12E-02	9.60E-01	FALSE	FALSE	2	11819328	6.26E-02	5.59E-03	TRUE	2	11679202	4.22E-02	0.89	5462	2.89E-06	TRUE	TRUE	482,730	21.93
Phosphatidylethanolamine (16:0_18:2) levels	Parkinson's disease	rs174564	G	A	G	A	3.08E-01	-4.60E-03	4.09E-01	6.66E-01	FALSE	FALSE	11	61588305	1.78E-02	9.84E-02	TRUE	11	61820833	1.89E-02	1.00	5462	5.58E-58	TRUE	TRUE	482,730	263.82
Phosphatidylethanolamine (16:0_18:2) levels	Parkinson's disease	rs1891637	T	C	T	C	-1.02E-01	1.26E-02	7.71E-01	2.26E-01	FALSE	FALSE	9	126083523	3.25E-02	1.56E-01	TRUE	9	123321244	2.29E-02	0.97	5462	7.64E-06	TRUE	TRUE	482,730	20.06
Phosphatidylethanolamine (16:0_18:2) levels	Parkinson's disease	rs2043085	C	T	C	T	-2.63E-01	-2.04E-02	5.76E-01	3.79E-01	FALSE	FALSE	15	58680954	1.75E-02	6.16E-01	TRUE	15	58388755	1.88E-02	1.00	5462	1.18E-43	TRUE	TRUE	482,730	195.36
Phosphatidylethanolamine (16:0_18:2) levels	Parkinson's disease	rs34278620	G	A	G	A	2.25E-01	5.08E-02	1.25E-01	9.32E-01	FALSE	FALSE	15	59324390	4.57E-02	5.75E-01	TRUE	15	59032191	2.85E-02	1.00	5462	2.95E-15	TRUE	TRUE	482,730	62.66
Phosphatidylethanolamine (16:0_18:2) levels	Parkinson's disease	rs35319363	T	C	T	C	1.73E-01	3.52E-02	6.76E-02	9.72E-01	FALSE	FALSE	5	162771536	7.79E-02	1.86E-01	TRUE	5	163344530	3.84E-02	0.99	5462	6.97E-06	TRUE	TRUE	482,730	20.24
Phosphatidylethanolamine (16:0_18:2) levels	Parkinson's disease	rs4129186	C	T	C	T	-1.53E-01	5.81E-02	9.57E-02	8.81E-01	FALSE	FALSE	1	214569107	3.51E-02	4.89E-02	TRUE	1	214395764	3.29E-02	0.97	5462	3.37E-06	TRUE	TRUE	482,730	21.63
Phosphatidylethanolamine (16:0_18:2) levels	Parkinson's disease	rs4269529	C	T	C	T	-5.04E-01	-3.23E-02	9.96E-03	9.71E-01	FALSE	FALSE	8	67224046	6.82E-02	1.97E-01	TRUE	8	66311811	9.80E-02	0.99	5462	2.76E-07	TRUE	TRUE	482,730	26.47
Phosphatidylethanolamine (16:0_18:2) levels	Parkinson's disease	rs4363488	G	T	G	T	-1.09E-01	-1.34E-02	8.11E-01	2.24E-01	FALSE	FALSE	10	64862314	2.33E-02	2.49E-01	TRUE	10	63102554	2.44E-02	1.00	5462	8.20E-06	TRUE	TRUE	482,730	19.93
Phosphatidylethanolamine (16:0_18:2) levels	Parkinson's disease	rs436509	C	T	C	T	1.22E-01	2.30E-02	8.51E-01	2.00E-01	FALSE	FALSE	6	169789993	2.80E-02	3.86E-01	TRUE	6	169389898	2.70E-02	0.99	5462	6.32E-06	TRUE	TRUE	482,730	20.43
Phosphatidylethanolamine (16:0_18:2) levels	Parkinson's disease	rs510988	T	C	T	C	-1.46E-01	5.67E-02	8.29E-01	1.15E-01	FALSE	FALSE	11	116813804	2.66E-02	1.65E-02	TRUE	11	116943088	2.53E-02	1.00	5462	7.40E-09	TRUE	TRUE	482,730	33.53
Phosphatidylethanolamine (16:0_18:2) levels	Parkinson's disease	rs544282	A	G	A	G	1.06E-01	7.80E-03	2.80E-01	8.31E-01	FALSE	FALSE	15	59022102	2.65E-02	1.14E-01	TRUE	15	58729903	2.11E-02	1.00	5462	5.18E-07	TRUE	TRUE	482,730	25.26
Phosphatidylethanolamine (16:0_18:2) levels	Parkinson's disease	rs61696472	G	A	G	A	9.22E-02	-1.80E-02	3.90E-01	6.04E-01	FALSE	FALSE	7	43503828	2.38E-02	3.47E-01	TRUE	7	43464229	1.94E-02	1.00	5462	1.99E-06	TRUE	TRUE	482,730	22.65
Phosphatidylethanolamine (16:0_18:2) levels	Parkinson's disease	rs6415128	A	G	A	G	9.02E-02	2.28E-02	6.63E-01	4.02E-01	FALSE	FALSE	6	32330894	1.93E-02	6.23E-01	TRUE	6	32363117	2.01E-02	1.00	5462	7.65E-06	TRUE	TRUE	482,730	20.06
Phosphatidylethanolamine (16:0_18:2) levels	Parkinson's disease	rs7180275	G	A	G	A	-1.42E-01	-1.16E-02	1.53E-01	8.43E-01	FALSE	FALSE	15	74672161	2.54E-02	1.87E-01	TRUE	15	74379820	2.73E-02	0.99	5462	2.05E-07	TRUE	TRUE	482,730	27.05
Phosphatidylethanolamine (16:0_18:2) levels	Parkinson's disease	rs7550598	G	T	G	T	1.71E-01	-5.60E-02	7.15E-02	9.37E-01	FALSE	FALSE	1	15628252	3.66E-02	8.99E-01	TRUE	1	15301756	3.75E-02	1.00	5462	5.20E-06	TRUE	TRUE	482,730	20.80
Phosphatidylethanolamine (16:0_18:2) levels	Parkinson's disease	rs894342	T	C	T	C	9.56E-02	8.10E-03	5.30E-01	4.05E-01	FALSE	FALSE	15	58177591	1.92E-02	1.73E-01	TRUE	15	57885393	1.93E-02	0.99	5462	7.35E-07	TRUE	TRUE	482,730	24.58
Phosphatidylethanolamine (16:0_20:4) levels	Parkinson's disease	rs10892181	G	T	G	T	8.03E-02	-5.00E-04	4.66E-01	6.36E-01	FALSE	FALSE	11	117699586	2.78E-02	6.21E-03	TRUE	11	117828871	1.74E-02	0.98	6666	4.17E-06	TRUE	TRUE	482,730	21.22
Phosphatidylethanolamine (16:0_20:4) levels	Parkinson's disease	rs115265221	G	C	G	C	-4.67E-01	6.50E-02	8.21E-03	2.96E-02	TRUE	FALSE	3	140877487	6.78E-02	4.72E-01	TRUE	3	141158645	9.67E-02	0.97	6666	1.37E-06	TRUE	TRUE	482,730	23.36
Phosphatidylethanolamine (16:0_20:4) levels	Parkinson's disease	rs1193168	C	A	C	A	8.46E-02	-2.23E-02	6.55E-01	3.72E-01	FALSE	FALSE	1	7591122	1.80E-02	6.64E-01	TRUE	1	7531062	1.85E-02	0.98	6666	4.64E-06	TRUE	TRUE	482,730	21.01
Phosphatidylethanolamine (16:0_20:4) levels	Parkinson's disease	rs1260326	C	T	C	T	-8.62E-02	-6.78E-02	6.51E-01	4.20E-01	FALSE	FALSE	2	27730940	1.72E-02	4.04E-05	TRUE	2	27508073	1.82E-02	1.00	6666	2.11E-06	TRUE	TRUE	482,730	22.53
Phosphatidylethanolamine (16:0_20:4) levels	Parkinson's disease	rs139848079	T	C	T	C	-2.54E-01	-4.94E-02	3.04E-02	9.47E-01	FALSE	FALSE	10	61128500	5.39E-02	4.45E-01	TRUE	10	59368742	5.31E-02	0.91	6666	1.66E-06	TRUE	TRUE	482,730	22.99
Phosphatidylethanolamine (16:0_20:4) levels	Parkinson's disease	rs142259144	C	T	C	T	1.48E-01	4.41E-02	7.83E-02	9.41E-01	FALSE	FALSE	14	57798333	5.06E-02	4.15E-01	TRUE	14	57331615	3.32E-02	0.96	6666	8.74E-06	TRUE	TRUE	482,730	19.80
Phosphatidylethanolamine (16:0_20:4) levels	Parkinson's disease	rs149460121	A	T	A	T	1.45E-01	-7.80E-02	1.09E-01	2.74E-02	TRUE	FALSE	9	140752952	8.70E-02	4.32E-01	TRUE	9	137858500	2.91E-02	0.93	6666	5.91E-07	TRUE	TRUE	482,730	24.99
Phosphatidylethanolamine (16:0_20:4) levels	Parkinson's disease	rs1690333	C	T	C	T	9.96E-02	-6.60E-03	4.39E-01	6.10E-01	FALSE	FALSE	15	57909186	1.77E-02	1.49E-01	TRUE	15	57616988	1.75E-02	1.00	6666	1.20E-08	TRUE	TRUE	482,730	32.56
Phosphatidylethanolamine (16:0_20:4) levels	Parkinson's disease	rs1800588	T	C	T	C	3.93E-01	-3.30E-03	2.58E-01	7.75E-01	FALSE	FALSE	15	58723675	2.12E-02	5.73E-02	TRUE	15	58431476	1.94E-02	0.99	6666	1.26E-88	TRUE	TRUE	482,730	410.38
Phosphatidylethanolamine (16:0_20:4) levels	Parkinson's disease	rs1815786	C	T	C	T	-1.34E-01	3.42E-02	8.66E-01	9.32E-02	FALSE	FALSE	11	116921390	2.94E-02	6.12E-01	TRUE	11	117050674	2.54E-02	1.00	6666	1.43E-07	TRUE	TRUE	482,730	27.74
Phosphatidylethanolamine (16:0_20:4) levels	Parkinson's disease	rs2043085	C	T	C	T	-3.72E-01	-2.04E-02	5.76E-01	3.79E-01	FALSE	FALSE	15	58680954	1.75E-02	6.16E-01	TRUE	15	58388755	1.69E-02	1.00	6666	3.62E-104	TRUE	TRUE	482,730	486.44
Phosphatidylethanolamine (16:0_20:4) levels	Parkinson's disease	rs34278620	G	A	G	A	2.13E-01	5.08E-02	1.25E-01	9.32E-01	FALSE	FALSE	15	59324390	4.57E-02	5.75E-01	TRUE	15	59032191	2.62E-02	1.00	6666	4.39E-16	TRUE	TRUE	482,730	66.40
Phosphatidylethanolamine (16:0_20:4) levels	Parkinson's disease	rs34733845	T	C	T	C	1.67E-01	5.54E-02	5.92E-02	9.72E-01	FALSE	FALSE	12	61255422	7.14E-02	3.59E-01	TRUE	12	60861641	3.69E-02	0.97	6666	5.96E-06	TRUE	TRUE	482,730	20.53
Phosphatidylethanolamine (16:0_20:4) levels	Parkinson's disease	rs62155727	G	A	G	A	-1.21E-01	-4.84E-02	1.51E-01	8.43E-01	FALSE	FALSE	2	102356997	2.40E-02	2.19E-02	TRUE	2	101740535	2.40E-02	1.00	6666	4.42E-07	TRUE	TRUE	482,730	25.55
Phosphatidylethanolamine (16:0_20:4) levels	Parkinson's disease	rs72679383	T	G	T	G	3.13E-01	6.22E-02	2.16E-02	9.84E-01	FALSE	FALSE	4	101850410	1.18E-01	2.24E-01	TRUE	4	100929253	6.08E-02	0.96	6666	2.65E-07	TRUE	TRUE	482,730	26.54
Phosphatidylethanolamine (16:0_20:4) levels	Parkinson's disease	rs74929979	C	T	C	T	-1.87E-01	-3.90E-03	4.66E-02	9.58E-01	FALSE	FALSE	3	8910651	5.93E-02	2.36E-02	TRUE	3	8868967	4.20E-02	0.98	6666	9.13E-06	TRUE	TRUE	482,730	19.71
Phosphatidylethanolamine (16:0_20:4) levels	Parkinson's disease	rs75344234	C	T	C	T	-1.78E-01	1.04E-01	6.58E-02	9.39E-01	FALSE	FALSE	8	139176960	6.71E-02	9.15E-01	TRUE	8	138164717	3.74E-02	0.87	6666	1.98E-06	TRUE	TRUE	482,730	22.66
Phosphatidylethanolamine (16:0_20:4) levels	Parkinson's disease	rs76435838	A	C	A	C	1.47E-01	-4.63E-02	7.47E-02	9.34E-01	FALSE	FALSE	7	35610009	4.51E-02	5.17E-01	TRUE	7	35570399	3.28E-02	1.00	6666	7.46E-06	TRUE	TRUE	482,730	20.10
Phosphatidylethanolamine (16:0_20:4) levels	Parkinson's disease	rs79450270	A	G	A	G	3.83E-01	2.20E-03	1.27E-02	9.91E-01	FALSE	FALSE	2	139503514	2.03E-01	3.75E-03	TRUE	2	138745944	8.20E-02	0.90	6666	3.03E-06	TRUE	TRUE	482,730	21.83
Phosphatidylethanolamine (16:0_20:4) levels	Parkinson's disease	rs9943332	A	G	A	G	1.20E-01	-1.76E-02	1.35E-01	8.62E-01	FALSE	FALSE	10	129859769	2.76E-02	2.81E-01	TRUE	10	128061505	2.57E-02	0.99	6666	3.01E-06	TRUE	TRUE	482,730	21.85
Phosphatidylethanolamine (18:0_18:2) levels	Parkinson's disease	rs10069895	C	T	C	T	9.72E-02	3.63E-02	7.64E-01	1.43E-01	FALSE	FALSE	5	85593102	3.17E-02	5.98E-01	TRUE	5	86297284	1.96E-02	1.00	7128	7.18E-07	TRUE	TRUE	482,730	24.61
Phosphatidylethanolamine (18:0_18:2) levels	Parkinson's disease	rs10276778	C	T	C	T	-1.82E-01	-5.68E-02	4.40E-02	9.76E-01	FALSE	FALSE	7	146691509	7.64E-02	3.40E-01	TRUE	7	146994417	4.03E-02	1.00	7128	6.58E-06	TRUE	TRUE	482,730	20.34
Phosphatidylethanolamine (18:0_18:2) levels	Parkinson's disease	rs10468017	T	C	T	C	2.98E-01	2.75E-02	3.38E-01	7.16E-01	FALSE	FALSE	15	58678512	1.87E-02	8.48E-01	TRUE	15	58386313	1.72E-02	1.00	7128	9.03E-66	TRUE	TRUE	482,730	299.64
Phosphatidylethanolamine (18:0_18:2) levels	Parkinson's disease	rs111543310	C	T	C	T	2.80E-01	2.90E-03	2.23E-02	9.80E-01	FALSE	FALSE	15	59531818	9.70E-02	1.05E-02	TRUE	15	59239619	6.04E-02	0.89	7128	3.55E-06	TRUE	TRUE	482,730	21.53
Phosphatidylethanolamine (18:0_18:2) levels	Parkinson's disease	rs112129861	C	G	C	G	1.56E-01	-1.08E-01	6.48E-02	5.60E-02	TRUE	FALSE	15	59339649	4.96E-02	1.45E-02	TRUE	15	59047450	3.38E-02	1.00	7128	3.91E-06	TRUE	TRUE	482,730	21.34
Phosphatidylethanolamine (18:0_18:2) levels	Parkinson's disease	rs117376818	A	G	A	G	6.30E-01	1.24E-01	1.58E-02	9.88E-01	FALSE	FALSE	15	58798961	1.67E-01	3.41E-01	TRUE	15	58506762	7.04E-02	0.90	7128	4.16E-19	TRUE	TRUE	482,730	80.24
Phosphatidylethanolamine (18:0_18:2) levels	Parkinson's disease	rs117900629	A	G	A	G	-2.13E-01	-2.16E-02	6.18E-02	9.78E-01	FALSE	FALSE	11	61972800	7.58E-02	1.11E-01	TRUE	11	62205328	3.45E-02	0.99	7128	6.25E-10	TRUE	TRUE	482,730	38.35
Phosphatidylethanolamine (18:0_18:2) levels	Parkinson's disease	rs1260326	C	T	C	T	-1.22E-01	-6.78E-02	6.51E-01	4.20E-01	FALSE	FALSE	2	27730940	1.72E-02	4.04E-05	TRUE	2	27508073	1.74E-02	1.00	7128	2.57E-12	TRUE	TRUE	482,730	49.15
Phosphatidylethanolamine (18:0_18:2) levels	Parkinson's disease	rs12928099	A	C	A	C	-1.13E-01	-1.09E-02	2.74E-01	6.96E-01	FALSE	FALSE	16	15150505	1.84E-02	2.58E-01	TRUE	16	15056648	1.86E-02	1.00	7128	1.50E-09	TRUE	TRUE	482,730	36.63
Phosphatidylethanolamine (18:0_18:2) levels	Parkinson's disease	rs13110318	A	G	A	G	-1.21E-01	6.23E-02	1.14E-01	9.06E-01	FALSE	FALSE	4	38138856	3.73E-02	4.74E-02	TRUE	4	38137235	2.61E-02	1.00	7128	3.74E-06	TRUE	TRUE	482,730	21.43
Phosphatidylethanolamine (18:0_18:2) levels	Parkinson's disease	rs138283783	C	T	C	T	3.52E-01	1.05E-01	2.66E-02	9.88E-01	FALSE	FALSE	15	58536527	1.36E-01	3.58E-01	TRUE	15	58244328	5.36E-02	0.96	7128	5.56E-11	TRUE	TRUE	482,730	43.10
Phosphatidylethanolamine (18:0_18:2) levels	Parkinson's disease	rs141883259	G	A	G	A	3.73E-01	1.48E-01	1.02E-02	9.87E-01	FALSE	FALSE	5	119396702	1.01E-01	8.40E-01	TRUE	5	120061007	8.26E-02	0.98	7128	6.59E-06	TRUE	TRUE	482,730	20.34
Phosphatidylethanolamine (18:0_18:2) levels	Parkinson's disease	rs149431055	T	C	T	C	-2.43E-01	4.62E-02	2.48E-02	9.57E-01	FALSE	FALSE	4	116795833	6.01E-02	3.54E-01	TRUE	4	115874677	5.47E-02	0.97	7128	9.10E-06	TRUE	TRUE	482,730	19.72
Phosphatidylethanolamine (18:0_18:2) levels	Parkinson's disease	rs2946493	C	T	C	T	1.05E-01	-2.73E-02	2.06E-01	8.07E-01	FALSE	FALSE	8	12823671	2.60E-02	5.32E-01	TRUE	8	12966162	2.08E-02	0.98	7128	4.35E-07	TRUE	TRUE	482,730	25.58
Phosphatidylethanolamine (18:0_18:2) levels	Parkinson's disease	rs34733845	T	C	T	C	1.65E-01	5.54E-02	5.92E-02	9.72E-01	FALSE	FALSE	12	61255422	7.14E-02	3.59E-01	TRUE	12	60861641	3.57E-02	0.97	7128	3.86E-06	TRUE	TRUE	482,730	21.37
Phosphatidylethanolamine (18:0_18:2) levels	Parkinson's disease	rs4296402	A	G	A	G	1.06E-01	1.01E-02	1.60E-01	8.95E-01	FALSE	FALSE	2	175561291	3.80E-02	1.02E-01	TRUE	2	174696563	2.30E-02	0.98	7128	4.49E-06	TRUE	TRUE	482,730	21.07
Phosphatidylethanolamine (18:0_18:2) levels	Parkinson's disease	rs62291572	T	C	T	C	1.00E-01	-2.71E-02	2.14E-01	7.47E-01	FALSE	FALSE	4	4997927	2.75E-02	4.89E-01	TRUE	4	4996200	2.10E-02	0.94	7128	1.96E-06	TRUE	TRUE	482,730	22.67
Phosphatidylethanolamine (18:0_18:2) levels	Parkinson's disease	rs62434228	A	G	A	G	1.09E-01	1.69E-02	1.60E-01	9.08E-01	FALSE	FALSE	6	155002491	3.92E-02	1.76E-01	TRUE	6	154681357	2.30E-02	0.99	7128	2.35E-06	TRUE	TRUE	482,730	22.32
Phosphatidylethanolamine (18:0_18:2) levels	Parkinson's disease	rs633695	G	A	G	A	2.55E-01	2.32E-02	3.14E-01	6.47E-01	FALSE	FALSE	15	58725839	2.19E-02	5.39E-01	TRUE	15	58433640	1.80E-02	1.00	7128	5.56E-45	TRUE	TRUE	482,730	200.84
Phosphatidylethanolamine (18:0_18:2) levels	Parkinson's disease	rs7226158	C	T	C	T	-8.08E-02	1.94E-02	6.84E-01	3.01E-01	FALSE	FALSE	17	77390725	2.46E-02	3.67E-01	TRUE	17	79394643	1.78E-02	1.00	7128	6.06E-06	TRUE	TRUE	482,730	20.50
Phosphatidylethanolamine (18:0_18:2) levels	Parkinson's disease	rs72679383	T	G	T	G	2.86E-01	6.22E-02	2.16E-02	9.84E-01	FALSE	FALSE	4	101850410	1.18E-01	2.24E-01	TRUE	4	100929253	5.85E-02	0.96	7128	1.04E-06	TRUE	TRUE	482,730	23.89
Phosphatidylethanolamine (18:0_18:2) levels	Parkinson's disease	rs73424597	C	T	C	T	3.55E-01	2.31E-02	3.50E-02	9.68E-01	FALSE	FALSE	15	58899609	6.42E-02	1.43E-01	TRUE	15	58607410	4.53E-02	0.99	7128	5.88E-15	TRUE	TRUE	482,730	61.20
Phosphatidylethanolamine (18:0_18:2) levels	Parkinson's disease	rs805292	A	G	A	G	8.50E-02	-5.20E-03	3.78E-01	6.89E-01	FALSE	FALSE	6	31690009	2.24E-02	8.72E-02	TRUE	6	31722232	1.74E-02	1.00	7128	1.10E-06	TRUE	TRUE	482,730	23.79
Phosphatidylethanolamine (18:0_18:2) levels	Parkinson's disease	rs964184	C	G	C	G	-1.54E-01	-5.68E-02	8.49E-01	8.63E-01	TRUE	FALSE	11	116648917	2.43E-02	9.71E-03	TRUE	11	116778201	2.32E-02	1.00	7128	2.75E-11	TRUE	TRUE	482,730	44.48
Phosphatidylethanolamine (18:0_20:4) levels	Parkinson's disease	rs10468017	T	C	T	C	3.54E-01	2.75E-02	3.38E-01	7.16E-01	FALSE	FALSE	15	58678512	1.87E-02	8.48E-01	TRUE	15	58386313	1.71E-02	1.00	7164	7.59E-93	TRUE	TRUE	482,730	430.24
Phosphatidylethanolamine (18:0_20:4) levels	Parkinson's disease	rs10750098	T	G	T	G	-1.49E-01	2.43E-02	8.63E-01	9.23E-02	FALSE	FALSE	11	116705568	2.96E-02	3.85E-01	TRUE	11	116834852	2.45E-02	1.00	7164	1.09E-09	TRUE	TRUE	482,730	37.25
Phosphatidylethanolamine (18:0_20:4) levels	Parkinson's disease	rs11031419	T	A	T	A	1.77E-01	5.74E-02	5.86E-02	3.39E-02	TRUE	FALSE	11	31605896	6.38E-02	4.33E-01	TRUE	11	31584349	3.54E-02	0.99	7164	6.13E-07	TRUE	TRUE	482,730	24.91
Phosphatidylethanolamine (18:0_20:4) levels	Parkinson's disease	rs111543310	C	T	C	T	2.71E-01	2.90E-03	2.23E-02	9.80E-01	FALSE	FALSE	15	59531818	9.70E-02	1.05E-02	TRUE	15	59239619	6.01E-02	0.89	7164	6.69E-06	TRUE	TRUE	482,730	20.31
Phosphatidylethanolamine (18:0_20:4) levels	Parkinson's disease	rs112129861	C	G	C	G	1.74E-01	-1.08E-01	6.48E-02	5.60E-02	TRUE	FALSE	15	59339649	4.96E-02	1.45E-02	TRUE	15	59047450	3.37E-02	1.00	7164	2.51E-07	TRUE	TRUE	482,730	26.65
Phosphatidylethanolamine (18:0_20:4) levels	Parkinson's disease	rs113887618	A	G	A	G	-8.19E-02	-1.08E-02	3.01E-01	7.34E-01	FALSE	FALSE	3	71883321	2.53E-02	1.75E-01	TRUE	3	71834170	1.82E-02	1.00	7164	6.81E-06	TRUE	TRUE	482,730	20.27
Phosphatidylethanolamine (18:0_20:4) levels	Parkinson's disease	rs116866162	A	G	A	G	-2.60E-01	2.03E-02	2.51E-02	9.79E-01	FALSE	FALSE	8	11482360	8.99E-02	8.53E-02	TRUE	8	11624851	5.48E-02	0.93	7164	2.07E-06	TRUE	TRUE	482,730	22.56
Phosphatidylethanolamine (18:0_20:4) levels	Parkinson's disease	rs117376818	A	G	A	G	6.64E-01	1.24E-01	1.58E-02	9.88E-01	FALSE	FALSE	15	58798961	1.67E-01	3.41E-01	TRUE	15	58506762	7.01E-02	0.90	7164	3.43E-21	TRUE	TRUE	482,730	89.85

Phosphatidylethanolamine (18:0_20:4) levels	Parkinson's disease	rs1260326	C	T	C	T	-8.75E-02	-6.78E-02	6.51E-01	4.20E-01	FALSE	FALSE	2	27730940	1.72E-02	4.04E-05	TRUE	2	27508073	1.74E-02	1.00	7164	5.43E-07	TRUE	TRUE	482,730	25.15
Phosphatidylethanolamine (18:0_20:4) levels	Parkinson's disease	rs138283783	C	T	C	T	3.65E-01	1.05E-01	2.66E-02	9.88E-01	FALSE	FALSE	15	58536527	1.36E-01	3.58E-01	TRUE	15	58244328	5.34E-02	0.96	7164	9.22E-12	TRUE	TRUE	482,730	46.64
Phosphatidylethanolamine (18:0_20:4) levels	Parkinson's disease	rs143607354	T	C	T	C	-1.88E-01	3.33E-02	4.24E-02	9.81E-01	FALSE	FALSE	4	62413208	9.98E-02	1.32E-01	TRUE	4	61547490	4.13E-02	0.99	7164	5.48E-06	TRUE	TRUE	482,730	20.69
Phosphatidylethanolamine (18:0_20:4) levels	Parkinson's disease	rs174537	T	G	T	G	-1.88E-01	-2.50E-03	4.08E-01	6.71E-01	FALSE	FALSE	11	61552680	1.79E-02	5.23E-02	TRUE	11	61785208	1.69E-02	1.00	7164	1.33E-28	TRUE	TRUE	482,730	124.17
Phosphatidylethanolamine (18:0_20:4) levels	Parkinson's disease	rs62549032	A	G	A	G	2.26E-01	-3.00E-03	3.07E-02	9.85E-01	FALSE	FALSE	9	76707664	1.64E-01	6.30E-03	TRUE	9	74092748	4.88E-02	0.95	7164	3.71E-06	TRUE	TRUE	482,730	21.44
Phosphatidylethanolamine (18:0_20:4) levels	Parkinson's disease	rs633695	G	A	G	A	2.88E-01	2.32E-02	3.14E-01	6.47E-01	FALSE	FALSE	15	58725839	2.19E-02	5.39E-01	TRUE	15	58433640	1.79E-02	1.00	7164	2.75E-57	TRUE	TRUE	482,730	259.05
Phosphatidylethanolamine (18:0_20:4) levels	Parkinson's disease	rs72753379	T	C	T	C	2.21E-01	2.30E-02	3.92E-02	9.80E-01	FALSE	FALSE	9	123112954	9.08E-02	9.67E-02	TRUE	9	120350676	4.47E-02	0.95	7164	8.09E-07	TRUE	TRUE	482,730	24.38
Phosphatidylethanolamine (18:0_20:4) levels	Parkinson's disease	rs73424577	G	A	G	A	3.46E-01	7.40E-03	3.45E-02	9.69E-01	FALSE	FALSE	15	58869185	6.53E-02	4.12E-02	TRUE	15	58576986	4.56E-02	0.99	7164	3.66E-14	TRUE	TRUE	482,730	57.58
Phosphatidylethanolamine (18:0_20:4) levels	Parkinson's disease	rs77144832	A	G	A	G	2.77E-01	-2.08E-01	1.87E-02	9.73E-01	FALSE	FALSE	13	76632839	7.22E-02	2.00E-03	TRUE	13	76058703	6.23E-02	0.96	7164	8.93E-06	TRUE	TRUE	482,730	19.76
Phosphatidylethanolamine (18:1_18:1) levels	Parkinson's disease	rs10751670	G	A	G	A	-9.84E-02	-2.11E-02	8.14E-01	1.13E-01	FALSE	FALSE	11	944466	2.99E-02	3.17E-01	TRUE	11	944466	2.17E-02	1.00	7045	5.99E-06	TRUE	TRUE	482,730	20.52
Phosphatidylethanolamine (18:1_18:1) levels	Parkinson's disease	rs10915321	A	G	A	G	1.69E-01	9.10E-03	6.95E-02	9.25E-01	FALSE	FALSE	1	4106146	4.69E-02	7.27E-02	TRUE	1	4046086	3.57E-02	0.85	7045	2.38E-06	TRUE	TRUE	482,730	22.29
Phosphatidylethanolamine (18:1_18:1) levels	Parkinson's disease	rs112129861	C	G	C	G	1.94E-01	-1.08E-01	6.48E-02	5.60E-02	TRUE	FALSE	15	59339649	4.96E-02	1.45E-02	TRUE	15	59047450	3.40E-02	1.00	7045	1.08E-08	TRUE	TRUE	482,730	32.76
Phosphatidylethanolamine (18:1_18:1) levels	Parkinson's disease	rs112314129	T	C	T	C	1.47E-01	-2.12E-02	7.44E-02	9.16E-01	FALSE	FALSE	6	138332019	4.20E-02	2.12E-01	TRUE	6	138010882	3.28E-02	0.95	7045	7.26E-06	TRUE	TRUE	482,730	20.15
Phosphatidylethanolamine (18:1_18:1) levels	Parkinson's disease	rs11633043	A	G	A	G	1.35E-01	9.90E-03	1.91E-01	8.60E-01	FALSE	FALSE	15	58837722	3.25E-02	1.20E-01	TRUE	15	58545523	2.17E-02	0.97	7045	4.34E-10	TRUE	TRUE	482,730	39.06
Phosphatidylethanolamine (18:1_18:1) levels	Parkinson's disease	rs1260326	C	T	C	T	-8.30E-02	-6.78E-02	6.51E-01	4.20E-01	FALSE	FALSE	2	27730940	1.72E-02	4.04E-05	TRUE	2	27508073	1.76E-02	1.00	7045	2.56E-06	TRUE	TRUE	482,730	22.15
Phosphatidylethanolamine (18:1_18:1) levels	Parkinson's disease	rs140764699	A	G	A	G	-4.76E-01	7.62E-02	7.05E-03	9.84E-01	FALSE	FALSE	9	95390032	1.05E-01	3.29E-01	TRUE	9	92627750	1.02E-01	0.97	7045	3.08E-06	TRUE	TRUE	482,730	21.80
Phosphatidylethanolamine (18:1_18:1) levels	Parkinson's disease	rs151000252	T	G	T	G	7.89E-01	-1.57E-01	2.48E-03	9.92E-01	FALSE	FALSE	6	86033449	1.75E-01	4.33E-01	TRUE	6	85323731	1.78E-01	0.92	7045	9.22E-06	TRUE	TRUE	482,730	19.69
Phosphatidylethanolamine (18:1_18:1) levels	Parkinson's disease	rs16874868	T	A	T	A	-1.27E-01	5.00E-04	1.06E-01	1.29E-01	TRUE	FALSE	5	5149361	3.03E-02	6.12E-03	TRUE	5	5149248	2.78E-02	1.00	7045	4.62E-06	TRUE	TRUE	482,730	21.02
Phosphatidylethanolamine (18:1_18:1) levels	Parkinson's disease	rs17151028	G	A	G	A	9.15E-02	-1.89E-02	2.14E-01	7.07E-01	FALSE	FALSE	7	43544507	2.44E-02	3.59E-01	TRUE	7	43504908	2.04E-02	1.00	7045	7.40E-06	TRUE	TRUE	482,730	20.12
Phosphatidylethanolamine (18:1_18:1) levels	Parkinson's disease	rs17174502	A	G	A	G	1.36E-01	-5.35E-02	1.74E-01	8.94E-01	FALSE	FALSE	11	116759655	2.78E-02	2.71E-02	TRUE	11	116888939	2.23E-02	0.99	7045	1.14E-09	TRUE	TRUE	482,730	37.16
Phosphatidylethanolamine (18:1_18:1) levels	Parkinson's disease	rs174548	G	C	G	C	1.90E-01	2.00E-03	3.84E-01	3.00E-01	TRUE	FALSE	11	61571348	1.83E-02	3.90E-02	TRUE	11	61803876	1.73E-02	1.00	7045	1.12E-27	TRUE	TRUE	482,730	119.86
Phosphatidylethanolamine (18:1_18:1) levels	Parkinson's disease	rs2070895	A	G	A	G	2.59E-01	-4.20E-03	2.67E-01	7.58E-01	FALSE	FALSE	15	58723939	2.23E-02	7.08E-02	TRUE	15	58431740	1.92E-02	0.98	7045	3.11E-41	TRUE	TRUE	482,730	183.22
Phosphatidylethanolamine (18:1_18:1) levels	Parkinson's disease	rs2503980	T	A	T	A	-8.27E-02	8.70E-03	3.25E-01	3.88E-01	TRUE	FALSE	6	67839458	1.95E-02	1.83E-01	TRUE	6	67129565	1.82E-02	1.00	7045	5.68E-06	TRUE	TRUE	482,730	20.62
Phosphatidylethanolamine (18:1_18:1) levels	Parkinson's disease	rs2946819	T	G	T	G	-1.43E-01	-2.17E-02	9.12E-01	9.64E-02	FALSE	FALSE	3	118218911	3.81E-02	2.45E-01	TRUE	3	118500064	3.03E-02	0.99	7045	2.38E-06	TRUE	TRUE	482,730	22.30
Phosphatidylethanolamine (18:1_18:1) levels	Parkinson's disease	rs72880096	C	T	C	T	1.36E-01	-7.38E-02	9.91E-02	8.76E-01	FALSE	FALSE	17	75014147	3.38E-02	1.45E-02	TRUE	17	77018065	2.85E-02	1.00	7045	1.75E-06	TRUE	TRUE	482,730	22.88
Phosphatidylethanolamine (18:1_18:1) levels	Parkinson's disease	rs74899875	T	C	T	C	1.84E-01	-6.50E-03	5.16E-02	9.58E-01	FALSE	FALSE	2	12010336	6.01E-02	3.90E-02	TRUE	2	11870210	3.97E-02	0.93	7045	3.60E-06	TRUE	TRUE	482,730	21.50
Phosphatidylethanolamine (18:1_18:1) levels	Parkinson's disease	rs7779208	A	G	A	G	-4.22E-01	-8.00E-02	1.02E-02	9.78E-01	FALSE	FALSE	7	146201517	8.17E-02	4.85E-01	TRUE	7	146504425	8.70E-02	0.92	7045	1.22E-06	TRUE	TRUE	482,730	23.59
Phosphatidylethanolamine (18:1_18:1) levels	Parkinson's disease	rs78564380	A	G	A	G	3.46E-01	-7.33E-02	1.60E-02	9.64E-01	FALSE	FALSE	2	13498588	6.81E-02	5.50E-01	TRUE	2	13358463	7.18E-02	0.88	7045	1.46E-06	TRUE	TRUE	482,730	23.23
Phosphatidylethanolamine (18:1_18:1) levels	Parkinson's disease	rs9837048	G	A	G	A	-7.58E-02	-5.58E-02	5.58E-01	5.22E-01	FALSE	FALSE	3	132780117	2.23E-02	6.17E-03	TRUE	3	133061273	1.69E-02	1.00	7045	7.59E-06	TRUE	TRUE	482,730	20.07
Phosphatidylethanolamine (18:1_18:1) levels	Parkinson's disease	rs9920308	A	C	A	C	8.73E-02	-2.20E-03	4.18E-01	6.15E-01	FALSE	FALSE	15	57902231	1.81E-02	4.51E-02	TRUE	15	57610033	1.72E-02	1.00	7045	4.26E-07	TRUE	TRUE	482,730	25.62
Phosphatidylethanolamine (O-16:1_18:2) levels	Parkinson's disease	rs10949554	G	A	G	A	-9.75E-02	6.80E-03	3.00E-01	7.15E-01	FALSE	FALSE	6	10113526	2.11E-02	1.26E-01	TRUE	6	10113293	2.16E-02	0.93	5430	6.67E-06	TRUE	TRUE	482,730	20.32
Phosphatidylethanolamine (O-16:1_18:2) levels	Parkinson's disease	rs111680982	T	C	T	C	-5.64E-01	6.26E-02	6.91E-03	9.67E-01	FALSE	FALSE	8	122348782	6.72E-02	4.55E-01	TRUE	8	121336542	1.26E-01	0.86	5430	8.44E-06	TRUE	TRUE	482,730	19.87
Phosphatidylethanolamine (O-16:1_18:2) levels	Parkinson's disease	rs111761575	C	T	C	T	5.64E-01	-2.41E-02	6.24E-03	9.84E-01	FALSE	FALSE	13	27695675	9.01E-02	1.03E-01	TRUE	13	27121538	1.27E-01	0.99	5430	8.76E-06	TRUE	TRUE	482,730	19.80
Phosphatidylethanolamine (O-16:1_18:2) levels	Parkinson's disease	rs115261885	A	G	A	G	1.85E-01	8.31E-02	5.81E-02	9.43E-01	FALSE	FALSE	1	67069745	4.35E-02	2.80E-02	TRUE	1	66604062	4.14E-02	0.97	5430	7.78E-06	TRUE	TRUE	482,730	20.03
Phosphatidylethanolamine (O-16:1_18:2) levels	Parkinson's disease	rs12997068	A	G	A	G	2.73E-01	-5.74E-02	2.94E-02	9.35E-01	FALSE	FALSE	2	42424857	4.77E-02	6.41E-01	TRUE	2	42197717	5.65E-02	0.97	5430	1.36E-06	TRUE	TRUE	482,730	23.38
Phosphatidylethanolamine (O-16:1_18:2) levels	Parkinson's disease	rs145863254	T	C	T	C	2.73E-01	-1.43E-01	2.72E-02	9.85E-01	FALSE	FALSE	15	69988580	1.38E-01	5.20E-01	TRUE	15	69696241	6.15E-02	0.93	5430	9.08E-06	TRUE	TRUE	482,730	19.73
Phosphatidylethanolamine (O-16:1_18:2) levels	Parkinson's disease	rs174530	G	A	G	A	1.49E-01	-1.80E-03	4.14E-01	6.44E-01	FALSE	FALSE	11	61546592	1.79E-02	3.58E-02	TRUE	11	61779120	1.93E-02	1.00	5430	1.33E-14	TRUE	TRUE	482,730	59.64
Phosphatidylethanolamine (O-16:1_18:2) levels	Parkinson's disease	rs311415	G	C	G	C	-9.18E-02	-4.48E-02	5.88E-01	6.25E-01	TRUE	FALSE	8	55111214	2.29E-02	2.52E-02	TRUE	8	54198654	1.95E-02	1.00	5430	2.65E-06	TRUE	TRUE	482,730	22.10
Phosphatidylethanolamine (O-16:1_18:2) levels	Parkinson's disease	rs35485513	C	T	C	T	-3.56E-01	4.64E-02	1.70E-02	9.66E-01	FALSE	FALSE	4	28424704	6.54E-02	3.21E-01	TRUE	4	28423082	7.94E-02	0.93	5430	7.74E-06	TRUE	TRUE	482,730	20.04
Phosphatidylethanolamine (O-16:1_18:2) levels	Parkinson's disease	rs62294904	T	A	T	A	1.62E-01	3.83E-02	9.76E-02	8.51E-02	TRUE	FALSE	3	182180352	4.50E-02	4.04E-01	TRUE	3	182462564	3.49E-02	0.87	5430	3.30E-06	TRUE	TRUE	482,730	21.68
Phosphatidylethanolamine (O-16:1_18:2) levels	Parkinson's disease	rs71520868	G	A	G	A	4.59E-01	-1.37E-01	9.92E-03	9.83E-01	FALSE	FALSE	8	105162724	1.02E-01	7.54E-01	TRUE	8	104150496	1.04E-01	0.95	5430	9.92E-06	TRUE	TRUE	482,730	19.56
Phosphatidylethanolamine (O-16:1_18:2) levels	Parkinson's disease	rs75480840	G	C	G	C	3.69E-01	1.10E-02	1.37E-02	1.79E-02	TRUE	FALSE	16	79936417	9.21E-02	4.34E-02	TRUE	16	79902520	8.13E-02	0.96	5430	5.87E-06	TRUE	TRUE	482,730	20.57
Phosphatidylethanolamine (O-16:1_18:2) levels	Parkinson's disease	rs78655391	C	T	C	T	1.90E-01	1.16E-01	6.58E-02	9.29E-01	FALSE	FALSE	1	111068053	4.65E-02	6.27E-03	TRUE	1	110525431	3.99E-02	0.96	5430	1.97E-06	TRUE	TRUE	482,730	22.67
Phosphatidylethanolamine (O-16:1_18:2) levels	Parkinson's disease	rs78816612	T	C	T	C	-5.91E-01	2.14E-02	6.05E-03	9.80E-01	FALSE	FALSE	1	111382656	7.26E-02	1.15E-01	TRUE	1	110840034	1.33E-01	0.87	5430	9.31E-06	TRUE	TRUE	482,730	19.68
Phosphatidylethanolamine (O-16:1_18:2) levels	Parkinson's disease	rs9590678	A	T	A	T	9.82E-02	-3.95E-02	4.10E-01	3.78E-01	TRUE	FALSE	13	42811550	2.77E-02	8.16E-01	TRUE	13	42237414	1.98E-02	0.97	5430	7.54E-07	TRUE	TRUE	482,730	24.53
Phosphatidylethanolamine (O-16:1_20:4) levels	Parkinson's disease	rs10234951	G	T	G	T	-1.51E-01	-4.75E-02	8.00E-02	8.81E-01	FALSE	FALSE	7	94048534	3.70E-02	7.00E-01	TRUE	7	94419222	3.10E-02	0.99	7169	1.17E-06	TRUE	TRUE	482,730	23.67
Phosphatidylethanolamine (O-16:1_20:4) levels	Parkinson's disease	rs1034755	T	C	T	C	8.50E-02	1.95E-02	3.19E-01	6.71E-01	FALSE	FALSE	7	8850785	2.32E-02	3.96E-01	TRUE	7	8811155	1.79E-02	0.98	7169	2.13E-06	TRUE	TRUE	482,730	22.50
Phosphatidylethanolamine (O-16:1_20:4) levels	Parkinson's disease	rs10487877	G	C	G	C	1.82E-01	1.13E-01	4.73E-02	1.84E-02	TRUE	FALSE	7	80467301	1.35E-01	3.93E-01	TRUE	7	80837985	3.98E-02	0.98	7169	5.12E-06	TRUE	TRUE	482,730	20.82
Phosphatidylethanolamine (O-16:1_20:4) levels	Parkinson's disease	rs11136432	G	T	G	T	-8.05E-02	1.90E-02	6.02E-01	4.40E-01	FALSE	FALSE	8	1791638	2.29E-02	3.90E-01	TRUE	8	1843472	1.71E-02	0.98	7169	2.74E-06	TRUE	TRUE	482,730	22.03
Phosphatidylethanolamine (O-16:1_20:4) levels	Parkinson's disease	rs11725298	C	T	C	T	2.59E-01	-5.85E-02	2.23E-02	9.79E-01	FALSE	FALSE	4	53583422	8.74E-02	2.98E-01	TRUE	4	52717255	5.67E-02	0.96	7169	4.96E-06	TRUE	TRUE	482,730	20.88
Phosphatidylethanolamine (O-16:1_20:4) levels	Parkinson's disease	rs12923459	A	G	A	G	-7.49E-02	-1.19E-02	4.57E-01	5.56E-01	FALSE	FALSE	16	56989830	1.77E-02	3.02E-01	TRUE	16	56955918	1.68E-02	0.99	7169	8.79E-06	TRUE	TRUE	482,730	19.78
Phosphatidylethanolamine (O-16:1_20:4) levels	Parkinson's disease	rs143745495	T	C	T	C	-9.82E-02	3.60E-03	1.72E-01	8.76E-01	FALSE	FALSE	1	119715178	3.01E-02	4.31E-02	TRUE	1	119172555	2.21E-02	1.00	7169	8.71E-06	TRUE	TRUE	482,730	19.80
Phosphatidylethanolamine (O-16:1_20:4) levels	Parkinson's disease	rs2070895	A	G	A	G	8.50E-02	-4.20E-03	2.67E-01	7.58E-01	FALSE	FALSE	15	58723939	2.23E-02	7.08E-02	TRUE	15	58431740	1.92E-02	0.98	7169	9.89E-06	TRUE	TRUE	482,730	19.56
Phosphatidylethanolamine (O-16:1_20:4) levels	Parkinson's disease	rs2277289	C	T	C	T	8.04E-02	2.23E-02	3.26E-01	6.81E-01	FALSE	FALSE	11	118182660	2.06E-02	5.56E-01	TRUE	11	118311945	1.78E-02	1.00	7169	6.69E-06	TRUE	TRUE	482,730	20.31
Phosphatidylethanolamine (O-16:1_20:4) levels	Parkinson's disease	rs2573210	A	G	A	G	1.13E-01	9.30E-03	8.38E-01	2.02E-01	FALSE	FALSE	2	233385025	2.30E-02	1.64E-01	TRUE	2	232520315	2.27E-02	0.98	7169	6.34E-07	TRUE	TRUE	482,730	24.85
Phosphatidylethanolamine (O-16:1_20:4) levels	Parkinson's disease	rs3820438	C	G	C	G	8.99E-02	5.55E-02	2.91E-01	2.27E-01	TRUE	FALSE	1	201924749	3.28E-02	4.53E-02	TRUE	1	201955621	1.84E-02	0.98	7169	1.03E-06	TRUE	TRUE	482,730	23.92
Phosphatidylethanolamine (O-16:1_20:4) levels	Parkinson's disease	rs6601694	A	G	A	G	8.58E-02	-2.82E-02	3.34E-01	7.30E-01	FALSE	FALSE	8	8248058	2.21E-02	6.94E-01	TRUE	8	8390548	1.78E-02	0.99	7169	1.47E-06	TRUE	TRUE	482,730	23.23
Phosphatidylethanolamine (O-16:1_20:4) levels	Parkinson's disease	rs7445920	T	C	T	C	9.66E-02	1.10E-03	1.93E-01	8.13E-01	FALSE	FALSE	5	73973825	2.27E-02	1.71E-02	TRUE	5	74678000	2.11E-02	0.99	7169	4.82E-06	TRUE	TRUE	482,730	20.94
Phosphatidylethanolamine (O-16:1_20:4) levels	Parkinson's disease	rs74677222	A	G	A	G	1.46E-01	-8.28E-02	8.30E-02	9.32E-01	FALSE	FALSE	12	122220917	3.96E-02	1.83E-02	TRUE	12	121783011	3.05E-02	0.98	7169	1.79E-06	TRUE	TRUE	482,730	22.85
Phosphatidylethanolamine (O-16:1_20:4) levels	Parkinson's disease	rs79337987	A	T	A	T	-2.94E-01	-1.54E-01	1.95E-02	1.23E-02	TRUE	FALSE	7	137839857	1.10E-01	7.90E-01	TRUE	7	138155111	6.08E-02	0.97	7169	1.32E-06	TRUE	TRUE	482,730	23.43
Phosphatidylethanolamine (O-16:1_20:4) levels	Parkinson's disease	rs9265944	C	T	C	T	8.35E-02	-2.37E-02	3.91E-01	5.51E-01	FALSE	FALSE	6	31315109	2.09E-02	5.92E-01	TRUE	6	31347332	1.71E-02	0.99	7169	1.13E-06	TRUE	TRUE	482,730	23.74
Phosphatidylethanolamine (O-16:1_20:4) levels	Parkinson's disease	rs97384	C	T	C	T	2.10E-01	7.50E-03	5.80E-01	3.66E-01	FALSE	FALSE	11	61624181	1.98E-02	1.53E-01	TRUE	11	61856709	1.69E-02	0.99	7169	4.34E-35	TRUE	TRUE	482,730	154.39
Phosphatidylethanolamine (O-16:1_22:5) levels	Parkinson's disease	rs10187082	G	C	G	C	-5.27E-01	-2.83E-02	6.66E-03	2.47E-02	TRUE	FALSE	2	141519001	7.51E-02	1.51E-01	TRUE	2	140761432	1.15E-01	0.99	5609	4.40E-06	TRUE	TRUE	482,730	21.13
Phosphatidylethanolamine (O-16:1_22:5) levels	Parkinson's disease	rs111698799	T	C	T	C	-3.91E-01	-1.99E-02	1.17E-02	9.81E-01	FALSE	FALSE	10	111275755	8.45E-02	8.97E-02	TRUE	10	109515997	8.60E-02	0.97	5609	5.47E-06	TRUE	TRUE	482,730	20.70
Phosphatidylethanolamine (O-16:1_22:5) levels	Parkinson's disease	rs115002881	T	G	T	G	2.43E-01	1.68E-01	3.08E-02	9.87E-01	FALSE	FALSE	2	62499087	1.75E-01	4.70E-01	TRUE	2	62271952	5.49E-02	0.99	5609	9.44E-06	TRUE	TRUE	482,730	19.66
Phosphatidylethanolamine (O-16:1_22:5) levels	Parkinson's disease	rs115536895	A	G	A	G	2.71E-01	1.72E-01	2.70E-02	9.82E-01	FALSE	FALSE	3	192927733	7.76E-02	1.34E-02	TRUE	3	193209944	5.99E-02	0.97	5609	6.09E-06	TRUE	TRUE	482,730	20.50
Phosphatidylethanolamine (O-16:1_22:5) levels	Parkinson's disease	rs116250572	C	G	C	G	5.79E-01	-1.02E-01	5.41E-03	1.53E-02	TRUE	FALSE	5	106903533	1.06E-01	4.70E-01	TRUE	5	107567832	1.29E-01	0.90	5609	6.85E-06	TRUE	TRUE	482,730	20.27
Phosphatidylethanolamine (O-16:1_22:5) levels	Parkinson's disease	rs150942220	C	G	C	G	1.81E-01	-4.39E-02	6.21E-02	6.34E-02	TRUE	FALSE	5	32055367	6.01E-02	3.33E-01	TRUE	5	32055261	4.09E-02	0.90	5609	9.64E-06	TRUE	TRUE	482,730	19.62
Phosphatidylethanolamine (O-16:1_22:5) levels	Parkinson's disease	rs190716231	C	G	C	G	-3.09E-01	-1.23E-01	2.12E-02	1.58E-02	TRUE	FALSE	7	132783436	9.54E-02	7.08E-01	TRUE	7	133098676	6.69E-02	0.97	5609	3.97E-06	TRUE	TRUE	482,730	21.32
Phosphatidylethanolamine (O-16:1_22:5) levels	Parkinson's disease	rs2940919	G	A	G	A	1.33E-01	-2.90E-02	1.15E-01	8.10E-01	FALSE	FALSE	5	42470730	2.72E-02	5.42E-01	TRUE	5	42470628	2.92E-02	1.00	5609	5.21E-06	TRUE	TRUE	482,730	20.80
Phosphatidylethanolamine (O-16:1_22:5) levels	Parkinson's disease	rs41264497	G	A	G	A	-6.34E-01	5.92E-02	5.74E-03	9.86E-01	FALSE	FALSE	1	40090422	1.00E-01	2.55E-01	TRUE	1	39624750	1.27E-01	0.97	5609	5.96E-07	TRUE	TRUE	482,730	24.98
Phosphatidylethanolamine (O-16:1_22:5) levels	Parkinson's disease	rs4974228	C	T	C	T	1.54E-01	1.20E-02	9.21E-02	8.85E-01	FALSE	FALSE	3	60763597	3.30E-02	1.45E-01	TRUE	3	60777864	3.27E-02	1.00	5609	2.43E-06	TRUE	TRUE	482,730	22.27
Phosphatidylethanolamine (O-16:1_22:5) levels	Parkinson's disease	rs55892660	T	C	T	C	-1.20E-01	-2.90E-03	1.48E-01	8.39E-01	FALSE	FALSE	1	98835679	3.06E-02	3.46E-02	TRUE	1	98370123	2.66E-02	0.99	5609	7.05E-06	TRUE	TRUE	482,730	20.22
Phosphatidylethanolamine (O-16:1_22:5) levels	Parkinson's disease	rs7028232	T	C	T	C	9.34E-02	1.99E-02	3.74E-01	6.95E-01	FALSE	FALSE	9	117017793	2.45E-02	3.80E-01	TRUE	9	114255513	2.00E-02	0.98	5609	2.95E-06	TRUE	TRUE	482,730	21.89
Phosphatidylethanolamine (O-16:1_22:5) levels	Parkinson's disease	rs72993205	G	A	G	A	3.11E-01	5.49E-02	2.36E-02	9.68E-01	FALSE	FALSE	2	230490365	6.98E-02	3.65E-01	TRUE	2	229625649	6.80E-02	0.86	5609	5.07E-06	TRUE	TRUE	482,730	20.85
Phosphatidylethanolamine (O-16:1_22:5) levels	Parkinson's disease	rs73199626	C	T	C	T	-1.04E-01	3.74E-02	2.09E-01	8.34E-01	FALSE	FALSE	3	190888563	3.13E-02	6.35E-01	TRUE	3	191170774	2.31E-02	0.98	5609	6.31E-06	TRUE	TRUE	482,730	20.43
Phosphatidylethanolamine (O-16:1_22:5) levels	Parkinson's disease	rs76268189	T	C	T	C	3.23E-01	6.90E-02	1.91E-02	9.58E-01	FALSE	FALSE	8	63895236	6.09E-02	5.90E-01	TRUE	8	62982677	7.22E-02	0.94	5609	8.04E-06	TRUE	TRUE	482,730	19.96
Phosphatidylethanolamine (O-16:1_22:5) levels	Parkinson's disease	rs7761485	C	T	C	T	-8.73E-02	-1.26E-02	3.85E-01	6.07E-01	FALSE	FALSE	6	86113968	2.24E-02	2.40E-01	TRUE	6	85404250	1.97E-02	1.00	5609	9.05E-06	TRUE	TRUE	482,730	19.73
Phosphatidylethanolamine (O-18:1_18:2) levels	Parkinson's disease	rs10811102	T	G	T	G	-1.07E-01	-4.00E-03	1.95E-01	7.40E-01	FALSE	FALSE	9	1911291	2.70E-02	5.37E-02	TRUE	9	1911291	2.25E-02	0.91	6870	2.28E-06	TRUE	TRUE	482,730	22.38
Phosphatidylethanolamine (O-18:1_18:2) levels	Parkinson's disease	rs10848158	C	T	C	T	9.34E-02	-3.00E-04	4.34E-01	5.86E-01	FALSE	FALSE	12	131091966	2.30E-02	3.93E-03	TRUE	12	130607421	1.73E-02	0.97	6870	7.17E-08	TRUE	TRUE	482,730	29.08
Phosphatidylethanolamine (O-18:1_18:2) levels	Parkinson's disease	rs10926263	T	G	T	G	-8.60E-02	2.23E-02	6.98E-01	2.60E-01	FALSE	FALSE	1	240611348	2.60E-02	4.08E-01	TRUE	1	240448048	1.89E-02	0.98	6870	5.29E-06	TRUE	TRUE	482,730	20.76
Phosphatidylethanolamine (O-18:1_18:2) levels	Parkinson's disease	rs111703312	G	A	G	A	3.34E-01	-5.39E-02	1.57E-02	9.90E-01	FALSE	FALSE	2	205373798	2.04E-01	1.01E-01	TRUE	2	204509075	7.06E-02	0.94	6870	2.19E-06	TRUE	TRUE	482,730	22.45
Phosphatidylethanolamine (O-18:1_18:2) levels	Parkinson's disease	rs118048608	C	T	C	T	2.47E-01	-8.24E-02	2.56E-02	9.71E-01	FALSE	FALSE	8	115278115	6.57E-02	6.78E-01	TRUE	8	114265886	5.49E-02	1.00	6870	6.92E-06	TRUE	TRUE	482,730	20.25
Phosphatidylethanolamine (O-18:1_18:2) levels	Parkinson's disease	rs11936543	G	A	G	A	-1.43E-01	5.67E-02	9.98E-02	9.62E-01	FALSE	FALSE	4	152191381	7.37E-02	3.54E-01	TRUE	4	151270229	2.89E-02	0.97	6870	7.70E-07	TRUE	TRUE	482,730	24.47
Phosphatidylethanolamine (O-18:1_18:2) levels	Parkinson's disease	rs149968069	T	C	T	C	-2.50E-01	8.40E-02	2.53E-02	9.80E-01	FALSE	FALSE	1	242237754	8.77E-02	4.71E-01	TRUE	1	242074452	5.48E-02	0.95	6870	5.34E-06	TRUE	TRUE	482,730	20.74
Phosphatidylethanolamine (O-18:1_18:2) levels	Parkinson's disease	rs17019568	G	C	G	C	1.51E-01	-5.18E-02	8.80E-02	1.20E-01	TRUE	FALSE	4	93479889	3.01E-02	4.26E-02	TRUE	4	92558738	3.04E-02	1.00	6870	7.10E-07	TRUE	TRUE	482,730	24.63
Phosphatidylethanolamine (O-18:1_18:2) levels	Parkinson's disease	rs17138200	G	C	G	C	9.14E-02	-5.96E-02	2.14E-01	2.19E-01	TRUE	FALSE	7	17736907	2.69E-02	1.34E-02	TRUE	7	17697283	2.05E-02	1.00	6870	8.84E-06	TRUE	TRUE	482,730	19.78
Phosphatidylethanolamine (O-18:1_18:2) levels	Parkinson's disease	rs174547	C	T	C	T	1.94E-01	-3.30E-03	4.07E-01	6.72E-01	FALSE	FALSE	11	61570783	1.79E-02	6.93E-02	TRUE	11	61803311	1.73E-02	1.00	6870	3.60E-29	TRUE	TRUE	482,730	126.83
Phosphatidylethanolamine (O-18:1_18:2) levels	Parkinson's disease	rs182992622	T	C	T	C	6.38E-01	-1.69E-01	3.68E-03	9.92E-01	FALSE	FALSE	1	153793197	1.75E-01	4.77E-01	TRUE	1	153820721	1.43E-01	0.93	6870	8.01E-06	TRUE	TRUE	482,730	19.96
Phosphatidylethanolamine (O-18:1_18:2) levels	Parkinson's disease	rs187748847	G	A	G	A	-5.87E-01	-1.56E-01	5.16E-03	9.95E-01	FALSE	FALSE	3	181321122	2.83E-01	2.35E-01	TRUE	3	181603334	1.24E-01	0.93	6870	2.29E-06	TRUE	TRUE	482,730	22.37
Phosphatidylethanolamine (O-18:1_18:2) levels	Parkinson's disease	rs261334	C	G	C	G	-1.04E-01	2.22E-02	7.58E-01	6.16E-01	TRUE	FALSE	15	58726744	2.16E-02	5.19E-01	TRUE	15	58434545	2.00E-02	1.00	6870	1.91E-07	TRUE	TRUE	482,730	27.17
Phosphatidylethanolamine (O-18:1_18:2) levels	Parkinson's disease	rs28415525	G	A	G	A	-1.29E-01	1.98E-02	9.46E-02	8.76E-01	FALSE	FALSE	17	68143434	3.37E-02	2.54E-01	TRUE	17	70147293	2.90E-02	1.00	6870	8.81E-06	TRUE	TRUE	482,730	19.78
Phosphatidylethanolamine (O-18:1_18:2) levels	Parkinson's disease	rs28844909	A	G	A	G	-1.67E-01	-4.91E-02	6.66E-02	9.73E-01	FALSE	FALSE	4	146628373	8.04E-02	2.67E-01	TRUE	4	145707221	3.48E-02	0.95	6870	1.69E-06	TRUE	TRUE	482,730	22.95
Phosphatidylethanolamine (O-18:1_18:2) levels	Parkinson's disease	rs4696957	A	G	A	G	-1.67E-01	-1.63E-02	5.75E-02	9.25E-01	FALSE	FALSE	4	18852672	4.37E-02	1.49E-01	TRUE	4	18851049	3.73E-02	0.94	6870	7.14E-06	TRUE	TRUE	482,730	20.19
Phosphatidylethanolamine (O-18:1_18:2) levels	Parkinson's disease	rs55683433	A	C	A	C	-2.10E-01	-1.70E-02	4.24E-02	9.30E-01	FALSE	FALSE	4	75812514	4.88E-02	1.38E-01	TRUE	4	74887304	4.36E-02	0.94	6870	1.52E-06	TRUE	TRUE	482,730	23.16
Phosphatidylethanolamine (O-18:1_18:2) levels	Parkinson's disease	rs68103316	C	T	C	T	9.84E-02	1.20E-03	1.84E-01	8.74E-01	FALSE	FALSE	7	81320077	3.43E-02	1.28E-02	TRUE	7	81690761	2.22E-02	0.97	6870	9.14E-06	TRUE	TRUE	482,730	19.71
Phosphatidylethanolamine (O-18:1_18:2) levels	Parkinson's disease	rs7003304	T	C	T	C	-1.20E-01	4.80E-03	1.39E-01	8.39E-01	FALSE	FALSE	8	14191967	3.07E-02	5.83E-02	TRUE	8	14334458	2.50E-02	0.99	6870	1.54E-06	TRUE	TRUE	482,730	23.14
Phosphatidylethanolamine (O-18:1_18:2) levels	Parkinson's disease	rs709166	A	G	A	G	-7.78E-02	6.00E-03	4.73E-01	5.37E-01	FALSE	FALSE	3	12511652	2.18E-02	1.06E-01	TRUE	3	12470153	1.70E-02	1.00	6870	5.08E-06	TRUE	TRUE	482,730	20.84
Phosphatidylethanolamine (O-18:1_18:2) levels	Parkinson's disease	rs76085699	A	G	A	G	-1.22E-01	-2.87E-02	1.34E-01	8.93E-01	FALSE	FALSE	1	206911375	3.93E-02	3.32E-01	TRUE	1	206738030	2.53E-02	0.98	6870	1.42E-06	TRUE	TRUE	482,730	23.29
Phosphatidylethanolamine (O-18:1_18:2) levels	Parkinson's disease	rs9644026	C	T	C	T	8.24E-02	2.33E-02	3.10E-01	6.52E-01	FALSE	FALSE	8	22793887	2.33E-02	4.99E-01	TRUE	8	22936374	1.85E-02	1.00	6870	9.06E-06	TRUE	TRUE	482,730	19.73
Phosphatidylethanolamine (O-18:1_18:2) levels	Parkinson's disease	rs9652580	T	C	T	C	-8.05E-02	1.80E-02	4.23E-01	5.64E-01	FALSE	FALSE	16	24308825	2.28E-02	3.65E-01	TRUE	16	24297504	1.76E-02	0.98	6870	5.02E-06	TRUE	TRUE	482,730	20.86
Phosphatidylethanolamine (O-18:1_20:4) levels	Parkinson's disease	rs10234951	G	T	G	T	-1.49E-01	-4.75E-02	8.00E-02	8.81E-01	FALSE	FALSE	7	94048534	3.70E-02	7.00E-01	TRUE	7	94419222	3.11E-02	0.99	7172	1.69E-06	TRUE	TRUE	482,730	22.95
Phosphatidylethanolamine (O-18:1_20:4) levels	Parkinson's disease	rs10520189	A	G	A	G	1.22E-01	-1.70E-02	1.14E-01	9.41E-01	FALSE	FALSE	4	171641235	5.64E-02	1.17E-01	TRUE	4	170720084	2.71E-02	0.93	7172	7.55E-06	TRUE	TRUE	482,730	20.08
Phosphatidylethanolamine (O-18:1_20:4) levels	Parkinson's disease	rs10756627	C	T	C	T	7.50E-02	7.30E-03	5.51E-01	4.88E-01	FALSE	FALSE	9	14928594	2.26E-02	1.27E-01	TRUE	9	14928596	1.68E-02	0.99	7172	8.04E-06	TRUE	TRUE	482,730	19.96
Phosphatidylethanolamine (O-18:1_20:4) levels	Parkinson's disease	rs11062287	A	G	A	G	2.55E-01	1.77E-02	3.39E-02	9.64E-01	FALSE	FALSE	12	2739704	7.57E-02	8.90E-02	TRUE	12	2630538	5.04E-02	0.83	7172	4.45E-07	TRUE	TRUE	482,730	25.54
Phosphatidylethanolamine (O-18:1_20:4) levels	Parkinson's disease	rs113913626	T	C	T	C	-2.25E-01	-1.93E-01	3.23E-02	9.82E-01	FALSE	FALSE	12	76649394	8.81E-02	1.42E-02	TRUE	12	76255614	4.82E-02	0.94	7172	3.27E-06	TRUE	TRUE	482,730	21.68
Phosphatidylethanolamine (O-18:1_20:4) levels	Parkinson's disease	rs117002537	T	C	T	C	2.91E-01	1.80E-01	1.81E-02	9.82E-01	FALSE	FALSE	9	133819302	9.20E-02	2.53E-02	TRUE	9	130943915	6.53E-02	0.91	7172	8.52E-06	TRUE	TRUE	482,730	19.85
Phosphatidylethanolamine (O-18:1_20:4) levels	Parkinson's disease	rs11725298	C	T	C	T	2.56E-01	-5.85E-02	2.23E-02	9.79E-01	FALSE	FALSE	4	53583422	8.74E-02	2.98E-01	TRUE	4	52717255	5.67E-02	0.96	7172	6.50E-06	TRUE	TRUE	482,730	20.36
Phosphatidylethanolamine (O-18:1_20:4) levels	Parkinson's disease	rs117565387	T	C	T	C	1.75E-01	-1.06E-01	5.32E-02	9.73E-01	FALSE	FALSE	16	5974671	6.91E-02	9.01E-01	TRUE	16	5924670	3.77E-02	0.96	7172	3.51E-06	TRUE	TRUE	482,730	21.55
Phosphatidylethanolamine (O-18:1_20:4) levels	Parkinson's disease	rs1203148	G	A	G	A	9.07E-02	-5.07E-02	2.40E-01	7.90E-01	FALSE	FALSE	1	35968899	2.72E-02	3.12E-02	TRUE	1	35503298	2.02E-02	0.93	7172	7.34E-06	TRUE	TRUE	482,730	20.13
Phosphatidylethanolamine (O-18:1_20:4) levels	Parkinson's disease	rs1513795	C	T	C	T	8.00E-02	-2.32E-02	4.29E-01	4.79E-01	FALSE	FALSE	2	124404961	2.22E-02	5.28E-01	TRUE	2	123647385	1.70E-02	1.00	7172	2.47E-06	TRUE	TRUE	482,730	22.22
Phosphatidylethanolamine (O-18:1_20:4) levels	Parkinson's disease	rs174536	C	A	C	A	-2.51E-01	-2.00E-03	4.08E-01	6.68E-01	FALSE	FALSE	11	61551927	1.78E-02	4.16E-02	TRUE	11	61784455	1.68E-02	1.00	7172	1.09E-49	TRUE	TRUE	482,730	223.03
Phosphatidylethanolamine (O-18:1_20:4) levels	Parkinson's disease	rs17703316	T	G	T	G	1.79E-01	-4.03E-02	5.04E-02	9.05E-01	FALSE	FALSE	13	76170694	3.93E-02	5.15E-01	TRUE	13	75596558	3.89E-02	0.97	7172	4.25E-06	TRUE	TRUE	482,730	21.18
Phosphatidylethanolamine (O-18:1_20:4) levels	Parkinson's disease	rs2277289	C	T	C	T	9.11E-02	2.23E-02	3.26E-01	6.81E-01	FALSE	FALSE	11	118182660	2.06E-02	5.56E-01	TRUE	11	118311945	1.78E-02	1.00	7172	3.31E-07	TRUE	TRUE	482,730	26.11
Phosphatidylethanolamine (O-18:1_20:4) levels	Parkinson's disease	rs2705007	G	C	G	C	-3.61E-01	-3.84E-02	9.87E-01	9.86E-01	TRUE	FALSE	7	106000968	1.16E-01	1.30E-01	TRUE	7	106360522	7.91E-02	0.89	7172	5.12E-06	TRUE	TRUE	482,730	20.82
Phosphatidylethanolamine (O-18:1_20:4) levels	Parkinson's disease	rs311416	T	C	T	C	-7.93E-02	3.54E-02	6.49E-01	3.50E-01	FALSE	FALSE	8	55110809	2.32E-02	8.95E-01	TRUE	8	54198249	1.76E-02	1.00	7172	6.58E-06	TRUE	TRUE	482,730	20.34

Phosphatidylethanolamine (O-18:1_20:4) levels	Parkinson's disease	rs35427954	C	T	C	T	-1.40E-01	-6.24E-02	8.70E-02	9.06E-01	FALSE	FALSE	16	76496994	2.97E-02	1.78E-02	TRUE	16	76463097	2.96E-02	1.00	7172	2.30E-06	TRUE	TRUE	482,730	22.36
Phosphatidylethanolamine (O-18:1_20:4) levels	Parkinson's disease	rs3820438	C	G	C	G	8.19E-02	5.55E-02	2.91E-01	2.27E-01	TRUE	FALSE	1	201924749	3.28E-02	4.53E-02	TRUE	1	201955621	1.84E-02	0.98	7172	8.42E-06	TRUE	TRUE	482,730	19.87
Phosphatidylethanolamine (O-18:1_20:4) levels	Parkinson's disease	rs6601694	A	G	A	G	8.75E-02	-2.82E-02	3.34E-01	7.30E-01	FALSE	FALSE	8	8248058	2.21E-02	6.94E-01	TRUE	8	8390548	1.78E-02	0.99	7172	8.85E-07	TRUE	TRUE	482,730	24.20
Phosphatidylethanolamine (O-18:1_20:4) levels	Parkinson's disease	rs72635955	T	C	T	C	1.26E-01	-1.33E-01	1.25E-01	8.96E-01	FALSE	FALSE	1	245146108	3.53E-02	7.87E-05	TRUE	1	244982806	2.51E-02	1.00	7172	5.16E-07	TRUE	TRUE	482,730	25.25
Phosphatidylethanolamine (O-18:2_18:1) levels	Parkinson's disease	rs11174276	A	G	A	G	8.31E-02	3.87E-02	3.18E-01	8.01E-01	FALSE	FALSE	12	62399624	2.84E-02	7.61E-01	TRUE	12	62005843	1.85E-02	0.99	6717	7.22E-06	TRUE	TRUE	482,730	20.17
Phosphatidylethanolamine (O-18:2_18:1) levels	Parkinson's disease	rs112243303	T	C	T	C	1.38E-01	6.83E-02	1.12E-01	9.35E-01	FALSE	FALSE	1	224380630	5.96E-02	6.00E-01	TRUE	1	224192928	2.77E-02	0.95	6717	5.86E-07	TRUE	TRUE	482,730	25.00
Phosphatidylethanolamine (O-18:2_18:1) levels	Parkinson's disease	rs112779958	C	T	C	T	-2.34E-01	2.72E-02	3.03E-02	9.63E-01	FALSE	FALSE	2	14455552	6.65E-02	1.66E-01	TRUE	2	14315428	5.16E-02	0.96	6717	6.07E-06	TRUE	TRUE	482,730	20.50
Phosphatidylethanolamine (O-18:2_18:1) levels	Parkinson's disease	rs116075306	G	C	G	C	-3.71E-01	-3.49E-02	1.09E-02	2.11E-02	TRUE	FALSE	1	241506146	8.48E-02	1.67E-01	TRUE	1	241342846	8.35E-02	0.93	6717	9.30E-06	TRUE	TRUE	482,730	19.68
Phosphatidylethanolamine (O-18:2_18:1) levels	Parkinson's disease	rs116140603	C	T	C	T	5.76E-01	-1.25E-01	4.50E-03	9.87E-01	FALSE	FALSE	5	179476450	9.68E-02	7.07E-01	TRUE	5	180049450	1.30E-01	0.97	6717	9.21E-06	TRUE	TRUE	482,730	19.70
Phosphatidylethanolamine (O-18:2_18:1) levels	Parkinson's disease	rs117049823	G	A	G	A	4.89E-01	-1.09E-01	6.47E-03	9.72E-01	FALSE	FALSE	9	2495519	6.76E-02	9.73E-01	TRUE	9	2495519	1.10E-01	0.99	6717	8.55E-06	TRUE	TRUE	482,730	19.84
Phosphatidylethanolamine (O-18:2_18:1) levels	Parkinson's disease	rs12496575	G	A	G	A	-8.97E-02	-7.10E-03	6.93E-01	3.22E-01	FALSE	FALSE	3	278270	2.39E-02	1.15E-01	TRUE	3	236587	1.87E-02	1.00	6717	1.65E-06	TRUE	TRUE	482,730	23.01
Phosphatidylethanolamine (O-18:2_18:1) levels	Parkinson's disease	rs13257713	T	C	T	C	-1.19E-01	-4.10E-03	1.39E-01	9.15E-01	FALSE	FALSE	8	113429636	4.19E-02	3.56E-02	TRUE	8	112417407	2.50E-02	0.99	6717	2.06E-06	TRUE	TRUE	482,730	22.57
Phosphatidylethanolamine (O-18:2_18:1) levels	Parkinson's disease	rs140484398	A	G	A	G	5.19E-01	9.48E-02	6.89E-03	9.91E-01	FALSE	FALSE	9	105430778	1.48E-01	2.82E-01	TRUE	9	102668496	1.06E-01	0.98	6717	1.02E-06	TRUE	TRUE	482,730	23.94
Phosphatidylethanolamine (O-18:2_18:1) levels	Parkinson's disease	rs148727465	A	G	A	G	2.09E-01	-1.14E-01	3.97E-02	9.83E-01	FALSE	FALSE	1	167150475	1.58E-01	3.27E-01	TRUE	1	167181238	4.63E-02	0.92	6717	6.67E-06	TRUE	TRUE	482,730	20.32
Phosphatidylethanolamine (O-18:2_18:1) levels	Parkinson's disease	rs150046733	C	T	C	T	-1.81E-01	2.44E-02	4.92E-02	9.46E-01	FALSE	FALSE	11	732354	4.31E-02	2.43E-01	TRUE	11	732354	4.06E-02	0.97	6717	8.64E-06	TRUE	TRUE	482,730	19.82
Phosphatidylethanolamine (O-18:2_18:1) levels	Parkinson's disease	rs17368528	T	C	T	C	1.61E-01	-3.18E-02	8.43E-02	8.74E-01	FALSE	FALSE	1	9324213	2.57E-02	6.66E-01	TRUE	1	9264154	3.09E-02	1.00	6717	2.04E-07	TRUE	TRUE	482,730	27.05
Phosphatidylethanolamine (O-18:2_18:1) levels	Parkinson's disease	rs1800588	T	C	T	C	9.53E-02	-3.30E-03	2.58E-01	7.75E-01	FALSE	FALSE	15	58723675	2.12E-02	5.73E-02	TRUE	15	58431476	2.00E-02	0.99	6717	1.94E-06	TRUE	TRUE	482,730	22.69
Phosphatidylethanolamine (O-18:2_18:1) levels	Parkinson's disease	rs2271489	G	C	G	C	-1.72E-01	-3.40E-02	8.15E-02	1.08E-01	TRUE	FALSE	3	39276659	2.97E-02	5.97E-01	TRUE	3	39235168	3.18E-02	0.99	6717	6.67E-08	TRUE	TRUE	482,730	29.22
Phosphatidylethanolamine (O-18:2_18:1) levels	Parkinson's disease	rs35253870	G	A	G	A	1.18E-01	3.20E-03	1.51E-01	8.33E-01	FALSE	FALSE	2	12372589	3.28E-02	3.49E-02	TRUE	2	12232463	2.45E-02	0.98	6717	1.39E-06	TRUE	TRUE	482,730	23.33
Phosphatidylethanolamine (O-18:2_18:1) levels	Parkinson's disease	rs3752651	C	T	C	T	1.15E-01	-2.73E-02	1.36E-01	7.94E-01	FALSE	FALSE	7	55229543	2.40E-02	5.93E-01	TRUE	7	55161850	2.53E-02	0.99	6717	5.07E-06	TRUE	TRUE	482,730	20.85
Phosphatidylethanolamine (O-18:2_18:1) levels	Parkinson's disease	rs4674246	T	C	T	C	1.02E-01	-4.13E-02	1.86E-01	8.71E-01	FALSE	FALSE	2	218940418	3.55E-02	6.11E-01	TRUE	2	218075695	2.27E-02	0.96	6717	7.63E-06	TRUE	TRUE	482,730	20.06
Phosphatidylethanolamine (O-18:2_18:1) levels	Parkinson's disease	rs6749797	A	G	A	G	-9.80E-02	1.10E-03	2.29E-01	7.93E-01	FALSE	FALSE	2	121156062	2.74E-02	1.43E-02	TRUE	2	120398486	2.05E-02	1.00	6717	1.74E-06	TRUE	TRUE	482,730	22.90
Phosphatidylethanolamine (O-18:2_18:1) levels	Parkinson's disease	rs79878108	T	C	T	C	-2.61E-01	-2.82E-02	2.91E-02	9.56E-01	FALSE	FALSE	1	245286218	5.67E-02	2.09E-01	TRUE	1	245122916	5.18E-02	0.98	6717	5.08E-07	TRUE	TRUE	482,730	25.28
Phosphatidylethanolamine (O-18:2_18:2) levels	Parkinson's disease	rs11870168	T	C	T	C	-8.25E-02	-2.22E-02	4.20E-01	4.66E-01	FALSE	FALSE	17	74477691	2.27E-02	4.86E-01	TRUE	17	76481609	1.83E-02	0.98	6337	6.62E-06	TRUE	TRUE	482,730	20.33
Phosphatidylethanolamine (O-18:2_18:2) levels	Parkinson's disease	rs11936543	G	A	G	A	-1.36E-01	5.67E-02	9.98E-02	9.62E-01	FALSE	FALSE	4	152191381	7.37E-02	3.54E-01	TRUE	4	151270229	3.02E-02	0.97	6337	6.52E-06	TRUE	TRUE	482,730	20.36
Phosphatidylethanolamine (O-18:2_18:2) levels	Parkinson's disease	rs181750334	T	A	T	A	-4.92E-01	3.30E-02	7.47E-03	1.37E-02	TRUE	FALSE	16	14739018	1.04E-01	1.24E-01	TRUE	16	14645161	1.10E-01	0.91	6337	8.28E-06	TRUE	TRUE	482,730	19.90
Phosphatidylethanolamine (O-18:2_18:2) levels	Parkinson's disease	rs2550882	G	C	G	C	-8.50E-02	7.90E-03	4.14E-01	3.63E-01	TRUE	FALSE	16	75549345	1.89E-02	1.70E-01	TRUE	16	75515447	1.80E-02	0.99	6337	2.36E-06	TRUE	TRUE	482,730	22.32
Phosphatidylethanolamine (O-18:2_18:2) levels	Parkinson's disease	rs2727270	T	C	T	C	1.77E-01	3.83E-02	2.58E-01	8.80E-01	FALSE	FALSE	11	61603237	2.62E-02	8.43E-01	TRUE	11	61835765	2.02E-02	1.00	6337	2.43E-18	TRUE	TRUE	482,730	76.76
Phosphatidylethanolamine (O-18:2_18:2) levels	Parkinson's disease	rs34683829	T	C	T	C	-1.04E-01	2.16E-02	2.06E-01	8.44E-01	FALSE	FALSE	2	217573445	3.15E-02	3.08E-01	TRUE	2	216708722	2.20E-02	0.98	6337	2.36E-06	TRUE	TRUE	482,730	22.32
Phosphatidylethanolamine (O-18:2_18:2) levels	Parkinson's disease	rs4603290	C	G	C	G	1.53E-01	-3.40E-03	9.86E-02	9.34E-02	TRUE	FALSE	11	15322753	4.05E-02	2.99E-02	TRUE	11	15301207	3.06E-02	0.95	6337	5.94E-07	TRUE	TRUE	482,730	24.98
Phosphatidylethanolamine (O-18:2_18:2) levels	Parkinson's disease	rs497849	T	C	T	C	-1.07E-01	7.65E-02	1.90E-01	7.73E-01	FALSE	FALSE	10	69753620	2.96E-02	4.88E-03	TRUE	10	67993863	2.35E-02	0.92	6337	5.60E-06	TRUE	TRUE	482,730	20.66
Phosphatidylethanolamine (O-18:2_18:2) levels	Parkinson's disease	rs72840788	A	G	A	G	9.49E-02	-9.10E-02	2.29E-01	7.84E-01	FALSE	FALSE	10	121415685	2.06E-02	4.99E-06	TRUE	10	119656173	2.13E-02	0.99	6337	8.98E-06	TRUE	TRUE	482,730	19.75
Phosphatidylethanolamine (O-18:2_18:2) levels	Parkinson's disease	rs7459809	T	A	T	A	-6.86E-01	-9.38E-02	4.48E-03	1.85E-02	TRUE	FALSE	8	140140565	1.02E-01	4.48E-01	TRUE	8	139128322	1.44E-01	0.88	6337	2.08E-06	TRUE	TRUE	482,730	22.56
Phosphatidylethanolamine (O-18:2_18:2) levels	Parkinson's disease	rs79087995	G	A	G	A	-2.71E-01	-1.57E-01	2.25E-02	9.82E-01	FALSE	FALSE	3	114176514	8.56E-02	3.38E-02	TRUE	3	114457667	6.12E-02	0.99	6337	9.55E-06	TRUE	TRUE	482,730	19.63
Phosphatidylethanolamine (O-18:2_18:2) levels	Parkinson's disease	rs79998109	T	C	T	C	5.40E-01	-3.06E-02	7.07E-03	9.82E-01	FALSE	FALSE	12	109508248	9.48E-02	1.27E-01	TRUE	12	109070443	1.14E-01	0.89	6337	2.24E-06	TRUE	TRUE	482,730	22.42
Phosphatidylethanolamine (O-18:2_20:4) levels	Parkinson's disease	rs1034755	T	C	T	C	8.04E-02	1.95E-02	3.19E-01	6.71E-01	FALSE	FALSE	7	8850785	2.32E-02	3.96E-01	TRUE	7	8811155	1.80E-02	0.98	7154	7.79E-06	TRUE	TRUE	482,730	20.01
Phosphatidylethanolamine (O-18:2_20:4) levels	Parkinson's disease	rs115069241	C	T	C	T	-2.47E-01	-1.09E-02	2.49E-02	9.56E-01	FALSE	FALSE	2	147524359	5.69E-02	7.19E-02	TRUE	2	146766791	5.44E-02	0.97	7154	5.54E-06	TRUE	TRUE	482,730	20.67
Phosphatidylethanolamine (O-18:2_20:4) levels	Parkinson's disease	rs115623452	A	G	A	G	-8.03E-01	-1.23E-01	2.75E-03	9.94E-01	FALSE	FALSE	3	105846629	2.90E-01	1.74E-01	TRUE	3	106127782	1.74E-01	0.92	7154	4.14E-06	TRUE	TRUE	482,730	21.23
Phosphatidylethanolamine (O-18:2_20:4) levels	Parkinson's disease	rs13280051	G	A	G	A	9.25E-02	-1.43E-02	2.47E-01	8.20E-01	FALSE	FALSE	8	8231691	2.60E-02	2.35E-01	TRUE	8	8374175	1.94E-02	1.00	7154	1.88E-06	TRUE	TRUE	482,730	22.75
Phosphatidylethanolamine (O-18:2_20:4) levels	Parkinson's disease	rs143745495	T	C	T	C	-1.04E-01	3.60E-03	1.72E-01	8.76E-01	FALSE	FALSE	1	119715178	3.01E-02	4.31E-02	TRUE	1	119172555	2.21E-02	1.00	7154	2.38E-06	TRUE	TRUE	482,730	22.30
Phosphatidylethanolamine (O-18:2_20:4) levels	Parkinson's disease	rs145522126	T	C	T	C	2.67E-01	1.67E-01	2.31E-02	9.89E-01	FALSE	FALSE	10	36472745	2.04E-01	3.82E-01	TRUE	10	36183817	5.94E-02	0.89	7154	6.81E-06	TRUE	TRUE	482,730	20.27
Phosphatidylethanolamine (O-18:2_20:4) levels	Parkinson's disease	rs174556	T	C	T	C	-1.83E-01	1.00E-04	3.80E-01	7.09E-01	FALSE	FALSE	11	61580635	1.85E-02	1.04E-03	TRUE	11	61813163	1.72E-02	1.00	7154	4.16E-26	TRUE	TRUE	482,730	112.58
Phosphatidylethanolamine (O-18:2_20:4) levels	Parkinson's disease	rs17703316	T	G	T	G	1.75E-01	-4.03E-02	5.04E-02	9.05E-01	FALSE	FALSE	13	76170694	3.93E-02	5.15E-01	TRUE	13	75596558	3.90E-02	0.97	7154	7.40E-06	TRUE	TRUE	482,730	20.11
Phosphatidylethanolamine (O-18:2_20:4) levels	Parkinson's disease	rs17864186	A	C	A	C	1.47E-01	-4.08E-02	7.43E-02	9.30E-01	FALSE	FALSE	7	126943656	4.48E-02	4.40E-01	TRUE	7	127303602	3.22E-02	0.98	7154	4.97E-06	TRUE	TRUE	482,730	20.88
Phosphatidylethanolamine (O-18:2_20:4) levels	Parkinson's disease	rs181750334	T	A	T	A	-5.37E-01	3.30E-02	7.47E-03	1.37E-02	TRUE	FALSE	16	14739018	1.04E-01	1.24E-01	TRUE	16	14645161	1.03E-01	0.91	7154	1.91E-07	TRUE	TRUE	482,730	27.18
Phosphatidylethanolamine (O-18:2_20:4) levels	Parkinson's disease	rs2323249	G	A	G	A	-2.41E-01	-1.55E-02	2.76E-02	9.45E-01	FALSE	FALSE	3	5516124	5.33E-02	1.13E-01	TRUE	3	5474437	5.39E-02	0.90	7154	8.02E-06	TRUE	TRUE	482,730	19.96
Phosphatidylethanolamine (O-18:2_20:4) levels	Parkinson's disease	rs3820438	C	G	C	G	8.89E-02	5.55E-02	2.91E-01	2.27E-01	TRUE	FALSE	1	201924749	3.28E-02	4.53E-02	TRUE	1	201955621	1.84E-02	0.98	7154	1.38E-06	TRUE	TRUE	482,730	23.35
Phosphatidylethanolamine (O-18:2_20:4) levels	Parkinson's disease	rs4877665	C	A	C	A	7.89E-02	2.26E-02	6.00E-01	5.44E-01	FALSE	FALSE	9	84236917	2.35E-02	4.72E-01	TRUE	9	81622002	1.76E-02	0.94	7154	7.61E-06	TRUE	TRUE	482,730	20.06
Phosphatidylethanolamine (O-18:2_20:4) levels	Parkinson's disease	rs55853808	G	A	G	A	1.11E-01	-2.96E-02	1.42E-01	8.48E-01	FALSE	FALSE	3	182053946	3.34E-02	4.25E-01	TRUE	3	182336158	2.51E-02	0.92	7154	9.77E-06	TRUE	TRUE	482,730	19.58
Phosphatidylethanolamine (O-18:2_20:4) levels	Parkinson's disease	rs61792775	T	C	T	C	-1.53E-01	-6.60E-03	7.30E-02	9.29E-01	FALSE	FALSE	3	131981244	4.61E-02	5.25E-02	TRUE	3	132262400	3.28E-02	0.97	7154	3.23E-06	TRUE	TRUE	482,730	21.71
Phosphatidylethanolamine (O-18:2_20:4) levels	Parkinson's disease	rs71366199	G	A	G	A	1.21E-01	-2.11E-02	1.48E-01	8.89E-01	FALSE	FALSE	17	15297187	3.83E-02	2.35E-01	TRUE	17	15393870	2.41E-02	0.95	7154	4.87E-07	TRUE	TRUE	482,730	25.36
Phosphatidylethanolamine (O-18:2_20:4) levels	Parkinson's disease	rs72627458	G	T	G	T	-2.31E-01	-4.84E-02	2.88E-02	9.71E-01	FALSE	FALSE	2	107424732	6.10E-02	3.69E-01	TRUE	2	106808276	5.11E-02	0.97	7154	6.48E-06	TRUE	TRUE	482,730	20.37
Phosphatidylethanolamine (O-18:2_20:4) levels	Parkinson's disease	rs77754835	A	G	A	G	1.03E-01	6.83E-02	1.56E-01	7.72E-01	FALSE	FALSE	8	70900782	2.73E-02	6.18E-03	TRUE	8	69988547	2.34E-02	0.99	7154	9.77E-06	TRUE	TRUE	482,730	19.58
Phosphatidylethanolamine (O-18:2_20:4) levels	Parkinson's disease	rs79337987	A	T	A	T	-2.76E-01	-1.54E-01	1.95E-02	1.23E-02	TRUE	FALSE	7	137839857	1.10E-01	7.90E-01	TRUE	7	138155111	6.08E-02	0.97	7154	5.74E-06	TRUE	TRUE	482,730	20.60
Phosphatidylethanolamine (O-18:2_20:4) levels	Parkinson's disease	rs80299509	A	C	A	C	-1.12E-01	-6.68E-02	1.28E-01	9.29E-01	FALSE	FALSE	16	75589413	3.59E-02	3.14E-02	TRUE	16	75555515	2.48E-02	1.00	7154	5.91E-06	TRUE	TRUE	482,730	20.55
Phosphatidylethanolamine (O-18:2_20:4) levels	Parkinson's disease	rs9564073	A	G	A	G	-1.93E-01	-7.65E-02	4.12E-02	9.74E-01	FALSE	FALSE	13	34693980	7.41E-02	5.20E-01	TRUE	13	34119843	4.33E-02	0.94	7154	8.13E-06	TRUE	TRUE	482,730	19.94
Phosphatidylinositol (16:0_18:1) levels	Parkinson's disease	rs10490343	A	T	A	T	-7.91E-02	9.60E-03	3.62E-01	3.04E-01	TRUE	FALSE	2	45845041	2.43E-02	1.59E-01	TRUE	2	45617902	1.74E-02	1.00	7157	5.90E-06	TRUE	TRUE	482,730	20.55
Phosphatidylinositol (16:0_18:1) levels	Parkinson's disease	rs11208007	T	A	T	A	-8.55E-02	3.60E-03	2.49E-01	3.20E-01	TRUE	FALSE	1	63176083	1.86E-02	7.13E-02	TRUE	1	62710412	1.92E-02	1.00	7157	8.47E-06	TRUE	TRUE	482,730	19.86
Phosphatidylinositol (16:0_18:1) levels	Parkinson's disease	rs117226992	G	C	G	C	7.34E-01	-4.81E-02	3.15E-03	1.10E-02	TRUE	FALSE	16	51347509	1.59E-01	1.18E-01	TRUE	16	51313598	1.59E-01	0.93	7157	3.78E-06	TRUE	TRUE	482,730	21.41
Phosphatidylinositol (16:0_18:1) levels	Parkinson's disease	rs13113877	A	C	A	C	-7.78E-02	-3.91E-02	3.78E-01	6.78E-01	FALSE	FALSE	4	59977257	2.38E-02	9.98E-01	TRUE	4	59111539	1.73E-02	0.99	7157	7.13E-06	TRUE	TRUE	482,730	20.19
Phosphatidylinositol (16:0_18:1) levels	Parkinson's disease	rs174556	T	C	T	C	9.97E-02	1.00E-04	3.80E-01	7.09E-01	FALSE	FALSE	11	61580635	1.85E-02	1.04E-03	TRUE	11	61813163	1.73E-02	1.00	7157	8.51E-09	TRUE	TRUE	482,730	33.23
Phosphatidylinositol (16:0_18:1) levels	Parkinson's disease	rs1918704	A	G	A	G	1.03E-01	-3.50E-02	8.40E-01	1.34E-01	FALSE	FALSE	2	10693327	3.42E-02	5.16E-01	TRUE	2	10553201	2.32E-02	0.97	7157	9.15E-06	TRUE	TRUE	482,730	19.71
Phosphatidylinositol (16:0_18:1) levels	Parkinson's disease	rs3103985	A	G	A	G	-1.19E-01	-2.71E-02	8.82E-01	1.33E-01	FALSE	FALSE	8	119742870	3.06E-02	4.25E-01	TRUE	8	118730631	2.66E-02	0.96	7157	8.20E-06	TRUE	TRUE	482,730	19.92
Phosphatidylinositol (16:0_18:1) levels	Parkinson's disease	rs75678679	T	C	T	C	2.43E-01	-9.30E-03	2.46E-02	9.80E-01	FALSE	FALSE	10	64318160	8.30E-02	4.04E-02	TRUE	10	62558401	5.48E-02	0.99	7157	9.53E-06	TRUE	TRUE	482,730	19.63
Phosphatidylinositol (16:0_18:1) levels	Parkinson's disease	rs76902173	G	C	G	C	-2.93E-01	2.54E-02	2.17E-02	1.92E-02	TRUE	FALSE	12	29153882	8.57E-02	1.15E-01	TRUE	12	29000949	5.87E-02	0.97	7157	5.81E-07	TRUE	TRUE	482,730	25.02
Phosphatidylinositol (16:0_18:1) levels	Parkinson's disease	rs79107687	G	A	G	A	-1.45E-01	-1.30E-01	8.19E-02	9.62E-01	FALSE	FALSE	4	80991966	6.62E-02	2.48E-02	TRUE	4	80070812	3.25E-02	0.89	7157	8.22E-06	TRUE	TRUE	482,730	19.91
Phosphatidylinositol (16:0_18:1) levels	Parkinson's disease	rs80310784	A	G	A	G	1.68E-01	-6.42E-02	6.00E-02	9.03E-01	FALSE	FALSE	10	54142641	4.13E-02	9.22E-01	TRUE	10	52382881	3.61E-02	0.92	7157	3.44E-06	TRUE	TRUE	482,730	21.59
Phosphatidylinositol (16:0_18:1) levels	Parkinson's disease	rs964184	C	G	C	G	-1.23E-01	-5.68E-02	8.49E-01	8.63E-01	TRUE	FALSE	11	116648917	2.43E-02	9.71E-03	TRUE	11	116778201	2.32E-02	1.00	7157	1.20E-07	TRUE	TRUE	482,730	28.07
Phosphatidylinositol (16:0_18:2) levels	Parkinson's disease	rs10466588	G	A	G	A	1.51E-01	-6.78E-02	9.58E-02	9.22E-01	FALSE	FALSE	11	116610249	3.17E-02	1.62E-02	TRUE	11	116739533	2.85E-02	1.00	6998	1.11E-07	TRUE	TRUE	482,730	28.23
Phosphatidylinositol (16:0_18:2) levels	Parkinson's disease	rs141824100	T	C	T	C	-1.85E-01	-1.09E-01	5.05E-02	9.72E-01	FALSE	FALSE	16	89052208	7.18E-02	8.82E-01	TRUE	16	88985800	3.87E-02	0.96	6998	1.93E-06	TRUE	TRUE	482,730	22.71
Phosphatidylinositol (16:0_18:2) levels	Parkinson's disease	rs143705793	T	C	T	C	2.44E-01	-1.15E-02	2.41E-02	9.82E-01	FALSE	FALSE	8	176933	9.08E-02	4.63E-02	TRUE	8	226933	5.48E-02	0.98	6998	8.49E-06	TRUE	TRUE	482,730	19.85
Phosphatidylinositol (16:0_18:2) levels	Parkinson's disease	rs17427389	A	G	A	G	1.08E-01	-7.30E-03	1.91E-01	8.20E-01	FALSE	FALSE	6	151163115	2.19E-02	1.32E-01	TRUE	6	150841979	2.15E-02	1.00	6998	4.96E-07	TRUE	TRUE	482,730	25.33
Phosphatidylinositol (16:0_18:2) levels	Parkinson's disease	rs174549	A	G	A	G	1.64E-01	5.00E-04	3.82E-01	7.07E-01	FALSE	FALSE	11	61571382	1.85E-02	1.03E-02	TRUE	11	61803910	1.74E-02	1.00	6998	5.75E-21	TRUE	TRUE	482,730	88.81
Phosphatidylinositol (16:0_18:2) levels	Parkinson's disease	rs1748199	C	T	C	T	-1.13E-01	1.00E-03	2.62E-01	6.73E-01	FALSE	FALSE	1	63057136	1.79E-02	1.94E-02	TRUE	1	62591465	1.91E-02	1.00	6998	3.20E-09	TRUE	TRUE	482,730	35.15
Phosphatidylinositol (16:0_18:2) levels	Parkinson's disease	rs184003290	C	T	C	T	6.10E-01	-1.95E-01	4.59E-03	9.89E-01	FALSE	FALSE	13	26528130	1.52E-01	6.99E-01	TRUE	13	25953992	1.36E-01	0.88	6998	7.26E-06	TRUE	TRUE	482,730	20.15
Phosphatidylinositol (16:0_18:2) levels	Parkinson's disease	rs28675901	C	T	C	T	1.24E-01	6.79E-02	1.28E-01	8.82E-01	FALSE	FALSE	4	109760367	3.48E-02	2.55E-02	TRUE	4	108839211	2.55E-02	0.98	6998	1.11E-06	TRUE	TRUE	482,730	23.77
Phosphatidylinositol (16:0_18:2) levels	Parkinson's disease	rs34777947	C	T	C	T	1.03E-01	-2.55E-02	1.89E-01	7.34E-01	FALSE	FALSE	7	105223880	2.59E-02	4.87E-01	TRUE	7	105583433	2.16E-02	0.97	6998	1.99E-06	TRUE	TRUE	482,730	22.64
Phosphatidylinositol (16:0_18:2) levels	Parkinson's disease	rs4665972	C	T	C	T	-8.90E-02	-7.12E-02	6.20E-01	4.20E-01	FALSE	FALSE	2	27598097	1.79E-02	3.48E-05	TRUE	2	27375230	1.74E-02	1.00	6998	3.19E-07	TRUE	TRUE	482,730	26.18
Phosphatidylinositol (16:0_18:2) levels	Parkinson's disease	rs57107736	T	C	T	C	1.15E-01	4.35E-02	1.41E-01	8.27E-01	FALSE	FALSE	16	76661694	2.97E-02	8.46E-01	TRUE	16	76627797	2.46E-02	0.99	6998	2.94E-06	TRUE	TRUE	482,730	21.89
Phosphatidylinositol (16:0_18:2) levels	Parkinson's disease	rs73127058	A	G	A	G	-3.13E-01	8.65E-02	1.65E-02	9.60E-01	FALSE	FALSE	5	77131170	6.07E-02	8.13E-01	TRUE	5	77835346	6.83E-02	0.94	6998	4.47E-06	TRUE	TRUE	482,730	21.08
Phosphatidylinositol (16:0_18:2) levels	Parkinson's disease	rs9344829	T	C	T	C	8.59E-02	-2.70E-03	3.73E-01	6.88E-01	FALSE	FALSE	6	64887066	1.90E-02	5.16E-02	TRUE	6	64177173	1.75E-02	0.99	6998	9.08E-07	TRUE	TRUE	482,730	24.15
Phosphatidylinositol (16:0_18:2) levels	Parkinson's disease	rs9666759	G	T	G	T	-7.76E-02	-1.82E-02	4.20E-01	5.02E-01	FALSE	FALSE	11	11283618	2.23E-02	3.84E-01	TRUE	11	11262071	1.71E-02	0.99	6998	5.86E-06	TRUE	TRUE	482,730	20.56
Phosphatidylinositol (16:0_20:4) levels	Parkinson's disease	rs111243657	C	T	C	T	3.19E-01	-8.26E-02	1.77E-02	9.75E-01	FALSE	FALSE	1	239494391	7.13E-02	6.08E-01	TRUE	1	239331091	7.17E-02	0.98	5519	8.88E-06	TRUE	TRUE	482,730	19.78
Phosphatidylinositol (16:0_20:4) levels	Parkinson's disease	rs112184984	T	C	T	C	2.02E-01	-1.30E-02	5.27E-02	9.22E-01	FALSE	FALSE	11	1690272	3.62E-02	1.43E-01	TRUE	11	1669042	4.42E-02	0.96	5519	5.26E-06	TRUE	TRUE	482,730	20.78
Phosphatidylinositol (16:0_20:4) levels	Parkinson's disease	rs114728714	A	G	A	G	2.25E-01	1.92E-02	4.13E-02	9.77E-01	FALSE	FALSE	2	173045072	8.72E-02	8.32E-02	TRUE	2	172180344	4.94E-02	0.94	5519	5.35E-06	TRUE	TRUE	482,730	20.75
Phosphatidylinositol (16:0_20:4) levels	Parkinson's disease	rs115609318	T	C	T	C	-3.97E-01	-1.36E-01	1.22E-02	9.74E-01	FALSE	FALSE	2	23620163	7.32E-02	3.15E-02	TRUE	2	23397292	8.87E-02	0.95	5519	7.63E-06	TRUE	TRUE	482,730	20.06
Phosphatidylinositol (16:0_20:4) levels	Parkinson's disease	rs12494536	G	C	G	C	9.12E-02	-3.15E-02	3.40E-01	2.41E-01	TRUE	FALSE	3	27267046	2.41E-02	7.17E-01	TRUE	3	27225555	2.03E-02	0.97	5519	7.09E-06	TRUE	TRUE	482,730	20.21
Phosphatidylinositol (16:0_20:4) levels	Parkinson's disease	rs12545907	A	G	A	G	1.06E-01	4.45E-02	2.05E-01	8.67E-01	FALSE	FALSE	8	40426103	2.97E-02	8.71E-01	TRUE	8	40568584	2.40E-02	0.98	5519	9.10E-06	TRUE	TRUE	482,730	19.73
Phosphatidylinositol (16:0_20:4) levels	Parkinson's disease	rs1260326	C	T	C	T	-1.10E-01	-6.78E-02	6.51E-01	4.20E-01	FALSE	FALSE	2	27730940	1.72E-02	4.04E-05	TRUE	2	27508073	1.99E-02	1.00	5519	3.99E-08	TRUE	TRUE	482,730	30.24
Phosphatidylinositol (16:0_20:4) levels	Parkinson's disease	rs141039423	A	G	A	G	-3.98E-01	-8.34E-02	1.26E-02	9.89E-01	FALSE	FALSE	6	37272860	1.56E-01	2.27E-01	TRUE	6	37305084	8.77E-02	0.95	5519	5.75E-06	TRUE	TRUE	482,730	20.61
Phosphatidylinositol (16:0_20:4) levels	Parkinson's disease	rs144018078	G	C	G	C	2.87E-01	-3.19E-02	2.61E-02	1.74E-02	TRUE	FALSE	6	116696631	7.21E-02	1.82E-01	TRUE	6	116375468	6.13E-02	0.99	5519	3.03E-06	TRUE	TRUE	482,730	21.84
Phosphatidylinositol (16:0_20:4) levels	Parkinson's disease	rs148590777	A	G	A	G	3.11E-01	2.09E-02	2.02E-02	9.74E-01	FALSE	FALSE	16	31923102	5.60E-02	1.49E-01	TRUE	16	31911781	6.96E-02	0.98	5519	8.04E-06	TRUE	TRUE	482,730	19.96
Phosphatidylinositol (16:0_20:4) levels	Parkinson's disease	rs149878915	A	G	A	G	2.40E-01	-2.61E-02	4.00E-02	9.81E-01	FALSE	FALSE	8	491839	9.43E-02	1.07E-01	TRUE	8	541839	4.83E-02	0.99	5519	6.91E-07	TRUE	TRUE	482,730	24.69
Phosphatidylinositol (16:0_20:4) levels	Parkinson's disease	rs174537	T	G	T	G	-1.36E-01	-2.50E-03	4.08E-01	6.71E-01	FALSE	FALSE	11	61552680	1.79E-02	5.23E-02	TRUE	11	61785208	1.94E-02	1.00	5519	2.51E-12	TRUE	TRUE	482,730	49.26
Phosphatidylinositol (16:0_20:4) levels	Parkinson's disease	rs4759322	G	A	G	A	-9.61E-02	-4.06E-02	4.00E-01	6.50E-01	FALSE	FALSE	12	54465664	2.31E-02	3.94E-02	TRUE	12	54071880	1.94E-02	0.99	5519	7.70E-07	TRUE	TRUE	482,730	24.48
Phosphatidylinositol (16:0_20:4) levels	Parkinson's disease	rs6884150	G	A	G	A	-9.47E-02	-1.90E-02	2.98E-01	7.07E-01	FALSE	FALSE	5	1767774	2.47E-02	3.54E-01	TRUE	5	1767659	2.10E-02	0.98	5519	6.88E-06	TRUE	TRUE	482,730	20.26
Phosphatidylinositol (16:0_20:4) levels	Parkinson's disease	rs73197087	C	A	C	A	-1.62E-01	2.44E-02	9.35E-02	9.23E-01	FALSE	FALSE	3	108601654	3.74E-02	2.90E-01	TRUE	3	108882807	3.21E-02	0.98	5519	4.68E-07	TRUE	TRUE	482,730	25.45
Phosphatidylinositol (16:0_20:4) levels	Parkinson's disease	rs7562707	G	A	G	A	-9.97E-02	-1.25E-02	7.54E-01	2.55E-01	FALSE	FALSE	2	206827385	2.04E-02	2.68E-01	TRUE	2	205962661	2.19E-02	1.00	5519	5.51E-06	TRUE	TRUE	482,730	20.69
Phosphatidylinositol (16:0_20:4) levels	Parkinson's disease	rs76609522	T	C	T	C	3.10E-01	-1.22E-01	2.32E-02	9.77E-01	FALSE	FALSE	12	6247923	8.14E-02	8.72E-01	TRUE	12	6138757	6.60E-02	0.91	5519	2.67E-06	TRUE	TRUE	482,730	22.09
Phosphatidylinositol (16:0_20:4) levels	Parkinson's disease	rs7782358	G	A	G	A	-9.79E-02	-6.70E-03	5.93E-01	4.00E-01	FALSE	FALSE	7	67820	2.96E-02	8.49E-02	TRUE	7	67820	2.02E-02	0.93	5519	1.25E-06	TRUE	TRUE	482,730	23.54
Phosphatidylinositol (16:0_20:4) levels	Parkinson's disease	rs9653945	A	G	A	G	-1.11E-01	-4.67E-02	3.64E-01	6.46E-01	FALSE	FALSE	3	142660706	2.32E-02	2.21E-02	TRUE	3	142941864	1.99E-02	1.00	5519	2.72E-08	TRUE	TRUE	482,730	30.99
Phosphatidylinositol (18:0_18:1) levels	Parkinson's disease	rs10490343	A	T	A	T	-7.72E-02	9.60E-03	3.62E-01	3.04E-01	TRUE	FALSE	2	45845041	2.43E-02	1.59E-01	TRUE	2	45617902	1.74E-02	1.00	7162	9.45E-06	TRUE	TRUE	482,730	19.65
Phosphatidylinositol (18:0_18:1) levels	Parkinson's disease	rs10889352	C	T	C	T	-1.60E-01	4.00E-04	2.62E-01	6.75E-01	FALSE	FALSE	1	63099023	1.80E-02	6.92E-03	TRUE	1	62633352	1.88E-02	1.00	7162	2.22E-17	TRUE	TRUE	482,730	72.30
Phosphatidylinositol (18:0_18:1) levels	Parkinson's disease	rs115510074	A	G	A	G	-2.10E-01	-8.08E-02	3.39E-02	9.81E-01	FALSE	FALSE	2	142724921	8.98E-02	4.34E-01	TRUE	2	141967352	4.67E-02	0.96	7162	7.06E-06	TRUE	TRUE	482,730	20.20
Phosphatidylinositol (18:0_18:1) levels	Parkinson's disease	rs11783515	G	A	G	A	-8.81E-02	-4.80E-03	6.26E-01	3.30E-01	FALSE	FALSE	8	59320389	2.28E-02	7.84E-02	TRUE	8	58407830	1.72E-02	1.00	7162	3.02E-07	TRUE	TRUE	482,730	26.29
Phosphatidylinositol (18:0_18:1) levels	Parkinson's disease	rs12122878	T	C	T	C	-8.56E-02	-2.16E-02	2.89E-01	7.35E-01	FALSE	FALSE	1	189896505	3.04E-02	3.21E-01	TRUE	1	189927375	1.86E-02	0.98	7162	3.98E-06	TRUE	TRUE	482,730	21.31
Phosphatidylinositol (18:0_18:1) levels	Parkinson's disease	rs12638256	G	A	G	A	1.60E-01	-3.66E-02	6.32E-01	4.63E-01	FALSE	FALSE	3	142655290	1.99E-02	3.29E-02	TRUE	3	142936448	1.74E-02	0.99	7162	4.88E-20	TRUE	TRUE	482,730	84.51
Phosphatidylinositol (18:0_18:1) levels	Parkinson's disease	rs1340414	G	A	G	A	8.30E-02	-3.50E-02	6.46E-01	3.80E-01	FALSE	FALSE	1	102946342	2.28E-02	9.04E-01	TRUE	1	102480786	1.75E-02	1.00	7162	2.24E-06	TRUE	TRUE	482,730	22.41
Phosphatidylinositol (18:0_18:1) levels	Parkinson's disease	rs1560063	G	A	G	A	8.23E-02	-1.12E-02	3.28E-01	7.18E-01	FALSE	FALSE	5	5210050	2.88E-02	1.56E-01	TRUE	5	5209937	1.79E-02	1.00	7162	4.51E-06	TRUE	TRUE	482,730	21.07
Phosphatidylinositol (18:0_18:1) levels	Parkinson's disease	rs174556	T	C	T	C	1.35E-01	1.00E-04	3.80E-01	7.09E-01	FALSE	FALSE	11	61580635	1.85E-02	1.04E-03	TRUE	11	61813163	1.73E-02	1.00	7162	5.98E-15	TRUE	TRUE	482,730	61.17
Phosphatidylinositol (18:0_18:1) levels	Parkinson's disease	rs183478874	A	C	A	C	-2.48E-01	-9.29E-02	2.35E-02	9.69E-01	FALSE	FALSE	5	176425594	7.33E-02	6.88E-01	TRUE	5	176998593	5.60E-02	0.95	7162	9.41E-06	TRUE	TRUE	482,730	19.65
Phosphatidylinositol (18:0_18:1) levels	Parkinson's disease	rs4846841	G	A	G	A	9.09E-02	7.00E-04	7.47E-01	2.07E-01	FALSE	FALSE	1	230302661	2.34E-02	1.06E-02	TRUE	1	230166915	1.90E-02	1.00	7162	1.85E-06	TRUE	TRUE	482,730	22.78
Phosphatidylinositol (18:0_18:1) levels	Parkinson's disease	rs67835422	T	C	T	C	-8.83E-02	-5.19E-02	2.77E-01	6.80E-01	FALSE	FALSE	4	72743636	2.37E-02	1.43E-02	TRUE	4	71877919	1.84E-02	1.00	7162	1.72E-06	TRUE	TRUE	482,730	22.92
Phosphatidylinositol (18:0_18:1) levels	Parkinson's disease	rs6813524	G	A	G	A	-7.66E-02	-1.47E-02	3.92E-01	6.36E-01	FALSE	FALSE	4	27127678	2.40E-02	2.67E-01	TRUE	4	27126056	1.73E-02	0.96	7162	9.64E-06	TRUE	TRUE	482,730	19.61
Phosphatidylinositol (18:0_18:1) levels	Parkinson's disease	rs7122803	G	C	G	C	-1.16E-01	-6.31E-02	8.60E-01	8.56E-01	TRUE	FALSE	11	107754336	3.20E-02	2.43E-02	TRUE	11	107883610	2.39E-02	1.00	7162	1.18E-06	TRUE	TRUE	482,730	23.65
Phosphatidylinositol (18:0_18:1) levels	Parkinson's disease	rs7742350	T	C	T	C	-1.90E-01	-7.30E-03	4.42E-02	9.37E-01	FALSE	FALSE	6	14804049	4.91E-02	5.47E-02	TRUE	6	14803818	4.15E-02	0.96	7162	4.85E-06	TRUE	TRUE	482,730	20.93

Phosphatidylinositol (18:0_18:1) levels	Parkinson's disease	rs964184	C	G	C	G	-1.95E-01	-5.68E-02	8.49E-01	8.63E-01	TRUE	FALSE	11	116648917	2.43E-02	9.71E-03	TRUE	11	116778201	2.31E-02	1.00	7162	3.67E-17	TRUE	TRUE	482,730	71.30
Phosphatidylinositol (18:0_18:2) levels	Parkinson's disease	rs112713750	T	C	T	C	-2.67E-01	-9.31E-02	2.05E-02	9.82E-01	FALSE	FALSE	7	134890075	8.33E-02	5.79E-01	TRUE	7	135205323	5.83E-02	1.00	7170	4.58E-06	TRUE	TRUE	482,730	21.04
Phosphatidylinositol (18:0_18:2) levels	Parkinson's disease	rs114488287	A	T	A	T	-2.63E-01	5.62E-02	2.20E-02	1.63E-02	TRUE	FALSE	8	84922868	8.88E-02	2.78E-01	TRUE	8	84010633	5.71E-02	0.97	7170	4.11E-06	TRUE	TRUE	482,730	21.24
Phosphatidylinositol (18:0_18:2) levels	Parkinson's disease	rs11780222	C	A	C	A	7.94E-02	-2.03E-02	3.18E-01	7.41E-01	FALSE	FALSE	8	67205241	1.95E-02	5.25E-01	TRUE	8	66293006	1.80E-02	1.00	7170	9.98E-06	TRUE	TRUE	482,730	19.54
Phosphatidylinositol (18:0_18:2) levels	Parkinson's disease	rs12147624	A	G	A	G	1.35E-01	3.77E-02	8.24E-02	9.17E-01	FALSE	FALSE	14	74258299	3.30E-02	5.96E-01	TRUE	14	73791596	3.05E-02	1.00	7170	9.03E-06	TRUE	TRUE	482,730	19.73
Phosphatidylinositol (18:0_18:2) levels	Parkinson's disease	rs139001006	C	T	C	T	7.38E-01	1.94E-01	2.78E-03	9.90E-01	FALSE	FALSE	6	160600278	1.32E-01	8.51E-01	TRUE	6	160179246	1.63E-01	0.92	7170	6.26E-06	TRUE	TRUE	482,730	20.43
Phosphatidylinositol (18:0_18:2) levels	Parkinson's disease	rs146893850	T	G	T	G	-3.51E-01	-2.54E-02	1.20E-02	9.78E-01	FALSE	FALSE	4	134148806	8.31E-02	1.19E-01	TRUE	4	133227651	7.88E-02	0.93	7170	8.84E-06	TRUE	TRUE	482,730	19.78
Phosphatidylinositol (18:0_18:2) levels	Parkinson's disease	rs150684478	T	G	T	G	3.08E-01	2.79E-01	2.40E-02	9.88E-01	FALSE	FALSE	11	61312726	1.87E-01	8.68E-01	TRUE	11	61545254	5.72E-02	0.90	7170	7.46E-08	TRUE	TRUE	482,730	29.00
Phosphatidylinositol (18:0_18:2) levels	Parkinson's disease	rs174566	G	A	G	A	2.92E-01	-5.70E-03	4.08E-01	6.64E-01	FALSE	FALSE	11	61592362	1.78E-02	1.24E-01	TRUE	11	61824890	1.67E-02	1.00	7170	3.90E-67	TRUE	TRUE	482,730	306.06
Phosphatidylinositol (18:0_18:2) levels	Parkinson's disease	rs1748199	C	T	C	T	-1.87E-01	1.00E-03	2.62E-01	6.73E-01	FALSE	FALSE	1	63057136	1.79E-02	1.94E-02	TRUE	1	62591465	1.87E-02	1.00	7170	1.99E-23	TRUE	TRUE	482,730	100.17
Phosphatidylinositol (18:0_18:2) levels	Parkinson's disease	rs184003290	C	T	C	T	6.08E-01	-1.95E-01	4.59E-03	9.89E-01	FALSE	FALSE	13	26528130	1.52E-01	6.99E-01	TRUE	13	25953992	1.35E-01	0.88	7170	7.25E-06	TRUE	TRUE	482,730	20.15
Phosphatidylinositol (18:0_18:2) levels	Parkinson's disease	rs2081687	C	T	C	T	-7.98E-02	-1.19E-02	6.18E-01	3.44E-01	FALSE	FALSE	8	59388565	2.03E-02	2.55E-01	TRUE	8	58476006	1.70E-02	1.00	7170	2.67E-06	TRUE	TRUE	482,730	22.07
Phosphatidylinositol (18:0_18:2) levels	Parkinson's disease	rs2713348	G	A	G	A	8.06E-02	1.80E-03	6.98E-01	2.66E-01	FALSE	FALSE	7	9510700	2.55E-02	2.49E-02	TRUE	7	9471070	1.82E-02	1.00	7170	9.26E-06	TRUE	TRUE	482,730	19.69
Phosphatidylinositol (18:0_18:2) levels	Parkinson's disease	rs34372369	A	G	A	G	-1.32E-01	4.23E-02	8.96E-02	9.46E-01	FALSE	FALSE	7	143092269	3.80E-02	5.76E-01	TRUE	7	143395176	2.91E-02	1.00	7170	5.79E-06	TRUE	TRUE	482,730	20.58
Phosphatidylinositol (18:0_18:2) levels	Parkinson's disease	rs34777947	C	T	C	T	1.02E-01	-2.55E-02	1.89E-01	7.34E-01	FALSE	FALSE	7	105223880	2.59E-02	4.87E-01	TRUE	7	105583433	2.13E-02	0.97	7170	1.74E-06	TRUE	TRUE	482,730	22.90
Phosphatidylinositol (18:0_18:2) levels	Parkinson's disease	rs41429348	T	C	T	C	1.83E-01	4.04E-02	4.86E-02	9.63E-01	FALSE	FALSE	15	74220000	5.36E-02	3.45E-01	TRUE	15	73927659	3.86E-02	0.99	7170	2.29E-06	TRUE	TRUE	482,730	22.37
Phosphatidylinositol (18:0_18:2) levels	Parkinson's disease	rs62039480	A	G	A	G	8.54E-02	1.69E-02	3.32E-01	6.66E-01	FALSE	FALSE	16	15137450	1.83E-02	4.52E-01	TRUE	16	15043593	1.77E-02	1.00	7170	1.50E-06	TRUE	TRUE	482,730	23.19
Phosphatidylinositol (18:0_18:2) levels	Parkinson's disease	rs72676136	G	A	G	A	-2.84E-01	1.68E-02	1.84E-02	9.67E-01	FALSE	FALSE	4	116513438	6.37E-02	1.01E-01	TRUE	4	115592282	6.27E-02	0.98	7170	6.00E-06	TRUE	TRUE	482,730	20.52
Phosphatidylinositol (18:0_18:2) levels	Parkinson's disease	rs72830461	C	T	C	T	6.38E-01	-1.05E-01	4.56E-03	9.72E-01	FALSE	FALSE	6	10040579	7.10E-02	8.59E-01	TRUE	6	10040346	1.33E-01	0.88	7170	1.56E-06	TRUE	TRUE	482,730	23.11
Phosphatidylinositol (18:0_18:2) levels	Parkinson's disease	rs73107473	A	G	A	G	9.44E-02	-1.46E-02	1.97E-01	7.81E-01	FALSE	FALSE	7	44577622	2.56E-02	2.44E-01	TRUE	7	44538023	2.10E-02	0.99	7170	6.83E-06	TRUE	TRUE	482,730	20.27
Phosphatidylinositol (18:0_18:2) levels	Parkinson's disease	rs7561644	T	G	T	G	8.62E-02	8.30E-03	4.62E-01	6.44E-01	FALSE	FALSE	2	79011900	2.36E-02	1.40E-01	TRUE	2	78784774	1.69E-02	0.99	7170	3.63E-07	TRUE	TRUE	482,730	25.93
Phosphatidylinositol (18:0_18:2) levels	Parkinson's disease	rs77470505	G	T	G	T	3.11E-01	-1.86E-01	1.79E-02	9.87E-01	FALSE	FALSE	11	61226469	1.48E-01	6.82E-01	TRUE	11	61458997	6.32E-02	1.00	7170	8.70E-07	TRUE	TRUE	482,730	24.24
Phosphatidylinositol (18:0_18:2) levels	Parkinson's disease	rs780093	C	T	C	T	-9.09E-02	-7.14E-02	6.46E-01	4.12E-01	FALSE	FALSE	2	27742603	1.73E-02	1.84E-05	TRUE	2	27519736	1.73E-02	1.00	7170	1.52E-07	TRUE	TRUE	482,730	27.62
Phosphatidylinositol (18:0_18:2) levels	Parkinson's disease	rs915894	G	T	G	T	-8.65E-02	1.30E-03	3.26E-01	6.25E-01	FALSE	FALSE	6	32190390	1.96E-02	2.40E-02	TRUE	6	32222613	1.77E-02	0.99	7170	1.07E-06	TRUE	TRUE	482,730	23.84
Phosphatidylinositol (18:0_18:2) levels	Parkinson's disease	rs9551866	A	G	A	G	-8.38E-02	2.36E-02	3.02E-01	6.50E-01	FALSE	FALSE	13	30769735	2.81E-02	3.96E-01	TRUE	13	30195598	1.84E-02	0.97	7170	5.27E-06	TRUE	TRUE	482,730	20.76
Phosphatidylinositol (18:0_18:2) levels	Parkinson's disease	rs964184	C	G	C	G	-2.39E-01	-5.68E-02	8.49E-01	8.63E-01	TRUE	FALSE	11	116648917	2.43E-02	9.71E-03	TRUE	11	116778201	2.30E-02	1.00	7170	5.59E-25	TRUE	TRUE	482,730	107.35
Phosphatidylinositol (18:0_18:2) levels	Parkinson's disease	rs976947	A	G	A	G	-7.78E-02	4.83E-02	6.35E-01	3.33E-01	FALSE	FALSE	11	115463786	2.36E-02	2.03E-02	TRUE	11	115593068	1.74E-02	1.00	7170	8.04E-06	TRUE	TRUE	482,730	19.96
Phosphatidylinositol (18:0_20:3) levels	Parkinson's disease	rs10897211	A	G	A	G	9.94E-02	-8.80E-03	3.35E-01	6.26E-01	FALSE	FALSE	11	61823576	2.27E-02	1.55E-01	TRUE	11	62056104	1.83E-02	0.99	6784	5.84E-08	TRUE	TRUE	482,730	29.48
Phosphatidylinositol (18:0_20:3) levels	Parkinson's disease	rs11769084	A	G	A	G	1.30E-01	-1.40E-01	1.05E-01	9.58E-01	FALSE	FALSE	7	71828688	6.17E-02	1.18E-02	TRUE	7	72363703	2.81E-02	0.99	6784	3.96E-06	TRUE	TRUE	482,730	21.32
Phosphatidylinositol (18:0_20:3) levels	Parkinson's disease	rs117761170	G	A	G	A	-1.98E-01	1.46E-02	3.95E-02	8.81E-01	FALSE	FALSE	9	34672500	3.16E-02	1.92E-01	TRUE	9	34672503	4.41E-02	0.99	6784	7.29E-06	TRUE	TRUE	482,730	20.15
Phosphatidylinositol (18:0_20:3) levels	Parkinson's disease	rs12598816	T	G	T	G	-9.05E-02	-2.23E-02	2.68E-01	7.22E-01	FALSE	FALSE	16	80287378	2.50E-02	4.31E-01	TRUE	16	80253481	1.94E-02	1.00	6784	3.12E-06	TRUE	TRUE	482,730	21.78
Phosphatidylinositol (18:0_20:3) levels	Parkinson's disease	rs1260326	C	T	C	T	-9.00E-02	-6.78E-02	6.51E-01	4.20E-01	FALSE	FALSE	2	27730940	1.72E-02	4.04E-05	TRUE	2	27508073	1.80E-02	1.00	6784	5.42E-07	TRUE	TRUE	482,730	25.15
Phosphatidylinositol (18:0_20:3) levels	Parkinson's disease	rs13147779	C	T	C	T	-1.04E-01	-9.50E-03	1.89E-01	7.55E-01	FALSE	FALSE	4	170208169	2.61E-02	1.46E-01	TRUE	4	169287018	2.20E-02	0.99	6784	2.03E-06	TRUE	TRUE	482,730	22.61
Phosphatidylinositol (18:0_20:3) levels	Parkinson's disease	rs145740269	C	T	C	T	-1.83E-01	1.14E-01	4.93E-02	9.82E-01	FALSE	FALSE	17	51274602	9.53E-02	6.33E-01	TRUE	17	53197241	4.03E-02	0.97	6784	6.00E-06	TRUE	TRUE	482,730	20.52
Phosphatidylinositol (18:0_20:3) levels	Parkinson's disease	rs16916055	C	T	C	T	4.62E-01	8.78E-02	8.81E-03	9.88E-01	FALSE	FALSE	11	91160029	1.27E-01	3.11E-01	TRUE	11	91426863	9.46E-02	0.97	6784	1.10E-06	TRUE	TRUE	482,730	23.79
Phosphatidylinositol (18:0_20:3) levels	Parkinson's disease	rs17085148	C	T	C	T	-1.93E-01	-3.45E-02	5.01E-02	9.56E-01	FALSE	FALSE	9	84501226	6.12E-02	2.42E-01	TRUE	9	81886311	4.29E-02	0.82	6784	6.95E-06	TRUE	TRUE	482,730	20.24
Phosphatidylinositol (18:0_20:3) levels	Parkinson's disease	rs1937711	G	C	G	C	9.08E-02	-4.76E-02	6.51E-01	6.22E-01	TRUE	FALSE	10	53615279	2.50E-02	2.85E-02	TRUE	10	51855519	1.90E-02	0.89	6784	1.84E-06	TRUE	TRUE	482,730	22.79
Phosphatidylinositol (18:0_20:3) levels	Parkinson's disease	rs2819082	C	T	C	T	-8.81E-02	1.31E-02	2.95E-01	6.85E-01	FALSE	FALSE	6	156700302	2.45E-02	2.28E-01	TRUE	6	156379168	1.88E-02	0.98	6784	2.97E-06	TRUE	TRUE	482,730	21.87
Phosphatidylinositol (18:0_20:3) levels	Parkinson's disease	rs28456	G	A	G	A	3.49E-01	-6.00E-04	3.84E-01	7.01E-01	FALSE	FALSE	11	61589481	1.84E-02	1.12E-02	TRUE	11	61822009	1.73E-02	1.00	6784	1.70E-88	TRUE	TRUE	482,730	409.65
Phosphatidylinositol (18:0_20:3) levels	Parkinson's disease	rs28514538	G	A	G	A	-9.25E-02	-2.20E-03	2.46E-01	7.32E-01	FALSE	FALSE	8	59398432	2.51E-02	3.20E-02	TRUE	8	58485873	2.00E-02	1.00	6784	3.67E-06	TRUE	TRUE	482,730	21.46
Phosphatidylinositol (18:0_20:3) levels	Parkinson's disease	rs319552	C	G	C	G	9.04E-02	-6.00E-03	2.55E-01	2.82E-01	TRUE	FALSE	13	93826052	3.07E-02	7.26E-02	TRUE	13	93173799	1.98E-02	0.96	6784	5.02E-06	TRUE	TRUE	482,730	20.86
Phosphatidylinositol (18:0_20:3) levels	Parkinson's disease	rs59382088	A	G	A	G	2.72E-01	-2.47E-02	2.01E-02	9.64E-01	FALSE	FALSE	12	5995398	5.80E-02	1.73E-01	TRUE	12	5886232	6.10E-02	0.99	6784	8.23E-06	TRUE	TRUE	482,730	19.91
Phosphatidylinositol (18:0_20:3) levels	Parkinson's disease	rs59739041	C	T	C	T	1.76E-01	2.12E-02	4.79E-02	9.50E-01	FALSE	FALSE	15	58661083	5.23E-02	1.64E-01	TRUE	15	58368884	3.97E-02	0.99	6784	8.81E-06	TRUE	TRUE	482,730	19.78
Phosphatidylinositol (18:0_20:3) levels	Parkinson's disease	rs6498540	G	A	G	A	-1.26E-01	1.82E-02	3.34E-01	6.94E-01	FALSE	FALSE	16	15130594	1.84E-02	4.93E-01	TRUE	16	15036737	1.82E-02	0.99	6784	5.24E-12	TRUE	TRUE	482,730	47.76
Phosphatidylinositol (18:0_20:3) levels	Parkinson's disease	rs67945876	C	T	C	T	-9.53E-02	-2.53E-02	2.34E-01	7.50E-01	FALSE	FALSE	4	78975931	2.37E-02	5.43E-01	TRUE	4	78054777	2.05E-02	0.98	6784	3.28E-06	TRUE	TRUE	482,730	21.68
Phosphatidylinositol (18:0_20:3) levels	Parkinson's disease	rs72914920	A	G	A	G	-9.57E-02	6.80E-03	3.06E-01	7.78E-01	FALSE	FALSE	11	60550325	2.43E-02	1.08E-01	TRUE	11	60782852	1.89E-02	0.98	6784	3.98E-07	TRUE	TRUE	482,730	25.75
Phosphatidylinositol (18:0_20:3) levels	Parkinson's disease	rs7355249	T	C	T	C	4.77E-01	1.26E-02	6.56E-03	9.77E-01	FALSE	FALSE	2	26584190	6.40E-02	7.37E-02	TRUE	2	26361322	1.06E-01	0.99	6784	7.50E-06	TRUE	TRUE	482,730	20.09
Phosphatidylinositol (18:0_20:3) levels	Parkinson's disease	rs76457276	G	A	G	A	-2.57E-01	1.58E-01	2.58E-02	9.82E-01	FALSE	FALSE	4	37897944	9.65E-02	9.97E-01	TRUE	4	37896323	5.59E-02	0.94	6784	4.21E-06	TRUE	TRUE	482,730	21.20
Phosphatidylinositol (18:0_20:3) levels	Parkinson's disease	rs7782961	T	A	T	A	9.42E-02	5.86E-02	2.32E-01	1.82E-01	TRUE	FALSE	7	6335583	2.64E-02	1.32E-02	TRUE	7	6295952	2.05E-02	0.97	6784	4.21E-06	TRUE	TRUE	482,730	21.20
Phosphatidylinositol (18:0_20:3) levels	Parkinson's disease	rs7845664	G	A	G	A	-8.22E-02	3.43E-02	5.76E-01	4.96E-01	FALSE	FALSE	8	40569597	1.99E-02	4.24E-02	TRUE	8	40712078	1.74E-02	0.99	6784	2.26E-06	TRUE	TRUE	482,730	22.39
Phosphatidylinositol (18:0_20:3) levels	Parkinson's disease	rs80011585	T	C	T	C	-1.51E-01	5.51E-02	7.30E-02	9.32E-01	FALSE	FALSE	16	84290054	4.66E-02	6.24E-01	TRUE	16	84256448	3.36E-02	0.95	6784	7.28E-06	TRUE	TRUE	482,730	20.15
Phosphatidylinositol (18:0_20:3) levels	Parkinson's disease	rs9530145	T	C	T	C	1.25E-01	-3.07E-02	1.05E-01	8.33E-01	FALSE	FALSE	13	73645300	2.73E-02	5.82E-01	TRUE	13	73071162	2.80E-02	1.00	6784	8.04E-06	TRUE	TRUE	482,730	19.96
Phosphatidylinositol (18:0_20:3) levels	Parkinson's disease	rs964184	C	G	C	G	-1.62E-01	-5.68E-02	8.49E-01	8.63E-01	TRUE	FALSE	11	116648917	2.43E-02	9.71E-03	TRUE	11	116778201	2.38E-02	1.00	6784	1.05E-11	TRUE	TRUE	482,730	46.39
Phosphatidylinositol (18:0_20:3) levels	Parkinson's disease	rs976947	A	G	A	G	-8.09E-02	4.83E-02	6.35E-01	3.33E-01	FALSE	FALSE	11	115463786	2.36E-02	2.03E-02	TRUE	11	115593068	1.79E-02	1.00	6784	6.42E-06	TRUE	TRUE	482,730	20.39
Phosphatidylinositol (18:0_20:4) levels	Parkinson's disease	rs10138449	C	G	C	G	1.17E-01	-1.67E-02	1.33E-01	8.59E-02	TRUE	FALSE	14	62013834	3.46E-02	2.01E-01	TRUE	14	61547116	2.47E-02	0.98	7174	2.05E-06	TRUE	TRUE	482,730	22.58
Phosphatidylinositol (18:0_20:4) levels	Parkinson's disease	rs111265437	A	T	A	T	3.91E-01	2.16E-02	9.97E-03	2.15E-02	TRUE	FALSE	7	28284715	9.37E-02	8.76E-02	TRUE	7	28245096	8.74E-02	0.90	7174	7.87E-06	TRUE	TRUE	482,730	19.99
Phosphatidylinositol (18:0_20:4) levels	Parkinson's disease	rs112944184	T	C	T	C	3.03E-01	-7.91E-02	1.74E-02	9.74E-01	FALSE	FALSE	1	239475430	7.02E-02	5.85E-01	TRUE	1	239312130	6.35E-02	0.99	7174	1.92E-06	TRUE	TRUE	482,730	22.71
Phosphatidylinositol (18:0_20:4) levels	Parkinson's disease	rs114051008	C	T	C	T	3.51E-01	7.24E-02	1.21E-02	9.76E-01	FALSE	FALSE	9	72170060	7.67E-02	4.62E-01	TRUE	9	69555144	7.88E-02	0.98	7174	8.37E-06	TRUE	TRUE	482,730	19.88
Phosphatidylinositol (18:0_20:4) levels	Parkinson's disease	rs116730430	T	C	T	C	-2.61E-01	1.49E-02	2.17E-02	9.86E-01	FALSE	FALSE	5	35798168	1.05E-01	5.18E-02	TRUE	5	35798066	5.81E-02	0.97	7174	7.11E-06	TRUE	TRUE	482,730	20.19
Phosphatidylinositol (18:0_20:4) levels	Parkinson's disease	rs11780610	T	C	T	C	-9.22E-02	-1.22E-02	6.80E-01	2.84E-01	FALSE	FALSE	8	18259876	1.92E-02	2.80E-01	TRUE	8	18402366	1.78E-02	1.00	7174	2.25E-07	TRUE	TRUE	482,730	26.86
Phosphatidylinositol (18:0_20:4) levels	Parkinson's disease	rs12213531	T	C	T	C	7.56E-02	7.70E-03	5.08E-01	4.17E-01	FALSE	FALSE	6	156712326	2.28E-02	1.34E-01	TRUE	6	156391192	1.66E-02	1.00	7174	5.56E-06	TRUE	TRUE	482,730	20.66
Phosphatidylinositol (18:0_20:4) levels	Parkinson's disease	rs1260326	C	T	C	T	-9.64E-02	-6.78E-02	6.51E-01	4.20E-01	FALSE	FALSE	2	27730940	1.72E-02	4.04E-05	TRUE	2	27508073	1.74E-02	1.00	7174	3.06E-08	TRUE	TRUE	482,730	30.74
Phosphatidylinositol (18:0_20:4) levels	Parkinson's disease	rs13068826	A	G	A	G	-7.96E-02	-6.10E-03	5.87E-01	4.12E-01	FALSE	FALSE	3	171113792	2.04E-02	1.16E-01	TRUE	3	171396003	1.67E-02	1.00	7174	2.02E-06	TRUE	TRUE	482,730	22.61
Phosphatidylinositol (18:0_20:4) levels	Parkinson's disease	rs174533	A	G	A	G	-2.49E-01	-9.00E-04	4.09E-01	6.69E-01	FALSE	FALSE	11	61549025	1.79E-02	1.84E-02	TRUE	11	61781553	1.68E-02	1.00	7174	3.40E-49	TRUE	TRUE	482,730	220.71
Phosphatidylinositol (18:0_20:4) levels	Parkinson's disease	rs186923696	T	C	T	C	2.76E-01	1.56E-01	1.99E-02	9.87E-01	FALSE	FALSE	5	105341485	1.09E-01	8.18E-01	TRUE	5	106005784	6.23E-02	0.90	7174	9.94E-06	TRUE	TRUE	482,730	19.55
Phosphatidylinositol (18:0_20:4) levels	Parkinson's disease	rs187491194	A	G	A	G	-2.06E-01	2.18E-02	3.56E-02	9.77E-01	FALSE	FALSE	7	80529555	8.88E-02	9.37E-02	TRUE	7	80900239	4.59E-02	0.94	7174	6.85E-06	TRUE	TRUE	482,730	20.26
Phosphatidylinositol (18:0_20:4) levels	Parkinson's disease	rs2149773	T	C	T	C	1.18E-01	-9.60E-03	1.09E-01	8.20E-01	FALSE	FALSE	13	114890232	2.96E-02	1.27E-01	TRUE	13	114124757	2.66E-02	0.99	7174	9.48E-06	TRUE	TRUE	482,730	19.64
Phosphatidylinositol (18:0_20:4) levels	Parkinson's disease	rs35332062	A	G	A	G	-1.29E-01	-4.60E-03	1.22E-01	8.81E-01	FALSE	FALSE	7	73012042	2.72E-02	6.20E-02	TRUE	7	73597712	2.53E-02	1.00	7174	4.08E-07	TRUE	TRUE	482,730	25.70
Phosphatidylinositol (18:0_20:4) levels	Parkinson's disease	rs4629190	C	T	C	T	8.25E-02	-3.08E-02	4.87E-01	5.08E-01	FALSE	FALSE	2	69142158	1.81E-02	4.44E-02	TRUE	2	68915026	1.66E-02	0.99	7174	6.30E-07	TRUE	TRUE	482,730	24.86
Phosphatidylinositol (18:0_20:4) levels	Parkinson's disease	rs4912056	T	C	T	C	1.14E-01	3.52E-02	1.52E-01	8.13E-01	FALSE	FALSE	1	19244678	3.15E-02	5.77E-01	TRUE	1	18918184	2.32E-02	0.99	7174	1.02E-06	TRUE	TRUE	482,730	23.92
Phosphatidylinositol (18:0_20:4) levels	Parkinson's disease	rs61872308	C	G	C	G	-1.44E-01	7.20E-03	8.55E-02	1.07E-01	TRUE	FALSE	10	114055948	3.85E-02	6.99E-02	TRUE	10	112296190	3.07E-02	0.92	7174	2.65E-06	TRUE	TRUE	482,730	22.09
Phosphatidylinositol (18:0_20:4) levels	Parkinson's disease	rs67945876	C	T	C	T	-9.42E-02	-2.53E-02	2.34E-01	7.50E-01	FALSE	FALSE	4	78975931	2.37E-02	5.43E-01	TRUE	4	78054777	1.99E-02	0.98	7174	2.11E-06	TRUE	TRUE	482,730	22.53
Phosphatidylinositol (18:0_20:4) levels	Parkinson's disease	rs964184	C	G	C	G	-2.67E-01	-5.68E-02	8.49E-01	8.63E-01	TRUE	FALSE	11	116648917	2.43E-02	9.71E-03	TRUE	11	116778201	2.30E-02	1.00	7174	5.97E-31	TRUE	TRUE	482,730	135.08
Phosphatidylinositol (18:0_20:4) levels	Parkinson's disease	rs9910521	A	G	A	G	-1.34E-01	-1.77E-02	9.05E-02	8.83E-01	FALSE	FALSE	17	11465720	3.43E-02	2.18E-01	TRUE	17	11562403	2.92E-02	0.99	7174	4.48E-06	TRUE	TRUE	482,730	21.08
Phosphatidylinositol (18:1_18:1) levels	Parkinson's disease	rs10468017	T	C	T	C	8.86E-02	2.75E-02	3.38E-01	7.16E-01	FALSE	FALSE	15	58678512	1.87E-02	8.48E-01	TRUE	15	58386313	1.76E-02	1.00	7164	4.59E-07	TRUE	TRUE	482,730	25.48
Phosphatidylinositol (18:1_18:1) levels	Parkinson's disease	rs10889352	C	T	C	T	-1.54E-01	4.00E-04	2.62E-01	6.75E-01	FALSE	FALSE	1	63099023	1.80E-02	6.92E-03	TRUE	1	62633352	1.88E-02	1.00	7164	2.63E-16	TRUE	TRUE	482,730	67.38
Phosphatidylinositol (18:1_18:1) levels	Parkinson's disease	rs116845732	C	T	C	T	7.15E-01	-3.50E-02	3.57E-03	9.80E-01	FALSE	FALSE	14	38885146	8.86E-02	1.60E-01	TRUE	14	38415942	1.39E-01	0.89	7164	2.86E-07	TRUE	TRUE	482,730	26.39
Phosphatidylinositol (18:1_18:1) levels	Parkinson's disease	rs11783515	G	A	G	A	-8.88E-02	-4.80E-03	6.26E-01	3.30E-01	FALSE	FALSE	8	59320389	2.28E-02	7.84E-02	TRUE	8	58407830	1.72E-02	1.00	7164	2.39E-07	TRUE	TRUE	482,730	26.74
Phosphatidylinositol (18:1_18:1) levels	Parkinson's disease	rs12428337	A	G	A	G	8.94E-02	2.47E-02	2.38E-01	8.03E-01	FALSE	FALSE	13	23175293	2.81E-02	4.21E-01	TRUE	13	22601154	1.95E-02	1.00	7164	4.56E-06	TRUE	TRUE	482,730	21.04
Phosphatidylinositol (18:1_18:1) levels	Parkinson's disease	rs145993592	A	C	A	C	4.72E-01	-2.76E-02	6.70E-03	9.87E-01	FALSE	FALSE	9	94531986	1.05E-01	1.01E-01	TRUE	9	91769704	1.04E-01	0.94	7164	5.87E-06	TRUE	TRUE	482,730	20.56
Phosphatidylinositol (18:1_18:1) levels	Parkinson's disease	rs149540646	G	A	G	A	-8.60E-02	-2.32E-02	2.58E-01	7.72E-01	FALSE	FALSE	3	74520102	2.69E-02	4.10E-01	TRUE	3	74470951	1.91E-02	1.00	7164	6.60E-06	TRUE	TRUE	482,730	20.33
Phosphatidylinositol (18:1_18:1) levels	Parkinson's disease	rs174568	T	C	T	C	1.57E-01	-4.40E-03	4.05E-01	6.73E-01	FALSE	FALSE	11	61593816	1.79E-02	9.37E-02	TRUE	11	61826344	1.70E-02	1.00	7164	3.37E-20	TRUE	TRUE	482,730	85.26
Phosphatidylinositol (18:1_18:1) levels	Parkinson's disease	rs1751064	G	A	G	A	-8.75E-02	-2.36E-02	7.60E-01	2.64E-01	FALSE	FALSE	13	95777748	2.57E-02	4.47E-01	TRUE	13	95125494	1.96E-02	1.00	7164	7.79E-06	TRUE	TRUE	482,730	20.02
Phosphatidylinositol (18:1_18:1) levels	Parkinson's disease	rs186422572	A	G	A	G	3.47E-01	-6.02E-02	1.32E-02	9.71E-01	FALSE	FALSE	14	104082970	7.26E-02	3.90E-01	TRUE	14	103616633	7.53E-02	0.94	7164	4.08E-06	TRUE	TRUE	482,730	21.26
Phosphatidylinositol (18:1_18:1) levels	Parkinson's disease	rs62409317	A	G	A	G	1.95E-01	1.57E-02	4.62E-02	9.42E-01	FALSE	FALSE	4	25951653	5.26E-02	1.16E-01	TRUE	4	25950031	4.20E-02	0.91	7164	3.65E-06	TRUE	TRUE	482,730	21.47
Phosphatidylinositol (18:1_18:1) levels	Parkinson's disease	rs627108	T	C	T	C	-8.46E-02	7.60E-03	3.96E-01	6.49E-01	FALSE	FALSE	1	230310568	1.98E-02	1.55E-01	TRUE	1	230174822	1.70E-02	0.99	7164	6.88E-07	TRUE	TRUE	482,730	24.69
Phosphatidylinositol (18:1_18:1) levels	Parkinson's disease	rs72846591	C	T	C	T	8.00E-02	1.16E-02	3.98E-01	6.01E-01	FALSE	FALSE	2	142470239	2.28E-02	2.14E-01	TRUE	2	141712670	1.71E-02	1.00	7164	3.00E-06	TRUE	TRUE	482,730	21.85
Phosphatidylinositol (18:1_18:1) levels	Parkinson's disease	rs78572804	G	A	G	A	4.06E-01	1.10E-01	9.68E-03	9.71E-01	FALSE	FALSE	10	76637703	7.70E-02	8.16E-01	TRUE	10	74877945	8.90E-02	0.92	7164	5.15E-06	TRUE	TRUE	482,730	20.81
Phosphatidylinositol (18:1_18:1) levels	Parkinson's disease	rs964184	C	G	C	G	-1.66E-01	-5.68E-02	8.49E-01	8.63E-01	TRUE	FALSE	11	116648917	2.43E-02	9.71E-03	TRUE	11	116778201	2.32E-02	1.00	7164	7.65E-13	TRUE	TRUE	482,730	51.56
Phosphatidylinositol (18:1_18:1) levels	Parkinson's disease	rs9652580	T	C	T	C	-8.05E-02	1.80E-02	4.23E-01	5.64E-01	FALSE	FALSE	16	24308825	2.28E-02	3.65E-01	TRUE	16	24297504	1.73E-02	0.98	7164	3.30E-06	TRUE	TRUE	482,730	21.67
Phosphatidylinositol (18:1_18:2) levels	Parkinson's disease	rs10889352	C	T	C	T	-1.24E-01	4.00E-04	2.62E-01	6.75E-01	FALSE	FALSE	1	63099023	1.80E-02	6.92E-03	TRUE	1	62633352	1.99E-02	1.00	6489	4.53E-10	TRUE	TRUE	482,730	38.99
Phosphatidylinositol (18:1_18:2) levels	Parkinson's disease	rs10911365	A	G	A	G	1.44E-01	-4.13E-02	8.96E-02	9.21E-01	FALSE	FALSE	1	183559105	3.63E-02	5.93E-01	TRUE	1	183589970	3.10E-02	0.98	6489	3.29E-06	TRUE	TRUE	482,730	21.67
Phosphatidylinositol (18:1_18:2) levels	Parkinson's disease	rs114782597	A	G	A	G	-4.66E-01	5.20E-03	7.60E-03	9.81E-01	FALSE	FALSE	2	52672311	8.70E-02	2.11E-02	TRUE	2	52445173	1.04E-01	0.97	6489	8.06E-06	TRUE	TRUE	482,730	19.95
Phosphatidylinositol (18:1_18:2) levels	Parkinson's disease	rs117234964	T	C	T	C	1.34E-01	1.58E-02	9.91E-02	9.57E-01	FALSE	FALSE	8	100603038	6.16E-02	9.79E-02	TRUE	8	99590810	2.98E-02	0.97	6489	7.08E-06	TRUE	TRUE	482,730	20.20
Phosphatidylinositol (18:1_18:2) levels	Parkinson's disease	rs11803708	A	G	A	G	1.97E-01	4.40E-03	4.86E-02	9.35E-01	FALSE	FALSE	1	236801119	3.82E-02	4.16E-02	TRUE	1	236637819	4.11E-02	1.00	6489	1.69E-06	TRUE	TRUE	482,730	22.97
Phosphatidylinositol (18:1_18:2) levels	Parkinson's disease	rs12103149	A	G	A	G	-1.39E-01	9.60E-03	9.12E-02	9.07E-01	FALSE	FALSE	16	24673898	3.85E-02	9.49E-02	TRUE	16	24662577	3.04E-02	1.00	6489	5.11E-06	TRUE	TRUE	482,730	20.83
Phosphatidylinositol (18:1_18:2) levels	Parkinson's disease	rs12943438	C	G	C	G	2.56E-01	1.71E-01	2.55E-02	2.28E-02	TRUE	FALSE	17	21119865	8.10E-02	1.74E-02	TRUE	17	21216552	5.67E-02	0.96	6489	6.16E-06	TRUE	TRUE	482,730	20.47
Phosphatidylinositol (18:1_18:2) levels	Parkinson's disease	rs144480172	T	C	T	C	2.79E-01	-2.34E-02	2.51E-02	9.80E-01	FALSE	FALSE	12	32701533	8.61E-02	1.05E-01	TRUE	12	32548599	5.65E-02	0.96	6489	8.07E-07	TRUE	TRUE	482,730	24.39
Phosphatidylinositol (18:1_18:2) levels	Parkinson's disease	rs1544531	A	C	A	C	8.24E-02	-1.39E-02	5.17E-01	5.08E-01	FALSE	FALSE	16	5642868	2.42E-02	2.48E-01	TRUE	16	5592867	1.74E-02	0.99	6489	2.37E-06	TRUE	TRUE	482,730	22.31
Phosphatidylinositol (18:1_18:2) levels	Parkinson's disease	rs17074477	G	A	G	A	-1.40E-01	3.04E-02	1.09E-01	8.99E-01	FALSE	FALSE	5	171649520	3.74E-02	3.80E-01	TRUE	5	172222516	2.80E-02	0.99	6489	6.51E-07	TRUE	TRUE	482,730	24.80
Phosphatidylinositol (18:1_18:2) levels	Parkinson's disease	rs174568	T	C	T	C	1.61E-01	-4.40E-03	4.05E-01	6.73E-01	FALSE	FALSE	11	61593816	1.79E-02	9.37E-02	TRUE	11	61826344	1.78E-02	1.00	6489	1.47E-19	TRUE	TRUE	482,730	82.35
Phosphatidylinositol (18:1_18:2) levels	Parkinson's disease	rs2701185	G	A	G	A	7.99E-02	2.46E-02	5.39E-01	4.68E-01	FALSE	FALSE	12	121352974	1.80E-02	7.63E-01	TRUE	12	120915171	1.76E-02	1.00	6489	5.94E-06	TRUE	TRUE	482,730	20.54
Phosphatidylinositol (18:1_18:2) levels	Parkinson's disease	rs4848460	T	C	T	C	1.87E-01	-9.50E-03	5.02E-02	9.27E-01	FALSE	FALSE	2	118399351	4.47E-02	7.99E-02	TRUE	2	117641775	4.05E-02	0.96	6489	3.94E-06	TRUE	TRUE	482,730	21.33
Phosphatidylinositol (18:1_18:2) levels	Parkinson's disease	rs72753955	G	A	G	A	-4.17E-01	2.60E-02	1.07E-02	9.81E-01	FALSE	FALSE	15	83439818	7.03E-02	1.48E-01	TRUE	15	82771066	8.78E-02	0.98	6489	2.09E-06	TRUE	TRUE	482,730	22.55
Phosphatidylinositol (18:1_18:2) levels	Parkinson's disease	rs73109128	G	C	G	C	2.59E-01	1.39E-02	2.78E-02	6.07E-02	TRUE	FALSE	3	74359882	3.67E-02	1.51E-01	TRUE	3	74310731	5.46E-02	1.00	6489	2.20E-06	TRUE	TRUE	482,730	22.45
Phosphatidylinositol (18:1_18:2) levels	Parkinson's disease	rs75519325	A	G	A	G	1.62E-01	-6.10E-03	6.93E-02	9.56E-01	FALSE	FALSE	3	196299444	6.19E-02	3.53E-02	TRUE	3	196572573	3.55E-02	0.93	6489	5.23E-06	TRUE	TRUE	482,730	20.78
Phosphatidylinositol (18:1_18:2) levels	Parkinson's disease	rs79507869	A	C	A	C	-1.25E-01	-4.50E-03	1.29E-01	8.99E-01	FALSE	FALSE	2	42401076	3.83E-02	4.22E-02	TRUE	2	42173936	2.62E-02	1.00	6489	1.79E-06	TRUE	TRUE	482,730	22.84
Phosphatidylinositol (18:1_18:2) levels	Parkinson's disease	rs964184	C	G	C	G	-1.23E-01	-5.68E-02	8.49E-01	8.63E-01	TRUE	FALSE	11	116648917	2.43E-02	9.71E-03	TRUE	11	116778201	2.42E-02	1.00	6489	4.28E-07	TRUE	TRUE	482,730	25.62
Phosphatidylinositol (18:1_20:4) levels	Parkinson's disease	rs10775270	C	A	C	A	-9.59E-02	-2.05E-02	7.53E-01	3.34E-01	FALSE	FALSE	16	10229982	2.02E-02	5.09E-01	TRUE	16	10136125	2.16E-02	1.00	5882	9.48E-06	TRUE	TRUE	482,730	19.65
Phosphatidylinositol (18:1_20:4) levels	Parkinson's disease	rs111645166	A	G	A	G	-4.65E-01	-4.85E-02	8.70E-03	9.76E-01	FALSE	FALSE	13	32126907	8.16E-02	2.58E-01	TRUE	13	31552770	1.05E-01	0.92	5882	9.76E-06	TRUE	TRUE	482,730	19.59
Phosphatidylinositol (18:1_20:4) levels	Parkinson's disease	rs117210444	G	A	G	A	-2.87E-01	2.10E-01	3.45E-02	9.75E-01	FALSE	FALSE	16	73892971	7.99E-02	4.37E-03	TRUE	16	73859072	4.98E-02	0.97	5882	9.19E-09	TRUE	TRUE	482,730	33.10
Phosphatidylinositol (18:1_20:4) levels	Parkinson's disease	rs117598891	C	T	C	T	-4.82E-01	5.45E-02	8.15E-03	9.81E-01	FALSE	FALSE	14	95498315	9.62E-02	2.43E-01	TRUE	14	95031978	1.05E-01	0.94	5882	4.91E-06	TRUE	TRUE	482,730	20.91
Phosphatidylinositol (18:1_20:4) levels	Parkinson's disease	rs117960039	G	A	G	A	2.50E-01	-1.68E-01	2.86E-02	9.73E-01	FALSE	FALSE	1	97949304	6.95E-02	7.73E-03	TRUE	1	97483748	5.57E-02	0.94	5882	7.25E-06	TRUE	TRUE	482,730	20.16
Phosphatidylinositol (18:1_20:4) levels	Parkinson's disease	rs12467458	G	T	G	T	1.26E-01	1.00E-04	1.26E-01	9.01E-01	FALSE	FALSE	2	214838013	3.70E-02	6.52E-04	TRUE	2	213973289	2.80E-02	0.98	5882	6.52E-06	TRUE	TRUE	482,730	20.36
Phosphatidylinositol (18:1_20:4) levels	Parkinson's disease	rs12554791	G	T	G	T	1.36E-01	-4.76E-02	1.10E-01	9.15E-01	FALSE	FALSE	9	31860326	4.28E-02	5.75E-01	TRUE	9	31860328	3.02E-02	0.94	5882	6.69E-06	TRUE	TRUE	482,730	20.31
Phosphatidylinositol (18:1_20:4) levels	Parkinson's disease	rs12938892	G	A	G	A	-8.72E-02	8.20E-03	4.37E-01	6.54E-01	FALSE	FALSE	17	10007092	3.02E-02	1.05E-01	TRUE	17	10103775	1.93E-02	0.91	5882	6.54E-06	TRUE	TRUE	482,730	20.36

Phosphatidylinositol (18:1_20:4) levels	Parkinson's disease	rs13291383	C	T	C	T	9.60E-02	-1.81E-02	2.76E-01	6.96E-01	FALSE	FALSE	9	136762812	3.13E-02	2.50E-01	TRUE	9	133897690	2.14E-02	0.93	5882	7.48E-06	TRUE	TRUE	482,730	20.10
Phosphatidylinositol (18:1_20:4) levels	Parkinson's disease	rs140854080	A	G	A	G	3.70E-01	1.83E-01	1.40E-02	9.85E-01	FALSE	FALSE	4	88328404	9.92E-02	3.22E-02	TRUE	4	87407252	7.85E-02	0.99	5882	2.47E-06	TRUE	TRUE	482,730	22.23
Phosphatidylinositol (18:1_20:4) levels	Parkinson's disease	rs2819083	A	G	A	G	-9.24E-02	1.40E-02	4.86E-01	6.22E-01	FALSE	FALSE	6	156700816	2.34E-02	2.59E-01	TRUE	6	156379682	1.84E-02	0.99	5882	4.92E-07	TRUE	TRUE	482,730	25.35
Phosphatidylinositol (18:1_20:4) levels	Parkinson's disease	rs36061340	T	C	T	C	1.94E-01	-2.37E-02	5.21E-02	9.41E-01	FALSE	FALSE	8	101807230	4.55E-02	2.20E-01	TRUE	8	100795002	4.14E-02	1.00	5882	2.94E-06	TRUE	TRUE	482,730	21.90
Phosphatidylinositol (18:1_20:4) levels	Parkinson's disease	rs3741298	T	C	T	C	-1.27E-01	4.06E-02	7.65E-01	1.98E-01	FALSE	FALSE	11	116657561	2.12E-02	2.77E-02	TRUE	11	116786845	2.16E-02	1.00	5882	4.35E-09	TRUE	TRUE	482,730	34.56
Phosphatidylinositol (18:1_20:4) levels	Parkinson's disease	rs4920209	A	G	A	G	8.77E-02	1.78E-02	4.75E-01	5.55E-01	FALSE	FALSE	1	234164516	2.01E-02	4.27E-01	TRUE	1	234028770	1.83E-02	1.00	5882	1.63E-06	TRUE	TRUE	482,730	23.03
Phosphatidylinositol (18:1_20:4) levels	Parkinson's disease	rs55976852	C	A	C	A	-9.88E-02	-1.46E-02	2.74E-01	7.97E-01	FALSE	FALSE	6	94947385	2.27E-02	2.84E-01	TRUE	6	94237667	2.07E-02	0.99	5882	1.92E-06	TRUE	TRUE	482,730	22.72
Phosphatidylinositol (18:1_20:4) levels	Parkinson's disease	rs61915733	A	G	A	G	1.52E-01	3.39E-02	8.97E-02	8.80E-01	FALSE	FALSE	12	18323957	3.57E-02	4.65E-01	TRUE	12	18171023	3.20E-02	0.97	5882	2.09E-06	TRUE	TRUE	482,730	22.56
Phosphatidylinositol (18:1_20:4) levels	Parkinson's disease	rs670975	T	A	T	A	1.73E-01	1.43E-02	6.69E-02	7.38E-02	TRUE	FALSE	11	75933188	3.82E-02	1.50E-01	TRUE	11	76222144	3.76E-02	1.00	5882	4.20E-06	TRUE	TRUE	482,730	21.21
Phosphatidylinositol (18:1_20:4) levels	Parkinson's disease	rs74559802	A	G	A	G	-4.32E-01	6.09E-02	1.08E-02	9.81E-01	FALSE	FALSE	10	6227442	1.02E-01	2.59E-01	TRUE	10	6185479	9.74E-02	0.81	5882	9.56E-06	TRUE	TRUE	482,730	19.63
Phosphatidylinositol (18:1_20:4) levels	Parkinson's disease	rs74572381	A	G	A	G	1.73E-01	3.45E-02	6.23E-02	8.91E-01	FALSE	FALSE	14	35521865	3.12E-02	5.72E-01	TRUE	14	35052659	3.89E-02	1.00	5882	8.84E-06	TRUE	TRUE	482,730	19.78
Sphingomyelin (d32:1) levels	Parkinson's disease	rs10192465	C	G	C	G	-8.63E-02	-4.65E-02	7.21E-01	7.87E-01	TRUE	FALSE	2	57758855	3.55E-02	7.21E-01	TRUE	2	57531720	1.90E-02	0.94	7174	5.41E-06	TRUE	TRUE	482,730	20.72
Sphingomyelin (d32:1) levels	Parkinson's disease	rs10468017	T	C	T	C	8.35E-02	2.75E-02	3.38E-01	7.16E-01	FALSE	FALSE	15	58678512	1.87E-02	8.48E-01	TRUE	15	58386313	1.75E-02	1.00	7174	1.82E-06	TRUE	TRUE	482,730	22.82
Sphingomyelin (d32:1) levels	Parkinson's disease	rs10968815	G	A	G	A	1.30E-01	-1.23E-02	9.22E-02	9.43E-01	FALSE	FALSE	9	28896198	5.37E-02	8.67E-02	TRUE	9	28896200	2.92E-02	0.99	7174	8.82E-06	TRUE	TRUE	482,730	19.78
Sphingomyelin (d32:1) levels	Parkinson's disease	rs10975312	A	G	A	G	2.46E-01	0.00E+00	3.12E-02	9.80E-01	FALSE	FALSE	9	5837780	8.29E-02	8.69E-05	TRUE	9	5837780	4.87E-02	0.98	7174	4.55E-07	TRUE	TRUE	482,730	25.49
Sphingomyelin (d32:1) levels	Parkinson's disease	rs11591147	T	G	T	G	-2.33E-01	-3.77E-02	3.32E-02	9.82E-01	FALSE	FALSE	1	55505647	6.83E-02	2.36E-01	TRUE	1	55039974	4.65E-02	1.00	7174	5.44E-07	TRUE	TRUE	482,730	25.15
Sphingomyelin (d32:1) levels	Parkinson's disease	rs1218344	C	A	C	A	9.96E-02	1.39E-02	1.70E-01	8.48E-01	FALSE	FALSE	10	14311683	3.09E-02	1.85E-01	TRUE	10	14269684	2.23E-02	1.00	7174	7.92E-06	TRUE	TRUE	482,730	19.99
Sphingomyelin (d32:1) levels	Parkinson's disease	rs12192157	T	C	T	C	7.89E-02	3.64E-02	4.65E-01	4.47E-01	FALSE	FALSE	6	169720903	2.29E-02	9.52E-01	TRUE	6	169320808	1.68E-02	0.98	7174	2.78E-06	TRUE	TRUE	482,730	21.99
Sphingomyelin (d32:1) levels	Parkinson's disease	rs12622488	T	C	T	C	-1.20E-01	-5.80E-03	1.13E-01	8.21E-01	FALSE	FALSE	2	47443962	2.52E-02	8.80E-02	TRUE	2	47216823	2.62E-02	0.98	7174	5.02E-06	TRUE	TRUE	482,730	20.86
Sphingomyelin (d32:1) levels	Parkinson's disease	rs16851199	G	A	G	A	-1.88E-01	6.27E-02	4.28E-02	9.75E-01	FALSE	FALSE	1	230379953	8.05E-02	3.61E-01	TRUE	1	230244207	4.23E-02	0.94	7174	9.37E-06	TRUE	TRUE	482,730	19.66
Sphingomyelin (d32:1) levels	Parkinson's disease	rs217184	C	T	C	T	-9.55E-02	-4.18E-02	2.28E-01	8.02E-01	FALSE	FALSE	16	72105965	2.27E-02	3.28E-02	TRUE	16	72072066	1.98E-02	0.99	7174	1.49E-06	TRUE	TRUE	482,730	23.20
Sphingomyelin (d32:1) levels	Parkinson's disease	rs2553631	T	A	T	A	-1.51E-01	-7.42E-02	6.99E-02	4.58E-02	TRUE	FALSE	2	125121775	5.46E-02	7.59E-01	TRUE	2	124364198	3.27E-02	1.00	7174	4.24E-06	TRUE	TRUE	482,730	21.18
Sphingomyelin (d32:1) levels	Parkinson's disease	rs2604631	A	G	A	G	-8.96E-02	2.96E-02	7.54E-01	3.27E-01	FALSE	FALSE	4	61803239	2.36E-02	6.75E-01	TRUE	4	60937521	1.94E-02	1.00	7174	4.03E-06	TRUE	TRUE	482,730	21.28
Sphingomyelin (d32:1) levels	Parkinson's disease	rs3026120	A	G	A	G	-1.75E-01	-1.28E-02	7.46E-02	9.54E-01	FALSE	FALSE	17	5287415	4.14E-02	1.20E-01	TRUE	17	5384095	3.12E-02	1.00	7174	2.14E-08	TRUE	TRUE	482,730	31.43
Sphingomyelin (d32:1) levels	Parkinson's disease	rs3741111	G	A	G	A	8.16E-02	-5.00E-04	4.83E-01	5.00E-01	FALSE	FALSE	11	123947295	1.95E-02	8.69E-03	TRUE	11	124076588	1.67E-02	0.99	7174	1.02E-06	TRUE	TRUE	482,730	23.93
Sphingomyelin (d32:1) levels	Parkinson's disease	rs58001441	A	G	A	G	-1.04E-01	2.73E-02	1.65E-01	8.76E-01	FALSE	FALSE	9	6764828	3.38E-02	3.76E-01	TRUE	9	6764828	2.25E-02	1.00	7174	4.21E-06	TRUE	TRUE	482,730	21.20
Sphingomyelin (d32:1) levels	Parkinson's disease	rs61927425	T	C	T	C	-2.21E-01	2.15E-01	3.29E-02	9.84E-01	FALSE	FALSE	12	80115934	1.55E-01	7.82E-01	TRUE	12	79722154	4.86E-02	0.89	7174	5.53E-06	TRUE	TRUE	482,730	20.67
Sphingomyelin (d32:1) levels	Parkinson's disease	rs61977690	G	C	G	C	2.82E-01	1.96E-02	2.01E-02	3.07E-02	TRUE	FALSE	14	23289292	6.07E-02	1.27E-01	TRUE	14	22820083	6.26E-02	0.89	7174	7.03E-06	TRUE	TRUE	482,730	20.21
Sphingomyelin (d32:1) levels	Parkinson's disease	rs6444176	T	C	T	C	1.80E-01	9.82E-02	5.20E-02	9.72E-01	FALSE	FALSE	3	186586060	6.39E-02	9.04E-01	TRUE	3	186868271	3.75E-02	0.96	7174	1.59E-06	TRUE	TRUE	482,730	23.07
Sphingomyelin (d32:1) levels	Parkinson's disease	rs7157785	T	G	T	G	5.13E-01	-3.20E-02	1.25E-01	8.32E-01	FALSE	FALSE	14	64235556	2.25E-02	8.11E-01	TRUE	14	63768838	2.44E-02	1.00	7174	3.08E-95	TRUE	TRUE	482,730	441.86
Sphingomyelin (d32:1) levels	Parkinson's disease	rs72649550	C	G	C	G	8.15E-02	5.68E-02	3.32E-01	1.98E-01	TRUE	FALSE	15	94154562	2.87E-02	2.39E-02	TRUE	15	93611333	1.78E-02	0.98	7174	4.73E-06	TRUE	TRUE	482,730	20.97
Sphingomyelin (d32:1) levels	Parkinson's disease	rs75418091	T	C	T	C	4.06E-01	2.24E-02	1.19E-02	9.88E-01	FALSE	FALSE	4	4503361	1.15E-01	7.32E-02	TRUE	4	4501634	7.92E-02	0.94	7174	3.03E-07	TRUE	TRUE	482,730	26.28
Sphingomyelin (d32:1) levels	Parkinson's disease	rs75679663	A	C	A	C	-2.56E-01	1.35E-01	2.58E-02	9.87E-01	FALSE	FALSE	17	4667972	9.72E-02	7.79E-01	TRUE	17	4764677	5.37E-02	0.98	7174	1.86E-06	TRUE	TRUE	482,730	22.77
Sphingomyelin (d32:1) levels	Parkinson's disease	rs8017356	G	A	G	A	-3.48E-01	-7.87E-02	9.86E-01	3.47E-02	FALSE	FALSE	14	65581781	4.82E-02	9.90E-01	TRUE	14	65115063	7.17E-02	0.99	7174	1.29E-06	TRUE	TRUE	482,730	23.48
Sphingomyelin (d34:0) levels	Parkinson's disease	rs112585713	A	G	A	G	2.62E-01	1.07E-01	3.16E-02	9.80E-01	FALSE	FALSE	4	73742418	9.00E-02	6.32E-01	TRUE	4	72876701	5.10E-02	0.98	6207	2.96E-07	TRUE	TRUE	482,730	26.33
Sphingomyelin (d34:0) levels	Parkinson's disease	rs113312978	A	G	A	G	-6.45E-01	2.40E-01	3.74E-03	9.86E-01	FALSE	FALSE	11	59498713	1.07E-01	1.28E-02	TRUE	11	59731240	1.46E-01	0.89	6207	9.79E-06	TRUE	TRUE	482,730	19.58
Sphingomyelin (d34:0) levels	Parkinson's disease	rs114372832	C	G	C	G	-3.00E-01	-8.50E-02	1.97E-02	3.45E-02	TRUE	FALSE	2	75951334	5.56E-02	8.99E-01	TRUE	2	75724208	6.69E-02	0.99	6207	7.69E-06	TRUE	TRUE	482,730	20.05
Sphingomyelin (d34:0) levels	Parkinson's disease	rs11591147	T	G	T	G	-2.56E-01	-3.77E-02	3.32E-02	9.82E-01	FALSE	FALSE	1	55505647	6.83E-02	2.36E-01	TRUE	1	55039974	5.28E-02	1.00	6207	1.33E-06	TRUE	TRUE	482,730	23.42
Sphingomyelin (d34:0) levels	Parkinson's disease	rs11631073	T	C	T	C	-9.12E-02	-3.60E-03	6.18E-01	4.72E-01	FALSE	FALSE	15	57614545	1.80E-02	7.39E-02	TRUE	15	57322347	1.87E-02	0.99	6207	1.11E-06	TRUE	TRUE	482,730	23.78
Sphingomyelin (d34:0) levels	Parkinson's disease	rs1324162	G	T	G	T	9.00E-02	3.63E-02	2.71E-01	7.18E-01	FALSE	FALSE	9	83472933	2.48E-02	8.43E-01	TRUE	9	80858018	2.01E-02	1.00	6207	7.81E-06	TRUE	TRUE	482,730	20.02
Sphingomyelin (d34:0) levels	Parkinson's disease	rs16850360	G	A	G	A	2.27E-01	-3.83E-02	3.33E-02	9.51E-01	FALSE	FALSE	4	74872445	6.18E-02	2.71E-01	TRUE	4	74006728	5.04E-02	0.99	6207	6.51E-06	TRUE	TRUE	482,730	20.36
Sphingomyelin (d34:0) levels	Parkinson's disease	rs174535	C	T	C	T	-9.76E-02	-2.60E-03	4.09E-01	6.66E-01	FALSE	FALSE	11	61551356	1.78E-02	5.31E-02	TRUE	11	61783884	1.84E-02	1.00	6207	1.14E-07	TRUE	TRUE	482,730	28.19
Sphingomyelin (d34:0) levels	Parkinson's disease	rs17497891	G	A	G	A	1.19E-01	1.54E-02	1.50E-01	9.17E-01	FALSE	FALSE	1	222357810	4.24E-02	1.45E-01	TRUE	1	222184468	2.52E-02	0.99	6207	2.51E-06	TRUE	TRUE	482,730	22.20
Sphingomyelin (d34:0) levels	Parkinson's disease	rs2808569	C	A	C	A	-9.79E-02	8.10E-03	2.42E-01	7.78E-01	FALSE	FALSE	9	101299559	2.67E-02	1.19E-01	TRUE	9	98537277	2.11E-02	1.00	6207	3.51E-06	TRUE	TRUE	482,730	21.55
Sphingomyelin (d34:0) levels	Parkinson's disease	rs3026120	A	G	A	G	-2.07E-01	-1.28E-02	7.46E-02	9.54E-01	FALSE	FALSE	17	5287415	4.14E-02	1.20E-01	TRUE	17	5384095	3.42E-02	1.00	6207	1.44E-09	TRUE	TRUE	482,730	36.72
Sphingomyelin (d34:0) levels	Parkinson's disease	rs55811539	C	T	C	T	-3.99E-01	1.31E-01	1.24E-02	9.78E-01	FALSE	FALSE	3	113032399	7.01E-02	3.12E-02	TRUE	3	113313552	8.36E-02	0.99	6207	1.83E-06	TRUE	TRUE	482,730	22.80
Sphingomyelin (d34:0) levels	Parkinson's disease	rs55985238	A	T	A	T	-5.60E-01	4.56E-01	8.82E-03	6.60E-03	TRUE	FALSE	17	3672503	2.71E-01	4.63E-02	TRUE	17	3769209	1.01E-01	0.90	6207	2.72E-08	TRUE	TRUE	482,730	30.97
Sphingomyelin (d34:0) levels	Parkinson's disease	rs6683774	T	C	T	C	1.78E-01	5.80E-03	9.34E-01	5.40E-02	FALSE	FALSE	1	198594595	5.56E-02	3.79E-02	TRUE	1	198625465	3.65E-02	0.97	6207	1.17E-06	TRUE	TRUE	482,730	23.67
Sphingomyelin (d34:0) levels	Parkinson's disease	rs75679663	A	C	A	C	-6.89E-01	1.35E-01	2.58E-02	9.87E-01	FALSE	FALSE	17	4667972	9.72E-02	7.79E-01	TRUE	17	4764677	6.23E-02	0.98	6207	3.31E-28	TRUE	TRUE	482,730	122.50
Sphingomyelin (d34:0) levels	Parkinson's disease	rs7814780	T	G	T	G	-8.70E-02	-3.90E-03	3.28E-01	6.29E-01	FALSE	FALSE	8	123239940	2.32E-02	6.19E-02	TRUE	8	122227701	1.93E-02	1.00	6207	6.75E-06	TRUE	TRUE	482,730	20.30
Sphingomyelin (d34:0) levels	Parkinson's disease	rs80130206	T	C	T	C	-1.82E-01	3.00E-04	5.50E-02	9.68E-01	FALSE	FALSE	10	107989164	6.79E-02	1.48E-03	TRUE	10	106229406	3.90E-02	0.99	6207	2.99E-06	TRUE	TRUE	482,730	21.86
Sphingomyelin (d34:0) levels	Parkinson's disease	rs934198	T	G	T	G	9.78E-02	3.40E-03	2.97E-01	6.77E-01	FALSE	FALSE	2	21278773	1.85E-02	6.75E-02	TRUE	2	21055901	1.98E-02	1.00	6207	8.40E-07	TRUE	TRUE	482,730	24.31
Sphingomyelin (d34:1) levels	Parkinson's disease	rs114483871	T	C	T	C	2.82E-01	-2.94E-02	3.81E-02	9.77E-01	FALSE	FALSE	4	73990168	8.47E-02	1.38E-01	TRUE	4	73124451	4.48E-02	0.93	7174	3.24E-10	TRUE	TRUE	482,730	39.64
Sphingomyelin (d34:1) levels	Parkinson's disease	rs11591147	T	G	T	G	-4.19E-01	-3.77E-02	3.32E-02	9.82E-01	FALSE	FALSE	1	55505647	6.83E-02	2.36E-01	TRUE	1	55039974	4.64E-02	1.00	7174	2.32E-19	TRUE	TRUE	482,730	81.41
Sphingomyelin (d34:1) levels	Parkinson's disease	rs116302332	T	C	T	C	3.77E-01	1.00E-01	2.81E-02	9.75E-01	FALSE	FALSE	4	75370891	8.11E-02	6.62E-01	TRUE	4	74505174	5.07E-02	0.96	7174	1.28E-13	TRUE	TRUE	482,730	55.10
Sphingomyelin (d34:1) levels	Parkinson's disease	rs117279275	A	G	A	G	-3.34E-01	1.36E-02	1.44E-02	9.73E-01	FALSE	FALSE	12	53286039	5.33E-02	9.80E-02	TRUE	12	52892255	6.94E-02	1.00	7174	1.52E-06	TRUE	TRUE	482,730	23.17
Sphingomyelin (d34:1) levels	Parkinson's disease	rs141286087	G	A	G	A	2.19E-01	-1.71E-01	4.08E-02	9.79E-01	FALSE	FALSE	16	51063903	8.95E-02	2.80E-02	TRUE	16	51029992	4.52E-02	0.86	7174	1.31E-06	TRUE	TRUE	482,730	23.44
Sphingomyelin (d34:1) levels	Parkinson's disease	rs191640693	T	C	T	C	-2.44E-01	1.60E-01	2.72E-02	9.86E-01	FALSE	FALSE	13	47775477	1.05E-01	8.93E-01	TRUE	13	47201342	5.30E-02	0.95	7174	4.13E-06	TRUE	TRUE	482,730	21.24
Sphingomyelin (d34:1) levels	Parkinson's disease	rs1934410	T	G	T	G	8.16E-02	2.82E-02	6.27E-01	3.88E-01	FALSE	FALSE	1	180521764	1.76E-02	9.63E-01	TRUE	1	180552628	1.74E-02	1.00	7174	2.86E-06	TRUE	TRUE	482,730	21.94
Sphingomyelin (d34:1) levels	Parkinson's disease	rs2306619	G	A	G	A	-1.01E-01	-4.54E-02	1.83E-01	8.27E-01	FALSE	FALSE	5	125887856	2.62E-02	4.16E-02	TRUE	5	126552164	2.14E-02	1.00	7174	2.14E-06	TRUE	TRUE	482,730	22.50
Sphingomyelin (d34:1) levels	Parkinson's disease	rs2894147	G	A	G	A	-7.61E-02	2.60E-02	3.74E-01	5.69E-01	FALSE	FALSE	10	73000943	2.26E-02	6.02E-01	TRUE	10	71241186	1.72E-02	1.00	7174	9.47E-06	TRUE	TRUE	482,730	19.64
Sphingomyelin (d34:1) levels	Parkinson's disease	rs3026120	A	G	A	G	-1.62E-01	-1.28E-02	7.46E-02	9.54E-01	FALSE	FALSE	17	5287415	4.14E-02	1.20E-01	TRUE	17	5384095	3.12E-02	1.00	7174	2.01E-07	TRUE	TRUE	482,730	27.08
Sphingomyelin (d34:1) levels	Parkinson's disease	rs34087767	G	C	G	C	5.98E-01	1.32E-01	5.41E-03	1.81E-02	TRUE	FALSE	2	182767178	9.47E-02	7.85E-01	TRUE	2	181902451	1.25E-01	0.87	7174	1.78E-06	TRUE	TRUE	482,730	22.85
Sphingomyelin (d34:1) levels	Parkinson's disease	rs35577358	G	A	G	A	1.26E-01	-2.18E-02	1.35E-01	8.08E-01	FALSE	FALSE	4	163464201	2.90E-02	3.44E-01	TRUE	4	162543049	2.46E-02	0.99	7174	2.84E-07	TRUE	TRUE	482,730	26.40
Sphingomyelin (d34:1) levels	Parkinson's disease	rs3912622	C	A	C	A	-9.27E-02	4.00E-03	2.00E-01	7.39E-01	FALSE	FALSE	11	103121473	2.20E-02	6.73E-02	TRUE	11	103250744	2.09E-02	1.00	7174	9.57E-06	TRUE	TRUE	482,730	19.62
Sphingomyelin (d34:1) levels	Parkinson's disease	rs4246215	T	G	T	G	-7.49E-02	-5.30E-03	4.36E-01	6.58E-01	FALSE	FALSE	11	61564299	1.79E-02	1.14E-01	TRUE	11	61796827	1.68E-02	0.99	7174	8.47E-06	TRUE	TRUE	482,730	19.86
Sphingomyelin (d34:1) levels	Parkinson's disease	rs4954960	C	T	C	T	8.42E-02	-1.30E-03	7.38E-01	3.23E-01	FALSE	FALSE	2	139574143	2.40E-02	1.87E-02	TRUE	2	138816573	1.90E-02	0.99	7174	9.90E-06	TRUE	TRUE	482,730	19.56
Sphingomyelin (d34:1) levels	Parkinson's disease	rs56108088	C	T	C	T	7.71E-02	-1.43E-02	5.63E-01	5.53E-01	FALSE	FALSE	14	87206069	1.78E-02	3.75E-01	TRUE	14	86739725	1.67E-02	0.99	7174	4.11E-06	TRUE	TRUE	482,730	21.25
Sphingomyelin (d34:1) levels	Parkinson's disease	rs7012814	A	G	A	G	7.99E-02	-3.76E-02	5.12E-01	5.23E-01	FALSE	FALSE	8	9173358	2.54E-02	8.59E-01	TRUE	8	9315848	1.68E-02	0.99	7174	2.09E-06	TRUE	TRUE	482,730	22.54
Sphingomyelin (d34:1) levels	Parkinson's disease	rs7032795	C	T	C	T	8.42E-02	-1.21E-02	4.81E-01	5.11E-01	FALSE	FALSE	9	117134945	1.70E-02	3.21E-01	TRUE	9	114372665	1.67E-02	1.00	7174	4.47E-07	TRUE	TRUE	482,730	25.52
Sphingomyelin (d34:1) levels	Parkinson's disease	rs722814	A	C	A	C	1.42E-01	-2.97E-02	7.89E-02	9.28E-01	FALSE	FALSE	14	70356168	4.40E-02	3.01E-01	TRUE	14	69889451	3.11E-02	0.99	7174	4.74E-06	TRUE	TRUE	482,730	20.97
Sphingomyelin (d34:1) levels	Parkinson's disease	rs72794954	T	C	T	C	-4.99E-01	5.13E-02	7.15E-03	9.80E-01	FALSE	FALSE	16	76376559	9.22E-02	2.38E-01	TRUE	16	76342662	1.05E-01	0.93	7174	1.93E-06	TRUE	TRUE	482,730	22.70
Sphingomyelin (d34:1) levels	Parkinson's disease	rs7350789	A	G	A	G	1.10E-01	1.67E-02	3.93E-01	6.61E-01	FALSE	FALSE	15	58679668	1.79E-02	4.57E-01	TRUE	15	58387469	1.69E-02	1.00	7174	6.71E-11	TRUE	TRUE	482,730	42.73
Sphingomyelin (d34:1) levels	Parkinson's disease	rs74915447	A	G	A	G	2.88E-01	5.94E-02	3.78E-02	9.71E-01	FALSE	FALSE	4	73765477	7.20E-02	3.88E-01	TRUE	4	72899760	4.42E-02	0.97	7174	7.50E-11	TRUE	TRUE	482,730	42.51
Sphingomyelin (d34:1) levels	Parkinson's disease	rs75679663	A	C	A	C	-3.04E-01	1.35E-01	2.58E-02	9.87E-01	FALSE	FALSE	17	4667972	9.72E-02	7.79E-01	TRUE	17	4764677	5.37E-02	0.98	7174	1.50E-08	TRUE	TRUE	482,730	32.12
Sphingomyelin (d34:1) levels	Parkinson's disease	rs7575840	T	G	T	G	1.00E-01	7.50E-03	2.96E-01	6.76E-01	FALSE	FALSE	2	21273490	1.85E-02	1.64E-01	TRUE	2	21050618	1.85E-02	1.00	7174	5.50E-08	TRUE	TRUE	482,730	29.60
Sphingomyelin (d34:1) levels	Parkinson's disease	rs78143047	G	A	G	A	3.43E-01	-7.18E-02	1.49E-02	9.71E-01	FALSE	FALSE	9	5527351	7.07E-02	5.08E-01	TRUE	9	5527351	7.05E-02	0.94	7174	1.19E-06	TRUE	TRUE	482,730	23.63
Sphingomyelin (d34:2) levels	Parkinson's disease	rs1033030	A	G	A	G	-1.05E-01	-8.84E-02	1.76E-01	8.15E-01	FALSE	FALSE	14	99775651	3.56E-02	6.51E-03	TRUE	14	99309314	2.22E-02	0.96	7174	2.16E-06	TRUE	TRUE	482,730	22.48
Sphingomyelin (d34:2) levels	Parkinson's disease	rs10471676	G	C	G	C	-9.19E-02	-3.30E-02	2.26E-01	2.47E-01	TRUE	FALSE	5	65580391	3.45E-02	4.71E-01	TRUE	5	66284563	2.04E-02	0.92	7174	6.75E-06	TRUE	TRUE	482,730	20.29
Sphingomyelin (d34:2) levels	Parkinson's disease	rs10867632	G	C	G	C	9.20E-02	-6.70E-02	2.07E-01	1.61E-01	TRUE	FALSE	9	83477078	3.06E-02	1.43E-02	TRUE	9	80862163	2.07E-02	0.99	7174	8.84E-06	TRUE	TRUE	482,730	19.78
Sphingomyelin (d34:2) levels	Parkinson's disease	rs10975312	A	G	A	G	2.37E-01	0.00E+00	3.12E-02	9.80E-01	FALSE	FALSE	9	5837780	8.29E-02	8.69E-05	TRUE	9	5837780	4.87E-02	0.98	7174	1.18E-06	TRUE	TRUE	482,730	23.65
Sphingomyelin (d34:2) levels	Parkinson's disease	rs111507730	C	T	C	T	1.71E-01	-6.51E-02	5.37E-02	9.44E-01	FALSE	FALSE	13	34328013	4.93E-02	7.29E-01	TRUE	13	33753876	3.67E-02	1.00	7174	3.33E-06	TRUE	TRUE	482,730	21.64
Sphingomyelin (d34:2) levels	Parkinson's disease	rs113127305	A	G	A	G	-2.17E-01	1.10E-03	4.98E-02	9.31E-01	FALSE	FALSE	10	72596921	4.27E-02	9.39E-03	TRUE	10	70837164	3.83E-02	1.00	7174	1.46E-08	TRUE	TRUE	482,730	32.18
Sphingomyelin (d34:2) levels	Parkinson's disease	rs11591147	T	G	T	G	-2.46E-01	-3.77E-02	3.32E-02	9.82E-01	FALSE	FALSE	1	55505647	6.83E-02	2.36E-01	TRUE	1	55039974	4.65E-02	1.00	7174	1.27E-07	TRUE	TRUE	482,730	27.97
Sphingomyelin (d34:2) levels	Parkinson's disease	rs116302332	T	C	T	C	2.98E-01	1.00E-01	2.81E-02	9.75E-01	FALSE	FALSE	4	75370891	8.11E-02	6.62E-01	TRUE	4	74505174	5.08E-02	0.96	7174	4.74E-09	TRUE	TRUE	482,730	34.37
Sphingomyelin (d34:2) levels	Parkinson's disease	rs117009181	C	T	C	T	1.10E-01	1.08E-02	1.50E-01	8.51E-01	FALSE	FALSE	12	125331024	2.85E-02	1.52E-01	TRUE	12	124846478	2.31E-02	0.98	7174	2.03E-06	TRUE	TRUE	482,730	22.60
Sphingomyelin (d34:2) levels	Parkinson's disease	rs12952341	T	C	T	C	8.53E-02	1.09E-02	3.26E-01	6.79E-01	FALSE	FALSE	17	4385503	2.59E-02	1.72E-01	TRUE	17	4482208	1.86E-02	0.90	7174	4.84E-06	TRUE	TRUE	482,730	20.93
Sphingomyelin (d34:2) levels	Parkinson's disease	rs1404846	C	T	C	T	6.96E-01	-5.68E-02	3.46E-03	9.78E-01	FALSE	FALSE	7	109770869	8.08E-02	3.17E-01	TRUE	7	110130812	1.54E-01	0.92	7174	6.24E-06	TRUE	TRUE	482,730	20.44
Sphingomyelin (d34:2) levels	Parkinson's disease	rs146136464	T	C	T	C	7.93E-01	1.48E-01	2.70E-03	9.81E-01	FALSE	FALSE	5	146763986	9.23E-02	9.65E-01	TRUE	5	147384423	1.76E-01	0.87	7174	6.79E-06	TRUE	TRUE	482,730	20.28
Sphingomyelin (d34:2) levels	Parkinson's disease	rs17276940	T	C	T	C	-2.14E-01	4.88E-02	3.35E-02	9.79E-01	FALSE	FALSE	14	20486115	8.48E-02	2.48E-01	TRUE	14	20017956	4.60E-02	0.99	7174	3.48E-06	TRUE	TRUE	482,730	21.56
Sphingomyelin (d34:2) levels	Parkinson's disease	rs174561	C	T	C	T	-1.70E-01	8.00E-04	3.81E-01	7.08E-01	FALSE	FALSE	11	61582708	1.85E-02	1.52E-02	TRUE	11	61815236	1.72E-02	1.00	7174	7.26E-23	TRUE	TRUE	482,730	97.59
Sphingomyelin (d34:2) levels	Parkinson's disease	rs2531191	T	C	T	C	3.71E-01	-1.03E-01	1.05E-02	9.91E-01	FALSE	FALSE	4	10617220	1.89E-01	2.32E-01	TRUE	4	10615596	8.33E-02	0.95	7174	8.23E-06	TRUE	TRUE	482,730	19.91
Sphingomyelin (d34:2) levels	Parkinson's disease	rs3912622	C	A	C	A	-9.29E-02	4.00E-03	2.00E-01	7.39E-01	FALSE	FALSE	11	103121473	2.20E-02	6.73E-02	TRUE	11	103250744	2.09E-02	1.00	7174	9.05E-06	TRUE	TRUE	482,730	19.73
Sphingomyelin (d34:2) levels	Parkinson's disease	rs56163454	G	C	G	C	2.67E-01	1.53E-01	2.02E-02	1.05E-02	TRUE	FALSE	1	44639367	1.60E-01	4.73E-01	TRUE	1	44173695	5.88E-02	0.99	7174	5.58E-06	TRUE	TRUE	482,730	20.66
Sphingomyelin (d34:2) levels	Parkinson's disease	rs62412280	T	A	T	A	-6.56E-01	-8.20E-02	3.91E-03	1.26E-02	TRUE	FALSE	6	68434598	1.55E-01	2.24E-01	TRUE	6	67724705	1.40E-01	0.90	7174	2.97E-06	TRUE	TRUE	482,730	21.87
Sphingomyelin (d34:2) levels	Parkinson's disease	rs7012814	A	G	A	G	8.66E-02	-3.76E-02	5.12E-01	5.23E-01	FALSE	FALSE	8	9173358	2.54E-02	8.59E-01	TRUE	8	9315848	1.68E-02	0.99	7174	2.67E-07	TRUE	TRUE	482,730	26.52
Sphingomyelin (d34:2) levels	Parkinson's disease	rs74915447	A	G	A	G	1.96E-01	5.94E-02	3.78E-02	9.71E-01	FALSE	FALSE	4	73765477	7.20E-02	3.88E-01	TRUE	4	72899760	4.43E-02	0.97	7174	9.99E-06	TRUE	TRUE	482,730	19.54
Sphingomyelin (d34:2) levels	Parkinson's disease	rs939971	A	C	A	C	-9.62E-02	-3.89E-02	2.11E-01	6.66E-01	FALSE	FALSE	15	80322269	2.35E-02	4.89E-02	TRUE	15	80029927	2.04E-02	1.00	7174	2.43E-06	TRUE	TRUE	482,730	22.26
Sphingomyelin (d34:2) levels	Parkinson's disease	rs9835223	G	C	G	C	-9.53E-02	2.23E-02	8.01E-01	8.29E-01	TRUE	FALSE	3	186586284	3.11E-02	3.25E-01	TRUE	3	186868495	2.11E-02	0.96	7174	6.54E-06	TRUE	TRUE	482,730	20.35
Sphingomyelin (d36:1) levels	Parkinson's disease	rs10838826	T	G	T	G	9.16E-02	-6.30E-03	7.83E-01	2.93E-01	FALSE	FALSE	11	48219812	1.85E-02	1.34E-01	TRUE	11	48198260	2.01E-02	1.00	7174	5.13E-06	TRUE	TRUE	482,730	20.82
Sphingomyelin (d36:1) levels	Parkinson's disease	rs112585713	A	G	A	G	2.82E-01	1.07E-01	3.16E-02	9.80E-01	FALSE	FALSE	4	73742418	9.00E-02	6.32E-01	TRUE	4	72876701	4.80E-02	0.98	7174	4.41E-09	TRUE	TRUE	482,730	34.51
Sphingomyelin (d36:1) levels	Parkinson's disease	rs114839471	G	A	G	A	-4.45E-01	5.45E-02	8.23E-03	9.87E-01	FALSE	FALSE	5	89545035	1.13E-01	2.01E-01	TRUE	5	90249218	9.46E-02	0.96	7174	2.59E-06	TRUE	TRUE	482,730	22.14
Sphingomyelin (d36:1) levels	Parkinson's disease	rs11591147	T	G	T	G	-2.59E-01	-3.77E-02	3.32E-02	9.82E-01	FALSE	FALSE	1	55505647	6.83E-02	2.36E-01	TRUE	1	55039974	4.66E-02	1.00	7174	2.96E-08	TRUE	TRUE	482,730	30.80
Sphingomyelin (d36:1) levels	Parkinson's disease	rs1169306	T	C	T	C	-8.53E-02	2.60E-03	3.60E-01	6.36E-01	FALSE	FALSE	12	121438311	1.75E-02	5.42E-02	TRUE	12	121000508	1.74E-02	1.00	7174	9.27E-07	TRUE	TRUE	482,730	24.11
Sphingomyelin (d36:1) levels	Parkinson's disease	rs117442205	A	G	A	G	6.09E-01	9.65E-02	3.87E-03	9.87E-01	FALSE	FALSE	11	4988071	9.84E-02	4.86E-01	TRUE	11	4966841	1.38E-01	0.93	7174	9.71E-06	TRUE	TRUE	482,730	19.59
Sphingomyelin (d36:1) levels	Parkinson's disease	rs117643293	T	C	T	C	-1.71E-01	9.73E-02	6.20E-02	9.71E-01	FALSE	FALSE	17	4968964	5.79E-02	4.64E-02	TRUE	17	5065669	3.41E-02	0.99	7174	5.50E-07	TRUE	TRUE	482,730	25.12
Sphingomyelin (d36:1) levels	Parkinson's disease	rs11779209	G	T	G	T	1.14E-01	3.22E-02	1.26E-01	8.44E-01	FALSE	FALSE	8	71985773	3.17E-02	5.09E-01	TRUE	8	71073538	2.56E-02	0.97	7174	9.39E-06	TRUE	TRUE	482,730	19.66
Sphingomyelin (d36:1) levels	Parkinson's disease	rs12464353	A	G	A	G	2.29E-01	4.56E-01	3.26E-02	9.76E-01	FALSE	FALSE	2	61210028	3.12E-01	8.45E-01	TRUE	2	60982893	4.79E-02	0.99	7174	1.74E-06	TRUE	TRUE	482,730	22.90
Sphingomyelin (d36:1) levels	Parkinson's disease	rs138524673	A	G	A	G	1.93E-01	-1.26E-01	4.49E-02	9.81E-01	FALSE	FALSE	15	68673137	1.21E-01	5.26E-01	TRUE	15	68380799	4.24E-02	0.92	7174	5.75E-06	TRUE	TRUE	482,730	20.60
Sphingomyelin (d36:1) levels	Parkinson's disease	rs1452773	G	T	G	T	-1.17E-01	4.96E-02	1.38E-01	8.61E-01	FALSE	FALSE	2	50277949	3.27E-02	8.89E-01	TRUE	2	50050811	2.40E-02	0.99	7174	1.10E-06	TRUE	TRUE	482,730	23.78
Sphingomyelin (d36:1) levels	Parkinson's disease	rs17664981	C	T	C	T	1.67E-01	-3.90E-03	5.74E-02	9.47E-01	FALSE	FALSE	6	13187603	3.96E-02	3.55E-02	TRUE	6	13187371	3.67E-02	0.96	7174	5.34E-06	TRUE	TRUE	482,730	20.74
Sphingomyelin (d36:1) levels	Parkinson's disease	rs182695896	C	A	C	A	3.39E-01	1.53E-01	2.53E-02	9.84E-01	FALSE	FALSE	4	74813227	1.83E-01	3.94E-01	TRUE	4	73947510	5.29E-02	0.96	7174	1.58E-10	TRUE	TRUE	482,730	41.04
Sphingomyelin (d36:1) levels	Parkinson's disease	rs186039163	G	T	G	T	-4.16E-01	-2.60E-02	1.01E-02	9.88E-01	FALSE	FALSE	17	3450893	1.46E-01	6.63E-02	TRUE	17	3547599	8.40E-02	0.97	7174	7.74E-07	TRUE	TRUE	482,730	24.46
Sphingomyelin (d36:1) levels	Parkinson's disease	rs3742023	T	C	T	C	-7.66E-02	-1.68E-02	4.23E-01	6.33E-01	FALSE	FALSE	12	109693982	1.97E-02	4.05E-01	TRUE	12	109256177	1.69E-02	1.00	7174	5.63E-06	TRUE	TRUE	482,730	20.64
Sphingomyelin (d36:1) levels	Parkinson's disease	rs4144237	G	A	G	A	-1.13E-01	-2.88E-02	1.26E-01	8.12E-01	FALSE	FALSE	6	2365268	2.94E-02	4.85E-01	TRUE	6	2365034	2.53E-02	0.98	7174	8.50E-06	TRUE	TRUE	482,730	19.85
Sphingomyelin (d36:1) levels	Parkinson's disease	rs4246215	T	G	T	G	-7.48E-02	-5.30E-03	4.36E-01	6.58E-01	FALSE	FALSE	11	61564299	1.79E-02	1.14E-01	TRUE	11	61796827	1.68E-02	0.99	7174	8.88E-06	TRUE	TRUE	482,730	19.77

Sphingomyelin (d36:1) levels	Parkinson's disease	rs4981443	A	G	A	G	-7.71E-02	2.40E-03	3.60E-01	6.87E-01	FALSE	FALSE	14	23300663	1.84E-02	4.69E-02	TRUE	14	22831454	1.74E-02	1.00	7174	9.63E-06	TRUE	TRUE	482,730	19.61
Sphingomyelin (d36:1) levels	Parkinson's disease	rs581080	C	G	C	G	1.14E-01	3.05E-02	8.56E-01	8.10E-01	TRUE	FALSE	9	15305378	2.15E-02	8.05E-01	TRUE	9	15305380	2.37E-02	1.00	7174	1.47E-06	TRUE	TRUE	482,730	23.23
Sphingomyelin (d36:1) levels	Parkinson's disease	rs6558122	C	T	C	T	-1.40E-01	-7.00E-04	1.01E-01	8.14E-01	FALSE	FALSE	8	29375767	2.85E-02	9.22E-03	TRUE	8	29518250	2.77E-02	0.99	7174	4.58E-07	TRUE	TRUE	482,730	25.48
Sphingomyelin (d36:1) levels	Parkinson's disease	rs6829637	G	A	G	A	-7.87E-02	-3.08E-02	4.16E-01	6.36E-01	FALSE	FALSE	4	111326909	2.44E-02	6.83E-01	TRUE	4	110405753	1.72E-02	0.97	7174	4.77E-06	TRUE	TRUE	482,730	20.96
Sphingomyelin (d36:1) levels	Parkinson's disease	rs7157785	T	G	T	G	1.36E-01	-3.20E-02	1.25E-01	8.32E-01	FALSE	FALSE	14	64235556	2.25E-02	8.11E-01	TRUE	14	63768838	2.51E-02	1.00	7174	6.56E-08	TRUE	TRUE	482,730	29.25
Sphingomyelin (d36:1) levels	Parkinson's disease	rs72898481	T	C	T	C	2.86E-01	1.52E-02	1.93E-02	9.72E-01	FALSE	FALSE	17	75751850	9.02E-02	6.23E-02	TRUE	17	77755768	6.35E-02	0.91	7174	6.72E-06	TRUE	TRUE	482,730	20.30
Sphingomyelin (d36:1) levels	Parkinson's disease	rs73073606	C	A	C	A	-9.00E-02	2.07E-02	2.41E-01	6.67E-01	FALSE	FALSE	3	193894366	2.49E-02	3.91E-01	TRUE	3	194176577	2.02E-02	0.94	7174	8.25E-06	TRUE	TRUE	482,730	19.91
Sphingomyelin (d36:1) levels	Parkinson's disease	rs75679663	A	C	A	C	-3.25E-01	1.35E-01	2.58E-02	9.87E-01	FALSE	FALSE	17	4667972	9.72E-02	7.79E-01	TRUE	17	4764677	5.37E-02	0.98	7174	1.42E-09	TRUE	TRUE	482,730	36.73
Sphingomyelin (d36:1) levels	Parkinson's disease	rs79146711	T	C	T	C	2.34E-01	-6.90E-03	4.51E-02	9.76E-01	FALSE	FALSE	4	74290628	8.45E-02	2.92E-02	TRUE	4	73424911	4.09E-02	0.94	7174	1.02E-08	TRUE	TRUE	482,730	32.88
Sphingomyelin (d36:1) levels	Parkinson's disease	rs884532	T	C	T	C	-7.53E-02	1.68E-02	5.10E-01	5.07E-01	FALSE	FALSE	5	173666484	2.04E-02	3.87E-01	TRUE	5	174239481	1.67E-02	1.00	7174	6.95E-06	TRUE	TRUE	482,730	20.24
Sphingomyelin (d36:2) levels	Parkinson's disease	rs117546336	G	T	G	T	-1.90E-01	-6.76E-02	4.73E-02	9.64E-01	FALSE	FALSE	6	98545963	6.63E-02	5.11E-01	TRUE	6	98098087	3.99E-02	0.96	7173	2.00E-06	TRUE	TRUE	482,730	22.63
Sphingomyelin (d36:2) levels	Parkinson's disease	rs12207488	A	G	A	G	-9.64E-02	-2.94E-02	1.82E-01	7.35E-01	FALSE	FALSE	6	10952336	2.12E-02	7.79E-01	TRUE	6	10952103	2.16E-02	1.00	7173	8.21E-06	TRUE	TRUE	482,730	19.92
Sphingomyelin (d36:2) levels	Parkinson's disease	rs12464353	A	G	A	G	2.21E-01	4.56E-01	3.26E-02	9.76E-01	FALSE	FALSE	2	61210028	3.12E-01	8.45E-01	TRUE	2	60982893	4.79E-02	0.99	7173	4.04E-06	TRUE	TRUE	482,730	21.28
Sphingomyelin (d36:2) levels	Parkinson's disease	rs1452773	G	T	G	T	-1.09E-01	4.96E-02	1.38E-01	8.61E-01	FALSE	FALSE	2	50277949	3.27E-02	8.89E-01	TRUE	2	50050811	2.40E-02	0.99	7173	5.98E-06	TRUE	TRUE	482,730	20.52
Sphingomyelin (d36:2) levels	Parkinson's disease	rs146253188	A	G	A	G	-2.88E-01	1.48E-01	1.68E-02	9.91E-01	FALSE	FALSE	5	35226066	1.84E-01	3.78E-01	TRUE	5	35225964	6.45E-02	1.00	7173	8.07E-06	TRUE	TRUE	482,730	19.95
Sphingomyelin (d36:2) levels	Parkinson's disease	rs146465816	A	G	A	G	-3.61E-01	6.27E-02	1.09E-02	9.75E-01	FALSE	FALSE	3	19365490	7.76E-02	3.78E-01	TRUE	3	19323998	8.08E-02	0.96	7173	8.07E-06	TRUE	TRUE	482,730	19.95
Sphingomyelin (d36:2) levels	Parkinson's disease	rs17276940	T	C	T	C	-2.19E-01	4.88E-02	3.35E-02	9.79E-01	FALSE	FALSE	14	20486115	8.48E-02	2.48E-01	TRUE	14	20017956	4.61E-02	0.99	7173	2.12E-06	TRUE	TRUE	482,730	22.52
Sphingomyelin (d36:2) levels	Parkinson's disease	rs174544	A	C	A	C	-1.78E-01	-9.00E-04	3.80E-01	7.09E-01	FALSE	FALSE	11	61567753	1.85E-02	1.67E-02	TRUE	11	61800281	1.72E-02	1.00	7173	5.30E-25	TRUE	TRUE	482,730	107.46
Sphingomyelin (d36:2) levels	Parkinson's disease	rs182695896	C	A	C	A	3.03E-01	1.53E-01	2.53E-02	9.84E-01	FALSE	FALSE	4	74813227	1.83E-01	3.94E-01	TRUE	4	73947510	5.30E-02	0.96	7173	1.12E-08	TRUE	TRUE	482,730	32.69
Sphingomyelin (d36:2) levels	Parkinson's disease	rs313950	A	G	A	G	-7.65E-02	-1.96E-02	3.96E-01	5.94E-01	FALSE	FALSE	4	114021169	2.25E-02	4.17E-01	TRUE	4	113100013	1.70E-02	1.00	7173	7.32E-06	TRUE	TRUE	482,730	20.14
Sphingomyelin (d36:2) levels	Parkinson's disease	rs581080	C	G	C	G	1.08E-01	3.05E-02	8.56E-01	8.10E-01	TRUE	FALSE	9	15305378	2.15E-02	8.05E-01	TRUE	9	15305380	2.37E-02	1.00	7173	5.20E-06	TRUE	TRUE	482,730	20.79
Sphingomyelin (d36:2) levels	Parkinson's disease	rs6444176	T	C	T	C	1.75E-01	9.82E-02	5.20E-02	9.72E-01	FALSE	FALSE	3	186586060	6.39E-02	9.04E-01	TRUE	3	186868271	3.76E-02	0.96	7173	3.22E-06	TRUE	TRUE	482,730	21.72
Sphingomyelin (d36:2) levels	Parkinson's disease	rs7700432	A	G	A	G	-7.95E-02	5.90E-03	3.54E-01	7.26E-01	FALSE	FALSE	5	130194694	2.13E-02	1.07E-01	TRUE	5	130859001	1.73E-02	1.00	7173	4.55E-06	TRUE	TRUE	482,730	21.05
Sphingomyelin (d36:2) levels	Parkinson's disease	rs77645768	A	G	A	G	2.43E-01	5.69E-02	2.56E-02	9.74E-01	FALSE	FALSE	4	73668828	7.75E-02	3.34E-01	TRUE	4	72803111	5.38E-02	0.97	7173	6.45E-06	TRUE	TRUE	482,730	20.38
Sphingomyelin (d36:2) levels	Parkinson's disease	rs78999781	C	T	C	T	-1.48E-01	-4.01E-02	9.10E-02	8.89E-01	FALSE	FALSE	2	204290037	2.99E-02	7.44E-01	TRUE	2	203425314	2.91E-02	1.00	7173	3.95E-07	TRUE	TRUE	482,730	25.76
Sphingomyelin (d36:2) levels	Parkinson's disease	rs7920200	A	G	A	G	-1.88E-01	-4.80E-02	4.19E-02	9.20E-01	FALSE	FALSE	10	72659304	4.27E-02	5.84E-01	TRUE	10	70899547	4.23E-02	0.96	7173	8.75E-06	TRUE	TRUE	482,730	19.79
Sphingomyelin (d36:2) levels	Parkinson's disease	rs9738226	G	A	G	A	1.01E-01	7.40E-03	5.79E-01	3.92E-01	FALSE	FALSE	12	121423659	1.72E-02	1.76E-01	TRUE	12	120985856	1.69E-02	1.00	7173	2.37E-09	TRUE	TRUE	482,730	35.73
Sphingomyelin (d38:1) levels	Parkinson's disease	rs114483871	T	C	T	C	2.68E-01	-2.94E-02	3.81E-02	9.77E-01	FALSE	FALSE	4	73990168	8.47E-02	1.38E-01	TRUE	4	73124451	4.48E-02	0.93	7174	2.19E-09	TRUE	TRUE	482,730	35.89
Sphingomyelin (d38:1) levels	Parkinson's disease	rs11591147	T	G	T	G	-3.01E-01	-3.77E-02	3.32E-02	9.82E-01	FALSE	FALSE	1	55505647	6.83E-02	2.36E-01	TRUE	1	55039974	4.65E-02	1.00	7174	1.07E-10	TRUE	TRUE	482,730	41.81
Sphingomyelin (d38:1) levels	Parkinson's disease	rs117146578	C	T	C	T	1.83E-01	-1.42E-01	4.82E-02	9.31E-01	FALSE	FALSE	12	102316763	3.85E-02	1.18E-04	TRUE	12	101922985	3.92E-02	1.00	7174	3.27E-06	TRUE	TRUE	482,730	21.68
Sphingomyelin (d38:1) levels	Parkinson's disease	rs117643293	T	C	T	C	-2.43E-01	9.73E-02	6.20E-02	9.71E-01	FALSE	FALSE	17	4968964	5.79E-02	4.64E-02	TRUE	17	5065669	3.39E-02	0.99	7174	8.25E-13	TRUE	TRUE	482,730	51.41
Sphingomyelin (d38:1) levels	Parkinson's disease	rs118070067	A	C	A	C	2.58E-01	5.38E-02	2.22E-02	9.74E-01	FALSE	FALSE	12	61644723	8.01E-02	3.00E-01	TRUE	12	61250942	5.74E-02	0.93	7174	7.31E-06	TRUE	TRUE	482,730	20.14
Sphingomyelin (d38:1) levels	Parkinson's disease	rs13258507	G	T	G	T	9.26E-02	-1.41E-02	2.41E-01	8.17E-01	FALSE	FALSE	8	126492597	2.24E-02	2.77E-01	TRUE	8	125480355	1.94E-02	1.00	7174	1.92E-06	TRUE	TRUE	482,730	22.70
Sphingomyelin (d38:1) levels	Parkinson's disease	rs1452773	G	T	G	T	-1.10E-01	4.96E-02	1.38E-01	8.61E-01	FALSE	FALSE	2	50277949	3.27E-02	8.89E-01	TRUE	2	50050811	2.39E-02	0.99	7174	4.26E-06	TRUE	TRUE	482,730	21.18
Sphingomyelin (d38:1) levels	Parkinson's disease	rs147426822	T	C	T	C	3.59E-01	2.09E-02	1.32E-02	9.71E-01	FALSE	FALSE	8	23817383	7.75E-02	1.04E-01	TRUE	8	23959870	7.86E-02	0.88	7174	4.97E-06	TRUE	TRUE	482,730	20.88
Sphingomyelin (d38:1) levels	Parkinson's disease	rs182695896	C	A	C	A	3.71E-01	1.53E-01	2.53E-02	9.84E-01	FALSE	FALSE	4	74813227	1.83E-01	3.94E-01	TRUE	4	73947510	5.28E-02	0.96	7174	2.53E-12	TRUE	TRUE	482,730	49.20
Sphingomyelin (d38:1) levels	Parkinson's disease	rs186039163	G	T	G	T	-3.81E-01	-2.60E-02	1.01E-02	9.88E-01	FALSE	FALSE	17	3450893	1.46E-01	6.63E-02	TRUE	17	3547599	8.40E-02	0.97	7174	5.68E-06	TRUE	TRUE	482,730	20.62
Sphingomyelin (d38:1) levels	Parkinson's disease	rs188346987	G	C	G	C	-1.78E-01	-1.93E-02	5.12E-02	3.47E-02	TRUE	FALSE	2	157953943	7.37E-02	1.01E-01	TRUE	2	157097431	3.95E-02	0.91	7174	6.67E-06	TRUE	TRUE	482,730	20.32
Sphingomyelin (d38:1) levels	Parkinson's disease	rs2867985	A	G	A	G	8.20E-02	1.07E-02	3.05E-01	6.70E-01	FALSE	FALSE	15	78363775	2.04E-02	2.21E-01	TRUE	15	78071433	1.82E-02	1.00	7174	6.90E-06	TRUE	TRUE	482,730	20.25
Sphingomyelin (d38:1) levels	Parkinson's disease	rs3741111	G	A	G	A	8.64E-02	-5.00E-04	4.83E-01	5.00E-01	FALSE	FALSE	11	123947295	1.95E-02	8.69E-03	TRUE	11	124076588	1.67E-02	0.99	7174	2.19E-07	TRUE	TRUE	482,730	26.91
Sphingomyelin (d38:1) levels	Parkinson's disease	rs715119	T	C	T	C	-9.19E-02	6.90E-03	2.33E-01	7.62E-01	FALSE	FALSE	9	107737393	2.62E-02	1.01E-01	TRUE	9	104975112	1.97E-02	1.00	7174	3.11E-06	TRUE	TRUE	482,730	21.78
Sphingomyelin (d38:1) levels	Parkinson's disease	rs7157785	T	G	T	G	3.70E-01	-3.20E-02	1.25E-01	8.32E-01	FALSE	FALSE	14	64235556	2.25E-02	8.11E-01	TRUE	14	63768838	2.48E-02	1.00	7174	1.11E-49	TRUE	TRUE	482,730	223.06
Sphingomyelin (d38:1) levels	Parkinson's disease	rs7172051	G	A	G	A	7.71E-02	-6.60E-03	3.73E-01	5.68E-01	FALSE	FALSE	15	70288315	2.29E-02	1.11E-01	TRUE	15	69995976	1.74E-02	0.97	7174	9.32E-06	TRUE	TRUE	482,730	19.67
Sphingomyelin (d38:1) levels	Parkinson's disease	rs73073606	C	A	C	A	-9.65E-02	2.07E-02	2.41E-01	6.67E-01	FALSE	FALSE	3	193894366	2.49E-02	3.91E-01	TRUE	3	194176577	2.01E-02	0.94	7174	1.68E-06	TRUE	TRUE	482,730	22.96
Sphingomyelin (d38:1) levels	Parkinson's disease	rs73193962	A	C	A	C	-2.43E-01	2.13E-02	2.60E-02	9.69E-01	FALSE	FALSE	3	105693782	6.98E-02	1.19E-01	TRUE	3	105974935	5.39E-02	0.95	7174	6.41E-06	TRUE	TRUE	482,730	20.39
Sphingomyelin (d38:1) levels	Parkinson's disease	rs7478167	A	T	A	T	-8.30E-02	-3.50E-03	6.69E-01	6.66E-01	TRUE	FALSE	10	103476258	1.91E-02	6.80E-02	TRUE	10	101716501	1.78E-02	0.99	7174	3.36E-06	TRUE	TRUE	482,730	21.63
Sphingomyelin (d38:1) levels	Parkinson's disease	rs75679663	A	C	A	C	-4.74E-01	1.35E-01	2.58E-02	9.87E-01	FALSE	FALSE	17	4667972	9.72E-02	7.79E-01	TRUE	17	4764677	5.35E-02	0.98	7174	9.06E-19	TRUE	TRUE	482,730	78.68
Sphingomyelin (d38:1) levels	Parkinson's disease	rs75740927	G	C	G	C	2.30E-01	-2.02E-02	3.07E-02	1.80E-02	TRUE	FALSE	11	95347575	9.58E-02	7.94E-02	TRUE	11	95614411	5.11E-02	0.89	7174	7.25E-06	TRUE	TRUE	482,730	20.15
Sphingomyelin (d38:1) levels	Parkinson's disease	rs76174849	T	C	T	C	3.34E-01	-2.82E-02	1.40E-02	9.79E-01	FALSE	FALSE	1	8798765	8.49E-02	1.31E-01	TRUE	1	8738706	7.19E-02	0.97	7174	3.44E-06	TRUE	TRUE	482,730	21.59
Sphingomyelin (d38:1) levels	Parkinson's disease	rs77645768	A	G	A	G	3.04E-01	5.69E-02	2.56E-02	9.74E-01	FALSE	FALSE	4	73668828	7.75E-02	3.34E-01	TRUE	4	72803111	5.36E-02	0.97	7174	1.53E-08	TRUE	TRUE	482,730	32.09
Sphingomyelin (d38:1) levels	Parkinson's disease	rs79231873	G	A	G	A	2.94E-01	6.94E-02	1.98E-02	9.73E-01	FALSE	FALSE	8	54064174	7.57E-02	4.44E-01	TRUE	8	53151614	6.55E-02	0.83	7174	7.37E-06	TRUE	TRUE	482,730	20.12
Sphingomyelin (d38:1) levels	Parkinson's disease	rs7970512	A	C	A	C	-2.25E-01	7.19E-02	3.50E-02	9.81E-01	FALSE	FALSE	12	67907600	1.01E-01	3.23E-01	TRUE	12	67513820	4.78E-02	0.87	7174	2.46E-06	TRUE	TRUE	482,730	22.23
Sphingomyelin (d38:2) levels	Parkinson's disease	rs10206976	T	G	T	G	9.05E-02	-6.00E-04	2.68E-01	8.24E-01	FALSE	FALSE	2	211614638	2.48E-02	8.91E-03	TRUE	2	210749914	1.90E-02	1.00	7150	1.88E-06	TRUE	TRUE	482,730	22.75
Sphingomyelin (d38:2) levels	Parkinson's disease	rs113118892	T	C	T	C	3.83E-01	-7.96E-02	1.23E-02	9.88E-01	FALSE	FALSE	15	59566385	1.69E-01	1.96E-01	TRUE	15	59274186	7.97E-02	0.91	7150	1.58E-06	TRUE	TRUE	482,730	23.09
Sphingomyelin (d38:2) levels	Parkinson's disease	rs114483871	T	C	T	C	2.20E-01	-2.94E-02	3.81E-02	9.77E-01	FALSE	FALSE	4	73990168	8.47E-02	1.38E-01	TRUE	4	73124451	4.49E-02	0.93	7150	9.38E-07	TRUE	TRUE	482,730	24.09
Sphingomyelin (d38:2) levels	Parkinson's disease	rs114717988	A	T	A	T	1.97E-01	2.60E-02	4.57E-02	4.97E-02	TRUE	FALSE	6	48755681	5.05E-02	2.17E-01	TRUE	6	48788044	3.96E-02	0.99	7150	6.37E-07	TRUE	TRUE	482,730	24.84
Sphingomyelin (d38:2) levels	Parkinson's disease	rs11591147	T	G	T	G	-2.19E-01	-3.77E-02	3.32E-02	9.82E-01	FALSE	FALSE	1	55505647	6.83E-02	2.36E-01	TRUE	1	55039974	4.66E-02	1.00	7150	2.84E-06	TRUE	TRUE	482,730	21.95
Sphingomyelin (d38:2) levels	Parkinson's disease	rs116411822	G	A	G	A	-4.20E-01	-3.65E-01	8.15E-03	9.92E-01	FALSE	FALSE	2	46721260	1.96E-01	3.10E-02	TRUE	2	46494121	9.37E-02	0.97	7150	7.29E-06	TRUE	TRUE	482,730	20.14
Sphingomyelin (d38:2) levels	Parkinson's disease	rs1169306	T	C	T	C	-1.21E-01	2.60E-03	3.60E-01	6.36E-01	FALSE	FALSE	12	121438311	1.75E-02	5.42E-02	TRUE	12	121000508	1.74E-02	1.00	7150	4.14E-12	TRUE	TRUE	482,730	48.22
Sphingomyelin (d38:2) levels	Parkinson's disease	rs139150589	T	C	T	C	5.21E-01	1.81E-01	7.71E-03	9.93E-01	FALSE	FALSE	10	72219604	2.04E-01	4.26E-01	TRUE	10	70459848	1.00E-01	0.91	7150	1.87E-07	TRUE	TRUE	482,730	27.21
Sphingomyelin (d38:2) levels	Parkinson's disease	rs146723536	A	G	A	G	-2.56E-01	-1.80E-01	2.19E-02	9.86E-01	FALSE	FALSE	16	12241416	1.33E-01	7.54E-01	TRUE	16	12147559	5.75E-02	0.96	7150	8.40E-06	TRUE	TRUE	482,730	19.87
Sphingomyelin (d38:2) levels	Parkinson's disease	rs17276940	T	C	T	C	-2.45E-01	4.88E-02	3.35E-02	9.79E-01	FALSE	FALSE	14	20486115	8.48E-02	2.48E-01	TRUE	14	20017956	4.61E-02	0.99	7150	1.02E-07	TRUE	TRUE	482,730	28.39
Sphingomyelin (d38:2) levels	Parkinson's disease	rs174556	T	C	T	C	-1.44E-01	1.00E-04	3.80E-01	7.09E-01	FALSE	FALSE	11	61580635	1.85E-02	1.04E-03	TRUE	11	61813163	1.72E-02	1.00	7150	6.46E-17	TRUE	TRUE	482,730	70.19
Sphingomyelin (d38:2) levels	Parkinson's disease	rs182695896	C	A	C	A	3.26E-01	1.53E-01	2.53E-02	9.84E-01	FALSE	FALSE	4	74813227	1.83E-01	3.94E-01	TRUE	4	73947510	5.30E-02	0.96	7150	8.57E-10	TRUE	TRUE	482,730	37.73
Sphingomyelin (d38:2) levels	Parkinson's disease	rs3936109	G	A	G	A	-1.04E-01	1.66E-02	1.80E-01	7.94E-01	FALSE	FALSE	3	184341438	2.80E-02	2.56E-01	TRUE	3	184623650	2.21E-02	0.98	7150	2.72E-06	TRUE	TRUE	482,730	22.04
Sphingomyelin (d38:2) levels	Parkinson's disease	rs581080	C	G	C	G	1.15E-01	3.05E-02	8.56E-01	8.10E-01	TRUE	FALSE	9	15305378	2.15E-02	8.05E-01	TRUE	9	15305380	2.37E-02	1.00	7150	1.19E-06	TRUE	TRUE	482,730	23.63
Sphingomyelin (d38:2) levels	Parkinson's disease	rs657315	T	C	T	C	8.17E-02	-2.21E-02	2.84E-01	7.92E-01	FALSE	FALSE	11	69332712	2.07E-02	5.44E-01	TRUE	11	69517944	1.84E-02	1.00	7150	8.75E-06	TRUE	TRUE	482,730	19.79
Sphingomyelin (d38:2) levels	Parkinson's disease	rs672959	G	A	G	A	-4.59E-01	1.08E-01	7.87E-03	9.85E-01	FALSE	FALSE	10	14460294	1.04E-01	5.22E-01	TRUE	10	14418295	1.00E-01	0.89	7150	4.67E-06	TRUE	TRUE	482,730	21.00
Sphingomyelin (d38:2) levels	Parkinson's disease	rs72794954	T	C	T	C	-4.90E-01	5.13E-02	7.15E-03	9.80E-01	FALSE	FALSE	16	76376559	9.22E-02	2.38E-01	TRUE	16	76342662	1.05E-01	0.93	7150	3.02E-06	TRUE	TRUE	482,730	21.84
Sphingomyelin (d38:2) levels	Parkinson's disease	rs72839716	A	G	A	G	-1.08E-01	1.35E-02	1.59E-01	8.44E-01	FALSE	FALSE	10	124592971	2.73E-02	2.06E-01	TRUE	10	122833455	2.29E-02	0.98	7150	2.53E-06	TRUE	TRUE	482,730	22.18
Sphingomyelin (d38:2) levels	Parkinson's disease	rs75038600	C	G	C	G	7.26E-01	1.44E-02	3.10E-03	2.54E-02	TRUE	FALSE	12	12494616	7.80E-02	6.85E-02	TRUE	12	12341682	1.60E-01	0.85	7150	5.94E-06	TRUE	TRUE	482,730	20.54
Sphingomyelin (d38:2) levels	Parkinson's disease	rs75098294	G	A	G	A	5.27E-01	1.22E-01	6.17E-03	9.81E-01	FALSE	FALSE	13	63562778	9.34E-02	7.16E-01	TRUE	13	62988645	1.07E-01	0.95	7150	9.06E-07	TRUE	TRUE	482,730	24.16
Sphingomyelin (d38:2) levels	Parkinson's disease	rs77645768	A	G	A	G	2.68E-01	5.69E-02	2.56E-02	9.74E-01	FALSE	FALSE	4	73668828	7.75E-02	3.34E-01	TRUE	4	72803111	5.38E-02	0.97	7150	6.16E-07	TRUE	TRUE	482,730	24.90
Sphingomyelin (d40:1) levels	Parkinson's disease	rs10927285	G	C	G	C	8.23E-02	-1.84E-02	3.27E-01	3.21E-01	TRUE	FALSE	1	236188283	1.91E-02	4.72E-01	TRUE	1	236024983	1.76E-02	0.99	7174	2.86E-06	TRUE	TRUE	482,730	21.94
Sphingomyelin (d40:1) levels	Parkinson's disease	rs11050950	T	G	T	G	1.47E-01	-1.79E-01	6.97E-02	9.73E-01	FALSE	FALSE	12	30710636	6.90E-02	4.70E-03	TRUE	12	30557703	3.24E-02	0.98	7174	6.29E-06	TRUE	TRUE	482,730	20.43
Sphingomyelin (d40:1) levels	Parkinson's disease	rs113118892	T	C	T	C	3.59E-01	-7.96E-02	1.23E-02	9.88E-01	FALSE	FALSE	15	59566385	1.69E-01	1.96E-01	TRUE	15	59274186	7.93E-02	0.91	7174	6.26E-06	TRUE	TRUE	482,730	20.44
Sphingomyelin (d40:1) levels	Parkinson's disease	rs113318071	T	C	T	C	1.62E-01	-1.06E-01	6.32E-02	9.74E-01	FALSE	FALSE	11	46870078	8.51E-02	6.69E-01	TRUE	11	46848527	3.42E-02	0.97	7174	2.33E-06	TRUE	TRUE	482,730	22.34
Sphingomyelin (d40:1) levels	Parkinson's disease	rs11591147	T	G	T	G	-3.32E-01	-3.77E-02	3.32E-02	9.82E-01	FALSE	FALSE	1	55505647	6.83E-02	2.36E-01	TRUE	1	55039974	4.65E-02	1.00	7174	1.05E-12	TRUE	TRUE	482,730	50.93
Sphingomyelin (d40:1) levels	Parkinson's disease	rs117146578	C	T	C	T	1.79E-01	-1.42E-01	4.82E-02	9.31E-01	FALSE	FALSE	12	102316763	3.85E-02	1.18E-04	TRUE	12	101922985	3.92E-02	1.00	7174	5.15E-06	TRUE	TRUE	482,730	20.81
Sphingomyelin (d40:1) levels	Parkinson's disease	rs117643293	T	C	T	C	-3.25E-01	9.73E-02	6.20E-02	9.71E-01	FALSE	FALSE	17	4968964	5.79E-02	4.64E-02	TRUE	17	5065669	3.39E-02	0.99	7174	1.08E-21	TRUE	TRUE	482,730	92.15
Sphingomyelin (d40:1) levels	Parkinson's disease	rs138255545	A	G	A	G	2.11E-01	-6.14E-02	3.31E-02	9.76E-01	FALSE	FALSE	11	22349283	7.58E-02	3.79E-01	TRUE	11	22327737	4.74E-02	0.97	7174	8.58E-06	TRUE	TRUE	482,730	19.83
Sphingomyelin (d40:1) levels	Parkinson's disease	rs145797759	A	T	A	T	8.29E-01	-1.02E-01	2.23E-03	8.70E-03	TRUE	FALSE	13	104581365	2.07E-01	2.06E-01	TRUE	13	103929015	1.85E-01	0.94	7174	7.39E-06	TRUE	TRUE	482,730	20.12
Sphingomyelin (d40:1) levels	Parkinson's disease	rs182695896	C	A	C	A	3.77E-01	1.53E-01	2.53E-02	9.84E-01	FALSE	FALSE	4	74813227	1.83E-01	3.94E-01	TRUE	4	73947510	5.28E-02	0.96	7174	1.11E-12	TRUE	TRUE	482,730	50.82
Sphingomyelin (d40:1) levels	Parkinson's disease	rs186039163	G	T	G	T	-3.99E-01	-2.60E-02	1.01E-02	9.88E-01	FALSE	FALSE	17	3450893	1.46E-01	6.63E-02	TRUE	17	3547599	8.40E-02	0.97	7174	2.09E-06	TRUE	TRUE	482,730	22.55
Sphingomyelin (d40:1) levels	Parkinson's disease	rs188346987	G	C	G	C	-1.76E-01	-1.93E-02	5.12E-02	3.47E-02	TRUE	FALSE	2	157953943	7.37E-02	1.01E-01	TRUE	2	157097431	3.95E-02	0.91	7174	8.76E-06	TRUE	TRUE	482,730	19.79
Sphingomyelin (d40:1) levels	Parkinson's disease	rs192913735	T	C	T	C	-5.62E-01	8.33E-02	9.06E-03	9.89E-01	FALSE	FALSE	17	4682640	1.51E-01	2.36E-01	TRUE	17	4779345	8.74E-02	0.98	7174	1.36E-10	TRUE	TRUE	482,730	41.34
Sphingomyelin (d40:1) levels	Parkinson's disease	rs2275544	C	T	C	T	-1.23E-01	1.25E-02	1.25E-01	8.56E-01	FALSE	FALSE	9	107651212	2.38E-02	2.22E-01	TRUE	9	104888931	2.52E-02	1.00	7174	1.13E-06	TRUE	TRUE	482,730	23.72
Sphingomyelin (d40:1) levels	Parkinson's disease	rs2552134	T	G	T	G	-9.28E-02	-2.90E-03	2.21E-01	7.40E-01	FALSE	FALSE	8	3933252	2.55E-02	4.08E-02	TRUE	8	4075730	2.01E-02	0.99	7174	3.88E-06	TRUE	TRUE	482,730	21.36
Sphingomyelin (d40:1) levels	Parkinson's disease	rs279967	G	A	G	A	-1.57E-01	-1.34E-02	9.38E-01	6.77E-02	FALSE	FALSE	8	94060273	4.85E-02	1.07E-01	TRUE	8	93048045	3.45E-02	0.98	7174	5.45E-06	TRUE	TRUE	482,730	20.70
Sphingomyelin (d40:1) levels	Parkinson's disease	rs283819	C	A	C	A	-1.06E-01	3.13E-02	8.16E-01	2.54E-01	FALSE	FALSE	2	79227243	2.61E-02	6.39E-01	TRUE	2	79000117	2.16E-02	0.99	7174	1.01E-06	TRUE	TRUE	482,730	23.95
Sphingomyelin (d40:1) levels	Parkinson's disease	rs34359547	A	G	A	G	1.77E-01	3.54E-02	4.50E-02	9.29E-01	FALSE	FALSE	13	97128574	3.76E-02	4.59E-01	TRUE	13	96476320	3.98E-02	0.99	7174	9.05E-06	TRUE	TRUE	482,730	19.73
Sphingomyelin (d40:1) levels	Parkinson's disease	rs4767003	C	T	C	T	8.73E-02	1.19E-02	6.65E-01	4.37E-01	FALSE	FALSE	12	113190918	2.15E-02	2.37E-01	TRUE	12	112753113	1.75E-02	1.00	7174	6.52E-07	TRUE	TRUE	482,730	24.80
Sphingomyelin (d40:1) levels	Parkinson's disease	rs4949190	T	C	T	C	-9.86E-02	-9.80E-03	7.73E-01	2.94E-01	FALSE	FALSE	1	31485357	2.53E-02	1.56E-01	TRUE	1	31012510	1.99E-02	0.99	7174	7.78E-07	TRUE	TRUE	482,730	24.46
Sphingomyelin (d40:1) levels	Parkinson's disease	rs4954960	C	T	C	T	9.06E-02	-1.30E-03	7.38E-01	3.23E-01	FALSE	FALSE	2	139574143	2.40E-02	1.87E-02	TRUE	2	138816573	1.90E-02	0.99	7174	1.90E-06	TRUE	TRUE	482,730	22.74
Sphingomyelin (d40:1) levels	Parkinson's disease	rs4962634	G	A	G	A	7.54E-02	1.48E-02	5.11E-01	3.88E-01	FALSE	FALSE	10	126181491	2.04E-02	3.30E-01	TRUE	10	124492922	1.67E-02	0.99	7174	6.80E-06	TRUE	TRUE	482,730	20.28
Sphingomyelin (d40:1) levels	Parkinson's disease	rs66626762	G	A	G	A	1.41E-01	1.32E-02	7.47E-02	9.47E-01	FALSE	FALSE	5	100974164	4.30E-02	1.19E-01	TRUE	5	101638460	3.18E-02	0.99	7174	9.67E-06	TRUE	TRUE	482,730	19.60
Sphingomyelin (d40:1) levels	Parkinson's disease	rs7478167	A	T	A	T	-8.06E-02	-3.50E-03	6.69E-01	6.66E-01	TRUE	FALSE	10	103476258	1.91E-02	6.80E-02	TRUE	10	101716501	1.78E-02	0.99	7174	6.39E-06	TRUE	TRUE	482,730	20.40
Sphingomyelin (d40:1) levels	Parkinson's disease	rs75679663	A	C	A	C	-7.74E-01	1.35E-01	2.58E-02	9.87E-01	FALSE	FALSE	17	4667972	9.72E-02	7.79E-01	TRUE	17	4764677	5.30E-02	0.98	7174	1.26E-47	TRUE	TRUE	482,730	213.28
Sphingomyelin (d40:1) levels	Parkinson's disease	rs76174849	T	C	T	C	3.67E-01	-2.82E-02	1.40E-02	9.79E-01	FALSE	FALSE	1	8798765	8.49E-02	1.31E-01	TRUE	1	8738706	7.19E-02	0.97	7174	3.41E-07	TRUE	TRUE	482,730	26.05
Sphingomyelin (d40:1) levels	Parkinson's disease	rs76790474	A	G	A	G	-1.37E-01	2.64E-02	2.53E-01	7.71E-01	FALSE	FALSE	17	4401974	3.35E-02	3.66E-01	TRUE	17	4498679	1.94E-02	0.94	7174	1.64E-12	TRUE	TRUE	482,730	50.05
Sphingomyelin (d40:1) levels	Parkinson's disease	rs77030061	T	C	T	C	2.25E-01	5.87E-02	3.76E-02	9.65E-01	FALSE	FALSE	4	169820130	7.33E-02	3.73E-01	TRUE	4	168898979	4.79E-02	0.83	7174	2.68E-06	TRUE	TRUE	482,730	22.07
Sphingomyelin (d40:1) levels	Parkinson's disease	rs77645768	A	G	A	G	3.20E-01	5.69E-02	2.56E-02	9.74E-01	FALSE	FALSE	4	73668828	7.75E-02	3.34E-01	TRUE	4	72803111	5.36E-02	0.97	7174	2.56E-09	TRUE	TRUE	482,730	35.57
Sphingomyelin (d40:1) levels	Parkinson's disease	rs7970512	A	C	A	C	-2.29E-01	7.19E-02	3.50E-02	9.81E-01	FALSE	FALSE	12	67907600	1.01E-01	3.23E-01	TRUE	12	67513820	4.78E-02	0.87	7174	1.65E-06	TRUE	TRUE	482,730	23.00
Sphingomyelin (d40:1) levels	Parkinson's disease	rs80019356	G	C	G	C	2.52E-01	-2.98E-02	2.35E-02	2.38E-02	TRUE	FALSE	1	155881837	5.97E-02	2.09E-01	TRUE	1	155912046	5.63E-02	0.99	7174	7.92E-06	TRUE	TRUE	482,730	19.99
Sphingomyelin (d40:1) levels	Parkinson's disease	rs80101718	A	G	A	G	3.34E-01	-2.60E-03	2.96E-02	9.72E-01	FALSE	FALSE	4	73240747	7.86E-02	1.16E-02	TRUE	4	72375030	5.08E-02	0.93	7174	4.97E-11	TRUE	TRUE	482,730	43.32
Sphingomyelin (d40:1) levels	Parkinson's disease	rs8189230	A	G	A	G	1.56E-01	-1.16E-01	7.02E-02	9.67E-01	FALSE	FALSE	11	48827607	7.89E-02	8.48E-01	TRUE	11	48806055	3.38E-02	0.93	7174	3.88E-06	TRUE	TRUE	482,730	21.36
Sphingomyelin (d40:1) levels	Parkinson's disease	rs9835223	G	C	G	C	-1.04E-01	2.23E-02	8.01E-01	8.29E-01	TRUE	FALSE	3	186586284	3.11E-02	3.25E-01	TRUE	3	186868495	2.11E-02	0.96	7174	9.29E-07	TRUE	TRUE	482,730	24.11
Sphingomyelin (d40:2) levels	Parkinson's disease	rs10490233	A	T	A	T	-1.31E-01	8.10E-03	1.11E-01	1.08E-01	TRUE	FALSE	2	50429128	3.58E-02	8.53E-02	TRUE	2	50201990	2.64E-02	1.00	7174	7.65E-07	TRUE	TRUE	482,730	24.49
Sphingomyelin (d40:2) levels	Parkinson's disease	rs113118892	T	C	T	C	3.62E-01	-7.96E-02	1.23E-02	9.88E-01	FALSE	FALSE	15	59566385	1.69E-01	1.96E-01	TRUE	15	59274186	7.94E-02	0.91	7174	5.09E-06	TRUE	TRUE	482,730	20.83
Sphingomyelin (d40:2) levels	Parkinson's disease	rs114483871	T	C	T	C	2.36E-01	-2.94E-02	3.81E-02	9.77E-01	FALSE	FALSE	4	73990168	8.47E-02	1.38E-01	TRUE	4	73124451	4.49E-02	0.93	7174	1.41E-07	TRUE	TRUE	482,730	27.76
Sphingomyelin (d40:2) levels	Parkinson's disease	rs11591147	T	G	T	G	-2.59E-01	-3.77E-02	3.32E-02	9.82E-01	FALSE	FALSE	1	55505647	6.83E-02	2.36E-01	TRUE	1	55039974	4.66E-02	1.00	7174	2.73E-08	TRUE	TRUE	482,730	30.96
Sphingomyelin (d40:2) levels	Parkinson's disease	rs1237196	A	C	A	C	-8.94E-02	-1.35E-02	7.82E-01	2.95E-01	FALSE	FALSE	1	164860158	2.43E-02	2.38E-01	TRUE	1	164890921	2.02E-02	1.00	7174	9.98E-06	TRUE	TRUE	482,730	19.54
Sphingomyelin (d40:2) levels	Parkinson's disease	rs12595641	A	G	A	G	9.81E-02	-2.62E-02	1.83E-01	8.43E-01	FALSE	FALSE	15	31668706	3.17E-02	3.89E-01	TRUE	15	31376503	2.17E-02	0.98	7174	6.00E-06	TRUE	TRUE	482,730	20.51
Sphingomyelin (d40:2) levels	Parkinson's disease	rs146855398	T	C	T	C	-2.37E-01	9.34E-02	2.78E-02	9.86E-01	FALSE	FALSE	14	57187443	1.09E-01	4.09E-01	TRUE	14	56720725	5.28E-02	0.92	7174	7.06E-06	TRUE	TRUE	482,730	20.21
Sphingomyelin (d40:2) levels	Parkinson's disease	rs148316008	A	G	A	G	-3.52E-01	1.32E-01	1.24E-02	9.83E-01	FALSE	FALSE	2	129057401	8.92E-02	8.60E-01	TRUE	2	128299827	7.73E-02	0.97	7174	5.30E-06	TRUE	TRUE	482,730	20.76
Sphingomyelin (d40:2) levels	Parkinson's disease	rs16851199	G	A	G	A	-1.89E-01	6.27E-02	4.28E-02	9.75E-01	FALSE	FALSE	1	230379953	8.05E-02	3.61E-01	TRUE	1	230244207	4.24E-02	0.94	7174	8.33E-06	TRUE	TRUE	482,730	19.89

Sphingomyelin (d40:2) levels	Parkinson's disease	rs17276933	A	T	A	T	-1.65E-01	-3.59E-02	5.67E-02	7.95E-02	TRUE	FALSE	14	20485905	4.08E-02	4.21E-01	TRUE	14	20017746	3.61E-02	1.00	7174	4.64E-06	TRUE	TRUE	482,730	21.01
Sphingomyelin (d40:2) levels	Parkinson's disease	rs174561	C	T	C	T	-1.21E-01	8.00E-04	3.81E-01	7.08E-01	FALSE	FALSE	11	61582708	1.85E-02	1.52E-02	TRUE	11	61815236	1.72E-02	1.00	7174	2.13E-12	TRUE	TRUE	482,730	49.54
Sphingomyelin (d40:2) levels	Parkinson's disease	rs17604972	A	G	A	G	1.07E-01	7.40E-03	1.44E-01	8.89E-01	FALSE	FALSE	12	45970721	4.77E-02	5.69E-02	TRUE	12	45576938	2.42E-02	0.96	7174	9.37E-06	TRUE	TRUE	482,730	19.66
Sphingomyelin (d40:2) levels	Parkinson's disease	rs1797734	A	G	A	G	7.88E-02	2.28E-02	6.56E-01	3.56E-01	FALSE	FALSE	12	31947194	1.90E-02	6.39E-01	TRUE	12	31794260	1.76E-02	0.99	7174	7.57E-06	TRUE	TRUE	482,730	20.07
Sphingomyelin (d40:2) levels	Parkinson's disease	rs182695896	C	A	C	A	3.53E-01	1.53E-01	2.53E-02	9.84E-01	FALSE	FALSE	4	74813227	1.83E-01	3.94E-01	TRUE	4	73947510	5.29E-02	0.96	7174	2.77E-11	TRUE	TRUE	482,730	44.48
Sphingomyelin (d40:2) levels	Parkinson's disease	rs187144380	G	A	G	A	3.80E-01	1.78E-01	1.11E-02	9.93E-01	FALSE	FALSE	6	152241906	1.71E-01	5.24E-01	TRUE	6	151920771	8.16E-02	0.94	7174	3.36E-06	TRUE	TRUE	482,730	21.63
Sphingomyelin (d40:2) levels	Parkinson's disease	rs2760512	G	A	G	A	8.35E-02	2.78E-02	3.33E-01	6.67E-01	FALSE	FALSE	1	192561545	2.37E-02	6.21E-01	TRUE	1	192592415	1.79E-02	1.00	7174	3.31E-06	TRUE	TRUE	482,730	21.66
Sphingomyelin (d40:2) levels	Parkinson's disease	rs3026120	A	G	A	G	-1.88E-01	-1.28E-02	7.46E-02	9.54E-01	FALSE	FALSE	17	5287415	4.14E-02	1.20E-01	TRUE	17	5384095	3.12E-02	1.00	7174	1.61E-09	TRUE	TRUE	482,730	36.49
Sphingomyelin (d40:2) levels	Parkinson's disease	rs56298236	T	C	T	C	9.57E-02	-1.70E-03	2.68E-01	8.22E-01	FALSE	FALSE	2	211606024	2.47E-02	2.51E-02	TRUE	2	210741300	1.90E-02	1.00	7174	4.61E-07	TRUE	TRUE	482,730	25.46
Sphingomyelin (d40:2) levels	Parkinson's disease	rs646488	C	T	C	T	8.17E-02	1.45E-02	6.79E-01	3.29E-01	FALSE	FALSE	11	125790722	1.85E-02	3.62E-01	TRUE	11	125920827	1.79E-02	0.99	7174	5.12E-06	TRUE	TRUE	482,730	20.82
Sphingomyelin (d40:2) levels	Parkinson's disease	rs7157785	T	G	T	G	3.37E-01	-3.20E-02	1.25E-01	8.32E-01	FALSE	FALSE	14	64235556	2.25E-02	8.11E-01	TRUE	14	63768838	2.49E-02	1.00	7174	2.10E-41	TRUE	TRUE	482,730	184.03
Sphingomyelin (d40:2) levels	Parkinson's disease	rs73073606	C	A	C	A	-9.48E-02	2.07E-02	2.41E-01	6.67E-01	FALSE	FALSE	3	193894366	2.49E-02	3.91E-01	TRUE	3	194176577	2.02E-02	0.94	7174	2.59E-06	TRUE	TRUE	482,730	22.14
Sphingomyelin (d40:2) levels	Parkinson's disease	rs73641617	T	A	T	A	2.94E-01	-8.64E-02	1.76E-02	3.00E-02	TRUE	FALSE	9	5454500	6.66E-02	7.11E-01	TRUE	9	5454500	6.37E-02	0.98	7174	3.84E-06	TRUE	TRUE	482,730	21.37
Sphingomyelin (d40:2) levels	Parkinson's disease	rs7478167	A	T	A	T	-8.16E-02	-3.50E-03	6.69E-01	6.66E-01	TRUE	FALSE	10	103476258	1.91E-02	6.80E-02	TRUE	10	101716501	1.79E-02	0.99	7174	5.02E-06	TRUE	TRUE	482,730	20.86
Sphingomyelin (d40:2) levels	Parkinson's disease	rs7536561	G	A	G	A	7.62E-02	-9.00E-04	5.78E-01	4.77E-01	FALSE	FALSE	1	180243524	1.74E-02	1.88E-02	TRUE	1	180274389	1.69E-02	1.00	7174	6.94E-06	TRUE	TRUE	482,730	20.24
Sphingomyelin (d40:2) levels	Parkinson's disease	rs75679663	A	C	A	C	-2.77E-01	1.35E-01	2.58E-02	9.87E-01	FALSE	FALSE	17	4667972	9.72E-02	7.79E-01	TRUE	17	4764677	5.37E-02	0.98	7174	2.59E-07	TRUE	TRUE	482,730	26.58
Sphingomyelin (d40:2) levels	Parkinson's disease	rs77645768	A	G	A	G	3.04E-01	5.69E-02	2.56E-02	9.74E-01	FALSE	FALSE	4	73668828	7.75E-02	3.34E-01	TRUE	4	72803111	5.37E-02	0.97	7174	1.55E-08	TRUE	TRUE	482,730	32.06
Sphingomyelin (d40:2) levels	Parkinson's disease	rs79402526	C	T	C	T	2.14E-01	3.06E-02	3.88E-02	9.63E-01	FALSE	FALSE	4	169786002	7.10E-02	1.76E-01	TRUE	4	168864851	4.65E-02	0.85	7174	4.43E-06	TRUE	TRUE	482,730	21.10
Sphingomyelin (d40:2) levels	Parkinson's disease	rs9835223	G	C	G	C	-1.05E-01	2.23E-02	8.01E-01	8.29E-01	TRUE	FALSE	3	186586284	3.11E-02	3.25E-01	TRUE	3	186868495	2.11E-02	0.96	7174	6.33E-07	TRUE	TRUE	482,730	24.85
Sphingomyelin (d40:2) levels	Parkinson's disease	rs9839427	G	A	G	A	-8.12E-02	-2.33E-02	3.25E-01	6.36E-01	FALSE	FALSE	3	50301584	1.90E-02	6.59E-01	TRUE	3	50264152	1.77E-02	0.99	7174	4.75E-06	TRUE	TRUE	482,730	20.97
Sphingomyelin (d42:2) levels	Parkinson's disease	rs10252648	C	G	C	G	2.57E-01	-1.13E-02	9.78E-01	9.63E-01	TRUE	FALSE	7	151638803	5.89E-02	7.14E-02	TRUE	7	151941718	5.81E-02	0.99	7174	9.95E-06	TRUE	TRUE	482,730	19.55
Sphingomyelin (d42:2) levels	Parkinson's disease	rs112585713	A	G	A	G	2.88E-01	1.07E-01	3.16E-02	9.80E-01	FALSE	FALSE	4	73742418	9.00E-02	6.32E-01	TRUE	4	72876701	4.79E-02	0.98	7174	1.86E-09	TRUE	TRUE	482,730	36.20
Sphingomyelin (d42:2) levels	Parkinson's disease	rs114483871	T	C	T	C	2.79E-01	-2.94E-02	3.81E-02	9.77E-01	FALSE	FALSE	4	73990168	8.47E-02	1.38E-01	TRUE	4	73124451	4.48E-02	0.93	7174	4.78E-10	TRUE	TRUE	482,730	38.87
Sphingomyelin (d42:2) levels	Parkinson's disease	rs115350073	T	C	T	C	2.94E-01	-9.46E-02	1.81E-02	9.89E-01	FALSE	FALSE	5	90165912	1.38E-01	3.07E-01	TRUE	5	90870095	6.33E-02	0.97	7174	3.56E-06	TRUE	TRUE	482,730	21.52
Sphingomyelin (d42:2) levels	Parkinson's disease	rs11591147	T	G	T	G	-3.62E-01	-3.77E-02	3.32E-02	9.82E-01	FALSE	FALSE	1	55505647	6.83E-02	2.36E-01	TRUE	1	55039974	4.65E-02	1.00	7174	8.29E-15	TRUE	TRUE	482,730	60.52
Sphingomyelin (d42:2) levels	Parkinson's disease	rs11610836	T	C	T	C	7.93E-02	2.08E-02	4.22E-01	5.85E-01	FALSE	FALSE	12	113202967	2.15E-02	4.75E-01	TRUE	12	112765162	1.66E-02	1.00	7174	1.75E-06	TRUE	TRUE	482,730	22.89
Sphingomyelin (d42:2) levels	Parkinson's disease	rs116893213	C	G	C	G	2.27E-01	1.67E-02	3.70E-02	2.21E-02	TRUE	FALSE	11	101610303	8.38E-02	7.46E-02	TRUE	11	101739572	4.48E-02	0.98	7174	4.17E-07	TRUE	TRUE	482,730	25.66
Sphingomyelin (d42:2) levels	Parkinson's disease	rs116919723	A	G	A	G	-3.34E-01	7.40E-03	1.33E-02	9.81E-01	FALSE	FALSE	17	3434834	1.43E-01	1.82E-02	TRUE	17	3531540	7.46E-02	0.94	7174	7.56E-06	TRUE	TRUE	482,730	20.07
Sphingomyelin (d42:2) levels	Parkinson's disease	rs12530474	G	A	G	A	-1.11E-01	-1.60E-02	1.33E-01	9.03E-01	FALSE	FALSE	6	14104590	2.99E-02	2.27E-01	TRUE	6	14104359	2.42E-02	1.00	7174	4.56E-06	TRUE	TRUE	482,730	21.05
Sphingomyelin (d42:2) levels	Parkinson's disease	rs1367117	A	G	A	G	9.95E-02	6.00E-03	2.85E-01	6.89E-01	FALSE	FALSE	2	21263900	1.81E-02	1.31E-01	TRUE	2	21041028	1.84E-02	1.00	7174	6.88E-08	TRUE	TRUE	482,730	29.16
Sphingomyelin (d42:2) levels	Parkinson's disease	rs139474488	C	A	C	A	3.44E-01	-1.11E-01	1.39E-02	9.87E-01	FALSE	FALSE	10	130541154	1.11E-01	4.99E-01	TRUE	10	128742890	7.59E-02	0.87	7174	6.01E-06	TRUE	TRUE	482,730	20.51
Sphingomyelin (d42:2) levels	Parkinson's disease	rs146331166	C	T	C	T	-2.19E-01	-9.60E-03	5.96E-02	9.76E-01	FALSE	FALSE	17	5367550	6.93E-02	5.05E-02	TRUE	17	5464230	3.45E-02	0.99	7174	2.56E-10	TRUE	TRUE	482,730	40.10
Sphingomyelin (d42:2) levels	Parkinson's disease	rs17018185	C	A	C	A	-8.15E-02	-2.10E-02	2.79E-01	7.90E-01	FALSE	FALSE	1	107579295	2.12E-02	4.92E-01	TRUE	1	107036673	1.83E-02	1.00	7174	8.28E-06	TRUE	TRUE	482,730	19.90
Sphingomyelin (d42:2) levels	Parkinson's disease	rs17110517	T	C	T	C	-1.67E-01	-3.06E-02	6.10E-02	9.48E-01	FALSE	FALSE	1	54834840	5.36E-02	2.46E-01	TRUE	1	54369167	3.53E-02	0.98	7174	2.26E-06	TRUE	TRUE	482,730	22.40
Sphingomyelin (d42:2) levels	Parkinson's disease	rs17145750	T	C	T	C	1.32E-01	-4.00E-04	1.41E-01	8.50E-01	FALSE	FALSE	7	73026378	2.45E-02	6.30E-03	TRUE	7	73612048	2.39E-02	1.00	7174	3.07E-08	TRUE	TRUE	482,730	30.73
Sphingomyelin (d42:2) levels	Parkinson's disease	rs17261772	C	T	C	T	9.00E-02	1.26E-02	3.26E-01	6.46E-01	FALSE	FALSE	2	135911422	2.08E-02	2.65E-01	TRUE	2	135153852	1.77E-02	1.00	7174	3.94E-07	TRUE	TRUE	482,730	25.77
Sphingomyelin (d42:2) levels	Parkinson's disease	rs17276933	A	T	A	T	-1.71E-01	-3.59E-02	5.67E-02	7.95E-02	TRUE	FALSE	14	20485905	4.08E-02	4.21E-01	TRUE	14	20017746	3.61E-02	1.00	7174	2.11E-06	TRUE	TRUE	482,730	22.53
Sphingomyelin (d42:2) levels	Parkinson's disease	rs182695896	C	A	C	A	3.82E-01	1.53E-01	2.53E-02	9.84E-01	FALSE	FALSE	4	74813227	1.83E-01	3.94E-01	TRUE	4	73947510	5.28E-02	0.96	7174	5.36E-13	TRUE	TRUE	482,730	52.26
Sphingomyelin (d42:2) levels	Parkinson's disease	rs2034007	G	A	G	A	-8.17E-02	8.10E-03	3.00E-01	7.41E-01	FALSE	FALSE	3	19371858	2.25E-02	1.44E-01	TRUE	3	19330366	1.82E-02	0.99	7174	7.52E-06	TRUE	TRUE	482,730	20.09
Sphingomyelin (d42:2) levels	Parkinson's disease	rs2177333	A	G	A	G	7.48E-02	1.87E-02	5.34E-01	4.35E-01	FALSE	FALSE	3	153293917	2.23E-02	3.95E-01	TRUE	3	153576128	1.67E-02	1.00	7174	7.76E-06	TRUE	TRUE	482,730	20.02
Sphingomyelin (d42:2) levels	Parkinson's disease	rs3912622	C	A	C	A	-1.09E-01	4.00E-03	2.00E-01	7.39E-01	FALSE	FALSE	11	103121473	2.20E-02	6.73E-02	TRUE	11	103250744	2.09E-02	1.00	7174	2.00E-07	TRUE	TRUE	482,730	27.09
Sphingomyelin (d42:2) levels	Parkinson's disease	rs4100654	C	T	C	T	-1.56E-01	7.60E-03	6.62E-02	9.00E-01	FALSE	FALSE	9	107669241	2.92E-02	1.00E-01	TRUE	9	104906960	3.37E-02	0.97	7174	3.76E-06	TRUE	TRUE	482,730	21.42
Sphingomyelin (d42:2) levels	Parkinson's disease	rs515081	G	A	G	A	-9.17E-02	3.18E-02	6.88E-01	3.39E-01	FALSE	FALSE	15	58716257	2.95E-02	5.51E-01	TRUE	15	58424058	1.89E-02	0.89	7174	1.31E-06	TRUE	TRUE	482,730	23.45
Sphingomyelin (d42:2) levels	Parkinson's disease	rs59916403	T	G	T	G	7.82E-02	1.15E-02	4.13E-01	6.56E-01	FALSE	FALSE	2	242370751	1.95E-02	2.54E-01	TRUE	2	241431336	1.69E-02	1.00	7174	3.85E-06	TRUE	TRUE	482,730	21.37
Sphingomyelin (d42:2) levels	Parkinson's disease	rs62274367	C	T	C	T	-6.80E-01	1.28E-01	3.81E-03	9.81E-01	FALSE	FALSE	3	103234448	9.15E-02	7.89E-01	TRUE	3	103515604	1.46E-01	0.88	7174	3.03E-06	TRUE	TRUE	482,730	21.83
Sphingomyelin (d42:2) levels	Parkinson's disease	rs6680361	T	C	T	C	9.66E-02	2.46E-02	7.95E-01	1.64E-01	FALSE	FALSE	1	180246261	3.11E-02	3.67E-01	TRUE	1	180277126	2.11E-02	0.99	7174	4.53E-06	TRUE	TRUE	482,730	21.06
Sphingomyelin (d42:2) levels	Parkinson's disease	rs67718253	C	A	C	A	-1.02E-01	-3.17E-02	1.76E-01	8.10E-01	FALSE	FALSE	5	107977118	2.85E-02	5.74E-01	TRUE	5	108641417	2.19E-02	0.99	7174	3.65E-06	TRUE	TRUE	482,730	21.47
Sphingomyelin (d42:2) levels	Parkinson's disease	rs6843498	G	T	G	T	9.46E-02	-2.90E-03	3.12E-01	6.58E-01	FALSE	FALSE	4	16521723	2.53E-02	4.13E-02	TRUE	4	16520100	1.88E-02	0.92	7174	5.00E-07	TRUE	TRUE	482,730	25.31
Sphingomyelin (d42:2) levels	Parkinson's disease	rs7104343	T	C	T	C	9.28E-02	1.83E-02	2.05E-01	8.98E-01	FALSE	FALSE	11	116529024	3.60E-02	2.14E-01	TRUE	11	116658307	2.06E-02	1.00	7174	6.92E-06	TRUE	TRUE	482,730	20.24
Sphingomyelin (d42:2) levels	Parkinson's disease	rs75444922	G	A	G	A	2.36E-01	-1.76E-02	2.65E-02	9.53E-01	FALSE	FALSE	8	47869478	5.91E-02	1.16E-01	TRUE	8	46957856	5.33E-02	0.94	7174	9.45E-06	TRUE	TRUE	482,730	19.65
Sphingomyelin (d42:2) levels	Parkinson's disease	rs75679663	A	C	A	C	-5.38E-01	1.35E-01	2.58E-02	9.87E-01	FALSE	FALSE	17	4667972	9.72E-02	7.79E-01	TRUE	17	4764677	5.34E-02	0.98	7174	1.09E-23	TRUE	TRUE	482,730	101.37
Triacylglycerol (46:1) levels	Parkinson's disease	rs10521320	C	A	C	A	1.07E-01	-1.64E-02	1.95E-01	8.01E-01	FALSE	FALSE	16	55576161	2.46E-02	2.97E-01	TRUE	16	55542249	2.34E-02	0.95	6070	5.44E-06	TRUE	TRUE	482,730	20.71
Triacylglycerol (46:1) levels	Parkinson's disease	rs111568723	G	A	G	A	2.79E-01	-1.20E-01	2.59E-02	9.47E-01	FALSE	FALSE	9	135345889	5.14E-02	9.73E-03	TRUE	9	132470502	5.93E-02	0.95	6070	2.63E-06	TRUE	TRUE	482,730	22.11
Triacylglycerol (46:1) levels	Parkinson's disease	rs117591451	A	G	A	G	-4.02E-01	-2.41E-02	1.31E-02	9.93E-01	FALSE	FALSE	8	40225327	2.27E-01	3.84E-02	TRUE	8	40367808	8.49E-02	0.92	6070	2.27E-06	TRUE	TRUE	482,730	22.39
Triacylglycerol (46:1) levels	Parkinson's disease	rs12225049	T	C	T	C	-1.17E-01	2.19E-02	1.69E-01	8.78E-01	FALSE	FALSE	11	110250339	3.33E-02	2.92E-01	TRUE	11	110379615	2.42E-02	0.99	6070	1.44E-06	TRUE	TRUE	482,730	23.27
Triacylglycerol (46:1) levels	Parkinson's disease	rs12301917	T	C	T	C	1.13E-01	6.30E-02	1.89E-01	8.07E-01	FALSE	FALSE	12	82066608	2.85E-02	1.35E-02	TRUE	12	81672829	2.33E-02	0.99	6070	1.16E-06	TRUE	TRUE	482,730	23.68
Triacylglycerol (46:1) levels	Parkinson's disease	rs1260326	C	T	C	T	-1.01E-01	-6.78E-02	6.51E-01	4.20E-01	FALSE	FALSE	2	27730940	1.72E-02	4.04E-05	TRUE	2	27508073	1.89E-02	1.00	6070	8.99E-08	TRUE	TRUE	482,730	28.65
Triacylglycerol (46:1) levels	Parkinson's disease	rs16853823	G	A	G	A	1.00E-01	4.51E-02	2.23E-01	7.95E-01	FALSE	FALSE	2	216203296	2.77E-02	9.87E-01	TRUE	2	215338573	2.19E-02	1.00	6070	4.54E-06	TRUE	TRUE	482,730	21.06
Triacylglycerol (46:1) levels	Parkinson's disease	rs16923137	T	A	T	A	1.86E-01	-2.36E-01	5.21E-02	2.99E-02	TRUE	FALSE	8	59070917	1.70E-01	7.78E-01	TRUE	8	58158358	4.05E-02	0.98	6070	4.37E-06	TRUE	TRUE	482,730	21.13
Triacylglycerol (46:1) levels	Parkinson's disease	rs4318703	G	A	G	A	8.88E-02	3.27E-02	3.00E-01	7.43E-01	FALSE	FALSE	4	136910271	2.54E-02	7.04E-01	TRUE	4	135989116	2.00E-02	1.00	6070	8.71E-06	TRUE	TRUE	482,730	19.81
Triacylglycerol (46:1) levels	Parkinson's disease	rs478562	C	T	C	T	7.88E-01	4.95E-02	9.97E-01	2.34E-02	FALSE	FALSE	2	137164045	8.56E-02	2.49E-01	TRUE	2	136406475	1.78E-01	0.86	6070	9.57E-06	TRUE	TRUE	482,730	19.63
Triacylglycerol (46:1) levels	Parkinson's disease	rs73259585	G	A	G	A	-2.16E-01	2.69E-02	3.72E-02	9.66E-01	FALSE	FALSE	14	30287597	6.23E-02	1.76E-01	TRUE	14	29818391	4.85E-02	0.98	6070	8.36E-06	TRUE	TRUE	482,730	19.89
Triacylglycerol (46:1) levels	Parkinson's disease	rs78056622	G	C	G	C	4.06E-01	1.20E-01	1.10E-02	1.60E-02	TRUE	FALSE	1	184385697	1.07E-01	5.83E-01	TRUE	1	184416563	9.03E-02	0.91	6070	6.91E-06	TRUE	TRUE	482,730	20.25
Triacylglycerol (46:1) levels	Parkinson's disease	rs9796793	A	G	A	G	-1.31E-01	-6.30E-02	1.25E-01	9.58E-01	FALSE	FALSE	16	30495652	5.46E-02	6.04E-01	TRUE	16	30484331	2.83E-02	0.95	6070	3.81E-06	TRUE	TRUE	482,730	21.40
Triacylglycerol (46:1) levels	Parkinson's disease	rs9837572	T	G	T	G	-9.73E-02	2.68E-02	2.36E-01	8.28E-01	FALSE	FALSE	3	72309600	3.00E-02	4.29E-01	TRUE	3	72260449	2.15E-02	0.99	6070	6.10E-06	TRUE	TRUE	482,730	20.49
Triacylglycerol (46:2) levels	Parkinson's disease	rs11613792	G	A	G	A	-1.18E-01	5.26E-02	1.63E-01	8.62E-01	FALSE	FALSE	12	8760610	4.22E-02	6.73E-01	TRUE	12	8608014	2.65E-02	0.93	5448	8.83E-06	TRUE	TRUE	482,730	19.78
Triacylglycerol (46:2) levels	Parkinson's disease	rs116923150	A	G	A	G	8.30E-01	1.63E-01	3.23E-03	9.84E-01	FALSE	FALSE	9	114876489	1.55E-01	5.32E-01	TRUE	9	112114209	1.83E-01	0.90	5448	5.75E-06	TRUE	TRUE	482,730	20.61
Triacylglycerol (46:2) levels	Parkinson's disease	rs117529982	T	A	T	A	-4.77E-01	-3.12E-02	8.74E-03	2.33E-02	TRUE	FALSE	9	102418171	7.57E-02	1.67E-01	TRUE	9	99655889	1.06E-01	0.94	5448	6.46E-06	TRUE	TRUE	482,730	20.38
Triacylglycerol (46:2) levels	Parkinson's disease	rs117591451	A	G	A	G	-4.08E-01	-2.41E-02	1.31E-02	9.93E-01	FALSE	FALSE	8	40225327	2.27E-01	3.84E-02	TRUE	8	40367808	9.07E-02	0.92	5448	7.07E-06	TRUE	TRUE	482,730	20.21
Triacylglycerol (46:2) levels	Parkinson's disease	rs12225049	T	C	T	C	-1.18E-01	2.19E-02	1.69E-01	8.78E-01	FALSE	FALSE	11	110250339	3.33E-02	2.92E-01	TRUE	11	110379615	2.57E-02	0.99	5448	4.24E-06	TRUE	TRUE	482,730	21.19
Triacylglycerol (46:2) levels	Parkinson's disease	rs12301917	T	C	T	C	1.10E-01	6.30E-02	1.89E-01	8.07E-01	FALSE	FALSE	12	82066608	2.85E-02	1.35E-02	TRUE	12	81672829	2.46E-02	0.99	5448	7.62E-06	TRUE	TRUE	482,730	20.07
Triacylglycerol (46:2) levels	Parkinson's disease	rs1260326	C	T	C	T	-1.14E-01	-6.78E-02	6.51E-01	4.20E-01	FALSE	FALSE	2	27730940	1.72E-02	4.04E-05	TRUE	2	27508073	2.00E-02	1.00	5448	1.35E-08	TRUE	TRUE	482,730	32.35
Triacylglycerol (46:2) levels	Parkinson's disease	rs140796055	C	T	C	T	-2.28E-01	2.28E-02	3.89E-02	9.80E-01	FALSE	FALSE	9	2283431	8.66E-02	1.01E-01	TRUE	9	2283431	5.06E-02	0.98	5448	6.76E-06	TRUE	TRUE	482,730	20.30
Triacylglycerol (46:2) levels	Parkinson's disease	rs141935215	A	G	A	G	2.98E-01	-9.30E-03	2.35E-02	9.87E-01	FALSE	FALSE	6	33953514	1.11E-01	3.01E-02	TRUE	6	33985737	6.60E-02	0.92	5448	6.61E-06	TRUE	TRUE	482,730	20.34
Triacylglycerol (46:2) levels	Parkinson's disease	rs17189819	T	C	T	C	-1.42E-01	6.74E-02	1.03E-01	8.10E-01	FALSE	FALSE	6	30940125	2.84E-02	8.82E-03	TRUE	6	30972348	3.17E-02	1.00	5448	7.30E-06	TRUE	TRUE	482,730	20.15
Triacylglycerol (46:2) levels	Parkinson's disease	rs191616475	G	A	G	A	2.25E-01	2.15E-01	4.81E-02	9.51E-01	FALSE	FALSE	8	59242606	2.39E-01	4.33E-01	TRUE	8	58330047	4.41E-02	1.00	5448	3.64E-07	TRUE	TRUE	482,730	25.94
Triacylglycerol (46:2) levels	Parkinson's disease	rs2084594	A	G	A	G	9.13E-02	2.00E-04	4.61E-01	5.99E-01	FALSE	FALSE	11	26805374	2.30E-02	3.09E-03	TRUE	11	26783827	1.91E-02	1.00	5448	1.79E-06	TRUE	TRUE	482,730	22.85
Triacylglycerol (46:2) levels	Parkinson's disease	rs6474079	T	C	T	C	-1.15E-01	2.78E-02	8.17E-01	1.69E-01	FALSE	FALSE	8	57623583	3.08E-02	4.36E-01	TRUE	8	56711024	2.46E-02	0.98	5448	2.88E-06	TRUE	TRUE	482,730	21.94
Triacylglycerol (46:2) levels	Parkinson's disease	rs6502975	T	C	T	C	1.48E-01	7.11E-02	1.03E-01	9.65E-01	FALSE	FALSE	17	6667350	6.81E-02	5.28E-01	TRUE	17	6764031	3.25E-02	0.98	5448	5.20E-06	TRUE	TRUE	482,730	20.80
Triacylglycerol (46:2) levels	Parkinson's disease	rs7323268	T	C	T	C	1.05E-01	1.23E-02	2.20E-01	7.97E-01	FALSE	FALSE	13	33909738	2.80E-02	1.80E-01	TRUE	13	33335601	2.31E-02	1.00	5448	5.24E-06	TRUE	TRUE	482,730	20.79
Triacylglycerol (46:2) levels	Parkinson's disease	rs778957	C	G	C	G	-1.43E-01	9.80E-03	1.20E-01	1.03E-01	TRUE	FALSE	4	63252005	3.67E-02	1.03E-01	TRUE	4	62386287	2.96E-02	0.99	5448	1.30E-06	TRUE	TRUE	482,730	23.48
Triacylglycerol (46:2) levels	Parkinson's disease	rs78056622	G	C	G	C	4.72E-01	1.20E-01	1.10E-02	1.60E-02	TRUE	FALSE	1	184385697	1.07E-01	5.83E-01	TRUE	1	184416563	9.60E-02	0.91	5448	8.72E-07	TRUE	TRUE	482,730	24.24
Triacylglycerol (46:2) levels	Parkinson's disease	rs9796793	A	G	A	G	-1.50E-01	-6.30E-02	1.25E-01	9.58E-01	FALSE	FALSE	16	30495652	5.46E-02	6.04E-01	TRUE	16	30484331	2.98E-02	0.95	5448	4.57E-07	TRUE	TRUE	482,730	25.50
Triacylglycerol (48:0) levels	Parkinson's disease	rs117264209	G	C	G	C	-3.31E-01	4.21E-02	1.90E-02	3.48E-02	TRUE	FALSE	9	138884524	6.36E-02	2.94E-01	TRUE	9	135992678	7.24E-02	0.96	5463	4.84E-06	TRUE	TRUE	482,730	20.94
Triacylglycerol (48:0) levels	Parkinson's disease	rs117945317	A	T	A	T	-4.75E-01	-1.61E-01	9.26E-03	1.31E-02	TRUE	FALSE	8	11395772	1.26E-01	6.94E-01	TRUE	8	11538263	1.07E-01	0.86	5463	9.91E-06	TRUE	TRUE	482,730	19.56
Triacylglycerol (48:0) levels	Parkinson's disease	rs140828820	G	A	G	A	4.24E-01	1.72E-01	1.06E-02	9.91E-01	FALSE	FALSE	4	34769093	2.99E-01	2.48E-01	TRUE	4	34767471	9.49E-02	0.93	5463	7.93E-06	TRUE	TRUE	482,730	19.99
Triacylglycerol (48:0) levels	Parkinson's disease	rs222507	T	G	T	G	-3.23E-01	-1.39E-01	9.79E-01	3.37E-02	FALSE	FALSE	12	102118873	8.40E-02	4.95E-02	TRUE	12	101725095	6.97E-02	0.93	5463	3.68E-06	TRUE	TRUE	482,730	21.46
Triacylglycerol (48:0) levels	Parkinson's disease	rs2242104	G	A	G	A	-1.03E-01	-6.60E-03	7.52E-01	3.76E-01	FALSE	FALSE	9	2640492	2.32E-02	1.09E-01	TRUE	9	2640492	2.22E-02	0.99	5463	4.16E-06	TRUE	TRUE	482,730	21.23
Triacylglycerol (48:0) levels	Parkinson's disease	rs4955801	C	T	C	T	-1.07E-01	-3.90E-02	7.85E-01	2.48E-01	FALSE	FALSE	3	178810499	2.61E-02	8.70E-01	TRUE	3	179092711	2.32E-02	1.00	5463	4.09E-06	TRUE	TRUE	482,730	21.26
Triacylglycerol (48:0) levels	Parkinson's disease	rs60243822	C	A	C	A	1.14E-01	6.69E-02	1.72E-01	8.30E-01	FALSE	FALSE	12	82030834	3.02E-02	1.34E-02	TRUE	12	81637055	2.55E-02	0.99	5463	7.93E-06	TRUE	TRUE	482,730	19.99
Triacylglycerol (48:0) levels	Parkinson's disease	rs62348866	A	G	A	G	-1.41E-01	2.94E-02	1.18E-01	8.66E-01	FALSE	FALSE	4	187374398	3.29E-02	4.30E-01	TRUE	4	186453244	2.97E-02	0.99	5463	2.01E-06	TRUE	TRUE	482,730	22.63
Triacylglycerol (48:0) levels	Parkinson's disease	rs73302224	C	T	C	T	5.18E-01	1.02E-01	7.44E-03	9.91E-01	FALSE	FALSE	10	49801412	1.39E-01	3.34E-01	TRUE	10	48593367	1.16E-01	0.98	5463	7.88E-06	TRUE	TRUE	482,730	20.00
Triacylglycerol (48:0) levels	Parkinson's disease	rs769432	T	C	T	C	9.10E-02	5.85E-02	5.09E-01	4.13E-01	FALSE	FALSE	17	3324255	1.75E-02	4.15E-04	TRUE	17	3420961	1.91E-02	1.00	5463	2.04E-06	TRUE	TRUE	482,730	22.60
Triacylglycerol (48:0) levels	Parkinson's disease	rs7721676	C	T	C	T	-1.04E-01	-1.91E-02	2.18E-01	7.53E-01	FALSE	FALSE	5	149083207	2.55E-02	3.42E-01	TRUE	5	149703644	2.33E-02	1.00	5463	8.45E-06	TRUE	TRUE	482,730	19.87
Triacylglycerol (48:0) levels	Parkinson's disease	rs79159286	G	T	G	T	1.63E-01	4.20E-02	8.00E-02	8.63E-01	FALSE	FALSE	7	31717311	3.38E-02	6.69E-01	TRUE	7	31677697	3.57E-02	0.97	5463	5.20E-06	TRUE	TRUE	482,730	20.80
Triacylglycerol (48:0) levels	Parkinson's disease	rs969816	G	A	G	A	1.33E-01	-4.59E-02	1.20E-01	9.29E-01	FALSE	FALSE	2	211643761	4.45E-02	5.19E-01	TRUE	2	210779037	3.00E-02	1.00	5463	9.09E-06	TRUE	TRUE	482,730	19.73
Triacylglycerol (48:1) levels	Parkinson's disease	rs10861498	A	G	A	G	-9.52E-02	-1.05E-02	2.42E-01	8.15E-01	FALSE	FALSE	12	106151792	2.88E-02	1.46E-01	TRUE	12	105758014	1.94E-02	0.99	7019	9.10E-07	TRUE	TRUE	482,730	24.15
Triacylglycerol (48:1) levels	Parkinson's disease	rs117387197	C	G	C	G	1.41E-01	-1.93E-02	8.43E-02	5.17E-02	TRUE	FALSE	12	132107451	5.62E-02	1.36E-01	TRUE	12	131622906	3.17E-02	0.93	7019	9.39E-06	TRUE	TRUE	482,730	19.66
Triacylglycerol (48:1) levels	Parkinson's disease	rs12565526	G	A	G	A	7.95E-02	1.85E-02	6.24E-01	3.92E-01	FALSE	FALSE	1	77828321	2.28E-02	3.81E-01	TRUE	1	77362636	1.76E-02	1.00	7019	6.17E-06	TRUE	TRUE	482,730	20.46
Triacylglycerol (48:1) levels	Parkinson's disease	rs1260326	C	T	C	T	-1.19E-01	-6.78E-02	6.51E-01	4.20E-01	FALSE	FALSE	2	27730940	1.72E-02	4.04E-05	TRUE	2	27508073	1.76E-02	1.00	7019	1.89E-11	TRUE	TRUE	482,730	45.23
Triacylglycerol (48:1) levels	Parkinson's disease	rs147463852	T	C	T	C	-4.40E-01	-1.20E-01	7.55E-03	9.86E-01	FALSE	FALSE	3	171205885	9.74E-02	6.62E-01	TRUE	3	171488096	9.69E-02	1.00	7019	5.62E-06	TRUE	TRUE	482,730	20.64
Triacylglycerol (48:1) levels	Parkinson's disease	rs1515104	A	T	A	T	7.84E-02	3.40E-03	6.31E-01	6.47E-01	TRUE	FALSE	2	227092405	1.79E-02	7.04E-02	TRUE	2	226227689	1.76E-02	1.00	7019	8.22E-06	TRUE	TRUE	482,730	19.91
Triacylglycerol (48:1) levels	Parkinson's disease	rs16923137	T	A	T	A	1.72E-01	-2.36E-01	5.21E-02	2.99E-02	TRUE	FALSE	8	59070917	1.70E-01	7.78E-01	TRUE	8	58158358	3.78E-02	0.98	7019	5.08E-06	TRUE	TRUE	482,730	20.84
Triacylglycerol (48:1) levels	Parkinson's disease	rs16970164	C	A	C	A	-2.02E-01	1.01E-01	3.94E-02	9.70E-01	FALSE	FALSE	15	40292090	5.79E-02	4.13E-02	TRUE	15	39999889	4.26E-02	0.99	7019	2.04E-06	TRUE	TRUE	482,730	22.59
Triacylglycerol (48:1) levels	Parkinson's disease	rs17192812	A	G	A	G	1.66E-01	-4.00E-03	5.85E-02	9.61E-01	FALSE	FALSE	2	164412011	5.98E-02	2.41E-02	TRUE	2	163555501	3.66E-02	0.95	7019	6.03E-06	TRUE	TRUE	482,730	20.51
Triacylglycerol (48:1) levels	Parkinson's disease	rs3127050	A	G	A	G	8.83E-02	-1.83E-02	7.18E-01	2.83E-01	FALSE	FALSE	13	61406737	2.47E-02	3.38E-01	TRUE	13	60832603	1.86E-02	1.00	7019	2.18E-06	TRUE	TRUE	482,730	22.47
Triacylglycerol (48:1) levels	Parkinson's disease	rs56082136	T	C	T	C	1.73E-01	2.15E-02	5.26E-02	9.15E-01	FALSE	FALSE	12	52637176	3.69E-02	2.51E-01	TRUE	12	52243392	3.88E-02	0.93	7019	8.47E-06	TRUE	TRUE	482,730	19.86
Triacylglycerol (48:1) levels	Parkinson's disease	rs6664147	T	C	T	C	-9.68E-02	2.77E-02	1.90E-01	7.84E-01	FALSE	FALSE	1	227617519	2.38E-02	6.11E-01	TRUE	1	227429818	2.16E-02	1.00	7019	7.94E-06	TRUE	TRUE	482,730	19.98
Triacylglycerol (48:1) levels	Parkinson's disease	rs67093838	G	A	G	A	-2.20E-01	-9.00E-03	3.00E-02	9.43E-01	FALSE	FALSE	2	62350070	4.94E-02	6.74E-02	TRUE	2	62122935	4.94E-02	0.97	7019	8.76E-06	TRUE	TRUE	482,730	19.79
Triacylglycerol (48:1) levels	Parkinson's disease	rs6721083	C	G	C	G	1.58E-01	1.00E-02	9.37E-01	9.34E-01	TRUE	FALSE	2	171081652	4.72E-02	7.96E-02	TRUE	2	170225142	3.54E-02	0.95	7019	8.31E-06	TRUE	TRUE	482,730	19.89
Triacylglycerol (48:1) levels	Parkinson's disease	rs6804331	G	T	G	T	-8.03E-02	2.28E-02	3.37E-01	5.95E-01	FALSE	FALSE	3	103754387	2.27E-02	5.01E-01	TRUE	3	104035543	1.80E-02	0.99	7019	8.25E-06	TRUE	TRUE	482,730	19.91
Triacylglycerol (48:1) levels	Parkinson's disease	rs72753379	T	C	T	C	2.07E-01	2.30E-02	3.92E-02	9.80E-01	FALSE	FALSE	9	123112954	9.08E-02	9.67E-02	TRUE	9	120350676	4.53E-02	0.95	7019	4.90E-06	TRUE	TRUE	482,730	20.91
Triacylglycerol (48:1) levels	Parkinson's disease	rs75178403	C	T	C	T	-4.14E-01	9.64E-02	9.63E-03	9.79E-01	FALSE	FALSE	8	2683218	8.23E-02	6.17E-01	TRUE	8	2825696	8.99E-02	0.88	7019	4.19E-06	TRUE	TRUE	482,730	21.21
Triacylglycerol (48:1) levels	Parkinson's disease	rs7721676	C	T	C	T	-9.08E-02	-1.91E-02	2.18E-01	7.53E-01	FALSE	FALSE	5	149083207	2.55E-02	3.42E-01	TRUE	5	149703644	2.04E-02	1.00	7019	8.92E-06	TRUE	TRUE	482,730	19.76
Triacylglycerol (48:1) levels	Parkinson's disease	rs79416109	G	T	G	T	1.26E-01	5.42E-02	1.04E-01	9.34E-01	FALSE	FALSE	1	103312658	5.36E-02	5.06E-01	TRUE	1	102847102	2.83E-02	0.95	7019	8.66E-06	TRUE	TRUE	482,730	19.81
Triacylglycerol (48:1) levels	Parkinson's disease	rs964184	C	G	C	G	-1.29E-01	-5.68E-02	8.49E-01	8.63E-01	TRUE	FALSE	11	116648917	2.43E-02	9.71E-03	TRUE	11	116778201	2.34E-02	1.00	7019	3.48E-08	TRUE	TRUE	482,730	30.48

Triacylglycerol (48:2) levels	Parkinson's disease	rs10084264	T	G	T	G	1.72E-01	2.64E-02	5.91E-02	9.45E-01	FALSE	FALSE	2	164415281	5.11E-02	2.18E-01	TRUE	2	163558771	3.64E-02	0.95	7071	2.26E-06	TRUE	TRUE	482,730	22.40
Triacylglycerol (48:2) levels	Parkinson's disease	rs10861498	A	G	A	G	-8.80E-02	-1.05E-02	2.42E-01	8.15E-01	FALSE	FALSE	12	106151792	2.88E-02	1.46E-01	TRUE	12	105758014	1.93E-02	0.99	7071	5.21E-06	TRUE	TRUE	482,730	20.79
Triacylglycerol (48:2) levels	Parkinson's disease	rs117591451	A	G	A	G	-3.60E-01	-2.41E-02	1.31E-02	9.93E-01	FALSE	FALSE	8	40225327	2.27E-01	3.84E-02	TRUE	8	40367808	7.76E-02	0.92	7071	3.43E-06	TRUE	TRUE	482,730	21.59
Triacylglycerol (48:2) levels	Parkinson's disease	rs1260326	C	T	C	T	-1.38E-01	-6.78E-02	6.51E-01	4.20E-01	FALSE	FALSE	2	27730940	1.72E-02	4.04E-05	TRUE	2	27508073	1.75E-02	1.00	7071	4.46E-15	TRUE	TRUE	482,730	61.75
Triacylglycerol (48:2) levels	Parkinson's disease	rs138672261	A	G	A	G	2.82E-01	7.99E-02	2.02E-02	9.87E-01	FALSE	FALSE	8	86379014	1.20E-01	2.96E-01	TRUE	8	85466785	6.30E-02	0.93	7071	7.50E-06	TRUE	TRUE	482,730	20.09
Triacylglycerol (48:2) levels	Parkinson's disease	rs173848	T	A	T	A	8.29E-02	2.47E-02	6.63E-01	6.80E-01	TRUE	FALSE	7	30317163	2.62E-02	4.62E-01	TRUE	7	30277547	1.85E-02	0.93	7071	7.34E-06	TRUE	TRUE	482,730	20.13
Triacylglycerol (48:2) levels	Parkinson's disease	rs1832326	C	A	C	A	8.32E-02	-1.39E-02	7.25E-01	2.50E-01	FALSE	FALSE	9	33656917	2.18E-02	2.80E-01	TRUE	9	33656919	1.88E-02	1.00	7071	9.60E-06	TRUE	TRUE	482,730	19.62
Triacylglycerol (48:2) levels	Parkinson's disease	rs191616475	G	A	G	A	1.89E-01	2.15E-01	4.81E-02	9.51E-01	FALSE	FALSE	8	59242606	2.39E-01	4.33E-01	TRUE	8	58330047	3.88E-02	1.00	7071	1.15E-06	TRUE	TRUE	482,730	23.70
Triacylglycerol (48:2) levels	Parkinson's disease	rs3127050	A	G	A	G	8.85E-02	-1.83E-02	7.18E-01	2.83E-01	FALSE	FALSE	13	61406737	2.47E-02	3.38E-01	TRUE	13	60832603	1.86E-02	1.00	7071	1.87E-06	TRUE	TRUE	482,730	22.76
Triacylglycerol (48:2) levels	Parkinson's disease	rs56082136	T	C	T	C	1.74E-01	2.15E-02	5.26E-02	9.15E-01	FALSE	FALSE	12	52637176	3.69E-02	2.51E-01	TRUE	12	52243392	3.86E-02	0.93	7071	6.20E-06	TRUE	TRUE	482,730	20.45
Triacylglycerol (48:2) levels	Parkinson's disease	rs6664147	T	C	T	C	-9.59E-02	2.77E-02	1.90E-01	7.84E-01	FALSE	FALSE	1	227617519	2.38E-02	6.11E-01	TRUE	1	227429818	2.16E-02	1.00	7071	8.72E-06	TRUE	TRUE	482,730	19.80
Triacylglycerol (48:2) levels	Parkinson's disease	rs6804331	G	T	G	T	-8.13E-02	2.28E-02	3.37E-01	5.95E-01	FALSE	FALSE	3	103754387	2.27E-02	5.01E-01	TRUE	3	104035543	1.79E-02	0.99	7071	5.87E-06	TRUE	TRUE	482,730	20.56
Triacylglycerol (48:2) levels	Parkinson's disease	rs72738698	A	G	A	G	1.02E-01	-3.20E-02	1.79E-01	7.90E-01	FALSE	FALSE	9	82705280	2.76E-02	6.10E-01	TRUE	9	80090365	2.21E-02	0.99	7071	4.16E-06	TRUE	TRUE	482,730	21.22
Triacylglycerol (48:2) levels	Parkinson's disease	rs72753379	T	C	T	C	2.08E-01	2.30E-02	3.92E-02	9.80E-01	FALSE	FALSE	9	123112954	9.08E-02	9.67E-02	TRUE	9	120350676	4.51E-02	0.95	7071	3.94E-06	TRUE	TRUE	482,730	21.33
Triacylglycerol (48:2) levels	Parkinson's disease	rs79934453	G	A	G	A	-6.10E-01	-9.28E-02	4.04E-03	9.90E-01	FALSE	FALSE	6	23919234	1.77E-01	2.22E-01	TRUE	6	23919006	1.37E-01	0.91	7071	8.65E-06	TRUE	TRUE	482,730	19.82
Triacylglycerol (48:2) levels	Parkinson's disease	rs964184	C	G	C	G	-1.43E-01	-5.68E-02	8.49E-01	8.63E-01	TRUE	FALSE	11	116648917	2.43E-02	9.71E-03	TRUE	11	116778201	2.33E-02	1.00	7071	9.60E-10	TRUE	TRUE	482,730	37.50
Triacylglycerol (48:3) levels	Parkinson's disease	rs1042034	T	C	T	C	8.98E-02	-5.30E-03	7.28E-01	2.18E-01	FALSE	FALSE	2	21225281	2.03E-02	9.96E-02	TRUE	2	21002409	1.90E-02	1.00	6855	2.44E-06	TRUE	TRUE	482,730	22.25
Triacylglycerol (48:3) levels	Parkinson's disease	rs10861498	A	G	A	G	-9.14E-02	-1.05E-02	2.42E-01	8.15E-01	FALSE	FALSE	12	106151792	2.88E-02	1.46E-01	TRUE	12	105758014	1.96E-02	0.99	6855	3.26E-06	TRUE	TRUE	482,730	21.69
Triacylglycerol (48:3) levels	Parkinson's disease	rs111430300	G	A	G	A	-1.11E-01	2.48E-02	1.35E-01	8.07E-01	FALSE	FALSE	2	36618053	2.76E-02	4.33E-01	TRUE	2	36390910	2.47E-02	1.00	6855	6.78E-06	TRUE	TRUE	482,730	20.29
Triacylglycerol (48:3) levels	Parkinson's disease	rs112578388	T	C	T	C	-5.61E-01	-2.36E-02	5.33E-03	9.88E-01	FALSE	FALSE	13	26460581	2.04E-01	4.20E-02	TRUE	13	25886443	1.24E-01	0.86	6855	6.36E-06	TRUE	TRUE	482,730	20.41
Triacylglycerol (48:3) levels	Parkinson's disease	rs117591451	A	G	A	G	-3.87E-01	-2.41E-02	1.31E-02	9.93E-01	FALSE	FALSE	8	40225327	2.27E-01	3.84E-02	TRUE	8	40367808	7.78E-02	0.92	6855	6.57E-07	TRUE	TRUE	482,730	24.78
Triacylglycerol (48:3) levels	Parkinson's disease	rs1260326	C	T	C	T	-1.42E-01	-6.78E-02	6.51E-01	4.20E-01	FALSE	FALSE	2	27730940	1.72E-02	4.04E-05	TRUE	2	27508073	1.78E-02	1.00	6855	1.98E-15	TRUE	TRUE	482,730	63.37
Triacylglycerol (48:3) levels	Parkinson's disease	rs191616475	G	A	G	A	2.05E-01	2.15E-01	4.81E-02	9.51E-01	FALSE	FALSE	8	59242606	2.39E-01	4.33E-01	TRUE	8	58330047	3.95E-02	1.00	6855	2.08E-07	TRUE	TRUE	482,730	27.01
Triacylglycerol (48:3) levels	Parkinson's disease	rs2280065	A	G	A	G	-1.38E-01	3.32E-02	8.62E-02	8.47E-01	FALSE	FALSE	15	57762740	3.29E-02	5.04E-01	TRUE	15	57470542	3.12E-02	0.93	6855	9.58E-06	TRUE	TRUE	482,730	19.62
Triacylglycerol (48:3) levels	Parkinson's disease	rs4353433	A	C	A	C	7.60E-02	1.81E-02	5.78E-01	3.48E-01	FALSE	FALSE	14	94417802	2.15E-02	3.99E-01	TRUE	14	93951456	1.72E-02	1.00	6855	9.85E-06	TRUE	TRUE	482,730	19.57
Triacylglycerol (48:3) levels	Parkinson's disease	rs56082136	T	C	T	C	1.89E-01	2.15E-02	5.26E-02	9.15E-01	FALSE	FALSE	12	52637176	3.69E-02	2.51E-01	TRUE	12	52243392	3.93E-02	0.93	6855	1.49E-06	TRUE	TRUE	482,730	23.20
Triacylglycerol (48:3) levels	Parkinson's disease	rs62410903	T	C	T	C	8.25E-02	2.21E-02	3.35E-01	5.85E-01	FALSE	FALSE	4	44886410	2.03E-02	5.58E-01	TRUE	4	44884393	1.82E-02	1.00	6855	5.78E-06	TRUE	TRUE	482,730	20.59
Triacylglycerol (48:3) levels	Parkinson's disease	rs72738698	A	G	A	G	1.09E-01	-3.20E-02	1.79E-01	7.90E-01	FALSE	FALSE	9	82705280	2.76E-02	6.10E-01	TRUE	9	80090365	2.24E-02	0.99	6855	1.25E-06	TRUE	TRUE	482,730	23.53
Triacylglycerol (48:3) levels	Parkinson's disease	rs77745982	T	C	T	C	-1.41E-01	3.97E-02	8.08E-02	9.01E-01	FALSE	FALSE	13	21649012	3.72E-02	5.42E-01	TRUE	13	21074873	3.16E-02	1.00	6855	8.49E-06	TRUE	TRUE	482,730	19.85
Triacylglycerol (48:3) levels	Parkinson's disease	rs8047793	T	C	T	C	-1.09E-01	-1.49E-02	1.44E-01	8.64E-01	FALSE	FALSE	16	84577993	3.22E-02	1.91E-01	TRUE	16	84544387	2.46E-02	1.00	6855	9.34E-06	TRUE	TRUE	482,730	19.67
Triacylglycerol (48:3) levels	Parkinson's disease	rs820903	G	A	G	A	1.46E-01	-1.46E-02	9.17E-01	1.61E-01	FALSE	FALSE	11	38182666	3.05E-02	1.99E-01	TRUE	11	38161116	3.22E-02	0.95	6855	5.39E-06	TRUE	TRUE	482,730	20.72
Triacylglycerol (48:3) levels	Parkinson's disease	rs964184	C	G	C	G	-1.63E-01	-5.68E-02	8.49E-01	8.63E-01	TRUE	FALSE	11	116648917	2.43E-02	9.71E-03	TRUE	11	116778201	2.37E-02	1.00	6855	6.67E-12	TRUE	TRUE	482,730	47.28
Triacylglycerol (48:3) levels	Parkinson's disease	rs9796793	A	G	A	G	-1.26E-01	-6.30E-02	1.25E-01	9.58E-01	FALSE	FALSE	16	30495652	5.46E-02	6.04E-01	TRUE	16	30484331	2.63E-02	0.95	6855	1.80E-06	TRUE	TRUE	482,730	22.83
Triacylglycerol (48:3) levels	Parkinson's disease	rs9837572	T	G	T	G	-9.25E-02	2.68E-02	2.36E-01	8.28E-01	FALSE	FALSE	3	72309600	3.00E-02	4.29E-01	TRUE	3	72260449	2.02E-02	0.99	6855	4.82E-06	TRUE	TRUE	482,730	20.94
Triacylglycerol (49:1) levels	Parkinson's disease	rs10812660	A	T	A	T	8.90E-02	2.06E-02	3.75E-01	3.26E-01	TRUE	FALSE	9	27742535	2.93E-02	3.18E-01	TRUE	9	27742537	1.98E-02	0.93	5843	7.02E-06	TRUE	TRUE	482,730	20.22
Triacylglycerol (49:1) levels	Parkinson's disease	rs10861498	A	G	A	G	-9.99E-02	-1.05E-02	2.42E-01	8.15E-01	FALSE	FALSE	12	106151792	2.88E-02	1.46E-01	TRUE	12	105758014	2.14E-02	0.99	5843	3.17E-06	TRUE	TRUE	482,730	21.75
Triacylglycerol (49:1) levels	Parkinson's disease	rs112902435	T	C	T	C	1.83E-01	-1.49E-02	5.59E-02	9.47E-01	FALSE	FALSE	5	149115470	4.62E-02	1.27E-01	TRUE	5	149735907	4.04E-02	0.98	5843	5.99E-06	TRUE	TRUE	482,730	20.53
Triacylglycerol (49:1) levels	Parkinson's disease	rs117591451	A	G	A	G	-4.08E-01	-2.41E-02	1.31E-02	9.93E-01	FALSE	FALSE	8	40225327	2.27E-01	3.84E-02	TRUE	8	40367808	8.54E-02	0.92	5843	1.87E-06	TRUE	TRUE	482,730	22.77
Triacylglycerol (49:1) levels	Parkinson's disease	rs1233660	A	T	A	T	-1.02E-01	-2.80E-03	2.21E-01	3.51E-01	TRUE	FALSE	6	28260249	1.81E-02	5.72E-02	TRUE	6	28292472	2.21E-02	1.00	5843	4.45E-06	TRUE	TRUE	482,730	21.10
Triacylglycerol (49:1) levels	Parkinson's disease	rs1260326	C	T	C	T	-9.44E-02	-6.78E-02	6.51E-01	4.20E-01	FALSE	FALSE	2	27730940	1.72E-02	4.04E-05	TRUE	2	27508073	1.93E-02	1.00	5843	1.02E-06	TRUE	TRUE	482,730	23.93
Triacylglycerol (49:1) levels	Parkinson's disease	rs138672261	A	G	A	G	3.08E-01	7.99E-02	2.02E-02	9.87E-01	FALSE	FALSE	8	86379014	1.20E-01	2.96E-01	TRUE	8	85466785	6.87E-02	0.93	5843	7.60E-06	TRUE	TRUE	482,730	20.07
Triacylglycerol (49:1) levels	Parkinson's disease	rs146238333	T	A	T	A	6.27E-01	5.84E-02	5.21E-03	1.67E-02	TRUE	FALSE	12	84101712	9.77E-02	2.60E-01	TRUE	12	83707933	1.34E-01	0.89	5843	2.75E-06	TRUE	TRUE	482,730	22.03
Triacylglycerol (49:1) levels	Parkinson's disease	rs149371861	A	G	A	G	2.31E-01	6.46E-02	3.58E-02	9.57E-01	FALSE	FALSE	2	242637785	5.20E-02	6.71E-01	TRUE	2	241698370	5.06E-02	0.98	5843	5.13E-06	TRUE	TRUE	482,730	20.82
Triacylglycerol (49:1) levels	Parkinson's disease	rs16970164	C	A	C	A	-2.11E-01	1.01E-01	3.94E-02	9.70E-01	FALSE	FALSE	15	40292090	5.79E-02	4.13E-02	TRUE	15	39999889	4.77E-02	0.99	5843	9.81E-06	TRUE	TRUE	482,730	19.58
Triacylglycerol (49:1) levels	Parkinson's disease	rs2004776	T	C	T	C	-1.05E-01	2.69E-02	1.96E-01	7.61E-01	FALSE	FALSE	1	230848702	2.05E-02	7.19E-01	TRUE	1	230712956	2.35E-02	1.00	5843	8.53E-06	TRUE	TRUE	482,730	19.85
Triacylglycerol (49:1) levels	Parkinson's disease	rs2031531	T	C	T	C	-1.02E-01	3.59E-02	2.06E-01	8.32E-01	FALSE	FALSE	13	50011149	2.63E-02	7.63E-01	TRUE	13	49437013	2.30E-02	0.98	5843	9.14E-06	TRUE	TRUE	482,730	19.72
Triacylglycerol (49:1) levels	Parkinson's disease	rs33951980	T	C	T	C	-1.30E-01	-6.00E-03	1.22E-01	8.79E-01	FALSE	FALSE	7	73029437	2.71E-02	8.41E-02	TRUE	7	73615107	2.84E-02	1.00	5843	4.91E-06	TRUE	TRUE	482,730	20.91
Triacylglycerol (49:1) levels	Parkinson's disease	rs34379959	C	T	C	T	1.50E-01	2.27E-02	8.77E-02	9.22E-01	FALSE	FALSE	2	12637503	4.50E-02	2.12E-01	TRUE	2	12497377	3.37E-02	0.96	5843	9.49E-06	TRUE	TRUE	482,730	19.65
Triacylglycerol (49:1) levels	Parkinson's disease	rs6429254	T	C	T	C	8.50E-02	1.00E-04	3.65E-01	6.37E-01	FALSE	FALSE	1	241384872	2.30E-02	9.57E-04	TRUE	1	241221572	1.91E-02	1.00	5843	8.53E-06	TRUE	TRUE	482,730	19.85
Triacylglycerol (49:1) levels	Parkinson's disease	rs71620944	T	G	T	G	1.90E-01	8.87E-02	5.15E-02	9.66E-01	FALSE	FALSE	4	183657915	6.98E-02	6.90E-01	TRUE	4	182736762	4.29E-02	0.96	5843	9.58E-06	TRUE	TRUE	482,730	19.63
Triacylglycerol (49:1) levels	Parkinson's disease	rs72753379	T	C	T	C	2.32E-01	2.30E-02	3.92E-02	9.80E-01	FALSE	FALSE	9	123112954	9.08E-02	9.67E-02	TRUE	9	120350676	4.96E-02	0.95	5843	2.96E-06	TRUE	TRUE	482,730	21.88
Triacylglycerol (49:1) levels	Parkinson's disease	rs73216924	G	T	G	T	-1.30E-01	4.78E-02	1.18E-01	8.57E-01	FALSE	FALSE	4	35019797	2.87E-02	4.79E-02	TRUE	4	35018175	2.88E-02	1.00	5843	6.80E-06	TRUE	TRUE	482,730	20.28
Triacylglycerol (49:1) levels	Parkinson's disease	rs73259585	G	A	G	A	-2.27E-01	2.69E-02	3.72E-02	9.66E-01	FALSE	FALSE	14	30287597	6.23E-02	1.76E-01	TRUE	14	29818391	5.01E-02	0.98	5843	5.85E-06	TRUE	TRUE	482,730	20.57
Triacylglycerol (49:1) levels	Parkinson's disease	rs79725259	G	C	G	C	-3.13E-01	-9.23E-02	1.81E-02	3.56E-02	TRUE	FALSE	5	107962480	6.21E-02	8.63E-01	TRUE	5	108626779	6.93E-02	0.97	5843	6.43E-06	TRUE	TRUE	482,730	20.39
Triacylglycerol (49:1) levels	Parkinson's disease	rs964184	C	G	C	G	-1.31E-01	-5.68E-02	8.49E-01	8.63E-01	TRUE	FALSE	11	116648917	2.43E-02	9.71E-03	TRUE	11	116778201	2.53E-02	1.00	5843	2.24E-07	TRUE	TRUE	482,730	26.88
Triacylglycerol (49:2) levels	Parkinson's disease	rs10468471	C	T	C	T	-1.64E-01	-6.22E-02	6.54E-02	9.40E-01	FALSE	FALSE	17	12125885	4.71E-02	7.29E-01	TRUE	17	12222568	3.70E-02	0.98	6076	9.83E-06	TRUE	TRUE	482,730	19.58
Triacylglycerol (49:2) levels	Parkinson's disease	rs10861498	A	G	A	G	-9.96E-02	-1.05E-02	2.42E-01	8.15E-01	FALSE	FALSE	12	106151792	2.88E-02	1.46E-01	TRUE	12	105758014	2.10E-02	0.99	6076	2.27E-06	TRUE	TRUE	482,730	22.39
Triacylglycerol (49:2) levels	Parkinson's disease	rs1260326	C	T	C	T	-1.20E-01	-6.78E-02	6.51E-01	4.20E-01	FALSE	FALSE	2	27730940	1.72E-02	4.04E-05	TRUE	2	27508073	1.89E-02	1.00	6076	2.23E-10	TRUE	TRUE	482,730	40.39
Triacylglycerol (49:2) levels	Parkinson's disease	rs143838492	A	G	A	G	7.58E-01	1.82E-01	3.33E-03	9.90E-01	FALSE	FALSE	5	114228685	1.54E-01	6.27E-01	TRUE	5	114892988	1.59E-01	0.97	6076	1.88E-06	TRUE	TRUE	482,730	22.76
Triacylglycerol (49:2) levels	Parkinson's disease	rs145055632	G	A	G	A	-5.16E-01	6.11E-02	6.40E-03	9.87E-01	FALSE	FALSE	6	50750809	1.06E-01	2.49E-01	TRUE	6	50783096	1.12E-01	0.99	6076	4.36E-06	TRUE	TRUE	482,730	21.14
Triacylglycerol (49:2) levels	Parkinson's disease	rs2736863	G	T	G	T	4.63E-01	1.17E-01	9.92E-01	2.17E-02	FALSE	FALSE	8	134463832	8.11E-02	8.24E-01	TRUE	8	133451589	1.01E-01	0.96	6076	4.81E-06	TRUE	TRUE	482,730	20.95
Triacylglycerol (49:2) levels	Parkinson's disease	rs4937417	G	A	G	A	-8.49E-02	-3.74E-02	4.67E-01	5.57E-01	FALSE	FALSE	11	129218604	2.24E-02	4.75E-02	TRUE	11	129348709	1.83E-02	1.00	6076	3.39E-06	TRUE	TRUE	482,730	21.62
Triacylglycerol (49:2) levels	Parkinson's disease	rs6846577	A	G	A	G	-1.01E-01	-4.00E-03	2.15E-01	7.91E-01	FALSE	FALSE	4	65233589	2.19E-02	6.85E-02	TRUE	4	64367871	2.19E-02	1.00	6076	4.27E-06	TRUE	TRUE	482,730	21.18
Triacylglycerol (49:2) levels	Parkinson's disease	rs76411296	T	G	T	G	-1.36E-01	-7.10E-03	1.16E-01	8.66E-01	FALSE	FALSE	15	59809619	3.41E-02	7.87E-02	TRUE	15	59517420	2.87E-02	0.98	6076	2.28E-06	TRUE	TRUE	482,730	22.38
Triacylglycerol (49:2) levels	Parkinson's disease	rs964184	C	G	C	G	-1.55E-01	-5.68E-02	8.49E-01	8.63E-01	TRUE	FALSE	11	116648917	2.43E-02	9.71E-03	TRUE	11	116778201	2.49E-02	1.00	6076	5.55E-10	TRUE	TRUE	482,730	38.60
Triacylglycerol (50:1) levels	Parkinson's disease	rs1061808	G	T	G	T	9.98E-02	-2.62E-02	5.84E-01	3.18E-01	FALSE	FALSE	6	32136547	2.05E-02	6.96E-01	TRUE	6	32168770	1.69E-02	1.00	7143	3.91E-09	TRUE	TRUE	482,730	34.75
Triacylglycerol (50:1) levels	Parkinson's disease	rs10861498	A	G	A	G	-8.94E-02	-1.05E-02	2.42E-01	8.15E-01	FALSE	FALSE	12	106151792	2.88E-02	1.46E-01	TRUE	12	105758014	1.92E-02	0.99	7143	3.20E-06	TRUE	TRUE	482,730	21.72
Triacylglycerol (50:1) levels	Parkinson's disease	rs112122274	G	A	G	A	-2.40E-01	4.76E-02	3.60E-02	9.49E-01	FALSE	FALSE	9	139149840	6.87E-02	3.11E-01	TRUE	9	136257994	4.86E-02	0.85	7143	7.85E-07	TRUE	TRUE	482,730	24.44
Triacylglycerol (50:1) levels	Parkinson's disease	rs113739902	C	A	C	A	-5.72E-01	2.02E-01	4.89E-03	9.89E-01	FALSE	FALSE	6	67163155	1.65E-01	6.52E-01	TRUE	6	66453262	1.28E-01	0.92	7143	7.97E-06	TRUE	TRUE	482,730	19.97
Triacylglycerol (50:1) levels	Parkinson's disease	rs1260326	C	T	C	T	-1.09E-01	-6.78E-02	6.51E-01	4.20E-01	FALSE	FALSE	2	27730940	1.72E-02	4.04E-05	TRUE	2	27508073	1.75E-02	1.00	7143	4.18E-10	TRUE	TRUE	482,730	39.13
Triacylglycerol (50:1) levels	Parkinson's disease	rs1399627	G	A	G	A	8.34E-02	-2.80E-03	6.32E-01	3.52E-01	FALSE	FALSE	2	227083411	1.81E-02	5.71E-02	TRUE	2	226218695	1.74E-02	1.00	7143	1.76E-06	TRUE	TRUE	482,730	22.88
Triacylglycerol (50:1) levels	Parkinson's disease	rs143838492	A	G	A	G	6.73E-01	1.82E-01	3.33E-03	9.90E-01	FALSE	FALSE	5	114228685	1.54E-01	6.27E-01	TRUE	5	114892988	1.49E-01	0.97	7143	6.07E-06	TRUE	TRUE	482,730	20.50
Triacylglycerol (50:1) levels	Parkinson's disease	rs144960920	T	C	T	C	-2.34E-01	6.79E-02	2.71E-02	9.87E-01	FALSE	FALSE	3	122379712	8.54E-02	3.70E-01	TRUE	3	122660865	5.20E-02	0.99	7143	6.84E-06	TRUE	TRUE	482,730	20.27
Triacylglycerol (50:1) levels	Parkinson's disease	rs187952596	G	A	G	A	2.14E-01	6.50E-03	3.25E-02	9.71E-01	FALSE	FALSE	17	76116021	6.60E-02	3.53E-02	TRUE	17	78119940	4.79E-02	0.94	7143	8.37E-06	TRUE	TRUE	482,730	19.88
Triacylglycerol (50:1) levels	Parkinson's disease	rs480021	A	C	A	C	7.87E-02	-2.13E-02	3.40E-01	6.50E-01	FALSE	FALSE	9	135330975	1.94E-02	5.66E-01	TRUE	9	132455588	1.76E-02	1.00	7143	7.35E-06	TRUE	TRUE	482,730	20.13
Triacylglycerol (50:1) levels	Parkinson's disease	rs55857911	T	C	T	C	1.48E-01	-1.25E-02	9.25E-02	8.70E-01	FALSE	FALSE	1	230733978	3.62E-02	1.37E-01	TRUE	1	230598232	3.01E-02	0.92	7143	9.79E-07	TRUE	TRUE	482,730	24.01
Triacylglycerol (50:1) levels	Parkinson's disease	rs61906420	A	G	A	G	6.75E-01	4.46E-02	4.06E-03	9.85E-01	FALSE	FALSE	11	132530817	1.08E-01	1.67E-01	TRUE	11	132660922	1.41E-01	0.85	7143	1.64E-06	TRUE	TRUE	482,730	23.02
Triacylglycerol (50:1) levels	Parkinson's disease	rs62016118	C	A	C	A	-1.77E-01	6.00E-03	5.08E-02	9.41E-01	FALSE	FALSE	16	6841692	4.90E-02	4.43E-02	TRUE	16	6791691	3.84E-02	0.97	7143	4.08E-06	TRUE	TRUE	482,730	21.26
Triacylglycerol (50:1) levels	Parkinson's disease	rs6804331	G	T	G	T	-7.92E-02	2.28E-02	3.37E-01	5.95E-01	FALSE	FALSE	3	103754387	2.27E-02	5.01E-01	TRUE	3	104035543	1.78E-02	0.99	7143	9.07E-06	TRUE	TRUE	482,730	19.72
Triacylglycerol (50:1) levels	Parkinson's disease	rs72753379	T	C	T	C	2.04E-01	2.30E-02	3.92E-02	9.80E-01	FALSE	FALSE	9	123112954	9.08E-02	9.67E-02	TRUE	9	120350676	4.48E-02	0.95	7143	5.18E-06	TRUE	TRUE	482,730	20.80
Triacylglycerol (50:1) levels	Parkinson's disease	rs75178403	C	T	C	T	-3.98E-01	9.64E-02	9.63E-03	9.79E-01	FALSE	FALSE	8	2683218	8.23E-02	6.17E-01	TRUE	8	2825696	8.98E-02	0.88	7143	9.44E-06	TRUE	TRUE	482,730	19.65
Triacylglycerol (50:1) levels	Parkinson's disease	rs75931581	C	T	C	T	-2.02E-01	6.20E-02	4.19E-02	9.66E-01	FALSE	FALSE	15	40286536	5.44E-02	5.94E-01	TRUE	15	39994335	4.14E-02	0.99	7143	1.13E-06	TRUE	TRUE	482,730	23.73
Triacylglycerol (50:1) levels	Parkinson's disease	rs78275146	T	C	T	C	3.37E-01	7.90E-02	1.38E-02	9.87E-01	FALSE	FALSE	1	195998089	1.03E-01	3.52E-01	TRUE	1	196028959	7.56E-02	0.92	7143	8.42E-06	TRUE	TRUE	482,730	19.87
Triacylglycerol (50:1) levels	Parkinson's disease	rs79416109	G	T	G	T	1.33E-01	5.42E-02	1.04E-01	9.34E-01	FALSE	FALSE	1	103312658	5.36E-02	5.06E-01	TRUE	1	102847102	2.81E-02	0.95	7143	2.05E-06	TRUE	TRUE	482,730	22.59
Triacylglycerol (50:1) levels	Parkinson's disease	rs79934453	G	A	G	A	-6.16E-01	-9.28E-02	4.04E-03	9.90E-01	FALSE	FALSE	6	23919234	1.77E-01	2.22E-01	TRUE	6	23919006	1.37E-01	0.91	7143	7.04E-06	TRUE	TRUE	482,730	20.21
Triacylglycerol (50:1) levels	Parkinson's disease	rs9324675	T	C	T	C	1.01E-01	-5.92E-02	1.66E-01	8.66E-01	FALSE	FALSE	5	150489817	2.93E-02	2.17E-02	TRUE	5	151110256	2.26E-02	0.99	7143	8.16E-06	TRUE	TRUE	482,730	19.93
Triacylglycerol (50:1) levels	Parkinson's disease	rs964184	C	G	C	G	-1.62E-01	-5.68E-02	8.49E-01	8.63E-01	TRUE	FALSE	11	116648917	2.43E-02	9.71E-03	TRUE	11	116778201	2.32E-02	1.00	7143	3.12E-12	TRUE	TRUE	482,730	48.78
Triacylglycerol (50:2) levels	Parkinson's disease	rs1010313	T	C	T	C	9.27E-02	2.37E-02	1.98E-01	8.77E-01	FALSE	FALSE	8	60002542	2.73E-02	4.15E-01	TRUE	8	59089983	2.08E-02	1.00	7172	8.23E-06	TRUE	TRUE	482,730	19.91
Triacylglycerol (50:2) levels	Parkinson's disease	rs10213073	G	T	G	T	-8.09E-02	1.55E-02	3.06E-01	7.01E-01	FALSE	FALSE	4	82445334	1.99E-02	3.61E-01	TRUE	4	81524180	1.82E-02	1.00	7172	9.29E-06	TRUE	TRUE	482,730	19.68
Triacylglycerol (50:2) levels	Parkinson's disease	rs10861498	A	G	A	G	-8.46E-02	-1.05E-02	2.42E-01	8.15E-01	FALSE	FALSE	12	106151792	2.88E-02	1.46E-01	TRUE	12	105758014	1.91E-02	0.99	7172	9.89E-06	TRUE	TRUE	482,730	19.56
Triacylglycerol (50:2) levels	Parkinson's disease	rs111430300	G	A	G	A	-1.12E-01	2.48E-02	1.35E-01	8.07E-01	FALSE	FALSE	2	36618053	2.76E-02	4.33E-01	TRUE	2	36390910	2.42E-02	1.00	7172	3.36E-06	TRUE	TRUE	482,730	21.63
Triacylglycerol (50:2) levels	Parkinson's disease	rs112122274	G	A	G	A	-2.19E-01	4.76E-02	3.60E-02	9.49E-01	FALSE	FALSE	9	139149840	6.87E-02	3.11E-01	TRUE	9	136257994	4.85E-02	0.85	7172	6.84E-06	TRUE	TRUE	482,730	20.27
Triacylglycerol (50:2) levels	Parkinson's disease	rs1260326	C	T	C	T	-1.39E-01	-6.78E-02	6.51E-01	4.20E-01	FALSE	FALSE	2	27730940	1.72E-02	4.04E-05	TRUE	2	27508073	1.74E-02	1.00	7172	1.53E-15	TRUE	TRUE	482,730	63.88
Triacylglycerol (50:2) levels	Parkinson's disease	rs143838492	A	G	A	G	7.23E-01	1.82E-01	3.33E-03	9.90E-01	FALSE	FALSE	5	114228685	1.54E-01	6.27E-01	TRUE	5	114892988	1.48E-01	0.97	7172	1.15E-06	TRUE	TRUE	482,730	23.71
Triacylglycerol (50:2) levels	Parkinson's disease	rs17704001	T	G	T	G	8.89E-02	1.20E-02	2.26E-01	7.50E-01	FALSE	FALSE	15	93127165	2.58E-02	1.92E-01	TRUE	15	92583935	2.00E-02	1.00	7172	9.24E-06	TRUE	TRUE	482,730	19.69
Triacylglycerol (50:2) levels	Parkinson's disease	rs4615671	T	C	T	C	1.62E-01	-3.50E-03	5.82E-02	9.26E-01	FALSE	FALSE	9	24032281	4.55E-02	2.74E-02	TRUE	9	24032283	3.60E-02	0.95	7172	7.13E-06	TRUE	TRUE	482,730	20.19
Triacylglycerol (50:2) levels	Parkinson's disease	rs72753379	T	C	T	C	2.16E-01	2.30E-02	3.92E-02	9.80E-01	FALSE	FALSE	9	123112954	9.08E-02	9.67E-02	TRUE	9	120350676	4.47E-02	0.95	7172	1.42E-06	TRUE	TRUE	482,730	23.29
Triacylglycerol (50:2) levels	Parkinson's disease	rs78275146	T	C	T	C	3.40E-01	7.90E-02	1.38E-02	9.87E-01	FALSE	FALSE	1	195998089	1.03E-01	3.52E-01	TRUE	1	196028959	7.53E-02	0.92	7172	6.59E-06	TRUE	TRUE	482,730	20.33
Triacylglycerol (50:2) levels	Parkinson's disease	rs79416109	G	T	G	T	1.28E-01	5.42E-02	1.04E-01	9.34E-01	FALSE	FALSE	1	103312658	5.36E-02	5.06E-01	TRUE	1	102847102	2.80E-02	0.95	7172	5.23E-06	TRUE	TRUE	482,730	20.78
Triacylglycerol (50:2) levels	Parkinson's disease	rs79934453	G	A	G	A	-6.43E-01	-9.28E-02	4.04E-03	9.90E-01	FALSE	FALSE	6	23919234	1.77E-01	2.22E-01	TRUE	6	23919006	1.37E-01	0.91	7172	2.72E-06	TRUE	TRUE	482,730	22.04
Triacylglycerol (50:2) levels	Parkinson's disease	rs964184	C	G	C	G	-1.84E-01	-5.68E-02	8.49E-01	8.63E-01	TRUE	FALSE	11	116648917	2.43E-02	9.71E-03	TRUE	11	116778201	2.31E-02	1.00	7172	2.10E-15	TRUE	TRUE	482,730	63.24
Triacylglycerol (50:3) levels	Parkinson's disease	rs10105606	A	C	A	C	-1.01E-01	0.00E+00	2.79E-01	6.64E-01	FALSE	FALSE	8	19827848	1.77E-02	4.34E-05	TRUE	8	19970337	1.85E-02	1.00	7161	5.10E-08	TRUE	TRUE	482,730	29.74
Triacylglycerol (50:3) levels	Parkinson's disease	rs1042034	T	C	T	C	9.86E-02	-5.30E-03	7.28E-01	2.18E-01	FALSE	FALSE	2	21225281	2.03E-02	9.96E-02	TRUE	2	21002409	1.86E-02	1.00	7161	1.25E-07	TRUE	TRUE	482,730	27.99
Triacylglycerol (50:3) levels	Parkinson's disease	rs10741876	G	A	G	A	-1.01E-01	5.08E-02	8.32E-01	2.12E-01	FALSE	FALSE	11	3250208	2.49E-02	2.07E-02	TRUE	11	3228978	2.24E-02	0.99	7161	6.10E-06	TRUE	TRUE	482,730	20.49
Triacylglycerol (50:3) levels	Parkinson's disease	rs111430300	G	A	G	A	-1.11E-01	2.48E-02	1.35E-01	8.07E-01	FALSE	FALSE	2	36618053	2.76E-02	4.33E-01	TRUE	2	36390910	2.42E-02	1.00	7161	4.66E-06	TRUE	TRUE	482,730	21.00
Triacylglycerol (50:3) levels	Parkinson's disease	rs111526079	A	G	A	G	1.21E-01	5.76E-02	1.05E-01	9.31E-01	FALSE	FALSE	14	77437466	4.73E-02	6.52E-01	TRUE	14	76971123	2.74E-02	0.96	7161	9.51E-06	TRUE	TRUE	482,730	19.63
Triacylglycerol (50:3) levels	Parkinson's disease	rs116011373	A	G	A	G	-1.86E-01	-2.23E-02	4.88E-02	9.73E-01	FALSE	FALSE	3	87347791	8.48E-02	1.01E-01	TRUE	3	87298641	3.92E-02	0.96	7161	2.25E-06	TRUE	TRUE	482,730	22.41
Triacylglycerol (50:3) levels	Parkinson's disease	rs11849573	A	G	A	G	-7.51E-02	-1.68E-02	4.87E-01	5.04E-01	FALSE	FALSE	14	96033076	1.91E-02	4.23E-01	TRUE	14	95566739	1.65E-02	1.00	7161	5.58E-06	TRUE	TRUE	482,730	20.66
Triacylglycerol (50:3) levels	Parkinson's disease	rs12120691	C	T	C	T	-1.15E-01	3.59E-02	1.23E-01	9.03E-01	FALSE	FALSE	1	227215490	3.12E-02	6.03E-01	TRUE	1	227027789	2.56E-02	0.99	7161	6.66E-06	TRUE	TRUE	482,730	20.32
Triacylglycerol (50:3) levels	Parkinson's disease	rs12344868	A	C	A	C	2.94E-01	-1.55E-01	1.71E-02	9.77E-01	FALSE	FALSE	9	133447901	8.44E-02	3.29E-02	TRUE	9	130572514	6.64E-02	0.99	7161	9.56E-06	TRUE	TRUE	482,730	19.63
Triacylglycerol (50:3) levels	Parkinson's disease	rs1260326	C	T	C	T	-1.59E-01	-6.78E-02	6.51E-01	4.20E-01	FALSE	FALSE	2	27730940	1.72E-02	4.04E-05	TRUE	2	27508073	1.74E-02	1.00	7161	7.41E-20	TRUE	TRUE	482,730	83.68
Triacylglycerol (50:3) levels	Parkinson's disease	rs139499638	C	G	C	G	-6.43E-01	7.96E-02	4.41E-03	2.11E-02	TRUE	FALSE	4	121751160	8.87E-02	4.32E-01	TRUE	4	120830005	1.36E-01	0.92	7161	2.35E-06	TRUE	TRUE	482,730	22.32
Triacylglycerol (50:3) levels	Parkinson's disease	rs1428264	C	A	C	A	9.87E-02	2.10E-02	1.97E-01	8.77E-01	FALSE	FALSE	8	59994774	2.73E-02	3.55E-01	TRUE	8	59082215	2.09E-02	1.00	7161	2.24E-06	TRUE	TRUE	482,730	22.41
Triacylglycerol (50:3) levels	Parkinson's disease	rs143838492	A	G	A	G	6.76E-01	1.82E-01	3.33E-03	9.90E-01	FALSE	FALSE	5	114228685	1.54E-01	6.27E-01	TRUE	5	114892988	1.48E-01	0.97	7161	5.34E-06	TRUE	TRUE	482,730	20.74
Triacylglycerol (50:3) levels	Parkinson's disease	rs17704001	T	G	T	G	8.96E-02	1.20E-02	2.26E-01	7.50E-01	FALSE	FALSE	15	93127165	2.58E-02	1.92E-01	TRUE	15	92583935	2.00E-02	1.00	7161	7.71E-06	TRUE	TRUE	482,730	20.04
Triacylglycerol (50:3) levels	Parkinson's disease	rs35368205	T	C	T	C	-1.03E-01	3.10E-02	1.67E-01	8.17E-01	FALSE	FALSE	7	73017657	2.27E-02	7.64E-01	TRUE	7	73603327	2.23E-02	1.00	7161	4.03E-06	TRUE	TRUE	482,730	21.28

Triacylglycerol (50:3) levels	Parkinson's disease	rs35986054	C	T	C	T	9.59E-02	-1.07E-02	1.96E-01	8.03E-01	FALSE	FALSE	17	1526343	2.45E-02	1.79E-01	TRUE	17	1623049	2.14E-02	0.98	7161	7.59E-06	TRUE	TRUE	482,730	20.07
Triacylglycerol (50:3) levels	Parkinson's disease	rs72697234	G	A	G	A	2.11E-01	8.75E-02	3.57E-02	9.59E-01	FALSE	FALSE	14	93005264	7.44E-02	6.20E-01	TRUE	14	92538920	4.64E-02	0.93	7161	5.49E-06	TRUE	TRUE	482,730	20.69
Triacylglycerol (50:3) levels	Parkinson's disease	rs72738698	A	G	A	G	1.04E-01	-3.20E-02	1.79E-01	7.90E-01	FALSE	FALSE	9	82705280	2.76E-02	6.10E-01	TRUE	9	80090365	2.19E-02	0.99	7161	1.95E-06	TRUE	TRUE	482,730	22.68
Triacylglycerol (50:3) levels	Parkinson's disease	rs72753379	T	C	T	C	2.08E-01	2.30E-02	3.92E-02	9.80E-01	FALSE	FALSE	9	123112954	9.08E-02	9.67E-02	TRUE	9	120350676	4.47E-02	0.95	7161	3.22E-06	TRUE	TRUE	482,730	21.72
Triacylglycerol (50:3) levels	Parkinson's disease	rs79771052	G	A	G	A	3.59E-01	5.30E-03	1.20E-02	9.83E-01	FALSE	FALSE	3	72482879	9.20E-02	2.02E-02	TRUE	3	72433728	8.09E-02	0.91	7161	8.91E-06	TRUE	TRUE	482,730	19.76
Triacylglycerol (50:3) levels	Parkinson's disease	rs79934453	G	A	G	A	-6.37E-01	-9.28E-02	4.04E-03	9.90E-01	FALSE	FALSE	6	23919234	1.77E-01	2.22E-01	TRUE	6	23919006	1.37E-01	0.91	7161	3.41E-06	TRUE	TRUE	482,730	21.60
Triacylglycerol (50:3) levels	Parkinson's disease	rs79954170	A	C	A	C	-4.40E-01	-9.06E-02	8.14E-03	9.85E-01	FALSE	FALSE	6	49090758	8.53E-02	5.40E-01	TRUE	6	49123122	9.57E-02	0.96	7161	4.32E-06	TRUE	TRUE	482,730	21.15
Triacylglycerol (50:3) levels	Parkinson's disease	rs820903	G	A	G	A	1.40E-01	-1.46E-02	9.17E-01	1.61E-01	FALSE	FALSE	11	38182666	3.05E-02	1.99E-01	TRUE	11	38161116	3.13E-02	0.95	7161	7.51E-06	TRUE	TRUE	482,730	20.09
Triacylglycerol (50:3) levels	Parkinson's disease	rs9344829	T	C	T	C	8.01E-02	-2.70E-03	3.73E-01	6.88E-01	FALSE	FALSE	6	64887066	1.90E-02	5.16E-02	TRUE	6	64177173	1.72E-02	0.99	7161	3.39E-06	TRUE	TRUE	482,730	21.61
Triacylglycerol (50:3) levels	Parkinson's disease	rs964184	C	G	C	G	-2.13E-01	-5.68E-02	8.49E-01	8.63E-01	TRUE	FALSE	11	116648917	2.43E-02	9.71E-03	TRUE	11	116778201	2.31E-02	1.00	7161	4.05E-20	TRUE	TRUE	482,730	84.89
Triacylglycerol (50:4) levels	Parkinson's disease	rs10105606	A	C	A	C	-9.73E-02	0.00E+00	2.79E-01	6.64E-01	FALSE	FALSE	8	19827848	1.77E-02	4.34E-05	TRUE	8	19970337	1.85E-02	1.00	7166	1.54E-07	TRUE	TRUE	482,730	27.59
Triacylglycerol (50:4) levels	Parkinson's disease	rs1042034	T	C	T	C	9.32E-02	-5.30E-03	7.28E-01	2.18E-01	FALSE	FALSE	2	21225281	2.03E-02	9.96E-02	TRUE	2	21002409	1.86E-02	1.00	7166	5.96E-07	TRUE	TRUE	482,730	24.97
Triacylglycerol (50:4) levels	Parkinson's disease	rs116497138	T	G	T	G	-1.84E-01	5.25E-02	4.98E-02	9.53E-01	FALSE	FALSE	3	87229633	6.09E-02	4.10E-01	TRUE	3	87180483	3.92E-02	0.94	7166	2.68E-06	TRUE	TRUE	482,730	22.06
Triacylglycerol (50:4) levels	Parkinson's disease	rs116635711	A	G	A	G	2.30E-01	1.72E-02	2.79E-02	9.75E-01	FALSE	FALSE	3	151257854	7.13E-02	9.17E-02	TRUE	3	151540066	5.12E-02	0.99	7166	7.33E-06	TRUE	TRUE	482,730	20.13
Triacylglycerol (50:4) levels	Parkinson's disease	rs11783556	G	A	G	A	-9.96E-02	2.30E-02	2.00E-01	7.84E-01	FALSE	FALSE	8	714450	2.74E-02	3.95E-01	TRUE	8	764450	2.11E-02	0.99	7166	2.35E-06	TRUE	TRUE	482,730	22.32
Triacylglycerol (50:4) levels	Parkinson's disease	rs12120691	C	T	C	T	-1.18E-01	3.59E-02	1.23E-01	9.03E-01	FALSE	FALSE	1	227215490	3.12E-02	6.03E-01	TRUE	1	227027789	2.56E-02	0.99	7166	3.71E-06	TRUE	TRUE	482,730	21.44
Triacylglycerol (50:4) levels	Parkinson's disease	rs1260326	C	T	C	T	-1.69E-01	-6.78E-02	6.51E-01	4.20E-01	FALSE	FALSE	2	27730940	1.72E-02	4.04E-05	TRUE	2	27508073	1.74E-02	1.00	7166	3.53E-22	TRUE	TRUE	482,730	94.39
Triacylglycerol (50:4) levels	Parkinson's disease	rs17054866	C	T	C	T	1.16E-01	2.44E-02	1.19E-01	9.09E-01	FALSE	FALSE	5	157201270	3.87E-02	2.77E-01	TRUE	5	157774262	2.58E-02	0.99	7166	6.86E-06	TRUE	TRUE	482,730	20.26
Triacylglycerol (50:4) levels	Parkinson's disease	rs1832326	C	A	C	A	8.56E-02	-1.39E-02	7.25E-01	2.50E-01	FALSE	FALSE	9	33656917	2.18E-02	2.80E-01	TRUE	9	33656919	1.86E-02	1.00	7166	4.51E-06	TRUE	TRUE	482,730	21.07
Triacylglycerol (50:4) levels	Parkinson's disease	rs190411877	C	T	C	T	4.16E-01	5.44E-02	9.45E-03	9.93E-01	FALSE	FALSE	10	7913102	2.09E-01	9.96E-02	TRUE	10	7871139	8.80E-02	0.94	7166	2.38E-06	TRUE	TRUE	482,730	22.29
Triacylglycerol (50:4) levels	Parkinson's disease	rs191616475	G	A	G	A	1.85E-01	2.15E-01	4.81E-02	9.51E-01	FALSE	FALSE	8	59242606	2.39E-01	4.33E-01	TRUE	8	58330047	3.86E-02	1.00	7166	1.61E-06	TRUE	TRUE	482,730	23.05
Triacylglycerol (50:4) levels	Parkinson's disease	rs2249682	A	G	A	G	7.44E-02	-2.00E-02	5.25E-01	4.53E-01	FALSE	FALSE	15	61961353	1.87E-02	5.46E-01	TRUE	15	61669154	1.66E-02	1.00	7166	7.64E-06	TRUE	TRUE	482,730	20.05
Triacylglycerol (50:4) levels	Parkinson's disease	rs72697234	G	A	G	A	2.07E-01	8.75E-02	3.57E-02	9.59E-01	FALSE	FALSE	14	93005264	7.44E-02	6.20E-01	TRUE	14	92538920	4.64E-02	0.93	7166	7.93E-06	TRUE	TRUE	482,730	19.98
Triacylglycerol (50:4) levels	Parkinson's disease	rs72738698	A	G	A	G	1.05E-01	-3.20E-02	1.79E-01	7.90E-01	FALSE	FALSE	9	82705280	2.76E-02	6.10E-01	TRUE	9	80090365	2.19E-02	0.99	7166	1.44E-06	TRUE	TRUE	482,730	23.26
Triacylglycerol (50:4) levels	Parkinson's disease	rs759126	G	A	G	A	-2.14E-01	2.21E-02	9.64E-01	2.73E-02	FALSE	FALSE	9	123046579	7.32E-02	1.18E-01	TRUE	9	120284301	4.70E-02	0.92	7166	5.56E-06	TRUE	TRUE	482,730	20.67
Triacylglycerol (50:4) levels	Parkinson's disease	rs820903	G	A	G	A	1.40E-01	-1.46E-02	9.17E-01	1.61E-01	FALSE	FALSE	11	38182666	3.05E-02	1.99E-01	TRUE	11	38161116	3.13E-02	0.95	7166	7.48E-06	TRUE	TRUE	482,730	20.09
Triacylglycerol (50:4) levels	Parkinson's disease	rs9344829	T	C	T	C	7.70E-02	-2.70E-03	3.73E-01	6.88E-01	FALSE	FALSE	6	64887066	1.90E-02	5.16E-02	TRUE	6	64177173	1.72E-02	0.99	7166	7.85E-06	TRUE	TRUE	482,730	20.00
Triacylglycerol (50:4) levels	Parkinson's disease	rs964184	C	G	C	G	-2.03E-01	-5.68E-02	8.49E-01	8.63E-01	TRUE	FALSE	11	116648917	2.43E-02	9.71E-03	TRUE	11	116778201	2.31E-02	1.00	7166	2.01E-18	TRUE	TRUE	482,730	77.09
Triacylglycerol (50:5) levels	Parkinson's disease	rs11208199	A	G	A	G	-9.72E-02	-1.15E-02	2.67E-01	7.09E-01	FALSE	FALSE	1	63842972	2.20E-02	2.20E-01	TRUE	1	63377301	2.19E-02	0.97	5491	9.03E-06	TRUE	TRUE	482,730	19.74
Triacylglycerol (50:5) levels	Parkinson's disease	rs112578388	T	C	T	C	-6.72E-01	-2.36E-02	5.33E-03	9.88E-01	FALSE	FALSE	13	26460581	2.04E-01	4.20E-02	TRUE	13	25886443	1.48E-01	0.86	5491	5.82E-06	TRUE	TRUE	482,730	20.58
Triacylglycerol (50:5) levels	Parkinson's disease	rs11257904	T	C	T	C	5.73E-01	-1.44E-01	6.25E-03	9.72E-01	FALSE	FALSE	10	12624084	6.22E-02	1.03E-02	TRUE	10	12582085	1.26E-01	0.97	5491	5.45E-06	TRUE	TRUE	482,730	20.71
Triacylglycerol (50:5) levels	Parkinson's disease	rs115914883	T	G	T	G	3.04E-01	-6.22E-02	2.16E-02	9.87E-01	FALSE	FALSE	4	28036187	1.04E-01	2.60E-01	TRUE	4	28034565	6.79E-02	0.96	5491	7.61E-06	TRUE	TRUE	482,730	20.07
Triacylglycerol (50:5) levels	Parkinson's disease	rs116876951	A	G	A	G	4.95E-01	4.73E-02	7.67E-03	9.52E-01	FALSE	FALSE	16	27136935	5.36E-02	4.23E-01	TRUE	16	27125614	1.10E-01	0.99	5491	7.37E-06	TRUE	TRUE	482,730	20.13
Triacylglycerol (50:5) levels	Parkinson's disease	rs117906663	A	G	A	G	-6.10E-01	1.57E-02	5.81E-03	9.77E-01	FALSE	FALSE	8	3463002	8.99E-02	6.47E-02	TRUE	8	3605480	1.37E-01	0.89	5491	8.78E-06	TRUE	TRUE	482,730	19.80
Triacylglycerol (50:5) levels	Parkinson's disease	rs1260326	C	T	C	T	-1.57E-01	-6.78E-02	6.51E-01	4.20E-01	FALSE	FALSE	2	27730940	1.72E-02	4.04E-05	TRUE	2	27508073	1.98E-02	1.00	5491	2.77E-15	TRUE	TRUE	482,730	62.78
Triacylglycerol (50:5) levels	Parkinson's disease	rs143248091	G	C	G	C	-8.37E-01	2.48E-01	2.68E-03	1.21E-02	TRUE	FALSE	8	9677749	1.56E-01	9.47E-01	TRUE	8	9820239	1.84E-01	0.99	5491	5.73E-06	TRUE	TRUE	482,730	20.61
Triacylglycerol (50:5) levels	Parkinson's disease	rs150889723	C	T	C	T	-2.22E-01	-1.37E-02	5.05E-02	9.52E-01	FALSE	FALSE	11	21762505	5.89E-02	8.85E-02	TRUE	11	21740959	4.50E-02	0.93	5491	7.81E-07	TRUE	TRUE	482,730	24.46
Triacylglycerol (50:5) levels	Parkinson's disease	rs181240184	T	C	T	C	-3.05E-01	8.43E-02	2.12E-02	9.86E-01	FALSE	FALSE	11	77740984	9.95E-02	4.01E-01	TRUE	11	78029938	6.79E-02	0.97	5491	7.15E-06	TRUE	TRUE	482,730	20.19
Triacylglycerol (50:5) levels	Parkinson's disease	rs187087759	G	C	G	C	-4.22E-01	-2.83E-02	1.23E-02	1.19E-02	TRUE	FALSE	5	176302667	1.31E-01	8.19E-02	TRUE	5	176875666	9.20E-02	0.86	5491	4.65E-06	TRUE	TRUE	482,730	21.02
Triacylglycerol (50:5) levels	Parkinson's disease	rs2278983	A	C	A	C	8.85E-02	1.73E-02	5.36E-01	5.34E-01	FALSE	FALSE	16	70726795	2.33E-02	3.38E-01	TRUE	16	70692892	1.93E-02	0.98	5491	4.76E-06	TRUE	TRUE	482,730	20.97
Triacylglycerol (50:5) levels	Parkinson's disease	rs254668	A	G	A	G	1.01E-01	-4.50E-03	4.42E-01	4.82E-01	FALSE	FALSE	5	157272463	2.13E-02	8.01E-02	TRUE	5	157845455	1.91E-02	1.00	5491	1.28E-07	TRUE	TRUE	482,730	27.96
Triacylglycerol (50:5) levels	Parkinson's disease	rs2954038	A	C	A	C	-9.96E-02	-2.37E-02	7.43E-01	3.16E-01	FALSE	FALSE	8	126507389	1.82E-02	7.18E-01	TRUE	8	125495147	2.18E-02	1.00	5491	5.22E-06	TRUE	TRUE	482,730	20.80
Triacylglycerol (50:5) levels	Parkinson's disease	rs3767910	C	T	C	T	1.05E-01	-3.00E-04	2.11E-01	7.86E-01	FALSE	FALSE	1	25967562	2.73E-02	3.66E-03	TRUE	1	25641071	2.36E-02	0.99	5491	9.28E-06	TRUE	TRUE	482,730	19.69
Triacylglycerol (50:5) levels	Parkinson's disease	rs3906419	T	A	T	A	1.08E-01	8.80E-03	2.20E-01	2.10E-01	TRUE	FALSE	5	108287614	2.75E-02	1.26E-01	TRUE	5	108951913	2.29E-02	1.00	5491	2.28E-06	TRUE	TRUE	482,730	22.39
Triacylglycerol (50:5) levels	Parkinson's disease	rs67080320	C	T	C	T	-2.03E-01	-8.00E-04	5.08E-02	9.38E-01	FALSE	FALSE	8	140806696	4.67E-02	6.04E-03	TRUE	8	139794453	4.36E-02	1.00	5491	3.25E-06	TRUE	TRUE	482,730	21.71
Triacylglycerol (50:5) levels	Parkinson's disease	rs78571096	C	G	C	G	5.26E-01	-6.75E-02	7.15E-03	2.01E-02	TRUE	FALSE	7	71242924	8.89E-02	3.49E-01	TRUE	7	71777939	1.18E-01	0.87	5491	7.87E-06	TRUE	TRUE	482,730	20.00
Triacylglycerol (50:5) levels	Parkinson's disease	rs964184	C	G	C	G	-1.84E-01	-5.68E-02	8.49E-01	8.63E-01	TRUE	FALSE	11	116648917	2.43E-02	9.71E-03	TRUE	11	116778201	2.58E-02	1.00	5491	1.16E-12	TRUE	TRUE	482,730	50.78
Triacylglycerol (51:1) levels	Parkinson's disease	rs11102221	A	G	A	G	-1.10E-01	4.16E-02	1.98E-01	7.47E-01	FALSE	FALSE	1	111784816	1.96E-02	1.69E-02	TRUE	1	111242194	2.38E-02	1.00	5495	3.73E-06	TRUE	TRUE	482,730	21.44
Triacylglycerol (51:1) levels	Parkinson's disease	rs111568723	G	A	G	A	3.38E-01	-1.20E-01	2.59E-02	9.47E-01	FALSE	FALSE	9	135345889	5.14E-02	9.73E-03	TRUE	9	132470502	6.21E-02	0.95	5495	5.37E-08	TRUE	TRUE	482,730	29.65
Triacylglycerol (51:1) levels	Parkinson's disease	rs1260326	C	T	C	T	-1.02E-01	-6.78E-02	6.51E-01	4.20E-01	FALSE	FALSE	2	27730940	1.72E-02	4.04E-05	TRUE	2	27508073	1.99E-02	1.00	5495	2.96E-07	TRUE	TRUE	482,730	26.34
Triacylglycerol (51:1) levels	Parkinson's disease	rs12613782	C	T	C	T	2.14E-01	-2.63E-02	4.16E-02	9.75E-01	FALSE	FALSE	2	49266854	7.97E-02	1.30E-01	TRUE	2	49039715	4.84E-02	1.00	5495	9.64E-06	TRUE	TRUE	482,730	19.62
Triacylglycerol (51:1) levels	Parkinson's disease	rs140828820	G	A	G	A	4.51E-01	1.72E-01	1.06E-02	9.91E-01	FALSE	FALSE	4	34769093	2.99E-01	2.48E-01	TRUE	4	34767471	9.52E-02	0.93	5495	2.18E-06	TRUE	TRUE	482,730	22.48
Triacylglycerol (51:1) levels	Parkinson's disease	rs143186626	T	C	T	C	-1.73E-01	3.85E-02	7.01E-02	9.57E-01	FALSE	FALSE	11	95983462	5.77E-02	2.97E-01	TRUE	11	96250298	3.84E-02	0.97	5495	6.47E-06	TRUE	TRUE	482,730	20.38
Triacylglycerol (51:1) levels	Parkinson's disease	rs146238333	T	A	T	A	6.21E-01	5.84E-02	5.21E-03	1.67E-02	TRUE	FALSE	12	84101712	9.77E-02	2.60E-01	TRUE	12	83707933	1.34E-01	0.89	5495	3.60E-06	TRUE	TRUE	482,730	21.51
Triacylglycerol (51:1) levels	Parkinson's disease	rs1928423	C	A	C	A	-1.02E-01	-9.60E-03	6.47E-01	4.08E-01	FALSE	FALSE	9	74250387	2.74E-02	1.39E-01	TRUE	9	71635471	2.06E-02	0.95	5495	8.56E-07	TRUE	TRUE	482,730	24.28
Triacylglycerol (51:1) levels	Parkinson's disease	rs2169099	C	T	C	T	-1.17E-01	-1.21E-02	1.99E-01	8.10E-01	FALSE	FALSE	4	63226393	2.85E-02	1.73E-01	TRUE	4	62360675	2.41E-02	0.99	5495	1.21E-06	TRUE	TRUE	482,730	23.60
Triacylglycerol (51:1) levels	Parkinson's disease	rs2242104	G	A	G	A	-1.01E-01	-6.60E-03	7.52E-01	3.76E-01	FALSE	FALSE	9	2640492	2.32E-02	1.09E-01	TRUE	9	2640492	2.21E-02	0.99	5495	5.25E-06	TRUE	TRUE	482,730	20.78
Triacylglycerol (51:1) levels	Parkinson's disease	rs34496841	A	G	A	G	-1.12E-01	-6.99E-02	2.37E-01	8.42E-01	FALSE	FALSE	17	49516568	3.20E-02	1.45E-02	TRUE	17	51439207	2.24E-02	0.99	5495	5.15E-07	TRUE	TRUE	482,730	25.27
Triacylglycerol (51:1) levels	Parkinson's disease	rs4448624	A	G	A	G	-8.77E-02	2.16E-02	4.70E-01	5.59E-01	FALSE	FALSE	10	87739205	2.28E-02	4.65E-01	TRUE	10	85979448	1.92E-02	0.98	5495	4.82E-06	TRUE	TRUE	482,730	20.95
Triacylglycerol (51:1) levels	Parkinson's disease	rs4979284	A	G	A	G	-9.67E-02	-2.67E-02	6.77E-01	2.54E-01	FALSE	FALSE	9	116551303	2.19E-02	6.50E-01	TRUE	9	113789023	2.04E-02	0.99	5495	2.13E-06	TRUE	TRUE	482,730	22.52
Triacylglycerol (51:1) levels	Parkinson's disease	rs67093838	G	A	G	A	-2.51E-01	-9.00E-03	3.00E-02	9.43E-01	FALSE	FALSE	2	62350070	4.94E-02	6.74E-02	TRUE	2	62122935	5.68E-02	0.97	5495	9.88E-06	TRUE	TRUE	482,730	19.57
Triacylglycerol (51:1) levels	Parkinson's disease	rs7076657	G	C	G	C	1.80E-01	7.30E-03	6.51E-02	4.80E-02	TRUE	FALSE	10	134645860	5.99E-02	4.40E-02	TRUE	10	132832356	3.90E-02	0.97	5495	4.21E-06	TRUE	TRUE	482,730	21.21
Triacylglycerol (51:1) levels	Parkinson's disease	rs71556711	T	C	T	C	-1.47E-01	1.25E-02	9.53E-02	9.15E-01	FALSE	FALSE	7	72854549	3.50E-02	1.42E-01	TRUE	7	73440219	3.26E-02	0.99	5495	6.93E-06	TRUE	TRUE	482,730	20.25
Triacylglycerol (51:1) levels	Parkinson's disease	rs72801474	A	G	A	G	-1.97E-01	-1.22E-02	5.08E-02	9.13E-01	FALSE	FALSE	5	132444128	4.14E-02	1.14E-01	TRUE	5	133108436	4.42E-02	0.96	5495	8.77E-06	TRUE	TRUE	482,730	19.80
Triacylglycerol (51:1) levels	Parkinson's disease	rs832231	G	A	G	A	-8.85E-02	4.35E-02	5.99E-01	3.48E-01	FALSE	FALSE	9	33414020	2.79E-02	9.26E-01	TRUE	9	33414022	2.00E-02	0.96	5495	9.78E-06	TRUE	TRUE	482,730	19.59
Triacylglycerol (51:1) levels	Parkinson's disease	rs964184	C	G	C	G	-1.40E-01	-5.68E-02	8.49E-01	8.63E-01	TRUE	FALSE	11	116648917	2.43E-02	9.71E-03	TRUE	11	116778201	2.62E-02	1.00	5495	9.62E-08	TRUE	TRUE	482,730	28.52
Triacylglycerol (51:2) levels	Parkinson's disease	rs10147474	G	A	G	A	-2.27E-01	3.15E-02	3.72E-02	9.66E-01	FALSE	FALSE	14	30292648	6.22E-02	2.13E-01	TRUE	14	29823442	4.47E-02	0.98	7149	3.98E-07	TRUE	TRUE	482,730	25.75
Triacylglycerol (51:2) levels	Parkinson's disease	rs112122274	G	A	G	A	-2.21E-01	4.76E-02	3.60E-02	9.49E-01	FALSE	FALSE	9	139149840	6.87E-02	3.11E-01	TRUE	9	136257994	4.87E-02	0.85	7149	5.64E-06	TRUE	TRUE	482,730	20.64
Triacylglycerol (51:2) levels	Parkinson's disease	rs1260326	C	T	C	T	-1.28E-01	-6.78E-02	6.51E-01	4.20E-01	FALSE	FALSE	2	27730940	1.72E-02	4.04E-05	TRUE	2	27508073	1.74E-02	1.00	7149	2.46E-13	TRUE	TRUE	482,730	53.80
Triacylglycerol (51:2) levels	Parkinson's disease	rs139499638	C	G	C	G	-6.17E-01	7.96E-02	4.41E-03	2.11E-02	TRUE	FALSE	4	121751160	8.87E-02	4.32E-01	TRUE	4	120830005	1.36E-01	0.92	7149	6.00E-06	TRUE	TRUE	482,730	20.52
Triacylglycerol (51:2) levels	Parkinson's disease	rs143838492	A	G	A	G	6.81E-01	1.82E-01	3.33E-03	9.90E-01	FALSE	FALSE	5	114228685	1.54E-01	6.27E-01	TRUE	5	114892988	1.48E-01	0.97	7149	4.53E-06	TRUE	TRUE	482,730	21.06
Triacylglycerol (51:2) levels	Parkinson's disease	rs144241030	T	C	T	C	1.47E-01	3.37E-02	7.72E-02	9.57E-01	FALSE	FALSE	1	103708723	6.61E-02	2.14E-01	TRUE	1	103243167	3.22E-02	0.94	7149	5.05E-06	TRUE	TRUE	482,730	20.85
Triacylglycerol (51:2) levels	Parkinson's disease	rs17282355	T	C	T	C	-1.16E-01	-4.53E-02	1.19E-01	8.67E-01	FALSE	FALSE	3	134696331	3.36E-02	7.51E-01	TRUE	3	134977489	2.64E-02	0.96	7149	1.00E-05	TRUE	TRUE	482,730	19.54
Triacylglycerol (51:2) levels	Parkinson's disease	rs174556	T	C	T	C	8.45E-02	1.00E-04	3.80E-01	7.09E-01	FALSE	FALSE	11	61580635	1.85E-02	1.04E-03	TRUE	11	61813163	1.73E-02	1.00	7149	1.08E-06	TRUE	TRUE	482,730	23.81
Triacylglycerol (51:2) levels	Parkinson's disease	rs28368829	A	G	A	G	-8.84E-02	3.90E-03	2.40E-01	6.82E-01	FALSE	FALSE	9	138893579	2.42E-02	6.02E-02	TRUE	9	136001733	1.98E-02	0.99	7149	8.07E-06	TRUE	TRUE	482,730	19.95
Triacylglycerol (51:2) levels	Parkinson's disease	rs28437809	G	T	G	T	7.47E-02	-1.42E-02	5.16E-01	4.77E-01	FALSE	FALSE	4	183422600	2.24E-02	2.80E-01	TRUE	4	182501447	1.68E-02	0.99	7149	8.29E-06	TRUE	TRUE	482,730	19.90
Triacylglycerol (51:2) levels	Parkinson's disease	rs316343	T	C	T	C	-7.58E-02	9.90E-03	4.68E-01	4.54E-01	FALSE	FALSE	6	2840059	2.28E-02	1.77E-01	TRUE	6	2839825	1.71E-02	0.98	7149	9.37E-06	TRUE	TRUE	482,730	19.66
Triacylglycerol (51:2) levels	Parkinson's disease	rs33951980	T	C	T	C	-1.36E-01	-6.00E-03	1.22E-01	8.79E-01	FALSE	FALSE	7	73029437	2.71E-02	8.41E-02	TRUE	7	73615107	2.55E-02	1.00	7149	9.09E-08	TRUE	TRUE	482,730	28.62
Triacylglycerol (51:2) levels	Parkinson's disease	rs4266911	C	T	C	T	1.60E-01	-3.37E-02	9.41E-01	4.21E-02	FALSE	FALSE	1	27426796	5.78E-02	2.52E-01	TRUE	1	27100305	3.62E-02	0.94	7149	9.76E-06	TRUE	TRUE	482,730	19.58
Triacylglycerol (51:2) levels	Parkinson's disease	rs62043594	A	C	A	C	-7.96E-02	-1.90E-02	4.18E-01	6.26E-01	FALSE	FALSE	15	93697383	2.31E-02	3.86E-01	TRUE	15	93154154	1.68E-02	1.00	7149	2.16E-06	TRUE	TRUE	482,730	22.48
Triacylglycerol (51:2) levels	Parkinson's disease	rs6721083	C	G	C	G	1.61E-01	1.00E-02	9.37E-01	9.34E-01	TRUE	FALSE	2	171081652	4.72E-02	7.96E-02	TRUE	2	170225142	3.50E-02	0.95	7149	4.43E-06	TRUE	TRUE	482,730	21.10
Triacylglycerol (51:2) levels	Parkinson's disease	rs72753379	T	C	T	C	2.07E-01	2.30E-02	3.92E-02	9.80E-01	FALSE	FALSE	9	123112954	9.08E-02	9.67E-02	TRUE	9	120350676	4.48E-02	0.95	7149	3.94E-06	TRUE	TRUE	482,730	21.32
Triacylglycerol (51:2) levels	Parkinson's disease	rs75573052	T	C	T	C	-1.35E-01	3.91E-02	8.45E-02	8.89E-01	FALSE	FALSE	13	21612149	3.60E-02	5.57E-01	TRUE	13	21038010	3.05E-02	0.99	7149	9.70E-06	TRUE	TRUE	482,730	19.60
Triacylglycerol (51:2) levels	Parkinson's disease	rs76950187	G	A	G	A	1.63E-01	6.08E-02	7.45E-02	9.52E-01	FALSE	FALSE	2	129184649	5.53E-02	5.65E-01	TRUE	2	128427075	3.21E-02	0.99	7149	3.65E-07	TRUE	TRUE	482,730	25.92
Triacylglycerol (51:2) levels	Parkinson's disease	rs964184	C	G	C	G	-2.12E-01	-5.68E-02	8.49E-01	8.63E-01	TRUE	FALSE	11	116648917	2.43E-02	9.71E-03	TRUE	11	116778201	2.31E-02	1.00	7149	5.60E-20	TRUE	TRUE	482,730	84.25
Triacylglycerol (51:3) levels	Parkinson's disease	rs10147474	G	A	G	A	-2.05E-01	3.15E-02	3.72E-02	9.66E-01	FALSE	FALSE	14	30292648	6.22E-02	2.13E-01	TRUE	14	29823442	4.49E-02	0.98	7119	4.99E-06	TRUE	TRUE	482,730	20.87
Triacylglycerol (51:3) levels	Parkinson's disease	rs1042034	T	C	T	C	1.07E-01	-5.30E-03	7.28E-01	2.18E-01	FALSE	FALSE	2	21225281	2.03E-02	9.96E-02	TRUE	2	21002409	1.87E-02	1.00	7119	1.01E-08	TRUE	TRUE	482,730	32.89
Triacylglycerol (51:3) levels	Parkinson's disease	rs1047974	A	G	A	G	1.43E-01	-5.42E-02	7.60E-02	9.05E-01	FALSE	FALSE	12	108914596	3.12E-02	4.12E-02	TRUE	12	108520819	3.17E-02	1.00	7119	6.08E-06	TRUE	TRUE	482,730	20.49
Triacylglycerol (51:3) levels	Parkinson's disease	rs111526079	A	G	A	G	1.25E-01	5.76E-02	1.05E-01	9.31E-01	FALSE	FALSE	14	77437466	4.73E-02	6.52E-01	TRUE	14	76971123	2.74E-02	0.96	7119	5.14E-06	TRUE	TRUE	482,730	20.82
Triacylglycerol (51:3) levels	Parkinson's disease	rs111568723	G	A	G	A	2.42E-01	-1.20E-01	2.59E-02	9.47E-01	FALSE	FALSE	9	135345889	5.14E-02	9.73E-03	TRUE	9	132470502	5.47E-02	0.95	7119	9.58E-06	TRUE	TRUE	482,730	19.62
Triacylglycerol (51:3) levels	Parkinson's disease	rs116011373	A	G	A	G	-1.91E-01	-2.23E-02	4.88E-02	9.73E-01	FALSE	FALSE	3	87347791	8.48E-02	1.01E-01	TRUE	3	87298641	3.94E-02	0.96	7119	1.29E-06	TRUE	TRUE	482,730	23.47
Triacylglycerol (51:3) levels	Parkinson's disease	rs11728165	T	C	T	C	-1.48E-01	-4.76E-02	8.04E-02	8.98E-01	FALSE	FALSE	4	108808018	3.92E-02	6.47E-01	TRUE	4	107886862	3.22E-02	0.93	7119	4.08E-06	TRUE	TRUE	482,730	21.26
Triacylglycerol (51:3) levels	Parkinson's disease	rs117428564	T	C	T	C	4.66E-01	1.11E-01	7.25E-03	9.82E-01	FALSE	FALSE	12	21113599	9.08E-02	6.51E-01	TRUE	12	20960665	1.02E-01	0.97	7119	5.38E-06	TRUE	TRUE	482,730	20.73
Triacylglycerol (51:3) levels	Parkinson's disease	rs1260326	C	T	C	T	-1.38E-01	-6.78E-02	6.51E-01	4.20E-01	FALSE	FALSE	2	27730940	1.72E-02	4.04E-05	TRUE	2	27508073	1.75E-02	1.00	7119	3.28E-15	TRUE	TRUE	482,730	62.36
Triacylglycerol (51:3) levels	Parkinson's disease	rs12635725	G	A	G	A	7.53E-02	2.07E-02	5.60E-01	4.47E-01	FALSE	FALSE	3	1937149	2.20E-02	4.61E-01	TRUE	3	1895465	1.68E-02	0.99	7119	7.13E-06	TRUE	TRUE	482,730	20.19
Triacylglycerol (51:3) levels	Parkinson's disease	rs138427786	G	A	G	A	3.02E-01	-3.00E-04	1.62E-02	9.74E-01	FALSE	FALSE	4	164473300	7.15E-02	1.61E-03	TRUE	4	163552148	6.65E-02	0.97	7119	5.76E-06	TRUE	TRUE	482,730	20.60
Triacylglycerol (51:3) levels	Parkinson's disease	rs139278484	C	A	C	A	2.52E-01	4.45E-02	2.19E-02	9.83E-01	FALSE	FALSE	3	176741646	9.12E-02	2.04E-01	TRUE	3	177023858	5.63E-02	1.00	7119	7.54E-06	TRUE	TRUE	482,730	20.08
Triacylglycerol (51:3) levels	Parkinson's disease	rs139499638	C	G	C	G	-6.59E-01	7.96E-02	4.41E-03	2.11E-02	TRUE	FALSE	4	121751160	8.87E-02	4.32E-01	TRUE	4	120830005	1.36E-01	0.92	7119	1.28E-06	TRUE	TRUE	482,730	23.50
Triacylglycerol (51:3) levels	Parkinson's disease	rs139500046	C	A	C	A	-3.73E-01	-4.41E-02	1.12E-02	9.83E-01	FALSE	FALSE	12	97244448	8.65E-02	2.15E-01	TRUE	12	96850670	7.99E-02	0.99	7119	3.04E-06	TRUE	TRUE	482,730	21.83
Triacylglycerol (51:3) levels	Parkinson's disease	rs146131769	G	A	G	A	-4.48E-01	1.01E-02	8.35E-03	9.89E-01	FALSE	FALSE	6	49461707	9.17E-02	3.97E-02	TRUE	6	49493994	9.52E-02	0.97	7119	2.58E-06	TRUE	TRUE	482,730	22.14
Triacylglycerol (51:3) levels	Parkinson's disease	rs35332062	A	G	A	G	-1.51E-01	-4.60E-03	1.22E-01	8.81E-01	FALSE	FALSE	7	73012042	2.72E-02	6.20E-02	TRUE	7	73597712	2.55E-02	1.00	7119	3.41E-09	TRUE	TRUE	482,730	35.02
Triacylglycerol (51:3) levels	Parkinson's disease	rs35397574	G	A	G	A	-7.99E-02	-4.00E-03	4.83E-01	5.33E-01	FALSE	FALSE	3	148113373	2.36E-02	6.26E-02	TRUE	3	148395586	1.70E-02	0.98	7119	2.72E-06	TRUE	TRUE	482,730	22.04
Triacylglycerol (51:3) levels	Parkinson's disease	rs6692319	T	C	T	C	-8.82E-02	-1.94E-02	7.40E-01	1.99E-01	FALSE	FALSE	1	230284375	2.53E-02	3.53E-01	TRUE	1	230148628	1.89E-02	1.00	7119	2.93E-06	TRUE	TRUE	482,730	21.90
Triacylglycerol (51:3) levels	Parkinson's disease	rs7782961	T	A	T	A	8.98E-02	5.86E-02	2.32E-01	1.82E-01	TRUE	FALSE	7	6335583	2.64E-02	1.32E-02	TRUE	7	6295952	2.01E-02	0.97	7119	7.66E-06	TRUE	TRUE	482,730	20.05
Triacylglycerol (51:3) levels	Parkinson's disease	rs7846649	C	T	C	T	9.49E-02	6.90E-02	1.91E-01	9.09E-01	FALSE	FALSE	8	60001055	3.38E-02	2.06E-02	TRUE	8	59088496	2.13E-02	0.99	7119	8.14E-06	TRUE	TRUE	482,730	19.93
Triacylglycerol (51:3) levels	Parkinson's disease	rs964184	C	G	C	G	-2.49E-01	-5.68E-02	8.49E-01	8.63E-01	TRUE	FALSE	11	116648917	2.43E-02	9.71E-03	TRUE	11	116778201	2.31E-02	1.00	7119	5.67E-27	TRUE	TRUE	482,730	116.59
Triacylglycerol (51:4) levels	Parkinson's disease	rs112421492	T	A	T	A	-9.71E-01	1.03E-01	2.36E-03	6.80E-03	TRUE	FALSE	13	24612849	2.31E-01	1.83E-01	TRUE	13	24038710	1.97E-01	0.92	5237	8.22E-07	TRUE	TRUE	482,730	24.36
Triacylglycerol (51:4) levels	Parkinson's disease	rs116923150	A	G	A	G	9.55E-01	1.63E-01	3.23E-03	9.84E-01	FALSE	FALSE	9	114876489	1.55E-01	5.32E-01	TRUE	9	112114209	1.92E-01	0.90	5237	6.84E-07	TRUE	TRUE	482,730	24.72
Triacylglycerol (51:4) levels	Parkinson's disease	rs11782705	C	T	C	T	-2.00E-01	-1.94E-02	5.90E-02	9.11E-01	FALSE	FALSE	8	4490943	4.15E-02	1.94E-01	TRUE	8	4633421	4.21E-02	0.98	5237	2.02E-06	TRUE	TRUE	482,730	22.63
Triacylglycerol (51:4) levels	Parkinson's disease	rs1260326	C	T	C	T	-1.35E-01	-6.78E-02	6.51E-01	4.20E-01	FALSE	FALSE	2	27730940	1.72E-02	4.04E-05	TRUE	2	27508073	2.03E-02	1.00	5237	3.13E-11	TRUE	TRUE	482,730	44.28
Triacylglycerol (51:4) levels	Parkinson's disease	rs149992851	G	A	G	A	3.38E-01	-1.26E-01	1.98E-02	9.87E-01	FALSE	FALSE	3	55303104	1.02E-01	6.65E-01	TRUE	3	55269076	6.95E-02	0.98	5237	1.17E-06	TRUE	TRUE	482,730	23.67
Triacylglycerol (51:4) levels	Parkinson's disease	rs17514333	G	A	G	A	1.10E-01	8.50E-03	2.09E-01	8.20E-01	FALSE	FALSE	13	106591917	2.93E-02	1.12E-01	TRUE	13	105939568	2.38E-02	1.00	5237	3.41E-06	TRUE	TRUE	482,730	21.62
Triacylglycerol (51:4) levels	Parkinson's disease	rs182295482	C	T	C	T	-5.47E-01	2.97E-01	7.85E-03	9.88E-01	FALSE	FALSE	10	72951464	1.35E-01	1.42E-02	TRUE	10	71191707	1.19E-01	0.86	5237	4.54E-06	TRUE	TRUE	482,730	21.07
Triacylglycerol (51:4) levels	Parkinson's disease	rs2151252	C	G	C	G	1.01E-01	-2.39E-02	2.50E-01	2.25E-01	TRUE	FALSE	1	170351200	2.61E-02	4.42E-01	TRUE	1	170382059	2.24E-02	1.00	5237	7.12E-06	TRUE	TRUE	482,730	20.20
Triacylglycerol (51:4) levels	Parkinson's disease	rs4709886	C	A	C	A	1.08E-01	1.83E-02	7.94E-01	2.82E-01	FALSE	FALSE	6	165143420	2.18E-02	3.97E-01	TRUE	6	164722387	2.41E-02	1.00	5237	7.67E-06	TRUE	TRUE	482,730	20.06
Triacylglycerol (51:4) levels	Parkinson's disease	rs62002515	C	T	C	T	-3.28E-01	6.25E-02	1.93E-02	9.31E-01	FALSE	FALSE	15	59296838	4.02E-02	9.22E-01	TRUE	15	59004639	7.06E-02	0.99	5237	3.46E-06	TRUE	TRUE	482,730	21.59

Triacylglycerol (51:4) levels	Parkinson's disease	rs62291572	T	C	T	C	1.19E-01	-2.71E-02	2.14E-01	7.47E-01	FALSE	FALSE	4	4997927	2.75E-02	4.89E-01	TRUE	4	4996200	2.44E-02	0.94	5237	1.05E-06	TRUE	TRUE	482,730	23.89
Triacylglycerol (51:4) levels	Parkinson's disease	rs7112577	G	C	G	C	-1.90E-01	4.66E-02	5.84E-02	4.36E-02	TRUE	FALSE	11	117044603	4.75E-02	4.86E-01	TRUE	11	117173887	4.22E-02	1.00	5237	7.28E-06	TRUE	TRUE	482,730	20.16
Triacylglycerol (51:4) levels	Parkinson's disease	rs71605604	T	C	T	C	2.62E-01	1.12E-02	3.01E-02	9.59E-01	FALSE	FALSE	4	87457201	4.87E-02	8.74E-02	TRUE	4	86536048	5.68E-02	0.97	5237	4.16E-06	TRUE	TRUE	482,730	21.23
Triacylglycerol (51:4) levels	Parkinson's disease	rs76308170	C	T	C	T	1.63E-01	9.24E-02	7.75E-02	8.99E-01	FALSE	FALSE	3	187987160	3.84E-02	8.06E-03	TRUE	3	188269372	3.61E-02	0.97	5237	6.45E-06	TRUE	TRUE	482,730	20.39
Triacylglycerol (51:4) levels	Parkinson's disease	rs964184	C	G	C	G	-2.40E-01	-5.68E-02	8.49E-01	8.63E-01	TRUE	FALSE	11	116648917	2.43E-02	9.71E-03	TRUE	11	116778201	2.62E-02	1.00	5237	6.65E-20	TRUE	TRUE	482,730	84.08
Triacylglycerol (51:4) levels	Parkinson's disease	rs966039	G	A	G	A	-1.27E-01	-3.60E-02	1.64E-01	7.63E-01	FALSE	FALSE	3	66829256	2.69E-02	7.42E-01	TRUE	3	66778832	2.67E-02	0.98	5237	2.09E-06	TRUE	TRUE	482,730	22.56
Triacylglycerol (52:2) levels	Parkinson's disease	rs1010313	T	C	T	C	9.65E-02	2.37E-02	1.98E-01	8.77E-01	FALSE	FALSE	8	60002542	2.73E-02	4.15E-01	TRUE	8	59089983	2.08E-02	1.00	7168	3.42E-06	TRUE	TRUE	482,730	21.60
Triacylglycerol (52:2) levels	Parkinson's disease	rs10147474	G	A	G	A	-2.16E-01	3.15E-02	3.72E-02	9.66E-01	FALSE	FALSE	14	30292648	6.22E-02	2.13E-01	TRUE	14	29823442	4.47E-02	0.98	7168	1.32E-06	TRUE	TRUE	482,730	23.44
Triacylglycerol (52:2) levels	Parkinson's disease	rs10169217	G	A	G	A	-8.41E-02	-2.56E-02	6.76E-01	3.13E-01	FALSE	FALSE	2	212875346	2.37E-02	5.51E-01	TRUE	2	212010621	1.79E-02	1.00	7168	2.59E-06	TRUE	TRUE	482,730	22.13
Triacylglycerol (52:2) levels	Parkinson's disease	rs1042034	T	C	T	C	9.03E-02	-5.30E-03	7.28E-01	2.18E-01	FALSE	FALSE	2	21225281	2.03E-02	9.96E-02	TRUE	2	21002409	1.86E-02	1.00	7168	1.28E-06	TRUE	TRUE	482,730	23.49
Triacylglycerol (52:2) levels	Parkinson's disease	rs11017845	C	T	C	T	-1.76E-01	-3.36E-02	4.73E-02	9.53E-01	FALSE	FALSE	10	133052453	5.37E-02	2.75E-01	TRUE	10	131254190	3.96E-02	0.99	7168	9.65E-06	TRUE	TRUE	482,730	19.61
Triacylglycerol (52:2) levels	Parkinson's disease	rs112122274	G	A	G	A	-2.39E-01	4.76E-02	3.60E-02	9.49E-01	FALSE	FALSE	9	139149840	6.87E-02	3.11E-01	TRUE	9	136257994	4.85E-02	0.85	7168	8.47E-07	TRUE	TRUE	482,730	24.29
Triacylglycerol (52:2) levels	Parkinson's disease	rs1260326	C	T	C	T	-1.22E-01	-6.78E-02	6.51E-01	4.20E-01	FALSE	FALSE	2	27730940	1.72E-02	4.04E-05	TRUE	2	27508073	1.74E-02	1.00	7168	2.79E-12	TRUE	TRUE	482,730	49.00
Triacylglycerol (52:2) levels	Parkinson's disease	rs144241030	T	C	T	C	1.50E-01	3.37E-02	7.72E-02	9.57E-01	FALSE	FALSE	1	103708723	6.61E-02	2.14E-01	TRUE	1	103243167	3.22E-02	0.94	7168	3.26E-06	TRUE	TRUE	482,730	21.69
Triacylglycerol (52:2) levels	Parkinson's disease	rs147987331	A	T	A	T	-2.61E-01	-3.21E-02	2.27E-02	2.36E-02	TRUE	FALSE	2	104861307	7.92E-02	1.64E-01	TRUE	2	104244849	5.65E-02	0.97	7168	3.96E-06	TRUE	TRUE	482,730	21.32
Triacylglycerol (52:2) levels	Parkinson's disease	rs150741556	A	G	A	G	2.38E-01	1.04E-02	2.62E-02	9.88E-01	FALSE	FALSE	5	83102973	1.11E-01	3.35E-02	TRUE	5	83807154	5.23E-02	0.98	7168	5.63E-06	TRUE	TRUE	482,730	20.64
Triacylglycerol (52:2) levels	Parkinson's disease	rs2980888	C	T	C	T	-8.70E-02	-2.33E-02	7.43E-01	3.16E-01	FALSE	FALSE	8	126507308	1.82E-02	7.01E-01	TRUE	8	125495066	1.91E-02	1.00	7168	5.32E-06	TRUE	TRUE	482,730	20.75
Triacylglycerol (52:2) levels	Parkinson's disease	rs35332062	A	G	A	G	-1.41E-01	-4.60E-03	1.22E-01	8.81E-01	FALSE	FALSE	7	73012042	2.72E-02	6.20E-02	TRUE	7	73597712	2.54E-02	1.00	7168	3.06E-08	TRUE	TRUE	482,730	30.73
Triacylglycerol (52:2) levels	Parkinson's disease	rs501474	G	A	G	A	7.44E-02	-6.80E-03	4.25E-01	6.34E-01	FALSE	FALSE	5	6524005	2.31E-02	1.15E-01	TRUE	5	6523892	1.67E-02	1.00	7168	8.81E-06	TRUE	TRUE	482,730	19.78
Triacylglycerol (52:2) levels	Parkinson's disease	rs62291572	T	C	T	C	9.54E-02	-2.71E-02	2.14E-01	7.47E-01	FALSE	FALSE	4	4997927	2.75E-02	4.89E-01	TRUE	4	4996200	2.10E-02	0.94	7168	5.90E-06	TRUE	TRUE	482,730	20.55
Triacylglycerol (52:2) levels	Parkinson's disease	rs72753379	T	C	T	C	2.06E-01	2.30E-02	3.92E-02	9.80E-01	FALSE	FALSE	9	123112954	9.08E-02	9.67E-02	TRUE	9	120350676	4.47E-02	0.95	7168	4.08E-06	TRUE	TRUE	482,730	21.26
Triacylglycerol (52:2) levels	Parkinson's disease	rs76950187	G	A	G	A	1.54E-01	6.08E-02	7.45E-02	9.52E-01	FALSE	FALSE	2	129184649	5.53E-02	5.65E-01	TRUE	2	128427075	3.20E-02	0.99	7168	1.54E-06	TRUE	TRUE	482,730	23.13
Triacylglycerol (52:2) levels	Parkinson's disease	rs78275146	T	C	T	C	3.50E-01	7.90E-02	1.38E-02	9.87E-01	FALSE	FALSE	1	195998089	1.03E-01	3.52E-01	TRUE	1	196028959	7.53E-02	0.92	7168	3.49E-06	TRUE	TRUE	482,730	21.56
Triacylglycerol (52:2) levels	Parkinson's disease	rs79934453	G	A	G	A	-6.44E-01	-9.28E-02	4.04E-03	9.90E-01	FALSE	FALSE	6	23919234	1.77E-01	2.22E-01	TRUE	6	23919006	1.37E-01	0.91	7168	2.67E-06	TRUE	TRUE	482,730	22.08
Triacylglycerol (52:2) levels	Parkinson's disease	rs79954170	A	C	A	C	-4.43E-01	-9.06E-02	8.14E-03	9.85E-01	FALSE	FALSE	6	49090758	8.53E-02	5.40E-01	TRUE	6	49123122	9.57E-02	0.96	7168	3.85E-06	TRUE	TRUE	482,730	21.37
Triacylglycerol (52:2) levels	Parkinson's disease	rs964184	C	G	C	G	-2.48E-01	-5.68E-02	8.49E-01	8.63E-01	TRUE	FALSE	11	116648917	2.43E-02	9.71E-03	TRUE	11	116778201	2.30E-02	1.00	7168	7.83E-27	TRUE	TRUE	482,730	115.94
Triacylglycerol (52:3) levels	Parkinson's disease	rs1010313	T	C	T	C	1.03E-01	2.37E-02	1.98E-01	8.77E-01	FALSE	FALSE	8	60002542	2.73E-02	4.15E-01	TRUE	8	59089983	2.07E-02	1.00	7173	6.95E-07	TRUE	TRUE	482,730	24.67
Triacylglycerol (52:3) levels	Parkinson's disease	rs10105606	A	C	A	C	-1.29E-01	0.00E+00	2.79E-01	6.64E-01	FALSE	FALSE	8	19827848	1.77E-02	4.34E-05	TRUE	8	19970337	1.85E-02	1.00	7173	2.84E-12	TRUE	TRUE	482,730	48.96
Triacylglycerol (52:3) levels	Parkinson's disease	rs1042034	T	C	T	C	1.10E-01	-5.30E-03	7.28E-01	2.18E-01	FALSE	FALSE	2	21225281	2.03E-02	9.96E-02	TRUE	2	21002409	1.86E-02	1.00	7173	3.90E-09	TRUE	TRUE	482,730	34.75
Triacylglycerol (52:3) levels	Parkinson's disease	rs116011373	A	G	A	G	-1.76E-01	-2.23E-02	4.88E-02	9.73E-01	FALSE	FALSE	3	87347791	8.48E-02	1.01E-01	TRUE	3	87298641	3.92E-02	0.96	7173	7.27E-06	TRUE	TRUE	482,730	20.15
Triacylglycerol (52:3) levels	Parkinson's disease	rs12365864	G	A	G	A	1.12E-01	-2.95E-02	1.66E-01	7.86E-01	FALSE	FALSE	11	116489036	2.73E-02	5.55E-01	TRUE	11	116618319	2.26E-02	0.97	7173	6.71E-07	TRUE	TRUE	482,730	24.74
Triacylglycerol (52:3) levels	Parkinson's disease	rs1260326	C	T	C	T	-1.37E-01	-6.78E-02	6.51E-01	4.20E-01	FALSE	FALSE	2	27730940	1.72E-02	4.04E-05	TRUE	2	27508073	1.74E-02	1.00	7173	4.16E-15	TRUE	TRUE	482,730	61.89
Triacylglycerol (52:3) levels	Parkinson's disease	rs144072769	C	T	C	T	1.57E-01	-1.02E-01	6.82E-02	9.73E-01	FALSE	FALSE	6	155237379	1.71E-01	2.60E-01	TRUE	6	154916245	3.36E-02	0.96	7173	2.84E-06	TRUE	TRUE	482,730	21.95
Triacylglycerol (52:3) levels	Parkinson's disease	rs144313866	A	G	A	G	-1.35E-01	-1.78E-02	8.41E-02	9.25E-01	FALSE	FALSE	4	142471794	4.61E-02	1.56E-01	TRUE	4	141550641	3.04E-02	0.96	7173	8.80E-06	TRUE	TRUE	482,730	19.78
Triacylglycerol (52:3) levels	Parkinson's disease	rs147987331	A	T	A	T	-2.60E-01	-3.21E-02	2.27E-02	2.36E-02	TRUE	FALSE	2	104861307	7.92E-02	1.64E-01	TRUE	2	104244849	5.65E-02	0.97	7173	4.27E-06	TRUE	TRUE	482,730	21.17
Triacylglycerol (52:3) levels	Parkinson's disease	rs16913024	A	G	A	G	-4.57E-01	6.01E-02	8.06E-03	9.79E-01	FALSE	FALSE	10	60822279	7.68E-02	3.63E-01	TRUE	10	59062519	1.03E-01	0.85	7173	9.81E-06	TRUE	TRUE	482,730	19.57
Triacylglycerol (52:3) levels	Parkinson's disease	rs174560	C	T	C	T	7.68E-02	-1.90E-03	3.83E-01	7.01E-01	FALSE	FALSE	11	61581764	1.84E-02	3.82E-02	TRUE	11	61814292	1.73E-02	1.00	7173	8.73E-06	TRUE	TRUE	482,730	19.80
Triacylglycerol (52:3) levels	Parkinson's disease	rs2352723	C	T	C	T	-1.14E-01	-2.69E-02	7.69E-01	1.85E-01	FALSE	FALSE	1	230291868	2.61E-02	5.20E-01	TRUE	1	230156121	1.96E-02	1.00	7173	5.93E-09	TRUE	TRUE	482,730	33.94
Triacylglycerol (52:3) levels	Parkinson's disease	rs2980888	C	T	C	T	-8.86E-02	-2.33E-02	7.43E-01	3.16E-01	FALSE	FALSE	8	126507308	1.82E-02	7.01E-01	TRUE	8	125495066	1.91E-02	1.00	7173	3.51E-06	TRUE	TRUE	482,730	21.55
Triacylglycerol (52:3) levels	Parkinson's disease	rs35332062	A	G	A	G	-1.60E-01	-4.60E-03	1.22E-01	8.81E-01	FALSE	FALSE	7	73012042	2.72E-02	6.20E-02	TRUE	7	73597712	2.54E-02	1.00	7173	3.11E-10	TRUE	TRUE	482,730	39.71
Triacylglycerol (52:3) levels	Parkinson's disease	rs35986054	C	T	C	T	9.47E-02	-1.07E-02	1.96E-01	8.03E-01	FALSE	FALSE	17	1526343	2.45E-02	1.79E-01	TRUE	17	1623049	2.14E-02	0.98	7173	9.56E-06	TRUE	TRUE	482,730	19.62
Triacylglycerol (52:3) levels	Parkinson's disease	rs55724405	C	T	C	T	-8.18E-02	-7.00E-03	2.83E-01	6.70E-01	FALSE	FALSE	1	8259274	2.37E-02	1.14E-01	TRUE	1	8199214	1.85E-02	1.00	7173	9.56E-06	TRUE	TRUE	482,730	19.63
Triacylglycerol (52:3) levels	Parkinson's disease	rs57500102	A	G	A	G	-4.99E-01	8.37E-02	7.31E-03	9.81E-01	FALSE	FALSE	3	171254035	8.57E-02	4.83E-01	TRUE	3	171536246	1.07E-01	0.92	7173	3.10E-06	TRUE	TRUE	482,730	21.79
Triacylglycerol (52:3) levels	Parkinson's disease	rs61702480	A	G	A	G	-8.35E-02	5.00E-04	2.75E-01	7.12E-01	FALSE	FALSE	14	95684801	2.95E-02	6.43E-03	TRUE	14	95218464	1.87E-02	0.99	7173	8.15E-06	TRUE	TRUE	482,730	19.93
Triacylglycerol (52:3) levels	Parkinson's disease	rs6554273	T	C	T	C	7.48E-02	-2.10E-02	4.67E-01	5.58E-01	FALSE	FALSE	4	56226175	2.27E-02	4.50E-01	TRUE	4	55360008	1.68E-02	0.99	7173	8.50E-06	TRUE	TRUE	482,730	19.85
Triacylglycerol (52:3) levels	Parkinson's disease	rs72836628	T	C	T	C	-8.09E-02	3.49E-02	2.97E-01	7.34E-01	FALSE	FALSE	10	113902454	1.99E-02	3.97E-02	TRUE	10	112142696	1.82E-02	1.00	7173	9.11E-06	TRUE	TRUE	482,730	19.72
Triacylglycerol (52:3) levels	Parkinson's disease	rs75237020	C	G	C	G	-2.16E-01	3.98E-02	3.46E-02	3.80E-02	TRUE	FALSE	17	70173415	6.50E-02	2.67E-01	TRUE	17	72177274	4.73E-02	0.90	7173	4.96E-06	TRUE	TRUE	482,730	20.88
Triacylglycerol (52:3) levels	Parkinson's disease	rs75573052	T	C	T	C	-1.38E-01	3.91E-02	8.45E-02	8.89E-01	FALSE	FALSE	13	21612149	3.60E-02	5.57E-01	TRUE	13	21038010	3.04E-02	0.99	7173	5.67E-06	TRUE	TRUE	482,730	20.63
Triacylglycerol (52:3) levels	Parkinson's disease	rs76078494	A	G	A	G	2.09E-01	-4.91E-01	3.45E-02	9.76E-01	FALSE	FALSE	3	188840807	2.70E-01	3.45E-02	TRUE	3	189123018	4.63E-02	0.96	7173	6.73E-06	TRUE	TRUE	482,730	20.30
Triacylglycerol (52:3) levels	Parkinson's disease	rs79954170	A	C	A	C	-5.11E-01	-9.06E-02	8.14E-03	9.85E-01	FALSE	FALSE	6	49090758	8.53E-02	5.40E-01	TRUE	6	49123122	9.56E-02	0.96	7173	9.54E-08	TRUE	TRUE	482,730	28.52
Triacylglycerol (52:3) levels	Parkinson's disease	rs964184	C	G	C	G	-2.99E-01	-5.68E-02	8.49E-01	8.63E-01	TRUE	FALSE	11	116648917	2.43E-02	9.71E-03	TRUE	11	116778201	2.29E-02	1.00	7173	2.31E-38	TRUE	TRUE	482,730	169.71
Triacylglycerol (52:3) levels	Parkinson's disease	rs9997195	C	G	C	G	-8.10E-02	-3.10E-03	6.13E-01	6.86E-01	TRUE	FALSE	4	113925710	1.93E-02	5.86E-02	TRUE	4	113004554	1.72E-02	1.00	7173	2.61E-06	TRUE	TRUE	482,730	22.12
Triacylglycerol (52:4) levels	Parkinson's disease	rs10105606	A	C	A	C	-1.25E-01	0.00E+00	2.79E-01	6.64E-01	FALSE	FALSE	8	19827848	1.77E-02	4.34E-05	TRUE	8	19970337	1.85E-02	1.00	7174	1.44E-11	TRUE	TRUE	482,730	45.76
Triacylglycerol (52:4) levels	Parkinson's disease	rs1042034	T	C	T	C	1.06E-01	-5.30E-03	7.28E-01	2.18E-01	FALSE	FALSE	2	21225281	2.03E-02	9.96E-02	TRUE	2	21002409	1.86E-02	1.00	7174	1.28E-08	TRUE	TRUE	482,730	32.43
Triacylglycerol (52:4) levels	Parkinson's disease	rs10773708	A	G	A	G	-8.16E-02	3.91E-02	6.83E-01	2.93E-01	FALSE	FALSE	12	130281212	2.62E-02	8.67E-01	TRUE	12	129796667	1.80E-02	1.00	7174	6.20E-06	TRUE	TRUE	482,730	20.46
Triacylglycerol (52:4) levels	Parkinson's disease	rs10779836	T	C	T	C	-1.17E-01	-4.40E-03	7.77E-01	1.96E-01	FALSE	FALSE	1	230303150	2.40E-02	6.80E-02	TRUE	1	230167404	1.99E-02	1.00	7174	4.90E-09	TRUE	TRUE	482,730	34.31
Triacylglycerol (52:4) levels	Parkinson's disease	rs116497138	T	G	T	G	-1.83E-01	5.25E-02	4.98E-02	9.53E-01	FALSE	FALSE	3	87229633	6.09E-02	4.10E-01	TRUE	3	87180483	3.91E-02	0.94	7174	3.01E-06	TRUE	TRUE	482,730	21.84
Triacylglycerol (52:4) levels	Parkinson's disease	rs11783556	G	A	G	A	-9.53E-02	2.30E-02	2.00E-01	7.84E-01	FALSE	FALSE	8	714450	2.74E-02	3.95E-01	TRUE	8	764450	2.11E-02	0.99	7174	6.06E-06	TRUE	TRUE	482,730	20.50
Triacylglycerol (52:4) levels	Parkinson's disease	rs12365864	G	A	G	A	1.05E-01	-2.95E-02	1.66E-01	7.86E-01	FALSE	FALSE	11	116489036	2.73E-02	5.55E-01	TRUE	11	116618319	2.26E-02	0.97	7174	3.23E-06	TRUE	TRUE	482,730	21.71
Triacylglycerol (52:4) levels	Parkinson's disease	rs1260326	C	T	C	T	-1.39E-01	-6.78E-02	6.51E-01	4.20E-01	FALSE	FALSE	2	27730940	1.72E-02	4.04E-05	TRUE	2	27508073	1.74E-02	1.00	7174	1.80E-15	TRUE	TRUE	482,730	63.55
Triacylglycerol (52:4) levels	Parkinson's disease	rs13259370	A	G	A	G	9.88E-02	2.41E-02	1.97E-01	8.77E-01	FALSE	FALSE	8	60002813	2.73E-02	4.24E-01	TRUE	8	59090254	2.08E-02	1.00	7174	2.11E-06	TRUE	TRUE	482,730	22.53
Triacylglycerol (52:4) levels	Parkinson's disease	rs13261274	G	A	G	A	-7.44E-02	3.93E-02	5.31E-01	5.05E-01	FALSE	FALSE	8	98238267	2.22E-02	3.83E-02	TRUE	8	97226039	1.66E-02	1.00	7174	7.82E-06	TRUE	TRUE	482,730	20.01
Triacylglycerol (52:4) levels	Parkinson's disease	rs142146849	C	A	C	A	1.60E-01	7.85E-02	5.48E-02	9.38E-01	FALSE	FALSE	10	103726221	3.84E-02	2.05E-02	TRUE	10	101966464	3.62E-02	1.00	7174	9.83E-06	TRUE	TRUE	482,730	19.57
Triacylglycerol (52:4) levels	Parkinson's disease	rs144072769	C	T	C	T	1.63E-01	-1.02E-01	6.82E-02	9.73E-01	FALSE	FALSE	6	155237379	1.71E-01	2.60E-01	TRUE	6	154916245	3.36E-02	0.96	7174	1.17E-06	TRUE	TRUE	482,730	23.66
Triacylglycerol (52:4) levels	Parkinson's disease	rs56376607	T	C	T	C	-2.20E-01	5.94E-02	2.96E-02	9.18E-01	FALSE	FALSE	4	100728750	4.30E-02	7.77E-01	TRUE	4	99807593	4.96E-02	0.96	7174	9.24E-06	TRUE	TRUE	482,730	19.69
Triacylglycerol (52:4) levels	Parkinson's disease	rs61702480	A	G	A	G	-8.30E-02	5.00E-04	2.75E-01	7.12E-01	FALSE	FALSE	14	95684801	2.95E-02	6.43E-03	TRUE	14	95218464	1.87E-02	0.99	7174	9.36E-06	TRUE	TRUE	482,730	19.66
Triacylglycerol (52:4) levels	Parkinson's disease	rs72900657	A	G	A	G	-2.61E-01	-5.35E-02	2.31E-02	9.63E-01	FALSE	FALSE	11	45848147	6.50E-02	3.87E-01	TRUE	11	45826596	5.76E-02	0.92	7174	6.06E-06	TRUE	TRUE	482,730	20.50
Triacylglycerol (52:4) levels	Parkinson's disease	rs76330538	C	T	C	T	2.57E-01	1.21E-01	2.18E-02	9.90E-01	FALSE	FALSE	2	212926299	1.73E-01	3.15E-01	TRUE	2	212061574	5.81E-02	0.98	7174	9.76E-06	TRUE	TRUE	482,730	19.58
Triacylglycerol (52:4) levels	Parkinson's disease	rs79624003	G	A	G	A	-1.41E-01	-5.80E-03	1.22E-01	8.81E-01	FALSE	FALSE	7	73012785	2.71E-02	8.04E-02	TRUE	7	73598455	2.54E-02	1.00	7174	2.70E-08	TRUE	TRUE	482,730	30.98
Triacylglycerol (52:4) levels	Parkinson's disease	rs79954170	A	C	A	C	-4.66E-01	-9.06E-02	8.14E-03	9.85E-01	FALSE	FALSE	6	49090758	8.53E-02	5.40E-01	TRUE	6	49123122	9.57E-02	0.96	7174	1.15E-06	TRUE	TRUE	482,730	23.69
Triacylglycerol (52:4) levels	Parkinson's disease	rs9344829	T	C	T	C	7.84E-02	-2.70E-03	3.73E-01	6.88E-01	FALSE	FALSE	6	64887066	1.90E-02	5.16E-02	TRUE	6	64177173	1.72E-02	0.99	7174	5.25E-06	TRUE	TRUE	482,730	20.77
Triacylglycerol (52:4) levels	Parkinson's disease	rs964184	C	G	C	G	-2.84E-01	-5.68E-02	8.49E-01	8.63E-01	TRUE	FALSE	11	116648917	2.43E-02	9.71E-03	TRUE	11	116778201	2.30E-02	1.00	7174	6.53E-35	TRUE	TRUE	482,730	153.56
Triacylglycerol (52:5) levels	Parkinson's disease	rs10105606	A	C	A	C	-1.12E-01	0.00E+00	2.79E-01	6.64E-01	FALSE	FALSE	8	19827848	1.77E-02	4.34E-05	TRUE	8	19970337	1.85E-02	1.00	7172	1.58E-09	TRUE	TRUE	482,730	36.53
Triacylglycerol (52:5) levels	Parkinson's disease	rs1042034	T	C	T	C	9.04E-02	-5.30E-03	7.28E-01	2.18E-01	FALSE	FALSE	2	21225281	2.03E-02	9.96E-02	TRUE	2	21002409	1.86E-02	1.00	7172	1.22E-06	TRUE	TRUE	482,730	23.58
Triacylglycerol (52:5) levels	Parkinson's disease	rs10779836	T	C	T	C	-9.43E-02	-4.40E-03	7.77E-01	1.96E-01	FALSE	FALSE	1	230303150	2.40E-02	6.80E-02	TRUE	1	230167404	1.99E-02	1.00	7172	2.30E-06	TRUE	TRUE	482,730	22.36
Triacylglycerol (52:5) levels	Parkinson's disease	rs111568723	G	A	G	A	2.41E-01	-1.20E-01	2.59E-02	9.47E-01	FALSE	FALSE	9	135345889	5.14E-02	9.73E-03	TRUE	9	132470502	5.45E-02	0.95	7172	1.00E-05	TRUE	TRUE	482,730	19.54
Triacylglycerol (52:5) levels	Parkinson's disease	rs116497138	T	G	T	G	-1.74E-01	5.25E-02	4.98E-02	9.53E-01	FALSE	FALSE	3	87229633	6.09E-02	4.10E-01	TRUE	3	87180483	3.91E-02	0.94	7172	8.43E-06	TRUE	TRUE	482,730	19.87
Triacylglycerol (52:5) levels	Parkinson's disease	rs11783556	G	A	G	A	-9.84E-02	2.30E-02	2.00E-01	7.84E-01	FALSE	FALSE	8	714450	2.74E-02	3.95E-01	TRUE	8	764450	2.11E-02	0.99	7172	3.05E-06	TRUE	TRUE	482,730	21.82
Triacylglycerol (52:5) levels	Parkinson's disease	rs1260326	C	T	C	T	-1.54E-01	-6.78E-02	6.51E-01	4.20E-01	FALSE	FALSE	2	27730940	1.72E-02	4.04E-05	TRUE	2	27508073	1.74E-02	1.00	7172	1.01E-18	TRUE	TRUE	482,730	78.46
Triacylglycerol (52:5) levels	Parkinson's disease	rs13259370	A	G	A	G	9.31E-02	2.41E-02	1.97E-01	8.77E-01	FALSE	FALSE	8	60002813	2.73E-02	4.24E-01	TRUE	8	59090254	2.08E-02	1.00	7172	7.84E-06	TRUE	TRUE	482,730	20.00
Triacylglycerol (52:5) levels	Parkinson's disease	rs142146849	C	A	C	A	1.71E-01	7.85E-02	5.48E-02	9.38E-01	FALSE	FALSE	10	103726221	3.84E-02	2.05E-02	TRUE	10	101966464	3.62E-02	1.00	7172	2.46E-06	TRUE	TRUE	482,730	22.24
Triacylglycerol (52:5) levels	Parkinson's disease	rs144072769	C	T	C	T	1.57E-01	-1.02E-01	6.82E-02	9.73E-01	FALSE	FALSE	6	155237379	1.71E-01	2.60E-01	TRUE	6	154916245	3.36E-02	0.96	7172	2.86E-06	TRUE	TRUE	482,730	21.94
Triacylglycerol (52:5) levels	Parkinson's disease	rs17596144	T	C	T	C	1.10E-01	-2.90E-03	1.52E-01	8.43E-01	FALSE	FALSE	1	220978645	2.58E-02	4.12E-02	TRUE	1	220805303	2.34E-02	0.99	7172	2.60E-06	TRUE	TRUE	482,730	22.13
Triacylglycerol (52:5) levels	Parkinson's disease	rs190411877	C	T	C	T	4.28E-01	5.44E-02	9.45E-03	9.93E-01	FALSE	FALSE	10	7913102	2.09E-01	9.96E-02	TRUE	10	7871139	8.80E-02	0.94	7172	1.15E-06	TRUE	TRUE	482,730	23.70
Triacylglycerol (52:5) levels	Parkinson's disease	rs33951980	T	C	T	C	-1.13E-01	-6.00E-03	1.22E-01	8.79E-01	FALSE	FALSE	7	73029437	2.71E-02	8.41E-02	TRUE	7	73615107	2.54E-02	1.00	7172	8.70E-06	TRUE	TRUE	482,730	19.81
Triacylglycerol (52:5) levels	Parkinson's disease	rs35986054	C	T	C	T	1.00E-01	-1.07E-02	1.96E-01	8.03E-01	FALSE	FALSE	17	1526343	2.45E-02	1.79E-01	TRUE	17	1623049	2.14E-02	0.98	7172	2.77E-06	TRUE	TRUE	482,730	22.01
Triacylglycerol (52:5) levels	Parkinson's disease	rs4529433	G	T	G	T	8.43E-02	-1.49E-02	2.68E-01	7.72E-01	FALSE	FALSE	8	101394418	2.70E-02	2.36E-01	TRUE	8	100382190	1.90E-02	0.99	7172	9.02E-06	TRUE	TRUE	482,730	19.73
Triacylglycerol (52:5) levels	Parkinson's disease	rs6753694	G	C	G	C	-8.24E-02	-1.97E-02	2.84E-01	2.57E-01	TRUE	FALSE	2	42466280	2.56E-02	3.56E-01	TRUE	2	42239140	1.85E-02	1.00	7172	8.14E-06	TRUE	TRUE	482,730	19.93
Triacylglycerol (52:5) levels	Parkinson's disease	rs72900657	A	G	A	G	-2.73E-01	-5.35E-02	2.31E-02	9.63E-01	FALSE	FALSE	11	45848147	6.50E-02	3.87E-01	TRUE	11	45826596	5.76E-02	0.92	7172	2.21E-06	TRUE	TRUE	482,730	22.44
Triacylglycerol (52:5) levels	Parkinson's disease	rs75560495	G	A	G	A	-1.21E-01	2.01E-02	1.28E-01	8.69E-01	FALSE	FALSE	17	77402735	4.47E-02	1.86E-01	TRUE	17	79406653	2.68E-02	0.88	7172	6.03E-06	TRUE	TRUE	482,730	20.51
Triacylglycerol (52:5) levels	Parkinson's disease	rs7996305	T	C	T	C	8.60E-02	2.00E-04	2.50E-01	6.79E-01	FALSE	FALSE	13	51122510	1.93E-02	3.53E-03	TRUE	13	50548374	1.93E-02	1.00	7172	7.99E-06	TRUE	TRUE	482,730	19.97
Triacylglycerol (52:5) levels	Parkinson's disease	rs9344829	T	C	T	C	8.00E-02	-2.70E-03	3.73E-01	6.88E-01	FALSE	FALSE	6	64887066	1.90E-02	5.16E-02	TRUE	6	64177173	1.72E-02	0.99	7172	3.41E-06	TRUE	TRUE	482,730	21.60
Triacylglycerol (52:5) levels	Parkinson's disease	rs964184	C	G	C	G	-2.51E-01	-5.68E-02	8.49E-01	8.63E-01	TRUE	FALSE	11	116648917	2.43E-02	9.71E-03	TRUE	11	116778201	2.30E-02	1.00	7172	1.59E-27	TRUE	TRUE	482,730	119.15
Triacylglycerol (52:6) levels	Parkinson's disease	rs10105606	A	C	A	C	-9.48E-02	0.00E+00	2.79E-01	6.64E-01	FALSE	FALSE	8	19827848	1.77E-02	4.34E-05	TRUE	8	19970337	1.93E-02	1.00	6554	9.51E-07	TRUE	TRUE	482,730	24.07
Triacylglycerol (52:6) levels	Parkinson's disease	rs1017216	A	G	A	G	8.13E-02	-2.06E-02	4.59E-01	5.55E-01	FALSE	FALSE	4	181381842	2.23E-02	4.49E-01	TRUE	4	180460689	1.73E-02	1.00	6554	2.73E-06	TRUE	TRUE	482,730	22.04
Triacylglycerol (52:6) levels	Parkinson's disease	rs102274	C	T	C	T	-1.32E-01	-3.90E-03	4.09E-01	6.70E-01	FALSE	FALSE	11	61557826	1.79E-02	8.34E-02	TRUE	11	61790354	1.78E-02	1.00	6554	1.00E-13	TRUE	TRUE	482,730	55.60
Triacylglycerol (52:6) levels	Parkinson's disease	rs112578388	T	C	T	C	-5.76E-01	-2.36E-02	5.33E-03	9.88E-01	FALSE	FALSE	13	26460581	2.04E-01	4.20E-02	TRUE	13	25886443	1.26E-01	0.86	6554	5.22E-06	TRUE	TRUE	482,730	20.79
Triacylglycerol (52:6) levels	Parkinson's disease	rs114314612	A	C	A	C	4.13E-01	-6.57E-02	9.62E-03	9.82E-01	FALSE	FALSE	2	193891775	9.83E-02	2.98E-01	TRUE	2	193027049	9.19E-02	0.93	6554	6.97E-06	TRUE	TRUE	482,730	20.23
Triacylglycerol (52:6) levels	Parkinson's disease	rs117082735	T	C	T	C	-2.64E-01	-1.86E-02	2.23E-02	9.86E-01	FALSE	FALSE	9	82482394	9.81E-02	7.07E-02	TRUE	9	79867479	5.87E-02	0.98	6554	6.72E-06	TRUE	TRUE	482,730	20.30
Triacylglycerol (52:6) levels	Parkinson's disease	rs117518401	T	C	T	C	1.12E-01	3.10E-02	1.73E-01	8.68E-01	FALSE	FALSE	3	133614605	3.42E-02	4.37E-01	TRUE	3	133895761	2.34E-02	0.98	6554	1.70E-06	TRUE	TRUE	482,730	22.95
Triacylglycerol (52:6) levels	Parkinson's disease	rs1260326	C	T	C	T	-1.32E-01	-6.78E-02	6.51E-01	4.20E-01	FALSE	FALSE	2	27730940	1.72E-02	4.04E-05	TRUE	2	27508073	1.82E-02	1.00	6554	4.04E-13	TRUE	TRUE	482,730	52.83
Triacylglycerol (52:6) levels	Parkinson's disease	rs4508726	G	A	G	A	-8.71E-02	-1.15E-02	3.65E-01	5.63E-01	FALSE	FALSE	3	37429422	1.77E-02	2.89E-01	TRUE	3	37387931	1.82E-02	1.00	6554	1.77E-06	TRUE	TRUE	482,730	22.88
Triacylglycerol (52:6) levels	Parkinson's disease	rs56257715	A	G	A	G	4.28E-01	-2.63E-02	9.28E-03	9.86E-01	FALSE	FALSE	7	70995459	1.18E-01	8.41E-02	TRUE	7	71530474	9.25E-02	0.91	6554	3.70E-06	TRUE	TRUE	482,730	21.44
Triacylglycerol (52:6) levels	Parkinson's disease	rs61905503	C	G	C	G	-1.90E-01	2.24E-02	4.98E-02	8.68E-02	TRUE	FALSE	11	89360302	3.15E-02	3.22E-01	TRUE	11	89627134	4.01E-02	0.99	6554	2.23E-06	TRUE	TRUE	482,730	22.43
Triacylglycerol (52:6) levels	Parkinson's disease	rs6752909	T	C	T	C	-1.04E-01	3.28E-02	8.26E-01	1.72E-01	FALSE	FALSE	2	45710097	2.97E-02	5.70E-01	TRUE	2	45482958	2.31E-02	1.00	6554	7.19E-06	TRUE	TRUE	482,730	20.17
Triacylglycerol (52:6) levels	Parkinson's disease	rs78095833	T	C	T	C	-3.06E-01	-1.08E-01	1.99E-02	9.84E-01	FALSE	FALSE	1	237542217	9.79E-02	5.67E-01	TRUE	1	237378917	6.44E-02	0.90	6554	2.01E-06	TRUE	TRUE	482,730	22.62
Triacylglycerol (52:6) levels	Parkinson's disease	rs7815372	G	A	G	A	9.80E-02	1.07E-02	7.92E-01	2.77E-01	FALSE	FALSE	8	65608460	2.10E-02	2.16E-01	TRUE	8	64695903	2.15E-02	1.00	6554	5.52E-06	TRUE	TRUE	482,730	20.68
Triacylglycerol (52:6) levels	Parkinson's disease	rs78178542	C	T	C	T	-3.13E-01	5.70E-02	1.96E-02	9.78E-01	FALSE	FALSE	4	179936216	8.77E-02	2.88E-01	TRUE	4	179015062	6.52E-02	0.92	6554	1.59E-06	TRUE	TRUE	482,730	23.07
Triacylglycerol (52:6) levels	Parkinson's disease	rs9591384	A	T	A	T	-1.28E-01	-3.30E-02	1.24E-01	1.50E-01	TRUE	FALSE	13	51639473	3.15E-02	5.29E-01	TRUE	13	51065337	2.65E-02	1.00	6554	1.25E-06	TRUE	TRUE	482,730	23.55
Triacylglycerol (52:6) levels	Parkinson's disease	rs964184	C	G	C	G	-2.03E-01	-5.68E-02	8.49E-01	8.63E-01	TRUE	FALSE	11	116648917	2.43E-02	9.71E-03	TRUE	11	116778201	2.41E-02	1.00	6554	3.70E-17	TRUE	TRUE	482,730	71.32
Triacylglycerol (53:2) levels	Parkinson's disease	rs10147474	G	A	G	A	-2.19E-01	3.15E-02	3.72E-02	9.66E-01	FALSE	FALSE	14	30292648	6.22E-02	2.13E-01	TRUE	14	29823442	4.54E-02	0.98	6996	1.54E-06	TRUE	TRUE	482,730	23.14
Triacylglycerol (53:2) levels	Parkinson's disease	rs1042034	T	C	T	C	8.37E-02	-5.30E-03	7.28E-01	2.18E-01	FALSE	FALSE	2	21225281	2.03E-02	9.96E-02	TRUE	2	21002409	1.89E-02	1.00	6996	9.40E-06	TRUE	TRUE	482,730	19.66
Triacylglycerol (53:2) levels	Parkinson's disease	rs111241638	C	T	C	T	-2.22E-01	-5.13E-02	3.27E-02	9.60E-01	FALSE	FALSE	3	167830430	6.15E-02	3.94E-01	TRUE	3	168112642	4.99E-02	0.91	6996	8.97E-06	TRUE	TRUE	482,730	19.75
Triacylglycerol (53:2) levels	Parkinson's disease	rs116497138	T	G	T	G	-1.84E-01	5.25E-02	4.98E-02	9.53E-01	FALSE	FALSE	3	87229633	6.09E-02	4.10E-01	TRUE	3	87180483	3.97E-02	0.94	6996	3.54E-06	TRUE	TRUE	482,730	21.53
Triacylglycerol (53:2) levels	Parkinson's disease	rs11926107	G	A	G	A	-8.34E-02	-1.72E-02	2.84E-01	6.38E-01	FALSE	FALSE	3	134714338	2.32E-02	3.40E-01	TRUE	3	134995496	1.87E-02	1.00	6996	8.32E-06	TRUE	TRUE	482,730	19.89
Triacylglycerol (53:2) levels	Parkinson's disease	rs1260326	C	T	C	T	-1.19E-01	-6.78E-02	6.51E-01	4.20E-01	FALSE	FALSE	2	27730940	1.72E-02	4.04E-05	TRUE	2	27508073	1.76E-02	1.00	6996	1.73E-11	TRUE	TRUE	482,730	45.40

Triacylglycerol (53:2) levels	Parkinson's disease	rs12635725	G	A	G	A	7.58E-02	2.07E-02	5.60E-01	4.47E-01	FALSE	FALSE	FALSE	3	1937149	2.20E-02	4.61E-01	TRUE	3	1895465	1.69E-02	0.99	6996	7.41E-06	TRUE	TRUE	482,730	20.11
Triacylglycerol (53:2) levels	Parkinson's disease	rs150741556	A	G	A	G	2.35E-01	1.04E-02	2.62E-02	9.88E-01	FALSE	FALSE	FALSE	5	83102973	1.11E-01	3.35E-02	TRUE	5	83807154	5.27E-02	0.98	6996	8.45E-06	TRUE	TRUE	482,730	19.86
Triacylglycerol (53:2) levels	Parkinson's disease	rs174556	T	C	T	C	9.52E-02	1.00E-04	3.80E-01	7.09E-01	FALSE	FALSE	FALSE	11	61580635	1.85E-02	1.04E-03	TRUE	11	61813163	1.75E-02	1.00	6996	5.17E-08	TRUE	TRUE	482,730	29.71
Triacylglycerol (53:2) levels	Parkinson's disease	rs34021847	A	G	A	G	-8.84E-02	-3.21E-02	3.20E-01	6.61E-01	FALSE	FALSE	FALSE	7	2037761	2.02E-02	9.53E-01	TRUE	7	1998126	1.81E-02	0.99	6996	1.03E-06	TRUE	TRUE	482,730	23.92
Triacylglycerol (53:2) levels	Parkinson's disease	rs35332062	A	G	A	G	-1.34E-01	-4.60E-03	1.22E-01	8.81E-01	FALSE	FALSE	FALSE	7	73012042	2.72E-02	6.20E-02	TRUE	7	73597712	2.59E-02	1.00	6996	2.21E-07	TRUE	TRUE	482,730	26.89
Triacylglycerol (53:2) levels	Parkinson's disease	rs4266911	C	T	C	T	1.65E-01	-3.37E-02	9.41E-01	4.21E-02	FALSE	FALSE	FALSE	1	27426796	5.78E-02	2.52E-01	TRUE	1	27100305	3.67E-02	0.94	6996	6.96E-06	TRUE	TRUE	482,730	20.23
Triacylglycerol (53:2) levels	Parkinson's disease	rs55870249	G	C	G	C	9.06E-02	1.58E-02	2.25E-01	1.70E-01	TRUE	FALSE	FALSE	3	177221968	3.00E-02	2.23E-01	TRUE	3	177504180	2.03E-02	1.00	6996	7.99E-06	TRUE	TRUE	482,730	19.97
Triacylglycerol (53:2) levels	Parkinson's disease	rs62043594	A	C	A	C	-9.06E-02	-1.90E-02	4.18E-01	6.26E-01	FALSE	FALSE	FALSE	15	93697383	2.31E-02	3.86E-01	TRUE	15	93154154	1.70E-02	1.00	6996	9.63E-08	TRUE	TRUE	482,730	28.50
Triacylglycerol (53:2) levels	Parkinson's disease	rs62291572	T	C	T	C	1.03E-01	-2.71E-02	2.14E-01	7.47E-01	FALSE	FALSE	FALSE	4	4997927	2.75E-02	4.89E-01	TRUE	4	4996200	2.12E-02	0.94	6996	1.20E-06	TRUE	TRUE	482,730	23.62
Triacylglycerol (53:2) levels	Parkinson's disease	rs72679249	G	A	G	A	9.18E-02	-1.21E-02	2.70E-01	6.79E-01	FALSE	FALSE	FALSE	4	111122162	2.92E-02	1.69E-01	TRUE	4	110201006	1.93E-02	0.97	6996	2.06E-06	TRUE	TRUE	482,730	22.58
Triacylglycerol (53:2) levels	Parkinson's disease	rs75573052	T	C	T	C	-1.42E-01	3.91E-02	8.45E-02	8.89E-01	FALSE	FALSE	FALSE	13	21612149	3.60E-02	5.57E-01	TRUE	13	21038010	3.09E-02	0.99	6996	4.79E-06	TRUE	TRUE	482,730	20.95
Triacylglycerol (53:2) levels	Parkinson's disease	rs76950187	G	A	G	A	1.57E-01	6.08E-02	7.45E-02	9.52E-01	FALSE	FALSE	FALSE	2	129184649	5.53E-02	5.65E-01	TRUE	2	128427075	3.23E-02	0.99	6996	1.17E-06	TRUE	TRUE	482,730	23.66
Triacylglycerol (53:2) levels	Parkinson's disease	rs964184	C	G	C	G	-2.33E-01	-5.68E-02	8.49E-01	8.63E-01	TRUE	FALSE	FALSE	11	116648917	2.43E-02	9.71E-03	TRUE	11	116778201	2.33E-02	1.00	6996	2.38E-23	TRUE	TRUE	482,730	99.82
Triacylglycerol (53:3) levels	Parkinson's disease	rs1003966	T	C	T	C	-1.50E-01	3.83E-02	8.46E-02	8.89E-01	FALSE	FALSE	FALSE	13	21612159	3.60E-02	5.42E-01	TRUE	13	21038020	3.04E-02	0.99	7113	9.16E-07	TRUE	TRUE	482,730	24.14
Triacylglycerol (53:3) levels	Parkinson's disease	rs10147474	G	A	G	A	-2.11E-01	3.15E-02	3.72E-02	9.66E-01	FALSE	FALSE	FALSE	14	30292648	6.22E-02	2.13E-01	TRUE	14	29823442	4.49E-02	0.98	7113	2.67E-06	TRUE	TRUE	482,730	22.07
Triacylglycerol (53:3) levels	Parkinson's disease	rs1042034	T	C	T	C	9.71E-02	-5.30E-03	7.28E-01	2.18E-01	FALSE	FALSE	FALSE	2	21225281	2.03E-02	9.96E-02	TRUE	2	21002409	1.87E-02	1.00	7113	2.12E-07	TRUE	TRUE	482,730	26.97
Triacylglycerol (53:3) levels	Parkinson's disease	rs111568723	G	A	G	A	2.50E-01	-1.20E-01	2.59E-02	9.47E-01	FALSE	FALSE	FALSE	9	135345889	5.14E-02	9.73E-03	TRUE	9	132470502	5.46E-02	0.95	7113	4.67E-06	TRUE	TRUE	482,730	21.00
Triacylglycerol (53:3) levels	Parkinson's disease	rs11584070	G	A	G	A	-1.14E-01	3.60E-02	1.37E-01	8.81E-01	FALSE	FALSE	FALSE	1	150294925	2.95E-02	6.55E-01	TRUE	1	150322476	2.44E-02	1.00	7113	2.83E-06	TRUE	TRUE	482,730	21.96
Triacylglycerol (53:3) levels	Parkinson's disease	rs116011373	A	G	A	G	-1.92E-01	-2.23E-02	4.88E-02	9.73E-01	FALSE	FALSE	FALSE	3	87347791	8.48E-02	1.01E-01	TRUE	3	87298641	3.93E-02	0.96	7113	1.02E-06	TRUE	TRUE	482,730	23.94
Triacylglycerol (53:3) levels	Parkinson's disease	rs12448223	C	T	C	T	7.74E-02	1.20E-02	4.43E-01	5.34E-01	FALSE	FALSE	FALSE	16	4154551	2.19E-02	2.35E-01	TRUE	16	4104550	1.67E-02	1.00	7113	3.77E-06	TRUE	TRUE	482,730	21.41
Triacylglycerol (53:3) levels	Parkinson's disease	rs1260326	C	T	C	T	-1.37E-01	-6.78E-02	6.51E-01	4.20E-01	FALSE	FALSE	FALSE	2	27730940	1.72E-02	4.04E-05	TRUE	2	27508073	1.75E-02	1.00	7113	4.37E-15	TRUE	TRUE	482,730	61.78
Triacylglycerol (53:3) levels	Parkinson's disease	rs12635725	G	A	G	A	8.07E-02	2.07E-02	5.60E-01	4.47E-01	FALSE	FALSE	FALSE	3	1937149	2.20E-02	4.61E-01	TRUE	3	1895465	1.67E-02	0.99	7113	1.46E-06	TRUE	TRUE	482,730	23.23
Triacylglycerol (53:3) levels	Parkinson's disease	rs138622921	C	A	C	A	-2.91E-01	-4.47E-02	1.70E-02	9.86E-01	FALSE	FALSE	FALSE	12	26910068	1.13E-01	1.60E-01	TRUE	12	26757135	6.46E-02	0.99	7113	6.86E-06	TRUE	TRUE	482,730	20.26
Triacylglycerol (53:3) levels	Parkinson's disease	rs139500046	C	A	C	A	-3.81E-01	-4.41E-02	1.12E-02	9.83E-01	FALSE	FALSE	FALSE	12	97244448	8.65E-02	2.15E-01	TRUE	12	96850670	7.94E-02	0.99	7113	1.56E-06	TRUE	TRUE	482,730	23.11
Triacylglycerol (53:3) levels	Parkinson's disease	rs150741556	A	G	A	G	2.58E-01	1.04E-02	2.62E-02	9.88E-01	FALSE	FALSE	FALSE	5	83102973	1.11E-01	3.35E-02	TRUE	5	83807154	5.25E-02	0.98	7113	9.24E-07	TRUE	TRUE	482,730	24.12
Triacylglycerol (53:3) levels	Parkinson's disease	rs174560	C	T	C	T	1.04E-01	-1.90E-03	3.83E-01	7.01E-01	FALSE	FALSE	FALSE	11	61581764	1.84E-02	3.82E-02	TRUE	11	61814292	1.73E-02	1.00	7113	1.96E-09	TRUE	TRUE	482,730	36.11
Triacylglycerol (53:3) levels	Parkinson's disease	rs28450659	T	C	T	C	-1.19E-01	-3.90E-02	1.12E-01	8.29E-01	FALSE	FALSE	FALSE	3	15374050	2.63E-02	8.59E-01	TRUE	3	15332543	2.63E-02	0.99	7113	6.66E-06	TRUE	TRUE	482,730	20.32
Triacylglycerol (53:3) levels	Parkinson's disease	rs35332062	A	G	A	G	-1.48E-01	-4.60E-03	1.22E-01	8.81E-01	FALSE	FALSE	FALSE	7	73012042	2.72E-02	6.20E-02	TRUE	7	73597712	2.55E-02	1.00	7113	7.43E-09	TRUE	TRUE	482,730	33.50
Triacylglycerol (53:3) levels	Parkinson's disease	rs3829088	C	T	C	T	9.24E-02	2.59E-02	2.03E-01	8.53E-01	FALSE	FALSE	FALSE	9	23713680	3.15E-02	3.86E-01	TRUE	9	23713682	2.08E-02	1.00	7113	9.16E-06	TRUE	TRUE	482,730	19.71
Triacylglycerol (53:3) levels	Parkinson's disease	rs501474	G	A	G	A	7.66E-02	-6.80E-03	4.25E-01	6.34E-01	FALSE	FALSE	FALSE	5	6524005	2.31E-02	1.15E-01	TRUE	5	6523892	1.68E-02	1.00	7113	5.14E-06	TRUE	TRUE	482,730	20.82
Triacylglycerol (53:3) levels	Parkinson's disease	rs62291574	A	G	A	G	9.93E-02	-2.16E-02	2.14E-01	7.47E-01	FALSE	FALSE	FALSE	4	5005538	2.99E-02	3.29E-01	TRUE	4	5003811	2.11E-02	0.94	7113	2.61E-06	TRUE	TRUE	482,730	22.12
Triacylglycerol (53:3) levels	Parkinson's disease	rs6692319	T	C	T	C	-9.52E-02	-1.94E-02	7.40E-01	1.99E-01	FALSE	FALSE	FALSE	1	230284375	2.53E-02	3.53E-01	TRUE	1	230148628	1.88E-02	1.00	7113	4.41E-07	TRUE	TRUE	482,730	25.55
Triacylglycerol (53:3) levels	Parkinson's disease	rs76950187	G	A	G	A	1.48E-01	6.08E-02	7.45E-02	9.52E-01	FALSE	FALSE	FALSE	2	129184649	5.53E-02	5.65E-01	TRUE	2	128427075	3.21E-02	0.99	7113	4.23E-06	TRUE	TRUE	482,730	21.19
Triacylglycerol (53:3) levels	Parkinson's disease	rs935833	A	G	A	G	-7.87E-02	1.53E-02	6.35E-01	3.45E-01	FALSE	FALSE	FALSE	12	130276381	2.34E-02	2.89E-01	TRUE	12	129791836	1.75E-02	0.99	7113	7.19E-06	TRUE	TRUE	482,730	20.17
Triacylglycerol (53:3) levels	Parkinson's disease	rs964184	C	G	C	G	-2.83E-01	-5.68E-02	8.49E-01	8.63E-01	TRUE	FALSE	FALSE	11	116648917	2.43E-02	9.71E-03	TRUE	11	116778201	2.30E-02	1.00	7113	2.83E-34	TRUE	TRUE	482,730	150.59
Triacylglycerol (53:4) levels	Parkinson's disease	rs10105606	A	C	A	C	-1.12E-01	0.00E+00	2.79E-01	6.64E-01	FALSE	FALSE	FALSE	8	19827848	1.77E-02	4.34E-05	TRUE	8	19970337	1.96E-02	1.00	6479	1.29E-08	TRUE	TRUE	482,730	32.42
Triacylglycerol (53:4) levels	Parkinson's disease	rs116014876	T	C	T	C	4.20E-01	-2.42E-02	8.94E-03	9.73E-01	FALSE	FALSE	FALSE	1	110200131	6.12E-02	1.59E-01	TRUE	1	109657509	9.36E-02	0.97	6479	7.33E-06	TRUE	TRUE	482,730	20.14
Triacylglycerol (53:4) levels	Parkinson's disease	rs117829392	C	G	C	G	2.33E-01	-3.91E-02	3.20E-02	5.81E-02	TRUE	FALSE	FALSE	8	721605	5.06E-02	3.58E-01	TRUE	8	771605	5.12E-02	0.96	6479	5.50E-06	TRUE	TRUE	482,730	20.69
Triacylglycerol (53:4) levels	Parkinson's disease	rs117922651	A	G	A	G	-1.77E-01	1.42E-02	5.71E-02	8.84E-01	FALSE	FALSE	FALSE	17	39984746	3.42E-02	1.68E-01	TRUE	17	41828494	3.86E-02	1.00	6479	4.79E-06	TRUE	TRUE	482,730	20.95
Triacylglycerol (53:4) levels	Parkinson's disease	rs1260326	C	T	C	T	-1.36E-01	-6.78E-02	6.51E-01	4.20E-01	FALSE	FALSE	FALSE	2	27730940	1.72E-02	4.04E-05	TRUE	2	27508073	1.83E-02	1.00	6479	1.14E-13	TRUE	TRUE	482,730	55.34
Triacylglycerol (53:4) levels	Parkinson's disease	rs12635725	G	A	G	A	7.95E-02	2.07E-02	5.60E-01	4.47E-01	FALSE	FALSE	FALSE	3	1937149	2.20E-02	4.61E-01	TRUE	3	1895465	1.76E-02	0.99	6479	6.57E-06	TRUE	TRUE	482,730	20.35
Triacylglycerol (53:4) levels	Parkinson's disease	rs13288401	T	C	T	C	-2.36E-01	2.62E-02	3.15E-02	9.43E-01	FALSE	FALSE	FALSE	9	1147365	5.30E-02	2.07E-01	TRUE	9	1147365	5.24E-02	0.91	6479	6.82E-06	TRUE	TRUE	482,730	20.27
Triacylglycerol (53:4) levels	Parkinson's disease	rs138987797	T	A	T	A	7.76E-01	2.29E-01	3.39E-03	1.13E-02	TRUE	FALSE	FALSE	3	190274962	1.12E-01	2.04E-02	TRUE	3	190557173	1.59E-01	0.93	6479	1.04E-06	TRUE	TRUE	482,730	23.89
Triacylglycerol (53:4) levels	Parkinson's disease	rs139500046	C	A	C	A	-4.02E-01	-4.41E-02	1.12E-02	9.83E-01	FALSE	FALSE	FALSE	12	97244448	8.65E-02	2.15E-01	TRUE	12	96850670	8.34E-02	0.99	6479	1.52E-06	TRUE	TRUE	482,730	23.17
Triacylglycerol (53:4) levels	Parkinson's disease	rs144500033	T	A	T	A	-7.65E-01	-6.82E-02	3.12E-03	1.49E-02	TRUE	FALSE	FALSE	4	27063126	1.09E-01	2.76E-01	TRUE	4	27061504	1.70E-01	0.81	6479	7.15E-06	TRUE	TRUE	482,730	20.19
Triacylglycerol (53:4) levels	Parkinson's disease	rs17081481	G	A	G	A	8.65E-02	-2.07E-02	3.34E-01	6.94E-01	FALSE	FALSE	FALSE	13	35135179	2.61E-02	3.69E-01	TRUE	13	34561042	1.93E-02	0.90	6479	7.33E-06	TRUE	TRUE	482,730	20.14
Triacylglycerol (53:4) levels	Parkinson's disease	rs1949011	A	C	A	C	-8.71E-02	3.74E-02	4.29E-01	5.53E-01	FALSE	FALSE	FALSE	2	142499267	2.30E-02	9.83E-01	TRUE	2	141741698	1.79E-02	0.97	6479	1.19E-06	TRUE	TRUE	482,730	23.64
Triacylglycerol (53:4) levels	Parkinson's disease	rs2370511	C	T	C	T	1.16E-01	2.15E-02	1.57E-01	8.28E-01	FALSE	FALSE	FALSE	12	78783690	2.97E-02	3.29E-01	TRUE	12	78389910	2.41E-02	1.00	6479	1.44E-06	TRUE	TRUE	482,730	23.27
Triacylglycerol (53:4) levels	Parkinson's disease	rs351596	T	C	T	C	-2.62E-01	-3.40E-02	9.75E-01	6.21E-02	FALSE	FALSE	FALSE	1	4377911	4.93E-02	3.10E-01	TRUE	1	4317851	5.85E-02	0.92	6479	7.43E-06	TRUE	TRUE	482,730	20.11
Triacylglycerol (53:4) levels	Parkinson's disease	rs35832030	T	G	T	G	1.04E-01	-1.15E-02	1.96E-01	8.03E-01	FALSE	FALSE	FALSE	17	1527382	2.45E-02	1.95E-01	TRUE	17	1624088	2.26E-02	0.99	6479	4.30E-06	TRUE	TRUE	482,730	21.16
Triacylglycerol (53:4) levels	Parkinson's disease	rs3829088	C	T	C	T	9.73E-02	2.59E-02	2.03E-01	8.53E-01	FALSE	FALSE	FALSE	9	23713680	3.15E-02	3.86E-01	TRUE	9	23713682	2.20E-02	1.00	6479	9.59E-06	TRUE	TRUE	482,730	19.62
Triacylglycerol (53:4) levels	Parkinson's disease	rs4837740	G	A	G	A	-2.02E-01	-1.41E-02	9.55E-01	5.03E-02	FALSE	FALSE	FALSE	9	123018342	5.55E-02	9.69E-02	TRUE	9	120256064	4.46E-02	0.92	6479	6.30E-06	TRUE	TRUE	482,730	20.43
Triacylglycerol (53:4) levels	Parkinson's disease	rs55672000	A	G	A	G	-2.55E-01	-6.07E-02	2.74E-02	9.84E-01	FALSE	FALSE	FALSE	5	65530641	1.27E-01	1.98E-01	TRUE	5	66234813	5.68E-02	0.91	6479	7.10E-06	TRUE	TRUE	482,730	20.20
Triacylglycerol (53:4) levels	Parkinson's disease	rs62291573	C	T	C	T	1.00E-01	-2.81E-02	2.14E-01	7.47E-01	FALSE	FALSE	FALSE	4	5001376	2.75E-02	5.13E-01	TRUE	4	4999649	2.21E-02	0.94	6479	6.00E-06	TRUE	TRUE	482,730	20.52
Triacylglycerol (53:4) levels	Parkinson's disease	rs7152096	A	G	A	G	1.04E-01	9.87E-02	1.90E-01	8.62E-01	FALSE	FALSE	FALSE	14	98259535	4.33E-02	1.13E-02	TRUE	14	97793198	2.31E-02	0.94	6479	7.83E-06	TRUE	TRUE	482,730	20.01
Triacylglycerol (53:4) levels	Parkinson's disease	rs71556711	T	C	T	C	-1.41E-01	1.25E-02	9.53E-02	9.15E-01	FALSE	FALSE	FALSE	7	72854549	3.50E-02	1.42E-01	TRUE	7	73440219	3.00E-02	0.99	6479	2.40E-06	TRUE	TRUE	482,730	22.28
Triacylglycerol (53:4) levels	Parkinson's disease	rs7846649	C	T	C	T	1.26E-01	6.90E-02	1.91E-01	9.09E-01	FALSE	FALSE	FALSE	8	60001055	3.38E-02	2.06E-02	TRUE	8	59088496	2.23E-02	0.99	6479	1.58E-08	TRUE	TRUE	482,730	32.03
Triacylglycerol (53:4) levels	Parkinson's disease	rs79235240	A	C	A	C	2.18E-01	4.40E-03	3.79E-02	9.29E-01	FALSE	FALSE	FALSE	3	42857801	3.42E-02	4.66E-02	TRUE	3	42816309	4.67E-02	0.98	6479	3.14E-06	TRUE	TRUE	482,730	21.77
Triacylglycerol (53:4) levels	Parkinson's disease	rs825692	A	G	A	G	-1.39E-01	5.17E-02	1.10E-01	8.46E-01	FALSE	FALSE	FALSE	16	73590714	3.98E-02	7.13E-01	TRUE	16	73556815	2.89E-02	0.93	6479	1.65E-06	TRUE	TRUE	482,730	23.01
Triacylglycerol (53:4) levels	Parkinson's disease	rs964184	C	G	C	G	-2.74E-01	-5.68E-02	8.49E-01	8.63E-01	TRUE	FALSE	FALSE	11	116648917	2.43E-02	9.71E-03	TRUE	11	116778201	2.40E-02	1.00	6479	5.39E-30	TRUE	TRUE	482,730	130.76
Triacylglycerol (54:3) levels	Parkinson's disease	rs10105606	A	C	A	C	-1.13E-01	0.00E+00	2.79E-01	6.64E-01	FALSE	FALSE	FALSE	8	19827848	1.77E-02	4.34E-05	TRUE	8	19970337	1.85E-02	1.00	7162	9.69E-10	TRUE	TRUE	482,730	37.48
Triacylglycerol (54:3) levels	Parkinson's disease	rs10169217	G	A	G	A	-8.26E-02	-2.56E-02	6.76E-01	3.13E-01	FALSE	FALSE	FALSE	2	212875346	2.37E-02	5.51E-01	TRUE	2	212010621	1.79E-02	1.00	7162	3.95E-06	TRUE	TRUE	482,730	21.32
Triacylglycerol (54:3) levels	Parkinson's disease	rs1042034	T	C	T	C	9.46E-02	-5.30E-03	7.28E-01	2.18E-01	FALSE	FALSE	FALSE	2	21225281	2.03E-02	9.96E-02	TRUE	2	21002409	1.86E-02	1.00	7162	3.97E-07	TRUE	TRUE	482,730	25.75
Triacylglycerol (54:3) levels	Parkinson's disease	rs11068322	C	G	C	G	9.43E-02	-2.18E-02	1.92E-01	1.18E-01	TRUE	FALSE	FALSE	12	117517537	3.59E-02	2.64E-01	TRUE	12	117079732	2.11E-02	0.99	7162	7.76E-06	TRUE	TRUE	482,730	20.03
Triacylglycerol (54:3) levels	Parkinson's disease	rs112122274	G	A	G	A	-2.17E-01	4.76E-02	3.60E-02	9.49E-01	FALSE	FALSE	FALSE	9	139149840	6.87E-02	3.11E-01	TRUE	9	136257994	4.86E-02	0.85	7162	7.89E-06	TRUE	TRUE	482,730	19.99
Triacylglycerol (54:3) levels	Parkinson's disease	rs112576575	G	A	G	A	-4.08E-01	7.86E-02	9.58E-03	9.80E-01	FALSE	FALSE	FALSE	12	20885093	9.71E-02	3.79E-01	TRUE	12	20732159	9.05E-02	0.91	7162	6.74E-06	TRUE	TRUE	482,730	20.30
Triacylglycerol (54:3) levels	Parkinson's disease	rs12365864	G	A	G	A	1.16E-01	-2.95E-02	1.66E-01	7.86E-01	FALSE	FALSE	FALSE	11	116489036	2.73E-02	5.55E-01	TRUE	11	116618319	2.27E-02	0.97	7162	3.12E-07	TRUE	TRUE	482,730	26.22
Triacylglycerol (54:3) levels	Parkinson's disease	rs1260326	C	T	C	T	-9.49E-02	-6.78E-02	6.51E-01	4.20E-01	FALSE	FALSE	FALSE	2	27730940	1.72E-02	4.04E-05	TRUE	2	27508073	1.75E-02	1.00	7162	5.61E-08	TRUE	TRUE	482,730	29.55
Triacylglycerol (54:3) levels	Parkinson's disease	rs13237172	A	G	A	G	-1.72E-01	-7.84E-02	5.39E-02	9.47E-01	FALSE	FALSE	FALSE	7	155164591	5.33E-02	8.49E-01	TRUE	7	155371896	3.81E-02	0.95	7162	6.67E-06	TRUE	TRUE	482,730	20.32
Triacylglycerol (54:3) levels	Parkinson's disease	rs137871178	C	T	C	T	3.50E-01	-1.60E-03	1.73E-02	9.89E-01	FALSE	FALSE	FALSE	14	58604730	1.19E-01	4.80E-03	TRUE	14	58138012	6.60E-02	0.96	7162	1.15E-07	TRUE	TRUE	482,730	28.17
Triacylglycerol (54:3) levels	Parkinson's disease	rs141116593	G	C	G	C	1.50E-01	9.30E-02	7.40E-02	2.71E-02	TRUE	FALSE	FALSE	6	155201926	1.44E-01	2.85E-01	TRUE	6	154880792	3.21E-02	0.99	7162	3.15E-06	TRUE	TRUE	482,730	21.76
Triacylglycerol (54:3) levels	Parkinson's disease	rs144679576	C	T	C	T	-3.74E-01	-2.15E-01	1.18E-02	9.88E-01	FALSE	FALSE	FALSE	8	2753891	1.58E-01	7.63E-01	TRUE	8	2896369	8.02E-02	0.94	7162	3.18E-06	TRUE	TRUE	482,730	21.74
Triacylglycerol (54:3) levels	Parkinson's disease	rs147987331	A	T	A	T	-2.52E-01	-3.21E-02	2.27E-02	2.36E-02	TRUE	FALSE	FALSE	2	104861307	7.92E-02	1.64E-01	TRUE	2	104244849	5.66E-02	0.97	7162	8.45E-06	TRUE	TRUE	482,730	19.86
Triacylglycerol (54:3) levels	Parkinson's disease	rs17333900	T	G	T	G	1.18E-01	-6.90E-03	1.23E-01	8.93E-01	FALSE	FALSE	FALSE	10	90435280	3.11E-02	8.41E-02	TRUE	10	88675523	2.56E-02	0.99	7162	3.84E-06	TRUE	TRUE	482,730	21.38
Triacylglycerol (54:3) levels	Parkinson's disease	rs174560	C	T	C	T	1.33E-01	-1.90E-03	3.83E-01	7.01E-01	FALSE	FALSE	FALSE	11	61581764	1.84E-02	3.82E-02	TRUE	11	61814292	1.72E-02	1.00	7162	1.13E-14	TRUE	TRUE	482,730	59.91
Triacylglycerol (54:3) levels	Parkinson's disease	rs2281721	T	C	T	C	-7.78E-02	-6.20E-03	5.67E-01	3.86E-01	FALSE	FALSE	FALSE	1	230297136	1.74E-02	1.42E-01	TRUE	1	230161390	1.69E-02	1.00	7162	4.41E-06	TRUE	TRUE	482,730	21.11
Triacylglycerol (54:3) levels	Parkinson's disease	rs2642442	T	C	T	C	-1.17E-01	1.21E-02	6.93E-01	3.71E-01	FALSE	FALSE	FALSE	1	220973563	1.60E-02	3.45E-01	TRUE	1	220800221	1.82E-02	0.99	7162	1.42E-10	TRUE	TRUE	482,730	41.26
Triacylglycerol (54:3) levels	Parkinson's disease	rs2643912	T	G	T	G	-7.91E-02	1.80E-03	4.51E-01	5.72E-01	FALSE	FALSE	FALSE	1	2283842	2.24E-02	2.84E-02	TRUE	1	2352403	1.68E-02	1.00	7162	2.67E-06	TRUE	TRUE	482,730	22.08
Triacylglycerol (54:3) levels	Parkinson's disease	rs2980888	C	T	C	T	-8.76E-02	-2.33E-02	7.43E-01	3.16E-01	FALSE	FALSE	FALSE	8	126507308	1.82E-02	7.01E-01	TRUE	8	125495066	1.91E-02	1.00	7162	4.60E-06	TRUE	TRUE	482,730	21.03
Triacylglycerol (54:3) levels	Parkinson's disease	rs3812316	G	C	G	C	-1.20E-01	7.60E-03	1.21E-01	1.19E-01	TRUE	FALSE	FALSE	7	73020337	2.71E-02	1.08E-01	TRUE	7	73606007	2.55E-02	1.00	7162	2.41E-06	TRUE	TRUE	482,730	22.27
Triacylglycerol (54:3) levels	Parkinson's disease	rs3934642	G	C	G	C	-1.26E-01	3.38E-02	1.15E-01	1.55E-01	TRUE	FALSE	FALSE	4	82604736	3.27E-02	5.20E-01	TRUE	4	81683582	2.67E-02	0.96	7162	2.55E-06	TRUE	TRUE	482,730	22.16
Triacylglycerol (54:3) levels	Parkinson's disease	rs56252769	C	T	C	T	9.01E-02	-4.65E-02	2.29E-01	7.33E-01	FALSE	FALSE	FALSE	11	65718633	1.95E-02	8.55E-03	TRUE	11	65951162	2.01E-02	0.99	7162	7.50E-06	TRUE	TRUE	482,730	20.09
Triacylglycerol (54:3) levels	Parkinson's disease	rs62291572	T	C	T	C	1.10E-01	-2.71E-02	2.14E-01	7.47E-01	FALSE	FALSE	FALSE	4	4997927	2.75E-02	4.89E-01	TRUE	4	4996200	2.11E-02	0.94	7162	1.63E-07	TRUE	TRUE	482,730	27.48
Triacylglycerol (54:3) levels	Parkinson's disease	rs72836628	T	C	T	C	-8.72E-02	3.49E-02	2.97E-01	7.34E-01	FALSE	FALSE	FALSE	10	113902454	1.99E-02	3.97E-02	TRUE	10	112142696	1.82E-02	1.00	7162	1.79E-06	TRUE	TRUE	482,730	22.85
Triacylglycerol (54:3) levels	Parkinson's disease	rs7638534	T	G	T	G	7.77E-02	7.90E-03	3.49E-01	6.29E-01	FALSE	FALSE	FALSE	3	1940190	1.95E-02	1.63E-01	TRUE	3	1898506	1.74E-02	1.00	7162	8.59E-06	TRUE	TRUE	482,730	19.83
Triacylglycerol (54:3) levels	Parkinson's disease	rs76950187	G	A	G	A	1.50E-01	6.08E-02	7.45E-02	9.52E-01	FALSE	FALSE	FALSE	2	129184649	5.53E-02	5.65E-01	TRUE	2	128427075	3.20E-02	0.99	7162	2.79E-06	TRUE	TRUE	482,730	21.99
Triacylglycerol (54:3) levels	Parkinson's disease	rs78540742	A	T	A	T	2.46E-01	6.37E-02	2.38E-02	2.25E-02	TRUE	FALSE	FALSE	5	113907305	7.87E-02	3.79E-01	TRUE	5	114571608	5.52E-02	0.97	7162	8.35E-06	TRUE	TRUE	482,730	19.88
Triacylglycerol (54:3) levels	Parkinson's disease	rs9578336	G	C	G	C	9.83E-02	7.80E-03	1.82E-01	1.15E-01	TRUE	FALSE	FALSE	13	21546513	2.71E-02	1.12E-01	TRUE	13	20972374	2.17E-02	0.97	7162	5.84E-06	TRUE	TRUE	482,730	20.57
Triacylglycerol (54:3) levels	Parkinson's disease	rs964184	C	G	C	G	-2.86E-01	-5.68E-02	8.49E-01	8.63E-01	TRUE	FALSE	FALSE	11	116648917	2.43E-02	9.71E-03	TRUE	11	116778201	2.30E-02	1.00	7162	3.91E-35	TRUE	TRUE	482,730	154.60
Triacylglycerol (54:4) levels	Parkinson's disease	rs10105606	A	C	A	C	-1.24E-01	0.00E+00	2.79E-01	6.64E-01	FALSE	FALSE	FALSE	8	19827848	1.77E-02	4.34E-05	TRUE	8	19970337	1.85E-02	1.00	7165	2.21E-11	TRUE	TRUE	482,730	44.91
Triacylglycerol (54:4) levels	Parkinson's disease	rs1042034	T	C	T	C	1.02E-01	-5.30E-03	7.28E-01	2.18E-01	FALSE	FALSE	FALSE	2	21225281	2.03E-02	9.96E-02	TRUE	2	21002409	1.86E-02	1.00	7165	4.38E-08	TRUE	TRUE	482,730	30.03
Triacylglycerol (54:4) levels	Parkinson's disease	rs10773708	A	G	A	G	-8.86E-02	3.91E-02	6.83E-01	2.93E-01	FALSE	FALSE	FALSE	12	130281212	2.62E-02	8.67E-01	TRUE	12	129796667	1.81E-02	1.00	7165	9.43E-07	TRUE	TRUE	482,730	24.08
Triacylglycerol (54:4) levels	Parkinson's disease	rs10779833	G	A	G	A	-9.33E-02	6.80E-03	6.57E-01	2.62E-01	FALSE	FALSE	FALSE	1	230293037	2.60E-02	1.01E-01	TRUE	1	230157290	1.76E-02	0.97	7165	1.21E-07	TRUE	TRUE	482,730	28.05
Triacylglycerol (54:4) levels	Parkinson's disease	rs12365864	G	A	G	A	1.22E-01	-2.95E-02	1.66E-01	7.86E-01	FALSE	FALSE	FALSE	11	116489036	2.73E-02	5.55E-01	TRUE	11	116618319	2.26E-02	0.97	7165	7.80E-08	TRUE	TRUE	482,730	28.91
Triacylglycerol (54:4) levels	Parkinson's disease	rs1260326	C	T	C	T	-1.05E-01	-6.78E-02	6.51E-01	4.20E-01	FALSE	FALSE	FALSE	2	27730940	1.72E-02	4.04E-05	TRUE	2	27508073	1.74E-02	1.00	7165	1.95E-09	TRUE	TRUE	482,730	36.12
Triacylglycerol (54:4) levels	Parkinson's disease	rs137871178	C	T	C	T	3.37E-01	-1.60E-03	1.73E-02	9.89E-01	FALSE	FALSE	FALSE	14	58604730	1.19E-01	4.80E-03	TRUE	14	58138012	6.59E-02	0.96	7165	3.40E-07	TRUE	TRUE	482,730	26.05
Triacylglycerol (54:4) levels	Parkinson's disease	rs144072769	C	T	C	T	1.67E-01	-1.02E-01	6.82E-02	9.73E-01	FALSE	FALSE	FALSE	6	155237379	1.71E-01	2.60E-01	TRUE	6	154916245	3.36E-02	0.96	7165	6.72E-07	TRUE	TRUE	482,730	24.74
Triacylglycerol (54:4) levels	Parkinson's disease	rs17160706	C	A	C	A	9.82E-02	1.30E-03	1.80E-01	8.10E-01	FALSE	FALSE	FALSE	5	107228762	2.89E-02	1.62E-02	TRUE	5	107893061	2.16E-02	0.98	7165	5.77E-06	TRUE	TRUE	482,730	20.60
Triacylglycerol (54:4) levels	Parkinson's disease	rs174604	G	C	G	C	9.83E-02	-1.20E-03	3.72E-01	3.65E-01	TRUE	FALSE	FALSE	11	61626270	1.86E-02	2.30E-02	TRUE	11	61858798	1.74E-02	0.99	7165	1.71E-08	TRUE	TRUE	482,730	31.87
Triacylglycerol (54:4) levels	Parkinson's disease	rs2642442	T	C	T	C	-1.20E-01	1.21E-02	6.93E-01	3.71E-01	FALSE	FALSE	FALSE	1	220973563	1.60E-02	3.45E-01	TRUE	1	220800221	1.82E-02	0.99	7165	4.79E-11	TRUE	TRUE	482,730	43.39
Triacylglycerol (54:4) levels	Parkinson's disease	rs2643912	T	G	T	G	-7.46E-02	1.80E-03	4.51E-01	5.72E-01	FALSE	FALSE	FALSE	1	2283842	2.24E-02	2.84E-02	TRUE	1	2352403	1.68E-02	1.00	7165	9.42E-06	TRUE	TRUE	482,730	19.65
Triacylglycerol (54:4) levels	Parkinson's disease	rs2941755	C	T	C	T	-1.69E-01	2.70E-02	9.43E-01	8.43E-02	FALSE	FALSE	FALSE	2	80060995	4.13E-02	2.90E-01	TRUE	2	79833869	3.67E-02	0.97	7165	4.22E-06	TRUE	TRUE	482,730	21.19
Triacylglycerol (54:4) levels	Parkinson's disease	rs351596	T	C	T	C	-2.47E-01	-3.40E-02	9.75E-01	6.21E-02	FALSE	FALSE	FALSE	1	4377911	4.93E-02	3.10E-01	TRUE	1	4317851	5.57E-02	0.92	7165	9.46E-06	TRUE	TRUE	482,730	19.65
Triacylglycerol (54:4) levels	Parkinson's disease	rs3812316	G	C	G	C	-1.24E-01	7.60E-03	1.21E-01	1.19E-01	TRUE	FALSE	FALSE	7	73020337	2.71E-02	1.08E-01	TRUE	7	73606007	2.55E-02	1.00	7165	1.09E-06	TRUE	TRUE	482,730	23.80
Triacylglycerol (54:4) levels	Parkinson's disease	rs61702480	A	G	A	G	-8.34E-02	5.00E-04	2.75E-01	7.12E-01	FALSE	FALSE	FALSE	14	95684801	2.95E-02	6.43E-03	TRUE	14	95218464	1.87E-02	0.99	7165	8.61E-06	TRUE	TRUE	482,730	19.82
Triacylglycerol (54:4) levels	Parkinson's disease	rs62291573	C	T	C	T	9.80E-02	-2.81E-02	2.14E-01	7.47E-01	FALSE	FALSE	FALSE	4	5001376	2.75E-02	5.13E-01	TRUE	4	4999649	2.10E-02	0.94	7165	3.24E-06	TRUE	TRUE	482,730	21.70
Triacylglycerol (54:4) levels	Parkinson's disease	rs72836628	T	C	T	C	-8.98E-02	3.49E-02	2.97E-01	7.34E-01	FALSE	FALSE	FALSE	10	113902454	1.99E-02	3.97E-02	TRUE	10	112142696	1.82E-02	1.00	7165	8.55E-07	TRUE	TRUE	482,730	24.27
Triacylglycerol (54:4) levels	Parkinson's disease	rs73022136	T	C	T	C	-1.96E-01	1.67E-02	4.38E-02	9.74E-01	FALSE	FALSE	FALSE	6	165994574	8.38E-02	7.45E-02	TRUE	6	165581086	4.29E-02	0.92	7165	4.96E-06	TRUE	TRUE	482,730	20.88
Triacylglycerol (54:4) levels	Parkinson's disease	rs78433420	T	C	T	C	-2.68E-01	1.55E-02	2.10E-02	9.68E-01	FALSE	FALSE	FALSE	7	44961805	6.83E-02	8.62E-02	TRUE	7	44922206	5.91E-02	0.97	7165	5.58E-06	TRUE	TRUE	482,730	20.66
Triacylglycerol (54:4) levels	Parkinson's disease	rs964184	C	G	C	G	-3.01E-01	-5.68E-02	8.49E-01	8.63E-01	TRUE	FALSE	FALSE	11	116648917	2.43E-02	9.71E-03	TRUE	11	116778201	2.29E-02	1.00	7165	9.12E-39	TRUE	TRUE	482,730	171.60
Triacylglycerol (54:5) levels	Parkinson's disease	rs10105606	A	C	A	C	-1.16E-01	0.00E+00	2.79E-01	6.64E-01	FALSE	FALSE	FALSE	8	19827848	1.77E-02	4.34E-05	TRUE	8	19970337	1.85E-02	1.00	7146	4.47E-10	TRUE	TRUE	482,730	39.00
Triacylglycerol (54:5) levels	Parkinson's disease	rs1042034	T	C	T	C	9.21E-02	-5.30E-03	7.28E-01	2.18E-01	FALSE	FALSE	FALSE	2	21225281	2.03E-02	9.96E-02	TRUE	2	21002409	1.86E-02	1.00	7146	8.05E-07	TRUE	TRUE	482,730	24.39
Triacylglycerol (54:5) levels	Parkinson's disease	rs10773708	A	G	A	G	-8.71E-02	3.91E-02	6.83E-01	2.93E-01	FALSE	FALSE	FALSE	12	130281212	2.62E-02	8.67E-01	TRUE	12	129796667	1.81E-02	1.00	7146	1.43E-06	TRUE	TRUE	482,730	23.28
Triacylglycerol (54:5) levels	Parkinson's disease	rs12365864	G	A	G	A	1.13E-01	-2.95E-02	1.66E-01	7.86E-01	FALSE	FALSE	FALSE	11	116489036	2.73E-02	5.55E-01	TRUE	11	116618319	2.27E-02	0.97	7146	6.92E-07	TRUE	TRUE	482,730	24.68
Triacylglycerol (54:5) levels	Parkinson's disease	rs1260326	C	T	C	T	-1.22E-01	-6.78E-02	6.51E-01	4.20E-01	FALSE	FALSE	FALSE	2	27730940	1.72E-02	4.04E-05	TRUE	2	27508073	1.74E-02	1.00	7146	3.24E-12	TRUE	TRUE	482,730	48.70
Triacylglycerol (54:5) levels	Parkinson's disease	rs13178172	G	A	G	A	9.03E-02	4.23E-02	7.39E-01	3.24E-01	FALSE	FALSE	FALSE	5	117677555	2.42E-02	4.02E-02	TRUE	5	118341860	1.88E-02	1.00	7146	1.53E-06	TRUE	TRUE	482,730	23.15

Triacylglycerol (54:5) levels	Parkinson's disease	rs13246993	A	G	A	G	-1.22E-01	-9.10E-03	1.21E-01	8.81E-01	FALSE	FALSE	7	73022746	2.71E-02	1.33E-01	TRUE	7	73608416	2.55E-02	1.00	7146	1.57E-06	TRUE	TRUE	482,730	23.09
Triacylglycerol (54:5) levels	Parkinson's disease	rs137871178	C	T	C	T	2.98E-01	-1.60E-03	1.73E-02	9.89E-01	FALSE	FALSE	14	58604730	1.19E-01	4.80E-03	TRUE	14	58138012	6.61E-02	0.96	7146	6.67E-06	TRUE	TRUE	482,730	20.32
Triacylglycerol (54:5) levels	Parkinson's disease	rs139245678	T	C	T	C	-2.30E-01	-6.28E-02	3.00E-02	9.69E-01	FALSE	FALSE	8	16337302	7.08E-02	4.26E-01	TRUE	8	16479793	5.14E-02	0.91	7146	7.90E-06	TRUE	TRUE	482,730	19.99
Triacylglycerol (54:5) levels	Parkinson's disease	rs144072769	C	T	C	T	1.64E-01	-1.02E-01	6.82E-02	9.73E-01	FALSE	FALSE	6	155237379	1.71E-01	2.60E-01	TRUE	6	154916245	3.36E-02	0.96	7146	1.03E-06	TRUE	TRUE	482,730	23.92
Triacylglycerol (54:5) levels	Parkinson's disease	rs1456674	C	T	C	T	-4.51E-01	5.18E-02	8.76E-03	9.59E-01	FALSE	FALSE	3	147880828	6.10E-02	4.02E-01	TRUE	3	148163041	9.49E-02	0.89	7146	2.10E-06	TRUE	TRUE	482,730	22.54
Triacylglycerol (54:5) levels	Parkinson's disease	rs147151674	T	A	T	A	3.57E-01	-1.03E-01	1.18E-02	2.16E-02	TRUE	FALSE	6	98558213	8.54E-02	6.39E-01	TRUE	6	98110337	7.85E-02	0.95	7146	5.61E-06	TRUE	TRUE	482,730	20.65
Triacylglycerol (54:5) levels	Parkinson's disease	rs17160706	C	A	C	A	9.68E-02	1.30E-03	1.80E-01	8.10E-01	FALSE	FALSE	5	107228762	2.89E-02	1.62E-02	TRUE	5	107893061	2.17E-02	0.98	7146	8.00E-06	TRUE	TRUE	482,730	19.97
Triacylglycerol (54:5) levels	Parkinson's disease	rs17596144	T	C	T	C	1.30E-01	-2.90E-03	1.52E-01	8.43E-01	FALSE	FALSE	1	220978645	2.58E-02	4.12E-02	TRUE	1	220805303	2.34E-02	0.99	7146	2.84E-08	TRUE	TRUE	482,730	30.88
Triacylglycerol (54:5) levels	Parkinson's disease	rs2352723	C	T	C	T	-9.70E-02	-2.69E-02	7.69E-01	1.85E-01	FALSE	FALSE	1	230291868	2.61E-02	5.20E-01	TRUE	1	230156121	1.96E-02	1.00	7146	7.46E-07	TRUE	TRUE	482,730	24.53
Triacylglycerol (54:5) levels	Parkinson's disease	rs351596	T	C	T	C	-2.52E-01	-3.40E-02	9.75E-01	6.21E-02	FALSE	FALSE	1	4377911	4.93E-02	3.10E-01	TRUE	1	4317851	5.57E-02	0.92	7146	5.89E-06	TRUE	TRUE	482,730	20.55
Triacylglycerol (54:5) levels	Parkinson's disease	rs72671922	G	A	G	A	-1.50E-01	4.71E-02	6.71E-02	9.76E-01	FALSE	FALSE	14	22262835	8.19E-02	2.48E-01	TRUE	14	21794664	3.37E-02	0.98	7146	9.57E-06	TRUE	TRUE	482,730	19.62
Triacylglycerol (54:5) levels	Parkinson's disease	rs79380657	A	G	A	G	-2.61E-01	-4.80E-02	2.26E-02	9.88E-01	FALSE	FALSE	13	90010897	1.13E-01	1.73E-01	TRUE	13	89358643	5.77E-02	0.95	7146	5.97E-06	TRUE	TRUE	482,730	20.53
Triacylglycerol (54:5) levels	Parkinson's disease	rs9578336	G	C	G	C	9.72E-02	7.80E-03	1.82E-01	1.15E-01	TRUE	FALSE	13	21546513	2.71E-02	1.12E-01	TRUE	13	20972374	2.17E-02	0.97	7146	7.48E-06	TRUE	TRUE	482,730	20.09
Triacylglycerol (54:5) levels	Parkinson's disease	rs964184	C	G	C	G	-2.68E-01	-5.68E-02	8.49E-01	8.63E-01	TRUE	FALSE	11	116648917	2.43E-02	9.71E-03	TRUE	11	116778201	2.30E-02	1.00	7146	5.22E-31	TRUE	TRUE	482,730	135.36
Triacylglycerol (54:6) levels	Parkinson's disease	rs114561185	G	A	G	A	6.17E-01	9.99E-02	4.64E-03	9.81E-01	FALSE	FALSE	2	204931383	7.32E-02	7.64E-01	TRUE	2	204066660	1.29E-01	0.91	7066	1.70E-06	TRUE	TRUE	482,730	22.94
Triacylglycerol (54:6) levels	Parkinson's disease	rs12405870	T	A	T	A	-8.33E-02	2.23E-02	3.15E-01	2.60E-01	TRUE	FALSE	1	187887877	2.63E-02	4.00E-01	TRUE	1	187918746	1.82E-02	0.98	7066	5.13E-06	TRUE	TRUE	482,730	20.82
Triacylglycerol (54:6) levels	Parkinson's disease	rs1260326	C	T	C	T	-1.12E-01	-6.78E-02	6.51E-01	4.20E-01	FALSE	FALSE	2	27730940	1.72E-02	4.04E-05	TRUE	2	27508073	1.76E-02	1.00	7066	1.83E-10	TRUE	TRUE	482,730	40.76
Triacylglycerol (54:6) levels	Parkinson's disease	rs13178172	G	A	G	A	9.14E-02	4.23E-02	7.39E-01	3.24E-01	FALSE	FALSE	5	117677555	2.42E-02	4.02E-02	TRUE	5	118341860	1.89E-02	1.00	7066	1.30E-06	TRUE	TRUE	482,730	23.47
Triacylglycerol (54:6) levels	Parkinson's disease	rs140647506	A	G	A	G	4.67E-01	1.00E-01	7.05E-03	9.87E-01	FALSE	FALSE	9	31005161	1.47E-01	3.05E-01	TRUE	9	31005163	1.03E-01	0.94	7066	5.83E-06	TRUE	TRUE	482,730	20.57
Triacylglycerol (54:6) levels	Parkinson's disease	rs1456674	C	T	C	T	-4.37E-01	5.18E-02	8.76E-03	9.59E-01	FALSE	FALSE	3	147880828	6.10E-02	4.02E-01	TRUE	3	148163041	9.58E-02	0.89	7066	5.16E-06	TRUE	TRUE	482,730	20.81
Triacylglycerol (54:6) levels	Parkinson's disease	rs174554	G	A	G	A	-9.83E-02	-3.50E-03	4.06E-01	6.73E-01	FALSE	FALSE	11	61579463	1.79E-02	7.26E-02	TRUE	11	61811991	1.71E-02	1.00	7066	9.75E-09	TRUE	TRUE	482,730	32.97
Triacylglycerol (54:6) levels	Parkinson's disease	rs17596144	T	C	T	C	1.10E-01	-2.90E-03	1.52E-01	8.43E-01	FALSE	FALSE	1	220978645	2.58E-02	4.12E-02	TRUE	1	220805303	2.36E-02	0.99	7066	3.22E-06	TRUE	TRUE	482,730	21.72
Triacylglycerol (54:6) levels	Parkinson's disease	rs181609235	G	A	G	A	-3.51E-01	1.21E-02	1.23E-02	9.85E-01	FALSE	FALSE	6	165038123	9.66E-02	4.55E-02	TRUE	6	164617090	7.79E-02	0.97	7066	6.54E-06	TRUE	TRUE	482,730	20.35
Triacylglycerol (54:6) levels	Parkinson's disease	rs190411877	C	T	C	T	4.32E-01	5.44E-02	9.45E-03	9.93E-01	FALSE	FALSE	10	7913102	2.09E-01	9.96E-02	TRUE	10	7871139	8.86E-02	0.94	7066	1.08E-06	TRUE	TRUE	482,730	23.82
Triacylglycerol (54:6) levels	Parkinson's disease	rs268	G	A	G	A	2.94E-01	1.59E-02	2.27E-02	9.83E-01	FALSE	FALSE	8	19813529	6.69E-02	9.03E-02	TRUE	8	19956018	5.74E-02	0.97	7066	3.09E-07	TRUE	TRUE	482,730	26.24
Triacylglycerol (54:6) levels	Parkinson's disease	rs4057791	C	A	C	A	8.08E-02	-8.90E-03	5.46E-01	3.62E-01	FALSE	FALSE	4	164225132	2.07E-02	1.76E-01	TRUE	4	163303980	1.69E-02	1.00	7066	1.72E-06	TRUE	TRUE	482,730	22.93
Triacylglycerol (54:6) levels	Parkinson's disease	rs417237	T	G	T	G	7.58E-02	1.73E-02	5.90E-01	4.00E-01	FALSE	FALSE	1	228532195	1.71E-02	5.04E-01	TRUE	1	228344494	1.69E-02	1.00	7066	7.77E-06	TRUE	TRUE	482,730	20.02
Triacylglycerol (54:6) levels	Parkinson's disease	rs6501273	T	C	T	C	9.55E-02	-2.00E-04	7.93E-01	1.92E-01	FALSE	FALSE	17	76962364	2.92E-02	2.70E-03	TRUE	17	78966282	2.10E-02	0.98	7066	5.34E-06	TRUE	TRUE	482,730	20.74
Triacylglycerol (54:6) levels	Parkinson's disease	rs9344829	T	C	T	C	7.75E-02	-2.70E-03	3.73E-01	6.88E-01	FALSE	FALSE	6	64887066	1.90E-02	5.16E-02	TRUE	6	64177173	1.73E-02	0.99	7066	8.10E-06	TRUE	TRUE	482,730	19.94
Triacylglycerol (54:6) levels	Parkinson's disease	rs964184	C	G	C	G	-2.18E-01	-5.68E-02	8.49E-01	8.63E-01	TRUE	FALSE	11	116648917	2.43E-02	9.71E-03	TRUE	11	116778201	2.32E-02	1.00	7066	6.27E-21	TRUE	TRUE	482,730	88.64
Triacylglycerol (54:7) levels	Parkinson's disease	rs111853669	A	G	A	G	-1.58E-01	1.50E-02	7.08E-02	9.38E-01	FALSE	FALSE	1	231095407	4.76E-02	1.24E-01	TRUE	1	230959661	3.35E-02	0.98	6828	2.58E-06	TRUE	TRUE	482,730	22.14
Triacylglycerol (54:7) levels	Parkinson's disease	rs12120498	C	T	C	T	9.39E-02	-1.07E-02	6.97E-01	3.00E-01	FALSE	FALSE	1	246163381	2.46E-02	1.79E-01	TRUE	1	246000079	1.85E-02	0.99	6828	4.10E-07	TRUE	TRUE	482,730	25.69
Triacylglycerol (54:7) levels	Parkinson's disease	rs12210453	G	A	G	A	-1.88E-01	7.85E-02	4.68E-02	9.29E-01	FALSE	FALSE	6	130058445	3.93E-02	2.29E-02	TRUE	6	129737300	4.19E-02	0.94	6828	7.46E-06	TRUE	TRUE	482,730	20.10
Triacylglycerol (54:7) levels	Parkinson's disease	rs1260326	C	T	C	T	-1.03E-01	-6.78E-02	6.51E-01	4.20E-01	FALSE	FALSE	2	27730940	1.72E-02	4.04E-05	TRUE	2	27508073	1.78E-02	1.00	6828	8.72E-09	TRUE	TRUE	482,730	33.19
Triacylglycerol (54:7) levels	Parkinson's disease	rs138383428	T	G	T	G	-5.85E-01	1.58E-01	4.53E-03	9.78E-01	FALSE	FALSE	4	185931633	7.85E-02	2.21E-02	TRUE	4	185010479	1.31E-01	0.87	6828	8.67E-06	TRUE	TRUE	482,730	19.81
Triacylglycerol (54:7) levels	Parkinson's disease	rs140647506	A	G	A	G	4.64E-01	1.00E-01	7.05E-03	9.87E-01	FALSE	FALSE	9	31005161	1.47E-01	3.05E-01	TRUE	9	31005163	1.03E-01	0.94	6828	6.41E-06	TRUE	TRUE	482,730	20.39
Triacylglycerol (54:7) levels	Parkinson's disease	rs174584	A	G	A	G	-1.26E-01	-8.90E-03	4.10E-01	6.56E-01	FALSE	FALSE	11	61610750	1.77E-02	2.10E-01	TRUE	11	61843278	1.74E-02	1.00	6828	5.09E-13	TRUE	TRUE	482,730	52.37
Triacylglycerol (54:7) levels	Parkinson's disease	rs184609620	T	C	T	C	-3.95E-01	-4.40E-02	9.13E-03	9.88E-01	FALSE	FALSE	10	96146119	1.16E-01	1.53E-01	TRUE	10	94386362	8.86E-02	1.00	6828	8.31E-06	TRUE	TRUE	482,730	19.89
Triacylglycerol (54:7) levels	Parkinson's disease	rs190411877	C	T	C	T	4.23E-01	5.44E-02	9.45E-03	9.93E-01	FALSE	FALSE	10	7913102	2.09E-01	9.96E-02	TRUE	10	7871139	8.88E-02	0.94	6828	1.91E-06	TRUE	TRUE	482,730	22.72
Triacylglycerol (54:7) levels	Parkinson's disease	rs2192081	A	G	A	G	8.94E-02	-8.30E-03	2.69E-01	7.43E-01	FALSE	FALSE	10	59683005	2.56E-02	1.27E-01	TRUE	10	57923245	1.94E-02	1.00	6828	4.07E-06	TRUE	TRUE	482,730	21.26
Triacylglycerol (54:7) levels	Parkinson's disease	rs34942551	G	T	G	T	-9.16E-02	-2.20E-03	2.53E-01	7.30E-01	FALSE	FALSE	8	19870015	1.95E-02	4.04E-02	TRUE	8	20012504	1.96E-02	1.00	6828	3.11E-06	TRUE	TRUE	482,730	21.78
Triacylglycerol (54:7) levels	Parkinson's disease	rs35986054	C	T	C	T	9.73E-02	-1.07E-02	1.96E-01	8.03E-01	FALSE	FALSE	17	1526343	2.45E-02	1.79E-01	TRUE	17	1623049	2.19E-02	0.98	6828	9.38E-06	TRUE	TRUE	482,730	19.66
Triacylglycerol (54:7) levels	Parkinson's disease	rs417237	T	G	T	G	8.06E-02	1.73E-02	5.90E-01	4.00E-01	FALSE	FALSE	1	228532195	1.71E-02	5.04E-01	TRUE	1	228344494	1.72E-02	1.00	6828	3.02E-06	TRUE	TRUE	482,730	21.84
Triacylglycerol (54:7) levels	Parkinson's disease	rs4858271	C	T	C	T	-7.86E-02	5.80E-03	4.24E-01	4.96E-01	FALSE	FALSE	3	20856976	2.25E-02	9.83E-02	TRUE	3	20815484	1.74E-02	1.00	6828	6.08E-06	TRUE	TRUE	482,730	20.49
Triacylglycerol (54:7) levels	Parkinson's disease	rs56870907	C	G	C	G	1.22E-01	-1.81E-02	1.35E-01	1.30E-01	TRUE	FALSE	14	104756421	3.63E-02	2.09E-01	TRUE	14	104290084	2.63E-02	0.90	6828	3.61E-06	TRUE	TRUE	482,730	21.49
Triacylglycerol (54:7) levels	Parkinson's disease	rs62534834	G	A	G	A	-1.67E-01	-3.50E-03	6.05E-02	9.39E-01	FALSE	FALSE	9	1496121	4.87E-02	2.59E-02	TRUE	9	1496121	3.61E-02	0.98	6828	3.72E-06	TRUE	TRUE	482,730	21.44
Triacylglycerol (54:7) levels	Parkinson's disease	rs73192177	T	C	T	C	-3.19E-01	3.92E-02	1.52E-02	9.75E-01	FALSE	FALSE	3	122339936	6.44E-02	2.66E-01	TRUE	3	122621089	7.01E-02	0.96	6828	5.59E-06	TRUE	TRUE	482,730	20.65
Triacylglycerol (54:7) levels	Parkinson's disease	rs73660137	G	C	G	C	-3.65E-01	1.30E-01	1.05E-02	1.07E-02	TRUE	FALSE	8	5428512	1.13E-01	6.02E-01	TRUE	8	5570990	8.26E-02	0.99	6828	9.79E-06	TRUE	TRUE	482,730	19.58
Triacylglycerol (54:7) levels	Parkinson's disease	rs74516326	T	C	T	C	-9.78E-02	2.24E-02	1.96E-01	8.62E-01	FALSE	FALSE	7	70758150	4.14E-02	2.30E-01	TRUE	7	71293164	2.17E-02	0.98	6828	6.58E-06	TRUE	TRUE	482,730	20.34
Triacylglycerol (54:7) levels	Parkinson's disease	rs76481684	G	A	G	A	2.65E-01	8.58E-02	2.35E-02	9.86E-01	FALSE	FALSE	1	217491988	1.07E-01	3.75E-01	TRUE	1	217318646	5.68E-02	0.98	6828	3.18E-06	TRUE	TRUE	482,730	21.74
Triacylglycerol (54:7) levels	Parkinson's disease	rs964184	C	G	C	G	-1.74E-01	-5.68E-02	8.49E-01	8.63E-01	TRUE	FALSE	11	116648917	2.43E-02	9.71E-03	TRUE	11	116778201	2.36E-02	1.00	6828	1.70E-13	TRUE	TRUE	482,730	54.54
Triacylglycerol (54:7) levels	Parkinson's disease	rs9645690	T	C	T	C	-8.51E-02	1.85E-02	3.22E-01	6.77E-01	FALSE	FALSE	11	62206288	2.33E-02	3.70E-01	TRUE	11	62438816	1.81E-02	1.00	6828	2.74E-06	TRUE	TRUE	482,730	22.03
Triacylglycerol (56:3) levels	Parkinson's disease	rs10058653	T	G	T	G	-8.88E-02	1.91E-02	3.02E-01	8.11E-01	FALSE	FALSE	5	108938251	2.46E-02	3.58E-01	TRUE	5	109602550	1.94E-02	1.00	6172	4.77E-06	TRUE	TRUE	482,730	20.96
Triacylglycerol (56:3) levels	Parkinson's disease	rs10169217	G	A	G	A	-9.92E-02	-2.56E-02	6.76E-01	3.13E-01	FALSE	FALSE	2	212875346	2.37E-02	5.51E-01	TRUE	2	212010621	1.92E-02	1.00	6172	2.63E-07	TRUE	TRUE	482,730	26.56
Triacylglycerol (56:3) levels	Parkinson's disease	rs111568723	G	A	G	A	2.87E-01	-1.20E-01	2.59E-02	9.47E-01	FALSE	FALSE	9	135345889	5.14E-02	9.73E-03	TRUE	9	132470502	5.88E-02	0.95	6172	1.05E-06	TRUE	TRUE	482,730	23.87
Triacylglycerol (56:3) levels	Parkinson's disease	rs11399	T	C	T	C	1.46E-01	-4.71E-02	1.00E-01	8.84E-01	FALSE	FALSE	1	28212806	3.38E-02	7.86E-01	TRUE	1	27886295	3.01E-02	0.99	6172	1.19E-06	TRUE	TRUE	482,730	23.64
Triacylglycerol (56:3) levels	Parkinson's disease	rs116846412	A	G	A	G	-2.44E-01	3.57E-02	3.22E-02	9.56E-01	FALSE	FALSE	11	78777255	5.88E-02	2.65E-01	TRUE	11	79066210	5.29E-02	0.91	6172	3.92E-06	TRUE	TRUE	482,730	21.34
Triacylglycerol (56:3) levels	Parkinson's disease	rs117786044	A	G	A	G	-2.38E-01	2.03E-01	3.38E-02	9.63E-01	FALSE	FALSE	11	128626911	6.85E-02	1.52E-03	TRUE	11	128757016	5.09E-02	0.95	6172	2.83E-06	TRUE	TRUE	482,730	21.97
Triacylglycerol (56:3) levels	Parkinson's disease	rs117819583	A	G	A	G	-2.51E-01	-4.70E-02	2.88E-02	9.86E-01	FALSE	FALSE	16	23278676	1.26E-01	1.49E-01	TRUE	16	23267355	5.51E-02	0.92	6172	5.16E-06	TRUE	TRUE	482,730	20.81
Triacylglycerol (56:3) levels	Parkinson's disease	rs145737110	T	C	T	C	3.87E-01	5.98E-02	1.43E-02	9.80E-01	FALSE	FALSE	9	125736014	1.04E-01	2.47E-01	TRUE	9	122973735	8.45E-02	0.86	6172	4.77E-06	TRUE	TRUE	482,730	20.97
Triacylglycerol (56:3) levels	Parkinson's disease	rs149199066	A	G	A	G	2.08E-01	1.33E-01	4.44E-02	9.83E-01	FALSE	FALSE	11	65631203	8.50E-02	9.30E-01	TRUE	11	65863732	4.44E-02	0.98	6172	2.92E-06	TRUE	TRUE	482,730	21.91
Triacylglycerol (56:3) levels	Parkinson's disease	rs1539733	C	T	C	T	9.84E-02	-8.40E-03	7.94E-01	2.67E-01	FALSE	FALSE	1	162690246	2.53E-02	1.31E-01	TRUE	1	162720456	2.20E-02	0.99	6172	7.49E-06	TRUE	TRUE	482,730	20.10
Triacylglycerol (56:3) levels	Parkinson's disease	rs174560	C	T	C	T	1.70E-01	-1.90E-03	3.83E-01	7.01E-01	FALSE	FALSE	11	61581764	1.84E-02	3.82E-02	TRUE	11	61814292	1.84E-02	1.00	6172	3.95E-20	TRUE	TRUE	482,730	85.03
Triacylglycerol (56:3) levels	Parkinson's disease	rs17596144	T	C	T	C	1.62E-01	-2.90E-03	1.52E-01	8.43E-01	FALSE	FALSE	1	220978645	2.58E-02	4.12E-02	TRUE	1	220805303	2.51E-02	0.99	6172	1.11E-10	TRUE	TRUE	482,730	41.76
Triacylglycerol (56:3) levels	Parkinson's disease	rs181023072	A	C	A	C	-8.50E-01	-1.85E-01	2.49E-03	9.85E-01	FALSE	FALSE	16	63016233	9.63E-02	2.71E-02	TRUE	16	62982329	1.78E-01	0.97	6172	1.72E-06	TRUE	TRUE	482,730	22.93
Triacylglycerol (56:3) levels	Parkinson's disease	rs2980862	C	G	C	G	-1.01E-01	1.75E-02	7.43E-01	6.83E-01	TRUE	FALSE	8	126484638	1.81E-02	4.78E-01	TRUE	8	125472396	2.06E-02	1.00	6172	8.68E-07	TRUE	TRUE	482,730	24.25
Triacylglycerol (56:3) levels	Parkinson's disease	rs4908327	T	C	T	C	-1.27E-01	-2.83E-02	8.66E-01	1.09E-01	FALSE	FALSE	1	99993077	3.65E-02	3.58E-01	TRUE	1	99527521	2.67E-02	0.98	6172	2.03E-06	TRUE	TRUE	482,730	22.61
Triacylglycerol (56:3) levels	Parkinson's disease	rs58355654	A	G	A	G	1.19E-01	1.91E-02	1.29E-01	9.07E-01	FALSE	FALSE	13	23457820	3.92E-02	2.03E-01	TRUE	13	22883681	2.70E-02	0.99	6172	9.76E-06	TRUE	TRUE	482,730	19.59
Triacylglycerol (56:3) levels	Parkinson's disease	rs62016118	C	A	C	A	-1.85E-01	6.00E-03	5.08E-02	9.41E-01	FALSE	FALSE	16	6841692	4.90E-02	4.43E-02	TRUE	16	6791691	4.19E-02	0.97	6172	9.92E-06	TRUE	TRUE	482,730	19.56
Triacylglycerol (56:3) levels	Parkinson's disease	rs62291572	T	C	T	C	1.08E-01	-2.71E-02	2.14E-01	7.47E-01	FALSE	FALSE	4	4997927	2.75E-02	4.89E-01	TRUE	4	4996200	2.25E-02	0.94	6172	1.86E-06	TRUE	TRUE	482,730	22.77
Triacylglycerol (56:3) levels	Parkinson's disease	rs75008187	A	G	A	G	1.36E-01	1.58E-02	1.28E-01	8.85E-01	FALSE	FALSE	6	65151347	3.11E-02	2.14E-01	TRUE	6	64441454	2.72E-02	1.00	6172	6.10E-07	TRUE	TRUE	482,730	24.93
Triacylglycerol (56:3) levels	Parkinson's disease	rs964184	C	G	C	G	-2.38E-01	-5.68E-02	8.49E-01	8.63E-01	TRUE	FALSE	11	116648917	2.43E-02	9.71E-03	TRUE	11	116778201	2.45E-02	1.00	6172	4.19E-22	TRUE	TRUE	482,730	94.15
Triacylglycerol (56:3) levels	Parkinson's disease	rs9851701	A	G	A	G	1.19E-01	-2.75E-02	1.48E-01	8.77E-01	FALSE	FALSE	3	103001109	3.61E-02	3.51E-01	TRUE	3	103282265	2.57E-02	0.97	6172	3.58E-06	TRUE	TRUE	482,730	21.51
Triacylglycerol (56:4) levels	Parkinson's disease	rs10169217	G	A	G	A	-9.49E-02	-2.56E-02	6.76E-01	3.13E-01	FALSE	FALSE	2	212875346	2.37E-02	5.51E-01	TRUE	2	212010621	1.97E-02	1.00	5820	1.53E-06	TRUE	TRUE	482,730	23.15
Triacylglycerol (56:4) levels	Parkinson's disease	rs10430989	A	G	A	G	-1.16E-01	7.44E-02	2.02E-01	8.90E-01	FALSE	FALSE	11	43564232	3.78E-02	2.45E-02	TRUE	11	43542682	2.34E-02	0.97	5820	6.63E-07	TRUE	TRUE	482,730	24.77
Triacylglycerol (56:4) levels	Parkinson's disease	rs10966037	T	A	T	A	1.09E-01	-2.61E-02	2.02E-01	1.47E-01	TRUE	FALSE	9	23714475	3.15E-02	3.89E-01	TRUE	9	23714477	2.30E-02	1.00	5820	1.94E-06	TRUE	TRUE	482,730	22.70
Triacylglycerol (56:4) levels	Parkinson's disease	rs117883640	G	T	G	T	2.03E-01	-2.05E-01	5.16E-02	9.79E-01	FALSE	FALSE	8	57833816	1.51E-01	7.57E-01	TRUE	8	56921257	4.20E-02	0.96	5820	1.35E-06	TRUE	TRUE	482,730	23.40
Triacylglycerol (56:4) levels	Parkinson's disease	rs12448223	C	T	C	T	8.17E-02	1.20E-02	4.43E-01	5.34E-01	FALSE	FALSE	16	4154551	2.19E-02	2.35E-01	TRUE	16	4104550	1.85E-02	1.00	5820	1.00E-05	TRUE	TRUE	482,730	19.54
Triacylglycerol (56:4) levels	Parkinson's disease	rs141116593	G	C	G	C	1.61E-01	9.30E-02	7.40E-02	2.71E-02	TRUE	FALSE	6	155201926	1.44E-01	2.85E-01	TRUE	6	154880792	3.52E-02	0.99	5820	4.83E-06	TRUE	TRUE	482,730	20.94
Triacylglycerol (56:4) levels	Parkinson's disease	rs174592	G	A	G	A	1.36E-01	-6.80E-03	4.18E-01	6.42E-01	FALSE	FALSE	11	61618608	1.78E-02	1.54E-01	TRUE	11	61851136	1.87E-02	1.00	5820	4.89E-13	TRUE	TRUE	482,730	52.48
Triacylglycerol (56:4) levels	Parkinson's disease	rs2978946	G	T	G	T	8.54E-02	-2.20E-03	5.51E-01	5.27E-01	FALSE	FALSE	8	6623598	2.80E-02	2.75E-02	TRUE	8	6766077	1.90E-02	0.95	5820	7.07E-06	TRUE	TRUE	482,730	20.21
Triacylglycerol (56:4) levels	Parkinson's disease	rs36094040	C	T	C	T	-9.27E-02	-2.90E-03	3.48E-01	5.96E-01	FALSE	FALSE	12	72824963	2.33E-02	4.53E-02	TRUE	12	72431183	1.97E-02	0.99	5820	2.67E-06	TRUE	TRUE	482,730	22.08
Triacylglycerol (56:4) levels	Parkinson's disease	rs61942065	T	C	T	C	1.09E-01	-1.88E-02	2.16E-01	8.22E-01	FALSE	FALSE	12	130279271	2.90E-02	2.87E-01	TRUE	12	129794726	2.24E-02	1.00	5820	1.15E-06	TRUE	TRUE	482,730	23.71
Triacylglycerol (56:4) levels	Parkinson's disease	rs62291572	T	C	T	C	1.10E-01	-2.71E-02	2.14E-01	7.47E-01	FALSE	FALSE	4	4997927	2.75E-02	4.89E-01	TRUE	4	4996200	2.32E-02	0.94	5820	2.19E-06	TRUE	TRUE	482,730	22.46
Triacylglycerol (56:4) levels	Parkinson's disease	rs62475137	A	G	A	G	1.92E-01	-5.80E-03	5.21E-02	9.22E-01	FALSE	FALSE	7	115333007	5.07E-02	4.12E-02	TRUE	7	115692953	4.35E-02	0.90	5820	9.80E-06	TRUE	TRUE	482,730	19.58
Triacylglycerol (56:4) levels	Parkinson's disease	rs75533654	C	A	C	A	-3.36E-01	-4.27E-02	1.54E-02	9.70E-01	FALSE	FALSE	4	143096232	7.29E-02	2.54E-01	TRUE	4	142175079	7.61E-02	0.93	5820	9.90E-06	TRUE	TRUE	482,730	19.56
Triacylglycerol (56:4) levels	Parkinson's disease	rs964184	C	G	C	G	-2.42E-01	-5.68E-02	8.49E-01	8.63E-01	TRUE	FALSE	11	116648917	2.43E-02	9.71E-03	TRUE	11	116778201	2.53E-02	1.00	5820	2.14E-21	TRUE	TRUE	482,730	90.91
Triacylglycerol (56:5) levels	Parkinson's disease	rs10169217	G	A	G	A	-1.04E-01	-2.56E-02	6.76E-01	3.13E-01	FALSE	FALSE	2	212875346	2.37E-02	5.51E-01	TRUE	2	212010621	2.03E-02	1.00	5490	3.51E-07	TRUE	TRUE	482,730	26.01
Triacylglycerol (56:5) levels	Parkinson's disease	rs11623520	A	G	A	G	-9.21E-02	1.47E-02	6.50E-01	2.95E-01	FALSE	FALSE	14	95749101	2.48E-02	2.58E-01	TRUE	14	95282764	2.00E-02	1.00	5490	4.02E-06	TRUE	TRUE	482,730	21.30
Triacylglycerol (56:5) levels	Parkinson's disease	rs12193589	A	G	A	G	9.08E-02	-6.00E-03	4.50E-01	5.16E-01	FALSE	FALSE	6	164360807	2.88E-02	7.89E-02	TRUE	6	163939775	2.02E-02	0.90	5490	7.08E-06	TRUE	TRUE	482,730	20.21
Triacylglycerol (56:5) levels	Parkinson's disease	rs1441764	T	C	T	C	-1.33E-01	-7.00E-03	2.80E-01	6.65E-01	FALSE	FALSE	8	19863024	1.82E-02	1.56E-01	TRUE	8	20005513	2.12E-02	1.00	5490	3.60E-10	TRUE	TRUE	482,730	39.46
Triacylglycerol (56:5) levels	Parkinson's disease	rs1472230	A	G	A	G	8.78E-02	-3.64E-02	4.22E-01	4.91E-01	FALSE	FALSE	11	4675056	1.79E-02	2.10E-02	TRUE	11	4653826	1.95E-02	1.00	5490	6.60E-06	TRUE	TRUE	482,730	20.34
Triacylglycerol (56:5) levels	Parkinson's disease	rs149473233	A	G	A	G	6.99E-01	1.19E-01	5.17E-03	9.84E-01	FALSE	FALSE	3	6221580	9.64E-02	6.61E-01	TRUE	3	6179893	1.49E-01	0.89	5490	2.64E-06	TRUE	TRUE	482,730	22.10
Triacylglycerol (56:5) levels	Parkinson's disease	rs182009884	C	G	C	G	-2.37E-01	-1.53E-01	3.52E-02	1.36E-02	TRUE	FALSE	1	182692823	1.80E-01	4.03E-01	TRUE	1	182723688	5.19E-02	0.97	5490	4.98E-06	TRUE	TRUE	482,730	20.88
Triacylglycerol (56:5) levels	Parkinson's disease	rs34527930	T	C	T	C	-1.27E-01	1.60E-02	1.38E-01	8.27E-01	FALSE	FALSE	9	117026296	2.79E-02	2.48E-01	TRUE	9	114264016	2.79E-02	0.99	5490	5.54E-06	TRUE	TRUE	482,730	20.68
Triacylglycerol (56:5) levels	Parkinson's disease	rs4073968	T	C	T	C	-1.02E-01	-2.57E-02	2.34E-01	7.26E-01	FALSE	FALSE	4	38491835	2.64E-02	4.82E-01	TRUE	4	38490214	2.31E-02	0.96	5490	9.99E-06	TRUE	TRUE	482,730	19.55
Triacylglycerol (56:5) levels	Parkinson's disease	rs60102597	C	T	C	T	1.21E-01	-6.15E-02	1.54E-01	7.84E-01	FALSE	FALSE	17	76151697	2.42E-02	5.52E-03	TRUE	17	78155616	2.68E-02	0.95	5490	6.74E-06	TRUE	TRUE	482,730	20.31
Triacylglycerol (56:5) levels	Parkinson's disease	rs61960432	G	A	G	A	-1.51E-01	-1.02E-02	1.15E-01	9.01E-01	FALSE	FALSE	13	84556360	3.81E-02	1.03E-01	TRUE	13	83982225	3.05E-02	0.97	5490	8.22E-07	TRUE	TRUE	482,730	24.36
Triacylglycerol (56:5) levels	Parkinson's disease	rs73125564	T	A	T	A	1.05E-01	1.64E-02	2.78E-01	1.54E-01	TRUE	FALSE	4	32592630	3.35E-02	2.05E-01	TRUE	4	32591008	2.24E-02	0.90	5490	2.68E-06	TRUE	TRUE	482,730	22.08
Triacylglycerol (56:5) levels	Parkinson's disease	rs75768973	T	C	T	C	-3.25E-01	-8.54E-02	1.78E-02	9.69E-01	FALSE	FALSE	6	110099385	5.25E-02	9.84E-01	TRUE	6	109778182	7.35E-02	0.99	5490	9.80E-06	TRUE	TRUE	482,730	19.58
Triacylglycerol (56:5) levels	Parkinson's disease	rs78081886	A	G	A	G	3.58E-01	7.30E-03	1.54E-02	9.80E-01	FALSE	FALSE	4	27696057	9.56E-02	2.72E-02	TRUE	4	27694435	7.72E-02	0.96	5490	3.52E-06	TRUE	TRUE	482,730	21.56
Triacylglycerol (56:5) levels	Parkinson's disease	rs964184	C	G	C	G	-2.10E-01	-5.68E-02	8.49E-01	8.63E-01	TRUE	FALSE	11	116648917	2.43E-02	9.71E-03	TRUE	11	116778201	2.58E-02	1.00	5490	4.58E-16	TRUE	TRUE	482,730	66.37
Triacylglycerol (56:5) levels	Parkinson's disease	rs97384	C	T	C	T	1.54E-01	7.50E-03	5.80E-01	3.66E-01	FALSE	FALSE	11	61624181	1.98E-02	1.53E-01	TRUE	11	61856709	1.95E-02	0.99	5490	3.45E-15	TRUE	TRUE	482,730	62.34
Triacylglycerol (56:5) levels	Parkinson's disease	rs9997979	T	C	T	C	2.85E-01	-1.88E-02	2.45E-02	9.52E-01	FALSE	FALSE	4	100445359	4.35E-02	1.76E-01	TRUE	4	99524202	6.21E-02	0.98	5490	4.44E-06	TRUE	TRUE	482,730	21.11
Triacylglycerol (56:6) levels	Parkinson's disease	rs10105606	A	C	A	C	-1.30E-01	0.00E+00	2.79E-01	6.64E-01	FALSE	FALSE	8	19827848	1.77E-02	4.34E-05	TRUE	8	19970337	1.85E-02	1.00	7151	2.19E-12	TRUE	TRUE	482,730	49.48
Triacylglycerol (56:6) levels	Parkinson's disease	rs10739560	C	T	C	T	-2.19E-01	-1.11E-02	9.62E-01	3.81E-02	FALSE	FALSE	9	123030844	6.20E-02	6.63E-02	TRUE	9	120268566	4.61E-02	0.92	7151	2.07E-06	TRUE	TRUE	482,730	22.57
Triacylglycerol (56:6) levels	Parkinson's disease	rs1089588	G	T	G	T	8.33E-02	3.26E-02	2.64E-01	6.89E-01	FALSE	FALSE	7	86292935	2.13E-02	8.99E-01	TRUE	7	86663619	1.88E-02	1.00	7151	9.81E-06	TRUE	TRUE	482,730	19.57
Triacylglycerol (56:6) levels	Parkinson's disease	rs114971431	G	A	G	A	3.48E-01	-1.78E-02	1.27E-02	9.82E-01	FALSE	FALSE	2	56756418	9.34E-02	7.12E-02	TRUE	2	56529283	7.58E-02	0.97	7151	4.45E-06	TRUE	TRUE	482,730	21.09
Triacylglycerol (56:6) levels	Parkinson's disease	rs115007623	C	T	C	T	4.02E-01	-4.98E-02	9.91E-03	9.71E-01	FALSE	FALSE	6	44016412	5.91E-02	3.99E-01	TRUE	6	44048675	8.88E-02	0.90	7151	5.98E-06	TRUE	TRUE	482,730	20.52
Triacylglycerol (56:6) levels	Parkinson's disease	rs1260326	C	T	C	T	-1.18E-01	-6.78E-02	6.51E-01	4.20E-01	FALSE	FALSE	2	27730940	1.72E-02	4.04E-05	TRUE	2	27508073	1.74E-02	1.00	7151	1.60E-11	TRUE	TRUE	482,730	45.55
Triacylglycerol (56:6) levels	Parkinson's disease	rs174533	A	G	A	G	-2.33E-01	-9.00E-04	4.09E-01	6.69E-01	FALSE	FALSE	11	61549025	1.79E-02	1.84E-02	TRUE	11	61781553	1.68E-02	1.00	7151	8.17E-43	TRUE	TRUE	482,730	190.64
Triacylglycerol (56:6) levels	Parkinson's disease	rs17596144	T	C	T	C	1.11E-01	-2.90E-03	1.52E-01	8.43E-01	FALSE	FALSE	1	220978645	2.58E-02	4.12E-02	TRUE	1	220805303	2.34E-02	0.99	7151	2.05E-06	TRUE	TRUE	482,730	22.58
Triacylglycerol (56:6) levels	Parkinson's disease	rs187032204	C	T	C	T	2.80E-01	-9.41E-02	1.90E-02	9.83E-01	FALSE	FALSE	16	82461568	8.99E-02	5.30E-01	TRUE	16	82427963	6.20E-02	0.97	7151	6.51E-06	TRUE	TRUE	482,730	20.36
Triacylglycerol (56:6) levels	Parkinson's disease	rs187665388	T	C	T	C	-2.48E-01	2.57E-02	2.74E-02	9.76E-01	FALSE	FALSE	3	176044593	8.24E-02	1.22E-01	TRUE	3	176326805	5.20E-02	0.97	7151	1.93E-06	TRUE	TRUE	482,730	22.70
Triacylglycerol (56:6) levels	Parkinson's disease	rs197730	G	A	G	A	-8.66E-02	-1.51E-02	4.02E-01	5.40E-01	FALSE	FALSE	3	37496725	1.88E-02	3.73E-01	TRUE	3	37455234	1.70E-02	1.00	7151	3.38E-07	TRUE	TRUE	482,730	26.07
Triacylglycerol (56:6) levels	Parkinson's disease	rs2954021	G	A	G	A	-8.04E-02	-1.31E-02	5.38E-01	4.96E-01	FALSE	FALSE	8	126482077	1.69E-02	3.58E-01	TRUE	8	125469835	1.68E-02	1.00	7151	1.66E-06	TRUE	TRUE	482,730	23.00

Triacylglycerol (56:6) levels	Parkinson's disease	rs2968430	G	C	G	C	-1.04E-01	-1.61E-02	1.55E-01	1.26E-01	TRUE	FALSE	16	85325642	3.57E-02	1.86E-01	TRUE	16	85292036	2.34E-02	0.96	7151	8.42E-06	TRUE	TRUE	482,730	19.87
Triacylglycerol (56:6) levels	Parkinson's disease	rs3096698	C	T	C	T	-7.68E-02	-2.36E-02	6.31E-01	3.22E-01	FALSE	FALSE	6	32107139	2.04E-02	6.06E-01	TRUE	6	32139362	1.73E-02	0.99	7151	9.21E-06	TRUE	TRUE	482,730	19.70
Triacylglycerol (56:6) levels	Parkinson's disease	rs33951980	T	C	T	C	-1.28E-01	-6.00E-03	1.22E-01	8.79E-01	FALSE	FALSE	7	73029437	2.71E-02	8.41E-02	TRUE	7	73615107	2.54E-02	1.00	7151	4.81E-07	TRUE	TRUE	482,730	25.38
Triacylglycerol (56:6) levels	Parkinson's disease	rs4277987	G	A	G	A	-9.53E-02	-2.47E-02	2.41E-01	7.77E-01	FALSE	FALSE	6	169330769	2.96E-02	3.93E-01	TRUE	6	168930674	1.96E-02	0.98	7151	1.14E-06	TRUE	TRUE	482,730	23.72
Triacylglycerol (56:6) levels	Parkinson's disease	rs56013388	T	C	T	C	-3.79E-01	9.88E-02	1.03E-02	9.85E-01	FALSE	FALSE	10	76035911	1.01E-01	4.85E-01	TRUE	10	74276153	8.27E-02	0.97	7151	4.59E-06	TRUE	TRUE	482,730	21.03
Triacylglycerol (56:6) levels	Parkinson's disease	rs56296027	C	T	C	T	-8.79E-02	-2.20E-03	2.85E-01	7.68E-01	FALSE	FALSE	2	21134011	2.01E-02	3.97E-02	TRUE	2	20934251	1.83E-02	1.00	7151	1.60E-06	TRUE	TRUE	482,730	23.06
Triacylglycerol (56:6) levels	Parkinson's disease	rs7846376	A	G	A	G	-7.98E-02	-8.50E-03	6.37E-01	3.08E-01	FALSE	FALSE	8	131155443	2.09E-02	1.65E-01	TRUE	8	130143197	1.75E-02	1.00	7151	5.53E-06	TRUE	TRUE	482,730	20.67
Triacylglycerol (56:6) levels	Parkinson's disease	rs7863337	T	C	T	C	-1.37E-01	9.00E-03	9.49E-02	9.12E-01	FALSE	FALSE	9	74249202	4.04E-02	8.48E-02	TRUE	9	71634286	2.95E-02	0.95	7151	3.65E-06	TRUE	TRUE	482,730	21.47
Triacylglycerol (56:6) levels	Parkinson's disease	rs9578336	G	C	G	C	1.00E-01	7.80E-03	1.82E-01	1.15E-01	TRUE	FALSE	13	21546513	2.71E-02	1.12E-01	TRUE	13	20972374	2.17E-02	0.97	7151	4.07E-06	TRUE	TRUE	482,730	21.26
Triacylglycerol (56:6) levels	Parkinson's disease	rs964184	C	G	C	G	-2.41E-01	-5.68E-02	8.49E-01	8.63E-01	TRUE	FALSE	11	116648917	2.43E-02	9.71E-03	TRUE	11	116778201	2.31E-02	1.00	7151	2.07E-25	TRUE	TRUE	482,730	109.34
Triacylglycerol (56:7) levels	Parkinson's disease	rs10149017	G	A	G	A	9.33E-02	1.97E-02	7.96E-01	2.42E-01	FALSE	FALSE	14	95515519	2.59E-02	3.50E-01	TRUE	14	95049182	2.11E-02	0.99	7148	9.44E-06	TRUE	TRUE	482,730	19.65
Triacylglycerol (56:7) levels	Parkinson's disease	rs112214356	C	T	C	T	1.64E-01	1.71E-02	6.09E-02	9.25E-01	FALSE	FALSE	3	195923985	3.58E-02	1.99E-01	TRUE	3	196197114	3.53E-02	0.99	7148	3.46E-06	TRUE	TRUE	482,730	21.58
Triacylglycerol (56:7) levels	Parkinson's disease	rs115007623	C	T	C	T	4.03E-01	-4.98E-02	9.91E-03	9.71E-01	FALSE	FALSE	6	44016412	5.91E-02	3.99E-01	TRUE	6	44048675	8.93E-02	0.90	7148	6.41E-06	TRUE	TRUE	482,730	20.39
Triacylglycerol (56:7) levels	Parkinson's disease	rs1260326	C	T	C	T	-9.86E-02	-6.78E-02	6.51E-01	4.20E-01	FALSE	FALSE	2	27730940	1.72E-02	4.04E-05	TRUE	2	27508073	1.75E-02	1.00	7148	1.79E-08	TRUE	TRUE	482,730	31.78
Triacylglycerol (56:7) levels	Parkinson's disease	rs148132478	T	C	T	C	-7.36E-01	-6.59E-02	2.92E-03	9.84E-01	FALSE	FALSE	14	74767422	9.39E-02	3.16E-01	TRUE	14	74300719	1.63E-01	0.89	7148	6.59E-06	TRUE	TRUE	482,730	20.34
Triacylglycerol (56:7) levels	Parkinson's disease	rs174554	G	A	G	A	-1.61E-01	-3.50E-03	4.06E-01	6.73E-01	FALSE	FALSE	11	61579463	1.79E-02	7.26E-02	TRUE	11	61811991	1.70E-02	1.00	7148	2.72E-21	TRUE	TRUE	482,730	90.30
Triacylglycerol (56:7) levels	Parkinson's disease	rs1902483	G	T	G	T	7.56E-02	9.10E-03	5.05E-01	5.61E-01	FALSE	FALSE	4	155956844	2.25E-02	1.63E-01	TRUE	4	155035692	1.66E-02	1.00	7148	5.63E-06	TRUE	TRUE	482,730	20.64
Triacylglycerol (56:7) levels	Parkinson's disease	rs2229738	T	C	T	C	-1.21E-01	5.00E-04	1.56E-01	9.25E-01	FALSE	FALSE	11	68562328	3.29E-02	4.94E-03	TRUE	11	68794860	2.35E-02	0.94	7148	2.81E-07	TRUE	TRUE	482,730	26.43
Triacylglycerol (56:7) levels	Parkinson's disease	rs2626606	A	G	A	G	-1.10E-01	4.20E-03	8.62E-01	1.43E-01	FALSE	FALSE	14	97969335	3.14E-02	4.86E-02	TRUE	14	97502998	2.42E-02	0.98	7148	5.80E-06	TRUE	TRUE	482,730	20.58
Triacylglycerol (56:7) levels	Parkinson's disease	rs34001747	G	C	G	C	3.58E-01	8.43E-02	1.16E-02	1.51E-02	TRUE	FALSE	17	16863687	1.06E-01	3.69E-01	TRUE	17	16960373	8.10E-02	0.90	7148	1.00E-05	TRUE	TRUE	482,730	19.54
Triacylglycerol (56:7) levels	Parkinson's disease	rs3916027	A	G	A	G	-1.26E-01	3.60E-03	2.53E-01	7.21E-01	FALSE	FALSE	8	19824868	1.87E-02	7.30E-02	TRUE	8	19967357	1.92E-02	1.00	7148	6.22E-11	TRUE	TRUE	482,730	42.88
Triacylglycerol (56:7) levels	Parkinson's disease	rs513272	C	T	C	T	7.93E-02	-6.20E-03	5.64E-01	4.33E-01	FALSE	FALSE	12	129537939	2.31E-02	1.03E-01	TRUE	12	129053394	1.74E-02	0.95	7148	4.97E-06	TRUE	TRUE	482,730	20.88
Triacylglycerol (56:7) levels	Parkinson's disease	rs592586	G	A	G	A	8.04E-02	-1.44E-02	5.95E-01	4.29E-01	FALSE	FALSE	1	110193390	1.91E-02	3.46E-01	TRUE	1	109650768	1.69E-02	0.99	7148	2.10E-06	TRUE	TRUE	482,730	22.54
Triacylglycerol (56:7) levels	Parkinson's disease	rs62419698	G	C	G	C	-1.72E-01	1.60E-03	6.05E-02	3.52E-02	TRUE	FALSE	6	105911061	7.29E-02	7.80E-03	TRUE	6	105463186	3.73E-02	0.89	7148	3.81E-06	TRUE	TRUE	482,730	21.39
Triacylglycerol (56:7) levels	Parkinson's disease	rs6699175	T	C	T	C	1.24E-01	1.48E-02	1.14E-01	8.94E-01	FALSE	FALSE	1	72514896	3.93E-02	1.50E-01	TRUE	1	72049213	2.66E-02	0.96	7148	3.27E-06	TRUE	TRUE	482,730	21.68
Triacylglycerol (56:7) levels	Parkinson's disease	rs6904245	A	G	A	G	1.45E-01	-4.10E-03	8.06E-02	8.62E-01	FALSE	FALSE	6	134742161	3.25E-02	4.62E-02	TRUE	6	134421023	3.15E-02	0.98	7148	4.28E-06	TRUE	TRUE	482,730	21.17
Triacylglycerol (56:7) levels	Parkinson's disease	rs7002672	A	G	A	G	9.53E-02	1.73E-02	8.05E-01	2.49E-01	FALSE	FALSE	8	65606916	2.21E-02	3.62E-01	TRUE	8	64694359	2.11E-02	1.00	7148	6.36E-06	TRUE	TRUE	482,730	20.41
Triacylglycerol (56:7) levels	Parkinson's disease	rs7156531	G	C	G	C	1.22E-01	4.15E-02	1.14E-01	1.36E-01	TRUE	FALSE	14	36702807	4.30E-02	4.76E-01	TRUE	14	36233601	2.71E-02	0.95	7148	6.40E-06	TRUE	TRUE	482,730	20.39
Triacylglycerol (56:7) levels	Parkinson's disease	rs7170012	G	A	G	A	-7.61E-02	1.90E-02	5.79E-01	5.08E-01	FALSE	FALSE	15	60156653	1.92E-02	4.91E-01	TRUE	15	59864454	1.69E-02	1.00	7148	7.03E-06	TRUE	TRUE	482,730	20.21
Triacylglycerol (56:7) levels	Parkinson's disease	rs72623355	C	T	C	T	-1.33E-01	-1.00E-04	9.69E-02	8.96E-01	FALSE	FALSE	13	55582300	3.75E-02	1.04E-03	TRUE	13	55008165	2.84E-02	0.98	7148	2.64E-06	TRUE	TRUE	482,730	22.10
Triacylglycerol (56:7) levels	Parkinson's disease	rs72767411	A	T	A	T	2.96E-01	3.43E-02	1.66E-02	2.36E-02	TRUE	FALSE	5	56981164	7.72E-02	1.83E-01	TRUE	5	57685337	6.58E-02	0.96	7148	6.92E-06	TRUE	TRUE	482,730	20.24
Triacylglycerol (56:7) levels	Parkinson's disease	rs73095198	A	G	A	G	-2.28E-01	-1.97E-02	2.88E-02	9.64E-01	FALSE	FALSE	3	72123123	6.02E-02	1.29E-01	TRUE	3	72073972	5.12E-02	0.99	7148	8.52E-06	TRUE	TRUE	482,730	19.85
Triacylglycerol (56:7) levels	Parkinson's disease	rs75402627	C	T	C	T	1.56E-01	-7.86E-02	7.46E-02	9.46E-01	FALSE	FALSE	9	133239928	5.06E-02	9.19E-01	TRUE	9	130364541	3.25E-02	0.98	7148	1.59E-06	TRUE	TRUE	482,730	23.07
Triacylglycerol (56:7) levels	Parkinson's disease	rs7608044	A	G	A	G	-1.27E-01	3.60E-02	1.04E-01	8.67E-01	FALSE	FALSE	2	74232342	2.99E-02	6.42E-01	TRUE	2	74005215	2.82E-02	0.95	7148	7.11E-06	TRUE	TRUE	482,730	20.19
Triacylglycerol (56:7) levels	Parkinson's disease	rs80329613	A	G	A	G	-1.64E-01	-5.27E-02	5.37E-02	9.49E-01	FALSE	FALSE	4	76554752	4.12E-02	6.97E-01	TRUE	4	75629568	3.69E-02	0.99	7148	8.55E-06	TRUE	TRUE	482,730	19.84
Triacylglycerol (56:7) levels	Parkinson's disease	rs964184	C	G	C	G	-2.16E-01	-5.68E-02	8.49E-01	8.63E-01	TRUE	FALSE	11	116648917	2.43E-02	9.71E-03	TRUE	11	116778201	2.31E-02	1.00	7148	1.24E-20	TRUE	TRUE	482,730	87.27
Triacylglycerol (56:8) levels	Parkinson's disease	rs10480656	G	C	G	C	9.22E-02	1.19E-02	2.66E-01	2.87E-01	TRUE	FALSE	7	104192719	2.48E-02	2.01E-01	TRUE	7	104552272	1.92E-02	0.99	6800	1.63E-06	TRUE	TRUE	482,730	23.03
Triacylglycerol (56:8) levels	Parkinson's disease	rs11142645	A	G	A	G	-1.93E-01	-4.18E-02	4.29E-02	9.84E-01	FALSE	FALSE	9	73547045	1.08E-01	1.56E-01	TRUE	9	70932129	4.27E-02	0.97	6800	6.61E-06	TRUE	TRUE	482,730	20.33
Triacylglycerol (56:8) levels	Parkinson's disease	rs11649869	G	A	G	A	1.05E-01	-8.80E-03	1.82E-01	8.48E-01	FALSE	FALSE	17	47617669	3.24E-02	1.05E-01	TRUE	17	49540307	2.27E-02	0.96	6800	3.75E-06	TRUE	TRUE	482,730	21.42
Triacylglycerol (56:8) levels	Parkinson's disease	rs117564891	G	A	G	A	-2.12E-01	2.33E-02	3.66E-02	9.72E-01	FALSE	FALSE	15	82426870	6.02E-02	1.56E-01	TRUE	15	82134529	4.64E-02	0.98	6800	4.80E-06	TRUE	TRUE	482,730	20.95
Triacylglycerol (56:8) levels	Parkinson's disease	rs11892100	C	G	C	G	-1.70E-01	4.00E-02	5.62E-02	4.68E-02	TRUE	FALSE	2	40327176	5.41E-02	3.37E-01	TRUE	2	40100036	3.75E-02	0.98	6800	5.67E-06	TRUE	TRUE	482,730	20.63
Triacylglycerol (56:8) levels	Parkinson's disease	rs1260326	C	T	C	T	-8.57E-02	-6.78E-02	6.51E-01	4.20E-01	FALSE	FALSE	2	27730940	1.72E-02	4.04E-05	TRUE	2	27508073	1.80E-02	1.00	6800	1.90E-06	TRUE	TRUE	482,730	22.74
Triacylglycerol (56:8) levels	Parkinson's disease	rs13205789	C	T	C	T	-1.20E-01	1.72E-02	1.20E-01	8.49E-01	FALSE	FALSE	6	137901303	3.14E-02	2.34E-01	TRUE	6	137580166	2.64E-02	0.99	6800	5.38E-06	TRUE	TRUE	482,730	20.73
Triacylglycerol (56:8) levels	Parkinson's disease	rs141329532	G	C	G	C	6.86E-01	1.53E-01	3.35E-03	1.15E-02	TRUE	FALSE	7	144598365	1.69E-01	4.37E-01	TRUE	7	144901272	1.52E-01	0.89	6800	7.02E-06	TRUE	TRUE	482,730	20.22
Triacylglycerol (56:8) levels	Parkinson's disease	rs174530	G	A	G	A	-1.41E-01	-1.80E-03	4.14E-01	6.44E-01	FALSE	FALSE	11	61546592	1.79E-02	3.58E-02	TRUE	11	61779120	1.75E-02	1.00	6800	9.01E-16	TRUE	TRUE	482,730	64.95
Triacylglycerol (56:8) levels	Parkinson's disease	rs2165558	G	T	G	T	-1.11E-01	3.30E-03	2.71E-01	6.94E-01	FALSE	FALSE	8	19856260	1.85E-02	6.59E-02	TRUE	8	19998749	1.92E-02	1.00	6800	6.70E-09	TRUE	TRUE	482,730	33.70
Triacylglycerol (56:8) levels	Parkinson's disease	rs2229738	T	C	T	C	-1.37E-01	5.00E-04	1.56E-01	9.25E-01	FALSE	FALSE	11	68562328	3.29E-02	4.94E-03	TRUE	11	68794860	2.41E-02	0.94	6800	1.48E-08	TRUE	TRUE	482,730	32.15
Triacylglycerol (56:8) levels	Parkinson's disease	rs2647110	C	G	C	G	9.28E-02	-7.90E-03	2.40E-01	3.65E-01	TRUE	FALSE	4	125689202	2.34E-02	1.34E-01	TRUE	4	124768047	2.03E-02	0.99	6800	4.68E-06	TRUE	TRUE	482,730	21.00
Triacylglycerol (56:8) levels	Parkinson's disease	rs4711782	T	A	T	A	1.44E-01	6.47E-02	7.87E-02	3.36E-02	TRUE	FALSE	6	44363230	5.99E-02	5.53E-01	TRUE	6	44395493	3.23E-02	1.00	6800	8.44E-06	TRUE	TRUE	482,730	19.86
Triacylglycerol (56:8) levels	Parkinson's disease	rs4726178	T	C	T	C	-9.70E-02	6.00E-04	2.01E-01	8.25E-01	FALSE	FALSE	7	152272347	3.18E-02	6.56E-03	TRUE	7	152575262	2.15E-02	1.00	6800	6.81E-06	TRUE	TRUE	482,730	20.28
Triacylglycerol (56:8) levels	Parkinson's disease	rs592586	G	A	G	A	8.17E-02	-1.44E-02	5.95E-01	4.29E-01	FALSE	FALSE	1	110193390	1.91E-02	3.46E-01	TRUE	1	109650768	1.74E-02	0.99	6800	2.57E-06	TRUE	TRUE	482,730	22.15
Triacylglycerol (56:8) levels	Parkinson's disease	rs60543421	A	G	A	G	-2.92E-01	6.91E-02	1.82E-02	9.83E-01	FALSE	FALSE	13	108106475	8.71E-02	3.69E-01	TRUE	13	107454127	6.52E-02	0.99	6800	7.35E-06	TRUE	TRUE	482,730	20.13
Triacylglycerol (56:8) levels	Parkinson's disease	rs61967369	C	A	C	A	-1.61E-01	-5.28E-02	6.44E-02	9.23E-01	FALSE	FALSE	13	73666610	4.20E-02	6.81E-01	TRUE	13	73092472	3.48E-02	1.00	6800	3.71E-06	TRUE	TRUE	482,730	21.44
Triacylglycerol (56:8) levels	Parkinson's disease	rs61994001	A	T	A	T	-6.10E-01	1.00E-02	5.52E-03	2.08E-02	TRUE	FALSE	14	79372758	8.23E-02	4.42E-02	TRUE	14	78906415	1.20E-01	0.99	6800	4.12E-07	TRUE	TRUE	482,730	25.69
Triacylglycerol (56:8) levels	Parkinson's disease	rs62258666	A	G	A	G	4.99E-01	2.96E-02	6.81E-03	9.76E-01	FALSE	FALSE	3	35281457	7.79E-02	1.52E-01	TRUE	3	35239965	1.08E-01	0.94	6800	3.69E-06	TRUE	TRUE	482,730	21.45
Triacylglycerol (56:8) levels	Parkinson's disease	rs62419698	G	C	G	C	-1.84E-01	1.60E-03	6.05E-02	3.52E-02	TRUE	FALSE	6	105911061	7.29E-02	7.80E-03	TRUE	6	105463186	3.83E-02	0.89	6800	1.62E-06	TRUE	TRUE	482,730	23.05
Triacylglycerol (56:8) levels	Parkinson's disease	rs7031579	T	C	T	C	-1.47E-01	9.10E-02	9.20E-01	8.48E-02	FALSE	FALSE	9	133250275	4.01E-02	1.16E-02	TRUE	9	130374888	3.19E-02	1.00	6800	4.18E-06	TRUE	TRUE	482,730	21.22
Triacylglycerol (56:8) levels	Parkinson's disease	rs72623355	C	T	C	T	-1.32E-01	-1.00E-04	9.69E-02	8.96E-01	FALSE	FALSE	13	55582300	3.75E-02	1.04E-03	TRUE	13	55008165	2.93E-02	0.98	6800	6.70E-06	TRUE	TRUE	482,730	20.31
Triacylglycerol (56:8) levels	Parkinson's disease	rs7621165	A	G	A	G	-8.14E-02	5.90E-03	4.50E-01	5.81E-01	FALSE	FALSE	3	166390732	2.25E-02	1.00E-01	TRUE	3	166672944	1.73E-02	1.00	6800	2.75E-06	TRUE	TRUE	482,730	22.02
Triacylglycerol (56:8) levels	Parkinson's disease	rs78095833	T	C	T	C	-2.85E-01	-1.08E-01	1.99E-02	9.84E-01	FALSE	FALSE	1	237542217	9.79E-02	5.67E-01	TRUE	1	237378917	6.37E-02	0.90	6800	8.05E-06	TRUE	TRUE	482,730	19.95
Triacylglycerol (56:8) levels	Parkinson's disease	rs7815372	G	A	G	A	9.91E-02	1.07E-02	7.92E-01	2.77E-01	FALSE	FALSE	8	65608460	2.10E-02	2.16E-01	TRUE	8	64695903	2.11E-02	1.00	6800	2.68E-06	TRUE	TRUE	482,730	22.07
Triacylglycerol (56:8) levels	Parkinson's disease	rs8028182	T	G	T	G	-1.03E-01	-3.65E-02	1.88E-01	7.85E-01	FALSE	FALSE	15	75718669	2.12E-02	4.26E-02	TRUE	15	75426328	2.21E-02	1.00	6800	3.45E-06	TRUE	TRUE	482,730	21.59
Triacylglycerol (56:8) levels	Parkinson's disease	rs964184	C	G	C	G	-1.94E-01	-5.68E-02	8.49E-01	8.63E-01	TRUE	FALSE	11	116648917	2.43E-02	9.71E-03	TRUE	11	116778201	2.38E-02	1.00	6800	4.73E-16	TRUE	TRUE	482,730	66.23
Triacylglycerol (58:7) levels	Parkinson's disease	rs102275	C	T	C	T	-1.59E-01	-9.10E-03	4.10E-01	6.60E-01	FALSE	FALSE	11	61557803	1.78E-02	2.14E-01	TRUE	11	61790331	1.96E-02	1.00	5403	5.95E-16	TRUE	TRUE	482,730	65.85
Triacylglycerol (58:7) levels	Parkinson's disease	rs117926062	T	G	T	G	-4.54E-01	7.18E-02	8.80E-03	9.81E-01	FALSE	FALSE	6	139251654	8.70E-02	3.89E-01	TRUE	6	138930517	1.01E-01	0.97	5403	7.65E-06	TRUE	TRUE	482,730	20.06
Triacylglycerol (58:7) levels	Parkinson's disease	rs1258712	T	G	T	G	1.80E-01	7.77E-02	9.31E-01	7.38E-02	FALSE	FALSE	12	31466550	4.37E-02	3.77E-02	TRUE	12	31313616	3.90E-02	0.96	5403	4.08E-06	TRUE	TRUE	482,730	21.27
Triacylglycerol (58:7) levels	Parkinson's disease	rs1260326	C	T	C	T	-1.19E-01	-6.78E-02	6.51E-01	4.20E-01	FALSE	FALSE	2	27730940	1.72E-02	4.04E-05	TRUE	2	27508073	2.00E-02	1.00	5403	2.66E-09	TRUE	TRUE	482,730	35.54
Triacylglycerol (58:7) levels	Parkinson's disease	rs12700064	A	G	A	G	1.58E-01	-1.34E-02	9.00E-01	7.45E-02	FALSE	FALSE	7	19656705	5.56E-02	9.17E-02	TRUE	7	19617082	3.33E-02	0.93	5403	2.12E-06	TRUE	TRUE	482,730	22.53
Triacylglycerol (58:7) levels	Parkinson's disease	rs141765432	T	G	T	G	-2.63E-01	-1.04E-02	3.53E-02	9.88E-01	FALSE	FALSE	14	76982471	1.38E-01	2.69E-02	TRUE	14	76516128	5.32E-02	0.93	5403	8.36E-07	TRUE	TRUE	482,730	24.33
Triacylglycerol (58:7) levels	Parkinson's disease	rs3011337	G	A	G	A	9.91E-02	-9.10E-03	6.73E-01	3.49E-01	FALSE	FALSE	10	122533793	2.34E-02	1.56E-01	TRUE	10	120774281	2.08E-02	0.98	5403	1.85E-06	TRUE	TRUE	482,730	22.79
Triacylglycerol (58:7) levels	Parkinson's disease	rs592586	G	A	G	A	9.12E-02	-1.44E-02	5.95E-01	4.29E-01	FALSE	FALSE	1	110193390	1.91E-02	3.46E-01	TRUE	1	109650768	1.95E-02	0.99	5403	2.91E-06	TRUE	TRUE	482,730	21.92
Triacylglycerol (58:7) levels	Parkinson's disease	rs7764213	C	T	C	T	1.08E-01	-2.73E-02	1.95E-01	7.27E-01	FALSE	FALSE	6	112458606	2.04E-02	7.40E-01	TRUE	6	112137404	2.42E-02	1.00	5403	7.83E-06	TRUE	TRUE	482,730	20.01
Triacylglycerol (58:7) levels	Parkinson's disease	rs964184	C	G	C	G	-2.10E-01	-5.68E-02	8.49E-01	8.63E-01	TRUE	FALSE	11	116648917	2.43E-02	9.71E-03	TRUE	11	116778201	2.63E-02	1.00	5403	1.38E-15	TRUE	TRUE	482,730	64.17
Triacylglycerol (58:8) levels	Parkinson's disease	rs10521440	T	C	T	C	-1.83E-01	-4.19E-02	4.87E-02	9.83E-01	FALSE	FALSE	9	73535474	1.06E-01	1.60E-01	TRUE	9	70920558	4.14E-02	0.98	6384	9.72E-06	TRUE	TRUE	482,730	19.59
Triacylglycerol (58:8) levels	Parkinson's disease	rs112792028	A	C	A	C	-9.28E-02	-3.00E-03	3.22E-01	6.92E-01	FALSE	FALSE	9	19072290	2.99E-02	3.64E-02	TRUE	9	19072292	1.92E-02	0.98	6384	1.34E-06	TRUE	TRUE	482,730	23.40
Triacylglycerol (58:8) levels	Parkinson's disease	rs116095614	A	G	A	G	4.95E-01	-1.68E-01	7.37E-03	9.87E-01	FALSE	FALSE	3	2180753	1.08E-01	9.27E-01	TRUE	3	2139069	1.07E-01	0.94	6384	3.78E-06	TRUE	TRUE	482,730	21.41
Triacylglycerol (58:8) levels	Parkinson's disease	rs11776355	T	C	T	C	-8.73E-02	-3.89E-02	2.88E-01	7.30E-01	FALSE	FALSE	8	93474544	2.54E-02	9.01E-01	TRUE	8	92462316	1.96E-02	0.99	6384	8.51E-06	TRUE	TRUE	482,730	19.85
Triacylglycerol (58:8) levels	Parkinson's disease	rs12476545	G	C	G	C	1.26E-01	9.00E-04	1.15E-01	8.75E-02	TRUE	FALSE	2	29283385	3.07E-02	9.79E-03	TRUE	2	29060519	2.80E-02	0.98	6384	6.61E-06	TRUE	TRUE	482,730	20.34
Triacylglycerol (58:8) levels	Parkinson's disease	rs138578034	T	G	T	G	-6.78E-01	1.54E-01	3.22E-03	9.93E-01	FALSE	FALSE	11	17918702	1.95E-01	3.66E-01	TRUE	11	17897155	1.53E-01	0.94	6384	9.37E-06	TRUE	TRUE	482,730	19.66
Triacylglycerol (58:8) levels	Parkinson's disease	rs141329532	G	C	G	C	7.11E-01	1.53E-01	3.35E-03	1.15E-02	TRUE	FALSE	7	144598365	1.69E-01	4.37E-01	TRUE	7	144901272	1.60E-01	0.89	6384	9.39E-06	TRUE	TRUE	482,730	19.66
Triacylglycerol (58:8) levels	Parkinson's disease	rs143453636	G	C	G	C	-4.35E-01	-6.60E-03	9.02E-03	1.05E-02	TRUE	FALSE	9	129628531	1.24E-01	1.90E-02	TRUE	9	126866252	9.75E-02	0.95	6384	8.52E-06	TRUE	TRUE	482,730	19.85
Triacylglycerol (58:8) levels	Parkinson's disease	rs151093994	A	G	A	G	-2.84E-01	2.02E-02	2.00E-02	9.74E-01	FALSE	FALSE	6	163154991	8.09E-02	9.55E-02	TRUE	6	162733959	6.41E-02	0.95	6384	9.38E-06	TRUE	TRUE	482,730	19.66
Triacylglycerol (58:8) levels	Parkinson's disease	rs174544	A	C	A	C	-9.64E-02	-9.00E-04	3.80E-01	7.09E-01	FALSE	FALSE	11	61567753	1.85E-02	1.67E-02	TRUE	11	61800281	1.84E-02	1.00	6384	1.74E-07	TRUE	TRUE	482,730	27.36
Triacylglycerol (58:8) levels	Parkinson's disease	rs17765709	T	C	T	C	-1.14E-01	4.87E-02	1.40E-01	9.16E-01	FALSE	FALSE	14	37022572	4.25E-02	6.00E-01	TRUE	14	36553367	2.51E-02	0.99	6384	6.02E-06	TRUE	TRUE	482,730	20.51
Triacylglycerol (58:8) levels	Parkinson's disease	rs2229738	T	C	T	C	-1.19E-01	5.00E-04	1.56E-01	9.25E-01	FALSE	FALSE	11	68562328	3.29E-02	4.94E-03	TRUE	11	68794860	2.50E-02	0.94	6384	1.92E-06	TRUE	TRUE	482,730	22.71
Triacylglycerol (58:8) levels	Parkinson's disease	rs261291	C	T	C	T	8.13E-02	1.50E-02	3.95E-01	6.56E-01	FALSE	FALSE	15	58680178	1.78E-02	4.01E-01	TRUE	15	58387979	1.80E-02	1.00	6384	6.05E-06	TRUE	TRUE	482,730	20.51
Triacylglycerol (58:8) levels	Parkinson's disease	rs34718616	G	A	G	A	-1.24E-01	1.55E-02	1.20E-01	8.48E-01	FALSE	FALSE	6	137919709	3.15E-02	2.06E-01	TRUE	6	137598572	2.72E-02	0.99	6384	5.68E-06	TRUE	TRUE	482,730	20.62
Triacylglycerol (58:8) levels	Parkinson's disease	rs3916027	A	G	A	G	-1.08E-01	3.60E-03	2.53E-01	7.21E-01	FALSE	FALSE	8	19824868	1.87E-02	7.30E-02	TRUE	8	19967357	2.04E-02	1.00	6384	1.32E-07	TRUE	TRUE	482,730	27.89
Triacylglycerol (58:8) levels	Parkinson's disease	rs4726178	T	C	T	C	-1.06E-01	6.00E-04	2.01E-01	8.25E-01	FALSE	FALSE	7	152272347	3.18E-02	6.56E-03	TRUE	7	152575262	2.21E-02	1.00	6384	1.54E-06	TRUE	TRUE	482,730	23.14
Triacylglycerol (58:8) levels	Parkinson's disease	rs506912	A	C	A	C	9.29E-02	-1.62E-02	7.05E-01	3.47E-01	FALSE	FALSE	1	110191977	1.99E-02	3.80E-01	TRUE	1	109649355	1.93E-02	0.99	6384	1.46E-06	TRUE	TRUE	482,730	23.25
Triacylglycerol (58:8) levels	Parkinson's disease	rs61967369	C	A	C	A	-1.73E-01	-5.28E-02	6.44E-02	9.23E-01	FALSE	FALSE	13	73666610	4.20E-02	6.81E-01	TRUE	13	73092472	3.60E-02	1.00	6384	1.55E-06	TRUE	TRUE	482,730	23.13
Triacylglycerol (58:8) levels	Parkinson's disease	rs62321577	T	G	T	G	-2.06E-01	-3.49E-02	5.02E-02	9.40E-01	FALSE	FALSE	4	76781355	3.84E-02	4.39E-01	TRUE	4	75860202	4.03E-02	0.98	6384	3.13E-07	TRUE	TRUE	482,730	26.22
Triacylglycerol (58:8) levels	Parkinson's disease	rs62419698	G	C	G	C	-1.78E-01	1.60E-03	6.05E-02	3.52E-02	TRUE	FALSE	6	105911061	7.29E-02	7.80E-03	TRUE	6	105463186	3.94E-02	0.89	6384	6.66E-06	TRUE	TRUE	482,730	20.32
Triacylglycerol (58:8) levels	Parkinson's disease	rs6429013	A	C	A	C	1.16E-01	-1.79E-02	8.60E-01	2.32E-01	FALSE	FALSE	1	237592607	2.69E-02	2.96E-01	TRUE	1	237429307	2.59E-02	0.99	6384	6.87E-06	TRUE	TRUE	482,730	20.26
Triacylglycerol (58:8) levels	Parkinson's disease	rs67872791	G	T	G	T	5.05E-01	-3.81E-02	6.99E-03	9.71E-01	FALSE	FALSE	3	65533830	6.76E-02	2.41E-01	TRUE	3	65548155	1.07E-01	0.99	6384	2.40E-06	TRUE	TRUE	482,730	22.28
Triacylglycerol (58:8) levels	Parkinson's disease	rs7336059	G	A	G	A	-2.80E-01	-5.71E-02	2.13E-02	9.83E-01	FALSE	FALSE	13	22222773	9.18E-02	2.73E-01	TRUE	13	21648634	6.33E-02	0.92	6384	9.87E-06	TRUE	TRUE	482,730	19.56
Triacylglycerol (58:8) levels	Parkinson's disease	rs79294676	A	G	A	G	2.80E-01	6.86E-02	2.17E-02	9.89E-01	FALSE	FALSE	2	67504661	1.22E-01	2.41E-01	TRUE	2	67277529	6.21E-02	0.98	6384	6.81E-06	TRUE	TRUE	482,730	20.28
Triacylglycerol (58:8) levels	Parkinson's disease	rs964184	C	G	C	G	-1.71E-01	-5.68E-02	8.49E-01	8.63E-01	TRUE	FALSE	11	116648917	2.43E-02	9.71E-03	TRUE	11	116778201	2.44E-02	1.00	6384	2.38E-12	TRUE	TRUE	482,730	49.33

Significant association between lipid traits and Parkinson's disease by mendelian randomization

exposure	outcome	method	nsnp	b	se	pval	or	or_lci95	or_uci95
Sterol ester (27:1/14:0) levels	Parkinson's disease	MR Egger	13	2.56E-01	1.69E-01	1.60E-01	1.29	0.93	1.80
Sterol ester (27:1/14:0) levels	Parkinson's disease	Weighted median	13	2.25E-02	1.08E-01	8.35E-01	1.02	0.83	1.26
Sterol ester (27:1/14:0) levels	Parkinson's disease	Inverse variance weighted	13	7.86E-02	9.28E-02	3.97E-01	1.08	0.90	1.30
Sterol ester (27:1/14:0) levels	Parkinson's disease	Simple mode	13	-5.08E-02	1.90E-01	7.94E-01	0.95	0.66	1.38
Sterol ester (27:1/14:0) levels	Parkinson's disease	Weighted mode	13	-2.20E-02	2.37E-01	9.28E-01	0.98	0.61	1.56
Sterol ester (27:1/15:0) levels	Parkinson's disease	MR Egger	16	-1.14E-01	9.48E-02	2.50E-01	0.89	0.74	1.07
Sterol ester (27:1/15:0) levels	Parkinson's disease	Weighted median	16	1.28E-02	8.30E-02	8.78E-01	1.01	0.86	1.19
Sterol ester (27:1/15:0) levels	Parkinson's disease	Inverse variance weighted	16	4.57E-03	5.83E-02	9.38E-01	1.00	0.90	1.13
Sterol ester (27:1/15:0) levels	Parkinson's disease	Simple mode	16	1.09E-01	1.35E-01	4.32E-01	1.11	0.86	1.45
Sterol ester (27:1/15:0) levels	Parkinson's disease	Weighted mode	16	5.22E-03	9.76E-02	9.58E-01	1.01	0.83	1.22
Sterol ester (27:1/16:0) levels	Parkinson's disease	MR Egger	27	4.56E-02	7.96E-02	5.72E-01	1.05	0.90	1.22
Sterol ester (27:1/16:0) levels	Parkinson's disease	Weighted median	27	6.96E-03	6.20E-02	9.11E-01	1.01	0.89	1.14
Sterol ester (27:1/16:0) levels	Parkinson's disease	Inverse variance weighted	27	-1.03E-02	4.05E-02	7.98E-01	0.99	0.91	1.07
Sterol ester (27:1/16:0) levels	Parkinson's disease	Simple mode	27	6.89E-04	1.12E-01	9.95E-01	1.00	0.80	1.25
Sterol ester (27:1/16:0) levels	Parkinson's disease	Weighted mode	27	6.06E-03	7.76E-02	9.38E-01	1.01	0.86	1.17
Sterol ester (27:1/16:1) levels	Parkinson's disease	MR Egger	21	-1.81E-02	1.33E-01	8.93E-01	0.98	0.76	1.27
Sterol ester (27:1/16:1) levels	Parkinson's disease	Weighted median	21	4.40E-02	7.55E-02	5.60E-01	1.04	0.90	1.21
Sterol ester (27:1/16:1) levels	Parkinson's disease	Inverse variance weighted	21	9.03E-02	6.13E-02	1.41E-01	1.09	0.97	1.23
Sterol ester (27:1/16:1) levels	Parkinson's disease	Simple mode	21	-5.08E-02	1.18E-01	6.72E-01	0.95	0.75	1.20
Sterol ester (27:1/16:1) levels	Parkinson's disease	Weighted mode	21	5.30E-03	1.09E-01	9.62E-01	1.01	0.81	1.24
Sterol ester (27:1/17:0) levels	Parkinson's disease	MR Egger	25	-9.24E-02	1.50E-01	5.44E-01	0.91	0.68	1.22
Sterol ester (27:1/17:0) levels	Parkinson's disease	Weighted median	25	-1.06E-01	7.88E-02	1.79E-01	0.90	0.77	1.05
Sterol ester (27:1/17:0) levels	Parkinson's disease	Inverse variance weighted	25	-8.81E-02	5.97E-02	1.40E-01	0.92	0.81	1.03
Sterol ester (27:1/17:0) levels	Parkinson's disease	Simple mode	25	3.11E-03	1.44E-01	9.83E-01	1.00	0.76	1.33
Sterol ester (27:1/17:0) levels	Parkinson's disease	Weighted mode	25	-1.06E-01	1.30E-01	4.24E-01	0.90	0.70	1.16
Sterol ester (27:1/17:1) levels	Parkinson's disease	MR Egger	11	-1.24E-01	1.73E-01	4.91E-01	0.88	0.63	1.24
Sterol ester (27:1/17:1) levels	Parkinson's disease	Weighted median	11	-8.84E-03	1.15E-01	9.39E-01	0.99	0.79	1.24
Sterol ester (27:1/17:1) levels	Parkinson's disease	Inverse variance weighted	11	5.58E-02	8.17E-02	4.95E-01	1.06	0.90	1.24
Sterol ester (27:1/17:1) levels	Parkinson's disease	Simple mode	11	-2.57E-02	1.78E-01	8.88E-01	0.97	0.69	1.38
Sterol ester (27:1/17:1) levels	Parkinson's disease	Weighted mode	11	-3.68E-02	1.58E-01	8.21E-01	0.96	0.71	1.32
Sterol ester (27:1/18:0) levels	Parkinson's disease	MR Egger	19	-1.90E-01	1.63E-01	2.58E-01	0.83	0.60	1.14
Sterol ester (27:1/18:0) levels	Parkinson's disease	Weighted median	19	-7.25E-02	7.72E-02	3.48E-01	0.93	0.80	1.08
Sterol ester (27:1/18:0) levels	Parkinson's disease	Inverse variance weighted	19	-8.03E-02	5.52E-02	1.46E-01	0.92	0.83	1.03
Sterol ester (27:1/18:0) levels	Parkinson's disease	Simple mode	19	-6.85E-03	1.39E-01	9.61E-01	0.99	0.76	1.30
Sterol ester (27:1/18:0) levels	Parkinson's disease	Weighted mode	19	-3.86E-02	1.09E-01	7.26E-01	0.96	0.78	1.19
Sterol ester (27:1/18:1) levels	Parkinson's disease	MR Egger	23	7.70E-02	1.03E-01	4.65E-01	1.08	0.88	1.32
Sterol ester (27:1/18:1) levels	Parkinson's disease	Weighted median	23	1.01E-01	7.91E-02	2.04E-01	1.11	0.95	1.29
Sterol ester (27:1/18:1) levels	Parkinson's disease	Inverse variance weighted	23	3.86E-02	5.10E-02	4.50E-01	1.04	0.94	1.15
Sterol ester (27:1/18:1) levels	Parkinson's disease	Simple mode	23	2.02E-01	1.64E-01	2.33E-01	1.22	0.89	1.69
Sterol ester (27:1/18:1) levels	Parkinson's disease	Weighted mode	23	2.10E-01	1.71E-01	2.32E-01	1.23	0.88	1.72
Sterol ester (27:1/18:2) levels	Parkinson's disease	MR Egger	19	1.03E-01	1.05E-01	3.39E-01	1.11	0.90	1.36
Sterol ester (27:1/18:2) levels	Parkinson's disease	Weighted median	19	-3.61E-02	7.60E-02	6.35E-01	0.96	0.83	1.12
Sterol ester (27:1/18:2) levels	Parkinson's disease	Inverse variance weighted	19	-8.65E-02	6.39E-02	1.76E-01	0.92	0.81	1.04
Sterol ester (27:1/18:2) levels	Parkinson's disease	Simple mode	19	-2.23E-02	1.45E-01	8.79E-01	0.98	0.74	1.30

Sterol ester (27:1/18:2) levels	Parkinson's disease	Weighted mode	19	-1.00E-02	1.26E-01	9.38E-01	0.99	0.77	1.27
Sterol ester (27:1/18:3) levels	Parkinson's disease	MR Egger	13	-6.24E-02	1.52E-01	6.89E-01	0.94	0.70	1.27
Sterol ester (27:1/18:3) levels	Parkinson's disease	Weighted median	13	4.75E-02	7.97E-02	5.51E-01	1.05	0.90	1.23
Sterol ester (27:1/18:3) levels	Parkinson's disease	Inverse variance weighted	13	7.49E-02	6.72E-02	2.65E-01	1.08	0.94	1.23
Sterol ester (27:1/18:3) levels	Parkinson's disease	Simple mode	13	-6.97E-02	1.18E-01	5.65E-01	0.93	0.74	1.17
Sterol ester (27:1/18:3) levels	Parkinson's disease	Weighted mode	13	1.87E-02	8.47E-02	8.29E-01	1.02	0.86	1.20
Sterol ester (27:1/20:2) levels	Parkinson's disease	MR Egger	24	7.16E-02	1.10E-01	5.22E-01	1.07	0.87	1.33
Sterol ester (27:1/20:2) levels	Parkinson's disease	Weighted median	24	2.05E-02	5.84E-02	7.26E-01	1.02	0.91	1.14
Sterol ester (27:1/20:2) levels	Parkinson's disease	Inverse variance weighted	24	6.10E-02	4.52E-02	1.77E-01	1.06	0.97	1.16
Sterol ester (27:1/20:2) levels	Parkinson's disease	Simple mode	24	7.49E-02	1.04E-01	4.80E-01	1.08	0.88	1.32
Sterol ester (27:1/20:2) levels	Parkinson's disease	Weighted mode	24	2.49E-02	6.50E-02	7.05E-01	1.03	0.90	1.16
Sterol ester (27:1/20:3) levels	Parkinson's disease	MR Egger	20	6.34E-02	1.03E-01	5.46E-01	1.07	0.87	1.30
Sterol ester (27:1/20:3) levels	Parkinson's disease	Weighted median	20	-8.84E-02	7.34E-02	2.29E-01	0.92	0.79	1.06
Sterol ester (27:1/20:3) levels	Parkinson's disease	Inverse variance weighted	20	-1.55E-02	5.08E-02	7.60E-01	0.98	0.89	1.09
Sterol ester (27:1/20:3) levels	Parkinson's disease	Simple mode	20	-7.11E-02	1.32E-01	5.96E-01	0.93	0.72	1.21
Sterol ester (27:1/20:3) levels	Parkinson's disease	Weighted mode	20	-9.13E-02	7.36E-02	2.30E-01	0.91	0.79	1.05
Sterol ester (27:1/20:4) levels	Parkinson's disease	MR Egger	23	-4.86E-03	3.55E-02	8.92E-01	1.00	0.93	1.07
Sterol ester (27:1/20:4) levels	Parkinson's disease	Weighted median	23	8.00E-03	2.69E-02	7.66E-01	1.01	0.96	1.06
Sterol ester (27:1/20:4) levels	Parkinson's disease	Inverse variance weighted	23	-9.39E-05	2.45E-02	9.97E-01	1.00	0.95	1.05
Sterol ester (27:1/20:4) levels	Parkinson's disease	Simple mode	23	-8.72E-02	9.71E-02	3.79E-01	0.92	0.76	1.11
Sterol ester (27:1/20:4) levels	Parkinson's disease	Weighted mode	23	4.47E-03	2.74E-02	8.72E-01	1.00	0.95	1.06
Sterol ester (27:1/20:5) levels	Parkinson's disease	MR Egger	21	7.52E-02	7.05E-02	2.99E-01	1.08	0.94	1.24
Sterol ester (27:1/20:5) levels	Parkinson's disease	Weighted median	21	5.20E-03	4.01E-02	8.97E-01	1.01	0.93	1.09
Sterol ester (27:1/20:5) levels	Parkinson's disease	Inverse variance weighted	21	-1.69E-02	4.49E-02	7.07E-01	0.98	0.90	1.07
Sterol ester (27:1/20:5) levels	Parkinson's disease	Simple mode	21	-2.32E-01	1.39E-01	1.12E-01	0.79	0.60	1.04
Sterol ester (27:1/20:5) levels	Parkinson's disease	Weighted mode	21	1.19E-02	4.31E-02	7.85E-01	1.01	0.93	1.10
Sterol ester (27:1/22:6) levels	Parkinson's disease	MR Egger	19	-1.93E-01	1.31E-01	1.58E-01	0.82	0.64	1.07
Sterol ester (27:1/22:6) levels	Parkinson's disease	Weighted median	19	1.23E-03	8.70E-02	9.89E-01	1.00	0.84	1.19
Sterol ester (27:1/22:6) levels	Parkinson's disease	Inverse variance weighted	19	-5.21E-03	6.27E-02	9.34E-01	0.99	0.88	1.12
Sterol ester (27:1/22:6) levels	Parkinson's disease	Simple mode	19	4.92E-02	1.54E-01	7.54E-01	1.05	0.78	1.42
Sterol ester (27:1/22:6) levels	Parkinson's disease	Weighted mode	19	7.44E-03	1.18E-01	9.51E-01	1.01	0.80	1.27
Ceramide (d40:1) levels	Parkinson's disease	MR Egger	16	-1.51E-01	2.48E-01	5.52E-01	0.86	0.53	1.40
Ceramide (d40:1) levels	Parkinson's disease	Weighted median	16	-1.35E-01	8.97E-02	1.32E-01	0.87	0.73	1.04
Ceramide (d40:1) levels	Parkinson's disease	Inverse variance weighted	16	-8.14E-02	9.23E-02	3.78E-01	0.92	0.77	1.10
Ceramide (d40:1) levels	Parkinson's disease	Simple mode	16	-1.86E-01	1.37E-01	1.94E-01	0.83	0.64	1.09
Ceramide (d40:1) levels	Parkinson's disease	Weighted mode	16	-1.55E-01	1.08E-01	1.73E-01	0.86	0.69	1.06
Ceramide (d40:2) levels	Parkinson's disease	MR Egger	16	-9.11E-02	1.39E-01	5.22E-01	0.91	0.70	1.20
Ceramide (d40:2) levels	Parkinson's disease	Weighted median	16	-8.25E-02	7.41E-02	2.65E-01	0.92	0.80	1.06
Ceramide (d40:2) levels	Parkinson's disease	Inverse variance weighted	16	-3.88E-03	5.38E-02	9.42E-01	1.00	0.90	1.11
Ceramide (d40:2) levels	Parkinson's disease	Simple mode	16	-5.83E-02	1.35E-01	6.72E-01	0.94	0.72	1.23
Ceramide (d40:2) levels	Parkinson's disease	Weighted mode	16	-1.07E-01	8.69E-02	2.36E-01	0.90	0.76	1.07
Ceramide (d42:1) levels	Parkinson's disease	MR Egger	18	-9.42E-03	2.06E-01	9.64E-01	0.99	0.66	1.48
Ceramide (d42:1) levels	Parkinson's disease	Weighted median	18	2.05E-02	9.92E-02	8.36E-01	1.02	0.84	1.24
Ceramide (d42:1) levels	Parkinson's disease	Inverse variance weighted	18	-1.08E-02	1.05E-01	9.18E-01	0.99	0.81	1.21
Ceramide (d42:1) levels	Parkinson's disease	Simple mode	18	2.00E-01	1.90E-01	3.07E-01	1.22	0.84	1.77
Ceramide (d42:1) levels	Parkinson's disease	Weighted mode	18	1.36E-01	1.34E-01	3.21E-01	1.15	0.88	1.49

Ceramide (d42:2) levels	Parkinson's disease	MR Egger	20	-2.07E-02	2.62E-01	9.38E-01	0.98	0.59	1.64
Ceramide (d42:2) levels	Parkinson's disease	Weighted median	20	-4.96E-02	7.98E-02	5.34E-01	0.95	0.81	1.11
Ceramide (d42:2) levels	Parkinson's disease	Inverse variance weighted	20	2.61E-02	7.43E-02	7.25E-01	1.03	0.89	1.19
Ceramide (d42:2) levels	Parkinson's disease	Simple mode	20	-4.41E-02	1.43E-01	7.62E-01	0.96	0.72	1.27
Ceramide (d42:2) levels	Parkinson's disease	Weighted mode	20	-6.32E-02	1.11E-01	5.76E-01	0.94	0.76	1.17
Cholesterol levels	Parkinson's disease	MR Egger	24	1.34E-01	1.27E-01	3.03E-01	1.14	0.89	1.47
Cholesterol levels	Parkinson's disease	Weighted median	24	2.39E-02	7.70E-02	7.56E-01	1.02	0.88	1.19
Cholesterol levels	Parkinson's disease	Inverse variance weighted	24	5.26E-03	5.56E-02	9.25E-01	1.01	0.90	1.12
Cholesterol levels	Parkinson's disease	Simple mode	24	6.56E-02	1.21E-01	5.94E-01	1.07	0.84	1.35
Cholesterol levels	Parkinson's disease	Weighted mode	24	2.59E-02	1.02E-01	8.03E-01	1.03	0.84	1.25
Diacylglycerol (16:0_18:1) levels	Parkinson's disease	MR Egger	18	1.37E-02	1.56E-01	9.31E-01	1.01	0.75	1.38
Diacylglycerol (16:0_18:1) levels	Parkinson's disease	Weighted median	18	9.70E-02	8.84E-02	2.73E-01	1.10	0.93	1.31
Diacylglycerol (16:0_18:1) levels	Parkinson's disease	Inverse variance weighted	18	1.36E-01	7.48E-02	6.98E-02	1.15	0.99	1.33
Diacylglycerol (16:0_18:1) levels	Parkinson's disease	Simple mode	18	7.45E-02	1.51E-01	6.29E-01	1.08	0.80	1.45
Diacylglycerol (16:0_18:1) levels	Parkinson's disease	Weighted mode	18	4.21E-02	1.37E-01	7.63E-01	1.04	0.80	1.36
Diacylglycerol (16:0_18:2) levels	Parkinson's disease	MR Egger	25	-4.41E-02	1.17E-01	7.10E-01	0.96	0.76	1.20
Diacylglycerol (16:0_18:2) levels	Parkinson's disease	Weighted median	25	2.17E-02	6.50E-02	7.39E-01	1.02	0.90	1.16
Diacylglycerol (16:0_18:2) levels	Parkinson's disease	Inverse variance weighted	25	4.32E-02	5.12E-02	3.99E-01	1.04	0.94	1.15
Diacylglycerol (16:0_18:2) levels	Parkinson's disease	Simple mode	25	-3.07E-02	1.37E-01	8.25E-01	0.97	0.74	1.27
Diacylglycerol (16:0_18:2) levels	Parkinson's disease	Weighted mode	25	1.99E-02	1.35E-01	8.84E-01	1.02	0.78	1.33
Diacylglycerol (16:1_18:1) levels	Parkinson's disease	MR Egger	15	5.75E-02	1.88E-01	7.65E-01	1.06	0.73	1.53
Diacylglycerol (16:1_18:1) levels	Parkinson's disease	Weighted median	15	2.56E-01	8.96E-02	4.30E-03	1.29	1.08	1.54
Diacylglycerol (16:1_18:1) levels	Parkinson's disease	Inverse variance weighted	15	2.35E-01	6.83E-02	5.69E-04	1.27	1.11	1.45
Diacylglycerol (16:1_18:1) levels	Parkinson's disease	Simple mode	15	1.95E-02	1.79E-01	9.15E-01	1.02	0.72	1.45
Diacylglycerol (16:1_18:1) levels	Parkinson's disease	Weighted mode	15	3.35E-01	1.72E-01	7.22E-02	1.40	1.00	1.96
Diacylglycerol (18:1_18:1) levels	Parkinson's disease	MR Egger	20	1.87E-01	1.16E-01	1.25E-01	1.21	0.96	1.51
Diacylglycerol (18:1_18:1) levels	Parkinson's disease	Weighted median	20	1.12E-01	6.93E-02	1.06E-01	1.12	0.98	1.28
Diacylglycerol (18:1_18:1) levels	Parkinson's disease	Inverse variance weighted	20	1.60E-01	4.92E-02	1.11E-03	1.17	1.07	1.29
Diacylglycerol (18:1_18:1) levels	Parkinson's disease	Simple mode	20	5.34E-02	1.16E-01	6.50E-01	1.05	0.84	1.32
Diacylglycerol (18:1_18:1) levels	Parkinson's disease	Weighted mode	20	6.38E-02	8.21E-02	4.47E-01	1.07	0.91	1.25
Diacylglycerol (18:1_18:2) levels	Parkinson's disease	MR Egger	30	3.64E-02	9.38E-02	7.01E-01	1.04	0.86	1.25
Diacylglycerol (18:1_18:2) levels	Parkinson's disease	Weighted median	30	2.88E-02	6.02E-02	6.32E-01	1.03	0.91	1.16
Diacylglycerol (18:1_18:2) levels	Parkinson's disease	Inverse variance weighted	30	4.48E-02	4.82E-02	3.52E-01	1.05	0.95	1.15
Diacylglycerol (18:1_18:2) levels	Parkinson's disease	Simple mode	30	1.10E-02	1.11E-01	9.22E-01	1.01	0.81	1.26
Diacylglycerol (18:1_18:2) levels	Parkinson's disease	Weighted mode	30	4.31E-02	8.52E-02	6.17E-01	1.04	0.88	1.23
Diacylglycerol (18:1_18:3) levels	Parkinson's disease	MR Egger	19	-4.60E-02	1.72E-01	7.92E-01	0.96	0.68	1.34
Diacylglycerol (18:1_18:3) levels	Parkinson's disease	Weighted median	19	1.44E-01	8.65E-02	9.64E-02	1.15	0.97	1.37
Diacylglycerol (18:1_18:3) levels	Parkinson's disease	Inverse variance weighted	19	1.63E-01	7.23E-02	2.41E-02	1.18	1.02	1.36
Diacylglycerol (18:1_18:3) levels	Parkinson's disease	Simple mode	19	1.15E-01	1.54E-01	4.64E-01	1.12	0.83	1.52
Diacylglycerol (18:1_18:3) levels	Parkinson's disease	Weighted mode	19	1.33E-01	1.37E-01	3.45E-01	1.14	0.87	1.50
Lysophosphatidylcholine (16:0) levels	Parkinson's disease	MR Egger	26	1.61E-01	1.46E-01	2.79E-01	1.18	0.88	1.56
Lysophosphatidylcholine (16:0) levels	Parkinson's disease	Weighted median	26	5.56E-02	8.78E-02	5.27E-01	1.06	0.89	1.26
Lysophosphatidylcholine (16:0) levels	Parkinson's disease	Inverse variance weighted	26	1.06E-01	7.28E-02	1.44E-01	1.11	0.96	1.28
Lysophosphatidylcholine (16:0) levels	Parkinson's disease	Simple mode	26	1.25E-01	2.21E-01	5.75E-01	1.13	0.74	1.75
Lysophosphatidylcholine (16:0) levels	Parkinson's disease	Weighted mode	26	2.44E-01	1.97E-01	2.29E-01	1.28	0.87	1.88
Lysophosphatidylcholine (18:0) levels	Parkinson's disease	MR Egger	20	-1.01E-01	1.60E-01	5.35E-01	0.90	0.66	1.24

Lysophosphatidylcholine (18:0) levels	Parkinson's disease	Weighted median	20	-1.69E-01	8.94E-02	5.81E-02	0.84	0.71	1.01
Lysophosphatidylcholine (18:0) levels	Parkinson's disease	Inverse variance weighted	20	-1.98E-01	6.94E-02	4.36E-03	0.82	0.72	0.94
Lysophosphatidylcholine (18:0) levels	Parkinson's disease	Simple mode	20	-2.89E-01	1.59E-01	8.45E-02	0.75	0.55	1.02
Lysophosphatidylcholine (18:0) levels	Parkinson's disease	Weighted mode	20	-2.19E-01	1.46E-01	1.50E-01	0.80	0.60	1.07
Lysophosphatidylcholine (18:1) levels	Parkinson's disease	MR Egger	18	4.42E-01	1.88E-01	3.15E-02	1.56	1.08	2.25
Lysophosphatidylcholine (18:1) levels	Parkinson's disease	Weighted median	18	-1.58E-01	8.55E-02	6.54E-02	0.85	0.72	1.01
Lysophosphatidylcholine (18:1) levels	Parkinson's disease	Inverse variance weighted	18	-6.60E-02	6.76E-02	3.29E-01	0.94	0.82	1.07
Lysophosphatidylcholine (18:1) levels	Parkinson's disease	Simple mode	18	-1.89E-01	1.64E-01	2.65E-01	0.83	0.60	1.14
Lysophosphatidylcholine (18:1) levels	Parkinson's disease	Weighted mode	18	-1.97E-01	1.55E-01	2.20E-01	0.82	0.61	1.11
Lysophosphatidylcholine (18:2) levels	Parkinson's disease	MR Egger	15	-1.91E-01	2.30E-01	4.21E-01	0.83	0.53	1.30
Lysophosphatidylcholine (18:2) levels	Parkinson's disease	Weighted median	15	-2.04E-02	9.51E-02	8.30E-01	0.98	0.81	1.18
Lysophosphatidylcholine (18:2) levels	Parkinson's disease	Inverse variance weighted	15	3.53E-02	8.55E-02	6.80E-01	1.04	0.88	1.22
Lysophosphatidylcholine (18:2) levels	Parkinson's disease	Simple mode	15	-1.98E-02	1.29E-01	8.81E-01	0.98	0.76	1.26
Lysophosphatidylcholine (18:2) levels	Parkinson's disease	Weighted mode	15	-2.41E-02	1.06E-01	8.24E-01	0.98	0.79	1.20
Lysophosphatidylcholine (20:4) levels	Parkinson's disease	MR Egger	17	6.83E-02	1.26E-01	5.94E-01	1.07	0.84	1.37
Lysophosphatidylcholine (20:4) levels	Parkinson's disease	Weighted median	17	5.83E-03	8.72E-02	9.47E-01	1.01	0.85	1.19
Lysophosphatidylcholine (20:4) levels	Parkinson's disease	Inverse variance weighted	17	-5.19E-02	5.89E-02	3.78E-01	0.95	0.85	1.07
Lysophosphatidylcholine (20:4) levels	Parkinson's disease	Simple mode	17	5.63E-02	1.34E-01	6.80E-01	1.06	0.81	1.38
Lysophosphatidylcholine (20:4) levels	Parkinson's disease	Weighted mode	17	1.90E-02	1.10E-01	8.65E-01	1.02	0.82	1.27
Lysophosphatidylethanolamine (18:0) levels	Parkinson's disease	MR Egger	15	2.27E-01	4.35E-01	6.10E-01	1.26	0.54	2.94
Lysophosphatidylethanolamine (18:0) levels	Parkinson's disease	Weighted median	15	-2.99E-02	8.02E-02	7.09E-01	0.97	0.83	1.14
Lysophosphatidylethanolamine (18:0) levels	Parkinson's disease	Inverse variance weighted	15	1.36E-01	1.74E-01	4.37E-01	1.15	0.81	1.61
Lysophosphatidylethanolamine (18:0) levels	Parkinson's disease	Simple mode	15	-2.72E-02	1.12E-01	8.12E-01	0.97	0.78	1.21
Lysophosphatidylethanolamine (18:0) levels	Parkinson's disease	Weighted mode	15	-1.93E-02	9.81E-02	8.47E-01	0.98	0.81	1.19
Lysophosphatidylethanolamine (18:1) levels	Parkinson's disease	MR Egger	14	-7.50E-02	1.57E-01	6.41E-01	0.93	0.68	1.26
Lysophosphatidylethanolamine (18:1) levels	Parkinson's disease	Weighted median	14	5.81E-03	9.40E-02	9.51E-01	1.01	0.84	1.21
Lysophosphatidylethanolamine (18:1) levels	Parkinson's disease	Inverse variance weighted	14	1.35E-03	7.32E-02	9.85E-01	1.00	0.87	1.16
Lysophosphatidylethanolamine (18:1) levels	Parkinson's disease	Simple mode	14	3.09E-02	1.89E-01	8.72E-01	1.03	0.71	1.49
Lysophosphatidylethanolamine (18:1) levels	Parkinson's disease	Weighted mode	14	-4.22E-02	1.68E-01	8.06E-01	0.96	0.69	1.33
Lysophosphatidylethanolamine (18:2) levels	Parkinson's disease	MR Egger	22	-4.55E-02	7.99E-02	5.76E-01	0.96	0.82	1.12
Lysophosphatidylethanolamine (18:2) levels	Parkinson's disease	Weighted median	22	-5.00E-03	5.00E-02	9.20E-01	1.00	0.90	1.10
Lysophosphatidylethanolamine (18:2) levels	Parkinson's disease	Inverse variance weighted	22	4.56E-02	4.69E-02	3.30E-01	1.05	0.95	1.15
Lysophosphatidylethanolamine (18:2) levels	Parkinson's disease	Simple mode	22	-8.90E-02	1.37E-01	5.22E-01	0.91	0.70	1.20
Lysophosphatidylethanolamine (18:2) levels	Parkinson's disease	Weighted mode	22	-1.14E-02	4.94E-02	8.20E-01	0.99	0.90	1.09
Phosphatidylcholine (14:0_16:0) levels	Parkinson's disease	MR Egger	11	-4.48E-02	1.62E-01	7.88E-01	0.96	0.70	1.31
Phosphatidylcholine (14:0_16:0) levels	Parkinson's disease	Weighted median	11	1.22E-01	9.15E-02	1.83E-01	1.13	0.94	1.35
Phosphatidylcholine (14:0_16:0) levels	Parkinson's disease	Inverse variance weighted	11	-7.85E-03	7.97E-02	9.22E-01	0.99	0.85	1.16
Phosphatidylcholine (14:0_16:0) levels	Parkinson's disease	Simple mode	11	1.02E-01	1.47E-01	5.03E-01	1.11	0.83	1.48
Phosphatidylcholine (14:0_16:0) levels	Parkinson's disease	Weighted mode	11	1.18E-01	1.10E-01	3.07E-01	1.13	0.91	1.40
Phosphatidylcholine (14:0_18:1) levels	Parkinson's disease	MR Egger	11	-4.30E-01	7.09E-01	5.59E-01	0.65	0.16	2.61
Phosphatidylcholine (14:0_18:1) levels	Parkinson's disease	Weighted median	11	4.88E-02	1.12E-01	6.62E-01	1.05	0.84	1.31
Phosphatidylcholine (14:0_18:1) levels	Parkinson's disease	Inverse variance weighted	11	5.84E-02	1.13E-01	6.07E-01	1.06	0.85	1.32
Phosphatidylcholine (14:0_18:1) levels	Parkinson's disease	Simple mode	11	-2.81E-01	1.93E-01	1.76E-01	0.76	0.52	1.10
Phosphatidylcholine (14:0_18:1) levels	Parkinson's disease	Weighted mode	11	6.31E-02	1.87E-01	7.42E-01	1.07	0.74	1.54
Phosphatidylcholine (14:0_18:2) levels	Parkinson's disease	MR Egger	17	5.02E-02	2.00E-01	8.05E-01	1.05	0.71	1.56
Phosphatidylcholine (14:0_18:2) levels	Parkinson's disease	Weighted median	17	5.57E-02	7.71E-02	4.70E-01	1.06	0.91	1.23

Phosphatidylcholine (14:0_18:2) levels	Parkinson's disease	Inverse variance weighted	17	1.27E-01	6.63E-02	5.61E-02	1.14	1.00	1.29
Phosphatidylcholine (14:0_18:2) levels	Parkinson's disease	Simple mode	17	1.45E-01	1.22E-01	2.52E-01	1.16	0.91	1.47
Phosphatidylcholine (14:0_18:2) levels	Parkinson's disease	Weighted mode	17	5.21E-02	8.28E-02	5.38E-01	1.05	0.90	1.24
Phosphatidylcholine (15:0_18:1) levels	Parkinson's disease	MR Egger	16	-4.89E-02	2.41E-01	8.42E-01	0.95	0.59	1.53
Phosphatidylcholine (15:0_18:1) levels	Parkinson's disease	Weighted median	16	3.99E-02	9.79E-02	6.84E-01	1.04	0.86	1.26
Phosphatidylcholine (15:0_18:1) levels	Parkinson's disease	Inverse variance weighted	16	5.93E-02	8.99E-02	5.09E-01	1.06	0.89	1.27
Phosphatidylcholine (15:0_18:1) levels	Parkinson's disease	Simple mode	16	-2.51E-01	2.02E-01	2.33E-01	0.78	0.52	1.16
Phosphatidylcholine (15:0_18:1) levels	Parkinson's disease	Weighted mode	16	-2.33E-01	2.04E-01	2.71E-01	0.79	0.53	1.18
Phosphatidylcholine (15:0_18:2) levels	Parkinson's disease	MR Egger	35	3.31E-02	7.44E-02	6.60E-01	1.03	0.89	1.20
Phosphatidylcholine (15:0_18:2) levels	Parkinson's disease	Weighted median	35	3.64E-02	4.79E-02	4.47E-01	1.04	0.94	1.14
Phosphatidylcholine (15:0_18:2) levels	Parkinson's disease	Inverse variance weighted	35	3.95E-02	3.46E-02	2.53E-01	1.04	0.97	1.11
Phosphatidylcholine (15:0_18:2) levels	Parkinson's disease	Simple mode	35	7.36E-02	9.22E-02	4.30E-01	1.08	0.90	1.29
Phosphatidylcholine (15:0_18:2) levels	Parkinson's disease	Weighted mode	35	1.54E-02	4.92E-02	7.57E-01	1.02	0.92	1.12
Phosphatidylcholine (16:0_16:0) levels	Parkinson's disease	MR Egger	21	1.76E-01	1.49E-01	2.52E-01	1.19	0.89	1.59
Phosphatidylcholine (16:0_16:0) levels	Parkinson's disease	Weighted median	21	7.77E-02	6.22E-02	2.12E-01	1.08	0.96	1.22
Phosphatidylcholine (16:0_16:0) levels	Parkinson's disease	Inverse variance weighted	21	5.03E-02	4.67E-02	2.82E-01	1.05	0.96	1.15
Phosphatidylcholine (16:0_16:0) levels	Parkinson's disease	Simple mode	21	4.91E-02	1.06E-01	6.48E-01	1.05	0.85	1.29
Phosphatidylcholine (16:0_16:0) levels	Parkinson's disease	Weighted mode	21	7.30E-02	8.22E-02	3.85E-01	1.08	0.92	1.26
Phosphatidylcholine (16:0_16:1) levels	Parkinson's disease	MR Egger	14	5.68E-02	3.10E-01	8.58E-01	1.06	0.58	1.94
Phosphatidylcholine (16:0_16:1) levels	Parkinson's disease	Weighted median	14	3.76E-02	1.18E-01	7.49E-01	1.04	0.82	1.31
Phosphatidylcholine (16:0_16:1) levels	Parkinson's disease	Inverse variance weighted	14	1.92E-01	9.12E-02	3.54E-02	1.21	1.01	1.45
Phosphatidylcholine (16:0_16:1) levels	Parkinson's disease	Simple mode	14	-5.77E-03	2.12E-01	9.79E-01	0.99	0.66	1.51
Phosphatidylcholine (16:0_16:1) levels	Parkinson's disease	Weighted mode	14	-2.01E-02	2.29E-01	9.31E-01	0.98	0.63	1.54
Phosphatidylcholine (16:0_17:1) levels	Parkinson's disease	MR Egger	16	-2.46E-03	1.70E-01	9.89E-01	1.00	0.72	1.39
Phosphatidylcholine (16:0_17:1) levels	Parkinson's disease	Weighted median	16	4.34E-03	1.02E-01	9.66E-01	1.00	0.82	1.23
Phosphatidylcholine (16:0_17:1) levels	Parkinson's disease	Inverse variance weighted	16	-3.88E-02	7.10E-02	5.85E-01	0.96	0.84	1.11
Phosphatidylcholine (16:0_17:1) levels	Parkinson's disease	Simple mode	16	-2.12E-03	1.83E-01	9.91E-01	1.00	0.70	1.43
Phosphatidylcholine (16:0_17:1) levels	Parkinson's disease	Weighted mode	16	4.69E-02	1.45E-01	7.52E-01	1.05	0.79	1.39
Phosphatidylcholine (16:0_18:0) levels	Parkinson's disease	MR Egger	33	-1.03E-01	1.07E-01	3.42E-01	0.90	0.73	1.11
Phosphatidylcholine (16:0_18:0) levels	Parkinson's disease	Weighted median	33	-2.59E-02	6.35E-02	6.83E-01	0.97	0.86	1.10
Phosphatidylcholine (16:0_18:0) levels	Parkinson's disease	Inverse variance weighted	33	-1.15E-02	4.40E-02	7.94E-01	0.99	0.91	1.08
Phosphatidylcholine (16:0_18:0) levels	Parkinson's disease	Simple mode	33	1.98E-03	1.12E-01	9.86E-01	1.00	0.80	1.25
Phosphatidylcholine (16:0_18:0) levels	Parkinson's disease	Weighted mode	33	-2.47E-02	8.22E-02	7.65E-01	0.98	0.83	1.15
Phosphatidylcholine (16:0_18:1) levels	Parkinson's disease	MR Egger	16	-1.70E-01	2.32E-01	4.76E-01	0.84	0.54	1.33
Phosphatidylcholine (16:0_18:1) levels	Parkinson's disease	Weighted median	16	3.57E-02	9.29E-02	7.01E-01	1.04	0.86	1.24
Phosphatidylcholine (16:0_18:1) levels	Parkinson's disease	Inverse variance weighted	16	7.85E-02	8.10E-02	3.33E-01	1.08	0.92	1.27
Phosphatidylcholine (16:0_18:1) levels	Parkinson's disease	Simple mode	16	1.25E-01	1.61E-01	4.49E-01	1.13	0.83	1.55
Phosphatidylcholine (16:0_18:1) levels	Parkinson's disease	Weighted mode	16	-9.71E-02	1.61E-01	5.56E-01	0.91	0.66	1.24
Phosphatidylcholine (16:0_18:2) levels	Parkinson's disease	MR Egger	30	3.26E-02	7.58E-02	6.71E-01	1.03	0.89	1.20
Phosphatidylcholine (16:0_18:2) levels	Parkinson's disease	Weighted median	30	-1.72E-02	4.78E-02	7.19E-01	0.98	0.89	1.08
Phosphatidylcholine (16:0_18:2) levels	Parkinson's disease	Inverse variance weighted	30	1.01E-02	3.70E-02	7.84E-01	1.01	0.94	1.09
Phosphatidylcholine (16:0_18:2) levels	Parkinson's disease	Simple mode	30	1.72E-02	8.21E-02	8.36E-01	1.02	0.87	1.20
Phosphatidylcholine (16:0_18:2) levels	Parkinson's disease	Weighted mode	30	-4.28E-03	4.53E-02	9.25E-01	1.00	0.91	1.09
Phosphatidylcholine (16:0_18:3) levels	Parkinson's disease	MR Egger	18	5.26E-02	2.60E-01	8.42E-01	1.05	0.63	1.75
Phosphatidylcholine (16:0_18:3) levels	Parkinson's disease	Weighted median	18	1.28E-01	9.59E-02	1.81E-01	1.14	0.94	1.37
Phosphatidylcholine (16:0_18:3) levels	Parkinson's disease	Inverse variance weighted	18	1.42E-01	7.44E-02	5.68E-02	1.15	1.00	1.33

Phosphatidylcholine (16:0_18:3) levels	Parkinson's disease	Simple mode	18	3.90E-02	1.62E-01	8.13E-01	1.04	0.76	1.43
Phosphatidylcholine (16:0_18:3) levels	Parkinson's disease	Weighted mode	18	-2.02E-03	1.48E-01	9.89E-01	1.00	0.75	1.33
Phosphatidylcholine (16:0_20:1) levels	Parkinson's disease	MR Egger	11	-1.01E-01	2.65E-01	7.12E-01	0.90	0.54	1.52
Phosphatidylcholine (16:0_20:1) levels	Parkinson's disease	Weighted median	11	-3.04E-03	1.02E-01	9.76E-01	1.00	0.82	1.22
Phosphatidylcholine (16:0_20:1) levels	Parkinson's disease	Inverse variance weighted	11	-8.86E-03	7.94E-02	9.11E-01	0.99	0.85	1.16
Phosphatidylcholine (16:0_20:1) levels	Parkinson's disease	Simple mode	11	8.35E-02	1.43E-01	5.72E-01	1.09	0.82	1.44
Phosphatidylcholine (16:0_20:1) levels	Parkinson's disease	Weighted mode	11	1.99E-02	1.22E-01	8.74E-01	1.02	0.80	1.30
Phosphatidylcholine (16:0_20:2) levels	Parkinson's disease	MR Egger	22	-8.57E-02	7.30E-02	2.54E-01	0.92	0.80	1.06
Phosphatidylcholine (16:0_20:2) levels	Parkinson's disease	Weighted median	22	-2.61E-02	5.51E-02	6.36E-01	0.97	0.87	1.09
Phosphatidylcholine (16:0_20:2) levels	Parkinson's disease	Inverse variance weighted	22	9.30E-03	4.17E-02	8.24E-01	1.01	0.93	1.10
Phosphatidylcholine (16:0_20:2) levels	Parkinson's disease	Simple mode	22	-1.87E-02	8.21E-02	8.22E-01	0.98	0.84	1.15
Phosphatidylcholine (16:0_20:2) levels	Parkinson's disease	Weighted mode	22	-3.05E-02	5.61E-02	5.93E-01	0.97	0.87	1.08
Phosphatidylcholine (16:0_20:3) levels	Parkinson's disease	MR Egger	15	-1.54E-01	1.40E-01	2.92E-01	0.86	0.65	1.13
Phosphatidylcholine (16:0_20:3) levels	Parkinson's disease	Weighted median	15	-1.20E-01	6.42E-02	6.26E-02	0.89	0.78	1.01
Phosphatidylcholine (16:0_20:3) levels	Parkinson's disease	Inverse variance weighted	15	-4.88E-02	6.92E-02	4.80E-01	0.95	0.83	1.09
Phosphatidylcholine (16:0_20:3) levels	Parkinson's disease	Simple mode	15	-1.46E-01	1.10E-01	2.07E-01	0.86	0.70	1.07
Phosphatidylcholine (16:0_20:3) levels	Parkinson's disease	Weighted mode	15	-1.23E-01	6.18E-02	6.56E-02	0.88	0.78	1.00
Phosphatidylcholine (16:0_20:4) levels	Parkinson's disease	MR Egger	22	3.74E-02	4.27E-02	3.91E-01	1.04	0.95	1.13
Phosphatidylcholine (16:0_20:4) levels	Parkinson's disease	Weighted median	22	2.14E-03	2.87E-02	9.40E-01	1.00	0.95	1.06
Phosphatidylcholine (16:0_20:4) levels	Parkinson's disease	Inverse variance weighted	22	1.03E-02	2.91E-02	7.24E-01	1.01	0.95	1.07
Phosphatidylcholine (16:0_20:4) levels	Parkinson's disease	Simple mode	22	-3.99E-02	1.18E-01	7.38E-01	0.96	0.76	1.21
Phosphatidylcholine (16:0_20:4) levels	Parkinson's disease	Weighted mode	22	2.79E-03	3.09E-02	9.29E-01	1.00	0.94	1.07
Phosphatidylcholine (16:0_20:5) levels	Parkinson's disease	MR Egger	22	7.02E-02	6.54E-02	2.95E-01	1.07	0.94	1.22
Phosphatidylcholine (16:0_20:5) levels	Parkinson's disease	Weighted median	22	6.43E-03	4.68E-02	8.91E-01	1.01	0.92	1.10
Phosphatidylcholine (16:0_20:5) levels	Parkinson's disease	Inverse variance weighted	22	-1.82E-02	3.80E-02	6.32E-01	0.98	0.91	1.06
Phosphatidylcholine (16:0_20:5) levels	Parkinson's disease	Simple mode	22	-2.08E-01	1.38E-01	1.46E-01	0.81	0.62	1.06
Phosphatidylcholine (16:0_20:5) levels	Parkinson's disease	Weighted mode	22	1.67E-02	4.61E-02	7.21E-01	1.02	0.93	1.11
Phosphatidylcholine (16:0_22:4) levels	Parkinson's disease	MR Egger	24	-8.28E-02	1.45E-01	5.74E-01	0.92	0.69	1.22
Phosphatidylcholine (16:0_22:4) levels	Parkinson's disease	Weighted median	24	6.32E-02	7.57E-02	4.04E-01	1.07	0.92	1.24
Phosphatidylcholine (16:0_22:4) levels	Parkinson's disease	Inverse variance weighted	24	9.07E-02	6.77E-02	1.80E-01	1.09	0.96	1.25
Phosphatidylcholine (16:0_22:4) levels	Parkinson's disease	Simple mode	24	-2.51E-02	1.27E-01	8.45E-01	0.98	0.76	1.25
Phosphatidylcholine (16:0_22:4) levels	Parkinson's disease	Weighted mode	24	2.85E-02	9.56E-02	7.68E-01	1.03	0.85	1.24
Phosphatidylcholine (16:0_22:5) levels	Parkinson's disease	MR Egger	25	-2.90E-02	7.17E-02	6.90E-01	0.97	0.84	1.12
Phosphatidylcholine (16:0_22:5) levels	Parkinson's disease	Weighted median	25	2.13E-03	4.19E-02	9.60E-01	1.00	0.92	1.09
Phosphatidylcholine (16:0_22:5) levels	Parkinson's disease	Inverse variance weighted	25	1.49E-02	4.24E-02	7.25E-01	1.01	0.93	1.10
Phosphatidylcholine (16:0_22:5) levels	Parkinson's disease	Simple mode	25	-2.02E-02	9.91E-02	8.40E-01	0.98	0.81	1.19
Phosphatidylcholine (16:0_22:5) levels	Parkinson's disease	Weighted mode	25	-1.10E-03	4.13E-02	9.79E-01	1.00	0.92	1.08
Phosphatidylcholine (16:0_22:6) levels	Parkinson's disease	MR Egger	22	-9.74E-02	1.22E-01	4.34E-01	0.91	0.71	1.15
Phosphatidylcholine (16:0_22:6) levels	Parkinson's disease	Weighted median	22	-4.82E-02	7.90E-02	5.42E-01	0.95	0.82	1.11
Phosphatidylcholine (16:0_22:6) levels	Parkinson's disease	Inverse variance weighted	22	-4.75E-02	6.53E-02	4.66E-01	0.95	0.84	1.08
Phosphatidylcholine (16:0_22:6) levels	Parkinson's disease	Simple mode	22	2.34E-01	1.75E-01	1.95E-01	1.26	0.90	1.78
Phosphatidylcholine (16:0_22:6) levels	Parkinson's disease	Weighted mode	22	-1.72E-01	1.07E-01	1.22E-01	0.84	0.68	1.04
Phosphatidylcholine (16:1_18:0) levels	Parkinson's disease	MR Egger	15	-2.31E-01	2.15E-01	3.02E-01	0.79	0.52	1.21
Phosphatidylcholine (16:1_18:0) levels	Parkinson's disease	Weighted median	15	-9.72E-02	8.68E-02	2.63E-01	0.91	0.77	1.08
Phosphatidylcholine (16:1_18:0) levels	Parkinson's disease	Inverse variance weighted	15	-1.32E-02	8.51E-02	8.77E-01	0.99	0.84	1.17
Phosphatidylcholine (16:1_18:0) levels	Parkinson's disease	Simple mode	15	-1.47E-01	1.28E-01	2.71E-01	0.86	0.67	1.11

Phosphatidylcholine (16:1_18:0) levels	Parkinson's disease	Weighted mode	15	-1.59E-01	1.18E-01	2.01E-01	0.85	0.68	1.08
Phosphatidylcholine (16:1_18:1) levels	Parkinson's disease	MR Egger	22	-2.23E-01	1.66E-01	1.93E-01	0.80	0.58	1.11
Phosphatidylcholine (16:1_18:1) levels	Parkinson's disease	Weighted median	22	-4.62E-03	7.11E-02	9.48E-01	1.00	0.87	1.14
Phosphatidylcholine (16:1_18:1) levels	Parkinson's disease	Inverse variance weighted	22	1.75E-02	5.78E-02	7.62E-01	1.02	0.91	1.14
Phosphatidylcholine (16:1_18:1) levels	Parkinson's disease	Simple mode	22	2.90E-02	1.10E-01	7.94E-01	1.03	0.83	1.28
Phosphatidylcholine (16:1_18:1) levels	Parkinson's disease	Weighted mode	22	1.73E-02	7.96E-02	8.30E-01	1.02	0.87	1.19
Phosphatidylcholine (16:1_18:2) levels	Parkinson's disease	MR Egger	19	-4.60E-02	9.46E-02	6.33E-01	0.96	0.79	1.15
Phosphatidylcholine (16:1_18:2) levels	Parkinson's disease	Weighted median	19	-1.76E-02	4.65E-02	7.06E-01	0.98	0.90	1.08
Phosphatidylcholine (16:1_18:2) levels	Parkinson's disease	Inverse variance weighted	19	1.43E-02	4.97E-02	7.73E-01	1.01	0.92	1.12
Phosphatidylcholine (16:1_18:2) levels	Parkinson's disease	Simple mode	19	-8.35E-03	8.98E-02	9.27E-01	0.99	0.83	1.18
Phosphatidylcholine (16:1_18:2) levels	Parkinson's disease	Weighted mode	19	-1.25E-02	4.32E-02	7.76E-01	0.99	0.91	1.07
Phosphatidylcholine (16:1_20:4) levels	Parkinson's disease	MR Egger	15	-1.24E-01	8.91E-02	1.87E-01	0.88	0.74	1.05
Phosphatidylcholine (16:1_20:4) levels	Parkinson's disease	Weighted median	15	2.76E-02	5.28E-02	6.01E-01	1.03	0.93	1.14
Phosphatidylcholine (16:1_20:4) levels	Parkinson's disease	Inverse variance weighted	15	9.01E-02	5.58E-02	1.06E-01	1.09	0.98	1.22
Phosphatidylcholine (16:1_20:4) levels	Parkinson's disease	Simple mode	15	2.02E-01	1.55E-01	2.14E-01	1.22	0.90	1.66
Phosphatidylcholine (16:1_20:4) levels	Parkinson's disease	Weighted mode	15	1.83E-02	5.64E-02	7.50E-01	1.02	0.91	1.14
Phosphatidylcholine (17:0_18:1) levels	Parkinson's disease	MR Egger	14	5.93E-02	1.25E-01	6.43E-01	1.06	0.83	1.35
Phosphatidylcholine (17:0_18:1) levels	Parkinson's disease	Weighted median	14	1.39E-01	8.29E-02	9.47E-02	1.15	0.98	1.35
Phosphatidylcholine (17:0_18:1) levels	Parkinson's disease	Inverse variance weighted	14	5.99E-02	6.66E-02	3.69E-01	1.06	0.93	1.21
Phosphatidylcholine (17:0_18:1) levels	Parkinson's disease	Simple mode	14	1.46E-01	1.18E-01	2.41E-01	1.16	0.92	1.46
Phosphatidylcholine (17:0_18:1) levels	Parkinson's disease	Weighted mode	14	1.29E-01	9.05E-02	1.77E-01	1.14	0.95	1.36
Phosphatidylcholine (17:0_18:2) levels	Parkinson's disease	MR Egger	20	-4.03E-02	1.09E-01	7.17E-01	0.96	0.78	1.19
Phosphatidylcholine (17:0_18:2) levels	Parkinson's disease	Weighted median	20	-2.34E-02	6.33E-02	7.11E-01	0.98	0.86	1.11
Phosphatidylcholine (17:0_18:2) levels	Parkinson's disease	Inverse variance weighted	20	-2.13E-02	4.40E-02	6.28E-01	0.98	0.90	1.07
Phosphatidylcholine (17:0_18:2) levels	Parkinson's disease	Simple mode	20	-9.28E-02	1.20E-01	4.49E-01	0.91	0.72	1.15
Phosphatidylcholine (17:0_18:2) levels	Parkinson's disease	Weighted mode	20	-2.68E-02	6.63E-02	6.91E-01	0.97	0.86	1.11
Phosphatidylcholine (17:0_20:4) levels	Parkinson's disease	MR Egger	23	2.49E-03	4.55E-02	9.57E-01	1.00	0.92	1.10
Phosphatidylcholine (17:0_20:4) levels	Parkinson's disease	Weighted median	23	-2.24E-04	3.22E-02	9.94E-01	1.00	0.94	1.06
Phosphatidylcholine (17:0_20:4) levels	Parkinson's disease	Inverse variance weighted	23	-9.20E-03	3.07E-02	7.65E-01	0.99	0.93	1.05
Phosphatidylcholine (17:0_20:4) levels	Parkinson's disease	Simple mode	23	-1.41E-01	1.01E-01	1.77E-01	0.87	0.71	1.06
Phosphatidylcholine (17:0_20:4) levels	Parkinson's disease	Weighted mode	23	-2.56E-03	3.17E-02	9.36E-01	1.00	0.94	1.06
Phosphatidylcholine (18:0_18:1) levels	Parkinson's disease	MR Egger	25	-7.39E-02	1.06E-01	4.91E-01	0.93	0.76	1.14
Phosphatidylcholine (18:0_18:1) levels	Parkinson's disease	Weighted median	25	-4.03E-02	7.23E-02	5.77E-01	0.96	0.83	1.11
Phosphatidylcholine (18:0_18:1) levels	Parkinson's disease	Inverse variance weighted	25	-1.81E-02	5.21E-02	7.29E-01	0.98	0.89	1.09
Phosphatidylcholine (18:0_18:1) levels	Parkinson's disease	Simple mode	25	-1.12E-01	1.16E-01	3.43E-01	0.89	0.71	1.12
Phosphatidylcholine (18:0_18:1) levels	Parkinson's disease	Weighted mode	25	-5.37E-02	9.52E-02	5.78E-01	0.95	0.79	1.14
Phosphatidylcholine (18:0_18:2) levels	Parkinson's disease	MR Egger	26	-1.57E-01	1.18E-01	1.95E-01	0.85	0.68	1.08
Phosphatidylcholine (18:0_18:2) levels	Parkinson's disease	Weighted median	26	-3.63E-02	6.07E-02	5.49E-01	0.96	0.86	1.09
Phosphatidylcholine (18:0_18:2) levels	Parkinson's disease	Inverse variance weighted	26	-3.04E-02	4.65E-02	5.13E-01	0.97	0.89	1.06
Phosphatidylcholine (18:0_18:2) levels	Parkinson's disease	Simple mode	26	1.39E-01	1.21E-01	2.60E-01	1.15	0.91	1.46
Phosphatidylcholine (18:0_18:2) levels	Parkinson's disease	Weighted mode	26	-3.15E-02	6.10E-02	6.10E-01	0.97	0.86	1.09
Phosphatidylcholine (18:0_18:3) levels	Parkinson's disease	MR Egger	18	-1.13E-01	1.21E-01	3.65E-01	0.89	0.70	1.13
Phosphatidylcholine (18:0_18:3) levels	Parkinson's disease	Weighted median	18	-2.96E-03	7.61E-02	9.69E-01	1.00	0.86	1.16
Phosphatidylcholine (18:0_18:3) levels	Parkinson's disease	Inverse variance weighted	18	-4.23E-03	6.80E-02	9.50E-01	1.00	0.87	1.14
Phosphatidylcholine (18:0_18:3) levels	Parkinson's disease	Simple mode	18	-1.70E-02	1.20E-01	8.89E-01	0.98	0.78	1.24
Phosphatidylcholine (18:0_18:3) levels	Parkinson's disease	Weighted mode	18	-1.80E-03	1.04E-01	9.86E-01	1.00	0.81	1.22

Phosphatidylcholine (18:0_20:2) levels	Parkinson's disease	MR Egger	14	-1.57E-02	9.10E-02	8.66E-01	0.98	0.82	1.18
Phosphatidylcholine (18:0_20:2) levels	Parkinson's disease	Weighted median	14	-9.78E-03	5.71E-02	8.64E-01	0.99	0.89	1.11
Phosphatidylcholine (18:0_20:2) levels	Parkinson's disease	Inverse variance weighted	14	-4.58E-03	4.42E-02	9.17E-01	1.00	0.91	1.09
Phosphatidylcholine (18:0_20:2) levels	Parkinson's disease	Simple mode	14	-2.07E-02	1.00E-01	8.39E-01	0.98	0.81	1.19
Phosphatidylcholine (18:0_20:2) levels	Parkinson's disease	Weighted mode	14	-9.37E-03	5.78E-02	8.74E-01	0.99	0.88	1.11
Phosphatidylcholine (18:0_20:3) levels	Parkinson's disease	MR Egger	21	-1.56E-01	1.14E-01	1.87E-01	0.86	0.68	1.07
Phosphatidylcholine (18:0_20:3) levels	Parkinson's disease	Weighted median	21	-9.20E-02	6.48E-02	1.56E-01	0.91	0.80	1.04
Phosphatidylcholine (18:0_20:3) levels	Parkinson's disease	Inverse variance weighted	21	-4.76E-02	4.62E-02	3.02E-01	0.95	0.87	1.04
Phosphatidylcholine (18:0_20:3) levels	Parkinson's disease	Simple mode	21	-3.88E-02	1.02E-01	7.07E-01	0.96	0.79	1.17
Phosphatidylcholine (18:0_20:3) levels	Parkinson's disease	Weighted mode	21	-9.62E-02	6.64E-02	1.63E-01	0.91	0.80	1.03
Phosphatidylcholine (18:0_20:4) levels	Parkinson's disease	MR Egger	21	7.54E-03	3.28E-02	8.21E-01	1.01	0.94	1.07
Phosphatidylcholine (18:0_20:4) levels	Parkinson's disease	Weighted median	21	8.64E-03	2.60E-02	7.40E-01	1.01	0.96	1.06
Phosphatidylcholine (18:0_20:4) levels	Parkinson's disease	Inverse variance weighted	21	4.00E-03	2.38E-02	8.66E-01	1.00	0.96	1.05
Phosphatidylcholine (18:0_20:4) levels	Parkinson's disease	Simple mode	21	-4.89E-02	8.80E-02	5.84E-01	0.95	0.80	1.13
Phosphatidylcholine (18:0_20:4) levels	Parkinson's disease	Weighted mode	21	8.23E-03	2.57E-02	7.52E-01	1.01	0.96	1.06
Phosphatidylcholine (18:0_20:5) levels	Parkinson's disease	MR Egger	22	5.40E-02	5.32E-02	3.23E-01	1.06	0.95	1.17
Phosphatidylcholine (18:0_20:5) levels	Parkinson's disease	Weighted median	22	2.13E-02	3.94E-02	5.88E-01	1.02	0.95	1.10
Phosphatidylcholine (18:0_20:5) levels	Parkinson's disease	Inverse variance weighted	22	-5.29E-03	3.37E-02	8.75E-01	0.99	0.93	1.06
Phosphatidylcholine (18:0_20:5) levels	Parkinson's disease	Simple mode	22	-1.64E-01	1.10E-01	1.51E-01	0.85	0.68	1.05
Phosphatidylcholine (18:0_20:5) levels	Parkinson's disease	Weighted mode	22	1.91E-02	3.96E-02	6.36E-01	1.02	0.94	1.10
Phosphatidylcholine (18:0_22:5) levels	Parkinson's disease	MR Egger	24	-3.82E-02	1.29E-01	7.69E-01	0.96	0.75	1.24
Phosphatidylcholine (18:0_22:5) levels	Parkinson's disease	Weighted median	24	3.60E-02	5.94E-02	5.45E-01	1.04	0.92	1.16
Phosphatidylcholine (18:0_22:5) levels	Parkinson's disease	Inverse variance weighted	24	5.69E-02	5.99E-02	3.42E-01	1.06	0.94	1.19
Phosphatidylcholine (18:0_22:5) levels	Parkinson's disease	Simple mode	24	6.41E-02	1.16E-01	5.85E-01	1.07	0.85	1.34
Phosphatidylcholine (18:0_22:5) levels	Parkinson's disease	Weighted mode	24	3.33E-02	6.15E-02	5.93E-01	1.03	0.92	1.17
Phosphatidylcholine (18:0_22:6) levels	Parkinson's disease	MR Egger	19	4.18E-02	1.48E-01	7.80E-01	1.04	0.78	1.39
Phosphatidylcholine (18:0_22:6) levels	Parkinson's disease	Weighted median	19	-9.99E-02	7.78E-02	1.99E-01	0.90	0.78	1.05
Phosphatidylcholine (18:0_22:6) levels	Parkinson's disease	Inverse variance weighted	19	-1.59E-01	5.31E-02	2.85E-03	0.85	0.77	0.95
Phosphatidylcholine (18:0_22:6) levels	Parkinson's disease	Simple mode	19	-1.59E-01	1.43E-01	2.83E-01	0.85	0.64	1.13
Phosphatidylcholine (18:0_22:6) levels	Parkinson's disease	Weighted mode	19	-4.75E-02	9.65E-02	6.29E-01	0.95	0.79	1.15
Phosphatidylcholine (18:1_18:1) levels	Parkinson's disease	MR Egger	18	8.69E-02	1.28E-01	5.07E-01	1.09	0.85	1.40
Phosphatidylcholine (18:1_18:1) levels	Parkinson's disease	Weighted median	18	-3.29E-02	7.00E-02	6.39E-01	0.97	0.84	1.11
Phosphatidylcholine (18:1_18:1) levels	Parkinson's disease	Inverse variance weighted	18	-7.19E-02	5.27E-02	1.73E-01	0.93	0.84	1.03
Phosphatidylcholine (18:1_18:1) levels	Parkinson's disease	Simple mode	18	-3.26E-02	1.08E-01	7.67E-01	0.97	0.78	1.20
Phosphatidylcholine (18:1_18:1) levels	Parkinson's disease	Weighted mode	18	-2.57E-02	8.25E-02	7.59E-01	0.97	0.83	1.15
Phosphatidylcholine (18:1_18:2) levels	Parkinson's disease	MR Egger	20	-3.84E-02	7.90E-02	6.33E-01	0.96	0.82	1.12
Phosphatidylcholine (18:1_18:2) levels	Parkinson's disease	Weighted median	20	-1.68E-02	4.25E-02	6.92E-01	0.98	0.90	1.07
Phosphatidylcholine (18:1_18:2) levels	Parkinson's disease	Inverse variance weighted	20	-5.24E-04	4.31E-02	9.90E-01	1.00	0.92	1.09
Phosphatidylcholine (18:1_18:2) levels	Parkinson's disease	Simple mode	20	3.48E-02	8.78E-02	6.97E-01	1.04	0.87	1.23
Phosphatidylcholine (18:1_18:2) levels	Parkinson's disease	Weighted mode	20	-6.06E-03	4.27E-02	8.89E-01	0.99	0.91	1.08
Phosphatidylcholine (18:1_18:3) levels	Parkinson's disease	MR Egger	14	1.20E-01	1.26E-01	3.59E-01	1.13	0.88	1.44
Phosphatidylcholine (18:1_18:3) levels	Parkinson's disease	Weighted median	14	1.15E-01	8.72E-02	1.88E-01	1.12	0.95	1.33
Phosphatidylcholine (18:1_18:3) levels	Parkinson's disease	Inverse variance weighted	14	1.43E-01	6.55E-02	2.92E-02	1.15	1.01	1.31
Phosphatidylcholine (18:1_18:3) levels	Parkinson's disease	Simple mode	14	9.91E-02	1.33E-01	4.69E-01	1.10	0.85	1.43
Phosphatidylcholine (18:1_18:3) levels	Parkinson's disease	Weighted mode	14	9.22E-02	1.21E-01	4.61E-01	1.10	0.86	1.39
Phosphatidylcholine (18:1_20:2) levels	Parkinson's disease	MR Egger	21	-4.74E-02	6.43E-02	4.70E-01	0.95	0.84	1.08

Phosphatidylcholine (18:1_20:2) levels	Parkinson's disease	Weighted median	21	-1.92E-02	3.91E-02	6.24E-01	0.98	0.91	1.06
Phosphatidylcholine (18:1_20:2) levels	Parkinson's disease	Inverse variance weighted	21	-3.18E-02	3.48E-02	3.61E-01	0.97	0.90	1.04
Phosphatidylcholine (18:1_20:2) levels	Parkinson's disease	Simple mode	21	-6.66E-02	9.08E-02	4.72E-01	0.94	0.78	1.12
Phosphatidylcholine (18:1_20:2) levels	Parkinson's disease	Weighted mode	21	-2.50E-02	3.82E-02	5.20E-01	0.98	0.90	1.05
Phosphatidylcholine (18:1_20:3) levels	Parkinson's disease	MR Egger	19	-1.31E-01	1.10E-01	2.51E-01	0.88	0.71	1.09
Phosphatidylcholine (18:1_20:3) levels	Parkinson's disease	Weighted median	19	-1.20E-01	6.46E-02	6.31E-02	0.89	0.78	1.01
Phosphatidylcholine (18:1_20:3) levels	Parkinson's disease	Inverse variance weighted	19	-7.48E-02	4.93E-02	1.29E-01	0.93	0.84	1.02
Phosphatidylcholine (18:1_20:3) levels	Parkinson's disease	Simple mode	19	-1.23E-01	9.50E-02	2.13E-01	0.88	0.73	1.07
Phosphatidylcholine (18:1_20:3) levels	Parkinson's disease	Weighted mode	19	-1.25E-01	6.90E-02	8.65E-02	0.88	0.77	1.01
Phosphatidylcholine (18:1_20:4) levels	Parkinson's disease	MR Egger	19	-3.35E-03	4.69E-02	9.44E-01	1.00	0.91	1.09
Phosphatidylcholine (18:1_20:4) levels	Parkinson's disease	Weighted median	19	4.94E-03	3.48E-02	8.87E-01	1.00	0.94	1.08
Phosphatidylcholine (18:1_20:4) levels	Parkinson's disease	Inverse variance weighted	19	5.76E-03	3.08E-02	8.52E-01	1.01	0.95	1.07
Phosphatidylcholine (18:1_20:4) levels	Parkinson's disease	Simple mode	19	-5.92E-02	1.18E-01	6.21E-01	0.94	0.75	1.19
Phosphatidylcholine (18:1_20:4) levels	Parkinson's disease	Weighted mode	19	1.86E-03	3.60E-02	9.59E-01	1.00	0.93	1.08
Phosphatidylcholine (18:2_18:2) levels	Parkinson's disease	MR Egger	18	-1.05E-01	1.21E-01	4.01E-01	0.90	0.71	1.14
Phosphatidylcholine (18:2_18:2) levels	Parkinson's disease	Weighted median	18	-2.75E-02	7.23E-02	7.03E-01	0.97	0.84	1.12
Phosphatidylcholine (18:2_18:2) levels	Parkinson's disease	Inverse variance weighted	18	1.83E-02	5.37E-02	7.33E-01	1.02	0.92	1.13
Phosphatidylcholine (18:2_18:2) levels	Parkinson's disease	Simple mode	18	4.94E-02	1.18E-01	6.81E-01	1.05	0.83	1.32
Phosphatidylcholine (18:2_18:2) levels	Parkinson's disease	Weighted mode	18	-1.47E-02	6.96E-02	8.36E-01	0.99	0.86	1.13
Phosphatidylcholine (18:2_20:1) levels	Parkinson's disease	MR Egger	15	-1.64E-02	8.18E-02	8.44E-01	0.98	0.84	1.15
Phosphatidylcholine (18:2_20:1) levels	Parkinson's disease	Weighted median	15	3.09E-02	5.22E-02	5.54E-01	1.03	0.93	1.14
Phosphatidylcholine (18:2_20:1) levels	Parkinson's disease	Inverse variance weighted	15	4.00E-02	4.29E-02	3.51E-01	1.04	0.96	1.13
Phosphatidylcholine (18:2_20:1) levels	Parkinson's disease	Simple mode	15	1.43E-01	1.11E-01	2.17E-01	1.15	0.93	1.43
Phosphatidylcholine (18:2_20:1) levels	Parkinson's disease	Weighted mode	15	2.90E-02	5.50E-02	6.06E-01	1.03	0.92	1.15
Phosphatidylcholine (18:2_20:3) levels	Parkinson's disease	MR Egger	17	-1.21E-01	1.03E-01	2.59E-01	0.89	0.72	1.08
Phosphatidylcholine (18:2_20:3) levels	Parkinson's disease	Weighted median	17	2.17E-03	7.46E-02	9.77E-01	1.00	0.87	1.16
Phosphatidylcholine (18:2_20:3) levels	Parkinson's disease	Inverse variance weighted	17	-1.22E-02	5.00E-02	8.08E-01	0.99	0.90	1.09
Phosphatidylcholine (18:2_20:3) levels	Parkinson's disease	Simple mode	17	2.96E-02	1.13E-01	7.97E-01	1.03	0.83	1.29
Phosphatidylcholine (18:2_20:3) levels	Parkinson's disease	Weighted mode	17	1.68E-02	7.01E-02	8.14E-01	1.02	0.89	1.17
Phosphatidylcholine (18:2_20:4) levels	Parkinson's disease	MR Egger	22	-2.01E-01	1.33E-01	1.48E-01	0.82	0.63	1.06
Phosphatidylcholine (18:2_20:4) levels	Parkinson's disease	Weighted median	22	-2.85E-02	7.56E-02	7.06E-01	0.97	0.84	1.13
Phosphatidylcholine (18:2_20:4) levels	Parkinson's disease	Inverse variance weighted	22	-5.55E-02	5.63E-02	3.24E-01	0.95	0.85	1.06
Phosphatidylcholine (18:2_20:4) levels	Parkinson's disease	Simple mode	22	-5.95E-02	1.19E-01	6.22E-01	0.94	0.75	1.19
Phosphatidylcholine (18:2_20:4) levels	Parkinson's disease	Weighted mode	22	-1.70E-02	8.38E-02	8.41E-01	0.98	0.83	1.16
Phosphatidylcholine (O-16:0_16:0) levels	Parkinson's disease	MR Egger	17	3.14E-01	1.75E-01	9.38E-02	1.37	0.97	1.93
Phosphatidylcholine (O-16:0_16:0) levels	Parkinson's disease	Weighted median	17	-3.73E-02	1.00E-01	7.10E-01	0.96	0.79	1.17
Phosphatidylcholine (O-16:0_16:0) levels	Parkinson's disease	Inverse variance weighted	17	3.95E-02	8.31E-02	6.35E-01	1.04	0.88	1.22
Phosphatidylcholine (O-16:0_16:0) levels	Parkinson's disease	Simple mode	17	-1.83E-02	1.43E-01	9.00E-01	0.98	0.74	1.30
Phosphatidylcholine (O-16:0_16:0) levels	Parkinson's disease	Weighted mode	17	-2.99E-02	1.33E-01	8.24E-01	0.97	0.75	1.26
Phosphatidylcholine (O-16:0_16:1) levels	Parkinson's disease	MR Egger	15	2.18E-01	2.37E-01	3.75E-01	1.24	0.78	1.98
Phosphatidylcholine (O-16:0_16:1) levels	Parkinson's disease	Weighted median	15	2.94E-02	8.24E-02	7.22E-01	1.03	0.88	1.21
Phosphatidylcholine (O-16:0_16:1) levels	Parkinson's disease	Inverse variance weighted	15	4.26E-02	7.42E-02	5.66E-01	1.04	0.90	1.21
Phosphatidylcholine (O-16:0_16:1) levels	Parkinson's disease	Simple mode	15	-4.50E-02	1.40E-01	7.53E-01	0.96	0.73	1.26
Phosphatidylcholine (O-16:0_16:1) levels	Parkinson's disease	Weighted mode	15	3.63E-02	9.83E-02	7.17E-01	1.04	0.86	1.26
Phosphatidylcholine (O-16:0_18:1) levels	Parkinson's disease	MR Egger	17	-5.52E-02	1.53E-01	7.24E-01	0.95	0.70	1.28
Phosphatidylcholine (O-16:0_18:1) levels	Parkinson's disease	Weighted median	17	6.75E-02	7.26E-02	3.53E-01	1.07	0.93	1.23

Phosphatidylcholine (O-16:0_18:1) levels	Parkinson's disease	Inverse variance weighted	17	3.03E-02	5.57E-02	5.86E-01	1.03	0.92	1.15
Phosphatidylcholine (O-16:0_18:1) levels	Parkinson's disease	Simple mode	17	8.14E-02	1.13E-01	4.81E-01	1.08	0.87	1.35
Phosphatidylcholine (O-16:0_18:1) levels	Parkinson's disease	Weighted mode	17	8.53E-02	1.07E-01	4.36E-01	1.09	0.88	1.34
Phosphatidylcholine (O-16:0_18:2) levels	Parkinson's disease	MR Egger	16	4.60E-03	1.06E-01	9.66E-01	1.00	0.82	1.24
Phosphatidylcholine (O-16:0_18:2) levels	Parkinson's disease	Weighted median	16	-5.16E-03	7.29E-02	9.44E-01	0.99	0.86	1.15
Phosphatidylcholine (O-16:0_18:2) levels	Parkinson's disease	Inverse variance weighted	16	-6.08E-04	5.11E-02	9.91E-01	1.00	0.90	1.10
Phosphatidylcholine (O-16:0_18:2) levels	Parkinson's disease	Simple mode	16	-1.44E-02	1.13E-01	9.00E-01	0.99	0.79	1.23
Phosphatidylcholine (O-16:0_18:2) levels	Parkinson's disease	Weighted mode	16	2.05E-03	7.68E-02	9.79E-01	1.00	0.86	1.16
Phosphatidylcholine (O-16:0_20:3) levels	Parkinson's disease	MR Egger	14	-6.03E-02	1.06E-01	5.80E-01	0.94	0.76	1.16
Phosphatidylcholine (O-16:0_20:3) levels	Parkinson's disease	Weighted median	14	-1.25E-01	6.51E-02	5.55E-02	0.88	0.78	1.00
Phosphatidylcholine (O-16:0_20:3) levels	Parkinson's disease	Inverse variance weighted	14	-7.10E-02	4.86E-02	1.44E-01	0.93	0.85	1.02
Phosphatidylcholine (O-16:0_20:3) levels	Parkinson's disease	Simple mode	14	-1.01E-01	1.14E-01	3.90E-01	0.90	0.72	1.13
Phosphatidylcholine (O-16:0_20:3) levels	Parkinson's disease	Weighted mode	14	-1.19E-01	7.28E-02	1.28E-01	0.89	0.77	1.02
Phosphatidylcholine (O-16:0_20:4) levels	Parkinson's disease	MR Egger	24	3.60E-02	6.13E-02	5.63E-01	1.04	0.92	1.17
Phosphatidylcholine (O-16:0_20:4) levels	Parkinson's disease	Weighted median	24	1.15E-02	4.60E-02	8.02E-01	1.01	0.92	1.11
Phosphatidylcholine (O-16:0_20:4) levels	Parkinson's disease	Inverse variance weighted	24	4.61E-03	3.63E-02	8.99E-01	1.00	0.94	1.08
Phosphatidylcholine (O-16:0_20:4) levels	Parkinson's disease	Simple mode	24	1.08E-02	1.22E-01	9.30E-01	1.01	0.80	1.28
Phosphatidylcholine (O-16:0_20:4) levels	Parkinson's disease	Weighted mode	24	1.08E-02	4.78E-02	8.23E-01	1.01	0.92	1.11
Phosphatidylcholine (O-16:0_22:5) levels	Parkinson's disease	MR Egger	10	-2.32E-01	1.80E-01	2.33E-01	0.79	0.56	1.13
Phosphatidylcholine (O-16:0_22:5) levels	Parkinson's disease	Weighted median	10	-1.63E-01	8.62E-02	5.79E-02	0.85	0.72	1.01
Phosphatidylcholine (O-16:0_22:5) levels	Parkinson's disease	Inverse variance weighted	10	-1.21E-01	7.26E-02	9.62E-02	0.89	0.77	1.02
Phosphatidylcholine (O-16:0_22:5) levels	Parkinson's disease	Simple mode	10	-2.31E-01	1.39E-01	1.32E-01	0.79	0.60	1.04
Phosphatidylcholine (O-16:0_22:5) levels	Parkinson's disease	Weighted mode	10	-1.00E-01	9.03E-02	2.96E-01	0.90	0.76	1.08
Phosphatidylcholine (O-16:1_16:0) levels	Parkinson's disease	MR Egger	16	1.81E-01	9.66E-02	8.26E-02	1.20	0.99	1.45
Phosphatidylcholine (O-16:1_16:0) levels	Parkinson's disease	Weighted median	16	7.48E-02	6.22E-02	2.29E-01	1.08	0.95	1.22
Phosphatidylcholine (O-16:1_16:0) levels	Parkinson's disease	Inverse variance weighted	16	2.87E-02	4.67E-02	5.39E-01	1.03	0.94	1.13
Phosphatidylcholine (O-16:1_16:0) levels	Parkinson's disease	Simple mode	16	-1.12E-01	1.04E-01	3.00E-01	0.89	0.73	1.10
Phosphatidylcholine (O-16:1_16:0) levels	Parkinson's disease	Weighted mode	16	1.03E-01	6.47E-02	1.34E-01	1.11	0.98	1.26
Phosphatidylcholine (O-16:1_18:0) levels	Parkinson's disease	MR Egger	14	-7.58E-02	1.53E-01	6.29E-01	0.93	0.69	1.25
Phosphatidylcholine (O-16:1_18:0) levels	Parkinson's disease	Weighted median	14	-4.02E-02	9.37E-02	6.68E-01	0.96	0.80	1.15
Phosphatidylcholine (O-16:1_18:0) levels	Parkinson's disease	Inverse variance weighted	14	-5.27E-02	8.09E-02	5.15E-01	0.95	0.81	1.11
Phosphatidylcholine (O-16:1_18:0) levels	Parkinson's disease	Simple mode	14	3.15E-02	1.74E-01	8.59E-01	1.03	0.73	1.45
Phosphatidylcholine (O-16:1_18:0) levels	Parkinson's disease	Weighted mode	14	-9.35E-02	1.21E-01	4.53E-01	0.91	0.72	1.15
Phosphatidylcholine (O-16:1_18:1) levels	Parkinson's disease	MR Egger	22	-1.03E-01	1.32E-01	4.44E-01	0.90	0.70	1.17
Phosphatidylcholine (O-16:1_18:1) levels	Parkinson's disease	Weighted median	22	-2.18E-02	7.81E-02	7.80E-01	0.98	0.84	1.14
Phosphatidylcholine (O-16:1_18:1) levels	Parkinson's disease	Inverse variance weighted	22	-4.29E-02	5.59E-02	4.43E-01	0.96	0.86	1.07
Phosphatidylcholine (O-16:1_18:1) levels	Parkinson's disease	Simple mode	22	2.12E-02	1.27E-01	8.70E-01	1.02	0.80	1.31
Phosphatidylcholine (O-16:1_18:1) levels	Parkinson's disease	Weighted mode	22	1.35E-02	1.24E-01	9.14E-01	1.01	0.79	1.29
Phosphatidylcholine (O-16:1_18:2) levels	Parkinson's disease	MR Egger	19	-6.53E-02	1.20E-01	5.95E-01	0.94	0.74	1.19
Phosphatidylcholine (O-16:1_18:2) levels	Parkinson's disease	Weighted median	19	-2.93E-02	8.31E-02	7.24E-01	0.97	0.83	1.14
Phosphatidylcholine (O-16:1_18:2) levels	Parkinson's disease	Inverse variance weighted	19	-1.55E-02	5.54E-02	7.80E-01	0.98	0.88	1.10
Phosphatidylcholine (O-16:1_18:2) levels	Parkinson's disease	Simple mode	19	-5.34E-02	1.11E-01	6.38E-01	0.95	0.76	1.18
Phosphatidylcholine (O-16:1_18:2) levels	Parkinson's disease	Weighted mode	19	-2.58E-02	9.94E-02	7.98E-01	0.97	0.80	1.18
Phosphatidylcholine (O-16:1_20:3) levels	Parkinson's disease	MR Egger	21	-1.04E-01	1.42E-01	4.71E-01	0.90	0.68	1.19
Phosphatidylcholine (O-16:1_20:3) levels	Parkinson's disease	Weighted median	21	-9.19E-02	6.43E-02	1.53E-01	0.91	0.80	1.03
Phosphatidylcholine (O-16:1_20:3) levels	Parkinson's disease	Inverse variance weighted	21	-3.60E-02	5.81E-02	5.35E-01	0.96	0.86	1.08

Phosphatidylcholine (O-16:1_20:3) levels	Parkinson's disease	Simple mode	21	-7.85E-02	1.11E-01	4.87E-01	0.92	0.74	1.15
Phosphatidylcholine (O-16:1_20:3) levels	Parkinson's disease	Weighted mode	21	-7.85E-02	7.12E-02	2.84E-01	0.92	0.80	1.06
Phosphatidylcholine (O-16:1_20:4) levels	Parkinson's disease	MR Egger	19	-1.37E-02	6.20E-02	8.28E-01	0.99	0.87	1.11
Phosphatidylcholine (O-16:1_20:4) levels	Parkinson's disease	Weighted median	19	2.94E-02	4.27E-02	4.92E-01	1.03	0.95	1.12
Phosphatidylcholine (O-16:1_20:4) levels	Parkinson's disease	Inverse variance weighted	19	5.58E-02	3.33E-02	9.41E-02	1.06	0.99	1.13
Phosphatidylcholine (O-16:1_20:4) levels	Parkinson's disease	Simple mode	19	1.89E-01	1.07E-01	9.34E-02	1.21	0.98	1.49
Phosphatidylcholine (O-16:1_20:4) levels	Parkinson's disease	Weighted mode	19	3.30E-02	4.07E-02	4.29E-01	1.03	0.95	1.12
Phosphatidylcholine (O-16:2_18:0) levels	Parkinson's disease	MR Egger	11	1.10E-01	1.96E-01	5.89E-01	1.12	0.76	1.64
Phosphatidylcholine (O-16:2_18:0) levels	Parkinson's disease	Weighted median	11	1.21E-01	9.58E-02	2.08E-01	1.13	0.93	1.36
Phosphatidylcholine (O-16:2_18:0) levels	Parkinson's disease	Inverse variance weighted	11	1.50E-01	8.27E-02	7.01E-02	1.16	0.99	1.37
Phosphatidylcholine (O-16:2_18:0) levels	Parkinson's disease	Simple mode	11	3.40E-02	1.44E-01	8.18E-01	1.03	0.78	1.37
Phosphatidylcholine (O-16:2_18:0) levels	Parkinson's disease	Weighted mode	11	7.40E-02	1.01E-01	4.80E-01	1.08	0.88	1.31
Phosphatidylcholine (O-17:0_15:0) levels	Parkinson's disease	MR Egger	16	6.68E-02	1.31E-01	6.17E-01	1.07	0.83	1.38
Phosphatidylcholine (O-17:0_15:0) levels	Parkinson's disease	Weighted median	16	-7.77E-02	9.42E-02	4.10E-01	0.93	0.77	1.11
Phosphatidylcholine (O-17:0_15:0) levels	Parkinson's disease	Inverse variance weighted	16	-4.05E-02	6.58E-02	5.38E-01	0.96	0.84	1.09
Phosphatidylcholine (O-17:0_15:0) levels	Parkinson's disease	Simple mode	16	-1.54E-01	1.64E-01	3.63E-01	0.86	0.62	1.18
Phosphatidylcholine (O-17:0_15:0) levels	Parkinson's disease	Weighted mode	16	-1.48E-01	1.48E-01	3.33E-01	0.86	0.65	1.15
Phosphatidylcholine (O-17:0_17:1) levels	Parkinson's disease	MR Egger	29	-1.21E-01	1.50E-01	4.26E-01	0.89	0.66	1.19
Phosphatidylcholine (O-17:0_17:1) levels	Parkinson's disease	Weighted median	29	-1.35E-02	6.78E-02	8.42E-01	0.99	0.86	1.13
Phosphatidylcholine (O-17:0_17:1) levels	Parkinson's disease	Inverse variance weighted	29	5.21E-03	5.77E-02	9.28E-01	1.01	0.90	1.13
Phosphatidylcholine (O-17:0_17:1) levels	Parkinson's disease	Simple mode	29	-7.68E-02	1.40E-01	5.87E-01	0.93	0.70	1.22
Phosphatidylcholine (O-17:0_17:1) levels	Parkinson's disease	Weighted mode	29	-1.46E-02	1.16E-01	9.00E-01	0.99	0.79	1.24
Phosphatidylcholine (O-18:0_14:0) levels	Parkinson's disease	MR Egger	13	-3.76E-02	1.53E-01	8.10E-01	0.96	0.71	1.30
Phosphatidylcholine (O-18:0_14:0) levels	Parkinson's disease	Weighted median	13	-1.61E-02	1.07E-01	8.80E-01	0.98	0.80	1.21
Phosphatidylcholine (O-18:0_14:0) levels	Parkinson's disease	Inverse variance weighted	13	-5.73E-02	7.18E-02	4.25E-01	0.94	0.82	1.09
Phosphatidylcholine (O-18:0_14:0) levels	Parkinson's disease	Simple mode	13	-6.31E-02	1.75E-01	7.24E-01	0.94	0.67	1.32
Phosphatidylcholine (O-18:0_14:0) levels	Parkinson's disease	Weighted mode	13	1.40E-01	1.71E-01	4.29E-01	1.15	0.82	1.61
Phosphatidylcholine (O-18:0_16:1) levels	Parkinson's disease	MR Egger	13	-1.96E-01	1.71E-01	2.76E-01	0.82	0.59	1.15
Phosphatidylcholine (O-18:0_16:1) levels	Parkinson's disease	Weighted median	13	-3.79E-02	9.33E-02	6.85E-01	0.96	0.80	1.16
Phosphatidylcholine (O-18:0_16:1) levels	Parkinson's disease	Inverse variance weighted	13	5.38E-02	6.48E-02	4.06E-01	1.06	0.93	1.20
Phosphatidylcholine (O-18:0_16:1) levels	Parkinson's disease	Simple mode	13	-1.09E-01	1.28E-01	4.13E-01	0.90	0.70	1.15
Phosphatidylcholine (O-18:0_16:1) levels	Parkinson's disease	Weighted mode	13	-5.56E-02	1.07E-01	6.13E-01	0.95	0.77	1.17
Phosphatidylcholine (O-18:0_20:4) levels	Parkinson's disease	MR Egger	19	-3.10E-02	7.92E-02	7.01E-01	0.97	0.83	1.13
Phosphatidylcholine (O-18:0_20:4) levels	Parkinson's disease	Weighted median	19	1.96E-02	5.18E-02	7.06E-01	1.02	0.92	1.13
Phosphatidylcholine (O-18:0_20:4) levels	Parkinson's disease	Inverse variance weighted	19	6.39E-02	4.28E-02	1.35E-01	1.07	0.98	1.16
Phosphatidylcholine (O-18:0_20:4) levels	Parkinson's disease	Simple mode	19	2.18E-01	1.16E-01	7.69E-02	1.24	0.99	1.56
Phosphatidylcholine (O-18:0_20:4) levels	Parkinson's disease	Weighted mode	19	1.37E-02	5.84E-02	8.16E-01	1.01	0.90	1.14
Phosphatidylcholine (O-18:1_16:0) levels	Parkinson's disease	MR Egger	26	-3.13E-02	1.37E-01	8.21E-01	0.97	0.74	1.27
Phosphatidylcholine (O-18:1_16:0) levels	Parkinson's disease	Weighted median	26	4.68E-02	6.52E-02	4.73E-01	1.05	0.92	1.19
Phosphatidylcholine (O-18:1_16:0) levels	Parkinson's disease	Inverse variance weighted	26	1.17E-02	4.51E-02	7.95E-01	1.01	0.93	1.11
Phosphatidylcholine (O-18:1_16:0) levels	Parkinson's disease	Simple mode	26	1.98E-01	1.21E-01	1.14E-01	1.22	0.96	1.54
Phosphatidylcholine (O-18:1_16:0) levels	Parkinson's disease	Weighted mode	26	6.92E-02	1.01E-01	4.99E-01	1.07	0.88	1.31
Phosphatidylcholine (O-18:1_18:2) levels	Parkinson's disease	MR Egger	20	-8.46E-02	1.81E-01	6.45E-01	0.92	0.64	1.31
Phosphatidylcholine (O-18:1_18:2) levels	Parkinson's disease	Weighted median	20	-6.01E-02	8.94E-02	5.01E-01	0.94	0.79	1.12
Phosphatidylcholine (O-18:1_18:2) levels	Parkinson's disease	Inverse variance weighted	20	-6.78E-02	7.09E-02	3.39E-01	0.93	0.81	1.07
Phosphatidylcholine (O-18:1_18:2) levels	Parkinson's disease	Simple mode	20	-9.92E-02	1.62E-01	5.48E-01	0.91	0.66	1.24

Phosphatidylcholine (O-18:1_18:2) levels	Parkinson's disease	Weighted mode	20	-6.73E-02	1.37E-01	6.28E-01	0.93	0.72	1.22
Phosphatidylcholine (O-18:1_20:3) levels	Parkinson's disease	MR Egger	16	1.45E-02	1.21E-01	9.06E-01	1.01	0.80	1.29
Phosphatidylcholine (O-18:1_20:3) levels	Parkinson's disease	Weighted median	16	-1.49E-02	6.57E-02	8.20E-01	0.99	0.87	1.12
Phosphatidylcholine (O-18:1_20:3) levels	Parkinson's disease	Inverse variance weighted	16	-9.37E-04	5.16E-02	9.86E-01	1.00	0.90	1.11
Phosphatidylcholine (O-18:1_20:3) levels	Parkinson's disease	Simple mode	16	-1.34E-01	1.27E-01	3.09E-01	0.87	0.68	1.12
Phosphatidylcholine (O-18:1_20:3) levels	Parkinson's disease	Weighted mode	16	-2.88E-02	6.40E-02	6.59E-01	0.97	0.86	1.10
Phosphatidylcholine (O-18:1_20:4) levels	Parkinson's disease	MR Egger	17	5.98E-02	8.82E-02	5.08E-01	1.06	0.89	1.26
Phosphatidylcholine (O-18:1_20:4) levels	Parkinson's disease	Weighted median	17	4.98E-03	5.85E-02	9.32E-01	1.00	0.90	1.13
Phosphatidylcholine (O-18:1_20:4) levels	Parkinson's disease	Inverse variance weighted	17	1.14E-02	4.51E-02	8.00E-01	1.01	0.93	1.11
Phosphatidylcholine (O-18:1_20:4) levels	Parkinson's disease	Simple mode	17	1.56E-02	1.30E-01	9.06E-01	1.02	0.79	1.31
Phosphatidylcholine (O-18:1_20:4) levels	Parkinson's disease	Weighted mode	17	3.47E-03	6.16E-02	9.56E-01	1.00	0.89	1.13
Phosphatidylcholine (O-18:2_16:0) levels	Parkinson's disease	MR Egger	20	-5.79E-02	8.83E-02	5.20E-01	0.94	0.79	1.12
Phosphatidylcholine (O-18:2_16:0) levels	Parkinson's disease	Weighted median	20	-3.43E-02	6.93E-02	6.20E-01	0.97	0.84	1.11
Phosphatidylcholine (O-18:2_16:0) levels	Parkinson's disease	Inverse variance weighted	20	-1.91E-02	4.99E-02	7.02E-01	0.98	0.89	1.08
Phosphatidylcholine (O-18:2_16:0) levels	Parkinson's disease	Simple mode	20	-4.02E-02	1.01E-01	6.95E-01	0.96	0.79	1.17
Phosphatidylcholine (O-18:2_16:0) levels	Parkinson's disease	Weighted mode	20	-2.68E-02	7.26E-02	7.16E-01	0.97	0.84	1.12
Phosphatidylcholine (O-18:2_18:1) levels	Parkinson's disease	MR Egger	14	4.84E-01	2.11E-01	4.05E-02	1.62	1.07	2.45
Phosphatidylcholine (O-18:2_18:1) levels	Parkinson's disease	Weighted median	14	-2.99E-02	9.16E-02	7.44E-01	0.97	0.81	1.16
Phosphatidylcholine (O-18:2_18:1) levels	Parkinson's disease	Inverse variance weighted	14	-1.15E-01	9.17E-02	2.12E-01	0.89	0.75	1.07
Phosphatidylcholine (O-18:2_18:1) levels	Parkinson's disease	Simple mode	14	-6.74E-03	1.35E-01	9.61E-01	0.99	0.76	1.29
Phosphatidylcholine (O-18:2_18:1) levels	Parkinson's disease	Weighted mode	14	-6.74E-03	1.04E-01	9.49E-01	0.99	0.81	1.22
Phosphatidylcholine (O-18:2_18:2) levels	Parkinson's disease	MR Egger	15	5.73E-02	1.63E-01	7.31E-01	1.06	0.77	1.46
Phosphatidylcholine (O-18:2_18:2) levels	Parkinson's disease	Weighted median	15	-3.85E-02	7.99E-02	6.30E-01	0.96	0.82	1.13
Phosphatidylcholine (O-18:2_18:2) levels	Parkinson's disease	Inverse variance weighted	15	-3.52E-02	5.87E-02	5.49E-01	0.97	0.86	1.08
Phosphatidylcholine (O-18:2_18:2) levels	Parkinson's disease	Simple mode	15	-3.73E-02	1.05E-01	7.29E-01	0.96	0.78	1.18
Phosphatidylcholine (O-18:2_18:2) levels	Parkinson's disease	Weighted mode	15	-3.43E-02	9.13E-02	7.13E-01	0.97	0.81	1.16
Phosphatidylcholine (O-18:2_20:4) levels	Parkinson's disease	MR Egger	18	-4.84E-02	1.11E-01	6.67E-01	0.95	0.77	1.18
Phosphatidylcholine (O-18:2_20:4) levels	Parkinson's disease	Weighted median	18	1.93E-02	7.14E-02	7.87E-01	1.02	0.89	1.17
Phosphatidylcholine (O-18:2_20:4) levels	Parkinson's disease	Inverse variance weighted	18	2.99E-02	4.99E-02	5.49E-01	1.03	0.93	1.14
Phosphatidylcholine (O-18:2_20:4) levels	Parkinson's disease	Simple mode	18	-5.03E-02	1.07E-01	6.44E-01	0.95	0.77	1.17
Phosphatidylcholine (O-18:2_20:4) levels	Parkinson's disease	Weighted mode	18	8.40E-03	8.19E-02	9.20E-01	1.01	0.86	1.18
Phosphatidylethanolamine (16:0_18:2) levels	Parkinson's disease	MR Egger	20	-3.84E-02	8.18E-02	6.44E-01	0.96	0.82	1.13
Phosphatidylethanolamine (16:0_18:2) levels	Parkinson's disease	Weighted median	20	-7.33E-03	4.30E-02	8.65E-01	0.99	0.91	1.08
Phosphatidylethanolamine (16:0_18:2) levels	Parkinson's disease	Inverse variance weighted	20	3.25E-02	3.79E-02	3.92E-01	1.03	0.96	1.11
Phosphatidylethanolamine (16:0_18:2) levels	Parkinson's disease	Simple mode	20	7.14E-02	7.12E-02	3.29E-01	1.07	0.93	1.23
Phosphatidylethanolamine (16:0_18:2) levels	Parkinson's disease	Weighted mode	20	2.14E-02	3.92E-02	5.91E-01	1.02	0.95	1.10
Phosphatidylethanolamine (16:0_20:4) levels	Parkinson's disease	MR Egger	20	-1.19E-02	7.04E-02	8.67E-01	0.99	0.86	1.13
Phosphatidylethanolamine (16:0_20:4) levels	Parkinson's disease	Weighted median	20	2.38E-02	4.27E-02	5.78E-01	1.02	0.94	1.11
Phosphatidylethanolamine (16:0_20:4) levels	Parkinson's disease	Inverse variance weighted	20	3.26E-02	3.93E-02	4.06E-01	1.03	0.96	1.12
Phosphatidylethanolamine (16:0_20:4) levels	Parkinson's disease	Simple mode	20	-1.12E-03	9.54E-02	9.91E-01	1.00	0.83	1.20
Phosphatidylethanolamine (16:0_20:4) levels	Parkinson's disease	Weighted mode	20	2.17E-02	3.68E-02	5.62E-01	1.02	0.95	1.10
Phosphatidylethanolamine (18:0_18:2) levels	Parkinson's disease	MR Egger	24	1.35E-01	9.99E-02	1.91E-01	1.14	0.94	1.39
Phosphatidylethanolamine (18:0_18:2) levels	Parkinson's disease	Weighted median	24	9.21E-02	5.18E-02	7.52E-02	1.10	0.99	1.21
Phosphatidylethanolamine (18:0_18:2) levels	Parkinson's disease	Inverse variance weighted	24	1.08E-01	4.46E-02	1.58E-02	1.11	1.02	1.22
Phosphatidylethanolamine (18:0_18:2) levels	Parkinson's disease	Simple mode	24	1.60E-01	1.06E-01	1.46E-01	1.17	0.95	1.45
Phosphatidylethanolamine (18:0_18:2) levels	Parkinson's disease	Weighted mode	24	9.40E-02	4.73E-02	5.88E-02	1.10	1.00	1.21

Phosphatidylethanolamine (18:0_20:4) levels	Parkinson's disease	MR Egger	17	-5.85E-02	1.07E-01	5.93E-01	0.94	0.76	1.16
Phosphatidylethanolamine (18:0_20:4) levels	Parkinson's disease	Weighted median	17	7.78E-02	4.46E-02	8.12E-02	1.08	0.99	1.18
Phosphatidylethanolamine (18:0_20:4) levels	Parkinson's disease	Inverse variance weighted	17	6.04E-02	4.94E-02	2.22E-01	1.06	0.96	1.17
Phosphatidylethanolamine (18:0_20:4) levels	Parkinson's disease	Simple mode	17	4.59E-02	7.97E-02	5.73E-01	1.05	0.90	1.22
Phosphatidylethanolamine (18:0_20:4) levels	Parkinson's disease	Weighted mode	17	6.97E-02	4.22E-02	1.18E-01	1.07	0.99	1.16
Phosphatidylethanolamine (18:1_18:1) levels	Parkinson's disease	MR Egger	21	-2.03E-01	1.22E-01	1.11E-01	0.82	0.64	1.04
Phosphatidylethanolamine (18:1_18:1) levels	Parkinson's disease	Weighted median	21	-1.31E-02	6.01E-02	8.28E-01	0.99	0.88	1.11
Phosphatidylethanolamine (18:1_18:1) levels	Parkinson's disease	Inverse variance weighted	21	-1.59E-02	5.92E-02	7.88E-01	0.98	0.88	1.11
Phosphatidylethanolamine (18:1_18:1) levels	Parkinson's disease	Simple mode	21	-4.33E-02	9.97E-02	6.69E-01	0.96	0.79	1.16
Phosphatidylethanolamine (18:1_18:1) levels	Parkinson's disease	Weighted mode	21	-1.41E-02	5.98E-02	8.16E-01	0.99	0.88	1.11
Phosphatidylethanolamine (O-16:1_18:2) levels	Parkinson's disease	MR Egger	15	-1.18E-01	1.04E-01	2.76E-01	0.89	0.73	1.09
Phosphatidylethanolamine (O-16:1_18:2) levels	Parkinson's disease	Weighted median	15	-4.12E-02	6.29E-02	5.12E-01	0.96	0.85	1.09
Phosphatidylethanolamine (O-16:1_18:2) levels	Parkinson's disease	Inverse variance weighted	15	-1.89E-02	5.98E-02	7.52E-01	0.98	0.87	1.10
Phosphatidylethanolamine (O-16:1_18:2) levels	Parkinson's disease	Simple mode	15	-7.40E-02	8.17E-02	3.80E-01	0.93	0.79	1.09
Phosphatidylethanolamine (O-16:1_18:2) levels	Parkinson's disease	Weighted mode	15	-6.65E-02	6.97E-02	3.56E-01	0.94	0.82	1.07
Phosphatidylethanolamine (O-16:1_20:4) levels	Parkinson's disease	MR Egger	17	5.90E-02	1.61E-01	7.19E-01	1.06	0.77	1.45
Phosphatidylethanolamine (O-16:1_20:4) levels	Parkinson's disease	Weighted median	17	3.48E-02	8.16E-02	6.70E-01	1.04	0.88	1.22
Phosphatidylethanolamine (O-16:1_20:4) levels	Parkinson's disease	Inverse variance weighted	17	2.43E-02	5.89E-02	6.80E-01	1.02	0.91	1.15
Phosphatidylethanolamine (O-16:1_20:4) levels	Parkinson's disease	Simple mode	17	3.34E-02	1.35E-01	8.08E-01	1.03	0.79	1.35
Phosphatidylethanolamine (O-16:1_20:4) levels	Parkinson's disease	Weighted mode	17	4.65E-02	8.04E-02	5.71E-01	1.05	0.89	1.23
Phosphatidylethanolamine (O-16:1_22:5) levels	Parkinson's disease	MR Egger	16	5.11E-02	9.76E-02	6.09E-01	1.05	0.87	1.27
Phosphatidylethanolamine (O-16:1_22:5) levels	Parkinson's disease	Weighted median	16	5.26E-02	7.73E-02	4.97E-01	1.05	0.91	1.23
Phosphatidylethanolamine (O-16:1_22:5) levels	Parkinson's disease	Inverse variance weighted	16	4.18E-02	5.56E-02	4.52E-01	1.04	0.94	1.16
Phosphatidylethanolamine (O-16:1_22:5) levels	Parkinson's disease	Simple mode	16	1.01E-01	1.26E-01	4.34E-01	1.11	0.86	1.42
Phosphatidylethanolamine (O-16:1_22:5) levels	Parkinson's disease	Weighted mode	16	6.72E-02	1.00E-01	5.13E-01	1.07	0.88	1.30
Phosphatidylethanolamine (O-18:1_18:2) levels	Parkinson's disease	MR Egger	23	-5.93E-02	1.33E-01	6.60E-01	0.94	0.73	1.22
Phosphatidylethanolamine (O-18:1_18:2) levels	Parkinson's disease	Weighted median	23	-2.66E-02	7.54E-02	7.24E-01	0.97	0.84	1.13
Phosphatidylethanolamine (O-18:1_18:2) levels	Parkinson's disease	Inverse variance weighted	23	-8.64E-02	5.06E-02	8.79E-02	0.92	0.83	1.01
Phosphatidylethanolamine (O-18:1_18:2) levels	Parkinson's disease	Simple mode	23	-1.78E-01	1.30E-01	1.86E-01	0.84	0.65	1.08
Phosphatidylethanolamine (O-18:1_18:2) levels	Parkinson's disease	Weighted mode	23	-2.22E-02	7.93E-02	7.82E-01	0.98	0.84	1.14
Phosphatidylethanolamine (O-18:1_20:4) levels	Parkinson's disease	MR Egger	19	1.28E-01	1.70E-01	4.61E-01	1.14	0.82	1.59
Phosphatidylethanolamine (O-18:1_20:4) levels	Parkinson's disease	Weighted median	19	1.08E-02	6.94E-02	8.77E-01	1.01	0.88	1.16
Phosphatidylethanolamine (O-18:1_20:4) levels	Parkinson's disease	Inverse variance weighted	19	-1.10E-02	7.81E-02	8.88E-01	0.99	0.85	1.15
Phosphatidylethanolamine (O-18:1_20:4) levels	Parkinson's disease	Simple mode	19	-1.16E-01	1.86E-01	5.42E-01	0.89	0.62	1.28
Phosphatidylethanolamine (O-18:1_20:4) levels	Parkinson's disease	Weighted mode	19	7.16E-03	7.39E-02	9.24E-01	1.01	0.87	1.16
Phosphatidylethanolamine (O-18:2_18:1) levels	Parkinson's disease	MR Egger	19	-1.43E-01	1.07E-01	1.97E-01	0.87	0.70	1.07
Phosphatidylethanolamine (O-18:2_18:1) levels	Parkinson's disease	Weighted median	19	-1.15E-01	7.50E-02	1.26E-01	0.89	0.77	1.03
Phosphatidylethanolamine (O-18:2_18:1) levels	Parkinson's disease	Inverse variance weighted	19	-5.64E-02	5.29E-02	2.86E-01	0.95	0.85	1.05
Phosphatidylethanolamine (O-18:2_18:1) levels	Parkinson's disease	Simple mode	19	-5.47E-03	1.20E-01	9.64E-01	0.99	0.79	1.26
Phosphatidylethanolamine (O-18:2_18:1) levels	Parkinson's disease	Weighted mode	19	-1.59E-01	1.06E-01	1.51E-01	0.85	0.69	1.05
Phosphatidylethanolamine (O-18:2_18:2) levels	Parkinson's disease	MR Egger	12	1.85E-01	1.86E-01	3.42E-01	1.20	0.84	1.73
Phosphatidylethanolamine (O-18:2_18:2) levels	Parkinson's disease	Weighted median	12	-2.12E-02	9.11E-02	8.16E-01	0.98	0.82	1.17
Phosphatidylethanolamine (O-18:2_18:2) levels	Parkinson's disease	Inverse variance weighted	12	-5.33E-02	1.11E-01	6.31E-01	0.95	0.76	1.18
Phosphatidylethanolamine (O-18:2_18:2) levels	Parkinson's disease	Simple mode	12	-1.92E-02	1.23E-01	8.79E-01	0.98	0.77	1.25
Phosphatidylethanolamine (O-18:2_18:2) levels	Parkinson's disease	Weighted mode	12	5.47E-02	9.69E-02	5.84E-01	1.06	0.87	1.28
Phosphatidylethanolamine (O-18:2_20:4) levels	Parkinson's disease	MR Egger	21	-6.55E-02	1.30E-01	6.20E-01	0.94	0.73	1.21

Phosphatidylethanolamine (O-18:2_20:4) levels	Parkinson's disease	Weighted median	21	1.27E-02	7.99E-02	8.73E-01	1.01	0.87	1.18
Phosphatidylethanolamine (O-18:2_20:4) levels	Parkinson's disease	Inverse variance weighted	21	6.95E-02	5.48E-02	2.05E-01	1.07	0.96	1.19
Phosphatidylethanolamine (O-18:2_20:4) levels	Parkinson's disease	Simple mode	21	-3.60E-03	1.28E-01	9.78E-01	1.00	0.77	1.28
Phosphatidylethanolamine (O-18:2_20:4) levels	Parkinson's disease	Weighted mode	21	-7.25E-03	8.68E-02	9.34E-01	0.99	0.84	1.18
Phosphatidylinositol (16:0_18:1) levels	Parkinson's disease	MR Egger	12	-7.41E-02	2.26E-01	7.50E-01	0.93	0.60	1.45
Phosphatidylinositol (16:0_18:1) levels	Parkinson's disease	Weighted median	12	-3.95E-02	1.07E-01	7.12E-01	0.96	0.78	1.19
Phosphatidylinositol (16:0_18:1) levels	Parkinson's disease	Inverse variance weighted	12	5.79E-02	8.90E-02	5.15E-01	1.06	0.89	1.26
Phosphatidylinositol (16:0_18:1) levels	Parkinson's disease	Simple mode	12	-6.63E-02	1.57E-01	6.80E-01	0.94	0.69	1.27
Phosphatidylinositol (16:0_18:1) levels	Parkinson's disease	Weighted mode	12	-4.54E-02	1.43E-01	7.56E-01	0.96	0.72	1.26
Phosphatidylinositol (16:0_18:2) levels	Parkinson's disease	MR Egger	14	-3.96E-01	2.30E-01	1.11E-01	0.67	0.43	1.06
Phosphatidylinositol (16:0_18:2) levels	Parkinson's disease	Weighted median	14	-7.73E-03	8.27E-02	9.26E-01	0.99	0.84	1.17
Phosphatidylinositol (16:0_18:2) levels	Parkinson's disease	Inverse variance weighted	14	3.04E-02	8.97E-02	7.35E-01	1.03	0.86	1.23
Phosphatidylinositol (16:0_18:2) levels	Parkinson's disease	Simple mode	14	-9.96E-02	1.25E-01	4.42E-01	0.91	0.71	1.16
Phosphatidylinositol (16:0_18:2) levels	Parkinson's disease	Weighted mode	14	-5.17E-02	8.23E-02	5.41E-01	0.95	0.81	1.12
Phosphatidylinositol (16:0_20:4) levels	Parkinson's disease	MR Egger	19	-1.28E-01	1.56E-01	4.22E-01	0.88	0.65	1.19
Phosphatidylinositol (16:0_20:4) levels	Parkinson's disease	Weighted median	19	5.96E-02	7.63E-02	4.35E-01	1.06	0.91	1.23
Phosphatidylinositol (16:0_20:4) levels	Parkinson's disease	Inverse variance weighted	19	1.17E-01	6.56E-02	7.55E-02	1.12	0.99	1.28
Phosphatidylinositol (16:0_20:4) levels	Parkinson's disease	Simple mode	19	4.05E-02	1.54E-01	7.96E-01	1.04	0.77	1.41
Phosphatidylinositol (16:0_20:4) levels	Parkinson's disease	Weighted mode	19	1.92E-02	1.11E-01	8.64E-01	1.02	0.82	1.27
Phosphatidylinositol (18:0_18:1) levels	Parkinson's disease	MR Egger	16	1.22E-01	1.92E-01	5.37E-01	1.13	0.78	1.65
Phosphatidylinositol (18:0_18:1) levels	Parkinson's disease	Weighted median	16	4.94E-04	7.75E-02	9.95E-01	1.00	0.86	1.16
Phosphatidylinositol (18:0_18:1) levels	Parkinson's disease	Inverse variance weighted	16	5.75E-02	6.08E-02	3.44E-01	1.06	0.94	1.19
Phosphatidylinositol (18:0_18:1) levels	Parkinson's disease	Simple mode	16	1.49E-02	1.48E-01	9.21E-01	1.02	0.76	1.36
Phosphatidylinositol (18:0_18:1) levels	Parkinson's disease	Weighted mode	16	-7.11E-03	9.70E-02	9.43E-01	0.99	0.82	1.20
Phosphatidylinositol (18:0_18:2) levels	Parkinson's disease	MR Egger	26	-9.72E-03	8.44E-02	9.09E-01	0.99	0.84	1.17
Phosphatidylinositol (18:0_18:2) levels	Parkinson's disease	Weighted median	26	-1.66E-02	5.19E-02	7.50E-01	0.98	0.89	1.09
Phosphatidylinositol (18:0_18:2) levels	Parkinson's disease	Inverse variance weighted	26	2.51E-02	4.54E-02	5.81E-01	1.03	0.94	1.12
Phosphatidylinositol (18:0_18:2) levels	Parkinson's disease	Simple mode	26	7.88E-03	1.02E-01	9.39E-01	1.01	0.82	1.23
Phosphatidylinositol (18:0_18:2) levels	Parkinson's disease	Weighted mode	26	-6.86E-03	5.44E-02	9.01E-01	0.99	0.89	1.10
Phosphatidylinositol (18:0_20:3) levels	Parkinson's disease	MR Egger	27	-2.78E-03	9.64E-02	9.77E-01	1.00	0.83	1.20
Phosphatidylinositol (18:0_20:3) levels	Parkinson's disease	Weighted median	27	-6.52E-03	4.84E-02	8.93E-01	0.99	0.90	1.09
Phosphatidylinositol (18:0_20:3) levels	Parkinson's disease	Inverse variance weighted	27	1.82E-03	4.93E-02	9.71E-01	1.00	0.91	1.10
Phosphatidylinositol (18:0_20:3) levels	Parkinson's disease	Simple mode	27	-1.24E-02	1.02E-01	9.04E-01	0.99	0.81	1.21
Phosphatidylinositol (18:0_20:3) levels	Parkinson's disease	Weighted mode	27	-1.24E-02	4.89E-02	8.02E-01	0.99	0.90	1.09
Phosphatidylinositol (18:0_20:4) levels	Parkinson's disease	MR Egger	20	5.22E-02	1.05E-01	6.26E-01	1.05	0.86	1.30
Phosphatidylinositol (18:0_20:4) levels	Parkinson's disease	Weighted median	20	3.42E-02	6.26E-02	5.85E-01	1.03	0.92	1.17
Phosphatidylinositol (18:0_20:4) levels	Parkinson's disease	Inverse variance weighted	20	9.46E-02	4.83E-02	5.01E-02	1.10	1.00	1.21
Phosphatidylinositol (18:0_20:4) levels	Parkinson's disease	Simple mode	20	4.99E-02	1.16E-01	6.71E-01	1.05	0.84	1.32
Phosphatidylinositol (18:0_20:4) levels	Parkinson's disease	Weighted mode	20	4.32E-02	6.74E-02	5.29E-01	1.04	0.91	1.19
Phosphatidylinositol (18:1_18:1) levels	Parkinson's disease	MR Egger	16	-1.13E-02	9.46E-02	9.07E-01	0.99	0.82	1.19
Phosphatidylinositol (18:1_18:1) levels	Parkinson's disease	Weighted median	16	-1.17E-02	6.46E-02	8.56E-01	0.99	0.87	1.12
Phosphatidylinositol (18:1_18:1) levels	Parkinson's disease	Inverse variance weighted	16	6.05E-02	4.67E-02	1.95E-01	1.06	0.97	1.16
Phosphatidylinositol (18:1_18:1) levels	Parkinson's disease	Simple mode	16	-2.46E-02	1.18E-01	8.38E-01	0.98	0.77	1.23
Phosphatidylinositol (18:1_18:1) levels	Parkinson's disease	Weighted mode	16	-2.90E-02	8.05E-02	7.23E-01	0.97	0.83	1.14
Phosphatidylinositol (18:1_18:2) levels	Parkinson's disease	MR Egger	18	-4.70E-02	1.21E-01	7.04E-01	0.95	0.75	1.21
Phosphatidylinositol (18:1_18:2) levels	Parkinson's disease	Weighted median	18	-1.37E-02	6.73E-02	8.39E-01	0.99	0.86	1.13

Phosphatidylinositol (18:1_18:2) levels	Parkinson's disease	Inverse variance weighted	18	2.67E-02	4.83E-02	5.81E-01	1.03	0.93	1.13
Phosphatidylinositol (18:1_18:2) levels	Parkinson's disease	Simple mode	18	-3.09E-02	9.20E-02	7.41E-01	0.97	0.81	1.16
Phosphatidylinositol (18:1_18:2) levels	Parkinson's disease	Weighted mode	18	-1.90E-02	7.69E-02	8.08E-01	0.98	0.84	1.14
Phosphatidylinositol (18:1_20:4) levels	Parkinson's disease	MR Egger	19	-9.79E-02	1.48E-01	5.18E-01	0.91	0.68	1.21
Phosphatidylinositol (18:1_20:4) levels	Parkinson's disease	Weighted median	19	3.51E-02	7.82E-02	6.54E-01	1.04	0.89	1.21
Phosphatidylinositol (18:1_20:4) levels	Parkinson's disease	Inverse variance weighted	19	-3.38E-02	6.53E-02	6.05E-01	0.97	0.85	1.10
Phosphatidylinositol (18:1_20:4) levels	Parkinson's disease	Simple mode	19	-1.03E-02	1.40E-01	9.43E-01	0.99	0.75	1.30
Phosphatidylinositol (18:1_20:4) levels	Parkinson's disease	Weighted mode	19	9.91E-02	1.24E-01	4.34E-01	1.10	0.87	1.41
Sphingomyelin (d32:1) levels	Parkinson's disease	MR Egger	23	-9.40E-02	5.59E-02	1.07E-01	0.91	0.82	1.02
Sphingomyelin (d32:1) levels	Parkinson's disease	Weighted median	23	-4.16E-02	4.22E-02	3.25E-01	0.96	0.88	1.04
Sphingomyelin (d32:1) levels	Parkinson's disease	Inverse variance weighted	23	1.05E-02	4.08E-02	7.97E-01	1.01	0.93	1.09
Sphingomyelin (d32:1) levels	Parkinson's disease	Simple mode	23	6.59E-02	1.37E-01	6.35E-01	1.07	0.82	1.40
Sphingomyelin (d32:1) levels	Parkinson's disease	Weighted mode	23	-3.75E-02	4.12E-02	3.73E-01	0.96	0.89	1.04
Sphingomyelin (d34:0) levels	Parkinson's disease	MR Egger	18	-2.14E-01	9.42E-02	3.70E-02	0.81	0.67	0.97
Sphingomyelin (d34:0) levels	Parkinson's disease	Weighted median	18	3.05E-02	7.81E-02	6.96E-01	1.03	0.88	1.20
Sphingomyelin (d34:0) levels	Parkinson's disease	Inverse variance weighted	18	-4.22E-02	5.64E-02	4.54E-01	0.96	0.86	1.07
Sphingomyelin (d34:0) levels	Parkinson's disease	Simple mode	18	4.15E-02	1.41E-01	7.72E-01	1.04	0.79	1.37
Sphingomyelin (d34:0) levels	Parkinson's disease	Weighted mode	18	3.80E-02	1.32E-01	7.76E-01	1.04	0.80	1.34
Sphingomyelin (d34:1) levels	Parkinson's disease	MR Egger	25	2.10E-02	8.99E-02	8.18E-01	1.02	0.86	1.22
Sphingomyelin (d34:1) levels	Parkinson's disease	Weighted median	25	2.36E-02	6.82E-02	7.29E-01	1.02	0.90	1.17
Sphingomyelin (d34:1) levels	Parkinson's disease	Inverse variance weighted	25	1.12E-03	4.69E-02	9.81E-01	1.00	0.91	1.10
Sphingomyelin (d34:1) levels	Parkinson's disease	Simple mode	25	-6.37E-02	1.14E-01	5.83E-01	0.94	0.75	1.17
Sphingomyelin (d34:1) levels	Parkinson's disease	Weighted mode	25	3.43E-02	9.84E-02	7.30E-01	1.03	0.85	1.26
Sphingomyelin (d34:2) levels	Parkinson's disease	MR Egger	22	7.34E-02	9.28E-02	4.38E-01	1.08	0.90	1.29
Sphingomyelin (d34:2) levels	Parkinson's disease	Weighted median	22	-3.87E-03	7.12E-02	9.57E-01	1.00	0.87	1.15
Sphingomyelin (d34:2) levels	Parkinson's disease	Inverse variance weighted	22	3.93E-02	5.26E-02	4.56E-01	1.04	0.94	1.15
Sphingomyelin (d34:2) levels	Parkinson's disease	Simple mode	22	7.37E-02	1.09E-01	5.07E-01	1.08	0.87	1.33
Sphingomyelin (d34:2) levels	Parkinson's disease	Weighted mode	22	2.80E-02	7.68E-02	7.19E-01	1.03	0.88	1.20
Sphingomyelin (d36:1) levels	Parkinson's disease	MR Egger	27	1.63E-02	1.10E-01	8.84E-01	1.02	0.82	1.26
Sphingomyelin (d36:1) levels	Parkinson's disease	Weighted median	27	-1.06E-02	6.90E-02	8.78E-01	0.99	0.86	1.13
Sphingomyelin (d36:1) levels	Parkinson's disease	Inverse variance weighted	27	5.89E-03	4.92E-02	9.05E-01	1.01	0.91	1.11
Sphingomyelin (d36:1) levels	Parkinson's disease	Simple mode	27	3.77E-02	1.27E-01	7.69E-01	1.04	0.81	1.33
Sphingomyelin (d36:1) levels	Parkinson's disease	Weighted mode	27	3.07E-02	1.16E-01	7.94E-01	1.03	0.82	1.30
Sphingomyelin (d36:2) levels	Parkinson's disease	MR Egger	17	9.17E-03	1.59E-01	9.55E-01	1.01	0.74	1.38
Sphingomyelin (d36:2) levels	Parkinson's disease	Weighted median	17	5.99E-02	8.42E-02	4.77E-01	1.06	0.90	1.25
Sphingomyelin (d36:2) levels	Parkinson's disease	Inverse variance weighted	17	9.66E-02	5.65E-02	8.74E-02	1.10	0.99	1.23
Sphingomyelin (d36:2) levels	Parkinson's disease	Simple mode	17	2.55E-01	1.28E-01	6.25E-02	1.29	1.01	1.66
Sphingomyelin (d36:2) levels	Parkinson's disease	Weighted mode	17	9.56E-02	8.80E-02	2.94E-01	1.10	0.93	1.31
Sphingomyelin (d38:1) levels	Parkinson's disease	MR Egger	25	-8.65E-02	6.99E-02	2.28E-01	0.92	0.80	1.05
Sphingomyelin (d38:1) levels	Parkinson's disease	Weighted median	25	-8.63E-02	5.90E-02	1.43E-01	0.92	0.82	1.03
Sphingomyelin (d38:1) levels	Parkinson's disease	Inverse variance weighted	25	-9.15E-02	4.07E-02	2.46E-02	0.91	0.84	0.99
Sphingomyelin (d38:1) levels	Parkinson's disease	Simple mode	25	-4.37E-02	1.23E-01	7.24E-01	0.96	0.75	1.22
Sphingomyelin (d38:1) levels	Parkinson's disease	Weighted mode	25	-7.96E-02	5.70E-02	1.75E-01	0.92	0.83	1.03
Sphingomyelin (d38:2) levels	Parkinson's disease	MR Egger	21	8.75E-02	8.71E-02	3.28E-01	1.09	0.92	1.29
Sphingomyelin (d38:2) levels	Parkinson's disease	Weighted median	21	2.84E-03	6.82E-02	9.67E-01	1.00	0.88	1.15
Sphingomyelin (d38:2) levels	Parkinson's disease	Inverse variance weighted	21	2.90E-02	4.78E-02	5.45E-01	1.03	0.94	1.13

Sphingomyelin (d38:2) levels	Parkinson's disease	Simple mode	21	-9.09E-02	1.03E-01	3.87E-01	0.91	0.75	1.12
Sphingomyelin (d38:2) levels	Parkinson's disease	Weighted mode	21	-8.65E-03	7.31E-02	9.07E-01	0.99	0.86	1.14
Sphingomyelin (d40:1) levels	Parkinson's disease	MR Egger	34	-1.52E-01	8.21E-02	7.42E-02	0.86	0.73	1.01
Sphingomyelin (d40:1) levels	Parkinson's disease	Weighted median	34	-1.04E-01	6.26E-02	9.60E-02	0.90	0.80	1.02
Sphingomyelin (d40:1) levels	Parkinson's disease	Inverse variance weighted	34	-1.03E-01	4.42E-02	2.03E-02	0.90	0.83	0.98
Sphingomyelin (d40:1) levels	Parkinson's disease	Simple mode	34	3.37E-02	1.19E-01	7.79E-01	1.03	0.82	1.31
Sphingomyelin (d40:1) levels	Parkinson's disease	Weighted mode	34	-1.24E-01	9.35E-02	1.95E-01	0.88	0.74	1.06
Sphingomyelin (d40:2) levels	Parkinson's disease	MR Egger	29	-1.57E-01	7.79E-02	5.34E-02	0.85	0.73	1.00
Sphingomyelin (d40:2) levels	Parkinson's disease	Weighted median	29	-7.01E-02	6.07E-02	2.48E-01	0.93	0.83	1.05
Sphingomyelin (d40:2) levels	Parkinson's disease	Inverse variance weighted	29	-2.67E-02	4.06E-02	5.12E-01	0.97	0.90	1.05
Sphingomyelin (d40:2) levels	Parkinson's disease	Simple mode	29	8.99E-02	1.15E-01	4.39E-01	1.09	0.87	1.37
Sphingomyelin (d40:2) levels	Parkinson's disease	Weighted mode	29	-6.60E-02	5.99E-02	2.80E-01	0.94	0.83	1.05
Sphingomyelin (d42:2) levels	Parkinson's disease	MR Egger	31	-1.41E-01	8.93E-02	1.25E-01	0.87	0.73	1.03
Sphingomyelin (d42:2) levels	Parkinson's disease	Weighted median	31	-5.61E-03	6.48E-02	9.31E-01	0.99	0.88	1.13
Sphingomyelin (d42:2) levels	Parkinson's disease	Inverse variance weighted	31	1.65E-02	4.45E-02	7.12E-01	1.02	0.93	1.11
Sphingomyelin (d42:2) levels	Parkinson's disease	Simple mode	31	2.04E-02	1.10E-01	8.55E-01	1.02	0.82	1.27
Sphingomyelin (d42:2) levels	Parkinson's disease	Weighted mode	31	-5.03E-03	1.04E-01	9.62E-01	0.99	0.81	1.22
Triacylglycerol (46:1) levels	Parkinson's disease	MR Egger	14	-8.67E-02	1.65E-01	6.09E-01	0.92	0.66	1.27
Triacylglycerol (46:1) levels	Parkinson's disease	Weighted median	14	6.28E-02	9.99E-02	5.30E-01	1.06	0.88	1.30
Triacylglycerol (46:1) levels	Parkinson's disease	Inverse variance weighted	14	1.16E-01	9.77E-02	2.35E-01	1.12	0.93	1.36
Triacylglycerol (46:1) levels	Parkinson's disease	Simple mode	14	9.78E-03	2.22E-01	9.66E-01	1.01	0.65	1.56
Triacylglycerol (46:1) levels	Parkinson's disease	Weighted mode	14	3.01E-02	1.17E-01	8.01E-01	1.03	0.82	1.30
Triacylglycerol (46:2) levels	Parkinson's disease	MR Egger	18	9.06E-02	1.76E-01	6.13E-01	1.09	0.78	1.55
Triacylglycerol (46:2) levels	Parkinson's disease	Weighted median	18	7.79E-02	9.60E-02	4.17E-01	1.08	0.90	1.30
Triacylglycerol (46:2) levels	Parkinson's disease	Inverse variance weighted	18	1.17E-01	8.06E-02	1.47E-01	1.12	0.96	1.32
Triacylglycerol (46:2) levels	Parkinson's disease	Simple mode	18	1.14E-02	1.74E-01	9.48E-01	1.01	0.72	1.42
Triacylglycerol (46:2) levels	Parkinson's disease	Weighted mode	18	6.81E-02	1.42E-01	6.37E-01	1.07	0.81	1.41
Triacylglycerol (48:0) levels	Parkinson's disease	MR Egger	13	2.26E-02	1.92E-01	9.08E-01	1.02	0.70	1.49
Triacylglycerol (48:0) levels	Parkinson's disease	Weighted median	13	2.07E-01	1.03E-01	4.35E-02	1.23	1.01	1.50
Triacylglycerol (48:0) levels	Parkinson's disease	Inverse variance weighted	13	2.14E-01	8.33E-02	1.01E-02	1.24	1.05	1.46
Triacylglycerol (48:0) levels	Parkinson's disease	Simple mode	13	3.21E-01	1.94E-01	1.23E-01	1.38	0.94	2.01
Triacylglycerol (48:0) levels	Parkinson's disease	Weighted mode	13	2.65E-01	1.87E-01	1.82E-01	1.30	0.90	1.88
Triacylglycerol (48:1) levels	Parkinson's disease	MR Egger	20	5.57E-02	1.88E-01	7.70E-01	1.06	0.73	1.53
Triacylglycerol (48:1) levels	Parkinson's disease	Weighted median	20	1.00E-01	9.13E-02	2.73E-01	1.11	0.92	1.32
Triacylglycerol (48:1) levels	Parkinson's disease	Inverse variance weighted	20	1.12E-01	7.25E-02	1.23E-01	1.12	0.97	1.29
Triacylglycerol (48:1) levels	Parkinson's disease	Simple mode	20	8.90E-02	1.73E-01	6.13E-01	1.09	0.78	1.53
Triacylglycerol (48:1) levels	Parkinson's disease	Weighted mode	20	1.06E-01	2.04E-01	6.10E-01	1.11	0.75	1.66
Triacylglycerol (48:2) levels	Parkinson's disease	MR Egger	16	6.03E-01	2.05E-01	1.07E-02	1.83	1.22	2.73
Triacylglycerol (48:2) levels	Parkinson's disease	Weighted median	16	1.52E-01	1.04E-01	1.45E-01	1.16	0.95	1.43
Triacylglycerol (48:2) levels	Parkinson's disease	Inverse variance weighted	16	1.66E-01	7.71E-02	3.13E-02	1.18	1.02	1.37
Triacylglycerol (48:2) levels	Parkinson's disease	Simple mode	16	1.55E-01	2.19E-01	4.90E-01	1.17	0.76	1.79
Triacylglycerol (48:2) levels	Parkinson's disease	Weighted mode	16	3.36E-01	1.37E-01	2.75E-02	1.40	1.07	1.83
Triacylglycerol (48:3) levels	Parkinson's disease	MR Egger	18	3.01E-01	2.37E-01	2.23E-01	1.35	0.85	2.15
Triacylglycerol (48:3) levels	Parkinson's disease	Weighted median	18	1.15E-01	9.68E-02	2.36E-01	1.12	0.93	1.36
Triacylglycerol (48:3) levels	Parkinson's disease	Inverse variance weighted	18	1.32E-01	6.83E-02	5.36E-02	1.14	1.00	1.30
Triacylglycerol (48:3) levels	Parkinson's disease	Simple mode	18	6.58E-02	1.74E-01	7.11E-01	1.07	0.76	1.50

Triacylglycerol (48:3) levels	Parkinson's disease	Weighted mode	18	2.86E-01	1.75E-01	1.19E-01	1.33	0.95	1.87
Triacylglycerol (49:1) levels	Parkinson's disease	MR Egger	21	7.85E-02	1.68E-01	6.46E-01	1.08	0.78	1.50
Triacylglycerol (49:1) levels	Parkinson's disease	Weighted median	21	6.85E-02	7.85E-02	3.83E-01	1.07	0.92	1.25
Triacylglycerol (49:1) levels	Parkinson's disease	Inverse variance weighted	21	9.49E-02	6.79E-02	1.63E-01	1.10	0.96	1.26
Triacylglycerol (49:1) levels	Parkinson's disease	Simple mode	21	9.63E-02	1.65E-01	5.67E-01	1.10	0.80	1.52
Triacylglycerol (49:1) levels	Parkinson's disease	Weighted mode	21	7.72E-02	1.35E-01	5.74E-01	1.08	0.83	1.41
Triacylglycerol (49:2) levels	Parkinson's disease	MR Egger	10	1.38E-01	1.47E-01	3.77E-01	1.15	0.86	1.53
Triacylglycerol (49:2) levels	Parkinson's disease	Weighted median	10	2.52E-01	9.40E-02	7.37E-03	1.29	1.07	1.55
Triacylglycerol (49:2) levels	Parkinson's disease	Inverse variance weighted	10	2.75E-01	7.04E-02	9.41E-05	1.32	1.15	1.51
Triacylglycerol (49:2) levels	Parkinson's disease	Simple mode	10	3.14E-01	1.69E-01	9.56E-02	1.37	0.98	1.91
Triacylglycerol (49:2) levels	Parkinson's disease	Weighted mode	10	3.11E-01	1.56E-01	7.68E-02	1.37	1.01	1.85
Triacylglycerol (50:1) levels	Parkinson's disease	MR Egger	22	6.83E-02	1.37E-01	6.23E-01	1.07	0.82	1.40
Triacylglycerol (50:1) levels	Parkinson's disease	Weighted median	22	1.55E-02	8.13E-02	8.49E-01	1.02	0.87	1.19
Triacylglycerol (50:1) levels	Parkinson's disease	Inverse variance weighted	22	3.90E-02	6.84E-02	5.69E-01	1.04	0.91	1.19
Triacylglycerol (50:1) levels	Parkinson's disease	Simple mode	22	-2.16E-01	1.64E-01	2.02E-01	0.81	0.58	1.11
Triacylglycerol (50:1) levels	Parkinson's disease	Weighted mode	22	-1.71E-01	1.77E-01	3.43E-01	0.84	0.60	1.19
Triacylglycerol (50:2) levels	Parkinson's disease	MR Egger	14	3.35E-01	1.53E-01	4.91E-02	1.40	1.04	1.89
Triacylglycerol (50:2) levels	Parkinson's disease	Weighted median	14	2.54E-01	9.03E-02	4.92E-03	1.29	1.08	1.54
Triacylglycerol (50:2) levels	Parkinson's disease	Inverse variance weighted	14	2.20E-01	6.43E-02	6.34E-04	1.25	1.10	1.41
Triacylglycerol (50:2) levels	Parkinson's disease	Simple mode	14	1.91E-01	1.91E-01	3.36E-01	1.21	0.83	1.76
Triacylglycerol (50:2) levels	Parkinson's disease	Weighted mode	14	2.79E-01	1.22E-01	4.03E-02	1.32	1.04	1.68
Triacylglycerol (50:3) levels	Parkinson's disease	MR Egger	25	1.80E-01	1.16E-01	1.35E-01	1.20	0.95	1.50
Triacylglycerol (50:3) levels	Parkinson's disease	Weighted median	25	9.99E-02	6.87E-02	1.46E-01	1.11	0.97	1.26
Triacylglycerol (50:3) levels	Parkinson's disease	Inverse variance weighted	25	7.13E-02	5.45E-02	1.91E-01	1.07	0.97	1.19
Triacylglycerol (50:3) levels	Parkinson's disease	Simple mode	25	1.15E-01	1.67E-01	4.99E-01	1.12	0.81	1.56
Triacylglycerol (50:3) levels	Parkinson's disease	Weighted mode	25	2.41E-01	1.51E-01	1.24E-01	1.27	0.95	1.71
Triacylglycerol (50:4) levels	Parkinson's disease	MR Egger	18	5.62E-01	1.47E-01	1.52E-03	1.75	1.31	2.34
Triacylglycerol (50:4) levels	Parkinson's disease	Weighted median	18	6.04E-02	8.60E-02	4.82E-01	1.06	0.90	1.26
Triacylglycerol (50:4) levels	Parkinson's disease	Inverse variance weighted	18	1.04E-01	6.33E-02	1.00E-01	1.11	0.98	1.26
Triacylglycerol (50:4) levels	Parkinson's disease	Simple mode	18	-1.33E-01	1.58E-01	4.12E-01	0.88	0.64	1.19
Triacylglycerol (50:4) levels	Parkinson's disease	Weighted mode	18	3.18E-01	1.13E-01	1.17E-02	1.37	1.10	1.71
Triacylglycerol (50:5) levels	Parkinson's disease	MR Egger	19	-1.15E-01	8.14E-02	1.77E-01	0.89	0.76	1.05
Triacylglycerol (50:5) levels	Parkinson's disease	Weighted median	19	6.99E-02	6.99E-02	3.17E-01	1.07	0.94	1.23
Triacylglycerol (50:5) levels	Parkinson's disease	Inverse variance weighted	19	5.98E-02	5.40E-02	2.68E-01	1.06	0.96	1.18
Triacylglycerol (50:5) levels	Parkinson's disease	Simple mode	19	4.56E-02	1.38E-01	7.45E-01	1.05	0.80	1.37
Triacylglycerol (50:5) levels	Parkinson's disease	Weighted mode	19	6.28E-02	1.50E-01	6.81E-01	1.06	0.79	1.43
Triacylglycerol (51:1) levels	Parkinson's disease	MR Egger	19	-1.05E-01	1.85E-01	5.79E-01	0.90	0.63	1.29
Triacylglycerol (51:1) levels	Parkinson's disease	Weighted median	19	6.34E-02	8.35E-02	4.48E-01	1.07	0.90	1.25
Triacylglycerol (51:1) levels	Parkinson's disease	Inverse variance weighted	19	5.54E-02	7.73E-02	4.73E-01	1.06	0.91	1.23
Triacylglycerol (51:1) levels	Parkinson's disease	Simple mode	19	3.87E-02	1.58E-01	8.09E-01	1.04	0.76	1.42
Triacylglycerol (51:1) levels	Parkinson's disease	Weighted mode	19	6.34E-02	1.54E-01	6.84E-01	1.07	0.79	1.44
Triacylglycerol (51:2) levels	Parkinson's disease	MR Egger	19	1.14E-01	1.29E-01	3.90E-01	1.12	0.87	1.44
Triacylglycerol (51:2) levels	Parkinson's disease	Weighted median	19	6.82E-02	8.23E-02	4.07E-01	1.07	0.91	1.26
Triacylglycerol (51:2) levels	Parkinson's disease	Inverse variance weighted	19	1.29E-01	5.90E-02	2.87E-02	1.14	1.01	1.28
Triacylglycerol (51:2) levels	Parkinson's disease	Simple mode	19	-1.16E-01	1.80E-01	5.27E-01	0.89	0.63	1.27
Triacylglycerol (51:2) levels	Parkinson's disease	Weighted mode	19	-9.22E-02	1.55E-01	5.58E-01	0.91	0.67	1.23

Triacylglycerol (51:3) levels	Parkinson's disease	MR Egger	21	-3.86E-02	1.18E-01	7.47E-01	0.96	0.76	1.21
Triacylglycerol (51:3) levels	Parkinson's disease	Weighted median	21	1.77E-01	7.38E-02	1.63E-02	1.19	1.03	1.38
Triacylglycerol (51:3) levels	Parkinson's disease	Inverse variance weighted	21	1.27E-01	6.08E-02	3.66E-02	1.14	1.01	1.28
Triacylglycerol (51:3) levels	Parkinson's disease	Simple mode	21	1.25E-01	1.44E-01	3.97E-01	1.13	0.85	1.50
Triacylglycerol (51:3) levels	Parkinson's disease	Weighted mode	21	1.96E-01	1.12E-01	9.67E-02	1.22	0.98	1.52
Triacylglycerol (51:4) levels	Parkinson's disease	MR Egger	16	-8.23E-02	1.53E-01	5.99E-01	0.92	0.68	1.24
Triacylglycerol (51:4) levels	Parkinson's disease	Weighted median	16	1.60E-01	7.55E-02	3.45E-02	1.17	1.01	1.36
Triacylglycerol (51:4) levels	Parkinson's disease	Inverse variance weighted	16	9.50E-02	7.20E-02	1.87E-01	1.10	0.96	1.27
Triacylglycerol (51:4) levels	Parkinson's disease	Simple mode	16	9.88E-02	1.77E-01	5.86E-01	1.10	0.78	1.56
Triacylglycerol (51:4) levels	Parkinson's disease	Weighted mode	16	1.76E-01	1.13E-01	1.40E-01	1.19	0.96	1.49
Triacylglycerol (52:2) levels	Parkinson's disease	MR Egger	20	1.98E-01	1.11E-01	9.13E-02	1.22	0.98	1.51
Triacylglycerol (52:2) levels	Parkinson's disease	Weighted median	20	2.27E-01	7.49E-02	2.48E-03	1.25	1.08	1.45
Triacylglycerol (52:2) levels	Parkinson's disease	Inverse variance weighted	20	1.90E-01	5.05E-02	1.70E-04	1.21	1.10	1.34
Triacylglycerol (52:2) levels	Parkinson's disease	Simple mode	20	2.05E-01	1.41E-01	1.62E-01	1.23	0.93	1.62
Triacylglycerol (52:2) levels	Parkinson's disease	Weighted mode	20	2.23E-01	9.36E-02	2.76E-02	1.25	1.04	1.50
Triacylglycerol (52:3) levels	Parkinson's disease	MR Egger	26	1.35E-01	9.70E-02	1.77E-01	1.14	0.95	1.38
Triacylglycerol (52:3) levels	Parkinson's disease	Weighted median	26	8.68E-02	6.28E-02	1.67E-01	1.09	0.96	1.23
Triacylglycerol (52:3) levels	Parkinson's disease	Inverse variance weighted	26	7.51E-02	4.58E-02	1.01E-01	1.08	0.99	1.18
Triacylglycerol (52:3) levels	Parkinson's disease	Simple mode	26	5.97E-02	1.19E-01	6.20E-01	1.06	0.84	1.34
Triacylglycerol (52:3) levels	Parkinson's disease	Weighted mode	26	1.30E-01	6.96E-02	7.45E-02	1.14	0.99	1.30
Triacylglycerol (52:4) levels	Parkinson's disease	MR Egger	20	3.37E-01	1.28E-01	1.71E-02	1.40	1.09	1.80
Triacylglycerol (52:4) levels	Parkinson's disease	Weighted median	20	1.91E-01	6.98E-02	6.24E-03	1.21	1.06	1.39
Triacylglycerol (52:4) levels	Parkinson's disease	Inverse variance weighted	20	1.03E-01	5.81E-02	7.76E-02	1.11	0.99	1.24
Triacylglycerol (52:4) levels	Parkinson's disease	Simple mode	20	3.29E-02	1.30E-01	8.03E-01	1.03	0.80	1.33
Triacylglycerol (52:4) levels	Parkinson's disease	Weighted mode	20	1.27E-01	7.83E-02	1.20E-01	1.14	0.97	1.32
Triacylglycerol (52:5) levels	Parkinson's disease	MR Egger	21	3.06E-01	1.45E-01	4.77E-02	1.36	1.02	1.80
Triacylglycerol (52:5) levels	Parkinson's disease	Weighted median	21	1.40E-01	7.07E-02	4.73E-02	1.15	1.00	1.32
Triacylglycerol (52:5) levels	Parkinson's disease	Inverse variance weighted	21	1.17E-01	5.51E-02	3.37E-02	1.12	1.01	1.25
Triacylglycerol (52:5) levels	Parkinson's disease	Simple mode	21	-1.20E-02	1.37E-01	9.31E-01	0.99	0.76	1.29
Triacylglycerol (52:5) levels	Parkinson's disease	Weighted mode	21	9.84E-02	9.90E-02	3.32E-01	1.10	0.91	1.34
Triacylglycerol (52:6) levels	Parkinson's disease	MR Egger	17	1.24E-01	1.59E-01	4.45E-01	1.13	0.83	1.55
Triacylglycerol (52:6) levels	Parkinson's disease	Weighted median	17	6.58E-02	7.68E-02	3.92E-01	1.07	0.92	1.24
Triacylglycerol (52:6) levels	Parkinson's disease	Inverse variance weighted	17	1.23E-01	5.76E-02	3.30E-02	1.13	1.01	1.27
Triacylglycerol (52:6) levels	Parkinson's disease	Simple mode	17	3.10E-02	1.45E-01	8.33E-01	1.03	0.78	1.37
Triacylglycerol (52:6) levels	Parkinson's disease	Weighted mode	17	3.40E-02	1.35E-01	8.04E-01	1.03	0.79	1.35
Triacylglycerol (53:2) levels	Parkinson's disease	MR Egger	19	1.73E-01	1.68E-01	3.18E-01	1.19	0.85	1.65
Triacylglycerol (53:2) levels	Parkinson's disease	Weighted median	19	2.18E-01	7.89E-02	5.74E-03	1.24	1.07	1.45
Triacylglycerol (53:2) levels	Parkinson's disease	Inverse variance weighted	19	1.57E-01	5.81E-02	6.92E-03	1.17	1.04	1.31
Triacylglycerol (53:2) levels	Parkinson's disease	Simple mode	19	2.04E-01	1.61E-01	2.22E-01	1.23	0.89	1.68
Triacylglycerol (53:2) levels	Parkinson's disease	Weighted mode	19	2.26E-01	1.10E-01	5.40E-02	1.25	1.01	1.55
Triacylglycerol (53:3) levels	Parkinson's disease	MR Egger	22	1.55E-01	1.32E-01	2.55E-01	1.17	0.90	1.51
Triacylglycerol (53:3) levels	Parkinson's disease	Weighted median	22	1.64E-01	7.00E-02	1.93E-02	1.18	1.03	1.35
Triacylglycerol (53:3) levels	Parkinson's disease	Inverse variance weighted	22	1.09E-01	5.44E-02	4.45E-02	1.12	1.00	1.24
Triacylglycerol (53:3) levels	Parkinson's disease	Simple mode	22	1.34E-01	1.42E-01	3.53E-01	1.14	0.87	1.51
Triacylglycerol (53:3) levels	Parkinson's disease	Weighted mode	22	1.74E-01	8.71E-02	5.85E-02	1.19	1.00	1.41
Triacylglycerol (53:4) levels	Parkinson's disease	MR Egger	25	1.52E-01	9.60E-02	1.26E-01	1.16	0.96	1.41

Triacylglycerol (53:4) levels	Parkinson's disease	Weighted median	25	9.80E-02	5.96E-02	9.98E-02	1.10	0.98	1.24
Triacylglycerol (53:4) levels	Parkinson's disease	Inverse variance weighted	25	1.09E-01	4.74E-02	2.12E-02	1.12	1.02	1.22
Triacylglycerol (53:4) levels	Parkinson's disease	Simple mode	25	5.03E-02	1.10E-01	6.52E-01	1.05	0.85	1.30
Triacylglycerol (53:4) levels	Parkinson's disease	Weighted mode	25	1.05E-01	8.55E-02	2.32E-01	1.11	0.94	1.31
Triacylglycerol (54:3) levels	Parkinson's disease	MR Egger	29	1.68E-01	1.27E-01	1.95E-01	1.18	0.92	1.52
Triacylglycerol (54:3) levels	Parkinson's disease	Weighted median	29	-1.74E-03	6.60E-02	9.79E-01	1.00	0.88	1.14
Triacylglycerol (54:3) levels	Parkinson's disease	Inverse variance weighted	29	4.73E-02	4.90E-02	3.35E-01	1.05	0.95	1.15
Triacylglycerol (54:3) levels	Parkinson's disease	Simple mode	29	-3.67E-02	1.14E-01	7.50E-01	0.96	0.77	1.21
Triacylglycerol (54:3) levels	Parkinson's disease	Weighted mode	29	3.22E-02	8.45E-02	7.06E-01	1.03	0.88	1.22
Triacylglycerol (54:4) levels	Parkinson's disease	MR Egger	21	2.29E-01	1.26E-01	8.40E-02	1.26	0.98	1.61
Triacylglycerol (54:4) levels	Parkinson's disease	Weighted median	21	-4.77E-03	6.83E-02	9.44E-01	1.00	0.87	1.14
Triacylglycerol (54:4) levels	Parkinson's disease	Inverse variance weighted	21	3.75E-02	5.22E-02	4.72E-01	1.04	0.94	1.15
Triacylglycerol (54:4) levels	Parkinson's disease	Simple mode	21	-3.41E-02	1.16E-01	7.71E-01	0.97	0.77	1.21
Triacylglycerol (54:4) levels	Parkinson's disease	Weighted mode	21	-3.71E-02	8.08E-02	6.51E-01	0.96	0.82	1.13
Triacylglycerol (54:5) levels	Parkinson's disease	MR Egger	20	6.51E-02	1.29E-01	6.19E-01	1.07	0.83	1.37
Triacylglycerol (54:5) levels	Parkinson's disease	Weighted median	20	7.94E-02	6.98E-02	2.55E-01	1.08	0.94	1.24
Triacylglycerol (54:5) levels	Parkinson's disease	Inverse variance weighted	20	1.03E-01	5.51E-02	6.13E-02	1.11	1.00	1.23
Triacylglycerol (54:5) levels	Parkinson's disease	Simple mode	20	5.23E-02	1.30E-01	6.91E-01	1.05	0.82	1.36
Triacylglycerol (54:5) levels	Parkinson's disease	Weighted mode	20	9.58E-02	9.43E-02	3.22E-01	1.10	0.91	1.32
Triacylglycerol (54:6) levels	Parkinson's disease	MR Egger	16	1.18E-01	1.05E-01	2.82E-01	1.12	0.92	1.38
Triacylglycerol (54:6) levels	Parkinson's disease	Weighted median	16	1.42E-01	7.40E-02	5.49E-02	1.15	1.00	1.33
Triacylglycerol (54:6) levels	Parkinson's disease	Inverse variance weighted	16	1.45E-01	5.61E-02	9.62E-03	1.16	1.04	1.29
Triacylglycerol (54:6) levels	Parkinson's disease	Simple mode	16	5.77E-03	1.30E-01	9.65E-01	1.01	0.78	1.30
Triacylglycerol (54:6) levels	Parkinson's disease	Weighted mode	16	1.70E-01	1.02E-01	1.16E-01	1.19	0.97	1.45
Triacylglycerol (54:7) levels	Parkinson's disease	MR Egger	22	-1.38E-01	1.32E-01	3.10E-01	0.87	0.67	1.13
Triacylglycerol (54:7) levels	Parkinson's disease	Weighted median	22	2.12E-02	7.53E-02	7.79E-01	1.02	0.88	1.18
Triacylglycerol (54:7) levels	Parkinson's disease	Inverse variance weighted	22	2.72E-02	6.18E-02	6.60E-01	1.03	0.91	1.16
Triacylglycerol (54:7) levels	Parkinson's disease	Simple mode	22	-1.03E-01	1.51E-01	5.02E-01	0.90	0.67	1.21
Triacylglycerol (54:7) levels	Parkinson's disease	Weighted mode	22	-7.95E-02	1.59E-01	6.23E-01	0.92	0.68	1.26
Triacylglycerol (56:3) levels	Parkinson's disease	MR Egger	21	1.58E-01	1.21E-01	2.09E-01	1.17	0.92	1.49
Triacylglycerol (56:3) levels	Parkinson's disease	Weighted median	21	2.77E-02	6.79E-02	6.84E-01	1.03	0.90	1.17
Triacylglycerol (56:3) levels	Parkinson's disease	Inverse variance weighted	21	1.35E-02	5.52E-02	8.07E-01	1.01	0.91	1.13
Triacylglycerol (56:3) levels	Parkinson's disease	Simple mode	21	1.02E-01	1.22E-01	4.12E-01	1.11	0.87	1.41
Triacylglycerol (56:3) levels	Parkinson's disease	Weighted mode	21	1.16E-01	8.75E-02	2.00E-01	1.12	0.95	1.33
Triacylglycerol (56:4) levels	Parkinson's disease	MR Egger	14	2.54E-01	1.55E-01	1.27E-01	1.29	0.95	1.75
Triacylglycerol (56:4) levels	Parkinson's disease	Weighted median	14	6.25E-02	8.23E-02	4.48E-01	1.06	0.91	1.25
Triacylglycerol (56:4) levels	Parkinson's disease	Inverse variance weighted	14	4.24E-02	6.20E-02	4.95E-01	1.04	0.92	1.18
Triacylglycerol (56:4) levels	Parkinson's disease	Simple mode	14	7.05E-03	1.35E-01	9.59E-01	1.01	0.77	1.31
Triacylglycerol (56:4) levels	Parkinson's disease	Weighted mode	14	1.24E-01	8.90E-02	1.86E-01	1.13	0.95	1.35
Triacylglycerol (56:5) levels	Parkinson's disease	MR Egger	17	2.73E-01	1.13E-01	2.89E-02	1.31	1.05	1.64
Triacylglycerol (56:5) levels	Parkinson's disease	Weighted median	17	5.52E-02	6.78E-02	4.15E-01	1.06	0.93	1.21
Triacylglycerol (56:5) levels	Parkinson's disease	Inverse variance weighted	17	5.40E-02	5.43E-02	3.20E-01	1.06	0.95	1.17
Triacylglycerol (56:5) levels	Parkinson's disease	Simple mode	17	9.24E-02	1.04E-01	3.88E-01	1.10	0.89	1.35
Triacylglycerol (56:5) levels	Parkinson's disease	Weighted mode	17	1.19E-01	8.83E-02	1.95E-01	1.13	0.95	1.34
Triacylglycerol (56:6) levels	Parkinson's disease	MR Egger	22	-7.71E-02	8.71E-02	3.86E-01	0.93	0.78	1.10
Triacylglycerol (56:6) levels	Parkinson's disease	Weighted median	22	1.79E-02	5.88E-02	7.61E-01	1.02	0.91	1.14

Triacylglycerol (56:6) levels	Parkinson's disease	Inverse variance weighted	22	9.28E-02	4.18E-02	2.64E-02	1.10	1.01	1.19
Triacylglycerol (56:6) levels	Parkinson's disease	Simple mode	22	2.50E-02	1.03E-01	8.11E-01	1.03	0.84	1.26
Triacylglycerol (56:6) levels	Parkinson's disease	Weighted mode	22	5.74E-03	6.93E-02	9.35E-01	1.01	0.88	1.15
Triacylglycerol (56:7) levels	Parkinson's disease	MR Egger	26	8.09E-02	9.85E-02	4.20E-01	1.08	0.89	1.32
Triacylglycerol (56:7) levels	Parkinson's disease	Weighted median	26	6.78E-02	5.92E-02	2.53E-01	1.07	0.95	1.20
Triacylglycerol (56:7) levels	Parkinson's disease	Inverse variance weighted	26	7.87E-02	4.34E-02	6.94E-02	1.08	0.99	1.18
Triacylglycerol (56:7) levels	Parkinson's disease	Simple mode	26	3.52E-02	1.07E-01	7.45E-01	1.04	0.84	1.28
Triacylglycerol (56:7) levels	Parkinson's disease	Weighted mode	26	3.20E-02	8.74E-02	7.17E-01	1.03	0.87	1.23
Triacylglycerol (56:8) levels	Parkinson's disease	MR Egger	27	2.96E-02	1.10E-01	7.90E-01	1.03	0.83	1.28
Triacylglycerol (56:8) levels	Parkinson's disease	Weighted median	27	7.13E-03	6.18E-02	9.08E-01	1.01	0.89	1.14
Triacylglycerol (56:8) levels	Parkinson's disease	Inverse variance weighted	27	7.87E-02	4.84E-02	1.03E-01	1.08	0.98	1.19
Triacylglycerol (56:8) levels	Parkinson's disease	Simple mode	27	-3.14E-02	1.07E-01	7.70E-01	0.97	0.79	1.19
Triacylglycerol (56:8) levels	Parkinson's disease	Weighted mode	27	-8.41E-03	8.80E-02	9.25E-01	0.99	0.83	1.18
Triacylglycerol (58:7) levels	Parkinson's disease	MR Egger	10	2.02E-01	2.72E-01	4.79E-01	1.22	0.72	2.08
Triacylglycerol (58:7) levels	Parkinson's disease	Weighted median	10	7.63E-02	8.79E-02	3.86E-01	1.08	0.91	1.28
Triacylglycerol (58:7) levels	Parkinson's disease	Inverse variance weighted	10	1.29E-01	8.68E-02	1.37E-01	1.14	0.96	1.35
Triacylglycerol (58:7) levels	Parkinson's disease	Simple mode	10	-1.06E-01	1.66E-01	5.41E-01	0.90	0.65	1.25
Triacylglycerol (58:7) levels	Parkinson's disease	Weighted mode	10	6.23E-03	1.26E-01	9.62E-01	1.01	0.79	1.29
Triacylglycerol (58:8) levels	Parkinson's disease	MR Egger	25	-1.58E-03	9.81E-02	9.87E-01	1.00	0.82	1.21
Triacylglycerol (58:8) levels	Parkinson's disease	Weighted median	25	-4.02E-04	6.81E-02	9.95E-01	1.00	0.87	1.14
Triacylglycerol (58:8) levels	Parkinson's disease	Inverse variance weighted	25	3.56E-02	4.68E-02	4.47E-01	1.04	0.95	1.14
Triacylglycerol (58:8) levels	Parkinson's disease	Simple mode	25	-2.18E-02	1.33E-01	8.71E-01	0.98	0.75	1.27
Triacylglycerol (58:8) levels	Parkinson's disease	Weighted mode	25	-4.20E-02	1.21E-01	7.31E-01	0.96	0.76	1.22

Detection of horizontal pleiotropy by Egger intercept				
**exposure**	**outcome**	**egger_intercept**	**se**	**pval**
Sterol ester (27:1/14:0) levels	Parkinson's disease	-2.24E-02	1.81E-02	0.24
Sterol ester (27:1/15:0) levels	Parkinson's disease	2.08E-02	1.31E-02	0.14
Sterol ester (27:1/16:0) levels	Parkinson's disease	-8.67E-03	1.06E-02	0.42
Sterol ester (27:1/16:1) levels	Parkinson's disease	1.50E-02	1.63E-02	0.37
Sterol ester (27:1/17:0) levels	Parkinson's disease	5.86E-04	1.89E-02	0.98
Sterol ester (27:1/17:1) levels	Parkinson's disease	2.29E-02	1.95E-02	0.27
Sterol ester (27:1/18:0) levels	Parkinson's disease	1.29E-02	1.79E-02	0.48
Sterol ester (27:1/18:1) levels	Parkinson's disease	-5.77E-03	1.35E-02	0.67
Sterol ester (27:1/18:2) levels	Parkinson's disease	-3.27E-02	1.50E-02	0.04
Sterol ester (27:1/18:3) levels	Parkinson's disease	2.28E-02	2.26E-02	0.34
Sterol ester (27:1/20:2) levels	Parkinson's disease	-1.72E-03	1.61E-02	0.92
Sterol ester (27:1/20:3) levels	Parkinson's disease	-1.50E-02	1.70E-02	0.39
Sterol ester (27:1/20:4) levels	Parkinson's disease	1.98E-03	1.06E-02	0.85
Sterol ester (27:1/20:5) levels	Parkinson's disease	-1.98E-02	1.20E-02	0.11
Sterol ester (27:1/22:6) levels	Parkinson's disease	3.09E-02	1.91E-02	0.12
Ceramide (d40:1) levels	Parkinson's disease	8.96E-03	2.94E-02	0.77
Ceramide (d40:2) levels	Parkinson's disease	1.58E-02	2.31E-02	0.50
Ceramide (d42:1) levels	Parkinson's disease	-2.09E-04	2.56E-02	0.99
Ceramide (d42:2) levels	Parkinson's disease	5.48E-03	2.92E-02	0.85
Cholesterol levels	Parkinson's disease	-1.78E-02	1.58E-02	0.27
Diacylglycerol (16:0_18:1) levels	Parkinson's disease	2.00E-02	2.24E-02	0.39
Diacylglycerol (16:0_18:2) levels	Parkinson's disease	1.34E-02	1.61E-02	0.41
Diacylglycerol (16:1_18:1) levels	Parkinson's disease	2.39E-02	2.36E-02	0.33
Diacylglycerol (18:1_18:1) levels	Parkinson's disease	-3.89E-03	1.55E-02	0.80
Diacylglycerol (18:1_18:2) levels	Parkinson's disease	1.48E-03	1.41E-02	0.92
Diacylglycerol (18:1_18:3) levels	Parkinson's disease	3.13E-02	2.34E-02	0.20
Lysophosphatidylcholine (16:0) levels	Parkinson's disease	-8.28E-03	1.89E-02	0.67
Lysophosphatidylcholine (18:0) levels	Parkinson's disease	-1.40E-02	2.09E-02	0.51
Lysophosphatidylcholine (18:1) levels	Parkinson's disease	-5.23E-02	1.83E-02	0.01
Lysophosphatidylcholine (18:2) levels	Parkinson's disease	2.81E-02	2.66E-02	0.31
Lysophosphatidylcholine (20:4) levels	Parkinson's disease	-1.76E-02	1.62E-02	0.30
Lysophosphatidylethanolamine (18:0) levels	Parkinson's disease	-1.32E-02	5.70E-02	0.82
Lysophosphatidylethanolamine (18:1) levels	Parkinson's disease	1.27E-02	2.29E-02	0.59
Lysophosphatidylethanolamine (18:2) levels	Parkinson's disease	1.77E-02	1.27E-02	0.18
Phosphatidylcholine (14:0_16:0) levels	Parkinson's disease	8.70E-03	3.26E-02	0.80
Phosphatidylcholine (14:0_18:1) levels	Parkinson's disease	4.85E-02	6.95E-02	0.50
Phosphatidylcholine (14:0_18:2) levels	Parkinson's disease	9.97E-03	2.46E-02	0.69
Phosphatidylcholine (15:0_18:1) levels	Parkinson's disease	1.43E-02	2.95E-02	0.63
Phosphatidylcholine (15:0_18:2) levels	Parkinson's disease	1.05E-03	1.08E-02	0.92
Phosphatidylcholine (16:0_16:0) levels	Parkinson's disease	-1.56E-02	1.75E-02	0.39
Phosphatidylcholine (16:0_16:1) levels	Parkinson's disease	1.56E-02	3.41E-02	0.66
Phosphatidylcholine (16:0_17:1) levels	Parkinson's disease	-4.50E-03	1.89E-02	0.82
Phosphatidylcholine (16:0_18:0) levels	Parkinson's disease	1.20E-02	1.27E-02	0.35
Phosphatidylcholine (16:0_18:1) levels	Parkinson's disease	2.72E-02	2.38E-02	0.27

Phosphatidylcholine (16:0_18:2) levels	Parkinson's disease	-4.17E-03	1.22E-02	0.74
Phosphatidylcholine (16:0_18:3) levels	Parkinson's disease	9.94E-03	2.76E-02	0.72
Phosphatidylcholine (16:0_20:1) levels	Parkinson's disease	9.84E-03	2.70E-02	0.72
Phosphatidylcholine (16:0_20:2) levels	Parkinson's disease	1.96E-02	1.25E-02	0.13
Phosphatidylcholine (16:0_20:3) levels	Parkinson's disease	1.84E-02	2.12E-02	0.40
Phosphatidylcholine (16:0_20:4) levels	Parkinson's disease	-9.66E-03	1.11E-02	0.39
Phosphatidylcholine (16:0_20:5) levels	Parkinson's disease	-1.78E-02	1.07E-02	0.11
Phosphatidylcholine (16:0_22:4) levels	Parkinson's disease	2.38E-02	1.77E-02	0.19
Phosphatidylcholine (16:0_22:5) levels	Parkinson's disease	8.52E-03	1.12E-02	0.45
Phosphatidylcholine (16:0_22:6) levels	Parkinson's disease	8.64E-03	1.77E-02	0.63
Phosphatidylcholine (16:1_18:0) levels	Parkinson's disease	3.00E-02	2.73E-02	0.29
Phosphatidylcholine (16:1_18:1) levels	Parkinson's disease	3.24E-02	2.10E-02	0.14
Phosphatidylcholine (16:1_18:2) levels	Parkinson's disease	1.17E-02	1.56E-02	0.46
Phosphatidylcholine (16:1_20:4) levels	Parkinson's disease	4.34E-02	1.55E-02	0.02
Phosphatidylcholine (17:0_18:1) levels	Parkinson's disease	9.95E-05	1.75E-02	1.00
Phosphatidylcholine (17:0_18:2) levels	Parkinson's disease	3.04E-03	1.60E-02	0.85
Phosphatidylcholine (17:0_20:4) levels	Parkinson's disease	-3.46E-03	9.74E-03	0.73
Phosphatidylcholine (18:0_18:1) levels	Parkinson's disease	7.79E-03	1.28E-02	0.55
Phosphatidylcholine (18:0_18:2) levels	Parkinson's disease	1.88E-02	1.61E-02	0.25
Phosphatidylcholine (18:0_18:3) levels	Parkinson's disease	1.86E-02	1.72E-02	0.30
Phosphatidylcholine (18:0_20:2) levels	Parkinson's disease	1.93E-03	1.38E-02	0.89
Phosphatidylcholine (18:0_20:3) levels	Parkinson's disease	1.73E-02	1.66E-02	0.31
Phosphatidylcholine (18:0_20:4) levels	Parkinson's disease	-1.21E-03	7.75E-03	0.88
Phosphatidylcholine (18:0_20:5) levels	Parkinson's disease	-1.47E-02	1.02E-02	0.17
Phosphatidylcholine (18:0_22:5) levels	Parkinson's disease	1.42E-02	1.69E-02	0.41
Phosphatidylcholine (18:0_22:6) levels	Parkinson's disease	-2.56E-02	1.77E-02	0.17
Phosphatidylcholine (18:1_18:1) levels	Parkinson's disease	-2.45E-02	1.81E-02	0.19
Phosphatidylcholine (18:1_18:2) levels	Parkinson's disease	7.65E-03	1.33E-02	0.57
Phosphatidylcholine (18:1_18:3) levels	Parkinson's disease	3.38E-03	1.59E-02	0.83
Phosphatidylcholine (18:1_20:2) levels	Parkinson's disease	4.11E-03	1.41E-02	0.77
Phosphatidylcholine (18:1_20:3) levels	Parkinson's disease	8.83E-03	1.55E-02	0.58
Phosphatidylcholine (18:1_20:4) levels	Parkinson's disease	2.74E-03	1.05E-02	0.80
Phosphatidylcholine (18:2_18:2) levels	Parkinson's disease	1.76E-02	1.56E-02	0.28
Phosphatidylcholine (18:2_20:1) levels	Parkinson's disease	1.20E-02	1.48E-02	0.43
Phosphatidylcholine (18:2_20:3) levels	Parkinson's disease	1.90E-02	1.58E-02	0.25
Phosphatidylcholine (18:2_20:4) levels	Parkinson's disease	1.95E-02	1.63E-02	0.25
Phosphatidylcholine (O-16:0_16:0) levels	Parkinson's disease	-4.15E-02	2.37E-02	0.10
Phosphatidylcholine (O-16:0_16:1) levels	Parkinson's disease	-2.50E-02	3.21E-02	0.45
Phosphatidylcholine (O-16:0_18:1) levels	Parkinson's disease	1.12E-02	1.87E-02	0.56
Phosphatidylcholine (O-16:0_18:2) levels	Parkinson's disease	-8.92E-04	1.59E-02	0.96
Phosphatidylcholine (O-16:0_20:3) levels	Parkinson's disease	-1.79E-03	1.57E-02	0.91
Phosphatidylcholine (O-16:0_20:4) levels	Parkinson's disease	-6.59E-03	1.04E-02	0.53
Phosphatidylcholine (O-16:0_22:5) levels	Parkinson's disease	2.12E-02	3.11E-02	0.52
Phosphatidylcholine (O-16:1_16:0) levels	Parkinson's disease	-2.86E-02	1.59E-02	0.09
Phosphatidylcholine (O-16:1_18:0) levels	Parkinson's disease	3.82E-03	2.11E-02	0.86
Phosphatidylcholine (O-16:1_18:1) levels	Parkinson's disease	8.94E-03	1.78E-02	0.62

Phosphatidylcholine (O-16:1_18:2) levels	Parkinson's disease	7.31E-03	1.57E-02	0.65
Phosphatidylcholine (O-16:1_20:3) levels	Parkinson's disease	1.07E-02	2.02E-02	0.60
Phosphatidylcholine (O-16:1_20:4) levels	Parkinson's disease	1.53E-02	1.16E-02	0.20
Phosphatidylcholine (O-16:2_18:0) levels	Parkinson's disease	6.39E-03	2.84E-02	0.83
Phosphatidylcholine (O-17:0_15:0) levels	Parkinson's disease	-2.04E-02	2.15E-02	0.36
Phosphatidylcholine (O-17:0_17:1) levels	Parkinson's disease	1.57E-02	1.71E-02	0.37
Phosphatidylcholine (O-18:0_14:0) levels	Parkinson's disease	-4.07E-03	2.76E-02	0.89
Phosphatidylcholine (O-18:0_16:1) levels	Parkinson's disease	3.31E-02	2.10E-02	0.14
Phosphatidylcholine (O-18:0_20:4) levels	Parkinson's disease	1.79E-02	1.26E-02	0.17
Phosphatidylcholine (O-18:1_16:0) levels	Parkinson's disease	5.49E-03	1.65E-02	0.74
Phosphatidylcholine (O-18:1_18:2) levels	Parkinson's disease	2.13E-03	2.10E-02	0.92
Phosphatidylcholine (O-18:1_20:3) levels	Parkinson's disease	-2.62E-03	1.83E-02	0.89
Phosphatidylcholine (O-18:1_20:4) levels	Parkinson's disease	-7.48E-03	1.17E-02	0.53
Phosphatidylcholine (O-18:2_16:0) levels	Parkinson's disease	8.35E-03	1.56E-02	0.60
Phosphatidylcholine (O-18:2_18:1) levels	Parkinson's disease	-8.21E-02	2.72E-02	0.01
Phosphatidylcholine (O-18:2_18:2) levels	Parkinson's disease	-1.21E-02	2.00E-02	0.55
Phosphatidylcholine (O-18:2_20:4) levels	Parkinson's disease	1.20E-02	1.51E-02	0.44
Phosphatidylethanolamine (16:0_18:2) levels	Parkinson's disease	1.54E-02	1.58E-02	0.34
Phosphatidylethanolamine (16:0_20:4) levels	Parkinson's disease	1.17E-02	1.52E-02	0.45
Phosphatidylethanolamine (18:0_18:2) levels	Parkinson's disease	-5.27E-03	1.73E-02	0.76
Phosphatidylethanolamine (18:0_20:4) levels	Parkinson's disease	3.03E-02	2.43E-02	0.23
Phosphatidylethanolamine (18:1_18:1) levels	Parkinson's disease	3.15E-02	1.81E-02	0.10
Phosphatidylethanolamine (O-16:1_18:2) levels	Parkinson's disease	2.37E-02	2.04E-02	0.27
Phosphatidylethanolamine (O-16:1_20:4) levels	Parkinson's disease	-4.22E-03	1.81E-02	0.82
Phosphatidylethanolamine (O-16:1_22:5) levels	Parkinson's disease	-2.10E-03	1.79E-02	0.91
Phosphatidylethanolamine (O-18:1_18:2) levels	Parkinson's disease	-3.77E-03	1.71E-02	0.83
Phosphatidylethanolamine (O-18:1_20:4) levels	Parkinson's disease	-2.26E-02	2.44E-02	0.37
Phosphatidylethanolamine (O-18:2_18:1) levels	Parkinson's disease	1.54E-02	1.64E-02	0.36
Phosphatidylethanolamine (O-18:2_18:2) levels	Parkinson's disease	-4.19E-02	2.70E-02	0.15
Phosphatidylethanolamine (O-18:2_20:4) levels	Parkinson's disease	2.13E-02	1.86E-02	0.27
Phosphatidylinositol (16:0_18:1) levels	Parkinson's disease	1.65E-02	2.59E-02	0.54
Phosphatidylinositol (16:0_18:2) levels	Parkinson's disease	5.64E-02	2.84E-02	0.07
Phosphatidylinositol (16:0_20:4) levels	Parkinson's disease	3.66E-02	2.14E-02	0.10
Phosphatidylinositol (18:0_18:1) levels	Parkinson's disease	-8.56E-03	2.42E-02	0.73
Phosphatidylinositol (18:0_18:2) levels	Parkinson's disease	6.65E-03	1.35E-02	0.63
Phosphatidylinositol (18:0_20:3) levels	Parkinson's disease	8.30E-04	1.49E-02	0.96
Phosphatidylinositol (18:0_20:4) levels	Parkinson's disease	7.19E-03	1.58E-02	0.65
Phosphatidylinositol (18:1_18:1) levels	Parkinson's disease	1.14E-02	1.30E-02	0.40
Phosphatidylinositol (18:1_18:2) levels	Parkinson's disease	1.22E-02	1.84E-02	0.52
Phosphatidylinositol (18:1_20:4) levels	Parkinson's disease	1.00E-02	2.08E-02	0.64
Sphingomyelin (d32:1) levels	Parkinson's disease	2.70E-02	1.09E-02	0.02
Sphingomyelin (d34:0) levels	Parkinson's disease	3.02E-02	1.37E-02	0.04
Sphingomyelin (d34:1) levels	Parkinson's disease	-2.94E-03	1.13E-02	0.80
Sphingomyelin (d34:2) levels	Parkinson's disease	-6.81E-03	1.51E-02	0.66
Sphingomyelin (d36:1) levels	Parkinson's disease	-1.34E-03	1.27E-02	0.92
Sphingomyelin (d36:2) levels	Parkinson's disease	1.22E-02	2.07E-02	0.56

Sphingomyelin (d38:1) levels	Parkinson's disease	-1.06E-03	1.20E-02	0.93
Sphingomyelin (d38:2) levels	Parkinson's disease	-1.14E-02	1.41E-02	0.43
Sphingomyelin (d40:1) levels	Parkinson's disease	8.40E-03	1.18E-02	0.48
Sphingomyelin (d40:2) levels	Parkinson's disease	2.11E-02	1.07E-02	0.06
Sphingomyelin (d42:2) levels	Parkinson's disease	2.32E-02	1.14E-02	0.05
Triacylglycerol (46:1) levels	Parkinson's disease	3.62E-02	2.43E-02	0.16
Triacylglycerol (46:2) levels	Parkinson's disease	4.32E-03	2.55E-02	0.87
Triacylglycerol (48:0) levels	Parkinson's disease	2.85E-02	2.57E-02	0.29
Triacylglycerol (48:1) levels	Parkinson's disease	7.34E-03	2.25E-02	0.75
Triacylglycerol (48:2) levels	Parkinson's disease	-5.46E-02	2.41E-02	0.04
Triacylglycerol (48:3) levels	Parkinson's disease	-2.15E-02	2.88E-02	0.47
Triacylglycerol (49:1) levels	Parkinson's disease	2.37E-03	2.22E-02	0.92
Triacylglycerol (49:2) levels	Parkinson's disease	2.24E-02	2.11E-02	0.32
Triacylglycerol (50:1) levels	Parkinson's disease	-4.66E-03	1.86E-02	0.81
Triacylglycerol (50:2) levels	Parkinson's disease	-1.74E-02	2.09E-02	0.42
Triacylglycerol (50:3) levels	Parkinson's disease	-1.67E-02	1.58E-02	0.30
Triacylglycerol (50:4) levels	Parkinson's disease	-6.05E-02	1.82E-02	0.00
Triacylglycerol (50:5) levels	Parkinson's disease	3.84E-02	1.47E-02	0.02
Triacylglycerol (51:1) levels	Parkinson's disease	2.36E-02	2.47E-02	0.35
Triacylglycerol (51:2) levels	Parkinson's disease	2.35E-03	1.76E-02	0.90
Triacylglycerol (51:3) levels	Parkinson's disease	3.03E-02	1.88E-02	0.12
Triacylglycerol (51:4) levels	Parkinson's disease	3.55E-02	2.72E-02	0.21
Triacylglycerol (52:2) levels	Parkinson's disease	-1.18E-03	1.54E-02	0.94
Triacylglycerol (52:3) levels	Parkinson's disease	-9.21E-03	1.31E-02	0.49
Triacylglycerol (52:4) levels	Parkinson's disease	-3.57E-02	1.77E-02	0.06
Triacylglycerol (52:5) levels	Parkinson's disease	-2.56E-02	1.82E-02	0.18
Triacylglycerol (52:6) levels	Parkinson's disease	-2.12E-04	2.11E-02	0.99
Triacylglycerol (53:2) levels	Parkinson's disease	-2.06E-03	2.01E-02	0.92
Triacylglycerol (53:3) levels	Parkinson's disease	-6.67E-03	1.76E-02	0.71
Triacylglycerol (53:4) levels	Parkinson's disease	-8.21E-03	1.58E-02	0.61
Triacylglycerol (54:3) levels	Parkinson's disease	-1.57E-02	1.52E-02	0.31
Triacylglycerol (54:4) levels	Parkinson's disease	-2.71E-02	1.63E-02	0.11
Triacylglycerol (54:5) levels	Parkinson's disease	6.07E-03	1.85E-02	0.75
Triacylglycerol (54:6) levels	Parkinson's disease	4.47E-03	1.42E-02	0.76
Triacylglycerol (54:7) levels	Parkinson's disease	2.37E-02	1.69E-02	0.18
Triacylglycerol (56:3) levels	Parkinson's disease	-2.61E-02	1.97E-02	0.20
Triacylglycerol (56:4) levels	Parkinson's disease	-3.09E-02	2.09E-02	0.17
Triacylglycerol (56:5) levels	Parkinson's disease	-3.62E-02	1.68E-02	0.05
Triacylglycerol (56:6) levels	Parkinson's disease	2.62E-02	1.20E-02	0.04
Triacylglycerol (56:7) levels	Parkinson's disease	-3.43E-04	1.36E-02	0.98
Triacylglycerol (56:8) levels	Parkinson's disease	7.31E-03	1.47E-02	0.62
Triacylglycerol (58:7) levels	Parkinson's disease	-1.10E-02	3.86E-02	0.78
Triacylglycerol (58:8) levels	Parkinson's disease	5.83E-03	1.35E-02	0.67

Detection of heterogeneity by Q statistics					
exposure	outcome	method	Q	Q_df	Q_pval
Sterol ester (27:1/14:0) levels	Parkinson's disease	MR Egger	21.01	11	0.03
Sterol ester (27:1/14:0) levels	Parkinson's disease	Inverse variance weighted	23.93	12	0.02
Sterol ester (27:1/15:0) levels	Parkinson's disease	MR Egger	12.60	14	0.56
Sterol ester (27:1/15:0) levels	Parkinson's disease	Inverse variance weighted	15.10	15	0.44
Sterol ester (27:1/16:0) levels	Parkinson's disease	MR Egger	21.75	25	0.65
Sterol ester (27:1/16:0) levels	Parkinson's disease	Inverse variance weighted	22.41	26	0.67
Sterol ester (27:1/16:1) levels	Parkinson's disease	MR Egger	23.50	19	0.22
Sterol ester (27:1/16:1) levels	Parkinson's disease	Inverse variance weighted	24.55	20	0.22
Sterol ester (27:1/17:0) levels	Parkinson's disease	MR Egger	30.89	23	0.13
Sterol ester (27:1/17:0) levels	Parkinson's disease	Inverse variance weighted	30.89	24	0.16
Sterol ester (27:1/17:1) levels	Parkinson's disease	MR Egger	7.50	9	0.58
Sterol ester (27:1/17:1) levels	Parkinson's disease	Inverse variance weighted	8.89	10	0.54
Sterol ester (27:1/18:0) levels	Parkinson's disease	MR Egger	16.22	17	0.51
Sterol ester (27:1/18:0) levels	Parkinson's disease	Inverse variance weighted	16.74	18	0.54
Sterol ester (27:1/18:1) levels	Parkinson's disease	MR Egger	19.54	21	0.55
Sterol ester (27:1/18:1) levels	Parkinson's disease	Inverse variance weighted	19.72	22	0.60
Sterol ester (27:1/18:2) levels	Parkinson's disease	MR Egger	20.92	17	0.23
Sterol ester (27:1/18:2) levels	Parkinson's disease	Inverse variance weighted	26.72	18	0.08
Sterol ester (27:1/18:3) levels	Parkinson's disease	MR Egger	15.86	11	0.15
Sterol ester (27:1/18:3) levels	Parkinson's disease	Inverse variance weighted	17.33	12	0.14
Sterol ester (27:1/20:2) levels	Parkinson's disease	MR Egger	30.32	22	0.11
Sterol ester (27:1/20:2) levels	Parkinson's disease	Inverse variance weighted	30.34	23	0.14
Sterol ester (27:1/20:3) levels	Parkinson's disease	MR Egger	10.77	18	0.90
Sterol ester (27:1/20:3) levels	Parkinson's disease	Inverse variance weighted	11.55	19	0.90
Sterol ester (27:1/20:4) levels	Parkinson's disease	MR Egger	17.53	21	0.68
Sterol ester (27:1/20:4) levels	Parkinson's disease	Inverse variance weighted	17.57	22	0.73
Sterol ester (27:1/20:5) levels	Parkinson's disease	MR Egger	27.00	19	0.10
Sterol ester (27:1/20:5) levels	Parkinson's disease	Inverse variance weighted	30.88	20	0.06
Sterol ester (27:1/22:6) levels	Parkinson's disease	MR Egger	17.67	17	0.41
Sterol ester (27:1/22:6) levels	Parkinson's disease	Inverse variance weighted	20.39	18	0.31
Ceramide (d40:1) levels	Parkinson's disease	MR Egger	37.79	14	0.00
Ceramide (d40:1) levels	Parkinson's disease	Inverse variance weighted	38.04	15	0.00
Ceramide (d40:2) levels	Parkinson's disease	MR Egger	14.42	14	0.42
Ceramide (d40:2) levels	Parkinson's disease	Inverse variance weighted	14.91	15	0.46
Ceramide (d42:1) levels	Parkinson's disease	MR Egger	49.16	16	0.00
Ceramide (d42:1) levels	Parkinson's disease	Inverse variance weighted	49.16	17	0.00
Ceramide (d42:2) levels	Parkinson's disease	MR Egger	37.12	18	0.01
Ceramide (d42:2) levels	Parkinson's disease	Inverse variance weighted	37.20	19	0.01
Cholesterol levels	Parkinson's disease	MR Egger	22.79	22	0.41
Cholesterol levels	Parkinson's disease	Inverse variance weighted	24.10	23	0.40
Diacylglycerol (16:0_18:1) levels	Parkinson's disease	MR Egger	29.81	16	0.02
Diacylglycerol (16:0_18:1) levels	Parkinson's disease	Inverse variance weighted	31.30	17	0.02
Diacylglycerol (16:0_18:2) levels	Parkinson's disease	MR Egger	34.99	23	0.05
Diacylglycerol (16:0_18:2) levels	Parkinson's disease	Inverse variance weighted	36.04	24	0.05

Diacylglycerol (16:1_18:1) levels	Parkinson's disease	MR Egger	17.49	13	0.18
Diacylglycerol (16:1_18:1) levels	Parkinson's disease	Inverse variance weighted	18.87	14	0.17
Diacylglycerol (18:1_18:1) levels	Parkinson's disease	MR Egger	22.16	18	0.23
Diacylglycerol (18:1_18:1) levels	Parkinson's disease	Inverse variance weighted	22.24	19	0.27
Diacylglycerol (18:1_18:2) levels	Parkinson's disease	MR Egger	49.08	28	0.01
Diacylglycerol (18:1_18:2) levels	Parkinson's disease	Inverse variance weighted	49.10	29	0.01
Diacylglycerol (18:1_18:3) levels	Parkinson's disease	MR Egger	25.22	17	0.09
Diacylglycerol (18:1_18:3) levels	Parkinson's disease	Inverse variance weighted	27.86	18	0.06
Lysophosphatidylcholine (16:0) levels	Parkinson's disease	MR Egger	50.06	24	0.00
Lysophosphatidylcholine (16:0) levels	Parkinson's disease	Inverse variance weighted	50.46	25	0.00
Lysophosphatidylcholine (18:0) levels	Parkinson's disease	MR Egger	24.97	18	0.13
Lysophosphatidylcholine (18:0) levels	Parkinson's disease	Inverse variance weighted	25.59	19	0.14
Lysophosphatidylcholine (18:1) levels	Parkinson's disease	MR Egger	12.83	16	0.68
Lysophosphatidylcholine (18:1) levels	Parkinson's disease	Inverse variance weighted	21.03	17	0.23
Lysophosphatidylcholine (18:2) levels	Parkinson's disease	MR Egger	22.43	13	0.05
Lysophosphatidylcholine (18:2) levels	Parkinson's disease	Inverse variance weighted	24.37	14	0.04
Lysophosphatidylcholine (20:4) levels	Parkinson's disease	MR Egger	11.45	15	0.72
Lysophosphatidylcholine (20:4) levels	Parkinson's disease	Inverse variance weighted	12.63	16	0.70
Lysophosphatidylethanolamine (18:0) levels	Parkinson's disease	MR Egger	135.26	13	0.00
Lysophosphatidylethanolamine (18:0) levels	Parkinson's disease	Inverse variance weighted	135.82	14	0.00
Lysophosphatidylethanolamine (18:1) levels	Parkinson's disease	MR Egger	18.67	12	0.10
Lysophosphatidylethanolamine (18:1) levels	Parkinson's disease	Inverse variance weighted	19.15	13	0.12
Lysophosphatidylethanolamine (18:2) levels	Parkinson's disease	MR Egger	31.14	20	0.05
Lysophosphatidylethanolamine (18:2) levels	Parkinson's disease	Inverse variance weighted	34.15	21	0.03
Phosphatidylcholine (14:0_16:0) levels	Parkinson's disease	MR Egger	15.28	9	0.08
Phosphatidylcholine (14:0_16:0) levels	Parkinson's disease	Inverse variance weighted	15.40	10	0.12
Phosphatidylcholine (14:0_18:1) levels	Parkinson's disease	MR Egger	23.10	9	0.01
Phosphatidylcholine (14:0_18:1) levels	Parkinson's disease	Inverse variance weighted	24.35	10	0.01
Phosphatidylcholine (14:0_18:2) levels	Parkinson's disease	MR Egger	22.44	15	0.10
Phosphatidylcholine (14:0_18:2) levels	Parkinson's disease	Inverse variance weighted	22.69	16	0.12
Phosphatidylcholine (15:0_18:1) levels	Parkinson's disease	MR Egger	32.44	14	0.00
Phosphatidylcholine (15:0_18:1) levels	Parkinson's disease	Inverse variance weighted	32.99	15	0.00
Phosphatidylcholine (15:0_18:2) levels	Parkinson's disease	MR Egger	39.53	33	0.20
Phosphatidylcholine (15:0_18:2) levels	Parkinson's disease	Inverse variance weighted	39.55	34	0.24
Phosphatidylcholine (16:0_16:0) levels	Parkinson's disease	MR Egger	10.48	19	0.94
Phosphatidylcholine (16:0_16:0) levels	Parkinson's disease	Inverse variance weighted	11.27	20	0.94
Phosphatidylcholine (16:0_16:1) levels	Parkinson's disease	MR Egger	18.32	12	0.11
Phosphatidylcholine (16:0_16:1) levels	Parkinson's disease	Inverse variance weighted	18.64	13	0.13
Phosphatidylcholine (16:0_17:1) levels	Parkinson's disease	MR Egger	15.30	14	0.36
Phosphatidylcholine (16:0_17:1) levels	Parkinson's disease	Inverse variance weighted	15.36	15	0.43
Phosphatidylcholine (16:0_18:0) levels	Parkinson's disease	MR Egger	33.85	31	0.33
Phosphatidylcholine (16:0_18:0) levels	Parkinson's disease	Inverse variance weighted	34.82	32	0.34
Phosphatidylcholine (16:0_18:1) levels	Parkinson's disease	MR Egger	23.60	14	0.05
Phosphatidylcholine (16:0_18:1) levels	Parkinson's disease	Inverse variance weighted	25.80	15	0.04
Phosphatidylcholine (16:0_18:2) levels	Parkinson's disease	MR Egger	37.09	28	0.12
Phosphatidylcholine (16:0_18:2) levels	Parkinson's disease	Inverse variance weighted	37.24	29	0.14

Phosphatidylcholine (16:0_18:3) levels	Parkinson's disease	MR Egger	26.80	16	0.04
Phosphatidylcholine (16:0_18:3) levels	Parkinson's disease	Inverse variance weighted	27.02	17	0.06
Phosphatidylcholine (16:0_20:1) levels	Parkinson's disease	MR Egger	3.51	9	0.94
Phosphatidylcholine (16:0_20:1) levels	Parkinson's disease	Inverse variance weighted	3.64	10	0.96
Phosphatidylcholine (16:0_20:2) levels	Parkinson's disease	MR Egger	24.43	20	0.22
Phosphatidylcholine (16:0_20:2) levels	Parkinson's disease	Inverse variance weighted	27.40	21	0.16
Phosphatidylcholine (16:0_20:3) levels	Parkinson's disease	MR Egger	25.53	13	0.02
Phosphatidylcholine (16:0_20:3) levels	Parkinson's disease	Inverse variance weighted	27.00	14	0.02
Phosphatidylcholine (16:0_20:4) levels	Parkinson's disease	MR Egger	24.22	20	0.23
Phosphatidylcholine (16:0_20:4) levels	Parkinson's disease	Inverse variance weighted	25.15	21	0.24
Phosphatidylcholine (16:0_20:5) levels	Parkinson's disease	MR Egger	15.59	20	0.74
Phosphatidylcholine (16:0_20:5) levels	Parkinson's disease	Inverse variance weighted	18.36	21	0.63
Phosphatidylcholine (16:0_22:4) levels	Parkinson's disease	MR Egger	35.62	22	0.03
Phosphatidylcholine (16:0_22:4) levels	Parkinson's disease	Inverse variance weighted	38.55	23	0.02
Phosphatidylcholine (16:0_22:5) levels	Parkinson's disease	MR Egger	38.18	23	0.02
Phosphatidylcholine (16:0_22:5) levels	Parkinson's disease	Inverse variance weighted	39.15	24	0.03
Phosphatidylcholine (16:0_22:6) levels	Parkinson's disease	MR Egger	41.32	20	0.00
Phosphatidylcholine (16:0_22:6) levels	Parkinson's disease	Inverse variance weighted	41.81	21	0.00
Phosphatidylcholine (16:1_18:0) levels	Parkinson's disease	MR Egger	25.23	13	0.02
Phosphatidylcholine (16:1_18:0) levels	Parkinson's disease	Inverse variance weighted	27.59	14	0.02
Phosphatidylcholine (16:1_18:1) levels	Parkinson's disease	MR Egger	26.42	20	0.15
Phosphatidylcholine (16:1_18:1) levels	Parkinson's disease	Inverse variance weighted	29.57	21	0.10
Phosphatidylcholine (16:1_18:2) levels	Parkinson's disease	MR Egger	32.75	17	0.01
Phosphatidylcholine (16:1_18:2) levels	Parkinson's disease	Inverse variance weighted	33.84	18	0.01
Phosphatidylcholine (16:1_20:4) levels	Parkinson's disease	MR Egger	15.20	13	0.29
Phosphatidylcholine (16:1_20:4) levels	Parkinson's disease	Inverse variance weighted	24.37	14	0.04
Phosphatidylcholine (17:0_18:1) levels	Parkinson's disease	MR Egger	15.24	12	0.23
Phosphatidylcholine (17:0_18:1) levels	Parkinson's disease	Inverse variance weighted	15.24	13	0.29
Phosphatidylcholine (17:0_18:2) levels	Parkinson's disease	MR Egger	18.64	18	0.41
Phosphatidylcholine (17:0_18:2) levels	Parkinson's disease	Inverse variance weighted	18.68	19	0.48
Phosphatidylcholine (17:0_20:4) levels	Parkinson's disease	MR Egger	24.48	21	0.27
Phosphatidylcholine (17:0_20:4) levels	Parkinson's disease	Inverse variance weighted	24.62	22	0.32
Phosphatidylcholine (18:0_18:1) levels	Parkinson's disease	MR Egger	25.64	23	0.32
Phosphatidylcholine (18:0_18:1) levels	Parkinson's disease	Inverse variance weighted	26.05	24	0.35
Phosphatidylcholine (18:0_18:2) levels	Parkinson's disease	MR Egger	34.95	24	0.07
Phosphatidylcholine (18:0_18:2) levels	Parkinson's disease	Inverse variance weighted	36.94	25	0.06
Phosphatidylcholine (18:0_18:3) levels	Parkinson's disease	MR Egger	26.97	16	0.04
Phosphatidylcholine (18:0_18:3) levels	Parkinson's disease	Inverse variance weighted	28.94	17	0.04
Phosphatidylcholine (18:0_20:2) levels	Parkinson's disease	MR Egger	9.78	12	0.64
Phosphatidylcholine (18:0_20:2) levels	Parkinson's disease	Inverse variance weighted	9.80	13	0.71
Phosphatidylcholine (18:0_20:3) levels	Parkinson's disease	MR Egger	12.02	19	0.88
Phosphatidylcholine (18:0_20:3) levels	Parkinson's disease	Inverse variance weighted	13.10	20	0.87
Phosphatidylcholine (18:0_20:4) levels	Parkinson's disease	MR Egger	14.27	19	0.77
Phosphatidylcholine (18:0_20:4) levels	Parkinson's disease	Inverse variance weighted	14.29	20	0.82
Phosphatidylcholine (18:0_20:5) levels	Parkinson's disease	MR Egger	11.64	20	0.93
Phosphatidylcholine (18:0_20:5) levels	Parkinson's disease	Inverse variance weighted	13.71	21	0.88

Phosphatidylcholine (18:0_22:5) levels	Parkinson's disease	MR Egger	46.27	22	0.00
Phosphatidylcholine (18:0_22:5) levels	Parkinson's disease	Inverse variance weighted	47.74	23	0.00
Phosphatidylcholine (18:0_22:6) levels	Parkinson's disease	MR Egger	17.24	17	0.44
Phosphatidylcholine (18:0_22:6) levels	Parkinson's disease	Inverse variance weighted	19.37	18	0.37
Phosphatidylcholine (18:1_18:1) levels	Parkinson's disease	MR Egger	18.52	16	0.29
Phosphatidylcholine (18:1_18:1) levels	Parkinson's disease	Inverse variance weighted	20.65	17	0.24
Phosphatidylcholine (18:1_18:2) levels	Parkinson's disease	MR Egger	30.22	18	0.04
Phosphatidylcholine (18:1_18:2) levels	Parkinson's disease	Inverse variance weighted	30.78	19	0.04
Phosphatidylcholine (18:1_18:3) levels	Parkinson's disease	MR Egger	8.01	12	0.78
Phosphatidylcholine (18:1_18:3) levels	Parkinson's disease	Inverse variance weighted	8.05	13	0.84
Phosphatidylcholine (18:1_20:2) levels	Parkinson's disease	MR Egger	24.51	19	0.18
Phosphatidylcholine (18:1_20:2) levels	Parkinson's disease	Inverse variance weighted	24.62	20	0.22
Phosphatidylcholine (18:1_20:3) levels	Parkinson's disease	MR Egger	9.45	17	0.93
Phosphatidylcholine (18:1_20:3) levels	Parkinson's disease	Inverse variance weighted	9.78	18	0.94
Phosphatidylcholine (18:1_20:4) levels	Parkinson's disease	MR Egger	17.59	17	0.42
Phosphatidylcholine (18:1_20:4) levels	Parkinson's disease	Inverse variance weighted	17.66	18	0.48
Phosphatidylcholine (18:2_18:2) levels	Parkinson's disease	MR Egger	19.00	16	0.27
Phosphatidylcholine (18:2_18:2) levels	Parkinson's disease	Inverse variance weighted	20.51	17	0.25
Phosphatidylcholine (18:2_20:1) levels	Parkinson's disease	MR Egger	7.52	13	0.87
Phosphatidylcholine (18:2_20:1) levels	Parkinson's disease	Inverse variance weighted	8.18	14	0.88
Phosphatidylcholine (18:2_20:3) levels	Parkinson's disease	MR Egger	13.49	15	0.56
Phosphatidylcholine (18:2_20:3) levels	Parkinson's disease	Inverse variance weighted	14.95	16	0.53
Phosphatidylcholine (18:2_20:4) levels	Parkinson's disease	MR Egger	25.73	20	0.17
Phosphatidylcholine (18:2_20:4) levels	Parkinson's disease	Inverse variance weighted	27.57	21	0.15
Phosphatidylcholine (O-16:0_16:0) levels	Parkinson's disease	MR Egger	20.27	15	0.16
Phosphatidylcholine (O-16:0_16:0) levels	Parkinson's disease	Inverse variance weighted	24.40	16	0.08
Phosphatidylcholine (O-16:0_16:1) levels	Parkinson's disease	MR Egger	21.48	13	0.06
Phosphatidylcholine (O-16:0_16:1) levels	Parkinson's disease	Inverse variance weighted	22.48	14	0.07
Phosphatidylcholine (O-16:0_18:1) levels	Parkinson's disease	MR Egger	6.47	15	0.97
Phosphatidylcholine (O-16:0_18:1) levels	Parkinson's disease	Inverse variance weighted	6.83	16	0.98
Phosphatidylcholine (O-16:0_18:2) levels	Parkinson's disease	MR Egger	14.31	14	0.43
Phosphatidylcholine (O-16:0_18:2) levels	Parkinson's disease	Inverse variance weighted	14.32	15	0.50
Phosphatidylcholine (O-16:0_20:3) levels	Parkinson's disease	MR Egger	12.73	12	0.39
Phosphatidylcholine (O-16:0_20:3) levels	Parkinson's disease	Inverse variance weighted	12.74	13	0.47
Phosphatidylcholine (O-16:0_20:4) levels	Parkinson's disease	MR Egger	21.28	22	0.50
Phosphatidylcholine (O-16:0_20:4) levels	Parkinson's disease	Inverse variance weighted	21.68	23	0.54
Phosphatidylcholine (O-16:0_22:5) levels	Parkinson's disease	MR Egger	10.58	8	0.23
Phosphatidylcholine (O-16:0_22:5) levels	Parkinson's disease	Inverse variance weighted	11.19	9	0.26
Phosphatidylcholine (O-16:1_16:0) levels	Parkinson's disease	MR Egger	9.52	14	0.80
Phosphatidylcholine (O-16:1_16:0) levels	Parkinson's disease	Inverse variance weighted	12.75	15	0.62
Phosphatidylcholine (O-16:1_18:0) levels	Parkinson's disease	MR Egger	21.28	12	0.05
Phosphatidylcholine (O-16:1_18:0) levels	Parkinson's disease	Inverse variance weighted	21.34	13	0.07
Phosphatidylcholine (O-16:1_18:1) levels	Parkinson's disease	MR Egger	14.45	20	0.81
Phosphatidylcholine (O-16:1_18:1) levels	Parkinson's disease	Inverse variance weighted	14.70	21	0.84
Phosphatidylcholine (O-16:1_18:2) levels	Parkinson's disease	MR Egger	15.37	17	0.57
Phosphatidylcholine (O-16:1_18:2) levels	Parkinson's disease	Inverse variance weighted	15.59	18	0.62

Phosphatidylcholine (O-16:1_20:3) levels	Parkinson's disease	MR Egger	34.60	19	0.02
Phosphatidylcholine (O-16:1_20:3) levels	Parkinson's disease	Inverse variance weighted	35.11	20	0.02
Phosphatidylcholine (O-16:1_20:4) levels	Parkinson's disease	MR Egger	16.50	17	0.49
Phosphatidylcholine (O-16:1_20:4) levels	Parkinson's disease	Inverse variance weighted	18.26	18	0.44
Phosphatidylcholine (O-16:2_18:0) levels	Parkinson's disease	MR Egger	14.31	9	0.11
Phosphatidylcholine (O-16:2_18:0) levels	Parkinson's disease	Inverse variance weighted	14.39	10	0.16
Phosphatidylcholine (O-17:0_15:0) levels	Parkinson's disease	MR Egger	13.10	14	0.52
Phosphatidylcholine (O-17:0_15:0) levels	Parkinson's disease	Inverse variance weighted	14.00	15	0.53
Phosphatidylcholine (O-17:0_17:1) levels	Parkinson's disease	MR Egger	44.93	27	0.02
Phosphatidylcholine (O-17:0_17:1) levels	Parkinson's disease	Inverse variance weighted	46.33	28	0.02
Phosphatidylcholine (O-18:0_14:0) levels	Parkinson's disease	MR Egger	12.10	11	0.36
Phosphatidylcholine (O-18:0_14:0) levels	Parkinson's disease	Inverse variance weighted	12.12	12	0.44
Phosphatidylcholine (O-18:0_16:1) levels	Parkinson's disease	MR Egger	7.76	11	0.73
Phosphatidylcholine (O-18:0_16:1) levels	Parkinson's disease	Inverse variance weighted	10.24	12	0.59
Phosphatidylcholine (O-18:0_20:4) levels	Parkinson's disease	MR Egger	11.07	17	0.85
Phosphatidylcholine (O-18:0_20:4) levels	Parkinson's disease	Inverse variance weighted	13.09	18	0.79
Phosphatidylcholine (O-18:1_16:0) levels	Parkinson's disease	MR Egger	23.24	24	0.51
Phosphatidylcholine (O-18:1_16:0) levels	Parkinson's disease	Inverse variance weighted	23.35	25	0.56
Phosphatidylcholine (O-18:1_18:2) levels	Parkinson's disease	MR Egger	28.58	18	0.05
Phosphatidylcholine (O-18:1_18:2) levels	Parkinson's disease	Inverse variance weighted	28.60	19	0.07
Phosphatidylcholine (O-18:1_20:3) levels	Parkinson's disease	MR Egger	14.55	14	0.41
Phosphatidylcholine (O-18:1_20:3) levels	Parkinson's disease	Inverse variance weighted	14.57	15	0.48
Phosphatidylcholine (O-18:1_20:4) levels	Parkinson's disease	MR Egger	14.81	15	0.46
Phosphatidylcholine (O-18:1_20:4) levels	Parkinson's disease	Inverse variance weighted	15.22	16	0.51
Phosphatidylcholine (O-18:2_16:0) levels	Parkinson's disease	MR Egger	18.16	18	0.45
Phosphatidylcholine (O-18:2_16:0) levels	Parkinson's disease	Inverse variance weighted	18.45	19	0.49
Phosphatidylcholine (O-18:2_18:1) levels	Parkinson's disease	MR Egger	17.44	12	0.13
Phosphatidylcholine (O-18:2_18:1) levels	Parkinson's disease	Inverse variance weighted	30.69	13	0.00
Phosphatidylcholine (O-18:2_18:2) levels	Parkinson's disease	MR Egger	10.37	13	0.66
Phosphatidylcholine (O-18:2_18:2) levels	Parkinson's disease	Inverse variance weighted	10.74	14	0.71
Phosphatidylcholine (O-18:2_20:4) levels	Parkinson's disease	MR Egger	16.20	16	0.44
Phosphatidylcholine (O-18:2_20:4) levels	Parkinson's disease	Inverse variance weighted	16.85	17	0.46
Phosphatidylethanolamine (16:0_18:2) levels	Parkinson's disease	MR Egger	29.24	18	0.05
Phosphatidylethanolamine (16:0_18:2) levels	Parkinson's disease	Inverse variance weighted	30.79	19	0.04
Phosphatidylethanolamine (16:0_20:4) levels	Parkinson's disease	MR Egger	31.13	18	0.03
Phosphatidylethanolamine (16:0_20:4) levels	Parkinson's disease	Inverse variance weighted	32.15	19	0.03
Phosphatidylethanolamine (18:0_18:2) levels	Parkinson's disease	MR Egger	33.40	22	0.06
Phosphatidylethanolamine (18:0_18:2) levels	Parkinson's disease	Inverse variance weighted	33.54	23	0.07
Phosphatidylethanolamine (18:0_20:4) levels	Parkinson's disease	MR Egger	28.97	15	0.02
Phosphatidylethanolamine (18:0_20:4) levels	Parkinson's disease	Inverse variance weighted	31.97	16	0.01
Phosphatidylethanolamine (18:1_18:1) levels	Parkinson's disease	MR Egger	34.88	19	0.01
Phosphatidylethanolamine (18:1_18:1) levels	Parkinson's disease	Inverse variance weighted	40.42	20	0.00
Phosphatidylethanolamine (O-16:1_18:2) levels	Parkinson's disease	MR Egger	20.22	13	0.09
Phosphatidylethanolamine (O-16:1_18:2) levels	Parkinson's disease	Inverse variance weighted	22.31	14	0.07
Phosphatidylethanolamine (O-16:1_20:4) levels	Parkinson's disease	MR Egger	18.01	15	0.26
Phosphatidylethanolamine (O-16:1_20:4) levels	Parkinson's disease	Inverse variance weighted	18.08	16	0.32

Phosphatidylethanolamine (O-16:1_22:5) levels	Parkinson's disease	MR Egger	14.49	14	0.41
Phosphatidylethanolamine (O-16:1_22:5) levels	Parkinson's disease	Inverse variance weighted	14.50	15	0.49
Phosphatidylethanolamine (O-18:1_18:2) levels	Parkinson's disease	MR Egger	14.38	21	0.85
Phosphatidylethanolamine (O-18:1_18:2) levels	Parkinson's disease	Inverse variance weighted	14.43	22	0.89
Phosphatidylethanolamine (O-18:1_20:4) levels	Parkinson's disease	MR Egger	43.52	17	0.00
Phosphatidylethanolamine (O-18:1_20:4) levels	Parkinson's disease	Inverse variance weighted	45.70	18	0.00
Phosphatidylethanolamine (O-18:2_18:1) levels	Parkinson's disease	MR Egger	12.89	17	0.74
Phosphatidylethanolamine (O-18:2_18:1) levels	Parkinson's disease	Inverse variance weighted	13.77	18	0.74
Phosphatidylethanolamine (O-18:2_18:2) levels	Parkinson's disease	MR Egger	27.55	10	0.00
Phosphatidylethanolamine (O-18:2_18:2) levels	Parkinson's disease	Inverse variance weighted	34.21	11	0.00
Phosphatidylethanolamine (O-18:2_20:4) levels	Parkinson's disease	MR Egger	19.26	19	0.44
Phosphatidylethanolamine (O-18:2_20:4) levels	Parkinson's disease	Inverse variance weighted	20.59	20	0.42
Phosphatidylinositol (16:0_18:1) levels	Parkinson's disease	MR Egger	15.40	10	0.12
Phosphatidylinositol (16:0_18:1) levels	Parkinson's disease	Inverse variance weighted	16.03	11	0.14
Phosphatidylinositol (16:0_18:2) levels	Parkinson's disease	MR Egger	25.46	12	0.01
Phosphatidylinositol (16:0_18:2) levels	Parkinson's disease	Inverse variance weighted	33.79	13	0.00
Phosphatidylinositol (16:0_20:4) levels	Parkinson's disease	MR Egger	26.13	17	0.07
Phosphatidylinositol (16:0_20:4) levels	Parkinson's disease	Inverse variance weighted	30.64	18	0.03
Phosphatidylinositol (18:0_18:1) levels	Parkinson's disease	MR Egger	22.04	14	0.08
Phosphatidylinositol (18:0_18:1) levels	Parkinson's disease	Inverse variance weighted	22.23	15	0.10
Phosphatidylinositol (18:0_18:2) levels	Parkinson's disease	MR Egger	44.94	24	0.01
Phosphatidylinositol (18:0_18:2) levels	Parkinson's disease	Inverse variance weighted	45.39	25	0.01
Phosphatidylinositol (18:0_20:3) levels	Parkinson's disease	MR Egger	53.60	25	0.00
Phosphatidylinositol (18:0_20:3) levels	Parkinson's disease	Inverse variance weighted	53.61	26	0.00
Phosphatidylinositol (18:0_20:4) levels	Parkinson's disease	MR Egger	26.25	18	0.09
Phosphatidylinositol (18:0_20:4) levels	Parkinson's disease	Inverse variance weighted	26.55	19	0.12
Phosphatidylinositol (18:1_18:1) levels	Parkinson's disease	MR Egger	11.73	14	0.63
Phosphatidylinositol (18:1_18:1) levels	Parkinson's disease	Inverse variance weighted	12.49	15	0.64
Phosphatidylinositol (18:1_18:2) levels	Parkinson's disease	MR Egger	13.95	16	0.60
Phosphatidylinositol (18:1_18:2) levels	Parkinson's disease	Inverse variance weighted	14.39	17	0.64
Phosphatidylinositol (18:1_20:4) levels	Parkinson's disease	MR Egger	26.85	17	0.06
Phosphatidylinositol (18:1_20:4) levels	Parkinson's disease	Inverse variance weighted	27.22	18	0.08
Sphingomyelin (d32:1) levels	Parkinson's disease	MR Egger	23.23	21	0.33
Sphingomyelin (d32:1) levels	Parkinson's disease	Inverse variance weighted	30.05	22	0.12
Sphingomyelin (d34:0) levels	Parkinson's disease	MR Egger	14.75	16	0.54
Sphingomyelin (d34:0) levels	Parkinson's disease	Inverse variance weighted	19.60	17	0.30
Sphingomyelin (d34:1) levels	Parkinson's disease	MR Egger	26.30	23	0.29
Sphingomyelin (d34:1) levels	Parkinson's disease	Inverse variance weighted	26.37	24	0.33
Sphingomyelin (d34:2) levels	Parkinson's disease	MR Egger	25.87	20	0.17
Sphingomyelin (d34:2) levels	Parkinson's disease	Inverse variance weighted	26.14	21	0.20
Sphingomyelin (d36:1) levels	Parkinson's disease	MR Egger	23.90	25	0.53
Sphingomyelin (d36:1) levels	Parkinson's disease	Inverse variance weighted	23.91	26	0.58
Sphingomyelin (d36:2) levels	Parkinson's disease	MR Egger	15.46	15	0.42
Sphingomyelin (d36:2) levels	Parkinson's disease	Inverse variance weighted	15.82	16	0.47
Sphingomyelin (d38:1) levels	Parkinson's disease	MR Egger	22.97	23	0.46
Sphingomyelin (d38:1) levels	Parkinson's disease	Inverse variance weighted	22.97	24	0.52

Sphingomyelin (d38:2) levels	Parkinson's disease	MR Egger	14.45	19	0.76
Sphingomyelin (d38:2) levels	Parkinson's disease	Inverse variance weighted	15.10	20	0.77
Sphingomyelin (d40:1) levels	Parkinson's disease	MR Egger	33.14	32	0.41
Sphingomyelin (d40:1) levels	Parkinson's disease	Inverse variance weighted	33.66	33	0.44
Sphingomyelin (d40:2) levels	Parkinson's disease	MR Egger	16.14	27	0.95
Sphingomyelin (d40:2) levels	Parkinson's disease	Inverse variance weighted	20.01	28	0.86
Sphingomyelin (d42:2) levels	Parkinson's disease	MR Egger	12.15	29	1.00
Sphingomyelin (d42:2) levels	Parkinson's disease	Inverse variance weighted	16.29	30	0.98
Triacylglycerol (46:1) levels	Parkinson's disease	MR Egger	28.09	12	0.01
Triacylglycerol (46:1) levels	Parkinson's disease	Inverse variance weighted	33.29	13	0.00
Triacylglycerol (46:2) levels	Parkinson's disease	MR Egger	31.06	16	0.01
Triacylglycerol (46:2) levels	Parkinson's disease	Inverse variance weighted	31.12	17	0.02
Triacylglycerol (48:0) levels	Parkinson's disease	MR Egger	16.24	11	0.13
Triacylglycerol (48:0) levels	Parkinson's disease	Inverse variance weighted	18.06	12	0.11
Triacylglycerol (48:1) levels	Parkinson's disease	MR Egger	30.70	18	0.03
Triacylglycerol (48:1) levels	Parkinson's disease	Inverse variance weighted	30.88	19	0.04
Triacylglycerol (48:2) levels	Parkinson's disease	MR Egger	16.15	14	0.30
Triacylglycerol (48:2) levels	Parkinson's disease	Inverse variance weighted	22.07	15	0.11
Triacylglycerol (48:3) levels	Parkinson's disease	MR Egger	24.69	16	0.08
Triacylglycerol (48:3) levels	Parkinson's disease	Inverse variance weighted	25.55	17	0.08
Triacylglycerol (49:1) levels	Parkinson's disease	MR Egger	34.48	19	0.02
Triacylglycerol (49:1) levels	Parkinson's disease	Inverse variance weighted	34.50	20	0.02
Triacylglycerol (49:2) levels	Parkinson's disease	MR Egger	9.62	8	0.29
Triacylglycerol (49:2) levels	Parkinson's disease	Inverse variance weighted	10.97	9	0.28
Triacylglycerol (50:1) levels	Parkinson's disease	MR Egger	37.33	20	0.01
Triacylglycerol (50:1) levels	Parkinson's disease	Inverse variance weighted	37.45	21	0.01
Triacylglycerol (50:2) levels	Parkinson's disease	MR Egger	13.63	12	0.33
Triacylglycerol (50:2) levels	Parkinson's disease	Inverse variance weighted	14.41	13	0.35
Triacylglycerol (50:3) levels	Parkinson's disease	MR Egger	37.91	23	0.03
Triacylglycerol (50:3) levels	Parkinson's disease	Inverse variance weighted	39.76	24	0.02
Triacylglycerol (50:4) levels	Parkinson's disease	MR Egger	14.69	16	0.55
Triacylglycerol (50:4) levels	Parkinson's disease	Inverse variance weighted	25.71	17	0.08
Triacylglycerol (50:5) levels	Parkinson's disease	MR Egger	22.75	17	0.16
Triacylglycerol (50:5) levels	Parkinson's disease	Inverse variance weighted	31.94	18	0.02
Triacylglycerol (51:1) levels	Parkinson's disease	MR Egger	39.16	17	0.00
Triacylglycerol (51:1) levels	Parkinson's disease	Inverse variance weighted	41.26	18	0.00
Triacylglycerol (51:2) levels	Parkinson's disease	MR Egger	23.84	17	0.12
Triacylglycerol (51:2) levels	Parkinson's disease	Inverse variance weighted	23.86	18	0.16
Triacylglycerol (51:3) levels	Parkinson's disease	MR Egger	33.39	19	0.02
Triacylglycerol (51:3) levels	Parkinson's disease	Inverse variance weighted	37.96	20	0.01
Triacylglycerol (51:4) levels	Parkinson's disease	MR Egger	33.91	14	0.00
Triacylglycerol (51:4) levels	Parkinson's disease	Inverse variance weighted	38.04	15	0.00
Triacylglycerol (52:2) levels	Parkinson's disease	MR Egger	16.39	18	0.57
Triacylglycerol (52:2) levels	Parkinson's disease	Inverse variance weighted	16.40	19	0.63
Triacylglycerol (52:3) levels	Parkinson's disease	MR Egger	33.85	24	0.09
Triacylglycerol (52:3) levels	Parkinson's disease	Inverse variance weighted	34.55	25	0.10

Triacylglycerol (52:4) levels	Parkinson's disease	MR Egger	27.11	18	0.08
Triacylglycerol (52:4) levels	Parkinson's disease	Inverse variance weighted	33.22	19	0.02
Triacylglycerol (52:5) levels	Parkinson's disease	MR Egger	26.19	19	0.13
Triacylglycerol (52:5) levels	Parkinson's disease	Inverse variance weighted	28.92	20	0.09
Triacylglycerol (52:6) levels	Parkinson's disease	MR Egger	22.12	15	0.10
Triacylglycerol (52:6) levels	Parkinson's disease	Inverse variance weighted	22.12	16	0.14
Triacylglycerol (53:2) levels	Parkinson's disease	MR Egger	22.47	17	0.17
Triacylglycerol (53:2) levels	Parkinson's disease	Inverse variance weighted	22.48	18	0.21
Triacylglycerol (53:3) levels	Parkinson's disease	MR Egger	30.67	20	0.06
Triacylglycerol (53:3) levels	Parkinson's disease	Inverse variance weighted	30.90	21	0.08
Triacylglycerol (53:4) levels	Parkinson's disease	MR Egger	36.91	23	0.03
Triacylglycerol (53:4) levels	Parkinson's disease	Inverse variance weighted	37.34	24	0.04
Triacylglycerol (54:3) levels	Parkinson's disease	MR Egger	41.80	27	0.03
Triacylglycerol (54:3) levels	Parkinson's disease	Inverse variance weighted	43.47	28	0.03
Triacylglycerol (54:4) levels	Parkinson's disease	MR Egger	26.10	19	0.13
Triacylglycerol (54:4) levels	Parkinson's disease	Inverse variance weighted	29.90	20	0.07
Triacylglycerol (54:5) levels	Parkinson's disease	MR Egger	27.77	18	0.07
Triacylglycerol (54:5) levels	Parkinson's disease	Inverse variance weighted	27.94	19	0.08
Triacylglycerol (54:6) levels	Parkinson's disease	MR Egger	20.07	14	0.13
Triacylglycerol (54:6) levels	Parkinson's disease	Inverse variance weighted	20.21	15	0.16
Triacylglycerol (54:7) levels	Parkinson's disease	MR Egger	31.72	20	0.05
Triacylglycerol (54:7) levels	Parkinson's disease	Inverse variance weighted	34.85	21	0.03
Triacylglycerol (56:3) levels	Parkinson's disease	MR Egger	31.14	19	0.04
Triacylglycerol (56:3) levels	Parkinson's disease	Inverse variance weighted	34.04	20	0.03
Triacylglycerol (56:4) levels	Parkinson's disease	MR Egger	12.79	12	0.38
Triacylglycerol (56:4) levels	Parkinson's disease	Inverse variance weighted	15.12	13	0.30
Triacylglycerol (56:5) levels	Parkinson's disease	MR Egger	17.69	15	0.28
Triacylglycerol (56:5) levels	Parkinson's disease	Inverse variance weighted	23.14	16	0.11
Triacylglycerol (56:6) levels	Parkinson's disease	MR Egger	19.66	20	0.48
Triacylglycerol (56:6) levels	Parkinson's disease	Inverse variance weighted	24.41	21	0.27
Triacylglycerol (56:7) levels	Parkinson's disease	MR Egger	29.17	24	0.21
Triacylglycerol (56:7) levels	Parkinson's disease	Inverse variance weighted	29.17	25	0.26
Triacylglycerol (56:8) levels	Parkinson's disease	MR Egger	33.41	25	0.12
Triacylglycerol (56:8) levels	Parkinson's disease	Inverse variance weighted	33.75	26	0.14
Triacylglycerol (58:7) levels	Parkinson's disease	MR Egger	22.00	8	0.00
Triacylglycerol (58:7) levels	Parkinson's disease	Inverse variance weighted	22.23	9	0.01
Triacylglycerol (58:8) levels	Parkinson's disease	MR Egger	18.00	23	0.76
Triacylglycerol (58:8) levels	Parkinson's disease	Inverse variance weighted	18.18	24	0.79

Detection of pleiotropy by MR-PRESSO global test

exposure	outcome	Global Test RSSobs	pval
Sterol ester (27:1/14:0) levels	Parkinson's disease	31.29	0.01
Sterol ester (27:1/15:0) levels	Parkinson's disease	17.16	0.48
Sterol ester (27:1/16:0) levels	Parkinson's disease	24.35	0.66
Sterol ester (27:1/16:1) levels	Parkinson's disease	27.97	0.18
Sterol ester (27:1/17:0) levels	Parkinson's disease	33.18	0.17
Sterol ester (27:1/17:1) levels	Parkinson's disease	10.64	0.57
Sterol ester (27:1/18:0) levels	Parkinson's disease	18.64	0.55
Sterol ester (27:1/18:1) levels	Parkinson's disease	22.77	0.55
Sterol ester (27:1/18:2) levels	Parkinson's disease	29.48	0.10
Sterol ester (27:1/18:3) levels	Parkinson's disease	21.67	0.12
Sterol ester (27:1/20:2) levels	Parkinson's disease	32.59	0.17
Sterol ester (27:1/20:3) levels	Parkinson's disease	13.87	0.86
Sterol ester (27:1/20:4) levels	Parkinson's disease	20.39	0.76
Sterol ester (27:1/20:5) levels	Parkinson's disease	38.69	0.18
Sterol ester (27:1/22:6) levels	Parkinson's disease	22.89	0.31
Ceramide (d40:1) levels	Parkinson's disease	45.46	0.00
Ceramide (d40:2) levels	Parkinson's disease	18.55	0.40
Ceramide (d42:1) levels	Parkinson's disease	56.89	0.00
Ceramide (d42:2) levels	Parkinson's disease	42.22	0.01
Cholesterol levels	Parkinson's disease	25.80	0.42
Diacylglycerol (16:0_18:1) levels	Parkinson's disease	37.16	0.01
Diacylglycerol (16:0_18:2) levels	Parkinson's disease	40.76	0.03
Diacylglycerol (16:1_18:1) levels	Parkinson's disease	22.93	0.16
Diacylglycerol (18:1_18:1) levels	Parkinson's disease	25.48	0.26
Diacylglycerol (18:1_18:2) levels	Parkinson's disease	54.66	0.01
Diacylglycerol (18:1_18:3) levels	Parkinson's disease	31.96	0.07
Lysophosphatidylcholine (16:0) levels	Parkinson's disease	56.37	0.00
Lysophosphatidylcholine (18:0) levels	Parkinson's disease	28.38	0.17
Lysophosphatidylcholine (18:1) levels	Parkinson's disease	24.14	0.19
Lysophosphatidylcholine (18:2) levels	Parkinson's disease	28.47	0.04
Lysophosphatidylcholine (20:4) levels	Parkinson's disease	14.01	0.71
Lysophosphatidylethanolamine (18:0) levels	Parkinson's disease	150.58	0.00
Lysophosphatidylethanolamine (18:1) levels	Parkinson's disease	22.62	0.11
Lysophosphatidylethanolamine (18:2) levels	Parkinson's disease	40.48	0.07
Phosphatidylcholine (14:0_16:0) levels	Parkinson's disease	19.98	0.13
Phosphatidylcholine (14:0_18:1) levels	Parkinson's disease	33.47	0.01
Phosphatidylcholine (14:0_18:2) levels	Parkinson's disease	27.89	0.10
Phosphatidylcholine (15:0_18:1) levels	Parkinson's disease	39.37	0.00
Phosphatidylcholine (15:0_18:2) levels	Parkinson's disease	42.16	0.25
Phosphatidylcholine (16:0_16:0) levels	Parkinson's disease	12.31	0.95
Phosphatidylcholine (16:0_16:1) levels	Parkinson's disease	25.43	0.12
Phosphatidylcholine (16:0_17:1) levels	Parkinson's disease	17.13	0.46
Phosphatidylcholine (16:0_18:0) levels	Parkinson's disease	36.62	0.35
Phosphatidylcholine (16:0_18:1) levels	Parkinson's disease	31.63	0.03

Phosphatidylcholine (16:0_18:2) levels	Parkinson's disease	39.16	0.17
Phosphatidylcholine (16:0_18:3) levels	Parkinson's disease	32.71	0.04
Phosphatidylcholine (16:0_20:1) levels	Parkinson's disease	4.41	0.95
Phosphatidylcholine (16:0_20:2) levels	Parkinson's disease	30.22	0.17
Phosphatidylcholine (16:0_20:3) levels	Parkinson's disease	30.85	0.03
Phosphatidylcholine (16:0_20:4) levels	Parkinson's disease	27.50	0.41
Phosphatidylcholine (16:0_20:5) levels	Parkinson's disease	21.31	0.65
Phosphatidylcholine (16:0_22:4) levels	Parkinson's disease	41.99	0.03
Phosphatidylcholine (16:0_22:5) levels	Parkinson's disease	41.13	0.07
Phosphatidylcholine (16:0_22:6) levels	Parkinson's disease	47.37	0.00
Phosphatidylcholine (16:1_18:0) levels	Parkinson's disease	34.90	0.01
Phosphatidylcholine (16:1_18:1) levels	Parkinson's disease	33.87	0.09
Phosphatidylcholine (16:1_18:2) levels	Parkinson's disease	37.64	0.05
Phosphatidylcholine (16:1_20:4) levels	Parkinson's disease	38.50	0.12
Phosphatidylcholine (17:0_18:1) levels	Parkinson's disease	16.99	0.34
Phosphatidylcholine (17:0_18:2) levels	Parkinson's disease	20.54	0.50
Phosphatidylcholine (17:0_20:4) levels	Parkinson's disease	27.07	0.46
Phosphatidylcholine (18:0_18:1) levels	Parkinson's disease	27.72	0.37
Phosphatidylcholine (18:0_18:2) levels	Parkinson's disease	39.17	0.07
Phosphatidylcholine (18:0_18:3) levels	Parkinson's disease	33.00	0.03
Phosphatidylcholine (18:0_20:2) levels	Parkinson's disease	10.55	0.78
Phosphatidylcholine (18:0_20:3) levels	Parkinson's disease	14.49	0.87
Phosphatidylcholine (18:0_20:4) levels	Parkinson's disease	15.35	0.86
Phosphatidylcholine (18:0_20:5) levels	Parkinson's disease	16.82	0.83
Phosphatidylcholine (18:0_22:5) levels	Parkinson's disease	51.17	0.00
Phosphatidylcholine (18:0_22:6) levels	Parkinson's disease	22.83	0.37
Phosphatidylcholine (18:1_18:1) levels	Parkinson's disease	22.47	0.31
Phosphatidylcholine (18:1_18:2) levels	Parkinson's disease	32.65	0.09
Phosphatidylcholine (18:1_18:3) levels	Parkinson's disease	9.02	0.85
Phosphatidylcholine (18:1_20:2) levels	Parkinson's disease	26.19	0.29
Phosphatidylcholine (18:1_20:3) levels	Parkinson's disease	10.80	0.94
Phosphatidylcholine (18:1_20:4) levels	Parkinson's disease	18.08	0.66
Phosphatidylcholine (18:2_18:2) levels	Parkinson's disease	22.45	0.30
Phosphatidylcholine (18:2_20:1) levels	Parkinson's disease	9.35	0.89
Phosphatidylcholine (18:2_20:3) levels	Parkinson's disease	16.65	0.57
Phosphatidylcholine (18:2_20:4) levels	Parkinson's disease	30.05	0.18
Phosphatidylcholine (O-16:0_16:0) levels	Parkinson's disease	27.29	0.10
Phosphatidylcholine (O-16:0_16:1) levels	Parkinson's disease	24.87	0.08
Phosphatidylcholine (O-16:0_18:1) levels	Parkinson's disease	7.58	0.98
Phosphatidylcholine (O-16:0_18:2) levels	Parkinson's disease	15.63	0.55
Phosphatidylcholine (O-16:0_20:3) levels	Parkinson's disease	15.06	0.50
Phosphatidylcholine (O-16:0_20:4) levels	Parkinson's disease	22.68	0.63
Phosphatidylcholine (O-16:0_22:5) levels	Parkinson's disease	13.77	0.31
Phosphatidylcholine (O-16:1_16:0) levels	Parkinson's disease	14.74	0.62
Phosphatidylcholine (O-16:1_18:0) levels	Parkinson's disease	25.11	0.08
Phosphatidylcholine (O-16:1_18:1) levels	Parkinson's disease	16.69	0.84

Phosphatidylcholine (O-16:1_18:2) levels	Parkinson's disease	17.64	0.62
Phosphatidylcholine (O-16:1_20:3) levels	Parkinson's disease	37.94	0.03
Phosphatidylcholine (O-16:1_20:4) levels	Parkinson's disease	24.41	0.41
Phosphatidylcholine (O-16:2_18:0) levels	Parkinson's disease	16.17	0.22
Phosphatidylcholine (O-17:0_15:0) levels	Parkinson's disease	15.94	0.54
Phosphatidylcholine (O-17:0_17:1) levels	Parkinson's disease	50.04	0.02
Phosphatidylcholine (O-18:0_14:0) levels	Parkinson's disease	15.55	0.36
Phosphatidylcholine (O-18:0_16:1) levels	Parkinson's disease	12.21	0.60
Phosphatidylcholine (O-18:0_20:4) levels	Parkinson's disease	17.60	0.69
Phosphatidylcholine (O-18:1_16:0) levels	Parkinson's disease	25.20	0.57
Phosphatidylcholine (O-18:1_18:2) levels	Parkinson's disease	32.63	0.06
Phosphatidylcholine (O-18:1_20:3) levels	Parkinson's disease	15.51	0.58
Phosphatidylcholine (O-18:1_20:4) levels	Parkinson's disease	16.13	0.59
Phosphatidylcholine (O-18:2_16:0) levels	Parkinson's disease	19.62	0.53
Phosphatidylcholine (O-18:2_18:1) levels	Parkinson's disease	35.81	0.01
Phosphatidylcholine (O-18:2_18:2) levels	Parkinson's disease	12.02	0.76
Phosphatidylcholine (O-18:2_20:4) levels	Parkinson's disease	18.27	0.50
Phosphatidylethanolamine (16:0_18:2) levels	Parkinson's disease	33.68	0.06
Phosphatidylethanolamine (16:0_20:4) levels	Parkinson's disease	34.53	0.08
Phosphatidylethanolamine (18:0_18:2) levels	Parkinson's disease	36.19	0.09
Phosphatidylethanolamine (18:0_20:4) levels	Parkinson's disease	33.86	0.02
Phosphatidylethanolamine (18:1_18:1) levels	Parkinson's disease	43.32	0.00
Phosphatidylethanolamine (O-16:1_18:2) levels	Parkinson's disease	24.35	0.10
Phosphatidylethanolamine (O-16:1_20:4) levels	Parkinson's disease	19.55	0.37
Phosphatidylethanolamine (O-16:1_22:5) levels	Parkinson's disease	16.18	0.52
Phosphatidylethanolamine (O-18:1_18:2) levels	Parkinson's disease	16.03	0.89
Phosphatidylethanolamine (O-18:1_20:4) levels	Parkinson's disease	48.76	0.00
Phosphatidylethanolamine (O-18:2_18:1) levels	Parkinson's disease	15.75	0.74
Phosphatidylethanolamine (O-18:2_18:2) levels	Parkinson's disease	41.17	0.00
Phosphatidylethanolamine (O-18:2_20:4) levels	Parkinson's disease	22.51	0.45
Phosphatidylinositol (16:0_18:1) levels	Parkinson's disease	19.12	0.14
Phosphatidylinositol (16:0_18:2) levels	Parkinson's disease	38.66	0.01
Phosphatidylinositol (16:0_20:4) levels	Parkinson's disease	35.33	0.03
Phosphatidylinositol (18:0_18:1) levels	Parkinson's disease	27.03	0.10
Phosphatidylinositol (18:0_18:2) levels	Parkinson's disease	49.46	0.02
Phosphatidylinositol (18:0_20:3) levels	Parkinson's disease	56.29	0.00
Phosphatidylinositol (18:0_20:4) levels	Parkinson's disease	31.41	0.12
Phosphatidylinositol (18:1_18:1) levels	Parkinson's disease	14.68	0.62
Phosphatidylinositol (18:1_18:2) levels	Parkinson's disease	15.81	0.67
Phosphatidylinositol (18:1_20:4) levels	Parkinson's disease	30.31	0.07
Sphingomyelin (d32:1) levels	Parkinson's disease	49.12	0.18
Sphingomyelin (d34:0) levels	Parkinson's disease	22.57	0.28
Sphingomyelin (d34:1) levels	Parkinson's disease	28.26	0.34
Sphingomyelin (d34:2) levels	Parkinson's disease	28.49	0.23
Sphingomyelin (d36:1) levels	Parkinson's disease	25.76	0.59
Sphingomyelin (d36:2) levels	Parkinson's disease	17.85	0.49

Sphingomyelin (d38:1) levels	Parkinson's disease	24.45	0.58
Sphingomyelin (d38:2) levels	Parkinson's disease	16.17	0.81
Sphingomyelin (d40:1) levels	Parkinson's disease	35.77	0.44
Sphingomyelin (d40:2) levels	Parkinson's disease	22.46	0.83
Sphingomyelin (d42:2) levels	Parkinson's disease	17.77	0.98
Triacylglycerol (46:1) levels	Parkinson's disease	40.51	0.01
Triacylglycerol (46:2) levels	Parkinson's disease	38.06	0.02
Triacylglycerol (48:0) levels	Parkinson's disease	22.03	0.12
Triacylglycerol (48:1) levels	Parkinson's disease	37.22	0.03
Triacylglycerol (48:2) levels	Parkinson's disease	29.92	0.07
Triacylglycerol (48:3) levels	Parkinson's disease	32.74	0.05
Triacylglycerol (49:1) levels	Parkinson's disease	39.26	0.02
Triacylglycerol (49:2) levels	Parkinson's disease	14.68	0.29
Triacylglycerol (50:1) levels	Parkinson's disease	43.72	0.01
Triacylglycerol (50:2) levels	Parkinson's disease	19.30	0.29
Triacylglycerol (50:3) levels	Parkinson's disease	46.86	0.01
Triacylglycerol (50:4) levels	Parkinson's disease	34.83	0.04
Triacylglycerol (50:5) levels	Parkinson's disease	40.33	0.01
Triacylglycerol (51:1) levels	Parkinson's disease	48.69	0.00
Triacylglycerol (51:2) levels	Parkinson's disease	29.68	0.09
Triacylglycerol (51:3) levels	Parkinson's disease	43.95	0.01
Triacylglycerol (51:4) levels	Parkinson's disease	45.60	0.00
Triacylglycerol (52:2) levels	Parkinson's disease	19.38	0.59
Triacylglycerol (52:3) levels	Parkinson's disease	39.61	0.07
Triacylglycerol (52:4) levels	Parkinson's disease	38.76	0.02
Triacylglycerol (52:5) levels	Parkinson's disease	35.06	0.06
Triacylglycerol (52:6) levels	Parkinson's disease	27.32	0.11
Triacylglycerol (53:2) levels	Parkinson's disease	26.70	0.19
Triacylglycerol (53:3) levels	Parkinson's disease	36.52	0.06
Triacylglycerol (53:4) levels	Parkinson's disease	41.41	0.04
Triacylglycerol (54:3) levels	Parkinson's disease	48.41	0.03
Triacylglycerol (54:4) levels	Parkinson's disease	36.19	0.05
Triacylglycerol (54:5) levels	Parkinson's disease	33.16	0.07
Triacylglycerol (54:6) levels	Parkinson's disease	24.38	0.16
Triacylglycerol (54:7) levels	Parkinson's disease	41.33	0.03
Triacylglycerol (56:3) levels	Parkinson's disease	39.16	0.02
Triacylglycerol (56:4) levels	Parkinson's disease	20.58	0.23
Triacylglycerol (56:5) levels	Parkinson's disease	26.74	0.10
Triacylglycerol (56:6) levels	Parkinson's disease	28.67	0.22
Triacylglycerol (56:7) levels	Parkinson's disease	32.45	0.25
Triacylglycerol (56:8) levels	Parkinson's disease	36.90	0.13
Triacylglycerol (58:7) levels	Parkinson's disease	28.83	0.01
Triacylglycerol (58:8) levels	Parkinson's disease	20.52	0.75

Supplementary table 3

Summary of instrumental variables used for all significant causal association evidence															
exposure	outcome	SNP	Effect_allele	Other_allele	Gene	Chr	Pos	EAF	F	Association with exposure			Association with outcome		
										beta	se	*P*-value	beta	se	*P*-value
Diacylglycerol (16:1_18:1) levels	Parkinson's Disease	rs10022344	T	C	NA	4	124943164	0.181	19.87	1.06E-01	2.37E-02	8.41E-06	7.97E-02	3.96E-02	2.21E-02
Diacylglycerol (16:1_18:1) levels	Parkinson's Disease	rs11182480	C	T	TMEM117	12	44386579	0.018	24.62	3.45E-01	6.96E-02	7.16E-07	-1.11E-02	6.60E-02	6.24E-02
Diacylglycerol (16:1_18:1) levels	Parkinson's Disease	rs112122274	G	A	CCDC187	9	136257994	0.036	24.51	-2.60E-01	5.25E-02	7.58E-07	4.76E-02	6.87E-02	3.11E-01
Diacylglycerol (16:1_18:1) levels	Parkinson's Disease	rs11775636	A	G	LYPD2	8	142752432	0.158	21.82	-1.13E-01	2.43E-02	3.05E-06	-2.10E-03	2.50E-02	3.05E-02
Diacylglycerol (16:1_18:1) levels	Parkinson's Disease	rs12050879	A	G	EIF2AK4	15	39983732	0.021	19.97	-2.78E-01	6.22E-02	7.99E-06	-4.75E-02	1.50E-01	1.24E-01
Diacylglycerol (16:1_18:1) levels	Parkinson's Disease	rs1260326	C	T	GCKR	2	27508073	0.651	35.93	-1.12E-01	1.87E-02	2.15E-09	-6.78E-02	1.72E-02	4.04E-05
Diacylglycerol (16:1_18:1) levels	Parkinson's Disease	rs143808674	G	A	ADGRL2	1	81671654	0.048	19.80	-1.86E-01	4.19E-02	8.76E-06	-2.87E-02	5.17E-02	2.37E-01
Diacylglycerol (16:1_18:1) levels	Parkinson's Disease	rs2182380	G	A	POLR1C, LINC01512, LOC105375070	6	43900875	0.031	23.01	2.61E-01	5.44E-02	1.65E-06	8.54E-02	5.97E-02	8.18E-01
Diacylglycerol (16:1_18:1) levels	Parkinson's Disease	rs2524075	T	C	NA	6	31276082	0.208	24.13	-1.07E-01	2.18E-02	9.22E-07	-4.53E-02	2.14E-02	1.71E-02
Diacylglycerol (16:1_18:1) levels	Parkinson's Disease	rs3829088	C	T	ELAVL2	9	23713682	0.203	20.64	1.02E-01	2.24E-02	5.65E-06	2.59E-02	3.15E-02	3.86E-01
Diacylglycerol (16:1_18:1) levels	Parkinson's Disease	rs4419068	A	G	LOC102723786	16	71309806	0.237	19.56	-9.31E-02	2.11E-02	9.89E-06	2.00E-04	1.94E-02	3.40E-03
Diacylglycerol (16:1_18:1) levels	Parkinson's Disease	rs77134026	A	G	NA	7	36495127	0.031	21.73	2.58E-01	5.53E-02	3.20E-06	-1.49E-02	8.36E-02	6.65E-02
Diacylglycerol (16:1_18:1) levels	Parkinson's Disease	rs7955732	T	G	TRHDE	12	72331215	0.423	23.55	-8.86E-02	1.83E-02	1.24E-06	8.90E-03	2.26E-02	1.58E-01
Diacylglycerol (16:1_18:1) levels	Parkinson's Disease	rs908150	C	T	PRKCA	17	66540315	0.428	19.63	8.07E-02	1.82E-02	9.56E-06	2.77E-02	2.26E-02	6.55E-01
Diacylglycerol (16:1_18:1) levels	Parkinson's Disease	rs964184	C	G	ZPR1	11	116778201	0.849	44.23	-1.64E-01	2.47E-02	3.17E-11	-5.68E-02	2.43E-02	9.71E-03
Diacylglycerol (18:1_18:1) levels	Parkinson's Disease	rs10169217	G	A	ERBB4	2	212010621	0.676	29.08	-9.83E-02	1.82E-02	7.18E-08	-2.56E-02	2.37E-02	5.51E-01
Diacylglycerol (18:1_18:1) levels	Parkinson's Disease	rs1042034	T	C	APOB	2	21002409	0.728	29.79	1.04E-01	1.90E-02	4.97E-08	-5.30E-03	2.03E-02	9.96E-02
Diacylglycerol (18:1_18:1) levels	Parkinson's Disease	rs1060743	G	A	BIN1	2	127068957	0.291	19.77	-8.35E-02	1.88E-02	8.86E-06	-9.20E-03	1.82E-02	2.13E-01
Diacylglycerol (18:1_18:1) levels	Parkinson's Disease	rs116011373	A	G	NA	3	87298641	0.049	21.82	-1.86E-01	3.98E-02	3.06E-06	-2.23E-02	8.48E-02	1.01E-01
Diacylglycerol (18:1_18:1) levels	Parkinson's Disease	rs116796527	T	C	NA	4	62344980	0.014	22.07	-3.37E-01	7.18E-02	2.68E-06	-1.78E-01	1.09E-01	9.93E-01
Diacylglycerol (18:1_18:1) levels	Parkinson's Disease	rs12365864	G	A	NA	11	116618319	0.166	20.67	1.05E-01	2.31E-02	5.55E-06	-2.95E-02	2.73E-02	5.55E-01
Diacylglycerol (18:1_18:1) levels	Parkinson's Disease	rs1260326	C	T	GCKR	2	27508073	0.651	47.53	-1.23E-01	1.78E-02	5.88E-12	-6.78E-02	1.72E-02	4.04E-05
Diacylglycerol (18:1_18:1) levels	Parkinson's Disease	rs137871178	C	T	ARMH4	14	58138012	0.017	19.87	2.99E-01	6.71E-02	8.42E-06	-1.60E-03	1.19E-01	4.80E-03
Diacylglycerol (18:1_18:1) levels	Parkinson's Disease	rs139500046	C	A	CFAP54	12	96850670	0.011	23.73	-3.93E-01	8.07E-02	1.13E-06	-4.41E-02	8.65E-02	2.15E-01
Diacylglycerol (18:1_18:1) levels	Parkinson's Disease	rs17596144	T	C	MTARC1	1	220805303	0.152	30.09	1.31E-01	2.39E-02	4.26E-08	-2.90E-03	2.58E-02	4.12E-02
Diacylglycerol (18:1_18:1) levels	Parkinson's Disease	rs268	G	A	LPL	8	19956018	0.023	23.52	2.81E-01	5.80E-02	1.26E-06	1.59E-02	6.69E-02	9.03E-02
Diacylglycerol (18:1_18:1) levels	Parkinson's Disease	rs2954021	G	A	NA	8	125469835	0.538	19.73	-7.58E-02	1.71E-02	9.05E-06	-1.31E-02	1.69E-02	3.58E-01
Diacylglycerol (18:1_18:1) levels	Parkinson's Disease	rs35332062	A	G	MLXIPL	7	73597712	0.122	30.70	-1.44E-01	2.59E-02	3.11E-08	-4.60E-03	2.72E-02	6.20E-02
Diacylglycerol (18:1_18:1) levels	Parkinson's Disease	rs36094040	C	T	TRHDE	12	72431183	0.348	20.88	-8.29E-02	1.81E-02	4.96E-06	-2.90E-03	2.33E-02	4.53E-02
Diacylglycerol (18:1_18:1) levels	Parkinson's Disease	rs62046303	C	T	NA	16	82018393	0.017	21.40	3.25E-01	7.03E-02	3.80E-06	1.59E-02	5.19E-02	1.19E-01
Diacylglycerol (18:1_18:1) levels	Parkinson's Disease	rs62291573	C	T	NA	4	4999649	0.214	24.68	1.07E-01	2.15E-02	6.91E-07	-2.81E-02	2.75E-02	5.13E-01
Diacylglycerol (18:1_18:1) levels	Parkinson's Disease	rs76950187	G	A	LOC105373611	2	128427075	0.074	24.05	1.60E-01	3.26E-02	9.58E-07	6.08E-02	5.53E-02	5.65E-01
Diacylglycerol (18:1_18:1) levels	Parkinson's Disease	rs7782961	T	A	NA	7	6295952	0.232	21.55	9.46E-02	2.04E-02	3.51E-06	5.86E-02	2.64E-02	1.32E-02
Diacylglycerol (18:1_18:1) levels	Parkinson's Disease	rs964184	C	G	ZPR1	11	116778201	0.849	126.77	-2.64E-01	2.34E-02	3.74E-29	-5.68E-02	2.43E-02	9.71E-03
Diacylglycerol (18:1_18:1) levels	Parkinson's Disease	rs9865586	G	A	CPNE4	3	132015999	0.165	22.33	-1.07E-01	2.26E-02	2.33E-06	-4.00E-03	2.72E-02	5.36E-02
Lysophosphatidylcholine (18:0) levels	Parkinson's Disease	rs10095602	T	C	NA	8	53669039	0.052	21.40	1.76E-01	3.80E-02	3.79E-06	5.12E-02	5.42E-02	4.63E-01
Lysophosphatidylcholine (18:0) levels	Parkinson's Disease	rs1018070	A	T	NA	8	18416117	0.061	25.01	1.77E-01	3.55E-02	5.83E-07	-6.56E-02	4.59E-02	8.14E-01
Lysophosphatidylcholine (18:0) levels	Parkinson's Disease	rs1146461	A	G	CNN3-DT	1	94942936	0.463	23.10	-7.99E-02	1.66E-02	1.57E-06	-9.90E-03	2.28E-02	1.77E-01
Lysophosphatidylcholine (18:0) levels	Parkinson's Disease	rs11582724	T	C	NA	1	201132736	0.575	21.01	-7.83E-02	1.71E-02	4.64E-06	3.95E-02	1.84E-02	1.59E-02
Lysophosphatidylcholine (18:0) levels	Parkinson's Disease	rs117140365	C	G	KAT6B	10	74861163	0.016	24.00	-3.29E-01	6.72E-02	9.83E-07	9.79E-02	8.33E-02	6.20E-01
Lysophosphatidylcholine (18:0) levels	Parkinson's Disease	rs11780085	T	A	GATA4	8	11680104	0.008	19.76	4.52E-01	1.02E-01	8.92E-06	-2.68E-01	1.34E-01	2.29E-02
Lysophosphatidylcholine (18:0) levels	Parkinson's Disease	rs145366219	G	A	APTX	9	33013634	0.067	22.07	-1.58E-01	3.37E-02	2.68E-06	6.62E-02	3.63E-02	3.41E-02
Lysophosphatidylcholine (18:0) levels	Parkinson's Disease	rs56121911	G	A	NA	3	45251126	0.097	20.78	1.31E-01	2.86E-02	5.24E-06	-2.77E-02	3.30E-02	3.97E-01
Lysophosphatidylcholine (18:0) levels	Parkinson's Disease	rs59634655	T	G	WWOX	16	78457680	0.163	22.23	-1.08E-01	2.29E-02	2.46E-06	5.70E-03	2.93E-02	7.32E-02
Lysophosphatidylcholine (18:0) levels	Parkinson's Disease	rs7048143	T	C	ROR2	9	91806878	0.247	19.58	-8.77E-02	1.98E-02	9.79E-06	1.19E-02	3.33E-02	1.43E-01
Lysophosphatidylcholine (18:0) levels	Parkinson's Disease	rs73040346	C	T	CCDC141	2	178827066	0.031	19.90	-2.17E-01	4.86E-02	8.28E-06	2.75E-02	4.05E-02	3.03E-01
Lysophosphatidylcholine (18:0) levels	Parkinson's Disease	rs7529794	T	G	NA	1	39937698	0.250	27.46	1.01E-01	1.94E-02	1.65E-07	-2.50E-02	2.57E-02	4.81E-01

Lysophosphatidylcholine (18:0) levels	Parkinson's Disease	rs75668753	G	A	LINC02147	5	117857598	0.021	20.48	-2.68E-01	5.92E-02	6.12E-06	-5.29E-02	7.47E-02	3.20E-01
Lysophosphatidylcholine (18:0) levels	Parkinson's Disease	rs76179782	A	C	PALD1	10	70458313	0.044	20.53	1.99E-01	4.38E-02	5.97E-06	-1.08E-01	4.90E-02	1.41E-02
Lysophosphatidylcholine (18:0) levels	Parkinson's Disease	rs76309137	T	C	NA	7	95573476	0.013	21.48	-3.51E-01	7.57E-02	3.63E-06	-1.37E-01	7.96E-02	4.22E-02
Lysophosphatidylcholine (18:0) levels	Parkinson's Disease	rs76530357	T	G	ROBO2	3	77008436	0.025	23.87	2.66E-01	5.45E-02	1.05E-06	-9.45E-02	1.44E-01	2.91E-01
Lysophosphatidylcholine (18:0) levels	Parkinson's Disease	rs77751297	T	G	SNRPF-DT	12	95853480	0.165	24.83	1.13E-01	2.26E-02	6.41E-07	-1.44E-02	2.57E-02	2.40E-01
Lysophosphatidylcholine (18:0) levels	Parkinson's Disease	rs78392466	T	A	LOC107984041	6	101082967	0.038	22.65	-2.09E-01	4.39E-02	1.98E-06	2.17E-01	8.64E-02	6.05E-03
Lysophosphatidylcholine (18:0) levels	Parkinson's Disease	rs79713567	T	C	SLC25A21	14	36841591	0.050	20.96	-1.87E-01	4.08E-02	4.75E-06	-6.50E-03	6.26E-02	3.78E-02
Lysophosphatidylcholine (18:0) levels	Parkinson's Disease	rs797821	C	T	SEMA3A	7	83961536	0.561	20.17	-7.54E-02	1.68E-02	7.21E-06	3.53E-02	2.24E-02	9.41E-01
Phosphatidylcholine (18:1_18:3) levels	Parkinson's Disease	rs10769099	A	G	NA	11	5586584	0.337	22.03	8.83E-02	1.88E-02	2.73E-06	2.20E-03	1.83E-02	4.42E-02
Phosphatidylcholine (18:1_18:3) levels	Parkinson's Disease	rs1116931	A	C	ABI3BP	3	100870486	0.272	22.00	-9.25E-02	1.97E-02	2.78E-06	-2.99E-02	2.40E-02	6.72E-01
Phosphatidylcholine (18:1_18:3) levels	Parkinson's Disease	rs114797690	T	C	NA	1	98597981	0.023	20.93	2.85E-01	6.22E-02	4.84E-06	2.25E-02	6.22E-02	1.44E-01
Phosphatidylcholine (18:1_18:3) levels	Parkinson's Disease	rs116910056	T	A	NA	11	80166090	0.009	19.57	-4.18E-01	9.46E-02	9.84E-06	-5.34E-02	8.70E-02	2.68E-01
Phosphatidylcholine (18:1_18:3) levels	Parkinson's Disease	rs140885981	A	G	CFDP1	16	75302415	0.008	21.36	-4.77E-01	1.03E-01	3.89E-06	1.05E-01	1.54E-01	3.04E-01
Phosphatidylcholine (18:1_18:3) levels	Parkinson's Disease	rs144128487	G	C	KLHL32	6	97082368	0.012	23.57	-3.99E-01	8.21E-02	1.23E-06	-1.40E-01	9.38E-02	8.67E-01
Phosphatidylcholine (18:1_18:3) levels	Parkinson's Disease	rs148879093	A	G	NA	12	96120840	0.079	20.01	-1.53E-01	3.43E-02	7.84E-06	-1.14E-01	7.12E-02	9.65E-01
Phosphatidylcholine (18:1_18:3) levels	Parkinson's Disease	rs149851240	C	G	LOC105374506	2	41556996	0.046	20.67	1.95E-01	4.29E-02	5.55E-06	2.34E-02	7.30E-02	1.26E-01
Phosphatidylcholine (18:1_18:3) levels	Parkinson's Disease	rs17208010	T	C	NRG2	5	139952900	0.084	19.78	1.44E-01	3.25E-02	8.83E-06	5.42E-02	3.78E-02	8.18E-01
Phosphatidylcholine (18:1_18:3) levels	Parkinson's Disease	rs17231506	T	C	CETP	16	56960616	0.278	20.29	8.90E-02	1.98E-02	6.76E-06	1.36E-02	1.81E-02	3.45E-01
Phosphatidylcholine (18:1_18:3) levels	Parkinson's Disease	rs174601	T	C	FADS2	11	61855668	0.419	19.90	8.01E-02	1.80E-02	8.29E-06	-3.70E-03	1.79E-02	7.75E-02
Phosphatidylcholine (18:1_18:3) levels	Parkinson's Disease	rs2774509	C	G	LY86-AS1	6	6411842	0.664	20.16	-8.38E-02	1.87E-02	7.22E-06	-3.69E-02	2.44E-02	8.85E-01
Phosphatidylcholine (18:1_18:3) levels	Parkinson's Disease	rs9480321	T	A	SNORD28B	6	156378387	0.050	24.91	-2.04E-01	4.09E-02	6.16E-07	2.13E-02	5.53E-02	1.55E-01
Phosphatidylcholine (18:1_18:3) levels	Parkinson's Disease	rs9559745	C	T	COL4A1	13	110209642	0.009	19.84	4.26E-01	9.55E-02	8.58E-06	4.29E-02	8.17E-02	2.22E-01
Phosphatidylethanolamine (18:0_18:2) levels	Parkinson's Disease	rs10069895	C	T	NBPF22P	5	86297284	0.764	24.61	9.72E-02	1.96E-02	7.18E-07	3.63E-02	3.17E-02	5.98E-01
Phosphatidylethanolamine (18:0_18:2) levels	Parkinson's Disease	rs10276778	C	T	CNTNAP2	7	146994417	0.044	20.34	-1.82E-01	4.03E-02	6.58E-06	-5.68E-02	7.64E-02	3.40E-01
Phosphatidylethanolamine (18:0_18:2) levels	Parkinson's Disease	rs10468017	T	C	NA	15	58386313	0.338	299.64	2.98E-01	1.72E-02	9.03E-66	2.75E-02	1.87E-02	8.48E-01
Phosphatidylethanolamine (18:0_18:2) levels	Parkinson's Disease	rs111543310	C	T	MYO1E	15	59239619	0.022	21.53	2.80E-01	6.04E-02	3.55E-06	2.90E-03	9.70E-02	1.05E-02
Phosphatidylethanolamine (18:0_18:2) levels	Parkinson's Disease	rs112129861	C	G	RNF111	15	59047450	0.065	21.34	1.56E-01	3.38E-02	3.91E-06	-1.08E-01	4.96E-02	1.45E-02
Phosphatidylethanolamine (18:0_18:2) levels	Parkinson's Disease	rs117376818	A	G	LIPC	15	58506762	0.016	80.24	6.30E-01	7.04E-02	4.16E-19	1.24E-01	1.67E-01	3.41E-01
Phosphatidylethanolamine (18:0_18:2) levels	Parkinson's Disease	rs117900629	A	G	NA	11	62205328	0.062	38.35	-2.13E-01	3.45E-02	6.25E-10	-2.16E-02	7.58E-02	1.11E-01
Phosphatidylethanolamine (18:0_18:2) levels	Parkinson's Disease	rs1260326	C	T	GCKR	2	27508073	0.651	49.15	-1.22E-01	1.74E-02	2.57E-12	-6.78E-02	1.72E-02	4.04E-05
Phosphatidylethanolamine (18:0_18:2) levels	Parkinson's Disease	rs12928099	A	C	PDXDC1, NTAN1	16	15056648	0.274	36.63	-1.13E-01	1.86E-02	1.50E-09	-1.09E-02	1.84E-02	2.58E-01
Phosphatidylethanolamine (18:0_18:2) levels	Parkinson's Disease	rs13110318	A	G	TBC1D1	4	38137235	0.114	21.43	-1.21E-01	2.61E-02	3.74E-06	6.23E-02	3.73E-02	4.74E-02
Phosphatidylethanolamine (18:0_18:2) levels	Parkinson's Disease	rs138283783	C	T	LOC124903499	15	58244328	0.027	43.10	3.52E-01	5.36E-02	5.56E-11	1.05E-01	1.36E-01	3.58E-01
Phosphatidylethanolamine (18:0_18:2) levels	Parkinson's Disease	rs141883259	G	A	NA	5	120061007	0.010	20.34	3.73E-01	8.26E-02	6.59E-06	1.48E-01	1.01E-01	8.40E-01
Phosphatidylethanolamine (18:0_18:2) levels	Parkinson's Disease	rs149431055	T	C	NA	4	115874677	0.025	19.72	-2.43E-01	5.47E-02	9.10E-06	4.62E-02	6.01E-02	3.54E-01
Phosphatidylethanolamine (18:0_18:2) levels	Parkinson's Disease	rs2946493	C	T	TRMT9B	8	12966162	0.206	25.58	1.05E-01	2.08E-02	4.35E-07	-2.73E-02	2.60E-02	5.32E-01
Phosphatidylethanolamine (18:0_18:2) levels	Parkinson's Disease	rs34733845	T	C	NA	12	60861641	0.059	21.37	1.65E-01	3.57E-02	3.86E-06	5.54E-02	7.14E-02	3.59E-01
Phosphatidylethanolamine (18:0_18:2) levels	Parkinson's Disease	rs4296402	A	G	NA	2	174696563	0.160	21.07	1.06E-01	2.30E-02	4.49E-06	1.01E-02	3.80E-02	1.02E-01
Phosphatidylethanolamine (18:0_18:2) levels	Parkinson's Disease	rs62291572	T	C	NA	4	4996200	0.214	22.67	1.00E-01	2.10E-02	1.96E-06	-2.71E-02	2.75E-02	4.89E-01
Phosphatidylethanolamine (18:0_18:2) levels	Parkinson's Disease	rs62434228	A	G	NA	6	154681357	0.160	22.32	1.09E-01	2.30E-02	2.35E-06	1.69E-02	3.92E-02	1.76E-01
Phosphatidylethanolamine (18:0_18:2) levels	Parkinson's Disease	rs633695	G	A	LIPC	15	58433640	0.314	200.84	2.55E-01	1.80E-02	5.56E-45	2.32E-02	2.19E-02	5.39E-01
Phosphatidylethanolamine (18:0_18:2) levels	Parkinson's Disease	rs7226158	C	T	RBFOX3	17	79394643	0.684	20.50	-8.08E-02	1.78E-02	6.06E-06	1.94E-02	2.46E-02	3.67E-01
Phosphatidylethanolamine (18:0_18:2) levels	Parkinson's Disease	rs72679383	T	G	NA	4	100929253	0.022	23.89	2.86E-01	5.85E-02	1.04E-06	6.22E-02	1.18E-01	2.24E-01
Phosphatidylethanolamine (18:0_18:2) levels	Parkinson's Disease	rs73424597	C	T	ADAM10	15	58607410	0.035	61.20	3.55E-01	4.53E-02	5.88E-15	2.31E-02	6.42E-02	1.43E-01
Phosphatidylethanolamine (18:0_18:2) levels	Parkinson's Disease	rs805292	A	G	MPIG6B, LY6G6C	6	31722232	0.378	23.79	8.50E-02	1.74E-02	1.10E-06	-5.20E-03	2.24E-02	8.72E-02
Phosphatidylethanolamine (18:0_18:2) levels	Parkinson's Disease	rs964184	C	G	ZPR1	11	116778201	0.849	44.48	-1.54E-01	2.32E-02	2.75E-11	-5.68E-02	2.43E-02	9.71E-03
Sphingomyelin (d38:1) levels	Parkinson's Disease	rs114483871	T	C	ANKRD17	4	73124451	0.038	35.89	2.68E-01	4.48E-02	2.19E-09	-2.94E-02	8.47E-02	1.38E-01
Sphingomyelin (d38:1) levels	Parkinson's Disease	rs11591147	T	G	PCSK9	1	55039974	0.033	41.81	-3.01E-01	4.65E-02	1.07E-10	-3.77E-02	6.83E-02	2.36E-01
Sphingomyelin (d38:1) levels	Parkinson's Disease	rs117146578	C	T	DRAM1	12	101922985	0.048	21.68	1.83E-01	3.92E-02	3.27E-06	-1.42E-01	3.85E-02	1.18E-04
Sphingomyelin (d38:1) levels	Parkinson's Disease	rs117643293	T	C	NA	17	5065669	0.062	51.41	-2.43E-01	3.39E-02	8.25E-13	9.73E-02	5.79E-02	4.64E-02
Sphingomyelin (d38:1) levels	Parkinson's Disease	rs118070067	A	C	NA	12	61250942	0.022	20.14	2.58E-01	5.74E-02	7.31E-06	5.38E-02	8.01E-02	3.00E-01
Sphingomyelin (d38:1) levels	Parkinson's Disease	rs13258507	G	T	NA	8	125480355	0.241	22.70	9.26E-02	1.94E-02	1.92E-06	-1.41E-02	2.24E-02	2.77E-01
Sphingomyelin (d38:1) levels	Parkinson's Disease	rs1452773	G	T	NRXN1	2	50050811	0.138	21.18	-1.10E-01	2.39E-02	4.26E-06	4.96E-02	3.27E-02	8.89E-01

Sphingomyelin (d38:1) levels	Parkinson's Disease	rs147426822	T	C	LOC107986931	8	23959870	0.013	20.88	3.59E-01	7.86E-02	4.97E-06	2.09E-02	7.75E-02	1.04E-01
Sphingomyelin (d38:1) levels	Parkinson's Disease	rs182695896	C	A	NA	4	73947510	0.025	49.20	3.71E-01	5.28E-02	2.53E-12	1.53E-01	1.83E-01	3.94E-01
Sphingomyelin (d38:1) levels	Parkinson's Disease	rs186039163	G	T	TRPV3	17	3547599	0.010	20.62	-3.81E-01	8.40E-02	5.68E-06	-2.60E-02	1.46E-01	6.63E-02
Sphingomyelin (d38:1) levels	Parkinson's Disease	rs188346987	G	C	NA	2	157097431	0.051	20.32	-1.78E-01	3.95E-02	6.67E-06	-1.93E-02	7.37E-02	1.01E-01
Sphingomyelin (d38:1) levels	Parkinson's Disease	rs2867985	A	G	TBC1D2B	15	78071433	0.305	20.25	8.20E-02	1.82E-02	6.90E-06	1.07E-02	2.04E-02	2.21E-01
Sphingomyelin (d38:1) levels	Parkinson's Disease	rs3741111	G	A	NA	11	124076588	0.483	26.91	8.64E-02	1.67E-02	2.19E-07	-5.00E-04	1.95E-02	8.69E-03
Sphingomyelin (d38:1) levels	Parkinson's Disease	rs715119	T	C	CT70	9	104975112	0.233	21.78	-9.19E-02	1.97E-02	3.11E-06	6.90E-03	2.62E-02	1.01E-01
Sphingomyelin (d38:1) levels	Parkinson's Disease	rs7157785	T	G	SYNE2	14	63768838	0.125	223.06	3.70E-01	2.48E-02	1.11E-49	-3.20E-02	2.25E-02	8.11E-01
Sphingomyelin (d38:1) levels	Parkinson's Disease	rs7172051	G	A	LOC101929129	15	69995976	0.373	19.67	7.71E-02	1.74E-02	9.32E-06	-6.60E-03	2.29E-02	1.11E-01
Sphingomyelin (d38:1) levels	Parkinson's Disease	rs73073606	C	A	NA	3	194176577	0.241	22.96	-9.65E-02	2.01E-02	1.68E-06	2.07E-02	2.49E-02	3.91E-01
Sphingomyelin (d38:1) levels	Parkinson's Disease	rs73193962	A	C	NA	3	105974935	0.026	20.39	-2.43E-01	5.39E-02	6.41E-06	2.13E-02	6.98E-02	1.19E-01
Sphingomyelin (d38:1) levels	Parkinson's Disease	rs7478167	A	T	NA	10	101716501	0.669	21.63	-8.30E-02	1.78E-02	3.36E-06	-3.50E-03	1.91E-02	6.80E-02
Sphingomyelin (d38:1) levels	Parkinson's Disease	rs75679663	A	C	NA	17	4764677	0.026	78.68	-4.74E-01	5.35E-02	9.06E-19	1.35E-01	9.72E-02	7.79E-01
Sphingomyelin (d38:1) levels	Parkinson's Disease	rs75740927	G	C	LOC105369441	11	95614411	0.031	20.15	2.30E-01	5.11E-02	7.25E-06	-2.02E-02	9.58E-02	7.94E-02
Sphingomyelin (d38:1) levels	Parkinson's Disease	rs76174849	T	C	RERE	1	8738706	0.014	21.59	3.34E-01	7.19E-02	3.44E-06	-2.82E-02	8.49E-02	1.31E-01
Sphingomyelin (d38:1) levels	Parkinson's Disease	rs77645768	A	G	NA	4	72803111	0.026	32.09	3.04E-01	5.36E-02	1.53E-08	5.69E-02	7.75E-02	3.34E-01
Sphingomyelin (d38:1) levels	Parkinson's Disease	rs79231873	G	A	NA	8	53151614	0.020	20.12	2.94E-01	6.55E-02	7.37E-06	6.94E-02	7.57E-02	4.44E-01
Sphingomyelin (d38:1) levels	Parkinson's Disease	rs7970512	A	C	NA	12	67513820	0.035	22.23	-2.25E-01	4.78E-02	2.46E-06	7.19E-02	1.01E-01	3.23E-01
Triacylglycerol (48:0) levels	Parkinson's Disease	rs117264209	G	C	NA	9	135992678	0.019	20.94	-3.31E-01	7.24E-02	4.84E-06	4.21E-02	6.36E-02	2.94E-01
Triacylglycerol (48:0) levels	Parkinson's Disease	rs117945317	A	T	BLK	8	11538263	0.009	19.56	-4.75E-01	1.07E-01	9.91E-06	-1.61E-01	1.26E-01	6.94E-01
Triacylglycerol (48:0) levels	Parkinson's Disease	rs140828820	G	A	NA	4	34767471	0.011	19.99	4.24E-01	9.49E-02	7.93E-06	1.72E-01	2.99E-01	2.48E-01
Triacylglycerol (48:0) levels	Parkinson's Disease	rs222507	T	G	CHPT1	12	101725095	0.979	21.46	-3.23E-01	6.97E-02	3.68E-06	-1.39E-01	8.40E-02	4.95E-02
Triacylglycerol (48:0) levels	Parkinson's Disease	rs2242104	G	A	VLDLR	9	2640492	0.752	21.23	-1.03E-01	2.22E-02	4.16E-06	-6.60E-03	2.32E-02	1.09E-01
Triacylglycerol (48:0) levels	Parkinson's Disease	rs4955801	C	T	NA	3	179092711	0.785	21.26	-1.07E-01	2.32E-02	4.09E-06	-3.90E-02	2.61E-02	8.70E-01
Triacylglycerol (48:0) levels	Parkinson's Disease	rs60243822	C	A	PPFIA2	12	81637055	0.172	19.99	1.14E-01	2.55E-02	7.93E-06	6.69E-02	3.02E-02	1.34E-02
Triacylglycerol (48:0) levels	Parkinson's Disease	rs62348866	A	G	F11-AS1	4	186453244	0.118	22.63	-1.41E-01	2.97E-02	2.01E-06	2.94E-02	3.29E-02	4.30E-01
Triacylglycerol (48:0) levels	Parkinson's Disease	rs73302224	C	T	ARHGAP22	10	48593367	0.007	20.00	5.18E-01	1.16E-01	7.88E-06	1.02E-01	1.39E-01	3.34E-01
Triacylglycerol (48:0) levels	Parkinson's Disease	rs769432	T	C	OR3A3	17	3420961	0.509	22.60	9.10E-02	1.91E-02	2.04E-06	5.85E-02	1.75E-02	4.15E-04
Triacylglycerol (48:0) levels	Parkinson's Disease	rs7721676	C	T	LOC105378224	5	149703644	0.218	19.87	-1.04E-01	2.33E-02	8.45E-06	-1.91E-02	2.55E-02	3.42E-01
Triacylglycerol (48:0) levels	Parkinson's Disease	rs79159286	G	T	PDE1C	7	31677697	0.080	20.80	1.63E-01	3.57E-02	5.20E-06	4.20E-02	3.38E-02	6.69E-01
Triacylglycerol (48:0) levels	Parkinson's Disease	rs969816	G	A	NA	2	210779037	0.120	19.73	1.33E-01	3.00E-02	9.09E-06	-4.59E-02	4.45E-02	5.19E-01
Triacylglycerol (49:2) levels	Parkinson's Disease	rs10468471	C	T	NA	17	12222568	0.065	19.58	-1.64E-01	3.70E-02	9.83E-06	-6.22E-02	4.71E-02	7.29E-01
Triacylglycerol (49:2) levels	Parkinson's Disease	rs10861498	A	G	LOC107984435	12	105758014	0.242	22.39	-9.96E-02	2.10E-02	2.27E-06	-1.05E-02	2.88E-02	1.46E-01
Triacylglycerol (49:2) levels	Parkinson's Disease	rs1260326	C	T	GCKR	2	27508073	0.651	40.39	-1.20E-01	1.89E-02	2.23E-10	-6.78E-02	1.72E-02	4.04E-05
Triacylglycerol (49:2) levels	Parkinson's Disease	rs143838492	A	G	NA	5	114892988	0.003	22.76	7.58E-01	1.59E-01	1.88E-06	1.82E-01	1.54E-01	6.27E-01
Triacylglycerol (49:2) levels	Parkinson's Disease	rs145055632	G	A	NA	6	50783096	0.006	21.14	-5.16E-01	1.12E-01	4.36E-06	6.11E-02	1.06E-01	2.49E-01
Triacylglycerol (49:2) levels	Parkinson's Disease	rs2736863	G	T	NA	8	133451589	0.992	20.95	4.63E-01	1.01E-01	4.81E-06	1.17E-01	8.11E-02	8.24E-01
Triacylglycerol (49:2) levels	Parkinson's Disease	rs4937417	G	A	NA	11	129348709	0.467	21.62	-8.49E-02	1.83E-02	3.39E-06	-3.74E-02	2.24E-02	4.75E-02
Triacylglycerol (49:2) levels	Parkinson's Disease	rs6846577	A	G	TECRL	4	64367871	0.215	21.18	-1.01E-01	2.19E-02	4.27E-06	-4.00E-03	2.19E-02	6.85E-02
Triacylglycerol (49:2) levels	Parkinson's Disease	rs76411296	T	G	FAM81A	15	59517420	0.116	22.38	-1.36E-01	2.87E-02	2.28E-06	-7.10E-03	3.41E-02	7.87E-02
Triacylglycerol (49:2) levels	Parkinson's Disease	rs964184	C	G	ZPR1	11	116778201	0.849	38.60	-1.55E-01	2.49E-02	5.55E-10	-5.68E-02	2.43E-02	9.71E-03
Triacylglycerol (50:2) levels	Parkinson's Disease	rs1010313	T	C	TOX	8	59089983	0.198	19.91	9.27E-02	2.08E-02	8.23E-06	2.37E-02	2.73E-02	4.15E-01
Triacylglycerol (50:2) levels	Parkinson's Disease	rs10213073	G	T	NA	4	81524180	0.306	19.68	-8.09E-02	1.82E-02	9.29E-06	1.55E-02	1.99E-02	3.61E-01
Triacylglycerol (50:2) levels	Parkinson's Disease	rs10861498	A	G	LOC107984435	12	105758014	0.242	19.56	-8.46E-02	1.91E-02	9.89E-06	-1.05E-02	2.88E-02	1.46E-01
Triacylglycerol (50:2) levels	Parkinson's Disease	rs111430300	G	A	CRIM1	2	36390910	0.135	21.63	-1.12E-01	2.42E-02	3.36E-06	2.48E-02	2.76E-02	4.33E-01
Triacylglycerol (50:2) levels	Parkinson's Disease	rs112122274	G	A	CCDC187	9	136257994	0.036	20.27	-2.19E-01	4.85E-02	6.84E-06	4.76E-02	6.87E-02	3.11E-01
Triacylglycerol (50:2) levels	Parkinson's Disease	rs1260326	C	T	GCKR	2	27508073	0.651	63.88	-1.39E-01	1.74E-02	1.53E-15	-6.78E-02	1.72E-02	4.04E-05
Triacylglycerol (50:2) levels	Parkinson's Disease	rs143838492	A	G	NA	5	114892988	0.003	23.71	7.23E-01	1.48E-01	1.15E-06	1.82E-01	1.54E-01	6.27E-01
Triacylglycerol (50:2) levels	Parkinson's Disease	rs17704001	T	G	NA	15	92583935	0.226	19.69	8.89E-02	2.00E-02	9.24E-06	1.20E-02	2.58E-02	1.92E-01
Triacylglycerol (50:2) levels	Parkinson's Disease	rs4615671	T	C	NA	9	24032283	0.058	20.19	1.62E-01	3.60E-02	7.13E-06	-3.50E-03	4.55E-02	2.74E-02
Triacylglycerol (50:2) levels	Parkinson's Disease	rs72753379	T	C	LOC105376253	9	120350676	0.039	23.29	2.16E-01	4.47E-02	1.42E-06	2.30E-02	9.08E-02	9.67E-02
Triacylglycerol (50:2) levels	Parkinson's Disease	rs78275146	T	C	NA	1	196028959	0.014	20.33	3.40E-01	7.53E-02	6.59E-06	7.90E-02	1.03E-01	3.52E-01
Triacylglycerol (50:2) levels	Parkinson's Disease	rs79416109	G	T	LOC107985096	1	102847102	0.104	20.78	1.28E-01	2.80E-02	5.23E-06	5.42E-02	5.36E-02	5.06E-01

Triacylglycerol (50:2) levels	Parkinson's Disease	rs79934453	G	A	NA	6	23919006	0.004	22.04	-6.43E-01	1.37E-01	2.72E-06	-9.28E-02	1.77E-01	2.22E-01
Triacylglycerol (50:2) levels	Parkinson's Disease	rs964184	C	G	ZPR1	11	116778201	0.849	63.24	-1.84E-01	2.31E-02	2.10E-15	-5.68E-02	2.43E-02	9.71E-03
Triacylglycerol (52:2) levels	Parkinson's Disease	rs1010313	T	C	TOX	8	59089983	0.198	21.60	9.65E-02	2.08E-02	3.42E-06	2.37E-02	2.73E-02	4.15E-01
Triacylglycerol (52:2) levels	Parkinson's Disease	rs10147474	G	A	PRKD1	14	29823442	0.037	23.44	-2.16E-01	4.47E-02	1.32E-06	3.15E-02	6.22E-02	2.13E-01
Triacylglycerol (52:2) levels	Parkinson's Disease	rs10169217	G	A	ERBB4	2	212010621	0.676	22.13	-8.41E-02	1.79E-02	2.59E-06	-2.56E-02	2.37E-02	5.51E-01
Triacylglycerol (52:2) levels	Parkinson's Disease	rs1042034	T	C	APOB	2	21002409	0.728	23.49	9.03E-02	1.86E-02	1.28E-06	-5.30E-03	2.03E-02	9.96E-02
Triacylglycerol (52:2) levels	Parkinson's Disease	rs11017845	C	T	TCERG1L	10	131254190	0.047	19.61	-1.76E-01	3.96E-02	9.65E-06	-3.36E-02	5.37E-02	2.75E-01
Triacylglycerol (52:2) levels	Parkinson's Disease	rs112122274	G	A	CCDC187	9	136257994	0.036	24.29	-2.39E-01	4.85E-02	8.47E-07	4.76E-02	6.87E-02	3.11E-01
Triacylglycerol (52:2) levels	Parkinson's Disease	rs1260326	C	T	GCKR	2	27508073	0.651	49.00	-1.22E-01	1.74E-02	2.79E-12	-6.78E-02	1.72E-02	4.04E-05
Triacylglycerol (52:2) levels	Parkinson's Disease	rs144241030	T	C	NA	1	103243167	0.077	21.69	1.50E-01	3.22E-02	3.26E-06	3.37E-02	6.61E-02	2.14E-01
Triacylglycerol (52:2) levels	Parkinson's Disease	rs147987331	A	T	NA	2	104244849	0.023	21.32	-2.61E-01	5.65E-02	3.96E-06	-3.21E-02	7.92E-02	1.64E-01
Triacylglycerol (52:2) levels	Parkinson's Disease	rs150741556	A	G	NA	5	83807154	0.026	20.64	2.38E-01	5.23E-02	5.63E-06	1.04E-02	1.11E-01	3.35E-02
Triacylglycerol (52:2) levels	Parkinson's Disease	rs2980888	C	T	NA	8	125495066	0.743	20.75	-8.70E-02	1.91E-02	5.32E-06	-2.33E-02	1.82E-02	7.01E-01
Triacylglycerol (52:2) levels	Parkinson's Disease	rs35332062	A	G	MLXIPL	7	73597712	0.122	30.73	-1.41E-01	2.54E-02	3.06E-08	-4.60E-03	2.72E-02	6.20E-02
Triacylglycerol (52:2) levels	Parkinson's Disease	rs501474	G	A	NA	5	6523892	0.425	19.78	7.44E-02	1.67E-02	8.81E-06	-6.80E-03	2.31E-02	1.15E-01
Triacylglycerol (52:2) levels	Parkinson's Disease	rs62291572	T	C	NA	4	4996200	0.214	20.55	9.54E-02	2.10E-02	5.90E-06	-2.71E-02	2.75E-02	4.89E-01
Triacylglycerol (52:2) levels	Parkinson's Disease	rs72753379	T	C	LOC105376253	9	120350676	0.039	21.26	2.06E-01	4.47E-02	4.08E-06	2.30E-02	9.08E-02	9.67E-02
Triacylglycerol (52:2) levels	Parkinson's Disease	rs76950187	G	A	LOC105373611	2	128427075	0.074	23.13	1.54E-01	3.20E-02	1.54E-06	6.08E-02	5.53E-02	5.65E-01
Triacylglycerol (52:2) levels	Parkinson's Disease	rs78275146	T	C	NA	1	196028959	0.014	21.56	3.50E-01	7.53E-02	3.49E-06	7.90E-02	1.03E-01	3.52E-01
Triacylglycerol (52:2) levels	Parkinson's Disease	rs79934453	G	A	NA	6	23919006	0.004	22.08	-6.44E-01	1.37E-01	2.67E-06	-9.28E-02	1.77E-01	2.22E-01
Triacylglycerol (52:2) levels	Parkinson's Disease	rs79954170	A	C	NA	6	49123122	0.008	21.37	-4.43E-01	9.57E-02	3.85E-06	-9.06E-02	8.53E-02	5.40E-01
Triacylglycerol (52:2) levels	Parkinson's Disease	rs964184	C	G	ZPR1	11	116778201	0.849	115.94	-2.48E-01	2.30E-02	7.83E-27	-5.68E-02	2.43E-02	9.71E-03
Triacylglycerol (52:6) levels	Parkinson's Disease	rs10105606	A	C	NA	8	19970337	0.279	24.07	-9.48E-02	1.93E-02	9.51E-07	0.00E+00	1.77E-02	4.34E-05
Triacylglycerol (52:6) levels	Parkinson's Disease	rs1017216	A	G	LOC105377565, LOC105377567	4	180460689	0.459	22.04	8.13E-02	1.73E-02	2.73E-06	-2.06E-02	2.23E-02	4.49E-01
Triacylglycerol (52:6) levels	Parkinson's Disease	rs102274	C	T	TMEM258	11	61790354	0.409	55.60	-1.32E-01	1.78E-02	1.00E-13	-3.90E-03	1.79E-02	8.34E-02
Triacylglycerol (52:6) levels	Parkinson's Disease	rs112578388	T	C	ATP8A2	13	25886443	0.005	20.79	-5.76E-01	1.26E-01	5.22E-06	-2.36E-02	2.04E-01	4.20E-02
Triacylglycerol (52:6) levels	Parkinson's Disease	rs114314612	A	C	NA	2	193027049	0.010	20.23	4.13E-01	9.19E-02	6.97E-06	-6.57E-02	9.83E-02	2.98E-01
Triacylglycerol (52:6) levels	Parkinson's Disease	rs117082735	T	C	LINC01507, LOC105376101	9	79867479	0.022	20.30	-2.64E-01	5.87E-02	6.72E-06	-1.86E-02	9.81E-02	7.07E-02
Triacylglycerol (52:6) levels	Parkinson's Disease	rs117518401	T	C	RAB6B	3	133895761	0.173	22.95	1.12E-01	2.34E-02	1.70E-06	3.10E-02	3.42E-02	4.37E-01
Triacylglycerol (52:6) levels	Parkinson's Disease	rs1260326	C	T	GCKR	2	27508073	0.651	52.83	-1.32E-01	1.82E-02	4.04E-13	-6.78E-02	1.72E-02	4.04E-05
Triacylglycerol (52:6) levels	Parkinson's Disease	rs4508726	G	A	APRG1	3	37387931	0.365	22.88	-8.71E-02	1.82E-02	1.77E-06	-1.15E-02	1.77E-02	2.89E-01
Triacylglycerol (52:6) levels	Parkinson's Disease	rs56257715	A	G	GALNT17	7	71530474	0.009	21.44	4.28E-01	9.25E-02	3.70E-06	-2.63E-02	1.18E-01	8.41E-02
Triacylglycerol (52:6) levels	Parkinson's Disease	rs61905503	C	G	NA	11	89627134	0.050	22.43	-1.90E-01	4.01E-02	2.23E-06	2.24E-02	3.15E-02	3.22E-01
Triacylglycerol (52:6) levels	Parkinson's Disease	rs6752909	T	C	SRBD1	2	45482958	0.826	20.17	-1.04E-01	2.31E-02	7.19E-06	3.28E-02	2.97E-02	5.70E-01
Triacylglycerol (52:6) levels	Parkinson's Disease	rs78095833	T	C	RYR2	1	237378917	0.020	22.62	-3.06E-01	6.44E-02	2.01E-06	-1.08E-01	9.79E-02	5.67E-01
Triacylglycerol (52:6) levels	Parkinson's Disease	rs7815372	G	A	CYP7B1	8	64695903	0.792	20.68	9.80E-02	2.15E-02	5.52E-06	1.07E-02	2.10E-02	2.16E-01
Triacylglycerol (52:6) levels	Parkinson's Disease	rs78178542	C	T	NA	4	179015062	0.020	23.07	-3.13E-01	6.52E-02	1.59E-06	5.70E-02	8.77E-02	2.88E-01
Triacylglycerol (52:6) levels	Parkinson's Disease	rs9591384	A	T	GUCY1B2	13	51065337	0.124	23.55	-1.28E-01	2.65E-02	1.25E-06	-3.30E-02	3.15E-02	5.29E-01
Triacylglycerol (52:6) levels	Parkinson's Disease	rs964184	C	G	ZPR1	11	116778201	0.849	71.32	-2.03E-01	2.41E-02	3.70E-17	-5.68E-02	2.43E-02	9.71E-03
Triacylglycerol (53:2) levels	Parkinson's Disease	rs10147474	G	A	PRKD1	14	29823442	0.037	23.14	-2.19E-01	4.54E-02	1.54E-06	3.15E-02	6.22E-02	2.13E-01
Triacylglycerol (53:2) levels	Parkinson's Disease	rs1042034	T	C	APOB	2	21002409	0.728	19.66	8.37E-02	1.89E-02	9.40E-06	-5.30E-03	2.03E-02	9.96E-02
Triacylglycerol (53:2) levels	Parkinson's Disease	rs111241638	C	T	NA	3	168112642	0.033	19.75	-2.22E-01	4.99E-02	8.97E-06	-5.13E-02	6.15E-02	3.94E-01
Triacylglycerol (53:2) levels	Parkinson's Disease	rs116497138	T	G	NA	3	87180483	0.050	21.53	-1.84E-01	3.97E-02	3.54E-06	5.25E-02	6.09E-02	4.10E-01
Triacylglycerol (53:2) levels	Parkinson's Disease	rs11926107	G	A	EPHB1	3	134995496	0.284	19.89	-8.34E-02	1.87E-02	8.32E-06	-1.72E-02	2.32E-02	3.40E-01
Triacylglycerol (53:2) levels	Parkinson's Disease	rs1260326	C	T	GCKR	2	27508073	0.651	45.40	-1.19E-01	1.76E-02	1.73E-11	-6.78E-02	1.72E-02	4.04E-05
Triacylglycerol (53:2) levels	Parkinson's Disease	rs12635725	G	A	NA	3	1895465	0.560	20.11	7.58E-02	1.69E-02	7.41E-06	2.07E-02	2.20E-02	4.61E-01
Triacylglycerol (53:2) levels	Parkinson's Disease	rs150741556	A	G	NA	5	83807154	0.026	19.86	2.35E-01	5.27E-02	8.45E-06	1.04E-02	1.11E-01	3.35E-02
Triacylglycerol (53:2) levels	Parkinson's Disease	rs174556	T	C	FADS1	11	61813163	0.380	29.71	9.52E-02	1.75E-02	5.17E-08	1.00E-04	1.85E-02	1.04E-03
Triacylglycerol (53:2) levels	Parkinson's Disease	rs34021847	A	G	MAD1L1	7	1998126	0.320	23.92	-8.84E-02	1.81E-02	1.03E-06	-3.21E-02	2.02E-02	9.53E-01
Triacylglycerol (53:2) levels	Parkinson's Disease	rs35332062	A	G	MLXIPL	7	73597712	0.122	26.89	-1.34E-01	2.59E-02	2.21E-07	-4.60E-03	2.72E-02	6.20E-02
Triacylglycerol (53:2) levels	Parkinson's Disease	rs4266911	C	T	SLC9A1	1	27100305	0.941	20.23	1.65E-01	3.67E-02	6.96E-06	-3.37E-02	5.78E-02	2.52E-01
Triacylglycerol (53:2) levels	Parkinson's Disease	rs55870249	G	C	LINC00578	3	177504180	0.225	19.97	9.06E-02	2.03E-02	7.99E-06	1.58E-02	3.00E-02	2.23E-01
Triacylglycerol (53:2) levels	Parkinson's Disease	rs62043594	A	C	LOC101927025	15	93154154	0.418	28.50	-9.06E-02	1.70E-02	9.63E-08	-1.90E-02	2.31E-02	3.86E-01

Triacylglycerol (53:2) levels	Parkinson's Disease	rs62291572	T	C	NA	4	4996200	0.214	23.62	1.03E-01	2.12E-02	1.20E-06	-2.71E-02	2.75E-02	4.89E-01
Triacylglycerol (53:2) levels	Parkinson's Disease	rs72679249	G	A	ELOVL6	4	110201006	0.270	22.58	9.18E-02	1.93E-02	2.06E-06	-1.21E-02	2.92E-02	1.69E-01
Triacylglycerol (53:2) levels	Parkinson's Disease	rs75573052	T	C	LATS2	13	21038010	0.085	20.95	-1.42E-01	3.09E-02	4.79E-06	3.91E-02	3.60E-02	5.57E-01
Triacylglycerol (53:2) levels	Parkinson's Disease	rs76950187	G	A	LOC105373611	2	128427075	0.074	23.66	1.57E-01	3.23E-02	1.17E-06	6.08E-02	5.53E-02	5.65E-01
Triacylglycerol (53:2) levels	Parkinson's Disease	rs964184	C	G	ZPR1	11	116778201	0.849	99.82	-2.33E-01	2.33E-02	2.38E-23	-5.68E-02	2.43E-02	9.71E-03
Triacylglycerol (53:3) levels	Parkinson's Disease	rs1003966	T	C	LATS2	13	21038020	0.085	24.14	-1.50E-01	3.04E-02	9.16E-07	3.83E-02	3.60E-02	5.42E-01
Triacylglycerol (53:3) levels	Parkinson's Disease	rs10147474	G	A	PRKD1	14	29823442	0.037	22.07	-2.11E-01	4.49E-02	2.67E-06	3.15E-02	6.22E-02	2.13E-01
Triacylglycerol (53:3) levels	Parkinson's Disease	rs1042034	T	C	APOB	2	21002409	0.728	26.97	9.71E-02	1.87E-02	2.12E-07	-5.30E-03	2.03E-02	9.96E-02
Triacylglycerol (53:3) levels	Parkinson's Disease	rs111568723	G	A	CFAP77	9	132470502	0.026	21.00	2.50E-01	5.46E-02	4.67E-06	-1.20E-01	5.14E-02	9.73E-03
Triacylglycerol (53:3) levels	Parkinson's Disease	rs11584070	G	A	PRPF3	1	150322476	0.137	21.96	-1.14E-01	2.44E-02	2.83E-06	3.60E-02	2.95E-02	6.55E-01
Triacylglycerol (53:3) levels	Parkinson's Disease	rs116011373	A	G	NA	3	87298641	0.049	23.94	-1.92E-01	3.93E-02	1.02E-06	-2.23E-02	8.48E-02	1.01E-01
Triacylglycerol (53:3) levels	Parkinson's Disease	rs12448223	C	T	ADCY9	16	4104550	0.443	21.41	7.74E-02	1.67E-02	3.77E-06	1.20E-02	2.19E-02	2.35E-01
Triacylglycerol (53:3) levels	Parkinson's Disease	rs1260326	C	T	GCKR	2	27508073	0.651	61.78	-1.37E-01	1.75E-02	4.37E-15	-6.78E-02	1.72E-02	4.04E-05
Triacylglycerol (53:3) levels	Parkinson's Disease	rs12635725	G	A	NA	3	1895465	0.560	23.23	8.07E-02	1.67E-02	1.46E-06	2.07E-02	2.20E-02	4.61E-01
Triacylglycerol (53:3) levels	Parkinson's Disease	rs138622921	C	A	ITPR2	12	26757135	0.017	20.26	-2.91E-01	6.46E-02	6.86E-06	-4.47E-02	1.13E-01	1.60E-01
Triacylglycerol (53:3) levels	Parkinson's Disease	rs139500046	C	A	CFAP54	12	96850670	0.011	23.11	-3.81E-01	7.94E-02	1.56E-06	-4.41E-02	8.65E-02	2.15E-01
Triacylglycerol (53:3) levels	Parkinson's Disease	rs150741556	A	G	NA	5	83807154	0.026	24.12	2.58E-01	5.25E-02	9.24E-07	1.04E-02	1.11E-01	3.35E-02
Triacylglycerol (53:3) levels	Parkinson's Disease	rs174560	C	T	FADS1,FADS2	11	61814292	0.383	36.11	1.04E-01	1.73E-02	1.96E-09	-1.90E-03	1.84E-02	3.82E-02
Triacylglycerol (53:3) levels	Parkinson's Disease	rs28450659	T	C	SH3BP5	3	15332543	0.112	20.32	-1.19E-01	2.63E-02	6.66E-06	-3.90E-02	2.63E-02	8.59E-01
Triacylglycerol (53:3) levels	Parkinson's Disease	rs35332062	A	G	MLXIPL	7	73597712	0.122	33.50	-1.48E-01	2.55E-02	7.43E-09	-4.60E-03	2.72E-02	6.20E-02
Triacylglycerol (53:3) levels	Parkinson's Disease	rs3829088	C	T	ELAVL2	9	23713682	0.203	19.71	9.24E-02	2.08E-02	9.16E-06	2.59E-02	3.15E-02	3.86E-01
Triacylglycerol (53:3) levels	Parkinson's Disease	rs501474	G	A	NA	5	6523892	0.425	20.82	7.66E-02	1.68E-02	5.14E-06	-6.80E-03	2.31E-02	1.15E-01
Triacylglycerol (53:3) levels	Parkinson's Disease	rs62291574	A	G	NA	4	5003811	0.214	22.12	9.93E-02	2.11E-02	2.61E-06	-2.16E-02	2.99E-02	3.29E-01
Triacylglycerol (53:3) levels	Parkinson's Disease	rs6692319	T	C	GALNT2	1	230148628	0.740	25.55	-9.52E-02	1.88E-02	4.41E-07	-1.94E-02	2.53E-02	3.53E-01
Triacylglycerol (53:3) levels	Parkinson's Disease	rs76950187	G	A	LOC105373611	2	128427075	0.074	21.19	1.48E-01	3.21E-02	4.23E-06	6.08E-02	5.53E-02	5.65E-01
Triacylglycerol (53:3) levels	Parkinson's Disease	rs935833	A	G	TMEM132D	12	129791836	0.635	20.17	-7.87E-02	1.75E-02	7.19E-06	1.53E-02	2.34E-02	2.89E-01
Triacylglycerol (53:3) levels	Parkinson's Disease	rs964184	C	G	ZPR1	11	116778201	0.849	150.59	-2.83E-01	2.30E-02	2.83E-34	-5.68E-02	2.43E-02	9.71E-03
Triacylglycerol (53:4) levels	Parkinson's Disease	rs10105606	A	C	NA	8	19970337	0.279	32.42	-1.12E-01	1.96E-02	1.29E-08	0.00E+00	1.77E-02	4.34E-05
Triacylglycerol (53:4) levels	Parkinson's Disease	rs116014876	T	C	GSTM4	1	109657509	0.009	20.14	4.20E-01	9.36E-02	7.33E-06	-2.42E-02	6.12E-02	1.59E-01
Triacylglycerol (53:4) levels	Parkinson's Disease	rs117829392	C	G	DLGAP2	8	771605	0.032	20.69	2.33E-01	5.12E-02	5.50E-06	-3.91E-02	5.06E-02	3.58E-01
Triacylglycerol (53:4) levels	Parkinson's Disease	rs117922651	A	G	NT5C3B	17	41828494	0.057	20.95	-1.77E-01	3.86E-02	4.79E-06	1.42E-02	3.42E-02	1.68E-01
Triacylglycerol (53:4) levels	Parkinson's Disease	rs1260326	C	T	GCKR	2	27508073	0.651	55.34	-1.36E-01	1.83E-02	1.14E-13	-6.78E-02	1.72E-02	4.04E-05
Triacylglycerol (53:4) levels	Parkinson's Disease	rs12635725	G	A	NA	3	1895465	0.560	20.35	7.95E-02	1.76E-02	6.57E-06	2.07E-02	2.20E-02	4.61E-01
Triacylglycerol (53:4) levels	Parkinson's Disease	rs13288401	T	C	NA	9	1147365	0.032	20.27	-2.36E-01	5.24E-02	6.82E-06	2.62E-02	5.30E-02	2.07E-01
Triacylglycerol (53:4) levels	Parkinson's Disease	rs138987797	T	A	IL1RAP	3	190557173	0.003	23.89	7.76E-01	1.59E-01	1.04E-06	2.29E-01	1.12E-01	2.04E-02
Triacylglycerol (53:4) levels	Parkinson's Disease	rs139500046	C	A	CFAP54	12	96850670	0.011	23.17	-4.02E-01	8.34E-02	1.52E-06	-4.41E-02	8.65E-02	2.15E-01
Triacylglycerol (53:4) levels	Parkinson's Disease	rs144500033	T	A	NA	4	27061504	0.003	20.19	-7.65E-01	1.70E-01	7.15E-06	-6.82E-02	1.09E-01	2.76E-01
Triacylglycerol (53:4) levels	Parkinson's Disease	rs17081481	G	A	LINC00457, LINC02343	13	34561042	0.334	20.14	8.65E-02	1.93E-02	7.33E-06	-2.07E-02	2.61E-02	3.69E-01
Triacylglycerol (53:4) levels	Parkinson's Disease	rs1949011	A	C	LRP1B, LOC107985779	2	141741698	0.429	23.64	-8.71E-02	1.79E-02	1.19E-06	3.74E-02	2.30E-02	9.83E-01
Triacylglycerol (53:4) levels	Parkinson's Disease	rs2370511	C	T	NA	12	78389910	0.157	23.27	1.16E-01	2.41E-02	1.44E-06	2.15E-02	2.97E-02	3.29E-01
Triacylglycerol (53:4) levels	Parkinson's Disease	rs351596	T	C	NA	1	4317851	0.975	20.11	-2.62E-01	5.85E-02	7.43E-06	-3.40E-02	4.93E-02	3.10E-01
Triacylglycerol (53:4) levels	Parkinson's Disease	rs35832030	T	G	SLC43A2	17	1624088	0.196	21.16	1.04E-01	2.26E-02	4.30E-06	-1.15E-02	2.45E-02	1.95E-01
Triacylglycerol (53:4) levels	Parkinson's Disease	rs3829088	C	T	ELAVL2	9	23713682	0.203	19.62	9.73E-02	2.20E-02	9.59E-06	2.59E-02	3.15E-02	3.86E-01
Triacylglycerol (53:4) levels	Parkinson's Disease	rs4837740	G	A	NA	9	120256064	0.955	20.43	-2.02E-01	4.46E-02	6.30E-06	-1.41E-02	5.55E-02	9.69E-02
Triacylglycerol (53:4) levels	Parkinson's Disease	rs55672000	A	G	NA	5	66234813	0.027	20.20	-2.55E-01	5.68E-02	7.10E-06	-6.07E-02	1.27E-01	1.98E-01
Triacylglycerol (53:4) levels	Parkinson's Disease	rs62291573	C	T	NA	4	4999649	0.214	20.52	1.00E-01	2.21E-02	6.00E-06	-2.81E-02	2.75E-02	5.13E-01
Triacylglycerol (53:4) levels	Parkinson's Disease	rs7152096	A	G	LOC105370651	14	97793198	0.190	20.01	1.04E-01	2.31E-02	7.83E-06	9.87E-02	4.33E-02	1.13E-02
Triacylglycerol (53:4) levels	Parkinson's Disease	rs71556711	T	C	BAZ1B	7	73440219	0.095	22.28	-1.41E-01	3.00E-02	2.40E-06	1.25E-02	3.50E-02	1.42E-01
Triacylglycerol (53:4) levels	Parkinson's Disease	rs7846649	C	T	TOX	8	59088496	0.191	32.03	1.26E-01	2.23E-02	1.58E-08	6.90E-02	3.38E-02	2.06E-02
Triacylglycerol (53:4) levels	Parkinson's Disease	rs79235240	A	C	ACKR2	3	42816309	0.038	21.77	2.18E-01	4.67E-02	3.14E-06	4.40E-03	3.42E-02	4.66E-02
Triacylglycerol (53:4) levels	Parkinson's Disease	rs825692	A	G	ZFHX3	16	73556815	0.110	23.01	-1.39E-01	2.89E-02	1.65E-06	5.17E-02	3.98E-02	7.13E-01
Triacylglycerol (53:4) levels	Parkinson's Disease	rs964184	C	G	ZPR1	11	116778201	0.849	130.76	-2.74E-01	2.40E-02	5.39E-30	-5.68E-02	2.43E-02	9.71E-03
Triacylglycerol (54:6) levels	Parkinson's Disease	rs114561185	G	A	NA	2	204066660	0.005	22.94	6.17E-01	1.29E-01	1.70E-06	9.99E-02	7.32E-02	7.64E-01

Triacylglycerol (54:6) levels	Parkinson's Disease	rs12405870	T	A	NA	1	187918746	0.315	20.82	-8.33E-02	1.82E-02	5.13E-06	2.23E-02	2.63E-02	4.00E-01
Triacylglycerol (54:6) levels	Parkinson's Disease	rs1260326	C	T	GCKR	2	27508073	0.651	40.76	-1.12E-01	1.76E-02	1.83E-10	-6.78E-02	1.72E-02	4.04E-05
Triacylglycerol (54:6) levels	Parkinson's Disease	rs13178172	G	A	NA	5	118341860	0.739	23.47	9.14E-02	1.89E-02	1.30E-06	4.23E-02	2.42E-02	4.02E-02
Triacylglycerol (54:6) levels	Parkinson's Disease	rs140647506	A	G	NA	9	31005163	0.007	20.57	4.67E-01	1.03E-01	5.83E-06	1.00E-01	1.47E-01	3.05E-01
Triacylglycerol (54:6) levels	Parkinson's Disease	rs1456674	C	T	NA	3	148163041	0.009	20.81	-4.37E-01	9.58E-02	5.16E-06	5.18E-02	6.10E-02	4.02E-01
Triacylglycerol (54:6) levels	Parkinson's Disease	rs174554	G	A	FADS1	11	61811991	0.406	32.97	-9.83E-02	1.71E-02	9.75E-09	-3.50E-03	1.79E-02	7.26E-02
Triacylglycerol (54:6) levels	Parkinson's Disease	rs17596144	T	C	MTARC1	1	220805303	0.152	21.72	1.10E-01	2.36E-02	3.22E-06	-2.90E-03	2.58E-02	4.12E-02
Triacylglycerol (54:6) levels	Parkinson's Disease	rs181609235	G	A	NA	6	164617090	0.012	20.35	-3.51E-01	7.79E-02	6.54E-06	1.21E-02	9.66E-02	4.55E-02
Triacylglycerol (54:6) levels	Parkinson's Disease	rs190411877	C	T	TAF3	10	7871139	0.009	23.82	4.32E-01	8.86E-02	1.08E-06	5.44E-02	2.09E-01	9.96E-02
Triacylglycerol (54:6) levels	Parkinson's Disease	rs268	G	A	LPL	8	19956018	0.023	26.24	2.94E-01	5.74E-02	3.09E-07	1.59E-02	6.69E-02	9.03E-02
Triacylglycerol (54:6) levels	Parkinson's Disease	rs4057791	C	A	NA	4	163303980	0.546	22.93	8.08E-02	1.69E-02	1.72E-06	-8.90E-03	2.07E-02	1.76E-01
Triacylglycerol (54:6) levels	Parkinson's Disease	rs417237	T	G	OBSCN	1	228344494	0.590	20.02	7.58E-02	1.69E-02	7.77E-06	1.73E-02	1.71E-02	5.04E-01
Triacylglycerol (54:6) levels	Parkinson's Disease	rs6501273	T	C	NA	17	78966282	0.793	20.74	9.55E-02	2.10E-02	5.34E-06	-2.00E-04	2.92E-02	2.70E-03
Triacylglycerol (54:6) levels	Parkinson's Disease	rs9344829	T	C	EYS	6	64177173	0.373	19.94	7.75E-02	1.73E-02	8.10E-06	-2.70E-03	1.90E-02	5.16E-02
Triacylglycerol (54:6) levels	Parkinson's Disease	rs964184	C	G	ZPR1	11	116778201	0.849	88.64	-2.18E-01	2.32E-02	6.27E-21	-5.68E-02	2.43E-02	9.71E-03
