# Supplementary material for: Investigating plasma lipid profiles in association with Parkinson’s disease risk
Source: NPJ Parkinsons Dis. 2025 Apr 28;11:99. doi: 10.1038/s41531-025-00955-8 (PMC12038023; doi:10.1038/s41531-025-00955-8)
Supplement: Supplementary file 2 — Financial Disclosures and Conflict of Interest declaration [file 41531_2025_955_MOESM2_ESM.docx]

**Supplementary information**

**Supplementary Table 1.** Used SNP date of the genetic predispositions to lipid traits and PD risk.

**Supplementary Table 2.** Result data of association of genetic predispositions to lipid traits with risk of PD.

**Supplementary Table 3.** Summary of SNPs used for all significant correlation association evidence.
